# Cardiac Safety of Modified Vaccinia Ankara for Vaccination against Smallpox in a Young, Healthy Study Population

**DOI:** 10.1371/journal.pone.0122653

**Published:** 2015-04-16

**Authors:** Eva-Maria Zitzmann-Roth, Frank von Sonnenburg, Stephan de la Motte, Nathaly Arndtz-Wiedemann, Alfred von Krempelhuber, Nadine Uebler, Jens Vollmar, Garth Virgin, Paul Chaplin

**Affiliations:** 1 Praxis Zitzmann-Roth, München, Germany; 2 Department of Infectious Diseases and Tropical Medicine, Section of International Medicine and Public Health, Ludwig-Maximilians-Universität, Munich, Germany; 3 Harrison Clinical Research GmbH, Munich, Germany; 4 Bavarian Nordic GmbH, Martinsried, Germany; Universidad de Valladolid, SPAIN

## Abstract

**Background:**

Conventional smallpox vaccines based on replicating vaccinia virus (VV) strains (e.g. Lister Elstree, NYCBOH) are associated with a high incidence of myo-/pericarditis, a severe inflammatory cardiac complication. A new smallpox vaccine candidate based on a non-replicating Modified Vaccinia Ankara (MVA) poxvirus has been assessed for cardiac safety in a large placebo-controlled clinical trial.

**Methods:**

Cardiac safety of one and two doses of MVA compared to placebo was assessed in 745 healthy subjects. Vaccinia-naïve subjects received either one dose of MVA and one dose of placebo, two doses of MVA, or two doses of placebo by subcutaneous injection four weeks apart; vaccinia-experienced subjects received a single dose of MVA. Solicited and unsolicited adverse events (AE) and cardiac safety parameters (recorded as Adverse Events of Special Interest, AESI) were monitored after each injection.

**Results:**

A total of 5 possibly related AESI (3 cases of palpitations, 2 of tachycardia) were reported during the study. No case of myo- or pericarditis occurred. One possibly related serious AE (SAE) was reported during the 6-month follow-up period (sarcoidosis). The most frequently observed AEs were injection site reactions.

**Conclusions:**

Vaccination with MVA was safe and well tolerated and did not increase the risk for development of myo-/pericarditis.

**Trial Registration:**

ClinicalTrials.gov NCT00316524

## Introduction

Due to the eradication of smallpox in 1980 and discontinuation of smallpox vaccinations worldwide, an increasing majority of the global population has no existing immunity to Variola virus (VARV). As the release of this highly contagious virus would have devastating effects, the potential use of VARV as an agent in bioterrorism or bio-warfare has raised significant concerns. Thus, many governments recognize the need for a safe and efficacious smallpox vaccine to secure public health interests.

MVA is a highly attenuated strain of VV that fails to replicate in human cells [1] and therefore cannot be transmitted or cause disseminated VV infection, which is an important safety concern of conventional smallpox vaccines[2–4]. Conventional vaccines are also associated with severe adverse events, including myo-/pericarditis or even death [3,5,6]. During the US National Smallpox Pre-Event Vaccination Programs initiated in 2002–2004, an unexpected high incidence of myo-/pericarditis was detected in close temporal relationship to Dryvax administration (> 1:10,000), which was notably higher than the expected rate in non-vaccinated US military personnel (0.21:10,000) [7–13]. In addition to clinical symptoms indicative of a cardiac event, criteria for diagnosis of suspected myo-/pericarditis included elevated serum levels of cardiac enzymes (CK-MB, troponin), usually in the presence of ST-segment elevation on ECG, and wall motion abnormalities on echocardiogram[14]. The vast majority of cases occurred within the first 14 days post-vaccination. Most subjects presented initially with chest pain or prodromal clinical symptoms including fever, chills, myalgia, arthralgia, and headache[15–17].

Moreover, a remarkably high number of suspected and probable myo-/pericarditis cases were reported in the pivotal clinical program of the second generation smallpox vaccine ACAM2000 [18]. In contrast to the national vaccination programs where cases were identified when symptomatic vaccinees presented for medical evaluation, the ACAM2000 trials included active post-vaccination cardiac monitoring, leading to detection of 8 myo-/pericarditis cases in 1,162 primary vaccinees after vaccination with either ACAM2000 or Dryvax (incidence of 1:145). Most cases were symptomatic (i.e. subjects presented with chest pain, reduced tolerance to exercise, dyspnea or palpitations) and diagnosis was confirmed by coexisting ECG changes and/or echocardiogram, findings indicative of myocardial inflammation.

Based on this experience, a causal association between conventional smallpox vaccines and myo-/pericarditis has been postulated, although the pathomechanism for cardiotoxicity remains unclear[13]. This has led to the inclusion of close cardiac monitoring in clinical development programs for poxvirus based smallpox vaccines like MVA.

BN has performed a large placebo-controlled Phase II study to determine immunogenicity and safety of MVA with special focus on cardiac safety. Monitoring of ECG changes, new onset of cardiac symptoms and increase of cardiac enzymes was performed to assess the potential risk of developing myo-/pericarditis after vaccination with MVA.

## Material and Methods

The protocol for this study and supporting CONSORT checklist are available as supporting information; see S1 CONSORT Checklist and S1 Protocol.

### Vaccine

The MVA strain was derived from the MVA vaccine licensed in Germany by additional passages and serial dilutions in chicken embryo fibroblast (CEF) cells[5,6]. MVA smallpox vaccine was produced by IDT Biologika GmbH (Dessau-Roßlau, Germany) according to GMP and provided by Bavarian Nordic A/S (Kvistgaard, Denmark) in aliquots of 0.5 ml liquid-frozen vaccine containing at least 1 x 10^8^ TCID_50_ (Tissue Culture Infectious Dose 50) /ml.

### Participants and Study Procedures

The study was performed at a Clinical Research Organization in compliance with current standards of the International Conference on Harmonization of Technical Requirements for Registration of Pharmaceuticals for Human Use—Good Clinical Practice (ICH-GCP), the Declaration of Helsinki and local legal and regulatory requirements. A total of 1,322 volunteers between 18 and 55 years of age were screened (Table 1). Healthy subjects with normal baseline ECG, cardiac troponin I within normal limits and no active or history of cardiac disease were eligible for inclusion in the study.

**Table 1:** Demographics.

		Group 1 (MM) vaccinia-naïve (N = 183)	Group 2 (MP) vaccinia-naïve (N = 181)	Group 3 (PP) vaccinia-naïve (N = 181)	Group 4 (M-) vaccinia-exp. (N = 200)
**Age [years]**	Mean	25.3	25.4	26.0	41.5
	SD	5.0	4.4	5.1	7.6
	Median	24	25	25	42
	Min	18	18	18	22
	Max	50	44	50	55
**BMI [kg/m^2^]**	Mean	23.6	23.7	23.9	24.6
	SD	3.86	3.41	4.31	3.93
	Median	23.0	23.2	23.1	23.9
	Min	17.5	17.4	16.2	17.6
	Max	46.7	35.8	46.5	40.7
**Gender (n/%)**	Male	86 (47.0)	69 (38.1)	74 (40.9)	85 (42.5)
	Female	97 (53.0)	112 (61.9)	107 (59.1)	115 (57.5)
**Ethnic group (n/%)**	Caucasian	178 (97.3)	176 (97.2)	177 (97.8)	198 (99.0)
	Asian	1 (0.5)	2 (1.1)	1 (0.6)	0 (0.0)
	Black	0 (0.0)	1 (0.6)	0 (0.0)	1 (0.5)
	Arabic	2 (1.1)	1 (0.6)	1 (0.6)	0 (0.0)
	Other	2 (1.1)	1 (0.6)	2 (1.1)	1 (0.5)

MM = two vaccinations with MVA; MP = first vaccination with MVA, second vaccination with placebo; PP = two vaccinations with placebo; M- = single vaccination with MVA; BMI = body mass index; N = Number of subjects in each group; n = Number of subjects with data available; % = Percentage based on N; SD = Standard deviation

Subjects with coronary heart disease, myocardial infarction, angina, congestive heart failure, cardiomyopathy, stroke or transient ischemic attack, uncontrolled high blood pressure, any other medically treated heart condition, a family history of death due to ischemic heart disease before age 50 and/or a ≥ 10 percent risk for myocardial infarction or coronary death within the next 10 years were excluded. Exclusionary ECG findings included any kind of atrioventricular or intraventricular conditions or blocks such as complete left or right bundle branch block, AV node block, QTc or PR prolongation; premature atrial contractions or other atrial arrhythmia, sustained ventricular arrhythmia, two premature ventricular contractions (PVC) in a row; ST elevation consistent with ischemia.

**Fig 1:**
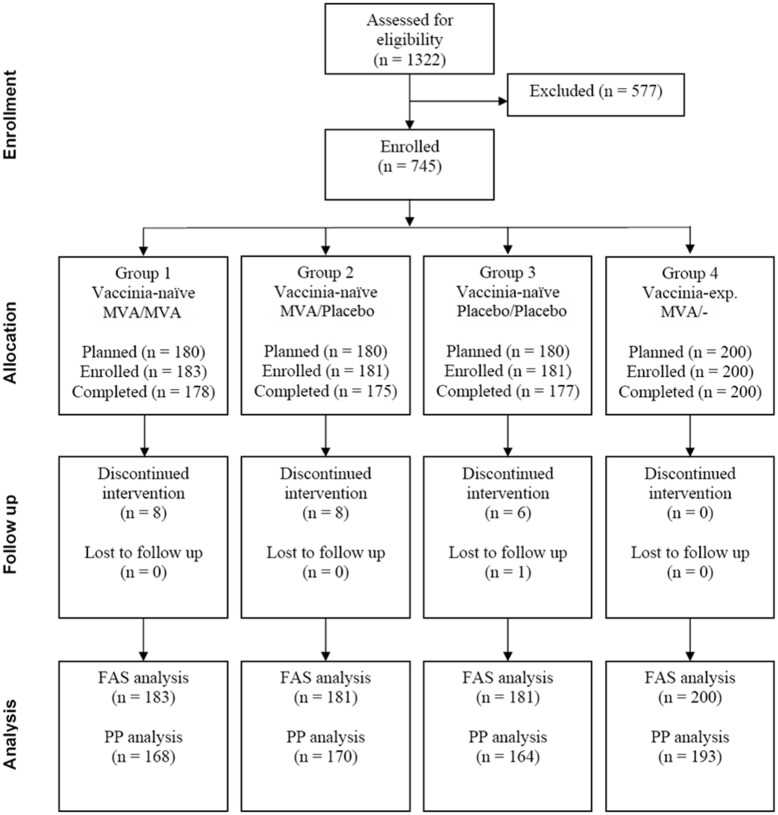
Consolidated Standards of Reporting Trials (CONSORT) subject flow diagram demonstrating the number of patients recruited into the study, number of subjects randomized and vaccinated, and number of subjects analyzed. MVA: Modified Vaccinia Ankara; FAS: full analysis set: PP: per protocol

The study was approved by the ethics committee of the Ludwig-Maximilians-Universitaet, Munich, Germany, under the approval number 365–05. Written informed consent was obtained from all subjects. Seven hundred and forty-five (745) subjects were assigned to four groups (Fig 1): Subjects with no history of previous smallpox vaccination (vaccinia-naïve) were randomly assigned to Groups 1 to 3 to receive (under double-blind conditions, including subject handling, trial personnel and data analysis) either two subcutaneous (s.c.) injections of MVA, one dose of MVA and one dose of placebo or two doses of placebo, respectively. Group 4 (open-label) consisted of subjects with a history of previous smallpox vaccination (vaccinia-experienced) that received a single vaccination with MVA. Enrolment started in April 2006, the last follow-up visit was performed in August 2007.

### Safety Assessments

Safety laboratory tests, including troponin I and ECGs were performed at the screening visit and 10–15 days after each vaccination, and 28 to 35 days after the second (Groups 1–3) or single vaccination (Group 4), as well as at the 6-month follow-up visit, if clinically indicated. All subjects were monitored for ECG changes and cardiac symptoms, such as chest pain, shortness of breath and palpitations, by a physical examination at all trial visits including heart and lung auscultation specifically looking for heart failure, presence of rubs, gallops, murmurs, crackles, and rales.

ECGs were evaluated by the investigator and transmitted electronically to a central database. Subjects with abnormal ECG findings were referred to a cardiologist, which included a thorough examination and performance of an echocardiogram and/or treadmill ECG.

Any cardiac symptom, clinically significant ECG changes or elevated cardiac enzymes (specifically troponin I above upper limit of normal [ULN]) were recorded as an adverse event of special interest (AESI) and followed-up thoroughly to identify or rule-out any cardiac abnormalities. The case definitions as published by the US Center of Disease Control (CDC) [14] were applied to recognize possible cases of acute myocarditis and pericarditis and to distinguish unspecific and isolated ECG changes without or with unclear clinical meaning and ECG changes related to possible or probable cases of acute myocarditis and/or pericarditis.

Unsolicited AE were recorded by the investigator at every study visit by asking the subject for any change in health status since the last visit. Solicited local and general AE (injection site erythema, swelling and induration as well as increased body temperature, headache, myalgia, nausea and fatigue) and any additional symptoms were recorded by the subjects on a diary on the day of vaccination and for the following 7 days, or longer, if lasting beyond the 7-day period. AEs not resolved at the last visit were followed-up and any potentially new SAE and AESI were recorded at the follow-up visit.

### Statistical Methods

ECGs were evaluated by a centralized ECG laboratory. Standard ECG results like PQ, QRS, QT and QTc durations and formations as well as heart rate were summarized per visit and group.

Occurrence, relationship and intensity of ECG changes and any other cardiac symptom at any time during the study were assessed and compared between groups by means of the exact test of Fisher. The comparison of the four treatment groups with regard to ECG changes and cardiac symptoms was the primary safety objective of this trial.

Detailed QTc analysis was performed according to FDA/EMA guidance [19]. PR, QT and QRS durations as well as the reported QTc duration based on the Bazett correction, and heart rate were tabulated per group and visit.

AESI were defined as any cardiac symptom, clinically significant ECG changes or cardiac enzymes elevated above the ULN, and were separately listed and tabulated; incidence was compared between groups taking Group 3 (placebo) as the reference group by means of the exact test of Fisher. Here the following commonly used classification of significance was used to display the results: NS = Not Significant (i.e. p≥0.05); * p< 0.05; ** p<0.01; *** p < 0.001).

Unsolicited AE were coded using the MedDRA coding terminology and graded based on intensity. The number of AE and number of subjects with at least one AE for each preferred term were descriptively compared between groups. The occurrence of any Grade 3 or higher AE *possibly*, *probably or definitely* related to the study vaccine within 28 days after vaccination was compared between groups.

Occurrence and intensity of solicited general AE within one week after each vaccination was summarized per patient and vaccination. Occurrence and intensity of solicited local AE were documented and graded according to maximum severity. The maximum intensity over the 7-day period after vaccination was used, categorized as Grades 1 to 4 and compared between groups groups using Group 3 as the reference group by means of Fisher’s exact test.

SAE were listed separately and individually described. The number of subjects with at least one SAE was compared between groups using Group 3 as the reference group by means of Fisher’s exact test.

## Results

### Participants and Demographic Data

Data of 745 randomized subjects who were administered at least one vaccination (564 with MVA and 181 with placebo) were included in the analysis (Fig 1). Of the vaccinia-naïve subjects, 180 subjects (Group 1) received two doses of MVA and three subjects from Group 1 as well as 181 subjects from Group 2 received a single dose of MVA. 174 subjects in Group 2 received placebo with the second injection. 200 vaccinia-experienced subjects received a single dose of MVA (Group 4).

There were slightly more female than male subjects in all groups (Table 1). The vaccinia-naïve population was younger than the vaccinia-experienced population. The median body mass index (BMI) between groups was similar.

### Cardiac Monitoring

To ensure that subjects did not have any cardiac abnormalities prior to enrollment, all subjects received an ECG assessment at screening. All ECGs were assessed by the investigator and a central ECG laboratory, and any abnormal or unclear findings were further assessed by a cardiologist. Of the 745 enrolled subjects, 324 (43.5%) had initial ECGs which led to a more thorough cardiac work-up. The ECGs from all enrolled subjects were ultimately assessed to be normal, of these 358 (48.1%) normal ECGs and 386 (51.6%) had ECGs with normal variations such as sinus arrhythmia.

A total of 11 AESI were reported for 10 subjects during the active phase (successful screening until 28 days after last vaccination) of the study. The reported events were palpitations, tachycardia and sinus tachycardia, of which 5 events in 4 subjects were considered as possibly related to the study vaccine (Tables 2 and 3). A further five AESI were reported during follow-up (later than 28 days after last vaccination until end of trial) in five subjects consisting of palpitations, tachycardia and mild pericardial effusion, all assessed as unlikely related.

**Table 2:** Overview of Unsolicited Adverse Events per Subject.

Subject based	Group 1	Group 2	Group 3	Group 4
	(MM)	(MP)	(PP)	(M/-)
	vaccinia-naïve	vaccinia-naïve	vaccinia-naïve	vaccinia-exp.
	(N = 183)	(N = 181)	(N = 181)	(N = 200)
SAE	3 (1.6) NS	2 (1.1) NS	5 (2.8)	1 (0.5) NS
Possibly related SAE*	1 (0.5) NS	0 (0.0) NS	0 (0.0)	0 (0.0) NS
AESI	3 (1.6) NS	4 (2.2) NS	1 (0.6)	8 (4.0) *
Possibly related AESI	0 (0.0) NS	2 (1.1) NS	0 (0.0)	2 (1.0) NS
At least one unsolicited AE	113 (61.7) ***	107 (59.1) **	78 (43.1)	99 (49.5) NS
Related unsolicited AE	61 (33.3) ***	61 (33.7) ***	23 (12.7)	74 (37.0) ***
Related unsolicited AE ≥ Grade 3	4 (2.2) NS	8 (4.4) NS	4 (2.2)	1 (0.5) NS
AE leading to withdrawal from study	0 (0.0) NS	1 (0.6) NS	1 (0.6)	0 (0.0) NS

AE = adverse event (up to 28 days after the first vaccination); SAE = serious adverse event (up to 6 months after the last vaccination); AESI = adverse event of special interest (cardiac event; up to 6 months after the last vaccination); * reported during the 6-month follow-up period. Fishers Exact test of comparison to Group 3; NS = Not Significant (p≥0.05);

* p< 0.05;

** p<0.01;

*** p < 0.001).

**Table 3:** Cardiac Adverse Events (AESI).

Subject #	AESI (Diagnosis)	Relationship to vaccine	Onset of symptoms (post last vaccination)	Duration	Other conditions	Cardiac work-up/safety lab	Outcome
**Group 1 (MM) vaccinia-naïve (N = 183)**							
287	Sinus tachycardia (102 bpm)	Unlikely	14 days	Minutes	Thyroxin treatment for hypothyreosis	ECG; TSH, T3, T4 (normal)	Recovered
317	Tachycardia (116 bpm)	Unlikely	31 days	4 months	Psychological stress	TSH, T3, T4 (normal)	Recovered
557	Palpitations	Unlikely	2 days	5 seconds	Past history of palpitations during exercise; treatment for hypothyreosis	-	Recovered
**Group 2 (MP) vaccinia-naïve (N = 181)**							
220	Palpitations	Possibly	15 hours	2.5 hours	Treatment for allergic rhinitis	-	Recovered
228	Sinus tachycardia (111 bpm)	Unlikely	13 days	< 1 day	Pre-existing HR 98 bpm	Echocardiogram, ECG and TSH/T3/T4 (normal)	Normal Recovered
563	Tachycardia (105 bpm)	Possibly	28 days	2 days	Nervousness, pre-existing HR 81 bpm	-	Recovered
710	Palpitations (FU)	Unlikely	193 days	intermittent	Personal distress	-	Ongoing
**Group 3 (PP) vaccinia-naïve (N = 181)**							
689	Palpitations	Unlikely	23 days	intermittent	Recurrent pain in shoulder	-	Ongoing
**Group 4 (M-) vaccinia-exp. (N = 200)**							
024	Palpitations (FU)	Unlikely	>90 days	intermittent	Anxiety, depression	-	Recovered
028	Palpitations	Possibly	3 hours	1 hour	Sweating	-	Recovered
	Palpitations	Possibly	3 days	~ 5 hours	Sweating	ECG, cardiac enzymes (normal)	Recovered
	Palpitations (FU)	Unlikely	92 days	1,5 hours	Common cold, sweating	Echocardiogram, ECG (normal)	Recovered
246	Tachycardia (105 bpm) (FU)	Unlikely	182 days	13 days	Nervousness	Echocardiogram, ergometry, TSH, T3, T4 (normal)	Recovered
397	Tachycardia (bpm 120)	Possibly	~ 4 hours	3 days	Nervousness	TSH, T3, T4 (normal)	Recovered
411	Palpitations	Unlikely	34 hours	10 minutes	Hypotension, post-traumatic pain right shoulder	ECG (normal)	
	Mild pericardial effusion (FU)	Unlikely	196	ongoing		Echocardiogram (abnormal); ECG, ergometry (normal)	Abnormal, but clinically not significant Ongoing

Bpm = beats per minute (heart rate); HR = heart rate; Adverse events of special interest (AESI) that were reported during the half-year follow-up phase are marked with “FU”

Subjects showing any ECG abnormalities at any of the post-vaccination visits underwent a thorough cardiac work-up by a cardiologist. Nearly one third of all subjects received echocardiograms and/or treadmill ECGs during the study and all examinations completely ruled out any cardiac abnormalities. Table 4 summarizes all ECG abnormalities observed by group and by visit. Approxinately 20–30% of subjects in each group with normal ECGs at baseline had ECG changes at visit 2 consistent with normal variation and approximately 20–30% of subjects with ECGs demonstrating normal variation at baseline had normal ECGs at visit 2 (Table 4). Similar percentages of subjects with ECG changes were observed at Visit 4, whereby nearly half had reverted back to the original screening assessment.

**Table 4:** Summary of Electrocardiogram Abnormalities (Safety Analysis Set, N = 745).

	Group 1	Group 2	Group 3	Group 4
	n (%) N = 183	n (%) N = 181	n (%) N = 181	n (%) N = 200
**Screening**				
Assessment missing	0	0	0	0
Normal	87 (47.5%) NS	78 (43.1%) NS	76 (42.0%)	118 (59.0%) **
Abnormal, CS	0	0	0	0
Abnormal, NCS	96 (52.5%)	103 (56.9%)	105 (58.0%)	82 (41.0%)
AV block (PQ time > 0.20 sec)—first degree	0	0	1 (0.6%)	1 (0.5%)
Right bundle branch block—incomplete	10 (5.5%)	18 (9.9%)	4 (2.2%)	1 (0.5%)
ST elevation	5 (2.7%)	1 (0.6%)	4 (2.2%)	1 (0.5%)
ST depression	0	1 (0.6%)	0	1 (0.5%)
T inversion—pathological	42 (23.0%)	54 (29.8%)	50 (27.6%)	48 (24.0%)
T inversion—other	14 (7.7%)	19 (10.5%)	22 (12.2%)	16 (8.0%)
Low voltage	0	0	0	1 (0.5%)
Other*	23 (12.6%)	23 (12.7%)	26 (14.4%)	5 (2.5%)
Bradycardia	30 (16.4%)	28 (15.5%)	30 (16.6%)	6 (3.0%)
Arrhythmia	4 (2.2%)	3 (1.7%)	8 (4.4%)	1 (0.5%)
Repolarisation	3 (1.6%)	3 (1.7%)	3 (1.7%)	0
Q-Abnormalities	1 (0.5%)	1 (0.6%)	0	4 (2.0%)
Tall T-waves	0	0	0	0
**Two weeks post vaccination 1**				
Assessment missing	1 (0.5%)	2 (1.1%)	2 (1.1%)	0
Normal	91 (49.7%) NS	81 (44.8%) NS	84 (46.4%)	129 (64.5%) ***
Abnormal, CS	0	0	0	0
Abnormal, NCS	91 (49.7%)	98 (54.1%)	95 (52.4%)	71 (35.5%)
Supraventricular arrhythmia	0	1 (0.6%)	0	0
Ventricular arrhythmia	0	1 (0.6%)	1 (0.6%)	0
AV block (PQ time > 0.20 sec)—first degree	0	0	1 (0.6%)	1 (0.5%)
Right bundle branch block—incomplete	9 (4.9%)	17 ((9.4%)	15 (8.3%)	15 (7.5%)
ST elevation	3 (1.6%)	1 (0.6%)	2 (1.1%)	0
ST depression	0	1 (0.6%)	1 (0.6%)	1 (0.5%)
T inversion—pathological	34 (18.6%)	46 (25.4%)	40 (22.1%)	31 (15.5%)
T inversion—other	11 (6.0%)	9 (5.0%)	10 (5.5%)	7 (3.5%)
Other*	34 (18.6%)	29 (16.0%)	34 (18.8%)	14 (7.0%)
Bradycardia	31 (16.9%)	25 (13.8%)	33 (18.2)	6 (3.0%)
Arrhythmia	4 (2.2%)	4 (2.2%)	2 (1.1%)	5 (2.5%)
Repolarisation	1 (0.5%)	3 (1.7%)	2 (1.1%)	1 (0.5%)
Q-Abnormalities	0	3 (1.7%)	1 (0.6%)	4 (2.0%)
Tall T-waves	1 (0.5%)	3 (1.7%)	0	0
**Two weeks post vaccination 2**				
Assessment missing	7 (3.8%)	7 (3.9%)	6 (3.3%)	NA
Normal	89 (48.6%) NS	82 (45.3%) NS	82 (45.3%)	
Abnormal, CS	0	0	0	
Abnormal, NCS	87 (47.5%)	92 (50.1%)	93 (51.4%)	
Supraventricular arrhythmia	1 (0.5%)	1 (0.6%)	0	
AV block (PQ time > 0.20 sec)—first degree	2 (1.1%)	0	1 (0.6%)	
Right bundle branch block—incomplete	10 (5.5%)	20 (11.0%)	12 (6.6%)	
ST elevation	1 (0.5%)	3 (1.7%)	1 (0.6%)	
ST depression	1 (0.5%)	0	0	
T inversion—pathological	28 (15.3%)	37 (20.4%)	39 (21.5%)	
T inversion—other	3 (1.6%)	5 (2.8%)	4 (2.2%)	
Other*	35 (19.1%)	35 (19.3%)	33 (18.2%)	
Bradycardia	30 (16.4%)	23 (12.7%)	28 (15.5%)	
Arrhythmia	4 (2.2%)	4 (2.2%)	2 (1.1%)	
Repolarisation	2 (1.1%)	3 (1.7%)	7 (3.9%)	
Q-Abnormalities	1 (0.6%)	0	1 (0.6%)	
Tall T-waves	1 (0.6%)	1 (0.6%)	2 (1.1%)	

N = number of subjects in each group, % = percentage based on N. NA = not applicable; AV = atrioventricular, CS = clinically significant, NCS = not clinically significant, PQ = PQ interval in electrocardiogram, QTc = QT interval corrected for hart rate, ST = ST segment in electrocardiogram,

*Other: non-specific ECG findings not consistent with one of the terms specified in this table

This table includes ECGs obtained in routine study visits only, but no ECGs obtained in potential further workups.

Fishers Exact test of comparison to Group 3; NS = Not Significant (p≥0.05);

* p< 0.05;

** p<0.01;

*** p < 0.001).

Despite the unexpectedly large number of subjects with ECG findings which triggered further work-up, all ECG recordings were ultimately assessed by the investigator and/or the cardiologist to be normal or represent normal variations. No significant QTc prolongations nor changes in PR, QRS and/or RR intervals were considered related to vaccination. Differences in the rates of normal ECGs between the active treatment groups versus placebo were not significant for groups 1 and 2, and showed more normal ECGs than in the placebo group for group 4 (Vaccinia experienced subjects). There were no differences between groups including placebo with regard to ECG changes and cardiac symptoms and no cases of myo- or pericarditis were reported.

### Solicited and unsolicited Adverse Events

A total of 718 AEs were reported during the active study phase. The majority of unsolicited AEs were mild or moderate (Grades 1 and 2); of the 18 cases documented as severe (Grade 3), only chills and injection site urticaria were assessed as related to study vaccine. Furthermore, there were no vaccine-related clinically significant laboratory abnormalities.

During the active phase of the study, three SAEs were reported which were assessed as unrelated (ruptured finger tendon) or unlikely related (nervous breakdown, transient motoric hemiparesis) to the study vaccine. The case of transient motoric hemiparesis occurred in a 38-year old female subject 6 days after the first vaccination and was judged by the investigator to be unlikely related to study vaccine but rather related to a previous history of migraine accompagnée,

During the 6-month follow up, nine SAEs were reported, thereof for one (sarcoidosis, diagnosed by bronchoscopy and biopsy), a causal relationship could not be ruled out. Ten weeks following the second vaccination with MVA, a 30 year old, otherwise healthy male subject contracted a urinary tract infection (Chlamydia trachomatis), which was treated with antibiotics. Concomitant arthralgia and fever continued, leading to a chest x-ray, bronchoscopy and finally the diagnosis of sarcoidosis. The responsible Data Safety Monitoring Board members assessed the event to be unlikely related to vaccination and expressed no concerns regarding continuation of the MVA development program. Two further SAEs, colon carcinoma and depression, were judged to be unlikely related to vaccination, while the remaining were considered to be not related.

Solicited general as well as local symptoms were generally moderate in severity and none were considered life-threatening or disabling (Grade 4). Table 5 shows related AEs with a frequency of ≥2% in at least one study group. Most AEs belong to the System Organ Class (SOC) “General disorders and administration site conditions”. As expected, a clear difference between placebo and MVA was observed: 98.9%, 97.8% and 97.0% of subjects in Groups 1, 2 and 4 had at least one vaccine-related solicited or unsolicited AE, respectively, compared to 56.9% receiving placebo. Local injection site reactions such as pain, erythema, induration, swelling, pruritus and warmth presented with a significantly lower frequency in the placebo group and indicate a direct relationship to vaccination with MVA compared to placebo. Nevertheless, pain and erythema did still occur in 20.4% and 21.5%, respectively, of subjects receiving placebo.

**Table 5:** Related Adverse Events with a frequency of ≥2% in at least one study group; MedDRA Coding by System Organ Class and Preferred Term (Safety dataset).

		N = Number of subjects in the specified group			
SOC	Preferred Term (PT)	Group 1 (N = 183)	Group 2 (N = 181)	Group 3 (N = 181)	Group 4 (N = 200)
		Number of subjects (%)—Number of events			
**General disorders and administration site conditions**					
	Any PT	181 (98.9) 1392 ***	177 (97.8) 824 ***	103 (56.9) 252	194 (97.0) 894 ***
	Injection site pain	166 (90.7) 300	155 (85.6) 168	37 (20.4) 46	167 (83.5) 167
	Injection site erythema	166 (90.7) 304	146 (80.7) 160	39 (21.5) 51	169 (84.5) 169
	Injection site induration	162 (88.5) 273	146 (80.7) 155	5 (2.8) 6	155 (77.5) 155
	Injection site swelling	149 (81.4) 247	103 (56.9) 112	10 (5.5) 12	149 (74.5) 149
	Fatigue	57 (31.1) 75	42 (23.2) 52	45 (24.9) 53	71 (35.5) 71
	Headache	42 (23.0) 49	58 (32.0) 66	31 (17.1) 36	51 (25.5) 51
	Injection site pruritus	43 (23.5) 61	36 (19.9) 37	4 (2.2) 4	47 (23.5) 47
	Myalgia	23 (12.6) 24	15 (8.3) 15	14 (7.7) 18	39 (19.5) 39
	Body temperature increased	16 (8.7) 18	19 (10.5) 19	10 (5.5) 10	10 (5.0) 10
	Nausea	12 (6.6) 14	17 (9.4) 20	8 (4.4) 8	16 (8.0) 16
	Injection site hematoma	6 (3.3) 6	9 (5.0) 9	4 (2.2) 4	4 (2.0) 4
	Injection site warmth	9 (4.9) 9	5 (2.8) 5	0 (0.0) 0	8 (4.0) 8
	Injection site discoloration	1 (0.5) 1	4 (2.2) 4	0 (0.0) 0	0 (0.0) 0
**Nervous system disorders**					
	Any PT	1 (0.5) 1	6 (3.3) 6	3 (1.7) 3	6 (3.0) 7
	Dizziness	1 (0.5) 1	2 (1.1) 2	2 (1.1) 2	4 (2.0) 4
**Infections and infestations**					
	Any PT	6 (3.3) 7	1 (0.6) 1	5 (2.8) 6	2 (1.0) 3
	Nasopharyngitis	5 (2.7) 5	0 (0.0) 0	4 (2.2) 4	1 (0.5) 1
**Gastrointestinal disorders**					
	Any PT	2 (1.1) 3	1 (0.6) 1	2 (1.1) 3	7 (3.5) 7
	Diarrhoea	0 (0.0) 0	1 (0.6) 1	1 (0.6) 1	4 (2.0) 4
**Respiratory, thoracic and mediastinal disorders**					
	Any PT	2 (1.1) 2	2 (1.1) 2	2 (1.1) 2	5 (2.5) 5
	Pharyngolaryngeal pain	2 (1.1) 2	1 (0.6) 1	1 (0.6) 1	4 (2.0) 4
**Blood and lymphatic system disorders**					
	Any PT	2 (1.1) 4	5 (2.8) 5	0 (0.0) 0	2 (1.0) 2
	Lymphadenopathy	2 (1.1) 3 NS	4 (2.2) 4 NS	0 (0.0) 0	2 (1.0) 2 NS
**Vascular disorders**					
	Any PT	0 (0.0) 0	0 (0.0) 0	0 (0.0) 0	4 (2.0) 4
	Hot flush	0 (0.0) 0	0 (0.0) 0	0 (0.0) 0	4 (2.0) 4

Fishers Exact test of comparison to Group 3; NS = Not Significant (p≥0.05);

* p< 0.05;

** p<0.01;

*** p < 0.001).

When comparing Group 1 with Group 3, fatigue, headache, myalgia and nasopharyngitis occurred slightly less frequently in the placebo group (Table 5). Comparing Group 2 with Group 3, fatigue and myalgia were similarly frequent (23.2% vs. 24.9% and 7.7% vs. 8.3%, respectively). The highest incidence of fatigue and myalgia was observed in subjects in Group 4 (35.5% and 19.5%, respectively).

Overall, there were significantly more AEs in the active treatment groups versus placebo. However, the AE profile was consistent with that of other, approved live-attenuated vaccines.

## Discussion

The primary safety objective of this study was to compare the four vaccination groups with regard to ECG changes and cardiac symptoms. Despite rigorous cardiac monitoring of all subjects enrolled in this trial, no cardiac safety signals including cardiac enzyme elevations such as troponin I were detected, reported neither as cardiac symptoms nor as abnormal ECG recordings. Not a single case of myo-/pericarditis was reported following vaccination with MVA, in contrast to the high incidence of myo-/pericarditis observed with Dryvax (10.38 events per thousand vaccinations) and ACAM2000 (5.73 events per thousand immunizations)[20].

Screening revealed an unexpectedly high rate of “abnormal” ECGs in this young healthy population. Of the 543 subjects that failed screening, 139 could not be enrolled due to abnormal ECG findings. Furthermore, routine ECGs performed at visits following the vaccinations also resulted in large numbers of falsely abnormal findings. Extensive cardiac work-up did not reveal any true cardiac condition in all cases. Therefore, it seems that normal variations, rather than cardiac abnormalities were actually being measured. Even in young and healthy individuals, ECG screening frequently reveals abnormalities such as right bundle branch block, fascicular block, first-degree AV blocks or non-specific T-wave abnormalities[21]. When performing routine measurements of ECGs in presumably healthy subjects, one therefore has to consider a potentially high level of background noise [22,23]. This questions whether an ECG represents an appropriate criterion to determine subject eligibility for clinical trials, particularly in healthy young populations. In addition, control ECGs performed throughout ongoing studies without accompanying cardiac symptoms or other clinical signs can lead to the necessity of communicating (falsely) abnormal ECG findings to the volunteers, as was the case in this study. This inevitably leads to anxiety as well as time- and resource-consuming cardiac work up for ruling out any true cardiac condition. Particularly in vaccine studies, which generally by default enroll only healthy volunteers, the often confounding and misleading results of routine ECG monitoring seem to outweigh the benefits. Exceptions are of course vaccines known to cause cardiac complications, such as replication-competent smallpox vaccines [15–17]. For the non-replicating MVA vaccine no confirmed cases of severe cardiac events have been reported in the clinical development program[24–31].

Similar to performing ECGs without a clinical indication, however, the value of routine troponin I measurements in the absence of cardiac symptoms has also been questioned. There are reports that many factors can lead to falsely positive troponin values [32–34]. Furthermore, it is known that isolated elevated troponin I does not necessarily correlate with cardiac damage [35]. Therefore, routine troponin I measurements within the context of a clinical trial have to be regarded as a screening parameter only, as any observed abnormality requires a thorough cardiologist workup to confirm a clinically significant cardiac finding.

Secondary safety objectives of this study were comparisons with regard to overall safety of the four vaccination groups and specifically of Groups 1 and 2 with the placebo group (Group 3) in vaccinia-naive subjects. Commonly observed AEs in this study were local symptoms (pain, erythema, induration and swelling) at the injection site and general symptoms, such as fatigue, headache and myalgia. The significantly lower frequency of local symptoms in the placebo group was expected as these AE are known to be associated with injectable vaccines such as MVA. By far the majority of local symptoms were mild or moderate. Only 3.3% or less of the subjects in any group had Grade 3 local reactions and no Grade 4 events were reported.

None of the SAE historically associated with replicating smallpox vaccines, such as Dryvax or ACAM2000 [7,11–13,36], were reported in this study. Thus, the large amount of safety data, including extensive cardiac monitoring results, collected from the 745 subjects who completed this study confirm the excellent safety and tolerability profile of MVA. The results revealed no safety concerns in this healthy study population, particularly with regard to the development of cardiac events following vaccination with MVA. A Phase III clinical trial evaluating the cardiac safety of MVA in 3,000 subjects compared to placebo is currently ongoing and expected to provide further confirmative data in this regard.

## Supporting Information

S1 CONSORT Checklist CONSORT Checklist. (DOC)

S1 Protocol Trial protocol. (PDF)

S1 Data Full clinical trial report incl. tables, figures and listings. (ZIP)
