# Supplementary material for: Cardiac Safety of Modified Vaccinia Ankara for Vaccination against Smallpox in a Young, Healthy Study Population
Source: PLoS One. 2015 Apr 16;10(4):e0122653. doi: 10.1371/journal.pone.0122653 (PMC4399887; doi:10.1371/journal.pone.0122653)
Supplement: S1 Data — (ZIP) [file POX-MVA-005_Final clinical study report Sections 1-16.3_21Dec2007.pdf › POX-MVA-005_Final clinical study report Sections 1-16.3_21Dec2007.pdf]

Clinical Study Report

POX-MVA-005

EudraCT number: 2005-001781-14

**Study title:**	A partially randomized, partially double-blind, placebo-controlled Phase II non-inferiority study to evaluate immunogenicity and safety of one and two doses of MVA-BN® (IMVAMUNE™) smallpox vaccine in 18-55 year old healthy subjects
**Protocol code:**	POX-MVA-005, (HCR: 1029/BN)
**Sponsor:**	Bavarian Nordic A/S<br>Hejreskovvej 10A, 3490 Kvistgård, Denmark
**Study vaccine:**	MVA-BN® (Modified Vaccinia virus Ankara – Bavarian Nordic®)
**Study indication:**	Immunogenicity and safety of a third generation smallpox vaccine MVA-BN® (IMVAMUNE™)
**Medical indication:**	Smallpox prevention
**Phase:**	II
**Principal investigator:**	Prof. Dr. Frank von Sonnenburg<br>Section of International Medicine & Public Health<br>Department of Infectious Diseases and Tropical Medicine<br>University of Munich, Georgenstrasse 5, 80799 Munich, Germany
**Initiation:**	27 April 2006, enrolment of first subject
**Completion:**	27 August 2007, last subject completed follow-up visit
**GCP statement:**	This study was performed in compliance with current Good Clinical Practice (cGCP), the Declaration of Helsinki (with amendments) and local legal and regulatory requirements.
**Archiving:**	The study documents will be archived according to ICH-GCP regulations.
**Date of report:**	19 December 2007
**Earlier reports:**	Clinical Study Safety Report from 12 July 2007

1 SIGNATURES

I have read this clinical study report and confirm that to the best of my knowledge it accurately describes the conduct and results of the clinical study as outlined in the protocol. The study was performed according to the Good Clinical Practice guidelines in operation at the time of the initiation of the study.

Prof. Frank von Sonnenburg, M.D.<br>Principal Investigator<br>University of Munich	Dec-21-2007<br>Date / Signature
Alfred von Krempelhuber, Ph.D.<br>Project Coordinator<br>Bavarian Nordic GmbH	Date / Signature
Garth Virgin, M.D.<br>Medical Monitor<br>Bavarian Nordic GmbH	Date / Signature
Paul Chaplin, Ph.D.<br>Chief Scientific Officer<br>Bavarian Nordic GmbH	Date / Signature
Robert Semich, Ph.D.<br>GCP Specialist<br>Bavarian Nordic GmbH	Date / Signature

1 SIGNATURES

I have read this clinical study report and confirm that to the best of my knowledge it accurately describes the conduct and results of the clinical study as outlined in the protocol. The study was performed according to the Good Clinical Practice guidelines in operation at the time of the initiation of the study.

Prof. Frank von Sonnenburg, M.D.<br>Principal Investigator<br>University of Munich	Date / Signature
Alfred von Krempelhuber, Ph.D.<br>Project Coordinator<br>Bavarian Nordic GmbH	21-Dec-2007 [signature]<br>Date / Signature
Garth Virgin, M.D.<br>Medical Monitor<br>Bavarian Nordic GmbH	21-Dec-2007 Garth Virgin<br>Date / Signature
Paul Chaplin, Ph.D.<br>Chief Scientific Officer<br>Bavarian Nordic GmbH	21-Dec-2007 [signature]<br>Date / Signature
Robert Semich, Ph.D.<br>GCP Specialist<br>Bavarian Nordic GmbH	21-Dec-2007 Robert Semich<br>Date / Signature

SIGNATURES (continued)

I have read this clinical study report and confirm that to the best of my knowledge it accurately describes the conduct and results of the clinical study as outlined in the protocol. The study was performed according to the Good Clinical Practice guidelines in operation at the time of the initiation of the study.

Stephan de la Motte, M.D.
Investigator
Harrison Clinical Research GmbH

20 DEC 2007, [signature]
Date / Signature

Marianne Sievers
Project Manager
Harrison Clinical Research GmbH

Date / Signature

Joachim Klinger
Director Data Management and Statistics
Harrison Clinical Research GmbH

20 Dec 07 J. Klinger
Date / Signature

Caterina Milazzo, Ph.D.
Medical Writer
Harrison Clinical Research GmbH

20 DEC 07 C. Milazzo
Date / Signature

[*] Stephan de la Motte, M.D.
Investigator
Harrison Clinical Research GmbH

Date / Signature

Marianne Sievers
Project Manager
Harrison Clinical Research GmbH

20.12.07 [signature]
Date / Signature

Joachim Klinger
Director Data Management and Statistics
Harrison Clinical Research GmbH

Date / Signature

Caterina Milazzo, Ph.D.
Medical Writer
Harrison Clinical Research GmbH

Date / Signature

0049810631578

19-DEZ-07 14:02 Sievers 0049810631578 SEITE: 1

2 SYNOPSIS

**Name of Sponsor/Company:** Bavarian Nordic A/S	**Individual Study Table Referring to Part of the Dossier**	(For National Authority Use Only)
**Name of Finished Product:** IMVAMUNE®	Volume:	
**Name of Active Ingredient(s):** Modified Vaccinia virus Ankara	Page:	

Title of the study:

A partially randomized, partially double-blind, placebo-controlled Phase II non-inferiority study to evaluate immunogenicity and safety of one and two doses of MVA-BN® (IMVAMUNE™) smallpox vaccine in 18-55 year old healthy subjects

Investigators:

Principal Investigator:	Prof. Frank von Sonnenburg, Institute for Tropical Medicine, University of Munich
Investigator:	Dr. S. de la Motte, Harrison Clinical Research GmbH

Study centre(s):

Clinical Unit: Harrison Clinical Research GmbH, Albrechtstr. 43, 80636 Munich, Germany

Publication:

None at the date of this report

**Study period:**	**Phase of Development:**
27 April 2006 (first enrolment)<br>27 August 2007 (last subject completed follow-up visit)	Phase II

Objectives:

The primary immunogenicity objective of the trial was:

- To compare the ELISA specific humoral immune response of subjects with a history of smallpox vaccination (Group 4: 1 dose MVA-BN®) to subjects without a history of smallpox vaccination (Group 1: 2 doses MVA-BN®), i.e. to prove that a single dose schedule would be sufficient to boost the immunity in a previously vaccinated population.

The primary safety objective of the trial was:

- To compare the four different vaccination groups with regard to ECG changes and cardiac symptoms.

The secondary objectives of the trial were:

- To compare the ELISA specific immune response of the four different vaccination groups - comparison combinations not performed for the primary objective.
- To compare the four different vaccination groups with regard to immune response measured with a plaque reduction neutralization test (PRNT) assay.
- To assess the kinetics of the immune responses after 1 (Group 2) and 2 (Group 1) doses of MVA-BN® in vaccinia-naïve subjects.
- To compare the four different vaccination groups with regard to safety and reactogenicity.
- To compare the safety of one (Group 2) and two (Group 1) vaccinations with MVA-BN® in vaccinia-naïve subjects with placebo (Group 3) and historical / recently published data of Dryvax® immunization in vaccinia-naive subjects.

**Name of Sponsor/Company:** Bavarian Nordic A/S	**Individual Study Table Referring to Part of the Dossier**	(For National Authority Use Only)
**Name of Finished Product:** IMVAMUNE®	Volume:	
**Name of Active Ingredient(s):** Modified Vaccinia virus Ankara	Page:	

Methodology:

This study was a partially randomized, partially double-blind, placebo-controlled, phase II non-inferiority trial to evaluate immunogenicity and safety of one and two doses of MVA-BN® smallpox vaccine in 18-55 year old healthy subjects.

In this study subjects in the following four groups received one (Group 4) or two injections with either MVA-BN® or placebo administered at an interval of 4 weeks (28 – 35 days) (Groups 1-3):

Group		1. Vaccination	2. Vaccination
1	Subjects without history of smallpox vaccination	MVA-BN®	MVA-BN®
2		MVA-BN®	Placebo
3		Placebo	Placebo
4	Subjects with history of smallpox vaccination	MVA-BN®	-

Subjects in Groups 1-3 were treated under double-blind conditions while treatment in Group 4 was open-label. Subjects eligible for Groups 1, 2 and 3 were randomly allocated to one of these three treatment groups in order of their appearance. The randomization list was provided by Kendle GmbH. At the investigational site, only the study independent person preparing the study medication had access to the randomization list.

An overview of the assessment schedule and study procedures is given in the Flow Chart in Table 9-1.

Safety laboratory blood samples were taken and a standard 12-lead ECG was recorded at the screening visit and 10-15 days after each vaccination (Visits 2 and 4 for Groups 1-3 and Visit 2 for Group 4). At Visit 5 (Groups 1-3) and Visit 3 (Group 4) as well as at the 6-month follow-up visit safety laboratory and ECG were only performed if clinically indicated.

To analyze the immune response, a blood sample was taken during Visits 1-5 (Groups 1-3) and Visits 1-3 (Group 4) as well as at the follow-up visit. Antibody responses against MVA-BN® were measured using validated ELISA and PRNT assays.

Number of subjects:

800 subjects; changed according to Amendment no. 1 to a total of 740 subjects (200 subjects in Group 4 and 180 subjects each in Groups 1, 2 and 3).
Vaccinated: 745 subjects received at least one dose of the study vaccine.

Diagnosis and main criteria for inclusion:

Healthy male and female subjects between 18 and 55 years of age with no abnormal ECG findings and no active or history of cardiac disease.
Groups 1-3: Subjects with no history of known or suspected previous smallpox vaccination and no detectable vaccinia scar.
Group 4: Subjects with a history of previous smallpox vaccination (written documentation and/or typical vaccinia scar), most recent smallpox vaccination ≥ 5 years ago.

Name of Sponsor/Company: Bavarian Nordic A/S	Individual Study Table Referring to Part of the Dossier	(For National Authority Use Only)
**Name of Finished Product:** IMVAMUNE®	Volume:	
**Name of Active Ingredient(s):** Modified Vaccinia virus Ankara	Page:	

Test product, dose and mode of administration, batch number:

MVA-BN®, 1 x 10^8 $TCID_{50}$ (Tissue Culture Infectious Dose 50) per 0.5 mL and dose, administered subcutaneously in the non-dominant upper arm
Batch no.: 170505

Duration of study:

Groups 1/2/3: Up to 39 weeks for each subject
Group 4: Up to 34 weeks for each subject

Reference therapy, dose and mode of administration, batch number:

Tris buffer (placebo), 0.5 mL per dose, administered subcutaneously in the non-dominant upper arm
Batch no.: 030505

Criteria for evaluation:

Immunogenicity:

- Vaccinia-specific seroconversion rate derived from the ELISA specific antibody titers
- Vaccinia-specific seroconversion rate derived from the PRNT specific antibody titers

Safety:

- Occurrence, relationship and intensity of any specific or unspecific ECG change and any other cardiac symptom at any time during the study.
- Occurrence, intensity, duration and relationship of adverse events

Statistical methods:

Primary endpoints:

Immunogenicity:
Vaccinia-specific seroconversion rate derived from the ELISA specific antibody titers 2 weeks after the last vaccination (Groups 1-3: Visit 4, Group 4: Visit 2). Seroconversion as the primary immunogenicity variable was defined as an antibody titer of ≥ 50 in subjects who were seronegative at baseline, or a two-fold increase of the antibody titer compared to the pre-existing titer in subjects showing an antibody response against vaccinia at baseline.

Safety:
- Occurrence, relationship and intensity of any specific or unspecific ECG change at any time during the study.
- Occurrence, relationship and intensity of any other cardiac symptom at any time during the study.

Secondary endpoints:

Immunogenicity:
- Vaccinia-specific seroconversion rate derived from the ELISA specific antibody titers 4 weeks after the last vaccination (Groups 1-3: Visit 5, Group 4: Visit 3).
- Vaccinia-specific seroconversion rate derived from the PRNT specific antibody titers 2

Name of Sponsor/Company: Bavarian Nordic A/S	Individual Study Table Referring to Part of the Dossier	(For National Authority Use Only)
Name of Finished Product: IMVAMUNE®	Volume:	
Name of Active Ingredient(s): Modified Vaccinia virus Ankara	Page:	

weeks after the last vaccination (Groups 1-3: Visit 4, Group 4: Visit 2) and 4 weeks after the last vaccination (Groups 1-3: Visit 5, Group 4: Visit 3).

Safety:

- Occurrence of any serious adverse event possibly, probably or definitely related to the study vaccine at any time during the study.
- Occurrence of any Grade 3 or higher adverse reaction possibly, probably or definitely related to the study vaccine within 28 days after vaccination.
- Occurrence of solicited local adverse events within 1 week (Days 0-7) after vaccination: Intensity, duration, and relationship to vaccination.
- Occurrence of solicited general adverse events within 1 week (Days 0-7) after vaccination: Intensity, duration, and relationship to vaccination.
- Occurrence of unsolicited non-serious adverse events within 28 days after vaccination: Intensity, duration and relationship to vaccination.

SUMMARY – CONCLUSIONS

IMMUNOGENICITY

Based on antibody titer analysis by ELISA it was shown in this study that a single dose of MVA-BN® injected in vaccinia-experienced subjects was sufficient to elicit seroconversion in 95.5% of the subjects measured two weeks after a single vaccination (Group 4). This was only slightly lower than the seroconversion rate observed in vaccinia-naïve subjects (98.9%) measured four weeks after the second vaccination (Group 1). Statistically these seroconversion rates were not significantly different; however, at four weeks after the last vaccination a significant difference was observed in the seroconversion rates between Group 1 (98.9%) and Group 4 (93.0%).

The primary hypothesis of the seroconversion rate in Group 4 being non-inferior to the seroconversion rate in Group 1 could not be confirmed at two, as well as at four weeks after the last vaccination. This was essentially because the criteria of seroconversion was set too strict and in retrospect had little biological relevance as to the evaluation of the immunogenicity of MVA-BN®. Indeed, many of the non-seroconverters in Group 4 actually met the criteria at later time points, while subjects with high starting titers failed to meet the 2-fold increase, although a boost in the total antibody response to vaccinia was observed in all, but two subjects in Group 4 following the single vaccination with MVA-BN®. Indeed, although the lower seroconversion rate in Group 4 compared to Group 1 implied a lower relative change in antibody titers in Group 4, the vaccinia-specific peak GMT as measured by ELISA was in fact higher in the Group 4 subjects. A peak GMT of 568.819 in Group 4 was measured two weeks after the MVA-BN® vaccination (Visit 2) and a GMT of 495.843 in Group 1 two weeks after the second MVA-BN® vaccination (Visit 4). Therefore, the results of the study revealed that following two vaccinations with MVA-BN® strong antibody responses were induced in the majority of the vaccinia naïve subjects and these responses were comparable to those generated in vaccinia-experienced subjects that received a single booster vaccination.

In comparison, a single vaccination with MVA-BN® followed by a placebo injection in subjects who had no history of smallpox vaccination (Group 2) resulted in a seroconversion in 87.4% of the

**Name of Sponsor/Company:** Bavarian Nordic A/S	**Individual Study Table Referring to Part of the Dossier**	(For National Authority Use Only)
**Name of Finished Product:** IMVAMUNE®	Volume:	
**Name of Active Ingredient(s):** Modified Vaccinia virus Ankara	Page:	

subjects at four weeks after the MVA-BN® vaccination (87.4% was the highest seroconversion rate measured in Group 2).

Similarly, only 78.5% of the vaccinia-experienced subjects seroconverted by PRNT, 2 weeks post vaccination with MVA-BN®. However, out of the 43 non-seroconverters (2-fold increase) in Group 4, 19 would actually have fulfilled the criteria at later visits; clearly indicating that MVA-BN® vaccination induced a strong booster response to the neutralizing antibodies in this population. Seroconversion rates were actually higher in the vaccinia naïve subjects (Group 1) after the second vaccination, which again may be accounted by the strict criteria set for seroconversion particularly for the vaccinia-experienced subjects. Indeed, this was again highlighted by comparing the GMT generated by PRNT. The GMT in Group 1, 2 weeks post the second vaccination with MVA-BN® was 45.577, compared to 175.150 two weeks post MVA-BN® vaccination in vaccinia-experienced subjects (Group 4).

Approx. six months after the second vaccination the GMT derived from ELISA and PRNT in the subjects who received two doses of MVA-BN® (Group 1) was still at 27.900 and 7.236 respectively, which are in the same order of magnitude at the antibody levels recorded in vaccinia-experienced subjects at baseline. In these subjects re-exposure to the vaccinia virus might induce a similar booster effect as observed in the subjects in Group 4. In Group 4, the GMT by both ELISA and PRNT at the follow-up visit (179.907 and 106.463 respectively) were still markedly higher than the baseline titers.

Importantly, when all outliers were taken into account the ELISA and PRNT titers were highly correlated at all visits for all groups. In general the GMT and seroconversion rates were lower by PRNT compared to ELISA and this is probably related to the fact the PRNT is only measuring a component of the total antibody responses measured by the ELISA. Moreover, the ELISA represents a more sensitive assay compared to the PRNT.

In this study, a limited ability to boost pre-existing baseline titers above a certain value was observed. This leads to the assumption that the criteria for seroconversion as defined for the vaccinia-experienced population may have been too strict and a two-fold increase in titers does not seem to be an appropriate measure of immunity. Therefore, the inability to prove non-inferiority between seroconversion rates in vaccinia-naïve compared to vaccinia-experienced subjects does not seem to have any clinical relevance with regard to the clearly demonstrated immunogenicity of MVA-BN®.

SAFETY

There were no deaths reported throughout the entire study (up to Visit 5).

A total of 3 serious adverse events were reported during the active phase of this study, in two subjects from Group 2 (Subjects 536 and 706) and in one subject from Group 1 (Subject 148). One SAE, a ruptured tendon of the finger, was considered not related to the study vaccine and is still ongoing. The other two SAEs were nervous breakdown and transient motoric hemiparesis, from which the two subjects completely recovered. Both events were considered unlikely to be related

Name of Sponsor/Company: Bavarian Nordic A/S	Individual Study Table Referring to Part of the Dossier	(For National Authority Use Only)
**Name of Finished Product:** IMVAMUNE®	Volume:	
**Name of Active Ingredient(s):** Modified Vaccinia virus Ankara	Page:	

to the study vaccine.

During follow-up (6 months-period after the last vaccination) nine SAEs were reported in 9 subjects, of which only one SAE was considered to be possibly related to the study vaccine: ongoing sarcoidosis in subject 521 in Group 1. Two SAEs were judged to be unlikely related to the study treatment and consisted of colon carcinoma (Subject 685, Group 3) and depression (Subject 751, Group 3). Six SAEs were assessed to be unrelated: tonsillectomy, pregnancy, salmonella enteritis, thyroidectomy, lower limb fracture, and tendon rupture.

The primary safety objective of this study was to compare the four different vaccination groups with regard to ECG changes and cardiac symptoms. All ECG recordings were considered normal or represented normal variations as assessed by the investigator and/or the local cardiologist. Neither significant QTc prolongations nor changes in PR, QRS and/or RR intervals resulting from vaccination were identified.

Secondary safety objectives of this study compared the safety of the four different vaccination groups and specifically of Groups 1 and 2 with the placebo group (Group 3) as well as with historical data following Dryvax® immunization in vaccinia-naïve subjects.

Comparing Group 1 with the placebo group, a clearly higher frequency of common local injection site reactions was observed in Group 1, indicating a relationship to MVA-BN®. Very frequent local AEs were pain, erythema, induration and swelling. No clear relationship to MVA-BN® could be determined for general symptoms, such as fatigue, headache and myalgia. Headache and myalgia were most frequent in vaccinia-experienced subjects in Group 4.

After the second vaccination with MVA-BN® in Group 1, local Grade 2 and Grade 3 erythema, swelling and induration tended to be more frequent than after the first vaccination. Comparing vaccinia-naïve subjects (Groups 1 and 2) with vaccinia-experienced subjects (Group 4) after the first vaccination, overall frequencies of local solicited symptoms were similar, however, there was a tendency of an increased frequency of Grade 2 and Grade 3 erythema and induration in Group 4. After the first vaccination swelling was overall more frequent in Group 4, as compared to Groups 1 and 2.

After the second injection (MVA-BN® in Group 1 and placebo in Group 2) there were no major differences in solicited general AEs between Groups 1 and 2.

There were no treatment-related clinically significant abnormalities observed in the clinical laboratory results, vital signs and physical examinations in this study.

As compared to the unexpectedly high incidence of myo-/pericarditis following Dryvax® immunizations, in this study not a single case of myo-/pericarditis was reported.

Thus, the large amount of safety data, including extensive cardiac monitoring results, collected from the 745 subjects who completed this study confirm the excellent safety and tolerability profile of MVA-BN® and revealed no particular safety concerns in this healthy study population, also with regard to the development of cardiac events following vaccination with MVA-BN®.

Date of the report: 19 December 2007

3 TABLE OF CONTENTS

PAGE

1 SIGNATURES 2

2 SYNOPSIS 4

3 TABLE OF CONTENTS 10

4 LIST OF ABBREVIATIONS AND DEFINITION OF TERMS 15

5 ETHICS 17

5.1 INDEPENDENT ETHICS COMMITTEE (IEC) OR INSTITUTIONAL REVIEW BOARD (IRB) 17
5.2 ETHICAL CONDUCT OF THE STUDY 17
5.3 SUBJECT INFORMATION AND CONSENT 17

6 INVESTIGATIONS AND STUDY ADMINISTRATIVE STRUCTURE 18

7 INTRODUCTION 20

8 STUDY OBJECTIVES 21

9 INVESTIGATIONAL PLAN 22

9.1 OVERALL STUDY DESIGN AND PLAN 22
9.2 DISCUSSION OF STUDY DESIGN AND THE CHOICE OF CONTROL GROUPS 24
9.3 SELECTION OF STUDY POPULATION 25
9.3.1 INCLUSION CRITERIA 25
9.3.2 EXCLUSION CRITERIA 26
9.3.3 REMOVAL OF SUBJECTS FROM THERAPY OR ASSESSMENT 27
9.4 TREATMENTS 28
9.4.1 TREATMENTS ADMINISTERED 28
9.4.2 IDENTITY OF INVESTIGATIONAL PRODUCT(S) 28
9.4.3 METHOD OF ASSIGNING PATIENTS TO TREATMENT GROUPS 29
9.4.4 SELECTION OF DOSES IN THE STUDY 29
9.4.5 SELECTION AND TIMING OF DOSE FOR EACH PATIENT 29
9.4.6 BLINDING 29
9.4.7 PRIOR AND CONCOMITANT THERAPY 30
9.4.8 TREATMENT COMPLIANCE 30
9.5 EFFICACY AND SAFETY VARIABLES 30
9.5.1 IMMUNOLOGY MEASUREMENTS ASSESSED 30
9.5.2 SAFETY MEASUREMENTS ASSESSED 30
9.6 DATA QUALITY ASSURANCE 31
9.7 STATISTICAL METHODS PLANNED IN THE PROTOCOL AND DETERMINATION OF SAMPLE SIZE 32
9.7.1 STATISTICAL AND ANALYSIS PLAN 32
9.7.2 DETERMINATION OF SAMPLE SIZE 36
9.8 CHANGES IN THE CONDUCT OF THE STUDY OR PLANNED ANALYSES 36

10 STUDY SUBJECTS 38

10.1 DISPOSITION OF SUBJECTS 38

10.2 PROTOCOL DEVIATIONS 40

11 IMMUNOGENICITY EVALUATION 41

11.1 DATASETS ANALYSED....41
11.2 DEMOGRAPHIC AND OTHER BASELINE CHARACTERISTICS 41
11.3 MEASUREMENTS OF TREATMENT COMPLIANCE....42
11.4 IMMUNOGENICITY RESULTS AND TABULATIONS OF INDIVIDUAL SUBJECT DATA 42
11.4.1 ANALYSIS OF IMMUNOGENICITY 42
11.4.2 STATISTICAL/ANALYTICAL ISSUES 58
11.4.3 TABULATION OF INDIVIDUAL RESPONSE DATA 59
11.4.4 DRUG DOSE, DRUG CONCENTRATION RELATIONSHIPS TO RESPONSE....59
11.4.5 DRUG-DRUG AND DRUG-DISEASE INTERACTIONS 59
11.4.6 BY-PATIENT DISPLAYS 59
11.4.7 EFFICACY CONCLUSIONS 59

12 SAFETY EVALUATION 62

12.1 EXTENT OF EXPOSURE 62
12.2 ADVERSE EVENTS (AEs) 62
12.2.1 BRIEF SUMMARY OF ADVERSE EVENTS....62
12.2.2 DISPLAY OF ADVERSE EVENTS....68
12.2.3 ANALYSIS OF ADVERSE EVENTS....75
12.2.4 LISTING OF ADVERSE EVENTS BY SUBJECTS....77
12.3 DEATHS, OTHER SERIOUS ADVERSE EVENTS, AND ADVERSE EVENTS OF SPECIAL INTEREST....78
12.3.1 LISTING OF DEATHS, OTHER SERIOUS ADVERSE EVENTS AND ADVERSE EVENTS OF SPECIAL INTEREST 78
12.3.2 NARRATIVES OF DEATHS, OTHER SERIOUS ADVERSE EVENTS AND ADVERSE EVENTS OF SPECIAL INTEREST 81
12.3.3 ANALYSIS AND DISCUSSION OF DEATHS, OTHER SERIOUS ADVERSE EVENTS AND CERTAIN OTHER SIGNIFICANT ADVERSE EVENTS....85
12.4 CLINICAL LABORATORY EVALUATION....86
12.4.1 LISTING OF INDIVIDUAL LABORATORY MEASUREMENTS BY SUBJECT (16.2.8) AND EACH ABNORMAL LABORATORY VALUE (14.3.4)....86
12.4.2 EVALUATION OF EACH LABORATORY PARAMETER 86
12.5 VITAL SIGNS, PHYSICAL FINDINGS AND OTHER OBSERVATIONS RELATED TO SAFETY 86
12.6 ECG ANALYSIS....87
12.6.1 PR, QRS, QT AND RR INTERVALS 87
12.6.2 QTC INTERVAL ANALYSIS....87
12.7 COMPARISON WITH SAFETY DATA OF DRYVAX® IMMUNIZATION 90
12.8 SAFETY CONCLUSIONS....91

13 DISCUSSION AND OVERALL CONCLUSIONS 92

The detailed Table of Contents for the following sections can be found in the respective section.

**14**	**TABLES, FIGURES AND GRAPHS REFERRED TO BUT NOT INCLUDED IN THE TEXT**	PAGE
14.1	DEMOGRAPHIC DATA	5
14.2	IMMUNOGENICITY DATA	19
14.3	LABORATORY DATA	83
14.4	ECG DATA	209
14.5	ADVERSE EVENT DATA	237
14.6	FURTHER SAFETY DATA	312
14.7	ADDITIONAL EXPLORATORY SUBGROUP ANALYSIS BY AGE GROUP AND GENDER	339

		Page(s) total
**15**	**REFERENCES**	2
**16**	**APPENDICES**	
16.1	STUDY INFORMATION	573
16.2	SUBJECT DATA LISTINGS	5989
16.3	REPORT FORMS FOR SERIOUS ADVERSE EVENTS, ADVERSE EVENTS OF SPECIAL INTEREST AND PREGNANCIES	128

TABLE OF IN-TEXT TABLES

Table 9-1 Flow Chart 23
Table 10-1 Violations against visit windows 40
Table 11-1 Primary efficacy parameter: Vaccinia-specific seroconversion rate derived from the ELISA specific antibody titers 2 weeks after the last vaccination (Group 1-3: Visit 4, Group 4: Visit 2) 47
Table 11-2 Secondary efficacy parameter: Vaccinia-specific seroconversion rate derived from the ELISA specific antibody titers 4 weeks after the last vaccination (Group 1 3: Visit 5, Group 4: Visit 3) 48
Table 11-3 Group 4 subjects in the FA dataset not meeting the criterion of seroconversion at Visit 249
Table 11-4 Primary efficacy parameter: Vaccinia-specific seroconversion rate derived from the PRNT specific antibody titers 2 weeks after the last vaccination (Group 1-3: Visit 4, Group 4: Visit 2) 54
Table 11-5 Secondary efficacy parameter: Vaccinia-specific seroconversion rate derived from the PRNT specific antibody titers 4 weeks after the last vaccination (Group 1 3: Visit 5, Group 4: Visit 3) 54
Table 11-6 Subjects in Group 4 (FA dataset) not meeting the criterion of seroconversion at Visit 2..55
Table 11-7 Subjects in Group 1 (FA dataset) not meeting the criterion of seroconversion at Visit 4..57
Table 11-8 Correlation analysis between ELISA and PRNT antibody titers 58
Table 12-1 Unsolicited Adverse Events: Overview, Safety dataset, active study phase 64
Table 12-2 Summary of Maximum Intensity of Solicited General Symptoms (7-day Follow-up Period after Vaccination) as Recorded in the Subject Diary, Safety dataset 66
Table 12-3 Summary of Maximum Intensity of Solicited Local Symptoms (7-day Follow-up Period after Vaccination) as Recorded in the Subject Diary, Safety dataset 67
Table 12-4 Unsolicited Adverse Events: MedDRA coding by SOC and subject, Safety dataset 69
Table 12-5 Solicited (General / Local) and Unsolicited Adverse Events Assessed as Being Possibly/Probably/Definitely Related to Study Vaccine: MedDRA Coding by SOC, PT, Safety dataset 71
Table 12-6 Deaths, other serious adverse events and adverse events of special interest: 80
Table 12-7 Absolute QTc values - threshold <=/> 450ms (Fridericia´s correction) 88
Table 12-8 Absolute QTc values (Fridericia´s correction) 89

TABLE OF IN-TEXT FIGURES

Figure 10-1 Disposition of subjects 39
Figure 11-1 Vaccinia-specific antibody response, measured by ELISA; FA dataset 44
Figure 11-2 Vaccinia-specific antibody response measured by ELISA /Time response; FA dataset..44
Figure 11-3 Seroconversion rate, measured by ELISA, FA dataset 45
Figure 11-4 Seroconversion rate, measured by ELISA, two and four weeks after last vaccination, FA dataset 46
Figure 11-5 Vaccinia-specific antibody response, measured by PRNT; FA dataset 50
Figure 11-6 Vaccinia-specific antibody response measured by PRNT /Time response; FA dataset....51
Figure 11-7 Seroconversion rate, measured by PRNT, FA dataset 52
Figure 11-8 Seroconversion rate, measured by PRNT, two and four weeks after last vaccination, FA dataset 53
Figure 12-1 Frequencies of solicited and unsolicited treatment-related adverse events in the safety dataset with a statistically significant difference between any of the four groups as determined by a p-value < 0.05. 77

4 LIST OF ABBREVIATIONS AND DEFINITION OF TERMS

AE	Adverse event
ALT	Alanine aminotransferase
ANC	Absolute neutrophil count
ANOVA	Analysis Of Variance
AP	Alkaline phosphatase
AST	Aspartate aminotransferase
BCG	Bacillus of Calmette and Guerin
BMI	Body mass index
BN	Bavarian Nordic
BPM	Beats per minute
CA	Competent authority
CK	Creatine (phospho-)kinase
CK-MB	Creatine (phospho-)kinase - muscle brain
CRF(s)	Case Report Form(s)
eCRF(s)	Electronic case report form(s)
CRO	Contract research organization
DHHS	Department of Health and Human Services
DMID	Division of Microbiology and Infectious Diseases, National Institute of Allergy and Infectious Diseases
DSMB	Data Safety Monitoring Board
ECG	Electro cardiogram
ELISA	Enzyme-linked Immunosorbent Assay
FA	Full Analysis
FU	Follow-up
GCP	Good clinical practice
GMT	Geometric Mean Titer
HCR	Harrison Clinical Research
HDL	High density lipoprotein
HIV	Human immunodeficiency virus
ICH	International Conference of Harmonization of Technical Requirements for Registration of Pharmaceuticals for Human Use
IEC	Independent Ethics Committee
IRB	Institutional Review Board
LDL	Low density lipoprotein

LLN	Lower limit of normal
LMU	Ludwig Maximilian University of Munich
MedDRA	Medical Dictionary for Regulatory Activities
MVA	Modified Vaccinia Ankara
MVA-BN®	Modified Vaccinia virus Ankara – Bavarian Nordic
N/n	Number
NYCBH	New York City Board of Health
PP	Per protocol<br>Or in *table headers* Placebo Placebo (Group 3, receiving 2 doses of placebo)
PRNT	Plaque reduction neutralization test
PT	Preferred term
PVC	Premature ventricular contractions
QTc_B	QT correction according to Bazett
QTc_F	QT correction according to Fridericia
SAE	Serious adverse event
s.c.	Subcutaneous
SCR	Screening
SGOT	Serum glutamic oxaloacetic transaminase
SGPT	Serum glutamic pyruvic transaminase
SIAE	Adverse event of special interest
SMC	Safety Monitoring Committee
ST	ST segment in electrocardiogram
SOC	System organ class
$TCID_{50}$	Tissue culture infectious dose 50
ULN	Upper limit of normal
V	Visit
WBC	White blood cell count
WHO	World Health Organization

5 ETHICS

5.1 INDEPENDENT ETHICS COMMITTEE (IEC) OR INSTITUTIONAL REVIEW BOARD (IRB)

The clinical trial authorization by the competent authority (CA) for this trial (Paul-Ehrlich-Institut, Federal Agency for Sera and Vaccines, Langen, Germany) and a favorable opinion from the Ethics Committee of the Medical Faculty of the Ludwig-Maximilians University, Munich, Germany were obtained prior to the start of the study. The local authorities were also notified about the study as required by law.

The addresses of the IEC and regulatory authorities consulted and informed are provided in Section 16.1.3 of this report.

5.2 ETHICAL CONDUCT OF THE STUDY

This study was conducted in accordance with the ethical principles set forth in the Declaration of Helsinki.

5.3 SUBJECT INFORMATION AND CONSENT

Written informed consent was obtained from all subjects prior to entry into the study. The investigator explained to each subject, orally and in writing (subject information sheet), the nature, significance, risks and implications of the trial.

A copy of the subject information sheet and the informed consent form are included in Section 16.1.3.

6 INVESTIGATIONS AND STUDY ADMINISTRATIVE STRUCTURE

Bavarian Nordic was the sponsor of this study. Harrison Clinical Research GmbH (HCR), a contract research organization (CRO), organized the performance of this study. The responsibilities were allocated as specified below:

Institution/ person	Responsibility
Sponsor	• Test product<br>• Packaging and labeling of test product<br>• Storage and transportation of test product<br>• Relevant product information<br>• Drug handling instructions<br>• Certificate of analysis<br>• Sponsor Project management<br>• Archiving of trial master file after the end of the study<br>• Notification of Ethics Committee and Competent Authority (CA) about the end of the trial<br>• Notification of Ethics Committee and CA about the occurrence of unexpected serious adverse events (AEs) and other events which require a re-evaluation of the risk/benefit ratio<br>• Annual (or on-demand) reporting of serious AEs to CA<br>• Submission of final study report to ethics committee and CA<br>• Archiving of study documentation at the end of the trial<br>• Protocol<br>• Investigator's brochure<br>• Writing of subject information sheet /informed consent form
CRO	• Writing of electronic case report form (eCRF)<br>• Monitoring activities<br>• CRO project management<br>• Maintenance and storage of trial master file during the performance of the study<br>• Submission of study documentation to the relevant Ethics Committee and governmental / health authorities<br>• Quality control and quality assurance<br>• Data management and statistical evaluation<br>• Subject insurance<br>• Preparation of final study report
Investigator	• Recruitment of subjects<br>• Conduct of clinical phase<br>• Medical care of subjects, including cases of emergency<br>• Investigator file<br>• Drug accountability<br>• Documentation in eCRFs<br>• Notification of sponsor about serious adverse events and other relevant events supply of sponsor, Ethics Committee and CA with any information requested about reported deaths
Immunological laboratory	• Immune response analysis

Institution/ person	Responsibility
Analytical laboratory	• Safety laboratory measurements
Kendle GmbH	• Randomization list

A detailed list of persons and institutions involved in this study is provided in Section 16.1.4.

7 INTRODUCTION

After the World Health Organization (WHO) formally declared the global eradication of smallpox in May 1980, smallpox vaccinations were discontinued in many countries. However, recent concerns about the potential use of the highly contagious Variola virus as an agent in bioterrorism or bio-warfare initiated the development of a third generation smallpox vaccine by Bavarian Nordic (BN). Derived from Professor Anton Mayr's Modified Vaccinia virus Ankara (MVA) strain, BN developed the proprietary strain MVA–Bavarian Nordic (MVA-BN®, IMVAMUNE®).

MVA-BN® is a highly attenuated strain of the vaccinia virus that does not replicate in human cells and therefore cannot be transmitted or cause dispersed vaccinia-infection, which is a very important safety concern of conventional smallpox vaccines in at-risk populations, e.g. subjects with Human Immunodeficiency Virus (HIV) infection, atopic dermatitis, etc.

BN has performed an extensive preclinical development program that has demonstrated the safety, efficacy and bio-equivalence of MVA-BN® compared to other traditional smallpox vaccines (e.g. Dryvax®, Elstree and MVA-571).

In 8 completed and on-going phase I and phase II clinical studies in more than 1,500 subjects, MVA-BN® has shown a favorable safety profile in parallel with a strong and rapid immune response. In addition, the safety of MVA-based recombinant vaccines has been demonstrated in more than 150 subjects also including risk populations (HIV and melanoma).

There were no unexpected findings and no target organ toxicity observed with MVA-BN®. Furthermore, available data from subjects with contraindications to conventional smallpox vaccines such as patients with atopic dermatitis and HIV infected subjects, revealed no special risks of MVA-BN® administration in these populations.

8 STUDY OBJECTIVES

The primary immunogenicity objective of the trial was:

- To compare the ELISA (Enzyme-linked Immunosorbent Assay) specific humoral immune response of the group with history of smallpox vaccination (Group 4: 1 dose MVA-BN®) versus the group without history of smallpox vaccination (Group 1: 2 doses MVA-BN®), i.e. to prove that a single dose schedule would be sufficient to boost the immunity in a previously vaccinated population.

The primary safety objective of the trial was:

- To compare the four different vaccination groups with regard to ECG changes and cardiac symptoms.

The secondary objectives of the trial were:

- To compare the ELISA specific immune response of the four different vaccination groups - comparison combinations not done for the primary objective.
- To compare the four different vaccination groups with regard to immune response measured with a plaque reduction neutralization test (PRNT).
- To assess the kinetics of the immune responses after one (Group 2) and two (Group 1) doses of MVA-BN® in vaccinia-naïve subjects.
- To compare the four different vaccination groups with regard to safety and reactogenicity.
- To compare the safety of one (Group 2) and two (Group 1) vaccinations with MVA-BN® in vaccinia-naïve subjects with placebo (Group 3) and historical / recently published data of Dryvax® immunization in vaccinia-naive subjects.

9 INVESTIGATIONAL PLAN

9.1 OVERALL STUDY DESIGN AND PLAN

The following sections describe the investigational plan valid for the performance of this study. The plan was defined in the Study Protocol, dated 12 December 2005 and one Protocol Amendment (No. 1, dated 26 March 2007).

Copies of the Protocol and the Amendment are provided in Section 16.1.1.

This study was a partially randomized, partially double-blind, placebo-controlled, phase II non-inferiority trial to evaluate immunogenicity and safety of one and two doses of MVA-BN® smallpox vaccine in 18-55 year old healthy subjects.

In this study subjects in the following four groups, with or without history of smallpox vaccination, received one (Group 4) or two treatments (vaccine or placebo) administered at an interval of 4 weeks (28–35 days) apart (Groups 1-3):

Group		1. Vaccination	2. Vaccination	Abbrev. used in tables/listings
1	Subjects without history of smallpox vaccination	MVA-BN®	MVA-BN®	MM
2		MVA-BN®	Placebo	MP
3		Placebo	Placebo	PP
4	Subjects with history of smallpox vaccination	MVA-BN®	-	M-

Subjects in Groups 1-3 were treated under double-blind conditions, while treatment in Group 4 was open-label.

Safety laboratory blood samples were taken and a standard 12-lead ECG was recorded at the screening visit and 10-15 days after each vaccination (Visit 2 and 4 for Groups 1-3 and Visit 2 for Group 4). At Visit 5 (Groups 1-3) and Visit 3 (Group 4) as well as at the follow-up visit, safety laboratory and ECGs were only performed if clinically indicated.

To analyze the immune response raised against the vaccination, blood samples were taken during Visits 1-5 (Groups 1-3) and Visits 1-3 (Group 4) as well as at the follow-up Visit. Antibody responses against vaccinia were measured using validated ELISA and PRNT assays.

An overview of the assessment schedule and study procedures is given in the Flow Chart in Table 9-1.

Table 9-1 Flow Chart

Group 1, 2 and 3:

VISIT (V)	SCR	V 1	V 2	V 3	V 4	V 5	V FU
	DAY -28- -1	DAY 0	V 1 + 10-15 DAYS	V 1 + 28-35 Days	V 3 + 10-15 DAYS	V 3 + 28-35 DAYS	V 3 + 182-210 DAYS
Informed consent	X						
Check incl. / excl. criteria	X	X		X			
Medical history	X						
Vaccination		I		II			
Phys. exam incl. vital signs	X	X	X	X	X	X	X
ECG	X		X		X	(X)[2]	(X)[2]
Pregnancy test[1]	X	X		X		X	
Urine analysis	X						
Safety laboratory	X		X		X	(X)[2]	(X)[2]
Antibody analysis		X	X	X	X	X	X
Handout of diary card[3]		X		X			
Collection of diary card			X		X		
AE reporting		X	X	X	X	X	X[4]
Review baseline signs & symptoms	X	X					
Review prior / concomitant medication	X	X	X	X	X	X	
Blood sampling in mL[5]	15	8	23	8	23	8 (+15mL)[2]	8 (+15mL)[2]

[1] Urine or serum pregnancy test for women of childbearing potential only. At screening visit, a serum test was performed.

[2] Only required if clinically indicated.

[3]The diary card was completed daily for 7 days. If symptoms persisted at Day 7, temperature/symptom measurements were recorded each day until resolved.

[4] Serious adverse events only.

[5] Amount of single blood draws: Safety laboratory: 15 mL, Antibody analysis: 8 mL. Total amount of blood taken during study: Up to 123 mL for vaccinia-naïve subjects; up to 77mL for vaccinia-experienced subjects

Group 4:

VISIT (V)	SCR	V 1	V 2	V 3	V FU
	DAY -28 - 1	DAY 0	V1 + 10-15 DAYS	V 1 + 28-35 DAYS	V 1 + 182-210 DAYS
Informed consent	X				
Check incl. / excl. criteria	X	X			
Medical history	X				
Vaccination		I			
Phys. exam incl. vital signs	X	X	X	X	X
ECG	X		X	(X)[2]	(X)[2]
Pregnancy test[1]	X	X		X	
Urine analysis	X				
Safety laboratory	X		X	(X)[2]	(X)[2]
Antibody analysis		X	X	X	X
Handout of diary card[3]		X			
Collection of diary card			X		
AE reporting		X	X	X	X[4]
Review baseline signs & symptoms	X	X			
Review concomitant medication	X	X	X	X	
Blood sampling in mL[5]	15	8	23	8	8 (+15 mL)[2]

[1] Urine or serum pregnancy test for women of childbearing potential only. At screening visit, a serum test was performed.
[2] Only required if clinically indicated.
[3]The diary card was completed daily for 7 days. If symptoms persisted at Day 7, temperature/symptom measurements were recorded each day until resolved.
[4] Serious adverse events only.
[5] Amount of single blood draws: Safety laboratory: 15 mL, Antibody analysis: 8 mL. Total amount of blood taken during study: Up to 123 mL for vaccinia-naïve subjects; up to 77mL for vaccinia-experienced subjects

9.2 DISCUSSION OF STUDY DESIGN AND THE CHOICE OF CONTROL GROUPS

The primary objective of this study was to demonstrate that the booster immune response elicited by a single vaccination in a population previously vaccinated against smallpox (Group 4) was non-inferior to a vaccinia-naïve population which received two vaccinations (Group 1), i.e. to prove that a single dose schedule would be sufficient to boost the immunity in a previously vaccinated population.

Group 3 (two doses of placebo) served as an internal control mainly for safety / cardiac monitoring to validate the trial results and to show sensitivity of the trial.

Group 2, which received only a single vaccination with MVA-BN® followed by a placebo vaccination was used to compare the immune responses induced following a single vaccination with MVA-BN® in a vaccinia-naïve population, to a similar population that received two vaccinations (Group 1), or a vaccinia-experienced population also only receiving a single vaccination (Group 4).

In addition, the study was designed to further investigate the safety profile of MVA-BN® in healthy subjects. Recent findings from the US vaccination smallpox campaign

(Grabenstein et al.) and previous clinical trials indicated that after vaccination with the replication competent New York City Board of Health (NYCBH) vaccinia strain (Dryvax®), cardiac side effects occurred in an unexpectedly high frequency. Results of recently completed controlled clinical trials with the first generation smallpox vaccine (Dryvax®) and Acambis' second generation smallpox vaccine ACAM2000 demonstrated 10 cases of myo-/pericarditis in 1,162 vaccinated study subjects, i.e. a combined incidence of 5.97 events per thousand vaccinations (Vaccines and Related Biological Products Advisory Committee Briefing Document, 18 April 2007). Importantly, the events occurred only in vaccinia-naïve populations, whereas for a population previously vaccinated against smallpox no cardiac adverse reactions were reported. While the underlying mechanism of NYCBH strain related myo-/pericarditis is unknown, phenomena such as "molecular mimicry" as well as unspecific immunologically triggered mechanisms are being discussed. Thus, it has been stipulated by regulatory authorities to focus on the cardiac safety profile of all other vaccinia-based vaccines in development.

To date, more than 1,400 subjects have been vaccinated in clinical trials with MVA-BN® and not a single case of myo-/pericarditis has been detected. To further evaluate the cardiac safety profile of MVA-BN®, the comparison of the four groups with regard to ECG changes and cardiac symptoms was chosen as the co-primary safety objective. The main comparisons were Group 1 (vaccinia-naïve, two vaccinations) with placebo (Group 3), Group 2 (one vaccination) with placebo and Group 4 with placebo. Additionally, a comparison of Group 1 vs. Group 2 and Group 1 vs. Group 4, as well as pooled analyses of Groups 1, 2 and Groups 1, 2, 4 vs. Group 3 were performed.

Sample sizes of 200, 400 and 600 subjects were calculated to be sufficient to detect reactions reported at a frequency of less than 1/50, 1/100 and 1/200 respectively.

9.3 SELECTION OF STUDY POPULATION

9.3.1 INCLUSION CRITERIA

To be eligible for participation in the study the subjects had to fulfill the following criteria.

<u>All groups:</u>

1. Male and female subjects between 18 and 55 years of age.
2. Women must have a negative serum pregnancy test at screening and a negative urine or serum pregnancy test within 24 hours prior to vaccination.
3. Women of childbearing potential must have used an acceptable method of contraception for 30 days prior to the first vaccination, must agree to use an acceptable method of contraception during the study, and must not become pregnant for at least 28 days after the last vaccination. A woman was considered of childbearing potential unless post-menopausal or surgically sterilized. (Acceptable contraception methods were restricted to abstinence, barrier contraceptives, intrauterine contraceptive devices or licensed hormonal products.)
4. Read, signed and dated informed consent document after being advised of the risks and benefits of the study in a language understood by the subject signed, and prior to performance of any study specific procedure.
5. Troponin I within normal laboratory limits.
6. White blood cells ≥ 2500/mm3 and ≤ 11,000/ mm3.
7. Absolute neutrophil count (ANC) ≥ 1000/mm3.
8. Negative urine glucose by dipstick or urinalysis.
9. Hemoglobin ≥ LLN.

10. Platelets 100 – 450/nL.
11. Adequate renal function defined as:
    a. Serum creatinine without clinically significant findings.
    b. Urine protein ≤ 30 mg/dL or none or trace proteinuria (by urinalysis or dip stick).
12. Adequate hepatic function defined as:
    a. Total bilirubin ≤ 1.5 x ULN in the absence of other evidence of significant liver disease (healthy subjects without clinical disease with Morbus Meulengracht can be included).
    b. AST (SGOT), ALT (SGPT) and alkaline phosphatase without clinically significant findings.
13. Electrocardiogram (ECG) without abnormal findings (e.g. any kind of atrioventricular or intraventricular conditions or blocks such as complete left or right bundle branch block, AV node block, QTc or PR prolongation, premature atrial contractions or other atrial arrhythmia, sustained ventricular arrhythmia, 2 premature ventricular contractions (PVC) in a row, ST elevation consistent with ischemia).
14. Availability for follow-up for the planned duration of the study (26 weeks after last vaccination).

<u>Groups 1, 2 and 3 (vaccinia-naïve subjects) additionally:</u>
1. No history of known or suspected previous smallpox vaccination.
2. No detectable vaccinia scar.

<u>Group 4 (All previously vaccinated subjects) additionally:</u>
1. History of previous smallpox vaccination (documented and/or typical vaccinia scar).
2. Most recent smallpox vaccination ≥ 5 years ago.

9.3.2 EXCLUSION CRITERIA

Subjects who met any of the following criteria were excluded from study participation.

<u>Groups 1, 2, 3, and 4 (All subjects):</u>
1. Pregnant or breast-feeding women.
2. Uncontrolled serious infection i.e. not responding to antimicrobial therapy.
3. History of any serious medical condition, which in the opinion of the investigator would compromise the safety of the subject.
4. History of or active autoimmune disease. Persons with vitiligo or thyroid disease taking thyroid replacement were not excluded.
5. Known or suspected impairment of immunologic function including, but not limited to, clinically significant liver disease; diabetes mellitus; moderate to severe kidney impairment.
6. History of malignancy, other than squamous cell or basal cell skin cancer, unless there has been surgical excision that was considered to have achieved cure. Subjects with history of skin cancer at the vaccination site were excluded.
7. History or clinical manifestation of clinically significant and severe hematological, renal, hepatic, pulmonary, central nervous, cardiovascular or gastrointestinal disorders.
8. Clinically significant mental disorder not adequately controlled by medical treatment.
9. Any condition which might interfere with study objectives or would limit the subject's ability to complete the study or to be compliant in the opinion of the investigator.
10. History of coronary heart disease, myocardial infarction, angina, congestive heart failure, cardiomyopathy, stroke or transient ischemic attack, uncontrolled high blood pressure, or any other heart condition under the care of a doctor.

11. History of an immediate family member (father, mother, brother, or sister) who died due to ischemic heart disease before age 50 years.
12. Ten percent or greater risk of developing a myocardial infarction or coronary death within the next 10 years using the National Cholesterol Education Program's risk assessment tool. (http://hin.nhlbi.nih.gov/atpiii/calculator.asp?usertype=prof) NOTE: This criterion applies only to volunteers 20 years of age and older.
13. History of intravenous drug abuse.
14. History of allergic disease or reactions likely to be exacerbated by any component of the vaccine.
15. Known allergy to egg or aminoglycoside (gentamicin).
16. History of anaphylaxis or severe allergic reaction.
17. Having received any vaccinations or planned vaccinations with a live vaccine within 30 days prior or after study vaccination.
18. Having received any vaccinations or planned vaccinations with a killed vaccine within 14 days prior or after study vaccination.
19. Chronic administration (defined as more than 14 days) of systemic immuno-suppressant drugs during a period starting from six months prior to administration of the vaccine and ending at study conclusion. (Corticosteroid nasal sprays are permissible. Persons who have used topical and inhaled steroids could be enrolled after their therapy was completed).
20. Post organ transplant subjects whether or not receiving chronic immunosuppressive therapy.
21. Administration or planned administration of immunoglobulins and/or any blood products during a period starting from 3 months prior to administration of the vaccine and ending at study conclusion.
22. Use of any investigational or non-registered drug or vaccine other than the study vaccine within 30 days preceding the first dose of the study vaccine, or planned administration of such a drug during the study period.
23. Study personnel.

9.3.3 REMOVAL OF SUBJECTS FROM THERAPY OR ASSESSMENT

A temporary halting or termination for single study subjects or for the study as a whole could be decided in case of an occurrence of

- a serious adverse event (SAE)
- an unexpected Grade 3 or higher systemic reaction or lab toxicity

with an at least reasonable possibility of a causal relationship to the administration of MVA-BN®, i.e. the relationship could not be ruled out. These parameters were not all-inclusive. Other adverse events could have occurred that would have triggered a Data Safety Monitoring Board (DSMB) review. Any member of the DSMB, the Coordinating Investigator, BN Safety Officer, or DMID Medical Officer could request a DSMB review based on any observation.

The investigator had the right to terminate participation of any subject at any time. The following were justifiable reasons for premature withdrawal:

- intolerable adverse event
- intolerable side effect (adverse reaction) due to the study medication, including a medically relevant change in clinical laboratory parameters
- relevant non-compliance with the protocol
- concomitant disease
- if the investigator deemed it in the subject's best interest

Participation in the study was strictly voluntary. A subject had the right to withdraw his consent at any time for any reason or without giving reason. If he chose to withdraw, the investigator had to be informed immediately.

Any subject who was withdrawn or withdrew his consent and who had received the study vaccine had to undergo a final, post-study examination. The reason and circumstances for premature discontinuation had to be documented in the Case Report Form.

If study participation was terminated due to an adverse event possibly related to the study medication or study examinations, the subject had to be followed by additional examinations, according to the medical judgment of the investigator, until the abnormal condition was resolved or the investigator deemed further observations or examinations to be no longer medically indicated.

9.4 TREATMENTS

9.4.1 TREATMENTS ADMINISTERED

Test treatment:

Compound: MVA-BN® (Modified Vaccinia virus Ankara – Bavarian Nordic) (IMVAMUNE®)
Route: subcutaneous injection
Single dose: 0.5 mL (1 x 10^8 $TCID_{50}$) MVA-BN® vaccine
Dosage schedule: Group 1:
Two s.c. vaccinations with 0.5 mL MVA-BN® vaccine containing 1 x 10^8 $TCID_{50}$, four weeks apart (Visit 1/Day 0 and Visit 3/Visit 1 + 28-35 days)
Group 2:
First s.c. vaccination with 0.5 mL MVA-BN® vaccine containing 1 x 10^8 $TCID_{50}$ and second s.c. vaccination with placebo (0.5 mL Tris buffer), four weeks apart (Visit 1/Day 0 and Visit 3/Visit 1 + 28-35 days)
Group 3:
Two s.c. vaccinations with placebo (0.5 mL Tris buffer), four weeks apart (Visit 1/Day 0 and Visit 3/Visit 1 + 28-35 days)
Group 4:
One s.c. vaccination with 0.5 mL MVA-BN® vaccine containing 1 x 10^8 $TCID_{50}$ (Visit 1/Day 0)

Reference treatment (Placebo):

0.5 mL Tris buffer

9.4.2 IDENTITY OF INVESTIGATIONAL PRODUCT(S)

Test product:

Formulation: Modified Vaccinia Ankara Vector (MVA-BN®)
Strength: 1 x 10^8 $TCID_{50}$ per 0.5 mL
Container: Single dose 2 mL 2R glass vial
Batch number(s): 170505

Expiry date(s):	31 May 2007
Manufacturer:	IDT Biologika GmbH<br>Am Pharmapark<br>06861 Dessau-Rosslau, Germany
Trade name:	IMVAMUNE®
Storage requirements:	-20 ± 5°C

Placebo:

Formulation:	Tris buffer
Container:	Single dose 2 mL 2R glass vial
Batch number(s):	030505
Expiry date(s):	31 May 2007
Manufacturer:	IDT Biologika GmbH<br>Am Pharmapark<br>06861 Dessau-Rosslau, Germany
Storage requirements:	-20 ± 5°C

Composition: 1.21 g/L Tris, 8.526 g/L sodium chloride, 19.2 g/L dextran FP-40, 45.2 g/L sucrose and 0.108 g/L L-glutamic acid mono-potassium salt, pH 7.5

9.4.3 METHOD OF ASSIGNING PATIENTS TO TREATMENT GROUPS

Assignment of patients:

Subjects eligible for Groups 1, 2 and 3 were randomly allocated to one of these three treatment groups in order of their appearance. Subjects eligible for Group 4 were allocated to Group 4 in order of their appearance.

The complete randomization list with patient identifiers and assigned treatment is provided in Section 16.1.7.

9.4.4 SELECTION OF DOSES IN THE STUDY

The dose selected for a single vaccination was 1 x 10^8 $TCID_{50}$ MVA-BN®.

9.4.5 SELECTION AND TIMING OF DOSE FOR EACH PATIENT

All subjects in Groups 1-3 received two vaccinations each with a dose of 0.5 mL MVA-BN® containing 1 x 10^8 $TCID_{50}$ or placebo. Injections were administered subcutaneously in the non-dominant upper arm at an interval of approx. 4 weeks (Day 0 / Day 28-35). Subjects in Group 4 received one vaccination with a dose of 0.5 mL MVA-BN® containing 1 x 10^8 $TCID_{50}$.

9.4.6 BLINDING

The study was performed under double-blind conditions for Groups 1, 2 and 3. The randomization list was provided by Kendle GmbH and was included in the eCRF for randomization. Each vial of the study medication had a randomization number. By entering the

subject number in the eCRF, the investigator received the respective randomization number from the eCRF system.
In case of emergency, which would have made unblinding necessary, unblinding of the respective subject could have been done by the investigator by means of the eCRF. In addition, the Safety Department of Bavarian Nordic also had access to a sealed copy of the randomization list as a back up for a safety case making unblinding necessary.

9.4.7 PRIOR AND CONCOMITANT THERAPY

All concomitant medication was recorded in the eCRF with the reason for administration, the dosage regimen, and the onset and end of treatment (see Sections 14.6.5 and 16.2.9.7).

9.4.8 TREATMENT COMPLIANCE

The injections (either the study vaccine or placebo) were administered by the investigator and therefore treatment compliance was not an issue.

9.5 EFFICACY AND SAFETY VARIABLES

9.5.1 IMMUNOLOGY MEASUREMENTS ASSESSED

Immune response analysis was planned at all study visits (except the screening visit), including the follow-up visit. The baseline assessment for immunogenicity parameters was performed during Visit 1 (before vaccination).

The method of collection, storage and handling of laboratory specimens for the immune analysis was specified in SOP/CA/20: "Collection preparation and storage instructions for immune response serum specimens" (Doc. No. 10000213).

Antibody responses against vaccinia were measured by a direct ELISA and a PRNT using established and validated in-house assays.

Immune analyses were performed at Bavarian Nordic's laboratory at Bavarian Nordic GmbH, 82152 Martinsried, Germany.

9.5.2 SAFETY MEASUREMENTS ASSESSED

1. Medical history

Medical history was documented during the screening visit (visit SCR). For subjects in Group 4, the presence of a typical vaccinia scar and/or documentation of smallpox vaccination (if any vaccination certificate was available) was entered into the CRF.

2. Physical examination and vital signs

A physical examination and a check of vital signs were performed during the screening period (visit SCR) for baseline assessment and at every following visit (Visits 1 – 5 and FU).
The exam included a review of major organ systems and weight, as well as listening to the heart and lungs. Blood pressure and pulse rate were taken after the subject was seated for two minutes, and oral temperature was measured.

3. Laboratory measurements

Safety laboratory was determined at the screening visit (Visit SCR) and 10 – 15 days after each vaccination (Groups 1, 2 and 3: Visits 2 and 4 / Group 4: Visit 2). At Visit 5 (only Groups 1, 2 and 3) and Visit 3 (only Group 4) as well as at the follow-up visit (all Groups: Visit FU) safety laboratory was only done if clinically indicated. The safety laboratory measurements were performed at a central laboratory.

The following safety laboratory parameters were evaluated:

- Hematology:

Red blood cell count, hemoglobin, total and differential white blood cell count (WBC), platelet count

- Serum chemistry:

Total bilirubin, alkaline phosphatase, AST, ALT, serum creatinine, sodium, potassium, calcium, troponin I.

4. ECG assessments

A standard 12-lead ECG was recorded from all subjects at screening (Visit SCR) and 10-15 days after each vaccination (Groups 1, 2 and 3: Visits 2 and 4 / Group 4: Visit 2). At Visit 5 (only Groups 1, 2 and 3) and Visit 3 (Group 4) as well as at the follow-up visit (all Groups: Visit FU) an ECG was only done if clinically indicated.

ECGs were evaluated by the investigator and transmitted electronically to a central database. Abnormal and unclear ECG findings at screening or throughout the study were evaluated and followed-up by an external cardiologist, including a thorough examination and performance of an echocardiogram and/or treadmill ECG.

5. Adverse events

After each vaccination the subjects received a diary card on which they were instructed to record any solicited local and general adverse events, including intensity and course of the event. During the next scheduled visit, the investigator collected the diary cards and assessed the relationship of the solicited general events to study medication. Per definition, all local adverse events were considered to be at least possibly related to study vaccination.

Unsolicited adverse events following the vaccination were recorded by the subjects in a special section of the diary card and transferred by the investigator to the adverse event section of the CRF.

9.6 DATA QUALITY ASSURANCE

HCR was responsible for data quality assurance. In addition, Bavarian Nordic performed an audit of the study center on February 15th, 2007. The audit certificate is provided in Section 16.1.8.

9.7 STATISTICAL METHODS PLANNED IN THE PROTOCOL AND DETERMINATION OF SAMPLE SIZE

As soon as the last subject had completed the follow-up visit (Visit FU) and after all necessary settlement of queries in the CRFs, the database was locked. A full safety analysis of the data available was performed.

9.7.1 STATISTICAL AND ANALYSIS PLAN

See Section 16.1.9 for a detailed statistical analysis plan.

9.7.1.1 Analysis of demographics and baseline characteristics

An analysis of variance (ANOVA) for the main baseline characteristics (age, weight, height) was performed. The ANOVA was performed to detect any global treatment effect. The model does not include a term for location by treatment interaction (according to ICH guideline E9, Statistical Principles for Clinical Trials, p.16).

9.7.1.2 Analysis of Immunogenicity

Antibody titers and resulting seroconversion rates were assessed by validated ELISA and PRNT.

The primary immunogenicity variable was the vaccinia-specific seroconversion rate. This parameter was derived from the ELISA specific antibody titers.

The primary hypothesis was to show that the humoral immune response of the group with history of smallpox vaccination (Group 4) was not statistically inferior compared to the group without history of smallpox vaccination (Group 1).

The above hypothesis was tested based on an exact, unconditional test for binomial differences. In addition an exact one-sided 97.5% unconditional confidence interval for the difference of proportions was calculated. If the lower limit of this confidence interval was greater than 5% (or equivalent the p-value of the non-inferiority test was less than 5%), then the null hypothesis was rejected (StatXact®, Chan, 1998; Agresti and Min, 2001).

In order to limit the overall type-I error to a nominal level of 5%, a hierarchical test procedure was chosen:

In a first step, the primary null hypothesis was tested on the measurements of samples collected two weeks after the last vaccination (Groups 1-3: Visit 4, Group 4: Visit 2). Only if this comparison showed a significant result, the comparison of data of samples collected four weeks after vaccination (Groups 1-3: Visit 5, Group 4: Visit 3) was performed.

A secondary analysis with the neutralization assay specific seroconversion rates (from PRNT) was performed similar to the method described above.

In addition to the main comparison of Group 4 versus Group 1, secondary comparisons were also made among all other groups.

All statistical tests for secondary time points and comparisons were considered descriptive. Therefore, no adjustment for multiple testing was done.

Tables and graphs showing the seroconversion rates for each group at each visit were prepared. Descriptive statistics and graphical displays were given for the geometric mean titer for all groups at all visits.

9.7.1.3 Analysis of safety and reactogenicity

Solicited local adverse events

The occurrence of solicited local adverse events within one week after each vaccination was summarized on a per patient and per vaccination basis.
The maximum intensity over the 7-day period after vaccination was used and categorized as follows:

Pain:
Grade = 0 / > 0
Grade < 2 / ≥ 2
Grade < 3 / ≥ 3

For measurements of diameter size:
Diameter = 0 / > 0
Diameter < 30mm / ≥ 30 mm
Diameter < 100mm/ ≥ 100 mm

These categories were compared between treatment groups by means of the exact test of Fisher (or its normal approximation, the chi-squared test).

Solicited general adverse events

Occurrence of solicited general adverse events within one week after each vaccination was summarized per patient and vaccination. The maximum intensity over the 7-day period after vaccination was used and categorized as follows:
Grade = 0 / > 0
Grade < 2 / ≥ 2
Grade < 3 / ≥ 3

These categories were compared between treatment groups by means of the exact test of Fisher (or its normal approximation, the chi-squared test).

Unsolicited adverse events:

Unsolicited adverse events were coded using the Medical Dictionary of Regulatory Activities (MedDRA) coding terminology. The intensity of adverse events was graded according to the following descriptions:

1 = An adverse event which is easily tolerated by the subject, causing minimal discomfort and not interfering with daily activities.
2 = An adverse event which is sufficiently discomforting to interfere with daily activities.

3 = An adverse event which prevents daily activities. (Such an adverse event would, for example, prevent attendance at work and would necessitate the administration of corrective therapy.)
4 = Life-threatening or disabling

The number of adverse events and number of patients with at least one adverse event for each preferred term were descriptively compared between treatment groups.

The occurrence of any Grade 3 or higher adverse event possibly, probably or definitely related to the study vaccine within 28 days after vaccination was compared between treatment groups.

Adverse events of special interest were separately listed and tabulated. The incidence of these AEs was compared between treatment groups by means of the exact test of Fisher (or its normal approximation, the chi-squared test).

Serious adverse events were listed separately. Each SAE was described individually in detail. The number of subjects with at least one SAE was compared between treatment groups by means of the exact test of Fisher (or its normal approximation, the chi-squared test).

The occurrence, relationship and intensity of any cardiac symptoms at any time during the study were listed. The number of subjects with at least one cardiac symptom was compared between treatment groups by means of the exact test of Fisher (or its normal approximation, the chi-squared test).

Safety laboratory and urine analysis:
Clinical laboratory test results were marked whether the result is below, within or above the respective reference range. The number of values outside of the reference range was counted.

ECG
ECGs were evaluated by a centralized procedure. The transmitted standard ECG results like PQ, QRS, QT and QTc duration and heart rate were summarized per visit and treatment group.

1. Overall assessment
   a. The number of subjects with normal/abnormal ECGs and in case of abnormal results clinically significant ECGs (resulting in AEs) was compared between treatment groups by means of the exact test of Fisher (or its normal approximation, the chi-squared test).
   b. The number of subjects with myo-/pericarditis was compared between treatment groups by means of the exact test of Fisher (or its normal approximation, the chi-squared test).
   c. For all clinically significant ECGs, individual data listings showing the type of ECG abnormality were prepared.

2. Analysis of QTc

According to the FDA (Food and Drug Administration) concept paper for *'Clinical Evaluation Of QT/QTc Interval Prolongation And Proarrhythmic Potential For Non-Antiarrhythmic Drugs'* and the EMEA (European Medicines Agency) *'Guidance for Industry E14 Clinical Evaluation of QT/QTc Interval Prolongation and Proarrhythmic Potential for Non-Antiarrhythmic Drugs'* a detailed QTc analysis was performed:

a. Correction of QT interval data

QT interval data was corrected for heart rate based upon Fridericia's formula:

Fridericia's correction: $QTc = QT/RR^{0.33}$, where RR is the resting rate.

The QTc values reported from the conventional ECG are based on Bazett's correction ($QTc = QT/RR^{0.5}$).
In general, however, Bazett's correction over-corrects at elevated heart rates and under-corrects at heart rates below 60 beats per minute (bpm) and hence is not an ideal correction. Fridericia's correction is more accurate than Bazett's correction in subjects with such altered heart rates. Therefore, Fridericia's correction only was used for summary tables.

b. Analyses of Central Tendency

Absolute and baseline-adjusted QTc values were tabulated with descriptive statistics per treatment group and per visit. In addition, the maximum absolute and baseline-adjusted change was tabulated per treatment group.

c. Categorical Analyses

Categorical analyses of QTc interval data were based on the number and percentage of subjects meeting or exceeding some predefined upper limit value. The limits were defined as follows:

→ Absolute QTc interval prolongation:

- QTc interval > 450 ms
- QTc interval > 480 ms
- QTc interval > 500 ms

→ Change from baseline in QTc interval:

- QTc interval increases from baseline ≥ 30 ms
- QTc interval increases from baseline ≥ 60 ms

Frequency tables showing the absolute and relative frequency for each category per treatment group and visit were prepared. In addition, the maximal QTc prolongation, respectively the maximum change from baseline over time was used for classification into the categories.

d. Further ECG data
PR-, QT- and QRS-durations as well as the reported QTc-duration based on the Bazett correction and heart rate were listed and tabulated per treatment group and visit.

9.7.1.4 Data handling

All data obtained in this study and documented in the CRFs were listed. For parameters of interest, summary tables with descriptive group statistics (mean, standard deviation, minimum, maximum, number of valid cases) for metrical variables were prepared. For ordinal/ dichotomous variables summary tables showing the absolute and relative count in each category were prepared.

Further statistical analysis was performed as appropriate.

9.7.2 DETERMINATION OF SAMPLE SIZE

The sample size calculation was based on the primary immunogenicity variable 'vaccinia-specific seroconversion rate'. This parameter was derived from the ELISA specific antibody titers. Seroconversion was defined as the appearance of antibody titers ≥ 1:50 in a vaccinia-specific IgG ELISA for initially seronegative subjects or two-fold increase of the antibody titer compared to the pre-existing baseline titer for subjects with a pre-existing antibody titer in the ELISA.

The primary hypothesis was to show that the humoral immune response of the group with history of smallpox vaccination (Group 4) was not statistically inferior compared to the group without history of smallpox vaccination (Group 1). The study was designed to demonstrate that the Group 4 seroconversion rate is not worse than the Group 1 seroconversion rate by more than a pre-specified amount. This amount is called the non-inferiority margin (D).

Suppose p_1 is the seroconversion rate in the group without history of smallpox vaccination and p_4 is the seroconversion rate in the subjects with history of smallpox vaccination.
The test on non-inferiority was applied for the following hypothesis:

H_0: $p_4 - p_1 \leq -\square$ versus H_1: $p_4 - p_1 > -\square$, where

□ is the non-inferiority margin and is chosen in this trial as 5%.
From the experience in a pilot trial with MVA-BN® in healthy subjects, it was anticipated that the seroconversion rate in healthy vaccinia-naive subjects reaches 98-100%.

Assuming a significance level of 5%, a power of 80% and expected seroconversion rates of 98% in both groups, this yielded a sample size of 175 subjects per group (700 subjects in total). In order to account for drop outs, at least 180 subjects per group were treated.

For reference see Agresti A, Min Y, 2001 and Chan I, 1998.

9.8 CHANGES IN THE CONDUCT OF THE STUDY OR PLANNED ANALYSES

One Amendment to the Study Protocol (version 1.0 from 12 December 2005), dated 26 March 2007, was released. The contents of this Amendment No. 1 were fully incorporated in the above description of the investigational plan.

The Amendment introduced the following changes:

The number of subjects to be enrolled in Groups 1-3 was reduced from the originally planned 200 to 180 subjects per group. 200 subjects had been originally calculated based on the sample size calculation, which required 175 subjects per group for the evaluation, accounting for 25 drop-outs per group. As the actual number of drop-outs proved to be much lower than expected, the compensation to account for drop-outs was reduced accordingly, i.e. 20 subjects fewer for each of the Groups 1-3. At the time of implementation of the Amendment, Group 4 had already been completely enrolled (N=200) as planned.

The Safety Monitoring Committee (SMC) was changed to a Data Safety Monitoring Board (DSMB).

The workflow with regard to evaluation of cardiac complications was simplified. The division of the primary responsibility between two parties (centralized ECG service provider and investigator) was removed and the primary responsibility for the assessment of cardiac events was given to the investigator. The electronic transmission of all ECGs to a central database by the investigator was continued. A local, external cardiologist was responsible for evaluation and follow-up of abnormal and unclear ECG findings sent to him/her by the investigator.

The study halting and termination rules were extended and the time constraint was removed. Subjects could be discontinued or temporarily halted or the study could be terminated or halted in case any SAE, unexpected systemic reaction (Grade 3 or higher) or Grade 3 laboratory toxicity occurred, independent of duration (previously lasting > 48 hours).

The background section of the protocol was updated to include results obtained from a recently completed clinical trial with MVA-BN®.

The range specified for the inclusion criterion "platelets" was adjusted to the reference values of the safety laboratory used in this trial.

The names of the contact persons at Bavarian Nordic, the centralized laboratory (Interlab) and ECG service provider (ganiMed) were updated and a local, external cardiologist was added.

Garth Virgin, MD replaced Jens Vollmar, MD as the medical monitor for this trial.

In addition to the changes introduced in the amendment the lower detection limit of the PRNT was changed from the planned 20 to 6. Correspondingly PRNT specific seroconversion for subjects with a baseline titer was defined as an antibody titer of ≥6 and not ≥20.

10 STUDY SUBJECTS

10.1 DISPOSITION OF SUBJECTS

The exact numbers of subjects attending each visit is given in Section 14.1.2. An overview of the disposition of subjects from screening up to the last visit of the active study period (Visit 5 for subjects in Groups 1, 2 and 3; Visit 3 for subjects in Group 4) can be seen in Figure 10-1. 753 subjects were enrolled and randomized in this study. Eight subjects were withdrawn prior to vaccination because they were no longer eligible after re-check of the inclusion and exclusion criteria (see Section 16.2.3.1). 745 subjects received at least one study vaccine administration with 183 subjects being randomized to Group 1, 181 subjects each to Groups 2 and 3 and 200 subjects to Group 4.

In Group 4, all 200 subjects completed Visits 1 to 3. From the 545 subjects in Groups 1-3, 529 subjects received the second injection and 523 subjects completed all 5 study visits.

22 subjects discontinued the study prematurely, 8 subjects each in Groups 1 and 2 and 6 subjects in Group 3. Reasons for discontinuation were withdrawal of consent (11 subjects), subject unwilling /unable to comply with study requirements (8 subjects), adverse events (2 subjects), and lost to follow-up (1 subject). See Section 14.6.1 and a listing by subject in Section 16.2.1.1.

In Group 1 all 178 subjects who had completed Visit 5 returned for the follow-up visit. In each Group 2 and Group 4 only one subject of the subjects who completed Visit 5 (175 subjects) and Visit 3 (200 subjects) respectively did not return for the follow-up visit. Of the 177 subjects in Group 3 two subjects did not return for the follow-up visit (see Section 14.1.2).

The first subject was enrolled on 27 April 2006; the last subject completed the follow-up visit on 27 August 2007 (see Section 16.2.5.6).

The visit dates and time between visits are listed in Section 16.2.5.6 and 16.2.5.7.

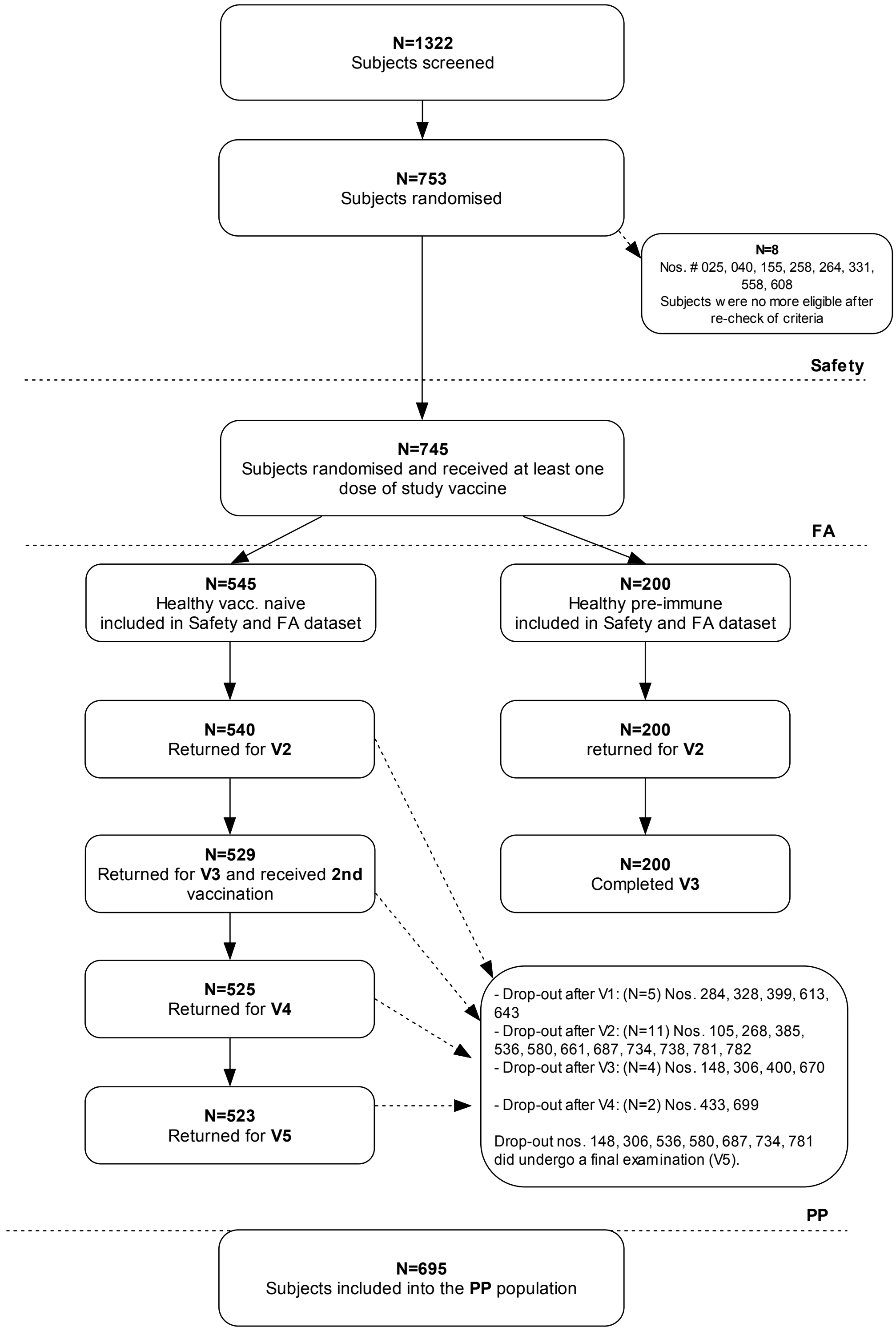

Figure 10-1 Disposition of subjects

10.2 PROTOCOL DEVIATIONS

No subjects were excluded from the safety population because of protocol deviations. All protocol deviations are listed in detail in Section 16.2.2 sorted by protocol deviation and subject number.

Four subjects were falsely and one subject was possibly falsely allocated into Group 4. The subjects had been allocated into Group 4 at screening, because of the existence of a vaccination scar. It was detected later that in four subjects (numbers 043, 050, 061 and 089) the scar was the result of a documented BCG (Bacillus of Calmette and Guerin, tuberculosis) vaccination. In subject 115 the vaccination status remained unclear, but according to the subject's mother the subject had had no smallpox vaccination and the scar might have been the result of a BCG vaccination.
Subject 594 (26 years old) was falsely allocated into Group 3, because of the subject's statement that she had not had a smallpox vaccination. Later the subject provided a document proving that she had been vaccinated against smallpox.
All six subjects were excluded from the PP (per protocol) population.

The most frequent protocol violations were violations against the time windows between visits (see Section 16.2.2 and Table 10-1 below). 24 violations in 23 subjects were considered relevant for exclusion of the respective subjects from the PP population.

Table 10-1 Violations against visit windows

Visit windows	Time between visits according to the protocol	Time between visits considered not relevant for exclusion from PP population	Number of subjects excluded from PP population because of relevant violations against visit windows
Days between V1 and Screening	< -28 days	-35	-
Days between V2 and V1	10 – 15 days	9 – 18 days	6
Days between V3 and V1	28 – 35 days	25 – 38 days	9
Days between V4 and V3	10 – 15 days	9 – 18 days	7
Days between V5 and V3		25 – 38 days	2

Source: See section 16.2.2.

The second most frequent protocol violations were violations against inclusion and exclusion criteria regarding safety laboratory values being outside of the specified range. Most frequently inclusion criteria no. 9 was not fulfilled, i.e. in some subjects hemoglobin was below LLN. These violations were all considered minor and all subjects remained in the PP analysis.

A total of 29 subjects were excluded from the PP population because of the protocol deviations described above, of whom one subject dropped out of the study before Visit 5 in addition to the protocol deviation.

11 IMMUNOGENICITY EVALUATION

11.1 DATASETS ANALYSED

Two datasets were used for immunologic analysis, the FA (full analysis) dataset and the PP dataset (see Section 16.1.9). In the FA dataset data from a total of 745 randomized healthy subjects who received at least one vaccination (one injection of MVA-BN® or placebo) were included. In the PP dataset only data of those subjects were included who received two injections (Groups 1–3) or one injection (Group 4), completed Visits 1-5 in Groups 1–3 and Visits 1–3 in Group 4 and adhered to all protocol conditions. A total of 50 subjects were excluded from the PP dataset, due to relevant protocol deviations (29 subjects, see Section 10.2) and/or premature drop out (22 subjects) (see Section 16.2.1 and Section 10.1), including one subject who was excluded because of both a relevant protocol deviation and premature drop out. As outlined in the Statistical Analysis Plan, the primary analysis was performed on the FA dataset. Therefore, the following descriptions mainly refer to the FA dataset. Additionally, differences between FA and PP datasets are explained.

11.2 DEMOGRAPHIC AND OTHER BASELINE CHARACTERISTICS

Details on the demographic data and other baseline characteristics are listed in Section 16.2.4, corresponding summary statistics are provided in Section 14.1. The description of the study population refers to the FA dataset (see Section 11.1) and consists of 314 male and 431 female subjects.

In each treatment group there were more female than male subjects (53.0%, 61.9%, 59.1% and 57.5% in Groups 1–4, respectively). As can be expected due to the discrimination concerning history of smallpox vaccination, the vaccinia-naïve population (Groups 1–3) in this study was younger (mean age 25.3, 25.4 and 26.0 years, respectively) than the vaccinia-experienced population (mean age 41.5 years in Group 4). In Groups 1–3, the subjects' age ranged from 18 to 50 years, compared to a minimum age of 22 years and a maximum age of 55 years in Group 4. The one subject (no. 043) in Group 4 who was 22 years old was probably falsely allocated into Group 4 (see Section 10.2); therefore the actual minimum age of vaccinia-experienced subjects was 24 years.

There were no apparent differences between Groups 1–4 regarding weight and BMI (body mass index). The mean BMI of the subjects in the Groups 1–4 ranged from 23.6 to 24.6 kg/m2.

Except for a total of five subjects who were falsely or probably falsely allocated into Group 4 (see protocol deviations in Section 10.2), all subjects in Group 4 had a history of smallpox vaccination as determined by the presence of a vaccination scar and/or by documentation. Two subjects had no vaccination scar, but provided documentation about a previous smallpox vaccination.

The population targeted was healthy and there were no clinically significant findings in the medical histories of the subjects that led to exclusion according to the opinion of the investigator (see Section 16.2.4.3). 28 subjects had a known history of cardiovascular disease; however, all conditions were either no longer active and/or were adequately controlled with medication. All of these subjects complied with the inclusion/exclusion criteria.

11.3 MEASUREMENTS OF TREATMENT COMPLIANCE

The injections were administered by the study personnel. Treatment compliance was therefore not an issue. Details on date and time of the injection and information about whether the injection was made in the left or right arm are provided in Section 16.2.5.9.

11.4 IMMUNOGENICITY RESULTS AND TABULATIONS OF INDIVIDUAL SUBJECT DATA

11.4.1 ANALYSIS OF IMMUNOGENICITY

Immune responses after vaccination with MVA-BN® were analyzed by measuring the vaccinia-specific antibody response using a direct validated ELISA and a validated PRNT. Antibody titers were measured from samples taken at all study visits (except at screening) and follow-up. Baseline anti-vaccinia antibody levels were determined at Visit 1.

Antibody titers sorted by subject and visit and information about seroconversion for the FA dataset are provided in Section 16.2.6.1 (ELISA analysis) and 16.2.6.2 (PRNT analysis). When a subject dropped out of the study prematurely (see Section 10.1), the missing titer values were specified with ND (Not done). One serum sample in subject 120 (Group 3) and one sample in subject 037 (Group 4) could not be evaluated by ELISA and PRNT. These missing values were marked with NE (Not evaluable).
The PRNT analysis of the follow-up sample of Subject 173 (Group 4) was performed after databank closure. Therefore in Section 16.2.6.2 this value is marked with NA (Not analyzed), the titer however was determined and was 174.

11.4.1.1 Anti-vaccinia antibody responses measured by direct ELISA

A validated ELISA was used to determine vaccinia-specific antibody titers. The cut-off of the ELISA was an antibody titer of 50, meaning that antibody titers below 50 were considered seronegative and given the arbitrary value of 1 in all analyses.

11.4.1.2 ELISA specific antibody titers

As expected most subjects in Groups 1–3, who had no history of previous smallpox vaccination prior to the start of the study, were seronegative at baseline or in sporadic cases had titers at or close to the lower detection limit (50 or 100).
In only six subjects a notable titer (>100) was observed at baseline in Groups 1-3. The highest ELISA baseline titer was measured in Subject 102 (1134), who had however titers of 50 both at Visits 2 and 3, indicating a possible testing artifact.

Most subjects who received two doses of placebo and who did not receive any vaccination with MVA-BN® (Group 3) remained seronegative throughout the study. Some subjects in Group 3 had ELISA specific titers ≤ 100 throughout the study. Elevated titers were observed in only three subjects in Group 3. Two of theses higher titers were measured at Visit 4 in two subjects who were, however, seronegative at all other visits (subject 099: titer=316, subject 656: titer=910), indicating that these two outliers might be test deviations. Subject 594 who had a

titer greater than 100 was obviously incorrectly assessed as being vaccinia-naïve, as evident from antibody titers of 100 or greater at all study visits, including the follow-up visit.

The geometric mean titer (GMT) was calculated by taking the antilogarithm of the mean of the log10 titer transformations. For the FA dataset the GMT for each group and visit is illustrated as columns in Figure 11-1 and as graphs in Figure 11-2, showing the course of the GMT for the four treatment groups throughout the study (Visit 1 to follow-up visit). At baseline (Visit 1) a GMT of 1.440 in Group 1, 1.271 in Group 2 and 1.147 in Group 3 confirmed the vaccinia-naïve status of the subjects in Groups 1–3, as compared to a GMT of 38.845 in Group 4 containing subjects previously vaccinated against smallpox.
As expected the GMTs in Groups 1 and 2 were in the same range until 4 weeks after the first vaccination with MVA-BN® (Group 1: GMT =71.847, Group 2: GMT=60.343).
For Group 1 subjects who received then a second vaccination of MVA-BN® at study Visit 3, a strong booster effect was observed two weeks after this second vaccination (Visit 4) with the GMT increasing to 495.843 (with titers ranging from 1 to 9101). In contrast, after the second vaccination with placebo for Group 2 subjects, the GMT decreased slightly to values of 42.327 measured at Visit 4 and 23.193 at Visit 5.

As described above, negligible antibody responses against MVA-BN® were observed in subjects who only received placebo (Group 3), which is accordingly reflected in low GMTs throughout the study (ranging from 1.1 to 1.3).

A strong booster effect could be observed in the vaccinia-experienced subjects (Group 4) with an increase in the GMT from a baseline value of 38.845 to 568.819 (with titers ranging from 1 to 8875) two weeks after the vaccination with MVA-BN® and reaching an even higher GMT compared to Group 1 subjects (GMT: 495.843) at two weeks after their second MVA-BN® vaccination.

In summary, these results demonstrate a rapid re-emergence of the humoral immune response in vaccinia-experienced subjects, with the maximal GMT measurable already two weeks after the vaccination, compared to the much lower peak response in vaccinia-naïve subjects after only one dose of MVA-BN® (Groups 1 and 2). However, the antibody response to a second ("booster") vaccination with MVA-BN® in vaccinia-naïve subjects reached similar GMT levels at the study visit two weeks following the booster vaccination (Visit 4) (see Figure 11-2).

At the follow-up visit, approx. 6 months (26-30 weeks) after the last vaccination, the GMT in Group 1 had decreased from 328.666 at 4 weeks after the second vaccination to a still high GMT level of 27.900. This GMT approx. corresponds to the GMT level measured in the vaccinia-experienced subjects in Group 4 at baseline (38.845). It can therefore be speculated that these subjects might experience a similar booster effect as observed in Group 4 when re-vaccinated with MVA-BN®.
The high GMT observed in Group 4 at four weeks after the vaccination (452.321) decreased to 179.907 at 26-30 weeks after the vaccination. Subjects in Group 2 who had only received one dose of MVA-BN® still had a GMT of 5.279 at the follow-up visit.

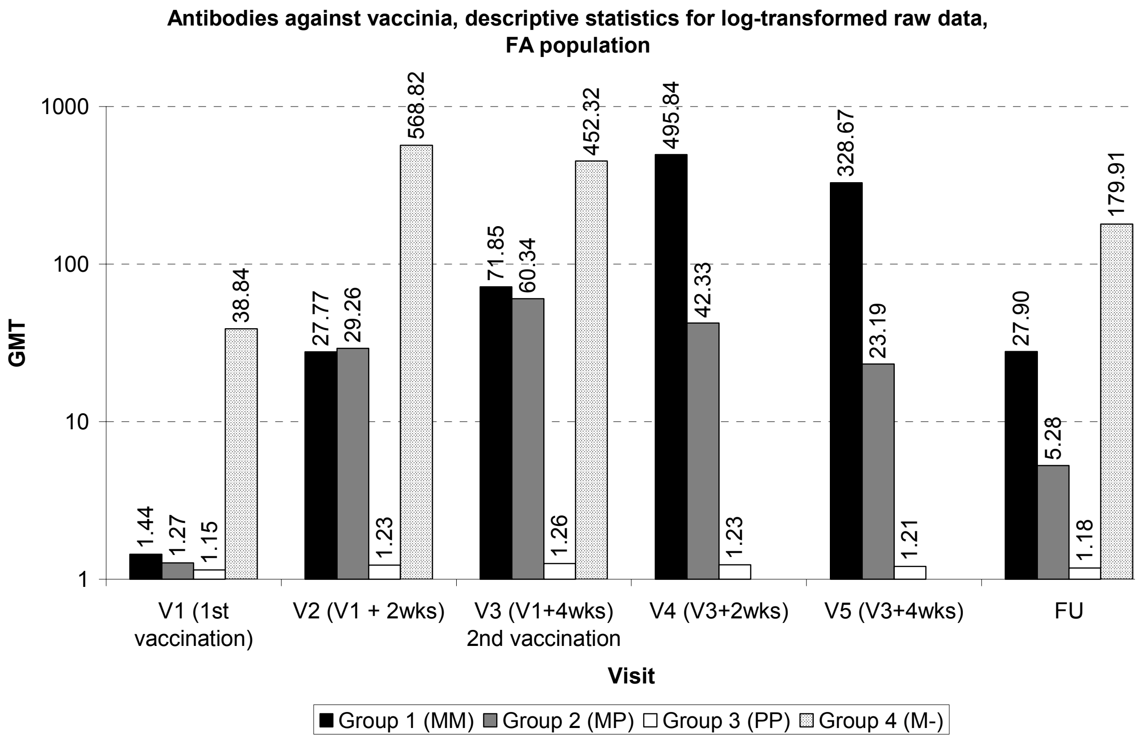

Figure 11-1 Vaccinia-specific antibody response, measured by ELISA; FA dataset
Source: Section 14.2.1.5

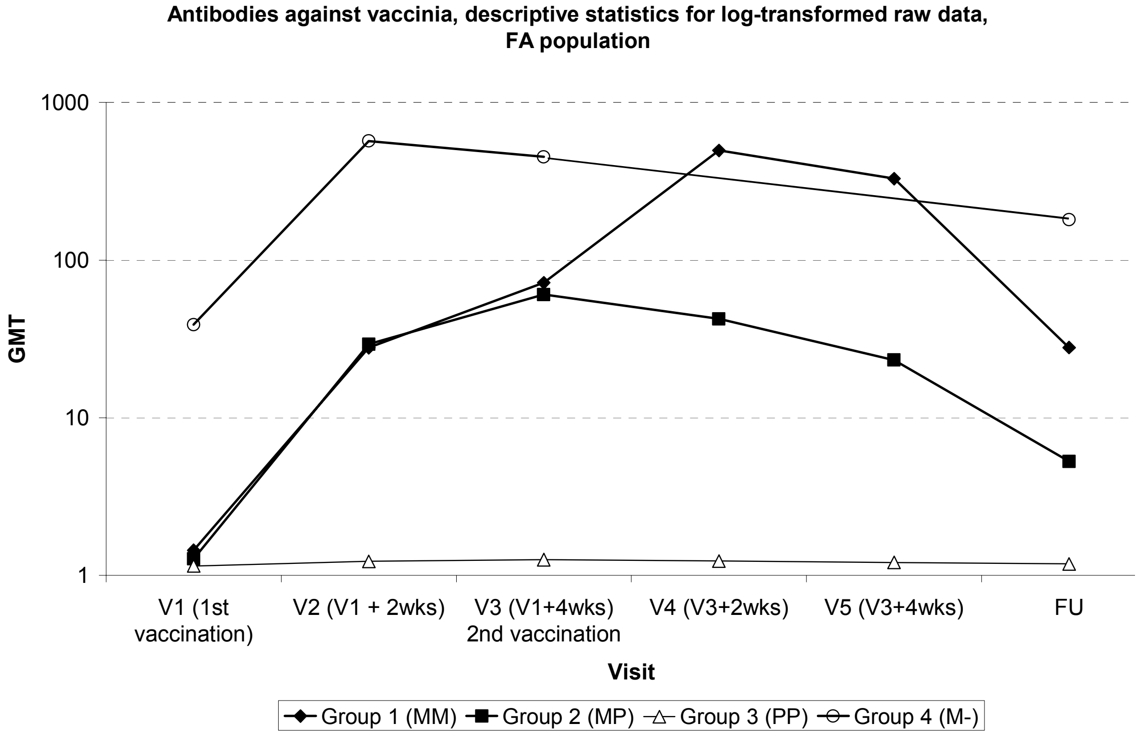

Figure 11-2 Vaccinia-specific antibody response measured by ELISA /Time response; FA dataset
Source: Section 14.2.1.5

The corresponding figures for the PP dataset are provided in Section 14.2.1.6. Differences in the GMT values between the FA and PP dataset were minor.

11.4.1.3 Seroconversion derived from ELISA specific antibody titers

Seroconversion derived from ELISA specific antibody titers was defined as an antibody titer of ≥ 50 in subjects who were seronegative at baseline, or a two-fold increase of the antibody titer compared to the pre-existing baseline titer in subjects showing an antibody response against vaccinia at baseline.

The seroconversion rates for the FA and PP dataset are tabulated in Section 14.2.1.1 and 14.2.1.2 respectively; column charts are provided in Sections 14.2.1.7 – 14.2.1.10 (for the FA dataset see also Figure 11-3 and Figure 11-4). Individual information about seroconversion for the FA dataset is listed in Section 16.2.6.1.

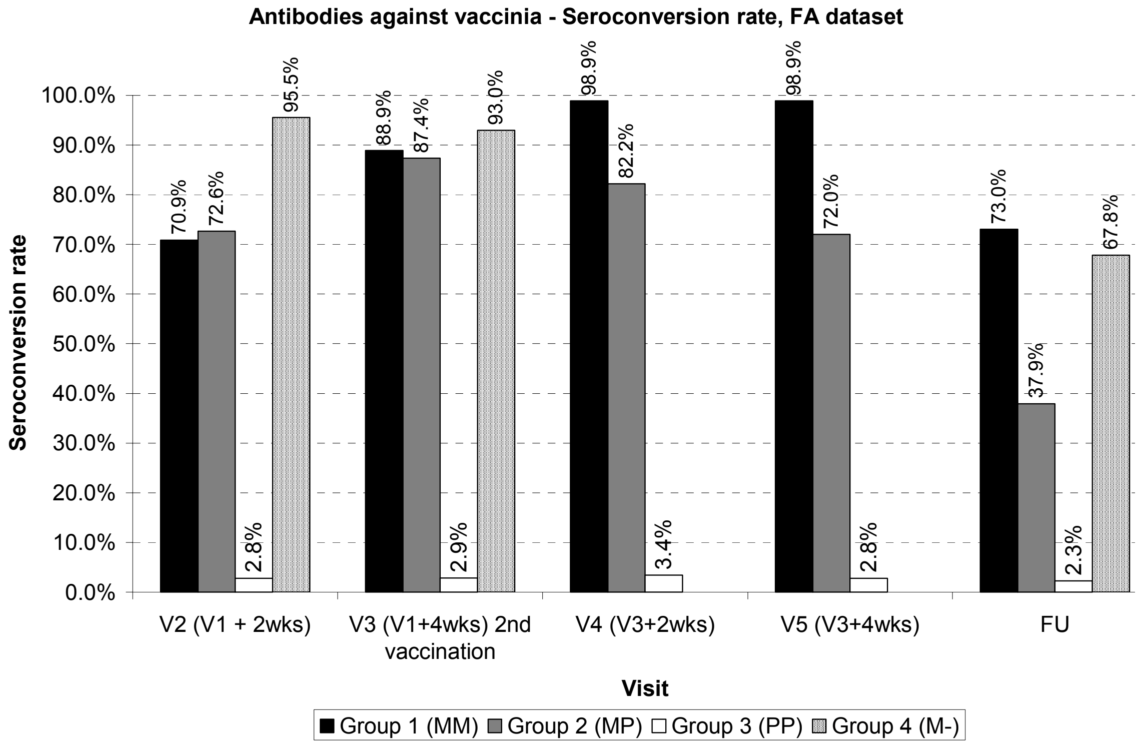

Figure 11-3 Seroconversion rate, measured by ELISA, FA dataset
Source: Section 14.2.1.7

Of the vaccinia-naïve subjects having received a first dose of MVA-BN® in the FA dataset, 70.9% of the subjects in Group 1 and 72.6% of the subjects in Group 2 had seroconverted two weeks after the first vaccination. In this first vaccination period, seroconversion rates slightly increased over time to 88.9% in Group 1 and 87.4% in Group 2 four weeks after the first vaccination (see Figure 11-3).

Two weeks after the second vaccination (Visit 4) the seroconversion rate increased to 98.9% in vaccinia-naïve subjects (Group 1) and remained at the same level 4 weeks after the second dose (Visit 5). 26-30 weeks after the second dose 73.0% of the subjects in Group 1 still met the

criteria for seroconversion. In subjects who received placebo as the second injection (Group 2), the seroconversion rate had decreased to 82.2% after two weeks (Visit 4), 72.0% after four weeks (Visit 5) and to 37.9% after 26-30 weeks (follow-up) following the placebo injection.

Subjects from Group 3 having received two doses of placebo showed low seropositivity which remained stable over the course of the study (2.8%, 2.9%, 3.4%, 2.8% and 2.3% at Visits 2, 3, 4 5 and follow-up, respectively) indicating that subjects of this study group, indeed received placebo only.

After administration of a single dose of MVA-BN® to vaccinia-experienced subjects (Group 4), 95.5% of these subjects (191 out of 200 subjects) had seroconverted at Visit 2 two weeks later. Four weeks after the vaccination, 93.0% of the subjects still fulfilled the criteria for seroconversion. At the follow-up visit, 26-30 weeks after the vaccination, a seroconversion rate of 67.8% was observed. An overview of the seroconversion rates of Groups 1-4 in the FA dataset specifically for the time points two and four weeks after the last vaccination, as well as the follow-up is provided in Figure 11-4 below.

In the PP dataset similar GMTs were observed for all groups and time points.

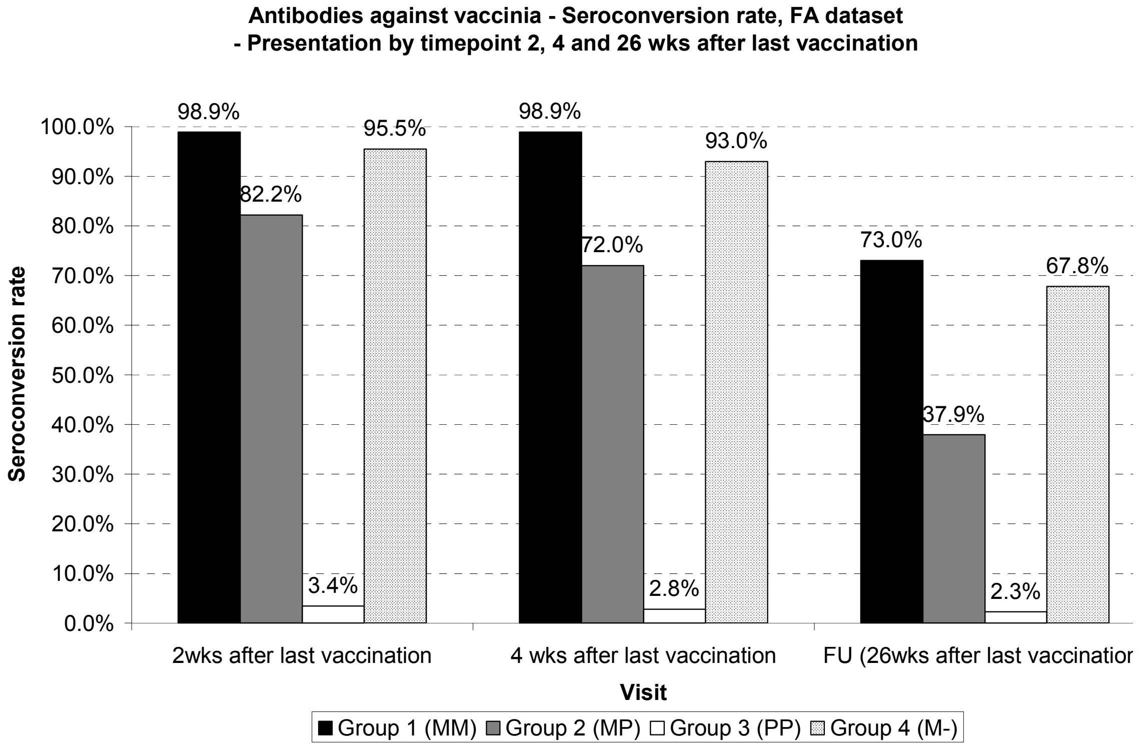

Figure 11-4 Seroconversion rate, measured by ELISA, two and four weeks after last vaccination, FA dataset
Source: Section 14.2.1.8

The differences in the seroconversion rates between Group 4 and 1 were –3.4% in the FA dataset and –2.4% in the PP dataset at two weeks after the last vaccination. In both datasets these differences were not statistically different, as shown by the p-values provided in Table 11-1 (0.0675 for the FA and 0.1837 for the PP dataset). At four weeks after the last vaccination

a statistical difference in the seroconversion rates between Group 4 and 1 was observed in the FA dataset (difference: -5.9%; p-value: 0.0043) and PP dataset (difference: -6.1%; p-value: 0.0046) (see Table 11-2).

The primary immunogenicity variable was to test whether the seroconversion rate in Group 4 was non-inferior to the seroconversion rate in Group 1, based on a non-inferiority margin of (-5%; +∞) pre-defined in the protocol (see Table 11-1 and Table 11-2). In the FA dataset the confidence intervals for the difference of seroconversion rates between Group 4 and Group 1 exceeded the pre-defined margin mentioned above at two weeks (-7.36%; +∞) and at four weeks after the last vaccination (-10.52%; +∞) (see Section 16.1.9). In the PP dataset the confidence intervals similarly exceeded the pre-defined margin.

Therefore, statistically, it could not be shown that the seroconversion rate in Group 4 after a single booster vaccination was non-inferior to the seroconversion rate in Group 1 after two vaccinations with MVA-BN®, which is also reflected in the p-values for the rejection of the null hypothesis of inferiority (p-values were non-significant (≥ 0.05) at two weeks (0.2162) and at four weeks (0.7055) after the last vaccination in the FA dataset, see Section 16.1.9). Similar non-significant p-values were observed in the PP dataset.

Table 11-1 Primary efficacy parameter: Vaccinia-specific seroconversion rate derived from the ELISA specific antibody titers 2 weeks after the last vaccination (Group 1-3: Visit 4, Group 4: Visit 2)

Population	Seroconversion rate<br>Group 1 (MM)	Seroconversion rate<br>Group 4 (M-)	Difference Group 4 – Group 1	p-value for the test of the null hypothesis of equality of proportions[1] (Fisher's exact test)	p-value for the rejection of the null hypothesis of inferiority	Exact one-sided 97.5% confidence interval for the difference of rates between Group 4 and Group 1
FA	98.9% (174/176)	95.5% (191/200)	-3.4%	0.0675	0.2162	(-7.36%; +∞)
PP	98.8% (166/168)	96.4% (186/193)	-2.4%	0.1837	0.0857	(-6.27%; +∞)

[1] This was only done for descriptive purposes and is not the p-value for the actual primary null and alternative hypothesis for testing the non-inferiority

Unconditional exact test for non-inferiority for a binomial difference

The predefined non-inferiority margin was (-5%; +□).

Table 11-2 Secondary efficacy parameter: Vaccinia-specific seroconversion rate derived from the ELISA specific antibody titers 4 weeks after the last vaccination (Group 1 3: Visit 5, Group 4: Visit 3)

Population	Seroconversion rate Group 1 (MM)	Seroconversion rate Group 4 (M-)	Difference Group 4 – Group 1	p-value for the test of the null hypothesis of equality of proportions[1] (Fisher's exact test)	p-value for the rejection of the null hypothesis of inferiority	Exact one-sided 97.5% confidence interval for the difference of rates between Group 4 and Group 1
FA	98.9% (176/178)	93.0% (185/199)	-5.9%	0.0043	0.7055	(-10.52%; +∞)
PP	98.8% (166/168)	92.7% (178/192)	-6.1%	0.0046	0.7452	(-10.84%; +∞)

[1] This was only done for descriptive purposes and is not the p-value for the actual primary null and alternative hypothesis for testing the non-inferiority
Unconditional exact test for non-inferiority for a binomial difference
The predefined non-inferiority margin was (-5%; +□).

Examination of the reason for the rejection of the null hypothesis on a per subject basis showed that nine of the 200 vaccinated subjects in the Group 4 FA dataset did not meet the criterion of seroconversion at Visit 2 (see Section 16.2.6). Table 11-3 gives a detailed listing of all Group 4 subjects in the FA dataset who did not meet the definition of seroconversion at Visit 2, two weeks after the vaccination, including information on gender, age (see also Section 16.2.4.1) and individual history of smallpox vaccination (see also Section 16.2.4.2).

Although four of these subjects (nos. 010, 060, 252 and 411) showed a titer increase after vaccination with IMVAMUNE®, they did not meet the stringent seroconversion criterion of a two-fold titer increase at Visit 2 presenting already high titers at baseline. Three subjects (nos. 016, 043 and 115) with baseline titers below the detection limit reached seroconversion only four weeks after the vaccination (Visit 3) with relatively low titers of 100, 50 and 100, respectively. One subject (no. 185) with a baseline titer of 50 experienced no titer increase after vaccination (Visit 2: 1, Visit 3: 50). Only one subject (no. 181) had no measurable titers at all examined time-points.

Table 11-3 Group 4 subjects in the FA dataset not meeting the criterion of seroconversion at Visit 2

Subject No.	Gender	Age on signing informed consent	V1	V2	V3	Typical vaccination scar	Vaccination date
			ELISA specific antibody titer				
010	Female	50	1011	1533	1135	Yes	25-Apr-58<br>3 Mar 66
016	Male	28	1	1	100	Yes	Unknown
043	Female	22	1	1	50	Yes (BCG)[1]	1984
060	Female	46	358	589	495	Yes	28-Apr-61
115	Female	26	1	1	100	Yes (BCG)[1]	Unknown
181	Male	32	1	1	1	Yes	Unknown
185	Female	26	50	1	50	Yes	(USSR)
252	Female	41	209	287	250	Yes	1970
411	Female	47	1169	1373	731	Yes	Unknown

[1] In subject no. 043 the vaccination scar was a result of a documented BCG vaccination; in subject no. 115 the vaccination status remained unclear, but the vaccination scar was likely caused by a previous BCG vaccination.
Source: Sections 16.2.6.1, 16.2.4.1 and 16.2.4.2.

In the PP dataset subjects no. 043 and no. 115 were excluded, because the vaccination scar was definitely (no. 043) or probably (no. 115) the result of a BCG vaccination (see Section 10.2). Both subjects had no measurable antibody titer at baseline.

Therefore, a total of seven subjects (3.6%) in the Group 4 PP dataset did not meet the criterion for seroconversion of a two-fold titer increase or an antibody titer of ≥ 50 (in subjects who were seronegative at baseline) at Visit 2.

11.4.1.4 Anti-vaccinia antibody responses measured by PRNT

Neutralizing antibodies were measured by PRNT. The cut-off of the PRNT was an antibody titer of 6, i.e. antibody titers below 6 were considered seronegative and given the arbitrary value of 1 in all analyses.

11.4.1.5 PRNT specific antibody titers

The PRNT specific GMT was calculated similar to the ELISA specific GMT. The GMTs for Groups 1-4 derived from PRNT specific antibody titers for Visits 1-5 (Groups 1-3) or Visits 1-3 (Group 4) and the follow-up visits are illustrated in Figure 11-5. The change over time is shown in Figure 11-6.

In Groups 1-3, baseline PRNT specific titers below or at the lower detection limit (6) were measured in the majority of the subjects. Only seven subjects had an elevated titer greater than 6 at baseline.

After the first vaccination with MVA-BN® the GMT in Group 1 gradually increased from a GMT of 1.125 at baseline to 4.780 at two weeks and 7.544 at four weeks after the vaccination. As expected, subjects in Group 2 responded to the first vaccination with MVA-BN® nearly

identically as subjects in Group 1. The GMT in Group 2 increased from 1.055 at baseline to 5.118 at two weeks and 7.189 at four weeks after the first vaccination.

The second vaccination with MVA-BN® in subjects in Group 1 effected a rapid increase of the GMT to 45.577 only two weeks after the second vaccination. Two weeks after this peak response the GMT slightly decreased to 34.007. At the follow-up visit 26-30 weeks after the first vaccination the GMT was still at approx. the same level as four weeks after the first vaccination (7.236).

Subjects in Group 2 who received placebo as the second injection, showed no booster effect as observed in Group 1. The GMT slowly decreased after the peak at 4 weeks after the vaccination with MVA-BN®, to 5.878 at two weeks and 4.192 at four weeks after the placebo injection. A slightly elevated GMT of 1.871, compared to a baseline value of 1.055, could still be observed at follow-up.

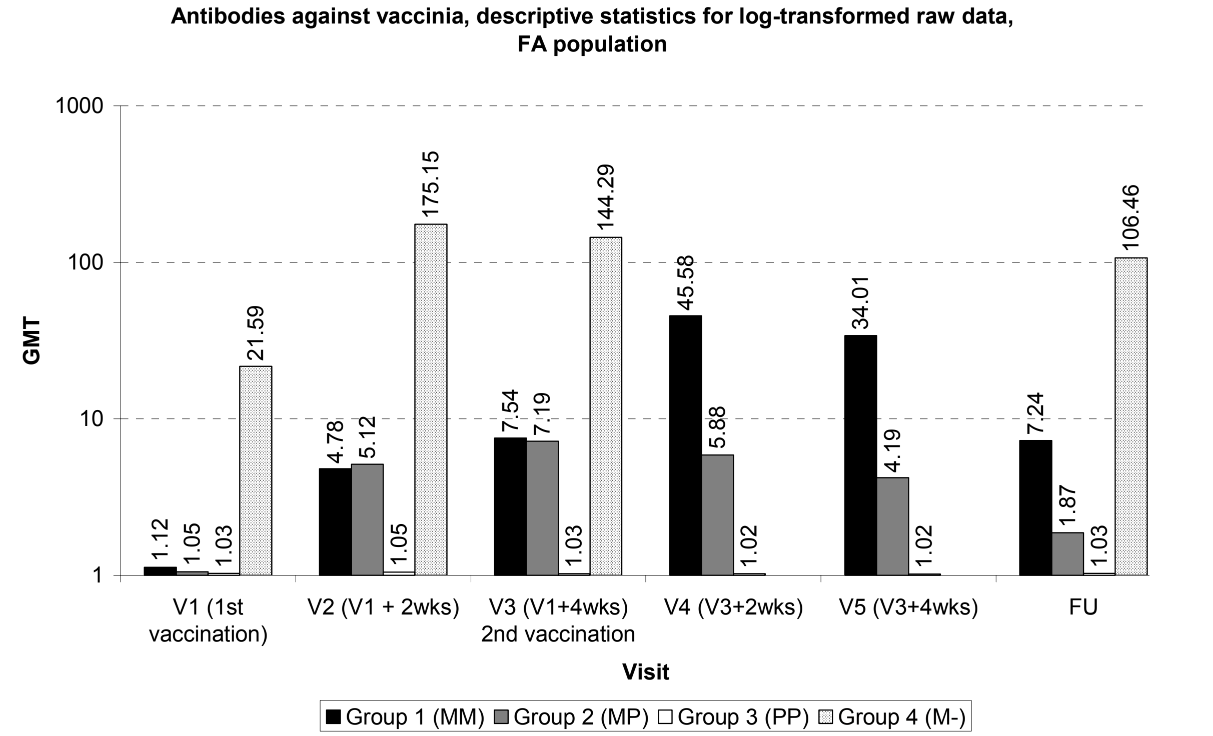

Figure 11-5 Vaccinia-specific antibody response, measured by PRNT; FA dataset
Source: Section 14.2.2.5

Throughout the study no neutralizing antibody titers were observed in subjects in the placebo group (Group 3), except for sporadic titers at the lower detection limit (6) in a total of three subjects and one subject (Subject 594) with pronounced titers observed at every visit. In this subject elevated titers had also been measured by ELISA (see Section 11.4.1.1.1). The GMTs in this group ranged from 1.020 to 1.050.

Vaccinia-experienced subjects in Group 4 had, as expected, a marked PRNT baseline GMT of 21.586. Exposure to the study vaccine MVA-BN® initiated a rapid increase in the neutralizing antibody titers. Two weeks after the vaccination a GMT of 175.150 was measured that only

slightly decreased to 144.285 two weeks later. At approx. 6 months after the vaccination the GMT was still elevated (106.463).

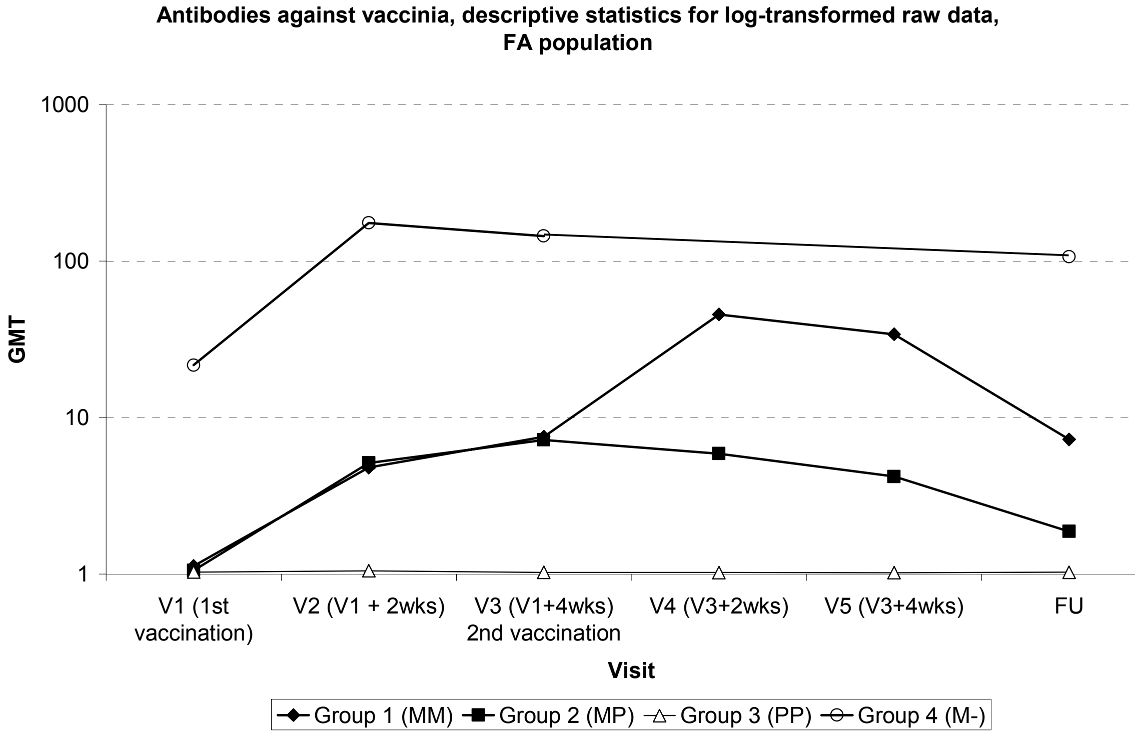

Figure 11-6 Vaccinia-specific antibody response measured by PRNT /Time response; FA dataset
Source: Section 14.2.2.5

11.4.1.6 Seroconversion derived from PRNT specific antibody titers

Seroconversion derived from neutralizing antibody titers was defined as an antibody titer of ≥ 6 (and not 20, as specified in the protocol) in subjects who were seronegative at baseline, or a two-fold increase of the baseline antibody titer in subjects showing an antibody response against vaccinia at baseline.

Seroconversion rates are tabulated in Section 14.2.2.1 for the FA dataset and in Section 14.2.2.2 for the PP dataset; corresponding Figures are provided in Sections 14.2.2.7 and 14.2.2.8 for the FA dataset and in Sections 14.2.2.9 and 14.2.2.10 for the PP dataset. Individual information about seroconversion sorted by subject and visit is provided in Section 16.2.6.2.

Of the subjects in Groups 1 and 2 who had received one dose of MVA-BN®, 45.1% and 52.0% respectively had seroconverted at two weeks and 56.7% and 62.1% respectively had seroconverted at four weeks after the first vaccination. A second dose of MVA-BN® elicited a maximum seroconversion of 89.2% of the subjects in Group 1 two weeks after the second vaccination. Four weeks after the second dose of MVA-BN® the seroconversion rate was still at 86.0%.

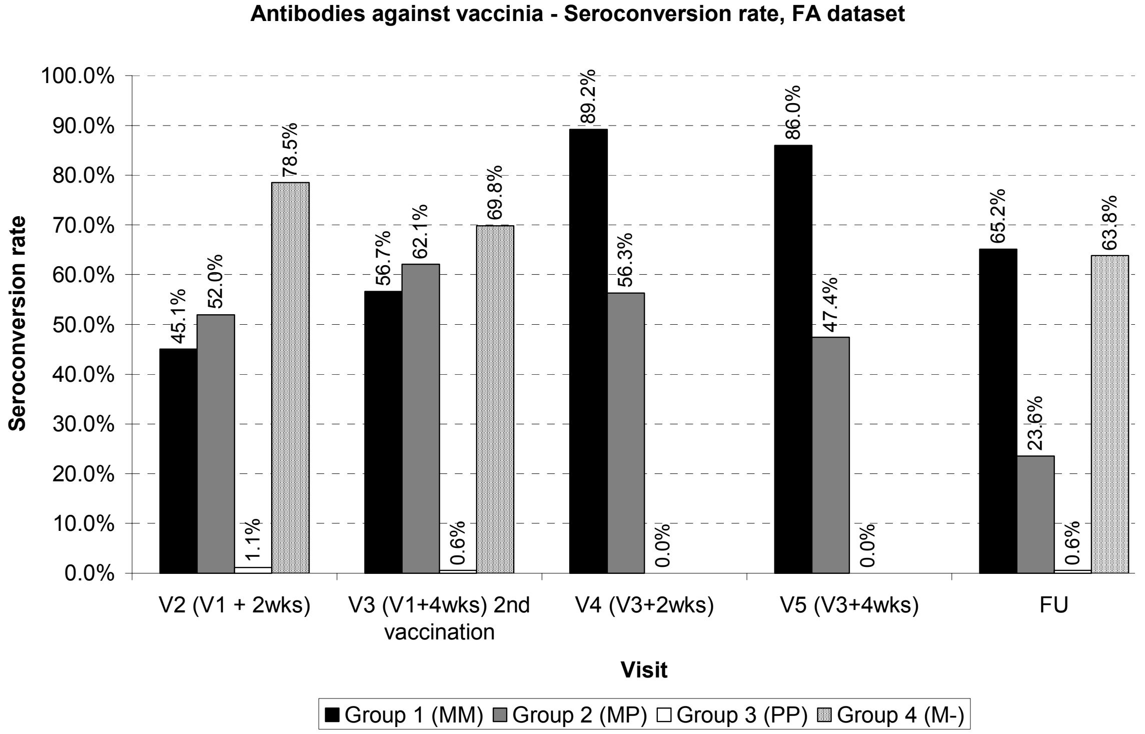

Figure 11-7 Seroconversion rate, measured by PRNT, FA dataset
Source: Section 14.2.2.7

In subjects in Group 2 seroconversion was maximal at four weeks after the vaccination with MVA-BN® (62.1%). Following the placebo treatment at Visit 3, the number of subjects remaining positive for seroconversion decreased to 56.3% at Visit 4, 47.4% at Visit 5 and to 23.6% at follow-up.
Sporadic titers measured in a very low number of subjects in Group 3 account for a seroconversion rate ranging between 0.0% and 1.1%.

A single dose of MVA-BN® elicited a maximum seroconversion of 78.5% of the vaccinia-experienced subjects in Group 4 at two weeks after the vaccination. Four weeks after the vaccination 69.8% of the subjects still met the criteria for seroconversion.

At follow-up, approx. six months after the last vaccination a similar percentage of subjects in Group 1 and 4 still had neutralizing antibody titers meeting the criteria for seroconversion: 65.2% in Group 1 and 63.8% in Group 4.

In Figure 11-8 below only the GMTs at two and four weeks after the last vaccination and at follow-up are shown.

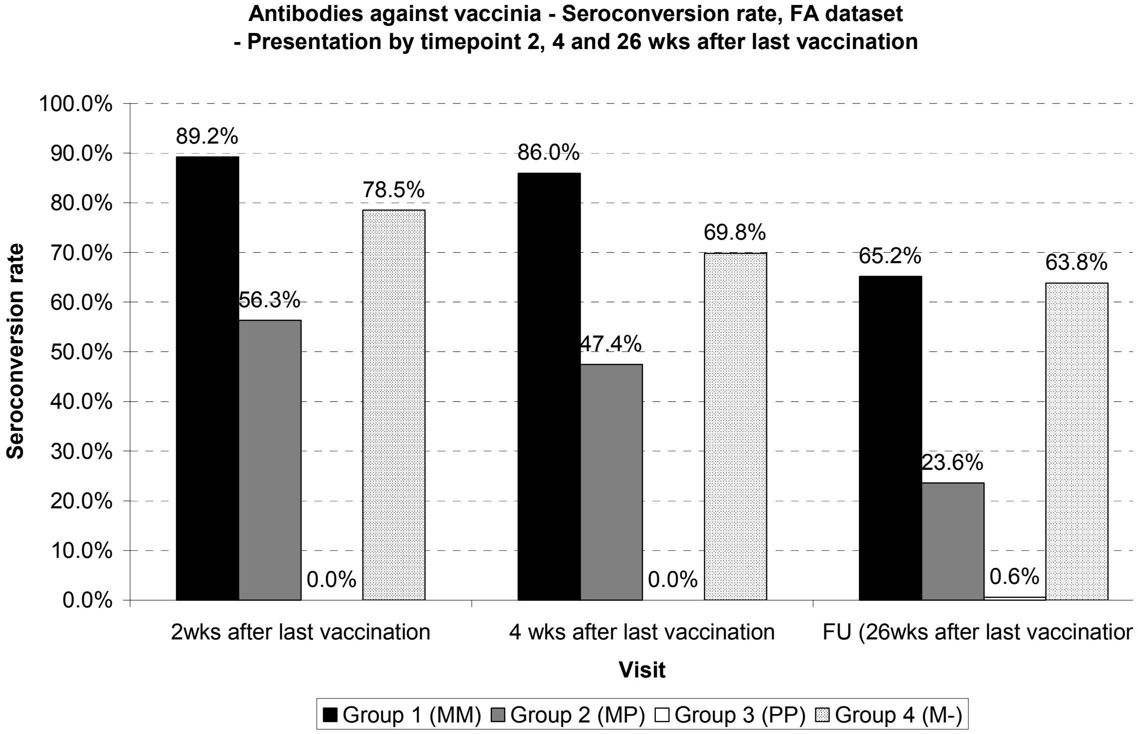

Figure 11-8 Seroconversion rate, measured by PRNT, two and four weeks after last vaccination, FA dataset
Source: Section 14.2.2.8

Comparing the seroconversion rates of Group 1 and Group 4 (see Figure 11-8) a significant difference of 10.7% (p-value: 0.0054) at two weeks and 16.2% (p-value: 0.0002) at four weeks after the last vaccination was observed in the FA dataset (see Table 11-4 and Table 11-5). Similar significant differences were observed in the PP dataset (9.9% at two weeks and 16.9% at four weeks after the vaccination).

It could not be statistically shown that the PRNT specific seroconversion rate in Group 4 was non-inferior to the seroconversion rate in Group 1 in the FA and the PP dataset at two and four weeks after the last vaccination. The confidence intervals provided in Table 11-4 and Table 11-5 exceeded the pre-defined margin of [-5%; +□]; p-values were all >0.9 and therefore not significant (p-values <0.05 are significant).

Table 11-4 Primary efficacy parameter: Vaccinia-specific seroconversion rate derived from the PRNT specific antibody titers 2 weeks after the last vaccination (Group 1-3: Visit 4, Group 4: Visit 2)

Population	Seroconversion rate Group 1 (MM)	Seroconversion rate Group 4 (M-)	Difference Group 4 – Group 1	p-value for the test of the null hypothesis of equality of proportions (Fisher's exact test)	p-value for the rejection of the null hypothesis of inferiority	Exact one-sided 97.5% confidence interval for the difference of rates between Group 4 and Group 1
FA	89.2% (157/176)	78.5% (157/200)	-10.7%	**0.0054**	0.9353	(-18.18%; +∞)
PP	88.7% (149/168)	78.8% (152/193)	-9.9%	**0.0155**	0.9017	(-17.60%; +∞)

[1] This was only done for descriptive purposes and is not the p-value for the actual primary null and alternative hypothesis for testing the non-inferiority
Unconditional exact test for non-inferiority for a binomial difference
The predefined non-inferiority margin was (-5%; +□).
Significant p-values are bolded.

Table 11-5 Secondary efficacy parameter: Vaccinia-specific seroconversion rate derived from the PRNT specific antibody titers 4 weeks after the last vaccination (Group 1 3: Visit 5, Group 4: Visit 3)

Population	Seroconversion rate Group 1 (MM)	Seroconversion rate Group 4 (M-)	Difference Group 4 – Group 1	p-value for the test of the null hypothesis of equality of proportions (Fisher's exact test)	p-value for the rejection of the null hypothesis of inferiority	Exact one-sided 97.5% confidence interval for the difference of rates between Group 4 and Group 1
FA	86.0% (153/178)	69.8% (139/199)	-16.2%	**0.0002**	0.9957	(-24.40%; +∞)
PP	85.7% (144/168)	68.8% (132/192)	-16.9%	**0.0002**	0.9969	(-25.54%; +∞)

[1] This was only done for descriptive purposes and is not the p-value for the actual primary null and alternative hypothesis for testing the non-inferiority
Unconditional exact test for non-inferiority for a binomial difference
The predefined non-inferiority margin was (-5%; +□).
Significant p-values are bolded.

In Group 4 a total of 43 of 200 subjects in the FA dataset (see Table 11-6 below) had not seroconverted two weeks after the vaccination with MVA-BN®. Of these 43 subjects however, 17 subjects had seroconverted at four weeks after the vaccination and 7 subjects had seroconverted at the follow-up visit (approx. six months after the vaccination) (see shaded fields in Table 11-6). In these subjects initiation of neutralizing antibodies simply took longer than two weeks. Out of the 43 subjects in Group 4 that failed to meet the seroconversion criteria two weeks post vaccination, only 19 subjects actually failed to seroconvert at all study visits.

Subjects 043 and 050 were excluded from the PP dataset, because they had been allocated into Group 4 on the basis of a vaccination scar that however was the result of a documented BCG vaccination.

Table 11-6 Subjects in Group 4 (FA dataset) not meeting the criterion of seroconversion at Visit 2

Subject No.	Gender	Age on signing informed consent	V1	V2	V3	FU	V2	V3	FU	Vaccination date
			PRNT specific antibody titer				Seroconversion			
006	female	44	69	61	104	148	no	no	yes	1971
016	male	28	1	1	79	1	no	yes	no	Unknown
017	female	49	44	49	299	227	no	yes	yes	Unknown
022	female	46	6	6	136	102	no	yes	yes	01MAR1960<br>15FEB1971
029	male	43	6	6	363	40	no	yes	yes	Unknown
043	female	22	1	1	30	1	no	yes	no	1984
048	male	33	9496	6513	5661	6351	no	no	no	Unknown
050	female	26	1	1	36	1	no	yes	no	Unknown
063	female	43	128	161	135	41	no	no	no	Unknown
097	female	48	184	203	333	173	no	no	no	Unknown
117	male	27	203	325	585	227	no	yes	no	Unknown
122	male	37	49	86	68	69	no	no	no	Unknown
132	female	47	97	142	148	176	no	no	no	Unknown
134	female	38	253	324	321	216	no	no	no	Unknown
139	male	51	57	88	244	168	no	yes	yes	Unknown
145	female	44	166	236	837	205	no	yes	no	14MAY1973
151	male	49	381	457	304	122	no	no	no	Unknown
157	female	46	99	160	153	6	no	no	no	09JUN1971
181	male	32	1	1	6	1	no	yes	no	Unknown
183	male	52	213	109	62	126	no	no	no	Unknown
242	female	54	27	51	247	130	no	yes	yes	Unknown
243	female	36	148	222	284	171	no	no	no	15JUN1971
252	female	41	183	32	6	80	no	no	no	1970
257	female	29	114	209	500	189	no	yes	no	11MAY1978
261	male	46	37	45	1	6	no	no	no	Unknown
262	female	27	78	78	45	66	no	no	no	02OCT1979
269	female	42	110	64	40	191	no	no	no	15FEB1966<br>30APR1976
270	female	41	47	88	221	58	no	yes	no	Unknown

Subject No.	Gender	Age on signing informed consent	V1	V2	V3	FU	V2	V3	FU	Vaccination date
			PRNT specific antibody titer				Seroconversion			
279	male	48	199	251	27	168	no	no	no	23AUG1978
286	male	46	87	6	6	103	no	no	no	27JUN1973<br>29APR1974
289	male	52	72	26	125	132	no	no	no	Unknown
291	female	36	96	164	68	95	no	no	no	Unknown
298	male	36	110	69	401	209	no	yes	no	18JUL1971
299	male	32	217	134	311	598	no	no	yes	11MAR1977<br>23APR1980
302	female	38	6	6	43	74	no	yes	yes	Unknown
311	female	45	104	61	159	222	no	no	yes	Unknown
314	female	46	96	6	49	306	no	no	yes	Unknown
315	male	43	50	6	96	206	no	no	yes	13FEB1963<br>21MAY1974
324	female	51	26	1	1	6	no	no	no	17APR1956<br>02MAY1967
333	female	39	24	6	6	49	no	no	yes	Unknown
359	female	53	32	6	31	80	no	no	yes	Unknown
411	female	47	231	423	527	249	no	yes	no	Unknown
451	male	47	81	157	225	200	no	yes	yes	Unknown

Subjects meeting the criteria for seroconversion at Visit 3 or at follow-up are highlighted by shading.
Source: Sections 16.2.4.1 and 16.2.6.2

In comparison in Group 1 nineteen of 176 subjects had not seroconverted with regard to PRNT specific antibody titers at two weeks after the second vaccination. Similar to Group 4, of these 19 subjects, 17 subjects seroconverted at a later time point (12 subjects had seroconverted at four weeks after the second vaccination and five subjects had seroconverted at the follow-up). Only two subjects in Group 1 (Subjects nos. 236 and 525) did not meet the criteria for seroconversion at either Visit 4, Visit 5 or at follow-up. Subject 525 was however positive for seroconversion at Visit 2.

Table 11-7 Subjects in Group 1 (FA dataset) not meeting the criterion of seroconversion at Visit 4

Subject No.	Gender	Age on signing informed consent	V1	V4	V5	FU	V4	V5	FU
			PRNT specific antibody titer				Seroconversion		
129	female	20	1	1	25	6	no	yes	yes
140	male	28	1	1	1	45	no	no	yes
205	female	30	1	1	1	6	no	no	yes
215	female	23	1	1	1	28	no	no	yes
236	female	19	1	1	1	1	no	no	no
254	male	25	1	1	1	26	no	no	yes
283	female	21	1	1	55	125	no	yes	yes
317	male	45	113	213	168	232	no	no	yes
323	female	24	1	1	6	36	no	yes	yes
386	female	29	1	1	6	1	no	yes	no
432	female	28	1	1	25	38	no	yes	yes
447	female	31	1	1	49	6	no	yes	yes
449	female	29	1	1	6	1	no	yes	no
455	male	28	1	1	99	1	no	yes	no
495	female	22	1	1	62	22	no	yes	yes
521	male	29	1	1	155	6	no	yes	yes
525	male	27	1	1	1	1	no	no	no
537	male	31	1	1	140	25	no	yes	yes
572	female	24	1	1	66	1	no	yes	no

Subjects meeting the criteria for seroconversion at Visit 3 or at follow-up are highlighted by shading.
Source: Sections 16.2.4.1 and 16.2.6.2

11.4.1.7 Correlation analysis of ELISA and PRNT specific antibody titers

The log10-transformed antibody titers derived from ELISA and PRNT analysis were tested for correlation by treatment group and visit (see Section 14.2.3). Group 3 was excluded from the correlation analysis, because, as mentioned above, the majority of the subjects in this group were seronegative throughout the study. Correlation coefficients, p-values and the number of correlated values per analysis are listed in Table 11-8.

Table 11-8 Correlation analysis between ELISA and PRNT antibody titers

Treatment Group	Visit	Correlation Coefficient	P-value	N
Group 1	1	0.57820	<0.0001	183
	2	0.43279	<0.0001	182
	3	0.27155	0.0002	180
	4	0.13852	0.0667	176
	5	0.37400	<0.0001	178
	Follow-up	0.46432	<0.0001	178
Group 2	1	0.50514	<0.0001	181
	2	0.45265	<0.0001	179
	3	0.30325	<0.0001	174
	4	0.31201	<0.0001	174
	5	0.44278	<0.0001	175
	Follow-up	0.46797	<0.0001	174
Group 4	1	0.56938	<0.0001	200
	2	0.62532	<0.0001	200
	3	0.47583	<0.0001	199
	Follow-up	0.75181	<0.0001	198

Source: Section 14.2.3

ELISA and PRNT specific titers measured in Groups 1, 2 and 4 correlated significantly, as demonstrated by p-values < 0.05 at all time points except for Visit 4 in Group 1. Significant correlation coefficients ranged from 0.27155 to 0.57820 in Group 1, 0.30325 to 0.50514 in Group 2 and from 0.47583 to 0.75181 in Group 4. Surprisingly, no significant correlation was observed in Group 1 at Visit 4 (correlation coefficient of 0.13852, p-value: 0.0667), however, this p-value was very close to the significance level. Since all other correlation coefficients were highly significant this can be classified as a statistical artifact.

Correlation plots (PRNT specific titers versus ELISA specific titers on a logarithmic scale) are provided in Section 14.2.3.

11.4.2 STATISTICAL/ANALYTICAL ISSUES

The statistical analysis was performed according to the statistical analysis plan (SAP) Version 2.0 (see Section 16.1.9). In addition a further exploratory subgroup analysis by age and gender regarding seroconversion was performed.
Seroconversion derived from PRNT specific antibody titers was defined as an antibody titer ≥6 for subjects seronegative at baseline and not as an antibody titer ≥20, as specified in the protocol.

Each subject number was allocated to one of the four treatment sequences according to the randomisation list.
Each subject was allocated to one of the three sequences (Groups 1-3). A separate list of vials ranging from 0001 to 2394 assigned each vial number to one of the two treatments ‘MVA-BN’ and ‘Placebo’.
The block length for the allocation of sequences was 9, i.e. after 9 subjects, 3 in each sequence group were allocated, the block length for the vial list was 18, i.e. after 18 vials, 9 of each treatment were used.

The subject randomisation number used in the randomization list could not be used in the e-CRF for technical reasons. Therefore, while the treatment sequence was stringently followed, the e-CRF system assigned consecutive numbers starting with 001 (see Section 16.1.9).

11.4.3 TABULATION OF INDIVIDUAL RESPONSE DATA

Individual response data is listed in Section 16.2.

11.4.4 DRUG DOSE, DRUG CONCENTRATION RELATIONSHIPS TO RESPONSE

Not applicable, because the drug dose was constant.

11.4.5 DRUG-DRUG AND DRUG-DISEASE INTERACTIONS

Not applicable, because in this study healthy subjects were vaccinated against smallpox to achieve a prophylactic protection against smallpox.

11.4.6 BY-PATIENT DISPLAYS

By-patient displays are provided in Section 16.2.

11.4.7 EFFICACY CONCLUSIONS

One vaccination of vaccinia-naïve healthy individuals with MVA-BN® resulted in a measurable ELISA specific seroconversion rate of 70.9% in Group 1 and 72.6% in Group 2 subjects two weeks after the first vaccination. Therefore, about one third of the vaccinia-naïve subjects did not reach measurable seroconversion at this early timepoint. However, comparing the immune response of these subjects after the second vaccination to the immune response of vaccinia-naïve subjects after the first vaccination with IMVAMUNE®, it could be shown that antibody titers measured by ELISA for these subjects 14 days after the **second** vaccination (Visit 4, Day 42) were higher compared to the average titers of vaccinia-naïve subjects with a measurable response at Study Visit 2 (14 days after the first vaccination). From this observation it can be concluded that even for subjects who did not show a measurable response after the first vaccination, a priming of the immune system had still occurred, albeit at a level below the detection limit of the assays. Following a vaccination the immune response of these individuals could be boostered to high antibody titers.

Two vaccinations with MVA-BN® in subjects without a history of smallpox vaccination (Group 1) were shown to result in a seroconversion rate of 98.9% at both two and four weeks after the second vaccination. In comparison, a single vaccination with MVA-BN® in subjects with a history of smallpox vaccination (Group 4) was sufficient to result in a seroconversion rate of 95.5% that statistically was not significantly different from the Group 1 peak response. Four weeks after the vaccination the percentage of subjects meeting the definition of seroconversion in the vaccinia-experienced study population (Group 4) had only slightly decreased to 93.0%.
Statistically, however, non-inferiority with regard to the seroconversion rate in vaccinia-experienced subjects compared to the seroconversion rate in initially vaccinia-naïve subjects

could not be confirmed. Taking a closer look to this part of Group 4 subjects who missed the primary endpoint, a total of nine subjects in the Group 4 FA dataset (n= 200) did not meet the criterion for seroconversion at Visit 2. However, three (subject nos. 016, 043 and 115) of these nine subjects reached the titer defining seroconversion only four weeks after the vaccination indicating that some individuals could need longer to provide a measurable humoral immune response reflecting biological diversity. Two of these three subjects (nos. 043 and 115) were excluded from the PP dataset because the presented vaccination scar were suspected (subject 115) or confirmed (subject 043) as derived from a BCG vaccination.

From the six remaining subjects, four (subject nos. 010, 060, 252 and 411) showed a titer increase at Visit 2, which, however, did not meet the criterion of a two-fold titer increase. Nevertheless, these titer increases reflect a clear booster reaction of the immune system induced by the vaccination with MVA-BN®. It is assumed that subjects with relatively high (>500) baseline titers cannot double their titers compared to baseline, whereas they still show some titer increase representing a booster response. For these subjects, the study protocol definition of a two-fold titer increase to fulfill seroconversion was clearly set too strict; this observation should be taken into consideration for future study protocols evaluating immunogenicity parameter of vaccinia-experienced subjects.

Only two subjects (nos. 181 and 185) of Group 4 did not show any increase in antibody titers and could be determined as vaccination failures. One case of vaccination failure was also observed in the vaccinia-naive group (Subject no. 102 in Group 1).

The weakness of setting a 2-fold increase in titer as a definition of seroconversion for people previously vaccinated against smallpox, was further demonstrated to be biologically irrelevant when the GMT between the groups were compared. Although the lower seroconversion rate in Group 4 compared to Group 1 implied a lower relative change in antibody titers in Group 4, the vaccinia-specific peak GMT was in fact higher in the Group 4 subjects. A peak GMT of 568.819 in Group 4 was measured two weeks after the MVA-BN® vaccination (Visit 2) and a GMT of 495.843 in Group 1 two weeks after the second MVA-BN® vaccination (Visit 4). These peak GMTs decreased to 452.321 in Group 4 and 328.666 in Group 1 at the individual last study visit two weeks later (Visit 3 for Group 4 and visit 5 for Group 1).

A further statistical analysis of the relationship of gender and age to seroconversion was performed revealing no significant differences.

As seen for two subjects in Group 4 (010 and 411) that had high initial antibody titers, there is a clear indication that the ability to increase the individual antibody titer by a factor of 2 decreases with a high baseline titer. With regard to the observed limitations to boost antibody titers that are still high even some decades after immunization with a traditional smallpox vaccine, the criteria for seroconversion as defined for the vaccinia-experienced population was likely too stringent and did not take into account high starting titers or the biological diversity in the time for individuals to seroconvert. Indeed, the fact that significant increases in GMT (by both ELISA and PRNT) were observed in both vaccinia naïve and experienced subjects would suggest that the criteria set for seroconversion has little biological relevance for evaluating the immunogenicity of MVA-BN®.

Similarly, only 78.5% of the vaccinia-experienced subjects seroconverted by PRNT, 2 weeks post vaccination with MVA-BN®. However, out of the 43 non-seroconverters (2-fold increase) in Group 4, 19 would actually have fulfilled the criteria at later visits; clearly indicating that MVA-BN® vaccination induced a strong booster response to the neutralizing antibodies in this

population. Seroconversion rates were actually higher in the vaccinia naïve subjects (Group 1) after the second vaccination, which again may be accounted by the strict criteria set for seroconversion particularly for the vaccinia-experienced subjects. Indeed, this was again highlighted by comparing the GMT generated by PRNT. The GMT in Group 1, 2 weeks post the secondary vaccination with MVA-BN® was 45.577, compared to 175.150 2 weeks post MVA-BN® vaccination in vaccinia-experienced subjects (Group 4).

Importantly, when all outliers were taken into account the ELISA and PRNT titers were highly correlated at all visits for all groups. In general the GMT and seroconversion rates were lower by PRNT compared to ELISA and this is probably related to the fact the PRNT is only measuring a component of the total antibody responses measured by the ELISA. Moreover, the ELISA represents a more sensitive assay compared to the PRNT.

In conclusion, humoral immune response measured by ELISA demonstrated that two vaccinations with MVA-BN® resulted in seroconversion of almost all vaccinia-naïve subjects (98.8%) and that one dose of MVA-BN® in vaccinia-experienced subjects elicited an immunologically relevant increase of vaccinia-specific antibody titers leading to a more than two-fold increase (or at titer ≥50 in subjects being seronegative at baseline) in >95% of the vaccinia-experienced subjects. Similarly, seroconversion rates by PRNT were also seen in the majority of the vaccinia naïve subjects following two vaccinations (89.2%), which were also seen in the majority of the vaccinia-experienced subjects (78.5%).

Approx. six months after the second vaccination the GMT derived from ELISA and PRNT in the subjects who received two doses of MVA-BN® was still at 27.900 and 7.236 respectively, which are in the same order of magnitude at the antibody levels recorded in vaccinia-experienced subjects at baseline. In these subjects re-exposure to the vaccinia virus might induce a similar booster effect as observed in the subjects in Group 4. In Group 4, the GMT by both ELISA and PRNT at the follow-up visit (179.907 and 106.463 respectively) were still markedly higher than the baseline titers.

12 SAFETY EVALUATION

12.1 EXTENT OF EXPOSURE

A total of 745 randomized healthy subjects received at least one vaccination (one injection of MVA-BN® or placebo) and were included in the safety analysis.

A total of 564 subjects received at least one dose of 1 x 10^8 $TCID_{50}$ MVA-BN®. Of the vaccinia-naïve subjects, 180 subjects (Group 1) received two doses of MVA-BN® and 3 subjects from Group 1 as well as 181 subjects from Group 2 received a single dose of MVA-BN®. Two hundred vaccinia-experienced subjects (Group 4) received one dose of MVA-BN®.

The following subjects received placebo: 174 subjects in Group 2 received placebo with the second injection. In Group 3 six subjects received only one injection with placebo and 175 subjects received two injections of placebo (see Section 14.1.2).

The mean duration between the two injections in the respective Groups 1-3 was 30.1-30.4 days (see Section 14.6.4).

12.2 ADVERSE EVENTS (AEs)

Summary tables for adverse event (AE) data are provided in Section 14.5, individual AE data are listed in Section 16.2.7. Individual follow-up AE data are provided in Section 16.2.10.1.

12.2.1 BRIEF SUMMARY OF ADVERSE EVENTS

In this study unsolicited and solicited adverse events were assessed. Unsolicited adverse events were recorded by the investigator at every study visit by asking the subject if he/she had experienced any change in health status since his/her last visit. Solicited adverse events were recorded by the subjects in the diary card and transferred to the CRF. After each injection the subjects monitored defined local and general symptoms and recorded any additional symptom that occurred under the category "other" beginning on the day of vaccination (Day 0) and for the following 7 days or longer, if the event lasted more than 7 days.
At the follow-up visit AEs that had not resolved at the last visit of the active study phase (Groups 1-3: Visit 5; Group 4: Visit 3) were followed-up and any SAEs and SIAEs were recorded.

12.2.1.1 Unsolicited adverse events

12.2.1.1.1 Unsolicited adverse events observed during the active study phase

A total of 718 adverse events were verbatim reported during the active phase of this study (Group 1: Visits 1-5; Group 4: Visits 1-3): 241 AEs in Group 1, 188 in Group 2, 136 in Group 3 and 153 in Group 4 (See Table 12-1).
Of the subjects who received at least one dose of MVA-BN®, 113 subjects (61.7%) in Group 1, 107 subjects (59.1%) in Group 2 and 99 subjects (49.5%) in Group 4 experienced at least one

adverse event. In the placebo group (Group 3, one or two doses of placebo) 78 subjects (43.1%) experienced at least one adverse event.

The majority of the unsolicited adverse events were mild or moderate (Grades 1 and 2). There were only 18 cases of severe (Grade 3) unsolicited AEs (see Section 14.5.1.7).

33.3%- 37.0% of the subjects in Groups 1, 2 and 4, compared to 12.7% of the subjects in Group 3 (placebo), experienced at least one AE that was judged as treatment-related (definitely, probably, possibly or unknown) by the investigator while still blinded to the treatment allocation.

One serious adverse event (SAE) in Group 1 (unlikely related to study vaccine) and two SAEs in two subjects in Group 2 (one event not related, the other unlikely related to study vaccine) were reported during the active phase of this study. Two of these subjects completely recovered. 1 subject in Group 2 had an ongoing rupture of a finger tendon.

All subjects were particularly monitored with regard to ECG changes and cardiac symptoms. Any cardiac symptom, clinically significant ECG changes or cardiac enzymes elevated above ULN (upper limit of normal) were recorded as an “adverse event of special interest” (SIAE). In this study a total of 11 SIAEs were observed in 10 subjects: 3 SIAEs each in Groups 1 and 2, 1 SIAE in Group 3 and 4 SIAEs in Group 4. These included 6 cases of palpitations, 3 cases of tachycardia and 2 cases of sinus tachycardia. 9 subjects completely recovered. One subject in Group 3 (placebo) continued to experience palpitations.

Two subjects were withdrawn after Visit 2 because of adverse events. Subject 536 (Group 2) experienced an SAE (transient motoric hemiparesis, see Section 12.3.2.1) assessed as being unlikely related to study vaccine. In subject 580 (Group 3, placebo) a mild pericardial effusion was incidentally detected. The event was not classified as an SIAE, because the cardiologist assessed the event to be not clinically significant (see also Section 12.4.1.3).

Table 12-1 Unsolicited Adverse Events: Overview, Safety dataset, active study phase

	Group 1 (MM)		Group 2 (MP)		Group 3 (PP)		Group 4 (M-)		All subjects	
	Count	%	Count	%	Count	%	Count	%	Count	%
**Total no. of subjects included**	183	24.6	181	24.3	181	24.3	200	26.8	745	100.0
**No. of subjects with at least one AE**	113	61.7	107	59.1	78	43.1	99	49.5	397	53.3
**No. of subjects with at least one treatment-related[1] AE**	61	33.3	61	33.7	23	12.7	74	37.0	219	29.4
**No. of subjects with no AE**	70	38.3	74	40.9	103	56.9	101	50.5	348	46.7
**No. of subjects with at least one SAE**	1	0.5	2	1.1	-	-	-	-	3	0.4
**No. of subjects with at least one related SAE**	-	-	-	-	-	-	-	-	-	-
**No. of subjects with at least one SIAE**	3	1.6	3	1.7	1	0.6	3	1.5	10	1.3
**No. of subjects with at least one related SIAE**	-	-	2	1.1	-	-	2	1.0	4	0.5
**No. of verbatim reported AEs**	241	33.6	188	26.2	136	18.9	153	21.3	718	100.0
**No. of reported SAEs**	1	33.3	2	66.7	-	-	-	-	3	100.0
**No. of reported SIAEs**	3	27.3	3	27.3	1	9.1	4	36.4	11	100.0
**No. of coded MedDRA PTs[2]**	260	34.5	197	26.2	141	18.7	155	20.6	753	100.0
**No. of Preferred Terms (PTs) by subject[3]**	229	32.3	193	27.3	135	19.1	151	21.3	708	100.0

Percentages for 'No. of subjects with...' are based on the total number of subjects included per treatment
[1] Treatment-related: definite, probable, possible or unknown relationship to study vaccine
[2] This includes all coded symptoms including multiple occurrences of the same PT within one subject
Splitting of verbatim reported AEs possible.
[3] A specific PT was only counted once even when it occurred more than one time in the same subject.
Source data: Table 14.5.1.3

12.2.1.2 Unsolicited adverse events observed during follow-up

During the 6-months follow-up after the last vaccination (Visit 3 for Groups 1-3, Visit 1 for Group 4) nine SAEs and five SIAEs were reported (see Section 14.5.5.2). Of these events, however, only one SAE (ongoing sarcoidosis in Subject 521 in Group 1) and no SIAE were considered to be related to the study treatment by the investigator (see Section 14.5.5.1). Please refer to Section 12.3.1 for a detailed description of these cases.

In one subject a previously ongoing AE was updated. In subject 067 (Group 3) iron deficiency was reported at follow-up; the investigator was of the opinion that the deficiency already existed during the active study phase and therefore the event was neither classified as initial nor as follow-up.

12.2.1.3 Solicited adverse events

Diaries monitoring solicited adverse events were available from 182 subjects in Group 1, 179 subjects each in Groups 2 and 3 and 200 subjects in Group 4. A tabulated overview of solicited adverse events is given in Sections 14.5.2 – 14.5.4; a detailed listing of solicited events by subject, symptom and vaccination is provided in Section 16.2.7.1.

12.2.1.3.1 Solicited general symptoms

After each vaccination the subjects were asked to document the occurrence of the five following general symptoms on the day of vaccination and the 7 days post-vaccination: increased body temperature, headache, myalgia, nausea and fatigue. Additionally, the grading of severity was recorded.

The frequencies described in the following apply to all subjects who experienced the respective symptom on at least one day during the 7 days after at least one of the vaccine administrations in Groups 1-3 and after the vaccination in Group 4. Of all subjects 59 subjects (8.0%) reported in their diary card an increased body temperature of 37.5°C (99.5°F) or higher, but below 39.0°C (102.2°F), i.e. all below Grade 3. Slightly more subjects (70 subjects, 9.5%) reported nausea and 113 subjects (15.3%) experienced myalgia. Most frequently headache and fatigue occurred in 246 subjects (33.2%) and 260 subjects (35.1%) of all subjects respectively (see Section 14.5.2.3).

None of the above mentioned symptoms were considered life-threatening or disabling (Grade 4). There were no episodes of severe myalgia and no cases of increased body temperature of 39°C (102.2°F) or higher. A total of 12 subjects reported Grade 3 headache, 13 subjects reported Grade 3 fatigue and a total of 6 subjects experienced Grade 3 nausea on at least one day of the 7 days after the injection(s). The proportions of severe symptoms and symptoms of severity below Grade 3 were not significantly different between the treatment groups regarding headache and fatigue, whereas Group 2 had a higher proportion of cases of Grade 3 nausea than the other groups.

Nearly no significant differences in the severity of particular adverse events were identified when comparing the four study groups. Only for increased body temperature, headache and myalgia were significant differences between the four treatment groups observed, when those subjects reporting none of the solicited symptoms (Grade 0) were compared to any symptom assigned a grade > 0, i.e. reported any symptom (see Section 14.5.2.3).

More subjects measured an increased body temperature in Groups 1 and 2 (9.9% and 11.7%) than in Groups 3 and 4 (5.6% and 5.0%). Headache was reported most frequently in subjects of Group 2 (46.9%), compared to subjects in the other groups (26.5 – 33.0%). Myalgia was most predominant in Group 4 (21.0%) and lowest in Groups 2 and 3 (12.3% and 11.2%) (see Section 14.5.2.3).

The individual numbers of subjects who experienced solicited general symptoms sorted by grade and group are listed in Table 12-2, whereas for each subject the maximum experienced intensity of the respective symptom is specified. If the subject did not experience the particular solicited symptom, he/she recorded “Grade 0” on the diary card. A p-value below 0.05 indicates a statistically significant difference in the proportions of Grade 0 (no symptom) to any symptom (Grade 1 to Grade 4) between any of the 4 treatment groups.

Table 12-2 Summary of Maximum Intensity of Solicited General Symptoms (7-day Follow-up Period after Vaccination) as Recorded in the Subject Diary, Safety dataset

	**Group 1 (N=182)**	**Group 2 (N=179)**	**Group 3 (N=179)**	**Group 4 (N=200)**	**p-value**
	**Number of subjects (%)**				
**Body temperature increased**					
Grade 0*	164 (90.1)	158 (88.3)	169 (94.4)	189 (94.5)	0.096~
Grade 1	13 (7.1)	17 (9.5)	8 (4.5)	10 (5.0)	
Grade 2	5 (2.7)	4 (2.2)	2 (1.1)	0 (0.0)	
Grade 3	0 (0.0)	0 (0.0)	0 (0.0)	0 (0.0)	
Grade 4	0 (0.0)	0 (0.0)	0 (0.0)	0 (0.0)	
Missing	0	0	0	1	
**Headache**					
Grade 0*	122 (67.0)	95 (53.1)	130 (72.6)	147 (73.5)	0.002~
Grade 1	39 (21.4)	57 (31.8)	35 (19.6)	41 (20.5)	
Grade 2	19 (10.4)	21 (11.7)	12 (6.7)	10 (5.0)	
Grade 3	2 (1.1)	6 (3.4)	2 (1.1)	2 (1.0)	
Grade 4	0 (0.0)	0 (0.0)	0 (0.0)	0 (0.0)	
**Myalgia**					
Grade 0*	153 (84.1)	157 (87.7)	159 (88.8)	158 (79.0)	0.054~
Grade 1	25 (13.7)	14 (7.8)	14 (7.8)	33 (16.5)	
Grade 2	4 (2.2)	8 (4.5)	6 (3.4)	9 (4.5)	
Grade 3	0 (0.0)	0 (0.0)	0 (0.0)	0 (0.0)	
Grade 4	0 (0.0)	0 (0.0)	0 (0.0)	0 (0.0)	
**Nausea**					
Grade 0*	165 (90.7)	157 (87.7)	166 (92.7)	182 (91.0)	0.073~
Grade 1	13 (7.1)	12 (6.7)	8 (4.5)	16 (8.0)	
Grade 2	4 (2.2)	5 (2.8)	4 (2.2)	2 (1.0)	
Grade 3	0 (0.0)	5 (2.8)	1 (0.6)	0 (0.0)	
Grade 4	0 (0.0)	0 (0.0)	0 (0.0)	0 (0.0)	
**Fatigue**					
Grade 0*	114 (62.6)	120 (67.0)	124 (69.3)	122 (61.0)	0.663~
Grade 1	51 (28.0)	41 (22.9)	38 (21.2)	52 (26.0)	
Grade 2	16 (8.8)	14 (7.8)	14 (7.8)	21 (10.5)	
Grade 3	1 (0.5)	4 (2.2)	3 (1.7)	5 (2.5)	
Grade 4	0 (0.0)	0 (0.0)	0 (0.0)	0 (0.0)	

N: Number of subjects in the specified group with subject diaries recording general symptoms

%: Percentage based on total number of subjects in each group

*Grade 0 indicates that the subject did not experience the particular symptom.

~ Chi-square test (p-values from Chi-square test to detect differences in proportions between all treatment groups) Source data: Table 14.5.2.2

12.2.1.3.2 Solicited local symptoms

The subjects documented the occurrence of local symptoms at the injection site (erythema, swelling, induration and pain) for each day of the 7 days post-injection. Additionally, the intensity of erythema, swelling, and induration was documented and graded according to size of the lesion (diameter measurement).

520 subjects (70.3%) and 525 subjects (70.9%) of all subjects indicated on their diary cards to have experienced erythema respectively pain at the injection site after at least one injection. 411 subjects (55.5%) and 468 subjects (63.2%) of all subjects experienced swelling and induration respectively (see Section 14.5.3.3).

In 19.2% – 35.0% of all subjects local reactions were moderate and only in 0.3% – 1.5% of all subjects local reactions were severe with no Grade 4 reactions having been reported (see Section 14.5.3.4). There were 20 incidences of Grade 3 symptoms in Groups 1 and 4. The percentages of subjects who experienced any severe local symptom in any group were never higher than 3.3%.

In the frequency of the above mentioned symptoms a significant difference was observed between subjects of the placebo group (Group 3) and subjects who received at least one dose of MVA-BN®. 20.7% and 21.8% of the subjects who received placebo only (Group 3) experienced pain respectively erythema, compared to 83.5% – 91.2% and 81.6% – 91.2% of the subjects who received at least one dose of MVA-BN® (Groups 1, 2 and 4). 5.6% and 2.8% of the subjects in Group 3 experienced swelling respectively induration, compared to 81.9%, 57.5% and 74.5% for swelling and 89.0%, 81.6% and 77.5% for induration in Groups 1, 2 and 4, respectively (see Section 14.5.3.3).

Table 12-3 lists the individual numbers of subjects who experienced solicited local symptoms sorted by grade and group, whereas for each subject the maximum experienced intensity of the respective symptom is specified.

Table 12-3 Summary of Maximum Intensity of Solicited Local Symptoms (7-day Follow-up Period after Vaccination) as Recorded in the Subject Diary, Safety dataset

	Group 1 (N=182)	Group 2 (N=179)	Group 3 (N=179)	Group 4 (N=200)	p-value
	Number of subjects (%)				
**Injection site pain**					
Grade 0*	16 (8.8)	24 (13.4)	142 (79.3)	33 (16.5)	<0.001~
Grade 1	117 (64.3)	114 (63.7)	25 (14.0)	123 (61.5)	
Grade 2	48 (26.4)	41 (22.9)	12 (6.7)	41 (20.5)	
Grade 3	1 (0.5)	0 (0.0)	0 (0.0)	3 (1.5)	
Grade 4	0 (0.0)	0 (0.0)	0 (0.0)	0 (0.0)	
Grade 0*	16 (8.8)	24 (13.4)	142 (79.3)	33 (16.5)	<0.001~
**Injection site erythema**					
Grade 0*	16 (8.8)	33 (18.4)	140 (78.2)	31 (15.5)	<0.001~
Grade 1	55 (30.2)	87 (48.6)	37 (20.7)	71 (35.5)	

	Group 1 (N=182)	Group 2 (N=179)	Group 3 (N=179)	Group 4 (N=200)	p-value
	Number of subjects (%)				
Grade 2	105 (57.7)	59 (33.0)	2 (1.1)	93 (46.5)	
Grade 3	6 (3.3)	0 (0.0)	0 (0.0)	5 (2.5)	
Grade 4	0 (0.0)	0 (0.0)	0 (0.0)	0 (0.0)	
**Injection site swelling**					
Grade 0*	33 (18.1)	76 (42.5)	169 (94.4)	51 (25.5)	<0.001~
Grade 1	58 (31.9)	71 (39.7)	9 (5.0)	91 (45.5)	
Grade 2	89 (48.9)	32 (17.9)	1 (0.6)	57 (28.5)	
Grade 3	2 (1.1)	0 (0.0)	0 (0.0)	1 (0.5)	
Grade 4	0 (0.0)	0 (0.0)	0 (0.0)	0 (0.0)	
**Injection site induration**					
Grade 0*	20 (11.0)	33 (18.4)	174 (97.2)	45 (22.5)	<0.001~
Grade 1	86 (47.3)	111 (62.0)	4 (2.2)	97 (48.5)	
Grade 2	75 (41.2)	35 (19.6)	1 (0.6)	57 (28.5)	
Grade 3	1 (0.5)	0 (0.0)	0 (0.0)	1 (0.5)	
Grade 4	0 (0.0)	0 (0.0)	0 (0.0)	0 (0.0)	

N: Number of subjects in the specified group with subject diaries recording general symptoms
%: Percentage based on total number of subjects in each group
*Grade 0 indicates that the subject did not experience the particular symptom.
~ Chi-square test (p-values from Chi-square test to detect differences in proportions between all treatment groups)
Source data: Table 14.5.3.2

12.2.2 DISPLAY OF ADVERSE EVENTS

Adverse events were coded according to MedDRA using MedDRA Version 7.1. Where an adverse event could not be adequately represented by a single MedDRA preferred term (PT), several terms were chosen ("splitting" of events). 718 AEs reported verbatim by the investigator in this study were coded to 753 MedDRA preferred terms.

Table 12-4 gives an overview grouped by the MedDRA system organ classes (SOCs) of the coded 753 preferred terms and provides information about the frequencies in the different vaccination groups.

Table 12-4 Unsolicited Adverse Events: MedDRA coding by SOC and subject, Safety dataset

	N=Number of subjects in the specified group				
**SOC**	**Group 1 (MM) (N=183)**	**Group 2 (MP) (N=181)**	**Group 3 (PP) (N=181)**	**Group 4 (M-) (N=200)**	**Total (N=745)**
	**Number of subjects (%) - Number of events**				
Any AE	113 (61.7) 260	107 (59.1) 197	78 (43.1) 141	99 (49.5) 155	397 (53.3) 753
General disorders and administration site conditions	55 (30.1) 93	49 (27.1) 60	15 (8.3) 16	57 (28.5) 70	176 (23.6) 239
Infections and infestations	54 (29.5) 64	38 (21.0) 42	36 (19.9) 45	12 (6.0) 15	140 (18.8) 166
Nervous system disorders	16 (8.7) 18	25 (13.8) 27	14 (7.7) 15	11 (5.5) 12	66 (8.9) 72
Gastrointestinal disorders	12 (6.6) 13	12 (6.6) 16	12 (6.6) 15	13 (6.5) 14	49 (6.6) 58
Respiratory, thoracic and mediastinal disorders	16 (8.7) 18	12 (6.6) 12	10 (5.5) 12	6 (3.0) 6	44 (5.9) 48
Musculoskeletal and connective tissue disorders	13 (7.1) 13	4 (2.2) 4	12 (6.6) 12	10 (5.0) 12	39 (5.2) 41
Blood and lymphatic system disorders	5 (2.7) 7	7 (3.9) 7	2 (1.1) 2	4 (2.0) 4	18 (2.4) 20
Injury, poisoning and procedural complications	5 (2.7) 5	6 (3.3) 7	6 (3.3) 6	1 (0.5) 1	18 (2.4) 19
Skin and subcutaneous tissue disorders	3 (1.6) 7	7 (3.9) 7	4 (2.2) 4	4 (2.0) 6	18 (2.4) 24
Cardiac disorders	4 (2.2) 4	4 (2.2) 4	3 (1.7) 3	3 (1.5) 4	14 (1.9) 15
Reproductive system and breast disorders	4 (2.2) 4	1 (0.6) 1	2 (1.1) 2	2 (1.0) 2	9 (1.2) 9
Surgical and medical procedures	2 (1.1) 2	3 (1.7) 3	2 (1.1) 2	2 (1.0) 2	9 (1.2) 9
Vascular disorders	2 (1.1) 2	1 (0.6) 1	1 (0.6) 1	5 (2.5) 5	9 (1.2) 9
Psychiatric disorders	2 (1.1) 3	3 (1.7) 3	2 (1.1) 2	1 (0.5) 1	8 (1.1) 9
Eye disorders	1 (0.5) 1	1 (0.6) 1	2 (1.1) 2	1 (0.5) 1	5 (0.7) 5
Ear and labyrinth disorders	3 (1.6) 3	1 (0.6) 1	0 (0.0) 0	0 (0.0) 0	4 (0.5) 4
Investigations	1 (0.5) 1	0 (0.0) 0	1 (0.6) 1	0 (0.0) 0	2 (0.3) 2
Metabolism and nutrition disorders	1 (0.5) 1	0 (0.0) 0	1 (0.6) 1	0 (0.0) 0	2 (0.3) 2
Endocrine disorders	1 (0.5) 1	0 (0.0) 0	0 (0.0) 0	0 (0.0) 0	1 (0.1) 1
Immune system disorders	0 (0.0) 0	1 (0.6) 1	0 (0.0) 0	0 (0.0) 0	1 (0.1) 1

A subject with more than one finding in a specific category was only counted once.
Percentages are based on total no. of subjects in each group (Safety dataset).
Table is sorted by descending subject count for the total of all treatment groups; Source data: Table 14.5.1.5

All treatment-related solicited and unsolicited AEs are listed below according to preferred term (PT) that were observed in this study at a frequency of 2% or more of all subjects in any

treatment group. The percentages in parentheses following the terms represent the range of frequency of each event within all study groups. The majority of these AEs were in the SOC "General disorders and administration site conditions".

Local symptoms at the injection site:

- Injection site pain (20.4–90.7%)
- Injection site erythema (21.5–90.7%)
- Injection site induration (2.8–88.5%)
- Injection site swelling (5.5–81.4%)
- Injection site pruritus (2.2–23.5%)
- Injection site hematoma (2.0–5.0%)
- Injection site warmth (0–4.9%)
- Injection site discoloration (0–2.2%)

General symptoms:

- Fatigue (23.2–35.5%)
- Headache (17.1–32.0%)
- Myalgia (7.7–19.5%)
- Body temperature increased (5.0–10.5%)
- Nausea (4.4–9.4%)
- Dizziness (0.5–2.0%)
- Nasopharyngitis (0–2.7%)
- Diarrhoea (0–2.0%)
- Pharyngolaryngeal pain (0.6–2.0%)
- Lymphadenopathy (0–2.2%)
- Hot flush (0–2.0%)

All solicited and unsolicited AEs that were considered treatment related including the above mentioned are listed in Table 12-5 sorted by frequency. A p-value below 0.05 indicates a statistically significant difference in the frequencies of the respective AE between any of the four treatment groups.

Table 12-5 Solicited (General / Local) and Unsolicited Adverse Events Assessed as Being Possibly/Probably/Definitely Related to Study Vaccine: MedDRA Coding by SOC, PT, Safety dataset

		N = Number of subjects in the specified group				
**SOC**	**Preferred Term**	**Group 1 (N=183)**	**Group 2 (N=181)**	**Group 3 (N=181)**	**Group 4 (N=200)**	**p-value**
		**Number of subjects (%) - Number of events**				
General disorders and administration site conditions						
	Any PT	181 (98.9) 1392	177 (97.8) 824	103 (56.9) 252	194 (97.0) 894	<0.001
	Injection site pain	166 (90.7) 300	155 (85.6) 168	37 (20.4) 46	167 (83.5) 167	<0.001
	Injection site erythema	166 (90.7) 304	146 (80.7) 160	39 (21.5) 51	169 (84.5) 169	<0.001
	Injection site induration	162 (88.5) 273	146 (80.7) 155	5 (2.8) 6	155 (77.5) 155	<0.001
	Injection site swelling	149 (81.4) 247	103 (56.9) 112	10 (5.5) 12	149 (74.5) 149	<0.001
	Fatigue	57 (31.1) 75	42 (23.2) 52	45 (24.9) 53	71 (35.5) 71	0.030
	Headache	42 (23.0) 49	58 (32.0) 66	31 (17.1) 36	51 (25.5) 51	0.010
	Injection site pruritus	43 (23.5) 61	36 (19.9) 37	4 (2.2) 4	47 (23.5) 47	<0.001
	Myalgia	23 (12.6) 24	15 (8.3) 15	14 (7.7) 18	39 (19.5) 39	0.002
	Body temperature increased	16 (8.7) 18	19 (10.5) 19	10 (5.5) 10	10 (5.0) 10	0.132
	Nausea	12 (6.6) 14	17 (9.4) 20	8 (4.4) 8	16 (8.0) 16	0.280
	Injection site hematoma	6 (3.3) 6	9 (5.0) 9	4 (2.2) 4	4 (2.0) 4	0.373
	Injection site warmth	9 (4.9) 9	5 (2.8) 5	0 (0.0) 0	8 (4.0) 8	0.009
	Injection site discoloration	1 (0.5) 1	4 (2.2) 4	0 (0.0) 0	0 (0.0) 0	0.021
	Injection site irritation	1 (0.5) 2	1 (0.6) 1	0 (0.0) 0	3 (1.5) 3	0.413
	Asthenia	2 (1.1) 2	0 (0.0) 0	1 (0.6) 1	1 (0.5) 1	0.751
	Axillary pain	1 (0.5) 1	1 (0.6) 1	0 (0.0) 0	0 (0.0) 0	0.607
	Injection site hypersensitivity	1 (0.5) 1	0 (0.0) 0	1 (0.6) 1	0 (0.0) 0	0.607
	Injection site reaction	1 (0.5) 1	0 (0.0) 0	0 (0.0) 0	1 (0.5) 1	>0.999
	Chills	1 (0.5) 1	0 (0.0) 0	0 (0.0) 0	0 (0.0) 0	0.732
	Feeling hot	0 (0.0) 0	0 (0.0) 0	0 (0.0) 0	1 (0.5) 1	>0.999
	Implant site pain	1 (0.5) 1	0 (0.0) 0	0 (0.0) 0	0 (0.0) 0	0.732

		N = Number of subjects in the specified group				
SOC	Preferred Term	Group 1 (N=183)	Group 2 (N=181)	Group 3 (N=181)	Group 4 (N=200)	p-value
		Number of subjects (%) - Number of events				
	Injection site paraesthesia	1 (0.5) 1	0 (0.0) 0	0 (0.0) 0	0 (0.0) 0	0.732
	Injection site urticaria	0 (0.0) 0	0 (0.0) 0	1 (0.6) 1	0 (0.0) 0	0.486
	Malaise	0 (0.0) 0	0 (0.0) 0	0 (0.0) 0	1 (0.5) 1	>0.999
	Non-cardiac chest pain	1 (0.5) 1	0 (0.0) 0	0 (0.0) 0	0 (0.0) 0	0.732
	Soft tissue inflammation	0 (0.0) 0	0 (0.0) 0	1 (0.6) 1	0 (0.0) 0	0.486
	Thirst	0 (0.0) 0	0 (0.0) 0	0 (0.0) 0	1 (0.5) 1	>0.999
Nervous system disorders						
	Any PT	1 (0.5) 1	6 (3.3) 6	3 (1.7) 3	6 (3.0) 7	0.214
	Dizziness	1 (0.5) 1	2 (1.1) 2	2 (1.1) 2	4 (2.0) 4	0.617
	Headache	0 (0.0) 0	3 (1.7) 3	1 (0.6) 1	1 (0.5) 1	0.267
	Disturbance in attention	0 (0.0) 0	0 (0.0) 0	0 (0.0) 0	1 (0.5) 1	>0.999
	Paraesthesia	0 (0.0) 0	1 (0.6) 1	0 (0.0) 0	0 (0.0) 0	0.486
	Sedation	0 (0.0) 0	0 (0.0) 0	0 (0.0) 0	1 (0.5) 1	>0.999
Infections and infestations						
	Any PT	6 (3.3) 7	1 (0.6) 1	5 (2.8) 6	2 (1.0) 3	0.154
	Nasopharyngitis	5 (2.7) 5	0 (0.0) 0	4 (2.2) 4	1 (0.5) 1	0.044
	Rhinitis	0 (0.0) 0	1 (0.6) 1	2 (1.1) 2	0 (0.0) 0	0.148
	Sinusitis	1 (0.5) 1	0 (0.0) 0	0 (0.0) 0	1 (0.5) 1	>0.999
	Bronchitis	0 (0.0) 0	0 (0.0) 0	0 (0.0) 0	1 (0.5) 1	>0.999
	Tonsillitis	1 (0.5) 1	0 (0.0) 0	0 (0.0) 0	0 (0.0) 0	0.732
Skin and subcutaneous tissue disorders						
	Any PT	1 (0.5) 1	5 (2.8) 5	1 (0.6) 1	4 (2.0) 5	0.251
	Rash	0 (0.0) 0	2 (1.1) 2	0 (0.0) 0	1 (0.5) 1	0.418
	Hyperhidrosis	0 (0.0) 0	0 (0.0) 0	0 (0.0) 0	2 (1.0) 3	0.249
	Acne	0 (0.0) 0	0 (0.0) 0	0 (0.0) 0	1 (0.5) 1	>0.999
	Dermatitis	0 (0.0) 0	1 (0.6) 1	0 (0.0) 0	0 (0.0) 0	0.486
	Dermatitis contact	0 (0.0) 0	1 (0.6) 1	0 (0.0) 0	0 (0.0) 0	0.486
	Eczema	0 (0.0) 0	0 (0.0) 0	1 (0.6) 1	0 (0.0) 0	0.486
	Erythema	0 (0.0) 0	1 (0.6) 1	0 (0.0) 0	0 (0.0) 0	0.486
	Rash papular	1 (0.5) 1	0 (0.0) 0	0 (0.0) 0	0 (0.0) 0	0.732
Gastrointestinal disorders						
	Any PT	2 (1.1) 3	1 (0.6) 1	2 (1.1) 3	7 (3.5) 7	0.167

		N = Number of subjects in the specified group				
SOC	Preferred Term	Group 1 (N=183)	Group 2 (N=181)	Group 3 (N=181)	Group 4 (N=200)	p-value
		Number of subjects (%) - Number of events				
	Diarrhoea	0 (0.0) 0	1 (0.6) 1	1 (0.6) 1	4 (2.0) 4	0.160
	Dry mouth	2 (1.1) 3	0 (0.0) 0	0 (0.0) 0	1 (0.5) 1	0.466
	Abdominal pain upper	0 (0.0) 0	0 (0.0) 0	2 (1.1) 2	0 (0.0) 0	0.118
	Dysphagia	0 (0.0) 0	0 (0.0) 0	0 (0.0) 0	1 (0.5) 1	>0.999
	Toothache	0 (0.0) 0	0 (0.0) 0	0 (0.0) 0	1 (0.5) 1	>0.999
Respiratory, thoracic and mediastinal disorders						
	Any PT	2 (1.1) 2	2 (1.1) 2	2 (1.1) 2	5 (2.5) 5	0.686
	Pharyngo-laryngeal pain	2 (1.1) 2	1 (0.6) 1	1 (0.6) 1	4 (2.0) 4	0.564
	Cough	0 (0.0) 0	0 (0.0) 0	1 (0.6) 1	0 (0.0) 0	0.486
	Dry throat	0 (0.0) 0	0 (0.0) 0	0 (0.0) 0	1 (0.5) 1	>0.999
	Sneezing	0 (0.0) 0	1 (0.6) 1	0 (0.0) 0	0 (0.0) 0	0.486
Musculoskeletal and connective tissue disorders						
	Any PT	5 (2.7) 5	1 (0.6) 1	1 (0.6) 1	3 (1.5) 4	0.292
	Muscular weakness	2 (1.1) 2	0 (0.0) 0	1 (0.6) 1	1 (0.5) 1	0.751
	Arthralgia	0 (0.0) 0	0 (0.0) 0	0 (0.0) 0	1 (0.5) 1	>0.999
	Back pain	1 (0.5) 1	0 (0.0) 0	0 (0.0) 0	0 (0.0) 0	0.732
	Muscle spasms	0 (0.0) 0	0 (0.0) 0	0 (0.0) 0	1 (0.5) 2	>0.999
	Neck pain	1 (0.5) 1	0 (0.0) 0	0 (0.0) 0	0 (0.0) 0	0.732
	Pain in extremity	1 (0.5) 1	0 (0.0) 0	0 (0.0) 0	0 (0.0) 0	0.732
	Sensation of heaviness	0 (0.0) 0	1 (0.6) 1	0 (0.0) 0	0 (0.0) 0	0.486
Blood and lymphatic system disorders						
	Any PT	2 (1.1) 4	5 (2.8) 5	0 (0.0) 0	2 (1.0) 2	0.125
	Lymph-adenopathy	2 (1.1) 3	4 (2.2) 4	0 (0.0) 0	2 (1.0) 2	0.250
	Lymph node pain	1 (0.5) 1	0 (0.0) 0	0 (0.0) 0	0 (0.0) 0	0.732
	Lymphadenitis	0 (0.0) 0	1 (0.6) 1	0 (0.0) 0	0 (0.0) 0	0.486
Cardiac disorders						
	Any PT	0 (0.0) 0	2 (1.1) 2	1 (0.6) 1	2 (1.0) 3	0.638
	Palpitations	0 (0.0) 0	1 (0.6) 1*	0 (0.0) 0	1 (0.5) 2	0.868
	Tachycardia	0 (0.0) 0	1 (0.6) 1	0 (0.0) 0	1 (0.5) 1	0.868
	Pericardial effusion	0 (0.0) 0	0 (0.0) 0	1 (0.6) 1	0 (0.0) 0	0.486
Vascular disorders						

		N = Number of subjects in the specified group				
**SOC**	**Preferred Term**	**Group 1 (N=183)**	**Group 2 (N=181)**	**Group 3 (N=181)**	**Group 4 (N=200)**	**p-value**
		**Number of subjects (%) - Number of events**				
	Any PT	0 (0.0) 0	0 (0.0) 0	0 (0.0) 0	4 (2.0) 4	0.015
	Hot flush	0 (0.0) 0	0 (0.0) 0	0 (0.0) 0	4 (2.0) 4	0.015
Psychiatric disorders						
	Any PT	1 (0.5) 1	1 (0.6) 1	0 (0.0) 0	1 (0.5) 1	>0.999
	Insomnia	0 (0.0) 0	1 (0.6) 1	0 (0.0) 0	0 (0.0) 0	0.486
	Restlessness	0 (0.0) 0	0 (0.0) 0	0 (0.0) 0	1 (0.5) 1	>0.999
	Sleep disorder	1 (0.5) 1	0 (0.0) 0	0 (0.0) 0	0 (0.0) 0	0.732
Eye disorders						
	Any PT	0 (0.0) 0	1 (0.6) 1	0 (0.0) 0	0 (0.0) 0	0.486
	Visual disturbance	0 (0.0) 0	1 (0.6) 1	0 (0.0) 0	0 (0.0) 0	0.486
Investigations						
	Any PT	1 (0.5) 1	0 (0.0) 0	0 (0.0) 0	0 (0.0) 0	0.732
	Lymph node palpable	1 (0.5) 1	0 (0.0) 0	0 (0.0) 0	0 (0.0) 0	0.732
Metabolism and nutrition disorders						
	Any PT	0 (0.0) 0	0 (0.0) 0	1 (0.6) 1	0 (0.0) 0	0.486
	Polydipsia	0 (0.0) 0	0 (0.0) 0	1 (0.6) 1	0 (0.0) 0	0.486
Reproductive system and breast disorders						
	Any PT	0 (0.0) 0	0 (0.0) 0	0 (0.0) 0	1 (0.5) 1	>0.999
	Menstruation irregular	0 (0.0) 0	0 (0.0) 0	0 (0.0) 0	1 (0.5) 1	>0.999

Relationship to study medication: possible/probable/definite for unsolicited AEs
Solicited Local Adverse Events are included if maximum intensity during first 7 days was > 0.
For Solicited Local Adverse Events 'other' category was excluded.
For Solicited General AEs, AEs recorded under the 'other' category have been transcribed to unsolicited AEs during data collection.
For Solicited General AEs, all AEs with missing or unknown relationship and possible/probable/definite relationship and maximum intensity during first 7 days > 0 (Grade 1-4) are included.
P-value from Fisher's Exact test to detect any global difference between all treatment groups
*The report of “palpitations” in the Group 2 subject occurred after the second vaccination with placebo.
Source data: Table 14.5.4.1

12.2.3 ANALYSIS OF ADVERSE EVENTS

12.2.3.1 Analysis of differences in the frequency of total unsolicited AEs between vaccination groups

The percentage of subjects who experienced at least one unsolicited AE (see Table 12-1 and Table 12-4) is lowest in the placebo group, Group 3 (43.1%), followed by Group 4 with 49.5%, and 59.1% and 61.7% in Groups 2 and 1 respectively.
These ratios change slightly when only treatment-related unsolicited AEs are taken into account. In Groups 1, 2 and 4 the percentages of subjects who experienced at least one treatment-related AE are 33.3%, 33.7% and 37.0%, respectively, while the low percentage in the placebo group is confirmed and becomes more pronounced (12.7%), indicative of an effect of MVA-BN® (see Table 12-1).

12.2.3.2 Analysis of differences in the frequency of individual treatment-related solicited and unsolicited AEs between vaccination groups

A statistically significant difference between any of the four treatment groups, as determined by a p-value < 0.05, can be observed in the frequencies of the following individual solicited and unsolicited AEs (p-values are indicated in parentheses after the term, see also Table 12-5). This can be a difference between placebo and other groups, as shown below for most of the local symptoms, or any other difference between any of the groups. All AEs listed were considered related to treatment by the investigator while still blinded.

<u>Local symptoms at the injection site:</u>
- Injection site pain (< 0.001)
- Injection site erythema (< 0.001)
- Injection site induration (< 0.001)
- Injection site swelling (< 0.001)
- Injection site pruritus (< 0.001)
- Injection site warmth (0.009)
- Injection site discoloration (0.021)

<u>General symptoms:</u>
- Fatigue (0.030)
- Headache (0.010)
- Myalgia (0.002)
- Nasopharyngitis (0.044)
- Hot flush (0.015)

Apart from nasopharyngitis and hot flush, these AEs belong to the SOC “General disorders and administration site conditions” (see Table 12-5). A clear difference between placebo and the other groups in the numbers of subjects who experienced at least one treatment-related solicited or unsolicited AE from this SOC can be seen: 98.9%, 97.8% and 97.0% for Groups 1, 2 and 4, respectively and 56.9% in Group 3. The number of subjects who experienced at least one unsolicited AE from this organ class regardless of relationship to treatment, reflects the same trend as above: 30.1%, 27.1% and 28.5% for Groups 1, 2 and 4, respectively and 8.3% in Group 3 (see Table 12-4).

Individual AEs that indicate a direct relationship to vaccination with MVA-BN® compared to placebo, i.e. that clearly show a significantly lower frequency in the placebo group, are the following local injection site reactions: pain, erythema, induration, swelling, pruritus and warmth (see Figure 12-1). Despite the lower frequency, pain and erythema did also occur in 20.4% respectively 21.5% of subjects in the placebo group and therefore could have been caused by the excipients of the placebo solution.
Injection site discoloration only occurred in 0.5% and 2.2% of subjects in Group 1 and 2.

When comparing Group 1 (two vaccinations with MVA-BN®) with Group 3 (two injections of placebo), fatigue, headache, myalgia and nasopharyngitis occurred marginally less frequently in the placebo group. The exact differences in frequency were between 6.2% (fatigue) and 0.5% (nasopharyngitis) (see Table 12-5).

Comparing Group 2 (one vaccination with MVA-BN® and one with placebo) with Group 3 (placebo group), fatigue and myalgia were similarly frequent (23.2% - 24.9% respectively 7.7% - 8.3%). The highest incidence of fatigue and myalgia was observed in subjects in Group 4 (35.5% and 19.5%, respectively). Nasopharyngitis did not occur in Group 2 and occurred at a frequency of 0.5% in Group 4. Hot flush was only reported in four subjects (2%) in Group 4 (see Table 12-5).

The moderately high, respectively minimally significantly different frequencies of naso-pharyngitis between the study groups can possibly be explained by the seasonal periods in which the subjects participated. Nearly all subjects in Group 4 were recruited early in the study and consequently participated during the summer months (April to October), while many of the subjects in Groups 1-3 also participated in the study over the winter as they were enrolled from May to February.

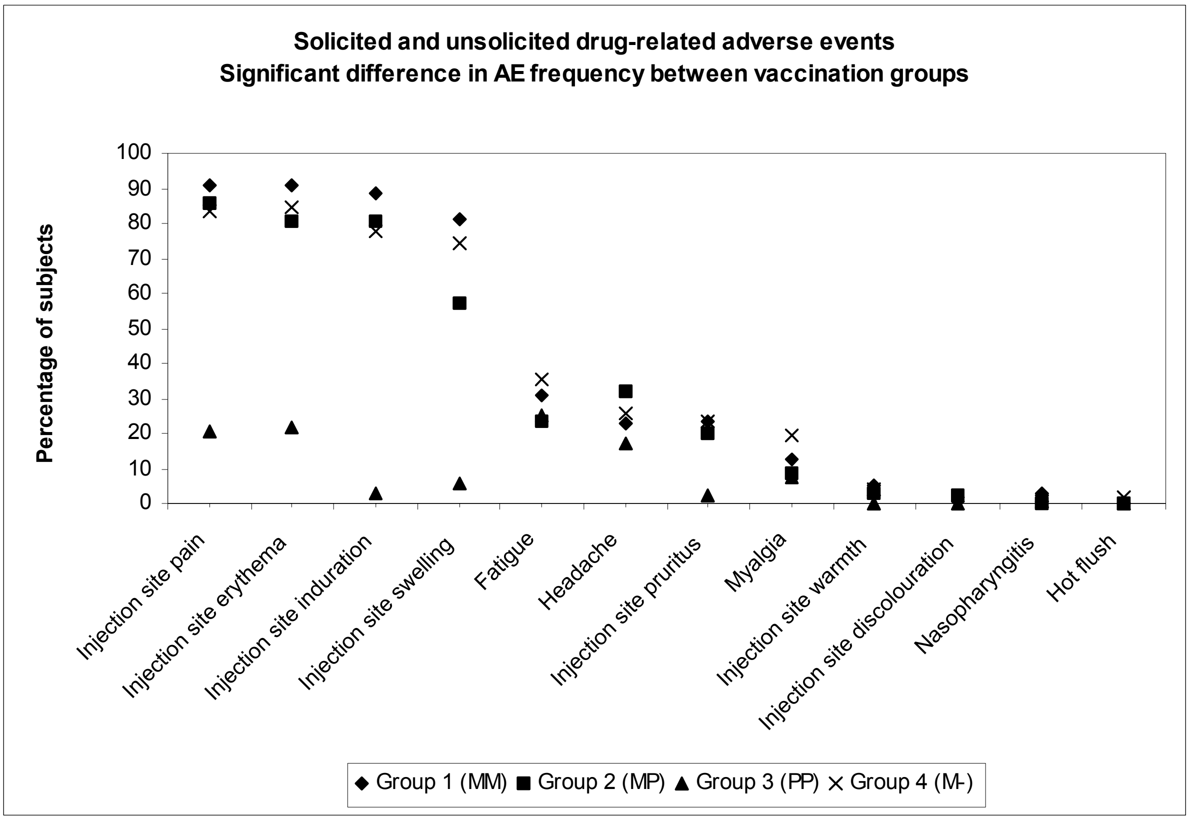

Figure 12-1 Frequencies of solicited and unsolicited treatment-related adverse events in the safety dataset with a statistically significant difference between any of the four groups as determined by a p-value < 0.05.
Source: Table 12-5

In Group 1 local injection site symptoms such as erythema, swelling and induration (except pain) tended to be slightly more severe after the second vaccination with MVA-BN® (increase of Grade 2 and 3 symptoms) than after the first vaccination (see Section 14.5.3.1).

Comparing vaccinia-naïve subjects (Groups 1 and 2) with vaccinia-experienced subjects (Group 4) after the first vaccination, overall frequencies of local solicited symptoms were similar, however, Grade 2 and 3 erythema and induration tended to be more frequent in Group 4. Swelling was overall more frequent in Group 4 after the first vaccination (see Section 14.5.3.1). Myalgia was especially common in Group 4, as compared to Groups 1 and 2 (see Section 14.5.2.1).

After the second injection (MVA-BN® in Group 1 and placebo in Group 2) there were no major differences (differences $\leq$ 4.3%) in solicited general AEs between Groups 1 and 2 (see Section 14.5.2.1).

12.2.4 LISTING OF ADVERSE EVENTS BY SUBJECTS

Individual AE data are listed in Section 16.2.7. Information about the follow-up of unresolved AEs is provided in Section 14.5.5.

12.3 DEATHS, OTHER SERIOUS ADVERSE EVENTS, AND ADVERSE EVENTS OF SPECIAL INTEREST

A summary of other serious adverse events and adverse events of special interest is given in Section 14.5.1.1, individual data are listed in Section 16.2.7.2.3. Individual data about SAEs and SIAEs reported during follow-up are provided in Section 14.5.5.

12.3.1 LISTING OF DEATHS, OTHER SERIOUS ADVERSE EVENTS AND ADVERSE EVENTS OF SPECIAL INTEREST

All SAEs and SIAEs that occurred during this study are listed in Table 12-6. SAEs and SIAEs that were reported during the follow-up are marked with FU; all SAEs and SIAEs considered related to the study vaccine are bolded.

12.3.1.1 Deaths

No deaths occurred in this study.

12.3.1.2 Other Serious Adverse Events

A total of 3 serious adverse events were reported during the active phase of this study, in two subjects from Group 2 (Subjects 536 and 706) and in one subject from Group 1 (Subject 148). One SAE, a ruptured tendon of the finger, was considered not related to the study vaccine and is still ongoing. The other two SAEs were nervous breakdown and transient motoric hemiparesis, from which the two subjects completely recovered. Both events were considered unlikely to be related to the study vaccine.

During follow-up (6 months-period after the last vaccination) nine SAEs were reported in 9 subjects, of which only one SAE was considered to be possibly related to the study vaccine: ongoing sarcoidosis in subject 521 in Group 1. Two SAEs were judged to be unlikely related to the study treatment and consisted of colon carcinoma (Subject 685, Group 3) and depression (Subject 751, Group 3). Six SAEs were assessed to be unrelated: tonsillectomy, pregnancy, salmonella enteritis, thyroidectomy, lower limb fracture, and tendon rupture.

12.3.1.3 Other Significant Adverse Events

“Adverse events of special interest” (SIAEs) were defined as any cardiac symptom, clinically significant ECG changes or cardiac enzymes elevated above ULN.
A total of 11 SIAEs were reported for 10 subjects during the active phase of the study (palpitations, tachycardia and sinus tachycardia). 5 events were considered as possibly related to the study vaccine. Two of these possibly related SIAEs occurred in two female subjects in Group 2 (Subjects 220 and 563) and three SIAEs occurred in Group 4 in one male (Subject 028, 2 reports of palpitations) and one female subject (Subject 397, tachycardia).

In subject 580 (Group 3, placebo) a mild pericardial effusion was incidentally found in the echocardiogram on 31 October 2006. The event was not classified as an SIAE, because

following extensive work-up the cardiologist concluded that the event was not clinically significant. A control echocardiogram on 23 November 2006 showed no pathological findings.

A further five SIAEs were reported during follow-up in five subjects consisting of palpitations (Subjects 710, 024 and 028), tachycardia (Subject 246) and mild pericardial effusion (Subject 411). All SIAEs were considered to be unlikely related to the study vaccine.
Of these subjects two subjects had already experienced at least one SIAE during the active study phase. Subject 028 in Group 4 experienced two events of palpitations during the active study phase and then again palpitations during the follow-up period, which however were considered to be unlikely related to the study vaccine. Subject 411, also in Group 4, experienced palpitations during the active study phase and had a mild pericardial effusion during follow-up. Both SIAEs in this subject were considered unlikely related to the study vaccine.

12.3.1.4 Pregnancies

A total of three pregnancies were reported in this study (Subject nos. 766, 695 and 486); see Section 16.3.3 for the pregnancy report forms. The pregnancy in subject 486 was classified as an SAE because the subject was hospitalized when she gave birth to a healthy child on 8th October, 2007. The other two women have not delivered yet as of the date of this report.

Table 12-6 Deaths, other serious adverse events and adverse events of special interest:

Subject	Adverse Event (Diagnosis)	SAE/SIAE	Relationship to study vaccine
**Group 1**			
148	Nervous breakdown	SAE	Unlikely related
287	Sinus tachycardia (102 bpm)	SIAE	Unlikely related
317	Tachycardia	SIAE	Unlikely related
495	Tonsillectomy	SAE (FU)[1]	Unrelated
**521**	**Sarcoidosis**	**SAE (FU)**	**Possibly related**
557	Palpitations (for about 5 seconds). subject known palpitations from years ago during sports, now without sports for the first time.	SIAE	Unlikely related
**Group 2**			
220	**Palpitations**	**SIAE**	**Possibly related**
228	Sinus tachycardia	SIAE	Unlikely related
486	Pregnancy	SAE (FU)	Unrelated
536	Transient motoric hemiparesis (right side)	SAE	Unlikely related
563	**Tachycardia (105 bpm)**	**SIAE**	**Possibly related**
706	Rupture of extension tendon 4th finger (ring finger) left hand 29.12.2006, op 30.12.2006, subject was in hospital 30.-31.12.2006. cut with knife while cooking	SAE	Unrelated
710	Palpitations	SIAE (FU)	Unlikely related
**Group 3**			
067	Samonella enteritis	SAE (FU)	Unrelated
444	Thyroidectomy (because of benign lump and cyst)	SAE (FU)	Unrelated
594	Fracture of right tibia and fibula	SAE (FU)	Unrelated
685	Colon-carcinoma	SAE (FU)	Unlikely related
689	Palpitations	SIAE	Unlikely related
751	Depression	SAE (FU)	Unlikely related
**Group 4**			
024	Palpitations	SIAE (FU)	Unlikely related
028	**Palpitations**	**SIAE**	**Possibly related**
	**Palpitations**	**SIAE**	**Possibly related**
	Palpitations	SIAE (FU)	Unlikely related
119	Rupture of the left tendon of the peroneus 5.11.2006; OP 8.12.2006	SAE (FU)	Unrelated
246	Tachycardia	SIAE (FU)	Unlikely related
397	**Tachycardia reported by subject**	**SIAE**	**Possibly related**
411	Palpitations	SIAE	Unlikely related
	Mild pericardial effusion	SIAE (FU)	Unlikely related

[1] SAEs or SIAEs that were reported at follow-up are marked with "FU"
Drug related (definite, probable, possible or unkown) SAEs or SIAEs are bolded.
Source data: Sections 14.5.1.1, 14.5.5.1 and 16.2.7..3

12.3.2 NARRATIVES OF DEATHS, OTHER SERIOUS ADVERSE EVENTS AND ADVERSE EVENTS OF SPECIAL INTEREST

Case report forms of SAEs and SIAEs are provided in Section 16.3.1.

12.3.2.1 Serious adverse events

A serious adverse event was reported in the following twelve subjects. All SAEs except one case of sarcoidosis were considered unlikely or not related to the treatment by the investigator. Sarcoidosis in Subject 521 (Group 1) was judged to be possibly related to the study vaccine.

Group 1 /Subject 148:
A 27 year old woman reported a nervous breakdown on August 6th, 2006, 5 days after the second vaccination with MVA-BN®. The subject was hospitalized for 5 days and recovered without sequelae. The subject stated that she had broken up the relationship with her partner. The investigator considered the event probably related to personal social problems and unlikely related to the study vaccine. The subject was treated with Cipramil due to suspicion of depression from August 6th until 8th, 2006.

Group 1 /Subject 495:
During the follow-up period (approx. 5 months, 158 days, after the second vaccination) a 22 year old woman underwent tonsillectomy from March 19th-25th, 2007. The subject recovered without sequelae. According to the investigator the tonsillectomy had been performed because of recurrent tonsillitis (definite relationship to this condition).

Group 1 /Subject 521:
A 30 year old man suffered from arthralgia since December 26th, 2006, 10 weeks after the second vaccination and reported fever up to 38°C and night sweat. Based on bronchoscopy and biopsy on February 6th, 2007 the subject was diagnosed with sarcoidosis. As the cause of sarcoidosis is unknown the investigator classified the event as an important medical event and as possibly related to the study vaccine or other condition or treatment, The subject was treated with 600 mg ibuprofen five times daily from January 15th to February 15th, 2007. The event is ongoing.

Group 2 /Subject 486:
Approx. four months (118 days) after the second vaccination a 20 year old woman was confirmed pregnant on February 8th, 2007. On October 8th, 2007 she gave birth to a healthy child.

Group 2 /Subject 536:
On October 3rd, 2006 at about 10 a.m., 6 days after the first vaccination, a 38 year old woman suddenly had problems moving her right arm and later also right leg while having a bath. The symptoms lasted for about 3 hours and were diagnosed in the clinic, where the subject was hospitalized, to be a transient motoric hemiparesis on the right side due to two small embolic ischemic events. The subject suffers from migraine accompagnée 1-2 times a year and hay fever (May to October) since 1992 and from tinnitus in both ears since childhood. The subject was hospitalized from October 3rd until 5th, 2006 and treated with ASS 100 and 40 mg Simvastatin. The subject recovered without sequelae. The cause for the transient ischemic events was not found. Duplex /doppler sonography, TTE (transthoracic echocardiogram), TEE (transesophageal echocardiogram), EEG (electroencephalogram) and clinical laboratory examinations performed in the clinic were all without pathological findings. The investigator

judged the event to be unlikely related to the study vaccine and possibly related to another condition/treatment (history of migraine accompagnée).

Group 2 /Subject 706:
An 18 year old man cut his finger while cooking and ruptured the extension tendon of the left 4th finger on December 29th, 2006, 25 days after the second vaccination. The subject was hospitalized from December 30th–31st, 2006 and required surgery. The event was assessed as not related to study vaccine and at the time of last visit, the event was still ongoing.

Group 3 /Subject 067:
A 50 year old woman was diagnosed with Salmonella enteritis and required hospitalization from February 1st-6th, 2007. The event started on 22 January 22nd, 2007, approx. six months (199 days) after the last injection, and had completely resolved on February 6th, 2007. Two days prior to onset she had been eaten dough containing raw eggs. The event was assessed by the investigator to be definitely related to a bacterial infection due to the consumption of raw eggs.

Group 3 /Subject 444:
A 40 year old man was hospitalized from January 2nd-4th, 2007 (approx. three months, 85 days, after the second injection) for a thyroidectomy. According to the investigator the thyroidectomy had been performed because of a benign lump and cyst (definite relationship). The subject recovered with sequelae.

Group 3 /Subject 594:
A 27 year old woman was hospitalized from February 17th, 2007, approx. 3 months (94 days) after the second injection, until March 4th, 2007 because of surgery of a fracture of the right tibia and fibula. She reported intermittent pain under pressure. The investigator confirmed that the event was definitely related to an accident. The subject recovered with sequelae.

Group 3 /Subject 685:
An examination to follow-up abdominal pain revealed a colon-carcinoma (sigma-tumor) in a 27 year old woman, which was subsequently removed during hospitalization from February 12th (more than 10 weeks after the last injection) to February 19th, 2007. The investigator considered the event to be unlikely related to the vaccine and probably related to other condition or treatment. The subject recovered with sequelae.

Group 3 /Subject 751:
A 32 year old man had recurrent depression since 2000. The subject had a new episode of depression from February 15th, 2007 (more than nine weeks, 66 days, after the second injection) to June 15th, 2007 and required hospitalization from March 22nd to April 26th, 2007. The subject recovered completely without sequelae. The event was judged by the investigator as unlikely related to the study vaccine and probably related to mobbing at work, as reported by the subject. Since 2005 the subject suffers from attention deficit disorder and is treated with 10 mg oral methylphenidate daily.

Group 4 /Subject 119:
A 35 year old man ruptured the tendon of the peroneus at the left ankle on November 5th, 2006, approx. 4 months (128 days) after the vaccination. He was hospitalized from December 7th-11th, 2006 for surgery of the tendon. The investigator reported that the event was definitely related to an accident. On January 18th, 2007 the subject had completely recovered.

12.3.2.2 Adverse events of special interest

The following describes all subjects who experienced SIAEs considered possibly related to treatment by the investigator. The event in Subject 563 was considered probably related to another condition and three of the other four events were assessed as being also possibly related to other conditions or circumstances.

Group 2 /Subject 220:
A 26 year old woman experienced palpitations on July 13th, 2006 lasting 2.5 hours, 14.8 hours after the first vaccination with MVA-BN®. The subject suffered from allergic rhinitis (hay fever) and had been taking Citericin since May 1st, 2006. The event was judged to be not related to Citericin. The subject recovered without sequelae.

Group 2 /Subject 563:
At Visit 5 on November 21st, 2006 a 25 year old woman was diagnosed with tachycardia (heart rate of 105 bpm). The event occurred 28 days after the second vaccination (October 24th, 2006) and lasted 2 days, until November 23rd, 2006. The subject was not treated for the event and recovered without sequelae. As the subject was nervous and under time pressure, the investigator also rated the event as probably related to these circumstances. The measured heart rates at all study visits were 81 bpm (screening), 79 bpm (Visit 1), 72 bpm (Visit 2), 99 bpm (Visit 3), 92 bpm (Visit 4) and 105 bpm (Visit 5). The subject reported that she was under time pressure also at Visits 3, 4 and 5.

Group 4 /Subject 028 (3 SIAE events):
On May 23rd, 2006, about 3 hours after the vaccination with MVA-BN®, a 46 year old man experienced palpitations (combined with sweating) from 17.30 until 18.30. The investigator considered the event to be possibly related to the study procedure (vaccination) or to another condition or treatment. Three days later, on May 26th, 2006, the subject experienced again palpitations (combined with sweating) from 14.00 hours until evening. For the second SIAE the subject was treated with Aspirin plus C once daily from May 26th–27th, 2006. The investigator judged this second SIAE as also possibly related to another condition or treatment. On May 30th, 2006 an additional ECG was recorded and an additional safety laboratory test performed, the results of which were all normal. The subject completely recovered.
On August 23rd, 2006, three months (92.3 days) after the vaccination, the subject had a common cold and experienced again sweating and palpitations from 22:00 until 23:30. He recovered without sequelae. The subject was sent to a cardiologist for an ECG and echocardiogram. No evidence of cardiac disease was found. As the subject had a common cold with the typical symptoms (conjunctivitis, rhinitis) the investigator considered the event to be unlikely related to the study vaccine and probably related to the common cold.

Group 4 /Subject 397:
In the evening of August 28th, 2006 after vaccination with MVA-BN® (administered at 15:38) a 37 year old woman reported tachycardia (heart rate up to 120 bpm). The subject measured her pulse herself and described constant tachycardia from the evening of August 28th, until 31st, 2006. The investigator considered the event to be possibly related to the study vaccine or study procedure, however, he also noted that the subject might have been nervous because of the injection, which may also have been a possible cause for the event. TSH, T3 and T4 were within the reference ranges. The subject recovered without sequelae.

The investigator classified all of the following SIAEs as unlikely related the study vaccine.

<u>Group 1 /Subject 287:</u>
A 28 year old woman had a short period of sinus tachycardia (102 bpm) on September 15th, 2006 during ECG measurement, 14 days after the second vaccination with MVA-BN®. The subject had had a high pulse at Visit 2 (93 bpm) and felt well and was healthy. The investigator considered the event probably related to hypothyroidism. The subject suffered from hypothyroidism since 2004 and is on medication since August 2004, taking 1 tablet of L-Thyrox-Jod Hexal 50/100 daily (orally). The subject also reported intermittent dizziness about every 4 months since 2005. The investigator also noted that the subject felt normal and that she was probably excited during the examination. The subject was not treated and recovered without sequelae. TSH, T3 and T4 values were within normal reference ranges.

<u>Group 1 /Subject 317:</u>
A 45 year old man experienced tachycardia (116 bpm) on October 9th, 2006, 31 days after the second vaccination, lasting approx. 4 months until February 13th, 2007 (the date of the foreseen Follow-up Visit). The investigator assessed the event as probably related to psychological stress due to personal reasons. The subject was not treated and recovered without sequelae. TSH, T3 and T4 and safety laboratory values were within the normal reference ranges. On February 13th, 2007 the heart rate was 96 bpm.

<u>Group 1 /Subject 557:</u>
2 days after the first vaccination on October 4th, 2006 a 35 year old man experienced palpitations that lasted about 5 seconds. The subject had experienced this symptom already years ago during athletic activities and this was the first time the symptoms occurred without doing sports. The investigator judged the event as possibly related to this medical history. The subject was not treated and recovered. The subject suffers from hypothyreosis since 1995 and takes orally 100 µg Jodid 100 per day since January 1995. He also reported intermittent headache since 2002.

<u>Group 2 /Subject 228:</u>
A 32 year old man was diagnosed with sinus tachycardia (111 bpm) on August 24th, 2006 at Visit 4, 13 days after the second vaccination, lasting 1 day or less. The subject had no complaints and a pre-existing fast pulse at screening visit (98 bpm). The investigator rated the event to be probably related to another condition/treatment. The cardiologist performed a control ECG and echocardiogram. All values were in the normal reference ranges. TSH, T3 and T4 values were within the normal reference ranges. The subject was not treated and recovered without sequelae.

<u>Group 2 /Subject 710:</u>
Since June 23rd, 2007, more than six months (193 days) after the second vaccination, a 26 year old woman experienced intermittent palpitations, especially in the evenings. The investigator judged the event to be unlikely related to the study vaccine and possibly related to personal distress. The event is still ongoing.

<u>Group 3 /Subject 689:</u>
A 30 year old man experienced palpitations several times per day for about 10 seconds each since November 15th, 2006, 23 days after the first vaccination with placebo on October 23rd, 2006. According to the investigator, the event was possibly related to another unknown condition or treatment. Since August 2006 the subject experienced recurrent pain in the right shoulder. The subject was not treated and the event is ongoing.

Group 4 /Subject 024:
A 47 year old woman experienced palpitations in August and October 2006. The vaccination had been on 23 May 2006. The subject reported that the symptoms always appeared after states of anxiety within periods of depressed mood. The investigator rated the event to be unlikely related to the study vaccine and probably related to anxiety. The subject recovered without sequelae.

Group 4 /Subject 246:
A 54 year old man experienced tachycardia (105 bpm) on January 18th, 2007, approx. six months, 182 days, after the vaccination. The event lasted until February 1st, 2007. The subject recovered without sequelae. The investigator considered the event to be unlikely related to the study vaccine and possibly related to the study procedure or to another condition or treatment. The heart rate at screening had been 89 bpm. The subject was nervous at visits. A follow-up revealed that TSH, T3 and T4 were within normal range. The echocardiogram and ergometry were within normal variations.

Group 4 /Subject 411 (2 SIAE events):
A 47 year old woman experienced nocturnal palpitations, lasting 10 minutes, on August 25th, 2006 (spontaneous onset and end), 33.6 hours after the first vaccination with MVA-BN®. The ECG performed on September 6th, 2006 was normal. The subject sometimes takes ayurvedic herbs and homeopathic medications. The subject did not feel discomfort and felt well all the time. The subject was not treated and recovered without sequelae. The investigator considered the event probably related to other conditions/treatments. The subject is known to have hypotension and an allergy to ampicillin, penicillin and Gelonida tablets. After a bicycle accident on May 15th, 2006 the subject required surgery of the shoulder and had ongoing pain in the right shoulder since then.
At the follow-up visit on March 6th, 2007 the subject described pain in the heart region in the evening prior to the visit. The subject was followed-up by a cardiologist who diagnosed a mild pericardial effusion by ultrasound on March 7th, 2007, 196 days, after the vaccination. The effusion had not changed at a control investigation of the cardiologist two weeks later. The cardiologist reported that the effusion was not clinically significant and that no treatment was warranted. Because of the long time gap (approx. 6 months) between the event and the vaccination, the investigator judged the event to be unlikely related to the vaccine and possibly related to another condition or treatment.

12.3.3 ANALYSIS AND DISCUSSION OF DEATHS, OTHER SERIOUS ADVERSE EVENTS AND CERTAIN OTHER SIGNIFICANT ADVERSE EVENTS

Five of the 16 SIAEs that were reported in this study were considered possibly related to the study vaccine. A 46 year old male subject in Group 4 experienced two possibly treatment-related SIAEs 3 days apart in the form of palpitations starting on the day of the vaccination. The other event of palpitations that was considered as a possibly treatment-related SIAE occurred in a female subject in Group 2, 14.8 hours after vaccination. Both subjects recovered without sequelae.
The other two possibly treatment-related SIAEs were tachycardia which occurred in one female subject in Group 2 (subject 563) and a female subject in Group 4 (subject 397). One event occurred on the day of the injection (subject 397), the other event occurred at Visit 5 (subject 563) 28 days after the second vaccination. The investigator assessed both events to be also possibly related to another condition / treatment and for subject 397 as possibly related to the study procedure. The investigator considered both events to be possibly respectively probably

related to nervousness about the injection in subject 397 and nervousness and time pressure in subject 563. For subject 563 an increased heart rate (99 and 92 bpm) was also measured at Visits 3 and 4 and the subject also reported being under time pressure at these two visits.

Only one (sarcoidosis in Subject 521) of the twelve reported SAEs was considered to be possibly related to the study vaccine. This SAE occurred more than two months after the second vaccination and is still ongoing. The investigator judged the event to be possibly also related to another condition or treatment.

12.4 CLINICAL LABORATORY EVALUATION

12.4.1 LISTING OF INDIVIDUAL LABORATORY MEASUREMENTS BY SUBJECT (16.2.8) AND EACH ABNORMAL LABORATORY VALUE (14.3.4)

Individual data listings of the results of the clinical laboratory tests are provided in Section 16.2.8.6; individual results of clinical laboratory tests performed at follow-up (if clinically indicated) are listed in Section 16.2.10.2.3. In these listings, laboratory values above or below the normal range are marked with '+' or '–', respectively, and classified as clinically significant (2) or not clinically significant (1). Corresponding summary tables are provided in Section 14.3. Reference ranges are provided in Section 16.2.8.1 and 16.2.10.2.1.

12.4.2 EVALUATION OF EACH LABORATORY PARAMETER

12.4.2.1 Laboratory Values Over Time

No obvious pattern or trend over time was observed.

12.4.2.2 Individual Subject Changes

At all examinations including screening there were deviations from the normal range, but the investigator considered none of these individual deviations to be clinically significant.

12.4.2.3 Individual Clinically Significant Abnormalities

There were no individual clinically significant abnormalities reported in this study.

12.5 VITAL SIGNS, PHYSICAL FINDINGS AND OTHER OBSERVATIONS RELATED TO SAFETY

Systolic and diastolic blood pressure, pulse rate and oral body temperature were measured at each visit and at follow-up. Summary tables are provided in Section 14.6.2 and individual data are listed in Sections 16.2.9.3 and 16.2.10.3.2 (follow-up data). No clinically significant abnormalities were observed for the above examinations.
Information about body temperature measured and recorded on the diary card by the subjects is provided in Section 12.2.1.3.1.

A physical examination was performed at screening for baseline assessments; any changes were recorded at each visit and at follow-up (see Sections 16.2.9.2 and 16.2.10.3.1 for follow-up data).

12.6 ECG ANALYSIS

ECGs were recorded at screening, Visit 2 and Visit 4 (Groups 1-3 only). If clinically indicated, additional ECGs were recorded at Visit 3, Visit 5 or follow-up (or whenever deemed necessary by the investigator). Individual data are listed in Section 16.2.9.4 and 16.2.10.3.3 (follow-up); summary tables are provided in Section 14.4.
All ECG recordings were considered normal or represented normal variations as assessed by the investigator and/or the local cardiologist.

12.6.1 PR, QRS, QT AND RR INTERVALS

No obvious difference between Groups 1-4 or a trend over time was observed for mean PR, QRS and RR intervals; see summary tables in Section 14.4.5.

There was no increase of the mean QT interval duration in Groups 1-4.

12.6.2 QTc INTERVAL ANALYSIS

The measured QT intervals were corrected for heart rate (QTc intervals) using Bazett's correction (QTc_B) and Fridericia's correction (QTc_F).

Absolute QTc_F values > 450 ms and ≤ 480 ms were observed at screening in a total of 14 subjects (1.9% of all subjects), 5 subjects each in Groups 1 and 3 and 2 subjects each in Groups 2 and 4. In 13 of these subjects the QTc_F values decreased to values < 450 ms during the course of the study, as determined at Visit 2 and Visit 4, if applicable. One subject in Group 3 with a QTc_F of 475.9 ms at screening still had an increased QTc_F at Visit 2 (468.6 ms) and Visit 5 (461.0 ms).

Of the subjects with QTc_F values < 450 ms at screening a QTc_F value greater than 450 ms was measured at Visit 2 in 3 subjects each in Groups 2 and 4 and in 1 subject in the placebo group (Group 3). Of these subjects only in 1 subject in Group 2 the absolute QTc_F value was also greater than 480 ms. All other QTc_F values measured at Visit 2 were 480 ms or less. All subjects in Groups 2 and 3 returned to QTc_F values < 450 ms at Visit 4.

Three subjects in Group 1 and one subject each in Groups 2 and 3 had QTc_F values > 450 ms and ≤ 480 ms for the first time after the second injection at Visit 4 (MVA-BN® in Group 1 and placebo in Groups 2 and 3) (see Table 12-7 and Section 16.2.9.4.3).

In one subject in Group 2 a QTc_F value greater than 480 ms was observed at Visit 5 (see Section 16.2.9.4.3).

There were no QTc_F values reported in this study that were greater than 500 ms (see Section 14.4.3.2).

A QTc_F change from baseline of 30 ms or more occurred in a total of 30 subjects at Visit 2 and in a total of 32 subjects at Visit 4 (see Section 14.4.3.3). A QTc_F change from baseline of 60 ms or more occurred only in one subject each in Groups 2 and 3 at Visit 2 (see Section 14.4.3.4).

Five subjects in Group 1, eleven subjects in Group 2, eight subjects in Group 3 (placebo) and six subjects in Group 4 experienced a QTc_F change from baseline of 30 ms or more at Visit 2.

At Visit 4 a QTc_F change from baseline of 30 ms or more occurred in 12 subjects in Group 1, 11 subjects in Group 2 and 9 subjects in Group 3.

Table 12-7 Absolute QTc values - threshold <=/> 450ms (Fridericia´s correction)

**Absolute QTc > 450ms**		**Screening**		**Visit 2**		**Visit 4**	
		**n**	**%**	**n**	**%**	**n**	**%**
Yes	Group 1 (MM)	5	2.7	-	0.0	3	1.7
	Group 2 (MP)	2	1.1	3	1.7	1	0.6
	Group 3 (PP)	5	2.8	2	1.1	1	0.6
	Group 4 (M-)	2	1.0	3	1.5	-	-
	Total	14	1.9	8	1.1	5	1.0
No	Group 1 (MM)	178	97.3	182	100.0	172	98.3
	Group 2 (MP)	178	98.9	175	98.3	171	99.4
	Group 3 (PP)	176	97.2	177	98.9	173	99.4
	Group 4 (M-)	197	99.0	196	98.5	-	-
	Total	729	98.1	730	98.9	516	99.0

n = number of subjects
Source data: Table 14.4.3.1

The mean, maximal and minimal absolute QTc_F values are listed in Table 12-8 by group and visit. Changes of the mean QTc_F values from baseline were minor and occurred in all groups, with the greatest absolute change being minus 2.4 ms observed in Group 3 (placebo) at Visit 4 (see Section 14.4.4.3). Also the QTc values corrected with the Bazett formulae show no significant changes in mean QTc_B values during the course of this study (see Section 14.4.5.4).

Table 12-8 Absolute QTc values (Fridericia´s correction)

[ms]		Visit		
		**Screening**	**Visit 2**	**Visit 4**
Group 1 (MM)	Mean	404.7	402.5	403.1
	Median	403.0	404.2	402.1
	StdDev	18.9	17.5	20.9
	StdErr	1.4	1.3	1.6
	Min	370.0	360.8	345.4
	Max	473.4	441.8	471.7
	N	183	182	175
Group 2 (MP)	Mean	407.1	407.3	408.5
	Median	409.2	408.2	407.4
	StdDev	19.9	20.4	19.6
	StdErr	1.5	1.5	1.5
	Min	357.4	356.9	355.0
	Max	456.1	480.9	457.3
	N	180	178	172
Group 3 (PP)	Mean	406.0	404.1	402.8
	Median	405.5	405.7	406.1
	StdDev	20.3	18.5	20.0
	StdErr	1.5	1.4	1.5
	Min	357.5	348.7	345.5
	Max	475.9	468.6	457.0
	N	181	179	174
Group 4 (M-)	Mean	406.8	406.5	-
	Median	407.4	407.5	-
	StdDev	20.3	19.3	-
	StdErr	1.4	1.4	-
	Min	336.9	355.1	-
	Max	466.8	454.1	-
	N	199	199	0
Total	Mean	406.2	405.1	404.8
	Median	406.4	406.7	405.1
	StdDev	19.8	19.0	20.3
	StdErr	0.7	0.7	0.9
	Min	336.9	348.7	345.4
	Max	475.9	480.9	471.7
	N	743	738	521

N= number of subjects Source data: Table 14.4.4.1

12.7 COMPARISON WITH SAFETY DATA OF DRYVAX® IMMUNIZATION

Since 2002, as a reaction to the threat of bioterrorism, the USA initiated anti-smallpox vaccination programs in which military personnel as well as civilians were vaccinated against smallpox using the Dryvax® vaccine (NYCBH live vaccinia strain).
During the recent US vaccination campaign, a statistically elevated rate of cases of myocarditis and pericarditis associated with Dryvax® immunization has been observed. Until then cardiac complications associated with the NYCBH strain had been rare or unrecognized according to historical literature.

From the 730,580 vaccinations that were performed by the US military (as of January 2005) a total of 89 cases (0.012%) of myo-/pericarditis were observed. As of January 2004 the Department of Health and Human Services (DHHS) initiated 40,422 vaccinations. Among these primary care responders, a total of 21 cases (0.052%) of myo-/pericarditis were reported. It is possible that cases of myo-/pericarditis were under-reported in both of the vaccination programs performed by the US military and DHHS, as only passive surveillance measures were used and sub-clinical cases might not have been detected. In the recently published results of a Phase I-III clinical program comparing an investigational 2nd generation smallpox vaccine (ACAM 2000) with Dryvax®, 3 cases of myo-/pericarditis in a total of 289 Dryvax® recipients (approx 1%) and 7 cases of myo-/pericarditis among the 873 subjects who received ACAM200 (approx. 1%) were reported (Vaccines and Related Biological Products Advisory Committee briefing document, 18 April 2007).

In this study being reported subjects were actively monitored for possible cardiac complications after vaccination with the study vaccine by regular ECG recordings and additional cardiac examinations, as necessary. Not a single case of myo-/pericarditis was reported in this study after injection of MVA-BN® or placebo. A total of 11 SIAEs were reported in the active phase of the study, of which 5 were classified as being possibly related to the study vaccine. During the FU phase further five SIAEs were reported. All were classified as unlikely related to the study vaccine. These included 3 incidences of palpitations and two incidences of tachycardia considered possibly related to the study vaccine. All subjects recovered.

As MVA-BN® is replication incompetent, severe AEs historically associated with the NYCBH strain (Dryvax®), such as inadvertent (auto-)inoculation, generalized vaccinia, eczema vaccinatum and progressive vaccinia, are not induced by MVA-BN® vaccination. Post-vaccinial encephalitis was a potentially fatal AE with a high incidence (42.3 cases per one million vaccinations) in small children under one year of age (Lane et al., 1970). There have been no cases of encephalitis reported in this study.

12.8 SAFETY CONCLUSIONS

One or two doses of MVA-BN® were shown to be safe and well tolerated.

Commonly observed AEs in this study were local symptoms at the injection site and general symptoms, such as fatigue, headache and myalgia. Common local signs included pain, erythema, induration and swelling, which occurred in 81.4% - 90.7% of the subjects who received two doses of MVA-BN® (Group 1). Less frequent were pruritus (23.5% of subjects in Group 1) and warmth (4.9% of subjects in Group 1) at the injection site.
Frequencies of local symptoms were significantly lower in the placebo group, which was expected as these AEs are known to be associated with injectable vaccines such as MVA-BN®. Injection site pain and erythema were moderately frequent also in the placebo group (approx. 20%) which could possibly be caused by other factors, e.g. the excipients in placebo, tissue damage due to the subcutaneous injection of 0.5 mL fluid, etc.

The majority of solicited local symptoms were mild or moderate. Only 3.3% or less of the subjects in any group had Grade 3 local reactions and no Grade 4 events were reported.

Fatigue, headache and myalgia occurred in 31.1%, 23.0% and 12.6% of the subjects in Group 1, respectively. The differences between Group 1 and placebo (Group 3), however, were minor and a relationship to MVA-BN® cannot be concluded but is possible. Fatigue and myalgia were more frequent in vaccinia-experienced subjects in Group 4, compared to the other groups.

Only one SAE was assessed by the investigator to be possibly treatment-related in this study. This event (sarcoidosis) was reported in a subject in Group 1 during the follow-up phase and was also judged to be possibly related to another (unknown) condition or treatment. Five SIAEs were considered possibly related to treatment and consisted of 3 cases of palpitations (2 events in one 46 year old subject) and two cases of tachycardia in subjects from Groups 2 and 4. All subjects completely recovered. The investigator judged both cases of tachycardia as also possibly related to nervousness and/or time pressure. The event of tachycardia in the subject from Group 2 occurred after the placebo injection.

ECG analyses did not show any clinically significant abnormal findings in any group. Neither significant QTc prolongations nor changes in mean PR, QRS and/or RR intervals associated with treatment were identified in this study.

No clinically significant abnormalities were observed in the clinical laboratory results, vital signs and physical examinations during this study.

13 DISCUSSION AND OVERALL CONCLUSIONS

Based on antibody titer analysis by ELISA it was shown in this study that a single dose of MVA-BN® injected in vaccinia-experienced subjects was sufficient to elicit seroconversion in 95.5% of the subjects at two weeks after the vaccination (Group 4). This was only slightly less then the seroconversion rate observed in vaccinia-naïve subjects (98.9%) who received two doses of MVA-BN® at two and four weeks after the second vaccination (Group 1). Statistically these seroconversion rates were not significantly different; however, at four weeks after the last vaccination a significant difference was observed in the seroconversion rates in Group 1 (98.9%) and Group 4 (93.0%).

The primary hypothesis of the seroconversion rate in Group 4 being non-inferior to the seroconversion rate in Group 1 could not be confirmed at two, as well as at four weeks after the last vaccination. This was essentially because the criteria of seroconversion was set too strict and in retrospect had little biological relevance as to the evaluation of the immunogenicity of MVA-BN®. Indeed, many of the non-seroconverters in Group 4 actually met the criteria at later time points, while subjects with high starting titers failed to meet the 2-fold increase, although a boost in the total antibody response to vaccinia was observed in all, but two subjects in Group 4 following the single vaccination with MVA-BN®. Indeed, although the lower seroconversion rate in Group 4 compared to Group 1 implied a lower relative change in antibody titers in Group 4, the vaccinia-specific peak GMT as measured by ELISA was in fact higher in the Group 4 subjects. A peak GMT of 568.819 in Group 4 was measured two weeks after the MVA-BN® vaccination (Visit 2) and a GMT of 495.843 in Group 1 two weeks after the second MVA-BN® vaccination (Visit 4). Therefore, the results of the study revealed that following two vaccinations with MVA-BN® strong antibody responses were induced in the majority of the vaccinia-naïve subjects and these responses were comparable to those generated in vaccinia-experienced subjects that received a single booster vaccination.

In comparison, a single vaccination with MVA-BN® followed by a placebo injection in subjects who had no history of smallpox vaccination (Group 2) resulted in a seroconversion in 87.4% of the subjects at four weeks after the MVA-BN® vaccination (87.4% was the highest seroconversion rate measured in Group 2).

Similarly, only 78.5% of the vaccinia-experienced subjects seroconverted by PRNT, 2 weeks post vaccination with MVA-BN®. However, out of the 43 non-seroconverters (2-fold increase) in Group 4, 19 would actually have fulfilled the criteria at later visits; clearly indicating that MVA-BN® vaccination induced a strong neutralizing antibody booster response in this population. Seroconversion rates were actually higher in the vaccinia-naïve subjects (Group 1) after the second vaccination, which again may be accounted by the strict criteria set for seroconversion particularly for the vaccinia-experienced subjects. Indeed, this was again highlighted by comparing the GMT generated by PRNT. The GMT in Group 1, 2 weeks post the second vaccination with MVA-BN® was 45.577, compared to 175.150 two weeks post MVA-BN® vaccination in vaccinia-experienced subjects (Group 4).

Importantly, when all outliers were taken into account the ELISA and PRNT titers were highly correlated at all visits for all groups. In general the GMT and seroconversion rates were lower by PRNT compared to ELISA and this is probably related to the fact the PRNT is only measuring a component of the total antibody responses measured by the ELISA. Moreover, the ELISA represents a more sensitive assay compared to the PRNT.

The primary safety objective of this study was to compare the four different vaccination groups with regard to ECG changes and cardiac symptoms. All ECG recordings were considered

normal or represented normal variations as assessed by the investigator and/or the local cardiologist. Neither significant QTc prolongations nor changes in PR, QRS and/or RR intervals resulting from vaccination were identified.

Secondary safety objectives of this study were a comparison with regard to safety of the four different vaccination groups and specifically of Groups 1 and 2 with the placebo group (Group 3) and with historical data of Dryvax® immunization in vaccinia-naïve subjects.

Comparing Group 1 with the placebo group, a clearly higher frequency of common local injection site symptoms was observed in Group 1, indicating a relationship to MVA-BN®. Very frequent local AEs were pain, erythema, induration and swelling. No clear relationship to MVA-BN® could be determined for general symptoms, such as fatigue, headache and myalgia. Headache and myalgia were most frequent in vaccinia-experienced subjects in Group 4.

After the second vaccination with MVA-BN® in Group 1, local Grade 2 and Grade 3 erythema, swelling and induration tended to be more frequent than after the first vaccination. Comparing vaccinia-naïve subjects (Groups 1 and 2) with vaccinia-experienced subjects (Group 4) after the first vaccination, overall frequencies of local solicited symptoms were similar, however, there was a tendency of an increased frequency of Grade 2 and Grade 3 erythema and induration in Group 4. Swelling was overall more frequent in Group 4 after the first vaccination.

After the second injection (MVA-BN® in Group 1 and placebo in Group 2) there were no major differences in solicited general AEs between Groups 1 and 2.

There were no treatment-related clinically significant abnormalities observed in the clinical laboratory results, vital signs and physical examinations in this study.

As compared to the unexpectedly high incidence of myo-/pericarditis following Dryvax® immunizations, in this study not a single case of myo-/pericarditis was reported.

Thus, the large amount of safety data, including extensive cardiac monitoring results, collected from the 745 subjects who completed this study confirm the excellent safety and tolerability profile of MVA-BN® and revealed no particular safety concerns in this healthy study population, also with regard to the development of cardiac events following vaccination with MVA-BN®.

14 TABLES, FIGURES AND GRAPHS REFERRED TO BUT NOT INCLUDED IN THE TEXT

Table of Contents

14. TABLES, FIGURES AND GRAPHS REFERRED TO BUT NOT INCLUDED IN THE TEXT 5

14.1 DEMOGRAPHIC DATA 5

14.1.1 Exception to inclusion/ exclusion criteria, Safety dataset 5
14.1.2 Overall Assessment, Reason for Premature Discontinuation - All Subjects 6
14.1.3 Subject characteristics - Safety (FA) dataset 7
14.1.4 Subject characteristics - PP dataset 9
14.1.5 Baseline signs and symptoms 11
14.1.5.1 Baseline signs and symptoms - outcome, Safety dataset 11
14.1.5.2 Baseline signs and symptoms - intensity, Safety dataset 12
14.1.5.3 Baseline signs and symptoms - action taken, Safety dataset 13
14.1.5.4 Baseline signs and symptoms - SAE or Special Interest AE (SIAE), Safety dataset 14
14.1.6 History of smallpox vaccination by presence of vaccination scar, Safety dataset 15
14.1.7 Medical history - number of normal/ abnormal findings per category, Safety dataset 16

14.2 IMMUNOGENICITY DATA 19

14.2.1 Antibody responses against vaccinia using a direct ELISA 19
14.2.1.1 ELISA-specific antibodies against vaccinia, seroconversion rates, responders - FA dataset 19
14.2.1.2 ELISA-specific antibodies against vaccinia, seroconversion rates, responders - PP dataset 20
14.2.1.3 ELISA-specific antibodies against vaccinia, descriptive statistics for log-transformed raw data, FA population 21
14.2.1.4 ELISA-specific antibodies against vaccinia, descriptive statistics for log-transformed raw data, PP population 24
14.2.1.5 ELISA-specific antibodies against vaccinia, figure of log-transformed data, FA population 27
14.2.1.6 ELISA-specific antibodies against vaccinia, figure of log-transformed data, PP population 29
14.2.1.7 ELISA-specific antibodies against vaccinia - figure of seroconversion rate, FA dataset 31
14.2.1.8 ELISA-specific antibodies against vaccinia - figure of seroconversion rate, FA dataset - Presentation by timepoint 2, 4 and 26 wks after last vaccination 32
14.2.1.9 ELISA-specific antibodies against vaccinia - figure of seroconversion rate, PP dataset 33
14.2.1.10 ELISA-specific antibodies against vaccinia - figure of seroconversion rate, PP dataset - Presentation by timepoint 2, 4 and 26 wks after last vaccination 34
14.2.2 Antibody responses against vaccinia using a plaque reduction neutralization test (PRNT) 35
14.2.2.1 PRNT-speficic antibodies against vaccinia, seroconversion rates, responders - FA dataset 35
14.2.2.2 PRNT-specific antibodies against vaccinia, seroconversion rates, responders - PP dataset 36
14.2.2.3 PRNT-specific antibodies against vaccinia, descriptive statistics for log-transformed raw data, FA population 37
14.2.2.4 PRNT-specific antibodies against vaccinia, descriptive statistics for log-transformed raw data, PP population 40
14.2.2.5 PRNT-specific antibodies against vaccinia, figure of log-transformed data, FA population 43

14.2.2.6 PRNT-specific antibodies against vaccinia, figure of log-transformed data, PP population....45
14.2.2.7 PRNT-specific antibodies against vaccinia - figure of seroconversion rate, FA dataset....47
14.2.2.8 PRNT-specific antibodies against vaccinia - figure of seroconversion rate, FA dataset - Presentation by timepoint 2, 4 and 26 wks after last vaccination....48
14.2.2.9 PRNT-specific antibodies against vaccinia - figure of seroconversion rate, PP dataset....49
14.2.2.10 PRNT-specific antibodies against vaccinia - figure of seroconversion rate, PP dataset - Presentation by timepoint 2, 4 and 26 wks after last vaccination....50
14.2.3 Correlation analysis between ELISA and PRNT antibody titer, FA dataset....51
14.2.3.1 Group 1 (MM)....51
14.2.3.2 Group 2 (MP)....63
14.2.3.3 Group 4 (M-)....75

14.3 LABORATORY DATA....83

14.3.1 Summary of laboratory data - Clinical Chemistry, descriptive statistics and count of abnormalities, Safety dataset....83
14.3.2 Summary of laboratory data - Haematology, descriptive statistics and count of abnormalities, Safety dataset....109
14.3.3 Laboratory abnormalities - Urinalysis (pH, specific gravity), Safety dataset....117
14.3.4 Laboratory abnormalities - Urinalysis (qualitative result parameters), Safety dataset....119
14.3.5 Laboratory abnormalities - Urine pregnancy test (qualitative result parameters), Safety dataset....127
14.3.6 Laboratory abnormalities, shift tables - Hematology, Safety dataset....128
14.3.6.1 Screening versus Visit 2....128
14.3.6.2 Screening versus Visit 4....140
14.3.6.3 Screening versus Visit 5....152
14.3.6.4 Screening versus Follow up....164
14.3.7 Laboratory abnormalities, shift tables - Serum Chemistry, Safety dataset....173
14.3.7.1 Screening versus Visit 2....173
14.3.7.2 Screening versus Visit 4....182
14.3.7.3 Screening versus Visit 5....191
14.3.7.4 Screening versus Follow up....200

14.4 ECG DATA....209

14.4.1 Count and percentage of subjects with normal/ abnormal and clinically significant ECG findings - Safety dataset....209
14.4.2 Count and percentage of subjects with Myo-/Pericarditis....215
14.4.3 ECG - abnormal ECGs....215
14.4.4 QTc analysis: categorical analysis....216
14.4.4.1 Absolute QTc values > 450ms (Fridericia´s correction)....216
14.4.4.2 Absolute QTc values > 480ms (Fridericia´s correction)....217
14.4.4.3 Absolute QTc value > 500ms (Fridericia´s correction)....218
14.4.4.4 Change from baseline >= 30ms (Fridericia´s correction)....219
14.4.4.5 Change from baseline >= 60ms (Fridericia´s correction)....220
14.4.5 QTc analysis: analysis of central tendency....221
14.4.5.1 Absolute QTc values (Fridericia´s correction)....221
14.4.5.2 Maximum post-dose values (Fridericia´s correction)....223
14.4.5.3 Change from baseline (Fridericia´s correction)....224
14.4.5.4 Mean maximal change from baseline (Fridericia´s correction)....226
14.4.6 Further ECG data....227

14.4.6.1 PR-duration 227
14.4.6.2 QRS-duration 229
14.4.6.3 QT-duration 231
14.4.6.4 QTc-duration (Bazett correction)....233
14.4.6.5 RR Interval 235

14.5 ADVERSE EVENT DATA 237

14.5.1 Unsolicted Adverse Events 237
14.5.1.1 Listings of deaths, other serious and significant adverse events....237
14.5.1.2 Narratives of deaths, other serious and certain other significant adverse events 242
14.5.1.3 Unsolicted Adverse Events: Overview, Safety dataset 243
14.5.1.4 Unsolicited adverse events: MedDRA coding by SOC, PT and subject, Safety dataset 245
14.5.1.5 Unsolicited adverse events: MedDRA coding by SOC and subject, Safety dataset...254
14.5.1.6 Unsolicited adverse events: MedDRA coding by PT and subject, Safety dataset 255
14.5.1.7 Unsolicited adverse events: MedDRA coding by SOC, PT, intensity and subject, Safety dataset 262
14.5.1.8 Unsolicited serious adverse events: MedDRA coding by SOC, PT and subject, Safety dataset 275
14.5.1.9 Unsolicited special interest adverse events: MedDRA coding by SOC, PT intensity and subject, Safety dataset....276
14.5.2 Solicited General Adverse Events 277
14.5.2.1 Summary of Maximum Intensity (7-day Follow-up Period after Vaccination) by vaccination period, Safety dataset 277
14.5.2.2 Summary of Maximum Intensity (7-day Follow-up Period after Vaccination) by subject, Safety dataset....281
14.5.2.3 Summary of Maximum Intensity by subject: categories grade=0/ >0, Safety dataset 283
14.5.2.4 Summary of Maximum Intensity by subject: categories grade<2/ >=2, Safety dataset 284
14.5.2.5 Summary of Maximum Intensity by subject: categories grade<3/ >=3, Safety dataset 285
14.5.2.6 Summary of Maximum Intensity (7-day Follow-up Period after Vaccination) by subject - related AEs, Safety dataset....286
14.5.3 Solicited Local Adverse Events....288
14.5.3.1 Summary of Maximum Intensity (7-Day Follow-up Period after Vaccination) by vaccination period, Safety dataset 288
14.5.3.2 Summary of Maximum Intensity (7-Day Follow-up Period after Vaccination) by subject, Safety dataset....292
14.5.3.3 Summary of Maximum Intensity by subject: categories grade=0/ >0, Safety dataset 294
14.5.3.4 Summary of Maximum Intensity by subject: categories grade<2/ >=2, Safety dataset 295
14.5.3.5 Summary of Maximum Intensity by subject: categories grade<3/ >=3, Safety dataset 296
14.5.4 Possibly/probably/definitely Related Adverse Events 297
14.5.4.1 Solicited (general / local) and unsolicited adverse events: MedDRA coding by SOC, PT, possible/probable/definite relationship to study medication, Safety dataset 297
14.5.5 SAEs, SIAEs and Follow up of Unresolved AEs during Follow up....302
14.5.5.1 Listings of SAE, SIAE and unresolved AEs 302
14.5.5.2 Unsolicted Adverse Events: Overview, Safety dataset 306
14.5.5.3 Unsolicited adverse events: MedDRA coding by SOC, PT and subject, Safety dataset 308
14.5.5.4 Unsolicited adverse events: MedDRA coding by SOC and subject, Safety dataset...310
14.5.5.5 Unsolicited adverse events: MedDRA coding by PT and subject, Safety dataset 311

14.6 FURTHER SAFETY DATA....312

14.6.1 Reason for study termination, Safety (FA) dataset 312
14.6.2 Vital signs 314
14.6.2.1 Vital signs - systolic blood pressure, Safety dataset 314
14.6.2.2 Vital signs - diastolic blood pressure, Safety dataset 315
14.6.2.3 Vital signs - pulse rate, Safety dataset 316
14.6.2.4 Vital signs - oral temperature, Safety dataset 317
14.6.3 Body weight, Safety dataset 318
14.6.4 Total study duration, time between visits, Safety dataset 319
14.6.5 Prior and concomitant medication by brand name, Safety dataset 322

14.7 ADDITIONAL EXPLORATORY SUBGROUP ANALYSIS BY AGE GROUP AND GENDER 339

14.7.1 Distribution of age and gender 339
14.7.1.1 Distribution of age - FA dataset 339
14.7.1.2 Distribution of gender - FA dataset 345
14.7.2 Antibodies against vaccinia, seroconversion rates 346
14.7.2.1 Tables 346
14.7.2.1.1 Antibodies against vaccinia, seroconversion rates, responders by gender - FA dataset 346
14.7.2.1.2 Antibodies against vaccinia, seroconversion rates, responders by gender - PP dataset 350
14.7.2.1.3 Antibodies against vaccinia, seroconversion rates, responders by age group - FA dataset 355
14.7.2.1.4 Antibodies against vaccinia, seroconversion rates, responders by age group - PP dataset 358
14.7.2.2 Figures 362
14.7.2.2.1 Age group 362
14.7.2.2.2 Gender 370
14.7.3 Antibodies against vaccinia - Presentation by timepoint 2 and 4 wks after last vaccination 378
14.7.3.1 Tables 378
14.7.3.1.1 Antibodies against vaccinia, seroconversion rates, responders by gender - FA dataset 378
14.7.3.1.2 Antibodies against vaccinia, seroconversion rates, responders by gender - PP dataset 383
14.7.3.1.3 Antibodies against vaccinia, seroconversion rates, responders by age group - FA dataset 387
14.7.3.1.4 Antibodies against vaccinia, seroconversion rates, responders by age group - PP dataset 390
14.7.3.2 Figures 394
14.7.3.2.1 Age group 394
14.7.3.2.2 Gender 398

14. TABLES, FIGURES AND GRAPHS REFERRED TO BUT NOT INCLUDED IN THE TEXT

14.1 DEMOGRAPHIC DATA

14.1.1 Exception to inclusion/ exclusion criteria, Safety dataset

	Group								Total	
	Group 1 (MM)		Group 2 (MP)		Group 3 (PP)		Group 4 (M-)			
	n	%	n	%	n	%	n	%	n	%
Any exceptions to eligibility criteria										
yes	2	1.1	5	2.8	11	6.1	11	5.5	29	3.9
no	181	98.9	176	97.2	170	93.9	189	94.5	716	96.1
Total	183	100.0	181	100.0	181	100.0	200	100.0	745	100.0

14.1.2 Overall Assessment, Reason for Premature Discontinuation - All Subjects

	Healthy vacc. naive						Healthy pre-immune		All Subjects		
	Group 1 (MM)		Group 2 (MP)		Group 3 (PP)		Group 4 (M-)				
	n	(%)	n	(%)	n	(%)	n	(%)	n	(%)	
Number of subjects screened	.		.		.		.		1322		[1]
Number of screening failures	.		.		.		.		577		
No. of subjects randomised	183		184		182		204		753		
No. of subjects who got at least one dose of vaccine	183	(100.0%)	181	(100.0%)	181	(100.0%)	200	(100.0%)	745	(100.0%)	
Returned for visit 2	182	(99.5%)	179	(98.9%)	179	(98.9%)	200	(100.0%)	740	(99.3%)	
Returned for/ completed visit 3	180	(98.4%)	174	(96.1%)	175	(96.7%)	200	(100.0%)	729	(97.9%)	
No. of subjects who got two doses of vaccine (Group 1-3 only)	180	(98.4%)	174	(96.1%)	175	(96.7%)	.		529	(97.1%)	[2]
Returned for visit 4	176	(96.2%)	174	(96.1%)	175	(96.7%)	.		525	(96.3%)	[2]
Completed visit 5/ Final Examination	178	(97.3%)	175	(96.7%)	177	(97.8%)	.		530	(97.2%)	[2]
Returned for follow up visit	178	(97.3%)	174	(96.1%)	175	(96.7%)	199	(99.5%)	726	(97.4%)	
Premature discontinuation (before V5)	8	(4.4%)	8	(4.4%)	6	(3.3%)	.		22	(3.0%)	[3]
Due to A	.		1	(0.6%)	1	(0.6%)	.		2	(0.3%)	
B	3	(1.6%)	6	(3.3%)	2	(1.1%)	.		11	(1.5%)	
C	5	(2.7%)	1	(0.6%)	2	(1.1%)	.		8	(1.1%)	
F	.		.		1	(0.6%)	.		1	(0.1%)	

n: Number of subjects %: Percentage based on number of subjects who got at least one dose of vaccine

[1] Number of subjects screened is based on status reports and is not based on the clinical database, because screening failures have not been necessarily entered

[2] Total percentage based on total number of subjects in group 1-3

[3] Reasons for premature termination (Percentages for totals are based on N=745):

A: An adverse event occurs that in the opinion of the investigator, makes it unsafe for the subject to continue the study.
B: Subject's request to withdraw
C: Subject unwilling or unable to comply with study requirements
D: Clinical need for concomitant or ancillary therapy not permitted in the study
E: Unrelated intercurrent illness that, in the judgment of the Principal Investigator, will affect assessment of clinical status to a significant degree F: Lost to follow-up G: Other

14.1.3 Subject characteristics - Safety (FA) dataset

Number of Patients (%) / Descriptive Statistics

		Group 1 (MM) (N=183)	Group 2 (MP) (N=181)	Group 3 (PP) (N=181)	Group 4 (M-) (N=200)	Total (N=745)	p-value
Age [years]							
	Mean	25.3	25.4	26.0	41.5	29.8	<0.001#
	Median	24.0	25.0	25.0	42.0	27.0	
	Std	4.98	4.38	5.10	7.59	9.07	
	SEM	0.37	0.33	0.38	0.54	0.33	
	Min	18.0	18.0	18.0	22.0	18.0	
	Max	50.0	44.0	50.0	55.0	55.0	
	N	183	181	181	200	745	
Height [cm]							
	Mean	174.4	172.6	173.8	172.2	173.2	0.103#
	Median	175.0	171.0	173.0	171.0	172.0	
	Std	9.97	8.98	9.75	9.36	9.54	
	SEM	0.74	0.67	0.72	0.66	0.35	
	Min	154.0	153.0	150.0	153.0	150.0	
	Max	204.0	197.0	200.0	195.0	204.0	
	N	183	181	181	200	745	
Weight [kg]							
	Mean	72.08	70.81	72.21	73.18	72.10	0.454#
	Median	70.30	69.40	69.10	70.60	69.80	
	Std	14.530	12.854	14.799	14.801	14.280	
	SEM	1.074	0.955	1.100	1.047	0.523	
	Min	45.10	47.40	47.30	46.10	45.10	
	Max	122.60	114.70	136.00	135.40	136.00	
	N	183	181	181	200	745	

Weight at screening visit
^ Fisher's Exact test
# ANOVA with factor treatment

Subject characteristics - Safety (FA) dataset

		Group 1 (MM) (N=183)	Group 2 (MP) (N=181)	Group 3 (PP) (N=181)	Group 4 (M-) (N=200)	Total (N=745)	p-value
		Number of Patients (%) / Descriptive Statistics					
Body mass index [kg/m²]							
	Mean	23.6	23.7	23.9	24.6	23.9	0.066#
	Median	23.0	23.2	23.1	23.9	23.2	
	Std	3.86	3.41	4.31	3.93	3.90	
	SEM	0.29	0.25	0.32	0.28	0.14	
	Min	17.5	17.4	16.2	17.6	16.2	
	Max	46.7	35.8	46.5	40.7	46.7	
	N	183	181	181	200	745	
Gender							
	male	86 (47.0)	69 (38.1)	74 (40.9)	85 (42.5)	314 (42.1)	0.379^
	female	97 (53.0)	112 (61.9)	107 (59.1)	115 (57.5)	431 (57.9)	
Ethnic group							
	caucasian	178 (97.3)	176 (97.2)	177 (97.8)	198 (99.0)	729 (97.9)	0.883^
	asian	1 (0.5)	2 (1.1)	1 (0.6)	0 (0.0)	4 (0.5)	
	black	0 (0.0)	1 (0.6)	0 (0.0)	1 (0.5)	2 (0.3)	
	arabic	2 (1.1)	1 (0.6)	1 (0.6)	0 (0.0)	4 (0.5)	
	other	2 (1.1)	1 (0.6)	2 (1.1)	1 (0.5)	6 (0.8)	

Weight at screening visit
^ Fisher's Exact test
# ANOVA with factor treatment

14.1.4 Subject characteristics - PP dataset

		Group 1 (MM) (N=168)	Group 2 (MP) (N=170)	Group 3 (PP) (N=164)	Group 4 (M-) (N=193)	Total (N=695)	p-value
		Number of Patients (%) / Descriptive Statistics					
Age [years]							
	Mean	25.4	25.5	26.0	41.9	30.2	<0.001#
	Median	24.0	25.0	25.0	42.0	27.0	
	Std	5.10	4.32	5.03	7.20	9.19	
	SEM	0.39	0.33	0.39	0.52	0.35	
	Min	18.0	18.0	18.0	24.0	18.0	
	Max	50.0	44.0	50.0	55.0	55.0	
	N	168	170	164	193	695	
Height [cm]							
	Mean	174.1	172.6	173.9	172.4	173.2	0.218#
	Median	174.0	171.5	173.0	171.0	173.0	
	Std	10.14	9.07	9.94	9.42	9.65	
	SEM	0.78	0.70	0.78	0.68	0.37	
	Min	154.0	153.0	150.0	153.0	150.0	
	Max	204.0	197.0	200.0	195.0	204.0	
	N	168	170	164	193	695	
Weight [kg]							
	Mean	72.31	70.46	72.79	73.53	72.31	0.222#
	Median	70.35	69.15	69.80	71.70	70.10	
	Std	14.727	12.550	14.991	14.941	14.366	
	SEM	1.136	0.963	1.171	1.075	0.545	
	Min	45.10	47.40	47.70	46.10	45.10	
	Max	122.60	114.70	136.00	135.40	136.00	
	N	168	170	164	193	695	

Weight at screening visit
^ Fisher's Exact test
# ANOVA with factor treatment

Subject characteristics - PP dataset

		Group 1 (MM) (N=168)	Group 2 (MP) (N=170)	Group 3 (PP) (N=164)	Group 4 (M-) (N=193)	Total (N=695)	p-value
		Number of Patients (%) / Descriptive Statistics					
Body mass index [kg/m²]							
	Mean	23.8	23.6	24.0	24.6	24.0	0.060#
	Median	23.1	23.2	23.1	24.0	23.3	
	Std	3.91	3.32	4.40	3.97	3.93	
	SEM	0.30	0.25	0.34	0.29	0.15	
	Min	17.5	17.4	18.3	17.6	17.4	
	Max	46.7	35.0	46.5	40.7	46.7	
	N	168	170	164	193	695	
Gender							
	male	78 (46.4)	67 (39.4)	70 (42.7)	85 (44.0)	300 (43.2)	0.620^
	female	90 (53.6)	103 (60.6)	94 (57.3)	108 (56.0)	395 (56.8)	
Ethnic group							
	caucasian	164 (97.6)	165 (97.1)	160 (97.6)	191 (99.0)	680 (97.8)	0.880^
	asian	1 (0.6)	2 (1.2)	1 (0.6)	0 (0.0)	4 (0.6)	
	black	0 (0.0)	1 (0.6)	0 (0.0)	1 (0.5)	2 (0.3)	
	arabic	2 (1.2)	1 (0.6)	1 (0.6)	0 (0.0)	4 (0.6)	
	other	1 (0.6)	1 (0.6)	2 (1.2)	1 (0.5)	5 (0.7)	

Weight at screening visit
^ Fisher's Exact test
# ANOVA with factor treatment

14.1.5 Baseline signs and symptoms

14.1.5.1 Baseline signs and symptoms - outcome, Safety dataset

	Number of Subjects (%) - Number of events				
Outcome	Group 1 (MM) (N=183)	Group 2 (MP) (N=181)	Group 3 (PP) (N=181)	Group 4 (M-) (N=200)	Total (N=745)
Total	28 (15.3) 34	28 (15.5) 29	28 (15.5) 30	29 (14.5) 32	113 (15.2) 125
recovery without sequelae	22 (12.0) 26	23 (12.7) 23	21 (11.6) 21	16 (8.0) 18	82 (11.0) 88
ongoing	8 (4.4) 8	5 (2.8) 6	9 (5.0) 9	13 (6.5) 13	35 (4.7) 36
recovery with sequelae	0 (0.0) 0	0 (0.0) 0	0 (0.0) 0	1 (0.5) 1	1 (0.1) 1

A subject with more than one finding in a specific category was only counted once
Percentages based on total no. of subjects in each group

14.1.5.2 Baseline signs and symptoms - intensity, Safety dataset

	Number of Subjects (%) - Number of events				
Intensity	Group 1 (MM) (N=183)	Group 2 (MP) (N=181)	Group 3 (PP) (N=181)	Group 4 (M-) (N=200)	Total (N=745)
Total	28 (15.3) 34	28 (15.5) 29	28 (15.5) 30	29 (14.5) 32	113 (15.2) 125
easily tolerated by subject	23 (12.6) 28	22 (12.2) 23	23 (12.7) 25	22 (11.0) 25	90 (12.1) 101
sufficiently discomforting to interfere with daily activities	5 (2.7) 6	6 (3.3) 6	5 (2.8) 5	7 (3.5) 7	23 (3.1) 24

A subject with more than one finding in a specific category was only counted once
Percentages based on total no. of subjects in each group

14.1.5.3 Baseline signs and symptoms - action taken, Safety dataset

	Number of Subjects (%) - Number of events				
Action taken	Group 1 (MM) (N=183)	Group 2 (MP) (N=181)	Group 3 (PP) (N=181)	Group 4 (M-) (N=200)	Total (N=745)
Total	28 (15.3) 34	28 (15.5) 29	28 (15.5) 30	29 (14.5) 32	113 (15.2) 125
concomitant medication	19 (10.4) 23	16 (8.8) 16	17 (9.4) 17	16 (8.0) 17	68 (9.1) 73
no action	9 (4.9) 9	13 (7.2) 13	11 (6.1) 11	14 (7.0) 14	47 (6.3) 47
non-drug therapy	2 (1.1) 2	0 (0.0) 0	1 (0.6) 1	1 (0.5) 1	4 (0.5) 4
hospitalization	0 (0.0) 0	0 (0.0) 0	1 (0.6) 1	0 (0.0) 0	1 (0.1) 1

A subject with more than one finding in a specific category was only counted once
Percentages based on total no. of subjects in each group

14.1.5.4 Baseline signs and symptoms - SAE or Special Interest AE (SIAE), Safety dataset

	Number of Subjects (%) - Number of events				
SAE or Special Interest AE (SIAE)	Group 1 (MM) (N=183)	Group 2 (MP) (N=181)	Group 3 (PP) (N=181)	Group 4 (M-) (N=200)	Total (N=745)
Total	28 (15.3) 34	28 (15.5) 29	28 (15.5) 30	29 (14.5) 32	113 (15.2) 125
no	28 (15.3) 34	28 (15.5) 29	28 (15.5) 30	29 (14.5) 32	113 (15.2) 125

A subject with more than one finding in a specific category was only counted once
Percentages based on total no. of subjects in each group

14.1.6 History of smallpox vaccination by presence of vaccination scar, Safety dataset

		Presence of typical vaccination scar?						Total	
		-		yes		no			
		n	%	n	%	n	%	n	%
Group 4 (M-)	History of smallpox vaccination?								
	yes	-	0.0	198	99.0	2	1.0	200	100.0
	no	-	0.0	-	0.0	-	0.0	-	-

14.1.7 Medical history - number of normal/ abnormal findings per category, Safety dataset

	Group 1 (MM) (N=183)	Group 2 (MP) (N=181)	Group 3 (PP) (N=181)	Group 4 (M-) (N=200)	Total (N=745)
skin					
Total	183 (100.0)	181 (100.0)	181 (100.0)	200 (100.0)	745 (100.0)
normal	179 (97.8)	177 (97.8)	177 (97.8)	186 (93.0)	719 (96.5)
abnormal	4 (2.2)	4 (2.2)	4 (2.2)	14 (7.0)	26 (3.5)
head, eyes, ears, nose and throat					
Total	183 (100.0)	181 (100.0)	181 (100.0)	200 (100.0)	745 (100.0)
normal	166 (90.7)	157 (86.7)	164 (90.6)	179 (89.5)	666 (89.4)
abnormal	17 (9.3)	24 (13.3)	17 (9.4)	21 (10.5)	79 (10.6)
respiratory					
Total	183 (100.0)	181 (100.0)	181 (100.0)	200 (100.0)	745 (100.0)
normal	178 (97.3)	171 (94.5)	177 (97.8)	193 (96.5)	719 (96.5)
abnormal	5 (2.7)	10 (5.5)	4 (2.2)	7 (3.5)	26 (3.5)
cardiovascular					
Total	183 (100.0)	181 (100.0)	181 (100.0)	200 (100.0)	745 (100.0)
normal	179 (97.8)	170 (93.9)	177 (97.8)	191 (95.5)	717 (96.2)
abnormal	4 (2.2)	11 (6.1)	4 (2.2)	9 (4.5)	28 (3.8)
gastrointestinal and hepatic					
Total	183 (100.0)	181 (100.0)	181 (100.0)	200 (100.0)	745 (100.0)
normal	165 (90.2)	158 (87.3)	168 (92.8)	180 (90.0)	671 (90.1)
abnormal	18 (9.8)	23 (12.7)	13 (7.2)	20 (10.0)	74 (9.9)
renal and genitourinary					
Total	183 (100.0)	181 (100.0)	181 (100.0)	200 (100.0)	745 (100.0)
normal	175 (95.6)	170 (93.9)	172 (95.0)	189 (94.5)	706 (94.8)
abnormal	8 (4.4)	11 (6.1)	9 (5.0)	11 (5.5)	39 (5.2)

A subject with more than one finding in a specific category was only counted once
Percentages based on total no. of subjects in each group

Medical history - number of normal/ abnormal findings per category, Safety dataset

	Group 1 (MM) (N=183)	Group 2 (MP) (N=181)	Group 3 (PP) (N=181)	Group 4 (M-) (N=200)	Total (N=745)
endocrine metabolic					
Total	183 (100.0)	181 (100.0)	181 (100.0)	200 (100.0)	745 (100.0)
normal	176 (96.2)	172 (95.0)	173 (95.6)	186 (93.0)	707 (94.9)
abnormal	7 (3.8)	9 (5.0)	8 (4.4)	14 (7.0)	38 (5.1)
hemopoietic and lymphatic					
Total	183 (100.0)	181 (100.0)	181 (100.0)	200 (100.0)	745 (100.0)
normal	182 (99.5)	178 (98.3)	180 (99.4)	198 (99.0)	738 (99.1)
abnormal	1 (0.5)	3 (1.7)	1 (0.6)	2 (1.0)	7 (0.9)
musculoskeletal					
Total	183 (100.0)	181 (100.0)	181 (100.0)	200 (100.0)	745 (100.0)
normal	158 (86.3)	152 (84.0)	157 (86.7)	162 (81.0)	629 (84.4)
abnormal	25 (13.7)	29 (16.0)	24 (13.3)	38 (19.0)	116 (15.6)
surgical history					
Total	183 (100.0)	181 (100.0)	181 (100.0)	200 (100.0)	745 (100.0)
normal	172 (94.0)	171 (94.5)	175 (96.7)	166 (83.0)	684 (91.8)
abnormal	11 (6.0)	10 (5.5)	6 (3.3)	34 (17.0)	61 (8.2)
psychiatric events					
Total	183 (100.0)	181 (100.0)	181 (100.0)	200 (100.0)	745 (100.0)
normal	173 (94.5)	174 (96.1)	178 (98.3)	196 (98.0)	721 (96.8)
abnormal	10 (5.5)	7 (3.9)	3 (1.7)	3 (1.5)	23 (3.1)
unknown	0 (0.0)	0 (0.0)	0 (0.0)	1 (0.5)	1 (0.1)
drug abuse of all kinds					
Total	183 (100.0)	181 (100.0)	181 (100.0)	200 (100.0)	745 (100.0)
normal	183 (100.0)	180 (99.4)	181 (100.0)	200 (100.0)	744 (99.9)
abnormal	0 (0.0)	1 (0.6)	0 (0.0)	0 (0.0)	1 (0.1)

A subject with more than one finding in a specific category was only counted once
Percentages based on total no. of subjects in each group

Medical history - number of normal/ abnormal findings per category, Safety dataset

	Group 1 (MM) (N=183)	Group 2 (MP) (N=181)	Group 3 (PP) (N=181)	Group 4 (M-) (N=200)	Total (N=745)
history of allergic reactions					
Total	183 (100.0)	181 (100.0)	181 (100.0)	200 (100.0)	745 (100.0)
normal	102 (55.7)	113 (62.4)	119 (65.7)	131 (65.5)	465 (62.4)
abnormal	81 (44.3)	68 (37.6)	62 (34.3)	69 (34.5)	280 (37.6)
other					
Total	183 (100.0)	181 (100.0)	181 (100.0)	200 (100.0)	745 (100.0)
normal	149 (81.4)	140 (77.3)	155 (85.6)	164 (82.0)	608 (81.6)
abnormal	34 (18.6)	41 (22.7)	26 (14.4)	36 (18.0)	137 (18.4)

A subject with more than one finding in a specific category was only counted once
Percentages based on total no. of subjects in each group

14.2 IMMUNOGENICITY DATA

14.2.1 Antibody responses against vaccinia using a direct ELISA

14.2.1.1 ELISA-specific antibodies against vaccinia, seroconversion rates, responders - FA dataset

		Number of Patients (%) / Descriptive Statistics			
		Group 1 (MM) (N=183)	Group 2 (MP) (N=181)	Group 3 (PP) (N=181)	Group 4 (M-) (N=200)
V2					
	yes	129 (70.9)	130 (72.6)	5 (2.8)	191 (95.5)
	no	53 (29.1)	49 (27.4)	173 (97.2)	9 (4.5)
V3					
	yes	160 (88.9)	152 (87.4)	5 (2.9)	185 (93.0)
	no	20 (11.1)	22 (12.6)	170 (97.1)	14 (7.0)
V4					
	yes	174 (98.9)	143 (82.2)	6 (3.4)	0 (0.0)
	no	2 (1.1)	31 (17.8)	169 (96.6)	0 (0.0)
V5					
	yes	176 (98.9)	126 (72.0)	5 (2.8)	0 (0.0)
	no	2 (1.1)	49 (28.0)	172 (97.2)	0 (0.0)
FU					
	yes	130 (73.0)	66 (37.9)	4 (2.3)	135 (67.8)
	no	48 (27.0)	108 (62.1)	171 (97.7)	64 (32.2)

Seroconversion is defined as the appearance of antibody titers >= 1:50 in a vaccinia specific IgG ELISA for initially seronegative subjects or twofold increase of the antibody titer compared to the pre-existing baseline titer for subjects with a pre-existing antibody titer in the ELISA.

14.2.1.2 ELISA-specific antibodies against vaccinia, seroconversion rates, responders - PP dataset

		Number of Patients (%) / Descriptive Statistics			
		Group 1 (MM) (N=168)	Group 2 (MP) (N=170)	Group 3 (PP) (N=164)	Group 4 (M-) (N=193)
V2					
	yes	117 (69.6)	123 (72.4)	5 (3.1)	186 (96.4)
	no	51 (30.4)	47 (27.6)	158 (96.9)	7 (3.6)
V3					
	yes	149 (88.7)	148 (87.1)	5 (3.0)	178 (92.7)
	no	19 (11.3)	22 (12.9)	159 (97.0)	14 (7.3)
V4					
	yes	166 (98.8)	139 (81.8)	6 (3.7)	0 (0.0)
	no	2 (1.2)	31 (18.2)	158 (96.3)	0 (0.0)
V5					
	yes	166 (98.8)	122 (71.8)	5 (3.0)	0 (0.0)
	no	2 (1.2)	48 (28.2)	159 (97.0)	0 (0.0)
FU					
	yes	121 (72.5)	64 (38.3)	4 (2.5)	131 (68.2)
	no	46 (27.5)	103 (61.7)	159 (97.5)	61 (31.8)

Seroconversion is defined as the appearance of antibody titers >= 1:50 in a vaccinia specific IgG ELISA for initially seronegative subjects or twofold increase of the antibody titer compared to the pre-existing baseline titer for subjects with a pre-existing antibody titer in the ELISA.

14.2.1.3 ELISA-specific antibodies against vaccinia, descriptive statistics for log-transformed raw data, FA population

	Number of Patients (%) / Descriptive statistics			
	Group 1 (MM) (N=183)	Group 2 (MP) (N=181)	Group 3 (PP) (N=181)	Group 4 (M-) (N=200)
V1				
Mean	0.158	0.104	0.059	1.589
Median	0.000	0.000	0.000	1.699
Geomean	1.440	1.271	1.147	38.845
Std	0.5425	0.4368	0.3239	0.8655
SEM	0.0401	0.0325	0.0241	0.0612
Min	0.000	0.000	0.000	0.000
Max	3.055	2.436	2.255	3.068
N	183	181	181	200
V2				
Mean	1.444	1.466	0.089	2.755
Median	1.699	1.699	0.000	2.811
Geomean	27.772	29.262	1.228	568.819
Std	0.9583	0.9105	0.3890	0.5725
SEM	0.0710	0.0681	0.0292	0.0405
Min	0.000	0.000	0.000	0.000
Max	3.751	3.533	2.000	3.948
N	182	179	178	200
V3				
Mean	1.856	1.781	0.099	2.655
Median	2.000	2.000	0.000	2.667
Geomean	71.847	60.343	1.255	452.321
Std	0.6872	0.6885	0.4031	0.4693
SEM	0.0512	0.0522	0.0305	0.0333

Geometric mean calculated from computing the arithmetic mean of the log-transformed values (i.e. the arithmetic mean on the log scale) and then using the exponentiation to return the geometric mean
Other descriptive statistics are based on log-transformed values

ELISA-sepcific antibodies against vaccinia, descriptive statistics for log-transformed raw data, FA population

	Number of Patients (%) / Descriptive statistics			
	Group 1 (MM) (N=183)	Group 2 (MP) (N=181)	Group 3 (PP) (N=181)	Group 4 (M-) (N=200)
Min	0.000	0.000	0.000	0.000
Max	4.065	3.314	2.000	3.845
N	180	174	175	199
V4				
Mean	2.695	1.627	0.091	.
Median	2.635	1.699	0.000	.
Geomean	495.843	42.327	1.234	
Std	0.4038	0.7485	0.4287	.
SEM	0.0304	0.0567	0.0324	.
Min	0.000	0.000	0.000	.
Max	3.959	3.455	2.959	.
N	176	174	175	0
V5				
Mean	2.517	1.365	0.082	.
Median	2.515	1.699	0.000	.
Geomean	328.666	23.193	1.206	
Std	0.3835	0.8435	0.3779	.
SEM	0.0287	0.0638	0.0284	.
Min	0.000	0.000	0.000	.
Max	3.888	2.896	2.233	.
N	178	175	177	0

Geometric mean calculated from computing the arithmetic mean of the log-transformed values (i.e. the arithmetic mean on the log scale) and then using the exponentiation to return the geometric mean
Other descriptive statistics are based on log-transformed values

ELISA-sepcific antibodies against vaccinia, descriptive statistics for log-transformed raw data, FA population

	Number of Patients (%) / Descriptive statistics			
	Group 1 (MM) (N=183)	Group 2 (MP) (N=181)	Group 3 (PP) (N=181)	Group 4 (M-) (N=200)
FU				
Mean	1.446	0.723	0.071	2.255
Median	1.699	0.000	0.000	2.356
Geomean	27.900	5.279	1.179	179.907
Std	0.8767	0.9060	0.3537	0.5887
SEM	0.0657	0.0687	0.0267	0.0417
Min	0.000	0.000	0.000	0.000
Max	3.945	2.898	2.312	3.897
N	178	174	175	199

Geometric mean calculated from computing the arithmetic mean of the log-transformed values (i.e. the arithmetic mean on the log scale) and then using the exponentiation to return the geometric mean
Other descriptive statistics are based on log-transformed values

14.2.1.4 ELISA-specific antibodies against vaccinia, descriptive statistics for log-transformed raw data, PP population

Number of Patients (%) / Descriptive statistics

	Group 1 (MM) (N=168)	Group 2 (MP) (N=170)	Group 3 (PP) (N=164)	Group 4 (M-) (N=193)
V1				
Mean	0.163	0.101	0.041	1.638
Median	0.000	0.000	0.000	2.000
Geomean	1.454	1.262	1.100	43.469
Std	0.5517	0.4331	0.2629	0.8337
SEM	0.0426	0.0332	0.0205	0.0600
Min	0.000	0.000	0.000	0.000
Max	3.055	2.436	1.699	3.068
N	168	170	164	193
V2				
Mean	1.425	1.464	0.085	2.797
Median	1.699	1.699	0.000	2.818
Geomean	26.588	29.094	1.217	627.020
Std	0.9745	0.9149	0.3770	0.4985
SEM	0.0752	0.0702	0.0295	0.0359
Min	0.000	0.000	0.000	0.000
Max	3.751	3.533	2.000	3.948
N	168	170	163	193
V3				
Mean	1.850	1.772	0.093	2.674
Median	2.000	2.000	0.000	2.690
Geomean	70.721	59.158	1.239	472.215
Std	0.7079	0.6939	0.3881	0.4643
SEM	0.0546	0.0532	0.0303	0.0335

Geometric mean calculated from computing the arithmetic mean of the log-transformed values (i.e. the arithmetic mean on the log scale) and then using the exponentiation to return the geometric mean
Other descriptive statistics are based on log-transformed values

ELISA-sepcific antibodies against vaccinia, descriptive statistics for log-transformed raw data, PP population

	Number of Patients (%) / Descriptive statistics			
	Group 1 (MM) (N=168)	Group 2 (MP) (N=170)	Group 3 (PP) (N=164)	Group 4 (M-) (N=193)
Min	0.000	0.000	0.000	0.000
Max	4.065	3.314	1.699	3.845
N	168	170	164	192
V4				
Mean	2.692	1.616	0.085	.
Median	2.631	1.699	0.000	.
Geomean	492.036	41.336	1.216	
Std	0.4057	0.7533	0.4163	.
SEM	0.0313	0.0578	0.0325	.
Min	0.000	0.000	0.000	.
Max	3.959	3.455	2.959	.
N	168	170	164	0
V5				
Mean	2.514	1.360	0.074	.
Median	2.509	1.699	0.000	.
Geomean	326.671	22.921	1.187	
Std	0.3824	0.8448	0.3539	.
SEM	0.0295	0.0648	0.0276	.
Min	0.000	0.000	0.000	.
Max	3.888	2.896	2.000	.
N	168	170	164	0

Geometric mean calculated from computing the arithmetic mean of the log-transformed values (i.e. the arithmetic mean on the log scale) and then using the exponentiation to return the geometric mean
Other descriptive statistics are based on log-transformed values

ELISA-sepcific antibodies against vaccinia, descriptive statistics for log-transformed raw data, PP population

	Number of Patients (%) / Descriptive statistics			
	Group 1 (MM) (N=168)	Group 2 (MP) (N=170)	Group 3 (PP) (N=164)	Group 4 (M-) (N=193)
FU				
Mean	1.439	0.731	0.063	2.295
Median	1.699	0.000	0.000	2.368
Geomean	27.481	5.379	1.155	197.113
Std	0.8833	0.9078	0.3209	0.5252
SEM	0.0683	0.0702	0.0251	0.0379
Min	0.000	0.000	0.000	0.000
Max	3.945	2.898	1.699	3.897
N	167	167	163	192

Geometric mean calculated from computing the arithmetic mean of the log-transformed values (i.e. the arithmetic mean on the log scale) and then using the exponentiation to return the geometric mean
Other descriptive statistics are based on log-transformed values

14.2.1.5 ELISA-specific antibodies against vaccinia, figure of log-transformed data, FA population

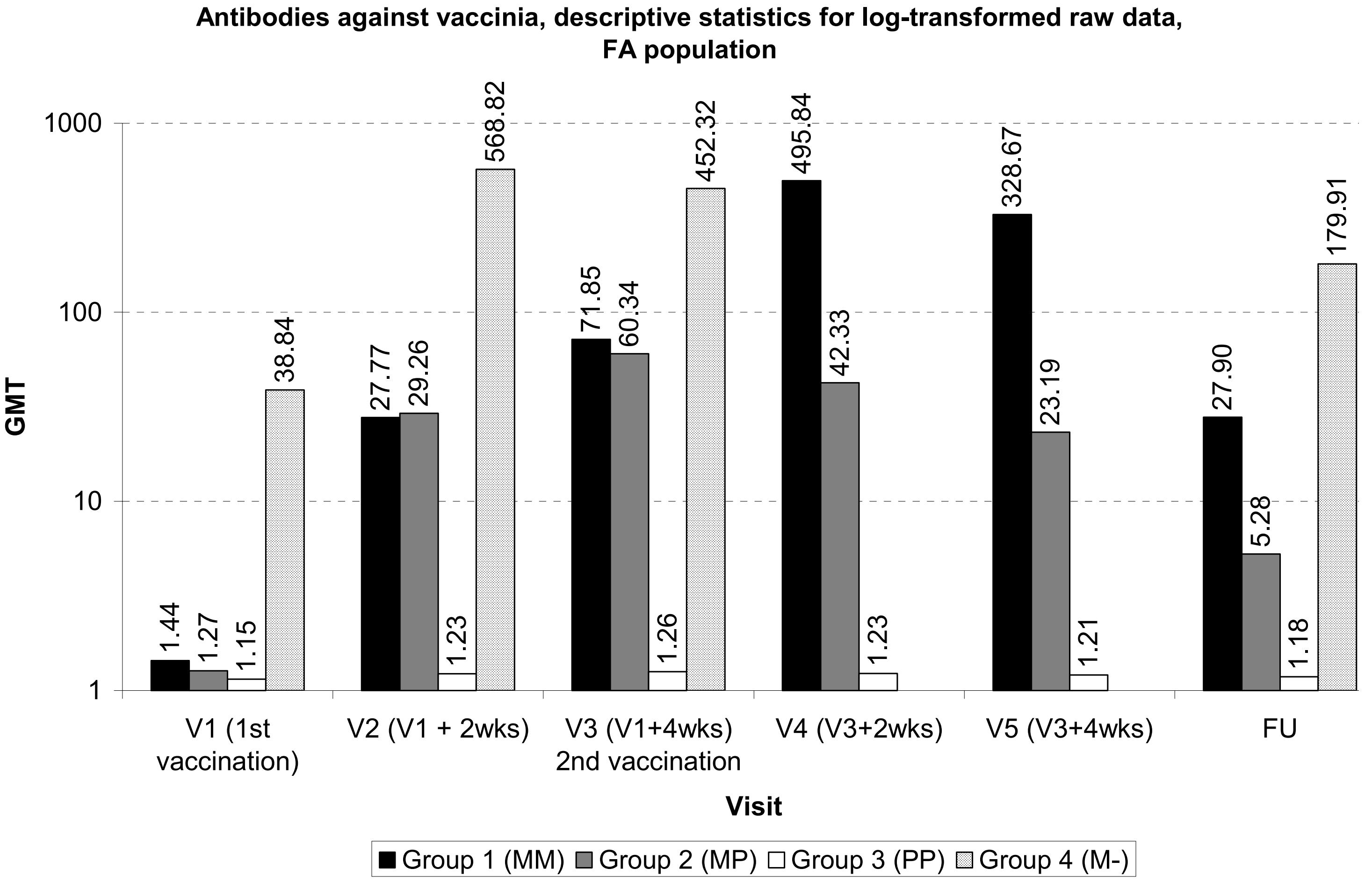

Antibodies against vaccinia, descriptive statistics for log-transformed raw data, FA population

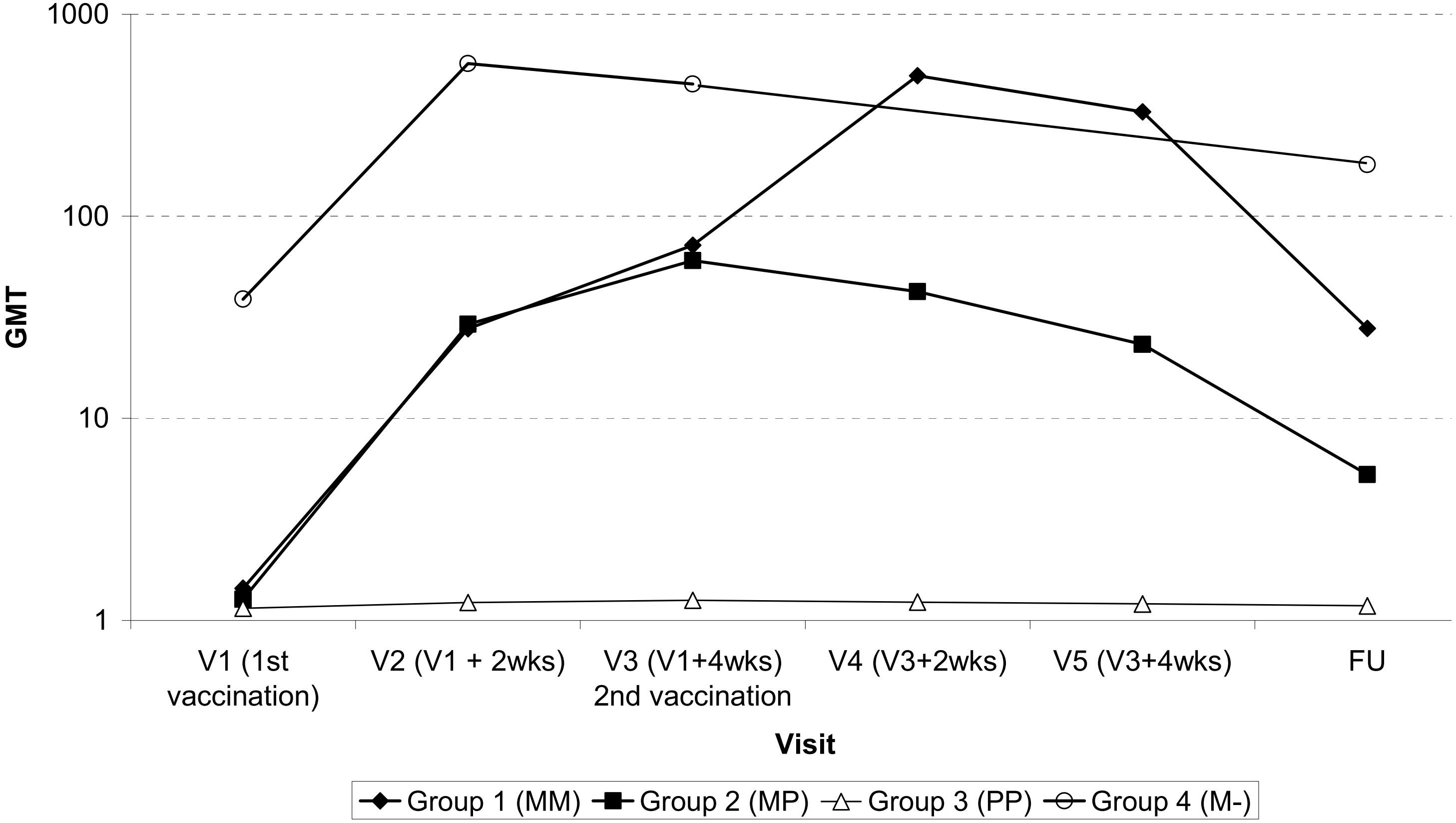

14.2.1.6 ELISA-specific antibodies against vaccinia, figure of log-transformed data, PP population

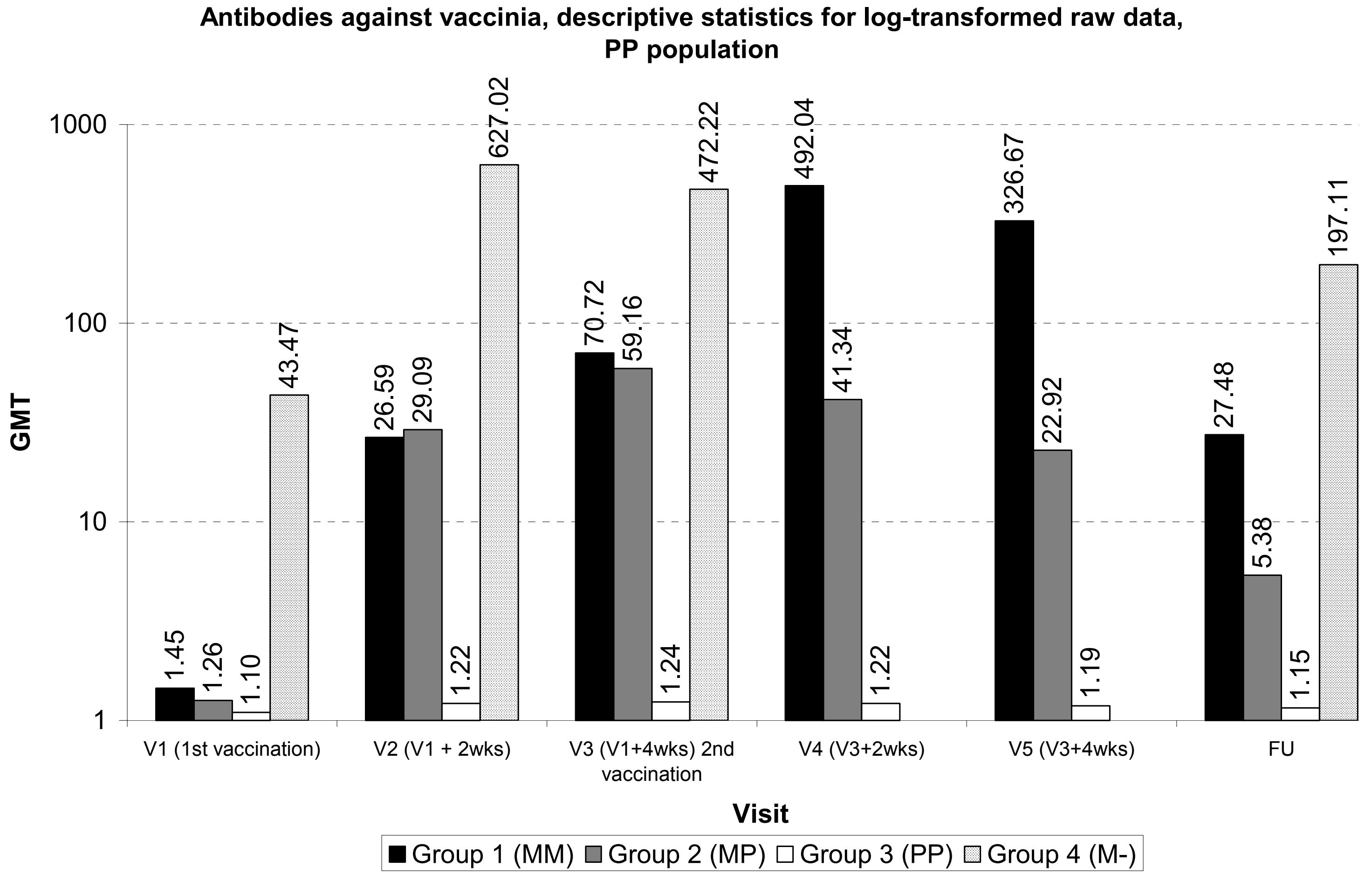

Antibodies against vaccinia, descriptive statistics for log-transformed raw data, PP population

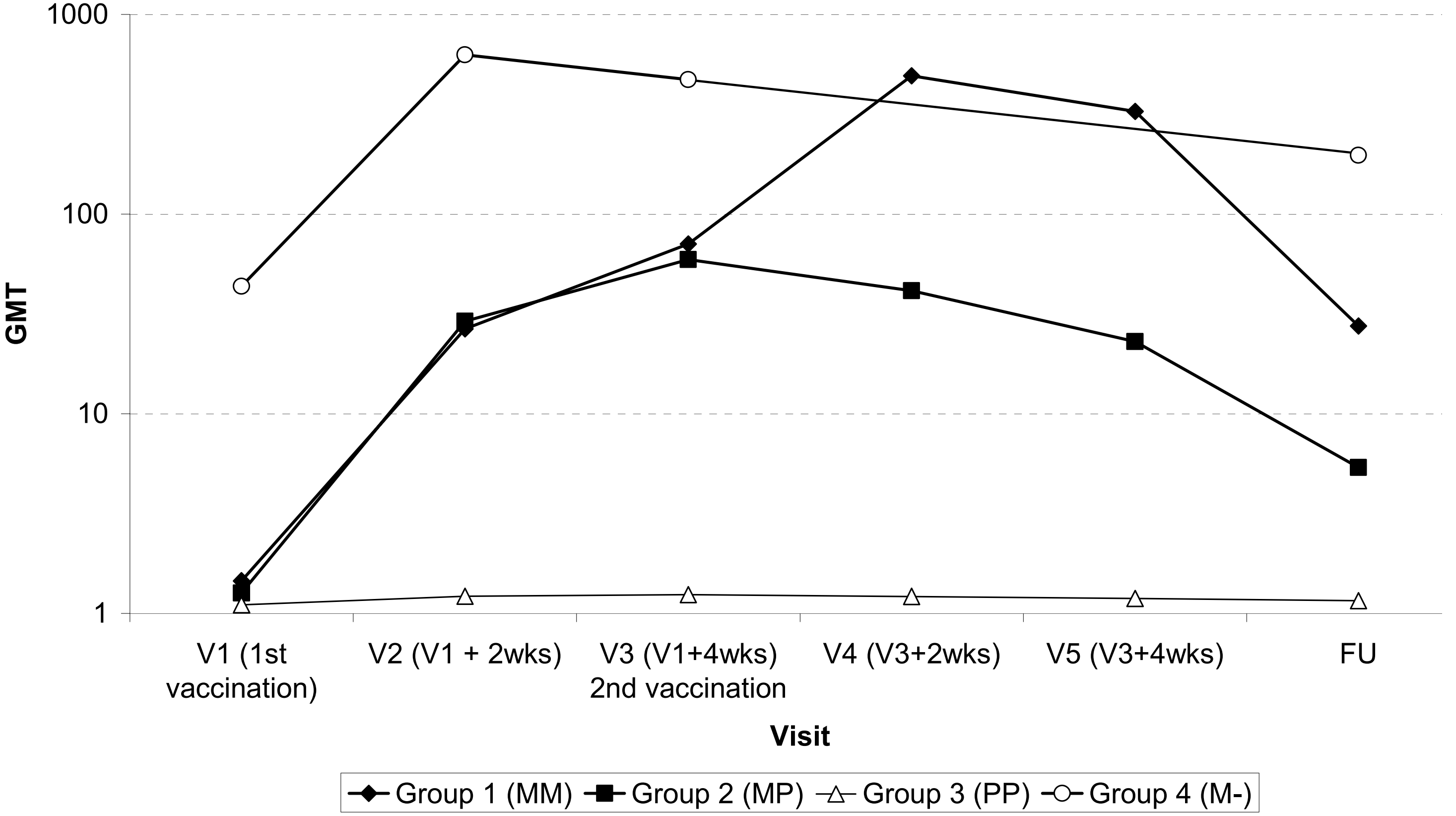

14.2.1.7 ELISA-specific antibodies against vaccinia - figure of seroconversion rate, FA dataset

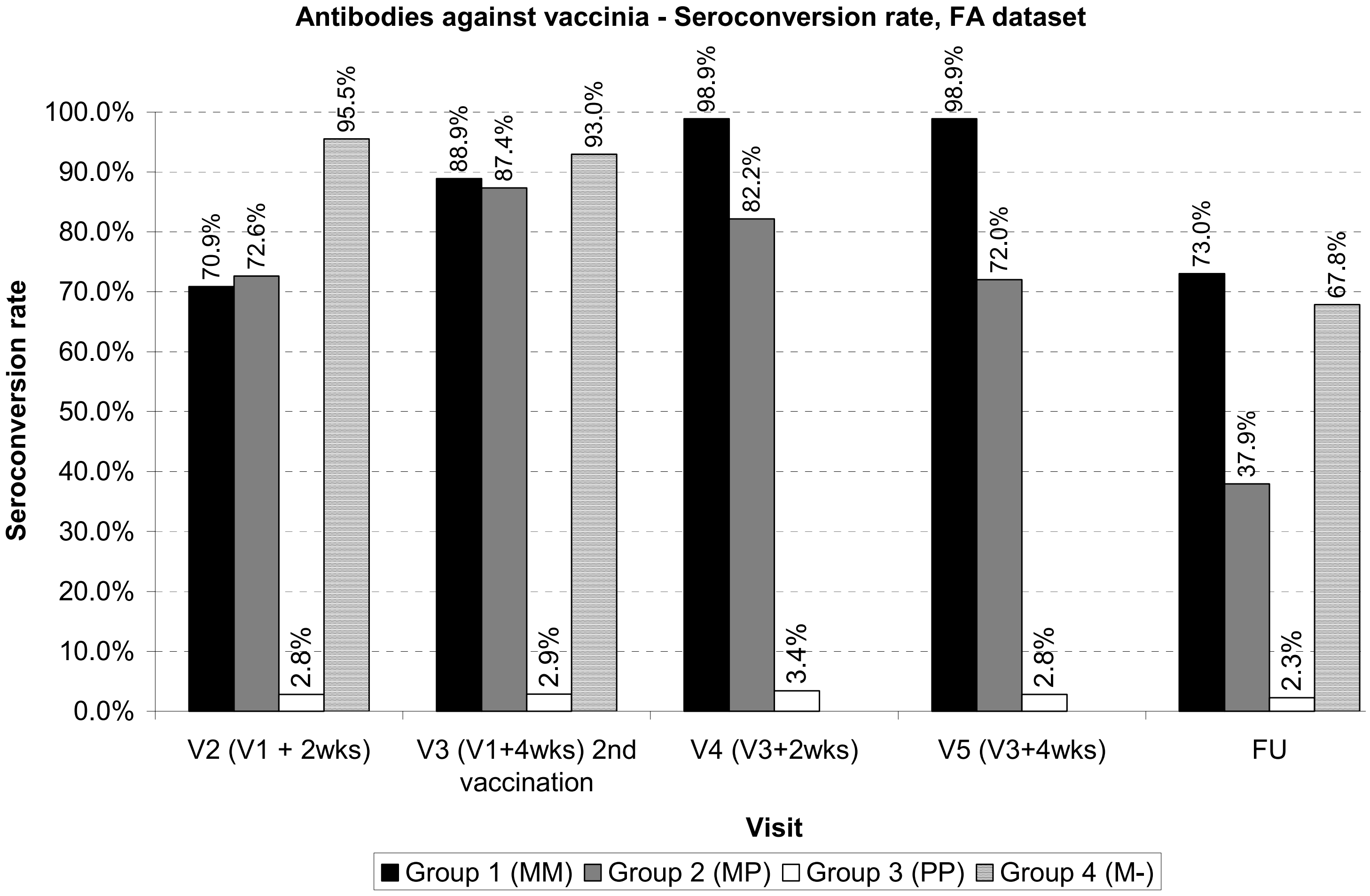

14.2.1.8 ELISA-specific antibodies against vaccinia - figure of seroconversion rate, FA dataset - Presentation by timepoint 2, 4 and 26 wks after last vaccination

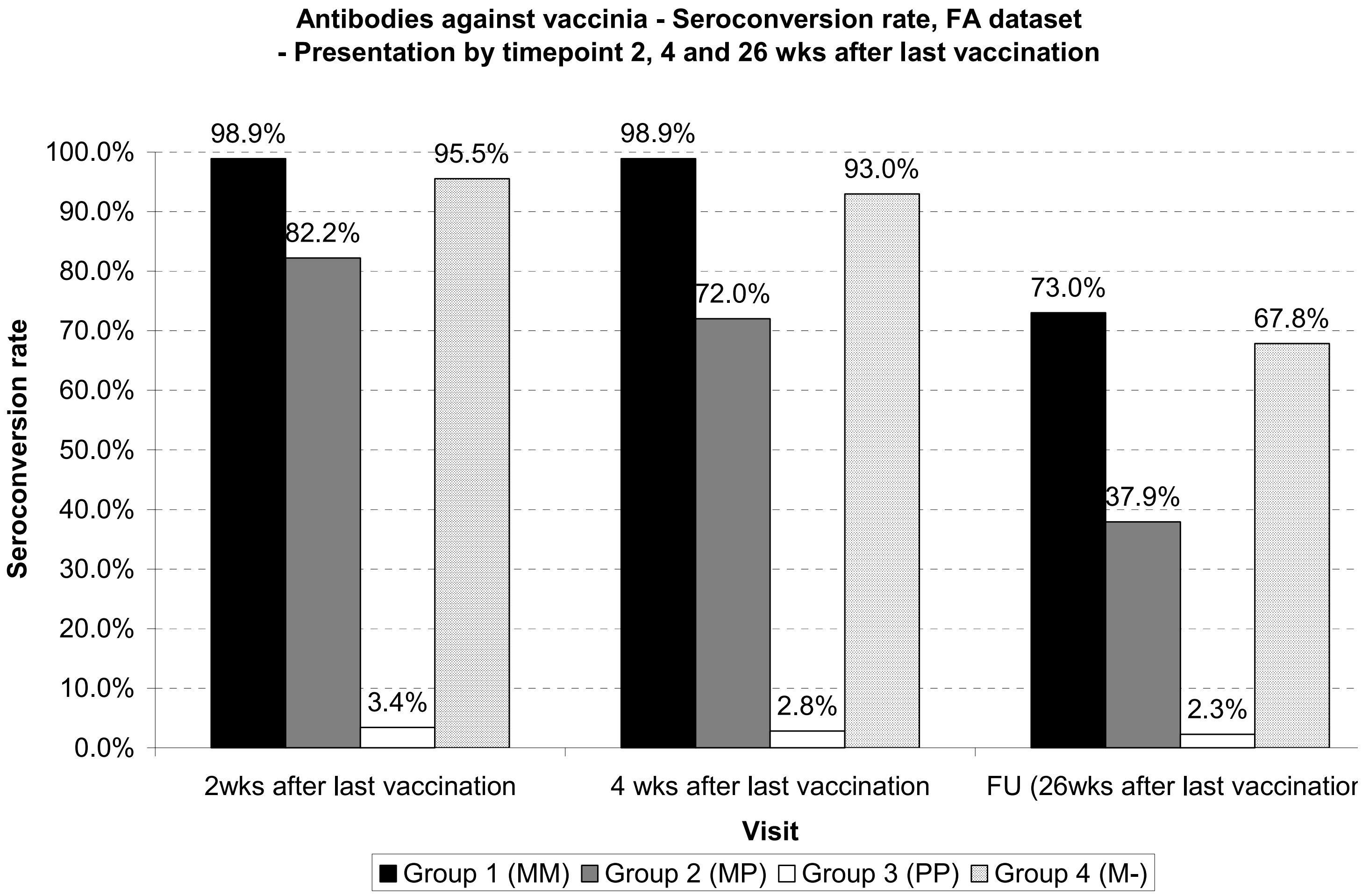

14.2.1.9 ELISA-specific antibodies against vaccinia - figure of seroconversion rate, PP dataset

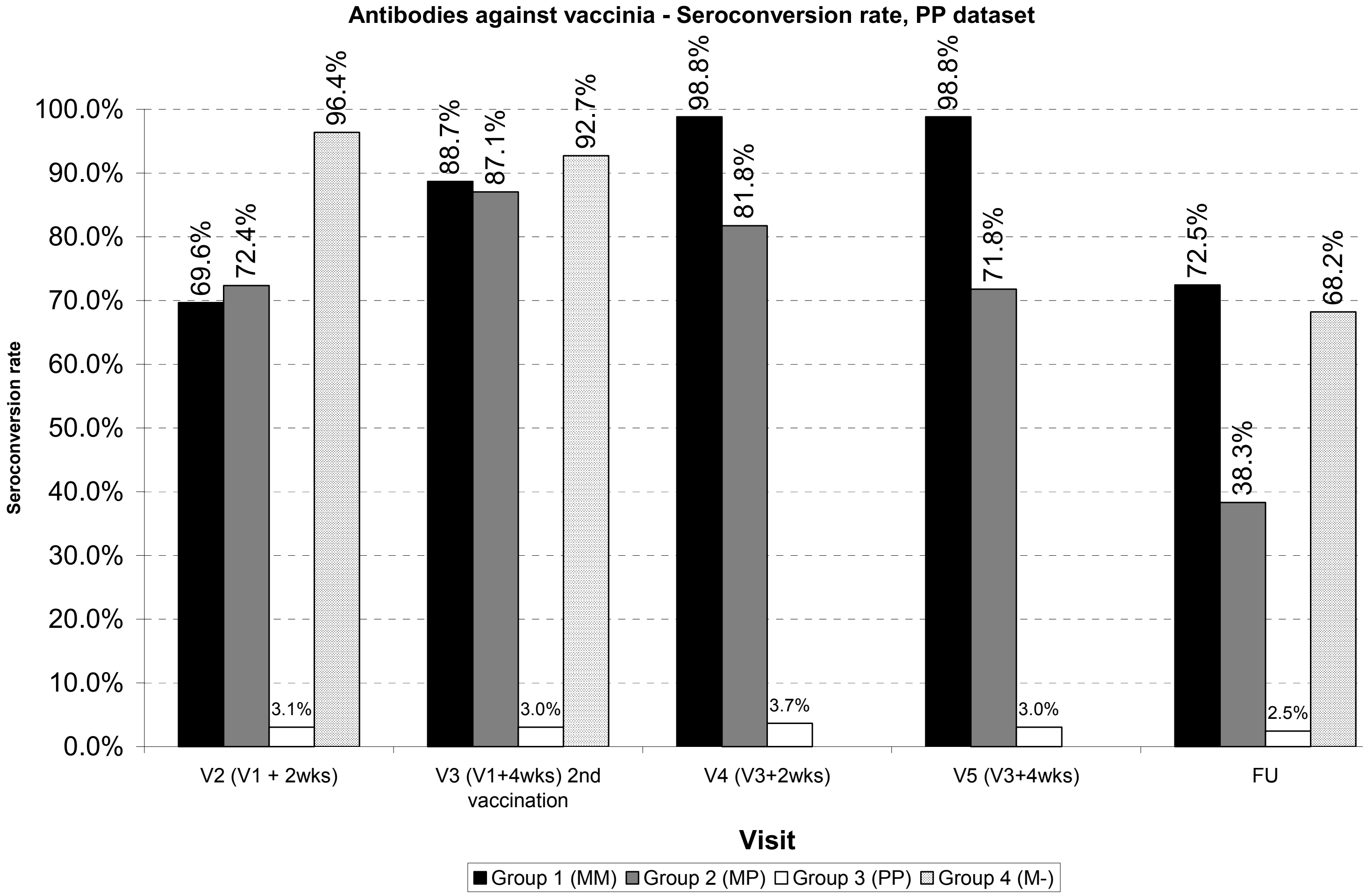

14.2.1.10 ELISA-specific antibodies against vaccinia - figure of seroconversion rate, PP dataset - Presentation by timepoint 2, 4 and 26 wks after last vaccination

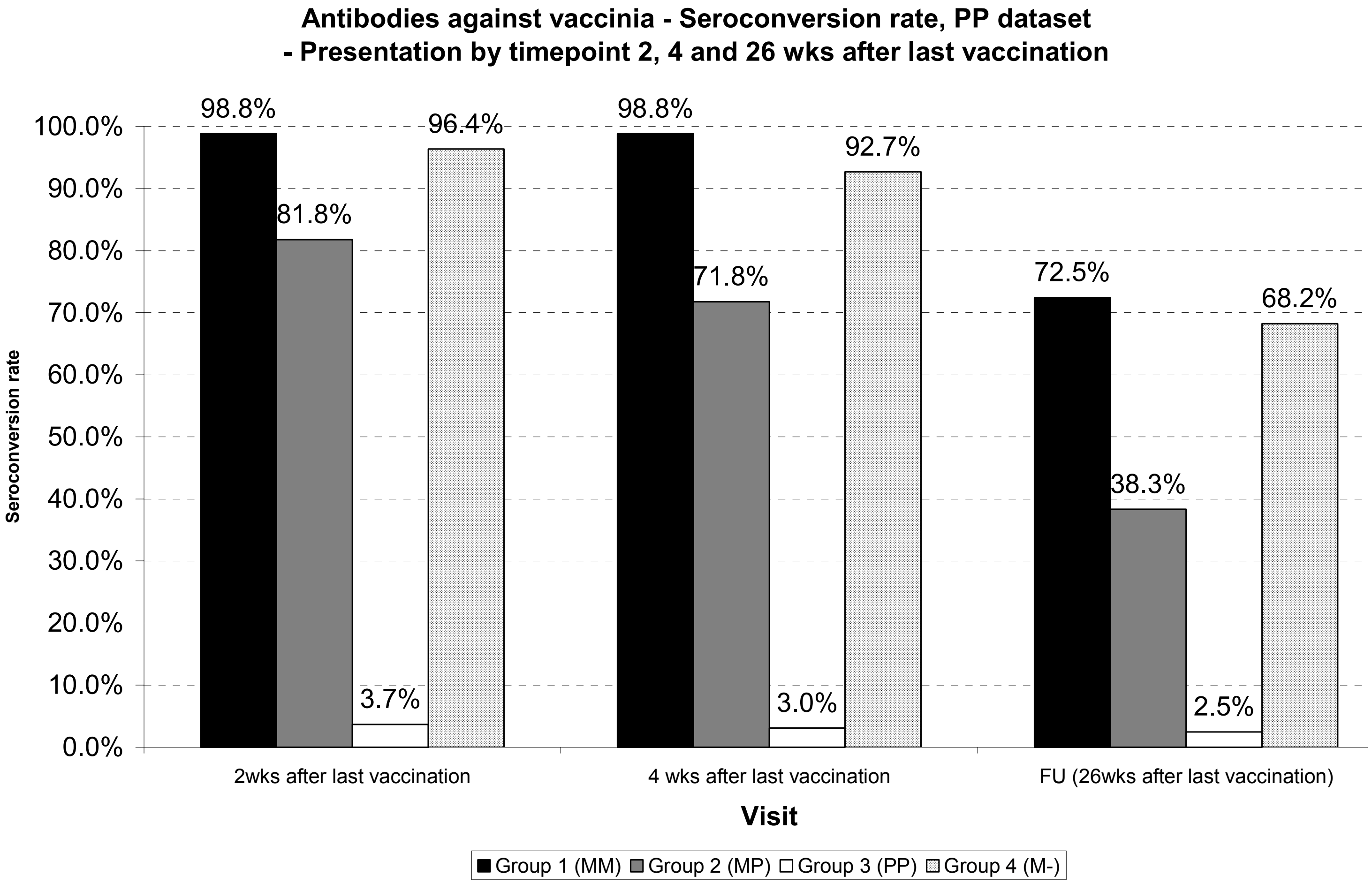

14.2.2 Antibody responses against vaccinia using a plaque reduction neutralization test (PRNT)

14.2.2.1 PRNT-speficic antibodies against vaccinia, seroconversion rates, responders - FA dataset

		Number of Patients (%) / Descriptive Statistics			
		Group 1 (MM) (N=183)	Group 2 (MP) (N=181)	Group 3 (PP) (N=181)	Group 4 (M-) (N=200)
V2					
	yes	82 (45.1)	93 (52.0)	2 (1.1)	157 (78.5)
	no	100 (54.9)	86 (48.0)	176 (98.9)	43 (21.5)
V3					
	yes	102 (56.7)	108 (62.1)	1 (0.6)	139 (69.8)
	no	78 (43.3)	66 (37.9)	174 (99.4)	60 (30.2)
V4					
	yes	157 (89.2)	98 (56.3)	0 (0.0)	0 (0.0)
	no	19 (10.8)	76 (43.7)	175 (100.0)	0 (0.0)
V5					
	yes	153 (86.0)	83 (47.4)	0 (0.0)	0 (0.0)
	no	25 (14.0)	92 (52.6)	177 (100.0)	0 (0.0)
FU					
	yes	116 (65.2)	41 (23.6)	1 (0.6)	127 (63.8)
	no	62 (34.8)	133 (76.4)	174 (99.4)	72 (36.2)

Seroconversion is defined as the appearance of antibody titers >= 1:6 in a vaccinia specific PRNT assay for initially seronegative subjects or twofold increase of the antibody titer compared to the pre-existing baseline titer for subjects with a pre-existing antibody titer in the PRNT.

14.2.2.2 PRNT-specific antibodies against vaccinia, seroconversion rates, responders - PP dataset

		Number of Patients (%) / Descriptive Statistics			
		Group 1 (MM) (N=168)	Group 2 (MP) (N=170)	Group 3 (PP) (N=164)	Group 4 (M-) (N=193)
V2					
	yes	75 (44.6)	89 (52.4)	2 (1.2)	152 (78.8)
	no	93 (55.4)	81 (47.6)	161 (98.8)	41 (21.2)
V3					
	yes	96 (57.1)	105 (61.8)	0 (0.0)	132 (68.8)
	no	72 (42.9)	65 (38.2)	164 (100.0)	60 (31.3)
V4					
	yes	149 (88.7)	95 (55.9)	0 (0.0)	0 (0.0)
	no	19 (11.3)	75 (44.1)	164 (100.0)	0 (0.0)
V5					
	yes	144 (85.7)	81 (47.6)	0 (0.0)	0 (0.0)
	no	24 (14.3)	89 (52.4)	164 (100.0)	0 (0.0)
FU					
	yes	107 (64.1)	39 (23.4)	0 (0.0)	123 (64.1)
	no	60 (35.9)	128 (76.6)	163 (100.0)	69 (35.9)

Seroconversion is defined as the appearance of antibody titers >= 1:6 in a vaccinia specific PRNT assay for initially seronegative subjects or twofold increase of the antibody titer compared to the pre-existing baseline titer for subjects with a pre-existing antibody titer in the PRNT.

14.2.2.3 PRNT-specific antibodies against vaccinia, descriptive statistics for log-transformed raw data, FA population

Number of Patients (%) / Descriptive statistics

	Group 1 (MM) (N=183)	Group 2 (MP) (N=181)	Group 3 (PP) (N=181)	Group 4 (M-) (N=200)
V1				
Mean	0.051	0.023	0.013	1.334
Median	0.000	0.000	0.000	1.607
Geomean	1.125	1.055	1.030	21.586
Std	0.3067	0.1702	0.1282	0.8669
SEM	0.0227	0.0126	0.0095	0.0613
Min	0.000	0.000	0.000	0.000
Max	2.053	1.869	1.544	3.978
N	183	181	181	200
V2				
Mean	0.679	0.709	0.021	2.243
Median	0.000	0.778	0.000	2.336
Geomean	4.780	5.118	1.050	175.150
Std	0.8318	0.7658	0.1454	0.6967
SEM	0.0617	0.0572	0.0109	0.0493
Min	0.000	0.000	0.000	0.000
Max	3.540	2.612	1.415	3.814
N	182	179	178	200

Geometric mean calculated from computing the arithmetic mean of the log-transformed values (i.e. the arithmetic mean on the log scale) and then using the exponentiation to return the geometric mean
Other descriptive statistics are based on log-transformed values

PRNT-specific antibodies against vaccinia, descriptive statistics for log-transformed raw data, FA population

	Number of Patients (%) / Descriptive statistics			
	Group 1 (MM) (N=183)	Group 2 (MP) (N=181)	Group 3 (PP) (N=181)	Group 4 (M-) (N=200)
V3				
Mean	0.878	0.857	0.011	2.159
Median	0.778	0.778	0.000	2.272
Geomean	7.544	7.189	1.025	144.285
Std	0.8405	0.7681	0.1426	0.6265
SEM	0.0626	0.0582	0.0108	0.0444
Min	0.000	0.000	0.000	0.000
Max	3.039	2.636	1.886	3.753
N	180	174	175	199
V4				
Mean	1.659	0.769	0.010	.
Median	1.949	0.778	0.000	.
Geomean	45.577	5.878	1.022	
Std	0.7660	0.7493	0.1257	.
SEM	0.0577	0.0568	0.0095	.
Min	0.000	0.000	0.000	.
Max	3.186	2.644	1.663	.
N	176	174	175	0
V5				
Mean	1.532	0.622	0.009	.
Median	1.763	0.000	0.000	.
Geomean	34.007	4.192	1.020	
Std	0.7488	0.7215	0.1161	.
SEM	0.0561	0.0545	0.0087	.
Min	0.000	0.000	0.000	.
Max	3.187	2.491	1.544	.
N	178	175	177	0

Geometric mean calculated from computing the arithmetic mean of the log-transformed values (i.e. the arithmetic mean on the log scale) and then using the exponentiation to return the geometric mean
Other descriptive statistics are based on log-transformed values

PRNT-specific antibodies against vaccinia, descriptive statistics for log-transformed raw data, FA population

	Number of Patients (%) / Descriptive statistics			
	Group 1 (MM) (N=183)	Group 2 (MP) (N=181)	Group 3 (PP) (N=181)	Group 4 (M-) (N=200)
FU				
Mean	0.860	0.272	0.011	2.027
Median	0.778	0.000	0.000	2.125
Geomean	7.236	1.871	1.026	106.463
Std	0.7533	0.5317	0.1470	0.5512
SEM	0.0565	0.0403	0.0111	0.0392
Min	0.000	0.000	0.000	0.000
Max	2.961	2.124	1.944	3.803
N	178	174	175	198

Geometric mean calculated from computing the arithmetic mean of the log-transformed values (i.e. the arithmetic mean on the log scale) and then using the exponentiation to return the geometric mean
Other descriptive statistics are based on log-transformed values

14.2.2.4 PRNT-specific antibodies against vaccinia, descriptive statistics for log-transformed raw data, PP population

Number of Patients (%) / Descriptive statistics

	Group 1 (MM) (N=168)	Group 2 (MP) (N=170)	Group 3 (PP) (N=164)	Group 4 (M-) (N=193)
V1				
Mean	0.056	0.025	0.005	1.383
Median	0.000	0.000	0.000	1.633
Geomean	1.137	1.059	1.011	24.131
Std	0.3197	0.1755	0.0608	0.8436
SEM	0.0247	0.0135	0.0047	0.0607
Min	0.000	0.000	0.000	0.000
Max	2.053	1.869	0.778	3.978
N	168	170	164	193
V2				
Mean	0.670	0.712	0.014	2.284
Median	0.000	0.778	0.000	2.373
Geomean	4.675	5.149	1.034	192.207
Std	0.8333	0.7644	0.1049	0.6605
SEM	0.0643	0.0586	0.0082	0.0475
Min	0.000	0.000	0.000	0.000
Max	3.540	2.612	0.778	3.814
N	168	170	163	193

Geometric mean calculated from computing the arithmetic mean of the log-transformed values (i.e. the arithmetic mean on the log scale) and then using the exponentiation to return the geometric mean
Other descriptive statistics are based on log-transformed values

PRNT-specific antibodies against vaccinia, descriptive statistics for log-transformed raw data, PP population

	Number of Patients (%) / Descriptive statistics			
	Group 1 (MM) (N=168)	Group 2 (MP) (N=170)	Group 3 (PP) (N=164)	Group 4 (M-) (N=193)
V3				
Mean	0.882	0.852	0.000	2.181
Median	0.778	0.778	0.000	2.321
Geomean	7.616	7.119	1.000	151.877
Std	0.8372	0.7689	0.0000	0.6225
SEM	0.0646	0.0590	0.0000	0.0449
Min	0.000	0.000	0.000	0.000
Max	3.039	2.636	0.000	3.753
N	168	170	164	192
V4				
Mean	1.649	0.759	0.000	.
Median	1.929	0.778	0.000	.
Geomean	44.551	5.742	1.000	
Std	0.7697	0.7474	0.0000	.
SEM	0.0594	0.0573	0.0000	.
Min	0.000	0.000	0.000	.
Max	3.186	2.644	0.000	.
N	168	170	164	0
V5				
Mean	1.522	0.622	0.000	.
Median	1.740	0.000	0.000	.
Geomean	33.289	4.190	1.000	
Std	0.7504	0.7197	0.0000	.
SEM	0.0579	0.0552	0.0000	.
Min	0.000	0.000	0.000	.
Max	3.187	2.491	0.000	.
N	168	170	164	0

Geometric mean calculated from computing the arithmetic mean of the log-transformed values (i.e. the arithmetic mean on the log scale)
and then using the exponentiation to return the geometric mean
Other descriptive statistics are based on log-transformed values

PRNT-specific antibodies against vaccinia, descriptive statistics for log-transformed raw data, PP population

	Number of Patients (%) / Descriptive statistics			
	Group 1 (MM) (N=168)	Group 2 (MP) (N=170)	Group 3 (PP) (N=164)	Group 4 (M-) (N=193)
FU				
Mean	0.841	0.270	0.000	2.065
Median	0.778	0.000	0.000	2.143
Geomean	6.927	1.861	1.000	116.186
Std	0.7536	0.5310	0.0000	0.4952
SEM	0.0583	0.0411	0.0000	0.0358
Min	0.000	0.000	0.000	0.000
Max	2.961	2.124	0.000	3.803
N	167	167	163	191

Geometric mean calculated from computing the arithmetic mean of the log-transformed values (i.e. the arithmetic mean on the log scale) and then using the exponentiation to return the geometric mean
Other descriptive statistics are based on log-transformed values

14.2.2.5 PRNT-specific antibodies against vaccinia, figure of log-transformed data, FA population

Antibodies against vaccinia, descriptive statistics for log-transformed raw data, FA population

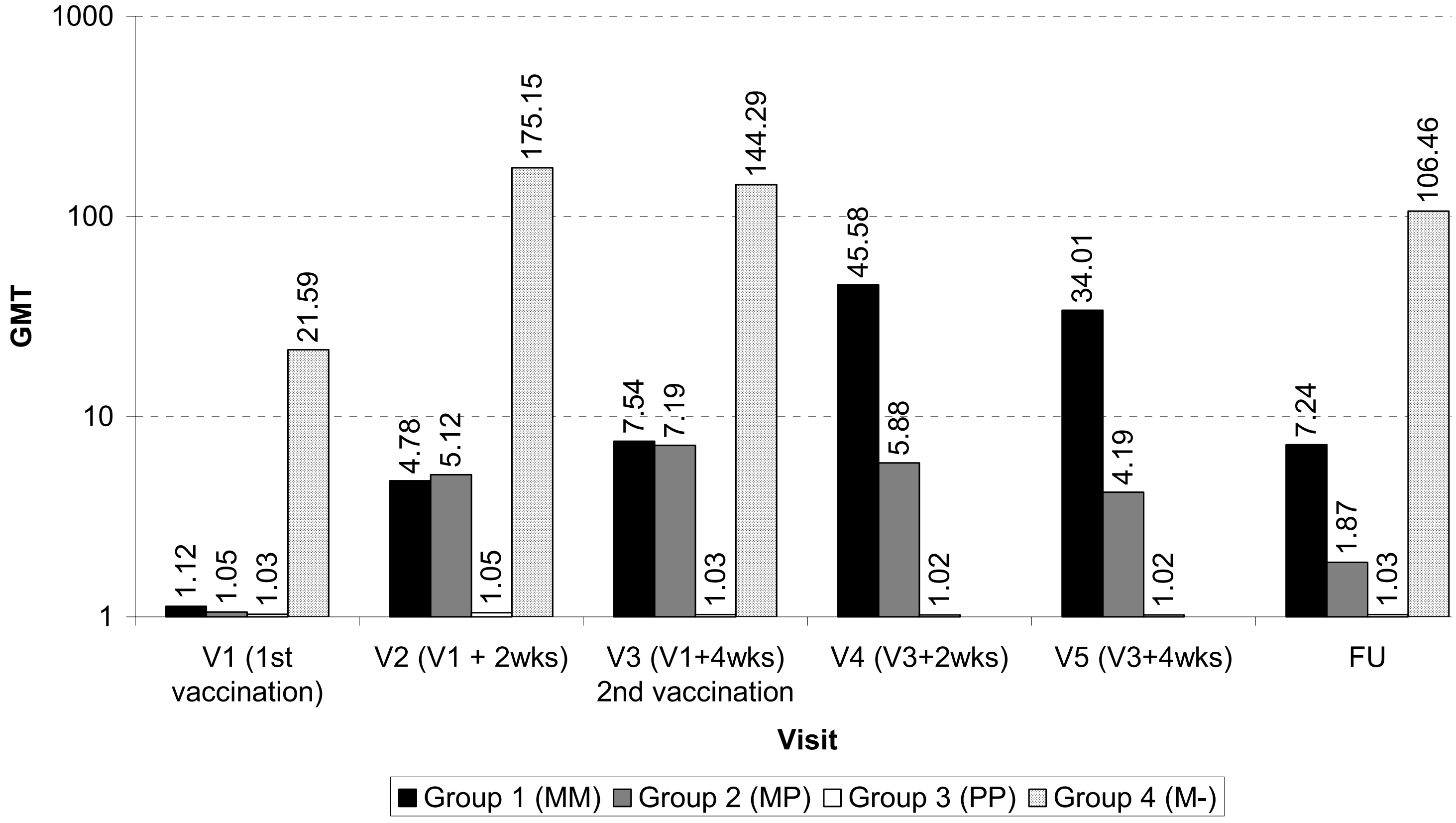

Antibodies against vaccinia, descriptive statistics for log-transformed raw data, FA population

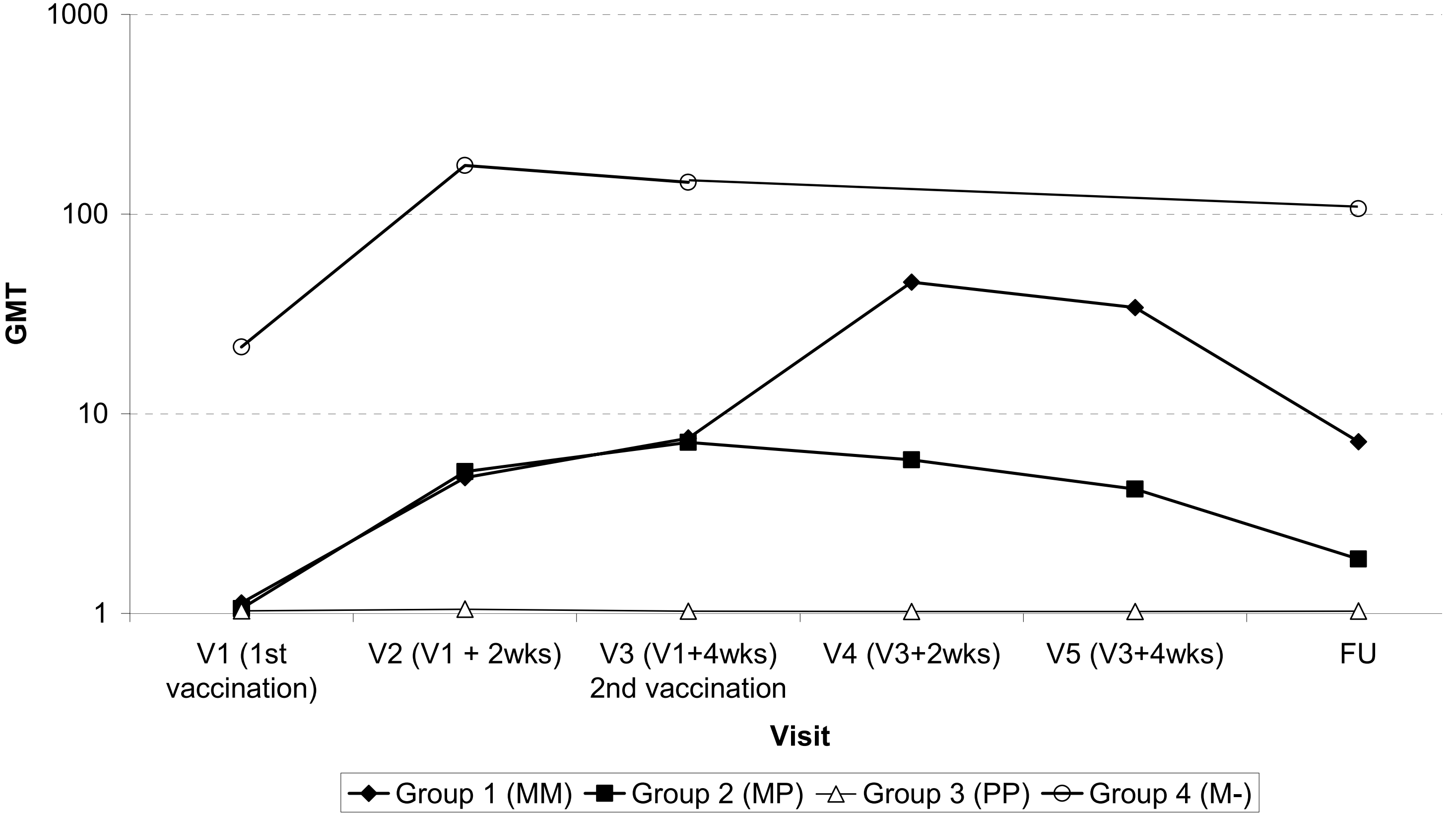

14.2.2.6 PRNT-specific antibodies against vaccinia, figure of log-transformed data, PP population

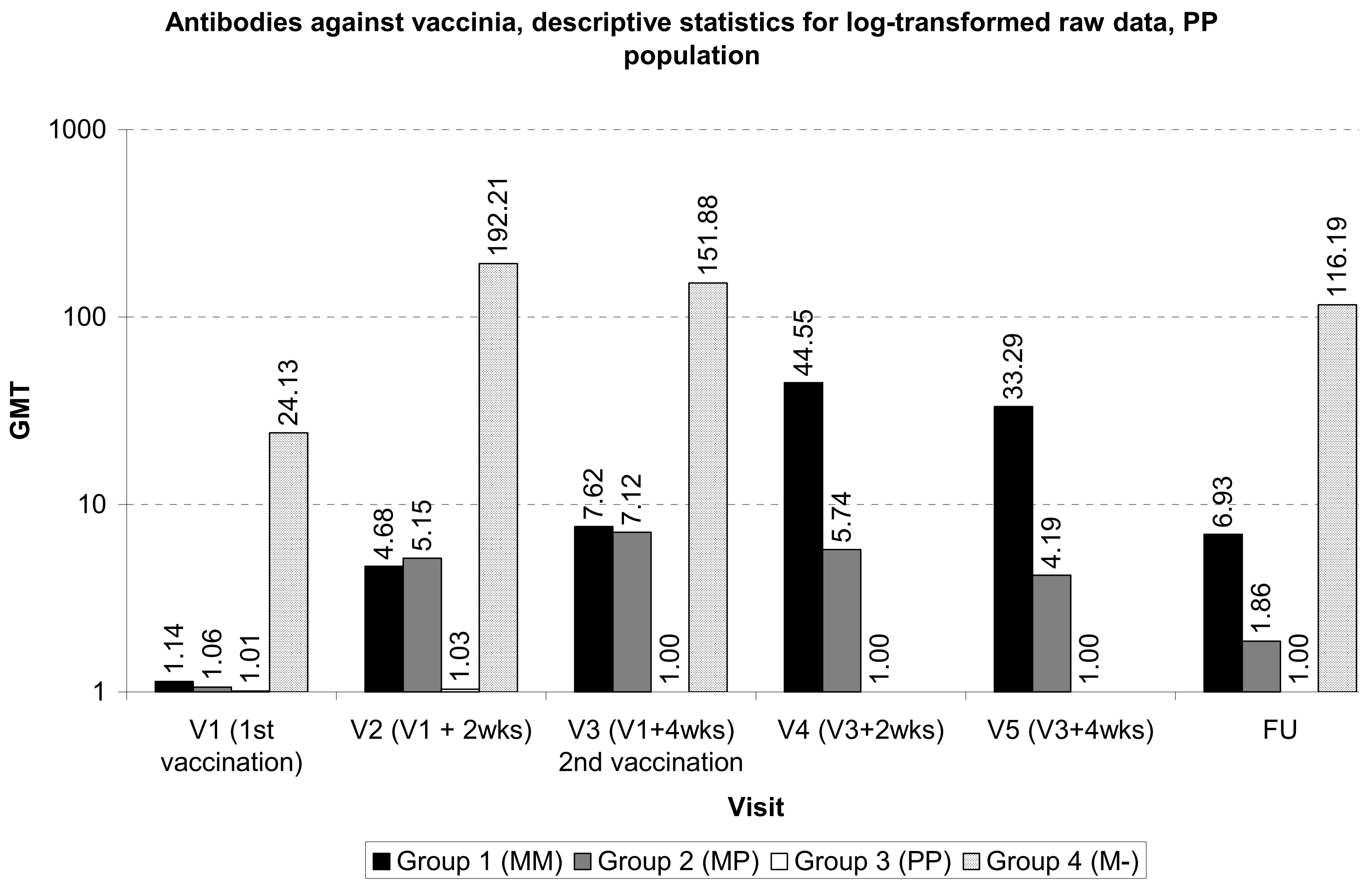

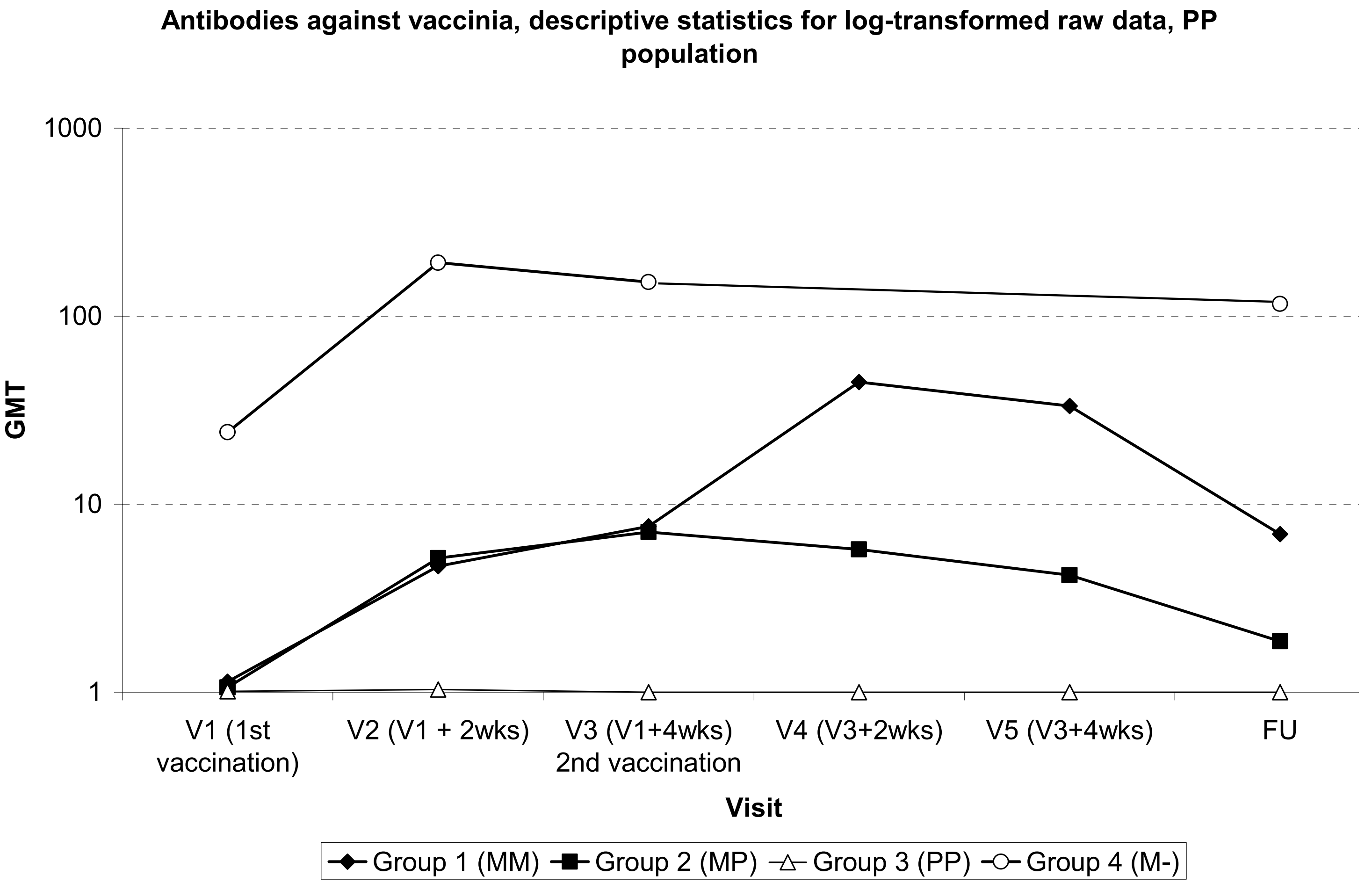
Antibodies against vaccinia, descriptive statistics for log-transformed raw data, PP population
1000
100
10
1
GMT
V1 (1st vaccination)
V2 (V1 + 2wks)
V3 (V1+4wks) 2nd vaccination
V4 (V3+2wks)
V5 (V3+4wks)
FU
Visit
Group 1 (MM)
Group 2 (MP)
Group 3 (PP)
Group 4 (M-)

14.2.2.7 PRNT-specific antibodies against vaccinia - figure of seroconversion rate, FA dataset

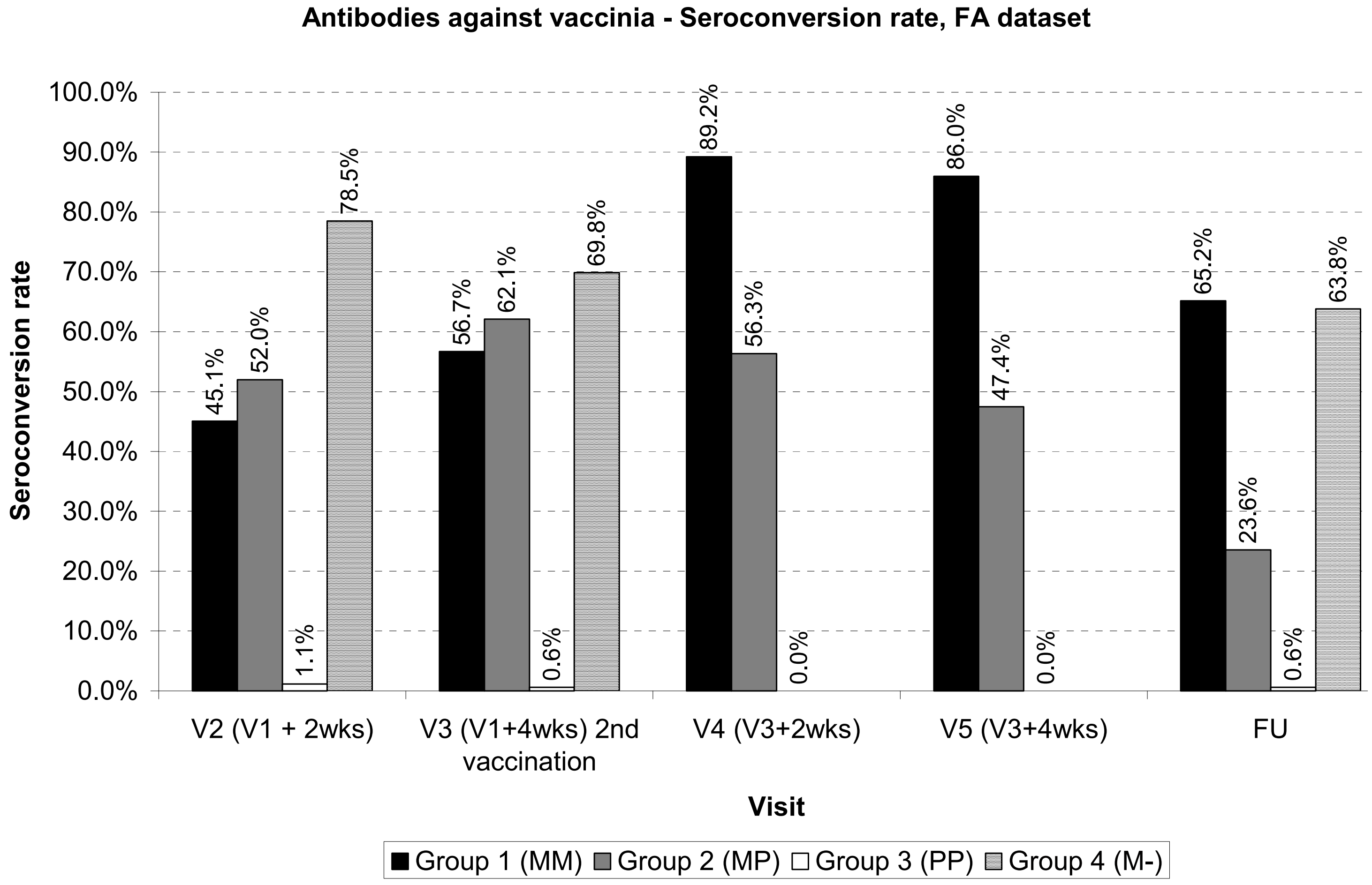

14.2.2.8 PRNT-specific antibodies against vaccinia - figure of seroconversion rate, FA dataset - Presentation by timepoint 2, 4 and 26 wks after last vaccination

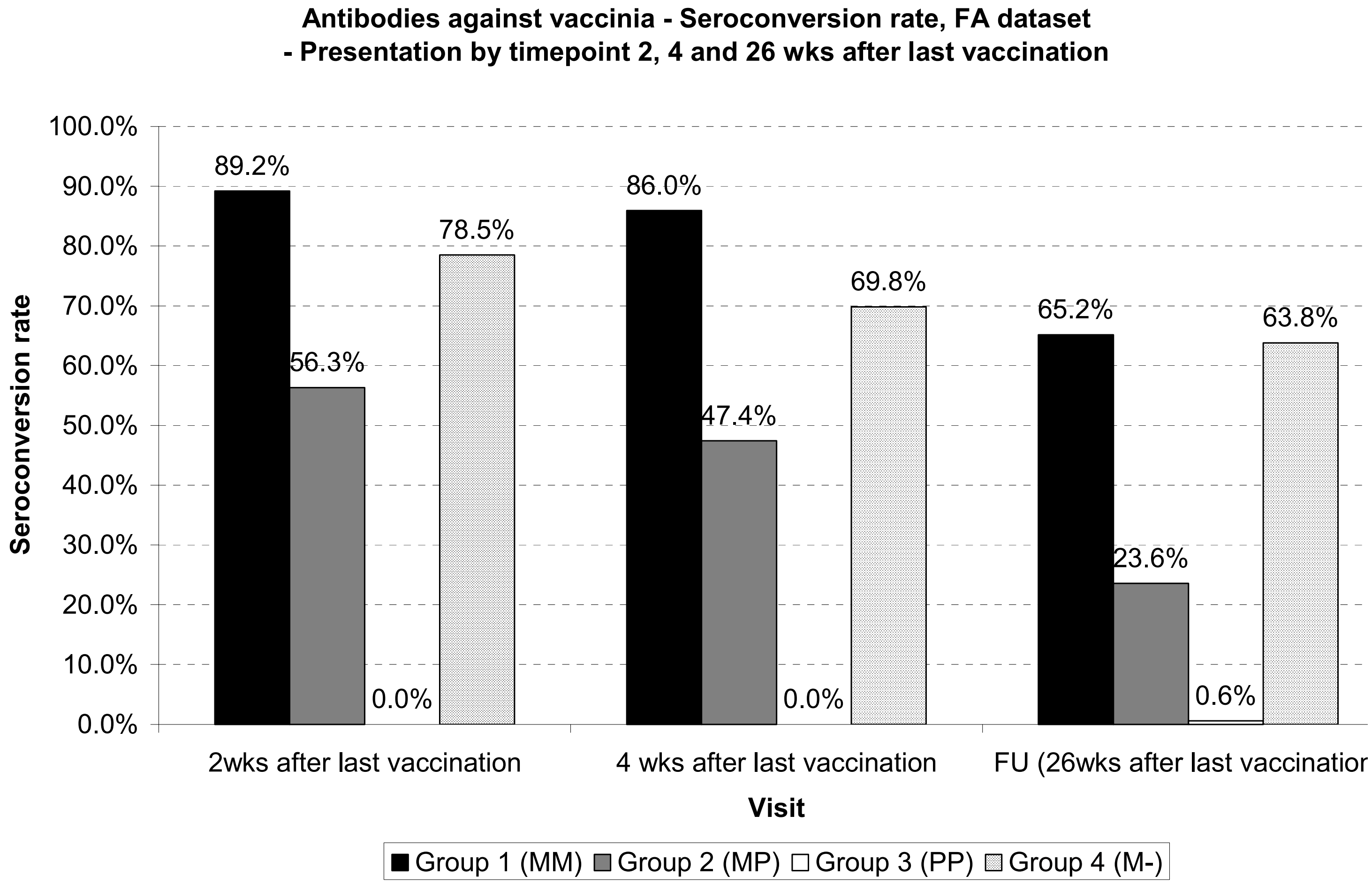

14.2.2.9 PRNT-specific antibodies against vaccinia - figure of seroconversion rate, PP dataset

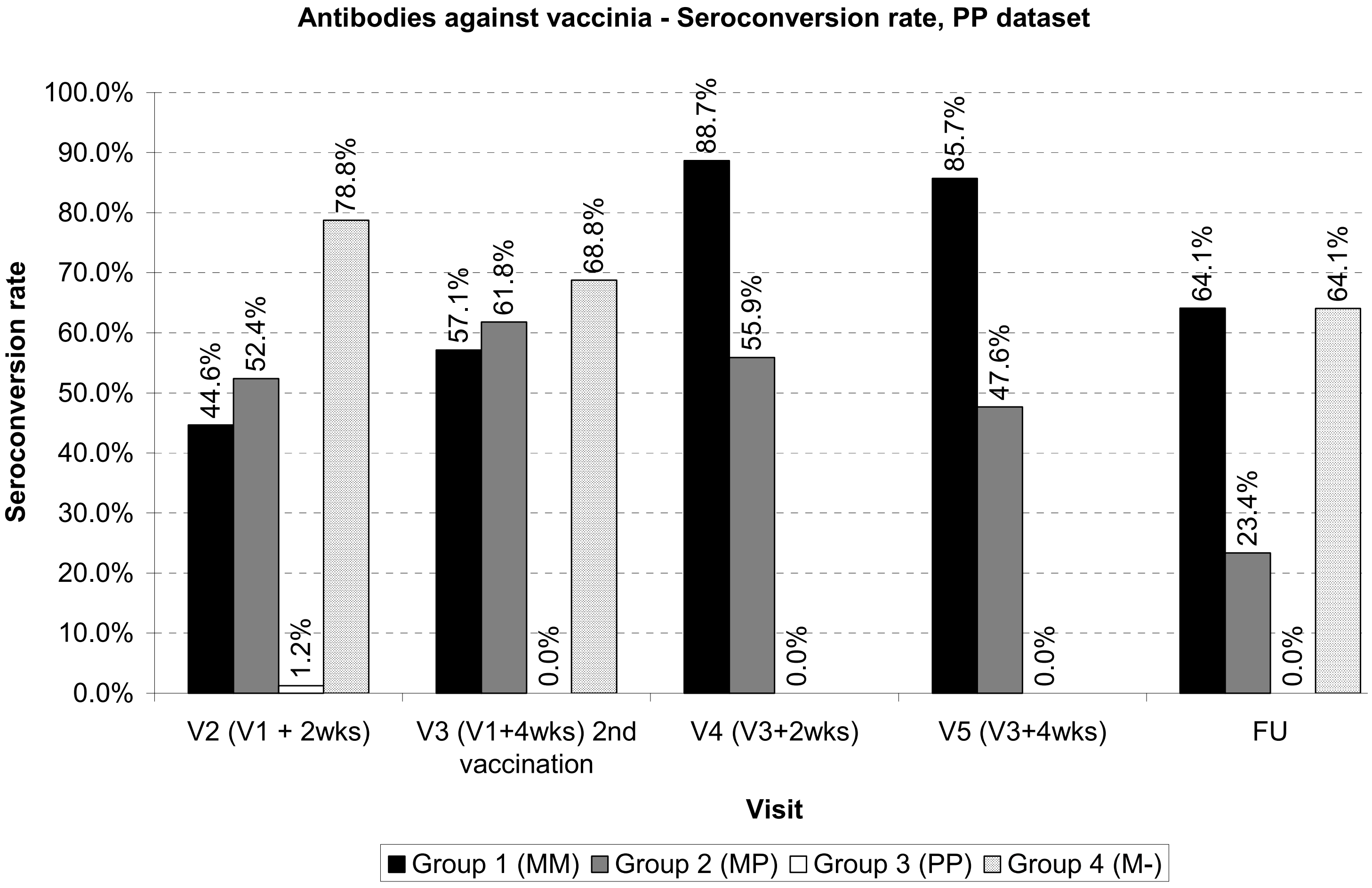

14.2.2.10 PRNT-specific antibodies against vaccinia - figure of seroconversion rate, PP dataset - Presentation by timepoint 2, 4 and 26 wks after last vaccination

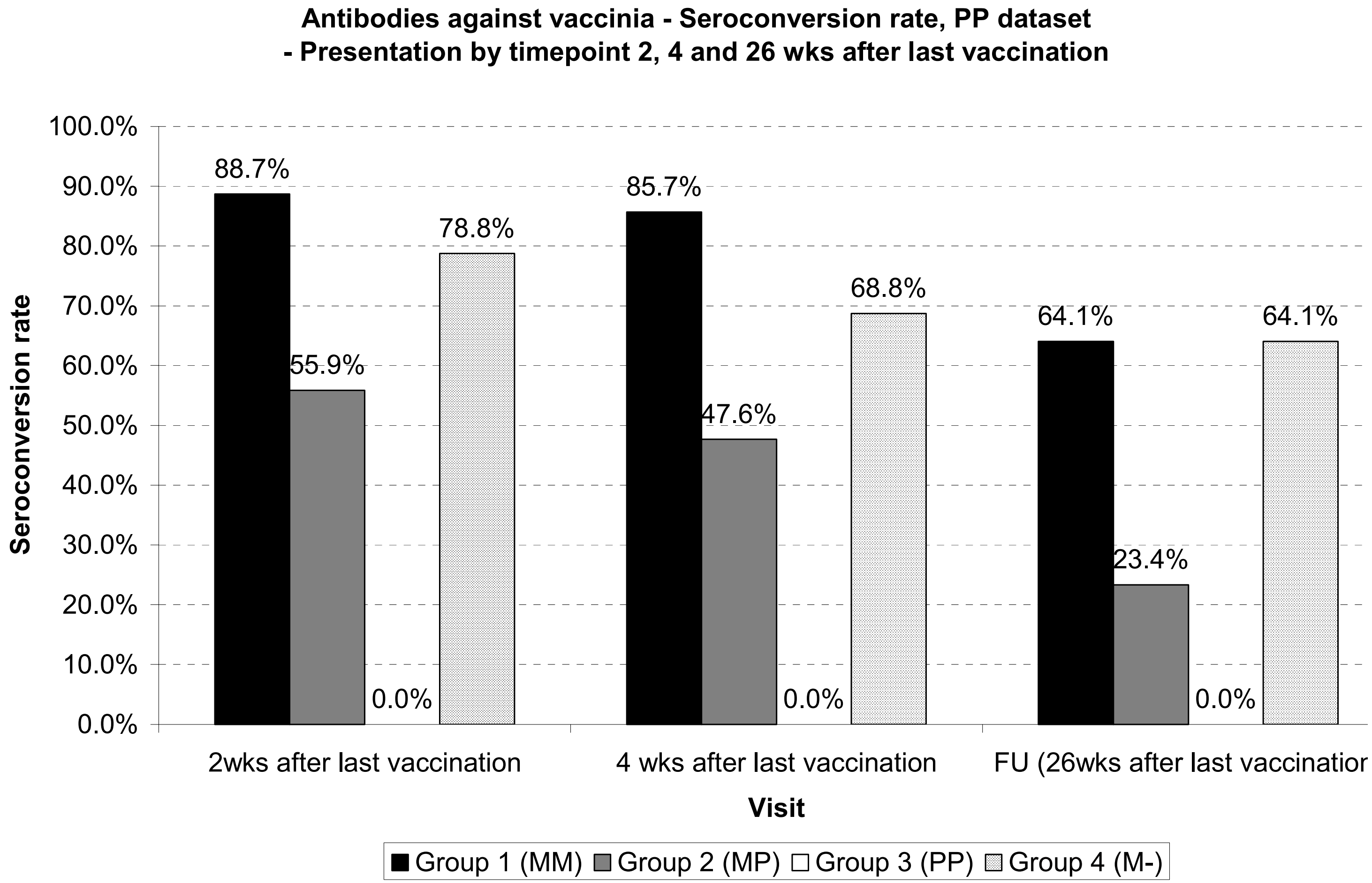

14.2.3 Correlation analysis between ELISA and PRNT antibody titer, FA dataset

14.2.3.1 Group 1 (MM)

Group=Group 1 (MM) VISIT =v1

**{tc "Corr " \f C \l 1}{tc "Group=Group 1 (MM) NAME OF FORMER VARIABLE=v1 " \f C \l 2}{tc "Variables Information " \f C \l 3}1 With Variables:**	prnt1
**1 Variables:**	elisa1

{tc "Simple Statistics " \f C \l 3}**Simple Statistics**							
**Variable**	**N**	**Mean**	**Std Dev**	**Sum**	**Minimum**	**Maximum**	**Label**
**prnt1**	183	0.05103	0.30666	9.33777	0	2.05308	PRNT antibody titer
**elisa1**	183	0.15848	0.54245	29.00163	0	3.05461	ELISA antibody titer

{tc "Pearson Correlations " \f C \l 3}**Pearson Correlation Coefficients, N = 183<br>Prob > |r| under H0: Rho=0**	
	**elisa1**
**prnt1**<br>PRNT antibody titer	0.57820<br><.0001

Correlation analysis - ELISA versus PRNT antibody titer by treatment group

Group=Group 1 (MM) Visit=v1

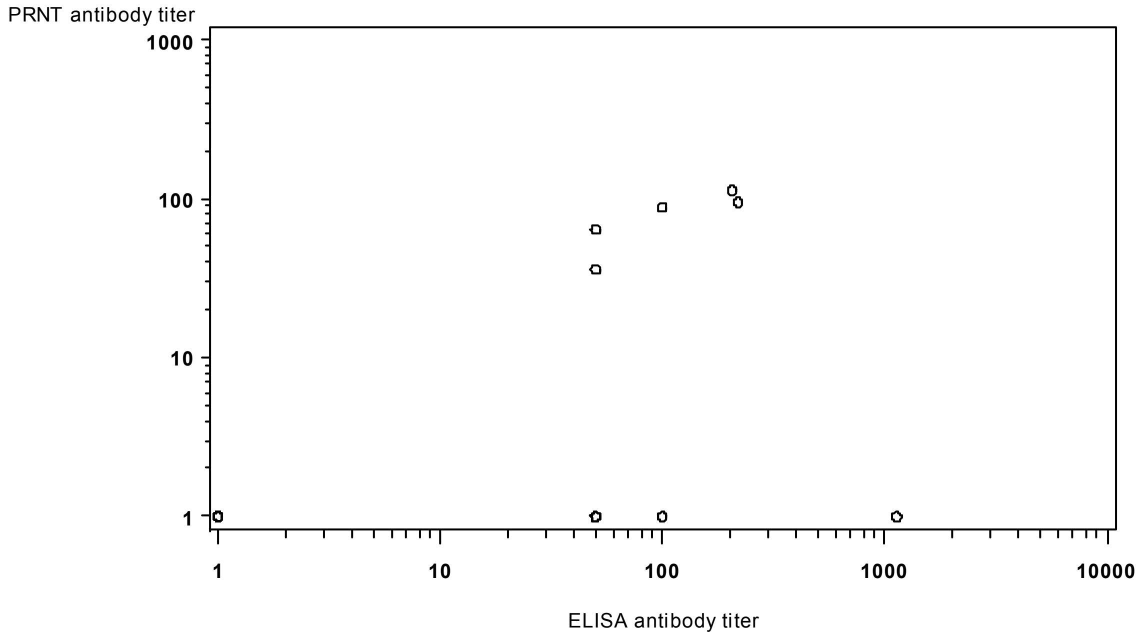

Antibody titer presented on a logarithmic scale

Group=Group 1 (MM) VISIT =v2

**{tc "Group=Group 1 (MM) NAME OF FORMER VARIABLE=v2 " \f C \l 2}{tc "Variables Information " \f C \l 3}1 With Variables:**	prnt1
**1 Variables:**	elisa1

{tc "Simple Statistics " \f C \l 3}**Simple Statistics**							
**Variable**	**N**	**Mean**	**Std Dev**	**Sum**	**Minimum**	**Maximum**	**Label**
**prnt1**	182	0.67944	0.83182	123.65871	0	3.53995	PRNT antibody titer
**elisa1**	182	1.44361	0.95830	262.73643	0	3.75136	ELISA antibody titer

{tc "Pearson Correlations " \f C \l 3}**Pearson Correlation Coefficients, N = 182 Prob > |r| under H0: Rho=0**	
	**elisa1**
**prnt1**<br>PRNT antibody titer	0.43279<br><.0001

Correlation analysis - ELISA versus PRNT antibody titer by treatment group

Group=Group 1 (MM) Visit=v2

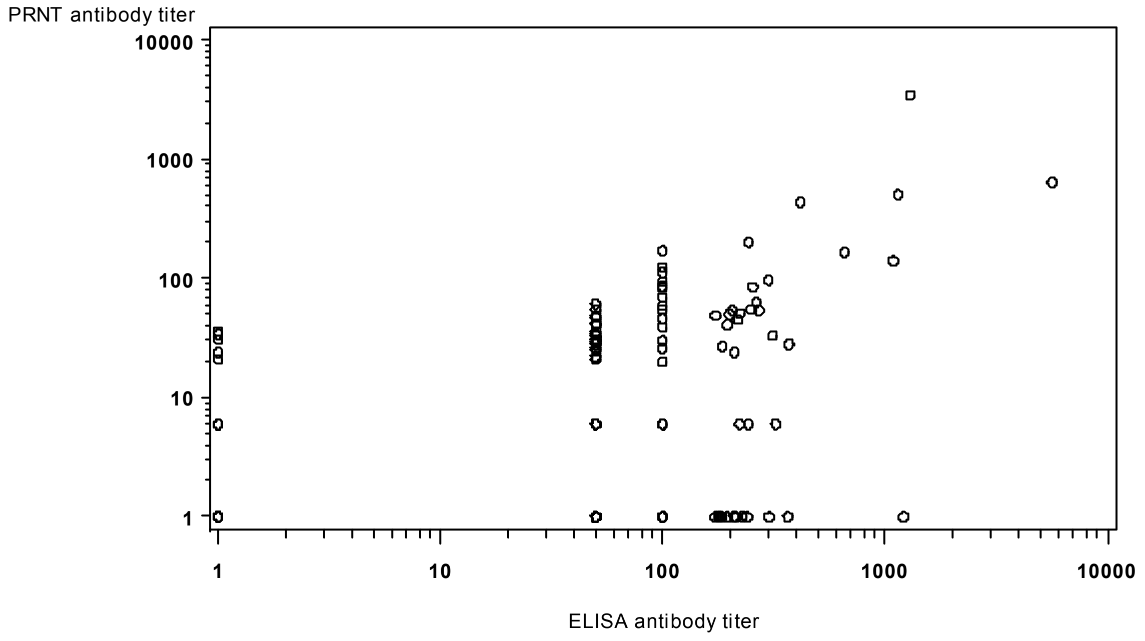

Antibody titer presented on a logarithmic scale

Group=Group 1 (MM) VISIT =v3

**{tc "Group=Group 1 (MM) NAME OF FORMER VARIABLE=v3 " \f C \l 2}{tc "Variables Information " \f C \l 3}1 With Variables:**	prnt1
**1 Variables:**	elisa1

{tc "Simple Statistics " \f C \l 3}**Simple Statistics**							
**Variable**	**N**	**Mean**	**Std Dev**	**Sum**	**Minimum**	**Maximum**	**Label**
**prnt1**	180	0.87760	0.84051	157.96754	0	3.03941	PRNT antibody titer
**elisa1**	180	1.85641	0.68723	334.15319	0	4.06528	ELISA antibody titer

{tc "Pearson Correlations " \f C \l 3}**Pearson Correlation Coefficients, N = 180<br>Prob > |r| under H0: Rho=0**	
	**elisa1**
**prnt1**<br>PRNT antibody titer	0.27155<br>0.0002

Correlation analysis - ELISA versus PRNT antibody titer by treatment group
Group=Group 1 (MM) Visit=v3

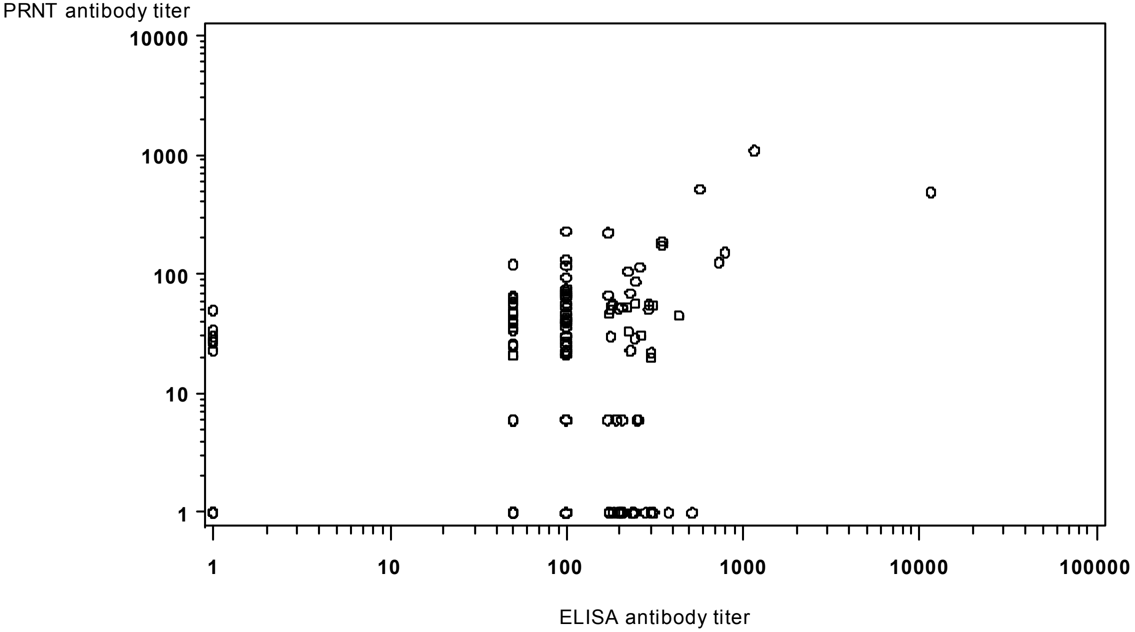

Antibody titer presented on a logarithmic scale

Group=Group 1 (MM) VISIT =v4

**{tc "Group=Group 1 (MM) NAME OF FORMER VARIABLE=v4 " \f C \l 2}{tc "Variables Information " \f C \l 3}1 With Variables:**	prnt1
**1 Variables:**	elisa1

{tc "Simple Statistics " \f C \l 3}**Simple Statistics**							
**Variable**	**N**	**Mean**	**Std Dev**	**Sum**	**Minimum**	**Maximum**	**Label**
**prnt1**	176	1.65875	0.76602	291.93982	0	3.18611	PRNT antibody titer
**elisa1**	176	2.69534	0.40384	474.38055	0	3.95909	ELISA antibody titer

{tc "Pearson Correlations " \f C \l 3}**Pearson Correlation Coefficients, N = 176 Prob > |r| under H0: Rho=0**	
	**elisa1**
**prnt1**<br>PRNT antibody titer	0.13852<br>0.0667

Correlation analysis - ELISA versus PRNT antibody titer by treatment group

Group=Group 1 (MM) Visit=v4

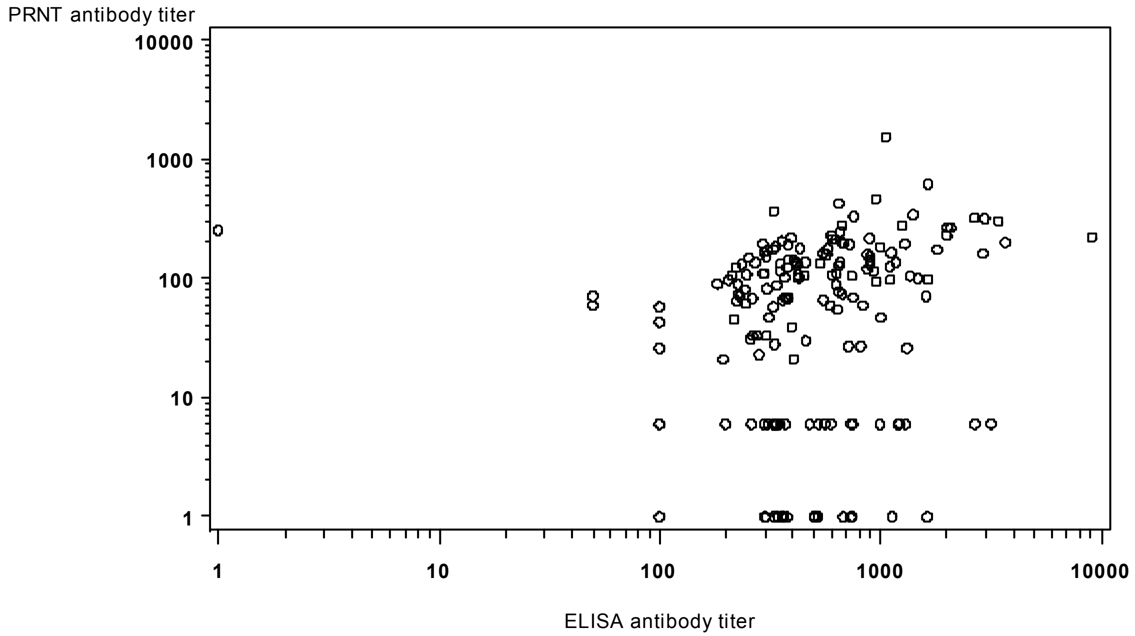

Antibody titer presented on a logarithmic scale

Group=Group 1 (MM) VISIT =v5

**{tc "Group=Group 1 (MM) NAME OF FORMER VARIABLE=v5 " \f C \l 2}{tc "Variables Information " \f C \l 3}1 With Variables:**	prnt1
**1 Variables:**	elisa1

**{**tc "Simple Statistics " \f C \l 3**}Simple Statistics**							
**Variable**	**N**	**Mean**	**Std Dev**	**Sum**	**Minimum**	**Maximum**	**Label**
**prnt1**	178	1.53157	0.74882	272.61935	0	3.18696	PRNT antibody titer
**elisa1**	178	2.51675	0.38346	447.98229	0	3.88795	ELISA antibody titer

**{**tc "Pearson Correlations " \f C \l 3**}Pearson Correlation Coefficients, N = 178 Prob > |r| under H0: Rho=0**	
	**elisa1**
**prnt1**<br>PRNT antibody titer	0.37400<br><.0001

Correlation analysis - ELISA versus PRNT antibody titer by treatment group

Group=Group 1 (MM) Visit=v5

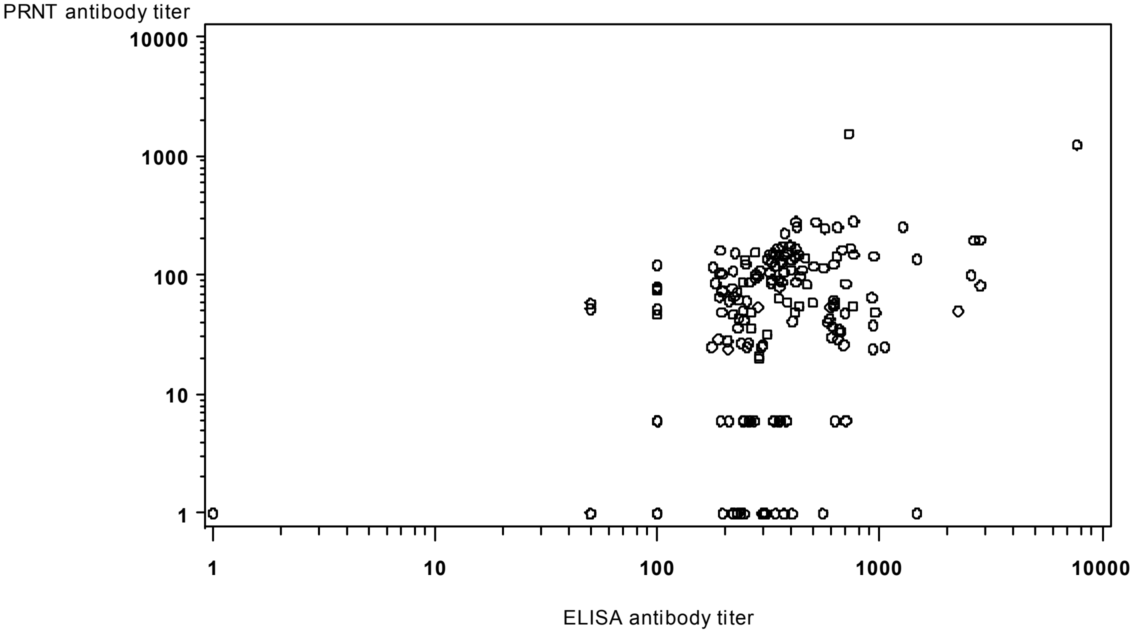

Antibody titer presented on a logarithmic scale

Group=Group 1 (MM) VISIT =FU

**{tc "Group=Group 1 (MM) NAME OF FORMER VARIABLE=v6 " \f C \l 2}{tc "Variables Information " \f C \l 3}1 With Variables:**	prnt1
**1 Variables:**	elisa1

{tc "Simple Statistics " \f C \l 3}**Simple Statistics**							
**Variable**	**N**	**Mean**	**Std Dev**	**Sum**	**Minimum**	**Maximum**	**Label**
**prnt1**	178	0.85952	0.75332	152.99425	0	2.96142	PRNT antibody titer
**elisa1**	178	1.44560	0.87673	257.31681	0	3.94527	ELISA antibody titer

{tc "Pearson Correlations " \f C \l 3}**Pearson Correlation Coefficients, N = 178**
Prob > |r| under H0: Rho=0

	**elisa1**
**prnt1**<br>PRNT antibody titer	0.46432<br><.0001

Correlation analysis - ELISA versus PRNT antibody titer by treatment group

Group=Group 1 (MM) Visit=v6

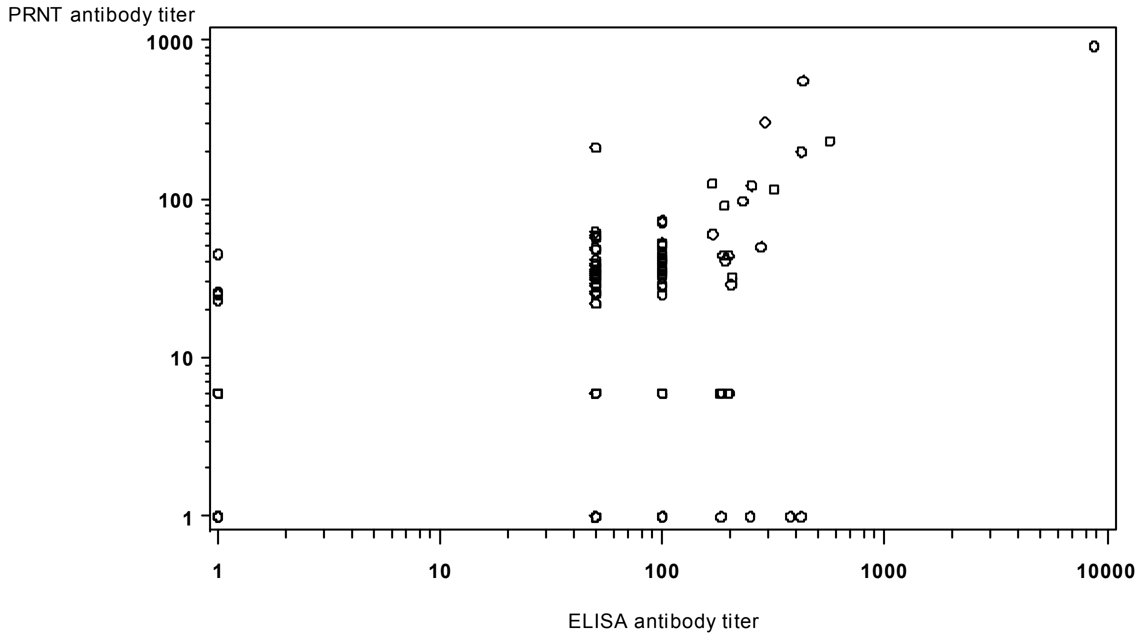

Antibody titer presented on a logarithmic scale

14.2.3.2 Group 2 (MP)

Group=Group 2 (MP) VISIT =v1

**{tc "Group=Group 2 (MP) NAME OF FORMER VARIABLE=v1 " \f C \l 2}{tc "Variables Information " \f C \l 3}1 With Variables:**	prnt1
**1 Variables:**	elisa1

**{tc "Simple Statistics " \f C \l 3}Simple Statistics**							
**Variable**	**N**	**Mean**	**Std Dev**	**Sum**	**Minimum**	**Maximum**	**Label**
**prnt1**	181	0.02322	0.17018	4.20369	0	1.86923	PRNT antibody titer
**elisa1**	181	0.10431	0.43684	18.88040	0	2.43616	ELISA antibody titer

**{tc "Pearson Correlations " \f C \l 3}Pearson Correlation Coefficients, N = 181 Prob > |r| under H0: Rho=0**	
	**elisa1**
**prnt1**<br>PRNT antibody titer	0.50514<br><.0001

Correlation analysis - ELISA versus PRNT antibody titer by treatment group

Group=Group 2 (MP) Visit=v1

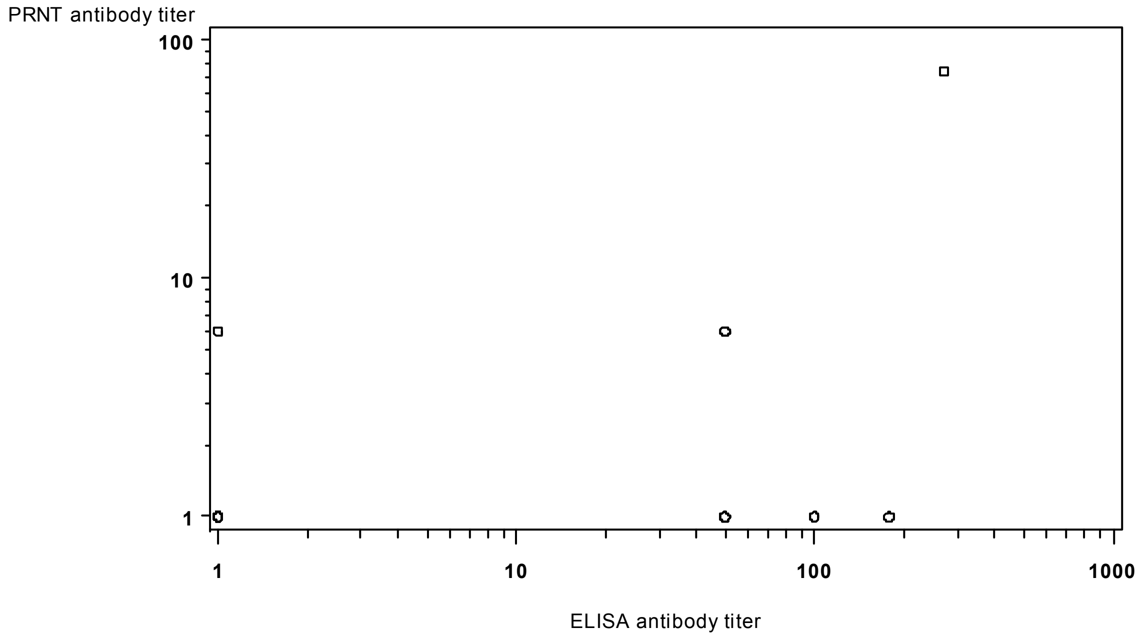

Antibody titer presented on a logarithmic scale

Group=Group 2 (MP) VISIT =v2

**{tc "Group=Group 2 (MP) NAME OF FORMER VARIABLE=v2 " \f C \l 2}{tc "Variables Information " \f C \l 3}1 With Variables:**	prnt1
**1 Variables:**	elisa1

{tc "Simple Statistics " \f C \l 3}**Simple Statistics**							
**Variable**	**N**	**Mean**	**Std Dev**	**Sum**	**Minimum**	**Maximum**	**Label**
**prnt1**	179	0.70906	0.76584	126.92210	0	2.61172	PRNT antibody titer
**elisa1**	179	1.46631	0.91054	262.46871	0	3.53301	ELISA antibody titer

{tc "Pearson Correlations " \f C \l 3}**Pearson Correlation Coefficients, N = 179 Prob > |r| under H0: Rho=0**	
	**elisa1**
**prnt1** PRNT antibody titer	0.45265 <.0001

Correlation analysis - ELISA versus PRNT antibody titer by treatment group
Group=Group 2 (MP) Visit=v2

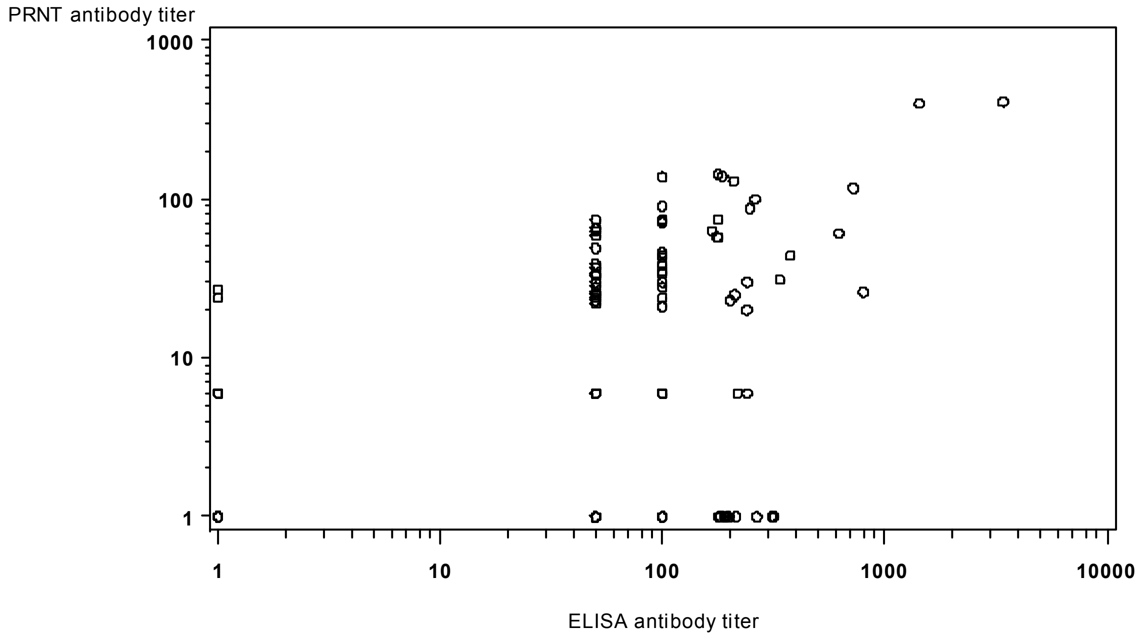

Antibody titer presented on a logarithmic scale

Group=Group 2 (MP) VISIT =v3

**{tc "Group=Group 2 (MP) NAME OF FORMER VARIABLE=v3 " \f C \l 2}{tc "Variables Information " \f C \l 3}1 With Variables:**	prnt1
**1 Variables:**	elisa1

{tc "Simple Statistics " \f C \l 3}**Simple Statistics**							
**Variable**	**N**	**Mean**	**Std Dev**	**Sum**	**Minimum**	**Maximum**	**Label**
**prnt1**	174	0.85667	0.76809	149.06054	0	2.63649	PRNT antibody titer
**elisa1**	174	1.78063	0.68855	309.82879	0	3.31408	ELISA antibody titer

{tc "Pearson Correlations " \f C \l 3}**Pearson Correlation Coefficients, N = 174 Prob > |r| under H0: Rho=0**	
	**elisa1**
**prnt1**<br>PRNT antibody titer	0.30325<br><.0001

Correlation analysis - ELISA versus PRNT antibody titer by treatment group

Group=Group 2 (MP) Visit=v3

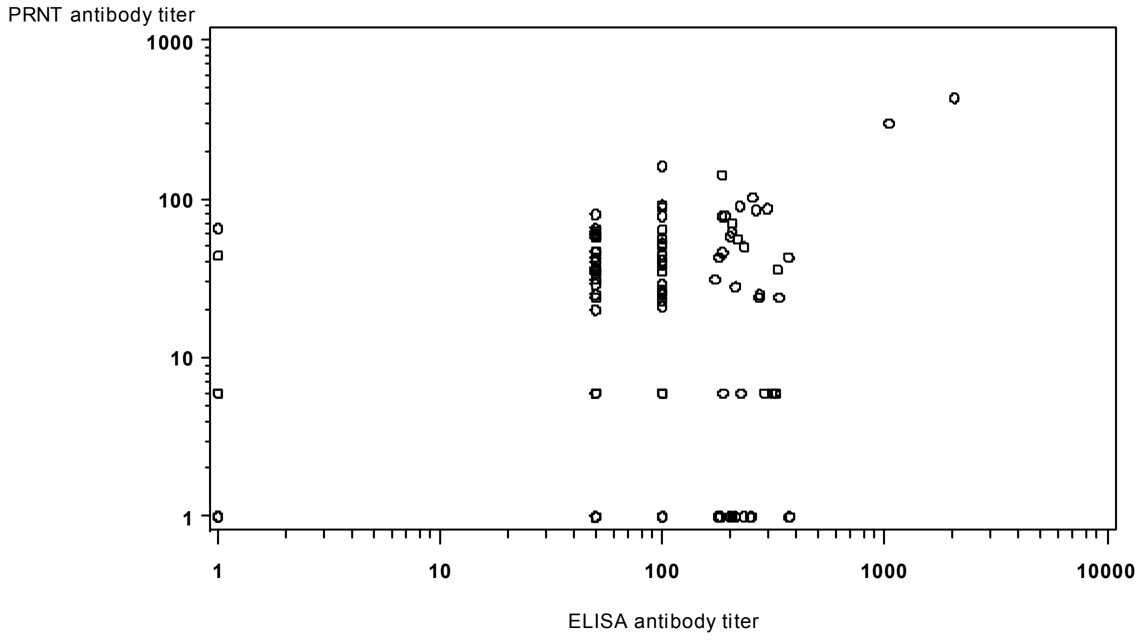

Antibody titer presented on a logarithmic scale

Group=Group 2 (MP) VISIT =v4

**{tc "Group=Group 2 (MP) NAME OF FORMER VARIABLE=v4 " \f C \l 2}{tc "Variables Information " \f C \l 3}1 With Variables:**	prnt1
**1 Variables:**	elisa1

{tc "Simple Statistics " \f C \l 3}**Simple Statistics**							
**Variable**	**N**	**Mean**	**Std Dev**	**Sum**	**Minimum**	**Maximum**	**Label**
**prnt1**	174	0.76925	0.74932	133.84957	0	2.64444	PRNT antibody titer
**elisa1**	174	1.62662	0.74845	283.03222	0	3.45515	ELISA antibody titer

{tc "Pearson Correlations " \f C \l 3}**Pearson Correlation Coefficients, N = 174 Prob > |r| under H0: Rho=0**	
	**elisa1**
**prnt1**<br>PRNT antibody titer	0.31201<br><.0001

Correlation analysis - ELISA versus PRNT antibody titer by treatment group
Group=Group 2 (MP) Visit=v4

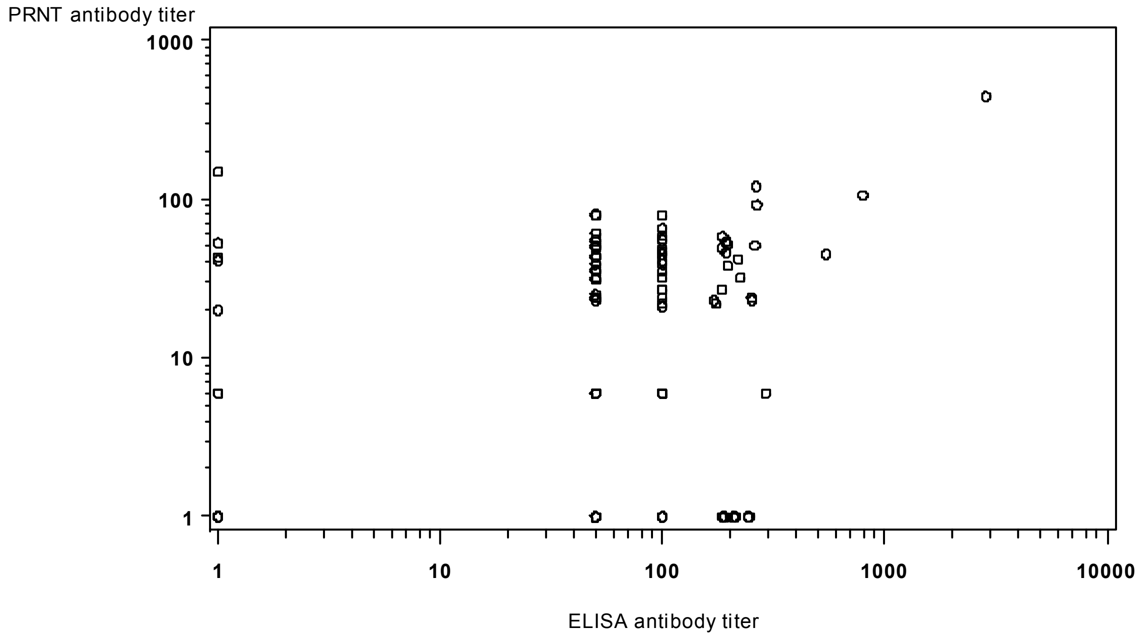

Antibody titer presented on a logarithmic scale

Group=Group 2 (MP) VISIT =v5

**{tc "Group=Group 2 (MP) NAME OF FORMER VARIABLE=v5 " \f C \l 2}{tc "Variables Information " \f C \l 3}1 With Variables:**	prnt1
**1 Variables:**	elisa1

{tc "Simple Statistics " \f C \l 3}**Simple Statistics**							
**Variable**	**N**	**Mean**	**Std Dev**	**Sum**	**Minimum**	**Maximum**	**Label**
**prnt1**	175	0.62241	0.72148	108.92206	0	2.49136	PRNT antibody titer
**elisa1**	175	1.36536	0.84350	238.93773	0	2.89597	ELISA antibody titer

{tc "Pearson Correlations " \f C \l 3}**Pearson Correlation Coefficients, N = 175 Prob > |r| under H0: Rho=0**	
	**elisa1**
**prnt1**<br>PRNT antibody titer	0.44278<br><.0001

Correlation analysis - ELISA versus PRNT antibody titer by treatment group
Group=Group 2 (MP) Visit=v5

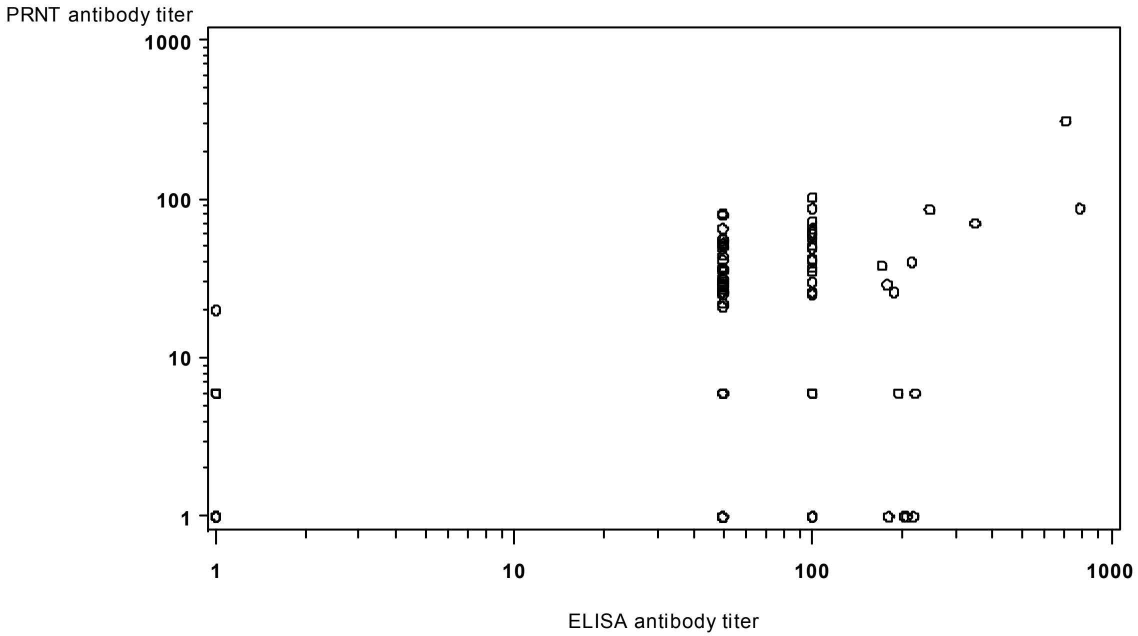

Antibody titer presented on a logarithmic scale

Group=Group 2 (MP) VISIT =FU

**{tc "Group=Group 2 (MP) NAME OF FORMER VARIABLE=v6 " \f C \l 2}{tc "Variables Information " \f C \l 3}1 With Variables:**	prnt1
**1 Variables:**	elisa1

{tc "Simple Statistics " \f C \l 3}**Simple Statistics**							
**Variable**	**N**	**Mean**	**Std Dev**	**Sum**	**Minimum**	**Maximum**	**Label**
**prnt1**	174	0.27203	0.53172	47.33295	0	2.12385	PRNT antibody titer
**elisa1**	174	0.72255	0.90598	125.72334	0	2.89818	ELISA antibody titer

{tc "Pearson Correlations " \f C \l 3}**Pearson Correlation Coefficients, N = 174**
Prob > |r| under H0: Rho=0

	**elisa1**
**prnt1**<br>PRNT antibody titer	0.46797<br><.0001

Correlation analysis - ELISA versus PRNT antibody titer by treatment group
Group=Group 2 (MP) Visit=v6

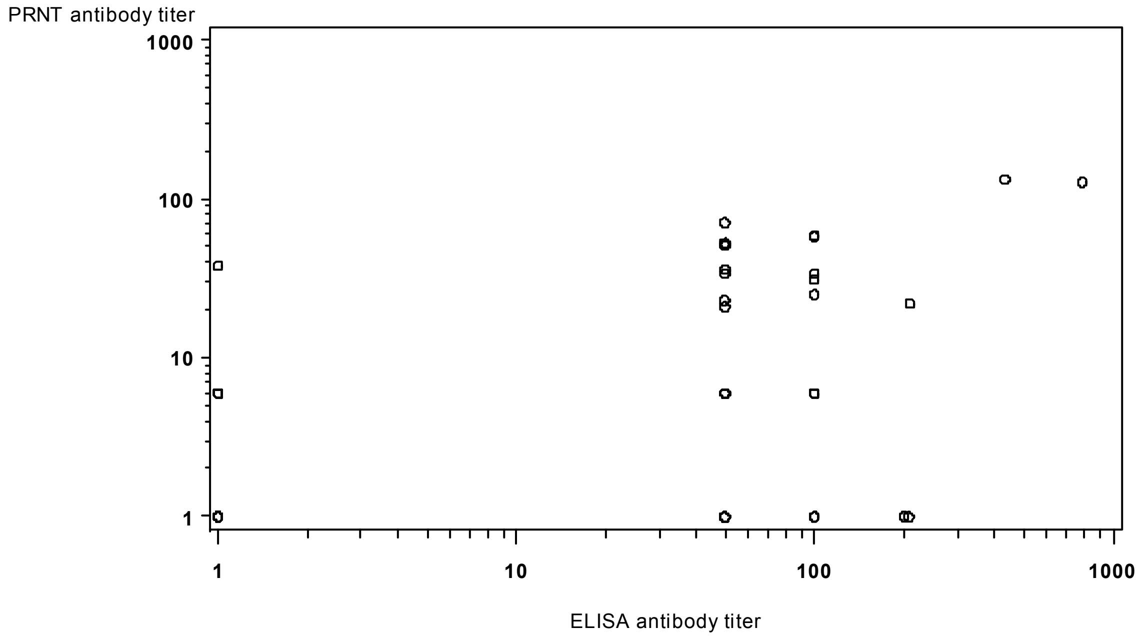

Antibody titer presented on a logarithmic scale

14.2.3.3 Group 4 (M-)

Group=Group 4 (M-) VISIT =v1

**{tc "Group=Group 4 (M-) NAME OF FORMER VARIABLE=v1 " \f C \l 2}{tc "Variables Information " \f C \l 3}1 With Variables:**	prnt1
**1 Variables:**	elisa1

{tc "Simple Statistics " \f C \l 3}**Simple Statistics**							
**Variable**	**N**	**Mean**	**Std Dev**	**Sum**	**Minimum**	**Maximum**	**Label**
**prnt1**	200	1.33418	0.86694	266.83571	0	3.97754	PRNT antibody titer
**elisa1**	200	1.58933	0.86551	317.86673	0	3.06781	ELISA antibody titer

{tc "Pearson Correlations " \f C \l 3}**Pearson Correlation Coefficients, N = 200 Prob > |r| under H0: Rho=0**	
	**elisa1**
**prnt1**<br>PRNT antibody titer	0.56938<br><.0001

Correlation analysis - ELISA versus PRNT antibody titer by treatment group

Group=Group 4 (M-) Visit=v1

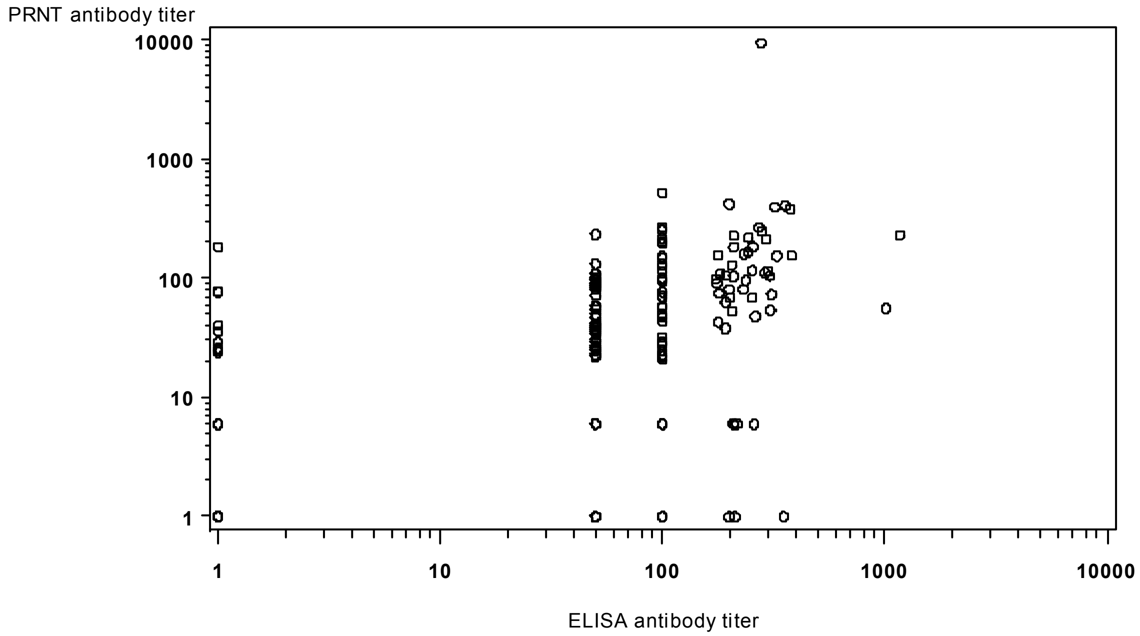

Antibody titer presented on a logarithmic scale

Group=Group 4 (M-) VISIT =v2

**{tc "Group=Group 4 (M-) NAME OF FORMER VARIABLE=v2 " \f C \l 2}{tc "Variables Information " \f C \l 3}1 With Variables:**	prnt1
**1 Variables:**	elisa1

{tc "Simple Statistics " \f C \l 3}**Simple Statistics**							
**Variable**	**N**	**Mean**	**Std Dev**	**Sum**	**Minimum**	**Maximum**	**Label**
**prnt1**	200	2.24341	0.69673	448.68201	0	3.81378	PRNT antibody titer
**elisa1**	200	2.75497	0.57246	550.99477	0	3.94817	ELISA antibody titer

{tc "Pearson Correlations " \f C \l 3}**Pearson Correlation Coefficients, N = 200 Prob > |r| under H0: Rho=0**	
	**elisa1**
**prnt1** PRNT antibody titer	0.62532 <.0001

Correlation analysis - ELISA versus PRNT antibody titer by treatment group
Group=Group 4 (M-) Visit=v2

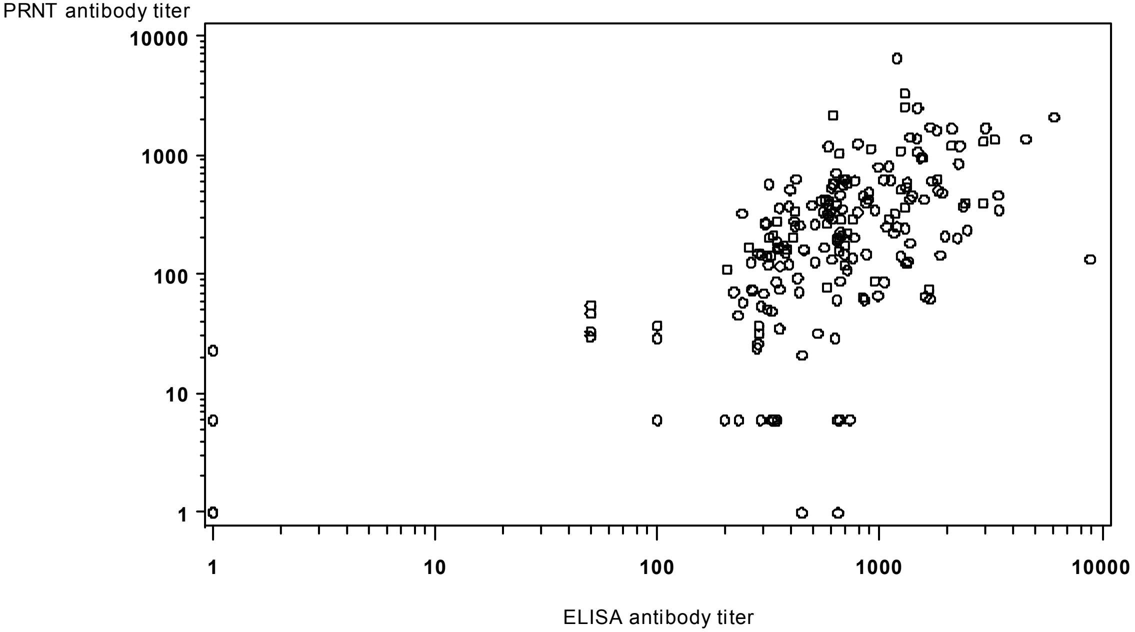

Antibody titer presented on a logarithmic scale

Group=Group 4 (M-) VISIT =v3

**{tc "Group=Group 4 (M-) NAME OF FORMER VARIABLE=v3 " \f C \l 2}{tc "Variables Information " \f C \l 3}1 With Variables:**	prnt1
**1 Variables:**	elisa1

**{**tc "Simple Statistics " \f C \l 3**}Simple Statistics**							
**Variable**	**N**	**Mean**	**Std Dev**	**Sum**	**Minimum**	**Maximum**	**Label**
**prnt1**	199	2.15922	0.62653	429.68509	0	3.75289	PRNT antibody titer
**elisa1**	199	2.65545	0.46933	528.43399	0	3.84479	ELISA antibody titer

**{**tc "Pearson Correlations " \f C \l 3**}Pearson Correlation Coefficients, N = 199 Prob > |r| under H0: Rho=0**	
	**elisa1**
**prnt1**<br>PRNT antibody titer	0.47583<br><.0001

Correlation analysis - ELISA versus PRNT antibody titer by treatment group

Group=Group 4 (M-) Visit=v3

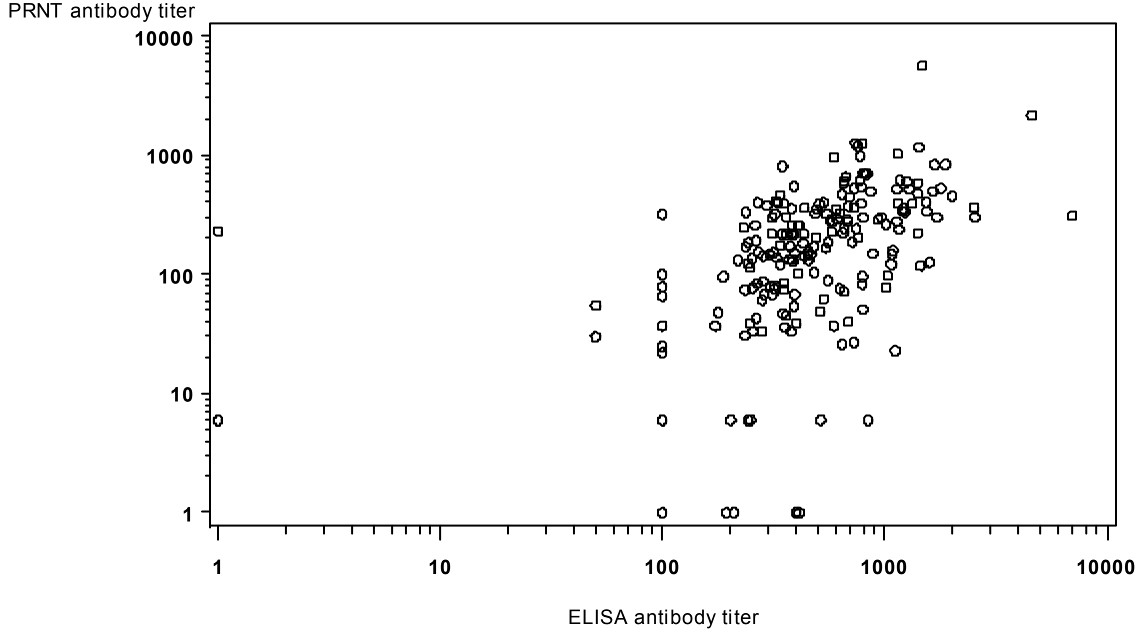

Antibody titer presented on a logarithmic scale

Group=Group 4 (M-) VISIT =FU

**{tc "Group=Group 4 (M-) NAME OF FORMER VARIABLE=v6 " \f C \l 2}{tc "Variables Information " \f C \l 3}1 With Variables:**	prnt1
**1 Variables:**	elisa1

**{**tc "Simple Statistics " \f C \l 3**}Simple Statistics**							
**Variable**	**N**	**Mean**	**Std Dev**	**Sum**	**Minimum**	**Maximum**	**Label**
**prnt1**	198	2.02720	0.55117	401.38553	0	3.80284	PRNT antibody titer
**elisa1**	199	2.25505	0.58865	448.75475	0	3.89746	ELISA antibody titer

{tc "Pearson Correlations " \f C \l 3**}Pearson Correlation Coefficients**
Prob > |r| under H0: Rho=0
Number of Observations

	**elisa1**
**prnt1**<br>PRNT antibody titer	0.75181<br><.0001<br>198

Correlation analysis - ELISA versus PRNT antibody titer by treatment group

Group=Group 4 (M-) Visit=v6

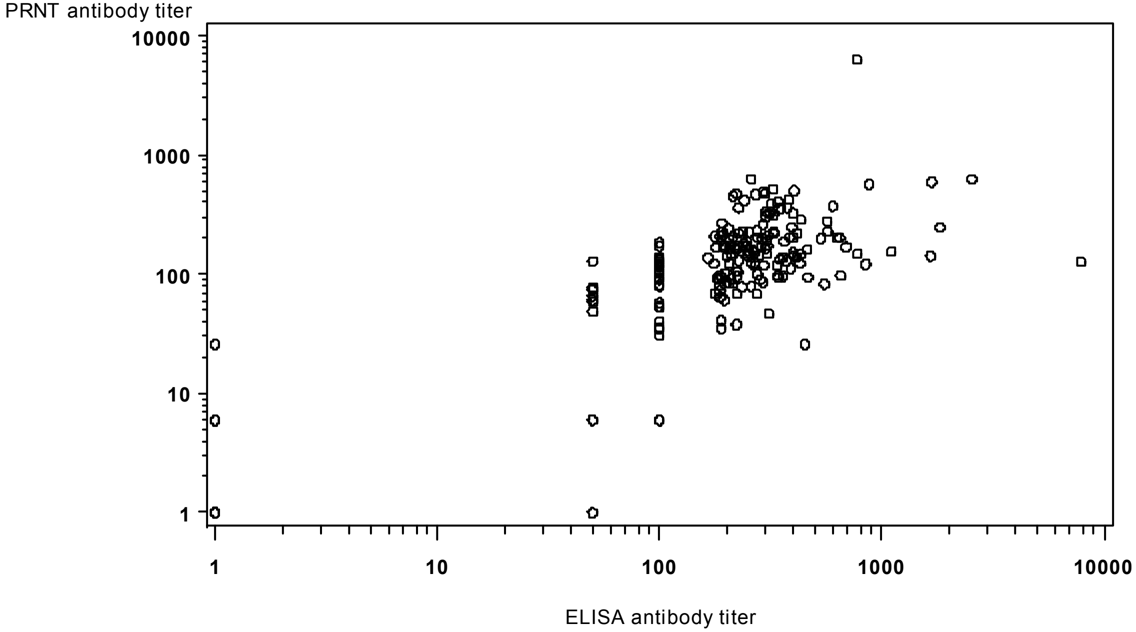

Antibody titer presented on a logarithmic scale

14.3 LABORATORY DATA

14.3.1 Summary of laboratory data - Clinical Chemistry, descriptive statistics and count of abnormalities, Safety dataset

Alkaline phosphatase (U/l)

		n	Mean	Median	Std	StdErr	Min	Max	Missing n	Missing %	Below LLN n	Below LLN %	Normal n	Normal %	Above ULN n	Above ULN %
Group 1 (MM) (N=183)																
	Screening	183	61.86	61.00	16.97	1.25	21.00	118.00	-	0.0	-	0.0	183	100.0	-	0.0
	Visit 2	180	62.32	61.00	16.10	1.20	22.00	104.00	-	0.0	-	0.0	180	100.0	-	0.0
	Visit 3	3	42.67	56.00	23.09	13.33	16.00	56.00	-	0.0	-	0.0	3	100.0	-	0.0
	Visit 4	175	61.90	62.00	16.39	1.24	22.00	127.00	1	0.6	-	0.0	174	98.9	1	0.6
	Visit 5	22	61.18	60.50	14.86	3.17	35.00	93.00	1	4.3	-	0.0	22	95.7	-	0.0
	Follow-Up	11	74.18	66.00	26.74	8.06	40.00	136.00	3	21.4	-	0.0	10	71.4	1	7.1
Group 2 (MP) (N=181)																
	Screening	180	58.83	56.00	15.26	1.14	29.00	107.00	1	0.6	-	0.0	180	99.4	-	0.0
	Visit 2	179	59.36	58.00	16.03	1.20	26.00	123.00	-	0.0	-	0.0	179	100.0	-	0.0
	Visit 3	0	-	-	-	-	-	-	-	-	-	-	-	-	-	-
	Visit 4	174	59.06	57.50	15.99	1.21	25.00	121.00	-	0.0	-	0.0	174	100.0	-	0.0
	Visit 5	19	55.26	50.00	13.84	3.18	39.00	87.00	1	5.0	-	0.0	19	95.0	-	0.0
	Follow-Up	6	66.33	64.00	12.27	5.01	53.00	86.00	2	25.0	-	0.0	6	75.0	-	0.0
Group 3 (PP) (N=181)																
	Screening	181	60.19	58.00	15.88	1.18	33.00	109.00	-	0.0	-	0.0	181	100.0	-	0.0
	Visit 2	176	59.59	57.50	16.70	1.26	19.00	113.00	2	1.1	-	0.0	176	98.9	-	0.0
	Visit 3	2	73.50	73.50	37.48	26.50	47.00	100.00	-	0.0	-	0.0	2	100.0	-	0.0
	Visit 4	175	60.74	58.00	17.13	1.29	25.00	113.00	-	0.0	-	0.0	175	100.0	-	0.0
	Visit 5	17	56.06	53.00	18.59	4.51	28.00	88.00	1	5.6	-	0.0	17	94.4	-	0.0
	Follow-Up	5	65.20	64.00	14.60	6.53	45.00	85.00	7	58.3	-	0.0	5	41.7	-	0.0

(Continued)

N: Number of subjects in the specified group
n: Number of subjects in the specified group (=Number of subjects with data available)
%: Percentage based on n
LLN: Lower limit of normal range
ULN: Upper limit of normal range
Cholesterol (Total, HDL, LDL) and beta-HCG only performed at Screening

Summary of laboratory data - Clinical Chemistry, descriptive statistics and count of abnormalities, Safety dataset

Alkaline phosphatase (U/l)

								Missing		Below LLN		Normal		Above ULN	
	n	Mean	Median	Std	StdErr	Min	Max	n	%	n	%	n	%	n	%
Group 4 (M-) (N=200)															
Screening	199	59.84	57.00	18.66	1.32	23.00	132.00	1	0.5	-	0.0	196	98.0	3	1.5
Visit 2	200	59.70	56.00	17.88	1.26	29.00	166.00	-	0.0	-	0.0	199	99.5	1	0.5
Visit 3	19	59.00	57.00	15.48	3.55	34.00	96.00	-	0.0	-	0.0	19	100.0	-	0.0
Visit 4	0	-	-	-	-	-	-	-	-	-	-	-	-	-	-
Visit 5	0	-	-	-	-	-	-	-	-	-	-	-	-	-	-
Follow-Up	7	50.00	48.00	7.16	2.71	41.00	60.00	-	0.0	-	0.0	7	100.0	-	0.0

N: Number of subjects in the specified group
n: Number of subjects in the specified group (=Number of subjects with data available)
%: Percentage based on n
LLN: Lower limit of normal range
ULN: Upper limit of normal range
Cholesterol (Total, HDL, LDL) and beta-HCG only performed at Screening

Summary of laboratory data - Clinical Chemistry, descriptive statistics and count of abnormalities, Safety dataset

Beta-HCG (IU/l)

									Missing		Below LLN		Normal		Above ULN	
		n	Mean	Median	Std	StdErr	Min	Max	n	%	n	%	n	%	n	%
Group 1 (MM) (N=183)																
	Screening	97	0.10	0.10	0.02	0.00	0.10	0.30	-	0.0	-	0.0	97	100.0	-	0.0
	Visit 2	0	-	-	-	-	-	-	-	-	-	-	-	-	-	-
	Visit 3	0	-	-	-	-	-	-	-	-	-	-	-	-	-	-
	Visit 4	0	-	-	-	-	-	-	-	-	-	-	-	-	-	-
	Visit 5	0	-	-	-	-	-	-	-	-	-	-	-	-	-	-
	Follow-Up	0	-	-	-	-	-	-	-	-	-	-	-	-	-	-
Group 2 (MP) (N=181)																
	Screening	111	0.11	0.10	0.05	0.00	0.10	0.50	1	0.9	-	0.0	111	99.1	-	0.0
	Visit 2	0	-	-	-	-	-	-	-	-	-	-	-	-	-	-
	Visit 3	0	-	-	-	-	-	-	-	-	-	-	-	-	-	-
	Visit 4	0	-	-	-	-	-	-	-	-	-	-	-	-	-	-
	Visit 5	0	-	-	-	-	-	-	-	-	-	-	-	-	-	-
	Follow-Up	0	-	-	-	-	-	-	-	-	-	-	-	-	-	-
Group 3 (PP) (N=181)																
	Screening	106	0.10	0.10	0.00	0.00	0.10	0.10	-	0.0	-	0.0	106	100.0	-	0.0
	Visit 2	0	-	-	-	-	-	-	-	-	-	-	-	-	-	-
	Visit 3	0	-	-	-	-	-	-	-	-	-	-	-	-	-	-
	Visit 4	0	-	-	-	-	-	-	-	-	-	-	-	-	-	-
	Visit 5	0	-	-	-	-	-	-	-	-	-	-	-	-	-	-
	Follow-Up	0	-	-	-	-	-	-	-	-	-	-	-	-	-	-

(Continued)

N: Number of subjects in the specified group
n: Number of subjects in the specified group (=Number of subjects with data available)
%: Percentage based on n
LLN: Lower limit of normal range
ULN: Upper limit of normal range
Cholesterol (Total, HDL, LDL) and beta-HCG only performed at Screening

Summary of laboratory data - Clinical Chemistry, descriptive statistics and count of abnormalities, Safety dataset

Beta-HCG (IU/l)

								Missing		Below LLN		Normal		Above ULN	
	n	Mean	Median	Std	StdErr	Min	Max	n	%	n	%	n	%	n	%
Group 4 (M-) (N=200)															
Screening	112	0.35	0.10	0.73	0.07	0.10	3.50	1	0.9	-	0.0	112	99.1	-	0.0
Visit 2	0	-	-	-	-	-	-	-	-	-	-	-	-	-	-
Visit 3	0	-	-	-	-	-	-	-	-	-	-	-	-	-	-
Visit 4	0	-	-	-	-	-	-	-	-	-	-	-	-	-	-
Visit 5	0	-	-	-	-	-	-	-	-	-	-	-	-	-	-
Follow-Up	0	-	-	-	-	-	-	-	-	-	-	-	-	-	-

N: Number of subjects in the specified group
n: Number of subjects in the specified group (=Number of subjects with data available)
%: Percentage based on n
LLN: Lower limit of normal range
ULN: Upper limit of normal range
Cholesterol (Total, HDL, LDL) and beta-HCG only performed at Screening

Summary of laboratory data - Clinical Chemistry, descriptive statistics and count of abnormalities, Safety dataset

Bilirubin, total (μmol/l)

		n	Mean	Median	Std	StdErr	Min	Max	Missing n	Missing %	Below LLN n	Below LLN %	Normal n	Normal %	Above ULN n	Above ULN %
Group 1 (MM) (N=183)																
	Screening	183	12.56	10.90	8.26	0.61	3.10	64.80	-	0.0	-	0.0	164	89.6	19	10.4
	Visit 2	180	11.06	9.10	6.90	0.51	3.40	56.80	-	0.0	-	0.0	167	92.8	13	7.2
	Visit 3	3	7.70	8.50	4.26	2.46	3.10	11.50	-	0.0	-	0.0	3	100.0	-	0.0
	Visit 4	175	11.37	9.40	6.89	0.52	2.70	42.40	1	0.6	-	0.0	158	89.8	17	9.7
	Visit 5	23	10.46	8.90	9.22	1.92	3.20	47.40	-	0.0	-	0.0	21	91.3	2	8.7
	Follow-Up	11	13.76	12.30	10.71	3.23	3.60	43.30	3	21.4	-	0.0	10	71.4	1	7.1
Group 2 (MP) (N=181)																
	Screening	180	12.60	10.00	8.05	0.60	2.60	52.80	1	0.6	-	0.0	156	86.2	24	13.3
	Visit 2	179	11.57	9.10	8.72	0.65	2.20	59.30	-	0.0	-	0.0	163	91.1	16	8.9
	Visit 3	0	-	-	-	-	-	-	-	-	-	-	-	-	-	-
	Visit 4	174	11.20	9.00	7.90	0.60	2.60	58.10	-	0.0	-	0.0	154	88.5	20	11.5
	Visit 5	19	10.92	9.40	6.29	1.44	5.10	33.00	1	5.0	-	0.0	18	90.0	1	5.0
	Follow-Up	6	9.82	10.10	2.88	1.18	5.80	13.00	2	25.0	-	0.0	6	75.0	-	0.0
Group 3 (PP) (N=181)																
	Screening	181	12.51	10.30	7.21	0.54	3.20	46.20	-	0.0	-	0.0	157	86.7	24	13.3
	Visit 2	176	10.86	8.80	7.51	0.57	3.10	41.70	2	1.1	-	0.0	156	87.6	20	11.2
	Visit 3	2	8.05	8.05	2.62	1.85	6.20	9.90	-	0.0	-	0.0	2	100.0	-	0.0
	Visit 4	175	11.08	8.40	7.75	0.59	2.90	55.10	-	0.0	-	0.0	154	88.0	21	12.0
	Visit 5	17	12.39	10.40	8.89	2.16	2.70	36.60	1	5.6	-	0.0	15	83.3	2	11.1
	Follow-Up	5	9.82	9.90	5.77	2.58	3.80	19.00	7	58.3	-	0.0	5	41.7	-	0.0

(Continued)

N: Number of subjects in the specified group
n: Number of subjects in the specified group (=Number of subjects with data available)
%: Percentage based on n
LLN: Lower limit of normal range
ULN: Upper limit of normal range
Cholesterol (Total, HDL, LDL) and beta-HCG only performed at Screening

Summary of laboratory data - Clinical Chemistry, descriptive statistics and count of abnormalities, Safety dataset

Bilirubin, total (µmol/l)

								Missing		Below LLN		Normal		Above ULN	
	n	Mean	Median	Std	StdErr	Min	Max	n	%	n	%	n	%	n	%
Group 4 (M-) (N=200)															
Screening	199	12.03	9.90	7.49	0.53	3.60	66.50	1	0.5	-	0.0	178	89.0	21	10.5
Visit 2	200	10.60	8.60	8.42	0.60	2.40	76.30	-	0.0	-	0.0	181	90.5	19	9.5
Visit 3	19	8.03	6.70	3.24	0.74	4.40	17.60	-	0.0	-	0.0	19	100.0	-	0.0
Visit 4	0	-	-	-	-	-	-	-	-	-	-	-	-	-	-
Visit 5	0	-	-	-	-	-	-	-	-	-	-	-	-	-	-
Follow-Up	7	7.87	8.50	2.63	0.99	5.30	11.30	-	0.0	-	0.0	7	100.0	-	0.0

N: Number of subjects in the specified group
n: Number of subjects in the specified group (=Number of subjects with data available)
%: Percentage based on n
LLN: Lower limit of normal range
ULN: Upper limit of normal range
Cholesterol (Total, HDL, LDL) and beta-HCG only performed at Screening

Summary of laboratory data - Clinical Chemistry, descriptive statistics and count of abnormalities, Safety dataset

Calcium, total (mmol/l)

								Missing		Below LLN		Normal		Above ULN	
		n	Mean	Median	Std	StdErr	Min	Max	n	%	n	%	n	%	n	%
Group 1 (MM) (N=183)																
	Screening	183	2.38	2.38	0.11	0.01	2.04	2.67	-	0.0	-	0.0	182	99.5	1	0.5
	Visit 2	180	2.36	2.36	0.12	0.01	2.08	3.13	-	0.0	-	0.0	177	98.3	3	1.7
	Visit 3	3	1.92	2.37	0.88	0.51	0.91	2.48	-	0.0	1	33.3	2	66.7	-	0.0
	Visit 4	174	2.34	2.34	0.15	0.01	0.97	2.64	2	1.1	2	1.1	172	97.7	-	0.0
	Visit 5	22	2.30	2.34	0.16	0.03	1.66	2.46	1	4.3	1	4.3	21	91.3	-	0.0
	Follow-Up	11	2.37	2.38	0.14	0.04	2.13	2.64	3	21.4	-	0.0	11	78.6	-	0.0
Group 2 (MP) (N=181)																
	Screening	180	2.37	2.37	0.10	0.01	2.14	2.67	1	0.6	-	0.0	179	98.9	1	0.6
	Visit 2	179	2.34	2.35	0.10	0.01	2.07	2.60	-	0.0	-	0.0	179	100.0	-	0.0
	Visit 3	0	-	-	-	-	-	-	-	-	-	-	-	-	-	-
	Visit 4	174	2.34	2.34	0.11	0.01	2.03	2.61	-	0.0	-	0.0	174	100.0	-	0.0
	Visit 5	19	2.33	2.32	0.08	0.02	2.21	2.55	1	5.0	-	0.0	19	95.0	-	0.0
	Follow-Up	6	2.37	2.33	0.10	0.04	2.28	2.54	2	25.0	-	0.0	6	75.0	-	0.0
Group 3 (PP) (N=181)																
	Screening	181	2.37	2.36	0.10	0.01	2.10	2.63	-	0.0	-	0.0	181	100.0	-	0.0
	Visit 2	176	2.34	2.35	0.14	0.01	1.13	2.71	2	1.1	1	0.6	174	97.8	1	0.6
	Visit 3	2	2.38	2.38	0.05	0.04	2.34	2.41	-	0.0	-	0.0	2	100.0	-	0.0
	Visit 4	175	2.35	2.34	0.11	0.01	2.14	2.73	-	0.0	-	0.0	173	98.9	2	1.1
	Visit 5	17	2.31	2.30	0.07	0.02	2.19	2.43	1	5.6	-	0.0	17	94.4	-	0.0
	Follow-Up	5	2.35	2.39	0.11	0.05	2.21	2.46	7	58.3	-	0.0	5	41.7	-	0.0

(Continued)

N: Number of subjects in the specified group
n: Number of subjects in the specified group (=Number of subjects with data available)
%: Percentage based on n
LLN: Lower limit of normal range
ULN: Upper limit of normal range
Cholesterol (Total, HDL, LDL) and beta-HCG only performed at Screening

Summary of laboratory data - Clinical Chemistry, descriptive statistics and count of abnormalities, Safety dataset

Calcium, total (mmol/l)

		n	Mean	Median	Std	StdErr	Min	Max	Missing n	Missing %	Below LLN n	Below LLN %	Normal n	Normal %	Above ULN n	Above ULN %
Group 4 (M-) (N=200)																
	Screening	199	2.31	2.32	0.10	0.01	2.02	2.59	1	0.5	-	0.0	199	99.5	-	0.0
	Visit 2	200	2.30	2.30	0.11	0.01	2.04	2.64	-	0.0	-	0.0	200	100.0	-	0.0
	Visit 3	19	2.32	2.34	0.09	0.02	2.14	2.46	-	0.0	-	0.0	19	100.0	-	0.0
	Visit 4	0	-	-	-	-	-	-	-	-	-	-	-	-	-	-
	Visit 5	0	-	-	-	-	-	-	-	-	-	-	-	-	-	-
	Follow-Up	7	2.33	2.33	0.13	0.05	2.12	2.53	-	0.0	-	0.0	7	100.0	-	0.0

N: Number of subjects in the specified group
n: Number of subjects in the specified group (=Number of subjects with data available)
%: Percentage based on n
LLN: Lower limit of normal range
ULN: Upper limit of normal range
Cholesterol (Total, HDL, LDL) and beta-HCG only performed at Screening

Summary of laboratory data - Clinical Chemistry, descriptive statistics and count of abnormalities, Safety dataset

Cholesterol - HDL (mmol/l)

								Missing		Below LLN		Normal		Above ULN	
	n	Mean	Median	Std	StdErr	Min	Max	n	%	n	%	n	%	n	%
Group 1 (MM) (N=183)															
Screening	183	1.67	1.58	0.44	0.03	0.96	3.37	-	0.0	-	0.0	183	100.0	-	0.0
Visit 2	0	-	-	-	-	-	-	-	-	-	-	-	-	-	-
Visit 3	0	-	-	-	-	-	-	-	-	-	-	-	-	-	-
Visit 4	0	-	-	-	-	-	-	-	-	-	-	-	-	-	-
Visit 5	0	-	-	-	-	-	-	-	-	-	-	-	-	-	-
Follow-Up	0	-	-	-	-	-	-	-	-	-	-	-	-	-	-
Group 2 (MP) (N=181)															
Screening	180	1.68	1.65	0.41	0.03	0.91	2.90	1	0.6	-	0.0	180	99.4	-	0.0
Visit 2	0	-	-	-	-	-	-	-	-	-	-	-	-	-	-
Visit 3	0	-	-	-	-	-	-	-	-	-	-	-	-	-	-
Visit 4	0	-	-	-	-	-	-	-	-	-	-	-	-	-	-
Visit 5	0	-	-	-	-	-	-	-	-	-	-	-	-	-	-
Follow-Up	0	-	-	-	-	-	-	-	-	-	-	-	-	-	-
Group 3 (PP) (N=181)															
Screening	181	1.67	1.61	0.40	0.03	0.62	2.75	-	0.0	3	1.7	178	98.3	-	0.0
Visit 2	0	-	-	-	-	-	-	-	-	-	-	-	-	-	-
Visit 3	0	-	-	-	-	-	-	-	-	-	-	-	-	-	-
Visit 4	0	-	-	-	-	-	-	-	-	-	-	-	-	-	-
Visit 5	0	-	-	-	-	-	-	-	-	-	-	-	-	-	-
Follow-Up	0	-	-	-	-	-	-	-	-	-	-	-	-	-	-

(Continued)

N: Number of subjects in the specified group
n: Number of subjects in the specified group (=Number of subjects with data available)
%: Percentage based on n
LLN: Lower limit of normal range
ULN: Upper limit of normal range
Cholesterol (Total, HDL, LDL) and beta-HCG only performed at Screening

Summary of laboratory data - Clinical Chemistry, descriptive statistics and count of abnormalities, Safety dataset

Cholesterol - HDL (mmol/l)

								Missing		Below LLN		Normal		Above ULN	
	n	Mean	Median	Std	StdErr	Min	Max	n	%	n	%	n	%	n	%
Group 4 (M-) (N=200)															
Screening	199	1.74	1.68	0.42	0.03	0.83	3.06	1	0.5	2	1.0	197	98.5	-	0.0
Visit 2	0	-	-	-	-	-	-	-	-	-	-	-	-	-	-
Visit 3	0	-	-	-	-	-	-	-	-	-	-	-	-	-	-
Visit 4	0	-	-	-	-	-	-	-	-	-	-	-	-	-	-
Visit 5	0	-	-	-	-	-	-	-	-	-	-	-	-	-	-
Follow-Up	0	-	-	-	-	-	-	-	-	-	-	-	-	-	-

N: Number of subjects in the specified group
n: Number of subjects in the specified group (=Number of subjects with data available)
%: Percentage based on n
LLN: Lower limit of normal range
ULN: Upper limit of normal range
Cholesterol (Total, HDL, LDL) and beta-HCG only performed at Screening

Summary of laboratory data - Clinical Chemistry, descriptive statistics and count of abnormalities, Safety dataset

Cholesterol - LDL (mmol/l)

								Missing		Below LLN		Normal		Above ULN	
	n	Mean	Median	Std	StdErr	Min	Max	n	%	n	%	n	%	n	%
Group 1 (MM) (N=183)															
Screening	183	2.80	2.64	0.80	0.06	1.19	5.65	-	0.0	-	0.0	171	93.4	12	6.6
Visit 2	0	-	-	-	-	-	-	-	-	-	-	-	-	-	-
Visit 3	0	-	-	-	-	-	-	-	-	-	-	-	-	-	-
Visit 4	0	-	-	-	-	-	-	-	-	-	-	-	-	-	-
Visit 5	0	-	-	-	-	-	-	-	-	-	-	-	-	-	-
Follow-Up	0	-	-	-	-	-	-	-	-	-	-	-	-	-	-
Group 2 (MP) (N=181)															
Screening	180	2.84	2.80	0.77	0.06	1.50	6.09	1	0.6	-	0.0	171	94.5	9	5.0
Visit 2	0	-	-	-	-	-	-	-	-	-	-	-	-	-	-
Visit 3	0	-	-	-	-	-	-	-	-	-	-	-	-	-	-
Visit 4	0	-	-	-	-	-	-	-	-	-	-	-	-	-	-
Visit 5	0	-	-	-	-	-	-	-	-	-	-	-	-	-	-
Follow-Up	0	-	-	-	-	-	-	-	-	-	-	-	-	-	-
Group 3 (PP) (N=181)															
Screening	181	2.84	2.80	0.73	0.05	1.11	4.56	-	0.0	-	0.0	176	97.2	5	2.8
Visit 2	0	-	-	-	-	-	-	-	-	-	-	-	-	-	-
Visit 3	0	-	-	-	-	-	-	-	-	-	-	-	-	-	-
Visit 4	0	-	-	-	-	-	-	-	-	-	-	-	-	-	-
Visit 5	0	-	-	-	-	-	-	-	-	-	-	-	-	-	-
Follow-Up	0	-	-	-	-	-	-	-	-	-	-	-	-	-	-

(Continued)

N: Number of subjects in the specified group
n: Number of subjects in the specified group (=Number of subjects with data available)
%: Percentage based on n
LLN: Lower limit of normal range
ULN: Upper limit of normal range
Cholesterol (Total, HDL, LDL) and beta-HCG only performed at Screening

Summary of laboratory data - Clinical Chemistry, descriptive statistics and count of abnormalities, Safety dataset

Cholesterol - LDL (mmol/l)

								Missing		Below LLN		Normal		Above ULN	
		n	Mean	Median	Std	StdErr	Min	Max	n	%	n	%	n	%	n	%
Group 4 (M-) (N=200)																
	Screening	199	3.15	3.16	0.82	0.06	1.37	5.85	1	0.5	-	0.0	181	90.5	18	9.0
	Visit 2	0	-	-	-	-	-	-	-	-	-	-	-	-	-	-
	Visit 3	0	-	-	-	-	-	-	-	-	-	-	-	-	-	-
	Visit 4	0	-	-	-	-	-	-	-	-	-	-	-	-	-	-
	Visit 5	0	-	-	-	-	-	-	-	-	-	-	-	-	-	-
	Follow-Up	0	-	-	-	-	-	-	-	-	-	-	-	-	-	-

N: Number of subjects in the specified group
n: Number of subjects in the specified group (=Number of subjects with data available)
%: Percentage based on n
LLN: Lower limit of normal range
ULN: Upper limit of normal range
Cholesterol (Total, HDL, LDL) and beta-HCG only performed at Screening

Summary of laboratory data - Clinical Chemistry, descriptive statistics and count of abnormalities, Safety dataset

Cholesterol, total (mmol/l)

								Missing		Below LLN		Normal		Above ULN	
	n	Mean	Median	Std	StdErr	Min	Max	n	%	n	%	n	%	n	%
Group 1 (MM) (N=183)															
Screening	183	4.68	4.56	0.91	0.07	2.75	8.31	-	0.0	-	0.0	166	90.7	17	9.3
Visit 2	0	-	-	-	-	-	-	-	-	-	-	-	-	-	-
Visit 3	0	-	-	-	-	-	-	-	-	-	-	-	-	-	-
Visit 4	0	-	-	-	-	-	-	-	-	-	-	-	-	-	-
Visit 5	0	-	-	-	-	-	-	-	-	-	-	-	-	-	-
Follow-Up	0	-	-	-	-	-	-	-	-	-	-	-	-	-	-
Group 2 (MP) (N=181)															
Screening	180	4.76	4.74	0.86	0.06	3.08	7.80	1	0.6	-	0.0	168	92.8	12	6.6
Visit 2	0	-	-	-	-	-	-	-	-	-	-	-	-	-	-
Visit 3	0	-	-	-	-	-	-	-	-	-	-	-	-	-	-
Visit 4	0	-	-	-	-	-	-	-	-	-	-	-	-	-	-
Visit 5	0	-	-	-	-	-	-	-	-	-	-	-	-	-	-
Follow-Up	0	-	-	-	-	-	-	-	-	-	-	-	-	-	-
Group 3 (PP) (N=181)															
Screening	181	4.73	4.71	0.87	0.06	2.64	6.55	-	0.0	-	0.0	165	91.2	16	8.8
Visit 2	0	-	-	-	-	-	-	-	-	-	-	-	-	-	-
Visit 3	0	-	-	-	-	-	-	-	-	-	-	-	-	-	-
Visit 4	0	-	-	-	-	-	-	-	-	-	-	-	-	-	-
Visit 5	0	-	-	-	-	-	-	-	-	-	-	-	-	-	-
Follow-Up	0	-	-	-	-	-	-	-	-	-	-	-	-	-	-

(Continued)

N: Number of subjects in the specified group
n: Number of subjects in the specified group (=Number of subjects with data available)
%: Percentage based on n
LLN: Lower limit of normal range
ULN: Upper limit of normal range
Cholesterol (Total, HDL, LDL) and beta-HCG only performed at Screening

Summary of laboratory data - Clinical Chemistry, descriptive statistics and count of abnormalities, Safety dataset

Cholesterol, total (mmol/l)

								Missing		Below LLN		Normal		Above ULN	
	n	Mean	Median	Std	StdErr	Min	Max	n	%	n	%	n	%	n	%
Group 4 (M-) (N=200)															
Screening	199	5.11	5.15	0.93	0.07	3.08	8.34	1	0.5	-	0.0	173	86.5	26	13.0
Visit 2	0	-	-	-	-	-	-	-	-	-	-	-	-	-	-
Visit 3	0	-	-	-	-	-	-	-	-	-	-	-	-	-	-
Visit 4	0	-	-	-	-	-	-	-	-	-	-	-	-	-	-
Visit 5	0	-	-	-	-	-	-	-	-	-	-	-	-	-	-
Follow-Up	0	-	-	-	-	-	-	-	-	-	-	-	-	-	-

N: Number of subjects in the specified group
n: Number of subjects in the specified group (=Number of subjects with data available)
%: Percentage based on n
LLN: Lower limit of normal range
ULN: Upper limit of normal range
Cholesterol (Total, HDL, LDL) and beta-HCG only performed at Screening

Summary of laboratory data - Clinical Chemistry, descriptive statistics and count of abnormalities, Safety dataset

Creatinine (µmol/l)

								Missing		Below LLN		Normal		Above ULN	
	n	Mean	Median	Std	StdErr	Min	Max	n	%	n	%	n	%	n	%
Group 1 (MM) (N=183)															
Screening	183	78.88	76.90	12.49	0.92	50.40	123.80	-	0.0	-	0.0	183	100.0	-	0.0
Visit 2	180	80.99	79.60	14.99	1.12	53.90	124.60	-	0.0	-	0.0	178	98.9	2	1.1
Visit 3	3	58.93	61.90	34.55	19.94	23.00	91.90	-	0.0	-	0.0	3	100.0	-	0.0
Visit 4	175	81.44	80.40	15.90	1.20	19.40	128.20	1	0.6	-	0.0	173	98.3	2	1.1
Visit 5	22	79.89	79.60	17.21	3.67	49.50	116.70	1	4.3	-	0.0	22	95.7	-	0.0
Follow-Up	11	88.88	89.30	16.58	5.00	60.10	117.60	3	21.4	-	0.0	11	78.6	-	0.0
Group 2 (MP) (N=181)															
Screening	180	78.81	76.90	12.17	0.91	58.30	114.90	1	0.6	-	0.0	178	98.3	2	1.1
Visit 2	179	79.65	78.70	14.10	1.05	55.70	146.70	-	0.0	-	0.0	176	98.3	3	1.7
Visit 3	0	-	-	-	-	-	-	-	-	-	-	-	-	-	-
Visit 4	174	79.63	77.80	13.59	1.03	56.60	122.00	-	0.0	-	0.0	173	99.4	1	0.6
Visit 5	19	81.61	80.40	13.61	3.12	61.90	114.00	1	5.0	-	0.0	18	90.0	1	5.0
Follow-Up	6	93.53	95.00	21.59	8.81	59.20	120.20	2	25.0	-	0.0	5	62.5	1	12.5
Group 3 (PP) (N=181)															
Screening	181	78.40	76.00	13.23	0.98	57.50	131.70	-	0.0	-	0.0	180	99.4	1	0.6
Visit 2	176	79.73	79.60	13.36	1.01	42.40	123.80	2	1.1	-	0.0	176	98.9	-	0.0
Visit 3	2	86.60	86.60	16.26	11.50	75.10	98.10	-	0.0	-	0.0	2	100.0	-	0.0
Visit 4	175	80.47	77.80	13.56	1.03	51.30	116.70	-	0.0	-	0.0	175	100.0	-	0.0
Visit 5	17	78.51	80.40	11.54	2.80	55.70	97.20	1	5.6	-	0.0	17	94.4	-	0.0
Follow-Up	5	89.28	85.70	23.27	10.41	62.80	124.60	7	58.3	-	0.0	4	33.3	1	8.3

(Continued)

N: Number of subjects in the specified group
n: Number of subjects in the specified group (=Number of subjects with data available)
%: Percentage based on n
LLN: Lower limit of normal range
ULN: Upper limit of normal range
Cholesterol (Total, HDL, LDL) and beta-HCG only performed at Screening

Summary of laboratory data - Clinical Chemistry, descriptive statistics and count of abnormalities, Safety dataset

Creatinine (µmol/l)

		n	Mean	Median	Std	StdErr	Min	Max	Missing n	Missing %	Below LLN n	Below LLN %	Normal n	Normal %	Above ULN n	Above ULN %
Group 4 (M-) (N=200)																
	Screening	199	78.06	76.90	12.67	0.90	53.00	112.30	1	0.5	-	0.0	199	99.5	-	0.0
	Visit 2	200	81.69	80.40	14.57	1.03	55.70	133.50	-	0.0	-	0.0	195	97.5	5	2.5
	Visit 3	19	76.35	76.00	11.92	2.73	55.70	96.40	-	0.0	-	0.0	19	100.0	-	0.0
	Visit 4	0	-	-	-	-	-	-	-	-	-	-	-	-	-	-
	Visit 5	0	-	-	-	-	-	-	-	-	-	-	-	-	-	-
	Follow-Up	7	77.27	75.10	9.19	3.48	65.40	94.60	-	0.0	-	0.0	7	100.0	-	0.0

N: Number of subjects in the specified group
n: Number of subjects in the specified group (=Number of subjects with data available)
%: Percentage based on n
LLN: Lower limit of normal range
ULN: Upper limit of normal range
Cholesterol (Total, HDL, LDL) and beta-HCG only performed at Screening

Summary of laboratory data - Clinical Chemistry, descriptive statistics and count of abnormalities, Safety dataset

GOT (AST) (U/l)

								Missing		Below LLN		Normal		Above ULN	
		n	Mean	Median	Std	StdErr	Min	Max	n	%	n	%	n	%	n	%
Group 1 (MM) (N=183)																
	Screening	183	21.54	20.00	5.91	0.44	12.00	49.00	-	0.0	-	0.0	183	100.0	-	0.0
	Visit 2	180	20.84	20.00	5.38	0.40	12.00	40.00	-	0.0	-	0.0	178	98.9	2	1.1
	Visit 3	3	14.33	17.00	7.37	4.26	6.00	20.00	-	0.0	-	0.0	3	100.0	-	0.0
	Visit 4	175	22.58	20.00	16.90	1.28	7.00	222.00	1	0.6	-	0.0	172	97.7	3	1.7
	Visit 5	22	21.27	21.00	4.55	0.97	14.00	30.00	1	4.3	-	0.0	22	95.7	-	0.0
	Follow-Up	11	24.55	23.00	7.87	2.37	13.00	41.00	3	21.4	-	0.0	11	78.6	-	0.0
Group 2 (MP) (N=181)																
	Screening	180	21.37	20.00	6.44	0.48	11.00	50.00	1	0.6	-	0.0	177	97.8	3	1.7
	Visit 2	179	21.02	19.00	6.84	0.51	12.00	57.00	-	0.0	-	0.0	173	96.6	6	3.4
	Visit 3	0	-	-	-	-	-	-	-	-	-	-	-	-	-	-
	Visit 4	174	20.50	19.00	6.23	0.47	11.00	68.00	-	0.0	-	0.0	172	98.9	2	1.1
	Visit 5	19	19.95	19.00	4.77	1.09	13.00	30.00	1	5.0	-	0.0	19	95.0	-	0.0
	Follow-Up	6	24.67	24.50	5.24	2.14	16.00	31.00	2	25.0	-	0.0	6	75.0	-	0.0
Group 3 (PP) (N=181)																
	Screening	181	20.84	19.00	6.85	0.51	10.00	69.00	-	0.0	-	0.0	179	98.9	2	1.1
	Visit 2	176	20.80	19.00	6.48	0.49	9.00	64.00	2	1.1	-	0.0	174	97.8	2	1.1
	Visit 3	2	46.00	46.00	33.94	24.00	22.00	70.00	-	0.0	-	0.0	1	50.0	1	50.0
	Visit 4	175	20.52	19.00	8.33	0.63	12.00	100.00	-	0.0	-	0.0	174	99.4	1	0.6
	Visit 5	17	20.82	21.00	5.35	1.30	13.00	34.00	1	5.6	-	0.0	17	94.4	-	0.0
	Follow-Up	5	21.60	22.00	7.44	3.33	12.00	31.00	7	58.3	-	0.0	5	41.7	-	0.0

(Continued)

N: Number of subjects in the specified group
n: Number of subjects in the specified group (=Number of subjects with data available)
%: Percentage based on n
LLN: Lower limit of normal range
ULN: Upper limit of normal range
Cholesterol (Total, HDL, LDL) and beta-HCG only performed at Screening

Summary of laboratory data - Clinical Chemistry, descriptive statistics and count of abnormalities, Safety dataset

GOT (AST) (U/l)

								Missing		Below LLN		Normal		Above ULN	
	n	Mean	Median	Std	StdErr	Min	Max	n	%	n	%	n	%	n	%
Group 4 (M-) (N=200)															
Screening	199	22.89	21.00	8.52	0.60	9.00	84.00	1	0.5	-	0.0	192	96.0	7	3.5
Visit 2	200	22.48	21.00	7.62	0.54	12.00	63.00	-	0.0	-	0.0	194	97.0	6	3.0
Visit 3	19	28.47	25.00	13.53	3.10	14.00	68.00	-	0.0	-	0.0	17	89.5	2	10.5
Visit 4	0	-	-	-	-	-	-	-	-	-	-	-	-	-	-
Visit 5	0	-	-	-	-	-	-	-	-	-	-	-	-	-	-
Follow-Up	7	19.71	20.00	4.79	1.81	14.00	27.00	-	0.0	-	0.0	7	100.0	-	0.0

N: Number of subjects in the specified group
n: Number of subjects in the specified group (=Number of subjects with data available)
%: Percentage based on n
LLN: Lower limit of normal range
ULN: Upper limit of normal range
Cholesterol (Total, HDL, LDL) and beta-HCG only performed at Screening

Summary of laboratory data - Clinical Chemistry, descriptive statistics and count of abnormalities, Safety dataset

GPT (ALT) (U/l)

									Missing		Below LLN		Normal		Above ULN	
		n	Mean	Median	Std	StdErr	Min	Max	n	%	n	%	n	%	n	%
Group 1 (MM) (N=183)																
	Screening	183	21.80	18.00	11.00	0.81	6.00	71.00	-	0.0	-	0.0	173	94.5	10	5.5
	Visit 2	180	20.71	18.00	10.17	0.76	5.00	75.00	-	0.0	-	0.0	178	98.9	2	1.1
	Visit 3	3	14.67	10.00	11.72	6.77	6.00	28.00	-	0.0	-	0.0	3	100.0	-	0.0
	Visit 4	175	22.11	19.00	12.70	0.96	5.00	79.00	1	0.6	-	0.0	165	93.8	10	5.7
	Visit 5	22	21.09	19.00	9.80	2.09	9.00	56.00	1	4.3	-	0.0	21	91.3	1	4.3
	Follow-Up	11	31.27	26.00	23.95	7.22	3.00	98.00	3	21.4	-	0.0	9	64.3	2	14.3
Group 2 (MP) (N=181)																
	Screening	180	21.76	17.00	13.70	1.02	7.00	108.00	1	0.6	-	0.0	171	94.5	9	5.0
	Visit 2	179	20.87	17.00	12.26	0.92	6.00	79.00	-	0.0	-	0.0	171	95.5	8	4.5
	Visit 3	0	-	-	-	-	-	-	-	-	-	-	-	-	-	-
	Visit 4	174	20.68	17.00	14.07	1.07	6.00	145.00	-	0.0	-	0.0	166	95.4	8	4.6
	Visit 5	19	17.11	16.00	7.04	1.61	8.00	34.00	1	5.0	-	0.0	19	95.0	-	0.0
	Follow-Up	6	32.83	30.50	17.24	7.04	12.00	55.00	2	25.0	-	0.0	4	50.0	2	25.0
Group 3 (PP) (N=181)																
	Screening	181	20.57	18.00	9.53	0.71	5.00	64.00	-	0.0	-	0.0	176	97.2	5	2.8
	Visit 2	176	20.51	18.00	9.97	0.75	6.00	73.00	2	1.1	-	0.0	171	96.1	5	2.8
	Visit 3	2	57.00	57.00	53.74	38.00	19.00	95.00	-	0.0	-	0.0	1	50.0	1	50.0
	Visit 4	175	21.02	18.00	14.52	1.10	5.00	124.00	-	0.0	-	0.0	166	94.9	9	5.1
	Visit 5	17	22.71	18.00	10.80	2.62	11.00	46.00	1	5.6	-	0.0	16	88.9	1	5.6
	Follow-Up	5	20.80	14.00	14.99	6.70	10.00	46.00	7	58.3	-	0.0	5	41.7	-	0.0

(Continued)

N: Number of subjects in the specified group
n: Number of subjects in the specified group (=Number of subjects with data available)
%: Percentage based on n
LLN: Lower limit of normal range
ULN: Upper limit of normal range
Cholesterol (Total, HDL, LDL) and beta-HCG only performed at Screening

Summary of laboratory data - Clinical Chemistry, descriptive statistics and count of abnormalities, Safety dataset

GPT (ALT) (U/l)

		n	Mean	Median	Std	StdErr	Min	Max	Missing		Below LLN		Normal		Above ULN	
									n	%	n	%	n	%	n	%
Group 4 (M-) (N=200)																
	Screening	199	25.21	21.00	14.61	1.04	6.00	95.00	1	0.5	-	0.0	179	89.5	20	10.0
	Visit 2	200	24.02	20.00	14.69	1.04	3.00	121.00	-	0.0	-	0.0	190	95.0	10	5.0
	Visit 3	19	34.68	29.00	26.88	6.17	12.00	117.00	-	0.0	-	0.0	15	78.9	4	21.1
	Visit 4	0	-	-	-	-	-	-	-	-	-	-	-	-	-	-
	Visit 5	0	-	-	-	-	-	-	-	-	-	-	-	-	-	-
	Follow-Up	7	19.14	19.00	7.40	2.80	12.00	34.00	-	0.0	-	0.0	7	100.0	-	0.0

N: Number of subjects in the specified group
n: Number of subjects in the specified group (=Number of subjects with data available)
%: Percentage based on n
LLN: Lower limit of normal range
ULN: Upper limit of normal range
Cholesterol (Total, HDL, LDL) and beta-HCG only performed at Screening

Summary of laboratory data - Clinical Chemistry, descriptive statistics and count of abnormalities, Safety dataset

Potassium (mmol/l)

		n	Mean	Median	Std	StdErr	Min	Max	Missing		Below LLN		Normal		Above ULN	
									n	%	n	%	n	%	n	%
Group 1 (MM) (N=183)																
	Screening	183	4.37	4.40	0.32	0.02	3.50	5.30	-	0.0	-	0.0	183	100.0	-	0.0
	Visit 2	180	4.21	4.20	0.31	0.02	3.60	5.60	-	0.0	-	0.0	179	99.4	1	0.6
	Visit 3	3	3.63	4.20	1.44	0.83	2.00	4.70	-	0.0	1	33.3	2	66.7	-	0.0
	Visit 4	175	4.21	4.20	0.36	0.03	2.50	5.20	1	0.6	2	1.1	173	98.3	-	0.0
	Visit 5	22	4.14	4.00	0.30	0.07	3.60	4.80	1	4.3	-	0.0	22	95.7	-	0.0
	Follow-Up	11	4.34	4.20	0.30	0.09	3.90	4.80	3	21.4	-	0.0	11	78.6	-	0.0
Group 2 (MP) (N=181)																
	Screening	180	4.38	4.30	0.32	0.02	3.70	5.30	1	0.6	-	0.0	180	99.4	-	0.0
	Visit 2	179	4.20	4.20	0.29	0.02	3.50	5.00	-	0.0	-	0.0	179	100.0	-	0.0
	Visit 3	0	-	-	-	-	-	-	-	-	-	-	-	-	-	-
	Visit 4	174	4.19	4.20	0.28	0.02	3.60	5.10	-	0.0	-	0.0	174	100.0	-	0.0
	Visit 5	19	4.29	4.20	0.36	0.08	3.80	5.20	1	5.0	-	0.0	19	95.0	-	0.0
	Follow-Up	6	4.15	4.05	0.39	0.16	3.60	4.60	2	25.0	-	0.0	6	75.0	-	0.0
Group 3 (PP) (N=181)																
	Screening	181	4.39	4.40	0.33	0.02	3.50	5.40	-	0.0	-	0.0	181	100.0	-	0.0
	Visit 2	176	4.22	4.20	0.29	0.02	3.00	5.30	2	1.1	1	0.6	175	98.3	-	0.0
	Visit 3	2	4.15	4.15	0.49	0.35	3.80	4.50	-	0.0	-	0.0	2	100.0	-	0.0
	Visit 4	174	4.25	4.20	0.36	0.03	3.60	6.40	1	0.6	-	0.0	172	98.3	2	1.1
	Visit 5	17	4.22	4.20	0.25	0.06	3.80	4.70	1	5.6	-	0.0	17	94.4	-	0.0
	Follow-Up	5	4.42	4.30	0.22	0.10	4.20	4.70	7	58.3	-	0.0	5	41.7	-	0.0

(Continued)

N: Number of subjects in the specified group
n: Number of subjects in the specified group (=Number of subjects with data available)
%: Percentage based on n
LLN: Lower limit of normal range
ULN: Upper limit of normal range
Cholesterol (Total, HDL, LDL) and beta-HCG only performed at Screening

Summary of laboratory data - Clinical Chemistry, descriptive statistics and count of abnormalities, Safety dataset

Potassium (mmol/l)

		n	Mean	Median	Std	StdErr	Min	Max	Missing		Below LLN		Normal		Above ULN	
									n	%	n	%	n	%	n	%
Group 4 (M-) (N=200)																
	Screening	199	4.44	4.40	0.36	0.03	3.70	5.70	1	0.5	-	0.0	197	98.5	2	1.0
	Visit 2	200	4.26	4.30	0.29	0.02	3.60	5.40	-	0.0	-	0.0	200	100.0	-	0.0
	Visit 3	19	4.12	4.20	0.36	0.08	3.30	4.80	-	0.0	-	0.0	19	100.0	-	0.0
	Visit 4	0	-	-	-	-	-	-	-	-	-	-	-	-	-	-
	Visit 5	0	-	-	-	-	-	-	-	-	-	-	-	-	-	-
	Follow-Up	7	4.29	4.20	0.40	0.15	3.80	5.00	-	0.0	-	0.0	7	100.0	-	0.0

N: Number of subjects in the specified group
n: Number of subjects in the specified group (=Number of subjects with data available)
%: Percentage based on n
LLN: Lower limit of normal range
ULN: Upper limit of normal range
Cholesterol (Total, HDL, LDL) and beta-HCG only performed at Screening

Summary of laboratory data - Clinical Chemistry, descriptive statistics and count of abnormalities, Safety dataset

Sodium (mmol/l)

		n	Mean	Median	Std	StdErr	Min	Max	Missing		Below LLN		Normal		Above ULN	
									n	%	n	%	n	%	n	%
Group 1 (MM) (N=183)																
	Screening	183	140.17	140.00	1.81	0.13	135.00	145.00	-	0.0	-	0.0	183	100.0	-	0.0
	Visit 2	179	140.40	140.00	2.10	0.16	132.00	149.00	1	0.6	-	0.0	176	97.8	3	1.7
	Visit 3	2	141.00	141.00	2.83	2.00	139.00	143.00	1	33.3	-	0.0	2	66.7	-	0.0
	Visit 4	173	140.52	141.00	2.05	0.16	136.00	147.00	3	1.7	-	0.0	171	97.2	2	1.1
	Visit 5	22	139.55	140.50	4.23	0.90	122.00	144.00	1	4.3	1	4.3	21	91.3	-	0.0
	Follow-Up	11	140.73	140.00	1.95	0.59	138.00	144.00	3	21.4	-	0.0	11	78.6	-	0.0
Group 2 (MP) (N=181)																
	Screening	180	140.19	140.00	1.79	0.13	135.00	145.00	1	0.6	-	0.0	180	99.4	-	0.0
	Visit 2	179	140.46	140.00	1.97	0.15	135.00	146.00	-	0.0	-	0.0	178	99.4	1	0.6
	Visit 3	0	-	-	-	-	-	-	-	-	-	-	-	-	-	-
	Visit 4	174	140.45	140.00	1.90	0.14	134.00	147.00	-	0.0	-	0.0	173	99.4	1	0.6
	Visit 5	19	140.00	140.00	1.45	0.33	137.00	143.00	1	5.0	-	0.0	19	95.0	-	0.0
	Follow-Up	6	140.67	141.00	2.34	0.95	138.00	143.00	2	25.0	-	0.0	6	75.0	-	0.0
Group 3 (PP) (N=181)																
	Screening	181	140.18	140.00	1.82	0.14	137.00	146.00	-	0.0	-	0.0	180	99.4	1	0.6
	Visit 2	175	140.19	140.00	1.80	0.14	134.00	145.00	3	1.7	-	0.0	175	98.3	-	0.0
	Visit 3	2	141.00	141.00	0.00	0.00	141.00	141.00	-	0.0	-	0.0	2	100.0	-	0.0
	Visit 4	175	140.32	140.00	2.16	0.16	130.00	147.00	-	0.0	1	0.6	171	97.7	3	1.7
	Visit 5	17	139.94	140.00	1.68	0.41	137.00	143.00	1	5.6	-	0.0	17	94.4	-	0.0
	Follow-Up	5	140.20	140.00	1.64	0.73	139.00	143.00	7	58.3	-	0.0	5	41.7	-	0.0

(Continued)

N: Number of subjects in the specified group
n: Number of subjects in the specified group (=Number of subjects with data available)
%: Percentage based on n
LLN: Lower limit of normal range
ULN: Upper limit of normal range
Cholesterol (Total, HDL, LDL) and beta-HCG only performed at Screening

Summary of laboratory data - Clinical Chemistry, descriptive statistics and count of abnormalities, Safety dataset

Sodium (mmol/l)

								Missing		Below LLN		Normal		Above ULN	
	n	Mean	Median	Std	StdErr	Min	Max	n	%	n	%	n	%	n	%
Group 4 (M-) (N=200)															
Screening	199	140.11	140.00	2.11	0.15	135.00	145.00	1	0.5	-	0.0	199	99.5	-	0.0
Visit 2	200	140.37	140.00	2.10	0.15	132.00	146.00	-	0.0	-	0.0	199	99.5	1	0.5
Visit 3	19	139.74	140.00	1.69	0.39	137.00	142.00	-	0.0	-	0.0	19	100.0	-	0.0
Visit 4	0	-	-	-	-	-	-	-	-	-	-	-	-	-	-
Visit 5	0	-	-	-	-	-	-	-	-	-	-	-	-	-	-
Follow-Up	7	138.86	138.00	2.19	0.83	137.00	142.00	-	0.0	-	0.0	7	100.0	-	0.0

N: Number of subjects in the specified group
n: Number of subjects in the specified group (=Number of subjects with data available)
%: Percentage based on n
LLN: Lower limit of normal range
ULN: Upper limit of normal range
Cholesterol (Total, HDL, LDL) and beta-HCG only performed at Screening

Summary of laboratory data - Clinical Chemistry, descriptive statistics and count of abnormalities, Safety dataset

Troponin I (µg/l)

								Missing		Below LLN		Normal		Above ULN	
		n	Mean	Median	Std	StdErr	Min	Max	n	%	n	%	n	%	n	%
Group 1 (MM) (N=183)																
	Screening	183	0.01	0.01	0.01	0.00	0.01	0.08	-	0.0	-	0.0	183	100.0	-	0.0
	Visit 2	180	0.01	0.01	0.00	0.00	0.01	0.04	-	0.0	-	0.0	180	100.0	-	0.0
	Visit 3	3	0.01	0.01	0.00	0.00	0.01	0.01	-	0.0	-	0.0	3	100.0	-	0.0
	Visit 4	175	0.01	0.01	0.02	0.00	0.01	0.23	1	0.6	-	0.0	175	99.4	-	0.0
	Visit 5	22	0.01	0.01	0.00	0.00	0.01	0.02	1	4.3	-	0.0	22	95.7	-	0.0
	Follow-Up	11	0.02	0.01	0.02	0.00	0.01	0.06	3	21.4	-	0.0	11	78.6	-	0.0
Group 2 (MP) (N=181)																
	Screening	180	0.01	0.01	0.02	0.00	0.01	0.18	1	0.6	-	0.0	180	99.4	-	0.0
	Visit 2	179	0.01	0.01	0.04	0.00	0.01	0.52	-	0.0	-	0.0	179	100.0	-	0.0
	Visit 3	0	-	-	-	-	-	-	-	-	-	-	-	-	-	-
	Visit 4	174	0.01	0.01	0.01	0.00	0.01	0.10	-	0.0	-	0.0	174	100.0	-	0.0
	Visit 5	19	0.02	0.01	0.01	0.00	0.01	0.07	1	5.0	-	0.0	19	95.0	-	0.0
	Follow-Up	6	0.02	0.01	0.01	0.01	0.01	0.04	2	25.0	-	0.0	6	75.0	-	0.0
Group 3 (PP) (N=181)																
	Screening	181	0.02	0.01	0.04	0.00	0.01	0.50	-	0.0	-	0.0	181	100.0	-	0.0
	Visit 2	176	0.01	0.01	0.02	0.00	0.01	0.25	2	1.1	-	0.0	176	98.9	-	0.0
	Visit 3	2	0.02	0.02	0.01	0.01	0.01	0.02	-	0.0	-	0.0	2	100.0	-	0.0
	Visit 4	175	0.01	0.01	0.00	0.00	0.01	0.05	-	0.0	-	0.0	175	100.0	-	0.0
	Visit 5	17	0.01	0.01	0.00	0.00	0.01	0.02	1	5.6	-	0.0	17	94.4	-	0.0
	Follow-Up	5	0.01	0.01	0.00	0.00	0.01	0.02	7	58.3	-	0.0	5	41.7	-	0.0

(Continued)

N: Number of subjects in the specified group
n: Number of subjects in the specified group (=Number of subjects with data available)
%: Percentage based on n
LLN: Lower limit of normal range
ULN: Upper limit of normal range
Cholesterol (Total, HDL, LDL) and beta-HCG only performed at Screening

Summary of laboratory data - Clinical Chemistry, descriptive statistics and count of abnormalities, Safety dataset

Troponin I (µg/l)

		n	Mean	Median	Std	StdErr	Min	Max	Missing		Below LLN		Normal		Above ULN	
									n	%	n	%	n	%	n	%
Group 4 (M-) (N=200)																
	Screening	199	0.02	0.01	0.02	0.00	0.01	0.14	1	0.5	-	0.0	199	99.5	-	0.0
	Visit 2	200	0.02	0.01	0.04	0.00	0.01	0.50	-	0.0	-	0.0	200	100.0	-	0.0
	Visit 3	19	0.04	0.01	0.11	0.03	0.01	0.50	-	0.0	-	0.0	19	100.0	-	0.0
	Visit 4	0	-	-	-	-	-	-	-	-	-	-	-	-	-	-
	Visit 5	0	-	-	-	-	-	-	-	-	-	-	-	-	-	-
	Follow-Up	7	0.02	0.02	0.01	0.00	0.01	0.04	-	0.0	-	0.0	7	100.0	-	0.0

N: Number of subjects in the specified group
n: Number of subjects in the specified group (=Number of subjects with data available)
%: Percentage based on n
LLN: Lower limit of normal range
ULN: Upper limit of normal range
Cholesterol (Total, HDL, LDL) and beta-HCG only performed at Screening

14.3.2 Summary of laboratory data - Haematology, descriptive statistics and count of abnormalities, Safety dataset

Erythrocytes (10E12/l)

		n	Mean	Median	Std	StdErr	Min	Max	Missing		Below LLN		Normal		Above ULN	
									n	%	n	%	n	%	n	%
Group 1 (MM) (N=183)																
	Screening	183	4.94	4.90	0.42	0.03	4.00	6.30	-	0.0	1	0.5	181	98.9	1	0.5
	Visit 2	180	4.85	4.80	0.41	0.03	3.80	5.90	-	0.0	4	2.2	176	97.8	-	0.0
	Visit 3	2	4.45	4.45	0.07	0.05	4.40	4.50	-	0.0	-	0.0	2	100.0	-	0.0
	Visit 4	176	4.83	4.80	0.43	0.03	3.70	5.90	-	0.0	2	1.1	174	98.9	-	0.0
	Visit 5	20	4.82	4.75	0.47	0.10	4.20	5.60	1	4.8	-	0.0	20	95.2	-	0.0
	Follow-Up	11	4.98	4.90	0.35	0.11	4.50	5.60	-	0.0	-	0.0	11	100.0	-	0.0
Group 2 (MP) (N=181)																
	Screening	181	4.91	4.90	0.43	0.03	4.00	6.10	-	0.0	2	1.1	179	98.9	-	0.0
	Visit 2	178	4.78	4.80	0.41	0.03	3.70	6.00	1	0.6	4	2.2	174	97.2	-	0.0
	Visit 3	0	-	-	-	-	-	-	-	-	-	-	-	-	-	-
	Visit 4	174	4.80	4.80	0.40	0.03	4.00	5.70	-	0.0	4	2.3	170	97.7	-	0.0
	Visit 5	20	4.77	4.75	0.34	0.08	4.10	5.50	-	0.0	-	0.0	20	100.0	-	0.0
	Follow-Up	7	4.73	4.60	0.52	0.20	4.40	5.90	1	12.5	-	0.0	7	87.5	-	0.0
Group 3 (PP) (N=181)																
	Screening	181	4.87	4.90	0.45	0.03	3.70	6.20	-	0.0	6	3.3	172	95.0	3	1.7
	Visit 2	178	4.75	4.80	0.43	0.03	3.80	5.70	-	0.0	8	4.5	169	94.9	1	0.6
	Visit 3	2	5.05	5.05	0.49	0.35	4.70	5.40	-	0.0	-	0.0	2	100.0	-	0.0
	Visit 4	175	4.76	4.80	0.42	0.03	3.80	5.70	-	0.0	7	4.0	167	95.4	1	0.6
	Visit 5	18	4.69	4.75	0.42	0.10	4.00	5.40	-	0.0	2	11.1	16	88.9	-	0.0
	Follow-Up	15	4.76	4.60	0.52	0.13	4.00	5.70	-	0.0	1	6.7	14	93.3	-	0.0
Group 4 (M-) (N=200)																
	Screening	200	4.83	4.80	0.45	0.03	3.40	6.50	-	0.0	9	4.5	190	95.0	1	0.5

(Continued)

N: Number of subjects in the specified group
n: Number of subjects in the specified group (=Number of subjects with data available)
%: Percentage based on n
LLN: Lower limit of normal range
ULN: Upper limit of normal range

Summary of laboratory data - Haematology, descriptive statistics and count of abnormalities, Safety dataset

Erythrocytes (10E12/l)

		n	Mean	Median	Std	StdErr	Min	Max	Missing n	Missing %	Below LLN n	Below LLN %	Normal n	Normal %	Above ULN n	Above ULN %
Group 4 (M-) (N=200)																
	Visit 2	200	4.70	4.70	0.44	0.03	3.30	6.30	-	0.0	11	5.5	188	94.0	1	0.5
	Visit 3	17	4.53	4.70	0.38	0.09	3.70	5.00	1	5.6	2	11.1	15	83.3	-	0.0
	Visit 4	0	-	-	-	-	-	-	-	-	-	-	-	-	-	-
	Visit 5	0	-	-	-	-	-	-	-	-	-	-	-	-	-	-
	Follow-Up	6	4.62	4.70	0.40	0.16	4.00	5.10	1	14.3	1	14.3	5	71.4	-	0.0

N: Number of subjects in the specified group
n: Number of subjects in the specified group (=Number of subjects with data available)
%: Percentage based on n
LLN: Lower limit of normal range
ULN: Upper limit of normal range

Summary of laboratory data - Haematology, descriptive statistics and count of abnormalities, Safety dataset

Hemoglobin (mmol/l)

		n	Mean	Median	Std	StdErr	Min	Max	Missing		Below LLN		Normal		Above ULN	
									n	%	n	%	n	%	n	%
Group 1 (MM) (N=183)																
	Screening	183	9.17	9.19	0.88	0.06	6.58	11.55	-	0.0	2	1.1	177	96.7	4	2.2
	Visit 2	180	9.01	9.00	0.84	0.06	7.20	11.05	-	0.0	5	2.8	174	96.7	1	0.6
	Visit 3	2	7.73	7.73	0.75	0.53	7.20	8.26	-	0.0	1	50.0	1	50.0	-	0.0
	Visit 4	176	8.98	8.88	0.87	0.07	6.58	10.99	-	0.0	5	2.8	170	96.6	1	0.6
	Visit 5	20	8.79	8.69	1.00	0.22	6.40	10.37	1	4.8	2	9.5	18	85.7	-	0.0
	Follow-Up	11	8.94	8.69	0.93	0.28	7.76	10.62	-	0.0	1	9.1	10	90.9	-	0.0
Group 2 (MP) (N=181)																
	Screening	181	9.05	9.00	0.86	0.06	7.14	11.12	-	0.0	4	2.2	174	96.1	3	1.7
	Visit 2	178	8.81	8.82	0.80	0.06	6.40	10.93	1	0.6	7	3.9	171	95.5	-	0.0
	Visit 3	0	-	-	-	-	-	-	-	-	-	-	-	-	-	-
	Visit 4	174	8.85	8.88	0.81	0.06	6.52	10.49	-	0.0	5	2.9	168	96.6	1	0.6
	Visit 5	20	8.43	8.45	0.75	0.17	7.08	10.00	-	0.0	2	10.0	18	90.0	-	0.0
	Follow-Up	7	8.69	8.38	1.29	0.49	7.70	11.49	1	12.5	-	0.0	6	75.0	1	12.5
Group 3 (PP) (N=181)																
	Screening	181	8.94	9.00	0.90	0.07	6.64	11.05	-	0.0	11	6.1	169	93.4	1	0.6
	Visit 2	178	8.73	8.76	0.88	0.07	6.52	10.56	-	0.0	14	7.9	164	92.1	-	0.0
	Visit 3	2	9.29	9.29	0.49	0.35	8.94	9.63	-	0.0	-	0.0	2	100.0	-	0.0
	Visit 4	175	8.76	8.69	0.84	0.06	6.02	10.43	-	0.0	9	5.1	165	94.3	1	0.6
	Visit 5	18	8.54	8.32	0.95	0.22	6.58	10.06	-	0.0	1	5.6	17	94.4	-	0.0
	Follow-Up	15	8.89	9.07	1.14	0.29	7.20	10.87	-	0.0	3	20.0	12	80.0	-	0.0
Group 4 (M-) (N=200)																
	Screening	200	8.95	8.94	0.86	0.06	6.33	11.18	-	0.0	9	4.5	188	94.0	3	1.5

(Continued)

N: Number of subjects in the specified group
n: Number of subjects in the specified group (=Number of subjects with data available)
%: Percentage based on n
LLN: Lower limit of normal range
ULN: Upper limit of normal range

Summary of laboratory data - Haematology, descriptive statistics and count of abnormalities, Safety dataset

Hemoglobin (mmol/l)

		n	Mean	Median	Std	StdErr	Min	Max	Missing n	Missing %	Below LLN n	Below LLN %	Normal n	Normal %	Above ULN n	Above ULN %
Group 4 (M-) (N=200)																
	Visit 2	200	8.70	8.66	0.85	0.06	6.15	10.87	-	0.0	17	8.5	182	91.0	1	0.5
	Visit 3	17	8.41	8.51	0.91	0.22	6.83	10.12	1	5.6	4	22.2	13	72.2	-	0.0
	Visit 4	0	-	-	-	-	-	-	-	-	-	-	-	-	-	-
	Visit 5	0	-	-	-	-	-	-	-	-	-	-	-	-	-	-
	Follow-Up	6	8.33	8.39	0.85	0.35	7.27	9.50	1	14.3	2	28.6	4	57.1	-	0.0

N: Number of subjects in the specified group
n: Number of subjects in the specified group (=Number of subjects with data available)
%: Percentage based on n
LLN: Lower limit of normal range
ULN: Upper limit of normal range

Summary of laboratory data - Haematology, descriptive statistics and count of abnormalities, Safety dataset

Leukocytes (10E9/l)

		n	Mean	Median	Std	StdErr	Min	Max	Missing		Below LLN		Normal		Above ULN	
									n	%	n	%	n	%	n	%
Group 1 (MM) (N=183)																
	Screening	183	6.23	5.90	1.67	0.12	2.70	11.00	-	0.0	7	3.8	176	96.2	-	0.0
	Visit 2	180	6.52	6.40	1.71	0.13	3.10	14.50	-	0.0	7	3.9	171	95.0	2	1.1
	Visit 3	2	5.75	5.75	1.06	0.75	5.00	6.50	-	0.0	-	0.0	2	100.0	-	0.0
	Visit 4	176	6.62	6.40	1.67	0.13	2.70	12.40	-	0.0	4	2.3	169	96.0	3	1.7
	Visit 5	20	6.94	6.70	1.65	0.37	4.20	10.30	1	4.8	-	0.0	20	95.2	-	0.0
	Follow-Up	11	7.31	6.80	2.41	0.73	4.00	11.20	-	0.0	-	0.0	10	90.9	1	9.1
Group 2 (MP) (N=181)																
	Screening	181	6.23	5.90	1.63	0.12	3.00	11.00	-	0.0	10	5.5	171	94.5	-	0.0
	Visit 2	178	6.66	6.30	1.76	0.13	3.30	14.20	1	0.6	7	3.9	168	93.9	3	1.7
	Visit 3	0	-	-	-	-	-	-	-	-	-	-	-	-	-	-
	Visit 4	174	6.62	6.40	1.64	0.12	2.90	12.40	-	0.0	5	2.9	164	94.3	5	2.9
	Visit 5	20	6.52	6.40	1.35	0.30	4.10	8.80	-	0.0	-	0.0	20	100.0	-	0.0
	Follow-Up	7	8.26	7.90	2.12	0.80	5.80	11.30	1	12.5	-	0.0	6	75.0	1	12.5
Group 3 (PP) (N=181)																
	Screening	181	6.35	6.00	1.66	0.12	3.50	11.80	-	0.0	7	3.9	173	95.6	1	0.6
	Visit 2	178	6.63	6.40	1.72	0.13	2.80	15.40	-	0.0	3	1.7	170	95.5	5	2.8
	Visit 3	2	7.25	7.25	2.76	1.95	5.30	9.20	-	0.0	-	0.0	2	100.0	-	0.0
	Visit 4	175	6.70	6.40	1.80	0.14	3.50	13.90	-	0.0	5	2.9	164	93.7	6	3.4
	Visit 5	18	6.93	6.40	2.39	0.56	3.90	13.90	-	0.0	1	5.6	16	88.9	1	5.6
	Follow-Up	15	7.07	6.30	2.49	0.64	3.80	11.90	-	0.0	1	6.7	12	80.0	2	13.3
Group 4 (M-) (N=200)																
	Screening	200	5.98	5.75	1.47	0.10	3.20	9.80	-	0.0	14	7.0	186	93.0	-	0.0

(Continued)

N: Number of subjects in the specified group
n: Number of subjects in the specified group (=Number of subjects with data available)
%: Percentage based on n
LLN: Lower limit of normal range
ULN: Upper limit of normal range

Summary of laboratory data - Haematology, descriptive statistics and count of abnormalities, Safety dataset

Leukocytes (10E9/l)

		n	Mean	Median	Std	StdErr	Min	Max	Missing		Below LLN		Normal		Above ULN	
									n	%	n	%	n	%	n	%
Group 4 (M-) (N=200)																
	Visit 2	200	6.53	6.40	1.57	0.11	3.10	11.60	-	0.0	5	2.5	193	96.5	2	1.0
	Visit 3	17	6.48	6.20	1.62	0.39	3.80	9.80	1	5.6	1	5.6	16	88.9	-	0.0
	Visit 4	0	-	-	-	-	-	-	-	-	-	-	-	-	-	-
	Visit 5	0	-	-	-	-	-	-	-	-	-	-	-	-	-	-
	Follow-Up	6	6.18	5.90	1.11	0.45	4.80	7.70	1	14.3	-	0.0	6	85.7	-	0.0

N: Number of subjects in the specified group
n: Number of subjects in the specified group (=Number of subjects with data available)
%: Percentage based on n
LLN: Lower limit of normal range
ULN: Upper limit of normal range

Summary of laboratory data - Haematology, descriptive statistics and count of abnormalities, Safety dataset

Platelets (10E9/l)

								Missing		Below LLN		Normal		Above ULN	
	n	Mean	Median	Std	StdErr	Min	Max	n	%	n	%	n	%	n	%
Group 1 (MM) (N=183)															
Screening	183	244.62	235.00	50.43	3.73	139.00	416.00	-	0.0	1	0.5	182	99.5	-	0.0
Visit 2	180	251.68	247.00	52.83	3.94	152.00	456.00	-	0.0	-	0.0	179	99.4	1	0.6
Visit 3	2	304.50	304.50	31.82	22.50	282.00	327.00	-	0.0	-	0.0	2	100.0	-	0.0
Visit 4	176	256.63	251.00	53.44	4.03	144.00	457.00	-	0.0	-	0.0	175	99.4	1	0.6
Visit 5	20	255.65	244.00	52.76	11.80	186.00	400.00	1	4.8	-	0.0	20	95.2	-	0.0
Follow-Up	11	282.09	258.00	90.66	27.34	194.00	483.00	-	0.0	-	0.0	10	90.9	1	9.1
Group 2 (MP) (N=181)															
Screening	181	255.71	248.00	54.32	4.04	149.00	409.00	-	0.0	-	0.0	181	100.0	-	0.0
Visit 2	178	262.71	255.00	58.51	4.39	148.00	450.00	1	0.6	-	0.0	178	99.4	-	0.0
Visit 3	0	-	-	-	-	-	-	-	-	-	-	-	-	-	-
Visit 4	174	265.78	263.50	57.00	4.32	152.00	409.00	-	0.0	-	0.0	174	100.0	-	0.0
Visit 5	20	290.05	292.50	69.39	15.52	191.00	471.00	-	0.0	-	0.0	19	95.0	1	5.0
Follow-Up	7	254.43	264.00	61.82	23.37	155.00	354.00	1	12.5	-	0.0	7	87.5	-	0.0
Group 3 (PP) (N=181)															
Screening	181	259.94	258.00	54.30	4.04	142.00	444.00	-	0.0	-	0.0	181	100.0	-	0.0
Visit 2	178	263.30	259.00	54.00	4.05	144.00	423.00	-	0.0	-	0.0	178	100.0	-	0.0
Visit 3	2	211.50	211.50	17.68	12.50	199.00	224.00	-	0.0	-	0.0	2	100.0	-	0.0
Visit 4	175	268.59	259.00	56.89	4.30	135.00	458.00	-	0.0	1	0.6	173	98.9	1	0.6
Visit 5	18	291.83	275.50	71.75	16.91	208.00	455.00	-	0.0	-	0.0	17	94.4	1	5.6
Follow-Up	15	285.27	268.00	64.39	16.62	204.00	390.00	-	0.0	-	0.0	15	100.0	-	0.0
Group 4 (M-) (N=200)															
Screening	200	249.00	248.00	51.87	3.67	127.00	418.00	-	0.0	1	0.5	199	99.5	-	0.0

(Continued)

N: Number of subjects in the specified group
n: Number of subjects in the specified group (=Number of subjects with data available)
%: Percentage based on n
LLN: Lower limit of normal range
ULN: Upper limit of normal range

Summary of laboratory data - Haematology, descriptive statistics and count of abnormalities, Safety dataset

Platelets (10E9/l)

		n	Mean	Median	Std	StdErr	Min	Max	Missing		Below LLN		Normal		Above ULN	
									n	%	n	%	n	%	n	%
Group 4 (M-) (N=200)																
	Visit 2	200	254.40	252.00	55.53	3.93	144.00	485.00	-	0.0	-	0.0	199	99.5	1	0.5
	Visit 3	17	272.71	271.00	54.86	13.30	200.00	432.00	1	5.6	-	0.0	17	94.4	-	0.0
	Visit 4	0	-	-	-	-	-	-	-	-	-	-	-	-	-	-
	Visit 5	0	-	-	-	-	-	-	-	-	-	-	-	-	-	-
	Follow-Up	6	246.00	249.50	43.71	17.85	176.00	306.00	1	14.3	-	0.0	6	85.7	-	0.0

N: Number of subjects in the specified group
n: Number of subjects in the specified group (=Number of subjects with data available)
%: Percentage based on n
LLN: Lower limit of normal range
ULN: Upper limit of normal range

14.3.3 Laboratory abnormalities - Urinalysis (pH, specific gravity), Safety dataset

Visit Screening
Urine: Specific gravity (g/ml)

								Missing		Below LLN		Normal		Above ULN	
	n	Mean	Median	Std	StdErr	Min	Max	n	%	n	%	n	%	n	%
Group 1 (MM) (N=183)	181	1.01	1.02	0.01	0.00	1.00	1.03	2	1.1	22	12.0	155	84.7	4	2.2
Group 2 (MP) (N=181)	180	1.01	1.01	0.01	0.00	1.00	1.03	3	1.6	32	17.5	146	79.8	2	1.1
Group 3 (PP) (N=181)	182	1.01	1.02	0.01	0.00	1.01	1.03	1	0.5	31	16.9	150	82.0	1	0.5
Group 4 (M-) (N=200)	200	1.01	1.02	0.01	0.00	1.00	1.03	2	1.0	22	10.9	172	85.1	6	3.0

N: Number of subjects in the specified group
n: Number of subjects in the specified group (=Number of subjects with data available)
%: Percentage based on n
LLN: Lower limit of normal range
ULN: Upper limit of normal range

Laboratory abnormalities - Urinalysis (pH, specific gravity), Safety dataset

Visit Screening
Urine: pH

	n	Mean	Median	Std	StdErr	Min	Max	Missing		Below LLN		Normal		Above ULN	
								n	%	n	%	n	%	n	%
Group 1 (MM) (N=183)	181	6.35	6.50	0.96	0.07	5.00	9.00	2	1.1	-	0.0	158	86.3	23	12.6
Group 2 (MP) (N=181)	180	6.36	6.50	0.92	0.07	5.00	8.00	3	1.6	-	0.0	163	89.1	17	9.3
Group 3 (PP) (N=181)	182	6.31	6.00	0.98	0.07	5.00	9.00	1	0.5	-	0.0	162	88.5	20	10.9
Group 4 (M-) (N=200)	200	6.33	6.50	0.88	0.06	5.00	9.00	2	1.0	-	0.0	185	91.6	15	7.4

N: Number of subjects in the specified group
n: Number of subjects in the specified group (=Number of subjects with data available)
%: Percentage based on n
LLN: Lower limit of normal range
ULN: Upper limit of normal range

14.3.4 Laboratory abnormalities - Urinalysis (qualitative result parameters), Safety dataset

Parameter Urine: Bilirubin

		Group							
		Group 1 (MM) (N=183)		Group 2 (MP) (N=181)		Group 3 (PP) (N=181)		Group 4 (M-) (N=200)	
		n	%	n	%	n	%	n	%
Screening									
	1 mg/dl	2	1.1	5	2.7	5	2.7	7	3.5
	10 /µl	-	0.0	-	0.0	-	0.0	-	0.0
	100 /µl	-	0.0	-	0.0	-	0.0	-	0.0
	15 mg/dl	-	0.0	-	0.0	-	0.0	-	0.0
	150 /µl	-	0.0	-	0.0	-	0.0	-	0.0
	25 /µl	-	0.0	-	0.0	-	0.0	-	0.0
	25 mg/dl	-	0.0	-	0.0	-	0.0	-	0.0
	250 /µl	-	0.0	-	0.0	-	0.0	-	0.0
	5 mg/dl	-	0.0	-	0.0	-	0.0	-	0.0
	50 /µl	-	0.0	-	0.0	-	0.0	-	0.0
	50 mg/dl	-	0.0	-	0.0	-	0.0	-	0.0
	500 /µl	-	0.0	-	0.0	-	0.0	-	0.0
	negative	179	97.8	175	95.6	177	96.7	193	95.5
	normal	-	0.0	-	0.0	-	0.0	-	0.0
	not done	2	1.1	3	1.6	1	0.5	2	1.0
	positive	-	0.0	-	0.0	-	0.0	-	0.0

N: Number of subjects in the specified group
n: Number of subjects in the specified group (=Number of subjects with data available)
%: Percentage based on n
LLN: Lower limit of normal range
ULN: Upper limit of normal range

Laboratory abnormalities - Urinalysis (qualitative result parameters), Safety dataset

Parameter Urine: Erythrocytes

		Group							
		Group 1 (MM) (N=183)		Group 2 (MP) (N=181)		Group 3 (PP) (N=181)		Group 4 (M-) (N=200)	
		n	%	n	%	n	%	n	%
Screening									
	1 mg/dl	-	0.0	-	0.0	-	0.0	-	0.0
	10 /µl	24	13.1	33	18.0	26	14.2	37	18.3
	100 /µl	-	0.0	-	0.0	-	0.0	-	0.0
	15 mg/dl	-	0.0	-	0.0	-	0.0	-	0.0
	150 /µl	3	1.6	3	1.6	3	1.6	2	1.0
	25 /µl	3	1.6	5	2.7	9	4.9	8	4.0
	25 mg/dl	-	0.0	-	0.0	-	0.0	-	0.0
	250 /µl	2	1.1	3	1.6	4	2.2	5	2.5
	5 mg/dl	-	0.0	-	0.0	-	0.0	-	0.0
	50 /µl	5	2.7	2	1.1	3	1.6	5	2.5
	50 mg/dl	-	0.0	-	0.0	-	0.0	-	0.0
	500 /µl	-	0.0	-	0.0	-	0.0	-	0.0
	negative	144	78.7	134	73.2	137	74.9	143	70.8
	normal	-	0.0	-	0.0	-	0.0	-	0.0
	not done	2	1.1	3	1.6	1	0.5	2	1.0
	positive	-	0.0	-	0.0	-	0.0	-	0.0

N: Number of subjects in the specified group
n: Number of subjects in the specified group (=Number of subjects with data available)
%: Percentage based on n
LLN: Lower limit of normal range
ULN: Upper limit of normal range

Laboratory abnormalities - Urinalysis (qualitative result parameters), Safety dataset

Parameter Urine: Glucose

		Group							
		Group 1 (MM) (N=183)		Group 2 (MP) (N=181)		Group 3 (PP) (N=181)		Group 4 (M-) (N=200)	
		n	%	n	%	n	%	n	%
Screening									
	1 mg/dl	-	0.0	-	0.0	-	0.0	-	0.0
	10 /µl	-	0.0	-	0.0	-	0.0	-	0.0
	100 /µl	-	0.0	-	0.0	-	0.0	-	0.0
	15 mg/dl	-	0.0	-	0.0	-	0.0	-	0.0
	150 /µl	-	0.0	-	0.0	-	0.0	-	0.0
	25 /µl	-	0.0	-	0.0	-	0.0	-	0.0
	25 mg/dl	-	0.0	-	0.0	-	0.0	-	0.0
	250 /µl	-	0.0	-	0.0	-	0.0	-	0.0
	5 mg/dl	-	0.0	-	0.0	-	0.0	-	0.0
	50 /µl	-	0.0	-	0.0	-	0.0	-	0.0
	50 mg/dl	-	0.0	-	0.0	-	0.0	-	0.0
	500 /µl	-	0.0	-	0.0	-	0.0	-	0.0
	negative	-	0.0	-	0.0	-	0.0	-	0.0
	normal	181	98.9	180	98.4	182	99.5	200	99.0
	not done	2	1.1	3	1.6	1	0.5	2	1.0
	positive	-	0.0	-	0.0	-	0.0	-	0.0

N: Number of subjects in the specified group
n: Number of subjects in the specified group (=Number of subjects with data available)
%: Percentage based on n
LLN: Lower limit of normal range
ULN: Upper limit of normal range

Laboratory abnormalities - Urinalysis (qualitative result parameters), Safety dataset

Parameter Urine: Ketone

	Group							
	Group 1 (MM) (N=183)		Group 2 (MP) (N=181)		Group 3 (PP) (N=181)		Group 4 (M-) (N=200)	
	n	%	n	%	n	%	n	%
Screening								
1 mg/dl	-	0.0	-	0.0	-	0.0	-	0.0
10 /µl	-	0.0	-	0.0	-	0.0	-	0.0
100 /µl	-	0.0	-	0.0	-	0.0	-	0.0
15 mg/dl	2	1.1	4	2.2	1	0.5	2	1.0
150 /µl	-	0.0	-	0.0	-	0.0	-	0.0
25 /µl	-	0.0	-	0.0	-	0.0	-	0.0
25 mg/dl	-	0.0	-	0.0	-	0.0	-	0.0
250 /µl	-	0.0	-	0.0	-	0.0	-	0.0
5 mg/dl	4	2.2	3	1.6	5	2.7	2	1.0
50 /µl	-	0.0	-	0.0	-	0.0	-	0.0
50 mg/dl	-	0.0	2	1.1	3	1.6	3	1.5
500 /µl	-	0.0	-	0.0	-	0.0	-	0.0
negative	175	95.6	171	93.4	173	94.5	193	95.5
normal	-	0.0	-	0.0	-	0.0	-	0.0
not done	2	1.1	3	1.6	1	0.5	2	1.0
positive	-	0.0	-	0.0	-	0.0	-	0.0

N: Number of subjects in the specified group
n: Number of subjects in the specified group (=Number of subjects with data available)
%: Percentage based on n
LLN: Lower limit of normal range
ULN: Upper limit of normal range

Laboratory abnormalities - Urinalysis (qualitative result parameters), Safety dataset

Parameter Urine: Leukocytes

		Group							
		Group 1 (MM) (N=183)		Group 2 (MP) (N=181)		Group 3 (PP) (N=181)		Group 4 (M-) (N=200)	
		n	%	n	%	n	%	n	%
Screening									
	1 mg/dl	-	0.0	-	0.0	-	0.0	-	0.0
	10 /µl	-	0.0	-	0.0	-	0.0	-	0.0
	100 /µl	6	3.3	11	6.0	16	8.7	3	1.5
	15 mg/dl	-	0.0	-	0.0	-	0.0	-	0.0
	150 /µl	-	0.0	-	0.0	-	0.0	-	0.0
	25 /µl	28	15.3	42	23.0	34	18.6	38	18.8
	25 mg/dl	-	0.0	-	0.0	-	0.0	-	0.0
	250 /µl	-	0.0	-	0.0	-	0.0	-	0.0
	5 mg/dl	-	0.0	-	0.0	-	0.0	-	0.0
	50 /µl	-	0.0	-	0.0	-	0.0	-	0.0
	50 mg/dl	-	0.0	-	0.0	-	0.0	-	0.0
	500 /µl	7	3.8	9	4.9	6	3.3	10	5.0
	negative	140	76.5	118	64.5	126	68.9	149	73.8
	normal	-	0.0	-	0.0	-	0.0	-	0.0
	not done	2	1.1	3	1.6	1	0.5	2	1.0
	positive	-	0.0	-	0.0	-	0.0	-	0.0

N: Number of subjects in the specified group
n: Number of subjects in the specified group (=Number of subjects with data available)
%: Percentage based on n
LLN: Lower limit of normal range
ULN: Upper limit of normal range

Laboratory abnormalities - Urinalysis (qualitative result parameters), Safety dataset

Parameter Urine: Nitrite

		Group						
		Group 1 (MM) (N=183)		Group 2 (MP) (N=181)		Group 3 (PP) (N=181)		Group 4 (M-) (N=200)
		n	%	n	%	n	%	n	%
Screening									
	1 mg/dl	-	0.0	-	0.0	-	0.0	-	0.0
	10 /µl	-	0.0	-	0.0	-	0.0	-	0.0
	100 /µl	-	0.0	-	0.0	-	0.0	-	0.0
	15 mg/dl	-	0.0	-	0.0	-	0.0	-	0.0
	150 /µl	-	0.0	-	0.0	-	0.0	-	0.0
	25 /µl	-	0.0	-	0.0	-	0.0	-	0.0
	25 mg/dl	-	0.0	-	0.0	-	0.0	-	0.0
	250 /µl	-	0.0	-	0.0	-	0.0	-	0.0
	5 mg/dl	-	0.0	-	0.0	-	0.0	-	0.0
	50 /µl	-	0.0	-	0.0	-	0.0	-	0.0
	50 mg/dl	-	0.0	-	0.0	-	0.0	-	0.0
	500 /µl	-	0.0	-	0.0	-	0.0	-	0.0
	negative	180	98.4	176	96.2	181	98.9	196	97.0
	normal	-	0.0	-	0.0	-	0.0	-	0.0
	not done	2	1.1	3	1.6	1	0.5	2	1.0
	positive	1	0.5	4	2.2	1	0.5	4	2.0

N: Number of subjects in the specified group
n: Number of subjects in the specified group (=Number of subjects with data available)
%: Percentage based on n
LLN: Lower limit of normal range
ULN: Upper limit of normal range

Laboratory abnormalities - Urinalysis (qualitative result parameters), Safety dataset

Parameter Urine: Protein

	Group							
	Group 1 (MM) (N=183)		Group 2 (MP) (N=181)		Group 3 (PP) (N=181)		Group 4 (M-) (N=200)	
	n	%	n	%	n	%	n	%
Screening								
1 mg/dl	-	0.0	-	0.0	-	0.0	-	0.0
10 /µl	-	0.0	-	0.0	-	0.0	-	0.0
100 /µl	-	0.0	-	0.0	-	0.0	-	0.0
15 mg/dl	-	0.0	-	0.0	-	0.0	-	0.0
150 /µl	-	0.0	-	0.0	-	0.0	-	0.0
25 /µl	-	0.0	-	0.0	-	0.0	-	0.0
25 mg/dl	21	11.5	33	18.0	24	13.1	14	6.9
250 /µl	-	0.0	-	0.0	-	0.0	-	0.0
5 mg/dl	-	0.0	-	0.0	-	0.0	-	0.0
50 /µl	-	0.0	-	0.0	-	0.0	-	0.0
50 mg/dl	-	0.0	-	0.0	-	0.0	-	0.0
500 /µl	-	0.0	-	0.0	-	0.0	-	0.0
negative	160	87.4	147	80.3	158	86.3	186	92.1
normal	-	0.0	-	0.0	-	0.0	-	0.0
not done	2	1.1	3	1.6	1	0.5	2	1.0
positive	-	0.0	-	0.0	-	0.0	-	0.0

N: Number of subjects in the specified group
n: Number of subjects in the specified group (=Number of subjects with data available)
%: Percentage based on n
LLN: Lower limit of normal range
ULN: Upper limit of normal range

Laboratory abnormalities - Urinalysis (qualitative result parameters), Safety dataset

Parameter Urine: Urobilinogen

		Group						
		Group 1 (MM) (N=183)		Group 2 (MP) (N=181)		Group 3 (PP) (N=181)		Group 4 (M-) (N=200)
		n	%	n	%	n	%	n	%
Screening									
	1 mg/dl	-	0.0	1	0.5	1	0.5	1	0.5
	10 /µl	-	0.0	-	0.0	-	0.0	-	0.0
	100 /µl	-	0.0	-	0.0	-	0.0	-	0.0
	15 mg/dl	-	0.0	-	0.0	-	0.0	-	0.0
	150 /µl	-	0.0	-	0.0	-	0.0	-	0.0
	25 /µl	-	0.0	-	0.0	-	0.0	-	0.0
	25 mg/dl	-	0.0	-	0.0	-	0.0	-	0.0
	250 /µl	-	0.0	-	0.0	-	0.0	-	0.0
	5 mg/dl	-	0.0	-	0.0	-	0.0	-	0.0
	50 /µl	-	0.0	-	0.0	-	0.0	-	0.0
	50 mg/dl	-	0.0	-	0.0	-	0.0	-	0.0
	500 /µl	-	0.0	-	0.0	-	0.0	-	0.0
	negative	-	0.0	-	0.0	-	0.0	-	0.0
	normal	181	98.9	179	97.8	181	98.9	199	98.5
	not done	2	1.1	3	1.6	1	0.5	2	1.0
	positive	-	0.0	-	0.0	-	0.0	-	0.0

N: Number of subjects in the specified group
n: Number of subjects in the specified group (=Number of subjects with data available)
%: Percentage based on n
LLN: Lower limit of normal range
ULN: Upper limit of normal range

14.3.5 Laboratory abnormalities - Urine pregnancy test (qualitative result parameters), Safety dataset

	Group							
	Group 1 (MM) (N=183)		Group 2 (MP) (N=181)		Group 3 (PP) (N=181)		Group 4 (M-) (N=200)	
	n	%	n	%	n	%	n	%
Visit 1								
negative	96	100.0	109	100.0	107	100.0	89	100.0
Visit 3								
negative	95	100.0	104	100.0	102	100.0	89	100.0
Visit 5								
negative	92	100.0	105	100.0	104	100.0	-	-

N: Number of subjects in the specified group
n: Number of subjects in the specified group (=Number of subjects with data available)
%: Percentage based on n
LLN: Lower limit of normal range
ULN: Upper limit of normal range

14.3.6 Laboratory abnormalities, shift tables - Hematology, Safety dataset

14.3.6.1 Screening versus Visit 2

Basophils (%)

Screening	Visit 2					
	Below LLN		Normal		Above ULN	
	n	%	n	%	n	%
Group 1 (MM) (N=183)						
Below LLN	-	0.0	-	0.0	-	0.0
Normal	-	0.0	180	99.4	-	0.0
Above ULN	-	0.0	1	0.6	-	0.0
Group 2 (MP) (N=181)						
Below LLN	-	0.0	-	0.0	-	0.0
Normal	-	0.0	177	99.4	1	0.6
Above ULN	-	0.0	-	0.0	-	0.0
Group 3 (PP) (N=181)						
Below LLN	-	0.0	-	0.0	-	0.0
Normal	-	0.0	178	99.4	-	0.0
Above ULN	-	0.0	1	0.6	-	0.0
Group 4 (M-) (N=200)						
Below LLN	-	0.0	-	0.0	-	0.0
Normal	-	0.0	198	99.0	1	0.5
Above ULN	-	0.0	-	0.0	1	0.5
Total						
Below LLN	-	0.0	-	0.0	-	0.0
Normal	-	0.0	733	99.3	2	0.3
Above ULN	-	0.0	2	0.3	1	0.1

Laboratory abnormalities, shift tables - Hematology, Safety dataset
Screening versus Visit 2

Eosinophils (%)

Screening		Visit 2					
		Below LLN		Normal		Above ULN	
		n	%	n	%	n	%
Group 1 (MM) (N=183)							
	Below LLN	-	0.0	-	0.0	-	0.0
	Normal	-	0.0	173	95.6	1	0.6
	Above ULN	-	0.0	6	3.3	1	0.6
Group 2 (MP) (N=181)							
	Below LLN	-	0.0	-	0.0	-	0.0
	Normal	-	0.0	169	94.9	4	2.2
	Above ULN	-	0.0	3	1.7	2	1.1
Group 3 (PP) (N=181)							
	Below LLN	-	0.0	-	0.0	-	0.0
	Normal	-	0.0	168	93.9	3	1.7
	Above ULN	-	0.0	4	2.2	4	2.2
Group 4 (M-) (N=200)							
	Below LLN	-	0.0	-	0.0	-	0.0
	Normal	-	0.0	192	96.0	-	0.0
	Above ULN	-	0.0	2	1.0	6	3.0
Total							
	Below LLN	-	0.0	-	0.0	-	0.0
	Normal	-	0.0	702	95.1	8	1.1
	Above ULN	-	0.0	15	2.0	13	1.8

Laboratory abnormalities, shift tables - Hematology, Safety dataset
Screening versus Visit 2

Erythrocytes (10E12/l)

Screening		Visit 2				
	Below LLN		Normal		Above ULN	
	n	%	n	%	n	%
Group 1 (MM) (N=183)						
Below LLN	-	0.0	1	0.6	-	0.0
Normal	4	2.2	175	96.7	-	0.0
Above ULN	-	0.0	1	0.6	-	0.0
Group 2 (MP) (N=181)						
Below LLN	1	0.6	1	0.6	-	0.0
Normal	3	1.7	173	97.2	-	0.0
Above ULN	-	0.0	-	0.0	-	0.0
Group 3 (PP) (N=181)						
Below LLN	4	2.2	3	1.7	-	0.0
Normal	4	2.2	165	92.2	-	0.0
Above ULN	-	0.0	2	1.1	1	0.6
Group 4 (M-) (N=200)						
Below LLN	5	2.5	3	1.5	-	0.0
Normal	6	3.0	185	92.5	-	0.0
Above ULN	-	0.0	-	0.0	1	0.5
Total						
Below LLN	10	1.4	8	1.1	-	0.0
Normal	17	2.3	698	94.6	-	0.0
Above ULN	-	0.0	3	0.4	2	0.3

Laboratory abnormalities, shift tables - Hematology, Safety dataset
Screening versus Visit 2

Hematocrit (%)

Screening		Visit 2					
		Below LLN		Normal		Above ULN	
		n	%	n	%	n	%
Group 1 (MM) (N=183)							
	Below LLN	1	0.6	1	0.6	-	0.0
	Normal	5	2.8	173	95.6	-	0.0
	Above ULN	-	0.0	1	0.6	-	0.0
Group 2 (MP) (N=181)							
	Below LLN	2	1.1	1	0.6	-	0.0
	Normal	5	2.8	169	94.9	-	0.0
	Above ULN	-	0.0	1	0.6	-	0.0
Group 3 (PP) (N=181)							
	Below LLN	2	1.1	4	2.2	-	0.0
	Normal	10	5.6	162	90.5	-	0.0
	Above ULN	-	0.0	1	0.6	-	0.0
Group 4 (M-) (N=200)							
	Below LLN	4	2.0	-	0.0	-	0.0
	Normal	7	3.5	186	93.0	1	0.5
	Above ULN	-	0.0	2	1.0	-	0.0
Total							
	Below LLN	9	1.2	6	0.8	-	0.0
	Normal	27	3.7	690	93.5	1	0.1
	Above ULN	-	0.0	5	0.7	-	0.0

Laboratory abnormalities, shift tables - Hematology, Safety dataset
Screening versus Visit 2

Hemoglobin (mmol/l)

Screening	Visit 2					
	Below LLN		Normal		Above ULN	
	n	%	n	%	n	%
Group 1 (MM) (N=183)						
Below LLN	2	1.1	-	0.0	-	0.0
Normal	3	1.7	171	94.5	1	0.6
Above ULN	-	0.0	4	2.2	-	0.0
Group 2 (MP) (N=181)						
Below LLN	2	1.1	2	1.1	-	0.0
Normal	5	2.8	166	93.3	-	0.0
Above ULN	-	0.0	3	1.7	-	0.0
Group 3 (PP) (N=181)						
Below LLN	4	2.2	6	3.4	-	0.0
Normal	10	5.6	158	88.3	-	0.0
Above ULN	-	0.0	1	0.6	-	0.0
Group 4 (M-) (N=200)						
Below LLN	6	3.0	2	1.0	-	0.0
Normal	10	5.0	178	89.0	1	0.5
Above ULN	1	0.5	2	1.0	-	0.0
Total						
Below LLN	14	1.9	10	1.4	-	0.0
Normal	28	3.8	673	91.2	2	0.3
Above ULN	1	0.1	10	1.4	-	0.0

Laboratory abnormalities, shift tables - Hematology, Safety dataset
Screening versus Visit 2

Leukocytes (10E9/l)

Screening		Visit 2					
		Below LLN		Normal		Above ULN	
		n	%	n	%	n	%
Group 1 (MM) (N=183)							
	Below LLN	2	1.1	5	2.8	-	0.0
	Normal	5	2.8	167	92.3	2	1.1
	Above ULN	-	0.0	-	0.0	-	0.0
Group 2 (MP) (N=181)							
	Below LLN	2	1.1	7	3.9	-	0.0
	Normal	5	2.8	160	89.9	3	1.7
	Above ULN	-	0.0	1	0.6	-	0.0
Group 3 (PP) (N=181)							
	Below LLN	1	0.6	7	3.9	-	0.0
	Normal	2	1.1	164	91.6	4	2.2
	Above ULN	-	0.0	-	0.0	1	0.6
Group 4 (M-) (N=200)							
	Below LLN	3	1.5	11	5.5	-	0.0
	Normal	2	1.0	182	91.0	2	1.0
	Above ULN	-	0.0	-	0.0	-	0.0
Total							
	Below LLN	8	1.1	30	4.1	-	0.0
	Normal	14	1.9	673	91.2	11	1.5
	Above ULN	-	0.0	1	0.1	1	0.1

Laboratory abnormalities, shift tables - Hematology, Safety dataset
Screening versus Visit 2

Lymphocytes (%)

Screening		Visit 2					
		Below LLN		Normal		Above ULN	
		n	%	n	%	n	%
Group 1 (MM) (N=183)							
	Below LLN	1	0.6	13	7.2	-	0.0
	Normal	3	1.7	163	90.1	1	0.6
	Above ULN	-	0.0	-	0.0	-	0.0
Group 2 (MP) (N=181)							
	Below LLN	2	1.1	11	6.2	1	0.6
	Normal	9	5.1	154	86.5	-	0.0
	Above ULN	-	0.0	1	0.6	-	0.0
Group 3 (PP) (N=181)							
	Below LLN	1	0.6	7	3.9	-	0.0
	Normal	5	2.8	166	92.7	-	0.0
	Above ULN	-	0.0	-	0.0	-	0.0
Group 4 (M-) (N=200)							
	Below LLN	1	0.5	8	4.0	-	0.0
	Normal	7	3.5	183	91.5	-	0.0
	Above ULN	-	0.0	-	0.0	1	0.5
Total							
	Below LLN	5	0.7	39	5.3	1	0.1
	Normal	24	3.3	666	90.2	1	0.1
	Above ULN	-	0.0	1	0.1	1	0.1

Laboratory abnormalities, shift tables - Hematology, Safety dataset
Screening versus Visit 2

MCH (fmol)

Screening		Visit 2					
		Below LLN		Normal		Above ULN	
		n	%	n	%	n	%
Group 1 (MM) (N=183)							
	Below LLN	11	6.1	1	0.6	-	0.0
	Normal	4	2.2	163	90.1	-	0.0
	Above ULN	-	0.0	1	0.6	1	0.6
Group 2 (MP) (N=181)							
	Below LLN	16	9.0	5	2.8	-	0.0
	Normal	5	2.8	151	84.8	-	0.0
	Above ULN	-	0.0	-	0.0	1	0.6
Group 3 (PP) (N=181)							
	Below LLN	17	9.5	3	1.7	-	0.0
	Normal	2	1.1	156	87.2	-	0.0
	Above ULN	-	0.0	1	0.6	-	0.0
Group 4 (M-) (N=200)							
	Below LLN	16	8.0	3	1.5	-	0.0
	Normal	4	2.0	174	87.0	-	0.0
	Above ULN	-	0.0	1	0.5	2	1.0
Total							
	Below LLN	60	8.1	12	1.6	-	0.0
	Normal	15	2.0	644	87.3	-	0.0
	Above ULN	-	0.0	3	0.4	4	0.5

Laboratory abnormalities, shift tables - Hematology, Safety dataset
Screening versus Visit 2

MCV (fl)

Screening		Visit 2				
	Below LLN		Normal		Above ULN	
	n	%	n	%	n	%
Group 1 (MM) (N=183)						
Below LLN	4	2.2	-	0.0	-	0.0
Normal	-	0.0	175	96.7	2	1.1
Above ULN	-	0.0	-	0.0	-	0.0
Group 2 (MP) (N=181)						
Below LLN	3	1.7	-	0.0	-	0.0
Normal	1	0.6	173	97.2	-	0.0
Above ULN	-	0.0	-	0.0	1	0.6
Group 3 (PP) (N=181)						
Below LLN	4	2.2	1	0.6	-	0.0
Normal	3	1.7	171	95.5	-	0.0
Above ULN	-	0.0	-	0.0	-	0.0
Group 4 (M-) (N=200)						
Below LLN	9	4.5	-	0.0	-	0.0
Normal	-	0.0	189	94.5	-	0.0
Above ULN	-	0.0	-	0.0	2	1.0
Total						
Below LLN	20	2.7	1	0.1	-	0.0
Normal	4	0.5	708	95.9	2	0.3
Above ULN	-	0.0	-	0.0	3	0.4

Laboratory abnormalities, shift tables - Hematology, Safety dataset
Screening versus Visit 2

Monocytes (%)

Screening		Visit 2					
		Below LLN		Normal		Above ULN	
		n	%	n	%	n	%
Group 1 (MM) (N=183)							
	Below LLN	-	0.0	-	0.0	-	0.0
	Normal	-	0.0	172	95.0	6	3.3
	Above ULN	-	0.0	1	0.6	2	1.1
Group 2 (MP) (N=181)							
	Below LLN	-	0.0	-	0.0	-	0.0
	Normal	-	0.0	171	96.1	3	1.7
	Above ULN	-	0.0	3	1.7	1	0.6
Group 3 (PP) (N=181)							
	Below LLN	-	0.0	-	0.0	-	0.0
	Normal	-	0.0	169	94.4	1	0.6
	Above ULN	-	0.0	6	3.4	3	1.7
Group 4 (M-) (N=200)							
	Below LLN	-	0.0	-	0.0	-	0.0
	Normal	-	0.0	198	99.0	-	0.0
	Above ULN	-	0.0	2	1.0	-	0.0
Total							
	Below LLN	-	0.0	-	0.0	-	0.0
	Normal	-	0.0	710	96.2	10	1.4
	Above ULN	-	0.0	12	1.6	6	0.8

Laboratory abnormalities, shift tables - Hematology, Safety dataset
Screening versus Visit 2

Neutrophils, total (%)

Screening		Visit 2					
		Below LLN		Normal		Above ULN	
		n	%	n	%	n	%
Group 1 (MM) (N=183)							
	Below LLN	1	0.6	4	2.2	-	0.0
	Normal	6	3.3	169	93.4	-	0.0
	Above ULN	-	0.0	1	0.6	-	0.0
Group 2 (MP) (N=181)							
	Below LLN	1	0.6	4	2.2	-	0.0
	Normal	3	1.7	170	95.5	-	0.0
	Above ULN	-	0.0	-	0.0	-	0.0
Group 3 (PP) (N=181)							
	Below LLN	1	0.6	5	2.8	-	0.0
	Normal	2	1.1	170	95.0	1	0.6
	Above ULN	-	0.0	-	0.0	-	0.0
Group 4 (M-) (N=200)							
	Below LLN	2	1.0	3	1.5	-	0.0
	Normal	4	2.0	190	95.0	-	0.0
	Above ULN	-	0.0	1	0.5	-	0.0
Total							
	Below LLN	5	0.7	16	2.2	-	0.0
	Normal	15	2.0	699	94.7	1	0.1
	Above ULN	-	0.0	2	0.3	-	0.0

Laboratory abnormalities, shift tables - Hematology, Safety dataset
Screening versus Visit 2

Platelets (10E9/l)

Screening	Visit 2					
	Below LLN		Normal		Above ULN	
	n	%	n	%	n	%
Group 1 (MM) (N=183)						
Below LLN	-	0.0	1	0.6	-	0.0
Normal	-	0.0	179	98.9	1	0.6
Above ULN	-	0.0	-	0.0	-	0.0
Group 2 (MP) (N=181)						
Below LLN	-	0.0	-	0.0	-	0.0
Normal	-	0.0	178	100.0	-	0.0
Above ULN	-	0.0	-	0.0	-	0.0
Group 3 (PP) (N=181)						
Below LLN	-	0.0	-	0.0	-	0.0
Normal	-	0.0	179	100.0	-	0.0
Above ULN	-	0.0	-	0.0	-	0.0
Group 4 (M-) (N=200)						
Below LLN	-	0.0	1	0.5	-	0.0
Normal	-	0.0	198	99.0	-	0.0
Above ULN	-	0.0	-	0.0	1	0.5
Total						
Below LLN	-	0.0	2	0.3	-	0.0
Normal	-	0.0	734	99.5	1	0.1
Above ULN	-	0.0	-	0.0	1	0.1

Laboratory abnormalities, shift tables - Hematology, Safety dataset

14.3.6.2 Screening versus Visit 4

Basophils (%)

Screening	Visit 4					
	Below LLN		Normal		Above ULN	
	n	%	n	%	n	%
Group 1 (MM) (N=183)						
Below LLN	-	0.0	-	0.0	-	0.0
Normal	-	0.0	174	98.9	1	0.6
Above ULN	-	0.0	1	0.6	-	0.0
Group 2 (MP) (N=181)						
Below LLN	-	0.0	-	0.0	-	0.0
Normal	-	0.0	174	100.0	-	0.0
Above ULN	-	0.0	-	0.0	-	0.0
Group 3 (PP) (N=181)						
Below LLN	-	0.0	-	0.0	-	0.0
Normal	-	0.0	174	99.4	-	0.0
Above ULN	-	0.0	1	0.6	-	0.0
Total						
Below LLN	-	0.0	-	0.0	-	0.0
Normal	-	0.0	522	99.4	1	0.2
Above ULN	-	0.0	2	0.4	-	0.0

Laboratory abnormalities, shift tables - Hematology, Safety dataset
Screening versus Visit 4

Eosinophils (%)

Screening	Visit 4					
	Below LLN		Normal		Above ULN	
	n	%	n	%	n	%
Group 1 (MM) (N=183)						
Below LLN	-	0.0	-	0.0	-	0.0
Normal	-	0.0	167	94.9	2	1.1
Above ULN	-	0.0	5	2.8	2	1.1
Group 2 (MP) (N=181)						
Below LLN	-	0.0	-	0.0	-	0.0
Normal	-	0.0	167	96.0	2	1.1
Above ULN	-	0.0	4	2.3	1	0.6
Group 3 (PP) (N=181)						
Below LLN	-	0.0	-	0.0	-	0.0
Normal	-	0.0	160	91.4	7	4.0
Above ULN	-	0.0	8	4.6	-	0.0
Total						
Below LLN	-	0.0	-	0.0	-	0.0
Normal	-	0.0	494	94.1	11	2.1
Above ULN	-	0.0	17	3.2	3	0.6

Laboratory abnormalities, shift tables - Hematology, Safety dataset
Screening versus Visit 4

Erythrocytes (10E12/l)

Screening	Visit 4					
	Below LLN		Normal		Above ULN	
	n	%	n	%	n	%
Group 1 (MM) (N=183)						
Below LLN	-	0.0	-	0.0	-	0.0
Normal	2	1.1	173	98.3	-	0.0
Above ULN	-	0.0	1	0.6	-	0.0
Group 2 (MP) (N=181)						
Below LLN	1	0.6	1	0.6	-	0.0
Normal	3	1.7	169	97.1	-	0.0
Above ULN	-	0.0	-	0.0	-	0.0
Group 3 (PP) (N=181)						
Below LLN	2	1.1	5	2.9	-	0.0
Normal	5	2.9	160	91.4	-	0.0
Above ULN	-	0.0	2	1.1	1	0.6
Total						
Below LLN	3	0.6	6	1.1	-	0.0
Normal	10	1.9	502	95.6	-	0.0
Above ULN	-	0.0	3	0.6	1	0.2

Laboratory abnormalities, shift tables - Hematology, Safety dataset
Screening versus Visit 4

Hematocrit (%)

Screening		Visit 4					
		Below LLN		Normal		Above ULN	
		n	%	n	%	n	%
Group 1 (MM) (N=183)							
	Below LLN	1	0.6	-	0.0	-	0.0
	Normal	2	1.1	171	97.2	1	0.6
	Above ULN	-	0.0	1	0.6	-	0.0
Group 2 (MP) (N=181)							
	Below LLN	1	0.6	2	1.1	-	0.0
	Normal	3	1.7	166	95.4	1	0.6
	Above ULN	-	0.0	1	0.6	-	0.0
Group 3 (PP) (N=181)							
	Below LLN	4	2.3	2	1.1	-	0.0
	Normal	5	2.9	163	93.1	-	0.0
	Above ULN	-	0.0	-	0.0	1	0.6
Total							
	Below LLN	6	1.1	4	0.8	-	0.0
	Normal	10	1.9	500	95.2	2	0.4
	Above ULN	-	0.0	2	0.4	1	0.2

Laboratory abnormalities, shift tables - Hematology, Safety dataset
Screening versus Visit 4

Hemoglobin (mmol/l)

Screening	Visit 4					
	Below LLN		Normal		Above ULN	
	n	%	n	%	n	%
Group 1 (MM) (N=183)						
Below LLN	2	1.1	-	0.0	-	0.0
Normal	3	1.7	166	94.3	1	0.6
Above ULN	-	0.0	4	2.3	-	0.0
Group 2 (MP) (N=181)						
Below LLN	2	1.1	2	1.1	-	0.0
Normal	3	1.7	163	93.7	1	0.6
Above ULN	-	0.0	3	1.7	-	0.0
Group 3 (PP) (N=181)						
Below LLN	5	2.9	4	2.3	-	0.0
Normal	4	2.3	161	92.0	-	0.0
Above ULN	-	0.0	-	0.0	1	0.6
Total						
Below LLN	9	1.7	6	1.1	-	0.0
Normal	10	1.9	490	93.3	2	0.4
Above ULN	-	0.0	7	1.3	1	0.2

Laboratory abnormalities, shift tables - Hematology, Safety dataset
Screening versus Visit 4

Leukocytes (10E9/l)

Screening	Visit 4					
	Below LLN		Normal		Above ULN	
	n	%	n	%	n	%
Group 1 (MM) (N=183)						
Below LLN	1	0.6	6	3.4	-	0.0
Normal	3	1.7	163	92.6	3	1.7
Above ULN	-	0.0	-	0.0	-	0.0
Group 2 (MP) (N=181)						
Below LLN	2	1.1	6	3.4	-	0.0
Normal	3	1.7	157	90.2	5	2.9
Above ULN	-	0.0	1	0.6	-	0.0
Group 3 (PP) (N=181)						
Below LLN	3	1.7	4	2.3	-	0.0
Normal	2	1.1	160	91.4	5	2.9
Above ULN	-	0.0	-	0.0	1	0.6
Total						
Below LLN	6	1.1	16	3.0	-	0.0
Normal	8	1.5	480	91.4	13	2.5
Above ULN	-	0.0	1	0.2	1	0.2

Laboratory abnormalities, shift tables - Hematology, Safety dataset
Screening versus Visit 4

Lymphocytes (%)

Screening	Visit 4					
	Below LLN		Normal		Above ULN	
	n	%	n	%	n	%
Group 1 (MM) (N=183)						
Below LLN	2	1.1	12	6.8	-	0.0
Normal	5	2.8	156	88.6	-	0.0
Above ULN	-	0.0	1	0.6	-	0.0
Group 2 (MP) (N=181)						
Below LLN	3	1.7	10	5.7	-	0.0
Normal	5	2.9	154	88.5	2	1.1
Above ULN	-	0.0	-	0.0	-	0.0
Group 3 (PP) (N=181)						
Below LLN	3	1.7	5	2.9	-	0.0
Normal	9	5.1	157	89.7	1	0.6
Above ULN	-	0.0	-	0.0	-	0.0
Total						
Below LLN	8	1.5	27	5.1	-	0.0
Normal	19	3.6	467	89.0	3	0.6
Above ULN	-	0.0	1	0.2	-	0.0

Laboratory abnormalities, shift tables - Hematology, Safety dataset
Screening versus Visit 4

MCH (fmol)

Screening		Visit 4					
		Below LLN		Normal		Above ULN	
		n	%	n	%	n	%
Group 1 (MM) (N=183)							
	Below LLN	10	5.7	3	1.7	-	0.0
	Normal	3	1.7	158	89.8	-	0.0
	Above ULN	-	0.0	-	0.0	2	1.1
Group 2 (MP) (N=181)							
	Below LLN	15	8.6	5	2.9	-	0.0
	Normal	7	4.0	146	83.9	-	0.0
	Above ULN	-	0.0	-	0.0	1	0.6
Group 3 (PP) (N=181)							
	Below LLN	16	9.1	1	0.6	-	0.0
	Normal	4	2.3	153	87.4	-	0.0
	Above ULN	-	0.0	1	0.6	-	0.0
Total							
	Below LLN	41	7.8	9	1.7	-	0.0
	Normal	14	2.7	457	87.0	-	0.0
	Above ULN	-	0.0	1	0.2	3	0.6

Laboratory abnormalities, shift tables - Hematology, Safety dataset
Screening versus Visit 4

MCV (fl)

Screening		Visit 4					
		Below LLN		Normal		Above ULN	
		n	%	n	%	n	%
Group 1 (MM) (N=183)							
	Below LLN	2	1.1	2	1.1	-	0.0
	Normal	1	0.6	170	96.6	1	0.6
	Above ULN	-	0.0	-	0.0	-	0.0
Group 2 (MP) (N=181)							
	Below LLN	2	1.1	-	0.0	-	0.0
	Normal	-	0.0	170	97.7	1	0.6
	Above ULN	-	0.0	-	0.0	1	0.6
Group 3 (PP) (N=181)							
	Below LLN	3	1.7	2	1.1	-	0.0
	Normal	3	1.7	167	95.4	-	0.0
	Above ULN	-	0.0	-	0.0	-	0.0
Total							
	Below LLN	7	1.3	4	0.8	-	0.0
	Normal	4	0.8	507	96.6	2	0.4
	Above ULN	-	0.0	-	0.0	1	0.2

Laboratory abnormalities, shift tables - Hematology, Safety dataset
Screening versus Visit 4

Monocytes (%)

Screening		Visit 4					
		Below LLN		Normal		Above ULN	
		n	%	n	%	n	%
Group 1 (MM) (N=183)							
	Below LLN	-	0.0	-	0.0	-	0.0
	Normal	-	0.0	171	97.2	1	0.6
	Above ULN	-	0.0	1	0.6	3	1.7
Group 2 (MP) (N=181)							
	Below LLN	-	0.0	-	0.0	-	0.0
	Normal	-	0.0	166	95.4	4	2.3
	Above ULN	-	0.0	4	2.3	-	0.0
Group 3 (PP) (N=181)							
	Below LLN	-	0.0	-	0.0	-	0.0
	Normal	-	0.0	164	93.7	3	1.7
	Above ULN	-	0.0	7	4.0	1	0.6
Total							
	Below LLN	-	0.0	-	0.0	-	0.0
	Normal	-	0.0	501	95.4	8	1.5
	Above ULN	-	0.0	12	2.3	4	0.8

Laboratory abnormalities, shift tables - Hematology, Safety dataset
Screening versus Visit 4

Neutrophils, total (%)

Screening		Visit 4				
	Below LLN		Normal		Above ULN	
	n	%	n	%	n	%
Group 1 (MM) (N=183)						
Below LLN	-	0.0	6	3.4	-	0.0
Normal	1	0.6	168	95.5	-	0.0
Above ULN	-	0.0	1	0.6	-	0.0
Group 2 (MP) (N=181)						
Below LLN	-	0.0	4	2.3	-	0.0
Normal	7	4.0	162	93.1	1	0.6
Above ULN	-	0.0	-	0.0	-	0.0
Group 3 (PP) (N=181)						
Below LLN	1	0.6	5	2.9	-	0.0
Normal	6	3.4	162	92.6	1	0.6
Above ULN	-	0.0	-	0.0	-	0.0
Total						
Below LLN	1	0.2	15	2.9	-	0.0
Normal	14	2.7	492	93.7	2	0.4
Above ULN	-	0.0	1	0.2	-	0.0

Laboratory abnormalities, shift tables - Hematology, Safety dataset
Screening versus Visit 4

Platelets (10E9/l)

Screening	Visit 4					
	Below LLN		Normal		Above ULN	
	n	%	n	%	n	%
Group 1 (MM) (N=183)						
Below LLN	-	0.0	1	0.6	-	0.0
Normal	-	0.0	174	98.9	1	0.6
Above ULN	-	0.0	-	0.0	-	0.0
Group 2 (MP) (N=181)						
Below LLN	-	0.0	-	0.0	-	0.0
Normal	-	0.0	174	100.0	-	0.0
Above ULN	-	0.0	-	0.0	-	0.0
Group 3 (PP) (N=181)						
Below LLN	-	0.0	-	0.0	-	0.0
Normal	1	0.6	173	98.9	1	0.6
Above ULN	-	0.0	-	0.0	-	0.0
Total						
Below LLN	-	0.0	1	0.2	-	0.0
Normal	1	0.2	521	99.2	2	0.4
Above ULN	-	0.0	-	0.0	-	0.0

Laboratory abnormalities, shift tables - Hematology, Safety dataset

14.3.6.3 Screening versus Visit 5

Basophils (%)

Screening		Visit 5				
		Below LLN		Normal		Above ULN	
	n	%	n	%	n	%
Group 1 (MM) (N=183)						
Below LLN	-	0.0	-	0.0	-	0.0
Normal	-	0.0	21	100.0	-	0.0
Above ULN	-	0.0	-	0.0	-	0.0
Group 2 (MP) (N=181)						
Below LLN	-	0.0	-	0.0	-	0.0
Normal	-	0.0	21	100.0	-	0.0
Above ULN	-	0.0	-	0.0	-	0.0
Group 3 (PP) (N=181)						
Below LLN	-	0.0	-	0.0	-	0.0
Normal	-	0.0	18	100.0	-	0.0
Above ULN	-	0.0	-	0.0	-	0.0
Total						
Below LLN	-	0.0	-	0.0	-	0.0
Normal	-	0.0	60	100.0	-	0.0
Above ULN	-	0.0	-	0.0	-	0.0

Laboratory abnormalities, shift tables - Hematology, Safety dataset
Screening versus Visit 5

Eosinophils (%)

Screening		Visit 5				
	Below LLN		Normal		Above ULN	
	n	%	n	%	n	%
Group 1 (MM) (N=183)						
Below LLN	-	0.0	-	0.0	-	0.0
Normal	-	0.0	20	95.2	-	0.0
Above ULN	-	0.0	1	4.8	-	0.0
Group 2 (MP) (N=181)						
Below LLN	-	0.0	-	0.0	-	0.0
Normal	-	0.0	20	95.2	-	0.0
Above ULN	-	0.0	1	4.8	-	0.0
Group 3 (PP) (N=181)						
Below LLN	-	0.0	-	0.0	-	0.0
Normal	-	0.0	18	100.0	-	0.0
Above ULN	-	0.0	-	0.0	-	0.0
Total						
Below LLN	-	0.0	-	0.0	-	0.0
Normal	-	0.0	58	96.7	-	0.0
Above ULN	-	0.0	2	3.3	-	0.0

Laboratory abnormalities, shift tables - Hematology, Safety dataset
Screening versus Visit 5

Erythrocytes (10E12/l)

Screening	Visit 5					
	Below LLN		Normal		Above ULN	
	n	%	n	%	n	%
Group 1 (MM) (N=183)						
Below LLN	-	0.0	1	4.8	-	0.0
Normal	-	0.0	20	95.2	-	0.0
Above ULN	-	0.0	-	0.0	-	0.0
Group 2 (MP) (N=181)						
Below LLN	-	0.0	-	0.0	-	0.0
Normal	-	0.0	21	100.0	-	0.0
Above ULN	-	0.0	-	0.0	-	0.0
Group 3 (PP) (N=181)						
Below LLN	1	5.6	-	0.0	-	0.0
Normal	1	5.6	16	88.9	-	0.0
Above ULN	-	0.0	-	0.0	-	0.0
Total						
Below LLN	1	1.7	1	1.7	-	0.0
Normal	1	1.7	57	95.0	-	0.0
Above ULN	-	0.0	-	0.0	-	0.0

Laboratory abnormalities, shift tables - Hematology, Safety dataset
Screening versus Visit 5

Hematocrit (%)

Screening	Visit 5					
	Below LLN		Normal		Above ULN	
	n	%	n	%	n	%
Group 1 (MM) (N=183)						
Below LLN	-	0.0	1	4.8	-	0.0
Normal	1	4.8	19	90.5	-	0.0
Above ULN	-	0.0	-	0.0	-	0.0
Group 2 (MP) (N=181)						
Below LLN	1	4.8	-	0.0	-	0.0
Normal	1	4.8	19	90.5	-	0.0
Above ULN	-	0.0	-	0.0	-	0.0
Group 3 (PP) (N=181)						
Below LLN	-	0.0	1	5.6	-	0.0
Normal	1	5.6	16	88.9	-	0.0
Above ULN	-	0.0	-	0.0	-	0.0
Total						
Below LLN	1	1.7	2	3.3	-	0.0
Normal	3	5.0	54	90.0	-	0.0
Above ULN	-	0.0	-	0.0	-	0.0

Laboratory abnormalities, shift tables - Hematology, Safety dataset
Screening versus Visit 5

Hemoglobin (mmol/l)

Screening		Visit 5				
	Below LLN		Normal		Above ULN	
	n	%	n	%	n	%
Group 1 (MM) (N=183)						
Below LLN	1	4.8	-	0.0	-	0.0
Normal	1	4.8	19	90.5	-	0.0
Above ULN	-	0.0	-	0.0	-	0.0
Group 2 (MP) (N=181)						
Below LLN	1	4.8	2	9.5	-	0.0
Normal	1	4.8	17	81.0	-	0.0
Above ULN	-	0.0	-	0.0	-	0.0
Group 3 (PP) (N=181)						
Below LLN	-	0.0	1	5.6	-	0.0
Normal	1	5.6	16	88.9	-	0.0
Above ULN	-	0.0	-	0.0	-	0.0
Total						
Below LLN	2	3.3	3	5.0	-	0.0
Normal	3	5.0	52	86.7	-	0.0
Above ULN	-	0.0	-	0.0	-	0.0

Laboratory abnormalities, shift tables - Hematology, Safety dataset
Screening versus Visit 5

Leukocytes (10E9/l)

Screening		Visit 5				
	Below LLN		Normal		Above ULN	
	n	%	n	%	n	%
Group 1 (MM) (N=183)						
Below LLN	-	0.0	2	9.5	-	0.0
Normal	-	0.0	19	90.5	-	0.0
Above ULN	-	0.0	-	0.0	-	0.0
Group 2 (MP) (N=181)						
Below LLN	-	0.0	2	9.5	-	0.0
Normal	-	0.0	19	90.5	-	0.0
Above ULN	-	0.0	-	0.0	-	0.0
Group 3 (PP) (N=181)						
Below LLN	1	5.6	-	0.0	-	0.0
Normal	-	0.0	16	88.9	-	0.0
Above ULN	-	0.0	-	0.0	1	5.6
Total						
Below LLN	1	1.7	4	6.7	-	0.0
Normal	-	0.0	54	90.0	-	0.0
Above ULN	-	0.0	-	0.0	1	1.7

Laboratory abnormalities, shift tables - Hematology, Safety dataset
Screening versus Visit 5

Lymphocytes (%)

Screening		Visit 5				
	Below LLN		Normal		Above ULN	
	n	%	n	%	n	%
Group 1 (MM) (N=183)						
Below LLN	1	4.8	2	9.5	-	0.0
Normal	2	9.5	16	76.2	-	0.0
Above ULN	-	0.0	-	0.0	-	0.0
Group 2 (MP) (N=181)						
Below LLN	-	0.0	-	0.0	-	0.0
Normal	2	9.5	19	90.5	-	0.0
Above ULN	-	0.0	-	0.0	-	0.0
Group 3 (PP) (N=181)						
Below LLN	-	0.0	2	11.1	-	0.0
Normal	-	0.0	16	88.9	-	0.0
Above ULN	-	0.0	-	0.0	-	0.0
Total						
Below LLN	1	1.7	4	6.7	-	0.0
Normal	4	6.7	51	85.0	-	0.0
Above ULN	-	0.0	-	0.0	-	0.0

Laboratory abnormalities, shift tables - Hematology, Safety dataset
Screening versus Visit 5

MCH (fmol)

Screening		Visit 5				
		Below LLN		Normal		Above ULN	
		n	%	n	%	n	%
Group 1 (MM) (N=183)							
	Below LLN	2	9.5	1	4.8	-	0.0
	Normal	-	0.0	18	85.7	-	0.0
	Above ULN	-	0.0	-	0.0	-	0.0
Group 2 (MP) (N=181)							
	Below LLN	7	33.3	-	0.0	-	0.0
	Normal	4	19.0	10	47.6	-	0.0
	Above ULN	-	0.0	-	0.0	-	0.0
Group 3 (PP) (N=181)							
	Below LLN	2	11.1	-	0.0	-	0.0
	Normal	1	5.6	15	83.3	-	0.0
	Above ULN	-	0.0	-	0.0	-	0.0
Total							
	Below LLN	11	18.3	1	1.7	-	0.0
	Normal	5	8.3	43	71.7	-	0.0
	Above ULN	-	0.0	-	0.0	-	0.0

Laboratory abnormalities, shift tables - Hematology, Safety dataset
Screening versus Visit 5

MCV (fl)

Screening	Visit 5					
	Below LLN		Normal		Above ULN	
	n	%	n	%	n	%
Group 1 (MM) (N=183)						
Below LLN	1	4.8	-	0.0	-	0.0
Normal	-	0.0	20	95.2	-	0.0
Above ULN	-	0.0	-	0.0	-	0.0
Group 2 (MP) (N=181)						
Below LLN	1	4.8	1	4.8	-	0.0
Normal	-	0.0	19	90.5	-	0.0
Above ULN	-	0.0	-	0.0	-	0.0
Group 3 (PP) (N=181)						
Below LLN	-	0.0	-	0.0	-	0.0
Normal	1	5.6	17	94.4	-	0.0
Above ULN	-	0.0	-	0.0	-	0.0
Total						
Below LLN	2	3.3	1	1.7	-	0.0
Normal	1	1.7	56	93.3	-	0.0
Above ULN	-	0.0	-	0.0	-	0.0

Laboratory abnormalities, shift tables - Hematology, Safety dataset
Screening versus Visit 5

Monocytes (%)

Screening		Visit 5				
	Below LLN		Normal		Above ULN	
	n	%	n	%	n	%
Group 1 (MM) (N=183)						
Below LLN	-	0.0	-	0.0	-	0.0
Normal	-	0.0	19	90.5	-	0.0
Above ULN	-	0.0	2	9.5	-	0.0
Group 2 (MP) (N=181)						
Below LLN	-	0.0	-	0.0	-	0.0
Normal	-	0.0	21	100.0	-	0.0
Above ULN	-	0.0	-	0.0	-	0.0
Group 3 (PP) (N=181)						
Below LLN	-	0.0	-	0.0	-	0.0
Normal	-	0.0	15	83.3	-	0.0
Above ULN	-	0.0	3	16.7	-	0.0
Total						
Below LLN	-	0.0	-	0.0	-	0.0
Normal	-	0.0	55	91.7	-	0.0
Above ULN	-	0.0	5	8.3	-	0.0

Laboratory abnormalities, shift tables - Hematology, Safety dataset
Screening versus Visit 5

Neutrophils, total (%)

Screening	Visit 5					
	Below LLN		Normal		Above ULN	
	n	%	n	%	n	%
Group 1 (MM) (N=183)						
Below LLN	-	0.0	-	0.0	-	0.0
Normal	-	0.0	21	100.0	-	0.0
Above ULN	-	0.0	-	0.0	-	0.0
Group 2 (MP) (N=181)						
Below LLN	-	0.0	-	0.0	-	0.0
Normal	1	4.8	20	95.2	-	0.0
Above ULN	-	0.0	-	0.0	-	0.0
Group 3 (PP) (N=181)						
Below LLN	-	0.0	2	11.1	-	0.0
Normal	-	0.0	16	88.9	-	0.0
Above ULN	-	0.0	-	0.0	-	0.0
Total						
Below LLN	-	0.0	2	3.3	-	0.0
Normal	1	1.7	57	95.0	-	0.0
Above ULN	-	0.0	-	0.0	-	0.0

Laboratory abnormalities, shift tables - Hematology, Safety dataset
Screening versus Visit 5

Platelets (10E9/l)

Screening	Visit 5					
	Below LLN		Normal		Above ULN	
	n	%	n	%	n	%
Group 1 (MM) (N=183)						
Below LLN	-	0.0	-	0.0	-	0.0
Normal	-	0.0	21	100.0	-	0.0
Above ULN	-	0.0	-	0.0	-	0.0
Group 2 (MP) (N=181)						
Below LLN	-	0.0	-	0.0	-	0.0
Normal	-	0.0	20	95.2	1	4.8
Above ULN	-	0.0	-	0.0	-	0.0
Group 3 (PP) (N=181)						
Below LLN	-	0.0	-	0.0	-	0.0
Normal	-	0.0	17	94.4	1	5.6
Above ULN	-	0.0	-	0.0	-	0.0
Total						
Below LLN	-	0.0	-	0.0	-	0.0
Normal	-	0.0	58	96.7	2	3.3
Above ULN	-	0.0	-	0.0	-	0.0

Laboratory abnormalities, shift tables - Hematology, Safety dataset

14.3.6.4 Screening versus Follow up

Basophils (%)

Screening	Follow up					
	Below LLN		Normal		Above ULN	
	n	%	n	%	n	%
Group 1 (MM) (N=183)						
Below LLN	-	0.0	-	0.0	-	0.0
Normal	-	0.0	12	100.0	-	0.0
Above ULN	-	0.0	-	0.0	-	0.0
Group 2 (MP) (N=181)						
Below LLN	-	0.0	-	0.0	-	0.0
Normal	-	0.0	7	100.0	-	0.0
Above ULN	-	0.0	-	0.0	-	0.0
Group 3 (PP) (N=181)						
Below LLN	-	0.0	-	0.0	-	0.0
Normal	-	0.0	16	100.0	-	0.0
Above ULN	-	0.0	-	0.0	-	0.0
Group 4 (M-) (N=200)						
Below LLN	-	0.0	-	0.0	-	0.0
Normal	-	0.0	6	100.0	-	0.0
Above ULN	-	0.0	-	0.0	-	0.0
Total						
Below LLN	-	0.0	-	0.0	-	0.0
Normal	-	0.0	41	100.0	-	0.0
Above ULN	-	0.0	-	0.0	-	0.0

Laboratory abnormalities, shift tables - Hematology, Safety dataset
Screening versus Follow up

Eosinophils (%)

Screening	Follow up					
	Below LLN		Normal		Above ULN	
	n	%	n	%	n	%
Group 1 (MM) (N=183)						
Below LLN	-	0.0	-	0.0	-	0.0
Normal	-	0.0	12	100.0	-	0.0
Above ULN	-	0.0	-	0.0	-	0.0
Group 2 (MP) (N=181)						
Below LLN	-	0.0	-	0.0	-	0.0
Normal	-	0.0	7	100.0	-	0.0
Above ULN	-	0.0	-	0.0	-	0.0
Group 3 (PP) (N=181)						
Below LLN	-	0.0	-	0.0	-	0.0
Normal	-	0.0	15	93.8	-	0.0
Above ULN	-	0.0	-	0.0	1	6.3
Group 4 (M-) (N=200)						
Below LLN	-	0.0	-	0.0	-	0.0
Normal	-	0.0	6	100.0	-	0.0
Above ULN	-	0.0	-	0.0	-	0.0
Total						
Below LLN	-	0.0	-	0.0	-	0.0
Normal	-	0.0	40	97.6	-	0.0
Above ULN	-	0.0	-	0.0	1	2.4

Laboratory abnormalities, shift tables - Hematology, Safety dataset
Screening versus Follow up

Erythrocytes (10E12/l)

Screening		Follow up					
		Below LLN		Normal		Above ULN	
		n	%	n	%	n	%
Group 1 (MM) (N=183)							
	Below LLN	-	0.0	-	0.0	-	0.0
	Normal	-	0.0	12	100.0	-	0.0
	Above ULN	-	0.0	-	0.0	-	0.0
Group 2 (MP) (N=181)							
	Below LLN	-	0.0	-	0.0	-	0.0
	Normal	-	0.0	7	100.0	-	0.0
	Above ULN	-	0.0	-	0.0	-	0.0
Group 3 (PP) (N=181)							
	Below LLN	1	6.3	-	0.0	-	0.0
	Normal	-	0.0	15	93.8	-	0.0
	Above ULN	-	0.0	-	0.0	-	0.0
Group 4 (M-) (N=200)							
	Below LLN	1	16.7	-	0.0	-	0.0
	Normal	-	0.0	5	83.3	-	0.0
	Above ULN	-	0.0	-	0.0	-	0.0
Total							
	Below LLN	2	4.9	-	0.0	-	0.0
	Normal	-	0.0	39	95.1	-	0.0
	Above ULN	-	0.0	-	0.0	-	0.0

Laboratory abnormalities, shift tables - Hematology, Safety dataset
Screening versus Follow up

Hemoglobin (mmol/l)

Screening		Follow up					
		Below LLN		Normal		Above ULN	
		n	%	n	%	n	%
Group 1 (MM) (N=183)							
	Below LLN	2	16.7	-	0.0	-	0.0
	Normal	-	0.0	10	83.3	-	0.0
	Above ULN	-	0.0	-	0.0	-	0.0
Group 2 (MP) (N=181)							
	Below LLN	-	0.0	-	0.0	-	0.0
	Normal	-	0.0	6	85.7	-	0.0
	Above ULN	-	0.0	-	0.0	1	14.3
Group 3 (PP) (N=181)							
	Below LLN	3	18.8	-	0.0	-	0.0
	Normal	-	0.0	13	81.3	-	0.0
	Above ULN	-	0.0	-	0.0	-	0.0
Group 4 (M-) (N=200)							
	Below LLN	2	33.3	-	0.0	-	0.0
	Normal	-	0.0	4	66.7	-	0.0
	Above ULN	-	0.0	-	0.0	-	0.0
Total							
	Below LLN	7	17.1	-	0.0	-	0.0
	Normal	-	0.0	33	80.5	-	0.0
	Above ULN	-	0.0	-	0.0	1	2.4

Laboratory abnormalities, shift tables - Hematology, Safety dataset
Screening versus Follow up

Leukocytes (10E9/l)

Screening		Follow up					
		Below LLN		Normal		Above ULN	
		n	%	n	%	n	%
Group 1 (MM) (N=183)							
	Below LLN	-	0.0	-	0.0	-	0.0
	Normal	-	0.0	11	91.7	-	0.0
	Above ULN	-	0.0	-	0.0	1	8.3
Group 2 (MP) (N=181)							
	Below LLN	-	0.0	-	0.0	-	0.0
	Normal	-	0.0	6	85.7	-	0.0
	Above ULN	-	0.0	-	0.0	1	14.3
Group 3 (PP) (N=181)							
	Below LLN	1	6.3	-	0.0	-	0.0
	Normal	-	0.0	13	81.3	-	0.0
	Above ULN	-	0.0	-	0.0	2	12.5
Group 4 (M-) (N=200)							
	Below LLN	-	0.0	-	0.0	-	0.0
	Normal	-	0.0	6	100.0	-	0.0
	Above ULN	-	0.0	-	0.0	-	0.0
Total							
	Below LLN	1	2.4	-	0.0	-	0.0
	Normal	-	0.0	36	87.8	-	0.0
	Above ULN	-	0.0	-	0.0	4	9.8

Laboratory abnormalities, shift tables - Hematology, Safety dataset
Screening versus Follow up

Lymphocytes (%)

Screening		Follow up				
	Below LLN		Normal		Above ULN	
	n	%	n	%	n	%
Group 1 (MM) (N=183)						
Below LLN	1	8.3	-	0.0	-	0.0
Normal	-	0.0	9	75.0	-	0.0
Above ULN	-	0.0	1	8.3	1	8.3
Group 2 (MP) (N=181)						
Below LLN	-	0.0	-	0.0	-	0.0
Normal	-	0.0	7	100.0	-	0.0
Above ULN	-	0.0	-	0.0	-	0.0
Group 3 (PP) (N=181)						
Below LLN	2	12.5	-	0.0	-	0.0
Normal	-	0.0	14	87.5	-	0.0
Above ULN	-	0.0	-	0.0	-	0.0
Group 4 (M-) (N=200)						
Below LLN	1	16.7	-	0.0	-	0.0
Normal	-	0.0	5	83.3	-	0.0
Above ULN	-	0.0	-	0.0	-	0.0
Total						
Below LLN	4	9.8	-	0.0	-	0.0
Normal	-	0.0	35	85.4	-	0.0
Above ULN	-	0.0	1	2.4	1	2.4

Laboratory abnormalities, shift tables - Hematology, Safety dataset
Screening versus Follow up

Monocytes (%)

Screening	Follow up					
	Below LLN		Normal		Above ULN	
	n	%	n	%	n	%
Group 1 (MM) (N=183)						
Below LLN	-	0.0	-	0.0	-	0.0
Normal	-	0.0	11	91.7	-	0.0
Above ULN	-	0.0	-	0.0	1	8.3
Group 2 (MP) (N=181)						
Below LLN	-	0.0	-	0.0	-	0.0
Normal	-	0.0	7	100.0	-	0.0
Above ULN	-	0.0	-	0.0	-	0.0
Group 3 (PP) (N=181)						
Below LLN	-	0.0	-	0.0	-	0.0
Normal	-	0.0	15	93.8	-	0.0
Above ULN	-	0.0	-	0.0	1	6.3
Group 4 (M-) (N=200)						
Below LLN	-	0.0	-	0.0	-	0.0
Normal	-	0.0	5	83.3	-	0.0
Above ULN	-	0.0	-	0.0	1	16.7
Total						
Below LLN	-	0.0	-	0.0	-	0.0
Normal	-	0.0	38	92.7	-	0.0
Above ULN	-	0.0	-	0.0	3	7.3

Laboratory abnormalities, shift tables - Hematology, Safety dataset
Screening versus Follow up

Neutrophils, total (%)

Screening		Follow up				
	Below LLN		Normal		Above ULN	
	n	%	n	%	n	%
Group 1 (MM) (N=183)						
Below LLN	2	16.7	-	0.0	-	0.0
Normal	-	0.0	10	83.3	-	0.0
Above ULN	-	0.0	-	0.0	-	0.0
Group 2 (MP) (N=181)						
Below LLN	-	0.0	-	0.0	-	0.0
Normal	-	0.0	7	100.0	-	0.0
Above ULN	-	0.0	-	0.0	-	0.0
Group 3 (PP) (N=181)						
Below LLN	-	0.0	-	0.0	-	0.0
Normal	-	0.0	16	100.0	-	0.0
Above ULN	-	0.0	-	0.0	-	0.0
Group 4 (M-) (N=200)						
Below LLN	-	0.0	-	0.0	-	0.0
Normal	-	0.0	6	100.0	-	0.0
Above ULN	-	0.0	-	0.0	-	0.0
Total						
Below LLN	2	4.9	-	0.0	-	0.0
Normal	-	0.0	39	95.1	-	0.0
Above ULN	-	0.0	-	0.0	-	0.0

Laboratory abnormalities, shift tables - Hematology, Safety dataset
Screening versus Follow up

Platelets (10E9/l)

Screening	Follow up					
	Below LLN		Normal		Above ULN	
	n	%	n	%	n	%
Group 1 (MM) (N=183)						
Below LLN	-	0.0	-	0.0	-	0.0
Normal	-	0.0	11	91.7	-	0.0
Above ULN	-	0.0	-	0.0	1	8.3
Group 2 (MP) (N=181)						
Below LLN	-	0.0	-	0.0	-	0.0
Normal	-	0.0	7	100.0	-	0.0
Above ULN	-	0.0	-	0.0	-	0.0
Group 3 (PP) (N=181)						
Below LLN	-	0.0	-	0.0	-	0.0
Normal	-	0.0	16	100.0	-	0.0
Above ULN	-	0.0	-	0.0	-	0.0
Group 4 (M-) (N=200)						
Below LLN	-	0.0	-	0.0	-	0.0
Normal	-	0.0	6	100.0	-	0.0
Above ULN	-	0.0	-	0.0	-	0.0
Total						
Below LLN	-	0.0	-	0.0	-	0.0
Normal	-	0.0	40	97.6	-	0.0
Above ULN	-	0.0	-	0.0	1	2.4

14.3.7 Laboratory abnormalities, shift tables - Serum Chemistry, Safety dataset

14.3.7.1 Screening versus Visit 2

Alkaline phosphatase (U/l)

Screening	Visit 2					
	Below LLN		Normal		Above ULN	
	n	%	n	%	n	%
Group 1 (MM) (N=183)						
Below LLN	-	0.0	-	0.0	-	0.0
Normal	-	0.0	181	100.0	-	0.0
Above ULN	-	0.0	-	0.0	-	0.0
Group 2 (MP) (N=181)						
Below LLN	-	0.0	-	0.0	-	0.0
Normal	-	0.0	179	100.0	-	0.0
Above ULN	-	0.0	-	0.0	-	0.0
Group 3 (PP) (N=181)						
Below LLN	-	0.0	-	0.0	-	0.0
Normal	-	0.0	177	100.0	-	0.0
Above ULN	-	0.0	-	0.0	-	0.0
Group 4 (M-) (N=200)						
Below LLN	-	0.0	-	0.0	-	0.0
Normal	-	0.0	196	98.0	1	0.5
Above ULN	-	0.0	3	1.5	-	0.0
Total						
Below LLN	-	0.0	-	0.0	-	0.0
Normal	-	0.0	733	99.5	1	0.1
Above ULN	-	0.0	3	0.4	-	0.0

Laboratory abnormalities, shift tables - Serum Chemistry, Safety dataset
Screening versus Visit 2

Bilirubin, total (µmol/l)

Screening	Visit 2					
	Below LLN		Normal		Above ULN	
	n	%	n	%	n	%
Group 1 (MM) (N=183)						
Below LLN	-	0.0	-	0.0	-	0.0
Normal	-	0.0	158	87.3	4	2.2
Above ULN	-	0.0	10	5.5	9	5.0
Group 2 (MP) (N=181)						
Below LLN	-	0.0	-	0.0	-	0.0
Normal	-	0.0	153	85.5	3	1.7
Above ULN	-	0.0	10	5.6	13	7.3
Group 3 (PP) (N=181)						
Below LLN	-	0.0	-	0.0	-	0.0
Normal	-	0.0	148	83.6	5	2.8
Above ULN	-	0.0	9	5.1	15	8.5
Group 4 (M-) (N=200)						
Below LLN	-	0.0	-	0.0	-	0.0
Normal	-	0.0	173	86.5	6	3.0
Above ULN	-	0.0	8	4.0	13	6.5
Total						
Below LLN	-	0.0	-	0.0	-	0.0
Normal	-	0.0	632	85.8	18	2.4
Above ULN	-	0.0	37	5.0	50	6.8

Laboratory abnormalities, shift tables - Serum Chemistry, Safety dataset
Screening versus Visit 2

Calcium, total (mmol/l)

Screening		Visit 2				
	Below LLN		Normal		Above ULN	
	n	%	n	%	n	%
Group 1 (MM) (N=183)						
Below LLN	-	0.0	-	0.0	-	0.0
Normal	-	0.0	178	98.3	2	1.1
Above ULN	-	0.0	-	0.0	1	0.6
Group 2 (MP) (N=181)						
Below LLN	-	0.0	-	0.0	-	0.0
Normal	-	0.0	178	99.4	-	0.0
Above ULN	-	0.0	1	0.6	-	0.0
Group 3 (PP) (N=181)						
Below LLN	-	0.0	-	0.0	-	0.0
Normal	1	0.6	175	98.9	1	0.6
Above ULN	-	0.0	-	0.0	-	0.0
Group 4 (M-) (N=200)						
Below LLN	-	0.0	-	0.0	-	0.0
Normal	-	0.0	200	100.0	-	0.0
Above ULN	-	0.0	-	0.0	-	0.0
Total						
Below LLN	-	0.0	-	0.0	-	0.0
Normal	1	0.1	731	99.2	3	0.4
Above ULN	-	0.0	1	0.1	1	0.1

Laboratory abnormalities, shift tables - Serum Chemistry, Safety dataset
Screening versus Visit 2

Creatinine (µmol/l)

Screening		Visit 2				
	Below LLN		Normal		Above ULN	
	n	%	n	%	n	%
Group 1 (MM) (N=183)						
Below LLN	-	0.0	-	0.0	-	0.0
Normal	-	0.0	179	98.9	2	1.1
Above ULN	-	0.0	-	0.0	-	0.0
Group 2 (MP) (N=181)						
Below LLN	-	0.0	-	0.0	-	0.0
Normal	-	0.0	175	97.8	2	1.1
Above ULN	-	0.0	1	0.6	1	0.6
Group 3 (PP) (N=181)						
Below LLN	-	0.0	-	0.0	-	0.0
Normal	-	0.0	176	99.4	-	0.0
Above ULN	-	0.0	1	0.6	-	0.0
Group 4 (M-) (N=200)						
Below LLN	-	0.0	-	0.0	-	0.0
Normal	-	0.0	195	97.5	5	2.5
Above ULN	-	0.0	-	0.0	-	0.0
Total						
Below LLN	-	0.0	-	0.0	-	0.0
Normal	-	0.0	725	98.4	9	1.2
Above ULN	-	0.0	2	0.3	1	0.1

Laboratory abnormalities, shift tables - Serum Chemistry, Safety dataset
Screening versus Visit 2

GOT (AST) (U/l)

Screening	Visit 2					
	Below LLN		Normal		Above ULN	
	n	%	n	%	n	%
Group 1 (MM) (N=183)						
Below LLN	-	0.0	-	0.0	-	0.0
Normal	-	0.0	178	98.3	3	1.7
Above ULN	-	0.0	-	0.0	-	0.0
Group 2 (MP) (N=181)						
Below LLN	-	0.0	-	0.0	-	0.0
Normal	-	0.0	170	95.0	6	3.4
Above ULN	-	0.0	3	1.7	-	0.0
Group 3 (PP) (N=181)						
Below LLN	-	0.0	-	0.0	-	0.0
Normal	-	0.0	173	97.7	2	1.1
Above ULN	-	0.0	2	1.1	-	0.0
Group 4 (M-) (N=200)						
Below LLN	-	0.0	-	0.0	-	0.0
Normal	-	0.0	189	94.5	4	2.0
Above ULN	-	0.0	5	2.5	2	1.0
Total						
Below LLN	-	0.0	-	0.0	-	0.0
Normal	-	0.0	710	96.3	15	2.0
Above ULN	-	0.0	10	1.4	2	0.3

Laboratory abnormalities, shift tables - Serum Chemistry, Safety dataset
Screening versus Visit 2

GPT (ALT) (U/l)

Screening	Visit 2					
	Below LLN		Normal		Above ULN	
	n	%	n	%	n	%
Group 1 (MM) (N=183)						
Below LLN	-	0.0	-	0.0	-	0.0
Normal	-	0.0	169	93.4	2	1.1
Above ULN	-	0.0	9	5.0	1	0.6
Group 2 (MP) (N=181)						
Below LLN	-	0.0	-	0.0	-	0.0
Normal	-	0.0	165	92.2	4	2.2
Above ULN	-	0.0	6	3.4	4	2.2
Group 3 (PP) (N=181)						
Below LLN	-	0.0	-	0.0	-	0.0
Normal	-	0.0	170	96.0	2	1.1
Above ULN	-	0.0	2	1.1	3	1.7
Group 4 (M-) (N=200)						
Below LLN	-	0.0	-	0.0	-	0.0
Normal	-	0.0	178	89.0	2	1.0
Above ULN	-	0.0	12	6.0	8	4.0
Total						
Below LLN	-	0.0	-	0.0	-	0.0
Normal	-	0.0	682	92.5	10	1.4
Above ULN	-	0.0	29	3.9	16	2.2

Laboratory abnormalities, shift tables - Serum Chemistry, Safety dataset
Screening versus Visit 2

Potassium (mmol/l)

Screening	Visit 2					
	Below LLN		Normal		Above ULN	
	n	%	n	%	n	%
Group 1 (MM) (N=183)						
Below LLN	-	0.0	-	0.0	-	0.0
Normal	-	0.0	180	99.4	1	0.6
Above ULN	-	0.0	-	0.0	-	0.0
Group 2 (MP) (N=181)						
Below LLN	-	0.0	-	0.0	-	0.0
Normal	-	0.0	178	99.4	-	0.0
Above ULN	-	0.0	1	0.6	-	0.0
Group 3 (PP) (N=181)						
Below LLN	-	0.0	-	0.0	-	0.0
Normal	1	0.6	176	99.4	-	0.0
Above ULN	-	0.0	-	0.0	-	0.0
Group 4 (M-) (N=200)						
Below LLN	-	0.0	-	0.0	-	0.0
Normal	-	0.0	198	99.0	-	0.0
Above ULN	-	0.0	2	1.0	-	0.0
Total						
Below LLN	-	0.0	-	0.0	-	0.0
Normal	1	0.1	732	99.3	1	0.1
Above ULN	-	0.0	3	0.4	-	0.0

Laboratory abnormalities, shift tables - Serum Chemistry, Safety dataset
Screening versus Visit 2

Sodium (mmol/l)

Screening		Visit 2				
	Below LLN		Normal		Above ULN	
	n	%	n	%	n	%
Group 1 (MM) (N=183)						
Below LLN	-	0.0	-	0.0	-	0.0
Normal	-	0.0	177	98.3	3	1.7
Above ULN	-	0.0	-	0.0	-	0.0
Group 2 (MP) (N=181)						
Below LLN	-	0.0	-	0.0	-	0.0
Normal	-	0.0	177	98.9	1	0.6
Above ULN	-	0.0	1	0.6	-	0.0
Group 3 (PP) (N=181)						
Below LLN	-	0.0	-	0.0	-	0.0
Normal	-	0.0	175	99.4	-	0.0
Above ULN	-	0.0	1	0.6	-	0.0
Group 4 (M-) (N=200)						
Below LLN	-	0.0	-	0.0	-	0.0
Normal	-	0.0	199	99.5	1	0.5
Above ULN	-	0.0	-	0.0	-	0.0
Total						
Below LLN	-	0.0	-	0.0	-	0.0
Normal	-	0.0	728	99.0	5	0.7
Above ULN	-	0.0	2	0.3	-	0.0

Laboratory abnormalities, shift tables - Serum Chemistry, Safety dataset
Screening versus Visit 2

Troponin I (µg/l)

Screening		Visit 2				
	Below LLN		Normal		Above ULN	
	n	%	n	%	n	%
Group 1 (MM) (N=183)						
Below LLN	-	0.0	-	0.0	-	0.0
Normal	-	0.0	181	100.0	-	0.0
Above ULN	-	0.0	-	0.0	-	0.0
Group 2 (MP) (N=181)						
Below LLN	-	0.0	-	0.0	-	0.0
Normal	-	0.0	179	100.0	-	0.0
Above ULN	-	0.0	-	0.0	-	0.0
Group 3 (PP) (N=181)						
Below LLN	-	0.0	-	0.0	-	0.0
Normal	-	0.0	177	100.0	-	0.0
Above ULN	-	0.0	-	0.0	-	0.0
Group 4 (M-) (N=200)						
Below LLN	-	0.0	-	0.0	-	0.0
Normal	-	0.0	200	100.0	-	0.0
Above ULN	-	0.0	-	0.0	-	0.0
Total						
Below LLN	-	0.0	-	0.0	-	0.0
Normal	-	0.0	737	100.0	-	0.0
Above ULN	-	0.0	-	0.0	-	0.0

Laboratory abnormalities, shift tables - Serum Chemistry, Safety dataset

14.3.7.2 Screening versus Visit 4

Alkaline phosphatase (U/l)

Screening	Visit 4					
	Below LLN		Normal		Above ULN	
	n	%	n	%	n	%
Group 1 (MM) (N=183)						
Below LLN	-	0.0	-	0.0	-	0.0
Normal	-	0.0	175	99.4	1	0.6
Above ULN	-	0.0	-	0.0	-	0.0
Group 2 (MP) (N=181)						
Below LLN	-	0.0	-	0.0	-	0.0
Normal	-	0.0	174	100.0	-	0.0
Above ULN	-	0.0	-	0.0	-	0.0
Group 3 (PP) (N=181)						
Below LLN	-	0.0	-	0.0	-	0.0
Normal	-	0.0	175	100.0	-	0.0
Above ULN	-	0.0	-	0.0	-	0.0
Total						
Below LLN	-	0.0	-	0.0	-	0.0
Normal	-	0.0	524	99.8	1	0.2
Above ULN	-	0.0	-	0.0	-	0.0

Laboratory abnormalities, shift tables - Serum Chemistry, Safety dataset
Screening versus Visit 4

Bilirubin, total (µmol/l)

Screening		Visit 4				
	Below LLN		Normal		Above ULN	
	n	%	n	%	n	%
Group 1 (MM) (N=183)						
Below LLN	-	0.0	-	0.0	-	0.0
Normal	-	0.0	149	84.7	8	4.5
Above ULN	-	0.0	10	5.7	9	5.1
Group 2 (MP) (N=181)						
Below LLN	-	0.0	-	0.0	-	0.0
Normal	-	0.0	145	83.3	7	4.0
Above ULN	-	0.0	9	5.2	13	7.5
Group 3 (PP) (N=181)						
Below LLN	-	0.0	-	0.0	-	0.0
Normal	-	0.0	144	82.3	8	4.6
Above ULN	-	0.0	10	5.7	13	7.4
Total						
Below LLN	-	0.0	-	0.0	-	0.0
Normal	-	0.0	438	83.4	23	4.4
Above ULN	-	0.0	29	5.5	35	6.7

Laboratory abnormalities, shift tables - Serum Chemistry, Safety dataset
Screening versus Visit 4

Calcium, total (mmol/l)

Screening		Visit 4					
		Below LLN		Normal		Above ULN	
		n	%	n	%	n	%
Group 1 (MM) (N=183)							
	Below LLN	-	0.0	-	0.0	-	0.0
	Normal	2	1.1	172	98.3	-	0.0
	Above ULN	-	0.0	1	0.6	-	0.0
Group 2 (MP) (N=181)							
	Below LLN	-	0.0	-	0.0	-	0.0
	Normal	-	0.0	173	99.4	-	0.0
	Above ULN	-	0.0	1	0.6	-	0.0
Group 3 (PP) (N=181)							
	Below LLN	-	0.0	-	0.0	-	0.0
	Normal	-	0.0	173	98.9	2	1.1
	Above ULN	-	0.0	-	0.0	-	0.0
Total							
	Below LLN	-	0.0	-	0.0	-	0.0
	Normal	2	0.4	518	98.9	2	0.4
	Above ULN	-	0.0	2	0.4	-	0.0

Laboratory abnormalities, shift tables - Serum Chemistry, Safety dataset
Screening versus Visit 4

Creatinine (µmol/l)

Screening		Visit 4					
		Below LLN		Normal		Above ULN	
		n	%	n	%	n	%
Group 1 (MM) (N=183)							
	Below LLN	-	0.0	-	0.0	-	0.0
	Normal	-	0.0	174	98.9	2	1.1
	Above ULN	-	0.0	-	0.0	-	0.0
Group 2 (MP) (N=181)							
	Below LLN	-	0.0	-	0.0	-	0.0
	Normal	-	0.0	172	98.9	-	0.0
	Above ULN	-	0.0	1	0.6	1	0.6
Group 3 (PP) (N=181)							
	Below LLN	-	0.0	-	0.0	-	0.0
	Normal	-	0.0	174	99.4	-	0.0
	Above ULN	-	0.0	1	0.6	-	0.0
Total							
	Below LLN	-	0.0	-	0.0	-	0.0
	Normal	-	0.0	520	99.0	2	0.4
	Above ULN	-	0.0	2	0.4	1	0.2

Laboratory abnormalities, shift tables - Serum Chemistry, Safety dataset
Screening versus Visit 4

GOT (AST) (U/l)

Screening		Visit 4				
	Below LLN		Normal		Above ULN	
	n	%	n	%	n	%
Group 1 (MM) (N=183)						
Below LLN	-	0.0	-	0.0	-	0.0
Normal	-	0.0	173	98.3	3	1.7
Above ULN	-	0.0	-	0.0	-	0.0
Group 2 (MP) (N=181)						
Below LLN	-	0.0	-	0.0	-	0.0
Normal	-	0.0	170	97.7	2	1.1
Above ULN	-	0.0	2	1.1	-	0.0
Group 3 (PP) (N=181)						
Below LLN	-	0.0	-	0.0	-	0.0
Normal	-	0.0	173	98.9	1	0.6
Above ULN	-	0.0	1	0.6	-	0.0
Total						
Below LLN	-	0.0	-	0.0	-	0.0
Normal	-	0.0	516	98.3	6	1.1
Above ULN	-	0.0	3	0.6	-	0.0

Laboratory abnormalities, shift tables - Serum Chemistry, Safety dataset
Screening versus Visit 4

GPT (ALT) (U/l)

Screening	Visit 4					
	Below LLN		Normal		Above ULN	
	n	%	n	%	n	%
Group 1 (MM) (N=183)						
Below LLN	-	0.0	-	0.0	-	0.0
Normal	-	0.0	158	89.8	8	4.5
Above ULN	-	0.0	8	4.5	2	1.1
Group 2 (MP) (N=181)						
Below LLN	-	0.0	-	0.0	-	0.0
Normal	-	0.0	160	92.0	4	2.3
Above ULN	-	0.0	6	3.4	4	2.3
Group 3 (PP) (N=181)						
Below LLN	-	0.0	-	0.0	-	0.0
Normal	-	0.0	164	93.7	6	3.4
Above ULN	-	0.0	2	1.1	3	1.7
Total						
Below LLN	-	0.0	-	0.0	-	0.0
Normal	-	0.0	482	91.8	18	3.4
Above ULN	-	0.0	16	3.0	9	1.7

Laboratory abnormalities, shift tables - Serum Chemistry, Safety dataset
Screening versus Visit 4

Potassium (mmol/l)

Screening		Visit 4					
		Below LLN		Normal		Above ULN	
		n	%	n	%	n	%
Group 1 (MM) (N=183)							
	Below LLN	-	0.0	-	0.0	-	0.0
	Normal	2	1.1	174	98.9	-	0.0
	Above ULN	-	0.0	-	0.0	-	0.0
Group 2 (MP) (N=181)							
	Below LLN	-	0.0	-	0.0	-	0.0
	Normal	-	0.0	173	99.4	-	0.0
	Above ULN	-	0.0	1	0.6	-	0.0
Group 3 (PP) (N=181)							
	Below LLN	-	0.0	-	0.0	-	0.0
	Normal	-	0.0	172	98.9	2	1.1
	Above ULN	-	0.0	-	0.0	-	0.0
Total							
	Below LLN	-	0.0	-	0.0	-	0.0
	Normal	2	0.4	519	99.0	2	0.4
	Above ULN	-	0.0	1	0.2	-	0.0

Laboratory abnormalities, shift tables - Serum Chemistry, Safety dataset
Screening versus Visit 4

Sodium (mmol/l)

Screening		Visit 4				
	Below LLN		Normal		Above ULN	
	n	%	n	%	n	%
Group 1 (MM) (N=183)						
Below LLN	-	0.0	-	0.0	-	0.0
Normal	-	0.0	172	98.9	2	1.1
Above ULN	-	0.0	-	0.0	-	0.0
Group 2 (MP) (N=181)						
Below LLN	-	0.0	-	0.0	-	0.0
Normal	-	0.0	172	98.9	1	0.6
Above ULN	-	0.0	1	0.6	-	0.0
Group 3 (PP) (N=181)						
Below LLN	-	0.0	-	0.0	-	0.0
Normal	1	0.6	170	97.1	3	1.7
Above ULN	-	0.0	1	0.6	-	0.0
Total						
Below LLN	-	0.0	-	0.0	-	0.0
Normal	1	0.2	514	98.3	6	1.1
Above ULN	-	0.0	2	0.4	-	0.0

Laboratory abnormalities, shift tables - Serum Chemistry, Safety dataset
Screening versus Visit 4

Troponin I (µg/l)

Screening		Visit 4				
	Below LLN		Normal		Above ULN	
	n	%	n	%	n	%
Group 1 (MM) (N=183)						
Below LLN	-	0.0	-	0.0	-	0.0
Normal	-	0.0	176	100.0	-	0.0
Above ULN	-	0.0	-	0.0	-	0.0
Group 2 (MP) (N=181)						
Below LLN	-	0.0	-	0.0	-	0.0
Normal	-	0.0	174	100.0	-	0.0
Above ULN	-	0.0	-	0.0	-	0.0
Group 3 (PP) (N=181)						
Below LLN	-	0.0	-	0.0	-	0.0
Normal	-	0.0	175	100.0	-	0.0
Above ULN	-	0.0	-	0.0	-	0.0
Total						
Below LLN	-	0.0	-	0.0	-	0.0
Normal	-	0.0	525	100.0	-	0.0
Above ULN	-	0.0	-	0.0	-	0.0

Laboratory abnormalities, shift tables - Serum Chemistry, Safety dataset

14.3.7.3 Screening versus Visit 5

Alkaline phosphatase (U/l)

Screening	Visit 5					
	Below LLN		Normal		Above ULN	
	n	%	n	%	n	%
Group 1 (MM) (N=183)						
Below LLN	-	0.0	-	0.0	-	0.0
Normal	-	0.0	23	100.0	-	0.0
Above ULN	-	0.0	-	0.0	-	0.0
Group 2 (MP) (N=181)						
Below LLN	-	0.0	-	0.0	-	0.0
Normal	-	0.0	20	100.0	-	0.0
Above ULN	-	0.0	-	0.0	-	0.0
Group 3 (PP) (N=181)						
Below LLN	-	0.0	-	0.0	-	0.0
Normal	-	0.0	17	100.0	-	0.0
Above ULN	-	0.0	-	0.0	-	0.0
Total						
Below LLN	-	0.0	-	0.0	-	0.0
Normal	-	0.0	60	100.0	-	0.0
Above ULN	-	0.0	-	0.0	-	0.0

Laboratory abnormalities, shift tables - Serum Chemistry, Safety dataset
Screening versus Visit 5

Bilirubin, total (µmol/l)

Screening	Visit 5					
	Below LLN		Normal		Above ULN	
	n	%	n	%	n	%
Group 1 (MM) (N=183)						
Below LLN	-	0.0	-	0.0	-	0.0
Normal	-	0.0	22	91.7	-	0.0
Above ULN	-	0.0	-	0.0	2	8.3
Group 2 (MP) (N=181)						
Below LLN	-	0.0	-	0.0	-	0.0
Normal	-	0.0	16	80.0	-	0.0
Above ULN	-	0.0	3	15.0	1	5.0
Group 3 (PP) (N=181)						
Below LLN	-	0.0	-	0.0	-	0.0
Normal	-	0.0	13	76.5	1	5.9
Above ULN	-	0.0	2	11.8	1	5.9
Total						
Below LLN	-	0.0	-	0.0	-	0.0
Normal	-	0.0	51	83.6	1	1.6
Above ULN	-	0.0	5	8.2	4	6.6

Laboratory abnormalities, shift tables - Serum Chemistry, Safety dataset
Screening versus Visit 5

Calcium, total (mmol/l)

Screening		Visit 5					
		Below LLN		Normal		Above ULN	
		n	%	n	%	n	%
Group 1 (MM) (N=183)							
	Below LLN	-	0.0	-	0.0	-	0.0
	Normal	1	4.3	22	95.7	-	0.0
	Above ULN	-	0.0	-	0.0	-	0.0
Group 2 (MP) (N=181)							
	Below LLN	-	0.0	-	0.0	-	0.0
	Normal	1	5.0	19	95.0	-	0.0
	Above ULN	-	0.0	-	0.0	-	0.0
Group 3 (PP) (N=181)							
	Below LLN	-	0.0	-	0.0	-	0.0
	Normal	-	0.0	17	100.0	-	0.0
	Above ULN	-	0.0	-	0.0	-	0.0
Total							
	Below LLN	-	0.0	-	0.0	-	0.0
	Normal	2	3.3	58	96.7	-	0.0
	Above ULN	-	0.0	-	0.0	-	0.0

Laboratory abnormalities, shift tables - Serum Chemistry, Safety dataset
Screening versus Visit 5

Creatinine (µmol/l)

Screening	Visit 5					
	Below LLN		Normal		Above ULN	
	n	%	n	%	n	%
Group 1 (MM) (N=183)						
Below LLN	-	0.0	-	0.0	-	0.0
Normal	-	0.0	23	100.0	-	0.0
Above ULN	-	0.0	-	0.0	-	0.0
Group 2 (MP) (N=181)						
Below LLN	-	0.0	-	0.0	-	0.0
Normal	-	0.0	19	95.0	-	0.0
Above ULN	-	0.0	-	0.0	1	5.0
Group 3 (PP) (N=181)						
Below LLN	-	0.0	-	0.0	-	0.0
Normal	-	0.0	17	100.0	-	0.0
Above ULN	-	0.0	-	0.0	-	0.0
Total						
Below LLN	-	0.0	-	0.0	-	0.0
Normal	-	0.0	59	98.3	-	0.0
Above ULN	-	0.0	-	0.0	1	1.7

Laboratory abnormalities, shift tables - Serum Chemistry, Safety dataset
Screening versus Visit 5

GOT (AST) (U/l)

Screening	Visit 5					
	Below LLN		Normal		Above ULN	
	n	%	n	%	n	%
Group 1 (MM) (N=183)						
Below LLN	-	0.0	-	0.0	-	0.0
Normal	-	0.0	23	100.0	-	0.0
Above ULN	-	0.0	-	0.0	-	0.0
Group 2 (MP) (N=181)						
Below LLN	-	0.0	-	0.0	-	0.0
Normal	-	0.0	19	95.0	-	0.0
Above ULN	-	0.0	1	5.0	-	0.0
Group 3 (PP) (N=181)						
Below LLN	-	0.0	-	0.0	-	0.0
Normal	-	0.0	17	100.0	-	0.0
Above ULN	-	0.0	-	0.0	-	0.0
Total						
Below LLN	-	0.0	-	0.0	-	0.0
Normal	-	0.0	59	98.3	-	0.0
Above ULN	-	0.0	1	1.7	-	0.0

Laboratory abnormalities, shift tables - Serum Chemistry, Safety dataset
Screening versus Visit 5

GPT (ALT) (U/l)

Screening	Visit 5					
	Below LLN		Normal		Above ULN	
	n	%	n	%	n	%
Group 1 (MM) (N=183)						
Below LLN	-	0.0	-	0.0	-	0.0
Normal	-	0.0	22	95.7	1	4.3
Above ULN	-	0.0	-	0.0	-	0.0
Group 2 (MP) (N=181)						
Below LLN	-	0.0	-	0.0	-	0.0
Normal	-	0.0	19	95.0	-	0.0
Above ULN	-	0.0	1	5.0	-	0.0
Group 3 (PP) (N=181)						
Below LLN	-	0.0	-	0.0	-	0.0
Normal	-	0.0	16	94.1	-	0.0
Above ULN	-	0.0	-	0.0	1	5.9
Total						
Below LLN	-	0.0	-	0.0	-	0.0
Normal	-	0.0	57	95.0	1	1.7
Above ULN	-	0.0	1	1.7	1	1.7

Laboratory abnormalities, shift tables - Serum Chemistry, Safety dataset
Screening versus Visit 5

Potassium (mmol/l)

Screening		Visit 5				
	Below LLN		Normal		Above ULN	
	n	%	n	%	n	%
Group 1 (MM) (N=183)						
Below LLN	-	0.0	-	0.0	-	0.0
Normal	-	0.0	23	100.0	-	0.0
Above ULN	-	0.0	-	0.0	-	0.0
Group 2 (MP) (N=181)						
Below LLN	-	0.0	-	0.0	-	0.0
Normal	-	0.0	20	100.0	-	0.0
Above ULN	-	0.0	-	0.0	-	0.0
Group 3 (PP) (N=181)						
Below LLN	-	0.0	-	0.0	-	0.0
Normal	-	0.0	17	100.0	-	0.0
Above ULN	-	0.0	-	0.0	-	0.0
Total						
Below LLN	-	0.0	-	0.0	-	0.0
Normal	-	0.0	60	100.0	-	0.0
Above ULN	-	0.0	-	0.0	-	0.0

Laboratory abnormalities, shift tables - Serum Chemistry, Safety dataset
Screening versus Visit 5

Sodium (mmol/l)

Screening		Visit 5				
		Below LLN		Normal		Above ULN	
		n	%	n	%	n	%
Group 1 (MM) (N=183)							
	Below LLN	-	0.0	-	0.0	-	0.0
	Normal	1	4.3	22	95.7	-	0.0
	Above ULN	-	0.0	-	0.0	-	0.0
Group 2 (MP) (N=181)							
	Below LLN	-	0.0	-	0.0	-	0.0
	Normal	1	5.0	19	95.0	-	0.0
	Above ULN	-	0.0	-	0.0	-	0.0
Group 3 (PP) (N=181)							
	Below LLN	-	0.0	-	0.0	-	0.0
	Normal	-	0.0	17	100.0	-	0.0
	Above ULN	-	0.0	-	0.0	-	0.0
Total							
	Below LLN	-	0.0	-	0.0	-	0.0
	Normal	2	3.3	58	96.7	-	0.0
	Above ULN	-	0.0	-	0.0	-	0.0

Laboratory abnormalities, shift tables - Serum Chemistry, Safety dataset
Screening versus Visit 5

Troponin I (µg/l)

Screening	Visit 5					
	Below LLN		Normal		Above ULN	
	n	%	n	%	n	%
Group 1 (MM) (N=183)						
Below LLN	-	0.0	-	0.0	-	0.0
Normal	-	0.0	23	100.0	-	0.0
Above ULN	-	0.0	-	0.0	-	0.0
Group 2 (MP) (N=181)						
Below LLN	-	0.0	-	0.0	-	0.0
Normal	-	0.0	20	100.0	-	0.0
Above ULN	-	0.0	-	0.0	-	0.0
Group 3 (PP) (N=181)						
Below LLN	-	0.0	-	0.0	-	0.0
Normal	-	0.0	17	100.0	-	0.0
Above ULN	-	0.0	-	0.0	-	0.0
Total						
Below LLN	-	0.0	-	0.0	-	0.0
Normal	-	0.0	60	100.0	-	0.0
Above ULN	-	0.0	-	0.0	-	0.0

Laboratory abnormalities, shift tables - Serum Chemistry, Safety dataset

14.3.7.4 Screening versus Follow up

Alkaline phosphatase (U/l)

Screening	Follow up					
	Below LLN		Normal		Above ULN	
	n	%	n	%	n	%
Group 1 (MM) (N=183)						
Below LLN	-	0.0	-	0.0	-	0.0
Normal	-	0.0	10	90.9	-	0.0
Above ULN	-	0.0	-	0.0	1	9.1
Group 2 (MP) (N=181)						
Below LLN	-	0.0	-	0.0	-	0.0
Normal	-	0.0	6	100.0	-	0.0
Above ULN	-	0.0	-	0.0	-	0.0
Group 3 (PP) (N=181)						
Below LLN	-	0.0	-	0.0	-	0.0
Normal	-	0.0	6	100.0	-	0.0
Above ULN	-	0.0	-	0.0	-	0.0
Group 4 (M-) (N=200)						
Below LLN	-	0.0	-	0.0	-	0.0
Normal	-	0.0	7	100.0	-	0.0
Above ULN	-	0.0	-	0.0	-	0.0
Total						
Below LLN	-	0.0	-	0.0	-	0.0
Normal	-	0.0	29	96.7	-	0.0
Above ULN	-	0.0	-	0.0	1	3.3

Laboratory abnormalities, shift tables - Serum Chemistry, Safety dataset
Screening versus Follow up

Bilirubin, total (µmol/l)

Screening		Follow up				
	Below LLN		Normal		Above ULN	
	n	%	n	%	n	%
Group 1 (MM) (N=183)						
Below LLN	-	0.0	-	0.0	-	0.0
Normal	-	0.0	10	90.9	-	0.0
Above ULN	-	0.0	-	0.0	1	9.1
Group 2 (MP) (N=181)						
Below LLN	-	0.0	-	0.0	-	0.0
Normal	-	0.0	6	100.0	-	0.0
Above ULN	-	0.0	-	0.0	-	0.0
Group 3 (PP) (N=181)						
Below LLN	-	0.0	-	0.0	-	0.0
Normal	-	0.0	4	66.7	-	0.0
Above ULN	-	0.0	1	16.7	1	16.7
Group 4 (M-) (N=200)						
Below LLN	-	0.0	-	0.0	-	0.0
Normal	-	0.0	7	100.0	-	0.0
Above ULN	-	0.0	-	0.0	-	0.0
Total						
Below LLN	-	0.0	-	0.0	-	0.0
Normal	-	0.0	27	90.0	-	0.0
Above ULN	-	0.0	1	3.3	2	6.7

Laboratory abnormalities, shift tables - Serum Chemistry, Safety dataset
Screening versus Follow up

Calcium, total (mmol/l)

Screening		Follow up					
		Below LLN		Normal		Above ULN	
		n	%	n	%	n	%
Group 1 (MM) (N=183)							
	Below LLN	-	0.0	-	0.0	-	0.0
	Normal	-	0.0	11	100.0	-	0.0
	Above ULN	-	0.0	-	0.0	-	0.0
Group 2 (MP) (N=181)							
	Below LLN	-	0.0	-	0.0	-	0.0
	Normal	-	0.0	6	100.0	-	0.0
	Above ULN	-	0.0	-	0.0	-	0.0
Group 3 (PP) (N=181)							
	Below LLN	-	0.0	-	0.0	-	0.0
	Normal	-	0.0	6	100.0	-	0.0
	Above ULN	-	0.0	-	0.0	-	0.0
Group 4 (M-) (N=200)							
	Below LLN	-	0.0	-	0.0	-	0.0
	Normal	-	0.0	7	100.0	-	0.0
	Above ULN	-	0.0	-	0.0	-	0.0
Total							
	Below LLN	-	0.0	-	0.0	-	0.0
	Normal	-	0.0	30	100.0	-	0.0
	Above ULN	-	0.0	-	0.0	-	0.0

Laboratory abnormalities, shift tables - Serum Chemistry, Safety dataset
Screening versus Follow up

Creatinine (µmol/l)

Screening		Follow up				
	Below LLN		Normal		Above ULN	
	n	%	n	%	n	%
Group 1 (MM) (N=183)						
Below LLN	-	0.0	-	0.0	-	0.0
Normal	-	0.0	11	100.0	-	0.0
Above ULN	-	0.0	-	0.0	-	0.0
Group 2 (MP) (N=181)						
Below LLN	-	0.0	-	0.0	-	0.0
Normal	-	0.0	5	83.3	-	0.0
Above ULN	-	0.0	-	0.0	1	16.7
Group 3 (PP) (N=181)						
Below LLN	-	0.0	-	0.0	-	0.0
Normal	-	0.0	5	83.3	1	16.7
Above ULN	-	0.0	-	0.0	-	0.0
Group 4 (M-) (N=200)						
Below LLN	-	0.0	-	0.0	-	0.0
Normal	-	0.0	7	100.0	-	0.0
Above ULN	-	0.0	-	0.0	-	0.0
Total						
Below LLN	-	0.0	-	0.0	-	0.0
Normal	-	0.0	28	93.3	1	3.3
Above ULN	-	0.0	-	0.0	1	3.3

Laboratory abnormalities, shift tables - Serum Chemistry, Safety dataset
Screening versus Follow up

GOT (AST) (U/l)

Screening		Follow up				
	Below LLN		Normal		Above ULN	
	n	%	n	%	n	%
Group 1 (MM) (N=183)						
Below LLN	-	0.0	-	0.0	-	0.0
Normal	-	0.0	11	100.0	-	0.0
Above ULN	-	0.0	-	0.0	-	0.0
Group 2 (MP) (N=181)						
Below LLN	-	0.0	-	0.0	-	0.0
Normal	-	0.0	6	100.0	-	0.0
Above ULN	-	0.0	-	0.0	-	0.0
Group 3 (PP) (N=181)						
Below LLN	-	0.0	-	0.0	-	0.0
Normal	-	0.0	6	100.0	-	0.0
Above ULN	-	0.0	-	0.0	-	0.0
Group 4 (M-) (N=200)						
Below LLN	-	0.0	-	0.0	-	0.0
Normal	-	0.0	7	100.0	-	0.0
Above ULN	-	0.0	-	0.0	-	0.0
Total						
Below LLN	-	0.0	-	0.0	-	0.0
Normal	-	0.0	30	100.0	-	0.0
Above ULN	-	0.0	-	0.0	-	0.0

Laboratory abnormalities, shift tables - Serum Chemistry, Safety dataset
Screening versus Follow up

GPT (ALT) (U/l)

Screening	Follow up					
	Below LLN		Normal		Above ULN	
	n	%	n	%	n	%
Group 1 (MM) (N=183)						
Below LLN	-	0.0	-	0.0	-	0.0
Normal	-	0.0	9	81.8	-	0.0
Above ULN	-	0.0	-	0.0	2	18.2
Group 2 (MP) (N=181)						
Below LLN	-	0.0	-	0.0	-	0.0
Normal	-	0.0	4	66.7	-	0.0
Above ULN	-	0.0	-	0.0	2	33.3
Group 3 (PP) (N=181)						
Below LLN	-	0.0	-	0.0	-	0.0
Normal	-	0.0	4	66.7	-	0.0
Above ULN	-	0.0	1	16.7	1	16.7
Group 4 (M-) (N=200)						
Below LLN	-	0.0	-	0.0	-	0.0
Normal	-	0.0	7	100.0	-	0.0
Above ULN	-	0.0	-	0.0	-	0.0
Total						
Below LLN	-	0.0	-	0.0	-	0.0
Normal	-	0.0	24	80.0	-	0.0
Above ULN	-	0.0	1	3.3	5	16.7

Laboratory abnormalities, shift tables - Serum Chemistry, Safety dataset
Screening versus Follow up

Potassium (mmol/l)

Screening		Follow up					
		Below LLN		Normal		Above ULN	
		n	%	n	%	n	%
Group 1 (MM) (N=183)							
	Below LLN	-	0.0	-	0.0	-	0.0
	Normal	-	0.0	11	100.0	-	0.0
	Above ULN	-	0.0	-	0.0	-	0.0
Group 2 (MP) (N=181)							
	Below LLN	-	0.0	-	0.0	-	0.0
	Normal	-	0.0	6	100.0	-	0.0
	Above ULN	-	0.0	-	0.0	-	0.0
Group 3 (PP) (N=181)							
	Below LLN	-	0.0	-	0.0	-	0.0
	Normal	-	0.0	6	100.0	-	0.0
	Above ULN	-	0.0	-	0.0	-	0.0
Group 4 (M-) (N=200)							
	Below LLN	-	0.0	-	0.0	-	0.0
	Normal	-	0.0	7	100.0	-	0.0
	Above ULN	-	0.0	-	0.0	-	0.0
Total							
	Below LLN	-	0.0	-	0.0	-	0.0
	Normal	-	0.0	30	100.0	-	0.0
	Above ULN	-	0.0	-	0.0	-	0.0

Laboratory abnormalities, shift tables - Serum Chemistry, Safety dataset
Screening versus Follow up

Sodium (mmol/l)

Screening		Follow up					
		Below LLN		Normal		Above ULN	
		n	%	n	%	n	%
Group 1 (MM) (N=183)							
	Below LLN	-	0.0	-	0.0	-	0.0
	Normal	-	0.0	11	100.0	-	0.0
	Above ULN	-	0.0	-	0.0	-	0.0
Group 2 (MP) (N=181)							
	Below LLN	-	0.0	-	0.0	-	0.0
	Normal	-	0.0	6	100.0	-	0.0
	Above ULN	-	0.0	-	0.0	-	0.0
Group 3 (PP) (N=181)							
	Below LLN	-	0.0	-	0.0	-	0.0
	Normal	-	0.0	6	100.0	-	0.0
	Above ULN	-	0.0	-	0.0	-	0.0
Group 4 (M-) (N=200)							
	Below LLN	-	0.0	-	0.0	-	0.0
	Normal	-	0.0	7	100.0	-	0.0
	Above ULN	-	0.0	-	0.0	-	0.0
Total							
	Below LLN	-	0.0	-	0.0	-	0.0
	Normal	-	0.0	30	100.0	-	0.0
	Above ULN	-	0.0	-	0.0	-	0.0

Laboratory abnormalities, shift tables - Serum Chemistry, Safety dataset
Screening versus Follow up

Troponin I (µg/l)

Screening		Follow up				
	Below LLN		Normal		Above ULN	
	n	%	n	%	n	%
Group 1 (MM) (N=183)						
Below LLN	-	0.0	-	0.0	-	0.0
Normal	-	0.0	11	100.0	-	0.0
Above ULN	-	0.0	-	0.0	-	0.0
Group 2 (MP) (N=181)						
Below LLN	-	0.0	-	0.0	-	0.0
Normal	-	0.0	6	100.0	-	0.0
Above ULN	-	0.0	-	0.0	-	0.0
Group 3 (PP) (N=181)						
Below LLN	-	0.0	-	0.0	-	0.0
Normal	-	0.0	6	100.0	-	0.0
Above ULN	-	0.0	-	0.0	-	0.0
Group 4 (M-) (N=200)						
Below LLN	-	0.0	-	0.0	-	0.0
Normal	-	0.0	7	100.0	-	0.0
Above ULN	-	0.0	-	0.0	-	0.0
Total						
Below LLN	-	0.0	-	0.0	-	0.0
Normal	-	0.0	30	100.0	-	0.0
Above ULN	-	0.0	-	0.0	-	0.0

14.4 ECG DATA

14.4.1 Count and percentage of subjects with normal/ abnormal and clinically significant ECG findings - Safety dataset

Visit=Screening

	Group 1 (MM) (N=183)	Group 2 (MP) (N=181)	Group 3 (PP) (N=181)	Group 4 (M-) (N=200)	Total (N=745)	p-value
	Number of subjects (%) / Descriptive Statistics					
Assessment						
-	0 (0.0)	0 (0.0)	0 (0.0)	0 (0.0)	0 (0.0)	
normal	183 (100.0)	181 (100.0)	181 (100.0)	200 (100.0)	745 (100.0)	
abnormal	0 (0.0)	0 (0.0)	0 (0.0)	0 (0.0)	0 (0.0)	
not evaluable	0 (0.0)	0 (0.0)	0 (0.0)	0 (0.0)	0 (0.0)	
ECG assessment performed by						
-	0 (0.0)	0 (0.0)	0 (0.0)	0 (0.0)	0 (0.0)	0.005~
not done	0 (0.0)	0 (0.0)	0 (0.0)	0 (0.0)	0 (0.0)	
investigator	115 (62.8)	107 (59.1)	106 (58.6)	92 (46.0)	420 (56.4)	
cardiologist	68 (37.2)	74 (40.9)	75 (41.4)	108 (54.0)	325 (43.6)	
not required, as not clinically indicated	0 (0.0)	0 (0.0)	0 (0.0)	0 (0.0)	0 (0.0)	

^ Fisher's Exact test
N/A: not applicable as not required -: Missing
For Group 1-3, no ECG was performed at Visit 3 and at Visit 5 only if clinically indicated
For Group 4, at Visit 3 an ECG was only performed if clinically indicated
In case of repeated ECGs, the last ECG was used in this table

Count and percentage of subjects with normal/ abnormal and clinically significant ECG findings - Safety dataset

Visit=Visit 2

	Number of subjects (%) / Descriptive Statistics					
	Group 1 (MM) (N=183)	Group 2 (MP) (N=181)	Group 3 (PP) (N=181)	Group 4 (M-) (N=200)	Total (N=745)	p-value
Assessment						
-	0 (0.0)	0 (0.0)	0 (0.0)	0 (0.0)	0 (0.0)	
normal	182 (99.5)	179 (98.9)	179 (98.9)	200 (100.0)	740 (99.3)	
abnormal	0 (0.0)	0 (0.0)	0 (0.0)	0 (0.0)	0 (0.0)	
not evaluable	0 (0.0)	0 (0.0)	0 (0.0)	0 (0.0)	0 (0.0)	
ECG assessment performed by						
-	0 (0.0)	0 (0.0)	0 (0.0)	0 (0.0)	0 (0.0)	0.290~
not done	0 (0.0)	0 (0.0)	0 (0.0)	0 (0.0)	0 (0.0)	
investigator	159 (86.9)	157 (86.7)	165 (91.2)	183 (91.5)	664 (89.1)	
cardiologist	23 (12.6)	22 (12.2)	14 (7.7)	17 (8.5)	76 (10.2)	
not required, as not clinically indicated	0 (0.0)	0 (0.0)	0 (0.0)	0 (0.0)	0 (0.0)	

^ Fisher's Exact test
N/A: not applicable as not required -: Missing
For Group 1-3, no ECG was performed at Visit 3 and at Visit 5 only if clinically indicated
For Group 4, at Visit 3 an ECG was only performed if clinically indicated
In case of repeated ECGs, the last ECG was used in this table

Count and percentage of subjects with normal/ abnormal and clinically significant ECG findings - Safety dataset

Visit=Visit 3

	Group 1 (MM) (N=183)	Group 2 (MP) (N=181)	Group 3 (PP) (N=181)	Group 4 (M-) (N=200)	Total (N=745)	p-value
	Number of subjects (%) / Descriptive Statistics					
Assessment						
-	0 (0.0)	0 (0.0)	0 (0.0)	0 (0.0)	0 (0.0)	
normal	0 (0.0)	0 (0.0)	0 (0.0)	7 (3.5)	7 (0.9)	
abnormal	0 (0.0)	0 (0.0)	0 (0.0)	0 (0.0)	0 (0.0)	
not evaluable	0 (0.0)	0 (0.0)	0 (0.0)	0 (0.0)	0 (0.0)	
N/A	0 (0.0)	0 (0.0)	0 (0.0)	193 (96.5)	193 (25.9)	
ECG assessment performed by						
-	0 (0.0)	0 (0.0)	0 (0.0)	0 (0.0)	0 (0.0)	
not done	0 (0.0)	0 (0.0)	0 (0.0)	0 (0.0)	0 (0.0)	
investigator	0 (0.0)	0 (0.0)	0 (0.0)	6 (3.0)	6 (0.8)	
cardiologist	0 (0.0)	0 (0.0)	0 (0.0)	1 (0.5)	1 (0.1)	
not required, as not clinically indicated	0 (0.0)	0 (0.0)	0 (0.0)	193 (96.5)	193 (25.9)	

^ Fisher's Exact test
N/A: not applicable as not required -: Missing
For Group 1-3, no ECG was performed at Visit 3 and at Visit 5 only if clinically indicated
For Group 4, at Visit 3 an ECG was only performed if clinically indicated
In case of repeated ECGs, the last ECG was used in this table

Count and percentage of subjects with normal/ abnormal and clinically significant ECG findings - Safety dataset

Visit=Visit 4

	Number of subjects (%) / Descriptive Statistics					
	Group 1 (MM) (N=183)	Group 2 (MP) (N=181)	Group 3 (PP) (N=181)	Group 4 (M-) (N=200)	Total (N=745)	p-value
Assessment						
-	0 (0.0)	0 (0.0)	0 (0.0)	0 (0.0)	0 (0.0)	>0.999^
normal	176 (96.2)	174 (96.1)	175 (96.7)	0 (0.0)	525 (70.5)	
abnormal	0 (0.0)	0 (0.0)	0 (0.0)	0 (0.0)	0 (0.0)	
not evaluable	0 (0.0)	0 (0.0)	0 (0.0)	0 (0.0)	0 (0.0)	
N/A	1 (0.5)	0 (0.0)	0 (0.0)	0 (0.0)	1 (0.1)	
ECG assessment performed by						
-	0 (0.0)	0 (0.0)	0 (0.0)	0 (0.0)	0 (0.0)	0.371^
not done	0 (0.0)	0 (0.0)	0 (0.0)	0 (0.0)	0 (0.0)	
investigator	166 (90.7)	168 (92.8)	163 (90.1)	0 (0.0)	497 (66.7)	
cardiologist	10 (5.5)	6 (3.3)	12 (6.6)	0 (0.0)	28 (3.8)	
not required, as not clinically indicated	0 (0.0)	0 (0.0)	0 (0.0)	0 (0.0)	0 (0.0)	
N/A	1 (0.5)	0 (0.0)	0 (0.0)	0 (0.0)	1 (0.1)	

^ Fisher's Exact test
N/A: not applicable as not required -: Missing
For Group 1-3, no ECG was performed at Visit 3 and at Visit 5 only if clinically indicated
For Group 4, at Visit 3 an ECG was only performed if clinically indicated
In case of repeated ECGs, the last ECG was used in this table

Count and percentage of subjects with normal/ abnormal and clinically significant ECG findings - Safety dataset

Visit=Visit 5

	Number of subjects (%) / Descriptive Statistics					
	Group 1 (MM) (N=183)	Group 2 (MP) (N=181)	Group 3 (PP) (N=181)	Group 4 (M-) (N=200)	Total (N=745)	p-value
Assessment						
-	0 (0.0)	0 (0.0)	0 (0.0)	0 (0.0)	0 (0.0)	0.797~
normal	7 (3.8)	5 (2.8)	5 (2.8)	0 (0.0)	17 (2.3)	
abnormal	0 (0.0)	0 (0.0)	0 (0.0)	0 (0.0)	0 (0.0)	
not evaluable	0 (0.0)	0 (0.0)	0 (0.0)	0 (0.0)	0 (0.0)	
N/A	171 (93.4)	170 (93.9)	172 (95.0)	0 (0.0)	513 (68.9)	
ECG assessment performed by						
-	0 (0.0)	0 (0.0)	0 (0.0)	0 (0.0)	0 (0.0)	0.964^
not done	0 (0.0)	0 (0.0)	0 (0.0)	0 (0.0)	0 (0.0)	
investigator	6 (3.3)	4 (2.2)	4 (2.2)	0 (0.0)	14 (1.9)	
cardiologist	1 (0.5)	1 (0.6)	1 (0.6)	0 (0.0)	3 (0.4)	
not required, as not clinically indicated	171 (93.4)	170 (93.9)	172 (95.0)	0 (0.0)	513 (68.9)	

^ Fisher's Exact test
N/A: not applicable as not required -: Missing
For Group 1-3, no ECG was performed at Visit 3 and at Visit 5 only if clinically indicated
For Group 4, at Visit 3 an ECG was only performed if clinically indicated
In case of repeated ECGs, the last ECG was used in this table

Count and percentage of subjects with normal/ abnormal and clinically significant ECG findings - Safety dataset

Visit=Follow-Up

	Group 1 (MM) (N=178)	Group 2 (MP) (N=174)	Group 3 (PP) (N=175)	Group 4 (M-) (N=199)	Total (N=726)
	Number of subjects (%) / Descriptive Statistics				
Assessment					
-	0 (0.0)	0 (0.0)	0 (0.0)	0 (0.0)	0 (0.0)
normal	2 (1.1)	3 (1.7)	3 (1.7)	5 (2.5)	13 (1.8)
abnormal	0 (0.0)	0 (0.0)	0 (0.0)	0 (0.0)	0 (0.0)
not evaluable	0 (0.0)	0 (0.0)	0 (0.0)	0 (0.0)	0 (0.0)
N/A	176 (98.9)	171 (98.3)	172 (98.3)	194 (97.5)	713 (98.2)
ECG assessment performed by					
-	0 (0.0)	0 (0.0)	0 (0.0)	0 (0.0)	0 (0.0)
not done	0 (0.0)	0 (0.0)	0 (0.0)	0 (0.0)	0 (0.0)
investigator	2 (1.1)	2 (1.1)	2 (1.1)	3 (1.5)	9 (1.2)
cardiologist	0 (0.0)	1 (0.6)	1 (0.6)	2 (1.0)	4 (0.6)
not required, as not clinically indicated	176 (98.9)	171 (98.3)	172 (98.3)	194 (97.5)	713 (98.2)

^ Fisher's Exact test
N/A: not applicable as not required -: Missing
For Group 1-3, no ECG was performed at Visit 3 and at Visit 5 only if clinically indicated
For Group 4, at Visit 3 an ECG was only performed if clinically indicated
In case of repeated ECGs, the last ECG was used in this table

14.4.2 Count and percentage of subjects with Myo-/Pericarditis

No cases of Myo-/Pericarditis were reported.

14.4.3 ECG - abnormal ECGs

No abnormal ECGs were recorded in the trial.

14.4.4 QTc analysis: categorical analysis

14.4.4.1 Absolute QTc values > 450ms (Fridericia´s correction)

Absolute QTc > 450ms	Screening		Visit 2		Visit 4		Follow-Up	
	n	%	n	%	n	%	n	%
Yes								
Group 1 (MM)	5	2.7	-	0.0	3	1.7	-	0.0
Group 2 (MP)	2	1.1	3	1.7	1	0.6	-	0.0
Group 3 (PP)	5	2.8	2	1.1	1	0.6	-	0.0
Group 4 (M-)	2	1.0	3	1.5	-	-	-	0.0
Total	14	1.9	8	1.1	5	1.0	-	0.0
No								
Group 1 (MM)	178	97.3	182	100.0	172	98.3	2	100.0
Group 2 (MP)	178	98.9	175	98.3	171	99.4	3	100.0
Group 3 (PP)	176	97.2	177	98.9	173	99.4	3	100.0
Group 4 (M-)	197	99.0	196	98.5	-	-	5	100.0
Total	729	98.1	730	98.9	516	99.0	13	100.0

14.4.4.2 Absolute QTc values > 480ms (Fridericia´s correction)

Absolute QTc > 480ms	Screening		Visit 2		Visit 4		Follow-Up	
	n	%	n	%	n	%	n	%
Yes								
Group 1 (MM)	-	0.0	-	0.0	-	0.0	-	0.0
Group 2 (MP)	-	0.0	1	0.6	-	0.0	-	0.0
Group 3 (PP)	-	0.0	-	0.0	-	0.0	-	0.0
Group 4 (M-)	-	0.0	-	0.0	-	-	-	0.0
Total	-	0.0	1	0.1	-	0.0	-	0.0
No								
Group 1 (MM)	183	100.0	182	100.0	175	100.0	2	100.0
Group 2 (MP)	180	100.0	177	99.4	172	100.0	3	100.0
Group 3 (PP)	181	100.0	179	100.0	174	100.0	3	100.0
Group 4 (M-)	199	100.0	199	100.0	-	-	5	100.0
Total	743	100.0	737	99.9	521	100.0	13	100.0

14.4.4.3 Absolute QTc value > 500ms (Fridericia´s correction)

Absolute QTc values > 500ms (Fridericia´s correction)

Absolute QTc > 500ms	Screening		Visit 2		Visit 4		Follow-Up	
	n	%	n	%	n	%	n	%
Yes								
Group 1 (MM)	-	0.0	-	0.0	-	0.0	-	0.0
Group 2 (MP)	-	0.0	-	0.0	-	0.0	-	0.0
Group 3 (PP)	-	0.0	-	0.0	-	0.0	-	0.0
Group 4 (M-)	-	0.0	-	0.0	-	-	-	0.0
Total	-	0.0	-	0.0	-	0.0	-	0.0
No								
Group 1 (MM)	183	100.0	182	100.0	175	100.0	2	100.0
Group 2 (MP)	180	100.0	178	100.0	172	100.0	3	100.0
Group 3 (PP)	181	100.0	179	100.0	174	100.0	3	100.0
Group 4 (M-)	199	100.0	199	100.0	-	-	5	100.0
Total	743	100.0	738	100.0	521	100.0	13	100.0

14.4.4.4 Change from baseline >= 30ms (Fridericia´s correction)

Change from baseline >= 30ms (Fridericia´s correction)

Change from baseline >= 30ms	Screening		Visit 2		Visit 4		Follow-Up	
	n	%	n	%	n	%	n	%
Yes								
Group 1 (MM)	-	0.0	5	2.7	12	6.9	-	0.0
Group 2 (MP)	-	0.0	11	6.2	11	6.4	-	0.0
Group 3 (PP)	-	0.0	8	4.5	9	5.2	-	0.0
Group 4 (M-)	-	0.0	6	3.0	-	-	-	0.0
All subjects	-	-	-	-	-	-	-	-
Total	-	0.0	30	4.1	32	6.2	-	0.0
No								
Group 1 (MM)	183	100.0	177	97.3	163	93.1	2	100.0
Group 2 (MP)	180	100.0	166	93.8	160	93.6	3	100.0
Group 3 (PP)	181	100.0	171	95.5	165	94.8	3	100.0
Group 4 (M-)	199	100.0	192	97.0	-	-	5	100.0
All subjects	-	-	-	-	-	-	-	-
Total	743	100.0	706	95.9	488	93.8	13	100.0

14.4.4.5 Change from baseline >= 60ms (Fridericia´s correction)

Change from baseline >= 60ms	Screening		Visit 2		Visit 4		Follow-Up	
	n	%	n	%	n	%	n	%
Yes								
Group 1 (MM)	-	0.0	-	0.0	-	0.0	-	0.0
Group 2 (MP)	-	0.0	1	0.6	-	0.0	-	0.0
Group 3 (PP)	-	0.0	1	0.6	-	0.0	-	0.0
Group 4 (M-)	-	0.0	-	0.0	-	-	-	0.0
All subjects	-	-	-	-	-	-	-	-
Total	-	0.0	2	0.3	-	0.0	-	0.0
No								
Group 1 (MM)	183	100.0	182	100.0	175	100.0	2	100.0
Group 2 (MP)	180	100.0	176	99.4	171	100.0	3	100.0
Group 3 (PP)	181	100.0	178	99.4	174	100.0	3	100.0
Group 4 (M-)	199	100.0	198	100.0	-	-	5	100.0
All subjects	-	-	-	-	-	-	-	-
Total	743	100.0	734	99.7	520	100.0	13	100.0

14.4.5 QTc analysis: analysis of central tendency

14.4.5.1 Absolute QTc values (Fridericia´s correction)

[ms]	Visit			
	Screening	Visit 2	Visit 4	Follow-Up
Group 1 (MM)				
Mean	404.7	402.5	403.1	395.2
Median	403.0	404.2	402.1	395.2
StdDev	18.9	17.5	20.9	2.8
StdErr	1.4	1.3	1.6	2.0
Min	370.0	360.8	345.4	393.2
Max	473.4	441.8	471.7	397.2
N	183	182	175	2
Group 2 (MP)				
Mean	407.1	407.3	408.5	402.2
Median	409.2	408.2	407.4	404.8
StdDev	19.9	20.4	19.6	12.1
StdErr	1.5	1.5	1.5	7.0
Min	357.4	356.9	355.0	389.0
Max	456.1	480.9	457.3	412.8
N	180	178	172	3
Group 3 (PP)				
Mean	406.0	404.1	402.8	420.6
Median	405.5	405.7	406.1	426.8
StdDev	20.3	18.5	20.0	32.4
StdErr	1.5	1.4	1.5	18.7
Min	357.5	348.7	345.5	385.6
Max	475.9	468.6	457.0	449.5
N	181	179	174	3
Group 4 (M-)				
Mean	406.8	406.5	-	396.1
Median	407.4	407.5	-	396.7
StdDev	20.3	19.3	-	18.7
StdErr	1.4	1.4	-	8.3
Min	336.9	355.1	-	370.1

(Continued)

Absolute QTc values (Fridericia´s correction)

[ms]	Visit			
	Screening	Visit 2	Visit 4	Follow-Up
Group 4 (M-)				
Max	466.8	454.1	-	421.7
N	199	199	0	5
Total				
Mean	406.2	405.1	404.8	403.0
Median	406.4	406.7	405.1	397.2
StdDev	19.8	19.0	20.3	20.6
StdErr	0.7	0.7	0.9	5.7
Min	336.9	348.7	345.4	370.1
Max	475.9	480.9	471.7	449.5
N	743	738	521	13

14.4.5.2 Maximum post-dose values (Fridericia´s correction)

[ms]	Group				Total
	Group 1 (MM)	Group 2 (MP)	Group 3 (PP)	Group 4 (M-)	
QTcF - duration [ms]					
Mean	410.5	415.6	410.6	406.7	410.7
Median	409.3	414.3	412.5	407.8	411.1
StdDev	18.0	18.9	17.7	19.3	18.7
StdErr	1.3	1.4	1.3	1.4	0.7
Min	360.8	370.0	360.4	355.1	355.1
Max	471.7	480.9	468.6	454.1	480.9
N	182	179	179	199	739

14.4.5.3 Change from baseline (Fridericia´s correction)

[ms]	Visit			
	Screening	Visit 2	Visit 4	Follow-Up
Group 1 (MM)				
Mean	0.0	-2.2	-1.5	-21.5
Median	0.0	-1.6	-1.7	-21.5
StdDev	0.0	18.6	19.1	11.6
StdErr	0.0	1.4	1.4	8.2
Min	0.0	-51.4	-46.5	-29.7
Max	0.0	53.0	40.1	-13.3
N	183	182	175	2
Group 2 (MP)				
Mean	0.0	-0.0	1.2	-8.9
Median	0.0	-0.4	-0.8	-7.2
StdDev	0.0	18.9	18.6	8.8
StdErr	0.0	1.4	1.4	5.1
Min	0.0	-47.3	-43.0	-18.4
Max	0.0	70.5	53.2	-1.1
N	180	177	171	3
Group 3 (PP)				
Mean	0.0	-1.7	-2.4	-11.7
Median	0.0	-0.6	-4.1	-16.0
StdDev	0.0	18.6	18.9	17.3
StdErr	0.0	1.4	1.4	10.0
Min	0.0	-51.8	-47.0	-26.5
Max	0.0	60.2	57.6	7.4
N	181	179	174	3
Group 4 (M-)				
Mean	0.0	-0.3	-	-12.9
Median	0.0	0.1	-	-10.9
StdDev	0.0	17.6	-	5.5
StdErr	0.0	1.3	-	2.5
Min	0.0	-48.0	-	-22.5

(Continued)

Change from baseline (Fridericia´s correction)

[ms]	Visit			
	Screening	Visit 2	Visit 4	Follow-Up
Group 4 (M-)				
Max	0.0	51.2	-	-9.1
N	199	198	0	5
Total				
Mean	0.0	-1.0	-0.9	-13.0
Median	0.0	-0.6	-2.2	-12.4
StdDev	0.0	18.4	18.9	10.0
StdErr	0.0	0.7	0.8	2.8
Min	0.0	-51.8	-47.0	-29.7
Max	0.0	70.5	57.6	7.4
N	743	736	520	13

14.4.5.4 Mean maximal change from baseline (Fridericia´s correction)

[ms]	Group				Total
	Group 1 (MM)	Group 2 (MP)	Group 3 (PP)	Group 4 (M-)	
Baseline- adjusted QTcF - duration [ms]					
Mean	5.8	8.4	4.7	-0.1	4.6
Median	5.0	8.3	5.6	0.1	4.5
StdDev	17.9	18.2	17.0	17.6	17.9
StdErr	1.3	1.4	1.3	1.3	0.7
Min	-41.0	-47.3	-38.0	-48.0	-48.0
Max	53.0	70.5	60.2	51.2	70.5
N	182	178	179	198	737

Values can differ from table 'Change from baseline (Fridericia´s correction)' because of consideration of results on optional visits e.g. Visit 3 for Group 4

14.4.6 Further ECG data

14.4.6.1 PR-duration

PR-duration

[ms]	Group				
	Group 1 (MM)	Group 2 (MP)	Group 3 (PP)	Group 4 (M-)	Total
Screening					
Mean	151.6	150.8	153.3	159.3	153.9
Median	152.0	150.0	153.0	161.0	153.5
StdDev	18.8	19.9	20.3	20.1	20.1
StdErr	1.4	1.5	1.5	1.4	0.7
Min	113.0	100.0	102.0	114.0	100.0
Max	210.0	208.0	247.0	215.0	247.0
N	183	181	181	199	744
Visit 2					
Mean	150.6	150.7	152.1	156.2	152.5
Median	149.0	149.0	151.0	157.0	152.0
StdDev	17.9	17.6	17.9	18.8	18.2
StdErr	1.3	1.3	1.3	1.3	0.7
Min	102.0	115.0	104.0	114.0	102.0
Max	200.0	203.0	223.0	210.0	223.0
N	182	178	179	199	738
Visit 4					
Mean	151.8	150.1	153.9	-	151.9
Median	151.0	151.0	152.0	-	151.0
StdDev	19.3	18.1	19.3	-	18.9
StdErr	1.5	1.4	1.5	-	0.8
Min	93.0	97.0	106.0	-	93.0
Max	222.0	194.0	250.0	-	250.0
N	175	172	174	0	521

(Continued)

PR-duration

[ms]	Group				Total
	Group 1 (MM)	Group 2 (MP)	Group 3 (PP)	Group 4 (M-)	
Follow-Up					
Mean	152.0	150.7	140.3	157.8	151.2
Median	152.0	146.0	132.0	158.0	150.0
StdDev	2.8	11.7	49.0	24.5	25.9
StdErr	2.0	6.8	28.3	10.9	7.2
Min	150.0	142.0	96.0	127.0	96.0
Max	154.0	164.0	193.0	195.0	195.0
N	2	3	3	5	13

14.4.6.2 QRS-duration

QRS-duration

[ms]	Group				Total
	Group 1 (MM)	Group 2 (MP)	Group 3 (PP)	Group 4 (M-)	
Screening					
Mean	95.4	95.6	95.0	93.7	94.9
Median	96.0	95.0	95.0	93.0	95.0
StdDev	8.2	9.2	9.1	9.7	9.1
StdErr	0.6	0.7	0.7	0.7	0.3
Min	74.0	80.0	76.0	66.0	66.0
Max	119.0	132.0	124.0	129.0	132.0
N	183	181	181	199	744
Visit 2					
Mean	95.4	96.5	94.9	94.2	95.2
Median	94.5	96.0	94.0	94.0	95.0
StdDev	8.9	9.9	9.2	9.1	9.3
StdErr	0.7	0.7	0.7	0.6	0.3
Min	75.0	74.0	70.0	72.0	70.0
Max	134.0	124.0	124.0	127.0	134.0
N	182	178	179	199	738
Visit 4					
Mean	95.0	95.4	95.5	-	95.3
Median	96.0	95.0	95.0	-	95.0
StdDev	8.8	8.9	9.3	-	9.0
StdErr	0.7	0.7	0.7	-	0.4
Min	72.0	79.0	74.0	-	72.0
Max	116.0	131.0	123.0	-	131.0
N	175	172	174	0	521

(Continued)

QRS-duration

[ms]	Group				
	Group 1 (MM)	Group 2 (MP)	Group 3 (PP)	Group 4 (M-)	Total
Follow-Up					
Mean	110.5	90.7	91.0	95.4	95.6
Median	110.5	90.0	89.0	94.0	94.0
StdDev	9.2	6.0	7.2	7.2	9.4
StdErr	6.5	3.5	4.2	3.2	2.6
Min	104.0	85.0	85.0	86.0	85.0
Max	117.0	97.0	99.0	104.0	117.0
N	2	3	3	5	13

14.4.6.3 QT-duration

QT-duration

[ms]	Group				Total
	Group 1 (MM)	Group 2 (MP)	Group 3 (PP)	Group 4 (M-)	
Screening					
Mean	402.1	405.6	402.4	401.4	402.8
Median	400.0	405.0	399.0	400.0	401.0
StdDev	30.0	27.4	29.9	24.9	28.0
StdErr	2.2	2.0	2.2	1.8	1.0
Min	333.0	330.0	337.0	342.0	330.0
Max	501.0	477.0	512.0	475.0	512.0
N	183	180	181	199	743
Visit 2					
Mean	388.3	392.2	392.9	391.2	391.1
Median	387.5	394.0	393.0	390.0	390.5
StdDev	28.9	29.1	29.7	24.6	28.1
StdErr	2.1	2.2	2.2	1.7	1.0
Min	330.0	317.0	313.0	341.0	313.0
Max	471.0	483.0	484.0	459.0	484.0
N	182	178	179	199	738
Visit 4					
Mean	391.1	392.1	390.3	-	391.1
Median	388.0	390.5	386.0	-	389.0
StdDev	29.8	26.5	27.9	-	28.1
StdErr	2.3	2.0	2.1	-	1.2
Min	329.0	303.0	316.0	-	303.0
Max	485.0	465.0	469.0	-	485.0
N	175	172	174	0	521

(Continued)

QT-duration

[ms]	Group				Total
	Group 1 (MM)	Group 2 (MP)	Group 3 (PP)	Group 4 (M-)	
Follow-Up					
Mean	399.5	375.7	414.3	362.8	383.3
Median	399.5	371.0	405.0	345.0	386.0
StdDev	19.1	17.5	40.8	28.8	33.3
StdErr	13.5	10.1	23.6	12.9	9.2
Min	386.0	361.0	379.0	338.0	338.0
Max	413.0	395.0	459.0	399.0	459.0
N	2	3	3	5	13

14.4.6.4 QTc-duration (Bazett correction)

QTc-duration (Bazett correction)

[ms]	Group				
	Group 1 (MM)	Group 2 (MP)	Group 3 (PP)	Group 4 (M-)	Total
Screening					
Mean	394.7	397.2	397.5	401.7	397.9
Median	393.0	396.5	398.0	405.0	397.0
StdDev	22.3	25.3	23.6	24.6	24.1
StdErr	1.6	1.9	1.8	1.7	0.9
Min	336.0	334.0	349.0	338.0	334.0
Max	469.0	476.0	454.0	465.0	476.0
N	183	180	181	199	743
Visit 2					
Mean	397.7	403.0	399.2	405.3	401.4
Median	397.0	404.0	400.0	406.5	402.0
StdDev	23.1	25.7	23.5	22.9	23.9
StdErr	1.7	1.9	1.8	1.6	0.9
Min	343.0	339.0	334.0	340.0	334.0
Max	454.0	491.0	457.0	459.0	491.0
N	182	178	179	198	737
Visit 4					
Mean	397.5	406.1	397.9	-	400.5
Median	399.0	407.0	398.5	-	401.0
StdDev	24.1	25.0	23.6	-	24.5
StdErr	1.8	1.9	1.8	-	1.1
Min	340.0	332.0	329.0	-	329.0
Max	465.0	469.0	464.0	-	469.0
N	175	172	174	0	521

(Continued)

QTc-duration (Bazett correction)

[ms]	Group				Total
	Group 1 (MM)	Group 2 (MP)	Group 3 (PP)	Group 4 (M-)	
Follow-Up					
Mean	388.5	406.3	417.7	405.6	405.9
Median	388.5	400.0	435.0	402.0	400.0
StdDev	2.1	15.5	41.8	23.6	24.6
StdErr	1.5	9.0	24.1	10.6	6.8
Min	387.0	395.0	370.0	377.0	370.0
Max	390.0	424.0	448.0	439.0	448.0
N	2	3	3	5	13

14.4.6.5 RR Interval

RR Interval

[ms]	Group				Total
	Group 1 (MM)	Group 2 (MP)	Group 3 (PP)	Group 4 (M-)	
Screening					
Mean	991.4	998.5	983.0	968.8	985.0
Median	976.0	988.0	971.0	952.0	971.0
StdDev	176.5	173.1	168.6	142.4	165.2
StdErr	13.0	12.9	12.5	10.1	6.1
Min	633.0	613.0	582.0	683.0	582.0
Max	1615.0	1556.0	1613.0	1558.0	1615.0
N	183	181	181	199	744
Visit 2					
Mean	909.0	904.1	928.9	899.6	910.1
Median	893.0	900.5	936.0	885.0	900.5
StdDev	172.2	170.2	170.9	141.2	163.6
StdErr	12.8	12.8	12.8	10.0	6.0
Min	601.0	587.0	607.0	600.0	587.0
Max	1455.0	1525.0	1532.0	1275.0	1532.0
N	182	178	179	199	738
Visit 4					
Mean	923.3	893.8	919.6	-	912.3
Median	898.0	879.5	882.0	-	887.0
StdDev	164.7	151.0	165.5	-	160.8
StdErr	12.5	11.5	12.5	-	7.0
Min	603.0	549.0	596.0	-	549.0
Max	1453.0	1371.0	1510.0	-	1510.0
N	175	172	174	0	521

(Continued)

RR Interval

[ms]	Group				Total
	Group 1 (MM)	Group 2 (MP)	Group 3 (PP)	Group 4 (M-)	
Follow-Up					
Mean	1035.0	818.0	974.7	774.0	870.6
Median	1035.0	799.0	1065.0	796.0	847.0
StdDev	125.9	102.8	242.5	131.6	174.5
StdErr	89.0	59.4	140.0	58.9	48.4
Min	946.0	726.0	700.0	635.0	635.0
Max	1124.0	929.0	1159.0	943.0	1159.0
N	2	3	3	5	13

14.5 ADVERSE EVENT DATA

14.5.1 Unsolicted Adverse Events

14.5.1.1 Listings of deaths, other serious and significant adverse events

Group=Group 1 (MM)

Subj	Scr. no	Gender	Age (years)	Body weight (kg)	Adverse Event (Diagnosis)	Onset relative to most recent vaccination	Onset Date	Onset Time (hh:mm)	End Date	End Time (hh:mm)	Duration	Frequency	Serious	Intensity	Relation	Action	Outcome
148	293	female	27	56.9	NERVOUS BREAKDOWN	5 days	06AUG2006	NK:NK	12AUG2006	NK:NK	6 days	1	SAE	3	unlikely	6	1
287	519	female	28	63.0	SINUS TACHYCARDIA (102 BPM)	14 days	15SEP2006	UNKNOWN	15SEP2006	UNKNOWN	<= 1 day	1	SIAE	1	unlikely	1	1
317	488	male	45	89.0	TACHYCARDIA	31.0 days	09OCT2006	14:10	13FEB2007	UNKNOWN	127 days	-1	SIAE	1	unlikely	1	1
557	938	male	35	69.3	PALPITATIONS (FOR ABOUT 5 SECONDS). SUBJECT KNOWN PALPITATIONS FROM YEARS AGO DURING SPORTS, NOW WITHOUT SPORTS FOR THE FIRST TIME.	2 days	04OCT2006	EVENING	04OCT2006	EVENING	<= 1 day	1	SIAE	1	unlikely	1	1

Frequency: 1=Single episode, 2=Intermittent, 3=Continuous, -1=Unknown

Intensity: 1=mild, 2=moderate, 3=severe, 4=life threatening/diasabling, -1=Unknown

Action: 1=no action, 2=study drug dosage adjusted, 3=study drug discontinued, 4=concomitant medication, 5=non-drug therapy, 6=hospitalization, -1=Unknown

Outcome: 1=recovered without sequelae, 2=recovered with sequelae, 3=ongoing, 4=death, -1=unknown

Listings of deaths, other serious and significant adverse events

Group=Group 2 (MP)

Subj	Scr. no	Gender	Age (years)	Body weight (kg)	Adverse Event (Diagnosis)	Onset relative to most recent vaccination	Onset Date	Onset Time (hh:mm)	End Date	End Time (hh:mm)	Duration	Frequency	Serious	Intensity	Relation	Action	Outcome
220	316	female	26	58.9	PALPITATIONS	14.8 hrs	13JUL2006	7:30	13JUL2006	10:00	2.5 hrs	1	SIAE	2	possible	1	1
228	430	male	32	84.8	SINUS TACHYCARDIA	13 days	24AUG2006		24AUG2006	UNKNOWN	<= 1 day	3	SIAE	1	unlikely	1	1
536	926	female	38	62.0	TRANSIENT MOTORIC HEMIPARESIS (RIGHT SIDE)	5.7 days	03OCT2006	10:00	03OCT2006	12:45	2.8 hrs	1	SAE	3	unlikely	6	1
563	966	female	25	73.9	TACHYCARDIA (105 BPM)	28 days	21NOV2006	AFTERNOON	23NOV2006	14:00	2 days	-1	SIAE	1	possible	1	1

Frequency: 1=Single episode, 2=Intermittent, 3=Continuous, -1=Unknown

Intensity: 1=mild, 2=moderate, 3=severe, 4=life threatening/diasabling, -1=Unknown

Action: 1=no action, 2=study drug dosage adjusted, 3=study drug discontinued, 4=concomitant medication, 5=non-drug therapy, 6=hospitalization, -1=Unknown

Outcome: 1=recovered without sequelae, 2=recovered with sequelae, 3=ongoing, 4=death, -1=unknown

Listings of deaths, other serious and significant adverse events

Group=Group 2 (MP)

Subj	Scr. no	Gender	Age (years)	Body weight (kg)	Adverse Event (Diagnosis)	Onset relative to most recent vaccination	Onset Date	Onset Time (hh:mm)	End Date	End Time (hh:mm)	Duration	Frequency	Serious	Intensity	Relation	Action	Outcome
706	1170	male	18	70.2	RUPTURE OF EXTENSION TENDON 4TH FINGER (RING FINGER) LEFT HAND 29.12.2006, OP 30.12.2006, SUBJECT WAS IN HOSPITAL 30.-31.12.2006. CUT WITH KNIFE WHILE COOKING	25.3 days	29DEC2006	23:00	Ongoing	CONTINU ED		1	SAE	3	none	6	3

Frequency: 1=Single episode, 2=Intermittent, 3=Continuous, -1=Unknown
Intensity: 1=mild, 2=moderate, 3=severe, 4=life threatening/diasabling, -1=Unknown
Action: 1=no action, 2=study drug dosage adjusted, 3=study drug discontinued, 4=concomitant medication, 5=non-drug therapy, 6=hospitalization, -1=Unknown
Outcome: 1=recovered without sequelae, 2=recovered with sequelae, 3=ongoing, 4=death, -1=unknown

Listings of deaths, other serious and significant adverse events

Group=Group 3 (PP)

Subj	Scr. no	Gender	Age (years)	Body weight (kg)	Adverse Event (Diagnosis)	Onset relative to most recent vaccination	Onset Date	Onset Time (hh:mm)	End Date	End Time (hh:mm)	Duration	Frequency	Serious	Intensity	Relation	Action	Outcome
689	1133	male	30	82.0	PALPITATIONS	23 days	15NOV2006	EVENING	Ongoing	CONT		2	SIAE	1	unlikely	1	3

Frequency: 1=Single episode, 2=Intermittent, 3=Continuous, -1=Unknown

Intensity: 1=mild, 2=moderate, 3=severe, 4=life threatening/diasabling, -1=Unknown

Action: 1=no action, 2=study drug dosage adjusted, 3=study drug discontinued, 4=concomitant medication, 5=non-drug therapy, 6=hospitalization, -1=Unknown

Outcome: 1=recovered without sequelae, 2=recovered with sequelae, 3=ongoing, 4=death, -1=unknown

Listings of deaths, other serious and significant adverse events

Group=Group 4 (M-)

Subj	Scr. no	Gender	Age (years)	Body weight (kg)	Adverse Event (Diagnosis)	Onset relative to most recent vaccination	Onset Date	Onset Time (hh:mm)	End Date	End Time (hh:mm)	Duration	Frequency	Serious	Intensity	Relation	Action	Outcome
028	85	male	46	71.7	PALPITATIONS	3.1 hrs	23MAY2006	17:30	23MAY2006	18:30	1.0 hrs	1	SIAE	2	possible	1	1
		male	46	71.7	PALPITATIONS	3.0 days	26MAY2006	14:00	26MAY2006	EVENING	<= 1 day	1	SIAE	2	possible	4	1
397	682	female	37	56.8	TACHYCARDIA REPORTED BY SUBJECT	<= 1 day	28AUG2006	EVENING	31AUG2006	MORNING	3 days	1	SIAE	1	possible	1	1
411	683	female	47	53.8	PALPITATIONS	1.4 days	25AUG2006	1:00	25AUG2006	1:10	10 min	1	SIAE	1	unlikely	1	1

Frequency: 1=Single episode, 2=Intermittent, 3=Continuous, -1=Unknown

Intensity: 1=mild, 2=moderate, 3=severe, 4=life threatening/diasabling, -1=Unknown

Action: 1=no action, 2=study drug dosage adjusted, 3=study drug discontinued, 4=concomitant medication, 5=non-drug therapy, 6=hospitalization, -1=Unknown

Outcome: 1=recovered without sequelae, 2=recovered with sequelae, 3=ongoing, 4=death, -1=unknown

14.5.1.2 Narratives of deaths, other serious and certain other significant adverse events

No deaths or other serious and/or significant adverse events occurred in this trial. See also section 12.3.2.

14.5.1.3 Unsolicted Adverse Events: Overview, Safety dataset

	Group									
	Group 1 (MM)		Group 2 (MP)		Group 3 (PP)		Group 4 (M-)		All subjects	
	Count	%	Count	%	Count	%	Count	%	Count	%
Total no. of subjects included	183	24.6	181	24.3	181	24.3	200	26.8	745	100.0
No. of subjects with at least one AE	113	61.7	107	59.1	78	43.1	99	49.5	397	53.3
No. of subjects with at least one drug-related[1] AE	61	33.3	61	33.7	23	12.7	74	37.0	219	29.4
No. of subjects with no AE	70	38.3	74	40.9	103	56.9	101	50.5	348	46.7
No. of subjects with at least one SAE	1	0.5	2	1.1	-	-	-	-	3	0.4
No. of subjects with at least one related SAE	-	-	-	-	-	-	-	-	-	-
No. of subjects with at least one SIAE	3	1.6	3	1.7	1	0.6	3	1.5	10	1.3
No. of subjects with at least one related SIAE	-	-	2	1.1	-	-	2	1.0	4	0.5
No. of verbatim reported AEs	241	33.6	188	26.2	136	18.9	153	21.3	718	100.0

(Continued)

Percentages for 'No. of subjects with...' are based on the total number of subjects included per treatment

[1]Drug related: definite, probable, possible or unknown
SIAE: special interest adverse events
[2]: This includes all coded symptoms including multiple occurrences of the same PT within one subject
Splitting of verbatim reported AEs possible.

[3]: A subject was only included once in each PT
(i.e. this represents the number of subjects with at least one event for the same PT)

Unsolicted Adverse Events: Overview, Safety dataset

	Group									
	Group 1 (MM)		Group 2 (MP)		Group 3 (PP)		Group 4 (M-)		All subjects	
	Count	%	Count	%	Count	%	Count	%	Count	%
No. of reported SAEs	1	33.3	2	66.7	-	-	-	-	3	100.0
No. of reported SIAEs	3	27.3	3	27.3	1	9.1	4	36.4	11	100.0
No. of coded MedDRA PTs[2]	260	34.5	197	26.2	141	18.7	155	20.6	753	100.0
No. of PTs by subject[3]	229	32.3	193	27.3	135	19.1	151	21.3	708	100.0

Percentages for 'No. of subjects with...' are based on the total number of subjects included per treatment

[1]Drug related: definite, probable, possible or unknown
SIAE: special interest adverse events
[2]: This includes all coded symptoms including multiple occurrences of the same PT within one subject
Splitting of verbatim reported AEs possible.

[3]: A subject was only included once in each PT
(i.e. this represents the number of subjects with at least one event for the same PT)

14.5.1.4 Unsolicited adverse events: MedDRA coding by SOC, PT and subject, Safety dataset

SOC<br>Preferred Term	Group 1 (MM) (N=183)		Group 2 (MP) (N=181)		Group 3 (PP) (N=181)		Group 4 (M-) (N=200)		Total (N=745)		p-value
	Number of Subjects (%) - Number of events										
General disorders and administration site conditions											
Any PT	55 (30.1)	93	49 (27.1)	60	15 (8.3)	16	57 (28.5)	70	176 (23.6)	239	<0.001
Injection site pruritus	43 (23.5)	61	36 (19.9)	37	4 (2.2)	4	49 (24.5)	49	132 (17.7)	151	<0.001
Injection site haematoma	6 (3.3)	6	9 (5.0)	9	5 (2.8)	5	4 (2.0)	4	24 (3.2)	24	0.431
Injection site warmth	9 (4.9)	10	5 (2.8)	5	0 (0.0)	0	8 (4.0)	8	22 (3.0)	23	0.009
Asthenia	2 (1.1)	2	0 (0.0)	0	2 (1.1)	2	1 (0.5)	1	5 (0.7)	5	0.576
Injection site discolouration	1 (0.5)	1	4 (2.2)	4	0 (0.0)	0	0 (0.0)	0	5 (0.7)	5	0.021
Injection site irritation	1 (0.5)	2	1 (0.6)	1	0 (0.0)	0	3 (1.5)	3	5 (0.7)	6	0.413
Axillary pain	1 (0.5)	1	1 (0.6)	1	0 (0.0)	0	1 (0.5)	1	3 (0.4)	3	>0.999
Injection site hypersensitivity	1 (0.5)	1	0 (0.0)	0	1 (0.6)	1	0 (0.0)	0	2 (0.3)	2	0.607
Injection site reaction	1 (0.5)	1	0 (0.0)	0	0 (0.0)	0	1 (0.5)	1	2 (0.3)	2	>0.999
Pyrexia	1 (0.5)	1	1 (0.6)	1	0 (0.0)	0	0 (0.0)	0	2 (0.3)	2	0.607
Thirst	0 (0.0)	0	1 (0.6)	1	0 (0.0)	0	1 (0.5)	1	2 (0.3)	2	0.868
Chest discomfort	0 (0.0)	0	1 (0.6)	1	0 (0.0)	0	0 (0.0)	0	1 (0.1)	1	0.486
Chest pain	1 (0.5)	1	0 (0.0)	0	0 (0.0)	0	0 (0.0)	0	1 (0.1)	1	0.732
Chills	1 (0.5)	1	0 (0.0)	0	0 (0.0)	0	0 (0.0)	0	1 (0.1)	1	0.732
Feeling hot	0 (0.0)	0	0 (0.0)	0	0 (0.0)	0	1 (0.5)	1	1 (0.1)	1	>0.999
Implant site pain	1 (0.5)	1	0 (0.0)	0	0 (0.0)	0	0 (0.0)	0	1 (0.1)	1	0.732
Induration	0 (0.0)	0	0 (0.0)	0	1 (0.6)	1	0 (0.0)	0	1 (0.1)	1	0.486
Influenza like illness	1 (0.5)	1	0 (0.0)	0	0 (0.0)	0	0 (0.0)	0	1 (0.1)	1	0.732
Injection site erythema	1 (0.5)	1	0 (0.0)	0	0 (0.0)	0	0 (0.0)	0	1 (0.1)	1	0.732
Injection site paraesthesia	1 (0.5)	1	0 (0.0)	0	0 (0.0)	0	0 (0.0)	0	1 (0.1)	1	0.732
Injection site urticaria	0 (0.0)	0	0 (0.0)	0	1 (0.6)	1	0 (0.0)	0	1 (0.1)	1	0.486
Malaise	0 (0.0)	0	0 (0.0)	0	0 (0.0)	0	1 (0.5)	1	1 (0.1)	1	>0.999
Non-cardiac chest pain	1 (0.5)	1	0 (0.0)	0	0 (0.0)	0	0 (0.0)	0	1 (0.1)	1	0.732
Pain	0 (0.0)	0	0 (0.0)	0	1 (0.6)	1	0 (0.0)	0	1 (0.1)	1	0.486
Soft tissue inflammation	0 (0.0)	0	0 (0.0)	0	1 (0.6)	1	0 (0.0)	0	1 (0.1)	1	0.486

A subject with more than one finding in a specific category was only counted once
Percentages based on total no. of subjects in each group
Table is sorted by descending subject count for the total of all treatment groups
p-value from Fisher's Exact test to detect any global difference between all treatment groups

Unsolicited adverse events: MedDRA coding by SOC, PT and subject, Safety dataset

SOC<br>Preferred Term	Group 1 (MM) (N=183)		Group 2 (MP) (N=181)		Group 3 (PP) (N=181)		Group 4 (M-) (N=200)		Total (N=745)		p-value
	Number of Subjects (%)	Number of events	Number of Subjects (%)	Number of events	Number of Subjects (%)	Number of events	Number of Subjects (%)	Number of events	Number of Subjects (%)	Number of events	
Infections and infestations											
Any PT	54 (29.5)	64	38 (21.0)	42	36 (19.9)	45	12 (6.0)	15	140 (18.8)	166	<0.001
Nasopharyngitis	42 (23.0)	45	20 (11.0)	20	26 (14.4)	28	4 (2.0)	4	92 (12.3)	97	<0.001
Bronchitis	3 (1.6)	3	4 (2.2)	4	2 (1.1)	2	1 (0.5)	1	10 (1.3)	10	0.469
Tonsillitis	4 (2.2)	4	5 (2.8)	5	1 (0.6)	1	0 (0.0)	0	10 (1.3)	10	0.034
Rhinitis	3 (1.6)	3	1 (0.6)	1	4 (2.2)	4	1 (0.5)	1	9 (1.2)	9	0.396
Cystitis	1 (0.5)	1	2 (1.1)	2	1 (0.6)	1	1 (0.5)	1	5 (0.7)	5	0.879
Herpes simplex	2 (1.1)	2	2 (1.1)	2	1 (0.6)	1	0 (0.0)	0	5 (0.7)	5	0.490
Sinusitis	2 (1.1)	2	1 (0.6)	1	1 (0.6)	1	1 (0.5)	1	5 (0.7)	5	0.937
Gastroenteritis	1 (0.5)	1	1 (0.6)	1	1 (0.6)	1	0 (0.0)	0	3 (0.4)	3	0.712
Otitis media	0 (0.0)	0	0 (0.0)	0	1 (0.6)	1	2 (1.0)	2	3 (0.4)	3	0.571
Pharyngitis	0 (0.0)	0	1 (0.6)	1	1 (0.6)	1	1 (0.5)	1	3 (0.4)	3	0.807
Otitis externa	1 (0.5)	1	0 (0.0)	0	0 (0.0)	0	1 (0.5)	1	2 (0.3)	2	>0.999
Vulvovaginal mycotic infection	0 (0.0)	0	0 (0.0)	0	1 (0.6)	2	1 (0.5)	1	2 (0.3)	3	0.868
Acute tonsillitis	0 (0.0)	0	1 (0.6)	1	0 (0.0)	0	0 (0.0)	0	1 (0.1)	1	0.486
Gastrointestinal infection	0 (0.0)	0	1 (0.6)	1	0 (0.0)	0	0 (0.0)	0	1 (0.1)	1	0.486
Herpes ophthalmic	1 (0.5)	1	0 (0.0)	0	0 (0.0)	0	0 (0.0)	0	1 (0.1)	1	0.732
Laryngitis	0 (0.0)	0	0 (0.0)	0	0 (0.0)	0	1 (0.5)	1	1 (0.1)	1	>0.999
Localised infection	0 (0.0)	0	0 (0.0)	0	1 (0.6)	1	0 (0.0)	0	1 (0.1)	1	0.486
Oophoritis	0 (0.0)	0	0 (0.0)	0	1 (0.6)	1	0 (0.0)	0	1 (0.1)	1	0.486
Sinobronchitis	0 (0.0)	0	1 (0.6)	1	0 (0.0)	0	0 (0.0)	0	1 (0.1)	1	0.486
Tinea pedis	0 (0.0)	0	0 (0.0)	0	0 (0.0)	0	1 (0.5)	1	1 (0.1)	1	>0.999
Urethritis	0 (0.0)	0	1 (0.6)	2	0 (0.0)	0	0 (0.0)	0	1 (0.1)	2	0.486
Urinary tract infection	1 (0.5)	1	0 (0.0)	0	0 (0.0)	0	0 (0.0)	0	1 (0.1)	1	0.732
Nervous system disorders											
Any PT	16 (8.7)	18	25 (13.8)	27	14 (7.7)	15	11 (5.5)	12	66 (8.9)	72	0.042

A subject with more than one finding in a specific category was only counted once
Percentages based on total no. of subjects in each group
Table is sorted by descending subject count for the total of all treatment groups
p-value from Fisher's Exact test to detect any global difference between all treatment groups

Unsolicited adverse events: MedDRA coding by SOC, PT and subject, Safety dataset

SOC<br>Preferred Term	Group 1 (MM)<br>(N=183)	Group 2 (MP)<br>(N=181)	Group 3 (PP)<br>(N=181)	Group 4 (M-)<br>(N=200)	Total<br>(N=745)	p-value
	Number of Subjects (%) - Number of events					
Headache	10 (5.5) 11	16 (8.8) 17	11 (6.1) 11	6 (3.0) 6	43 (5.8) 45	0.108
Dizziness	2 (1.1) 3	7 (3.9) 7	2 (1.1) 3	4 (2.0) 4	15 (2.0) 17	0.234
Tremor	1 (0.5) 1	1 (0.6) 1	0 (0.0) 0	0 (0.0) 0	2 (0.3) 2	0.607
Disturbance in attention	0 (0.0) 0	0 (0.0) 0	0 (0.0) 0	1 (0.5) 1	1 (0.1) 1	>0.999
Hemiparesis	0 (0.0) 0	1 (0.6) 1	0 (0.0) 0	0 (0.0) 0	1 (0.1) 1	0.486
Hypoaesthesia	1 (0.5) 1	0 (0.0) 0	0 (0.0) 0	0 (0.0) 0	1 (0.1) 1	0.732
Migraine	1 (0.5) 1	0 (0.0) 0	0 (0.0) 0	0 (0.0) 0	1 (0.1) 1	0.732
Paraesthesia	0 (0.0) 0	1 (0.6) 1	0 (0.0) 0	0 (0.0) 0	1 (0.1) 1	0.486
Sciatica	0 (0.0) 0	0 (0.0) 0	1 (0.6) 1	0 (0.0) 0	1 (0.1) 1	0.486
Sedation	0 (0.0) 0	0 (0.0) 0	0 (0.0) 0	1 (0.5) 1	1 (0.1) 1	>0.999
Syncope	1 (0.5) 1	0 (0.0) 0	0 (0.0) 0	0 (0.0) 0	1 (0.1) 1	0.732
Gastrointestinal disorders						
Any PT	12 (6.6) 13	12 (6.6) 16	12 (6.6) 15	13 (6.5) 14	49 (6.6) 58	>0.999
Diarrhoea	3 (1.6) 3	4 (2.2) 4	3 (1.7) 3	6 (3.0) 6	16 (2.1) 16	0.801
Abdominal pain	3 (1.6) 3	3 (1.7) 3	1 (0.6) 1	0 (0.0) 0	7 (0.9) 7	0.203
Toothache	2 (1.1) 2	1 (0.6) 1	2 (1.1) 3	2 (1.0) 2	7 (0.9) 8	>0.999
Abdominal pain upper	1 (0.5) 1	1 (0.6) 1	4 (2.2) 4	0 (0.0) 0	6 (0.8) 6	0.103
Dry mouth	2 (1.1) 3	0 (0.0) 0	0 (0.0) 0	1 (0.5) 1	3 (0.4) 4	0.466
Nausea	1 (0.5) 1	2 (1.1) 2	0 (0.0) 0	0 (0.0) 0	3 (0.4) 3	0.235
Dysphagia	0 (0.0) 0	0 (0.0) 0	0 (0.0) 0	2 (1.0) 2	2 (0.3) 2	0.249
Gastritis	0 (0.0) 0	0 (0.0) 0	2 (1.1) 2	0 (0.0) 0	2 (0.3) 2	0.118
Vomiting	0 (0.0) 0	2 (1.1) 2	0 (0.0) 0	0 (0.0) 0	2 (0.3) 2	0.118
Abdominal pain lower	0 (0.0) 0	0 (0.0) 0	0 (0.0) 0	1 (0.5) 1	1 (0.1) 1	>0.999
Cheilitis	0 (0.0) 0	1 (0.6) 1	0 (0.0) 0	0 (0.0) 0	1 (0.1) 1	0.486
Colitis	0 (0.0) 0	0 (0.0) 0	1 (0.6) 1	0 (0.0) 0	1 (0.1) 1	0.486
Constipation	0 (0.0) 0	0 (0.0) 0	0 (0.0) 0	1 (0.5) 1	1 (0.1) 1	>0.999

A subject with more than one finding in a specific category was only counted once
Percentages based on total no. of subjects in each group
Table is sorted by descending subject count for the total of all treatment groups
p-value from Fisher's Exact test to detect any global difference between all treatment groups

Unsolicited adverse events: MedDRA coding by SOC, PT and subject, Safety dataset

SOC<br>Preferred Term	Group 1 (MM)<br>(N=183)		Group 2 (MP)<br>(N=181)		Group 3 (PP)<br>(N=181)		Group 4 (M-)<br>(N=200)		Total<br>(N=745)		p-value
	Number of Subjects (%) - Number of events										
Dyspepsia	0 (0.0)	0	0 (0.0)	0	1 (0.6)	1	0 (0.0)	0	1 (0.1)	1	0.486
Flatulence	0 (0.0)	0	1 (0.6)	2	0 (0.0)	0	0 (0.0)	0	1 (0.1)	2	0.486
Sensitivity of teeth	0 (0.0)	0	0 (0.0)	0	0 (0.0)	0	1 (0.5)	1	1 (0.1)	1	>0.999
Respiratory, thoracic and mediastinal disorders											
Any PT	16 (8.7)	18	12 (6.6)	12	10 (5.5)	12	6 (3.0)	6	44 (5.9)	48	0.104
Pharyngolaryngeal pain	11 (6.0)	11	9 (5.0)	9	8 (4.4)	9	4 (2.0)	4	32 (4.3)	33	0.221
Cough	5 (2.7)	5	1 (0.6)	1	3 (1.7)	3	0 (0.0)	0	9 (1.2)	9	0.046
Sneezing	0 (0.0)	0	1 (0.6)	1	0 (0.0)	0	1 (0.5)	1	2 (0.3)	2	0.868
Dry throat	0 (0.0)	0	0 (0.0)	0	0 (0.0)	0	1 (0.5)	1	1 (0.1)	1	>0.999
Dysphonia	0 (0.0)	0	1 (0.6)	1	0 (0.0)	0	0 (0.0)	0	1 (0.1)	1	0.486
Hyperventilation	1 (0.5)	1	0 (0.0)	0	0 (0.0)	0	0 (0.0)	0	1 (0.1)	1	0.732
Rhinorrhoea	1 (0.5)	1	0 (0.0)	0	0 (0.0)	0	0 (0.0)	0	1 (0.1)	1	0.732
Musculoskeletal and connective tissue disorders											
Any PT	13 (7.1)	13	4 (2.2)	4	12 (6.6)	12	10 (5.0)	12	39 (5.2)	41	0.114
Back pain	4 (2.2)	4	1 (0.6)	1	5 (2.8)	5	2 (1.0)	2	12 (1.6)	12	0.316
Arthralgia	0 (0.0)	0	0 (0.0)	0	3 (1.7)	3	1 (0.5)	1	4 (0.5)	4	0.130
Muscular weakness	2 (1.1)	2	0 (0.0)	0	1 (0.6)	1	1 (0.5)	1	4 (0.5)	4	0.751
Musculoskeletal stiffness	3 (1.6)	3	0 (0.0)	0	0 (0.0)	0	1 (0.5)	1	4 (0.5)	4	0.146
Muscle spasms	1 (0.5)	1	0 (0.0)	0	0 (0.0)	0	1 (0.5)	2	2 (0.3)	3	>0.999
Myalgia	1 (0.5)	1	0 (0.0)	0	1 (0.6)	1	0 (0.0)	0	2 (0.3)	2	0.607
Neck pain	1 (0.5)	1	0 (0.0)	0	1 (0.6)	1	0 (0.0)	0	2 (0.3)	2	0.607
Pain in extremity	1 (0.5)	1	1 (0.6)	1	0 (0.0)	0	0 (0.0)	0	2 (0.3)	2	0.607
Arthritis	0 (0.0)	0	0 (0.0)	0	1 (0.6)	1	0 (0.0)	0	1 (0.1)	1	0.486
Flank pain	0 (0.0)	0	0 (0.0)	0	0 (0.0)	0	1 (0.5)	1	1 (0.1)	1	>0.999
Intervertebral disc protrusion	0 (0.0)	0	0 (0.0)	0	0 (0.0)	0	1 (0.5)	1	1 (0.1)	1	>0.999

A subject with more than one finding in a specific category was only counted once
Percentages based on total no. of subjects in each group
Table is sorted by descending subject count for the total of all treatment groups
p-value from Fisher's Exact test to detect any global difference between all treatment groups

Unsolicited adverse events: MedDRA coding by SOC, PT and subject, Safety dataset

SOC<br>Preferred Term	Group 1 (MM)<br>(N=183)		Group 2 (MP)<br>(N=181)		Group 3 (PP)<br>(N=181)		Group 4 (M-)<br>(N=200)		Total<br>(N=745)		p-value
	Number of Subjects (%) - Number of events										
Muscle tightness	0 (0.0)	0	1 (0.6)	1	0 (0.0)	0	0 (0.0)	0	1 (0.1)	1	0.486
Muscle twitching	0 (0.0)	0	0 (0.0)	0	0 (0.0)	0	1 (0.5)	1	1 (0.1)	1	>0.999
Musculoskeletal chest pain	0 (0.0)	0	0 (0.0)	0	0 (0.0)	0	1 (0.5)	1	1 (0.1)	1	>0.999
Pain in jaw	0 (0.0)	0	0 (0.0)	0	0 (0.0)	0	1 (0.5)	1	1 (0.1)	1	>0.999
Sensation of heaviness	0 (0.0)	0	1 (0.6)	1	0 (0.0)	0	0 (0.0)	0	1 (0.1)	1	0.486
Blood and lymphatic system disorders											
Any PT	5 (2.7)	7	7 (3.9)	7	2 (1.1)	2	4 (2.0)	4	18 (2.4)	20	0.376
Lymphadenopathy	4 (2.2)	5	4 (2.2)	4	2 (1.1)	2	4 (2.0)	4	14 (1.9)	15	0.890
Iron deficiency anaemia	0 (0.0)	0	2 (1.1)	2	0 (0.0)	0	0 (0.0)	0	2 (0.3)	2	0.118
Lymph node pain	2 (1.1)	2	0 (0.0)	0	0 (0.0)	0	0 (0.0)	0	2 (0.3)	2	0.178
Lymphadenitis	0 (0.0)	0	1 (0.6)	1	0 (0.0)	0	0 (0.0)	0	1 (0.1)	1	0.486
Injury, poisoning and procedural complications											
Any PT	5 (2.7)	5	6 (3.3)	7	6 (3.3)	6	1 (0.5)	1	18 (2.4)	19	0.140
Contusion	1 (0.5)	1	2 (1.1)	2	0 (0.0)	0	0 (0.0)	0	3 (0.4)	3	0.235
Limb injury	0 (0.0)	0	1 (0.6)	1	2 (1.1)	2	0 (0.0)	0	3 (0.4)	3	0.148
Arthropod bite	0 (0.0)	0	0 (0.0)	0	1 (0.6)	1	1 (0.5)	1	2 (0.3)	2	0.868
Excoriation	0 (0.0)	0	1 (0.6)	1	1 (0.6)	1	0 (0.0)	0	2 (0.3)	2	0.368
Procedural pain	0 (0.0)	0	1 (0.6)	1	1 (0.6)	1	0 (0.0)	0	2 (0.3)	2	0.368
Skin laceration	2 (1.1)	2	0 (0.0)	0	0 (0.0)	0	0 (0.0)	0	2 (0.3)	2	0.178
Back injury	1 (0.5)	1	0 (0.0)	0	0 (0.0)	0	0 (0.0)	0	1 (0.1)	1	0.732
Joint dislocation	0 (0.0)	0	0 (0.0)	0	1 (0.6)	1	0 (0.0)	0	1 (0.1)	1	0.486
Road traffic accident	0 (0.0)	0	1 (0.6)	1	0 (0.0)	0	0 (0.0)	0	1 (0.1)	1	0.486
Skin injury	1 (0.5)	1	0 (0.0)	0	0 (0.0)	0	0 (0.0)	0	1 (0.1)	1	0.732
Tendon injury	0 (0.0)	0	1 (0.6)	1	0 (0.0)	0	0 (0.0)	0	1 (0.1)	1	0.486

A subject with more than one finding in a specific category was only counted once
Percentages based on total no. of subjects in each group
Table is sorted by descending subject count for the total of all treatment groups
p-value from Fisher's Exact test to detect any global difference between all treatment groups

Unsolicited adverse events: MedDRA coding by SOC, PT and subject, Safety dataset

SOC / Preferred Term	Group 1 (MM) (N=183)		Group 2 (MP) (N=181)		Group 3 (PP) (N=181)		Group 4 (M-) (N=200)		Total (N=745)		p-value
	Number of Subjects (%) - Number of events										
Skin and subcutaneous tissue disorders											
Any PT	3 (1.6)	7	7 (3.9)	7	4 (2.2)	4	4 (2.0)	6	18 (2.4)	24	0.585
Eczema	1 (0.5)	3	1 (0.6)	1	1 (0.6)	1	0 (0.0)	0	3 (0.4)	5	0.712
Pruritus	1 (0.5)	1	1 (0.6)	1	1 (0.6)	1	0 (0.0)	0	3 (0.4)	3	0.712
Rash	0 (0.0)	0	2 (1.1)	2	0 (0.0)	0	1 (0.5)	2	3 (0.4)	4	0.418
Dermatitis contact	0 (0.0)	0	1 (0.6)	1	1 (0.6)	1	0 (0.0)	0	2 (0.3)	2	0.368
Hyperhidrosis	0 (0.0)	0	0 (0.0)	0	0 (0.0)	0	2 (1.0)	3	2 (0.3)	3	0.249
Acne	0 (0.0)	0	0 (0.0)	0	0 (0.0)	0	1 (0.5)	1	1 (0.1)	1	>0.999
Dermatitis	0 (0.0)	0	1 (0.6)	1	0 (0.0)	0	0 (0.0)	0	1 (0.1)	1	0.486
Erythema	0 (0.0)	0	1 (0.6)	1	0 (0.0)	0	0 (0.0)	0	1 (0.1)	1	0.486
Photodermatosis	1 (0.5)	1	0 (0.0)	0	0 (0.0)	0	0 (0.0)	0	1 (0.1)	1	0.732
Rash papular	1 (0.5)	2	0 (0.0)	0	0 (0.0)	0	0 (0.0)	0	1 (0.1)	2	0.732
Skin reaction	0 (0.0)	0	0 (0.0)	0	1 (0.6)	1	0 (0.0)	0	1 (0.1)	1	0.486
Cardiac disorders											
Any PT	4 (2.2)	4	4 (2.2)	4	3 (1.7)	3	3 (1.5)	4	14 (1.9)	15	0.950
Palpitations	2 (1.1)	2	2 (1.1)	2	2 (1.1)	2	2 (1.0)	3	8 (1.1)	9	>0.999
Tachycardia	1 (0.5)	1	1 (0.6)	1	0 (0.0)	0	1 (0.5)	1	3 (0.4)	3	>0.999
Sinus tachycardia	1 (0.5)	1	1 (0.6)	1	0 (0.0)	0	0 (0.0)	0	2 (0.3)	2	0.607
Pericardial effusion	0 (0.0)	0	0 (0.0)	0	1 (0.6)	1	0 (0.0)	0	1 (0.1)	1	0.486
Reproductive system and breast disorders											
Any PT	4 (2.2)	4	1 (0.6)	1	2 (1.1)	2	2 (1.0)	2	9 (1.2)	9	0.600
Dysmenorrhoea	3 (1.6)	3	1 (0.6)	1	2 (1.1)	2	0 (0.0)	0	6 (0.8)	6	0.254
Metrorrhagia	1 (0.5)	1	0 (0.0)	0	0 (0.0)	0	1 (0.5)	1	2 (0.3)	2	>0.999
Menstruation irregular	0 (0.0)	0	0 (0.0)	0	0 (0.0)	0	1 (0.5)	1	1 (0.1)	1	>0.999

A subject with more than one finding in a specific category was only counted once
Percentages based on total no. of subjects in each group
Table is sorted by descending subject count for the total of all treatment groups
p-value from Fisher's Exact test to detect any global difference between all treatment groups

Unsolicited adverse events: MedDRA coding by SOC, PT and subject, Safety dataset

SOC<br>Preferred Term	Group 1 (MM)<br>(N=183)		Group 2 (MP)<br>(N=181)		Group 3 (PP)<br>(N=181)		Group 4 (M-)<br>(N=200)		Total<br>(N=745)		p-value
	Number of Subjects (%) - Number of events										
Surgical and medical procedures											
Any PT	2 (1.1)	2	3 (1.7)	3	2 (1.1)	2	2 (1.0)	2	9 (1.2)	9	0.940
Tooth extraction	0 (0.0)	0	1 (0.6)	1	0 (0.0)	0	1 (0.5)	1	2 (0.3)	2	0.868
Dental treatment	0 (0.0)	0	0 (0.0)	0	0 (0.0)	0	1 (0.5)	1	1 (0.1)	1	>0.999
Meniscus operation	0 (0.0)	0	0 (0.0)	0	1 (0.6)	1	0 (0.0)	0	1 (0.1)	1	0.486
Nasal operation	1 (0.5)	1	0 (0.0)	0	0 (0.0)	0	0 (0.0)	0	1 (0.1)	1	0.732
Removal of internal fixation	1 (0.5)	1	0 (0.0)	0	0 (0.0)	0	0 (0.0)	0	1 (0.1)	1	0.732
Tooth repair	0 (0.0)	0	1 (0.6)	1	0 (0.0)	0	0 (0.0)	0	1 (0.1)	1	0.486
Varicose vein operation	0 (0.0)	0	0 (0.0)	0	1 (0.6)	1	0 (0.0)	0	1 (0.1)	1	0.486
Wisdom teeth removal	0 (0.0)	0	1 (0.6)	1	0 (0.0)	0	0 (0.0)	0	1 (0.1)	1	0.486
Vascular disorders											
Any PT	2 (1.1)	2	1 (0.6)	1	1 (0.6)	1	5 (2.5)	5	9 (1.2)	9	0.363
Hot flush	0 (0.0)	0	0 (0.0)	0	0 (0.0)	0	4 (2.0)	4	4 (0.5)	4	0.015
Haemorrhage	1 (0.5)	1	1 (0.6)	1	0 (0.0)	0	0 (0.0)	0	2 (0.3)	2	0.607
Flushing	0 (0.0)	0	0 (0.0)	0	1 (0.6)	1	0 (0.0)	0	1 (0.1)	1	0.486
Haematoma	1 (0.5)	1	0 (0.0)	0	0 (0.0)	0	0 (0.0)	0	1 (0.1)	1	0.732
Hypertension	0 (0.0)	0	0 (0.0)	0	0 (0.0)	0	1 (0.5)	1	1 (0.1)	1	>0.999
Psychiatric disorders											
Any PT	2 (1.1)	3	3 (1.7)	3	2 (1.1)	2	1 (0.5)	1	8 (1.1)	9	0.698
Insomnia	0 (0.0)	0	2 (1.1)	2	0 (0.0)	0	0 (0.0)	0	2 (0.3)	2	0.118
Sleep disorder	1 (0.5)	2	0 (0.0)	0	1 (0.6)	1	0 (0.0)	0	2 (0.3)	3	0.607
Disorientation	0 (0.0)	0	1 (0.6)	1	0 (0.0)	0	0 (0.0)	0	1 (0.1)	1	0.486
Initial insomnia	0 (0.0)	0	0 (0.0)	0	1 (0.6)	1	0 (0.0)	0	1 (0.1)	1	0.486
Mental disorder	1 (0.5)	1	0 (0.0)	0	0 (0.0)	0	0 (0.0)	0	1 (0.1)	1	0.732
Restlessness	0 (0.0)	0	0 (0.0)	0	0 (0.0)	0	1 (0.5)	1	1 (0.1)	1	>0.999

A subject with more than one finding in a specific category was only counted once
Percentages based on total no. of subjects in each group
Table is sorted by descending subject count for the total of all treatment groups
p-value from Fisher's Exact test to detect any global difference between all treatment groups

Unsolicited adverse events: MedDRA coding by SOC, PT and subject, Safety dataset

SOC<br>Preferred Term	Group 1 (MM)<br>(N=183)		Group 2 (MP)<br>(N=181)		Group 3 (PP)<br>(N=181)		Group 4 (M-)<br>(N=200)		Total<br>(N=745)		p-value
	Number of Subjects (%) - Number of events										
Eye disorders											
Any PT	1 (0.5)	1	1 (0.6)	1	2 (1.1)	2	1 (0.5)	1	5 (0.7)	5	0.879
Abnormal sensation in eye	0 (0.0)	0	0 (0.0)	0	0 (0.0)	0	1 (0.5)	1	1 (0.1)	1	>0.999
Blepharospasm	1 (0.5)	1	0 (0.0)	0	0 (0.0)	0	0 (0.0)	0	1 (0.1)	1	0.732
Conjunctivitis	0 (0.0)	0	0 (0.0)	0	1 (0.6)	1	0 (0.0)	0	1 (0.1)	1	0.486
Dry eye	0 (0.0)	0	0 (0.0)	0	1 (0.6)	1	0 (0.0)	0	1 (0.1)	1	0.486
Visual disturbance	0 (0.0)	0	1 (0.6)	1	0 (0.0)	0	0 (0.0)	0	1 (0.1)	1	0.486
Ear and labyrinth disorders											
Any PT	3 (1.6)	3	1 (0.6)	1	0 (0.0)	0	0 (0.0)	0	4 (0.5)	4	0.100
Ear pain	2 (1.1)	2	0 (0.0)	0	0 (0.0)	0	0 (0.0)	0	2 (0.3)	2	0.178
Vertigo	1 (0.5)	1	1 (0.6)	1	0 (0.0)	0	0 (0.0)	0	2 (0.3)	2	0.607
Investigations											
Any PT	1 (0.5)	1	0 (0.0)	0	1 (0.6)	1	0 (0.0)	0	2 (0.3)	2	0.607
Blood potassium increased	0 (0.0)	0	0 (0.0)	0	1 (0.6)	1	0 (0.0)	0	1 (0.1)	1	0.486
Lymph node palpable	1 (0.5)	1	0 (0.0)	0	0 (0.0)	0	0 (0.0)	0	1 (0.1)	1	0.732
Metabolism and nutrition disorders											
Any PT	1 (0.5)	1	0 (0.0)	0	1 (0.6)	1	0 (0.0)	0	2 (0.3)	2	0.607
Increased appetite	1 (0.5)	1	0 (0.0)	0	0 (0.0)	0	0 (0.0)	0	1 (0.1)	1	0.732
Polydipsia	0 (0.0)	0	0 (0.0)	0	1 (0.6)	1	0 (0.0)	0	1 (0.1)	1	0.486
Endocrine disorders											
Any PT	1 (0.5)	1	0 (0.0)	0	0 (0.0)	0	0 (0.0)	0	1 (0.1)	1	0.732
Hypothyroidism	1 (0.5)	1	0 (0.0)	0	0 (0.0)	0	0 (0.0)	0	1 (0.1)	1	0.732

A subject with more than one finding in a specific category was only counted once
Percentages based on total no. of subjects in each group
Table is sorted by descending subject count for the total of all treatment groups
p-value from Fisher's Exact test to detect any global difference between all treatment groups

Unsolicited adverse events: MedDRA coding by SOC, PT and subject, Safety dataset

SOC<br>Preferred Term	Group 1 (MM)<br>(N=183)	Group 2 (MP)<br>(N=181)	Group 3 (PP)<br>(N=181)	Group 4 (M-)<br>(N=200)	Total<br>(N=745)	p-value
	Number of Subjects (%) - Number of events					
Immune system disorders						
Any PT	0 (0.0) 0	1 (0.6) 1	0 (0.0) 0	0 (0.0) 0	1 (0.1) 1	0.486
Hypersensitivity	0 (0.0) 0	1 (0.6) 1	0 (0.0) 0	0 (0.0) 0	1 (0.1) 1	0.486

A subject with more than one finding in a specific category was only counted once
Percentages based on total no. of subjects in each group
Table is sorted by descending subject count for the total of all treatment groups
p-value from Fisher's Exact test to detect any global difference between all treatment groups

14.5.1.5 Unsolicited adverse events: MedDRA coding by SOC and subject, Safety dataset

SOC	Group 1 (MM) (N=183)		Group 2 (MP) (N=181)		Group 3 (PP) (N=181)		Group 4 (M-) (N=200)		Total (N=745)	
	Number of Subjects (%) - Number of events									
Any AE	113 (61.7)	260	107 (59.1)	197	78 (43.1)	141	99 (49.5)	155	397 (53.3)	753
General disorders and administration site conditions	55 (30.1)	93	49 (27.1)	60	15 (8.3)	16	57 (28.5)	70	176 (23.6)	239
Infections and infestations	54 (29.5)	64	38 (21.0)	42	36 (19.9)	45	12 (6.0)	15	140 (18.8)	166
Nervous system disorders	16 (8.7)	18	25 (13.8)	27	14 (7.7)	15	11 (5.5)	12	66 (8.9)	72
Gastrointestinal disorders	12 (6.6)	13	12 (6.6)	16	12 (6.6)	15	13 (6.5)	14	49 (6.6)	58
Respiratory, thoracic and mediastinal disorders	16 (8.7)	18	12 (6.6)	12	10 (5.5)	12	6 (3.0)	6	44 (5.9)	48
Musculoskeletal and connective tissue disorders	13 (7.1)	13	4 (2.2)	4	12 (6.6)	12	10 (5.0)	12	39 (5.2)	41
Blood and lymphatic system disorders	5 (2.7)	7	7 (3.9)	7	2 (1.1)	2	4 (2.0)	4	18 (2.4)	20
Injury, poisoning and procedural complications	5 (2.7)	5	6 (3.3)	7	6 (3.3)	6	1 (0.5)	1	18 (2.4)	19
Skin and subcutaneous tissue disorders	3 (1.6)	7	7 (3.9)	7	4 (2.2)	4	4 (2.0)	6	18 (2.4)	24
Cardiac disorders	4 (2.2)	4	4 (2.2)	4	3 (1.7)	3	3 (1.5)	4	14 (1.9)	15
Reproductive system and breast disorders	4 (2.2)	4	1 (0.6)	1	2 (1.1)	2	2 (1.0)	2	9 (1.2)	9
Surgical and medical procedures	2 (1.1)	2	3 (1.7)	3	2 (1.1)	2	2 (1.0)	2	9 (1.2)	9
Vascular disorders	2 (1.1)	2	1 (0.6)	1	1 (0.6)	1	5 (2.5)	5	9 (1.2)	9
Psychiatric disorders	2 (1.1)	3	3 (1.7)	3	2 (1.1)	2	1 (0.5)	1	8 (1.1)	9
Eye disorders	1 (0.5)	1	1 (0.6)	1	2 (1.1)	2	1 (0.5)	1	5 (0.7)	5
Ear and labyrinth disorders	3 (1.6)	3	1 (0.6)	1	0 (0.0)	0	0 (0.0)	0	4 (0.5)	4
Investigations	1 (0.5)	1	0 (0.0)	0	1 (0.6)	1	0 (0.0)	0	2 (0.3)	2
Metabolism and nutrition disorders	1 (0.5)	1	0 (0.0)	0	1 (0.6)	1	0 (0.0)	0	2 (0.3)	2
Endocrine disorders	1 (0.5)	1	0 (0.0)	0	0 (0.0)	0	0 (0.0)	0	1 (0.1)	1
Immune system disorders	0 (0.0)	0	1 (0.6)	1	0 (0.0)	0	0 (0.0)	0	1 (0.1)	1

A subject with more than one finding in a specific category was only counted once
Percentages based on total no. of subjects in each group
Table is sorted by descending subject count for the total of all treatment groups
p-value from Fisher's Exact test to detect any global difference between all treatment groups

14.5.1.6 Unsolicited adverse events: MedDRA coding by PT and subject, Safety dataset

	Number of Subjects (%) - Number of events				
	Group 1 (MM)	Group 2 (MP)	Group 3 (PP)	Group 4 (M-)	Total
Preferred Term	(N=183)	(N=181)	(N=181)	(N=200)	(N=745)
Any AE	113 (61.7) 260	107 (59.1) 197	78 (43.1) 141	99 (49.5) 155	397 (53.3) 753
Injection site pruritus	43 (23.5) 61	36 (19.9) 37	4 (2.2) 4	49 (24.5) 49	132 (17.7) 151
Nasopharyngitis	42 (23.0) 45	20 (11.0) 20	26 (14.4) 28	4 (2.0) 4	92 (12.3) 97
Headache	10 (5.5) 11	16 (8.8) 17	11 (6.1) 11	6 (3.0) 6	43 (5.8) 45
Pharyngolaryngeal pain	11 (6.0) 11	9 (5.0) 9	8 (4.4) 9	4 (2.0) 4	32 (4.3) 33
Injection site haematoma	6 (3.3) 6	9 (5.0) 9	5 (2.8) 5	4 (2.0) 4	24 (3.2) 24
Injection site warmth	9 (4.9) 10	5 (2.8) 5	0 (0.0) 0	8 (4.0) 8	22 (3.0) 23
Diarrhoea	3 (1.6) 3	4 (2.2) 4	3 (1.7) 3	6 (3.0) 6	16 (2.1) 16
Dizziness	2 (1.1) 3	7 (3.9) 7	2 (1.1) 3	4 (2.0) 4	15 (2.0) 17
Lymphadenopathy	4 (2.2) 5	4 (2.2) 4	2 (1.1) 2	4 (2.0) 4	14 (1.9) 15
Back pain	4 (2.2) 4	1 (0.6) 1	5 (2.8) 5	2 (1.0) 2	12 (1.6) 12
Bronchitis	3 (1.6) 3	4 (2.2) 4	2 (1.1) 2	1 (0.5) 1	10 (1.3) 10
Tonsillitis	4 (2.2) 4	5 (2.8) 5	1 (0.6) 1	0 (0.0) 0	10 (1.3) 10
Cough	5 (2.7) 5	1 (0.6) 1	3 (1.7) 3	0 (0.0) 0	9 (1.2) 9
Rhinitis	3 (1.6) 3	1 (0.6) 1	4 (2.2) 4	1 (0.5) 1	9 (1.2) 9
Palpitations	2 (1.1) 2	2 (1.1) 2	2 (1.1) 2	2 (1.0) 3	8 (1.1) 9
Abdominal pain	3 (1.6) 3	3 (1.7) 3	1 (0.6) 1	0 (0.0) 0	7 (0.9) 7
Toothache	2 (1.1) 2	1 (0.6) 1	2 (1.1) 3	2 (1.0) 2	7 (0.9) 8
Abdominal pain upper	1 (0.5) 1	1 (0.6) 1	4 (2.2) 4	0 (0.0) 0	6 (0.8) 6
Dysmenorrhoea	3 (1.6) 3	1 (0.6) 1	2 (1.1) 2	0 (0.0) 0	6 (0.8) 6
Asthenia	2 (1.1) 2	0 (0.0) 0	2 (1.1) 2	1 (0.5) 1	5 (0.7) 5
Cystitis	1 (0.5) 1	2 (1.1) 2	1 (0.6) 1	1 (0.5) 1	5 (0.7) 5
Herpes simplex	2 (1.1) 2	2 (1.1) 2	1 (0.6) 1	0 (0.0) 0	5 (0.7) 5
Injection site discolouration	1 (0.5) 1	4 (2.2) 4	0 (0.0) 0	0 (0.0) 0	5 (0.7) 5
Injection site irritation	1 (0.5) 2	1 (0.6) 1	0 (0.0) 0	3 (1.5) 3	5 (0.7) 6
Sinusitis	2 (1.1) 2	1 (0.6) 1	1 (0.6) 1	1 (0.5) 1	5 (0.7) 5
Arthralgia	0 (0.0) 0	0 (0.0) 0	3 (1.7) 3	1 (0.5) 1	4 (0.5) 4

A subject with more than one finding in a specific category was only counted once
Percentages based on total no. of subjects in each group
Table is sorted by descending subject count for the total of all treatment groups
p-value from Fisher's Exact test to detect any global difference between all treatment groups

Unsolicited adverse events: MedDRA coding by PT and subject, Safety dataset

Preferred Term	Group 1 (MM) (N=183)		Group 2 (MP) (N=181)		Group 3 (PP) (N=181)		Group 4 (M-) (N=200)		Total (N=745)	
	Number of Subjects (%) - Number of events									
Hot flush	0 (0.0)	0	0 (0.0)	0	0 (0.0)	0	4 (2.0)	4	4 (0.5)	4
Muscular weakness	2 (1.1)	2	0 (0.0)	0	1 (0.6)	1	1 (0.5)	1	4 (0.5)	4
Musculoskeletal stiffness	3 (1.6)	3	0 (0.0)	0	0 (0.0)	0	1 (0.5)	1	4 (0.5)	4
Axillary pain	1 (0.5)	1	1 (0.6)	1	0 (0.0)	0	1 (0.5)	1	3 (0.4)	3
Contusion	1 (0.5)	1	2 (1.1)	2	0 (0.0)	0	0 (0.0)	0	3 (0.4)	3
Dry mouth	2 (1.1)	3	0 (0.0)	0	0 (0.0)	0	1 (0.5)	1	3 (0.4)	4
Eczema	1 (0.5)	3	1 (0.6)	1	1 (0.6)	1	0 (0.0)	0	3 (0.4)	5
Gastroenteritis	1 (0.5)	1	1 (0.6)	1	1 (0.6)	1	0 (0.0)	0	3 (0.4)	3
Limb injury	0 (0.0)	0	1 (0.6)	1	2 (1.1)	2	0 (0.0)	0	3 (0.4)	3
Nausea	1 (0.5)	1	2 (1.1)	2	0 (0.0)	0	0 (0.0)	0	3 (0.4)	3
Otitis media	0 (0.0)	0	0 (0.0)	0	1 (0.6)	1	2 (1.0)	2	3 (0.4)	3
Pharyngitis	0 (0.0)	0	1 (0.6)	1	1 (0.6)	1	1 (0.5)	1	3 (0.4)	3
Pruritus	1 (0.5)	1	1 (0.6)	1	1 (0.6)	1	0 (0.0)	0	3 (0.4)	3
Rash	0 (0.0)	0	2 (1.1)	2	0 (0.0)	0	1 (0.5)	2	3 (0.4)	4
Tachycardia	1 (0.5)	1	1 (0.6)	1	0 (0.0)	0	1 (0.5)	1	3 (0.4)	3
Arthropod bite	0 (0.0)	0	0 (0.0)	0	1 (0.6)	1	1 (0.5)	1	2 (0.3)	2
Dermatitis contact	0 (0.0)	0	1 (0.6)	1	1 (0.6)	1	0 (0.0)	0	2 (0.3)	2
Dysphagia	0 (0.0)	0	0 (0.0)	0	0 (0.0)	0	2 (1.0)	2	2 (0.3)	2
Ear pain	2 (1.1)	2	0 (0.0)	0	0 (0.0)	0	0 (0.0)	0	2 (0.3)	2
Excoriation	0 (0.0)	0	1 (0.6)	1	1 (0.6)	1	0 (0.0)	0	2 (0.3)	2
Gastritis	0 (0.0)	0	0 (0.0)	0	2 (1.1)	2	0 (0.0)	0	2 (0.3)	2
Haemorrhage	1 (0.5)	1	1 (0.6)	1	0 (0.0)	0	0 (0.0)	0	2 (0.3)	2
Hyperhidrosis	0 (0.0)	0	0 (0.0)	0	0 (0.0)	0	2 (1.0)	3	2 (0.3)	3
Injection site hypersensitivity	1 (0.5)	1	0 (0.0)	0	1 (0.6)	1	0 (0.0)	0	2 (0.3)	2
Injection site reaction	1 (0.5)	1	0 (0.0)	0	0 (0.0)	0	1 (0.5)	1	2 (0.3)	2
Insomnia	0 (0.0)	0	2 (1.1)	2	0 (0.0)	0	0 (0.0)	0	2 (0.3)	2
Iron deficiency anaemia	0 (0.0)	0	2 (1.1)	2	0 (0.0)	0	0 (0.0)	0	2 (0.3)	2

A subject with more than one finding in a specific category was only counted once
Percentages based on total no. of subjects in each group
Table is sorted by descending subject count for the total of all treatment groups
p-value from Fisher's Exact test to detect any global difference between all treatment groups

Unsolicited adverse events: MedDRA coding by PT and subject, Safety dataset

Preferred Term	Group 1 (MM) (N=183)	Group 2 (MP) (N=181)	Group 3 (PP) (N=181)	Group 4 (M-) (N=200)	Total (N=745)
	Number of Subjects (%) - Number of events				
Lymph node pain	2 (1.1) 2	0 (0.0) 0	0 (0.0) 0	0 (0.0) 0	2 (0.3) 2
Metrorrhagia	1 (0.5) 1	0 (0.0) 0	0 (0.0) 0	1 (0.5) 1	2 (0.3) 2
Muscle spasms	1 (0.5) 1	0 (0.0) 0	0 (0.0) 0	1 (0.5) 2	2 (0.3) 3
Myalgia	1 (0.5) 1	0 (0.0) 0	1 (0.6) 1	0 (0.0) 0	2 (0.3) 2
Neck pain	1 (0.5) 1	0 (0.0) 0	1 (0.6) 1	0 (0.0) 0	2 (0.3) 2
Otitis externa	1 (0.5) 1	0 (0.0) 0	0 (0.0) 0	1 (0.5) 1	2 (0.3) 2
Pain in extremity	1 (0.5) 1	1 (0.6) 1	0 (0.0) 0	0 (0.0) 0	2 (0.3) 2
Procedural pain	0 (0.0) 0	1 (0.6) 1	1 (0.6) 1	0 (0.0) 0	2 (0.3) 2
Pyrexia	1 (0.5) 1	1 (0.6) 1	0 (0.0) 0	0 (0.0) 0	2 (0.3) 2
Sinus tachycardia	1 (0.5) 1	1 (0.6) 1	0 (0.0) 0	0 (0.0) 0	2 (0.3) 2
Skin laceration	2 (1.1) 2	0 (0.0) 0	0 (0.0) 0	0 (0.0) 0	2 (0.3) 2
Sleep disorder	1 (0.5) 2	0 (0.0) 0	1 (0.6) 1	0 (0.0) 0	2 (0.3) 3
Sneezing	0 (0.0) 0	1 (0.6) 1	0 (0.0) 0	1 (0.5) 1	2 (0.3) 2
Thirst	0 (0.0) 0	1 (0.6) 1	0 (0.0) 0	1 (0.5) 1	2 (0.3) 2
Tooth extraction	0 (0.0) 0	1 (0.6) 1	0 (0.0) 0	1 (0.5) 1	2 (0.3) 2
Tremor	1 (0.5) 1	1 (0.6) 1	0 (0.0) 0	0 (0.0) 0	2 (0.3) 2
Vertigo	1 (0.5) 1	1 (0.6) 1	0 (0.0) 0	0 (0.0) 0	2 (0.3) 2
Vomiting	0 (0.0) 0	2 (1.1) 2	0 (0.0) 0	0 (0.0) 0	2 (0.3) 2
Vulvovaginal mycotic infection	0 (0.0) 0	0 (0.0) 0	1 (0.6) 2	1 (0.5) 1	2 (0.3) 3
Abdominal pain lower	0 (0.0) 0	0 (0.0) 0	0 (0.0) 0	1 (0.5) 1	1 (0.1) 1
Abnormal sensation in eye	0 (0.0) 0	0 (0.0) 0	0 (0.0) 0	1 (0.5) 1	1 (0.1) 1
Acne	0 (0.0) 0	0 (0.0) 0	0 (0.0) 0	1 (0.5) 1	1 (0.1) 1
Acute tonsillitis	0 (0.0) 0	1 (0.6) 1	0 (0.0) 0	0 (0.0) 0	1 (0.1) 1
Arthritis	0 (0.0) 0	0 (0.0) 0	1 (0.6) 1	0 (0.0) 0	1 (0.1) 1
Back injury	1 (0.5) 1	0 (0.0) 0	0 (0.0) 0	0 (0.0) 0	1 (0.1) 1
Blepharospasm	1 (0.5) 1	0 (0.0) 0	0 (0.0) 0	0 (0.0) 0	1 (0.1) 1
Blood potassium increased	0 (0.0) 0	0 (0.0) 0	1 (0.6) 1	0 (0.0) 0	1 (0.1) 1

A subject with more than one finding in a specific category was only counted once
Percentages based on total no. of subjects in each group
Table is sorted by descending subject count for the total of all treatment groups
p-value from Fisher's Exact test to detect any global difference between all treatment groups

Unsolicited adverse events: MedDRA coding by PT and subject, Safety dataset

Preferred Term	Group 1 (MM) (N=183)		Group 2 (MP) (N=181)		Group 3 (PP) (N=181)		Group 4 (M-) (N=200)		Total (N=745)	
	Number of Subjects (%) - Number of events									
Cheilitis	0 (0.0)	0	1 (0.6)	1	0 (0.0)	0	0 (0.0)	0	1 (0.1)	1
Chest discomfort	0 (0.0)	0	1 (0.6)	1	0 (0.0)	0	0 (0.0)	0	1 (0.1)	1
Chest pain	1 (0.5)	1	0 (0.0)	0	0 (0.0)	0	0 (0.0)	0	1 (0.1)	1
Chills	1 (0.5)	1	0 (0.0)	0	0 (0.0)	0	0 (0.0)	0	1 (0.1)	1
Colitis	0 (0.0)	0	0 (0.0)	0	1 (0.6)	1	0 (0.0)	0	1 (0.1)	1
Conjunctivitis	0 (0.0)	0	0 (0.0)	0	1 (0.6)	1	0 (0.0)	0	1 (0.1)	1
Constipation	0 (0.0)	0	0 (0.0)	0	0 (0.0)	0	1 (0.5)	1	1 (0.1)	1
Dental treatment	0 (0.0)	0	0 (0.0)	0	0 (0.0)	0	1 (0.5)	1	1 (0.1)	1
Dermatitis	0 (0.0)	0	1 (0.6)	1	0 (0.0)	0	0 (0.0)	0	1 (0.1)	1
Disorientation	0 (0.0)	0	1 (0.6)	1	0 (0.0)	0	0 (0.0)	0	1 (0.1)	1
Disturbance in attention	0 (0.0)	0	0 (0.0)	0	0 (0.0)	0	1 (0.5)	1	1 (0.1)	1
Dry eye	0 (0.0)	0	0 (0.0)	0	1 (0.6)	1	0 (0.0)	0	1 (0.1)	1
Dry throat	0 (0.0)	0	0 (0.0)	0	0 (0.0)	0	1 (0.5)	1	1 (0.1)	1
Dyspepsia	0 (0.0)	0	0 (0.0)	0	1 (0.6)	1	0 (0.0)	0	1 (0.1)	1
Dysphonia	0 (0.0)	0	1 (0.6)	1	0 (0.0)	0	0 (0.0)	0	1 (0.1)	1
Erythema	0 (0.0)	0	1 (0.6)	1	0 (0.0)	0	0 (0.0)	0	1 (0.1)	1
Feeling hot	0 (0.0)	0	0 (0.0)	0	0 (0.0)	0	1 (0.5)	1	1 (0.1)	1
Flank pain	0 (0.0)	0	0 (0.0)	0	0 (0.0)	0	1 (0.5)	1	1 (0.1)	1
Flatulence	0 (0.0)	0	1 (0.6)	2	0 (0.0)	0	0 (0.0)	0	1 (0.1)	2
Flushing	0 (0.0)	0	0 (0.0)	0	1 (0.6)	1	0 (0.0)	0	1 (0.1)	1
Gastrointestinal infection	0 (0.0)	0	1 (0.6)	1	0 (0.0)	0	0 (0.0)	0	1 (0.1)	1
Haematoma	1 (0.5)	1	0 (0.0)	0	0 (0.0)	0	0 (0.0)	0	1 (0.1)	1
Hemiparesis	0 (0.0)	0	1 (0.6)	1	0 (0.0)	0	0 (0.0)	0	1 (0.1)	1
Herpes ophthalmic	1 (0.5)	1	0 (0.0)	0	0 (0.0)	0	0 (0.0)	0	1 (0.1)	1
Hypersensitivity	0 (0.0)	0	1 (0.6)	1	0 (0.0)	0	0 (0.0)	0	1 (0.1)	1
Hypertension	0 (0.0)	0	0 (0.0)	0	0 (0.0)	0	1 (0.5)	1	1 (0.1)	1
Hyperventilation	1 (0.5)	1	0 (0.0)	0	0 (0.0)	0	0 (0.0)	0	1 (0.1)	1

A subject with more than one finding in a specific category was only counted once
Percentages based on total no. of subjects in each group
Table is sorted by descending subject count for the total of all treatment groups
p-value from Fisher's Exact test to detect any global difference between all treatment groups

Unsolicited adverse events: MedDRA coding by PT and subject, Safety dataset

Preferred Term	Group 1 (MM) (N=183)	Group 2 (MP) (N=181)	Group 3 (PP) (N=181)	Group 4 (M-) (N=200)	Total (N=745)
	Number of Subjects (%) - Number of events				
Hypoaesthesia	1 (0.5) 1	0 (0.0) 0	0 (0.0) 0	0 (0.0) 0	1 (0.1) 1
Hypothyroidism	1 (0.5) 1	0 (0.0) 0	0 (0.0) 0	0 (0.0) 0	1 (0.1) 1
Implant site pain	1 (0.5) 1	0 (0.0) 0	0 (0.0) 0	0 (0.0) 0	1 (0.1) 1
Increased appetite	1 (0.5) 1	0 (0.0) 0	0 (0.0) 0	0 (0.0) 0	1 (0.1) 1
Induration	0 (0.0) 0	0 (0.0) 0	1 (0.6) 1	0 (0.0) 0	1 (0.1) 1
Influenza like illness	1 (0.5) 1	0 (0.0) 0	0 (0.0) 0	0 (0.0) 0	1 (0.1) 1
Initial insomnia	0 (0.0) 0	0 (0.0) 0	1 (0.6) 1	0 (0.0) 0	1 (0.1) 1
Injection site erythema	1 (0.5) 1	0 (0.0) 0	0 (0.0) 0	0 (0.0) 0	1 (0.1) 1
Injection site paraesthesia	1 (0.5) 1	0 (0.0) 0	0 (0.0) 0	0 (0.0) 0	1 (0.1) 1
Injection site urticaria	0 (0.0) 0	0 (0.0) 0	1 (0.6) 1	0 (0.0) 0	1 (0.1) 1
Intervertebral disc protrusion	0 (0.0) 0	0 (0.0) 0	0 (0.0) 0	1 (0.5) 1	1 (0.1) 1
Joint dislocation	0 (0.0) 0	0 (0.0) 0	1 (0.6) 1	0 (0.0) 0	1 (0.1) 1
Laryngitis	0 (0.0) 0	0 (0.0) 0	0 (0.0) 0	1 (0.5) 1	1 (0.1) 1
Localised infection	0 (0.0) 0	0 (0.0) 0	1 (0.6) 1	0 (0.0) 0	1 (0.1) 1
Lymph node palpable	1 (0.5) 1	0 (0.0) 0	0 (0.0) 0	0 (0.0) 0	1 (0.1) 1
Lymphadenitis	0 (0.0) 0	1 (0.6) 1	0 (0.0) 0	0 (0.0) 0	1 (0.1) 1
Malaise	0 (0.0) 0	0 (0.0) 0	0 (0.0) 0	1 (0.5) 1	1 (0.1) 1
Meniscus operation	0 (0.0) 0	0 (0.0) 0	1 (0.6) 1	0 (0.0) 0	1 (0.1) 1
Menstruation irregular	0 (0.0) 0	0 (0.0) 0	0 (0.0) 0	1 (0.5) 1	1 (0.1) 1
Mental disorder	1 (0.5) 1	0 (0.0) 0	0 (0.0) 0	0 (0.0) 0	1 (0.1) 1
Migraine	1 (0.5) 1	0 (0.0) 0	0 (0.0) 0	0 (0.0) 0	1 (0.1) 1
Muscle tightness	0 (0.0) 0	1 (0.6) 1	0 (0.0) 0	0 (0.0) 0	1 (0.1) 1
Muscle twitching	0 (0.0) 0	0 (0.0) 0	0 (0.0) 0	1 (0.5) 1	1 (0.1) 1
Musculoskeletal chest pain	0 (0.0) 0	0 (0.0) 0	0 (0.0) 0	1 (0.5) 1	1 (0.1) 1
Nasal operation	1 (0.5) 1	0 (0.0) 0	0 (0.0) 0	0 (0.0) 0	1 (0.1) 1
Non-cardiac chest pain	1 (0.5) 1	0 (0.0) 0	0 (0.0) 0	0 (0.0) 0	1 (0.1) 1
Oophoritis	0 (0.0) 0	0 (0.0) 0	1 (0.6) 1	0 (0.0) 0	1 (0.1) 1

A subject with more than one finding in a specific category was only counted once
Percentages based on total no. of subjects in each group
Table is sorted by descending subject count for the total of all treatment groups
p-value from Fisher's Exact test to detect any global difference between all treatment groups

Unsolicited adverse events: MedDRA coding by PT and subject, Safety dataset

Preferred Term	Group 1 (MM) (N=183)	Group 2 (MP) (N=181)	Group 3 (PP) (N=181)	Group 4 (M-) (N=200)	Total (N=745)
	Number of Subjects (%) - Number of events				
Pain	0 (0.0) 0	0 (0.0) 0	1 (0.6) 1	0 (0.0) 0	1 (0.1) 1
Pain in jaw	0 (0.0) 0	0 (0.0) 0	0 (0.0) 0	1 (0.5) 1	1 (0.1) 1
Paraesthesia	0 (0.0) 0	1 (0.6) 1	0 (0.0) 0	0 (0.0) 0	1 (0.1) 1
Pericardial effusion	0 (0.0) 0	0 (0.0) 0	1 (0.6) 1	0 (0.0) 0	1 (0.1) 1
Photodermatosis	1 (0.5) 1	0 (0.0) 0	0 (0.0) 0	0 (0.0) 0	1 (0.1) 1
Polydipsia	0 (0.0) 0	0 (0.0) 0	1 (0.6) 1	0 (0.0) 0	1 (0.1) 1
Rash papular	1 (0.5) 2	0 (0.0) 0	0 (0.0) 0	0 (0.0) 0	1 (0.1) 2
Removal of internal fixation	1 (0.5) 1	0 (0.0) 0	0 (0.0) 0	0 (0.0) 0	1 (0.1) 1
Restlessness	0 (0.0) 0	0 (0.0) 0	0 (0.0) 0	1 (0.5) 1	1 (0.1) 1
Rhinorrhoea	1 (0.5) 1	0 (0.0) 0	0 (0.0) 0	0 (0.0) 0	1 (0.1) 1
Road traffic accident	0 (0.0) 0	1 (0.6) 1	0 (0.0) 0	0 (0.0) 0	1 (0.1) 1
Sciatica	0 (0.0) 0	0 (0.0) 0	1 (0.6) 1	0 (0.0) 0	1 (0.1) 1
Sedation	0 (0.0) 0	0 (0.0) 0	0 (0.0) 0	1 (0.5) 1	1 (0.1) 1
Sensation of heaviness	0 (0.0) 0	1 (0.6) 1	0 (0.0) 0	0 (0.0) 0	1 (0.1) 1
Sensitivity of teeth	0 (0.0) 0	0 (0.0) 0	0 (0.0) 0	1 (0.5) 1	1 (0.1) 1
Sinobronchitis	0 (0.0) 0	1 (0.6) 1	0 (0.0) 0	0 (0.0) 0	1 (0.1) 1
Skin injury	1 (0.5) 1	0 (0.0) 0	0 (0.0) 0	0 (0.0) 0	1 (0.1) 1
Skin reaction	0 (0.0) 0	0 (0.0) 0	1 (0.6) 1	0 (0.0) 0	1 (0.1) 1
Soft tissue inflammation	0 (0.0) 0	0 (0.0) 0	1 (0.6) 1	0 (0.0) 0	1 (0.1) 1
Syncope	1 (0.5) 1	0 (0.0) 0	0 (0.0) 0	0 (0.0) 0	1 (0.1) 1
Tendon injury	0 (0.0) 0	1 (0.6) 1	0 (0.0) 0	0 (0.0) 0	1 (0.1) 1
Tinea pedis	0 (0.0) 0	0 (0.0) 0	0 (0.0) 0	1 (0.5) 1	1 (0.1) 1
Tooth repair	0 (0.0) 0	1 (0.6) 1	0 (0.0) 0	0 (0.0) 0	1 (0.1) 1
Urethritis	0 (0.0) 0	1 (0.6) 2	0 (0.0) 0	0 (0.0) 0	1 (0.1) 2
Urinary tract infection	1 (0.5) 1	0 (0.0) 0	0 (0.0) 0	0 (0.0) 0	1 (0.1) 1
Varicose vein operation	0 (0.0) 0	0 (0.0) 0	1 (0.6) 1	0 (0.0) 0	1 (0.1) 1
Visual disturbance	0 (0.0) 0	1 (0.6) 1	0 (0.0) 0	0 (0.0) 0	1 (0.1) 1

A subject with more than one finding in a specific category was only counted once
Percentages based on total no. of subjects in each group
Table is sorted by descending subject count for the total of all treatment groups
p-value from Fisher's Exact test to detect any global difference between all treatment groups

Unsolicited adverse events: MedDRA coding by PT and subject, Safety dataset

	Number of Subjects (%) - Number of events				
Preferred Term	Group 1 (MM) (N=183)	Group 2 (MP) (N=181)	Group 3 (PP) (N=181)	Group 4 (M-) (N=200)	Total (N=745)
Wisdom teeth removal	0 (0.0) 0	1 (0.6) 1	0 (0.0) 0	0 (0.0) 0	1 (0.1) 1

A subject with more than one finding in a specific category was only counted once
Percentages based on total no. of subjects in each group
Table is sorted by descending subject count for the total of all treatment groups
p-value from Fisher's Exact test to detect any global difference between all treatment groups

14.5.1.7 Unsolicited adverse events: MedDRA coding by SOC, PT, intensity and subject, Safety dataset

Intensity / SOC / Preferred Term	Group 1 (MM) (N=183)	Group 2 (MP) (N=181)	Group 3 (PP) (N=181)	Group 4 (M-) (N=200)	Total (N=745)
	Number of Subjects (%) - Number of events				
mild					
General disorders and administration site conditions					
Any PT	51 (27.9) 85	48 (26.5) 59	14 (7.7) 14	53 (26.5) 64	166 (22.3) 222
Injection site pruritus	42 (23.0) 60	36 (19.9) 37	4 (2.2) 4	46 (23.0) 46	128 (17.2) 147
Injection site haematoma	6 (3.3) 6	9 (5.0) 9	5 (2.8) 5	4 (2.0) 4	24 (3.2) 24
Injection site warmth	7 (3.8) 8	5 (2.8) 5	0 (0.0) 0	7 (3.5) 7	19 (2.6) 20
Injection site discolouration	1 (0.5) 1	4 (2.2) 4	0 (0.0) 0	0 (0.0) 0	5 (0.7) 5
Asthenia	1 (0.5) 1	0 (0.0) 0	2 (1.1) 2	1 (0.5) 1	4 (0.5) 4
Injection site irritation	1 (0.5) 2	1 (0.6) 1	0 (0.0) 0	2 (1.0) 2	4 (0.5) 5
Axillary pain	1 (0.5) 1	1 (0.6) 1	0 (0.0) 0	1 (0.5) 1	3 (0.4) 3
Injection site hypersensitivity	1 (0.5) 1	0 (0.0) 0	1 (0.6) 1	0 (0.0) 0	2 (0.3) 2
Injection site reaction	1 (0.5) 1	0 (0.0) 0	0 (0.0) 0	1 (0.5) 1	2 (0.3) 2
Thirst	0 (0.0) 0	1 (0.6) 1	0 (0.0) 0	1 (0.5) 1	2 (0.3) 2
Chest discomfort	0 (0.0) 0	1 (0.6) 1	0 (0.0) 0	0 (0.0) 0	1 (0.1) 1
Implant site pain	1 (0.5) 1	0 (0.0) 0	0 (0.0) 0	0 (0.0) 0	1 (0.1) 1
Induration	0 (0.0) 0	0 (0.0) 0	1 (0.6) 1	0 (0.0) 0	1 (0.1) 1
Injection site erythema	1 (0.5) 1	0 (0.0) 0	0 (0.0) 0	0 (0.0) 0	1 (0.1) 1
Malaise	0 (0.0) 0	0 (0.0) 0	0 (0.0) 0	1 (0.5) 1	1 (0.1) 1
Non-cardiac chest pain	1 (0.5) 1	0 (0.0) 0	0 (0.0) 0	0 (0.0) 0	1 (0.1) 1
Pyrexia	1 (0.5) 1	0 (0.0) 0	0 (0.0) 0	0 (0.0) 0	1 (0.1) 1
Soft tissue inflammation	0 (0.0) 0	0 (0.0) 0	1 (0.6) 1	0 (0.0) 0	1 (0.1) 1
Infections and infestations					
Any PT	35 (19.1) 41	25 (13.8) 26	29 (16.0) 33	7 (3.5) 8	96 (12.9) 108
Nasopharyngitis	25 (13.7) 27	11 (6.1) 11	21 (11.6) 22	3 (1.5) 3	60 (8.1) 63
Rhinitis	3 (1.6) 3	1 (0.6) 1	3 (1.7) 3	0 (0.0) 0	7 (0.9) 7
Bronchitis	2 (1.1) 2	2 (1.1) 2	1 (0.6) 1	0 (0.0) 0	5 (0.7) 5

A subject with more than one finding in a specific category was only counted once
Percentages based on total no. of subjects in each group
Table is sorted by descending subject count for the total of all treatment groups
Intensity grade 1/2/3/4 was decoded in mild/moderate/severe/life-threatening

Unsolicited adverse events: MedDRA coding by SOC, PT, intensity and subject, Safety dataset

Intensity<br>SOC<br>Preferred Term	Number of Subjects (%) - Number of events<br>Group 1 (MM)<br>(N=183)		Group 2 (MP)<br>(N=181)		Group 3 (PP)<br>(N=181)		Group 4 (M-)<br>(N=200)		Total<br>(N=745)	
Tonsillitis	1 (0.5)	1	4 (2.2)	4	0 (0.0)	0	0 (0.0)	0	5 (0.7)	5
Herpes simplex	1 (0.5)	1	2 (1.1)	2	1 (0.6)	1	0 (0.0)	0	4 (0.5)	4
Cystitis	1 (0.5)	1	0 (0.0)	0	1 (0.6)	1	1 (0.5)	1	3 (0.4)	3
Gastroenteritis	1 (0.5)	1	0 (0.0)	0	1 (0.6)	1	0 (0.0)	0	2 (0.3)	2
Pharyngitis	0 (0.0)	0	1 (0.6)	1	0 (0.0)	0	1 (0.5)	1	2 (0.3)	2
Sinusitis	2 (1.1)	2	0 (0.0)	0	0 (0.0)	0	0 (0.0)	0	2 (0.3)	2
Vulvovaginal mycotic infection	0 (0.0)	0	0 (0.0)	0	1 (0.6)	2	1 (0.5)	1	2 (0.3)	3
Acute tonsillitis	0 (0.0)	0	1 (0.6)	1	0 (0.0)	0	0 (0.0)	0	1 (0.1)	1
Gastrointestinal infection	0 (0.0)	0	1 (0.6)	1	0 (0.0)	0	0 (0.0)	0	1 (0.1)	1
Herpes ophthalmic	1 (0.5)	1	0 (0.0)	0	0 (0.0)	0	0 (0.0)	0	1 (0.1)	1
Localised infection	0 (0.0)	0	0 (0.0)	0	1 (0.6)	1	0 (0.0)	0	1 (0.1)	1
Oophoritis	0 (0.0)	0	0 (0.0)	0	1 (0.6)	1	0 (0.0)	0	1 (0.1)	1
Otitis externa	1 (0.5)	1	0 (0.0)	0	0 (0.0)	0	0 (0.0)	0	1 (0.1)	1
Otitis media	0 (0.0)	0	0 (0.0)	0	0 (0.0)	0	1 (0.5)	1	1 (0.1)	1
Sinobronchitis	0 (0.0)	0	1 (0.6)	1	0 (0.0)	0	0 (0.0)	0	1 (0.1)	1
Tinea pedis	0 (0.0)	0	0 (0.0)	0	0 (0.0)	0	1 (0.5)	1	1 (0.1)	1
Urethritis	0 (0.0)	0	1 (0.6)	2	0 (0.0)	0	0 (0.0)	0	1 (0.1)	2
Urinary tract infection	1 (0.5)	1	0 (0.0)	0	0 (0.0)	0	0 (0.0)	0	1 (0.1)	1
Nervous system disorders										
Any PT	13 (7.1)	14	15 (8.3)	15	9 (5.0)	9	8 (4.0)	8	45 (6.0)	46
Headache	10 (5.5)	11	11 (6.1)	11	7 (3.9)	7	5 (2.5)	5	33 (4.4)	34
Dizziness	2 (1.1)	2	2 (1.1)	2	2 (1.1)	2	2 (1.0)	2	8 (1.1)	8
Tremor	1 (0.5)	1	1 (0.6)	1	0 (0.0)	0	0 (0.0)	0	2 (0.3)	2
Disturbance in attention	0 (0.0)	0	0 (0.0)	0	0 (0.0)	0	1 (0.5)	1	1 (0.1)	1
Paraesthesia	0 (0.0)	0	1 (0.6)	1	0 (0.0)	0	0 (0.0)	0	1 (0.1)	1

A subject with more than one finding in a specific category was only counted once
Percentages based on total no. of subjects in each group
Table is sorted by descending subject count for the total of all treatment groups
Intensity grade 1/2/3/4 was decoded in mild/moderate/severe/life-threatening

Unsolicited adverse events: MedDRA coding by SOC, PT, intensity and subject, Safety dataset

Intensity<br>SOC<br>Preferred Term	Group 1 (MM)<br>(N=183)		Group 2 (MP)<br>(N=181)		Group 3 (PP)<br>(N=181)		Group 4 (M-)<br>(N=200)		Total<br>(N=745)	
	Number of Subjects (%) - Number of events									
Gastrointestinal disorders										
Any PT	7 (3.8)	7	8 (4.4)	9	8 (4.4)	10	9 (4.5)	9	32 (4.3)	35
Diarrhoea	2 (1.1)	2	2 (1.1)	2	2 (1.1)	2	5 (2.5)	5	11 (1.5)	11
Abdominal pain upper	0 (0.0)	0	1 (0.6)	1	3 (1.7)	3	0 (0.0)	0	4 (0.5)	4
Abdominal pain	2 (1.1)	2	1 (0.6)	1	0 (0.0)	0	0 (0.0)	0	3 (0.4)	3
Toothache	1 (0.5)	1	0 (0.0)	0	2 (1.1)	3	0 (0.0)	0	3 (0.4)	4
Dry mouth	2 (1.1)	2	0 (0.0)	0	0 (0.0)	0	0 (0.0)	0	2 (0.3)	2
Dysphagia	0 (0.0)	0	0 (0.0)	0	0 (0.0)	0	2 (1.0)	2	2 (0.3)	2
Nausea	0 (0.0)	0	2 (1.1)	2	0 (0.0)	0	0 (0.0)	0	2 (0.3)	2
Cheilitis	0 (0.0)	0	1 (0.6)	1	0 (0.0)	0	0 (0.0)	0	1 (0.1)	1
Constipation	0 (0.0)	0	0 (0.0)	0	0 (0.0)	0	1 (0.5)	1	1 (0.1)	1
Dyspepsia	0 (0.0)	0	0 (0.0)	0	1 (0.6)	1	0 (0.0)	0	1 (0.1)	1
Flatulence	0 (0.0)	0	1 (0.6)	2	0 (0.0)	0	0 (0.0)	0	1 (0.1)	2
Gastritis	0 (0.0)	0	0 (0.0)	0	1 (0.6)	1	0 (0.0)	0	1 (0.1)	1
Sensitivity of teeth	0 (0.0)	0	0 (0.0)	0	0 (0.0)	0	1 (0.5)	1	1 (0.1)	1
Respiratory, thoracic and mediastinal disorders										
Any PT	11 (6.0)	12	6 (3.3)	6	7 (3.9)	9	6 (3.0)	6	30 (4.0)	33
Pharyngolaryngeal pain	8 (4.4)	8	4 (2.2)	4	6 (3.3)	7	4 (2.0)	4	22 (3.0)	23
Cough	4 (2.2)	4	1 (0.6)	1	2 (1.1)	2	0 (0.0)	0	7 (0.9)	7
Sneezing	0 (0.0)	0	1 (0.6)	1	0 (0.0)	0	1 (0.5)	1	2 (0.3)	2
Dry throat	0 (0.0)	0	0 (0.0)	0	0 (0.0)	0	1 (0.5)	1	1 (0.1)	1
Musculoskeletal and connective tissue disorders										
Any PT	12 (6.6)	12	2 (1.1)	2	6 (3.3)	6	5 (2.5)	5	25 (3.4)	25
Back pain	4 (2.2)	4	0 (0.0)	0	3 (1.7)	3	1 (0.5)	1	8 (1.1)	8
Muscular weakness	2 (1.1)	2	0 (0.0)	0	1 (0.6)	1	1 (0.5)	1	4 (0.5)	4

A subject with more than one finding in a specific category was only counted once
Percentages based on total no. of subjects in each group
Table is sorted by descending subject count for the total of all treatment groups
Intensity grade 1/2/3/4 was decoded in mild/moderate/severe/life-threatening

Unsolicited adverse events: MedDRA coding by SOC, PT, intensity and subject, Safety dataset

Intensity<br>SOC<br>Preferred Term	Group 1 (MM)<br>(N=183)		Group 2 (MP)<br>(N=181)		Group 3 (PP)<br>(N=181)		Group 4 (M-)<br>(N=200)		Total<br>(N=745)	
	Number of Subjects (%) - Number of events									
Musculoskeletal stiffness	3 (1.6)	3	0 (0.0)	0	0 (0.0)	0	1 (0.5)	1	4 (0.5)	4
Myalgia	1 (0.5)	1	0 (0.0)	0	1 (0.6)	1	0 (0.0)	0	2 (0.3)	2
Pain in extremity	1 (0.5)	1	1 (0.6)	1	0 (0.0)	0	0 (0.0)	0	2 (0.3)	2
Arthralgia	0 (0.0)	0	0 (0.0)	0	1 (0.6)	1	0 (0.0)	0	1 (0.1)	1
Flank pain	0 (0.0)	0	0 (0.0)	0	0 (0.0)	0	1 (0.5)	1	1 (0.1)	1
Neck pain	1 (0.5)	1	0 (0.0)	0	0 (0.0)	0	0 (0.0)	0	1 (0.1)	1
Pain in jaw	0 (0.0)	0	0 (0.0)	0	0 (0.0)	0	1 (0.5)	1	1 (0.1)	1
Sensation of heaviness	0 (0.0)	0	1 (0.6)	1	0 (0.0)	0	0 (0.0)	0	1 (0.1)	1
Blood and lymphatic system disorders										
Any PT	4 (2.2)	6	7 (3.9)	7	1 (0.6)	1	4 (2.0)	4	16 (2.1)	18
Lymphadenopathy	4 (2.2)	5	4 (2.2)	4	1 (0.6)	1	4 (2.0)	4	13 (1.7)	14
Iron deficiency anaemia	0 (0.0)	0	2 (1.1)	2	0 (0.0)	0	0 (0.0)	0	2 (0.3)	2
Lymph node pain	1 (0.5)	1	0 (0.0)	0	0 (0.0)	0	0 (0.0)	0	1 (0.1)	1
Lymphadenitis	0 (0.0)	0	1 (0.6)	1	0 (0.0)	0	0 (0.0)	0	1 (0.1)	1
Skin and subcutaneous tissue disorders										
Any PT	3 (1.6)	6	6 (3.3)	6	4 (2.2)	4	3 (1.5)	4	16 (2.1)	20
Eczema	1 (0.5)	3	1 (0.6)	1	1 (0.6)	1	0 (0.0)	0	3 (0.4)	5
Pruritus	1 (0.5)	1	1 (0.6)	1	1 (0.6)	1	0 (0.0)	0	3 (0.4)	3
Dermatitis contact	0 (0.0)	0	1 (0.6)	1	1 (0.6)	1	0 (0.0)	0	2 (0.3)	2
Rash	0 (0.0)	0	1 (0.6)	1	0 (0.0)	0	1 (0.5)	2	2 (0.3)	3
Acne	0 (0.0)	0	0 (0.0)	0	0 (0.0)	0	1 (0.5)	1	1 (0.1)	1
Dermatitis	0 (0.0)	0	1 (0.6)	1	0 (0.0)	0	0 (0.0)	0	1 (0.1)	1
Erythema	0 (0.0)	0	1 (0.6)	1	0 (0.0)	0	0 (0.0)	0	1 (0.1)	1
Hyperhidrosis	0 (0.0)	0	0 (0.0)	0	0 (0.0)	0	1 (0.5)	1	1 (0.1)	1
Photodermatosis	1 (0.5)	1	0 (0.0)	0	0 (0.0)	0	0 (0.0)	0	1 (0.1)	1

A subject with more than one finding in a specific category was only counted once
Percentages based on total no. of subjects in each group
Table is sorted by descending subject count for the total of all treatment groups
Intensity grade 1/2/3/4 was decoded in mild/moderate/severe/life-threatening

Unsolicited adverse events: MedDRA coding by SOC, PT, intensity and subject, Safety dataset

Intensity / SOC / Preferred Term	Group 1 (MM) (N=183)		Group 2 (MP) (N=181)		Group 3 (PP) (N=181)		Group 4 (M-) (N=200)		Total (N=745)	
	Number of Subjects (%) - Number of events									
Rash papular	1 (0.5)	1	0 (0.0)	0	0 (0.0)	0	0 (0.0)	0	1 (0.1)	1
Skin reaction	0 (0.0)	0	0 (0.0)	0	1 (0.6)	1	0 (0.0)	0	1 (0.1)	1
Cardiac disorders										
Any PT	4 (2.2)	4	3 (1.7)	3	3 (1.7)	3	2 (1.0)	2	12 (1.6)	12
Palpitations	2 (1.1)	2	1 (0.6)	1	2 (1.1)	2	1 (0.5)	1	6 (0.8)	6
Tachycardia	1 (0.5)	1	1 (0.6)	1	0 (0.0)	0	1 (0.5)	1	3 (0.4)	3
Sinus tachycardia	1 (0.5)	1	1 (0.6)	1	0 (0.0)	0	0 (0.0)	0	2 (0.3)	2
Pericardial effusion	0 (0.0)	0	0 (0.0)	0	1 (0.6)	1	0 (0.0)	0	1 (0.1)	1
Vascular disorders										
Any PT	2 (1.1)	2	1 (0.6)	1	1 (0.6)	1	5 (2.5)	5	9 (1.2)	9
Hot flush	0 (0.0)	0	0 (0.0)	0	0 (0.0)	0	4 (2.0)	4	4 (0.5)	4
Haemorrhage	1 (0.5)	1	1 (0.6)	1	0 (0.0)	0	0 (0.0)	0	2 (0.3)	2
Flushing	0 (0.0)	0	0 (0.0)	0	1 (0.6)	1	0 (0.0)	0	1 (0.1)	1
Haematoma	1 (0.5)	1	0 (0.0)	0	0 (0.0)	0	0 (0.0)	0	1 (0.1)	1
Hypertension	0 (0.0)	0	0 (0.0)	0	0 (0.0)	0	1 (0.5)	1	1 (0.1)	1
Injury, poisoning and procedural complications										
Any PT	1 (0.5)	1	3 (1.7)	4	3 (1.7)	3	1 (0.5)	1	8 (1.1)	9
Arthropod bite	0 (0.0)	0	0 (0.0)	0	1 (0.6)	1	1 (0.5)	1	2 (0.3)	2
Excoriation	0 (0.0)	0	1 (0.6)	1	1 (0.6)	1	0 (0.0)	0	2 (0.3)	2
Limb injury	0 (0.0)	0	1 (0.6)	1	1 (0.6)	1	0 (0.0)	0	2 (0.3)	2
Contusion	1 (0.5)	1	0 (0.0)	0	0 (0.0)	0	0 (0.0)	0	1 (0.1)	1
Procedural pain	0 (0.0)	0	1 (0.6)	1	0 (0.0)	0	0 (0.0)	0	1 (0.1)	1
Road traffic accident	0 (0.0)	0	1 (0.6)	1	0 (0.0)	0	0 (0.0)	0	1 (0.1)	1

A subject with more than one finding in a specific category was only counted once
Percentages based on total no. of subjects in each group
Table is sorted by descending subject count for the total of all treatment groups
Intensity grade 1/2/3/4 was decoded in mild/moderate/severe/life-threatening

Unsolicited adverse events: MedDRA coding by SOC, PT, intensity and subject, Safety dataset

Intensity<br>SOC<br>Preferred Term	Group 1 (MM)<br>(N=183)		Group 2 (MP)<br>(N=181)		Group 3 (PP)<br>(N=181)		Group 4 (M-)<br>(N=200)		Total<br>(N=745)	
Reproductive system and breast disorders										
Any PT	2 (1.1)	2	0 (0.0)	0	2 (1.1)	2	2 (1.0)	2	6 (0.8)	6
Dysmenorrhoea	1 (0.5)	1	0 (0.0)	0	2 (1.1)	2	0 (0.0)	0	3 (0.4)	3
Metrorrhagia	1 (0.5)	1	0 (0.0)	0	0 (0.0)	0	1 (0.5)	1	2 (0.3)	2
Menstruation irregular	0 (0.0)	0	0 (0.0)	0	0 (0.0)	0	1 (0.5)	1	1 (0.1)	1
Eye disorders										
Any PT	1 (0.5)	1	0 (0.0)	0	2 (1.1)	2	1 (0.5)	1	4 (0.5)	4
Abnormal sensation in eye	0 (0.0)	0	0 (0.0)	0	0 (0.0)	0	1 (0.5)	1	1 (0.1)	1
Blepharospasm	1 (0.5)	1	0 (0.0)	0	0 (0.0)	0	0 (0.0)	0	1 (0.1)	1
Conjunctivitis	0 (0.0)	0	0 (0.0)	0	1 (0.6)	1	0 (0.0)	0	1 (0.1)	1
Dry eye	0 (0.0)	0	0 (0.0)	0	1 (0.6)	1	0 (0.0)	0	1 (0.1)	1
Surgical and medical procedures										
Any PT	1 (0.5)	1	1 (0.6)	1	0 (0.0)	0	2 (1.0)	2	4 (0.5)	4
Dental treatment	0 (0.0)	0	0 (0.0)	0	0 (0.0)	0	1 (0.5)	1	1 (0.1)	1
Removal of internal fixation	1 (0.5)	1	0 (0.0)	0	0 (0.0)	0	0 (0.0)	0	1 (0.1)	1
Tooth extraction	0 (0.0)	0	0 (0.0)	0	0 (0.0)	0	1 (0.5)	1	1 (0.1)	1
Wisdom teeth removal	0 (0.0)	0	1 (0.6)	1	0 (0.0)	0	0 (0.0)	0	1 (0.1)	1
Ear and labyrinth disorders										
Any PT	2 (1.1)	2	1 (0.6)	1	0 (0.0)	0	0 (0.0)	0	3 (0.4)	3
Vertigo	1 (0.5)	1	1 (0.6)	1	0 (0.0)	0	0 (0.0)	0	2 (0.3)	2
Ear pain	1 (0.5)	1	0 (0.0)	0	0 (0.0)	0	0 (0.0)	0	1 (0.1)	1
Psychiatric disorders										
Any PT	1 (0.5)	2	2 (1.1)	2	0 (0.0)	0	0 (0.0)	0	3 (0.4)	4

Number of Subjects (%) - Number of events

A subject with more than one finding in a specific category was only counted once
Percentages based on total no. of subjects in each group
Table is sorted by descending subject count for the total of all treatment groups
Intensity grade 1/2/3/4 was decoded in mild/moderate/severe/life-threatening

Unsolicited adverse events: MedDRA coding by SOC, PT, intensity and subject, Safety dataset

Intensity / SOC / Preferred Term	Group 1 (MM) (N=183)		Group 2 (MP) (N=181)		Group 3 (PP) (N=181)		Group 4 (M-) (N=200)		Total (N=745)	
Disorientation	0 (0.0)	0	1 (0.6)	1	0 (0.0)	0	0 (0.0)	0	1 (0.1)	1
Insomnia	0 (0.0)	0	1 (0.6)	1	0 (0.0)	0	0 (0.0)	0	1 (0.1)	1
Sleep disorder	1 (0.5)	2	0 (0.0)	0	0 (0.0)	0	0 (0.0)	0	1 (0.1)	2
Investigations										
Any PT	1 (0.5)	1	0 (0.0)	0	1 (0.6)	1	0 (0.0)	0	2 (0.3)	2
Blood potassium increased	0 (0.0)	0	0 (0.0)	0	1 (0.6)	1	0 (0.0)	0	1 (0.1)	1
Lymph node palpable	1 (0.5)	1	0 (0.0)	0	0 (0.0)	0	0 (0.0)	0	1 (0.1)	1
Endocrine disorders										
Any PT	1 (0.5)	1	0 (0.0)	0	0 (0.0)	0	0 (0.0)	0	1 (0.1)	1
Hypothyroidism	1 (0.5)	1	0 (0.0)	0	0 (0.0)	0	0 (0.0)	0	1 (0.1)	1
Metabolism and nutrition disorders										
Any PT	1 (0.5)	1	0 (0.0)	0	0 (0.0)	0	0 (0.0)	0	1 (0.1)	1
Increased appetite	1 (0.5)	1	0 (0.0)	0	0 (0.0)	0	0 (0.0)	0	1 (0.1)	1
moderate										
Infections and infestations										
Any PT	22 (12.0)	22	14 (7.7)	15	9 (5.0)	10	5 (2.5)	7	50 (6.7)	54
Nasopharyngitis	17 (9.3)	17	8 (4.4)	8	6 (3.3)	6	1 (0.5)	1	32 (4.3)	32
Bronchitis	1 (0.5)	1	2 (1.1)	2	1 (0.6)	1	1 (0.5)	1	5 (0.7)	5
Tonsillitis	3 (1.6)	3	1 (0.6)	1	1 (0.6)	1	0 (0.0)	0	5 (0.7)	5
Cystitis	0 (0.0)	0	2 (1.1)	2	0 (0.0)	0	0 (0.0)	0	2 (0.3)	2
Rhinitis	0 (0.0)	0	0 (0.0)	0	1 (0.6)	1	1 (0.5)	1	2 (0.3)	2
Sinusitis	0 (0.0)	0	1 (0.6)	1	0 (0.0)	0	1 (0.5)	1	2 (0.3)	2
Gastroenteritis	0 (0.0)	0	1 (0.6)	1	0 (0.0)	0	0 (0.0)	0	1 (0.1)	1

Number of Subjects (%) - Number of events

A subject with more than one finding in a specific category was only counted once
Percentages based on total no. of subjects in each group
Table is sorted by descending subject count for the total of all treatment groups
Intensity grade 1/2/3/4 was decoded in mild/moderate/severe/life-threatening

Unsolicited adverse events: MedDRA coding by SOC, PT, intensity and subject, Safety dataset

Intensity / SOC / Preferred Term	Group 1 (MM) (N=183)		Group 2 (MP) (N=181)		Group 3 (PP) (N=181)		Group 4 (M-) (N=200)		Total (N=745)	
Herpes simplex	1 (0.5)	1	0 (0.0)	0	0 (0.0)	0	0 (0.0)	0	1 (0.1)	1
Laryngitis	0 (0.0)	0	0 (0.0)	0	0 (0.0)	0	1 (0.5)	1	1 (0.1)	1
Otitis externa	0 (0.0)	0	0 (0.0)	0	0 (0.0)	0	1 (0.5)	1	1 (0.1)	1
Otitis media	0 (0.0)	0	0 (0.0)	0	0 (0.0)	0	1 (0.5)	1	1 (0.1)	1
Pharyngitis	0 (0.0)	0	0 (0.0)	0	1 (0.6)	1	0 (0.0)	0	1 (0.1)	1
Nervous system disorders										
Any PT	3 (1.6)	3	10 (5.5)	10	6 (3.3)	6	3 (1.5)	4	22 (3.0)	23
Headache	0 (0.0)	0	5 (2.8)	5	4 (2.2)	4	1 (0.5)	1	10 (1.3)	10
Dizziness	1 (0.5)	1	5 (2.8)	5	1 (0.6)	1	2 (1.0)	2	9 (1.2)	9
Hypoaesthesia	1 (0.5)	1	0 (0.0)	0	0 (0.0)	0	0 (0.0)	0	1 (0.1)	1
Migraine	1 (0.5)	1	0 (0.0)	0	0 (0.0)	0	0 (0.0)	0	1 (0.1)	1
Sciatica	0 (0.0)	0	0 (0.0)	0	1 (0.6)	1	0 (0.0)	0	1 (0.1)	1
Sedation	0 (0.0)	0	0 (0.0)	0	0 (0.0)	0	1 (0.5)	1	1 (0.1)	1
Gastrointestinal disorders										
Any PT	6 (3.3)	6	4 (2.2)	5	4 (2.2)	5	3 (1.5)	4	17 (2.3)	20
Abdominal pain	1 (0.5)	1	2 (1.1)	2	1 (0.6)	1	0 (0.0)	0	4 (0.5)	4
Diarrhoea	1 (0.5)	1	1 (0.6)	1	1 (0.6)	1	1 (0.5)	1	4 (0.5)	4
Toothache	1 (0.5)	1	1 (0.6)	1	0 (0.0)	0	1 (0.5)	1	3 (0.4)	3
Abdominal pain upper	1 (0.5)	1	0 (0.0)	0	1 (0.6)	1	0 (0.0)	0	2 (0.3)	2
Dry mouth	1 (0.5)	1	0 (0.0)	0	0 (0.0)	0	1 (0.5)	1	2 (0.3)	2
Abdominal pain lower	0 (0.0)	0	0 (0.0)	0	0 (0.0)	0	1 (0.5)	1	1 (0.1)	1
Colitis	0 (0.0)	0	0 (0.0)	0	1 (0.6)	1	0 (0.0)	0	1 (0.1)	1
Gastritis	0 (0.0)	0	0 (0.0)	0	1 (0.6)	1	0 (0.0)	0	1 (0.1)	1
Nausea	1 (0.5)	1	0 (0.0)	0	0 (0.0)	0	0 (0.0)	0	1 (0.1)	1
Vomiting	0 (0.0)	0	1 (0.6)	1	0 (0.0)	0	0 (0.0)	0	1 (0.1)	1

Number of Subjects (%) - Number of events

A subject with more than one finding in a specific category was only counted once
Percentages based on total no. of subjects in each group
Table is sorted by descending subject count for the total of all treatment groups
Intensity grade 1/2/3/4 was decoded in mild/moderate/severe/life-threatening

Unsolicited adverse events: MedDRA coding by SOC, PT, intensity and subject, Safety dataset

Intensity / SOC / Preferred Term	Group 1 (MM) (N=183)		Group 2 (MP) (N=181)		Group 3 (PP) (N=181)		Group 4 (M-) (N=200)		Total (N=745)	
	Number of Subjects (%) - Number of events									
Respiratory, thoracic and mediastinal disorders										
Any PT	5 (2.7)	6	6 (3.3)	6	3 (1.7)	3	0 (0.0)	0	14 (1.9)	15
Pharyngolaryngeal pain	3 (1.6)	3	5 (2.8)	5	2 (1.1)	2	0 (0.0)	0	10 (1.3)	10
Cough	1 (0.5)	1	0 (0.0)	0	1 (0.6)	1	0 (0.0)	0	2 (0.3)	2
Dysphonia	0 (0.0)	0	1 (0.6)	1	0 (0.0)	0	0 (0.0)	0	1 (0.1)	1
Hyperventilation	1 (0.5)	1	0 (0.0)	0	0 (0.0)	0	0 (0.0)	0	1 (0.1)	1
Rhinorrhoea	1 (0.5)	1	0 (0.0)	0	0 (0.0)	0	0 (0.0)	0	1 (0.1)	1
Musculoskeletal and connective tissue disorders										
Any PT	1 (0.5)	1	1 (0.6)	1	6 (3.3)	6	5 (2.5)	7	13 (1.7)	15
Back pain	0 (0.0)	0	1 (0.6)	1	2 (1.1)	2	1 (0.5)	1	4 (0.5)	4
Arthralgia	0 (0.0)	0	0 (0.0)	0	2 (1.1)	2	1 (0.5)	1	3 (0.4)	3
Muscle spasms	1 (0.5)	1	0 (0.0)	0	0 (0.0)	0	1 (0.5)	2	2 (0.3)	3
Arthritis	0 (0.0)	0	0 (0.0)	0	1 (0.6)	1	0 (0.0)	0	1 (0.1)	1
Intervertebral disc protrusion	0 (0.0)	0	0 (0.0)	0	0 (0.0)	0	1 (0.5)	1	1 (0.1)	1
Muscle twitching	0 (0.0)	0	0 (0.0)	0	0 (0.0)	0	1 (0.5)	1	1 (0.1)	1
Musculoskeletal chest pain	0 (0.0)	0	0 (0.0)	0	0 (0.0)	0	1 (0.5)	1	1 (0.1)	1
Neck pain	0 (0.0)	0	0 (0.0)	0	1 (0.6)	1	0 (0.0)	0	1 (0.1)	1
General disorders and administration site conditions										
Any PT	6 (3.3)	7	1 (0.6)	1	1 (0.6)	1	4 (2.0)	6	12 (1.6)	15
Injection site pruritus	1 (0.5)	1	0 (0.0)	0	0 (0.0)	0	3 (1.5)	3	4 (0.5)	4
Injection site warmth	2 (1.1)	2	0 (0.0)	0	0 (0.0)	0	1 (0.5)	1	3 (0.4)	3
Asthenia	1 (0.5)	1	0 (0.0)	0	0 (0.0)	0	0 (0.0)	0	1 (0.1)	1
Chest pain	1 (0.5)	1	0 (0.0)	0	0 (0.0)	0	0 (0.0)	0	1 (0.1)	1
Feeling hot	0 (0.0)	0	0 (0.0)	0	0 (0.0)	0	1 (0.5)	1	1 (0.1)	1
Influenza like illness	1 (0.5)	1	0 (0.0)	0	0 (0.0)	0	0 (0.0)	0	1 (0.1)	1

A subject with more than one finding in a specific category was only counted once
Percentages based on total no. of subjects in each group
Table is sorted by descending subject count for the total of all treatment groups
Intensity grade 1/2/3/4 was decoded in mild/moderate/severe/life-threatening

Unsolicited adverse events: MedDRA coding by SOC, PT, intensity and subject, Safety dataset

Intensity<br>SOC<br>Preferred Term	Number of Subjects (%) - Number of events<br>Group 1 (MM)<br>(N=183)		Group 2 (MP)<br>(N=181)		Group 3 (PP)<br>(N=181)		Group 4 (M-)<br>(N=200)		Total<br>(N=745)	
Injection site irritation	0 (0.0)	0	0 (0.0)	0	0 (0.0)	0	1 (0.5)	1	1 (0.1)	1
Injection site paraesthesia	1 (0.5)	1	0 (0.0)	0	0 (0.0)	0	0 (0.0)	0	1 (0.1)	1
Pain	0 (0.0)	0	0 (0.0)	0	1 (0.6)	1	0 (0.0)	0	1 (0.1)	1
Pyrexia	0 (0.0)	0	1 (0.6)	1	0 (0.0)	0	0 (0.0)	0	1 (0.1)	1
Injury, poisoning and procedural complications										
Any PT	4 (2.2)	4	2 (1.1)	2	3 (1.7)	3	0 (0.0)	0	9 (1.2)	9
Contusion	0 (0.0)	0	2 (1.1)	2	0 (0.0)	0	0 (0.0)	0	2 (0.3)	2
Skin laceration	2 (1.1)	2	0 (0.0)	0	0 (0.0)	0	0 (0.0)	0	2 (0.3)	2
Back injury	1 (0.5)	1	0 (0.0)	0	0 (0.0)	0	0 (0.0)	0	1 (0.1)	1
Joint dislocation	0 (0.0)	0	0 (0.0)	0	1 (0.6)	1	0 (0.0)	0	1 (0.1)	1
Limb injury	0 (0.0)	0	0 (0.0)	0	1 (0.6)	1	0 (0.0)	0	1 (0.1)	1
Procedural pain	0 (0.0)	0	0 (0.0)	0	1 (0.6)	1	0 (0.0)	0	1 (0.1)	1
Skin injury	1 (0.5)	1	0 (0.0)	0	0 (0.0)	0	0 (0.0)	0	1 (0.1)	1
Psychiatric disorders										
Any PT	0 (0.0)	0	1 (0.6)	1	2 (1.1)	2	1 (0.5)	1	4 (0.5)	4
Initial insomnia	0 (0.0)	0	0 (0.0)	0	1 (0.6)	1	0 (0.0)	0	1 (0.1)	1
Insomnia	0 (0.0)	0	1 (0.6)	1	0 (0.0)	0	0 (0.0)	0	1 (0.1)	1
Restlessness	0 (0.0)	0	0 (0.0)	0	0 (0.0)	0	1 (0.5)	1	1 (0.1)	1
Sleep disorder	0 (0.0)	0	0 (0.0)	0	1 (0.6)	1	0 (0.0)	0	1 (0.1)	1
Skin and subcutaneous tissue disorders										
Any PT	1 (0.5)	1	1 (0.6)	1	0 (0.0)	0	1 (0.5)	2	3 (0.4)	4
Hyperhidrosis	0 (0.0)	0	0 (0.0)	0	0 (0.0)	0	1 (0.5)	2	1 (0.1)	2
Rash	0 (0.0)	0	1 (0.6)	1	0 (0.0)	0	0 (0.0)	0	1 (0.1)	1
Rash papular	1 (0.5)	1	0 (0.0)	0	0 (0.0)	0	0 (0.0)	0	1 (0.1)	1

A subject with more than one finding in a specific category was only counted once
Percentages based on total no. of subjects in each group
Table is sorted by descending subject count for the total of all treatment groups
Intensity grade 1/2/3/4 was decoded in mild/moderate/severe/life-threatening

Unsolicited adverse events: MedDRA coding by SOC, PT, intensity and subject, Safety dataset

Intensity / SOC / Preferred Term	Group 1 (MM) (N=183)		Group 2 (MP) (N=181)		Group 3 (PP) (N=181)		Group 4 (M-) (N=200)		Total (N=745)	
	Number of Subjects (%) - Number of events									
Surgical and medical procedures										
Any PT	1 (0.5)	1	1 (0.6)	1	1 (0.6)	1	0 (0.0)	0	3 (0.4)	3
Nasal operation	1 (0.5)	1	0 (0.0)	0	0 (0.0)	0	0 (0.0)	0	1 (0.1)	1
Tooth repair	0 (0.0)	0	1 (0.6)	1	0 (0.0)	0	0 (0.0)	0	1 (0.1)	1
Varicose vein operation	0 (0.0)	0	0 (0.0)	0	1 (0.6)	1	0 (0.0)	0	1 (0.1)	1
Blood and lymphatic system disorders										
Any PT	1 (0.5)	1	0 (0.0)	0	1 (0.6)	1	0 (0.0)	0	2 (0.3)	2
Lymph node pain	1 (0.5)	1	0 (0.0)	0	0 (0.0)	0	0 (0.0)	0	1 (0.1)	1
Lymphadenopathy	0 (0.0)	0	0 (0.0)	0	1 (0.6)	1	0 (0.0)	0	1 (0.1)	1
Cardiac disorders										
Any PT	0 (0.0)	0	1 (0.6)	1	0 (0.0)	0	1 (0.5)	2	2 (0.3)	3
Palpitations	0 (0.0)	0	1 (0.6)	1	0 (0.0)	0	1 (0.5)	2	2 (0.3)	3
Reproductive system and breast disorders										
Any PT	2 (1.1)	2	0 (0.0)	0	0 (0.0)	0	0 (0.0)	0	2 (0.3)	2
Dysmenorrhoea	2 (1.1)	2	0 (0.0)	0	0 (0.0)	0	0 (0.0)	0	2 (0.3)	2
Ear and labyrinth disorders										
Any PT	1 (0.5)	1	0 (0.0)	0	0 (0.0)	0	0 (0.0)	0	1 (0.1)	1
Ear pain	1 (0.5)	1	0 (0.0)	0	0 (0.0)	0	0 (0.0)	0	1 (0.1)	1
Eye disorders										
Any PT	0 (0.0)	0	1 (0.6)	1	0 (0.0)	0	0 (0.0)	0	1 (0.1)	1
Visual disturbance	0 (0.0)	0	1 (0.6)	1	0 (0.0)	0	0 (0.0)	0	1 (0.1)	1

A subject with more than one finding in a specific category was only counted once
Percentages based on total no. of subjects in each group
Table is sorted by descending subject count for the total of all treatment groups
Intensity grade 1/2/3/4 was decoded in mild/moderate/severe/life-threatening

Unsolicited adverse events: MedDRA coding by SOC, PT, intensity and subject, Safety dataset

Intensity / SOC / Preferred Term	Group 1 (MM) (N=183)		Group 2 (MP) (N=181)		Group 3 (PP) (N=181)		Group 4 (M-) (N=200)		Total (N=745)	
	Number of Subjects (%) - Number of events									
Immune system disorders										
Any PT	0 (0.0)	0	1 (0.6)	1	0 (0.0)	0	0 (0.0)	0	1 (0.1)	1
Hypersensitivity	0 (0.0)	0	1 (0.6)	1	0 (0.0)	0	0 (0.0)	0	1 (0.1)	1
Metabolism and nutrition disorders										
Any PT	0 (0.0)	0	0 (0.0)	0	1 (0.6)	1	0 (0.0)	0	1 (0.1)	1
Polydipsia	0 (0.0)	0	0 (0.0)	0	1 (0.6)	1	0 (0.0)	0	1 (0.1)	1
severe										
Infections and infestations										
Any PT	1 (0.5)	1	1 (0.6)	1	2 (1.1)	2	0 (0.0)	0	4 (0.5)	4
Nasopharyngitis	1 (0.5)	1	1 (0.6)	1	0 (0.0)	0	0 (0.0)	0	2 (0.3)	2
Otitis media	0 (0.0)	0	0 (0.0)	0	1 (0.6)	1	0 (0.0)	0	1 (0.1)	1
Sinusitis	0 (0.0)	0	0 (0.0)	0	1 (0.6)	1	0 (0.0)	0	1 (0.1)	1
Nervous system disorders										
Any PT	1 (0.5)	1	2 (1.1)	2	0 (0.0)	0	0 (0.0)	0	3 (0.4)	3
Headache	0 (0.0)	0	1 (0.6)	1	0 (0.0)	0	0 (0.0)	0	1 (0.1)	1
Hemiparesis	0 (0.0)	0	1 (0.6)	1	0 (0.0)	0	0 (0.0)	0	1 (0.1)	1
Syncope	1 (0.5)	1	0 (0.0)	0	0 (0.0)	0	0 (0.0)	0	1 (0.1)	1
Gastrointestinal disorders										
Any PT	0 (0.0)	0	1 (0.6)	2	0 (0.0)	0	1 (0.5)	1	2 (0.3)	3
Diarrhoea	0 (0.0)	0	1 (0.6)	1	0 (0.0)	0	0 (0.0)	0	1 (0.1)	1
Toothache	0 (0.0)	0	0 (0.0)	0	0 (0.0)	0	1 (0.5)	1	1 (0.1)	1
Vomiting	0 (0.0)	0	1 (0.6)	1	0 (0.0)	0	0 (0.0)	0	1 (0.1)	1

A subject with more than one finding in a specific category was only counted once
Percentages based on total no. of subjects in each group
Table is sorted by descending subject count for the total of all treatment groups
Intensity grade 1/2/3/4 was decoded in mild/moderate/severe/life-threatening

Unsolicited adverse events: MedDRA coding by SOC, PT, intensity and subject, Safety dataset

Intensity<br>SOC<br>Preferred Term	Group 1 (MM)<br>(N=183)	Group 2 (MP)<br>(N=181)	Group 3 (PP)<br>(N=181)	Group 4 (M-)<br>(N=200)	Total<br>(N=745)
	Number of Subjects (%) - Number of events				
General disorders and administration site conditions					
Any PT	1 (0.5) 1	0 (0.0) 0	1 (0.6) 1	0 (0.0) 0	2 (0.3) 2
Chills	1 (0.5) 1	0 (0.0) 0	0 (0.0) 0	0 (0.0) 0	1 (0.1) 1
Injection site urticaria	0 (0.0) 0	0 (0.0) 0	1 (0.6) 1	0 (0.0) 0	1 (0.1) 1
Surgical and medical procedures					
Any PT	0 (0.0) 0	1 (0.6) 1	1 (0.6) 1	0 (0.0) 0	2 (0.3) 2
Meniscus operation	0 (0.0) 0	0 (0.0) 0	1 (0.6) 1	0 (0.0) 0	1 (0.1) 1
Tooth extraction	0 (0.0) 0	1 (0.6) 1	0 (0.0) 0	0 (0.0) 0	1 (0.1) 1
Injury, poisoning and procedural complications					
Any PT	0 (0.0) 0	1 (0.6) 1	0 (0.0) 0	0 (0.0) 0	1 (0.1) 1
Tendon injury	0 (0.0) 0	1 (0.6) 1	0 (0.0) 0	0 (0.0) 0	1 (0.1) 1
Musculoskeletal and connective tissue disorders					
Any PT	0 (0.0) 0	1 (0.6) 1	0 (0.0) 0	0 (0.0) 0	1 (0.1) 1
Muscle tightness	0 (0.0) 0	1 (0.6) 1	0 (0.0) 0	0 (0.0) 0	1 (0.1) 1
Psychiatric disorders					
Any PT	1 (0.5) 1	0 (0.0) 0	0 (0.0) 0	0 (0.0) 0	1 (0.1) 1
Mental disorder	1 (0.5) 1	0 (0.0) 0	0 (0.0) 0	0 (0.0) 0	1 (0.1) 1
Reproductive system and breast disorders					
Any PT	0 (0.0) 0	1 (0.6) 1	0 (0.0) 0	0 (0.0) 0	1 (0.1) 1
Dysmenorrhoea	0 (0.0) 0	1 (0.6) 1	0 (0.0) 0	0 (0.0) 0	1 (0.1) 1

A subject with more than one finding in a specific category was only counted once
Percentages based on total no. of subjects in each group
Table is sorted by descending subject count for the total of all treatment groups
Intensity grade 1/2/3/4 was decoded in mild/moderate/severe/life-threatening

14.5.1.8 Unsolicited serious adverse events: MedDRA coding by SOC, PT and subject, Safety dataset

SOC<br>Preferred Term	Group 1 (MM)<br>(N=183)		Group 2 (MP)<br>(N=181)		Group 3 (PP)<br>(N=181)		Group 4 (M-)<br>(N=200)		Total<br>(N=745)		p-value
	Number of Subjects (%) - Number of events										
Injury, poisoning and procedural complications											
Any PT	0 (0.0)	0	1 (0.6)	1	0 (0.0)	0	0 (0.0)	0	1 (0.1)	1	0.486
Tendon injury	0 (0.0)	0	1 (0.6)	1	0 (0.0)	0	0 (0.0)	0	1 (0.1)	1	0.486
Nervous system disorders											
Any PT	0 (0.0)	0	1 (0.6)	1	0 (0.0)	0	0 (0.0)	0	1 (0.1)	1	0.486
Hemiparesis	0 (0.0)	0	1 (0.6)	1	0 (0.0)	0	0 (0.0)	0	1 (0.1)	1	0.486
Psychiatric disorders											
Any PT	1 (0.5)	1	0 (0.0)	0	0 (0.0)	0	0 (0.0)	0	1 (0.1)	1	0.732
Mental disorder	1 (0.5)	1	0 (0.0)	0	0 (0.0)	0	0 (0.0)	0	1 (0.1)	1	0.732

A subject with more than one finding in a specific category was only counted once
Percentages based on total no. of subjects in each group
Table is sorted by descending subject count for the total of all treatment groups

14.5.1.9 Unsolicited special interest adverse events: MedDRA coding by SOC, PT intensity and subject, Safety dataset

Intensity / SOC / Preferred Term	Group 1 (MM) (N=183)		Group 2 (MP) (N=181)		Group 3 (PP) (N=181)		Group 4 (M-) (N=200)		Total (N=745)		p-value
	Number of Subjects (%) - Number of events										
mild											
Cardiac disorders											
Any PT	3 (1.6)	3	2 (1.1)	2	1 (0.6)	1	2 (1.0)	2	8 (1.1)	8	0.876
Palpitations	1 (0.5)	1	0 (0.0)	0	1 (0.6)	1	1 (0.5)	1	3 (0.4)	3	>0.999
Tachycardia	1 (0.5)	1	1 (0.6)	1	0 (0.0)	0	1 (0.5)	1	3 (0.4)	3	>0.999
Sinus tachycardia	1 (0.5)	1	1 (0.6)	1	0 (0.0)	0	0 (0.0)	0	2 (0.3)	2	0.607
moderate											
Cardiac disorders											
Any PT	0 (0.0)	0	1 (0.6)	1	0 (0.0)	0	1 (0.5)	2	2 (0.3)	3	0.868
Palpitations	0 (0.0)	0	1 (0.6)	1	0 (0.0)	0	1 (0.5)	2	2 (0.3)	3	0.868

A subject with more than one finding in a specific category was only counted once
Percentages based on total no. of subjects in each group
Table is sorted by descending subject count for the total of all treatment groups

14.5.2 Solicited General Adverse Events

14.5.2.1 Summary of Maximum Intensity (7-day Follow-up Period after Vaccination) by vaccination period, Safety dataset

	Number of Patients (%) / Descriptive Statistics					
1st Vaccination	Group 1 (MM) (N=182)	Group 2 (MP) (N=179)	Group 3 (PP) (N=179)	Group 4 (M-) (N=200)	Total (N=740)	p-value
Body temperature increased						
Grade 0	170 (93.4)	166 (92.7)	174 (97.2)	189 (94.5)	699 (94.5)	0.338~
Grade 1	8 (4.4)	10 (5.6)	5 (2.8)	10 (5.0)	33 (4.5)	
Grade 2	3 (1.6)	3 (1.7)	0 (0.0)	0 (0.0)	6 (0.8)	
Grade 3	0 (0.0)	0 (0.0)	0 (0.0)	0 (0.0)	0 (0.0)	
Grade 4	0 (0.0)	0 (0.0)	0 (0.0)	0 (0.0)	0 (0.0)	
Headache						
Grade 0	142 (78.0)	109 (60.9)	141 (78.8)	147 (73.5)	539 (72.8)	0.011~
Grade 1	28 (15.4)	50 (27.9)	31 (17.3)	41 (20.5)	150 (20.3)	
Grade 2	11 (6.0)	17 (9.5)	5 (2.8)	10 (5.0)	43 (5.8)	
Grade 3	1 (0.5)	3 (1.7)	2 (1.1)	2 (1.0)	8 (1.1)	
Grade 4	0 (0.0)	0 (0.0)	0 (0.0)	0 (0.0)	0 (0.0)	
Myalgia						
Grade 0	163 (89.6)	160 (89.4)	166 (92.7)	158 (79.0)	647 (87.4)	0.002~
Grade 1	17 (9.3)	14 (7.8)	9 (5.0)	33 (16.5)	73 (9.9)	
Grade 2	2 (1.1)	5 (2.8)	4 (2.2)	9 (4.5)	20 (2.7)	
Grade 3	0 (0.0)	0 (0.0)	0 (0.0)	0 (0.0)	0 (0.0)	
Grade 4	0 (0.0)	0 (0.0)	0 (0.0)	0 (0.0)	0 (0.0)	

N: Number of subjects in the specified group with patient diaries recording general symptoms

%: Percentage based on total number of subjects
~ Chi-square test

Summary of Maximum Intensity (7-day Follow-up Period after Vaccination) by vaccination period, Safety dataset

		Number of Patients (%) / Descriptive Statistics					
	1st Vaccination	Group 1 (MM) (N=182)	Group 2 (MP) (N=179)	Group 3 (PP) (N=179)	Group 4 (M-) (N=200)	Total (N=740)	p-value
Nausea							
	Grade 0	171 (94.0)	161 (89.9)	169 (94.4)	182 (91.0)	683 (92.3)	0.186~
	Grade 1	9 (4.9)	12 (6.7)	7 (3.9)	16 (8.0)	44 (5.9)	
	Grade 2	2 (1.1)	2 (1.1)	2 (1.1)	2 (1.0)	8 (1.1)	
	Grade 3	0 (0.0)	4 (2.2)	1 (0.6)	0 (0.0)	5 (0.7)	
	Grade 4	0 (0.0)	0 (0.0)	0 (0.0)	0 (0.0)	0 (0.0)	
Fatigue							
	Grade 0	127 (69.8)	128 (71.5)	137 (76.5)	122 (61.0)	514 (69.5)	0.061~
	Grade 1	43 (23.6)	36 (20.1)	34 (19.0)	52 (26.0)	165 (22.3)	
	Grade 2	11 (6.0)	13 (7.3)	6 (3.4)	21 (10.5)	51 (6.9)	
	Grade 3	1 (0.5)	2 (1.1)	2 (1.1)	5 (2.5)	10 (1.4)	
	Grade 4	0 (0.0)	0 (0.0)	0 (0.0)	0 (0.0)	0 (0.0)	

N: Number of subjects in the specified group with patient diaries recording general symptoms

%: Percentage based on total number of subjects
~ Chi-square test

Summary of Maximum Intensity (7-day Follow-up Period after Vaccination) by vaccination period, Safety dataset

2nd Vaccination		Group 1 (MM) (N=182)	Group 2 (MP) (N=179)	Group 3 (PP) (N=179)	Total (N=540)	p-value
		Number of Patients (%) / Descriptive Statistics				
Body temperature increased						
	Grade 0	169 (92.9)	166 (92.7)	170 (95.0)	505 (93.5)	0.682~
	Grade 1	7 (3.8)	7 (3.9)	3 (1.7)	17 (3.1)	
	Grade 2	2 (1.1)	1 (0.6)	2 (1.1)	5 (0.9)	
Headache						
	Grade 0	143 (78.6)	139 (77.7)	156 (87.2)	438 (81.1)	0.096~
	Grade 1	25 (13.7)	24 (13.4)	11 (6.1)	60 (11.1)	
	Grade 2	9 (4.9)	8 (4.5)	8 (4.5)	25 (4.6)	
	Grade 3	1 (0.5)	3 (1.7)	0 (0.0)	4 (0.7)	
Myalgia						
	Grade 0	166 (91.2)	170 (95.0)	162 (90.5)	498 (92.2)	0.068~
	Grade 1	10 (5.5)	1 (0.6)	11 (6.1)	22 (4.1)	
	Grade 2	2 (1.1)	3 (1.7)	2 (1.1)	7 (1.3)	
Nausea						
	Grade 0	170 (93.4)	166 (92.7)	171 (95.5)	507 (93.9)	0.633~
	Grade 1	6 (3.3)	4 (2.2)	2 (1.1)	12 (2.2)	
	Grade 2	2 (1.1)	3 (1.7)	2 (1.1)	7 (1.3)	

N: Number of subjects in the specified group with patient diaries recording general symptoms

%: Percentage based on total number of subjects
~ Chi-square test

Summary of Maximum Intensity (7-day Follow-up Period after Vaccination) by vaccination period, Safety dataset

	2nd Vaccination	Group 1 (MM) (N=182)	Group 2 (MP) (N=179)	Group 3 (PP) (N=179)	Total (N=540)	p-value
	Grade 3	0 (0.0)	1 (0.6)	0 (0.0)	1 (0.2)	
Fatigue						
	Grade 0	144 (79.1)	146 (81.6)	146 (81.6)	436 (80.7)	0.527~
	Grade 1	26 (14.3)	21 (11.7)	18 (10.1)	65 (12.0)	
	Grade 2	8 (4.4)	5 (2.8)	10 (5.6)	23 (4.3)	
	Grade 3	0 (0.0)	2 (1.1)	1 (0.6)	3 (0.6)	

Number of Patients (%) / Descriptive Statistics

N: Number of subjects in the specified group with patient diaries recording general symptoms

%: Percentage based on total number of subjects
~ Chi-square test

14.5.2.2 Summary of Maximum Intensity (7-day Follow-up Period after Vaccination) by subject, Safety dataset

	Number of Patients (%) / Descriptive Statistics					
	Group 1 (MM) (N=182)	Group 2 (MP) (N=179)	Group 3 (PP) (N=179)	Group 4 (M-) (N=200)	Total (N=740)	p-value
Body temperature increased						
Grade 0	164 (90.1)	158 (88.3)	169 (94.4)	189 (94.5)	680 (91.9)	0.096~
Grade 1	13 (7.1)	17 (9.5)	8 (4.5)	10 (5.0)	48 (6.5)	
Grade 2	5 (2.7)	4 (2.2)	2 (1.1)	0 (0.0)	11 (1.5)	
Grade 3	0 (0.0)	0 (0.0)	0 (0.0)	0 (0.0)	0 (0.0)	
Grade 4	0 (0.0)	0 (0.0)	0 (0.0)	0 (0.0)	0 (0.0)	
Missing	0	0	0	1	1	
Headache						
Grade 0	122 (67.0)	95 (53.1)	130 (72.6)	147 (73.5)	494 (66.8)	0.002~
Grade 1	39 (21.4)	57 (31.8)	35 (19.6)	41 (20.5)	172 (23.2)	
Grade 2	19 (10.4)	21 (11.7)	12 (6.7)	10 (5.0)	62 (8.4)	
Grade 3	2 (1.1)	6 (3.4)	2 (1.1)	2 (1.0)	12 (1.6)	
Grade 4	0 (0.0)	0 (0.0)	0 (0.0)	0 (0.0)	0 (0.0)	
Myalgia						
Grade 0	153 (84.1)	157 (87.7)	159 (88.8)	158 (79.0)	627 (84.7)	0.054~
Grade 1	25 (13.7)	14 (7.8)	14 (7.8)	33 (16.5)	86 (11.6)	
Grade 2	4 (2.2)	8 (4.5)	6 (3.4)	9 (4.5)	27 (3.6)	
Grade 3	0 (0.0)	0 (0.0)	0 (0.0)	0 (0.0)	0 (0.0)	

N: Number of subjects in the specified group with patient diaries recording general symptoms

%: Percentage based on total number of subjects
~ Chi-square test

Summary of Maximum Intensity (7-day Follow-up Period after Vaccination) by subject, Safety dataset

		Group 1 (MM) (N=182)	Group 2 (MP) (N=179)	Group 3 (PP) (N=179)	Group 4 (M-) (N=200)	Total (N=740)	p-value
		Number of Patients (%) / Descriptive Statistics					
	Grade 4	0 (0.0)	0 (0.0)	0 (0.0)	0 (0.0)	0 (0.0)	
Nausea							
	Grade 0	165 (90.7)	157 (87.7)	166 (92.7)	182 (91.0)	670 (90.5)	0.073~
	Grade 1	13 (7.1)	12 (6.7)	8 (4.5)	16 (8.0)	49 (6.6)	
	Grade 2	4 (2.2)	5 (2.8)	4 (2.2)	2 (1.0)	15 (2.0)	
	Grade 3	0 (0.0)	5 (2.8)	1 (0.6)	0 (0.0)	6 (0.8)	
	Grade 4	0 (0.0)	0 (0.0)	0 (0.0)	0 (0.0)	0 (0.0)	
Fatigue							
	Grade 0	114 (62.6)	120 (67.0)	124 (69.3)	122 (61.0)	480 (64.9)	0.663~
	Grade 1	51 (28.0)	41 (22.9)	38 (21.2)	52 (26.0)	182 (24.6)	
	Grade 2	16 (8.8)	14 (7.8)	14 (7.8)	21 (10.5)	65 (8.8)	
	Grade 3	1 (0.5)	4 (2.2)	3 (1.7)	5 (2.5)	13 (1.8)	
	Grade 4	0 (0.0)	0 (0.0)	0 (0.0)	0 (0.0)	0 (0.0)	

N: Number of subjects in the specified group with patient diaries recording general symptoms

%: Percentage based on total number of subjects
~ Chi-square test

14.5.2.3 Summary of Maximum Intensity by subject: categories grade=0/ >0, Safety dataset

	Number of Patients (%) / Descriptive Statistics					
	Group 1 (MM) (N=182)	Group 2 (MP) (N=179)	Group 3 (PP) (N=179)	Group 4 (M-) (N=200)	Total (N=740)	p-value
Body temperature increased						
Grade =0	164 (90.1)	158 (88.3)	169 (94.4)	189 (94.5)	680 (91.9)	0.044~
Grade >0	18 (9.9)	21 (11.7)	10 (5.6)	10 (5.0)	59 (8.0)	
Missing	0	0	0	1	1	
Headache						
Grade =0	122 (67.0)	95 (53.1)	130 (72.6)	147 (73.5)	494 (66.8)	<0.001~
Grade >0	60 (33.0)	84 (46.9)	49 (27.4)	53 (26.5)	246 (33.2)	
Myalgia						
Grade =0	153 (84.1)	157 (87.7)	159 (88.8)	158 (79.0)	627 (84.7)	0.034~
Grade >0	29 (15.9)	22 (12.3)	20 (11.2)	42 (21.0)	113 (15.3)	
Nausea						
Grade =0	165 (90.7)	157 (87.7)	166 (92.7)	182 (91.0)	670 (90.5)	0.434~
Grade >0	17 (9.3)	22 (12.3)	13 (7.3)	18 (9.0)	70 (9.5)	
Fatigue						
Grade =0	114 (62.6)	120 (67.0)	124 (69.3)	122 (61.0)	480 (64.9)	0.307~
Grade >0	68 (37.4)	59 (33.0)	55 (30.7)	78 (39.0)	260 (35.1)	

N: Number of subjects in the specified group with patient diaries recording general symptoms

%: Percentage based on total number of subjects
~ Chi-square test

14.5.2.4 Summary of Maximum Intensity by subject: categories grade<2/ >=2, Safety dataset

	Number of Patients (%) / Descriptive Statistics					
	Group 1 (MM) (N=182)	Group 2 (MP) (N=179)	Group 3 (PP) (N=179)	Group 4 (M-) (N=200)	Total (N=740)	p-value
Body temperature increased						
Grade <2	177 (97.3)	175 (97.8)	177 (98.9)	199 (99.5)	728 (98.4)	0.121~
Grade >=2	5 (2.7)	4 (2.2)	2 (1.1)	0 (0.0)	11 (1.5)	
Missing	0	0	0	1	1	
Headache						
Grade <2	161 (88.5)	152 (84.9)	165 (92.2)	188 (94.0)	666 (90.0)	0.018~
Grade >=2	21 (11.5)	27 (15.1)	14 (7.8)	12 (6.0)	74 (10.0)	
Myalgia						
Grade <2	178 (97.8)	171 (95.5)	173 (96.6)	191 (95.5)	713 (96.4)	0.596~
Grade >=2	4 (2.2)	8 (4.5)	6 (3.4)	9 (4.5)	27 (3.6)	
Nausea						
Grade <2	178 (97.8)	169 (94.4)	174 (97.2)	198 (99.0)	719 (97.2)	0.054~
Grade >=2	4 (2.2)	10 (5.6)	5 (2.8)	2 (1.0)	21 (2.8)	
Fatigue						
Grade <2	165 (90.7)	161 (89.9)	162 (90.5)	174 (87.0)	662 (89.5)	0.612~
Grade >=2	17 (9.3)	18 (10.1)	17 (9.5)	26 (13.0)	78 (10.5)	

N: Number of subjects in the specified group with patient diaries recording general symptoms

%: Percentage based on total number of subjects
~ Chi-square test

14.5.2.5 Summary of Maximum Intensity by subject: categories grade<3/ >=3, Safety dataset

	Number of Patients (%) / Descriptive Statistics Group 1 (MM) (N=182)	Group 2 (MP) (N=179)	Group 3 (PP) (N=179)	Group 4 (M-) (N=200)	Total (N=740)	p-value
Body temperature increased						
Grade <3	182 (100.0)	179 (100.0)	179 (100.0)	199 (99.5)	739 (99.9)	n/a~
Grade >=3	0 (0.0)	0 (0.0)	0 (0.0)	0 (0.0)	0 (0.0)	
Missing	0	0	0	1	1	
Headache						
Grade <3	180 (98.9)	173 (96.6)	177 (98.9)	198 (99.0)	728 (98.4)	0.218~
Grade >=3	2 (1.1)	6 (3.4)	2 (1.1)	2 (1.0)	12 (1.6)	
Myalgia						
Grade <3	182 (100.0)	179 (100.0)	179 (100.0)	200 (100.0)	740 (100.0)	n/a~
Grade >=3	0 (0.0)	0 (0.0)	0 (0.0)	0 (0.0)	0 (0.0)	
Nausea						
Grade <3	182 (100.0)	174 (97.2)	178 (99.4)	200 (100.0)	734 (99.2)	0.007~
Grade >=3	0 (0.0)	5 (2.8)	1 (0.6)	0 (0.0)	6 (0.8)	
Fatigue						
Grade <3	181 (99.5)	175 (97.8)	176 (98.3)	195 (97.5)	727 (98.2)	0.490~
Grade >=3	1 (0.5)	4 (2.2)	3 (1.7)	5 (2.5)	13 (1.8)	

N: Number of subjects in the specified group with patient diaries recording general symptoms

%: Percentage based on total number of subjects
~ Chi-square test

14.5.2.6 Summary of Maximum Intensity (7-day Follow-up Period after Vaccination) by subject - related AEs, Safety dataset

	Number of Patients (%) / Descriptive Statistics					
	Group 1 (MM) (N=182)	Group 2 (MP) (N=179)	Group 3 (PP) (N=179)	Group 4 (M-) (N=200)	Total (N=740)	p-value
Body temperature increased						
Grade 0	0 (0.0)	0 (0.0)	0 (0.0)	0 (0.0)	0 (0.0)	0.379~
Grade 1	1 (0.5)	0 (0.0)	1 (0.6)	0 (0.0)	2 (0.3)	
Grade 2	2 (1.1)	3 (1.7)	1 (0.6)	0 (0.0)	6 (0.8)	
Grade 3	0 (0.0)	0 (0.0)	0 (0.0)	0 (0.0)	0 (0.0)	
Grade 4	0 (0.0)	0 (0.0)	0 (0.0)	0 (0.0)	0 (0.0)	
Headache						
Grade 0	0 (0.0)	0 (0.0)	0 (0.0)	0 (0.0)	0 (0.0)	0.010~
Grade 1	25 (13.7)	39 (21.8)	21 (11.7)	41 (20.5)	126 (17.0)	
Grade 2	15 (8.2)	15 (8.4)	9 (5.0)	8 (4.0)	47 (6.4)	
Grade 3	2 (1.1)	4 (2.2)	1 (0.6)	2 (1.0)	9 (1.2)	
Grade 4	0 (0.0)	0 (0.0)	0 (0.0)	0 (0.0)	0 (0.0)	
Myalgia						
Grade 0	0 (0.0)	0 (0.0)	0 (0.0)	0 (0.0)	0 (0.0)	0.058~
Grade 1	19 (10.4)	9 (5.0)	12 (6.7)	32 (16.0)	72 (9.7)	
Grade 2	4 (2.2)	6 (3.4)	2 (1.1)	7 (3.5)	19 (2.6)	
Grade 3	0 (0.0)	0 (0.0)	0 (0.0)	0 (0.0)	0 (0.0)	
Grade 4	0 (0.0)	0 (0.0)	0 (0.0)	0 (0.0)	0 (0.0)	
Nausea						
Grade 0	0 (0.0)	0 (0.0)	0 (0.0)	0 (0.0)	0 (0.0)	0.157~
Grade 1	10 (5.5)	10 (5.6)	5 (2.8)	15 (7.5)	40 (5.4)	

Relationship to study medication: possible/probable/definite

Summary of Maximum Intensity (7-day Follow-up Period after Vaccination) by subject - related AEs, Safety dataset

	Group 1 (MM) (N=182)	Group 2 (MP) (N=179)	Group 3 (PP) (N=179)	Group 4 (M-) (N=200)	Total (N=740)	p-value
	Number of Patients (%) / Descriptive Statistics					
Grade 2	2 (1.1)	5 (2.8)	3 (1.7)	1 (0.5)	11 (1.5)	
Grade 3	0 (0.0)	2 (1.1)	0 (0.0)	0 (0.0)	2 (0.3)	
Grade 4	0 (0.0)	0 (0.0)	0 (0.0)	0 (0.0)	0 (0.0)	
Fatigue						
Grade 0	0 (0.0)	0 (0.0)	0 (0.0)	0 (0.0)	0 (0.0)	0.472~
Grade 1	41 (22.5)	29 (16.2)	29 (16.2)	47 (23.5)	146 (19.7)	
Grade 2	15 (8.2)	11 (6.1)	13 (7.3)	19 (9.5)	58 (7.8)	
Grade 3	1 (0.5)	2 (1.1)	3 (1.7)	5 (2.5)	11 (1.5)	
Grade 4	0 (0.0)	0 (0.0)	0 (0.0)	0 (0.0)	0 (0.0)	

Relationship to study medication: possible/probable/definite

14.5.3 Solicited Local Adverse Events

14.5.3.1 Summary of Maximum Intensity (7-Day Follow-up Period after Vaccination) by vaccination period, Safety dataset

Dose=1st vaccination

	Number of Patients (%) / Descriptive Statistics					
	Group 1 (MM) (N=182)	Group 2 (MP) (N=179)	Group 3 (PP) (N=179)	Group 4 (M-) (N=200)	Total (N=740)	p-value
Injection site pain						
Grade 0	27 (14.8)	26 (14.5)	157 (87.7)	33 (16.5)	243 (32.8)	<0.001~
Grade 1	121 (66.5)	113 (63.1)	16 (8.9)	123 (61.5)	373 (50.4)	
Grade 2	33 (18.1)	40 (22.3)	6 (3.4)	41 (20.5)	120 (16.2)	
Grade 3	1 (0.5)	0 (0.0)	0 (0.0)	3 (1.5)	4 (0.5)	
Grade 4	0 (0.0)	0 (0.0)	0 (0.0)	0 (0.0)	0 (0.0)	
Injection site erythema						
Grade 0	31 (17.0)	35 (19.6)	152 (84.9)	31 (15.5)	249 (33.6)	<0.001~
Grade 1	101 (55.5)	85 (47.5)	26 (14.5)	71 (35.5)	283 (38.2)	
Grade 2	50 (27.5)	59 (33.0)	1 (0.6)	93 (46.5)	203 (27.4)	
Grade 3	0 (0.0)	0 (0.0)	0 (0.0)	5 (2.5)	5 (0.7)	
Grade 4	0 (0.0)	0 (0.0)	0 (0.0)	0 (0.0)	0 (0.0)	
Injection site swelling						
Grade 0	76 (41.8)	77 (43.0)	174 (97.2)	51 (25.5)	378 (51.1)	<0.001~
Grade 1	71 (39.0)	70 (39.1)	5 (2.8)	91 (45.5)	237 (32.0)	
Grade 2	35 (19.2)	32 (17.9)	0 (0.0)	57 (28.5)	124 (16.8)	
Grade 3	0 (0.0)	0 (0.0)	0 (0.0)	1 (0.5)	1 (0.1)	
Grade 4	0 (0.0)	0 (0.0)	0 (0.0)	0 (0.0)	0 (0.0)	

N: Number of subjects in the specified group with patient diaries recording general symptoms

%: Percentage based on total number of subjects
~ Chi-square test

Summary of Maximum Intensity (7-Day Follow-up Period after Vaccination) by vaccination period, Safety dataset

Dose=1st vaccination

	Group 1 (MM) (N=182)	Group 2 (MP) (N=179)	Group 3 (PP) (N=179)	Group 4 (M-) (N=200)	Total (N=740)	p-value
	Number of Patients (%) / Descriptive Statistics					
Injection site induration						
Grade 0	41 (22.5)	33 (18.4)	175 (97.8)	45 (22.5)	294 (39.7)	<0.001~
Grade 1	107 (58.8)	111 (62.0)	4 (2.2)	97 (48.5)	319 (43.1)	
Grade 2	34 (18.7)	35 (19.6)	0 (0.0)	57 (28.5)	126 (17.0)	
Grade 3	0 (0.0)	0 (0.0)	0 (0.0)	1 (0.5)	1 (0.1)	
Grade 4	0 (0.0)	0 (0.0)	0 (0.0)	0 (0.0)	0 (0.0)	

N: Number of subjects in the specified group with patient diaries recording general symptoms

%: Percentage based on total number of subjects
~ Chi-square test

Summary of Maximum Intensity (7-Day Follow-up Period after Vaccination) by vaccination period, Safety dataset

Dose=2nd vaccination

	Group 1 (MM) (N=182)	Group 2 (MP) (N=179)	Group 3 (PP) (N=179)	Total (N=540)	p-value
	Number of Patients (%) / Descriptive Statistics				
Injection site pain					
Grade 0	33 (18.1)	159 (88.8)	151 (84.4)	343 (63.5)	<0.001~
Grade 1	117 (64.3)	14 (7.8)	17 (9.5)	148 (27.4)	
Grade 2	28 (15.4)	1 (0.6)	7 (3.9)	36 (6.7)	
Injection site erythema					
Grade 0	26 (14.3)	158 (88.3)	151 (84.4)	335 (62.0)	<0.001~
Grade 1	50 (27.5)	16 (8.9)	22 (12.3)	88 (16.3)	
Grade 2	96 (52.7)	0 (0.0)	2 (1.1)	98 (18.1)	
Grade 3	6 (3.3)	0 (0.0)	0 (0.0)	6 (1.1)	
Injection site swelling					
Grade 0	37 (20.3)	164 (91.6)	168 (93.9)	369 (68.3)	<0.001~
Grade 1	57 (31.3)	10 (5.6)	6 (3.4)	73 (13.5)	
Grade 2	82 (45.1)	0 (0.0)	1 (0.6)	83 (15.4)	
Grade 3	2 (1.1)	0 (0.0)	0 (0.0)	2 (0.4)	

N: Number of subjects in the specified group with patient diaries recording general symptoms

%: Percentage based on total number of subjects
~ Chi-square test

Summary of Maximum Intensity (7-Day Follow-up Period after Vaccination) by vaccination period, Safety dataset

Dose=2nd vaccination

	Group 1 (MM) (N=182)	Group 2 (MP) (N=179)	Group 3 (PP) (N=179)	Total (N=540)	p-value
	Number of Patients (%) / Descriptive Statistics				
Injection site induration					
Grade 0	46 (25.3)	165 (92.2)	173 (96.6)	384 (71.1)	<0.001~
Grade 1	65 (35.7)	9 (5.0)	1 (0.6)	75 (13.9)	
Grade 2	66 (36.3)	0 (0.0)	1 (0.6)	67 (12.4)	
Grade 3	1 (0.5)	0 (0.0)	0 (0.0)	1 (0.2)	

N: Number of subjects in the specified group with patient diaries recording general symptoms

%: Percentage based on total number of subjects
~ Chi-square test

14.5.3.2 Summary of Maximum Intensity (7-Day Follow-up Period after Vaccination) by subject, Safety dataset

	Number of Patients (%) / Descriptive Statistics					
	Group 1 (MM) (N=182)	Group 2 (MP) (N=179)	Group 3 (PP) (N=179)	Group 4 (M-) (N=200)	Total (N=740)	p-value
Injection site pain						
Grade 0	16 (8.8)	24 (13.4)	142 (79.3)	33 (16.5)	215 (29.1)	<0.001~
Grade 1	117 (64.3)	114 (63.7)	25 (14.0)	123 (61.5)	379 (51.2)	
Grade 2	48 (26.4)	41 (22.9)	12 (6.7)	41 (20.5)	142 (19.2)	
Grade 3	1 (0.5)	0 (0.0)	0 (0.0)	3 (1.5)	4 (0.5)	
Grade 4	0 (0.0)	0 (0.0)	0 (0.0)	0 (0.0)	0 (0.0)	
Injection site erythema						
Grade 0	16 (8.8)	33 (18.4)	140 (78.2)	31 (15.5)	220 (29.7)	<0.001~
Grade 1	55 (30.2)	87 (48.6)	37 (20.7)	71 (35.5)	250 (33.8)	
Grade 2	105 (57.7)	59 (33.0)	2 (1.1)	93 (46.5)	259 (35.0)	
Grade 3	6 (3.3)	0 (0.0)	0 (0.0)	5 (2.5)	11 (1.5)	
Grade 4	0 (0.0)	0 (0.0)	0 (0.0)	0 (0.0)	0 (0.0)	
Injection site swelling						
Grade 0	33 (18.1)	76 (42.5)	169 (94.4)	51 (25.5)	329 (44.5)	<0.001~
Grade 1	58 (31.9)	71 (39.7)	9 (5.0)	91 (45.5)	229 (30.9)	
Grade 2	89 (48.9)	32 (17.9)	1 (0.6)	57 (28.5)	179 (24.2)	
Grade 3	2 (1.1)	0 (0.0)	0 (0.0)	1 (0.5)	3 (0.4)	
Grade 4	0 (0.0)	0 (0.0)	0 (0.0)	0 (0.0)	0 (0.0)	

N: Number of subjects in the specified group with patient diaries recording general symptoms

%: Percentage based on total number of subjects
~ Chi-square test

Summary of Maximum Intensity (7-Day Follow-up Period after Vaccination) by subject, Safety dataset

	Number of Patients (%) / Descriptive Statistics					
	Group 1 (MM) (N=182)	Group 2 (MP) (N=179)	Group 3 (PP) (N=179)	Group 4 (M-) (N=200)	Total (N=740)	p-value
Injection site induration						
Grade 0	20 (11.0)	33 (18.4)	174 (97.2)	45 (22.5)	272 (36.8)	<0.001~
Grade 1	86 (47.3)	111 (62.0)	4 (2.2)	97 (48.5)	298 (40.3)	
Grade 2	75 (41.2)	35 (19.6)	1 (0.6)	57 (28.5)	168 (22.7)	
Grade 3	1 (0.5)	0 (0.0)	0 (0.0)	1 (0.5)	2 (0.3)	
Grade 4	0 (0.0)	0 (0.0)	0 (0.0)	0 (0.0)	0 (0.0)	

N: Number of subjects in the specified group with patient diaries recording general symptoms

%: Percentage based on total number of subjects
~ Chi-square test

14.5.3.3 Summary of Maximum Intensity by subject: categories grade=0/ >0, Safety dataset

	Number of Patients (%) / Descriptive Statistics					
	Group 1 (MM) (N=182)	Group 2 (MP) (N=179)	Group 3 (PP) (N=179)	Group 4 (M-) (N=200)	Total (N=740)	p-value
Injection site pain						
Grade =0	16 (8.8)	24 (13.4)	142 (79.3)	33 (16.5)	215 (29.1)	<0.001~
Grade >0	166 (91.2)	155 (86.6)	37 (20.7)	167 (83.5)	525 (70.9)	
Injection site erythema						
Grade =0	16 (8.8)	33 (18.4)	140 (78.2)	31 (15.5)	220 (29.7)	<0.001~
Grade >0	166 (91.2)	146 (81.6)	39 (21.8)	169 (84.5)	520 (70.3)	
Injection site swelling						
Grade =0	33 (18.1)	76 (42.5)	169 (94.4)	51 (25.5)	329 (44.5)	<0.001~
Grade >0	149 (81.9)	103 (57.5)	10 (5.6)	149 (74.5)	411 (55.5)	
Injection site induration						
Grade =0	20 (11.0)	33 (18.4)	174 (97.2)	45 (22.5)	272 (36.8)	<0.001~
Grade >0	162 (89.0)	146 (81.6)	5 (2.8)	155 (77.5)	468 (63.2)	

N: Number of subjects in the specified group with patient diaries recording general symptoms

%: Percentage based on total number of subjects
~ Chi-square test

14.5.3.4 Summary of Maximum Intensity by subject: categories grade<2/ >=2, Safety dataset

	Group 1 (MM) (N=182)	Group 2 (MP) (N=179)	Group 3 (PP) (N=179)	Group 4 (M-) (N=200)	Total (N=740)	p-value
	Number of Patients (%) / Descriptive Statistics					
Injection site pain						
Grade <2	133 (73.1)	138 (77.1)	167 (93.3)	156 (78.0)	594 (80.3)	<0.001~
Grade >=2	49 (26.9)	41 (22.9)	12 (6.7)	44 (22.0)	146 (19.7)	
Injection site erythema						
Grade <2	71 (39.0)	120 (67.0)	177 (98.9)	102 (51.0)	470 (63.5)	<0.001~
Grade >=2	111 (61.0)	59 (33.0)	2 (1.1)	98 (49.0)	270 (36.5)	
Injection site swelling						
Grade <2	91 (50.0)	147 (82.1)	178 (99.4)	142 (71.0)	558 (75.4)	<0.001~
Grade >=2	91 (50.0)	32 (17.9)	1 (0.6)	58 (29.0)	182 (24.6)	
Injection site induration						
Grade <2	106 (58.2)	144 (80.4)	178 (99.4)	142 (71.0)	570 (77.0)	<0.001~
Grade >=2	76 (41.8)	35 (19.6)	1 (0.6)	58 (29.0)	170 (23.0)	

N: Number of subjects in the specified group with patient diaries recording general symptoms

%: Percentage based on total number of subjects
~ Chi-square test

14.5.3.5 Summary of Maximum Intensity by subject: categories grade<3/ >=3, Safety dataset

Number of Patients (%) / Descriptive Statistics

	Group 1 (MM) (N=182)	Group 2 (MP) (N=179)	Group 3 (PP) (N=179)	Group 4 (M-) (N=200)	Total (N=740)	p-value
Injection site pain						
Grade <3	181 (99.5)	179 (100.0)	179 (100.0)	197 (98.5)	736 (99.5)	0.147~
Grade >=3	1 (0.5)	0 (0.0)	0 (0.0)	3 (1.5)	4 (0.5)	
Injection site erythema						
Grade <3	176 (96.7)	179 (100.0)	179 (100.0)	195 (97.5)	729 (98.5)	0.012~
Grade >=3	6 (3.3)	0 (0.0)	0 (0.0)	5 (2.5)	11 (1.5)	
Injection site swelling						
Grade <3	180 (98.9)	179 (100.0)	179 (100.0)	199 (99.5)	737 (99.6)	0.299~
Grade >=3	2 (1.1)	0 (0.0)	0 (0.0)	1 (0.5)	3 (0.4)	
Injection site induration						
Grade <3	181 (99.5)	179 (100.0)	179 (100.0)	199 (99.5)	738 (99.7)	0.596~
Grade >=3	1 (0.5)	0 (0.0)	0 (0.0)	1 (0.5)	2 (0.3)	

N: Number of subjects in the specified group with patient diaries recording general symptoms

%: Percentage based on total number of subjects
~ Chi-square test

14.5.4 Possibly/probably/definitely Related Adverse Events

14.5.4.1 Solicited (general / local) and unsolicited adverse events: MedDRA coding by SOC, PT, possible/probable/definite relationship to study medication, Safety dataset

	Number of Subjects (%) - Number of events										
SOC<br>Preferred Term	Group 1 (MM)<br>(N=183)		Group 2 (MP)<br>(N=181)		Group 3 (PP)<br>(N=181)		Group 4 (M-)<br>(N=200)		Total<br>(N=745)		p-value
General disorders and administration site conditions											
Any PT	181 (98.9)	1392	177 (97.8)	824	103 (56.9)	252	194 (97.0)	894	655 (87.9)	3362	<0.001
Injection site pain	166 (90.7)	300	155 (85.6)	168	37 (20.4)	46	167 (83.5)	167	525 (70.5)	681	<0.001
Injection site erythema	166 (90.7)	304	146 (80.7)	160	39 (21.5)	51	169 (84.5)	169	520 (69.8)	684	<0.001
Injection site induration	162 (88.5)	273	146 (80.7)	155	5 (2.8)	6	155 (77.5)	155	468 (62.8)	589	<0.001
Injection site swelling	149 (81.4)	247	103 (56.9)	112	10 (5.5)	12	149 (74.5)	149	411 (55.2)	520	<0.001
Fatigue	57 (31.1)	75	42 (23.2)	52	45 (24.9)	53	71 (35.5)	71	215 (28.9)	251	0.030
Headache	42 (23.0)	49	58 (32.0)	66	31 (17.1)	36	51 (25.5)	51	182 (24.4)	202	0.010
Injection site pruritus	43 (23.5)	61	36 (19.9)	37	4 (2.2)	4	47 (23.5)	47	130 (17.4)	149	<0.001
Myalgia	23 (12.6)	24	15 (8.3)	15	14 (7.7)	18	39 (19.5)	39	91 (12.2)	96	0.002
Body temperature increased	16 (8.7)	18	19 (10.5)	19	10 (5.5)	10	10 (5.0)	10	55 (7.4)	57	0.132
Nausea	12 (6.6)	14	17 (9.4)	20	8 (4.4)	8	16 (8.0)	16	53 (7.1)	58	0.280
Injection site haematoma	6 (3.3)	6	9 (5.0)	9	4 (2.2)	4	4 (2.0)	4	23 (3.1)	23	0.373
Injection site warmth	9 (4.9)	9	5 (2.8)	5	0 (0.0)	0	8 (4.0)	8	22 (3.0)	22	0.009
Injection site discolouration	1 (0.5)	1	4 (2.2)	4	0 (0.0)	0	0 (0.0)	0	5 (0.7)	5	0.021
Injection site irritation	1 (0.5)	2	1 (0.6)	1	0 (0.0)	0	3 (1.5)	3	5 (0.7)	6	0.413
Asthenia	2 (1.1)	2	0 (0.0)	0	1 (0.6)	1	1 (0.5)	1	4 (0.5)	4	0.751
Axillary pain	1 (0.5)	1	1 (0.6)	1	0 (0.0)	0	0 (0.0)	0	2 (0.3)	2	0.607
Injection site hypersensitivity	1 (0.5)	1	0 (0.0)	0	1 (0.6)	1	0 (0.0)	0	2 (0.3)	2	0.607
Injection site reaction	1 (0.5)	1	0 (0.0)	0	0 (0.0)	0	1 (0.5)	1	2 (0.3)	2	>0.999
Chills	1 (0.5)	1	0 (0.0)	0	0 (0.0)	0	0 (0.0)	0	1 (0.1)	1	0.732
Feeling hot	0 (0.0)	0	0 (0.0)	0	0 (0.0)	0	1 (0.5)	1	1 (0.1)	1	>0.999
Implant site pain	1 (0.5)	1	0 (0.0)	0	0 (0.0)	0	0 (0.0)	0	1 (0.1)	1	0.732
Injection site paraesthesia	1 (0.5)	1	0 (0.0)	0	0 (0.0)	0	0 (0.0)	0	1 (0.1)	1	0.732
Injection site urticaria	0 (0.0)	0	0 (0.0)	0	1 (0.6)	1	0 (0.0)	0	1 (0.1)	1	0.486

Relationship to study medication: possible/probable/definite for unsolicited AEs
Solicited Local Adverse Events are included if maximum intensity during first 7 days was >0
For Solicited Local Adverse Events 'other' category was excluded
For Solicited General AEs, AEs recorded under the 'other' category have been transcribed to unsolicted AEs during data collection
For Solicited General AEs, all AEs with missing or unknown relationship and possible/probable/definite relationship and maximum intensity during first 7 days >0

Solicited (general / local) and unsolicited adverse events: MedDRA coding by SOC, PT, possible/probable/definite relationship to study medication, Safety datase

SOC<br>Preferred Term	Group 1 (MM) (N=183)		Group 2 (MP) (N=181)		Group 3 (PP) (N=181)		Group 4 (M-) (N=200)		Total (N=745)		p-value
	Number of Subjects (%)	Number of events									
Malaise	0 (0.0)	0	0 (0.0)	0	0 (0.0)	0	1 (0.5)	1	1 (0.1)	1	>0.999
Non-cardiac chest pain	1 (0.5)	1	0 (0.0)	0	0 (0.0)	0	0 (0.0)	0	1 (0.1)	1	0.732
Soft tissue inflammation	0 (0.0)	0	0 (0.0)	0	1 (0.6)	1	0 (0.0)	0	1 (0.1)	1	0.486
Thirst	0 (0.0)	0	0 (0.0)	0	0 (0.0)	0	1 (0.5)	1	1 (0.1)	1	>0.999
Nervous system disorders											
Any PT	1 (0.5)	1	6 (3.3)	6	3 (1.7)	3	6 (3.0)	7	16 (2.1)	17	0.214
Dizziness	1 (0.5)	1	2 (1.1)	2	2 (1.1)	2	4 (2.0)	4	9 (1.2)	9	0.617
Headache	0 (0.0)	0	3 (1.7)	3	1 (0.6)	1	1 (0.5)	1	5 (0.7)	5	0.267
Disturbance in attention	0 (0.0)	0	0 (0.0)	0	0 (0.0)	0	1 (0.5)	1	1 (0.1)	1	>0.999
Paraesthesia	0 (0.0)	0	1 (0.6)	1	0 (0.0)	0	0 (0.0)	0	1 (0.1)	1	0.486
Sedation	0 (0.0)	0	0 (0.0)	0	0 (0.0)	0	1 (0.5)	1	1 (0.1)	1	>0.999
Infections and infestations											
Any PT	6 (3.3)	7	1 (0.6)	1	5 (2.8)	6	2 (1.0)	3	14 (1.9)	17	0.154
Nasopharyngitis	5 (2.7)	5	0 (0.0)	0	4 (2.2)	4	1 (0.5)	1	10 (1.3)	10	0.044
Rhinitis	0 (0.0)	0	1 (0.6)	1	2 (1.1)	2	0 (0.0)	0	3 (0.4)	3	0.148
Sinusitis	1 (0.5)	1	0 (0.0)	0	0 (0.0)	0	1 (0.5)	1	2 (0.3)	2	>0.999
Bronchitis	0 (0.0)	0	0 (0.0)	0	0 (0.0)	0	1 (0.5)	1	1 (0.1)	1	>0.999
Tonsillitis	1 (0.5)	1	0 (0.0)	0	0 (0.0)	0	0 (0.0)	0	1 (0.1)	1	0.732
Gastrointestinal disorders											
Any PT	2 (1.1)	3	1 (0.6)	1	2 (1.1)	3	7 (3.5)	7	12 (1.6)	14	0.167
Diarrhoea	0 (0.0)	0	1 (0.6)	1	1 (0.6)	1	4 (2.0)	4	6 (0.8)	6	0.160
Dry mouth	2 (1.1)	3	0 (0.0)	0	0 (0.0)	0	1 (0.5)	1	3 (0.4)	4	0.466

Relationship to study medication: possible/probable/definite for unsolicited AEs
Solicited Local Adverse Events are included if maximum intensity during first 7 days was >0
For Solicited Local Adverse Events 'other' category was excluded
For Solicited General AEs, AEs recorded under the 'other' category have been transcribed to unsolicted AEs during data collection
For Solicited General AEs, all AEs with missing or unknown relationship and possible/probable/definite relationship and maximum intensity during first 7 days >0

Solicited (general / local) and unsolicited adverse events: MedDRA coding by SOC, PT, possible/probable/definite relationship to study medication, Safety datase

SOC<br>Preferred Term	Group 1 (MM)<br>(N=183)		Group 2 (MP)<br>(N=181)		Group 3 (PP)<br>(N=181)		Group 4 (M-)<br>(N=200)		Total<br>(N=745)		p-value
	Number of Subjects (%) - Number of events										
Abdominal pain upper	0 (0.0)	0	0 (0.0)	0	2 (1.1)	2	0 (0.0)	0	2 (0.3)	2	0.118
Dysphagia	0 (0.0)	0	0 (0.0)	0	0 (0.0)	0	1 (0.5)	1	1 (0.1)	1	>0.999
Toothache	0 (0.0)	0	0 (0.0)	0	0 (0.0)	0	1 (0.5)	1	1 (0.1)	1	>0.999
Respiratory, thoracic and mediastinal disorders											
Any PT	2 (1.1)	2	2 (1.1)	2	2 (1.1)	2	5 (2.5)	5	11 (1.5)	11	0.686
Pharyngolaryngeal pain	2 (1.1)	2	1 (0.6)	1	1 (0.6)	1	4 (2.0)	4	8 (1.1)	8	0.564
Cough	0 (0.0)	0	0 (0.0)	0	1 (0.6)	1	0 (0.0)	0	1 (0.1)	1	0.486
Dry throat	0 (0.0)	0	0 (0.0)	0	0 (0.0)	0	1 (0.5)	1	1 (0.1)	1	>0.999
Sneezing	0 (0.0)	0	1 (0.6)	1	0 (0.0)	0	0 (0.0)	0	1 (0.1)	1	0.486
Skin and subcutaneous tissue disorders											
Any PT	1 (0.5)	1	5 (2.8)	5	1 (0.6)	1	4 (2.0)	5	11 (1.5)	12	0.251
Rash	0 (0.0)	0	2 (1.1)	2	0 (0.0)	0	1 (0.5)	1	3 (0.4)	3	0.418
Hyperhidrosis	0 (0.0)	0	0 (0.0)	0	0 (0.0)	0	2 (1.0)	3	2 (0.3)	3	0.249
Acne	0 (0.0)	0	0 (0.0)	0	0 (0.0)	0	1 (0.5)	1	1 (0.1)	1	>0.999
Dermatitis	0 (0.0)	0	1 (0.6)	1	0 (0.0)	0	0 (0.0)	0	1 (0.1)	1	0.486
Dermatitis contact	0 (0.0)	0	1 (0.6)	1	0 (0.0)	0	0 (0.0)	0	1 (0.1)	1	0.486
Eczema	0 (0.0)	0	0 (0.0)	0	1 (0.6)	1	0 (0.0)	0	1 (0.1)	1	0.486
Erythema	0 (0.0)	0	1 (0.6)	1	0 (0.0)	0	0 (0.0)	0	1 (0.1)	1	0.486
Rash papular	1 (0.5)	1	0 (0.0)	0	0 (0.0)	0	0 (0.0)	0	1 (0.1)	1	0.732
Musculoskeletal and connective tissue disorders											
Any PT	5 (2.7)	5	1 (0.6)	1	1 (0.6)	1	3 (1.5)	4	10 (1.3)	11	0.292
Muscular weakness	2 (1.1)	2	0 (0.0)	0	1 (0.6)	1	1 (0.5)	1	4 (0.5)	4	0.751

Relationship to study medication: possible/probable/definite for unsolicited AEs
Solicited Local Adverse Events are included if maximum intensity during first 7 days was >0
For Solicited Local Adverse Events 'other' category was excluded
For Solicited General AEs, AEs recorded under the 'other' category have been transcribed to unsolicted AEs during data collection
For Solicited General AEs, all AEs with missing or unknown relationship and possible/probable/definite relationship and maximum intensity during first 7 days >0

Solicited (general / local) and unsolicited adverse events: MedDRA coding by SOC, PT, possible/probable/definite relationship to study medication, Safety datase

SOC<br>Preferred Term	Group 1 (MM)<br>(N=183)		Group 2 (MP)<br>(N=181)		Group 3 (PP)<br>(N=181)		Group 4 (M-)<br>(N=200)		Total<br>(N=745)		p-value
	Number of Subjects (%) - Number of events										
Arthralgia	0 (0.0)	0	0 (0.0)	0	0 (0.0)	0	1 (0.5)	1	1 (0.1)	1	>0.999
Back pain	1 (0.5)	1	0 (0.0)	0	0 (0.0)	0	0 (0.0)	0	1 (0.1)	1	0.732
Muscle spasms	0 (0.0)	0	0 (0.0)	0	0 (0.0)	0	1 (0.5)	2	1 (0.1)	2	>0.999
Neck pain	1 (0.5)	1	0 (0.0)	0	0 (0.0)	0	0 (0.0)	0	1 (0.1)	1	0.732
Pain in extremity	1 (0.5)	1	0 (0.0)	0	0 (0.0)	0	0 (0.0)	0	1 (0.1)	1	0.732
Sensation of heaviness	0 (0.0)	0	1 (0.6)	1	0 (0.0)	0	0 (0.0)	0	1 (0.1)	1	0.486
Blood and lymphatic system disorders											
Any PT	2 (1.1)	4	5 (2.8)	5	0 (0.0)	0	2 (1.0)	2	9 (1.2)	11	0.125
Lymphadenopathy	2 (1.1)	3	4 (2.2)	4	0 (0.0)	0	2 (1.0)	2	8 (1.1)	9	0.250
Lymph node pain	1 (0.5)	1	0 (0.0)	0	0 (0.0)	0	0 (0.0)	0	1 (0.1)	1	0.732
Lymphadenitis	0 (0.0)	0	1 (0.6)	1	0 (0.0)	0	0 (0.0)	0	1 (0.1)	1	0.486
Cardiac disorders											
Any PT	0 (0.0)	0	2 (1.1)	2	1 (0.6)	1	2 (1.0)	3	5 (0.7)	6	0.638
Palpitations	0 (0.0)	0	1 (0.6)	1	0 (0.0)	0	1 (0.5)	2	2 (0.3)	3	0.868
Tachycardia	0 (0.0)	0	1 (0.6)	1	0 (0.0)	0	1 (0.5)	1	2 (0.3)	2	0.868
Pericardial effusion	0 (0.0)	0	0 (0.0)	0	1 (0.6)	1	0 (0.0)	0	1 (0.1)	1	0.486
Vascular disorders											
Any PT	0 (0.0)	0	0 (0.0)	0	0 (0.0)	0	4 (2.0)	4	4 (0.5)	4	0.015
Hot flush	0 (0.0)	0	0 (0.0)	0	0 (0.0)	0	4 (2.0)	4	4 (0.5)	4	0.015
Psychiatric disorders											
Any PT	1 (0.5)	1	1 (0.6)	1	0 (0.0)	0	1 (0.5)	1	3 (0.4)	3	>0.999

Relationship to study medication: possible/probable/definite for unsolicited AEs
Solicited Local Adverse Events are included if maximum intensity during first 7 days was >0
For Solicited Local Adverse Events 'other' category was excluded
For Solicited General AEs, AEs recorded under the 'other' category have been transcribed to unsolicted AEs during data collection
For Solicited General AEs, all AEs with missing or unknown relationship and possible/probable/definite relationship and maximum intensity during first 7 days >0

Solicited (general / local) and unsolicited adverse events: MedDRA coding by SOC, PT, possible/probable/definite relationship to study medication, Safety datase

SOC<br>Preferred Term	Group 1 (MM)<br>(N=183)		Group 2 (MP)<br>(N=181)		Group 3 (PP)<br>(N=181)		Group 4 (M-)<br>(N=200)		Total<br>(N=745)		p-value
Insomnia	0 (0.0)	0	1 (0.6)	1	0 (0.0)	0	0 (0.0)	0	1 (0.1)	1	0.486
Restlessness	0 (0.0)	0	0 (0.0)	0	0 (0.0)	0	1 (0.5)	1	1 (0.1)	1	>0.999
Sleep disorder	1 (0.5)	1	0 (0.0)	0	0 (0.0)	0	0 (0.0)	0	1 (0.1)	1	0.732
Eye disorders											
Any PT	0 (0.0)	0	1 (0.6)	1	0 (0.0)	0	0 (0.0)	0	1 (0.1)	1	0.486
Visual disturbance	0 (0.0)	0	1 (0.6)	1	0 (0.0)	0	0 (0.0)	0	1 (0.1)	1	0.486
Investigations											
Any PT	1 (0.5)	1	0 (0.0)	0	0 (0.0)	0	0 (0.0)	0	1 (0.1)	1	0.732
Lymph node palpable	1 (0.5)	1	0 (0.0)	0	0 (0.0)	0	0 (0.0)	0	1 (0.1)	1	0.732
Metabolism and nutrition disorders											
Any PT	0 (0.0)	0	0 (0.0)	0	1 (0.6)	1	0 (0.0)	0	1 (0.1)	1	0.486
Polydipsia	0 (0.0)	0	0 (0.0)	0	1 (0.6)	1	0 (0.0)	0	1 (0.1)	1	0.486
Reproductive system and breast disorders											
Any PT	0 (0.0)	0	0 (0.0)	0	0 (0.0)	0	1 (0.5)	1	1 (0.1)	1	>0.999
Menstruation irregular	0 (0.0)	0	0 (0.0)	0	0 (0.0)	0	1 (0.5)	1	1 (0.1)	1	>0.999

Number of Subjects (%) - Number of events

Relationship to study medication: possible/probable/definite for unsolicited AEs
Solicited Local Adverse Events are included if maximum intensity during first 7 days was >0
For Solicited Local Adverse Events 'other' category was excluded
For Solicited General AEs, AEs recorded under the 'other' category have been transcribed to unsolicted AEs during data collection
For Solicited General AEs, all AEs with missing or unknown relationship and possible/probable/definite relationship and maximum intensity during first 7 days >0

14.5.5 SAEs, SIAEs and Follow up of Unresolved AEs during Follow up

14.5.5.1 Listings of SAE, SIAE and unresolved AEs

Group=Group 1 (MM)

Subj	Screening number	Event no.	Adverse Event (Diagnosis)	Follow-up to previous event or initial event	Onset relative to most recent vaccination	Onset Date	Onset Time (hh:mm)	End Date	End Time (hh:mm)	Duration	Fre-quency	Serious	Inten-sity	Relation	Action	Outcome
428	570	1	COMMON COLD	follow-up	19.6 days	15OCT2006	6:00	OCT2006	UNKNOWN		1	no	1	unlikely	4	1
495	816	1	TONSILLECTOMY	initial	158 days	19MAR2007	UNKNOWN	25MAR2007	UNKNOWN	6 days	1	SAE	3	none	6	1
521	802	1	SARCOIDOSIS	initial	70 days	26DEC2006	UNKNOWN	Ongoing	CONTINUED		3	SAE	2	possible	4	3

Frequency: 1=Single episode, 2=Intermittent, 3=Continuous, -1=Unknown
Intensity: 1=mild, 2=moderate, 3=severe, 4=life threatening/diasabling, -1=Unknown
Action: 1=no action, 2=study drug dosage adjusted, 3=study drug discontinued, 4=concomitant medication, 5=non-drug therapy, 6=hospitalization, -1=Unknown
Outcome: 1=recovered without sequelae, 2=recovered with sequelae, 3=ongoing, 4=death, -1=unknown

Listings of SAE, SIAE and unresolved AEs

Group=Group 2 (MP)

Subj	Screening number	Event no.	Adverse Event (Diagnosis)	Follow-up to previous event or initial event	Onset relative to most recent vaccination	Onset Date	Onset Time (hh:mm)	End Date	End Time (hh:mm)	Duration	Fre-quency	Serious	Inten-sity	Relation	Action	Outcome
486	803	1	PREGNANCY	initial	118 days	08FEB2007	UNKNOWN		UNKNOWN		3	SAE	1	none	-1	-1
710	1118	1	PALPITATIONS	initial	193 days	23JUN2007	EVENING	Ongoing	ONGOING		2	SIAE	1	unlikely	1	3

Frequency: 1=Single episode, 2=Intermittent, 3=Continuous, -1=Unknown

Intensity: 1=mild, 2=moderate, 3=severe, 4=life threatening/diasabling, -1=Unknown

Action: 1=no action, 2=study drug dosage adjusted, 3=study drug discontinued, 4=concomitant medication, 5=non-drug therapy, 6=hospitalization, -1=Unknown

Outcome: 1=recovered without sequelae, 2=recovered with sequelae, 3=ongoing, 4=death, -1=unknown

Listings of SAE, SIAE and unresolved AEs

Group=Group 3 (PP)

Subj	Screening number	Event no.	Adverse Event (Diagnosis)	Follow-up to previous event or initial event	Onset relative to most recent vaccination	Onset Date	Onset Time (hh:mm)	End Date	End Time (hh:mm)	Duration	Fre-quency	Serious	Inten-sity	Relation	Action	Outcome
067	155	1	SALMONELLA ENTERITIS	initial	199 days	22JAN2007	UNKNOWN	06FEB2007	UNKNOWN	15 days	1	SAE	3	none	6	1
		2	IRON DEFICIENCY	unknown	18 days	25JUL2006	UNKNOWN	Ongoing	CONTINU ED		3	no	1	none	4	3
444	714	1	THYROIDECTOMY (BECAUSE OF BENIGN LUMP AND CYST)	initial	85 days	02JAN2007	UNKNOWN	04JAN2007	UNKNOWN	2 days	1	SAE	3	none	6	2
594	1009	1	FRACTURE OF RIGHT TIBIA AND FIBULA	initial	94 days	17FEB2007	UNKNOWN	04MAR2007	UNKNOWN	15 days	1	SAE	3	none	6	2
685	1001	1	COLON-CARCINOMA	initial	74.5 days	12FEB2007	8:00	19FEB2007	8:00	7.0 days	1	SAE	4	unlikely	6	2
751	1084	1	DEPRESSION	initial	66 days	15FEB2007	UNKNOWN	15JUN2007	UNKNOWN	120 days	1	SAE	3	unlikely	6	1

Frequency: 1=Single episode, 2=Intermittent, 3=Continuous, -1=Unknown

Intensity: 1=mild, 2=moderate, 3=severe, 4=life threatening/diasabling, -1=Unknown

Action: 1=no action, 2=study drug dosage adjusted, 3=study drug discontinued, 4=concomitant medication, 5=non-drug therapy, 6=hospitalization, -1=Unknown

Outcome: 1=recovered without sequelae, 2=recovered with sequelae, 3=ongoing, 4=death, -1=unknown

Listings of SAE, SIAE and unresolved AEs

Group=Group 4 (M-)

Subj	Screening number	Event no.	Adverse Event (Diagnosis)	Follow-up to previous event or initial event	Onset relative to most recent vaccination	Onset Date	Onset Time (hh:mm)	End Date	End Time (hh:mm)	Duration	Frequency	Serious	Intensity	Relation	Action	Outcome
024	67	1	PALPITATIONS	initial		AUG2006	UNKNOWN	OCT2006	UNKNOWN		2	SIAE	1	unlikely	1	1
028	85	1	PALPITATIONS	initial	92.3 days	23AUG2006	22:00	23AUG2006	23:30	1.5 hrs	1	SIAE	2	unlikely	1	1
119	256	1	RUPTURE OF THE LEFT TENDON OF THE PERONEUS 5.11.2006; OP 8.12.2006	initial	128 days	05NOV2006	UNKNOWN	18JAN2007	UNKNOWN	74 days	1	SAE	3	none	6	1
246	479	1	TACHYCARDIA	initial	182 days	18JAN2007	MORNING	01FEB2007	UNKNOWN	14 days	-1	SIAE	1	unlikely	1	1
411	683	1	MILD PERICARDIAL EFFUSION	initial	196 days	07MAR2007	UNK	Ongoing	ONGOING		3	SIAE	1	unlikely	1	3

Frequency: 1=Single episode, 2=Intermittent, 3=Continuous, -1=Unknown

Intensity: 1=mild, 2=moderate, 3=severe, 4=life threatening/diasabling, -1=Unknown

Action: 1=no action, 2=study drug dosage adjusted, 3=study drug discontinued, 4=concomitant medication, 5=non-drug therapy, 6=hospitalization, -1=Unknown

Outcome: 1=recovered without sequelae, 2=recovered with sequelae, 3=ongoing, 4=death, -1=unknown

14.5.5.2 Unsolicted Adverse Events: Overview, Safety dataset

	Group									
	Group 1 (MM)		Group 2 (MP)		Group 3 (PP)		Group 4 (M-)		All subjects	
	Count	%	Count	%	Count	%	Count	%	Count	%
Total no. of subjects included in FU	178	24.5	174	24.0	175	24.1	199	27.4	726	100.0
No. of subjects with at least one SAE, SIAE, update of AE	3	1.7	2	1.1	5	2.9	5	2.5	15	2.1
No. of subjects with at least one drug-related[1] SAE, SIAE, update of AE	1	0.6	-	-	-	-	-	-	1	0.1
No. of subjects with at least one update of AE	1	0.6	-	-	1	0.6	-	-	2	0.3
No. of subjects with at least one SAE	2	1.1	1	0.6	5	2.9	1	0.5	9	1.2
No. of subjects with at least one related SAE	1	0.6	-	-	-	-	-	-	1	0.1
No. of subjects with at least one SIAE	-	-	1	0.6	-	-	4	2.0	5	0.7
No. of subjects with at least one related SIAE	-	-	-	-	-	-	-	-	-	-

(Continued)

Percentages for 'No. of subjects with...' are based on the total number of subjects included per treatment

[1]Drug related: definite, probable, possible or unknown
SIAE: special interest adverse events
[2]: This includes all coded symptoms including multiple occurrences of the same PT within one subject
Splitting of verbatim reported AEs possible.

[3]: A subject was only included once in each PT
(i.e. this represents the number of subjects with at least one event for the same PT)

Unsolicted Adverse Events: Overview, Safety dataset

	Group									
	Group 1 (MM)		Group 2 (MP)		Group 3 (PP)		Group 4 (M-)		All subjects	
	Count	%	Count	%	Count	%	Count	%	Count	%
No. of verbatim reported SAE, SIAE, update of AE	3	18.8	2	12.5	6	37.5	5	31.3	16	100.0
No. of reported updates of AE	1	50.0	-	-	1	50.0	-	-	2	100.0
No. of reported SAEs	2	22.2	1	11.1	5	55.6	1	11.1	9	100.0
No. of reported SIAEs	-	-	1	20.0	-	-	4	80.0	5	100.0
No. of coded MedDRA PTs[2]	3	16.7	2	11.1	8	44.4	5	27.8	18	100.0
No. of PTs by subject[3]	3	16.7	2	11.1	8	44.4	5	27.8	18	100.0

Percentages for 'No. of subjects with...' are based on the total number of subjects included per treatment

[1]Drug related: definite, probable, possible or unknown
SIAE: special interest adverse events
[2]: This includes all coded symptoms including multiple occurrences of the same PT within one subject
Splitting of verbatim reported AEs possible.

[3]: A subject was only included once in each PT
(i.e. this represents the number of subjects with at least one event for the same PT)

14.5.5.3 Unsolicited adverse events: MedDRA coding by SOC, PT and subject, Safety dataset

SOC<br>Preferred Term	Group 1 (MM)<br>(N=178)		Group 2 (MP)<br>(N=174)		Group 3 (PP)<br>(N=175)		Group 4 (M-)<br>(N=199)		Total<br>(N=726)	
	Number of Subjects (%) - Number of events									
Cardiac disorders										
Any PT	0 (0.0)	0	1 (0.6)	1	0 (0.0)	0	4 (2.0)	4	5 (0.7)	5
Palpitations	0 (0.0)	0	1 (0.6)	1	0 (0.0)	0	2 (1.0)	2	3 (0.4)	3
Pericardial effusion	0 (0.0)	0	0 (0.0)	0	0 (0.0)	0	1 (0.5)	1	1 (0.1)	1
Tachycardia	0 (0.0)	0	0 (0.0)	0	0 (0.0)	0	1 (0.5)	1	1 (0.1)	1
Infections and infestations										
Any PT	1 (0.6)	1	0 (0.0)	0	1 (0.6)	1	0 (0.0)	0	2 (0.3)	2
Gastroenteritis salmonella	0 (0.0)	0	0 (0.0)	0	1 (0.6)	1	0 (0.0)	0	1 (0.1)	1
Nasopharyngitis	1 (0.6)	1	0 (0.0)	0	0 (0.0)	0	0 (0.0)	0	1 (0.1)	1
Injury, poisoning and procedural complications										
Any PT	0 (0.0)	0	0 (0.0)	0	1 (0.6)	1	1 (0.5)	1	2 (0.3)	2
Lower limb fracture	0 (0.0)	0	0 (0.0)	0	1 (0.6)	1	0 (0.0)	0	1 (0.1)	1
Tendon rupture	0 (0.0)	0	0 (0.0)	0	0 (0.0)	0	1 (0.5)	1	1 (0.1)	1
Neoplasms benign, malignant and unspecified (incl cysts and polyps)										
Any PT	0 (0.0)	0	0 (0.0)	0	2 (1.1)	2	0 (0.0)	0	2 (0.3)	2
Benign neoplasm of thyroid gland	0 (0.0)	0	0 (0.0)	0	1 (0.6)	1	0 (0.0)	0	1 (0.1)	1
Colon cancer	0 (0.0)	0	0 (0.0)	0	1 (0.6)	1	0 (0.0)	0	1 (0.1)	1
Surgical and medical procedures										
Any PT	1 (0.6)	1	0 (0.0)	0	1 (0.6)	1	0 (0.0)	0	2 (0.3)	2
Thyroidectomy	0 (0.0)	0	0 (0.0)	0	1 (0.6)	1	0 (0.0)	0	1 (0.1)	1
Tonsillectomy	1 (0.6)	1	0 (0.0)	0	0 (0.0)	0	0 (0.0)	0	1 (0.1)	1
Endocrine disorders										
Any PT	0 (0.0)	0	0 (0.0)	0	1 (0.6)	1	0 (0.0)	0	1 (0.1)	1

A subject with more than one finding in a specific category was only counted once
Percentages based on total no. of subjects in each group
Table is sorted by descending subject count for the total of all treatment groups

Unsolicited adverse events: MedDRA coding by SOC, PT and subject, Safety dataset

SOC<br>Preferred Term	Group 1 (MM)<br>(N=178)		Group 2 (MP)<br>(N=174)		Group 3 (PP)<br>(N=175)		Group 4 (M-)<br>(N=199)		Total<br>(N=726)	
	Number of Subjects (%) - Number of events									
Thyroid cyst	0 (0.0)	0	0 (0.0)	0	1 (0.6)	1	0 (0.0)	0	1 (0.1)	1
Immune system disorders										
Any PT	1 (0.6)	1	0 (0.0)	0	0 (0.0)	0	0 (0.0)	0	1 (0.1)	1
Sarcoidosis	1 (0.6)	1	0 (0.0)	0	0 (0.0)	0	0 (0.0)	0	1 (0.1)	1
Metabolism and nutrition disorders										
Any PT	0 (0.0)	0	0 (0.0)	0	1 (0.6)	1	0 (0.0)	0	1 (0.1)	1
Iron deficiency	0 (0.0)	0	0 (0.0)	0	1 (0.6)	1	0 (0.0)	0	1 (0.1)	1
Pregnancy, puerperium and perinatal conditions										
Any PT	0 (0.0)	0	1 (0.6)	1	0 (0.0)	0	0 (0.0)	0	1 (0.1)	1
Pregnancy	0 (0.0)	0	1 (0.6)	1	0 (0.0)	0	0 (0.0)	0	1 (0.1)	1
Psychiatric disorders										
Any PT	0 (0.0)	0	0 (0.0)	0	1 (0.6)	1	0 (0.0)	0	1 (0.1)	1
Depression	0 (0.0)	0	0 (0.0)	0	1 (0.6)	1	0 (0.0)	0	1 (0.1)	1

A subject with more than one finding in a specific category was only counted once
Percentages based on total no. of subjects in each group
Table is sorted by descending subject count for the total of all treatment groups

14.5.5.4 Unsolicited adverse events: MedDRA coding by SOC and subject, Safety dataset

SOC	Group 1 (MM) (N=178)		Group 2 (MP) (N=174)		Group 3 (PP) (N=175)		Group 4 (M-) (N=199)		Total (N=726)	
	Number of Subjects (%) - Number of events									
Any AE	3 (1.7)	3	2 (1.1)	2	5 (2.9)	8	5 (2.5)	5	15 (2.1)	18
Cardiac disorders	0 (0.0)	0	1 (0.6)	1	0 (0.0)	0	4 (2.0)	4	5 (0.7)	5
Infections and infestations	1 (0.6)	1	0 (0.0)	0	1 (0.6)	1	0 (0.0)	0	2 (0.3)	2
Injury, poisoning and procedural complications	0 (0.0)	0	0 (0.0)	0	1 (0.6)	1	1 (0.5)	1	2 (0.3)	2
Neoplasms benign, malignant and unspecified (incl cysts and polyps)	0 (0.0)	0	0 (0.0)	0	2 (1.1)	2	0 (0.0)	0	2 (0.3)	2
Surgical and medical procedures	1 (0.6)	1	0 (0.0)	0	1 (0.6)	1	0 (0.0)	0	2 (0.3)	2
Endocrine disorders	0 (0.0)	0	0 (0.0)	0	1 (0.6)	1	0 (0.0)	0	1 (0.1)	1
Immune system disorders	1 (0.6)	1	0 (0.0)	0	0 (0.0)	0	0 (0.0)	0	1 (0.1)	1
Metabolism and nutrition disorders	0 (0.0)	0	0 (0.0)	0	1 (0.6)	1	0 (0.0)	0	1 (0.1)	1
Pregnancy, puerperium and perinatal conditions	0 (0.0)	0	1 (0.6)	1	0 (0.0)	0	0 (0.0)	0	1 (0.1)	1
Psychiatric disorders	0 (0.0)	0	0 (0.0)	0	1 (0.6)	1	0 (0.0)	0	1 (0.1)	1

A subject with more than one finding in a specific category was only counted once
Percentages based on total no. of subjects in each group
Table is sorted by descending subject count for the total of all treatment groups

14.5.5.5 Unsolicited adverse events: MedDRA coding by PT and subject, Safety dataset

	Number of Subjects (%) - Number of events				
Preferred Term	Group 1 (MM) (N=178)	Group 2 (MP) (N=174)	Group 3 (PP) (N=175)	Group 4 (M-) (N=199)	Total (N=726)
Any AE	3 (1.7) 3	2 (1.1) 2	5 (2.9) 8	5 (2.5) 5	15 (2.1) 18
Palpitations	0 (0.0) 0	1 (0.6) 1	0 (0.0) 0	2 (1.0) 2	3 (0.4) 3
Benign neoplasm of thyroid gland	0 (0.0) 0	0 (0.0) 0	1 (0.6) 1	0 (0.0) 0	1 (0.1) 1
Colon cancer	0 (0.0) 0	0 (0.0) 0	1 (0.6) 1	0 (0.0) 0	1 (0.1) 1
Depression	0 (0.0) 0	0 (0.0) 0	1 (0.6) 1	0 (0.0) 0	1 (0.1) 1
Gastroenteritis salmonella	0 (0.0) 0	0 (0.0) 0	1 (0.6) 1	0 (0.0) 0	1 (0.1) 1
Iron deficiency	0 (0.0) 0	0 (0.0) 0	1 (0.6) 1	0 (0.0) 0	1 (0.1) 1
Lower limb fracture	0 (0.0) 0	0 (0.0) 0	1 (0.6) 1	0 (0.0) 0	1 (0.1) 1
Nasopharyngitis	1 (0.6) 1	0 (0.0) 0	0 (0.0) 0	0 (0.0) 0	1 (0.1) 1
Pericardial effusion	0 (0.0) 0	0 (0.0) 0	0 (0.0) 0	1 (0.5) 1	1 (0.1) 1
Pregnancy	0 (0.0) 0	1 (0.6) 1	0 (0.0) 0	0 (0.0) 0	1 (0.1) 1
Sarcoidosis	1 (0.6) 1	0 (0.0) 0	0 (0.0) 0	0 (0.0) 0	1 (0.1) 1
Tachycardia	0 (0.0) 0	0 (0.0) 0	0 (0.0) 0	1 (0.5) 1	1 (0.1) 1
Tendon rupture	0 (0.0) 0	0 (0.0) 0	0 (0.0) 0	1 (0.5) 1	1 (0.1) 1
Thyroid cyst	0 (0.0) 0	0 (0.0) 0	1 (0.6) 1	0 (0.0) 0	1 (0.1) 1
Thyroidectomy	0 (0.0) 0	0 (0.0) 0	1 (0.6) 1	0 (0.0) 0	1 (0.1) 1
Tonsillectomy	1 (0.6) 1	0 (0.0) 0	0 (0.0) 0	0 (0.0) 0	1 (0.1) 1

A subject with more than one finding in a specific category was only counted once
Percentages based on total no. of subjects in each group
Table is sorted by descending subject count for the total of all treatment groups

14.6 FURTHER SAFETY DATA

14.6.1 Reason for study termination, Safety (FA) dataset

Reason for study termination	Group								Total	
	Group 1 (MM)		Group 2 (MP)		Group 3 (PP)		Group 4 (M-)			
	n	%	n	%	n	%	n	%	n	%
-	-	0.0	-	0.0	-	0.0	-	0.0	-	0.0
the volunteer reached the planned observation time	175	95.6	173	95.6	175	96.7	200	100.0	723	97.0
adverse event, making it unsafe for the subject to continue	-	0.0	1	0.6	1	0.6	-	0.0	2	0.3
withdrawal of consent	3	1.6	6	3.3	2	1.1	-	0.0	11	1.5
subject unwilling/unable to comply with study requirements	5	2.7	1	0.6	2	1.1	-	0.0	8	1.1
clinical need for concomitant/ancillary therapy not permitted	-	0.0	-	0.0	-	0.0	-	0.0	-	0.0
unrelated intercurrent illness, affecting significantly assessment of clinical status	-	0.0	-	0.0	-	0.0	-	0.0	-	0.0
lost to follow-up	-	0.0	-	0.0	1	0.6	-	0.0	1	0.1
other	-	0.0	-	0.0	-	0.0	-	0.0	-	0.0
Total	183	100.0	181	100.0	181	100.0	200	100.0	745	100.0

-: Missing

Reason for study termination, PP dataset

Reason for study termination	Group								Total	
	Group 1 (MM)		Group 2 (MP)		Group 3 (PP)		Group 4 (M-)			
	n	%	n	%	n	%	n	%	n	%
-	-	0.0	-	0.0	-	0.0	-	0.0	-	0.0
the volunteer reached the planned observation time	168	100.0	170	100.0	164	100.0	193	100.0	695	100.0
adverse event, making it unsafe for the subject to continue	-	0.0	-	0.0	-	0.0	-	0.0	-	0.0
withdrawal of consent	-	0.0	-	0.0	-	0.0	-	0.0	-	0.0
subject unwilling/unable to comply with study requirements	-	0.0	-	0.0	-	0.0	-	0.0	-	0.0
clinical need for concomitant/ancillary therapy not permitted	-	0.0	-	0.0	-	0.0	-	0.0	-	0.0
unrelated intercurrent illness, affecting significantly assessment of clinical status	-	0.0	-	0.0	-	0.0	-	0.0	-	0.0
lost to follow-up	-	0.0	-	0.0	-	0.0	-	0.0	-	0.0
other	-	0.0	-	0.0	-	0.0	-	0.0	-	0.0
Total	168	100.0	170	100.0	164	100.0	193	100.0	695	100.0

-: Missing

14.6.2 Vital signs

14.6.2.1 Vital signs - systolic blood pressure, Safety dataset

Systolic blood pressure [mmHg]		Screening	Visit 1	Visit 2	Visit 3	Visit 4	Visit 5	Follow-Up
Group 1 (MM)								
	Mean	122.4	123.3	123.3	123.7	123.0	124.6	124.5
	Median	122.0	124.0	123.0	124.0	123.5	124.5	124.0
	StdDev	11.8	10.8	12.0	11.9	11.7	11.5	12.9
	SEM	0.9	0.8	0.9	0.9	0.9	0.9	1.0
	Min	87	94	88	91	99	92	92
	Max	152	152	162	160	153	154	156
	N	183	183	182	180	176	178	178
Group 2 (MP)								
	Mean	121.3	121.5	121.9	122.2	122.5	123.2	123.1
	Median	122.0	120.0	122.0	122.0	122.0	124.0	124.0
	StdDev	12.7	11.7	12.5	12.0	11.5	11.2	13.3
	SEM	0.9	0.9	0.9	0.9	0.9	0.8	1.0
	Min	89	87	85	93	94	96	89
	Max	158	148	149	158	157	151	161
	N	181	181	179	174	174	175	174
Group 3 (PP)								
	Mean	122.3	122.6	123.2	123.2	124.1	123.3	125.1
	Median	122.0	123.0	122.0	123.0	126.0	123.0	124.0
	StdDev	12.4	12.4	12.8	11.8	12.0	12.6	13.0
	SEM	0.9	0.9	1.0	0.9	0.9	0.9	1.0
	Min	93	89	97	95	85	96	92
	Max	163	152	163	152	166	160	156
	N	181	181	179	175	175	177	175
Group 4 (M-)								
	Mean	121.9	123.8	122.0	122.1	.	.	127.3
	Median	121.0	124.0	121.0	122.0	.	.	128.0
	StdDev	14.1	14.8	13.6	13.9	.	.	15.2
	SEM	1.0	1.0	1.0	1.0	.	.	1.1
	Min	87	88	96	90	.	.	94
	Max	166	200	179	181	.	.	188
	N	200	200	200	200	.	.	199

14.6.2.2 Vital signs - diastolic blood pressure, Safety dataset

Diastolic blood pressure [mmHg]		Screening	Visit 1	Visit 2	Visit 3	Visit 4	Visit 5	Follow-Up
Group 1 (MM)								
	Mean	75.5	73.6	73.0	73.5	73.0	73.6	72.1
	Median	76.0	75.0	73.0	73.0	72.5	73.5	72.0
	StdDev	8.8	9.3	8.6	8.8	8.5	8.9	9.0
	SEM	0.6	0.7	0.6	0.7	0.6	0.7	0.7
	Min	53	44	44	54	56	51	48
	Max	100	100	99	104	93	96	92
	N	183	183	182	180	176	178	178
Group 2 (MP)								
	Mean	74.9	72.4	73.8	72.5	72.9	73.1	72.0
	Median	75.0	72.0	74.0	72.0	72.5	72.0	72.0
	StdDev	8.6	8.9	9.3	8.3	7.9	7.9	8.8
	SEM	0.6	0.7	0.7	0.6	0.6	0.6	0.7
	Min	52	50	40	50	52	56	52
	Max	96	99	102	93	94	97	96
	N	181	181	179	174	174	175	174
Group 3 (PP)								
	Mean	76.6	74.2	73.3	73.5	73.8	72.9	74.2
	Median	77.0	73.0	73.0	73.0	73.0	72.0	74.0
	StdDev	9.2	9.3	8.7	8.8	9.6	8.6	9.1
	SEM	0.7	0.7	0.6	0.7	0.7	0.6	0.7
	Min	49	50	50	51	44	54	52
	Max	100	97	95	99	109	101	98
	N	181	181	179	175	175	177	175
Group 4 (M-)								
	Mean	80.0	78.0	77.6	77.5	.	.	79.0
	Median	80.0	77.0	77.5	76.0	.	.	79.0
	StdDev	9.3	9.4	9.4	9.3	.	.	9.9
	SEM	0.7	0.7	0.7	0.7	.	.	0.7
	Min	55	56	56	54	.	.	58
	Max	109	112	105	113	.	.	109
	N	200	200	200	200	.	.	199

14.6.2.3 Vital signs - pulse rate, Safety dataset

Pulse rate [bpm]		Screening	Visit 1	Visit 2	Visit 3	Visit 4	Visit 5	Follow-Up
Group 1 (MM)								
	Mean	67.8	75.6	74.0	74.2	72.6	73.8	73.8
	Median	67.0	76.0	73.0	74.0	73.0	73.0	73.5
	StdDev	11.6	12.0	12.8	11.3	11.4	12.1	11.5
	SEM	0.9	0.9	0.9	0.8	0.9	0.9	0.9
	Min	45	48	49	48	47	45	43
	Max	100	105	113	98	109	116	99
	N	183	183	182	180	176	178	178
Group 2 (MP)								
	Mean	68.3	74.2	74.3	74.8	74.3	75.2	74.1
	Median	67.0	73.0	73.0	74.0	74.0	73.0	74.0
	StdDev	10.9	11.6	12.1	11.0	11.2	11.4	11.9
	SEM	0.8	0.9	0.9	0.8	0.9	0.9	0.9
	Min	44	50	50	49	49	54	49
	Max	99	108	107	100	119	105	99
	N	181	181	179	174	174	175	174
Group 3 (PP)								
	Mean	68.9	74.7	72.6	74.5	73.2	73.9	74.9
	Median	69.0	74.0	71.0	74.0	73.0	73.0	75.0
	StdDev	10.2	11.4	11.6	10.7	11.5	11.5	11.7
	SEM	0.8	0.8	0.9	0.8	0.9	0.9	0.9
	Min	44	46	49	49	46	50	46
	Max	95	103	98	99	98	100	99
	N	181	181	179	175	175	177	175
Group 4 (M-)								
	Mean	69.4	75.8	74.3	74.7	.	.	75.4
	Median	69.0	74.5	73.0	75.0	.	.	74.0
	StdDev	9.9	10.6	10.8	10.9	.	.	11.4
	SEM	0.7	0.7	0.8	0.8	.	.	0.8
	Min	43	52	50	52	.	.	51
	Max	98	106	107	103	.	.	105
	N	200	200	200	200	.	.	199

14.6.2.4 Vital signs - oral temperature, Safety dataset

Oral temperature [˚C]		Screening	Visit 1	Visit 2	Visit 3	Visit 4	Visit 5	Follow-Up
Group 1 (MM)								
	Mean	36.32	36.56	36.44	36.48	36.41	36.37	36.39
	Median	36.30	36.60	36.40	36.50	36.40	36.30	36.30
	StdDev	0.30	0.37	0.35	0.34	0.33	0.33	0.33
	SEM	0.02	0.03	0.03	0.03	0.02	0.02	0.02
	Min	35.6	35.8	35.4	35.3	35.8	35.6	36.0
	Max	37.2	37.4	37.3	37.2	37.4	37.6	37.4
	N	183	183	182	179	176	178	178
Group 2 (MP)								
	Mean	36.33	36.57	36.51	36.48	36.46	36.42	36.46
	Median	36.30	36.60	36.50	36.40	36.45	36.40	36.50
	StdDev	0.27	0.36	0.36	0.36	0.33	0.35	0.36
	SEM	0.02	0.03	0.03	0.03	0.02	0.03	0.03
	Min	35.6	34.8	35.3	35.9	35.4	35.5	35.9
	Max	37.1	37.3	37.4	37.4	37.3	37.2	37.4
	N	181	181	179	174	174	175	174
Group 3 (PP)								
	Mean	36.38	36.54	36.56	36.50	36.45	36.42	36.48
	Median	36.40	36.60	36.60	36.50	36.40	36.40	36.50
	StdDev	0.33	0.36	0.34	0.32	0.33	0.33	0.36
	SEM	0.02	0.03	0.03	0.02	0.02	0.03	0.03
	Min	35.2	35.0	35.7	35.9	35.5	36.0	35.9
	Max	37.6	37.3	37.3	37.3	37.3	37.4	37.7
	N	181	181	179	175	175	177	175
Group 4 (M-)								
	Mean	36.34	36.52	36.53	36.50	.	.	36.34
	Median	36.30	36.50	36.60	36.50	.	.	36.30
	StdDev	0.29	0.36	0.34	0.34	.	.	0.31
	SEM	0.02	0.03	0.02	0.02	.	.	0.02
	Min	35.6	35.6	35.8	35.6	.	.	35.8
	Max	37.1	37.3	37.3	37.4	.	.	37.2
	N	200	200	200	200	.	.	199

14.6.3 Body weight, Safety dataset

Body weight [kg]		Screening	Visit 1	Visit 2	Visit 3	Visit 4	Visit 5	Follow-Up
Group 1 (MM)								
	Mean	72.08	72.23	72.23	72.19	72.51	72.59	72.57
	Median	70.30	70.40	70.45	70.60	70.00	70.40	69.35
	StdDev	14.53	14.51	14.57	14.54	14.81	14.83	15.34
	SEM	1.07	1.07	1.08	1.09	1.12	1.11	1.15
	Min	45.1	46.6	45.1	45.3	45.4	45.5	45.7
	Max	122.6	122.4	123.3	123.3	124.1	122.2	129.5
	N	183	183	182	179	176	178	178
Group 2 (MP)								
	Mean	70.81	71.20	70.81	71.11	71.11	71.26	71.55
	Median	69.40	69.50	69.10	69.85	69.95	70.00	69.20
	StdDev	12.85	12.81	12.52	12.56	12.65	12.79	13.32
	SEM	0.96	0.95	0.94	0.95	0.96	0.97	1.01
	Min	47.4	47.6	48.0	48.0	48.4	48.5	47.3
	Max	114.7	114.5	114.5	112.4	112.7	114.2	109.4
	N	181	181	179	174	174	175	174
Group 3 (PP)								
	Mean	72.21	72.41	72.59	72.96	72.93	72.93	73.04
	Median	69.10	69.40	69.40	69.80	70.30	69.40	70.80
	StdDev	14.80	14.86	14.88	14.99	15.05	14.92	15.08
	SEM	1.10	1.10	1.11	1.13	1.14	1.12	1.14
	Min	47.3	46.3	47.7	47.2	47.6	46.8	46.8
	Max	136.0	135.5	135.6	133.8	134.4	133.4	128.3
	N	181	181	179	175	175	177	175
Group 4 (M-)								
	Mean	73.18	73.44	73.36	73.43	.	.	74.17
	Median	70.60	71.00	70.50	70.60	.	.	71.80
	StdDev	14.80	14.92	15.00	14.95	.	.	15.58
	SEM	1.05	1.06	1.06	1.06	.	.	1.10
	Min	46.1	46.6	45.0	45.8	.	.	47.8
	Max	135.4	136.0	138.0	135.9	.	.	139.2
	N	200	200	200	200	.	.	199

14.6.4 Total study duration, time between visits, Safety dataset

Time between visits [days]		Group 1 (MM) (N=183)	Group 2 (MP) (N=181)	Group 3 (PP) (N=181)	Group 4 (M-) (N=200)	Total
Days between V1 and Screening						
	Mean	-16.4	-17.2	-16.7	-18.0	-17.1
	Median	-15.0	-17.0	-15.0	-17.0	-16.0
	StdDev	6.5	7.3	7.0	6.5	6.8
	SEM	0.5	0.5	0.5	0.5	0.3
	Min	-33	-35	-33	-31	-35
	Max	-4	-3	-1	-4	-1
	N	183	181	181	200	745
Days between V2 and V1						
	Mean	13.3	13.2	13.5	13.6	13.4
	Median	14.0	14.0	14.0	14.0	14.0
	StdDev	1.8	1.6	2.4	1.5	1.9
	SEM	0.1	0.1	0.2	0.1	0.1
	Min	10	9	7	9	7
	Max	22	18	31	18	31
	N	182	179	179	200	740
Days between V3 and V1						
	Mean	30.3	30.1	30.4	29.3	30.0
	Median	29.0	28.0	29.0	28.0	28.0
	StdDev	2.9	2.8	3.1	2.7	2.9
	SEM	0.2	0.2	0.2	0.2	0.1
	Min	26	28	28	24	24
	Max	44	43	42	52	52
	N	180	174	175	200	729
Days between V4 and V3						
	Mean	13.3	13.3	13.4	.	13.3
	Median	14.0	14.0	14.0	.	14.0
	StdDev	2.5	1.7	2.1	.	2.1
	SEM	0.2	0.1	0.2	.	0.1
	Min	9	10	9	.	9

(Continued)

Total study duration, time between visits, Safety dataset

Time between visits [days]		Group 1 (MM) (N=183)	Group 2 (MP) (N=181)	Group 3 (PP) (N=181)	Group 4 (M-) (N=200)	Total
Days between V4 and V3						
	Max	34	22	28	.	34
	N	176	174	175	0	525
Days between V5 and V3						
	Mean	29.9	30.7	30.6	.	30.4
	Median	28.0	28.0	29.0	.	28.0
	StdDev	2.9	4.5	3.7	.	3.7
	SEM	0.2	0.3	0.3	.	0.2
	Min	27	26	25	.	25
	Max	48	62	48	.	62
	N	177	173	175	0	525
Days between Follow up and V1(Group 4)/ V3(Group 1-3)						
	Mean	191.8	192.5	192.5	192.5	192.3
	Median	189.0	190.0	189.0	190.0	189.0
	StdDev	19.1	12.7	16.7	8.6	14.6
	SEM	1.4	1.0	1.3	0.6	0.5
	Min	111	153	132	176	111
	Max	298	252	312	229	312
	N	177	171	173	199	720
Duration of main study [days]						
	Mean	75.6	76.5	76.5	47.3	68.4
	Median	76.0	77.0	77.0	47.0	72.0
	StdDev	10.3	12.8	11.5	7.3	16.6
	SEM	0.8	0.9	0.9	0.5	0.6
	Min	13	15	10	32	10
	Max	98	104	103	66	104
	N	183	181	181	200	745

(Continued)

Total study duration, time between visits, Safety dataset

Time between visits [days]		Group 1 (MM) (N=183)	Group 2 (MP) (N=181)	Group 3 (PP) (N=181)	Group 4 (M-) (N=200)	Total
Duration of study [days] - Visit 1 to Follow up						
	Mean	233.4	232.7	233.5	209.7	226.9
	Median	235.0	237.0	237.0	209.5	231.0
	StdDev	37.5	40.1	39.3	15.1	35.7
	SEM	2.8	3.0	2.9	1.1	1.3
	Min	13	15	10	56	10
	Max	348	309	346	257	348
	N	183	181	181	200	745

14.6.5 Prior and concomitant medication by brand name, Safety dataset

Brand name	Group 1 (MM) (N=183)	Group 2 (MP) (N=181)	Group 3 (PP) (N=181)	Group 4 (M-) (N=200)	Total (N=745)
Any concomitant medication taken?	130 (71.0)	126 (69.6)	123 (68.0)	101 (50.5)	480 (64.4)
PARACETAMOL	23 (12.6)	19 (10.5)	20 (11.0)	11 (5.5)	73 (9.8)
ASPIRIN	22 (12.0)	9 (5.0)	11 (6.1)	11 (5.5)	53 (7.1)
IBUPROFEN	10 (5.5)	12 (6.6)	14 (7.7)	8 (4.0)	44 (5.9)
VALETTE	15 (8.2)	11 (6.1)	8 (4.4)	3 (1.5)	37 (5.0)
BELARA	4 (2.2)	7 (3.9)	6 (3.3)	1 (0.5)	18 (2.4)
ASS	5 (2.7)	6 (3.3)	5 (2.8)	1 (0.5)	17 (2.3)
MICROGYNON	3 (1.6)	5 (2.8)	3 (1.7)	3 (1.5)	14 (1.9)
PETIBELLE	0 (0.0)	6 (3.3)	4 (2.2)	2 (1.0)	12 (1.6)
MONOSTEP	5 (2.7)	4 (2.2)	2 (1.1)	0 (0.0)	11 (1.5)
DICLOFENAC	2 (1.1)	3 (1.7)	5 (2.8)	0 (0.0)	10 (1.3)
YASMIN	3 (1.6)	3 (1.7)	2 (1.1)	2 (1.0)	10 (1.3)
ACC	3 (1.6)	3 (1.7)	2 (1.1)	1 (0.5)	9 (1.2)
DIANE 35	1 (0.5)	1 (0.6)	5 (2.8)	1 (0.5)	8 (1.1)
IMPLANON	4 (2.2)	0 (0.0)	2 (1.1)	2 (1.0)	8 (1.1)
JODID	2 (1.1)	2 (1.1)	2 (1.1)	2 (1.0)	8 (1.1)
MIRANOVA	3 (1.6)	3 (1.7)	1 (0.6)	1 (0.5)	8 (1.1)
NUVA RING	3 (1.6)	2 (1.1)	3 (1.7)	0 (0.0)	8 (1.1)
ACC 600	1 (0.5)	1 (0.6)	5 (2.8)	0 (0.0)	7 (0.9)
DOLORMIN	3 (1.6)	2 (1.1)	0 (0.0)	2 (1.0)	7 (0.9)
L-THYROXIN	3 (1.6)	1 (0.6)	1 (0.6)	2 (1.0)	7 (0.9)
LEIOS	3 (1.6)	1 (0.6)	3 (1.7)	0 (0.0)	7 (0.9)
NUVARING	4 (2.2)	1 (0.6)	2 (1.1)	0 (0.0)	7 (0.9)
AMOXICILLIN	0 (0.0)	2 (1.1)	1 (0.6)	3 (1.5)	6 (0.8)
ASPIRIN COMPLEX	3 (1.6)	2 (1.1)	1 (0.6)	0 (0.0)	6 (0.8)
CILEST	1 (0.5)	3 (1.7)	2 (1.1)	0 (0.0)	6 (0.8)
GRIPPOSTAD	1 (0.5)	2 (1.1)	2 (1.1)	1 (0.5)	6 (0.8)
SINUPRET	3 (1.6)	2 (1.1)	0 (0.0)	1 (0.5)	6 (0.8)

A subject with more than one entry on the same drug was only counted once
Percentages based on total no. of subjects in each group
Table is sorted by total frequency

Prior and concomitant medication by brand name, Safety dataset

Brand name	Group 1 (MM) (N=183)	Group 2 (MP) (N=181)	Group 3 (PP) (N=181)	Group 4 (M-) (N=200)	Total (N=745)
VOLTAREN	2 (1.1)	2 (1.1)	1 (0.6)	1 (0.5)	6 (0.8)
ACETYLCYSTEIN	2 (1.1)	1 (0.6)	2 (1.1)	0 (0.0)	5 (0.7)
BUSCOPAN	1 (0.5)	2 (1.1)	1 (0.6)	1 (0.5)	5 (0.7)
EUTHYROX	0 (0.0)	1 (0.6)	2 (1.1)	2 (1.0)	5 (0.7)
JULIETTE	2 (1.1)	2 (1.1)	1 (0.6)	0 (0.0)	5 (0.7)
LAMUNA 20	0 (0.0)	2 (1.1)	2 (1.1)	1 (0.5)	5 (0.7)
MEDITONSIN	2 (1.1)	1 (0.6)	2 (1.1)	0 (0.0)	5 (0.7)
NASIC	2 (1.1)	2 (1.1)	1 (0.6)	0 (0.0)	5 (0.7)
NOVIAL	2 (1.1)	1 (0.6)	2 (1.1)	0 (0.0)	5 (0.7)
OLYNTH	3 (1.6)	1 (0.6)	1 (0.6)	0 (0.0)	5 (0.7)
SINUPRET FORTE	2 (1.1)	1 (0.6)	1 (0.6)	1 (0.5)	5 (0.7)
THOMAPYRIN	2 (1.1)	1 (0.6)	0 (0.0)	2 (1.0)	5 (0.7)
ASPIRIN PLUS C	1 (0.5)	1 (0.6)	0 (0.0)	2 (1.0)	4 (0.5)
CERAZETTE	2 (1.1)	0 (0.0)	2 (1.1)	0 (0.0)	4 (0.5)
CITALOPRAM	3 (1.6)	1 (0.6)	0 (0.0)	0 (0.0)	4 (0.5)
DOLORMIN EXTRA	1 (0.5)	0 (0.0)	2 (1.1)	1 (0.5)	4 (0.5)
GELOMYRTOL	3 (1.6)	0 (0.0)	1 (0.6)	0 (0.0)	4 (0.5)
JASMIN	0 (0.0)	0 (0.0)	3 (1.7)	1 (0.5)	4 (0.5)
MINISISTON	2 (1.1)	2 (1.1)	0 (0.0)	0 (0.0)	4 (0.5)
MIRENA	0 (0.0)	0 (0.0)	2 (1.1)	2 (1.0)	4 (0.5)
NOVALGIN	0 (0.0)	2 (1.1)	1 (0.6)	1 (0.5)	4 (0.5)
NUVA-RING	1 (0.5)	0 (0.0)	3 (1.7)	0 (0.0)	4 (0.5)
OTRIVEN	2 (1.1)	0 (0.0)	2 (1.1)	0 (0.0)	4 (0.5)
TRIGOA	0 (0.0)	3 (1.7)	0 (0.0)	1 (0.5)	4 (0.5)
ACC AKUT	1 (0.5)	2 (1.1)	0 (0.0)	0 (0.0)	3 (0.4)
ASPIRIN + C	0 (0.0)	1 (0.6)	2 (1.1)	0 (0.0)	3 (0.4)
ASPIRIN+C	1 (0.5)	1 (0.6)	1 (0.6)	0 (0.0)	3 (0.4)
BUSCOPAN PLUS	2 (1.1)	0 (0.0)	1 (0.6)	0 (0.0)	3 (0.4)

A subject with more than one entry on the same drug was only counted once
Percentages based on total no. of subjects in each group
Table is sorted by total frequency

Prior and concomitant medication by brand name, Safety dataset

Brand name	Group 1 (MM) (N=183)	Group 2 (MP) (N=181)	Group 3 (PP) (N=181)	Group 4 (M-) (N=200)	Total (N=745)
DOLO-DOBENDAN	1 (0.5)	1 (0.6)	1 (0.6)	0 (0.0)	3 (0.4)
GELOMYRTOL FORTE	0 (0.0)	2 (1.1)	1 (0.6)	0 (0.0)	3 (0.4)
IBUPROFEN 400	1 (0.5)	0 (0.0)	2 (1.1)	0 (0.0)	3 (0.4)
MUCOSOLVAN	2 (1.1)	1 (0.6)	0 (0.0)	0 (0.0)	3 (0.4)
NASIVIN	1 (0.5)	0 (0.0)	1 (0.6)	1 (0.5)	3 (0.4)
NEXIUM	1 (0.5)	0 (0.0)	1 (0.6)	1 (0.5)	3 (0.4)
THYRONAJOD	0 (0.0)	0 (0.0)	0 (0.0)	3 (1.5)	3 (0.4)
VOLTAREN DISPERS	0 (0.0)	1 (0.6)	0 (0.0)	2 (1.0)	3 (0.4)
VOLTAREN RESINAT	0 (0.0)	0 (0.0)	0 (0.0)	3 (1.5)	3 (0.4)
ZITHROMAX	0 (0.0)	2 (1.1)	1 (0.6)	0 (0.0)	3 (0.4)
"CORTISON"	0 (0.0)	1 (0.6)	1 (0.6)	0 (0.0)	2 (0.3)
AERIUS	1 (0.5)	0 (0.0)	1 (0.6)	0 (0.0)	2 (0.3)
ALLERGOSPASMIN	0 (0.0)	1 (0.6)	0 (0.0)	1 (0.5)	2 (0.3)
ANGOCIN	0 (0.0)	0 (0.0)	1 (0.6)	1 (0.5)	2 (0.3)
ASS+C RATIOPHARM	0 (0.0)	2 (1.1)	0 (0.0)	0 (0.0)	2 (0.3)
AZITHROMYCIN	1 (0.5)	1 (0.6)	0 (0.0)	0 (0.0)	2 (0.3)
BELLA HEXAL 35	0 (0.0)	1 (0.6)	1 (0.6)	0 (0.0)	2 (0.3)
BENURON	1 (0.5)	0 (0.0)	0 (0.0)	1 (0.5)	2 (0.3)
BEOFENAC	0 (0.0)	1 (0.6)	0 (0.0)	1 (0.5)	2 (0.3)
CEFUROXIM	0 (0.0)	0 (0.0)	1 (0.6)	1 (0.5)	2 (0.3)
CETIRIZIN	0 (0.0)	1 (0.6)	0 (0.0)	1 (0.5)	2 (0.3)
CLARITHROMYCIN	0 (0.0)	1 (0.6)	1 (0.6)	0 (0.0)	2 (0.3)
COTRIM FORTE	0 (0.0)	1 (0.6)	1 (0.6)	0 (0.0)	2 (0.3)
DESMIN 30	0 (0.0)	1 (0.6)	1 (0.6)	0 (0.0)	2 (0.3)
DICLAC	1 (0.5)	0 (0.0)	0 (0.0)	1 (0.5)	2 (0.3)
DOBENDAN	1 (0.5)	0 (0.0)	1 (0.6)	0 (0.0)	2 (0.3)
DOLOMO	1 (0.5)	0 (0.0)	0 (0.0)	1 (0.5)	2 (0.3)
FEMIGOA	0 (0.0)	1 (0.6)	1 (0.6)	0 (0.0)	2 (0.3)

A subject with more than one entry on the same drug was only counted once
Percentages based on total no. of subjects in each group
Table is sorted by total frequency

Prior and concomitant medication by brand name, Safety dataset

Brand name	Group 1 (MM) (N=183)	Group 2 (MP) (N=181)	Group 3 (PP) (N=181)	Group 4 (M-) (N=200)	Total (N=745)
FENISTIL GEL	1 (0.5)	0 (0.0)	0 (0.0)	1 (0.5)	2 (0.3)
FERRO SANOL	2 (1.1)	0 (0.0)	0 (0.0)	0 (0.0)	2 (0.3)
GRIPPOSTAD C	0 (0.0)	0 (0.0)	2 (1.1)	0 (0.0)	2 (0.3)
GYNODIAN	0 (0.0)	0 (0.0)	0 (0.0)	2 (1.0)	2 (0.3)
IBEROGAST	1 (0.5)	0 (0.0)	1 (0.6)	0 (0.0)	2 (0.3)
IODID	2 (1.1)	0 (0.0)	0 (0.0)	0 (0.0)	2 (0.3)
JODTHYROX	0 (0.0)	0 (0.0)	0 (0.0)	2 (1.0)	2 (0.3)
LAMUNA	0 (0.0)	1 (0.6)	1 (0.6)	0 (0.0)	2 (0.3)
LORANO	0 (0.0)	2 (1.1)	0 (0.0)	0 (0.0)	2 (0.3)
LORATADIN	1 (0.5)	0 (0.0)	0 (0.0)	1 (0.5)	2 (0.3)
LOVELLE	0 (0.0)	1 (0.6)	0 (0.0)	1 (0.5)	2 (0.3)
MERICOMB	0 (0.0)	0 (0.0)	0 (0.0)	2 (1.0)	2 (0.3)
MIRTAZAPIN	0 (0.0)	2 (1.1)	0 (0.0)	0 (0.0)	2 (0.3)
MYDOCALM	0 (0.0)	1 (0.6)	0 (0.0)	1 (0.5)	2 (0.3)
NEO-EUNOMIN	0 (0.0)	1 (0.6)	0 (0.0)	1 (0.5)	2 (0.3)
NEOANGIN	0 (0.0)	0 (0.0)	0 (0.0)	2 (1.0)	2 (0.3)
NEURALGIN	0 (0.0)	0 (0.0)	1 (0.6)	1 (0.5)	2 (0.3)
NUVA- RING	0 (0.0)	2 (1.1)	0 (0.0)	0 (0.0)	2 (0.3)
OMEPRAZOL	1 (0.5)	0 (0.0)	1 (0.6)	0 (0.0)	2 (0.3)
PAROXETIN	1 (0.5)	0 (0.0)	0 (0.0)	1 (0.5)	2 (0.3)
PENICILLIN	0 (0.0)	2 (1.1)	0 (0.0)	0 (0.0)	2 (0.3)
PERENTEROL	1 (0.5)	0 (0.0)	0 (0.0)	1 (0.5)	2 (0.3)
PROPOFOL	0 (0.0)	0 (0.0)	2 (1.1)	0 (0.0)	2 (0.3)
PROSPAN	1 (0.5)	1 (0.6)	0 (0.0)	0 (0.0)	2 (0.3)
SCANDICAIN	0 (0.0)	0 (0.0)	1 (0.6)	1 (0.5)	2 (0.3)
SEROQUEL	0 (0.0)	1 (0.6)	0 (0.0)	1 (0.5)	2 (0.3)
SILOMAT	2 (1.1)	0 (0.0)	0 (0.0)	0 (0.0)	2 (0.3)
TOMAPYRIN	1 (0.5)	1 (0.6)	0 (0.0)	0 (0.0)	2 (0.3)

A subject with more than one entry on the same drug was only counted once
Percentages based on total no. of subjects in each group
Table is sorted by total frequency

Prior and concomitant medication by brand name, Safety dataset

Brand name	Group 1 (MM) (N=183)	Group 2 (MP) (N=181)	Group 3 (PP) (N=181)	Group 4 (M-) (N=200)	Total (N=745)
TRAUMEEL	1 (0.5)	0 (0.0)	1 (0.6)	0 (0.0)	2 (0.3)
UMCKALOABO	1 (0.5)	0 (0.0)	1 (0.6)	0 (0.0)	2 (0.3)
UNOFEM	0 (0.0)	1 (0.6)	1 (0.6)	0 (0.0)	2 (0.3)
VIVIMED	1 (0.5)	1 (0.6)	0 (0.0)	0 (0.0)	2 (0.3)
VIVINOX	0 (0.0)	0 (0.0)	1 (0.6)	1 (0.5)	2 (0.3)
VOLTAREN EMULGEL	1 (0.5)	0 (0.0)	0 (0.0)	1 (0.5)	2 (0.3)
WICK MEDI NAIT	1 (0.5)	1 (0.6)	0 (0.0)	0 (0.0)	2 (0.3)
WICK MEDI NIGHT	1 (0.5)	0 (0.0)	1 (0.6)	0 (0.0)	2 (0.3)
WICK MEDINAIT	0 (0.0)	0 (0.0)	1 (0.6)	1 (0.5)	2 (0.3)
AARANE	0 (0.0)	1 (0.6)	0 (0.0)	0 (0.0)	1 (0.1)
ACIC 200	0 (0.0)	0 (0.0)	1 (0.6)	0 (0.0)	1 (0.1)
ACICLAV	0 (0.0)	1 (0.6)	0 (0.0)	0 (0.0)	1 (0.1)
ACICLOVIR	1 (0.5)	0 (0.0)	0 (0.0)	0 (0.0)	1 (0.1)
ACICLOVIR RATIOPHARM	0 (0.0)	0 (0.0)	1 (0.6)	0 (0.0)	1 (0.1)
ACITHROMYCIN	0 (0.0)	1 (0.6)	0 (0.0)	0 (0.0)	1 (0.1)
AGILAMIN	1 (0.5)	0 (0.0)	0 (0.0)	0 (0.0)	1 (0.1)
AGNUCASTON	0 (0.0)	0 (0.0)	0 (0.0)	1 (0.5)	1 (0.1)
AKTIVPUNKT EISEN PLUS	0 (0.0)	1 (0.6)	0 (0.0)	0 (0.0)	1 (0.1)
AKTIVPUNKT FOLSÄURE	0 (0.0)	1 (0.6)	0 (0.0)	0 (0.0)	1 (0.1)
AKTIVPUNKT JOD	0 (0.0)	1 (0.6)	0 (0.0)	0 (0.0)	1 (0.1)
AKTIVPUNKT ZINK PLUS	0 (0.0)	1 (0.6)	0 (0.0)	0 (0.0)	1 (0.1)
AKTREN SPEZIAL	0 (0.0)	1 (0.6)	0 (0.0)	0 (0.0)	1 (0.1)
AKTREN SPEZIAL (IBUPROFEN)	0 (0.0)	1 (0.6)	0 (0.0)	0 (0.0)	1 (0.1)
ALGAZONE	0 (0.0)	1 (0.6)	0 (0.0)	0 (0.0)	1 (0.1)
ALLEGRO	0 (0.0)	0 (0.0)	0 (0.0)	1 (0.5)	1 (0.1)
ALLERGOLACT	0 (0.0)	1 (0.6)	0 (0.0)	0 (0.0)	1 (0.1)
AMAXIBETA	1 (0.5)	0 (0.0)	0 (0.0)	0 (0.0)	1 (0.1)
AMITRIPTYLIN	0 (0.0)	1 (0.6)	0 (0.0)	0 (0.0)	1 (0.1)

A subject with more than one entry on the same drug was only counted once
Percentages based on total no. of subjects in each group
Table is sorted by total frequency

Prior and concomitant medication by brand name, Safety dataset

Brand name	Group 1 (MM) (N=183)	Group 2 (MP) (N=181)	Group 3 (PP) (N=181)	Group 4 (M-) (N=200)	Total (N=745)
AMOXILCILIN	0 (0.0)	0 (0.0)	1 (0.6)	0 (0.0)	1 (0.1)
ANALGIN	0 (0.0)	0 (0.0)	1 (0.6)	0 (0.0)	1 (0.1)
ANDROCUR	0 (0.0)	0 (0.0)	0 (0.0)	1 (0.5)	1 (0.1)
APSOMOL N	1 (0.5)	0 (0.0)	0 (0.0)	0 (0.0)	1 (0.1)
AQUALIBRA	1 (0.5)	0 (0.0)	0 (0.0)	0 (0.0)	1 (0.1)
ARTHOTEC	0 (0.0)	0 (0.0)	0 (0.0)	1 (0.5)	1 (0.1)
ASCOTOP	0 (0.0)	0 (0.0)	0 (0.0)	1 (0.5)	1 (0.1)
ASPIRIN +C	0 (0.0)	0 (0.0)	0 (0.0)	1 (0.5)	1 (0.1)
ASPIRIN PROTECT ASS	1 (0.5)	0 (0.0)	0 (0.0)	0 (0.0)	1 (0.1)
ASS 100	0 (0.0)	1 (0.6)	0 (0.0)	0 (0.0)	1 (0.1)
ASS 500	1 (0.5)	0 (0.0)	0 (0.0)	0 (0.0)	1 (0.1)
ASS 500 BRAUSE	0 (0.0)	0 (0.0)	0 (0.0)	1 (0.5)	1 (0.1)
ASS ASPIRIN	0 (0.0)	1 (0.6)	0 (0.0)	0 (0.0)	1 (0.1)
ASS RATIOPHARM	0 (0.0)	1 (0.6)	0 (0.0)	0 (0.0)	1 (0.1)
ATOMOXETIN	0 (0.0)	0 (0.0)	1 (0.6)	0 (0.0)	1 (0.1)
ATOSIL	0 (0.0)	1 (0.6)	0 (0.0)	0 (0.0)	1 (0.1)
AUGMENTAN	0 (0.0)	0 (0.0)	1 (0.6)	0 (0.0)	1 (0.1)
BASOCIN SOLUTION	0 (0.0)	1 (0.6)	0 (0.0)	0 (0.0)	1 (0.1)
BELLA	1 (0.5)	0 (0.0)	0 (0.0)	0 (0.0)	1 (0.1)
BELLA HEXAL	1 (0.5)	0 (0.0)	0 (0.0)	0 (0.0)	1 (0.1)
BELLARA	1 (0.5)	0 (0.0)	0 (0.0)	0 (0.0)	1 (0.1)
BEPANTHEN SALBE	0 (0.0)	1 (0.6)	0 (0.0)	0 (0.0)	1 (0.1)
BEPANTOL	0 (0.0)	0 (0.0)	1 (0.6)	0 (0.0)	1 (0.1)
BERBERIL N EDO	0 (0.0)	0 (0.0)	1 (0.6)	0 (0.0)	1 (0.1)
BEROTEC	1 (0.5)	0 (0.0)	0 (0.0)	0 (0.0)	1 (0.1)
BEROTEC FENOTEROL	0 (0.0)	0 (0.0)	1 (0.6)	0 (0.0)	1 (0.1)
BETAHISTIN	0 (0.0)	1 (0.6)	0 (0.0)	0 (0.0)	1 (0.1)
BIKALM	0 (0.0)	0 (0.0)	0 (0.0)	1 (0.5)	1 (0.1)

A subject with more than one entry on the same drug was only counted once
Percentages based on total no. of subjects in each group
Table is sorted by total frequency

Prior and concomitant medication by brand name, Safety dataset

Brand name	Group 1 (MM) (N=183)	Group 2 (MP) (N=181)	Group 3 (PP) (N=181)	Group 4 (M-) (N=200)	Total (N=745)
BIOTIN	0 (0.0)	0 (0.0)	0 (0.0)	1 (0.5)	1 (0.1)
BISOPROLOL	1 (0.5)	0 (0.0)	0 (0.0)	0 (0.0)	1 (0.1)
BIVIOL	1 (0.5)	0 (0.0)	0 (0.0)	0 (0.0)	1 (0.1)
BLEMAREN N	0 (0.0)	0 (0.0)	1 (0.6)	0 (0.0)	1 (0.1)
BROMELAIN POS	1 (0.5)	0 (0.0)	0 (0.0)	0 (0.0)	1 (0.1)
BRONCHICUM PASTILLEN	0 (0.0)	1 (0.6)	0 (0.0)	0 (0.0)	1 (0.1)
BRONCHOSPRAY	0 (0.0)	0 (0.0)	1 (0.6)	0 (0.0)	1 (0.1)
CALCIUM 500	0 (0.0)	0 (0.0)	0 (0.0)	1 (0.5)	1 (0.1)
CALCIUM SANDOZ FORTE	0 (0.0)	0 (0.0)	0 (0.0)	1 (0.5)	1 (0.1)
CANESTEN	0 (0.0)	0 (0.0)	0 (0.0)	1 (0.5)	1 (0.1)
CANESTEN EXTRA	0 (0.0)	0 (0.0)	0 (0.0)	1 (0.5)	1 (0.1)
CANESTEN EXTRA BIFONAZOL CREME	0 (0.0)	0 (0.0)	1 (0.6)	0 (0.0)	1 (0.1)
CANIFUG CREMOLUM 200 KOMBI	0 (0.0)	0 (0.0)	0 (0.0)	1 (0.5)	1 (0.1)
CANIFUG-CREME	0 (0.0)	0 (0.0)	1 (0.6)	0 (0.0)	1 (0.1)
CARBAMID + VAS CREME	0 (0.0)	0 (0.0)	0 (0.0)	1 (0.5)	1 (0.1)
CARBOSTESIN	1 (0.5)	0 (0.0)	0 (0.0)	0 (0.0)	1 (0.1)
CELEBREX	0 (0.0)	0 (0.0)	0 (0.0)	1 (0.5)	1 (0.1)
CENTRUM	1 (0.5)	0 (0.0)	0 (0.0)	0 (0.0)	1 (0.1)
CETRIZIN DIHYDRO CHLORID	0 (0.0)	0 (0.0)	0 (0.0)	1 (0.5)	1 (0.1)
CETU HEXAL	0 (0.0)	0 (0.0)	0 (0.0)	1 (0.5)	1 (0.1)
CHLORHEXAMED 0,1% LSG.	0 (0.0)	1 (0.6)	0 (0.0)	0 (0.0)	1 (0.1)
CILOXAN 3MG/ML	0 (0.0)	0 (0.0)	0 (0.0)	1 (0.5)	1 (0.1)
CIPRALEX	0 (0.0)	0 (0.0)	1 (0.6)	0 (0.0)	1 (0.1)
CIPRAMIL	1 (0.5)	0 (0.0)	0 (0.0)	0 (0.0)	1 (0.1)
CIPRO	0 (0.0)	0 (0.0)	0 (0.0)	1 (0.5)	1 (0.1)
CIPROBAY	0 (0.0)	1 (0.6)	0 (0.0)	0 (0.0)	1 (0.1)
CIPROFLOXACIN	0 (0.0)	0 (0.0)	0 (0.0)	1 (0.5)	1 (0.1)
CITERICIN	0 (0.0)	1 (0.6)	0 (0.0)	0 (0.0)	1 (0.1)

A subject with more than one entry on the same drug was only counted once
Percentages based on total no. of subjects in each group
Table is sorted by total frequency

Prior and concomitant medication by brand name, Safety dataset

Brand name	Group 1 (MM) (N=183)	Group 2 (MP) (N=181)	Group 3 (PP) (N=181)	Group 4 (M-) (N=200)	Total (N=745)
CLARITHROMYCIN-RATIOPHARM	0 (0.0)	0 (0.0)	0 (0.0)	1 (0.5)	1 (0.1)
CLARITROMYCIN	0 (0.0)	1 (0.6)	0 (0.0)	0 (0.0)	1 (0.1)
CLIMODIEN 2/2	1 (0.5)	0 (0.0)	0 (0.0)	0 (0.0)	1 (0.1)
CLINDAMYCIN	0 (0.0)	0 (0.0)	1 (0.6)	0 (0.0)	1 (0.1)
CLIVARIN 1.750	0 (0.0)	0 (0.0)	1 (0.6)	0 (0.0)	1 (0.1)
CLONIDIN	0 (0.0)	0 (0.0)	1 (0.6)	0 (0.0)	1 (0.1)
CODEIN	0 (0.0)	0 (0.0)	1 (0.6)	0 (0.0)	1 (0.1)
CONCEPLAN	1 (0.5)	0 (0.0)	0 (0.0)	0 (0.0)	1 (0.1)
CONCEPLAN M	0 (0.0)	0 (0.0)	0 (0.0)	1 (0.5)	1 (0.1)
CONCOR	0 (0.0)	0 (0.0)	0 (0.0)	1 (0.5)	1 (0.1)
CONVULEX	0 (0.0)	0 (0.0)	0 (0.0)	1 (0.5)	1 (0.1)
CORTISON	0 (0.0)	1 (0.6)	0 (0.0)	0 (0.0)	1 (0.1)
CYSTINOL AKUT	0 (0.0)	1 (0.6)	0 (0.0)	0 (0.0)	1 (0.1)
DAY PLUS MG	0 (0.0)	0 (0.0)	0 (0.0)	1 (0.5)	1 (0.1)
DEPON (N.K.)	0 (0.0)	1 (0.6)	0 (0.0)	0 (0.0)	1 (0.1)
DEPOT PROVERA	0 (0.0)	0 (0.0)	1 (0.6)	0 (0.0)	1 (0.1)
DERMAB	1 (0.5)	0 (0.0)	0 (0.0)	0 (0.0)	1 (0.1)
DERMATOP	0 (0.0)	1 (0.6)	0 (0.0)	0 (0.0)	1 (0.1)
DESLORATADIN	0 (0.0)	0 (0.0)	1 (0.6)	0 (0.0)	1 (0.1)
DESMIN	1 (0.5)	0 (0.0)	0 (0.0)	0 (0.0)	1 (0.1)
DESMIN20	0 (0.0)	0 (0.0)	1 (0.6)	0 (0.0)	1 (0.1)
DEXA-POLYSPECTRON	0 (0.0)	0 (0.0)	1 (0.6)	0 (0.0)	1 (0.1)
DEXAMETHASON	1 (0.5)	0 (0.0)	0 (0.0)	0 (0.0)	1 (0.1)
DHEA	0 (0.0)	0 (0.0)	0 (0.0)	1 (0.5)	1 (0.1)
DIAMOX	1 (0.5)	0 (0.0)	0 (0.0)	0 (0.0)	1 (0.1)
DIANE	0 (0.0)	1 (0.6)	0 (0.0)	0 (0.0)	1 (0.1)
DIAZEPAM	1 (0.5)	0 (0.0)	0 (0.0)	0 (0.0)	1 (0.1)
DIAZEPAM-TROPFEN	0 (0.0)	1 (0.6)	0 (0.0)	0 (0.0)	1 (0.1)

A subject with more than one entry on the same drug was only counted once
Percentages based on total no. of subjects in each group
Table is sorted by total frequency

Prior and concomitant medication by brand name, Safety dataset

Brand name	Group 1 (MM) (N=183)	Group 2 (MP) (N=181)	Group 3 (PP) (N=181)	Group 4 (M-) (N=200)	Total (N=745)
DIBONDRIN	1 (0.5)	0 (0.0)	0 (0.0)	0 (0.0)	1 (0.1)
DICLO-DIVIDO	0 (0.0)	0 (0.0)	0 (0.0)	1 (0.5)	1 (0.1)
DICLOFENAC RATIOPHARM 50 MG	0 (0.0)	0 (0.0)	1 (0.6)	0 (0.0)	1 (0.1)
DICLOFENAC RETARD	0 (0.0)	0 (0.0)	1 (0.6)	0 (0.0)	1 (0.1)
DIMENHYDRINAT	1 (0.5)	0 (0.0)	0 (0.0)	0 (0.0)	1 (0.1)
DOLO DOBENDAN	1 (0.5)	0 (0.0)	0 (0.0)	0 (0.0)	1 (0.1)
DOLODOBENDAN	0 (0.0)	0 (0.0)	1 (0.6)	0 (0.0)	1 (0.1)
DOLODOC	0 (0.0)	1 (0.6)	0 (0.0)	0 (0.0)	1 (0.1)
DOLOMO T/N	0 (0.0)	1 (0.6)	0 (0.0)	0 (0.0)	1 (0.1)
DOLORMIN FÜR FRAUEN	0 (0.0)	1 (0.6)	0 (0.0)	0 (0.0)	1 (0.1)
DOLORMIN MIGRÄNE	1 (0.5)	0 (0.0)	0 (0.0)	0 (0.0)	1 (0.1)
DONA S200	0 (0.0)	0 (0.0)	1 (0.6)	0 (0.0)	1 (0.1)
DONORMYL	1 (0.5)	0 (0.0)	0 (0.0)	0 (0.0)	1 (0.1)
DOREGRIPPIN	0 (0.0)	0 (0.0)	0 (0.0)	1 (0.5)	1 (0.1)
DORMICUM	0 (0.0)	0 (0.0)	1 (0.6)	0 (0.0)	1 (0.1)
DOXAM	0 (0.0)	0 (0.0)	1 (0.6)	0 (0.0)	1 (0.1)
DOXEPIN	0 (0.0)	1 (0.6)	0 (0.0)	0 (0.0)	1 (0.1)
DOXI-HEXAL DOXICYCLIN	1 (0.5)	0 (0.0)	0 (0.0)	0 (0.0)	1 (0.1)
DOXIMUCOL	0 (0.0)	0 (0.0)	1 (0.6)	0 (0.0)	1 (0.1)
DOXY	0 (0.0)	1 (0.6)	0 (0.0)	0 (0.0)	1 (0.1)
DOXYCYCLIN	0 (0.0)	1 (0.6)	0 (0.0)	0 (0.0)	1 (0.1)
DOXYCYCLIN AL 100 T	0 (0.0)	1 (0.6)	0 (0.0)	0 (0.0)	1 (0.1)
DOXYCYCLINE	0 (0.0)	1 (0.6)	0 (0.0)	0 (0.0)	1 (0.1)
DOXYHEXAL	0 (0.0)	0 (0.0)	0 (0.0)	1 (0.5)	1 (0.1)
DYSMENOL	0 (0.0)	0 (0.0)	1 (0.6)	0 (0.0)	1 (0.1)
EBASTEL	0 (0.0)	0 (0.0)	0 (0.0)	1 (0.5)	1 (0.1)
EBENOL CREME	1 (0.5)	0 (0.0)	0 (0.0)	0 (0.0)	1 (0.1)
ECHINAZIN	0 (0.0)	0 (0.0)	0 (0.0)	1 (0.5)	1 (0.1)

A subject with more than one entry on the same drug was only counted once
Percentages based on total no. of subjects in each group
Table is sorted by total frequency

Prior and concomitant medication by brand name, Safety dataset

Brand name	Group 1 (MM) (N=183)	Group 2 (MP) (N=181)	Group 3 (PP) (N=181)	Group 4 (M-) (N=200)	Total (N=745)
ECURAL CREME	0 (0.0)	0 (0.0)	0 (0.0)	1 (0.5)	1 (0.1)
EFEROX	0 (0.0)	0 (0.0)	0 (0.0)	1 (0.5)	1 (0.1)
EFFORTIL	0 (0.0)	0 (0.0)	0 (0.0)	1 (0.5)	1 (0.1)
EFFORTIL PLUS (PERLONGETTEN)	0 (0.0)	0 (0.0)	0 (0.0)	1 (0.5)	1 (0.1)
EISENBRAUSETABLETTE	0 (0.0)	0 (0.0)	1 (0.6)	0 (0.0)	1 (0.1)
ELECTROLYTE SOLUTION	0 (0.0)	0 (0.0)	1 (0.6)	0 (0.0)	1 (0.1)
ENTOCYD COMPOND (=STREPTOMYCINSULFAT)	1 (0.5)	0 (0.0)	0 (0.0)	0 (0.0)	1 (0.1)
ERY (HEXAL) 500	1 (0.5)	0 (0.0)	0 (0.0)	0 (0.0)	1 (0.1)
ESBERITOX	1 (0.5)	0 (0.0)	0 (0.0)	0 (0.0)	1 (0.1)
ESTICIA	0 (0.0)	0 (0.0)	0 (0.0)	1 (0.5)	1 (0.1)
ETIL CT	0 (0.0)	0 (0.0)	0 (0.0)	1 (0.5)	1 (0.1)
EUBIOLAC VERLA	1 (0.5)	0 (0.0)	0 (0.0)	0 (0.0)	1 (0.1)
EUCERIN UREA	1 (0.5)	0 (0.0)	0 (0.0)	0 (0.0)	1 (0.1)
EVRA	1 (0.5)	0 (0.0)	0 (0.0)	0 (0.0)	1 (0.1)
FENISTIL 24	0 (0.0)	1 (0.6)	0 (0.0)	0 (0.0)	1 (0.1)
FENISTIL HYDROCORT CREME	0 (0.0)	0 (0.0)	1 (0.6)	0 (0.0)	1 (0.1)
FENISTIL-GEL	0 (0.0)	1 (0.6)	0 (0.0)	0 (0.0)	1 (0.1)
FENSIUM	0 (0.0)	0 (0.0)	1 (0.6)	0 (0.0)	1 (0.1)
FERRO SANOL® DUODENAL MITE 50 MG/-DUODENAL	0 (0.0)	0 (0.0)	1 (0.6)	0 (0.0)	1 (0.1)
FERRO SONAL	0 (0.0)	1 (0.6)	0 (0.0)	0 (0.0)	1 (0.1)
FERROSANOL	0 (0.0)	0 (0.0)	0 (0.0)	1 (0.5)	1 (0.1)
FLUOXEITIN	0 (0.0)	1 (0.6)	0 (0.0)	0 (0.0)	1 (0.1)
FLUOXETIN	1 (0.5)	0 (0.0)	0 (0.0)	0 (0.0)	1 (0.1)
FRUBIASE CALCIUM FORTE 500	0 (0.0)	0 (0.0)	0 (0.0)	1 (0.5)	1 (0.1)
FRUBIENZYM	1 (0.5)	0 (0.0)	0 (0.0)	0 (0.0)	1 (0.1)
GAPAPENTIN	0 (0.0)	0 (0.0)	0 (0.0)	1 (0.5)	1 (0.1)
GASTROSIL	0 (0.0)	0 (0.0)	1 (0.6)	0 (0.0)	1 (0.1)
GELO-MYRTOL	0 (0.0)	0 (0.0)	0 (0.0)	1 (0.5)	1 (0.1)

A subject with more than one entry on the same drug was only counted once
Percentages based on total no. of subjects in each group
Table is sorted by total frequency

Prior and concomitant medication by brand name, Safety dataset

Brand name	Group 1 (MM) (N=183)	Group 2 (MP) (N=181)	Group 3 (PP) (N=181)	Group 4 (M-) (N=200)	Total (N=745)
GELONIDA	0 (0.0)	1 (0.6)	0 (0.0)	0 (0.0)	1 (0.1)
GLUCOSAMIN-CHONDROITIN-FORMULA	0 (0.0)	0 (0.0)	0 (0.0)	1 (0.5)	1 (0.1)
GRIPP- HEEL	0 (0.0)	0 (0.0)	0 (0.0)	1 (0.5)	1 (0.1)
GRIPPOCAPS	1 (0.5)	0 (0.0)	0 (0.0)	0 (0.0)	1 (0.1)
GRIPPOSTAD PARACETAMOL	0 (0.0)	1 (0.6)	0 (0.0)	0 (0.0)	1 (0.1)
HCT HEXAL	0 (0.0)	0 (0.0)	0 (0.0)	1 (0.5)	1 (0.1)
HEPARIN	0 (0.0)	0 (0.0)	1 (0.6)	0 (0.0)	1 (0.1)
HEXORALETTEN	0 (0.0)	0 (0.0)	1 (0.6)	0 (0.0)	1 (0.1)
HYDRODERM	0 (0.0)	1 (0.6)	0 (0.0)	0 (0.0)	1 (0.1)
HYLAK TRPF.+ACIDOPHILUS	0 (0.0)	0 (0.0)	0 (0.0)	1 (0.5)	1 (0.1)
IBU HEXAL AKUT	1 (0.5)	0 (0.0)	0 (0.0)	0 (0.0)	1 (0.1)
IBU-HEMOPHARM	1 (0.5)	0 (0.0)	0 (0.0)	0 (0.0)	1 (0.1)
IBU-RATIOPHARM 500 LYSINAT	1 (0.5)	0 (0.0)	0 (0.0)	0 (0.0)	1 (0.1)
IBUBETA 400 AKUT	1 (0.5)	0 (0.0)	0 (0.0)	0 (0.0)	1 (0.1)
IBUHEXAL	0 (0.0)	0 (0.0)	1 (0.6)	0 (0.0)	1 (0.1)
IBUPROFEN 200	0 (0.0)	0 (0.0)	1 (0.6)	0 (0.0)	1 (0.1)
IBUPROFEN 400 AKUT	0 (0.0)	1 (0.6)	0 (0.0)	0 (0.0)	1 (0.1)
IBUPROFEN 400 MG	0 (0.0)	0 (0.0)	0 (0.0)	1 (0.5)	1 (0.1)
IBUPROFEN 600	0 (0.0)	0 (0.0)	1 (0.6)	0 (0.0)	1 (0.1)
IBUPROFEN HEUMANN	1 (0.5)	0 (0.0)	0 (0.0)	0 (0.0)	1 (0.1)
IBUPROFEN STADA	1 (0.5)	0 (0.0)	0 (0.0)	0 (0.0)	1 (0.1)
IMODIUM	0 (0.0)	1 (0.6)	0 (0.0)	0 (0.0)	1 (0.1)
IMODIUM AKUT	1 (0.5)	0 (0.0)	0 (0.0)	0 (0.0)	1 (0.1)
IMOGAS (SIMETICON)	0 (0.0)	0 (0.0)	0 (0.0)	1 (0.5)	1 (0.1)
INIMUR	0 (0.0)	1 (0.6)	0 (0.0)	0 (0.0)	1 (0.1)
IODINE NUTR. SUPPLEMENT	0 (0.0)	0 (0.0)	1 (0.6)	0 (0.0)	1 (0.1)
ISOCILLIN 1,2 MEGA	0 (0.0)	1 (0.6)	0 (0.0)	0 (0.0)	1 (0.1)
ISOCILLIN 1.2 MEGA	0 (0.0)	1 (0.6)	0 (0.0)	0 (0.0)	1 (0.1)

A subject with more than one entry on the same drug was only counted once
Percentages based on total no. of subjects in each group
Table is sorted by total frequency

Prior and concomitant medication by brand name, Safety dataset

Brand name	Group 1 (MM) (N=183)	Group 2 (MP) (N=181)	Group 3 (PP) (N=181)	Group 4 (M-) (N=200)	Total (N=745)
ISOTREX GEL	0 (0.0)	1 (0.6)	0 (0.0)	0 (0.0)	1 (0.1)
JARSIN 600 MG	1 (0.5)	0 (0.0)	0 (0.0)	0 (0.0)	1 (0.1)
JOD-THYROX	0 (0.0)	1 (0.6)	0 (0.0)	0 (0.0)	1 (0.1)
JODETTEN	0 (0.0)	0 (0.0)	1 (0.6)	0 (0.0)	1 (0.1)
JODETTEN DEPOT HENNING	0 (0.0)	1 (0.6)	0 (0.0)	0 (0.0)	1 (0.1)
JODID 100	1 (0.5)	0 (0.0)	0 (0.0)	0 (0.0)	1 (0.1)
JODID 200	0 (0.0)	0 (0.0)	1 (0.6)	0 (0.0)	1 (0.1)
KABAN CREME	0 (0.0)	0 (0.0)	0 (0.0)	1 (0.5)	1 (0.1)
KEIMAX	1 (0.5)	0 (0.0)	0 (0.0)	0 (0.0)	1 (0.1)
KEPINOL	0 (0.0)	0 (0.0)	0 (0.0)	1 (0.5)	1 (0.1)
KETEK 400	0 (0.0)	0 (0.0)	1 (0.6)	0 (0.0)	1 (0.1)
KOLLAGENHYDROLYSAT	0 (0.0)	0 (0.0)	0 (0.0)	1 (0.5)	1 (0.1)
L-THYROX	1 (0.5)	0 (0.0)	0 (0.0)	0 (0.0)	1 (0.1)
L-THYROXIN (HENNING)	1 (0.5)	0 (0.0)	0 (0.0)	0 (0.0)	1 (0.1)
L-THYROXIN HENNING	0 (0.0)	0 (0.0)	1 (0.6)	0 (0.0)	1 (0.1)
LACTASE	0 (0.0)	0 (0.0)	1 (0.6)	0 (0.0)	1 (0.1)
LACTASE-ENCYME CAPSULES	0 (0.0)	1 (0.6)	0 (0.0)	0 (0.0)	1 (0.1)
LAIF 900	0 (0.0)	1 (0.6)	0 (0.0)	0 (0.0)	1 (0.1)
LAMINA 20	1 (0.5)	0 (0.0)	0 (0.0)	0 (0.0)	1 (0.1)
LAXOBERAL	1 (0.5)	0 (0.0)	0 (0.0)	0 (0.0)	1 (0.1)
LEDERMIX	0 (0.0)	1 (0.6)	0 (0.0)	0 (0.0)	1 (0.1)
LERAZETTE	1 (0.5)	0 (0.0)	0 (0.0)	0 (0.0)	1 (0.1)
LIDOCAIN	1 (0.5)	0 (0.0)	0 (0.0)	0 (0.0)	1 (0.1)
LIDOCAIN 1%	1 (0.5)	0 (0.0)	0 (0.0)	0 (0.0)	1 (0.1)
LINOLA CORT HYDRO 0.5	0 (0.0)	0 (0.0)	0 (0.0)	1 (0.5)	1 (0.1)
LINOLA UREA	0 (0.0)	0 (0.0)	0 (0.0)	1 (0.5)	1 (0.1)
LISIHEXAL	0 (0.0)	0 (0.0)	0 (0.0)	1 (0.5)	1 (0.1)
LIVIELLA	0 (0.0)	0 (0.0)	0 (0.0)	1 (0.5)	1 (0.1)

A subject with more than one entry on the same drug was only counted once
Percentages based on total no. of subjects in each group
Table is sorted by total frequency

Prior and concomitant medication by brand name, Safety dataset

Brand name	Group 1 (MM) (N=183)	Group 2 (MP) (N=181)	Group 3 (PP) (N=181)	Group 4 (M-) (N=200)	Total (N=745)
LIVOCAP	0 (0.0)	0 (0.0)	0 (0.0)	1 (0.5)	1 (0.1)
LOCABIOSOL	0 (0.0)	0 (0.0)	1 (0.6)	0 (0.0)	1 (0.1)
LOPERAMID	0 (0.0)	0 (0.0)	0 (0.0)	1 (0.5)	1 (0.1)
LORZAAR 50	0 (0.0)	0 (0.0)	0 (0.0)	1 (0.5)	1 (0.1)
LOVALADIN	0 (0.0)	0 (0.0)	0 (0.0)	1 (0.5)	1 (0.1)
MCP	0 (0.0)	1 (0.6)	0 (0.0)	0 (0.0)	1 (0.1)
MEAVERIN 0,5%-2ML	0 (0.0)	0 (0.0)	0 (0.0)	1 (0.5)	1 (0.1)
METHYLPHENIDAT	0 (0.0)	0 (0.0)	1 (0.6)	0 (0.0)	1 (0.1)
METOHEXAL SUCC	1 (0.5)	0 (0.0)	0 (0.0)	0 (0.0)	1 (0.1)
METRONIDAZOL	1 (0.5)	0 (0.0)	0 (0.0)	0 (0.0)	1 (0.1)
METRONIDAZOL (ARELIN)	0 (0.0)	1 (0.6)	0 (0.0)	0 (0.0)	1 (0.1)
MICROGYNOM	0 (0.0)	0 (0.0)	1 (0.6)	0 (0.0)	1 (0.1)
MICROGYNON 21	0 (0.0)	0 (0.0)	0 (0.0)	1 (0.5)	1 (0.1)
MIGRÄNE-KRANIT PHENAZON	1 (0.5)	0 (0.0)	0 (0.0)	0 (0.0)	1 (0.1)
MITRAZEPIN	0 (0.0)	1 (0.6)	0 (0.0)	0 (0.0)	1 (0.1)
MONO EMBOLEX 0,5	0 (0.0)	0 (0.0)	1 (0.6)	0 (0.0)	1 (0.1)
MONOSTEP 21	0 (0.0)	1 (0.6)	0 (0.0)	0 (0.0)	1 (0.1)
MULTILIND	0 (0.0)	0 (0.0)	0 (0.0)	1 (0.5)	1 (0.1)
MULTILOAD	0 (0.0)	0 (0.0)	0 (0.0)	1 (0.5)	1 (0.1)
MYOSON	0 (0.0)	0 (0.0)	0 (0.0)	1 (0.5)	1 (0.1)
NACL (NORMAL SALINE SOLUTION 0,9%)	1 (0.5)	0 (0.0)	0 (0.0)	0 (0.0)	1 (0.1)
NACL INFUSION	0 (0.0)	1 (0.6)	0 (0.0)	0 (0.0)	1 (0.1)
NASENSPRAY RATIOPHARM	1 (0.5)	0 (0.0)	0 (0.0)	0 (0.0)	1 (0.1)
NASONEX	0 (0.0)	0 (0.0)	0 (0.0)	1 (0.5)	1 (0.1)
NEO- EUNOMIN	1 (0.5)	0 (0.0)	0 (0.0)	0 (0.0)	1 (0.1)
NEOEUNOMIN	0 (0.0)	0 (0.0)	0 (0.0)	1 (0.5)	1 (0.1)
NIKORETTE	0 (0.0)	0 (0.0)	1 (0.6)	0 (0.0)	1 (0.1)
NOCTAMID	0 (0.0)	0 (0.0)	1 (0.6)	0 (0.0)	1 (0.1)

A subject with more than one entry on the same drug was only counted once
Percentages based on total no. of subjects in each group
Table is sorted by total frequency

Prior and concomitant medication by brand name, Safety dataset

Brand name	Group 1 (MM) (N=183)	Group 2 (MP) (N=181)	Group 3 (PP) (N=181)	Group 4 (M-) (N=200)	Total (N=745)
NORFLOXACIN	1 (0.5)	0 (0.0)	0 (0.0)	0 (0.0)	1 (0.1)
NOVALGIN TROPFEN	0 (0.0)	1 (0.6)	0 (0.0)	0 (0.0)	1 (0.1)
NUOVA RING	0 (0.0)	0 (0.0)	0 (0.0)	1 (0.5)	1 (0.1)
OFLO HEXAL (OFLOXACIN)	0 (0.0)	1 (0.6)	0 (0.0)	0 (0.0)	1 (0.1)
OMEP	0 (0.0)	0 (0.0)	1 (0.6)	0 (0.0)	1 (0.1)
ORFIRIL LONG 300 MG	0 (0.0)	1 (0.6)	0 (0.0)	0 (0.0)	1 (0.1)
OTALGAN	1 (0.5)	0 (0.0)	0 (0.0)	0 (0.0)	1 (0.1)
OTOBACID	0 (0.0)	0 (0.0)	0 (0.0)	1 (0.5)	1 (0.1)
OTOPAIN (IN INDONISIA)	0 (0.0)	1 (0.6)	0 (0.0)	0 (0.0)	1 (0.1)
PARACETAMOL 500	0 (0.0)	1 (0.6)	0 (0.0)	0 (0.0)	1 (0.1)
PARKEMED	1 (0.5)	0 (0.0)	0 (0.0)	0 (0.0)	1 (0.1)
PAROXAT	0 (0.0)	0 (0.0)	1 (0.6)	0 (0.0)	1 (0.1)
PENHEXAL 1.5 MEGA	0 (0.0)	0 (0.0)	0 (0.0)	1 (0.5)	1 (0.1)
PENICILLIN STADA 1.2 MEGA	0 (0.0)	1 (0.6)	0 (0.0)	0 (0.0)	1 (0.1)
PENICILLIN V	0 (0.0)	1 (0.6)	0 (0.0)	0 (0.0)	1 (0.1)
PENICILLIN V STADA	1 (0.5)	0 (0.0)	0 (0.0)	0 (0.0)	1 (0.1)
PENICILLIN V STADA 1.5 MEGA	0 (0.0)	1 (0.6)	0 (0.0)	0 (0.0)	1 (0.1)
PETI BELLE	1 (0.5)	0 (0.0)	0 (0.0)	0 (0.0)	1 (0.1)
PETITBELLE	1 (0.5)	0 (0.0)	0 (0.0)	0 (0.0)	1 (0.1)
PHLOGENZYM	0 (0.0)	0 (0.0)	1 (0.6)	0 (0.0)	1 (0.1)
PRACETAMOL	1 (0.5)	0 (0.0)	0 (0.0)	0 (0.0)	1 (0.1)
PRAMINO	0 (0.0)	1 (0.6)	0 (0.0)	0 (0.0)	1 (0.1)
PRIMPERAN	1 (0.5)	0 (0.0)	0 (0.0)	0 (0.0)	1 (0.1)
PROPECIA	0 (0.0)	0 (0.0)	0 (0.0)	1 (0.5)	1 (0.1)
PULMICORT BUDESONID	0 (0.0)	0 (0.0)	1 (0.6)	0 (0.0)	1 (0.1)
RATIOPHARM NASENSPRAY	0 (0.0)	0 (0.0)	1 (0.6)	0 (0.0)	1 (0.1)
REGAINE	1 (0.5)	0 (0.0)	0 (0.0)	0 (0.0)	1 (0.1)
REISEGOLD TABS (DIMENHYDRINAT)	0 (0.0)	1 (0.6)	0 (0.0)	0 (0.0)	1 (0.1)

A subject with more than one entry on the same drug was only counted once
Percentages based on total no. of subjects in each group
Table is sorted by total frequency

Prior and concomitant medication by brand name, Safety dataset

Brand name	Group 1 (MM) (N=183)	Group 2 (MP) (N=181)	Group 3 (PP) (N=181)	Group 4 (M-) (N=200)	Total (N=745)
RENNIE	1 (0.5)	0 (0.0)	0 (0.0)	0 (0.0)	1 (0.1)
REPARIL	1 (0.5)	0 (0.0)	0 (0.0)	0 (0.0)	1 (0.1)
RIFUN	0 (0.0)	0 (0.0)	1 (0.6)	0 (0.0)	1 (0.1)
RIOPAN GEL	0 (0.0)	0 (0.0)	0 (0.0)	1 (0.5)	1 (0.1)
ROXITHROMYCIN (ROXYHEXAL)	0 (0.0)	1 (0.6)	0 (0.0)	0 (0.0)	1 (0.1)
ROXITHROMYCIN RATIOPHARM 300	1 (0.5)	0 (0.0)	0 (0.0)	0 (0.0)	1 (0.1)
SALBUTAMOL	1 (0.5)	0 (0.0)	0 (0.0)	0 (0.0)	1 (0.1)
SALOFALK	0 (0.0)	0 (0.0)	1 (0.6)	0 (0.0)	1 (0.1)
SALVE MIXTURE	0 (0.0)	0 (0.0)	0 (0.0)	1 (0.5)	1 (0.1)
SANDOMIGRAN	0 (0.0)	0 (0.0)	1 (0.6)	0 (0.0)	1 (0.1)
SCANDICAIN 1%	1 (0.5)	0 (0.0)	0 (0.0)	0 (0.0)	1 (0.1)
SEDARISTON	0 (0.0)	0 (0.0)	0 (0.0)	1 (0.5)	1 (0.1)
SERTRALIN	0 (0.0)	0 (0.0)	0 (0.0)	1 (0.5)	1 (0.1)
SIMVASTATIN	0 (0.0)	1 (0.6)	0 (0.0)	0 (0.0)	1 (0.1)
SOBELIN 300	0 (0.0)	0 (0.0)	1 (0.6)	0 (0.0)	1 (0.1)
SOLVEX	0 (0.0)	0 (0.0)	0 (0.0)	1 (0.5)	1 (0.1)
SOSTRIL	0 (0.0)	0 (0.0)	1 (0.6)	0 (0.0)	1 (0.1)
SPALT LIQUA FORTE	0 (0.0)	0 (0.0)	0 (0.0)	1 (0.5)	1 (0.1)
SPIRALE	0 (0.0)	0 (0.0)	1 (0.6)	0 (0.0)	1 (0.1)
STALORAL	0 (0.0)	0 (0.0)	1 (0.6)	0 (0.0)	1 (0.1)
SUFENTANIL	0 (0.0)	0 (0.0)	1 (0.6)	0 (0.0)	1 (0.1)
SULFASALAZIN	1 (0.5)	0 (0.0)	0 (0.0)	0 (0.0)	1 (0.1)
TD-IMPFSTOFF MÉRIEUX	0 (0.0)	0 (0.0)	0 (0.0)	1 (0.5)	1 (0.1)
TELFAST 120	1 (0.5)	0 (0.0)	0 (0.0)	0 (0.0)	1 (0.1)
TETANOL	1 (0.5)	0 (0.0)	0 (0.0)	0 (0.0)	1 (0.1)
TETANOL PUR	1 (0.5)	0 (0.0)	0 (0.0)	0 (0.0)	1 (0.1)
TETRACEPAM	1 (0.5)	0 (0.0)	0 (0.0)	0 (0.0)	1 (0.1)
THEOPYLLIN	0 (0.0)	0 (0.0)	1 (0.6)	0 (0.0)	1 (0.1)

A subject with more than one entry on the same drug was only counted once
Percentages based on total no. of subjects in each group
Table is sorted by total frequency

Prior and concomitant medication by brand name, Safety dataset

Brand name	Group 1 (MM) (N=183)	Group 2 (MP) (N=181)	Group 3 (PP) (N=181)	Group 4 (M-) (N=200)	Total (N=745)
THYRANOJOD	0 (0.0)	0 (0.0)	0 (0.0)	1 (0.5)	1 (0.1)
TOGAL (IBUPROFEN)	0 (0.0)	0 (0.0)	0 (0.0)	1 (0.5)	1 (0.1)
TRAMADOL	0 (0.0)	0 (0.0)	0 (0.0)	1 (0.5)	1 (0.1)
TRAMAL	0 (0.0)	0 (0.0)	0 (0.0)	1 (0.5)	1 (0.1)
TREVILOR	1 (0.5)	0 (0.0)	0 (0.0)	0 (0.0)	1 (0.1)
TRIAMGALEN	1 (0.5)	0 (0.0)	0 (0.0)	0 (0.0)	1 (0.1)
TRIAMGALEN CREAM	1 (0.5)	0 (0.0)	0 (0.0)	0 (0.0)	1 (0.1)
TRIAMGALEN CREME	0 (0.0)	0 (0.0)	1 (0.6)	0 (0.0)	1 (0.1)
TRIETTE	0 (0.0)	1 (0.6)	0 (0.0)	0 (0.0)	1 (0.1)
TRIPTAN	0 (0.0)	0 (0.0)	0 (0.0)	1 (0.5)	1 (0.1)
TRISTEP	0 (0.0)	0 (0.0)	1 (0.6)	0 (0.0)	1 (0.1)
ULTRACAIN	0 (0.0)	1 (0.6)	0 (0.0)	0 (0.0)	1 (0.1)
ULTRACAIN / D-S FORTE	0 (0.0)	1 (0.6)	0 (0.0)	0 (0.0)	1 (0.1)
ULTRACAIN DS	0 (0.0)	1 (0.6)	0 (0.0)	0 (0.0)	1 (0.1)
ULTRACAIN DS FORTE	0 (0.0)	0 (0.0)	0 (0.0)	1 (0.5)	1 (0.1)
URO-TARIVID	0 (0.0)	0 (0.0)	1 (0.6)	0 (0.0)	1 (0.1)
VASOMOTAL	1 (0.5)	0 (0.0)	0 (0.0)	0 (0.0)	1 (0.1)
VENLAFAXIN	0 (0.0)	0 (0.0)	1 (0.6)	0 (0.0)	1 (0.1)
VERRUMAL	0 (0.0)	1 (0.6)	0 (0.0)	0 (0.0)	1 (0.1)
VERTIGOHEEL	0 (0.0)	1 (0.6)	0 (0.0)	0 (0.0)	1 (0.1)
VIANI 5 MIKROGRAMM DISKUS	0 (0.0)	0 (0.0)	1 (0.6)	0 (0.0)	1 (0.1)
VIDISIC	0 (0.0)	1 (0.6)	0 (0.0)	0 (0.0)	1 (0.1)
VITA C ASCOPHARM	0 (0.0)	0 (0.0)	0 (0.0)	1 (0.5)	1 (0.1)
VIVIMED MIGRÄNE	0 (0.0)	1 (0.6)	0 (0.0)	0 (0.0)	1 (0.1)
VIVINOX STARK	0 (0.0)	0 (0.0)	1 (0.6)	0 (0.0)	1 (0.1)
VOLON A TINKTUR	1 (0.5)	0 (0.0)	0 (0.0)	0 (0.0)	1 (0.1)
VOLTAREN EMUGEL	0 (0.0)	0 (0.0)	1 (0.6)	0 (0.0)	1 (0.1)
VOMEX	0 (0.0)	1 (0.6)	0 (0.0)	0 (0.0)	1 (0.1)

A subject with more than one entry on the same drug was only counted once
Percentages based on total no. of subjects in each group
Table is sorted by total frequency

Prior and concomitant medication by brand name, Safety dataset

Brand name	Group 1 (MM) (N=183)	Group 2 (MP) (N=181)	Group 3 (PP) (N=181)	Group 4 (M-) (N=200)	Total (N=745)
VOMEX A	1 (0.5)	0 (0.0)	0 (0.0)	0 (0.0)	1 (0.1)
VOMEX A SUPP.	0 (0.0)	1 (0.6)	0 (0.0)	0 (0.0)	1 (0.1)
WICK DAY MED	1 (0.5)	0 (0.0)	0 (0.0)	0 (0.0)	1 (0.1)
WICK MEDINIGHT	0 (0.0)	0 (0.0)	1 (0.6)	0 (0.0)	1 (0.1)
XICLAV	1 (0.5)	0 (0.0)	0 (0.0)	0 (0.0)	1 (0.1)
XUSAL	0 (0.0)	0 (0.0)	0 (0.0)	1 (0.5)	1 (0.1)
XUSAL LEVOCETIRIZIN	0 (0.0)	0 (0.0)	1 (0.6)	0 (0.0)	1 (0.1)
XYLONEST 3%	0 (0.0)	0 (0.0)	0 (0.0)	1 (0.5)	1 (0.1)
ZOVIRAX	0 (0.0)	1 (0.6)	0 (0.0)	0 (0.0)	1 (0.1)
ZYCLOVIRAN	0 (0.0)	1 (0.6)	0 (0.0)	0 (0.0)	1 (0.1)
ZYRTEC	0 (0.0)	0 (0.0)	1 (0.6)	0 (0.0)	1 (0.1)
ÎMPLANON	0 (0.0)	1 (0.6)	0 (0.0)	0 (0.0)	1 (0.1)

A subject with more than one entry on the same drug was only counted once
Percentages based on total no. of subjects in each group
Table is sorted by total frequency

14.7 ADDITIONAL EXPLORATORY SUBGROUP ANALYSIS BY AGE GROUP AND GENDER

14.7.1 Distribution of age and gender

14.7.1.1 Distribution of age - FA dataset

		Number of Patients (%) / Descriptive Statistics				
		Group 1 (MM) (N=183)	Group 2 (MP) (N=181)	Group 3 (PP) (N=181)	Group 4 (M-) (N=200)	p-value
agegrp						
	18 - 25 yrs	116 (63.4)	102 (56.4)	92 (50.8)	4 (2.0)	<0.001~
	26 - 35 yrs	59 (32.2)	73 (40.3)	81 (44.8)	36 (18.0)	
	36 - 45 yrs	7 (3.8)	6 (3.3)	6 (3.3)	88 (44.0)	
	46 - 55 yrs	1 (0.5)	0 (0.0)	2 (1.1)	72 (36.0)	

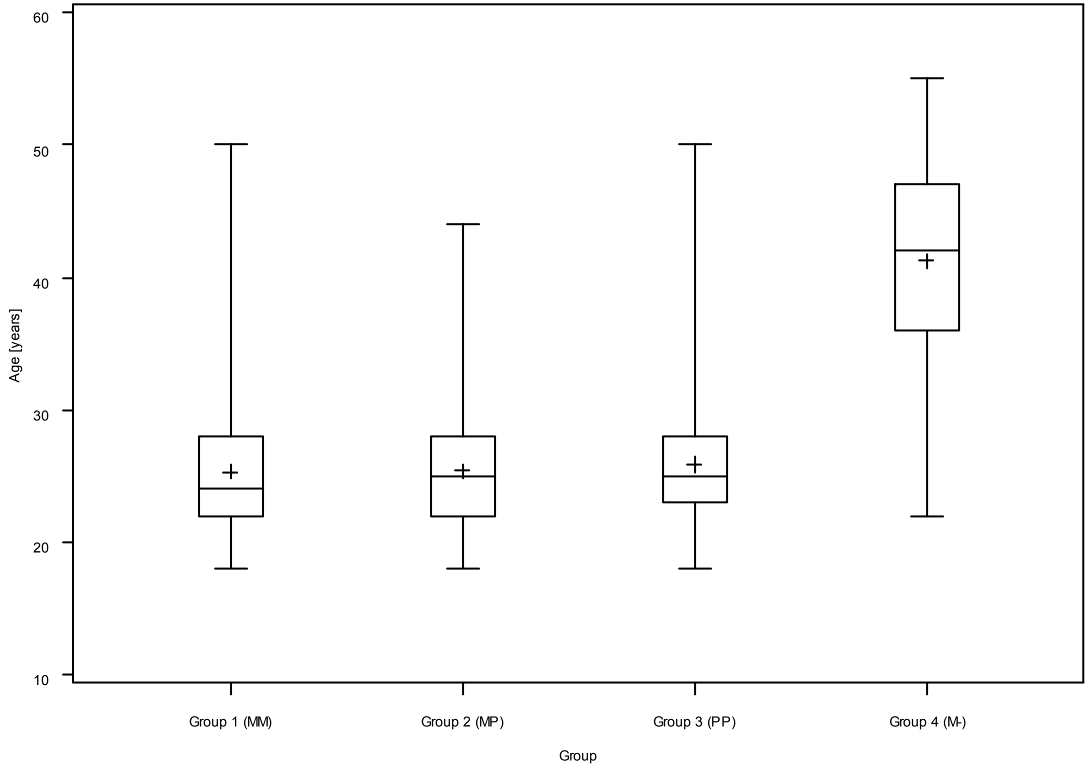
60
50
40
30
20
10
Age [years]
Group 1 (MM)
Group 2 (MP)
Group 3 (PP)
Group 4 (M-)
Group

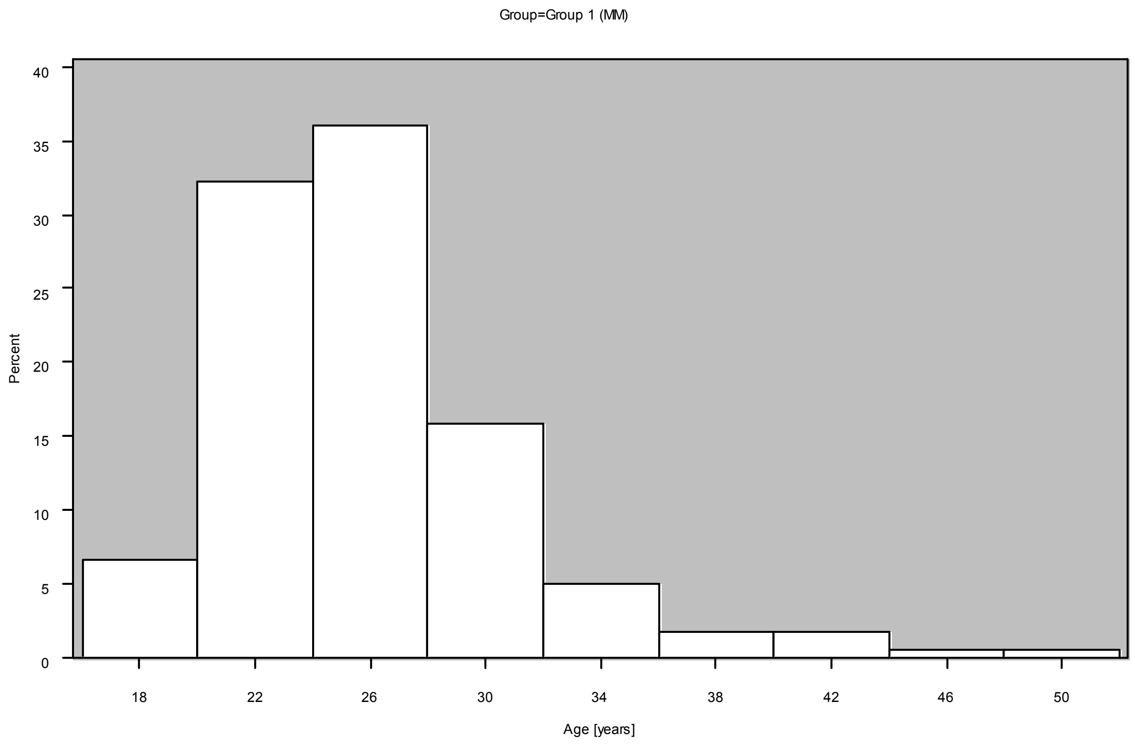
Group=Group 1 (MM)
Percent
40
35
30
25
20
15
10
5
0
18
22
26
30
34
38
42
46
50
Age [years]

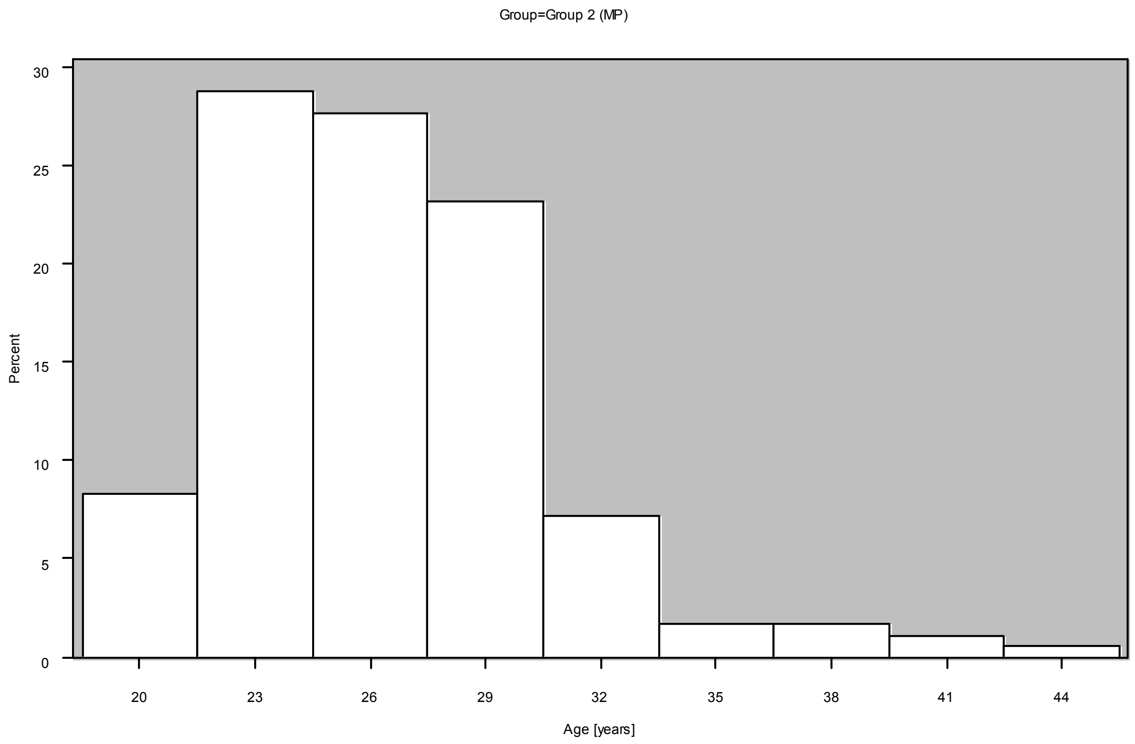
Group=Group 2 (MP)
Percent
0
5
10
15
20
25
30
20
23
26
29
32
35
38
41
44
Age [years]

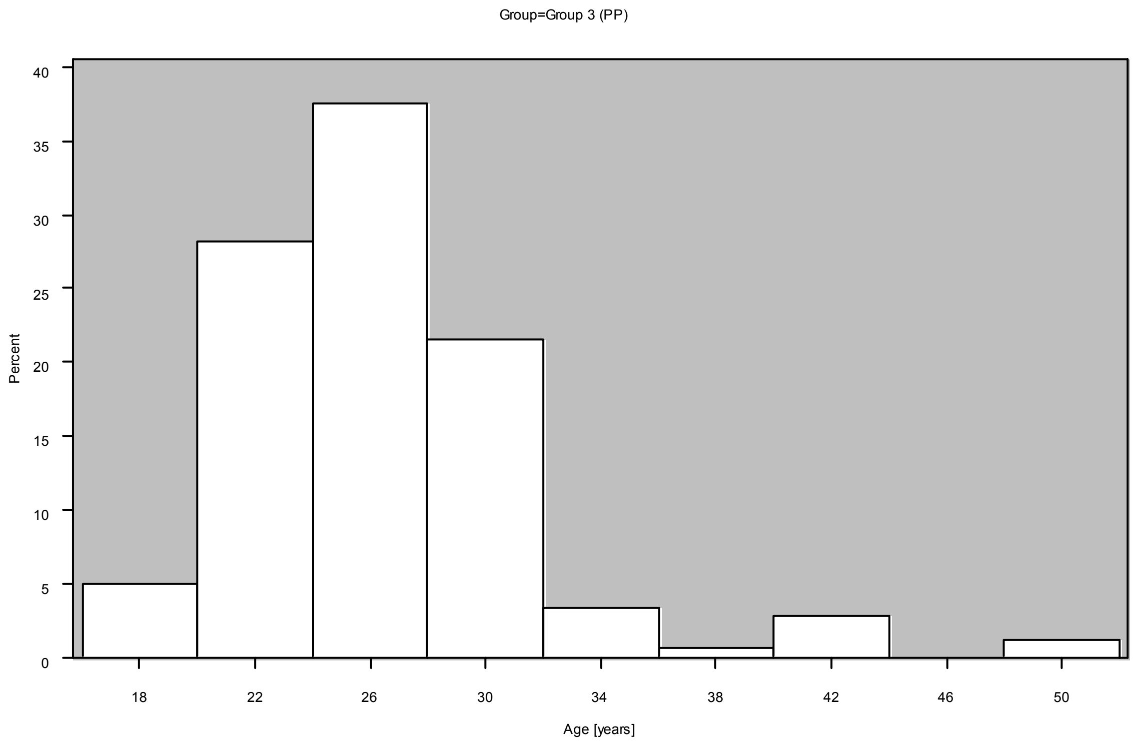
Group=Group 3 (PP)
Percent
0
5
10
15
20
25
30
35
40
18
22
26
30
34
38
42
46
50
Age [years]

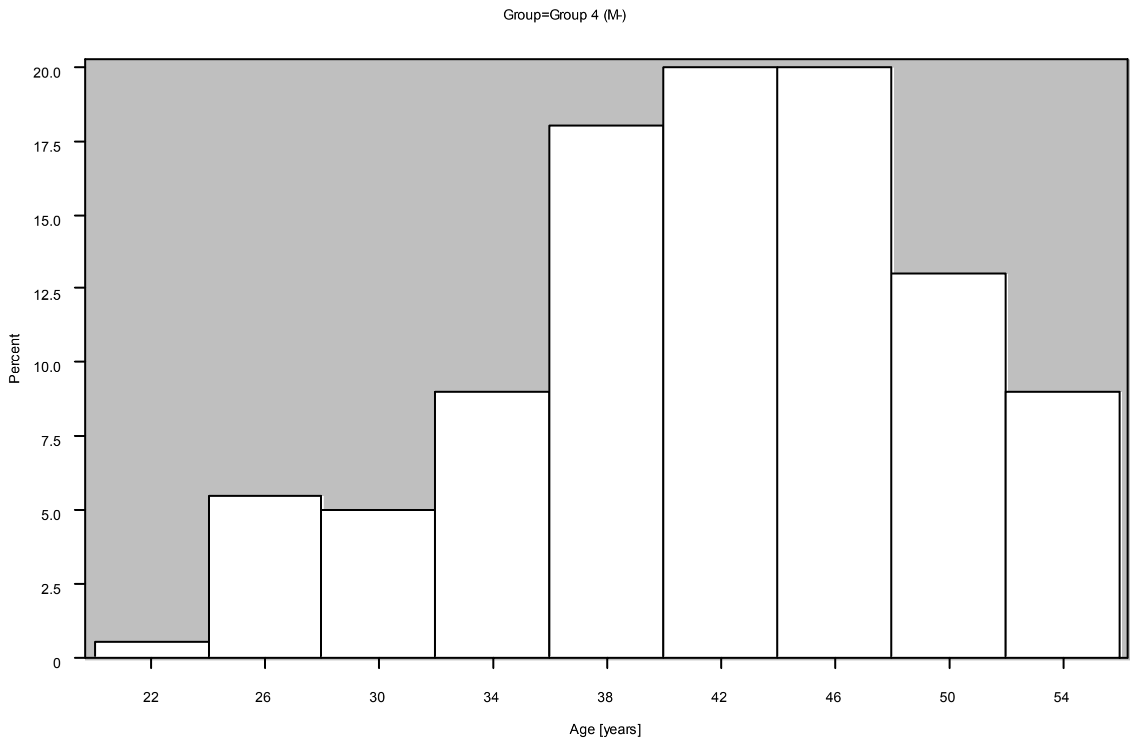
Group=Group 4 (M-)
Percent
20.0
17.5
15.0
12.5
10.0
7.5
5.0
2.5
0
22
26
30
34
38
42
46
50
54
Age [years]

14.7.1.2 Distribution of gender - FA dataset

		Number of Patients (%) / Descriptive Statistics				
		Group 1 (MM) (N=183)	Group 2 (MP) (N=181)	Group 3 (PP) (N=181)	Group 4 (M-) (N=200)	p-value
Gender						
	male	86 (47.0)	69 (38.1)	74 (40.9)	85 (42.5)	0.377~
	female	97 (53.0)	112 (61.9)	107 (59.1)	115 (57.5)	

14.7.2 Antibodies against vaccinia, seroconversion rates

14.7.2.1 Tables

14.7.2.1.1 Antibodies against vaccinia, seroconversion rates, responders by gender - FA dataset

Group=Group 1 (MM)

		Number of Patients (%) / Descriptive Statistics male (N=86)	female (N=97)	p-value
V2				
	yes	59 (68.6)	70 (72.9)	0.681
	no	27 (31.4)	26 (27.1)	
V3				
	yes	75 (89.3)	85 (88.5)	0.855
	no	9 (10.7)	11 (11.5)	
V4				
	yes	81 (98.8)	93 (98.9)	0.854
	no	1 (1.2)	1 (1.1)	
V5				
	yes	83 (98.8)	93 (98.9)	>0.999
	no	1 (1.2)	1 (1.1)	

Seroconversion is defined as the appearance of antibody titers >= 1:50 in a vaccinia specific IgG ELISA for initially seronegative subjects or twofold increase of the antibody titer compared to the pre-existing baseline titer for subjects with a pre-existing antibody titer in the ELISA.
p-value: exact test of Fisher

Antibodies against vaccinia, seroconversion rates, responders by gender - FA dataset

Group=Group 2 (MP)

		Number of Patients (%) / Descriptive Statistics male (N=69)	female (N=112)	p-value
V2				
	yes	50 (74.6)	80 (71.4)	0.191
	no	17 (25.4)	32 (28.6)	
V3				
	yes	57 (85.1)	95 (88.8)	0.708
	no	10 (14.9)	12 (11.2)	
V4				
	yes	53 (79.1)	90 (84.1)	0.668
	no	14 (20.9)	17 (15.9)	
V5				
	yes	50 (74.6)	76 (70.4)	0.872
	no	17 (25.4)	32 (29.6)	

Seroconversion is defined as the appearance of antibody titers >= 1:50 in a vaccinia specific IgG ELISA for initially seronegative subjects or twofold increase of the antibody titer compared to the pre-existing baseline titer for subjects with a pre-existing antibody titer in the ELISA.
p-value: exact test of Fisher

Antibodies against vaccinia, seroconversion rates, responders by gender - FA dataset

Group=Group 3 (PP)

		Number of Patients (%) / Descriptive Statistics male (N=74)	female (N=107)	p-value
V2				
	yes	1 (1.4)	4 (3.8)	0.533
	no	71 (98.6)	102 (96.2)	
V3				
	yes	2 (2.7)	3 (2.9)	0.499
	no	71 (97.3)	99 (97.1)	
V4				
	yes	1 (1.4)	5 (4.9)	0.261
	no	72 (98.6)	97 (95.1)	
V5				
	yes	3 (4.1)	2 (1.9)	0.500
	no	70 (95.9)	102 (98.1)	

Seroconversion is defined as the appearance of antibody titers >= 1:50 in a vaccinia specific IgG ELISA for initially seronegative subjects or twofold increase of the antibody titer compared to the pre-existing baseline titer for subjects with a pre-existing antibody titer in the ELISA.
p-value: exact test of Fisher

Antibodies against vaccinia, seroconversion rates, responders by gender - FA dataset

Group=Group 4 (M-)

		Number of Patients (%) / Descriptive Statistics male (N=85)	female (N=115)	p-value
V2				
	yes	83 (97.6)	108 (93.9)	0.306
	no	2 (2.4)	7 (6.1)	
V3				
	yes	80 (95.2)	105 (91.3)	0.262
	no	4 (4.8)	10 (8.7)	
V4				
		0 (0.0)	0 (0.0)	n/a
V5				
		0 (0.0)	0 (0.0)	n/a

Seroconversion is defined as the appearance of antibody titers >= 1:50 in a vaccinia specific IgG ELISA for initially seronegative subjects or twofold increase of the antibody titer compared to the pre-existing baseline titer for subjects with a pre-existing antibody titer in the ELISA.
p-value: exact test of Fisher

14.7.2.1.2 Antibodies against vaccinia, seroconversion rates, responders by gender - PP dataset

Group=Group 1 (MM)

		Number of Patients (%) / Descriptive Statistics		
		male (N=78)	female (N=90)	p-value
V2				
	yes	53 (67.9)	64 (71.1)	0.737
	no	25 (32.1)	26 (28.9)	
V3				
	yes	70 (89.7)	79 (87.8)	0.809
	no	8 (10.3)	11 (12.2)	
V4				
	yes	77 (98.7)	89 (98.9)	>0.999
	no	1 (1.3)	1 (1.1)	
V5				
	yes	77 (98.7)	89 (98.9)	>0.999
	no	1 (1.3)	1 (1.1)	

Seroconversion is defined as the appearance of antibody titers >= 1:50 in a vaccinia specific IgG ELISA for initially seronegative subjects or twofold increase of the antibody titer compared to the pre-existing baseline titer for subjects with a pre-existing antibody titer in the ELISA.
p-value: exact test of Fisher

Antibodies against vaccinia, seroconversion rates, responders by gender - PP dataset

Group=Group 2 (MP)

		Number of Patients (%) / Descriptive Statistics: male (N=67)	female (N=103)	p-value
V2				
	yes	50 (74.6)	73 (70.9)	0.726
	no	17 (25.4)	30 (29.1)	
V3				
	yes	57 (85.1)	91 (88.3)	0.641
	no	10 (14.9)	12 (11.7)	
V4				
	yes	53 (79.1)	86 (83.5)	0.543
	no	14 (20.9)	17 (16.5)	
V5				
	yes	50 (74.6)	72 (69.9)	0.602
	no	17 (25.4)	31 (30.1)	

Seroconversion is defined as the appearance of antibody titers >= 1:50 in a vaccinia specific IgG ELISA for initially seronegative subjects or twofold increase of the antibody titer compared to the pre-existing baseline titer for subjects with a pre-existing antibody titer in the ELISA.
p-value: exact test of Fisher

Antibodies against vaccinia, seroconversion rates, responders by gender - PP dataset

Group=Group 3 (PP)

		Number of Patients (%) / Descriptive Statistics male (N=70)	female (N=94)	p-value
V2				
	yes	1 (1.4)	4 (4.3)	0.264
	no	68 (98.6)	90 (95.7)	
V3				
	yes	2 (2.9)	3 (3.2)	>0.999
	no	68 (97.1)	91 (96.8)	
V4				
	yes	1 (1.4)	5 (5.3)	0.241
	no	69 (98.6)	89 (94.7)	
V5				
	yes	3 (4.3)	2 (2.1)	0.652
	no	67 (95.7)	92 (97.9)	

Seroconversion is defined as the appearance of antibody titers >= 1:50 in a vaccinia specific IgG ELISA for initially seronegative subjects or twofold increase of the antibody titer compared to the pre-existing baseline titer for subjects with a pre-existing antibody titer in the ELISA.
p-value: exact test of Fisher

Antibodies against vaccinia, seroconversion rates, responders by gender - PP dataset

Group=Group 4 (M-)

		Number of Patients (%) / Descriptive Statistics male (N=85)	female (N=108)	p-value
V2				
	yes	83 (97.6)	103 (95.4)	0.468
	no	2 (2.4)	5 (4.6)	
V3				
	yes	80 (95.2)	98 (90.7)	0.212
	no	4 (4.8)	10 (9.3)	
V4				
		0 (0.0)	0 (0.0)	n/a
V5				
		0 (0.0)	0 (0.0)	n/a

Seroconversion is defined as the appearance of antibody titers >= 1:50 in a vaccinia specific IgG ELISA for initially seronegative subjects or twofold increase of the antibody titer compared to the pre-existing baseline titer for subjects with a pre-existing antibody titer in the ELISA.
p-value: exact test of Fisher

Antibodies against vaccinia, seroconversion rates, responders by age group - FA dataset

Group=Group 1 (MM)

		Number of Patients (%) / Descriptive Statistics				
		18 - 25 yrs (N=116)	26 - 35 yrs (N=59)	36 - 45 yrs (N=7)	46 - 55 yrs (N=1)	p-value
V2						
	yes	82 (70.7)	41 (70.7)	6 (85.7)	0 (0.0)	0.331
	no	34 (29.3)	17 (29.3)	1 (14.3)	1 (100.0)	
V3						
	yes	103 (90.4)	50 (86.2)	7 (100.0)	0 (0.0)	0.253
	no	11 (9.6)	8 (13.8)	0 (0.0)	1 (100.0)	
V4						
	yes	110 (98.2)	56 (100.0)	7 (100.0)	1 (100.0)	0.739
	no	2 (1.8)	0 (0.0)	0 (0.0)	0 (0.0)	
V5						
	yes	113 (99.1)	56 (100.0)	6 (85.7)	1 (100.0)	0.133
	no	1 (0.9)	0 (0.0)	1 (14.3)	0 (0.0)	

Seroconversion is defined as the appearance of antibody titers >= 1:50 in a vaccinia specific IgG ELISA for initially seronegative subjects or twofold increase of the antibody titer compared to the pre-existing baseline titer for subjects with a pre-existing antibody titer in the ELISA.
p-value: exact test of Fisher

14.7.2.1.3 Antibodies against vaccinia, seroconversion rates, responders by age group - FA dataset

Group=Group 2 (MP)

		Number of Patients (%) / Descriptive Statistics: 18 - 25 yrs (N=102)	26 - 35 yrs (N=73)	36 - 45 yrs (N=6)	p-value
V2					
	yes	75 (75.0)	50 (68.5)	5 (83.3)	0.606
	no	25 (25.0)	23 (31.5)	1 (16.7)	
V3					
	yes	88 (90.7)	59 (81.9)	5 (100.0)	0.106
	no	9 (9.3)	13 (18.1)	0 (0.0)	
V4					
	yes	82 (84.5)	57 (79.2)	4 (80.0)	0.217
	no	15 (15.5)	15 (20.8)	1 (20.0)	
V5					
	yes	73 (74.5)	47 (66.2)	6 (100.0)	0.407
	no	25 (25.5)	24 (33.8)	0 (0.0)	

Seroconversion is defined as the appearance of antibody titers >= 1:50 in a vaccinia specific IgG ELISA for initially seronegative subjects or twofold increase of the antibody titer compared to the pre-existing baseline titer for subjects with a pre-existing antibody titer in the ELISA.
p-value: exact test of Fisher

Antibodies against vaccinia, seroconversion rates, responders by age group - FA dataset

Group=Group 3 (PP)

		Number of Patients (%) / Descriptive Statistics				
		18 - 25 yrs (N=92)	26 - 35 yrs (N=81)	36 - 45 yrs (N=6)	46 - 55 yrs (N=2)	p-value
V2						
	yes	3 (3.3)	2 (2.6)	0 (0.0)	0 (0.0)	0.448
	no	89 (96.7)	76 (97.4)	6 (100.0)	2 (100.0)	
V3						
	yes	2 (2.2)	3 (3.8)	0 (0.0)	0 (0.0)	0.423
	no	88 (97.8)	75 (96.2)	5 (100.0)	2 (100.0)	
V4						
	yes	3 (3.3)	2 (2.6)	1 (20.0)	0 (0.0)	0.174
	no	87 (96.7)	76 (97.4)	4 (80.0)	2 (100.0)	
V5						
	yes	3 (3.3)	2 (2.6)	0 (0.0)	0 (0.0)	0.727
	no	88 (96.7)	76 (97.4)	6 (100.0)	2 (100.0)	

Seroconversion is defined as the appearance of antibody titers >= 1:50 in a vaccinia specific IgG ELISA for initially seronegative subjects or twofold increase of the antibody titer compared to the pre-existing baseline titer for subjects with a pre-existing antibody titer in the ELISA.
p-value: exact test of Fisher

Antibodies against vaccinia, seroconversion rates, responders by age group - FA dataset

Group=Group 4 (M-)

		Number of Patients (%) / Descriptive Statistics				
		18 - 25 yrs (N=4)	26 - 35 yrs (N=36)	36 - 45 yrs (N=88)	46 - 55 yrs (N=72)	p-value
V2						
	yes	3 (75.0)	32 (88.9)	87 (98.9)	69 (95.8)	0.021
	no	1 (25.0)	4 (11.1)	1 (1.1)	3 (4.2)	
V3						
	yes	4 (100.0)	32 (88.9)	85 (97.7)	64 (88.9)	0.118
	no	0 (0.0)	4 (11.1)	2 (2.3)	8 (11.1)	
V4						
		0 (0.0)	0 (0.0)	0 (0.0)	0 (0.0)	n/a
V5						
		0 (0.0)	0 (0.0)	0 (0.0)	0 (0.0)	n/a

Seroconversion is defined as the appearance of antibody titers >= 1:50 in a vaccinia specific IgG ELISA for initially seronegative subjects or twofold increase of the antibody titer compared to the pre-existing baseline titer for subjects with a pre-existing antibody titer in the ELISA.
p-value: exact test of Fisher

14.7.2.1.4 Antibodies against vaccinia, seroconversion rates, responders by age group - PP dataset

Group=Group 1 (MM)

		Number of Patients (%) / Descriptive Statistics 18 - 25 yrs (N=107)	26 - 35 yrs (N=53)	36 - 45 yrs (N=7)	46 - 55 yrs (N=1)	p-value
V2						
	yes	74 (69.2)	37 (69.8)	6 (85.7)	0 (0.0)	0.493
	no	33 (30.8)	16 (30.2)	1 (14.3)	1 (100.0)	
V3						
	yes	97 (90.7)	45 (84.9)	7 (100.0)	0 (0.0)	0.101
	no	10 (9.3)	8 (15.1)	0 (0.0)	1 (100.0)	
V4						
	yes	105 (98.1)	53 (100.0)	7 (100.0)	1 (100.0)	>0.999
	no	2 (1.9)	0 (0.0)	0 (0.0)	0 (0.0)	
V5						
	yes	106 (99.1)	53 (100.0)	6 (85.7)	1 (100.0)	0.093
	no	1 (0.9)	0 (0.0)	1 (14.3)	0 (0.0)	

Seroconversion is defined as the appearance of antibody titers >= 1:50 in a vaccinia specific IgG ELISA for initially seronegative subjects or twofold increase of the antibody titer compared to the pre-existing baseline titer for subjects with a pre-existing antibody titer in the ELISA.
p-value: exact test of Fisher

Antibodies against vaccinia, seroconversion rates, responders by age group - PP dataset

Group=Group 2 (MP)

		Number of Patients (%) / Descriptive Statistics 18 - 25 yrs (N=96)	26 - 35 yrs (N=69)	36 - 45 yrs (N=5)	p-value
V2					
	yes	72 (75.0)	47 (68.1)	4 (80.0)	0.611
	no	24 (25.0)	22 (31.9)	1 (20.0)	
V3					
	yes	87 (90.6)	56 (81.2)	5 (100.0)	0.193
	no	9 (9.4)	13 (18.8)	0 (0.0)	
V4					
	yes	81 (84.4)	54 (78.3)	4 (80.0)	0.509
	no	15 (15.6)	15 (21.7)	1 (20.0)	
V5					
	yes	72 (75.0)	45 (65.2)	5 (100.0)	0.162
	no	24 (25.0)	24 (34.8)	0 (0.0)	

Seroconversion is defined as the appearance of antibody titers >= 1:50 in a vaccinia specific IgG ELISA for initially seronegative subjects or twofold increase of the antibody titer compared to the pre-existing baseline titer for subjects with a pre-existing antibody titer in the ELISA.
p-value: exact test of Fisher

Antibodies against vaccinia, seroconversion rates, responders by age group - PP dataset

Group=Group 3 (PP)

		Number of Patients (%) / Descriptive Statistics				
		18 - 25 yrs (N=84)	26 - 35 yrs (N=73)	36 - 45 yrs (N=5)	46 - 55 yrs (N=2)	p-value
V2						
	yes	3 (3.6)	2 (2.8)	0 (0.0)	0 (0.0)	0.863
	no	81 (96.4)	70 (97.2)	5 (100.0)	2 (100.0)	
V3						
	yes	2 (2.4)	3 (4.1)	0 (0.0)	0 (0.0)	0.731
	no	82 (97.6)	70 (95.9)	5 (100.0)	2 (100.0)	
V4						
	yes	3 (3.6)	2 (2.7)	1 (20.0)	0 (0.0)	0.257
	no	81 (96.4)	71 (97.3)	4 (80.0)	2 (100.0)	
V5						
	yes	3 (3.6)	2 (2.7)	0 (0.0)	0 (0.0)	>0.999
	no	81 (96.4)	71 (97.3)	5 (100.0)	2 (100.0)	

Seroconversion is defined as the appearance of antibody titers >= 1:50 in a vaccinia specific IgG ELISA for initially seronegative subjects or twofold increase of the antibody titer compared to the pre-existing baseline titer for subjects with a pre-existing antibody titer in the ELISA.
p-value: exact test of Fisher

Antibodies against vaccinia, seroconversion rates, responders by age group - PP dataset

Group=Group 4 (M-)

		Number of Patients (%) / Descriptive Statistics				
		18 - 25 yrs (N=2)	26 - 35 yrs (N=32)	36 - 45 yrs (N=87)	46 - 55 yrs (N=72)	p-value
V2						
	yes	2 (100.0)	29 (90.6)	86 (98.9)	69 (95.8)	0.148
	no	0 (0.0)	3 (9.4)	1 (1.1)	3 (4.2)	
V3						
	yes	2 (100.0)	28 (87.5)	84 (97.7)	64 (88.9)	0.095
	no	0 (0.0)	4 (12.5)	2 (2.3)	8 (11.1)	
V4						
		0 (0.0)	0 (0.0)	0 (0.0)	0 (0.0)	n/a
V5						
		0 (0.0)	0 (0.0)	0 (0.0)	0 (0.0)	n/a

Seroconversion is defined as the appearance of antibody titers >= 1:50 in a vaccinia specific IgG ELISA for initially seronegative subjects or twofold increase of the antibody titer compared to the pre-existing baseline titer for subjects with a pre-existing antibody titer in the ELISA.
p-value: exact test of Fisher

14.7.2.2 Figures

14.7.2.2.1 Age group

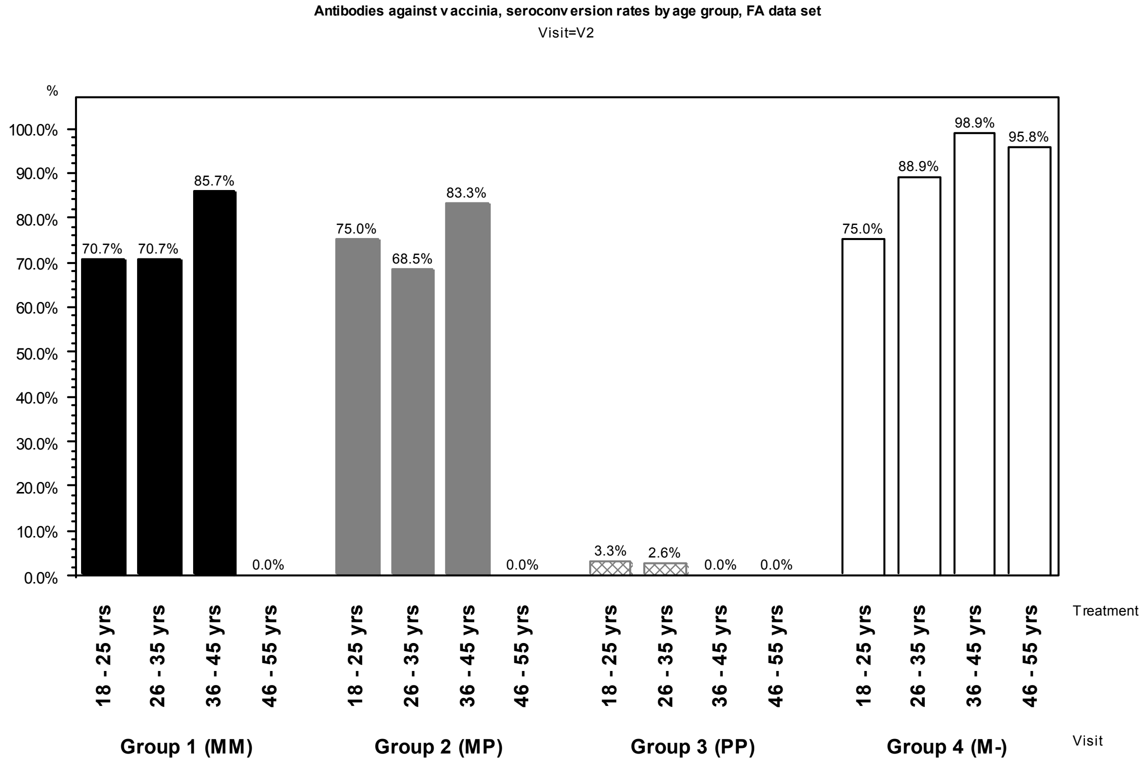

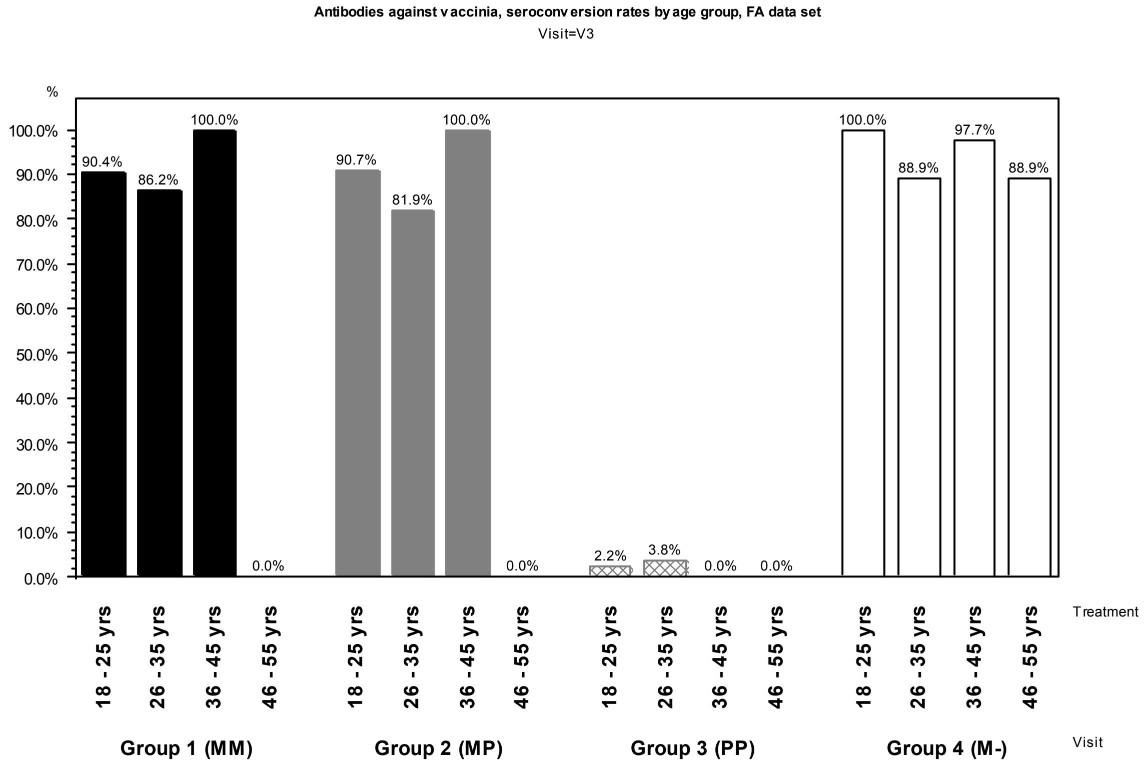
Antibodies against vaccinia, seroconversion rates by age group, FA data set
Visit=V3
%
100.0%
90.0%
80.0%
70.0%
60.0%
50.0%
40.0%
30.0%
20.0%
10.0%
0.0%
90.4%
86.2%
100.0%
0.0%
90.7%
81.9%
100.0%
0.0%
2.2%
3.8%
0.0%
0.0%
100.0%
88.9%
97.7%
88.9%
18 - 25 yrs
26 - 35 yrs
36 - 45 yrs
46 - 55 yrs
Group 1 (MM)
Group 2 (MP)
Group 3 (PP)
Group 4 (M-)
Treatment
Visit

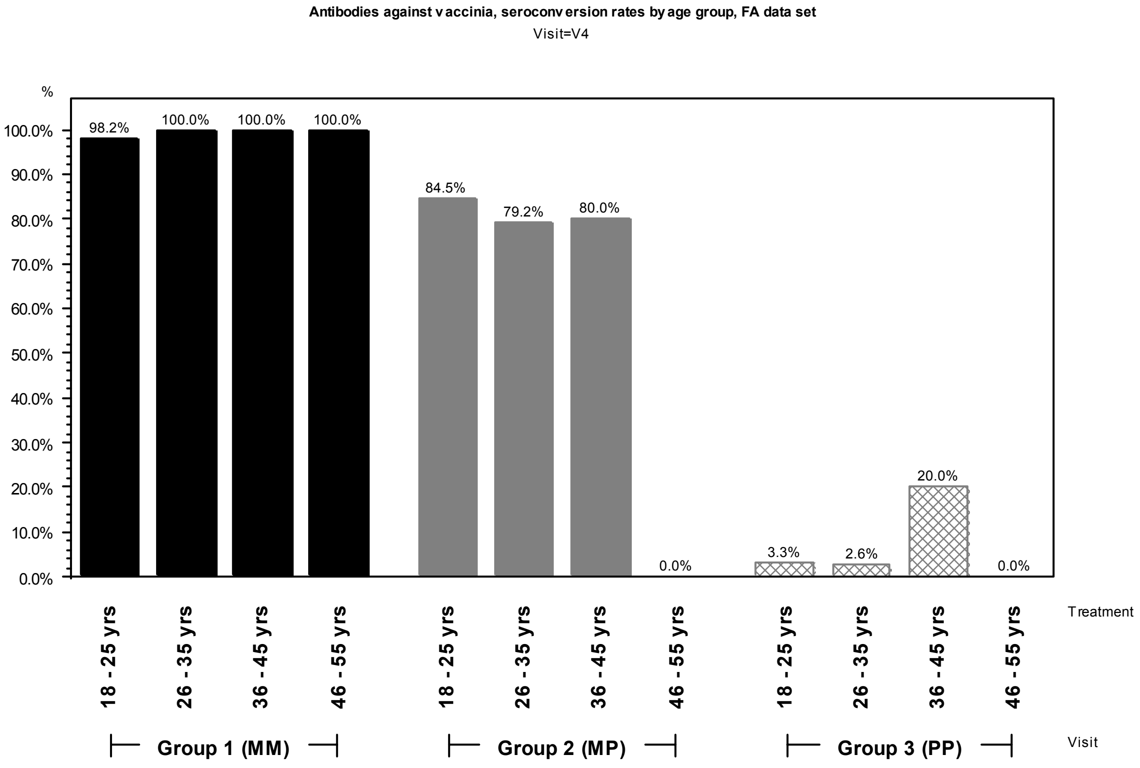

Antibodies against vaccinia, seroconversion rates by age group, FA data set
Visit=V4
%
100.0%
90.0%
80.0%
70.0%
60.0%
50.0%
40.0%
30.0%
20.0%
10.0%
0.0%
98.2%
100.0%
100.0%
100.0%
84.5%
79.2%
80.0%
0.0%
3.3%
2.6%
20.0%
0.0%
18 - 25 yrs
26 - 35 yrs
36 - 45 yrs
46 - 55 yrs
Treatment
Group 1 (MM)
Group 2 (MP)
Group 3 (PP)
Visit

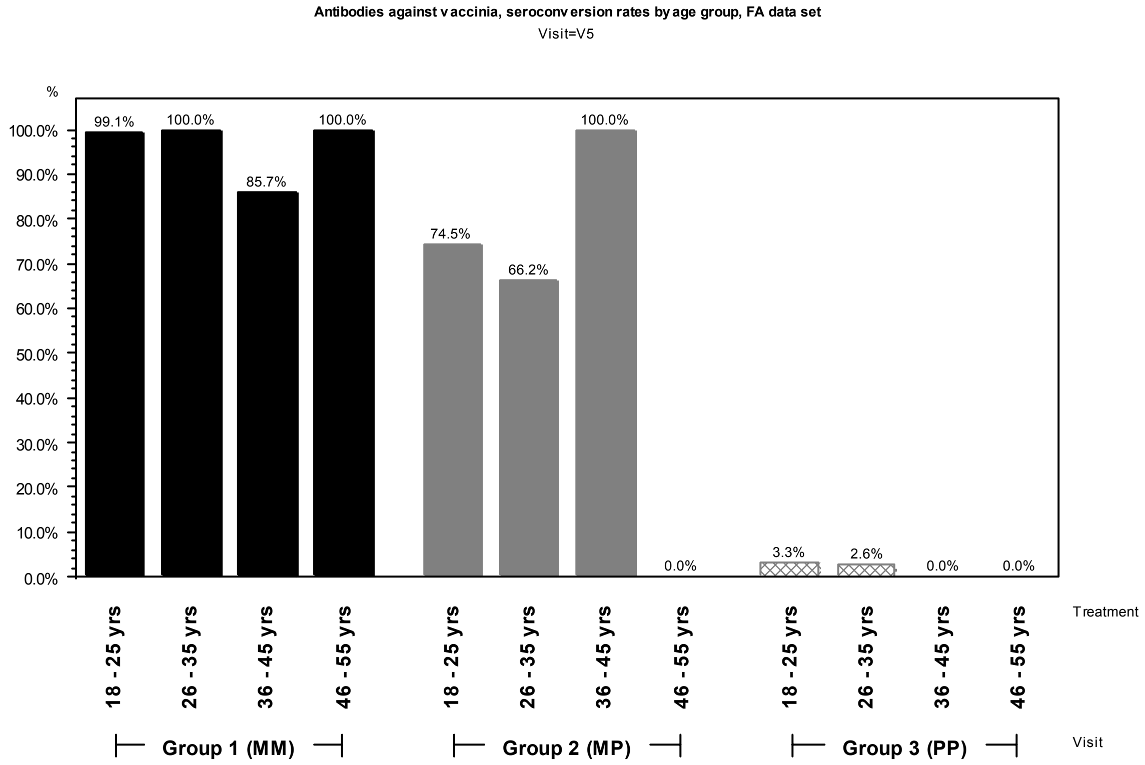
Antibodies against vaccinia, seroconversion rates by age group, FA data set
Visit=V5
%
100.0%
90.0%
80.0%
70.0%
60.0%
50.0%
40.0%
30.0%
20.0%
10.0%
0.0%
99.1%
100.0%
85.7%
100.0%
74.5%
66.2%
100.0%
0.0%
3.3%
2.6%
0.0%
0.0%
18 - 25 yrs
26 - 35 yrs
36 - 45 yrs
46 - 55 yrs
Treatment
Group 1 (MM)
Group 2 (MP)
Group 3 (PP)
Visit

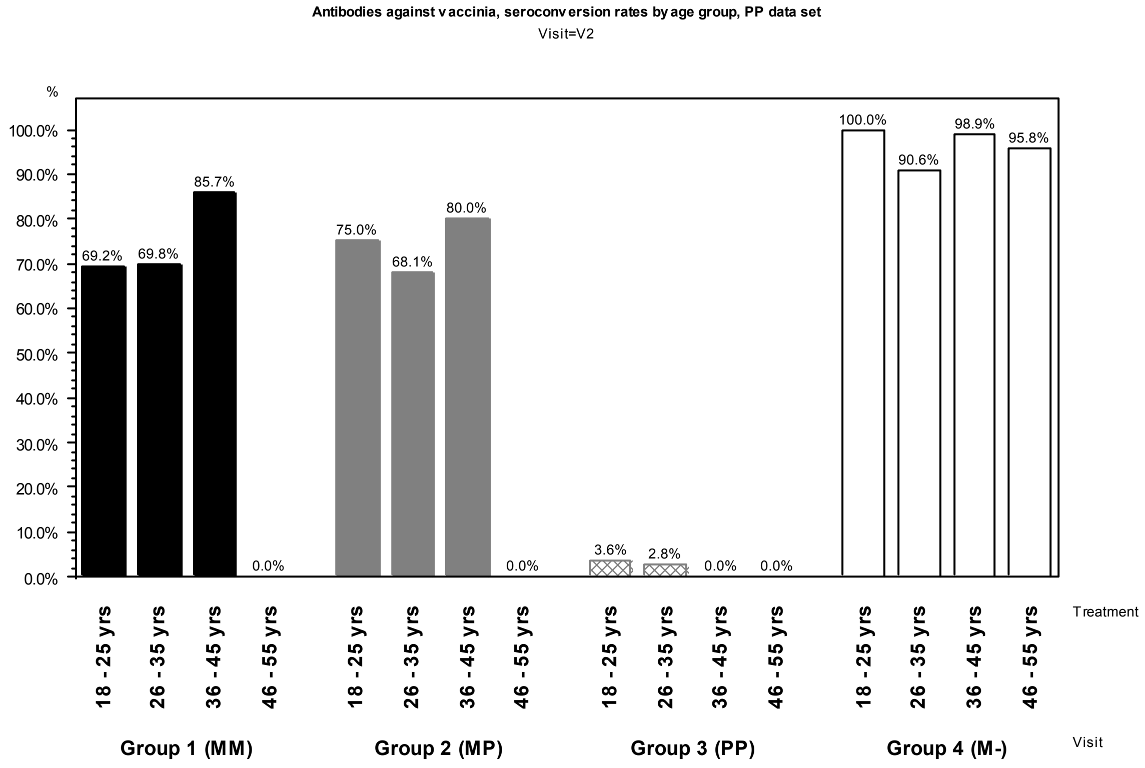
Antibodies against vaccinia, seroconversion rates by age group, PP data set
Visit=V2
%
100.0%
90.0%
80.0%
70.0%
60.0%
50.0%
40.0%
30.0%
20.0%
10.0%
0.0%
69.2%
69.8%
85.7%
0.0%
75.0%
68.1%
80.0%
0.0%
3.6%
2.8%
0.0%
0.0%
100.0%
90.6%
98.9%
95.8%
18 - 25 yrs
26 - 35 yrs
36 - 45 yrs
46 - 55 yrs
Treatment
Group 1 (MM)
Group 2 (MP)
Group 3 (PP)
Group 4 (M-)
Visit

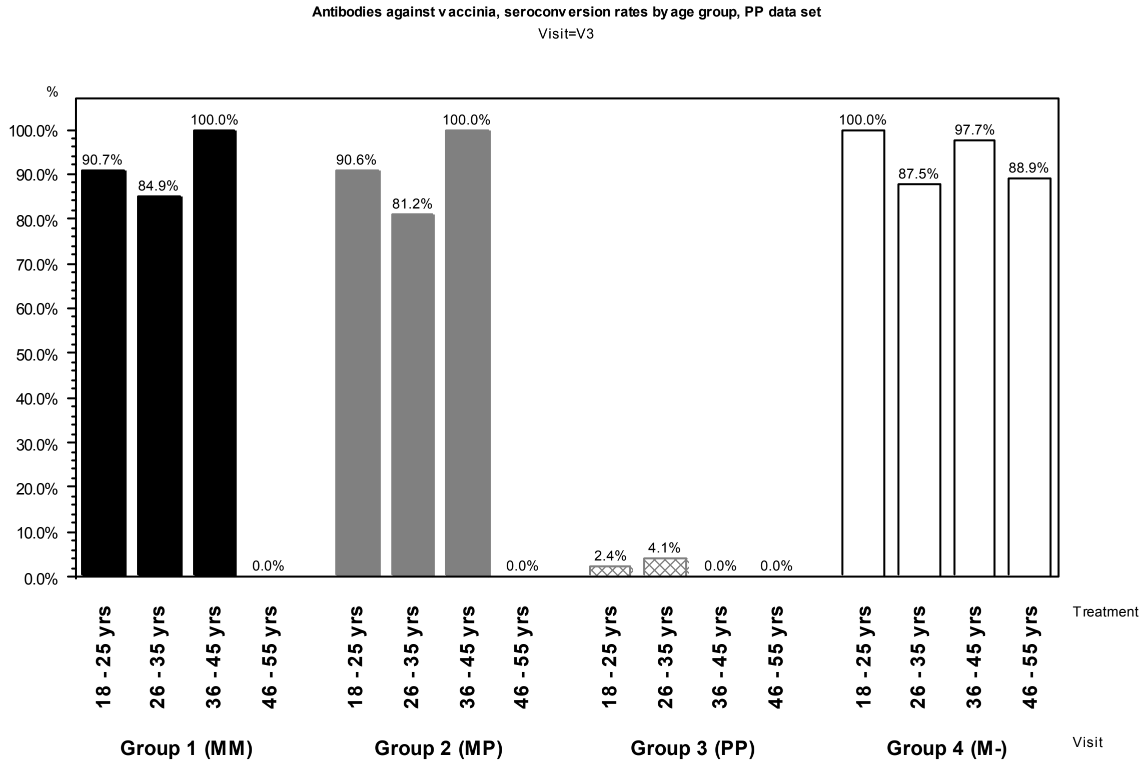
Antibodies against vaccinia, seroconversion rates by age group, PP data set
Visit=V3
%
100.0%
90.0%
80.0%
70.0%
60.0%
50.0%
40.0%
30.0%
20.0%
10.0%
0.0%
90.7%
84.9%
100.0%
0.0%
90.6%
81.2%
100.0%
0.0%
2.4%
4.1%
0.0%
0.0%
100.0%
87.5%
97.7%
88.9%
18 - 25 yrs
26 - 35 yrs
36 - 45 yrs
46 - 55 yrs
Treatment
Group 1 (MM)
Group 2 (MP)
Group 3 (PP)
Group 4 (M-)
Visit

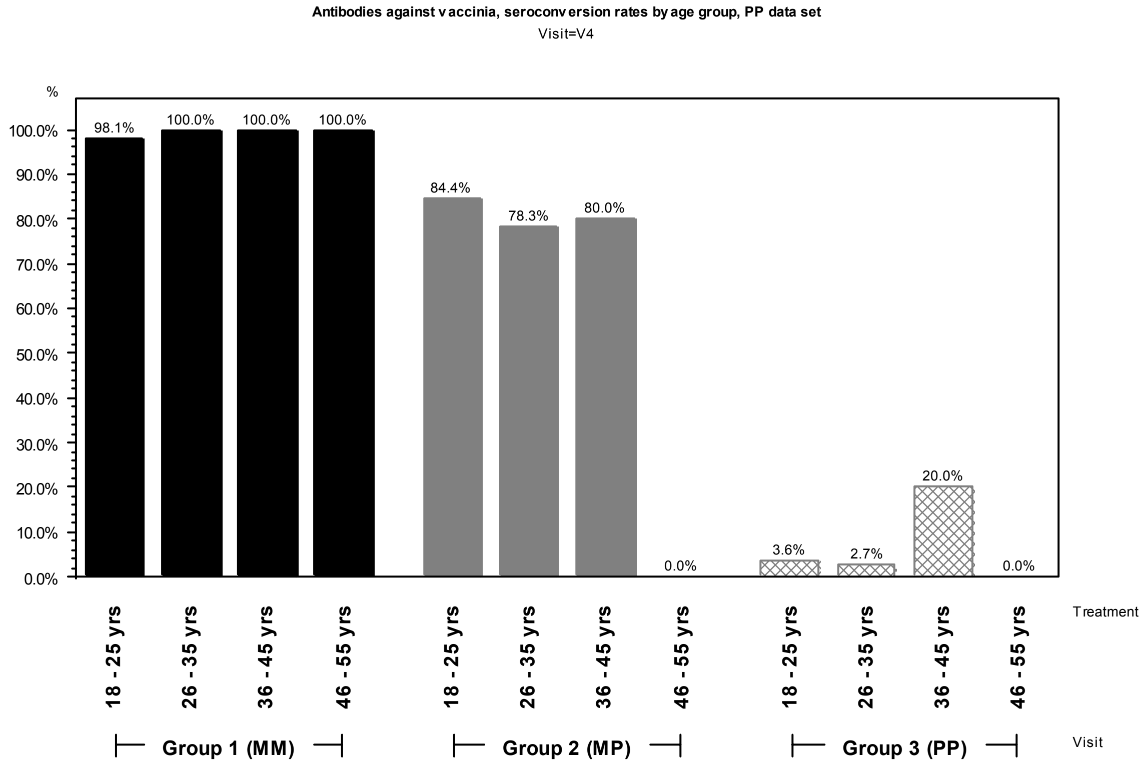
Antibodies against vaccinia, seroconversion rates by age group, PP data set
Visit=V4
%
100.0%
90.0%
80.0%
70.0%
60.0%
50.0%
40.0%
30.0%
20.0%
10.0%
0.0%
98.1%
100.0%
100.0%
100.0%
84.4%
78.3%
80.0%
0.0%
3.6%
2.7%
20.0%
0.0%
18 - 25 yrs
26 - 35 yrs
36 - 45 yrs
46 - 55 yrs
Treatment
Group 1 (MM)
Group 2 (MP)
Group 3 (PP)
Visit

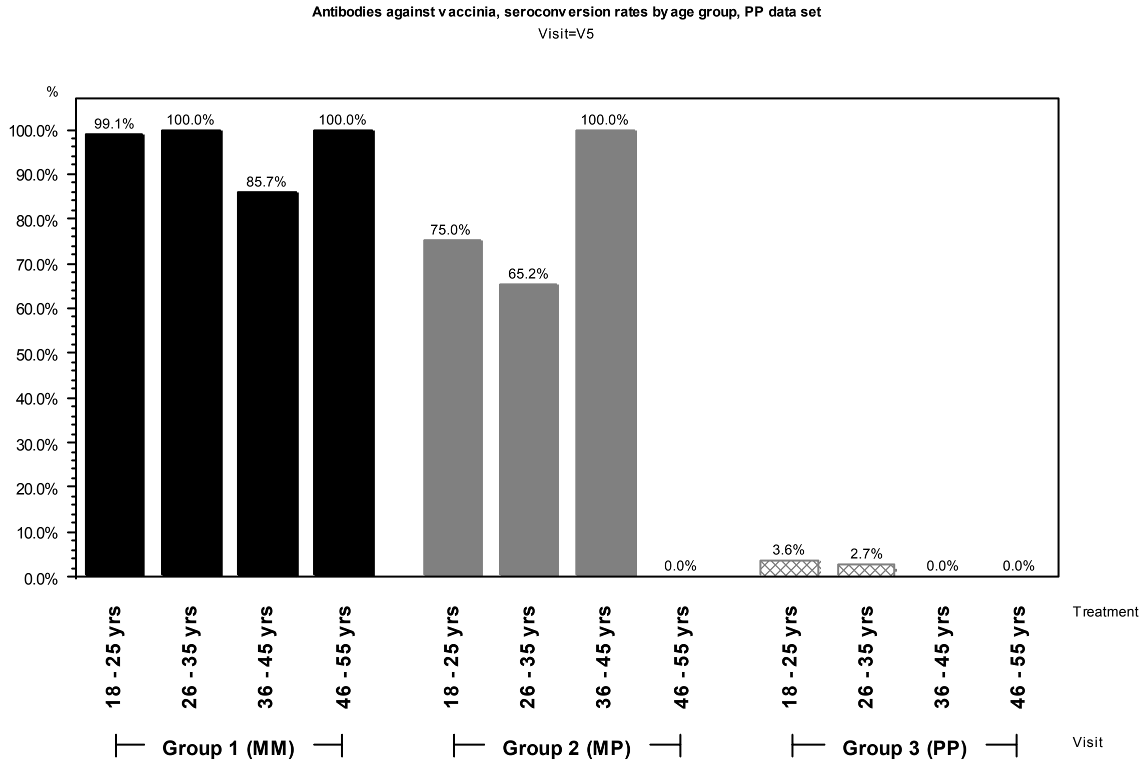

Antibodies against vaccinia, seroconversion rates by age group, PP data set
Visit=V5
%
100.0%
90.0%
80.0%
70.0%
60.0%
50.0%
40.0%
30.0%
20.0%
10.0%
0.0%
99.1%
100.0%
85.7%
100.0%
75.0%
65.2%
100.0%
0.0%
3.6%
2.7%
0.0%
0.0%
18 - 25 yrs
26 - 35 yrs
36 - 45 yrs
46 - 55 yrs
18 - 25 yrs
26 - 35 yrs
36 - 45 yrs
46 - 55 yrs
18 - 25 yrs
26 - 35 yrs
36 - 45 yrs
46 - 55 yrs
Treatment
Group 1 (MM)
Group 2 (MP)
Group 3 (PP)
Visit

14.7.2.2.2 Gender

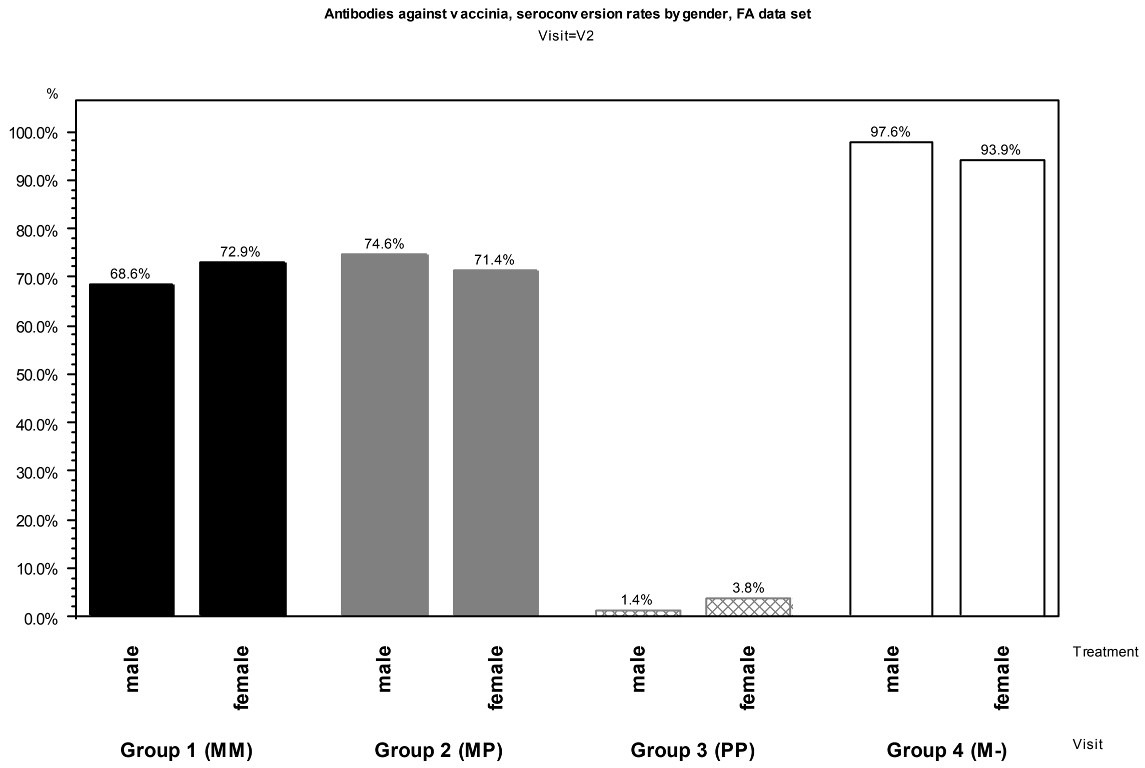

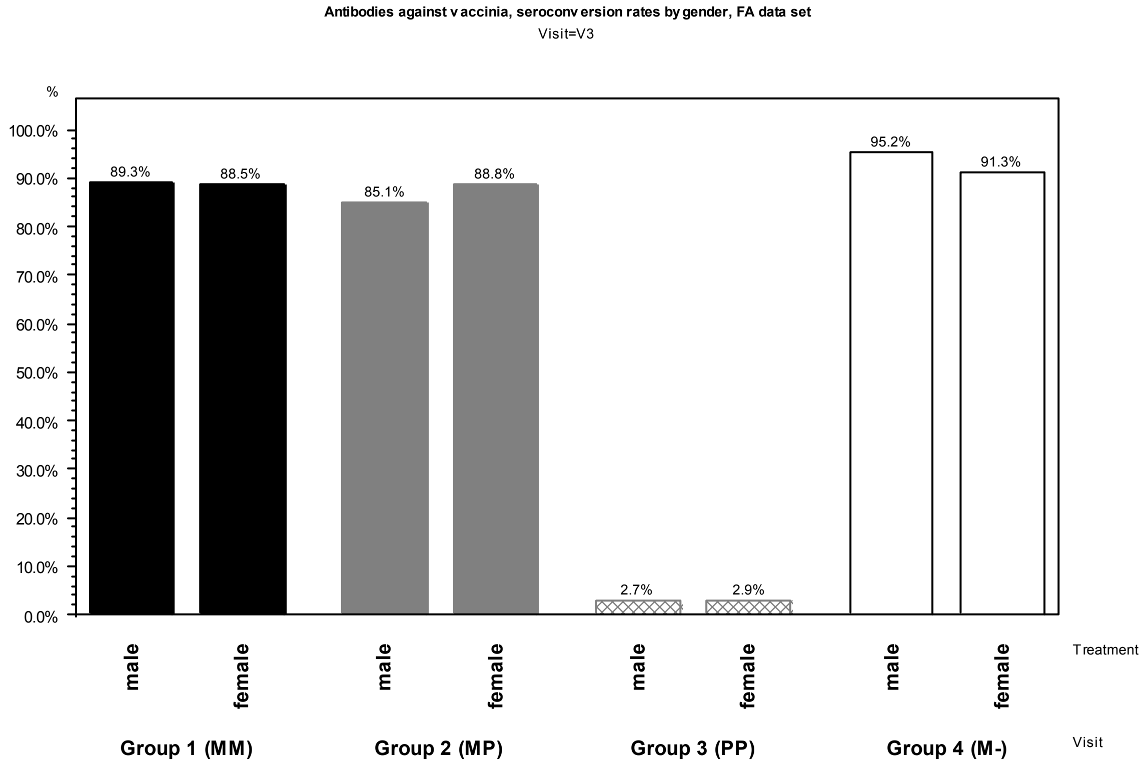
Antibodies against v accinia, seroconv ersion rates by gender, FA data set
Visit=V3
%
100.0%
90.0%
80.0%
70.0%
60.0%
50.0%
40.0%
30.0%
20.0%
10.0%
0.0%
89.3%
88.5%
85.1%
88.8%
2.7%
2.9%
95.2%
91.3%
male
female
male
female
male
female
male
female
Treatment
Group 1 (MM)
Group 2 (MP)
Group 3 (PP)
Group 4 (M-)
Visit

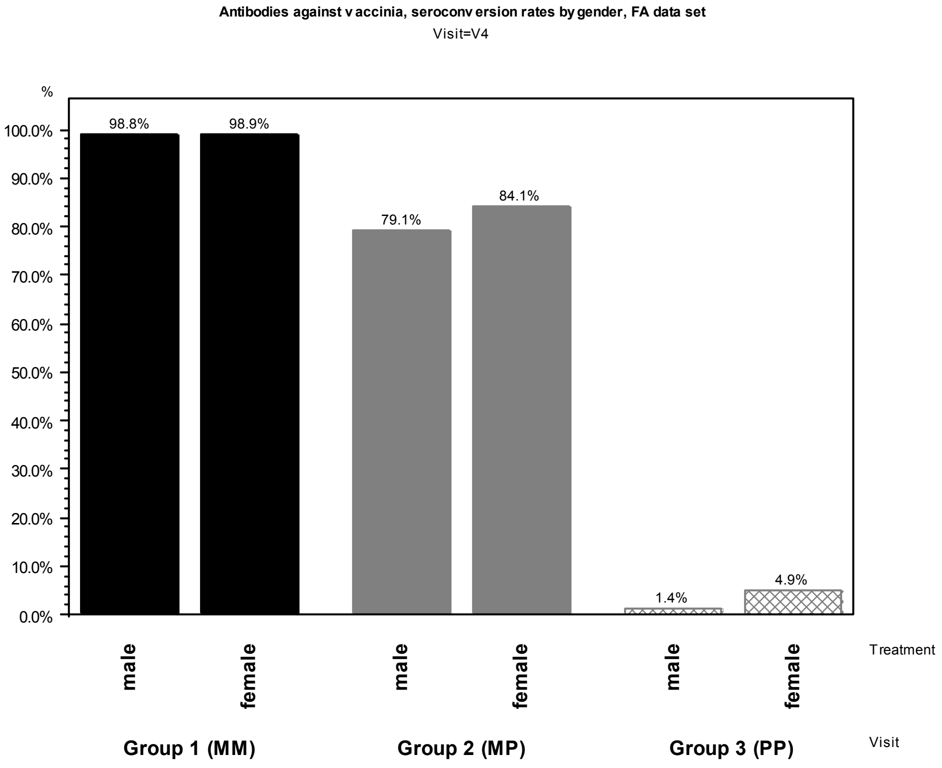
Antibodies against v accinia, seroconv ersion rates by gender, FA data set
Visit=V4
%
100.0%
90.0%
80.0%
70.0%
60.0%
50.0%
40.0%
30.0%
20.0%
10.0%
0.0%
98.8%
98.9%
79.1%
84.1%
1.4%
4.9%
male
female
male
female
male
female
Treatment
Group 1 (MM)
Group 2 (MP)
Group 3 (PP)
Visit

Antibodies against v accinia, seroconv ersion rates by gender, FA data set
Visit=V5

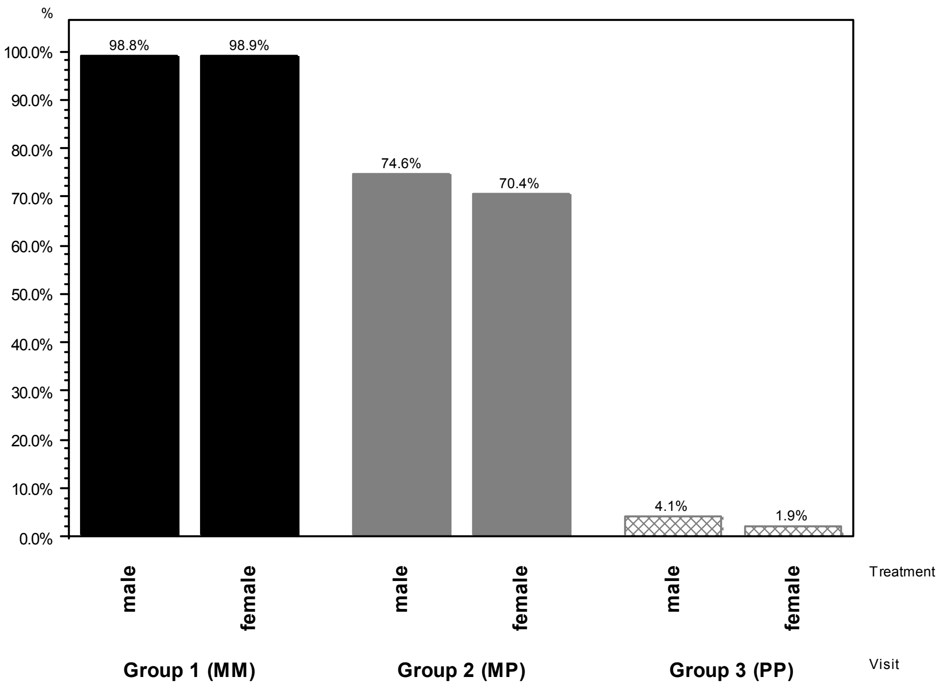

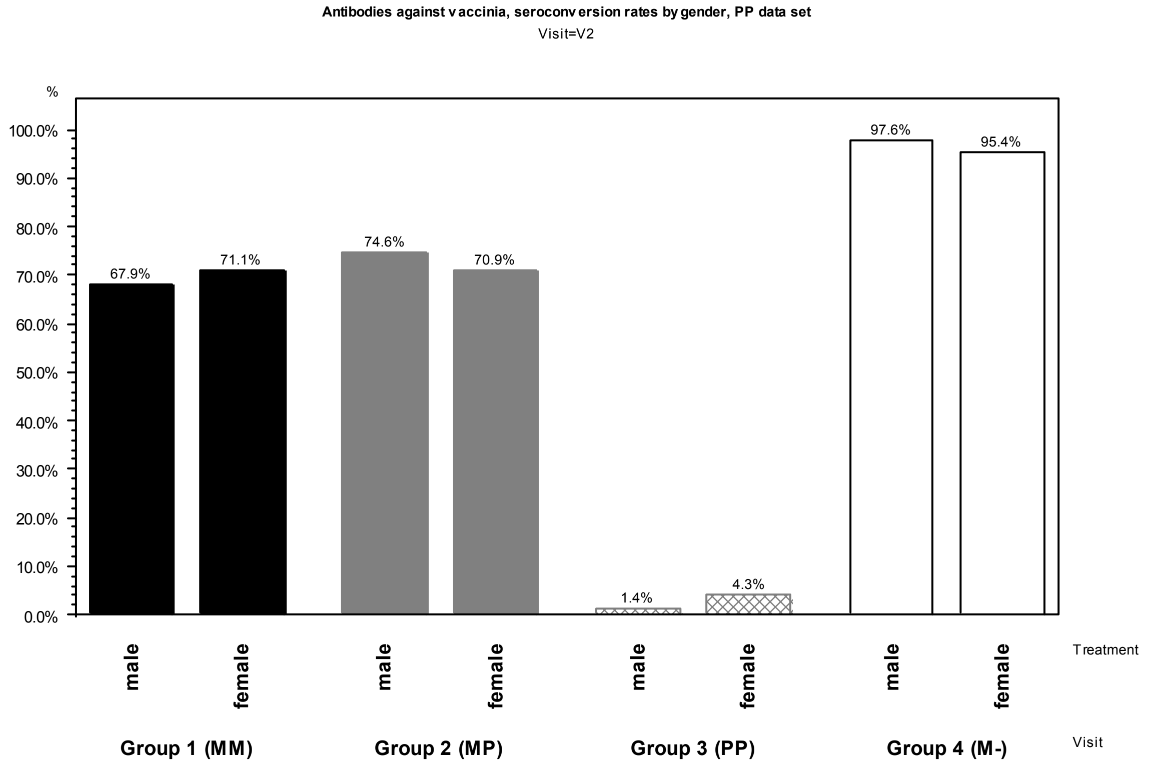

Antibodies against vaccinia, seroconversion rates by gender, PP data set
Visit=V2
%
100.0%
90.0%
80.0%
70.0%
60.0%
50.0%
40.0%
30.0%
20.0%
10.0%
0.0%
67.9%
71.1%
74.6%
70.9%
1.4%
4.3%
97.6%
95.4%
male
female
male
female
male
female
male
female
Treatment
Group 1 (MM)
Group 2 (MP)
Group 3 (PP)
Group 4 (M-)
Visit

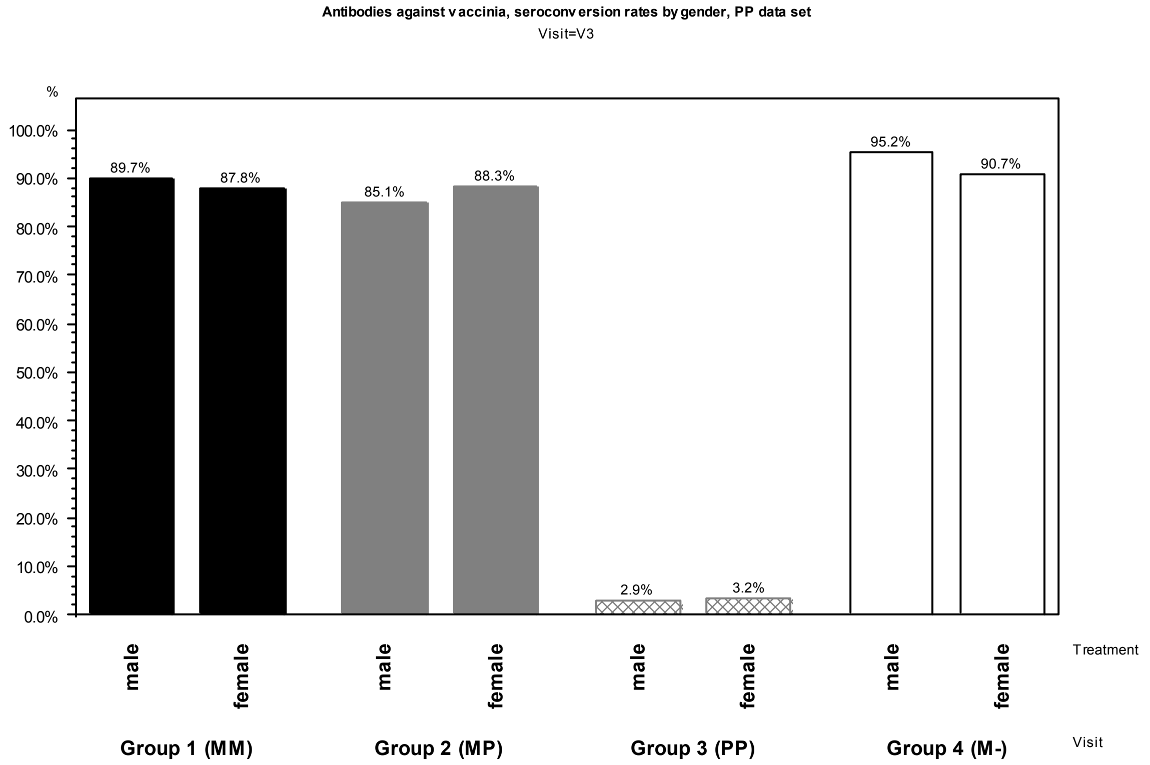
Antibodies against vaccinia, seroconversion rates by gender, PP data set
Visit=V3
%
100.0%
90.0%
80.0%
70.0%
60.0%
50.0%
40.0%
30.0%
20.0%
10.0%
0.0%
89.7%
87.8%
85.1%
88.3%
2.9%
3.2%
95.2%
90.7%
male
female
male
female
male
female
male
female
Treatment
Group 1 (MM)
Group 2 (MP)
Group 3 (PP)
Group 4 (M-)
Visit

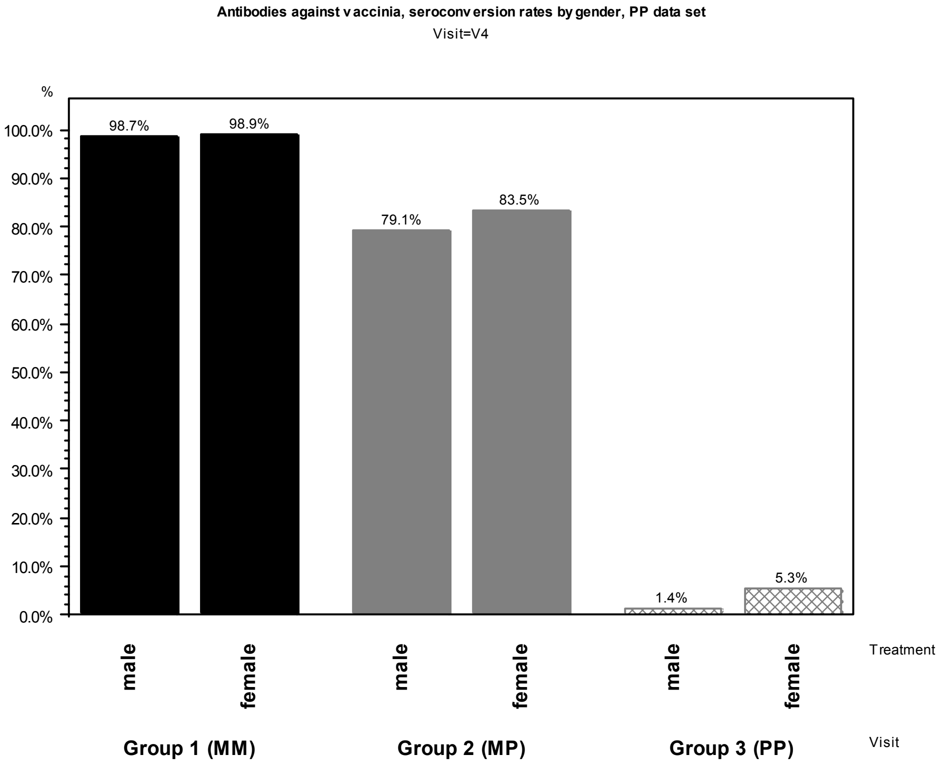
Antibodies against vaccinia, seroconversion rates by gender, PP data set
Visit=V4
%
100.0%
90.0%
80.0%
70.0%
60.0%
50.0%
40.0%
30.0%
20.0%
10.0%
0.0%
98.7%
98.9%
79.1%
83.5%
1.4%
5.3%
male
female
male
female
male
female
Treatment
Group 1 (MM)
Group 2 (MP)
Group 3 (PP)
Visit

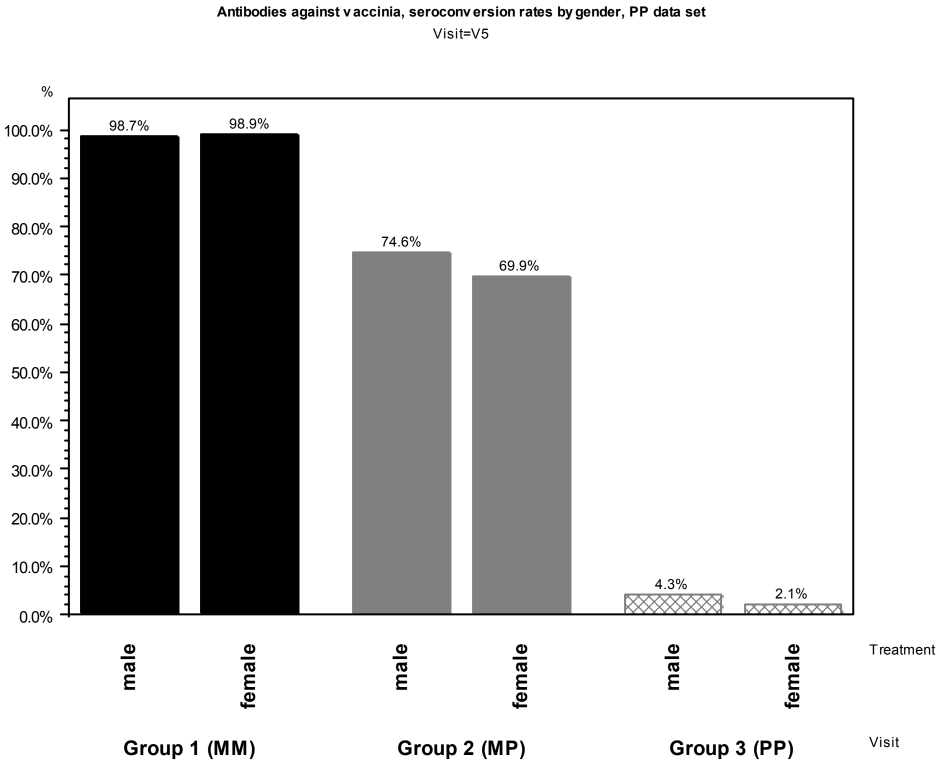
Antibodies against vaccinia, seroconversion rates by gender, PP data set
Visit=V5
%
100.0%
90.0%
80.0%
70.0%
60.0%
50.0%
40.0%
30.0%
20.0%
10.0%
0.0%
98.7%
98.9%
74.6%
69.9%
4.3%
2.1%
male
female
male
female
male
female
Treatment
Group 1 (MM)
Group 2 (MP)
Group 3 (PP)
Visit

14.7.3 Antibodies against vaccinia - Presentation by timepoint 2 and 4 wks after last vaccination

14.7.3.1 Tables

14.7.3.1.1 Antibodies against vaccinia, seroconversion rates, responders by gender - FA dataset

Group=Group 1 (MM)

	Number of Patients (%) / Descriptive Statistics male (N=86)	female (N=97)	p-value
2wks after last vaccination			
yes	81 (98.8)	93 (98.9)	0.854
no	1 (1.2)	1 (1.1)	
4wks after last vaccination			
yes	83 (98.8)	93 (98.9)	>0.999
no	1 (1.2)	1 (1.1)	

Seroconversion is defined as the appearance of antibody titers >= 1:50 in a vaccinia specific IgG ELISA for initially seronegative subjects or twofold increase of the antibody titer compared to the pre-existing baseline titer for subjects with a pre-existing antibody titer in the ELISA.
p-value: exact test of Fisher

Antibodies against vaccinia, seroconversion rates, responders by gender - FA dataset

Group=Group 2 (MP)

	Number of Patients (%) / Descriptive Statistics		
	male (N=69)	female (N=112)	p-value
2wks after last vaccination			
yes	53 (79.1)	90 (84.1)	0.668
no	14 (20.9)	17 (15.9)	
4wks after last vaccination			
yes	50 (74.6)	76 (70.4)	0.872
no	17 (25.4)	32 (29.6)	

Seroconversion is defined as the appearance of antibody titers >= 1:50 in a vaccinia specific IgG ELISA for initially seronegative subjects or twofold increase of the antibody titer compared to the pre-existing baseline titer for subjects with a pre-existing antibody titer in the ELISA.
p-value: exact test of Fisher

Antibodies against vaccinia, seroconversion rates, responders by gender - FA dataset

Group=Group 3 (PP)

	Number of Patients (%) / Descriptive Statistics		
	male (N=74)	female (N=107)	p-value
2wks after last vaccination			
yes	1 (1.4)	5 (4.9)	0.261
no	72 (98.6)	97 (95.1)	
4wks after last vaccination			
yes	3 (4.1)	2 (1.9)	0.500
no	70 (95.9)	102 (98.1)	

Seroconversion is defined as the appearance of antibody titers >= 1:50 in a vaccinia specific IgG ELISA for initially seronegative subjects or twofold increase of the antibody titer compared to the pre-existing baseline titer for subjects with a pre-existing antibody titer in the ELISA.
p-value: exact test of Fisher

Antibodies against vaccinia, seroconversion rates, responders by gender - FA dataset

Group=Group 4 (M-)

	Number of Patients (%) / Descriptive Statistics male (N=85)	female (N=115)	p-value
2wks after last vaccination			
yes	83 (97.6)	108 (93.9)	0.306
no	2 (2.4)	7 (6.1)	
4wks after last vaccination			
yes	80 (95.2)	105 (91.3)	0.262
no	4 (4.8)	10 (8.7)	

Seroconversion is defined as the appearance of antibody titers >= 1:50 in a vaccinia specific IgG ELISA for initially seronegative subjects or twofold increase of the antibody titer compared to the pre-existing baseline titer for subjects with a pre-existing antibody titer in the ELISA.
p-value: exact test of Fisher

Antibodies against vaccinia, seroconversion rates, responders by gender - PP dataset

Group=Group 1 (MM)

	Number of Patients (%) / Descriptive Statistics male (N=78)	female (N=90)	p-value
2wks after last vaccination			
yes	77 (98.7)	89 (98.9)	>0.999
no	1 (1.3)	1 (1.1)	
4wks after last vaccination			
yes	77 (98.7)	89 (98.9)	>0.999
no	1 (1.3)	1 (1.1)	

Seroconversion is defined as the appearance of antibody titers >= 1:50 in a vaccinia specific IgG ELISA for initially seronegative subjects or twofold increase of the antibody titer compared to the pre-existing baseline titer for subjects with a pre-existing antibody titer in the ELISA.
p-value: exact test of Fisher

14.7.3.1.2 Antibodies against vaccinia, seroconversion rates, responders by gender - PP dataset

Group=Group 2 (MP)

	Number of Patients (%) / Descriptive Statistics		
	male (N=67)	female (N=103)	p-value
2wks after last vaccination			
yes	53 (79.1)	86 (83.5)	0.543
no	14 (20.9)	17 (16.5)	
4wks after last vaccination			
yes	50 (74.6)	72 (69.9)	0.602
no	17 (25.4)	31 (30.1)	

Seroconversion is defined as the appearance of antibody titers >= 1:50 in a vaccinia specific IgG ELISA for initially seronegative subjects or twofold increase of the antibody titer compared to the pre-existing baseline titer for subjects with a pre-existing antibody titer in the ELISA.
p-value: exact test of Fisher

Antibodies against vaccinia, seroconversion rates, responders by gender - PP dataset

Group=Group 3 (PP)

	Number of Patients (%) / Descriptive Statistics		
	male (N=70)	female (N=94)	p-value
2wks after last vaccination			
yes	1 (1.4)	5 (5.3)	0.241
no	69 (98.6)	89 (94.7)	
4wks after last vaccination			
yes	3 (4.3)	2 (2.1)	0.652
no	67 (95.7)	92 (97.9)	

Seroconversion is defined as the appearance of antibody titers >= 1:50 in a vaccinia specific IgG ELISA for initially seronegative subjects or twofold increase of the antibody titer compared to the pre-existing baseline titer for subjects with a pre-existing antibody titer in the ELISA.
p-value: exact test of Fisher

Antibodies against vaccinia, seroconversion rates, responders by gender - PP dataset

Group=Group 4 (M-)

	Number of Patients (%) / Descriptive Statistics		
	male (N=85)	female (N=108)	p-value
2wks after last vaccination			
yes	83 (97.6)	103 (95.4)	0.468
no	2 (2.4)	5 (4.6)	
4wks after last vaccination			
yes	80 (95.2)	98 (90.7)	0.212
no	4 (4.8)	10 (9.3)	

Seroconversion is defined as the appearance of antibody titers >= 1:50 in a vaccinia specific IgG ELISA for initially seronegative subjects or twofold increase of the antibody titer compared to the pre-existing baseline titer for subjects with a pre-existing antibody titer in the ELISA.
p-value: exact test of Fisher

Antibodies against vaccinia, seroconversion rates, responders by age group - FA dataset

Group=Group 1 (MM)

	Number of Patients (%) / Descriptive Statistics				
	18 - 25 yrs (N=116)	26 - 35 yrs (N=59)	36 - 45 yrs (N=7)	46 - 55 yrs (N=1)	p-value
2wks after last vaccination					
yes	110 (98.2)	56 (100.0)	7 (100.0)	1 (100.0)	0.739
no	2 (1.8)	0 (0.0)	0 (0.0)	0 (0.0)	
4wks after last vaccination					
yes	113 (99.1)	56 (100.0)	6 (85.7)	1 (100.0)	0.133
no	1 (0.9)	0 (0.0)	1 (14.3)	0 (0.0)	

Seroconversion is defined as the appearance of antibody titers >= 1:50 in a vaccinia specific IgG ELISA for initially seronegative subjects or twofold increase of the antibody titer compared to the pre-existing baseline titer for subjects with a pre-existing antibody titer in the ELISA.
p-value: exact test of Fisher

14.7.3.1.3 Antibodies against vaccinia, seroconversion rates, responders by age group - FA dataset

Group=Group 2 (MP)

	Number of Patients (%) / Descriptive Statistics			
	18 - 25 yrs (N=102)	26 - 35 yrs (N=73)	36 - 45 yrs (N=6)	p-value
2wks after last vaccination				
yes	82 (84.5)	57 (79.2)	4 (80.0)	0.217
no	15 (15.5)	15 (20.8)	1 (20.0)	
4wks after last vaccination				
yes	73 (74.5)	47 (66.2)	6 (100.0)	0.407
no	25 (25.5)	24 (33.8)	0 (0.0)	

Seroconversion is defined as the appearance of antibody titers >= 1:50 in a vaccinia specific IgG ELISA for initially seronegative subjects or twofold increase of the antibody titer compared to the pre-existing baseline titer for subjects with a pre-existing antibody titer in the ELISA.
p-value: exact test of Fisher

Antibodies against vaccinia, seroconversion rates, responders by age group - FA dataset

Group=Group 3 (PP)

	Number of Patients (%) / Descriptive Statistics				
	18 - 25 yrs (N=92)	26 - 35 yrs (N=81)	36 - 45 yrs (N=6)	46 - 55 yrs (N=2)	p-value
2wks after last vaccination					
yes	3 (3.3)	2 (2.6)	1 (20.0)	0 (0.0)	0.174
no	87 (96.7)	76 (97.4)	4 (80.0)	2 (100.0)	
4wks after last vaccination					
yes	3 (3.3)	2 (2.6)	0 (0.0)	0 (0.0)	0.727
no	88 (96.7)	76 (97.4)	6 (100.0)	2 (100.0)	

Seroconversion is defined as the appearance of antibody titers >= 1:50 in a vaccinia specific IgG ELISA for initially seronegative subjects or twofold increase of the antibody titer compared to the pre-existing baseline titer for subjects with a pre-existing antibody titer in the ELISA.
p-value: exact test of Fisher

Antibodies against vaccinia, seroconversion rates, responders by age group - FA dataset

Group=Group 4 (M-)

	Number of Patients (%) / Descriptive Statistics 18 - 25 yrs (N=4)	26 - 35 yrs (N=36)	36 - 45 yrs (N=88)	46 - 55 yrs (N=72)	p-value
2wks after last vaccination					
yes	3 (75.0)	32 (88.9)	87 (98.9)	69 (95.8)	0.021
no	1 (25.0)	4 (11.1)	1 (1.1)	3 (4.2)	
4wks after last vaccination					
yes	4 (100.0)	32 (88.9)	85 (97.7)	64 (88.9)	0.118
no	0 (0.0)	4 (11.1)	2 (2.3)	8 (11.1)	

Seroconversion is defined as the appearance of antibody titers >= 1:50 in a vaccinia specific IgG ELISA for initially seronegative subjects or twofold increase of the antibody titer compared to the pre-existing baseline titer for subjects with a pre-existing antibody titer in the ELISA.
p-value: exact test of Fisher

14.7.3.1.4 Antibodies against vaccinia, seroconversion rates, responders by age group - PP dataset

Group=Group 1 (MM)

	Number of Patients (%) / Descriptive Statistics<br>18 - 25 yrs (N=107)	26 - 35 yrs (N=53)	36 - 45 yrs (N=7)	46 - 55 yrs (N=1)	p-value
2wks after last vaccination					
yes	105 (98.1)	53 (100.0)	7 (100.0)	1 (100.0)	>0.999
no	2 (1.9)	0 (0.0)	0 (0.0)	0 (0.0)	
4wks after last vaccination					
yes	106 (99.1)	53 (100.0)	6 (85.7)	1 (100.0)	0.093
no	1 (0.9)	0 (0.0)	1 (14.3)	0 (0.0)	

Seroconversion is defined as the appearance of antibody titers >= 1:50 in a vaccinia specific IgG ELISA for initially seronegative subjects or twofold increase of the antibody titer compared to the pre-existing baseline titer for subjects with a pre-existing antibody titer in the ELISA.
p-value: exact test of Fisher

Antibodies against vaccinia, seroconversion rates, responders by age group - PP dataset

Group=Group 2 (MP)

	Number of Patients (%) / Descriptive Statistics			
	18 - 25 yrs (N=96)	26 - 35 yrs (N=69)	36 - 45 yrs (N=5)	p-value
2wks after last vaccination				
yes	81 (84.4)	54 (78.3)	4 (80.0)	0.509
no	15 (15.6)	15 (21.7)	1 (20.0)	
4wks after last vaccination				
yes	72 (75.0)	45 (65.2)	5 (100.0)	0.162
no	24 (25.0)	24 (34.8)	0 (0.0)	

Seroconversion is defined as the appearance of antibody titers >= 1:50 in a vaccinia specific IgG ELISA for initially seronegative subjects or twofold increase of the antibody titer compared to the pre-existing baseline titer for subjects with a pre-existing antibody titer in the ELISA.
p-value: exact test of Fisher

Antibodies against vaccinia, seroconversion rates, responders by age group - PP dataset

Group=Group 3 (PP)

	Number of Patients (%) / Descriptive Statistics				
	18 - 25 yrs (N=84)	26 - 35 yrs (N=73)	36 - 45 yrs (N=5)	46 - 55 yrs (N=2)	p-value
2wks after last vaccination					
yes	3 (3.6)	2 (2.7)	1 (20.0)	0 (0.0)	0.257
no	81 (96.4)	71 (97.3)	4 (80.0)	2 (100.0)	
4wks after last vaccination					
yes	3 (3.6)	2 (2.7)	0 (0.0)	0 (0.0)	>0.999
no	81 (96.4)	71 (97.3)	5 (100.0)	2 (100.0)	

Seroconversion is defined as the appearance of antibody titers >= 1:50 in a vaccinia specific IgG ELISA for initially seronegative subjects or twofold increase of the antibody titer compared to the pre-existing baseline titer for subjects with a pre-existing antibody titer in the ELISA.
p-value: exact test of Fisher

Antibodies against vaccinia, seroconversion rates, responders by age group - PP dataset

Group=Group 4 (M-)

	Number of Patients (%) / Descriptive Statistics				
	18 - 25 yrs (N=2)	26 - 35 yrs (N=32)	36 - 45 yrs (N=87)	46 - 55 yrs (N=72)	p-value
2wks after last vaccination					
yes	2 (100.0)	29 (90.6)	86 (98.9)	69 (95.8)	0.148
no	0 (0.0)	3 (9.4)	1 (1.1)	3 (4.2)	
4wks after last vaccination					
yes	2 (100.0)	28 (87.5)	84 (97.7)	64 (88.9)	0.095
no	0 (0.0)	4 (12.5)	2 (2.3)	8 (11.1)	

Seroconversion is defined as the appearance of antibody titers >= 1:50 in a vaccinia specific IgG ELISA for initially seronegative subjects or twofold increase of the antibody titer compared to the pre-existing baseline titer for subjects with a pre-existing antibody titer in the ELISA.
p-value: exact test of Fisher

14.7.3.2 Figures

14.7.3.2.1 Age group

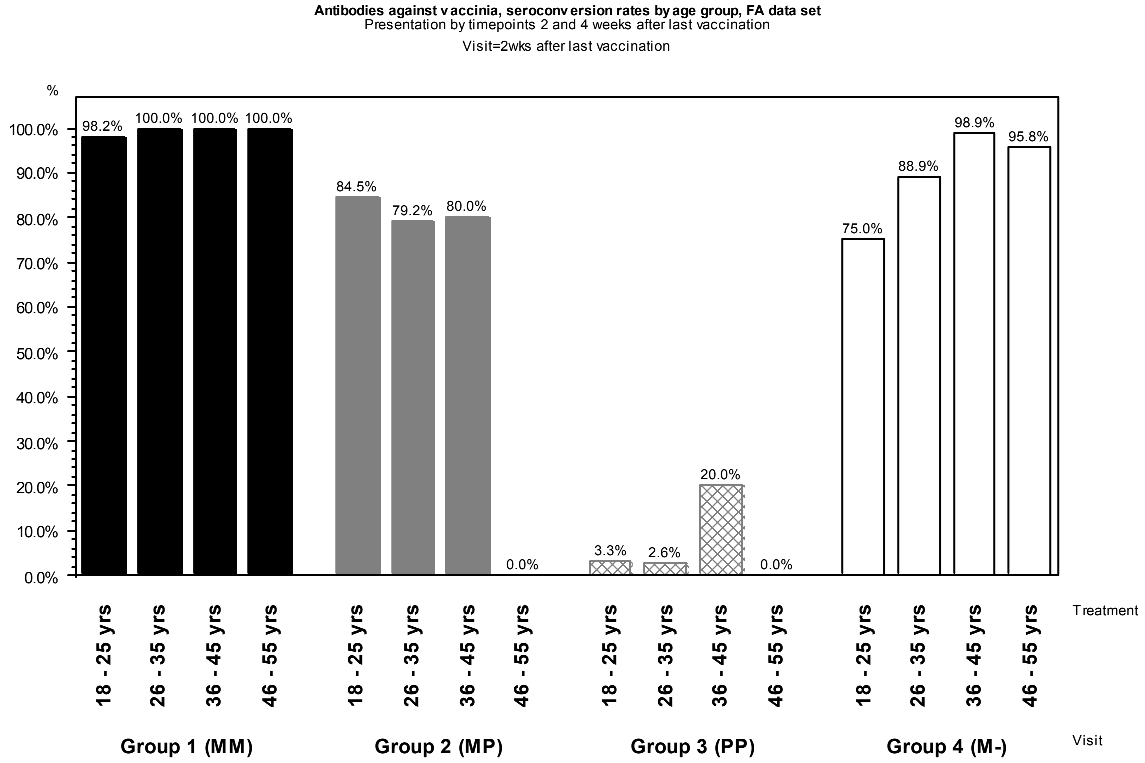

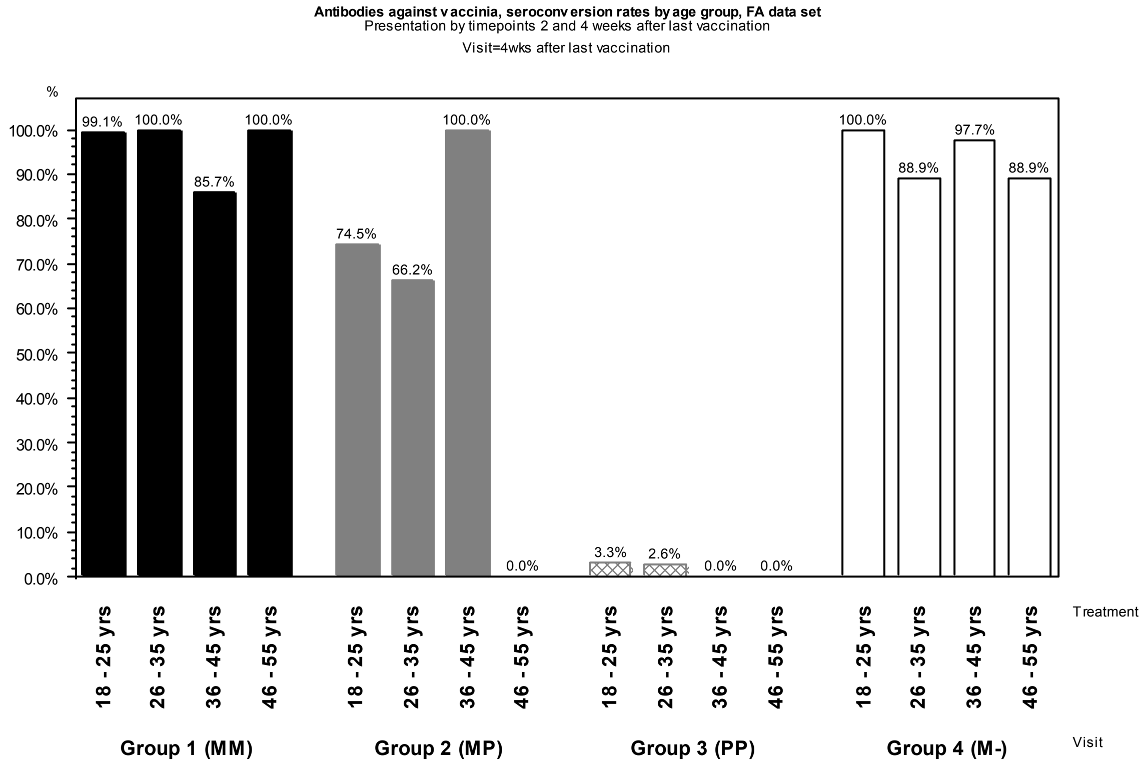
Antibodies against vaccinia, seroconversion rates by age group, FA data set
Presentation by timepoints 2 and 4 weeks after last vaccination
Visit=4wks after last vaccination
%
100.0%
90.0%
80.0%
70.0%
60.0%
50.0%
40.0%
30.0%
20.0%
10.0%
0.0%
99.1%
100.0%
85.7%
100.0%
74.5%
66.2%
100.0%
0.0%
3.3%
2.6%
0.0%
0.0%
100.0%
88.9%
97.7%
88.9%
18 - 25 yrs
26 - 35 yrs
36 - 45 yrs
46 - 55 yrs
Treatment
Group 1 (MM)
Group 2 (MP)
Group 3 (PP)
Group 4 (M-)
Visit

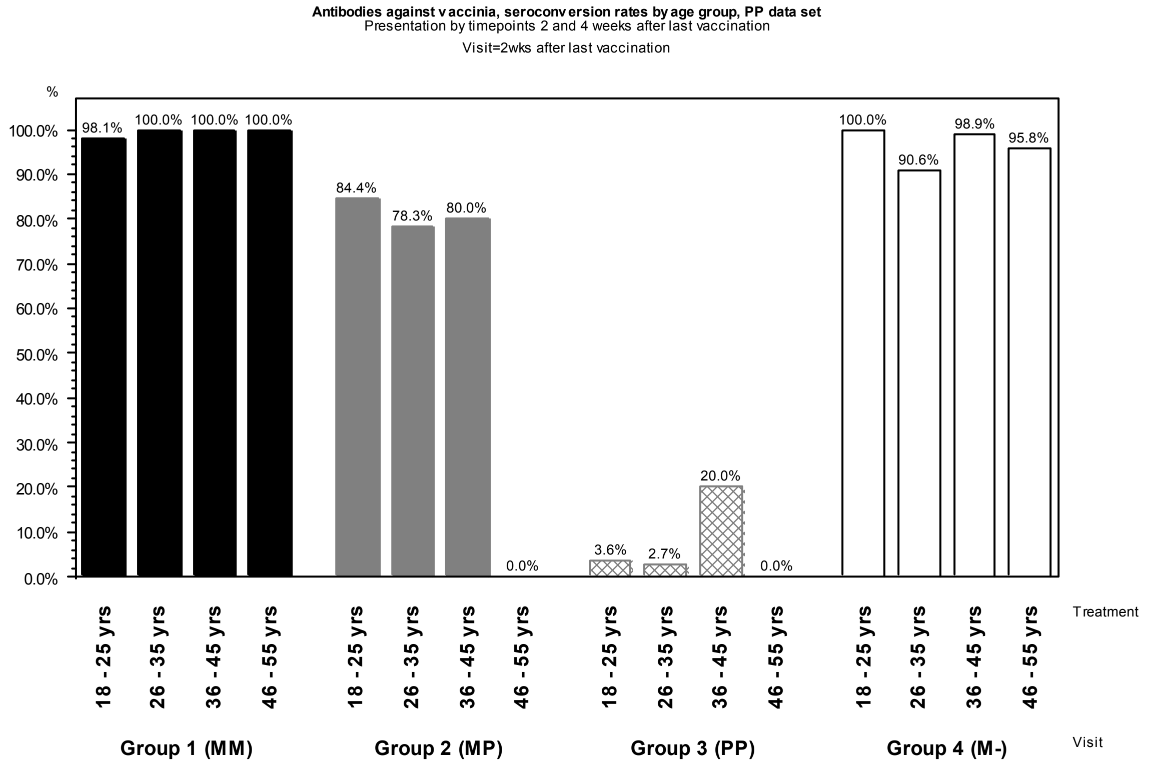
Antibodies against vaccinia, seroconversion rates by age group, PP data set
Presentation by timepoints 2 and 4 weeks after last vaccination
Visit=2wks after last vaccination
%
100.0%
90.0%
80.0%
70.0%
60.0%
50.0%
40.0%
30.0%
20.0%
10.0%
0.0%
98.1%
100.0%
100.0%
100.0%
84.4%
78.3%
80.0%
0.0%
3.6%
2.7%
20.0%
0.0%
100.0%
90.6%
98.9%
95.8%
18 - 25 yrs
26 - 35 yrs
36 - 45 yrs
46 - 55 yrs
Treatment
Group 1 (MM)
Group 2 (MP)
Group 3 (PP)
Group 4 (M-)
Visit

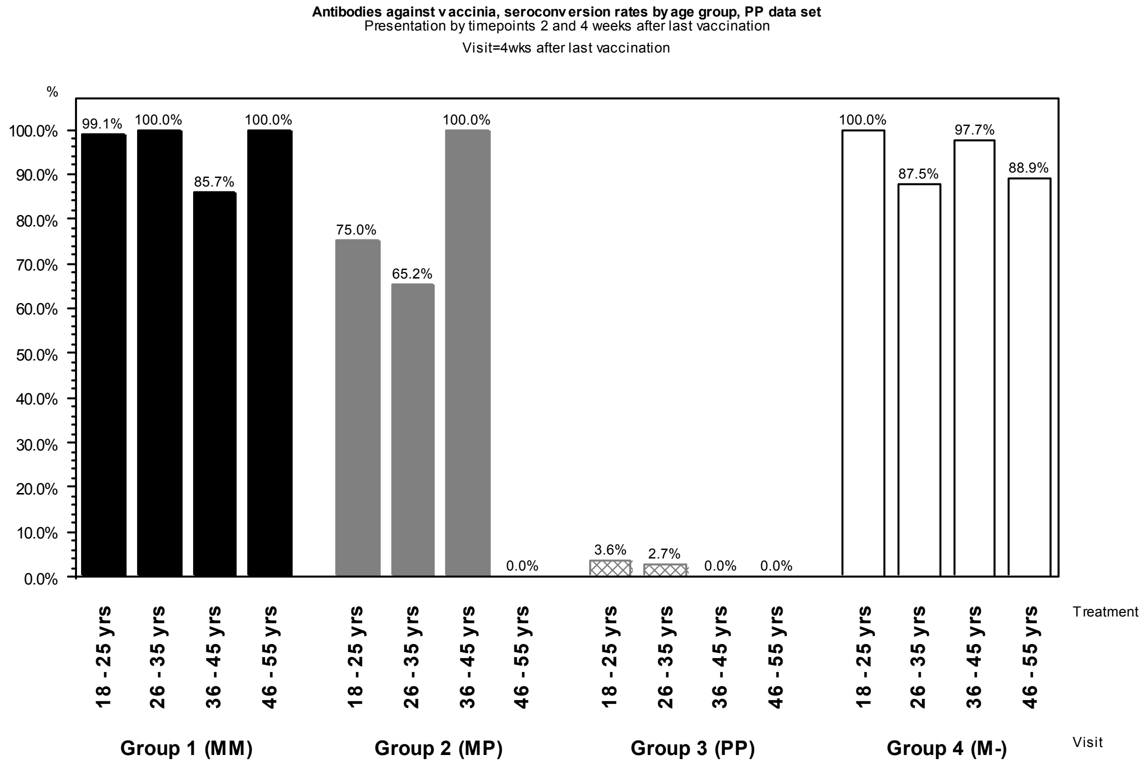
Antibodies against vaccinia, seroconversion rates by age group, PP data set
Presentation by timepoints 2 and 4 weeks after last vaccination
Visit=4wks after last vaccination
%
100.0%
90.0%
80.0%
70.0%
60.0%
50.0%
40.0%
30.0%
20.0%
10.0%
0.0%
99.1%
100.0%
85.7%
100.0%
75.0%
65.2%
100.0%
0.0%
3.6%
2.7%
0.0%
0.0%
100.0%
87.5%
97.7%
88.9%
18 - 25 yrs
26 - 35 yrs
36 - 45 yrs
46 - 55 yrs
Treatment
Group 1 (MM)
Group 2 (MP)
Group 3 (PP)
Group 4 (M-)
Visit

14.7.3.2.2 Gender

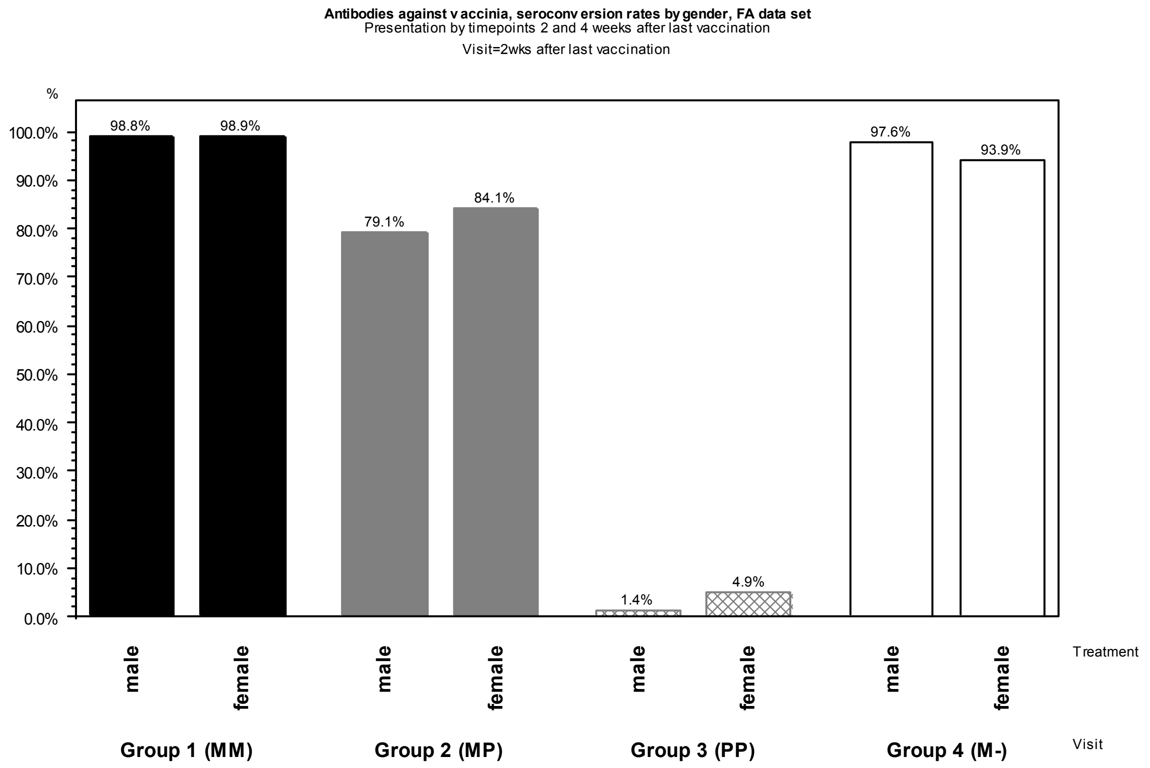

Antibodies against vaccinia, seroconversion rates by gender, FA data set
Presentation by timepoints 2 and 4 weeks after last vaccination
Visit=4wks after last vaccination

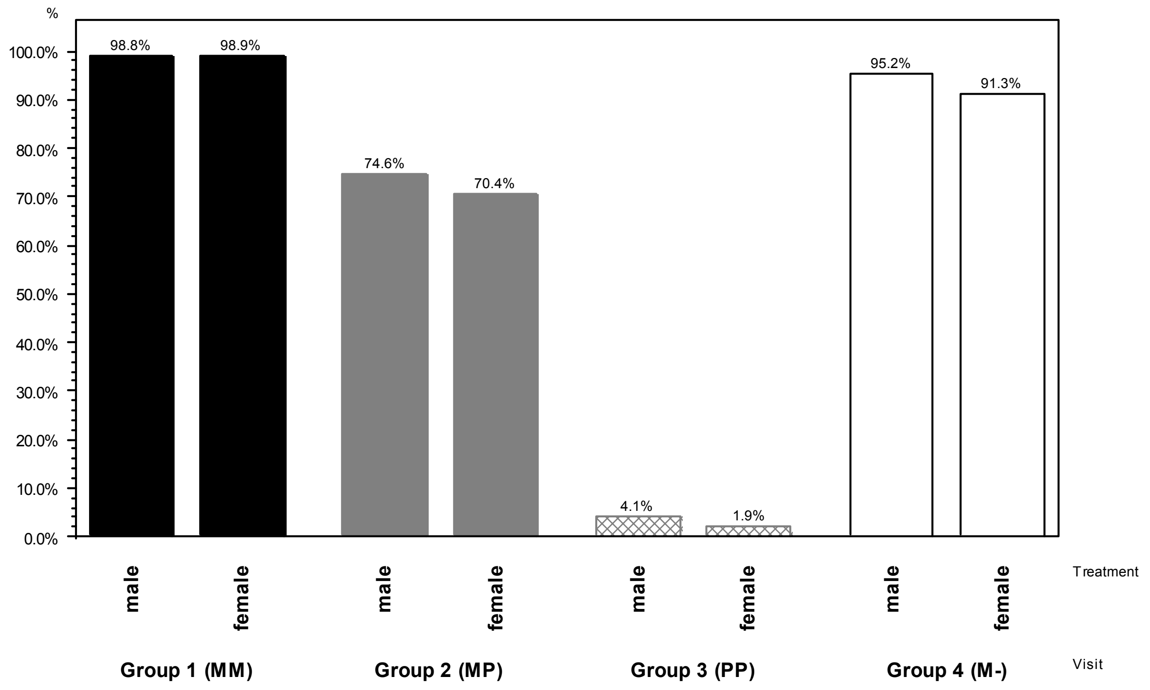

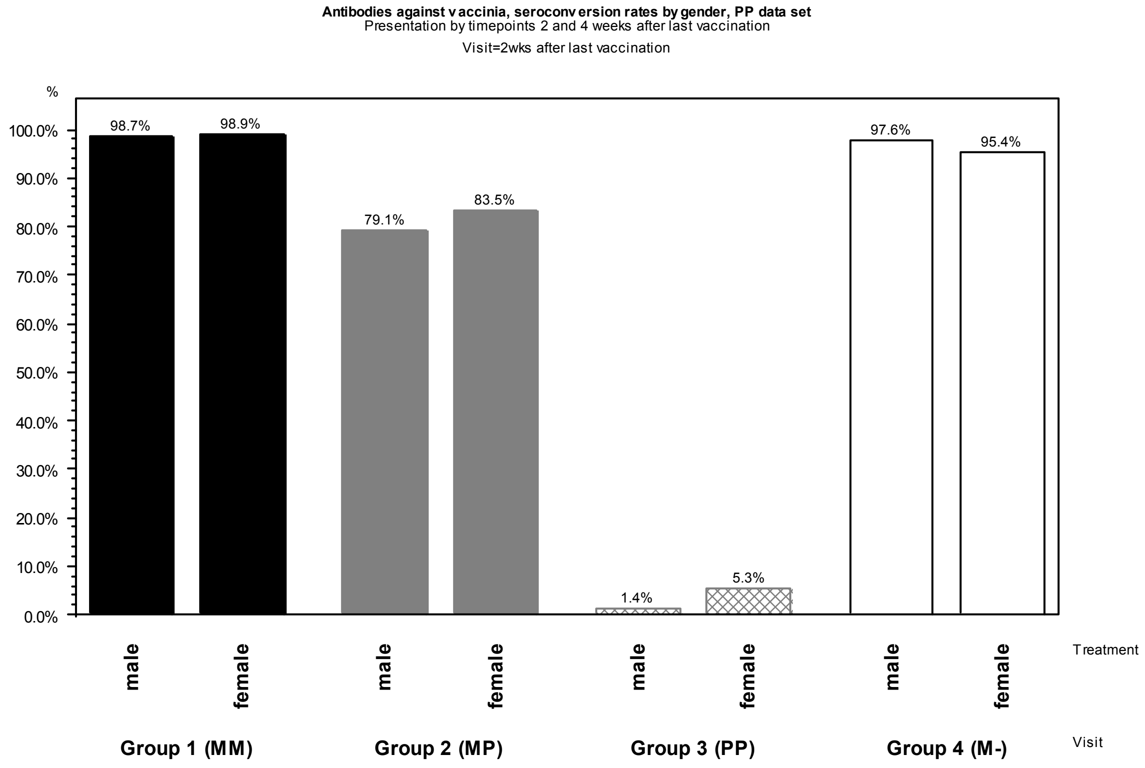
Antibodies against vaccinia, seroconversion rates by gender, PP data set
Presentation by timepoints 2 and 4 weeks after last vaccination
Visit=2wks after last vaccination
%
100.0%
90.0%
80.0%
70.0%
60.0%
50.0%
40.0%
30.0%
20.0%
10.0%
0.0%
98.7%
98.9%
79.1%
83.5%
1.4%
5.3%
97.6%
95.4%
male
female
male
female
male
female
male
female
Treatment
Group 1 (MM)
Group 2 (MP)
Group 3 (PP)
Group 4 (M-)
Visit

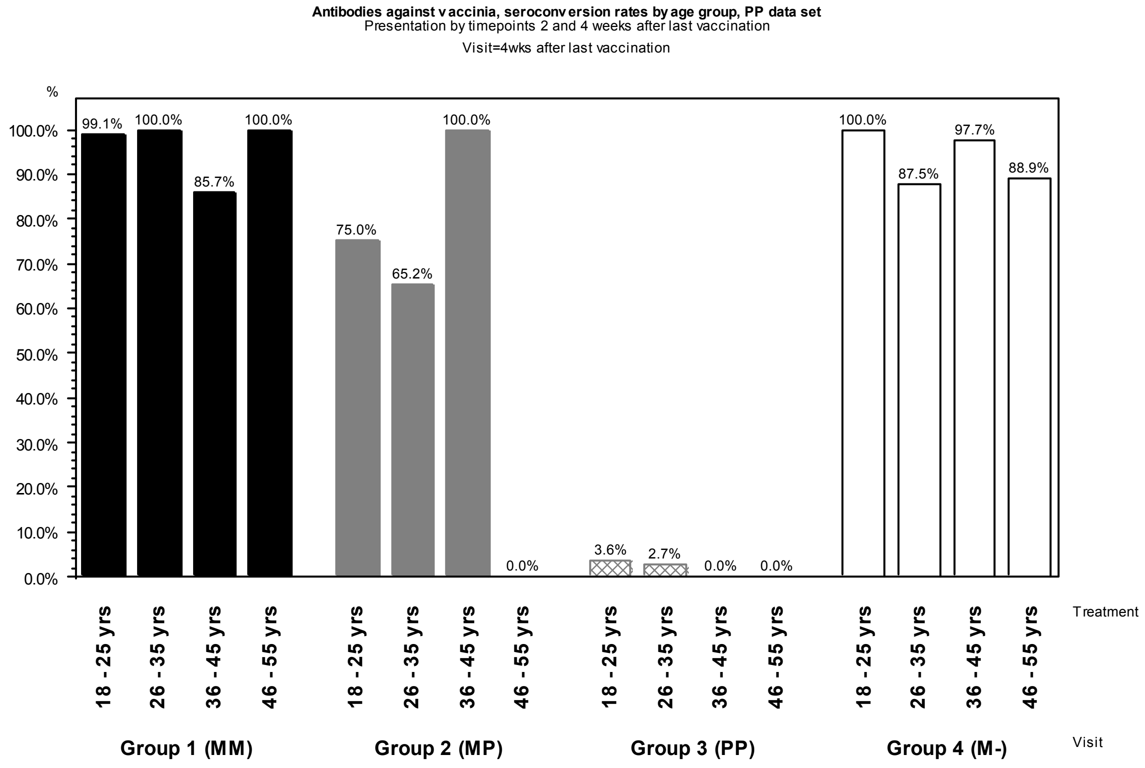
Antibodies against vaccinia, seroconversion rates by age group, PP data set
Presentation by timepoints 2 and 4 weeks after last vaccination
Visit=4wks after last vaccination
%
100.0%
90.0%
80.0%
70.0%
60.0%
50.0%
40.0%
30.0%
20.0%
10.0%
0.0%
99.1%
100.0%
85.7%
100.0%
75.0%
65.2%
100.0%
0.0%
3.6%
2.7%
0.0%
0.0%
100.0%
87.5%
97.7%
88.9%
18 - 25 yrs
26 - 35 yrs
36 - 45 yrs
46 - 55 yrs
Treatment
Group 1 (MM)
Group 2 (MP)
Group 3 (PP)
Group 4 (M-)
Visit

15 REFERENCES

REFERENCES

Agresti A, Min Y. On small-sample confidence intervals for parameters in discrete distributions. Biometrics 2001, Sep; 57(3):963-71.

Chan I. Exact tests of equivalence and efficacy with a non-zero lower bound for comparative studies. Statistics in Medicine 1998, 17:1403-13.

Clinical Evaluation Of QT/QTc Interval Prolongation And Proarrhythmic Potential For Non-Antiarrhythmic Drug, preliminary concept paper, FDA, draft 4 from 10 June 2004, printed out from http://www.fda.gov on 10 December 2007.

Grabenstein JD and Winkenwerder W Jr. US military smallpox vaccination program experience. JAMA 2003; 289(24): 3278-82.

Guidance for Industry E14 Clinical Evaluation of QT/QTc Interval Prolongation and Proarrhythmic Potential for Non-Antiarrhythmic Drugs, EMEA, November 2005, printed out from http://www.emea.eu.int on 10 December 2007.

Lane, J.M., Ruben, F.L., Neff, J.M., and Millar, J.D. Complications of smallpox vaccination, 1968: results of ten statewide surveys 1970; J.Infect.Dis., 122[4], 303-309.

Vaccines and Related Biological Products Advisory Committee Briefing Document, ACAM 2000, 18 April 2007, printed out from http://www.fda.gov.

16 APPENDICES

16.1 STUDY INFORMATION

16.1.1 Protocol, protocol amendments and file notes

POX-MVA-005
EudraCT No. 2005-001781-14
DMID 05-0128

A partially randomized, partially double-blind, placebo-controlled Phase II non-inferiority study to evaluate immunogenicity and safety of one and two doses of MVA-BN® (IMVAMUNE™) smallpox vaccine in 18-55 year old healthy subjects

PROPERTY OF BAVARIAN NORDIC A/S

CONFIDENTIAL

Site Signature page

Herewith I agree that I have read and fully understood this Protocol:

A partially randomized, partially double-blind, placebo-controlled Phase II non-inferiority study to evaluate immunogenicity and safety of one and two doses of MVA-BN® (IMVAMUNE™) smallpox vaccine in 18-55 year old healthy subjects Version 1.0, dated 12-Dec-2005, EudraCT No. 2005-001781-14, DMID 05-0128.

which reveals all the information necessary to conduct the study. I agree that I will conduct the study according to the instructions given by this protocol. Furthermore I agree that I will conduct this study according to ICH GCP, the current version of the declaration of Helsinki, Directive 2001/20/EC and the German Drug Law.

I agree that all information revealed in this protocol is handled strictly confidential.

Other than that I will permit trial related monitoring, audits, Ethics Committee review and regulatory inspections, providing direct access to source data/documents.

Dec 12 2005
Date

Prof. Frank von Sonnenburg, MD
Principal Investigator

Date

Stephan de la Motte, MD
Investigator

Site Signature page

Herewith I agree that I have read and fully understood this Protocol:

A partially randomized, partially double-blind, placebo-controlled Phase II non-inferiority study to evaluate immunogenicity and safety of one and two doses of MVA-BN® (IMVAMUNE™) smallpox vaccine in 18-55 year old healthy subjects Version 1.0, dated 12-Dec-2005, EudraCT No. 2005-001781-14, DMID 05-0128.

which reveals all the Information necessary to conduct the study. I agree that I will conduct the study according to the instructions given by this protocol. Furthermore I agree that I will conduct this study according to ICH GCP, the current version of the declaration of Helsinki, Directive 2001/20/EC and the German Drug Law.

I agree that all Information revealed in this protocol is handled strictly confidential.

Other than that I will permit trial related monitoring, audits, Ethics Committee review and regulatory inspections, providing direct access to source data/documents.

Date

Prof. Frank von Sonnenburg, MD
Principal Investigator

15 Dec 2005
Date

Stephan de la Motte, MD
Investigator

I. General information

Signature page

By signing the protocol:

A partially randomized, partially double-blind, placebo-controlled Phase II non-inferiority study to evaluate immunogenicity and safety of one and two doses of MVA-BN® (IMVAMUNE™) smallpox vaccine in 18-55 year old healthy subjects, Version 1.0, dated 12-Dec-2005, EudraCT No. 2005-001781-14, DMID 05-0128.

the undersigned parties agree, that the protocol was written according to international ethical and scientific quality standards (ICH-GCP), in compliance with Declaration of Helsinki, Directive 2001/20/EC and the German Drug Law.

15-Dez-2005<br>Date	Nadine Uebler<br>Nadine Uebler, M.Sc.<br>Protocol Author, Bavarian Nordic
15-Dez-2005<br>Date	[signature]<br>Jens Vollmar, M.D.<br>Medical Monitor, Bavarian Nordic
Date	Joachim Klinger<br>Director Data Management & Statistics,<br>Harrison Clinical Research
15 Dec 2005<br>Date	B L Cour<br>Birgitte Dornonville de la Cour<br>QA-Specialist, Bavarian Nordic
Dec 12 2005<br>Date	[signature]<br>Prof. Frank von Sonnenburg, MD<br>Principal Investigator

I. General information

Signature page

By signing the protocol:

A partially randomized, partially double-blind, placebo-controlled Phase II non-inferiority study to evaluate immunogenicity and safety of one and two doses of MVA-BN® (IMVAMUNE™) smallpox vaccine in 18-55 year old healthy subjects, Version 1.0, dated 12-Dec-2005, EudraCT No. 2005-001781-14, DMID 05-0128.

the undersigned parties agree, that the protocol was written according to international ethical and scientific quality standards (ICH-GCP), in compliance with Declaration of Helsinki, Directive 2001/20/EC and the German Drug Law.

Date	Nadine Uebler, M.Sc.<br>Protocol Author, Bavarian Nordic
Date	Jens Vollmar, M.D.<br>Medical Monitor, Bavarian Nordic
15 Dec 05<br>Date	J. Klinger<br>Joachim Klinger<br>Director Data Management & Statistics,<br>Harrison Clinical Research
Date	Birgitte Dornonville de la Cour<br>QA-Specialist, Bavarian Nordic
Date	Prof. Frank von Sonnenburg, MD<br>Principal Investigator

Responsibilities

**STUDY NUMBER**:	POX-MVA-005<br>DMID 05-0128
**STUDY TITLE**:	A partially randomized, partially double-blind, placebo-controlled Phase II non-inferiority study to evaluate immunogenicity and safety of one and two doses of MVA-BN® (IMVAMUNE™) smallpox vaccine in 18-55 year old healthy subjects.
**PRINCIPLE INVESTIGATOR**:	Prof. Frank von Sonnenburg<br>Section of International Medicine & Public Health<br>Department of Infectious Diseases and Tropical Medicine<br>Georgenstrasse 5<br>80799 Munich<br>Germany
Phone:	+49-89-2180 3830
Fax:	+49-89-33 60 38
E-mail:	sonnenburg@lrz.uni-muenchen.de
**STUDY SITE**	Harrison Clinical Research GmbH<br>Clinical Unit<br>Stephan de la Motte, MD<br>Hilblestr. 54<br>80636 Munich<br>Germany
Phone:	+49-89-126680-1210
Fax:	+49-89-126680-1444
**SPONSOR and PRODUCT SUPPLY**	Bavarian Nordic A/S<br>Bøgeskovvej 9<br>DK-3490 Kvistgård<br>Denmark
Phone:	+45-33-268383
Fax:	+45-33-268380

MEDICAL MONITOR: Jens Vollmar, M.D.
Bavarian Nordic GmbH
Fraunhoferstrasse 13
82152 Martinsried
Germany
Phone: +49-89-85651308
Fax: +49-89-85651333
E-mail: jens.vollmar@bavarian-nordic.com

PROJECT COORDINATOR: Nadine Uebler, M.Sc.
Bavarian Nordic GmbH
Fraunhoferstrasse 13
82152 Martinsried
Germany
Phone: +49-89-85651323
Fax: +49-89-85651333
E-mail: nadine.uebler@bavarian-nordic.com

STUDY STATISTICIAN(S): Joachim Klinger
Harrison Clinical Research GmbH
Albrechtstrasse 14
80636 Munich
Germany
Phone: +49-89-12 66 80-2190
Fax: +49-89-12 66 80-2444
E-mail: JKlinger@harrison-cro.com

Niels Holger Wulff, M.Sc.
Bavarian Nordic GmbH
Fraunhoferstrasse 13
82152 Martinsried
Germany
Phone: +49-89-85651366
Fax: +49-89-85651333
E-mail: niels.wulff@bavarian-nordic.com

CRO/PROJECT MANAGER: Sabine Zillikens
Harrison Clinical Research GmbH
Albrechtstrasse 14
80636 Munich
Germany
Phone: +49-89-12 66 80-2120
Fax: +49-89-12 66 80-2444
E-mail: szillikens@harrison-cro.com

CENTRALIZED ECG PROVIDER: Dr. Ulrich Herzfeld
ganiMed GmbH
Munzinger Strasse 1
79111 Freiburg
Germany
Phone: +49-761-120 666 3
Fax: +49-761-120 666 4
E-mail: info@ganimed.biz

LABORATORY: **(SAFETY)** Dr. Herrmann Schulz
INTERLAB GmbH
Bayerstrasse 53
80335 Munich
Germany
Phone: +49 89 741 393-0
Fax: +49 89 741 393-39
E-mail: Dr.Schulz@interlab.de

LABORATORY (IMMUNOLOGY) Nicole Bädeker, Ph.D.
Bavarian Nordic GmbH
Fraunhoferstrasse 13
82152 Martinsried
Germany
Phone: +49-89-85651386
Fax: +49-89-85651333
E-mail: nicole.baedeker@bavarian-nordic.com

Table of contents

I. GENERAL INFORMATION 3

SIGNATURE PAGE 3

II. PROTOCOL SYNOPSIS 11

III. FLOW CHART 18

IV. SCIENTIFIC SECTION 19

1. Introduction 19
1.1. Background information 19
1.2. Origin and characteristics of MVA 20
1.3. Summary of Preclinical Data 21
1.4. Summary of Clinical Data 22
1.5. Dose, route of administration, vaccination schedule 28
1.6. Benefit/risk assessment 28
2. Objectives 29
3. Study design 29
3.1. Experimental design 29
3.2. Discussion of study design and choice of control groups 30
3.3. Description of study procedures 31
3.4. Study duration 35
4. Selection of subjects 35
4.1. Recruitment procedure 35
4.2. Inclusion criteria 35
4.3. Exclusion criteria 36
5. Study halting/termination and withdrawal of subjects 37
5.1. Study halting and termination rule 37
5.2. Reporting of events fulfilling the study halting criteria 38
5.3. Safety Monitoring Committee (SMC) 38
5.4. Individual withdrawal criteria during the study 38
5.5. Subject withdrawal procedure 39
5.6. Contraindications and precautions for further study vaccinations 39
6. Study treatment 40
6.1. Investigational product 40
6.2. Placebo 40
6.3. Packaging and labeling 40
6.4. Vaccine storage, handling and dispensing 40
6.5. Dose, vaccination schedule and route of administration 41
6.6. Accountability and disposal 41
6.7. Concomitant medication 41
7. Clinical and laboratory assessments 41
7.1. Assessment of safety and reactogenicity 41
7.2. Assessment of immunogenicity: Antibody Response 48
8. Adverse event definitions and reporting 49
8.1. Definition of adverse event 49
8.2. Definition of solicited adverse events 49
8.3. Definition of unsolicited adverse events 49
8.4. Definition of “adverse event of special interest” 49
8.5. Definition of serious adverse events (SAEs) 50
8.6. Reporting of SAEs and adverse events of special interest 50
9. Statistical Considerations 51

9.1. Sample size calculation 51
9.2. Variables 52
9.3. Study cohorts/data sets to be evaluated 53
9.4. Statistical analysis 54
10. Ethical aspects 57
10.1. Ethical and legal regulations 57
10.2. Approval of an ethics committee 57
10.3. Confidentiality and data protection 57
11. Informed consent 58
12. Case report forms and retention of records 59
12.1. Electronic case report forms 59
12.2. Retention of records 59
13. Monitoring of the study 60
14. Responsibilities of the investigator 60
15. References 62
16. Appendices 64

List of abbreviations

AE	Adverse Event
AIDS	Acquired Immunodeficiency Syndrome
ALT	Alanine aminotransferase
AST	Aspartate aminotransferase
CD	Cluster of differentiation
CDISC	Clinical Data Interchange Standards Consortium
CEF	Chicken embryo fibroblast
CI	Confidence interval
CRF(s)	Case Report Form(s); can either be hardcopy or an electronic document
eCRF	Electronic CRF
eSource	Source data captured initially into a permanent electronic record
CRO	Contract Research Organization
CVA	Chorioallantois vaccinia virus Ankara
DMID	Division of Microbiology and Infectious Diseases, National Institute of Allergy and Infectious Diseases
ECG	Electrocardiogram
ELISA	Enzyme-linked Immunosorbent Assay
FU	Follow-up
GMT	Geometric mean titer
HIV	Human immunodeficiency virus
ICH	International Conference of Harmonization of Technical Requirements for Registration of Pharmaceuticals for Human Use
ICS	Intracellular Cytokine Staining
IgG	immunoglobulin G
i.d.	Intra-dermal
IFN	Interferon
i.m.	Intra-muscular
i.t.	Intra-tracheal
FA	Full Analysis
LLN	Lower Limit of Normal
LMU	Ludwig Maximilian University of Munich
MedDRA	Medical Dictionary for Regulatory Activities
MVA	Modified Vaccinia Ankara Strain
MVA-BN®	Modified Vaccinia Ankara – Bavarian Nordic

n/N	Number
NCI	National Cancer Institute
NYCBH	New York City Board of Health
ODM	Operational data model
PP	Per protocol
PRNT	Plaque reduction neutralization test
PVC	Premature ventricular contractions
SAE	Serious Adverse Event
s.c.	Subcutaneous
SCR	Screening
SMC	Safety Monitoring Committee
ST	ST segment in electrocardiogram
$TCID_{50}$	Tissue culture infectious dose 50
ULN	Upper Limit of Normal
V	Visit
VV	Vaccinia Virus
WBC	White Blood Cell Count
WHO	World Health Organization

II. Protocol Synopsis

TITLE	A partially randomized, partially double-blind, placebo-controlled Phase II non-inferiority study to evaluate immunogenicity and safety of one and two doses of MVA-BN® (IMVAMUNE™) smallpox vaccine in 18-55 year old healthy subjects.
SPONSOR	Bavarian Nordic A/S<br>Bøgeskovvej 9<br>3490 Kvistgård<br>Denmark
PRINCIPAL INVESTIGATOR	Prof. Frank von Sonnenburg<br>Section of International Medicine & Public Health<br>Department of Infectious Diseases and Tropical Medicine<br>Georgenstrasse 5<br>80799 Munich<br>Germany
Phone:	+49-89-2180 3830
Fax:	+49-89-33 60 38
E-mail:	sonnenburg@lrz.uni-muenchen.de
PROJECT PHASE	Phase II
INVESTIGATIONAL DRUG	MVA-BN® liquid-frozen, containing 1 x 10^8 $TCID_{50}$ Modified Vaccinia virus Ankara per 0.5 ml dose.
PLACEBO	0.5 ml Tris Buffer<br>(1.21 g/l Tris, 8.526 g/l sodium chloride, 19.2 g/l Dextran FP-40, 45.2 g/l Sucrose, 0.108 g/l L-Glutamic acid monopotassium salt, pH 7.5)
VACCINE DOSAGE, TIMING AND ROUTE OF ADMINISTRATION	**Group 1:**<br>200 vaccinia naive subjects receive two s.c.vaccinations with 0.5 ml MVA-BN® vaccine containing 1 x 10^8 $TCID_{50}$<br><br>**Group 2:**<br>200 vaccinia naive subjects receive first vaccination with 0.5 ml MVA-BN® vaccine containing 1 x 10^8 $TCID_{50}$ and second vaccination with placebo (0.5 ml Tris Buffer)

Group 3:
200 vaccinia naive subjects receive two s.c. vaccinations with placebo (0.5 ml Tris Buffer)

Group 4:
200 vaccinia non-naive subjects receive one s.c. vaccination with 0.5 ml MVA-BN® vaccine containing 1 x 10^8 $TCID_{50}$

PRIMARY IMMUNOGENICITY OBJECTIVE

To compare the ELISA specific humoral immune response of the group with history of smallpox vaccination (Group 4: 1 dose MVA-BN®) versus the group without history of smallpox vaccination (Group 1: 2 doses MVA-BN®), i.e. to prove that a single dose schedule would be sufficient to boost the immunity in a previously vaccinated population.

PRIMARY SAFETY OBJECTIVE

To compare the four different vaccination groups with regard to ECG changes and cardiac symptoms.

SECONDARY OBJECTIVES

IMMUNOGENICITY

To compare the ELISA specific immune response of the four different vaccination groups - comparison combinations not done for the primary objective.

To compare the four different vaccination groups with regard to immune response measured with a plaque reduction neutralization test.

To assess the kinetics of the immune responses after 1 (Group 2) and 2 (Group 1) doses of MVA-BN® in vaccinia naïve subjects.

SAFETY

To compare the four different vaccination groups with regard to safety and reactogenicity.

To compare the safety of one (Group 2) and two (Group 1) vaccinations with MVA-BN® in vaccinia-naïve subjects with placebo (Group 3) and historical / recently published data of Dryvax® immunization in vaccinia-naive subjects.

STUDY DESIGN

Partially randomized, partially double-blind, placebo-controlled, 4 study groups, non-inferiority trial:

Double-blinded:

Group 1: Subjects without history of smallpox vaccination (2 doses MVA-BN®)

Group 2: Subjects without history of smallpox vaccination (1st dose MVA-BN® / 2nd dose placebo)

Group 3: Subjects without history of smallpox vaccination (2 doses placebo)

Open-label:

Group 4: Subjects with history of smallpox vaccination (1 dose MVA-BN®)

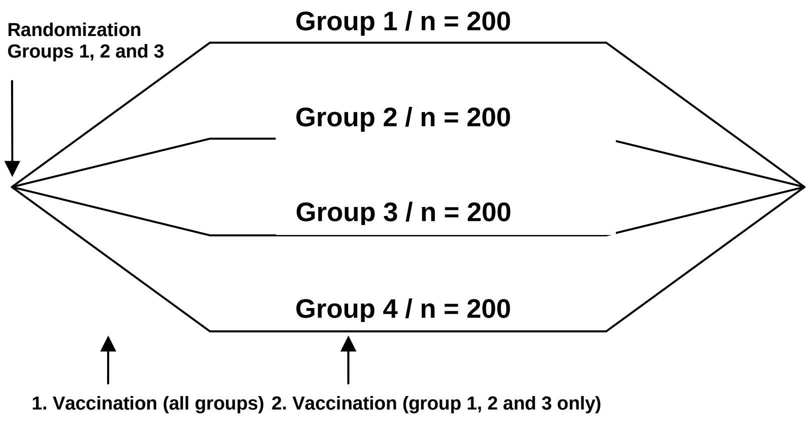

Visit Days (Groups 1, 2, 3)

-28	0	10-15	28 – 35	V3 + 10-15	V3 + 28-35	V3 + 182–210
Scr	V1	V2	V3	V4	V5	F-U

Visit Days (Group 4)

-28	0	10-15	28 - 35	V1 + 182–210
Scr	V1	V2	V3	F-U

STUDY DURATION

Groups 1/2/3: Up to 39 weeks for each subject
Groups 4: Up to 34 weeks for each subject

PLANNED TOTAL SAMPLE SIZE

The sample size is 800 (200 subjects per group). It is expected that 175 subjects per group are evaluable.

INCLUSION CRITERIA

All groups

1. Male and female subjects between 18 and 55 years of age.
2. Women must have a negative serum pregnancy test at screening and a negative urine or serum pregnancy test within 24 hours prior to vaccination.
3. Women of childbearing potential must have used an acceptable method of contraception for 30 days prior to the first vaccination, must agree to use an acceptable method of contraception during the study, and must not become pregnant for at least 28 days after the last vaccination. A woman is considered of childbearing potential unless post-menopausal or surgically sterilized. (Acceptable contraception methods are restricted to abstinence, barrier contraceptives, intrauterine contraceptive devices or licensed hormonal products.)
4. Read, signed and dated informed consent document after being advised of the risks and benefits of the study in a language understood by the subject signed, and prior to performance of any study specific procedure.
5. Troponin I within normal institutional limits.
6. White blood cells ≥ 2500/mm^3 and ≤ 11,000/ mm^3.
7. Absolute neutrophil count (ANC) ≥ 1000/mm^3.
8. Negative urine glucose by dipstick or urinalysis.
9. Hemoglobin ≥ LLN.
10. Platelets 100 – 440/nl.
11. Adequate renal function defined as:
    a. Serum creatinine without clinically significant findings.
    b. Urine protein ≤ 30 mg/dL or none or trace proteinuria (by urinalysis or dip stick).
12. Adequate hepatic function defined as:
    a. Total bilirubin ≤ 1.5 x ULN in the absence of other evidence of significant liver disease (healthy subjects without clinical disease with Morbus Meulengracht can be included).
    b. AST (SGOT), ALT (SGPT) and alkaline phosphatase without clinically significant findings.
13. Electrocardiogram (ECG) without abnormal findings (e.g. any kind of atrioventricular or intraventricular conditions or blocks such as complete left or right bundle branch block, AV-node block, QTc or PR prolongation, premature atrial contractions or other atrial arrhythmia, sustained ventricular arrhythmia, 2 premature ventricular contractions (PVC) in a row, ST elevation consistent with ischemia).
14. Availability for follow-up for the planned duration of the study (26 weeks after last vaccination).

Groups 1, 2 and 3 (All vaccinia-naïve subjects) additionally:

1. No history of known or suspected previous smallpox vaccination.
2. No detectable vaccinia scar.

Group 4 (All previously vaccinated subjects) additionally:

1. History of previous smallpox vaccination (documented and/or typical vaccinia scar).
2. Most recent smallpox vaccination ≥ 5 years.

EXCLUSION CRITERIA

Groups 1, 2, 3, and 4 (All subjects):

1. Pregnant or breast-feeding women.
2. Uncontrolled serious infection i.e. not responding to antimicrobial therapy.
3. History of any serious medical condition, which in the opinion of the investigator would compromise the safety of the subject.
4. History of or active autoimmune disease. Persons with vitiligo or thyroid disease taking thyroid replacement are not excluded.
5. Known or suspected impairment of immunologic function including, but not limited to, clinically significant liver disease; diabetes mellitus; moderate to severe kidney impairment.
6. History of malignancy, other than squamous cell or basal cell skin cancer, unless there has been surgical excision that is considered to have achieved cure. Subjects with history of skin cancer at the vaccination site are excluded.
7. History or clinical manifestation of clinically significant and severe hematological, renal, hepatic, pulmonary, central nervous, cardiovascular or gastrointestinal disorders.
8. Clinically significant mental disorder not adequately controlled by medical treatment.
9. Any condition which might interfere with study objectives or would limit the subject's ability to complete the study or to be compliant in the opinion of the investigator.
10. History of coronary heart disease, myocardial infarction, angina, congestive heart failure, cardiomyopathy, stroke or transient ischemic attack, uncontrolled high blood pressure, or any other heart condition under the care of a doctor.
11. History of an immediate family member (father, mother, brother, or sister) who died due to ischemic heart disease before age 50 years.
12. Ten percent or greater risk of developing a myocardial infarction or coronary death within the next 10 years using the National Cholesterol Education Program's risk assessment tool. (http://hin.nhlbi.nih.gov/atpiii/calculator.asp?usertype=prof) NOTE: This criterion applies only to volunteers 20 years of age and older.
13. History of intravenous drug abuse.
14. History of allergic disease or reactions likely to be exacerbated by any component of the vaccine.
15. Known allergy to egg or aminoglycoside (gentamicin).
16. History of anaphylaxis or severe allergic reaction.
17. Having received any vaccinations or planned vaccinations with a live vaccine within 30 days prior or after study vaccination.
18. Having received any vaccinations or planned vaccinations with a killed vaccine within14 days prior or after study vaccination.
19. Chronic administration (defined as more than 14 days) of systemic immuno-suppressant drugs during a period starting from six months prior to administration of the vaccine and ending at study conclusion. (Corticosteroid nasal sprays are permissible. Persons who have used topical and inhaled steroids can be enrolled after their therapy is completed).
20. Post organ transplant subjects whether or not receiving chronic immunosuppressive therapy.
21. Administration or planned administration of immunoglobulins and/or any blood products during a period starting from 3 months prior to administration of the vaccine and ending at study conclusion.
22. Use of any investigational or non-registered drug or vaccine other than the study vaccine within 30 days preceding the first dose of the study vaccine, or planned administration of such a drug during the study period.
23. Study personnel.

PRIMARY IMMUNOGENICITY VARIABLE

MVA -specific seroconversion rate derived from the ELISA specific antibody titers 2 weeks after the last vaccination (Group 1-3: Visit 4, Group 4: Visit 2).
Seroconversion is defined as the appearance of antibody titers ≥ 1:50 in a vaccinia specific IgG ELISA for initially seronegative subjects or twofold increase of the antibody titer compared to the pre-existing baseline titer for subjects with a pre-existing antibody titer in the ELISA.

PRIMARY SAFETY VARIABLES

Occurrence, relationship and intensity of any specific or unspecific ECG change at any time during the study.

Occurrence, relationship and intensity of any other cardiac symptom at any time during the study.

SECONDARY VARIABLES

IMMUNOGENICITY

MVA -specific seroconversion rate derived from the ELISA specific antibody titers 4 weeks after the last vaccination (Group 1-3: Visit 5, Group 4: Visit 3).

MVA -specific seroconversion rate derived from the PRNT specific antibody titers 2 weeks after the last vaccination (Group 1-3: Visit 4, Group 4: Visit 2) and 4 weeks after the last vaccination (Group 1-3: Visit 5, Group 4: Visit 3).
Seroconversion is defined as appearance of antibody titers ≥ 1:20 in a vaccinia specific plaque reduction neutralization assay for initially seronegative subjects or twofold increase of the antibody titer compared to the pre-existing baseline titer for subjects with a pre-existing antibody titer in the PRNT.

SAFETY

Occurrence of any serious adverse event probably, possibly or definitely related to the study vaccine at any time during the study.

Occurrence of any grade 3 or higher adverse reaction probably, possibly or definitely related to the study vaccine within 28 days after vaccination.

Occurrence of solicited local adverse events within 1 week (Days 0-7) after vaccination: Intensity, duration, and relationship to vaccination.

Occurrence of solicited general adverse events within 1 week (Days 0-7) after vaccination: Intensity, duration, and relationship to vaccination.

Occurrence of unsolicited non-serious adverse events within 28 days after vaccination: Intensity, duration and relationship to vaccination.

STATISTICAL CONSIDERATIONS

The sample size calculation is based on the primary immunogenicity variable '**MVA -specific seroconversion rate'**. This parameter is derived from the ELISA specific antibody titers.

The primary hypothesis is to show that the humoral immune response of the group with history of smallpox vaccination (Group 4) is not statistically inferior compared to the group without history of smallpox vaccination (Group 1). The study should demonstrate that the Group 4 seroconversion rate is not worse than the Group 1 seroconversion rate by more than a pre-specified amount. This amount is called the non-inferiority margin (Δ).

Suppose p_1 is the seroconversion rate in the group without history of smallpox vaccination and p_4 is the seroconversion rate in the subjects with history of smallpox vaccination.
The test on non-inferiority will be applied for the following hypothesis:

H_0: $p_4 - p_1 \leq -\Delta$ versus H_1: $p_4 - p_1 > -\Delta$,
where

Δ is the non-inferiority margin and is chosen in this trial as 5%.
From the experience in a pilot trial with MVA-BN® in healthy subjects it is anticipated that the seroconversion rate in healthy vaccinia-naive subjects reaches 98-100%.

Assuming a significance level of 5%, a power of 80%, expected seroconversion rates of 98% in both groups, this yields to a sample size of 175 per group (700 in total). In order to account for drop outs, **a total of 200 subjects per group (800 in total)** will be treated.

III. Flow Chart

GROUP 1, 2 AND 3 (SUBJECTS WITHOUT HISTORY OF SMALLPOX VACCINATION: 2 DOSES OF MVA-BN® OR 1 DOSE MVA-BN® / 1 DOSE PLACEBO OR 2 DOSES PLACEBO)

VISIT (V)	SCR	V 1	V 2	V 3	V 4	V 5	V F-U
	DAY -28- -1	DAY 0	V 1 + 10-15 DAYS	V 1 + 28-35 Days	V 3 + 10-15 DAYS	V 3 + 28-35 DAYS	V 3 + 182-210 DAYS
Informed consent	X						
Check incl. / excl. criteria	X	X		X			
Medical History	X						
Vaccination		I		II			
Phys. exam incl. vital signs	X	X	X	X	X	X	X
ECG	X		X		X	(X)[2]	(X)[2]
Pregnancy test[1]	X	X		X		X	
Urine analysis	X						
Safety Laboratory	X		X		X	(X)[2]	(X)[2]
Antibody analysis		X	X	X	X	X	X
Handout of diary card[3]		X		X			
Collection of diary card			X		X		
AE-reporting		X	X	X	X	X	X[4]
Review baseline signs & symptoms	X	X					
Review prior / concomitant medication	X	X	X	X	X	X	
Blood sampling in ml[5]	15	8	23	8	23	8 (+15 ML)[2]	8(+15 ML)[2]

GROUP 4 (SUBJECTS WITH HISTORY OF SMALLPOX VACCINATION): 1 DOSE OF MVA-BN®

VISIT (V)	SCR	V 1	V 2	V 3	V F-U
	DAY -28 - 1	DAY 0	V1 + 10-15 DAYS	V 1 + 28-35 DAYS	V 1 + 182-210 DAYS
Informed consent	X				
Check incl. / excl. criteria	X	X			
Medical History	X				
Vaccination		I			
Phys. exam incl. vital signs	X	X	X	X	X
ECG	X		X	(X)[2]	(X)[2]
Pregnancy test[1]	X	X		X	
Urine analysis	X				
Safety Laboratory	X		X	(X)[2]	(X)[2]
Antibody analysis		X	X	X	X
Handout of diary card[3]		X			
Collection of diary card			X		
AE-reporting		X	X	X	X[4]
Review baseline signs & symptoms	X	X			
Review concomitant medication	X	X	X	X	
Blood sampling in ml[5]	15	8	23	8	8 (+15 ML)[2]

[1] Urine or serum pregnancy test, women of childbearing potential only. At screening visit, a serum test must be performed.
[2] Only required if clinically indicated.
[3]The Diary Card should be completed daily for 7 days. If symptoms persist at day 7, temperature/symptom measurements should be recorded each day until resolved.
[4] Serious adverse events only.
[5] Amount of single blood draws: Safety laboratory: 15 ml; Antibody analysis: 8 ml. Total amount of blood taken during study: Up to 123 ml for vaccinia naïve subjects; up to 77ml for preimmune subjects

IV. Scientific Section

1. Introduction

1.1. Background information

In Germany during the period from 1960 to 1974, Professor Anton Mayr succeeded in attenuating the dermal vaccinia strain Ankara (CVA) by over 500 continuous passages in primary chicken embryo fibroblast cultures (CEF). The rationale was to attenuate the smallpox vaccine so that the complications associated with vaccination could be reduced or completely eliminated and make safer parenteral vaccination possible. This highly attenuated virus of CVA was named MVA (modified vaccinia Ankara). It was demonstrated that it had retained its original immunogenicity and its protective effect against variola and general Orthopox infections and offers a greatly diminished virulence for humans and animals combined with the loss of contagiousness. It was shown that vaccination-induced disease does not occur in newborn animals (mice, rabbits, and chickens) and immunosuppressed vaccinated animals (by radiation, iatrogenic immune suppression). Local (cutaneous, oral, and intranasal) and parenteral (subcutaneous, intramuscular) administration of MVA are both possible, safe and retain immunizing efficacy (Mayr & Munz 1964; Stickl *et al.* 1974; Stickl, Hochstein-Mintzel, Mayr, Huber, Schafer, & Holzner 1974; Mayr *et al.* 1975).

In Germany, the MVA strain was officially authorized for two-stage parenteral smallpox vaccination in children in 1976. It was used in approximately 120,000 Caucasian individuals with no reported side effects, even though many of the subjects were among the population with high risk of developing complications (Mayr *et al.* 1978).

In May 1980, the World Health Organization (WHO) declared the global eradication of smallpox. Since 1982, smallpox vaccination has not been required for international travelers' vaccination and International Certificates of Vaccination no longer include smallpox. As smallpox vaccination was deleted from the vaccination schedules, production of the vaccine was no longer required.

Despite the fact that the WHO officially declared smallpox eradicated in 1980, a new threat exists from the use of variola in biological warfare and/or bio-terrorism. Indeed, as mass vaccinations programs halted more than 20 years ago, most people have no existing immunity to smallpox, and as such the release of this highly contagious virus would have devastating effects. As a consequence, there is an urgent need by many Governments for a safe efficacious vaccine to protect the public against smallpox.

The original smallpox vaccines were based on a number of different vaccinia strains e.g. Lister-Elstree strain recommended by the WHO and used in Germany or the New York City Board of Health (NYCBH) strain used in the United States. While these proved to be highly effective immunizing agents they also showed considerable side effects. Besides from local reactions with scab development and scarring, general symptoms observed frequently after smallpox vaccination have been fever, weakness, muscular pain, headache, swelling and soreness of local lymph nodes and rashes. Fever occurred in the majority of vaccinees, especially in small children: up to 70% got temperatures of 38°C, 15-20% showed even higher rates. Apart from less dramatic and transient side effects like erythematous or urticarial rashes, severe and potentially fatal cutaneous complications of vaccinia vaccination include eczema vaccinatum and progressive vaccinia. Most feared are complications of the central nervous system, especially post-vaccinal encephalitis, followed in 15-25% by death and in 25% by neurological sequelae (Goldstein *et al.* 1975;Lane *et al.* 1969;Lane *et al.* 1970). In Germany, the occurrence of neurological complications in primary vaccinees was

reported in 1:20,000 to 30,000 vaccinees and while other countries such as the United States excluded high-risk individuals from vaccination, an average of seven persons a year still died from complications due to smallpox vaccination (McElwain 1972). Another consideration for the discontinued use of the classical smallpox vaccine is the world prevalence of HIV and AIDS. The classical replication competent smallpox vaccine could be lethal if given to immune compromised individuals. A study published in 1991 (Guillaume *et al.* 1991) reported the case of two HIV infected individuals that received an experimental immunotherapy in the form of paraformaldehyde fixed autologous T cells previously infected with recombinant vaccinia viruses. Both these patients were immune compromised and experienced necrotic skin lesions due to generalized vaccinia infections that led to death. However, complications can also occur in HIV infected individuals that have a good T cell count and appear healthy following vaccinations with vaccinia (Redfield *et al.* 1987) demonstrating the severe implications of a wide spread use of vaccinia based vaccines.

Bavarian Nordic's proprietary Modified Vaccinia Ankara vector (MVA-BN®) is a live attenuated pox virus derived from Anton Mayr's seed virus used to vaccinate more than 120,000 people in Germany during the smallpox vaccination program in the late seventies. This vaccine possesses a number of characteristics that makes it highly suitable for prophylactic and therapeutic vaccination. The vaccine has a superior safety record in comparison to other viruses. The vaccine's immunogenic potential has been demonstrated in animal studies and is known from data in humans vaccinated during the two-step vaccination program in Germany (Mayr et al. 1978).

Bavarian Nordic has established a state of the art cGMP (current Good Manufacturing Practice) rated production process for MVA-BN® as well as for MVA-BN® derived recombinant viruses.

1.2. Origin and characteristics of MVA

Vaccinia virus (VV) is considered the best known member of the poxvirus family and the prototype live viral vaccine. VV replicates in the cytoplasm of the host cell, its DNA does not integrate into the host cell genome and it is non-oncogenic.

MVA was derived from the VV strain Ankara by 574 serial passages in CEF. From passage 530 the virus is named MVA and has a stabilized immunogenicity, extremely reduced transmission and no induction of a carrier status. During passaging, MVA has suffered a multitude of mutations within its genome and six major deletions resulting in the loss of 15% (30kbp) of original genetic information (Antoine *et al.* 1998). The deletions affect a number of virulence and host range genes as well as the gene for the Type A inclusion bodies (Antoine, Scheiflinger, Dorner, & Falkner 1998;Rosel *et al.* 1986;Meyer *et al.* 1991). As a consequence, MVA exhibits a severely restricted host range, and replicates only very poorly, if at all, in most mammalian cell types, including primary human cells and most transformed human cell lines (Sutter & Moss 1992;Carroll & Moss 1997;Blanchard *et al.* 1998;Drexler *et al.* 1998).

Although MVA exhibits a strongly attenuated replication in these cell types its genes are efficiently transcribed, with the block in viral replication being at the level of virus assembly and egress (Sutter & Moss 1992; Carroll & Moss 1997). Genetic reconstitution of one of the deletions in the MVA genome affecting, besides other genes, the VV host range gene K1L restored MVA replication in some mammalian cell lines, but failed to restore the growth capacity of MVA in human cells (Meyer et al. 1991; Carroll & Moss 1997).

Taken together with the loss of 15% of its genome, MVA is therefore unlikely to be able to spontaneously regain its replication competency following injection into humans. Despite its high attenuation and reduced virulence, MVA has retained its stable immunogenic properties and variola-protective effect.

MVA is a potent inducer of type I interferon (IFN) in human cells and it expresses a soluble interleukin-1 receptor (Blanchard et al. 1998), which has been implicated as an anti-virulence factor for certain poxviruses (Alcami & Smith 1992;Alcami & Smith 1996). In contrast to VV, MVA does not express soluble receptors for IFN γ, IFN α/β, tumor necrosis factor and CC chemokines (Antoine et al. 1998; Blanchard et al. 1998). MVA is known to have protective immunogenic potential against any kind of orthopox virus.

Taken together with its restricted host range these factors may explain the avirulent phenotype observed for MVA *in vivo* in a wide variety of mammalian species including humans (see below). As a consequence, MVA was used in more than 120,000 human subjects as part of a two-step vaccination protocol, combined with conventional vaccinia virus, against smallpox in Germany (Mayr et al. 1978).

MVA-BN® is a highly attenuated, purified live vaccine produced under serum-free conditions in CEF cells. Extensive in-house studies have demonstrated that MVA-BN® has superior characteristics compared to other MVA strains, which include:

- MVA-BN® has a superior attenuation compared to other MVA strains that replicate in various human cells.
- MVA-BN® vaccinations are safe in severely immune compromised animals, whereas other MVA strains replicate resulting in pathology and death.
- MVA-BN® has a stable genotype, whereas other MVA strains rapidly lose their attenuation when cultured on mammalian cells.

Therefore, it can be concluded that MVA-BN® is a promising safe human smallpox vaccine candidate.

1.3. Summary of Preclinical Data

Bavarian Nordic has performed an extensive preclinical development program for MVA-BN® that has demonstrated the safety, efficacy and bio-equivalence of MVA-BN® compared to other traditional smallpox vaccines (e.g. Dryvax® and MVA-571). Bavarian Nordic has also developed challenge models including an intratracheal monkeypox challenge model and two murine orthopox challenge models. Some key features include:

- MVA-BN® has a superior attenuation compared to other MVA strains that replicate in various human cells.
- MVA-BN® vaccinations are safe in rabbits with minimal and (in case of subcutaneous administration: “potentially”) reversible effects.
- Administration of MVA-BN® resulted in the detection of vaccine transcripts in distinct tissues with clearance of those transcripts within 7 days (with only the injection site skin and muscle being positive to a low frequency).
- MVA-BN® vaccinations are safe in severely immune compromised animals, whereas other MVA strains replicate resulting in disease outbreak and death.
- MVA-BN® demonstrated a superior efficacy in a lethal Vaccinia virus challenge model in mice compared to other MVA strains.

- MVA-BN® vaccinations induce the same efficacy as traditional smallpox vaccine (e.g. Dryvax®) against a lethal orthopox challenge given via various routes (intraperitoneal and intranasal).
- A single vaccination with MVA-BN® induces equivalent protection in mice following intranasal challenge with high lethal doses of Ectromelia virus compared to a traditional smallpox vaccine (Dryvax®).
- MVA-BN® vaccinated mice are protected from a lethal Vaccinia virus challenge applied within 4 days, whereas Dryvax vaccinated mice are not protected.
- MVA-BN® is a potent inducer of both antibody and T cells responses and induces equivalent levels of immunity as traditional smallpox vaccines (Dryvax® and Elstree) in both murine and non-human primates.
- BN has developed the first intra-tracheal (i.t.) monkeypox challenge model that more closely mimics the natural route of a smallpox infection and results in a smallpox-like disease.
- Non-human primates vaccinated with MVA-BN® are protected from a lethal i.t. monkeypox challenge demonstrating an equivalent protection to traditional smallpox vaccines (Dryvax®).
- BN is in the process of validating the monkeypox and selected murine orthopox virus challenge models according to FDA approved protocols.

For more detailed information on preclinical data please refer to the respective section of the investigator brochure.

1.4. Summary of Clinical Data

1.4.1. Clinical Experience with the MVA precursor vaccine

Non-recombinant MVA has been extensively used in humans, forming part of a two-step smallpox vaccination program in combination with wild-type vaccinia virus in Germany in 1978. More than 120,000 human subjects received i.d. and s.c. injections with MVA as part of this program, with the majority of subjects being children and adults at risk for adverse reaction from the wild-type vaccine without prior immunization with an avirulent MVA (Mahnel & Mayr 1994;Mayr, Stickl, Muller, Danner, & Singer 1978;Stickl, Hochstein-Mintzel, Mayr, Huber, Schafer, & Holzner 1974; Stickl & Hochstein-Mintzel 1971). Mild local reactions including local reddening of the skin and infiltration were observed at the site of injection in ~75% of individuals vaccinated by the intradermal route (Mayr et al. 1978). Side effects on the injection site after MVA vaccination seen in 5308 vaccinia-naive individuals (0.2 ml intradermally in the forearm) are shown in table I.

Table I: Local reactogenicity 24-48 hours and 5-7 days after vaccination with MVA

LOCAL REACTION	24-48 HOURS	5-7 DAYS
No reaction	7.25%	9.17%
Reddening up to 10 mm in diameter	54.9%	75.45%
Reddening 10-20 mm in diameter	31.45%	14.05%
Reddening more than 20 mm in diameter	6.40%	1.53 %

No blistering, pustula or ulceration were observed at virus doses that produce such reactions in approximately 50% of naive individuals after application of conventional vaccinia. This indicated that the virulence of MVA for skin is very low. No encephalitic reaction was observed (Stickl et al. 1974).

Approximately 2-4% of the subjects (n = 7098) had slight fever and felt unwell (fever > 38°C in 2.28%, non-specific systemic symptoms in 4.11%), but the symptoms were much less than those observed with the normal smallpox vaccine. No serious adverse events were reported. In addition, vaccination with MVA before administering the normal smallpox vaccine (replication competent wild-type vaccinia virus) resulted in a reduced number of side effects from this administration and the development of smaller wild-type vaccinia-derived pocks.

Single dose vaccination with MVA elicited only weak hemagglutination inhibiting or virus neutralizing antibody response. However, after vaccination with wild-type vaccinia virus vaccinees presented with a strong booster effect, indicating the priming by MVA with specific humoral and cellular immune response (Mayr et al. 1978). In vaccinees having received a second dose of MVA, an increased level of antibody response was observed as well. It could be shown in animal experiments that protection against variola was provided even in the absence of an antibody response (Stickl et al. 1974).

1.4.2. Clinical Experience with MVA-BN®

To date, 11 clinical studies with MVA-based vaccines have been initiated and more than 500 subjects were vaccinated. The populations vaccinated include risk groups for which conventional smallpox vaccines are contraindicated such as HIV infected patients and patients with atopic dermatitis. The majority of the vaccinated subjects (401) received MVA-BN® and 115 subjects were vaccinated with several other recombinant MVA-based vaccines.

Vaccination of healthy population with MVA-BN®

Study POX-MVA-001: 86 healthy volunteers were vaccinated in this clinical phase I trial conducted in Europe. The subjects were healthy males, stratified based on the presence or absence of a prior history of smallpox vaccination. Primary objective was to assess safety and tolerability of MVA-BN® with secondary objectives comparing various routes of administration (subcutaneous [s.c.] versus intramuscular [i.m.]) with three different doses of vaccine being tested. There were five cohorts, with three cohorts receiving the vaccine s.c. at a dose of 10^6, 10^7, and 10^8 tissue culture infectious dose 50 ($TCID_{50}$), respectively. A fourth cohort received the vaccine i.m. at a dose of 10^8 $TCID_{50}$. All four cohorts were naïve to smallpox vaccination and received vaccine at baseline and week 4. A fifth cohort of subjects, who had been previously vaccinated against smallpox, received a single dose of $1x10^8$ $TCID_{50}$ of MVA-BN® s.c. In this study the vaccine induced a strong and dose dependent immune response and was safe and well tolerated even at high doses.

Study POX-MVA-004: This double-blind randomized, phase II, dose finding trial in 165 healthy, vaccinia naïve male and female volunteers aged 18–32 years evaluated the immunogenicity and safety of MVA-BN® at doses of $2x10^7$, $5x10^7$ or $1x10^8$ $TCID_{50}$. Subjects were randomly assigned to one of three treatment groups and were immunized twice at a 4 week interval using MVA-BN® (s.c.). Results of PRNT and ELISA, detecting neutralizing antibodies and total IgG against vaccinia respectively are shown in Table II.

Table II: POX-MVA-004 Humoral immune response (per protocol population

			ELISA			PRNT		
**Group**	**Visit Day**	**N**	**n (%)**	**GMT**	**95% CI (LL / UL)**	**n (%)**	**GMT**	**95% CI (LL / UL)**
Group 1 (N=54) 2x10^{7} TCID$_{50}$	0	54	0 (0%)	1.00	- / -	0 (0%)	1.00	- / -
	28	54	32 (59.3%)	14.37	7.71 / 26.8	4 (7.4%)	1.31	1.01 / 1.70
	42	54	54 (100%)	377.22	288.33 / 493.53	23 (42.6%)	5.51	3.17 / 9.59
	84	54	51 (94.4%)	134.33	91.05 / 198.19	10 (18.5%)	1.94	1.31 / 2.85
Group 2 (N=49) 5x10^{7} TCID$_{50}$	0	49	0 (0%)	1.00	- / -	0 (0%)	1.00	- / -
	28	49	40 (81.6%)	53.21	29.86 / 94.87	6 (12.2%)	1.55	1.10 / 2.19
	42	49	49 (100%)	583.62	461.58 / 737.94	29 (59.2%)	10.31	5.78 / 18.40
	84	49	49 (100%)	227.76	176.40 / 294.07	11 (22.4%)	2.32	1.47 / 3.66
Group 3 (N=52) 1x10^{8} TCID$_{50}$	0	52	0 (0%)	1.00	- / -	0 (0%)	1.00	- / -
	28	52	49 (94.2%)	98.52	67.57 / 143.65	5 (9.6%)	1.39	1.05 / 1.85
	42	52	52 (100%)	813.77	628.74 / 1053.26	37 (71.2%)	19.43	11.05 / 34.16
	84	52	52 (100%)	323.63	246.84 / 424.30	15 (28.8%)	2.94	1.81 / 4.76

N = number of subjects with samples for antibody response, n (%) = number of seropositive subjects / percentage of seropositive subjects based on N, GMT = Geometric Mean Titer, CI = Confidence Interval, LL: Lower Limit, UL: Upper Limit
Seropositivity rate was defined as the percentage of subjects with antibody titers ≥ the assay cut-off value.
ELISA: The assay cut-off value is 1:50; PRNT: The assay cut-off value is 1:20.

Study POX-MVA-004 revealed that all three dose levels induced a 100% seroconversion after the second vaccination. Seroconversion was defined as the appearance of antibody titers ≥ 1:50 in a vaccinia specific IgG ELISA for initially seronegative subjects. The strongest immune response with earliest onset of seroconversion was achieved with the highest dose tested. In this group, 94% of the subjects already seroconverted after only 1 vaccination. The data obtained with neutralizing antibodies closely correlated to the ELISA results (Figure I). A significance test of the correlation shows a highly significant result: p = 1.5E-18 and confirms that both assays are highly correlated. T cell response data: Cellular immune responses were analyzed in this trial with intracellular cytokine staining (ICS) detecting vaccinia-specific IFN-γ producing CD4+ / CD8+ cells. Cellular immune response resulted in a dose-dependent CD8+ response (Figure II).

Figure I: POX-MVA-004 ELISA/PRNT Correlation analysis

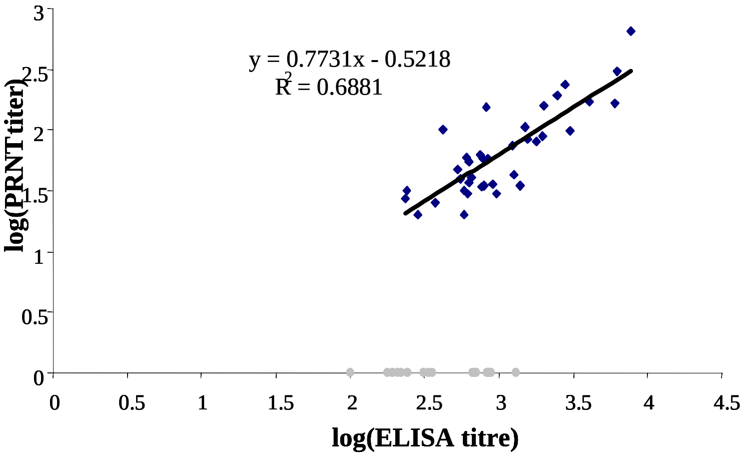

The ELISA titer versus the PRNT titer for all 162 available day 42 samples. The correlation calculation is based only on the samples where both titer values are positive – 94 positive pairs in all. 75% of the negative PRNT values had (log10 of) ELISA titers below 2.73, whereas all the samples with a positive PRNT titer had (log10 of) ELISA titers above 2.34.

Figure II: POX-MVA-004 Intracellular cytokine staining / vaccinia specific immunity

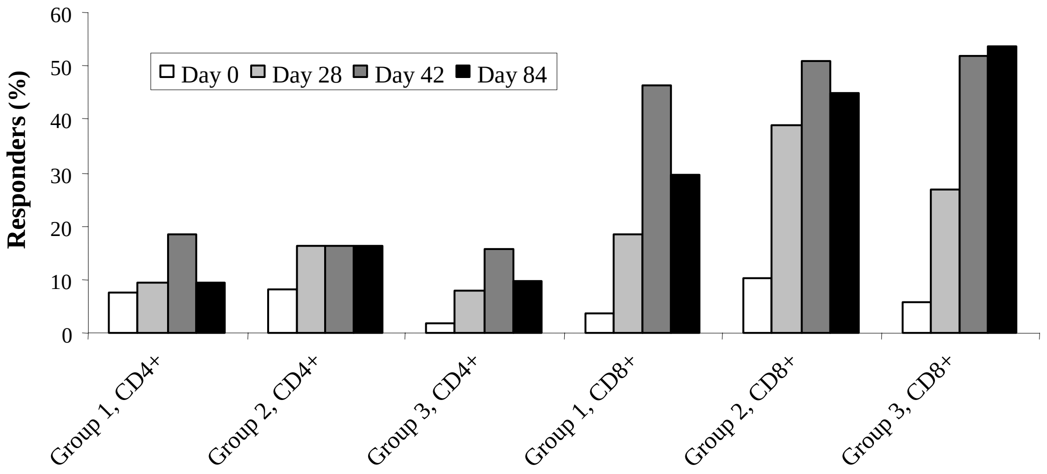

Group 1: 2x 10^7 $TCID_{50}$, subcutaneous in subjects not vaccinated against smallpox
Group 2: 5x 10^7 $TCID_{50}$, subcutaneous in subjects not vaccinated against smallpox
Group 3: 1x 10^8 $TCID_{50}$, subcutaneous in subjects not vaccinated against smallpox
Analysis was performed on the per protocol population

Study POX-MVA-002: In addition MVA-BN® is currently being tested in the US in a Phase I study (POX-MVA-002) in healthy subjects in combination with the licensed smallpox vaccine Dryvax®.

A total of 90 subjects were enrolled 75 of whom have received MVA-BN® in different doses. Currently, the study is still blinded and it is expected that final results of the study will become available during 2006. However no serious and unexpected adverse events related to MVA-BN® have been reported. One subject reported shortness of breath 2 weeks after vaccination with Dryvax® and this cardiac adverse event was assessed by the investigator as definitely being related to Dryvax®.

MVA-BN®-based vaccination of populations for which Dryvax® is contraindicated

Patients belonging to risk populations such as immune compromised (e.g. HIV infected patients) or otherwise immunologically impaired (e.g. patients with atopic disorders) are normally excluded from vaccination with traditional smallpox vaccines (e.g. Dryvax®). To show that MVA-BN® is safe and immunogenic even in these patients, Bavarian Nordic up to date initiated three clinical trials in HIV infected subjects and/or subjects with atopic dermatitis with MVA-BN® or MVA-BN® based vaccines. Initial trials in HIV-1 infected subjects using the recombinant MVA-BN® based Nef vaccine are completed and study reports available.

Studies HIV-NEF-001 and HIV-NEF-002: Both phase I vaccination studies conducted in Germany had the objective to evaluate safety and tolerability of a recombinant MVA vaccine expressing the HIV-1 nef-gene (MVA-nef) in HIV-1 infected patients on HAART. In study HIV-NEF-002 the subjects underwent a structured therapy interruption after the third vaccination.
Twenty-four patients with CD4 counts ≥ 400/µl were enrolled and immunized with $5x10^8$ $TCID_{50}$ of the MVA-nef vaccine, administered subcutaneously.
In both studies it was demonstrated that $5x10^8$ $TCID_{50}$ MVA-nef was safe and well tolerated in HIV-1 infected patients with CD4 counts ≥ 400/µl. The most frequent adverse reactions were mild injection site reactions like induration or pain.

Study POX-MVA-007: To evaluate safety and immunogenicity of MVA-BN® smallpox vaccine in subjects with atopic disorders, this open-label, controlled phase I pilot study was started in Germany in May 2004.

Primary objective of the trial is to expand the available data on the safety of MVA-BN® in a population with atopic disorders. Vaccinia-naïve volunteers between 18 and 40 years were assigned to the following study groups (15 subjects per group) depending on their medical history and current health status:

- Healthy subjects
- Subjects with history of atopic dermatitis
- Subjects with mild active atopic dermatitis
- Subjects with mild allergic rhinitis

All subjects are immunized twice at a 4 week interval using MVA-BN® subcutaneously in a dose of $1x10^8$ $TCID_{50.}$ The study duration is up to 36 weeks for each subject. All subjects will be followed for at least 24 weeks after having received the last study vaccination

An interim safety report of the first nineteen subjects in this study is available. This interim safety report demonstrates a low number of grade 3 adverse events (AEs) and confirms a favorable safety profile of MVA-BN® even in a population in which conventional smallpox vaccines are contraindicated. Non-serious unsolicited and solicited AEs documented in this report were comparable to typical adverse reactions expected with other modern injectable vaccines (Table III). Importantly, the results of the interim safety analysis revealed no special hazards of MVA-BN® smallpox vaccine in subjects with a history of mild or active atopic dermatitis. The interim data were also submitted to CBER (IND # 11596 amendment 6) and CBER confirmed that the study in atopic dermatitis was safe to be continued. All 60 subjects have since received two vaccinations without any serious or unexpected reactions reported.

Table III: Adverse reactions (per subject analysis - full analysis set, n = 19)

MedDra preferred term	Healthy		History of atopic dermatitis		Active atopic dermatitis		Allergic rhinitis		Total	
	(n = 5)		(n = 4)		(n = 5)		(n = 5)		(n = 19)	
	n	(%)	n	(%)	n	(%)	n	(%)	n	(%)
Lymphadenopathy	0	(0.0)	0	(0.0)	0	(0.0)	1	(20.0)	1	(5.3)
Injection site pain	5	(100.0)	4	(100.0)	5	(100.0)	5	(100.0)	19	(100.0)
Injection site erythema	5	(100.0)	4	(100.0)	5	(100.0)	5	(100.0)	19	(100.0)
Injection site swelling	5	(100.0)	3	(75.0)	3	(60.0)	4	(80.0)	15	(78.9)
Injection site induration	5	(100.0)	2	(50.0)	5	(100.0)	4	(100.0)	16	(84.2)
Injection site discoloration	0	(0.0)	0	(0.0)	0	(0.0)	1	(20.0)	1	(5.3)
Injection site hemorrhage	0	(0.0)	0	(0.0)	1	(20.0)	0	(0.0)	1	(5.3)
Injection site pruritus	0	(0.0)	0	(0.0)	1	(20.0)	1	(20.0)	2	(10.6)
Injection site warmth	0	(0.0)	0	(0.0)	1	(20.0)	0	(0.0)	1	(5.3)
Pain localized	0	(0.0)	0	(0.0)	0	(0.0)	1	(20.0)	1	(5.3)
Dermatitis atopic	0	(0.0)	0	(0.0)	1	(20.0)	0	(0.0)	1	(5.3)
Urticaria	0	(0.0)	0	(0.0)	1	(20.0)	0	(0.0)	1	(5.3)
Pyrexia	0	(0.0)	0	(0.0)	1	(20.0)	0	(0.0)	1	(5.3)
Headache	2	(40.0)	1	(25.0)	2	(40.0)	2	(40.0)	7	(36.8)
Myalgia	0	(0.0)	0	(0.0)	0	(0.0)	3	(60.0)	3	(15.8)
Nausea	0	(0.0)	0	(0.0)	0	(0.0)	2	(40.0)	2	(10.6)
Fatigue	3	(60.0)	1	(25.0)	3	(60.0)	5	(100.0)	12	(63.2)

Study POX-MVA-010: In July 2005, BN initiated a multi-center, open-label phase I/II study with the objective to generate safety and immunogenicity data with MVA-BN® in HIV infected subjects with CD4+ counts > 350 / µl compared to healthy subjects. 150 male and female vaccinia-naïve and pre-immune volunteers are assigned to the following study groups depending on their medical history and health status:

Group 1: 30 HIV infected subjects without history of previous smallpox vaccination
Group 2: 60 HIV infected subjects with history of previous smallpox vaccination
Group 3: 30 Healthy subjects without history of previous smallpox vaccination
Group 4: 30 Healthy subjects with history of previous smallpox vaccination

The subjects are immunized (s.c.) with a MVA-BN® dose of 1 x 10^8 $TCID_{50}$. Group 1 and 3 will receive two vaccine administrations 4 weeks apart; group 2 and 4 a single administration. The study is being performed at 5 study centers in the US and currently enrolling volunteers.

Study HIV-NEF-004: This phase II study is currently being conducted in Germany to evaluate immunogenicity and safety of two doses of a recombinant MVA vaccine expressing the HIV-1 nef-gene ($1x10^8$ and 5×10^8 MVA-nef) and one dose of $1x10^8$ $TCID_{50}$ MVA-BN®. By end of Oct. 2005 a total of 52 HIV-1 infected subjects with CD4 counts > 250/µl were enrolled. All subjects are stable on HAART and receive 3 vaccinations of either one of the three vaccines. To date, MVA-nef and MVA-BN® are well tolerated. Most frequent adverse reactions are mild injection site reactions like induration and pain.

1.5. Dose, route of administration, vaccination schedule

MVA-BN® will be given subcutaneously. The rationale for the selected dose and the route of administration comes from historical use as well as preclinical and clinical data (study V-POX-00.1 (POX-MVA-001)) that showed satisfactory immune responses after a $1x10^8$ $TCID_{50}$ dose and a good safety profile of MVA-BN® if administered via the subcutaneous route.

1.6. Benefit/risk assessment

The safety profile of MVA-BN® is supported by the historical experience with the MVA-BN® precursor vaccine, which was used in Germany during the smallpox vaccination for two-stage parenteral vaccination in children in 1976. MVA was used in over 120,000 Caucasian individuals with no reported major side effects, even though some of the subjects were among the population with high risk of developing complications (Mayr et al., 1978).

It could be concluded that from the previous experience with MVA based vaccines, adverse reactions to MVA-BN® are expected to be comparable to typical adverse reactions seen with other modern vaccines.

The severe and life-threatening adverse reactions like progressive vaccinia, eczema vaccinatum, generalized vaccinia and inadvertent inoculation that are observed after the administration of conventional smallpox vaccines are due to the replication of the vaccinia strains. Since MVA-BN® is replication incompetent in human cells it has a better safety and tolerability profile and can consequently not induce the severe side effects like progressive vaccinia which are associated with the replication of the vaccine virus. Apart from the better safety profile with regard to severe reactions the available clinical experience with MVA-BN® shows that it is generally better tolerated (e.g. local reaction without scar formation) than conventional smallpox vaccines.

Based on the available data with MVA-BN® the risk for the study participants is not expected to be higher than the risk observed with other modern vaccines. The main risk is the development of local reactions at the vaccination site.

In addition, the study participants can contribute significantly to the development of a safe smallpox vaccine. They might acquire a potential protection against smallpox. However, it cannot be concluded that the vaccine is efficacious against this disease, as MVA based vaccines are currently in an early phase of development. In view of the expected side effects of MVA-BN® the potential risks associated for the study participants in this study seem to be limited and justify the potential benefit for society.

Preclinical data with MVA-BN® reveal no special hazard for humans based on conventional studies of safety. Over 350 healthy and over 100 subjects with HIV or Melanoma have been vaccinated in clinical trials with vaccines using MVA-BN® alone (to develop an improved smallpox vaccine) or with MVA-BN® as vector (with HIV specific inserts or inserts to be used as melanoma vaccine).

2. Objectives

The primary immunogenicity objective of the trial is:

- To compare the ELISA specific humoral immune response of the group with history of smallpox vaccination (Group 4: 1 dose MVA-BN®) versus the group without history of smallpox vaccination (Group 1: 2 doses MVA-BN®), i.e. to prove that a single dose schedule would be sufficient to boost the immunity in a previously vaccinated population.

The primary safety objective of the trial is:

- To compare the four different vaccination groups with regard to ECG changes and cardiac symptoms.

The secondary objectives of the trial are:

- To compare the ELISA specific immune response of the four different vaccination groups - comparison combinations not done for the primary objective.
- To compare the four different vaccination groups with regard to immune response measured with a plaque reduction neutralization test.
- To assess the kinetics of the immune responses after 1 (Group 2) and 2 (Group 1) doses of MVA-BN® in vaccinia naïve subjects.
- To compare the four different vaccination groups with regard to safety and reactogenicity.
- To compare the safety of one (Group 2) and two (Group 1) vaccinations with MVA-BN® in vaccinia-naïve subjects with placebo (Group 3) and historical / recently published data of Dryvax® immunization in vaccinia-naive subjects.

3. Study design

3.1. Experimental design

This study is a partially randomized, partially double-blind, placebo-controlled, phase II non-inferiority trial to evaluate immunogenicity and safety of one and two doses of MVA-BN® smallpox vaccine in 18-55 year old healthy subjects.

In this study the following four subject groups will be treated as described below:

Double-blind:

Group 1: Subjects without history of smallpox vaccination (2 doses MVA-BN®)

Group 2: Subjects without history of smallpox vaccination (1st dose MVA-BN® / 2nd dose placebo)

Group 3: Subjects without history of smallpox vaccination (2 doses placebo)

Open-label:

Group 4: Subjects with history of smallpox vaccination (1 dose MVA-BN®)

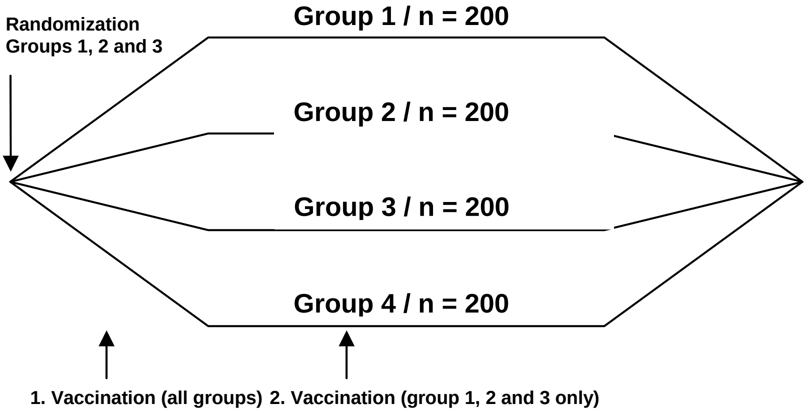

Visit Days (Groups 1, 2, 3)

-28	0	10-15	28 – 35	V3 + 10-15	V3 + 28-35	V3 + 182–210
Scr	V1	V2	V3	V4	V5	F-U

Visit Days (Group 4)

-28	0	10-15	28 - 35	V1 + 182–210
Scr	V1	V2	V3	F-U

3.2. Discussion of study design and choice of control groups

The primary objective of this study is to demonstrate that the booster immune response elicited by a single vaccination in a population previously vaccinated against smallpox (Group 4) is non-inferior to a vaccinia-naive population which receives two vaccinations (Group 1), i.e. to prove that a single dose schedule would be sufficient to boost the immunity in a previously vaccinated population.

Group 3 serves as an internal control to validate the trial results and to show sensitivity of the trial. In addition Groups 2 and 3 are important for the assessment of the secondary variables of the study and to generate hypotheses which might prove to be important and which should be addressed in the further clinical development plan for MVA-BN®.

Group 2, which receives only a single vaccination with MVA-BN® followed by a placebo vaccination, is necessary for the comparison of the immune response after only one vaccination in a vaccinia-naive population with the immune response induced by two vaccinations in such a population (Group 1) and the immune response after a single vaccination in a previously vaccinated population (Group 4).

In addition the study is designed to further investigate the safety profile of MVA-BN® in a healthy population. Recent findings of the US vaccination smallpox campaign (Grabenstein et. al.) and previous clinical trials indicated that after vaccination with the replication competent NYCBOH vaccinia strain, cardiac side effects occurred in an unexpected high frequency. Results of controlled clinical trials with the first and second generation smallpox vaccines Dryvax® and ACAM2000 demonstrated 8 cases of myo-/pericarditis in 1162 vaccinated study subjects, i.e. an incidence of 1:145 or 0.69% (Acambis news release Sep. 20th, 2004). Importantly, the events occurred only in a vaccinia-naive population, whereas a population previously vaccinated against smallpox did not report any cardiac adverse reactions. While the underlying mechanism of NYCBOH-related myo-/pericarditis is unknown, specific mechanisms ("molecular mimicry") as well as unspecific immunologically triggered mechanisms are being discussed. Thus, it has been stipulated by regulatory authorities to focus on the cardiac safety profile of all other vaccinia-based vaccines in development.

To date, >500 subjects have been vaccinated in clinical trials with MVA-BN®. No case of myo-/pericarditis or any other unexpected adverse reactions have been detected. To further evaluate the cardiac safety profile of MVA-BN®, the comparison of the four groups with regard to ECG changes and cardiac symptoms was chosen as co-primary safety objective. The main comparisons will be Group 1 (vaccinia naïve, 2 vaccinations) with placebo (Group 3), Group 2 (1 vaccination) with placebo and Group 4 with placebo. Additionally, a comparison of Group 1 vs. Group 2 and Group 1 vs. Group 4, as well as pooled analyses of Groups 1, 2 and Groups 1, 2, 4 vs. Group 3 will be performed.

A sample size of 200, 400 and 600 will be sufficient to detect reactions reported at a frequency of less than 1/50, 1/100 and 1/200 respectively.

3.3. Description of study procedures

The study will be conducted according to the Study Flow Chart (section III).

Visits should be scheduled within the given intervals.

3.3.1. Screening phase

Screening Visit (SCR, day -28 to –1 / week –4 to -1)

The informed consent must be reviewed with and signed and dated by the subject (volunteer) prior to the initiation of any evaluations or procedures required by the protocol. All subjects must be thoroughly informed of all aspects of the study (e.g. study visit schedule, required evaluations and procedures) as described in the informed consent document.

After informed consent has been collected, subjects will enter a screening period of up to four weeks before the first vaccination.

An extensive screening assessment will be performed during the screening period (visit SCR).

All Groups
**VISIT SCR (DAYS -28 TO -1)**<br>• Informed Consent<br>• Check inclusion/exclusion criteria<br>• Check medical history<br>• Physical examination including evaluation of vital signs, especially listening to heart and lung<br>• Perform baseline ECG<br>• Serum pregnancy test (if applicable)<br>• Urine analysis<br>• Safety laboratory<br>• Review baseline signs & symptoms<br>• Review prior medication

3.3.2. Allocation of subjects to treatment groups

Subjects eligible for Groups 1, 2 and 3 will be randomly allocated to one of these three treatment groups in order of their appearance. The randomization list will be provided by Bavarian Nordic A/S. At investigational site, only the study independent person preparing the study medication will have access to the randomization list. In case of emergency which makes unblinding necessary, the Medical Director of Bavarian Nordic (contact details listed in chapter 7.3) has also access to the randomization list.

Subjects eligible for Group 4 will be allocated to Group 4 in order of their appearance.

3.3.3. Active study phase

After successful screening period the subject will run in the active study phase starting with Visit 1.
The procedures performed at Visit 1 and all following visits are listed below. Blood draws and all other tasks mentioned in the list above the vaccination event must always be performed **prior** to vaccination!

Each immunization consists of 1 x 10^8 $TCID_{50}$ MVA-BN® vaccine or placebo (0.5 ml Tris Buffer), administered subcutaneously in the non-dominant upper arm. Subjects in Groups 1, 2 and 3 will receive two doses each, four weeks apart (optional up to +7 days); depending on the group two doses of MVA-BN®, one dose MVA-BN® and one dose placebo, or two doses of placebo (see section 3.1). Subjects in Groups 1, 2 and 3 will be double-blinded.
Study Group 4 will receive one vaccination with MVA-BN® at Visit 1. This group will be open-label.

Following vaccination subjects will be kept under close observation for at least 30 minutes, with appropriate medical treatment readily available in case of a rare anaphylactic reaction following the administration of vaccines.

Any adverse events (AE) that occur during or after vaccination will be recorded. The subjects will be issued with a diary card for the recording of solicited AEs occurring on the vaccination day and the seven days following vaccination.

<table>
<tr><th colspan="2">All Groups</th></tr>
<tr><td colspan="2">

VISIT 1 (DAY 0)

- Re-check inclusion/exclusion criteria
- Physical examination including evaluation of vital signs, especially listening to heart and lung
- Urine pregnancy test (if applicable)
- Blood for Antibody analysis
- Review baseline signs & symptoms
- Review prior medication

Blood draws and all other tasks mentioned above must always be performed prior to vaccination!

- 1st vaccination
- Handout of diary card for 1st vaccination
- Recording and documentation of AEs
- Review concomitant medication

</td></tr>
<tr><td colspan="2">

VISIT 2 (VISIT 1 + 10-15 DAYS)

- Physical examination including evaluation of vital signs, especially listening to heart and lung
- Perform ECG
- Safety laboratory
- Blood for Antibody analysis
- Collection of the diary card for (1st) vaccination, review together with subject and transfer to the electronic Case Report Form (CRF)
- Recording and documentation of AEs
- Review concomitant medication

</td></tr>
<tr><th>Groups 1, 2 and 3</th><th>Group 4</th></tr>
<tr><td>

VISIT 3 (VISIT 1 + 28-35 DAYS)

- Re-check inclusion/exclusion criteria
- Physical examination including evaluation of vital signs, especially listening to heart and lung
- Urine pregnancy test (if applicable)
- Blood for Antibody analysis

Blood draws and all other tasks mentioned above must always be performed prior to vaccination!

- 2nd vaccination
- Handing over of the diary card for 2nd vaccination
- Recording and documentation of AEs
- Review concomitant medication

</td><td>

VISIT 3 (VISIT 1 + 28-35 DAYS)

- Physical examination including evaluation of vital signs, especially listening to heart and lung
- Urine pregnancy test (if applicable)
- ECG, only if clinically indicated
- Safety laboratory, only if clinically indicated
- Blood for Antibody analysis
- Recording and documentation of AEs
- Review concomitant medication

</td></tr>
</table>

**VISIT 4 (VISIT 3 + 10-15 DAYS)**<br>• Physical examination including evaluation of vital signs, especially listening to heart and lung<br>• Perform ECG<br>• Safety laboratory<br>• Blood for Antibody analysis<br>• Collection of the diary card for 2nd vaccination, review together with subject and transfer to the electronic Case Report Form (CRF)<br>• Recording and documentation of AEs<br>• Review concomitant medication	
**VISIT 5 (VISIT 3 + 28-35 DAYS)**<br>• Physical examination including evaluation of vital signs, especially listening to heart and lung<br>• Urine pregnancy test (if applicable)<br>• ECG, only if clinically indicated<br>• Safety laboratory, only if clinically indicated<br>• Blood for Antibody analysis<br>• Recording and documentation of AEs<br>• Review concomitant medication	

3.3.4. Follow-up phase

To secure long-term safety, the subject has to come in for an examination in a follow-up phase 26 (+4) weeks after the last vaccination.

**Groups 1, 2 and 3**<br>**VISIT F-U (VISIT 3 + 182 – 210 DAYS)**	**Group 4**<br>**VISIT F-U (VISIT 1 + 182 – 210 DAYS)**
• Physical examination including evaluation of vital signs, especially listening to heart and lung<br>• ECG, only if clinically indicated<br>• Safety laboratory only if clinically indicated<br>• Blood for Antibody analysis<br>• Recording and documentation of Serious Adverse Events (SAEs)	

3.4. Study duration

The total duration of the study including the screening period and follow-up visit will be

- Up to 39 weeks for each subject in study Groups 1, 2 and 3 (vaccinia naïve subjects) and
- Up to 34 weeks for each subject in study Group 4 (pre-immune subjects)

All subjects will be followed for at least 26 weeks after having received the last study vaccination. It is expected to recruit subjects within a reasonable short time, to limit the total study duration to 11 months.

4. Selection of subjects

4.1. Recruitment procedure

Volunteers will be recruited actively. Methods of recruitment may include letters, e-mails, posters, paid advertisements, announcements on campuses and distribution of flyers.

In total, 800 individuals will be enrolled as defined in Section 4.2. After signing the Informed Consent, subjects undergo screening procedures to check eligibility due to the following inclusion/exclusion criteria. In the event of a screen failure secondary to mild or limited acute illness or abnormal laboratory values the patient may be rescreened after resolution of the event. Re-screening may require only an additional blood draw or a complete re-screening evaluation, depending on the circumstances of and the time interval from the initial screening failure.

The investigator will keep a log of subjects screened for the study, but not enrolled, and provide the reason for exclusion.

Bavarian Nordic will be informed about every patient entered in the study using a subject inclusion form.

4.2. Inclusion criteria

All groups

1. Male and female subjects between 18 and 55 years of age.
2. Women must have a negative serum pregnancy test at screening and a negative urine or serum pregnancy test within 24 hours prior to vaccination.
3. Women of childbearing potential must have used an acceptable method of contraception for 30 days prior to the first vaccination, must agree to use an acceptable method of contraception during the study, and must not become pregnant for at least 28 days after the last vaccination. A woman is considered of childbearing potential unless post-menopausal or surgically sterilized. (Acceptable contraception methods are restricted to abstinence, barrier contraceptives, intrauterine contraceptive devices or licensed hormonal products.)
4. Read, signed and dated informed consent document after being advised of the risks and benefits of the study in a language understood by the subject signed, and prior to performance of any study specific procedure.
5. Troponin I within normal institutional limits.
6. White blood cells ≥ 2500/mm^3 and ≤ 11,000/ mm^3.
7. Absolute neutrophil count (ANC) ≥ 1000/mm^3.

8. Negative urine glucose by dipstick or urinalysis.
9. Hemoglobin ≥ LLN.
10. Platelets 100 – 440/nl.
11. Adequate renal function defined as:
    c. Serum creatinine without clinically significant findings.
    d. Urine protein ≤ 30 mg/dL or none or trace proteinuria (by urinalysis or dip stick).
12. Adequate hepatic function defined as:
    c. Total bilirubin ≤ 1.5 x ULN in the absence of other evidence of significant liver disease (healthy subjects without clinical disease with Morbus Meulengracht can be included).
    d. AST (SGOT), ALT (SGPT) and alkaline phosphatase without clinically significant findings.
13. Electrocardiogram (ECG) without abnormal findings (e.g. any kind of atrioventricular or intraventricular conditions or blocks such as complete left or right bundle branch block, AV-node block, QTc or PR prolongation, premature atrial contractions or other atrial arrhythmia, sustained ventricular arrhythmia, 2 premature ventricular contractions (PVC) in a row, ST elevation consistent with ischemia).
14. Availability for follow-up for the planned duration of the study (26 weeks after last vaccination).

Groups 1, 2 and 3 (All vaccinia-naïve subjects) additionally:
1. No history of known or suspected previous smallpox vaccination.
2. No detectable vaccinia scar.

Group 4 (All previously vaccinated subjects) additionally:
1. History of previous smallpox vaccination (documented and/or typical vaccinia scar).
2. Most recent smallpox vaccination ≥ 5 years.

4.3. Exclusion criteria

Groups 1, 2, 3, and 4 (All subjects):
1. Pregnant or breast-feeding women.
2. Uncontrolled serious infection i.e. not responding to antimicrobial therapy.
3. History of any serious medical condition, which in the opinion of the investigator would compromise the safety of the subject.
4. History of or active autoimmune disease. Persons with vitiligo or thyroid disease taking thyroid replacement are not excluded.
5. Known or suspected impairment of immunologic function including, but not limited to, clinically significant liver disease; diabetes mellitus; moderate to severe kidney impairment.
6. History of malignancy, other than squamous cell or basal cell skin cancer, unless there has been surgical excision that is considered to have achieved cure. Subjects with history of skin cancer at the vaccination site are excluded.
7. History or clinical manifestation of clinically significant and severe hematological, renal, hepatic, pulmonary, central nervous, cardiovascular or gastrointestinal disorders.
8. Clinically significant mental disorder not adequately controlled by medical treatment.
9. Any condition which might interfere with study objectives or would limit the subject's ability to complete the study or to be compliant in the opinion of the investigator.
10. History of coronary heart disease, myocardial infarction, angina, congestive heart failure, cardiomyopathy, stroke or transient ischemic attack, uncontrolled high blood pressure, or any other heart condition under the care of a doctor.

11. History of an immediate family member (father, mother, brother, or sister) who died due to ischemic heart disease before age 50 years.
12. Ten percent or greater risk of developing a myocardial infarction or coronary death within the next 10 years using the National Cholesterol Education Program's risk assessment tool. (http://hin.nhlbi.nih.gov/atpiii/calculator.asp?usertype=prof) NOTE: This criterion applies only to volunteers 20 years of age and older.
13. History of intravenous drug abuse.
14. History of allergic disease or reactions likely to be exacerbated by any component of the vaccine.
15. Known allergy to egg or aminoglycoside (gentamicin).
16. History of anaphylaxis or severe allergic reaction.
17. Having received any vaccinations or planned vaccinations with a live vaccine within 30 days prior or after study vaccination.
18. Having received any vaccinations or planned vaccinations with a killed vaccine within14 days prior or after study vaccination.
19. Chronic administration (defined as more than 14 days) of systemic immuno-suppressant drugs during a period starting from six months prior to administration of the vaccine and ending at study conclusion. (Corticosteroid nasal sprays are permissible. Persons who have used topical and inhaled steroids can be enrolled after their therapy is completed).
20. Post organ transplant subjects whether or not receiving chronic immunosuppressive therapy.
21. Administration or planned administration of immunoglobulins and/or any blood products during a period starting from 3 months prior to administration of the vaccine and ending at study conclusion.
22. Use of any investigational or non-registered drug or vaccine other than the study vaccine within 30 days preceding the first dose of the study vaccine, or planned administration of such a drug during the study period.
23. Study personnel.

5. Study halting/termination and withdrawal of subjects

5.1. Study halting and termination rule

A temporary halting or termination for single study subjects or for the study as a whole can be decided in case of an occurrence of

- a serious adverse event (SAE)
- a grade 3 or higher systemic reaction or lab toxicity lasting >48 hours

with an at least reasonable possibility of a causal relationship to the administration of MVA-BN®, i.e. the relationship cannot be ruled out. These parameters are not all-inclusive. Other adverse events could occur that would trigger a Safety Monitoring Committee (SMC) review. Any member of the SMC, the Principal Investigator, BN Safety Officer, or DMID Medical Officer could request a Safety Monitoring Committee review based on any observation.

5.2. Reporting of events fulfilling the study halting criteria

If an event fulfilling the study halting criteria reaches the investigator's attention, the investigator has the liability to alert Bavarian Nordic's Safety Department immediately (within 24 hours) and provide a comprehensive documentation of the event.

5.3. Safety Monitoring Committee (SMC)

The SMC is an independent board that oversees the safety of volunteers participating in the study. The members of the SMC are selected by Bavarian Nordic and DMID in accordance to DMID guidelines. The primary responsibilities of the SMC are to periodically review and evaluate the accumulated study data for participant safety, study conduct and progress, and make recommendations to Bavarian Nordic, DMID and the Principal Investigator concerning the continuation, modification, or termination of the trial program. The SMC considers study specific data as well as relevant background knowledge about the disease, test agent, and patient population under study. A separate charter describes in detail relevant operational procedures, communication pathways, roles and responsibilities of the SMC.

In case an event occurs which fulfills the study halting criteria the Safety Monitoring Committee will review the event in a timely manner and give a recommendation to Bavarian Nordic, DMID and the Principal Investigator(s) to halt, resume or terminate the study participation of the affected subject and/or the study as a whole.

5.4. Individual withdrawal criteria during the study

Subjects may withdraw or be removed from the study for any of the reasons cited below:

- An adverse event occurs that, in the opinion of the investigator, makes it unsafe for the subject to continue the study. In this case, the appropriate measures will be taken.
- Subject's request to withdraw.
- Subject unwilling or unable to comply with study requirements.
- Clinical need for concomitant or ancillary therapy not permitted in the study.
- Unrelated intercurrent illness that, in the judgment of the Principal Investigator, will affect assessment of clinical status to a significant degree.

The following criteria should be checked at any visit after enrollment into the study. If any become applicable during the study, the subject may be withdrawn:

1. Use of any investigational or non-registered drug or vaccine other than the study vaccine(s).
2. Administration of a licensed vaccine not foreseen by the study protocol during the study period.
3. Start of chronic administration (defined as more than 14 days) of systemic immuno-suppressant drugs during the study period (Corticosteroid nasal sprays are permissible. Persons who have used topical and inhaled steroids can be enrolled after their therapy is completed).
4. Administration of immunoglobulins and/or any blood products during the study period.

5.5. Subject withdrawal procedure

If a subject discontinues prior to completion of the study, the date and reason for the discontinuation will be obtained. The date of the last dose of study medication will also be obtained.

Once a subject receives MVA-BN® he/she must be followed for safety as stated in the protocol. From the time of discontinuation, all diagnostic procedures and evaluations scheduled for Visit 5 for Group 1, 2 and 3 and Visit 3 for Group 4 should be performed (see III. FLOW CHART).

As a general rule, subjects who discontinued the trial after having received at least one vaccination will not be replaced.

Subjects included in the study but already discontinued the trial prior to the first vaccination should be replaced.

5.6. Contraindications and precautions for further study vaccinations

Absolute contra-indications:
The following events constitute absolute contra-indications to administration of the study vaccine; if any of these occur or become known during the study, the subject must not receive (additional) doses of the vaccine, but may continue other study procedures at the discretion of the investigator:

- Anaphylactic reaction following the administration of vaccine(s).
- Pregnancy up to and including 4 weeks (minimum 28 days) after receiving the last vaccine dose.

Subjects should be instructed to notify the investigator if it is determined after completion of the study that they became pregnant during the study or within 4 weeks (minimum 28 days) after receiving the last vaccine dose. Pregnancy must be reported to Bavarian Nordic on a Pregnancy Form within 24 hours of the investigator's becoming aware of the event.

A pregnancy should be followed to term, any premature terminations reported, and the health status of the mother and child including date of delivery and the child's gender and weight should be reported to Bavarian Nordic after delivery.

Temporary deferral of vaccination:
If the following symptom is present at the time scheduled for vaccination, the subject may be vaccinated at a later date or withdrawn at the discretion of the investigator:

- Acute disease at the time of vaccination. (Acute disease is defined as the presence of a moderate or severe illness with or without fever. The vaccine can be administered to persons with a minor illness such as diarrhea, mild upper respiratory infection with or without low-grade febrile illness, i.e., oral temperature ≤ 100.4°F (≤ 38.0°C).

6. Study treatment

6.1. Investigational product

MVA-BN® (IMVAMUNE™) is a highly attenuated live vaccinia virus. MVA-BN® will be provided in liquid-frozen aliquots of 0.6 ml. One dose of 0.5 ml liquid-frozen vaccine contains 1 x 10^8 $TCID_{50}$ Modified Vaccinia virus Ankara – Bavarian Nordic (MVA-BN®).

For details see current version of Investigator's Brochure.

6.2. Placebo

For this study 0.5 ml Tris Buffer will be used as placebo.

The Tris Buffer contains 1.21 g/l Tris, 8.526 g/l sodium chloride, 19.2 g/l Dextran FP-40, 45.2 g/l Sucrose and 0.108 g/l L-Glutamic acid monopotassium salt, pH 7.5.

6.3. Packaging and labeling

MVA-BN® and placebo are manufactured and labeled by IDT- Impfstoffwerke Dessau-Tornau GmbH.

Address:

IDT - Impfstoffwerke Dessau-Tornau GmbH
Dr. Margrit Gehrt
Streetzer Weg 15a
06862 Rodleben
Germany
Phone: +49-34901-885 0

The packages and vials will be labeled according to the national law.

6.4. Vaccine storage, handling and dispensing

The liquid frozen MVA-BN® vaccine has to be stored at -20 ± 5°C. Do not re-freeze a vial once it has been thawed.

For administration, the vaccine vial will be thawed at room temperature. To ensure homogeneity, upon thawing the vial will be swirled gently (not shaken!) for at least 30 seconds. After thawing, the drug product should appear as a pale milky colored suspension. The liquid vaccine should be visually inspected for any foreign particulate matter prior to administration. In case foreign particulate matter is visible, the vaccine must not be used anymore.
The injection volume of 0.5 ml per dose will be withdrawn with a syringe using an injection needle long enough to reach the bottom of the vial. After withdrawal of the vaccine, the injection needle should be changed and the vaccine administered to the subject immediately.

If the vaccine cannot be administered immediately, it is recommended to administer the product within 12 hours after thawing. During this time the thawed vaccine has to be stored at +2 to +8°C in the dark.

6.5. Dose, vaccination schedule and route of administration

Depending on the group subjects are allocated to, subjects will receive MVA-BN® vaccine and/or placebo as follows in the upper arm (deltoid region):

Group 1:
Two s.c. vaccinations with 0.5 ml MVA-BN® vaccine containing 1 x 10^8 $TCID_{50}$, four weeks apart (Visit 1/Day 0 and Visit 3/Visit 1 + 28-35 days)

Group 2:
First s.c. vaccination with 0.5 ml MVA-BN® vaccine containing 1 x 10^8 $TCID_{50}$ and second s.c. vaccination with placebo (0.5 ml Tris Buffer), four weeks apart (Visit 1/Day 0 and Visit 3/Visit 1 + 28-35 days)

Group 3:
Two s.c. vaccinations with placebo (0.5 ml Tris Buffer), four weeks apart (Visit 1/Day 0 and Visit 3/Visit 1 + 28-35 days)

Group 4:
One s.c. vaccination with 0.5 ml MVA-BN® vaccine containing 1 x 10^8 $TCID_{50}$ (Visit 1/Day 0)

6.6. Accountability and disposal

Used and unused vials should be stored in a safe place and remain the property of Bavarian Nordic. The Principal Investigator of the respective site or his designee is responsible for ensuring adequate accountability of all used and unused study drug. This includes acknowledgement of receipt of each shipment of study drug (quantity and condition) and drug inventory log. The drug inventory log will document quantity of study drug received from Bavarian Nordic, quantity of study drug used for vaccination (including lot number, date dispensed, subject identification number and initials of the person dispensing the study medication) and quantity of study drug returned to Bavarian Nordic or destroyed.
In case destruction is agreed, material should be discarded at site according to local regulations.

6.7. Concomitant medication

All concomitant medication must be recorded in the CRF with the reason for administration, the dosage regimen, and the onset and end of treatment.

7. Clinical and laboratory assessments

7.1. Assessment of safety and reactogenicity

Taking into account the medical history of the subject, safety will be monitored by performing physical examinations including vital signs, routine laboratory measurements and ECGs as well as by evaluating local and general solicited adverse events and unsolicited adverse events.

Details regarding definitions and reporting of adverse events are described in section 8 of this protocol.

7.1.1. Medical history

The baseline assessment for safety parameters will be performed during screening visit. Medical history will be documented during the screening period (visit SCR). History will focus particularly on any important diseases and in case of infections or tumors, the pathogen involved or the pathological diagnosis, respectively, if available. A special attention should be given to history of prior allergic reactions, especially to vaccines.

For subjects in group 4, the presence of a typical vaccinia scar and/or data about smallpox vaccination (if any vaccination certificate is available) should be documented in the CRF.

7.1.2. Physical examination and vital signs

A physical examination and a check of vital signs will be performed during the screening period (visit SCR) for baseline assessment and on every following visit (Visits 1 – 5 and F-U).

PHYSICAL EXAMINATION:

The exam includes a review of major organ systems and weight. The examination should be directed at finding evidence of any infections, tumors and lymphadenopathy.

In addition, listening to the heart and lungs specifically for heart failure, presence of rubs, gallops, murmurs, crackles, and rales will be performed.

VITAL SIGNS:

Blood pressure and pulse rate will be taken after the subject was seated for two minutes, and oral temperature will be taken.

7.1.3. Laboratory measurements

SAFETY LABORATORY:

Safety laboratory is determined at the screening visit (Visit SCR) and 10 – 15 days after each vaccination (groups 1, 2 and 3: Visits 2 and 4 / Group 4: Visit 2). At Visit 5 (only Groups 1, 2 and 3) and at follow-up visit (all groups: Visit F-U) safety laboratory is only done if clinically indicated. The safety laboratory measurements are performed at a central laboratory. Laboratory normal ranges are provided by the central laboratory and filed in the investigator file. Safety laboratory parameters to be evaluated are:

Hematology:
Red blood cell count, hemoglobin, total and differential WBC, platelet count.

Serum chemistry:
Total bilirubin, alkaline phosphatase, AST, ALT, serum creatinine, sodium, potassium, calcium, troponin I.

The intensity of laboratory / systemic quantitatively measured toxicities will be graded according to the toxicity scale in Appendix I. These grading scales include the laboratory values determined with the routine safety parameters. In case of other laboratory values not included in the routine safety laboratory and not listed in Appendix I, the NCI Common

Toxicity Criteria table, Version 2.0, published April 30, 1999 will be used for grading of laboratory toxicities.

PREGNANCY TEST:

β-HCG pregnancy test will be conducted for all women with reproductive potential at screening (Visit SCR), within 24 hours prior to each vaccination (Groups 1, 2 and 3: Visits 1 and 3 / Group 4: Visit 1) and 28-35 days after the last vaccination (Group 1-3: Visit 5 / Group 4: Visit 3). At screening a serum β-HCG pregnancy test will be conducted and all other pregnancy test will be conducted as urine β-HCG test.

The following parameters will only be evaluated during the screening period (Visit SCR) for check of inclusion / exclusion criteria:

CHOLESTEROL:

Total, HDL and LDL.

URINE ANALYSIS (E.G. WITH COMBUR-10 STICK):

Protein, glucose, occult blood, nitrites, leucocytes, pH, bilirubin, ketones, urobilinogen, specific gravity.

7.1.4. ECG assessments

A standard 12-lead ECG will be taken on all subjects at screening (Visit SCR) and 10-15 days after each vaccination (Groups 1, 2 and 3: Visits 2 and 4 / Group 4: Visit 2). At Visit 5 (only Groups 1, 2 and 3) and at follow-up visit (all groups: Visit F-U) an ECG is only done if clinically indicated.

ECGs will be evaluated by a centralized procedure. Unclear ECG abnormalities will additionally be evaluated by a central Cardiologist. The workflow / communication flow will be as follows:

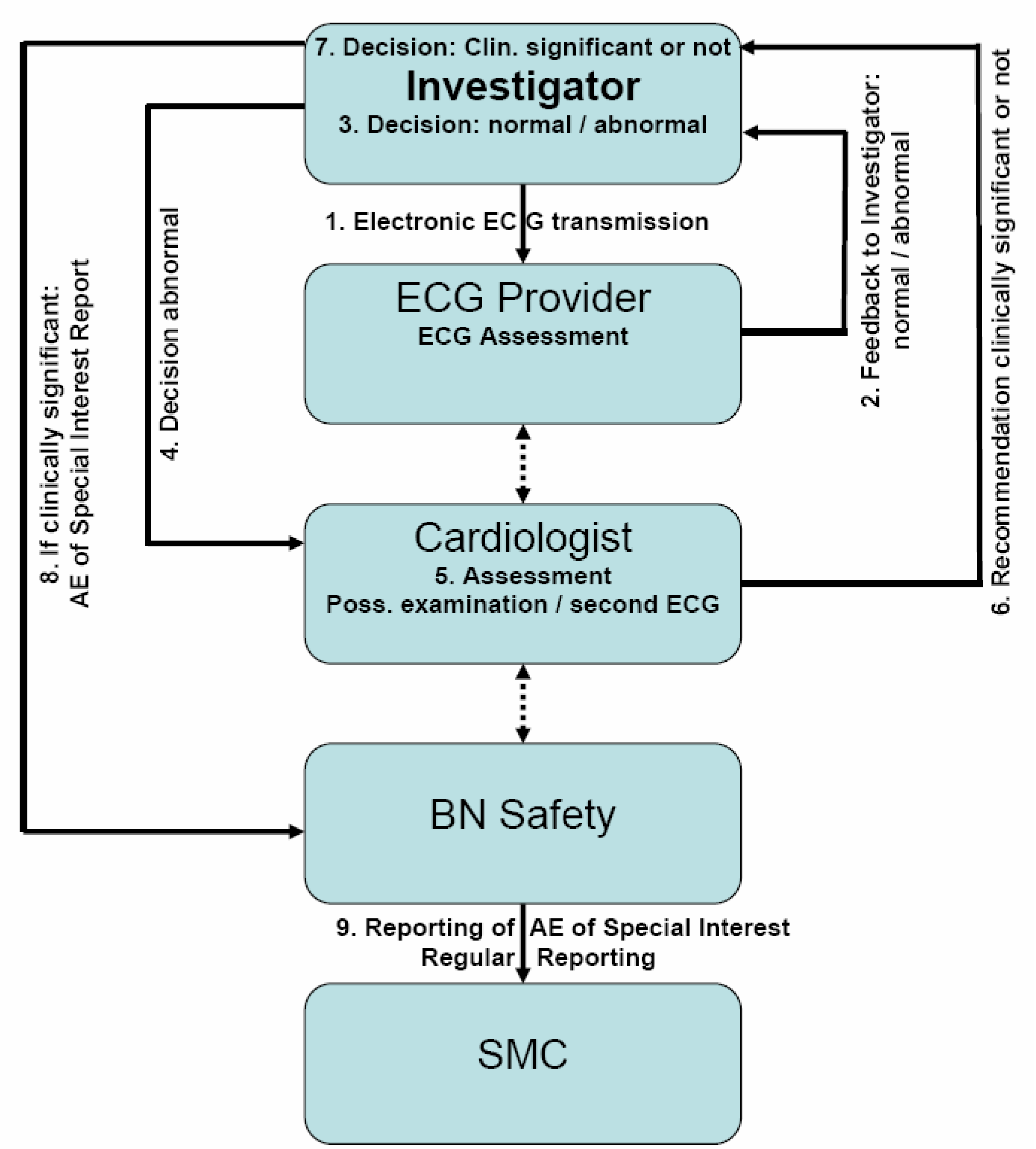

If clinically indicated, or in case of any kind of cardiac symptoms such as but not limited to chest pain, dyspnea, arrhythmia, or edema subjects will be referred for a cardiac evaluation by a Cardiologist and further diagnostic tests will be done as recommended by this Cardiologist (e.g. (exercise) ECG, cardiac enzymes, echocardiogram). Subjects will then be followed up for at least one year in a frequency determined by the Cardiologist.

Such adverse events fulfill the definition of an "adverse event of special interest" and are to be handled respectively reported as described under section 8.4.

Using replication-competent vaccinia-based smallpox vaccines during smallpox vaccination programs in the US during the last years, cases of acute myocarditis and pericarditis were observed (Grabenstein and Winkenwerder, 2003).

Case definitions as published by the US Center of Disease Control (CDC) ("Update: Cardiac-Related Events During the Civilian Smallpox Vaccination Program --- United States, MMWR May 30, 2003, Vol. 52, No. 21, p. 494") are provided in Appendix II to recognize possible cases of acute myocarditis and pericarditis and to distinguish unspecific and isolated ECG changes without or with unclear clinical meaning and ECG changes related to possible or probable cases of acute myocarditis and/or pericarditis.

7.1.5. Assessment of solicited adverse events (Diary)

After each vaccination each subject receives a diary to record solicited local and general adverse events most likely to occur on the day of vaccination or the following 7 days. All solicited adverse events observed after vaccination with details concerning the intensity and the course of the event should be documented there. The investigator will collect this information during the next scheduled visit and will assess the relationship of the solicited general events to study medication.

In case of severe and unexpected local and systemic reactions, the study physician should be contacted outside from scheduled study visits.

SOLICITED LOCAL ADVERSE EVENTS

The solicited local symptoms erythema, swelling, induration and pain at the injection site have to be documented in the diary by the subject and intensity is assessed in the following way:

Injection site erythema:	size measured in diameter
Injection site swelling:	size measured in diameter
Injection site induration:	size measured in diameter

The maximum intensity will be scored as follows:

0 =	0
1 =	< 30 mm
2 =	≥ 30 – <100 mm
3 =	≥ 100 mm

Injection site pain:

0 = Absent
1 = Painful on touch
2 = Painful when limb is moved
3 = Spontaneously painful / prevents normal activity

SOLICITED GENERAL ADVERSE EVENTS

Subjects are asked to document the solicited general adverse events as described in the table below on the day of vaccination and the following 7 days on their diary card.

In the subject's diary the grading of maximum symptom intensity is described in basic, easily understood language based on the following descriptions.

MedDRA coded Preferred Term General Adverse Events	Grade	Maximum Intensity
Body temperature*	0<br>1<br>2<br>3<br>4	< 99.5°F (< 37.5°C)<br>≥ 99.5 - <100.4°F (≥ 37.5° – < 38.0°C)<br>≥ 100.4 – 102.2°F (≥ 38.0 – < 39.0°C)<br>≥ 102.2 - 104°F (≥ 39.0 - < 40.0°C)<br>≥ 104°F (40.0°C)
Headache, Myalgia, Nausea and Fatigue	0<br>1<br><br>2<br>3<br>4	None<br>Mild: easily tolerated, minimal discomfort and no interference with daily activity<br>Moderate: Some interference with daily activity<br>Severe: Prevents daily activity<br>Life-threatening or disabling

***Pyrexia** is defined as oral temperature ≥ 100.4°F (≥38.0°C).

Causal relationship between solicited general adverse events and the study vaccine will be assessed by the investigator.

7.1.6. Assessment of unsolicited adverse events

During every study visit following screening, the investigator has to report any unsolicited adverse event experienced by the subject.

Unsolicited adverse events following the vaccination will generally be recorded by the subjects in a special section of the diary card and transferred by the investigator to the adverse event section of the CRF. In addition, all intercurrent diseases not recorded on the diary card but reported when the investigator actively inquires of the subject will be documented in the respective section of the case report form.

Adverse Events will be documented and followed up until completion of the study.
Serious adverse events ongoing at the time of the F-U Visit will be followed up until resolution or achievement of stable clinical conditions.

7.1.7. Assessment of intensity for adverse events

The scale for grading the maximum intensity of all adverse events will be based on the following descriptions:

1 = An adverse event which is easily tolerated by the subject, causing minimal discomfort and not interfering with daily activities.
2 = An adverse event which is sufficiently discomforting to interfere with daily activities.
3 = An adverse event which prevents daily activities. (Such an adverse event would, for example, prevent attendance at work and would necessitate the administration of corrective therapy.)
4 = Life threatening or disabling

7.1.8. Assessment of causality for adverse events

The relationship between the occurrence of an adverse event and the study drug will be assessed using the following categories:

**None**	The time interval between the administration of the study drug and the occurrence or worsening of the AE rules out a relationship
	and/or
	another cause is established and there is no evidence of a (concomitant) causal connection with or worsening caused by the study medication.
**Unlikely**	The time interval between administration of the study drug and the occurrence or worsening of the AE makes a causal relationship unlikely
	and/or
	the known effects of the study medication or substance class provide no indication of a (concomitant) causal connection with or worsening caused by the study medication and there is another cause which serves as an adequate explanation
	and/or
	although the known effects of the study medication or substance class make it possible to derive a plausible causal chain with regard to a (concomitant) causal connection or worsening, however, another cause is considerably more likely
	and/or
	another cause of the AE has been identified and a (concomitant) causal connection with or worsening caused by the study medication is unlikely.
**Possible**	A plausible causal chain with regard to a (concomitant) causal connection with / worsening of the AE can be derived from the pharmacological properties of the study medication or substance class. However, other approximately equally likely causes are known
	or
	although the pharmacological properties of the study medication or substance class provide no indication of a (concomitant) causal connection with / worsening of the AE, there is no other known cause which provides an adequate explanation.
**Probable**	The pharmacological properties of the study medication or substance class
	and/or
	the course of the AE after discontinuation of the study drug and possible subsequent re-exposure
	and/or
	specific findings (e.g. positive allergy test or antibodies against the trial drug / metabolites) suggest a (concomitant) causal connection with / worsening of the AE resulting from the study medication, however another cause cannot completely be ruled out.
**Definite**	The pharmacological properties of the study medication or substance class
	and/or
	the course of the AE after discontinuation of the study drug and possible subsequent re-exposure
	and/or
	specific findings (e.g. positive allergy test or antibodies against the trial drug / metabolites) definitely indicate that there is a (concomitant) causal connection with / worsening of the AE resulting from the study medication and there are no indications of other causes.

7.2. Assessment of immunogenicity: Antibody Response

Immune response analysis is planned at any study visit except the screening visit. The baseline assessment for immunogenicity parameters will be performed during Visit 1 (before vaccination).

The method of collection, storage and handling of laboratory specimen for the immune analysis is specified in SOP/CA/20: "Collection preparation and storage instructions for immune response serum specimens" (Doc. No. 10000213). A written instruction for this procedure will be provided to the investigators before enrollment. Additionally, the procedure will be introduced during the investigator meeting and/or at the initiation visit.

Antibody responses against MVA-BN® will be measured using a direct ELISA and a plaque reduction neutralization test (PRNT) using established and validated in house assays.

Immune analyses will be performed at Bavarian Nordic's laboratory at Bavarian Nordic GmbH, 82152 Martinsried, Germany. The protocols for the analytical tests performed are detailed in the following SOPs and will be provided in the Trial Master File:

SOP/CA/06: "Standard ELISA for detection of MVA specific antibodies in human sera" (Doc. No. 10000199).
SOP/PRE/17: "Vaccinia Virus (VV) Specific Plaque Reduction Assay for the Detection of Neutralizing Antibodies in Human Sera" (Doc. No. 10000210).
SOP/CA/29: "(Automated) Standard ELISA for detection of MVA specific antibodies in human sera" (Doc. No. 10000262).

The names and titles of the mentioned SOPs can be subject to changes or updates during the trial. A changed or updated SOP will always retain the same document number and will immediately be provided to the investigator upon availability.

<u>ELISA</u>
Seroconversion is defined as:
Appearance of antibody titers ≥ 1:50 in a vaccinia specific IgG ELISA for initially seronegative subjects or twofold increase of the antibody titer compared to the pre-existing baseline titer for subjects with a pre-existing antibody titer in the ELISA.

The geometric mean titer (GMT) is calculated by taking the antilogarithm of the mean of the log10 titer transformations. Antibody titers below the cut-off of the assay will be given an arbitrary value of one for the purpose of calculation.

<u>PRNT</u>
Seroconversion is defined as:
Appearance of antibody titers ≥ 1:20 in a vaccinia specific plaque reduction neutralization assay for initially seronegative subjects or twofold increase of the antibody titer compared to the pre-existing baseline titer for subjects with a pre-existing antibody titer in the PRNT.

The GMT is calculated by taking the antilogarithm of the mean of the log10 titer transformations. Antibody titers below the cut-off of the assay will be given an arbitrary value of one for the purpose of calculation.

8. Adverse event definitions and reporting

Any signs and symptoms that occur before first vaccination will be recorded in the baseline signs and symptoms section and will not be considered as adverse events.

8.1. Definition of adverse event

Adverse events are defined as any untoward (undesirable) medical occurrence in a clinical trial subject administered a medicinal product and which does not necessarily have a causal relationship with this medication. All adverse events (e.g. feeling of ill-health, subjective symptoms and objective signs, intercurrent diseases, accidents, etc.) observed by the investigator and/or reported by the subject must be recorded in the CRF regardless of the assessment of causality in relationship with the study drug.

Abnormal laboratory values that were assessed as clinically significant by the investigator are to be documented as AEs. In addition, abnormal laboratory values fulfilling the Grade 3 criterion according to the toxicity scale (Appendix I) are to be documented as AEs, regardless of whether they are considered clinically relevant or not.

8.2. Definition of solicited adverse events

Within this study protocol solicited adverse events are defined as all events recorded by the subjects in the diary provided to them following every vaccination. For each of the 7 days post-vaccination the subjects are requested to monitor and record local symptoms, i.e. erythema, swelling, induration and pain at the site of injection as well as general symptoms, i.e. body temperature, headache, myalgia, nausea and fatigue.

8.3. Definition of unsolicited adverse events

At every study visit the investigator should ask the subject if they have experienced any adverse events since their last visit. All intercurrent diseases reported by the subject, regardless of whether recorded in the subject diary or not, need to be recorded by the investigator in the appropriate page of the case report form.

The intensity and causality of the events will be graded according to the procedures described under 7.1.7 and 7.1.8 respectively.

8.4. Definition of "adverse event of special interest"

An "adverse event of special interest" is defined in this study as an adverse event fulfilling any of the following features:

- Any cardiac symptom
- ECG changes, which are determined to be clinically significant
- Cardiac enzymes elevated above ULN

Adverse events of special interest are to be reported according to the procedures and timelines applicable for serious adverse events.

8.5. Definition of serious adverse events (SAEs)

A serious adverse event (experience) or reaction is any untoward medical occurrence or effect that at any dose:

1. Results in death,
2. Is life-threatening*,
3. Requires inpatient hospitalization or prolongation of existing hospitalization,
4. Results in persistent or significant disability or incapacity,
5. Is a congenital anomaly or birth defect,
6. or is an otherwise important medical event.

**The term "life-threatening" in the definition of "serious" refers to an event in which the patient was at risk of death at the time of the event; it does not refer to an event which hypothetically might have caused death if it were more severe.*

Medical and scientific judgment should be exercised in deciding whether expedited reporting is appropriate in other situations, such as important medical events that may not be immediately life-threatening or result in death or hospitalization but may jeopardize the patient or may require intervention to prevent one of the other outcomes listed in the definition above. These should also usually be considered serious.

Examples of such events are intensive treatment in an emergency room or at home for allergic bronchospasm; blood dyscrasias or convulsions that do not result in hospitalization; or development of drug dependency or drug abuse.

8.6. Reporting of SAEs and adverse events of special interest

All serious adverse events (SAEs) or adverse events of special interest (see definition 8.4) occurring throughout the entire course of the study have to be reported to Bavarian Nordic´s Drug Safety Department. The study site has to send by e-mail or fax the completed serious adverse event form to Bavarian Nordic’s Drug Safety Department within 24 hours of becoming aware of the adverse event.

In compliance with regulations Bavarian Nordic will take care of expedited reporting of adverse events as required to the regulatory authorities and to the responsible NIH-DMID representative.

<u>SAEs</u>: BN will send copies of all SAEs and any supporting documents electronically to DMID within 3 business days of receipt by BN.
<u>Safety Reports</u>: BN will provide DMID with copies of all IND safety report submissions within 3 business days of the submission to the FDA.
<u>Site Monitoring Reports</u>: BN will provide DMID with a copy of all site monitoring reports on a monthly basis.

Please mail SAE reports to the following address: drug.safety@bavarian-nordic.com

***OR* fax the completed SAE report to the number: +49-89-8565 1419**

In case any problems occur when trying to transmit an SAE report or with all questions specifically related to the safety of a subject enrolled in the study please contact one of the below listed responsible persons:

Garth Virgin, MD<br>Drug Safety Officer, Bavarian Nordic<br>Phone: +49-89-8565 1319<br>(working hours)<br>Phone: +49-176-22 633 107<br>(after hours)	or (Backup)	Jens Vollmar, MD<br>Medical Monitor, Bavarian Nordic<br>Phone: +49-89-8565 1308<br>(working hours)<br>Phone: +49-172-86 23 292<br>(after hours)

The investigator should not delay reporting because of missing information. Nonetheless, the report should be as complete as possible. This initial notification should include, as a minimum, sufficient information to permit identification of the following:

- the reporter
- adverse event(s)
- involved study medication
- the subject
- date of onset

9. Statistical Considerations

The primary objective of this trial is to compare the ELISA specific humoral immune response of the group with history of smallpox vaccination versus the group without history of smallpox vaccination, i.e. to prove that a single dose schedule would be sufficient to boost the immunity in a previously vaccinated population.

9.1. Sample size calculation

The sample size calculation is based on the **primary immunogenicity variable 'MVA - specific seroconversion rate'**. This parameter is derived from the ELISA specific antibody titers. Seroconversion is defined as the appearance of antibody titers $\geq$ 1:50 in a vaccinia specific IgG ELISA for initially seronegative subjects or twofold increase of the antibody titer compared to the pre-existing baseline titer for subjects with a pre-existing antibody titer in the ELISA.

The primary hypothesis is to show that the humoral immune response of the group with history of smallpox vaccination (Group 4) is not statistically inferior compared to the group without history of smallpox vaccination (Group 1). The study should demonstrate that the Group 4 seroconversion rate is not worse than the Group 1 seroconversion rate by more than a pre-specified amount. This amount is called the non-inferiority margin (Δ).

Suppose p_1 is the seroconversion rate in the group without history of smallpox vaccination and p_4 is the seroconversion rate in the subjects with history of smallpox vaccination.
The test on non-inferiority will be applied for the following hypothesis:

H_0: $p_4 - p_1 \leq -\Delta$ versus H_1: $p_4 - p_1 > -\Delta$, where

Δ is the non-inferiority margin and is chosen in this trial as 5%.

From the experience in a pilot trial with MVA-BN® in healthy subjects it is anticipated that the seroconversion rate in healthy vaccinia-naive subjects reaches 98-100%.

Assuming a significance level of 5%, a power of 80%, expected seroconversion rates of 98% in both groups, this yields to a sample size of 175 per group (700 in total). In order to account for drop outs, **a total of 200 subjects per group (800 in total)** will be treated.

For reference see Agresti A, Min Y. (2001) and Chan I (1998).

9.2. Variables

9.2.1. Immunogenicity variables

Primary variable

MVA -specific seroconversion rate derived from the ELISA specific antibody titers 2 weeks after the last vaccination (Group 1-3: Visit 4, Group 4: Visit 2).
Seroconversion is defined as the appearance of antibody titers ≥ 1:50 in a vaccinia specific IgG ELISA for initially seronegative subjects or twofold increase of the antibody titer compared to the pre-existing baseline titer for subjects with a pre-existing antibody titer in the ELISA.

Secondary variables:

MVA -specific seroconversion rate derived from the ELISA specific antibody titers 4 weeks after the last vaccination (Group 1-3: Visit 5, Group 4: Visit 3).

MVA -specific seroconversion rate derived from the PRNT specific antibody titers 2 weeks after the last vaccination (Group 1-3: Visit 4, Group 4: Visit 2) and 4 weeks after the last vaccination (Group 1-3: Visit 5, Group 4: Visit 3).
Seroconversion is defined as appearance of antibody titers ≥ 1:20 in a vaccinia specific plaque reduction neutralization assay for initially seronegative subjects or twofold increase of the antibody titer compared to the pre-existing baseline titer for subjects with a pre-existing antibody titer in the PRNT.

9.2.2. Safety and reactogenicity variables

Primary variables:

Occurrence, relationship and intensity of any specific or unspecific ECG change at any time during the study.

Occurrence, relationship and intensity of any other cardiac symptom at any time during the study.

Secondary variables:

Occurrence of any serious adverse event probably, possibly or definitely related to the study vaccine at any time during the study.

Occurrence of any grade 3 or higher adverse reaction probably, possibly or definitely related to the study vaccine within 28 days after vaccination.

Occurrence of solicited local adverse events within 1 week (Days 0-7) after vaccination: Intensity, duration, and relationship to vaccination.

Occurrence of solicited general adverse events within 1 week (Days 0-7) after vaccination: Intensity, duration, and relationship to vaccination.

Occurrence of unsolicited non-serious adverse events within 28 days after vaccination: Intensity, duration and relationship to vaccination.

9.3. Study cohorts/data sets to be evaluated

For the statistical analysis the included subjects will be divided up into the following datasets:

Safety population: This is the subset of subjects who are randomized, have received at least one dose of study vaccine and for whom safety data are available.

The main analysis of safety will be performed on this population.

Full-analysis (FA): This is the subset of subjects who are randomized and had received at least one dose of study vaccine. Relevant protocol violators are included into this dataset. Patients who drop out before Visit 5 (for Group 1-3) or Visit 3 (for Group 4) are included here.

Per Protocol (PP): This is the subset of subjects who are randomized and had received two doses of study vaccine (group 1-3), respectively one dose of study vaccine (group 4). Subjects have to complete Visit 5 (for Group 1-3), respectively Visit 3 (for Group 4). Subjects have to adhere to all protocol conditions, however mild (not relevant) protocol violators can be included into this dataset.

The decision whether a protocol deviation is relevant or not for the classification of subjects to subsets should be made case-by case in a blind review meeting.

The primary population dataset will be the FA Set. All confirmatory testing is based on this subgroup. For further descriptive purposes, the same statistical procedures will be applied to the PP dataset.

Analysis of immunogenicity variables will be done on a valid case basis, i.e. for missing observations no imputation technique like LOCF will be applied, because this would imply a too optimistic approach.

9.4. Statistical analysis

As soon as the last subject has completed Visit 5 (for Group 1-3), respectively Visit 4 (for Group 4) and after any necessary settlement of queries etc. in the CRFs, data from those patients and visits will be locked. A full analysis of the data available will then be performed. Once all subjects have completed the follow-up (Visit F-U), an addendum to the clinical report will be prepared including all follow-up data.

9.4.1. Analysis of demographics and baseline characteristics

An analysis of variance (ANOVA) for the main baseline characteristics (age, weight, height) will be performed. The ANOVA includes treatment and location effects and will be performed to detect any global treatment or location effect. The model does not include a term for location by treatment interaction (according to ICH guideline E9, Statistical Principles for Clinical Trials, p.16).

9.4.2. Analysis of Immunogenicity

Antibody titers and resulting seroconversion rates will be assessed by direct ELISA and PRNT method as described in section 7.2.
The primary immunogenicity variable is the MVA -specific seroconversion rate. This parameter is derived from the ELISA specific antibody titers.

The primary hypothesis is to show that the humoral immune response of the group with history of smallpox vaccination (Group 4) is not statistically inferior compared to the group without history of smallpox vaccination (Group 1).

The above hypothesis will be tested based on an exact, unconditional test for binomial differences. In addition an exact one-sided 97.5% unconditional confidence interval for the difference of proportions will be calculated. If the lower limit of this confidence interval is greater than 5% (or equivalent the p-value of the non-inferiority test is less than 5%) then the null hypothesis will be rejected (StatXact®, Chan (1998), Agresti and Min (2001)).

In order to limit the overall type-I-error to a nominal level of 5%, a **hierarchical test procedure** will be chosen:

In a first step, the primary null hypothesis as stated in section 9.1 will be tested 2 weeks after the last vaccination (Group 1-3: Visit 4, Group 4: Visit 2). Only if this comparison shows a significant result, the comparison at 4 weeks after vaccination (Group 1-3: Visit 5, Group 4: Visit 3) will be done.

A secondary analysis will be done with the neutralization assay specific seroconversion rates (from PRNT) as above.

In addition to the main comparison of Group 4 versus Group 1, additional, secondary comparisons will be made among all other groups.

All statistical tests for secondary time points and comparisons are regarded descriptive. No adjustment for multiple testing will therefore be done.

Tables and graphs showing the seroconversion rates for each group at visit will be prepared.
Descriptive statistics and graphical displays will be given for the geometric mean titer for all groups at all visits.

9.4.3. Analysis of safety and reactogenicity

Solicited local adverse events

The occurrence of solicited local adverse events within 1 week after each vaccination will be summarized on a per patient and per vaccination basis.
The maximum intensity over the 7-day period after vaccination will be used and categorized as follows:

Pain:

Grade = 0 / > 0
Grade < 2 / ≥ 2
Grade < 3 / = 3

For measurements of diameter size:

Diameter = 0 / > 0
Diameter < 30mm / ≥ 30 mm
Diameter < 100mm/ ≥ 100 mm

These categories will be compared between treatment groups by means of the exact test of Fisher (or its normal approximation, the chi-squared test).

The occurrence of any grade 3 or higher adverse reaction probably, possibly or definitely related to the study vaccine within 28 days after vaccination will be compared between treatment groups.

Solicited general adverse events

Occurrence of solicited general adverse events within 1 week after each vaccination will be summarized per patient and vaccination.

The maximum intensity over the 7-day period after vaccination will be used and categorized as follows:

Grade = 0 > 0
Grade < 2 ≥ 2
Grade < 3 / ≥ 3

These categories will be compared between treatment groups by means of the exact test of Fisher (or its normal approximation, the chi-squared test).

The occurrence of any grade 3 or higher adverse reaction probably, possibly or definitely related to the study vaccine within 28 days after vaccination will be compared between treatment groups.

Unsolicited adverse events:

Unsolicited adverse events will be coded with the MedDRA coding terminology. The intensity of adverse events will be graded according to section 7.1.7.
The number of adverse events and number of patients with at least one adverse event for each preferred term will be descriptively compared between treatment groups.

The occurrence of any grade 3 or higher adverse reaction probably, possibly or definitely related to the study vaccine within 28 days after vaccination will be compared between treatment groups.

Adverse events of special interest will be separately listed and tabulated. The incidence of such AEs will be compared between treatment groups by means of the exact test of Fisher (or its normal approximation, the chi-squared test).

Serious adverse events will be listed separately. Each SAE will be described individually in detail. The number of subjects with at least one SAE will be compared between treatment groups by means of the exact test of Fisher (or its normal approximation, the chi-squared test).

The occurrence, relationship and intensity of any *other cardiac symptom* at any time during the study will be listed. The number of subjects with at least one cardiac symptom will be compared between treatment groups by means of the exact test of Fisher (or its normal approximation, the chi-squared test).

Safety laboratory and urine analysis:
Clinical laboratory test results will be marked whether the result is below, within or above the respective reference range. The number of values outside of the reference range will be counted.

ECG
The ECG will be evaluated by a centralized procedure. The transmitted standard ECG results like PQ, QRS, QT and QTc duration and heart rate will be summarized per visit and treatment group.

The number of subjects with normal/abnormal ECGs and clinically significant ECGs (resulting in AEs) will be compared between treatment groups by means of the exact test of Fisher (or its normal approximation, the chi-squared test).
Detailed descriptive analysis of the reasons (category) of abnormality will be done.

9.4.4. Data handling

All data obtained in this study and documented in the CRF's will be listed. For parameters of interest, summary tables with descriptive group statistics (mean, standard deviation, minimum, maximum, number of valid cases) for metrical variables will be prepared. For ordinal/dichotomous variables summary tables showing the absolute and relative count in each category will be prepared.
Further statistical analysis may be performed as appropriate.
9.4.5.

10. Ethical aspects

10.1. Ethical and legal regulations

The Principal Investigators make sure that this clinical trial is conducted in complete accordance with the provisions of the Declaration of Helsinki (and its amendments of Tokyo, Venice, Hong Kong, Somerset West and Edinburgh), the national laws and other guidelines for the conduct of clinical studies like the ICH Harmonized Tripartite Guideline for Good Clinical Practice to guarantee the greatest possible subject protection.

10.2. Approval of an ethics committee

The protocol must be reviewed by the competent ethics committee according to the national law of the respective site before the first subject is included in this study.

If one of the investigators is a member of one of these committees, he may not vote on any aspect of the review of this protocol.

The Sponsor will assure that the ethics committee is informed of any amendment to the protocol and any unanticipated problems involving risks to human subjects included in the study. Such information will be provided to the committee at intervals appropriate to the degree of subject risk involved, but not less than once a year. Copies of all correspondence between the investigator and the committee must be forwarded immediately to the sponsor. In case of withdrawal of ethics committee approval of the study, the sponsor has to be contacted immediately by facsimile or telephone.

10.3. Confidentiality and data protection

The Principal Investigator of the respective site is obliged to ensure anonymity of the subject. He/she has to make sure that all documents including CRFs provided (e.g. in the course of a marketing authorization procedure) to third parties (in this case: to the manufacturer of MVA-BN® or to an authority) contain no subject names.
Only a subject and centre number, not by their name or clinic and subject's file number, may identify subjects respectively. The Principal Investigators keep separate confidential subject logs for study enrollment, which allows subject numbers to be matched with names and addresses of subjects at any time. Documents not meant to be passed on to third parties have to be stored confidentially by the Principal Investigator.

Any information collected in the course of the study may be made available only to persons directly involved in this study (Principal Investigator and his staff members, monitor, statistician) or to authorized persons by the sponsor or the Principal Investigator or authorities.

11. Informed consent

No subject can participate in this study without having given informed consent in writing after the investigator or his delegate has informed the subject clearly and completely, verbally and in writing, over the purpose, procedures, potential benefits and risks of the current study and prior to study drug administration.

One signed copy of the informed consent must be given to each subject and one signed copy must remain in the study documentation file and be available for verification by the monitor or competent regulatory authorities at any time.

Subjects must be informed unequivocally that they may refuse participation in the study and that they may withdraw from the study at any time and for whatever reason and that withdrawal of consent will not affect their subsequent medical treatment or relationship with the treating physician.

Subjects also consent to authorize the monitor, quality assurance personnel and regulatory authorities to inspect source documents for quality assurance purposes. Such verifications will always be conducted on site and under the ethical supervision of the investigator. All aspects of the confidentiality of the subject's data will be guaranteed.

The informed consent form must be submitted to the ethics committee. Subjects will be informed about the following:

- Purpose of the research, duration of participation, procedures to be followed, including any that are experimental
- Reasonable foreseeable risks or discomforts
- Extent, if any, to which confidentiality of record will be maintained, and notification that regulatory authorities, the sponsor and the monitor may inspect the records
- Whether any compensation or medical treatments are available if injury occurs
- Who to contact for answers to question or in event of injury
- Statement that participation is voluntary and the subject has the right to withdraw at any time without penalty or loss of benefits
- Subject insurance & insurance conditions.

12. Case report forms and retention of records

12.1. Electronic case report forms

All of the clinical data will be captured via electronic data capture (EDC) using a web-based tool. The investigator site staff will enter and edit the data via a secure network, with secure access features (username and password). A complete electronic audit trail will be maintained. The investigator will approve the data using an electronic signature (Ref: 21 CFR Part 11), and this approval is used to confirm the accuracy of the data recorded.

eCRFs will be used for all patients. The investigator’s data will be accessible from the investigator’s site throughout the trial. The electronic CRFs must be kept current to reflect patient status at each phase during the course of the trial. The subjects are identified on the electronic CRF by name and initials. The investigator must make a separate confidential record of these details (subject identification and enrollment log). While the trial is ongoing and until the access to the database has been terminated, there will be no documentation of changes. All changes to data are done from the investigator through the EDC system. If a change is necessary once the investigator has no further access to the database, a request for change will be sent to the investigator for confirmation of the change.

It is the responsibility of the Principal Investigator of the respective site to ensure that all subject discontinuations or changes in study or other medications entered on the subject’s eCRF are also made on the subjects medical records.

The eCRFs for any subject leaving the study should be completed at the time of the final visit or shortly thereafter.

12.2. Retention of records

The Principal Investigator shall maintain the records of disposition of drug receipts and drug inventory logs, subject files and regulatory documents (informed consents, ethical approval) for 15 years after the end of the study.
To meet regulatory requirements, the eCRF data will be electronically stored at sites. The CDISC ODM (see www.cdisc.org for details) will be used to store and archive all electronic data at the sites. Since CDISC ODM is also the source for the EDC-web-based system, no transcription of data is necessary.
CDISC ODM is a plat-form independent standardized data format including the complete study metadata and audit trail. The data can be reviewed at a later stage using off-the-shelf tools. CDISC is providing a complete CDISC ODM Viewer for these purposes.
If needed, PDF-files can be created from the ODM file.

13. Monitoring of the study

The monitor is responsible for obtaining an overview of the course of the trial in co-operation with the investigator, checking if the trial protocol is being observed, and helping the investigators to solve any problems which may arise. All documents in the context with this clinical trial will be handled confidentially at any time.

Data reported in the eCRFs (electronic CRFs) that are transcribed from source documents must be consistent with the source documents or the discrepancies must be explained.

For data that is directly captured in the eCRF (eSource), the eCRF itself becomes source data and source data verification is not applicable.

The Principal Investigator of the respective site agrees to give the monitor access to relevant hospital or clinical records to confirm their consistency with the CRF entries and to obtain an adequate overview of the course of the trial. The monitor checks entries on the CRF for completeness, accuracy and correctness. The entries on the CRF will be verified against source documents. This will be done under preservation of data protection.

The items for source data verification will be specified in detail in the monitoring manual.

The source data verification must be performed by direct viewing. If a subject refuses to consent to this procedure he/she must not be enrolled in the study.
The investigator (or a representative) has further agreed to support the monitor in solving any problems he/she discovers during his/her visits.

Monitoring of drug accountability will be performed by an additional independent monitor to ensure that the monitor responsible for source data verification is kept blinded.

14. Responsibilities of the investigator

The Principal Investigator of the respective site agrees to carry out the study in accordance with the guidelines and procedures outlined in this trial protocol. The Principal Investigator especially consents to strictly adhere to the ethical principles (see section 10 of this protocol).

The Principal Investigator knows that he/she must, according to professional regulations for physicians, obtain the approval of the relevant Ethics Committee.

Any deviation from the trial protocol must, before its implementation, be agreed to by the sponsor in writing, and by the Ethics Committee initially consulted.

Changes to the protocol require written ”Amendments to the protocol” and written approval by the Principal Investigator. Changes are allowed only if study value is not reduced and if they are ethically justifiable. The statistician must agree to the amendment, if appropriate, his statement is to be submitted to the Ethics Committee. The amendment must be passed on to all participating investigators with the obligation to adhere to its provisions. If warranted, the subject information has to be changed accordingly.

It is within the responsibility of the investigator that a CRF is completed and signed after the subject has finished the trial for each subject participating in the study.

Since the electronic signature on the eCRF is a legally binding signature which is equivalent to a handwritten signature and dating on a document, it is the responsibility of the investigator not to pass any information about username and password to other persons.
At the conclusion of the study, the investigator will return all partly used, unused and empty drug containers to the sponsor.

The Principal Investigator may ask to terminate the study due to administrative or other reasons. If this should be the case, appropriate measures which safeguard the interests of the participating subjects must be taken after verification.

By signing this protocol, the Principal Investigator confirms that he/she has read the entire trial protocol, agrees to its procedures, and will comply strictly with the formulated guidelines.

15. References

1. Agresti A, Min Y. (2001). On small-sample confidence intervals for parameters in discretedistributions. *Biometrics. 2001 Sep;57(3):963-71*

2. Alcami A & Smith GL (1992) A soluble receptor for interleukin-1 beta encoded by vaccinia virus: a novel mechanism of virus modulation of the host response to infection.*Cell* 71, 153-167.

3. Alcami A & Smith GL (1996) A mechanism for the inhibition of fever by a virus.*Proc.Natl.Acad.Sci.U.S.A* 93, 11029-11034.

4. Antoine G, Scheiflinger F, Dorner F, & Falkner FG (1998) The complete genomic sequence of the modified vaccinia Ankara strain: comparison with other orthopoxviruses.*Virology* 244, 365-396.

5. Blanchard TJ, Alcami A, Andrea P, & Smith GL (1998) Modified vaccinia virus Ankara undergoes limited replication in human cells and lacks several immunomodulatory proteins: implications for use as a human vaccine. *J.Gen.Virol.* 79 (Pt 5), 1159-1167.

6. Carroll MW & Moss B (1997) Host range and cytopathogenicity of the highly attenuated MVA strain of vaccinia virus: propagation and generation of recombinant viruses in a nonhuman mammalian cell line. *Virology* 238, 198-211.

7. Chan I (1998). Exact tests of equivalence and efficacy with a non-zero lower bound for comparative studies. *Statistics in Medicine* 17, 1403-1413.

8. Drexler I, Heller K, Wahren B, Erfle V, & Sutter G (1998) Highly attenuated modified vaccinia virus Ankara replicates in baby hamster kidney cells, a potential host for virus propagation, but not in various human transformed and primary cells. *J.Gen.Virol.* 79 (Pt 2), 347-352.

9. Goldstein JA, Neff JM, Lane JM, & Koplan JP (1975) Smallpox vaccination reactions, prophylaxis, and therapy of complications. *Pediatrics* 55, 342-347.

10. Guillaume JC, Saiag P, Wechsler J, Lescs MC, & Roujeau JC (1991) Vaccinia from recombinant virus expressing HIV genes. *Lancet* 337, 1034-1035.

11. Grabenstein JD, Winkenwerder W. Jr. (2003) US Military Smallpox Vaccination Program Experience. JAMA 2003, Vol. 289, No. 24, 3278 - 3282

12. Lane JM, Ruben FL, Neff JM, & Millar JD (1969) Complications of smallpox vaccination, 1968.*N.Engl.J.Med.* 281, 1201-1208.

13. Lane JM, Ruben FL, Neff JM, & Millar JD (1970) Complications of smallpox vaccination, 1968: results of ten statewide surveys. *J.Infect.Dis.* 122, 303-309.

14. Mahnel H & Mayr A (1994) [Experiences with immunization against orthopox viruses of humans and animals using vaccine strain MVA]. *Berl Munch.Tierarztl.Wochenschr.* 107, 253-256.

15. Mayr A, Hochstein-Mintzel V, & Stickl H (1975) Passage History: Abstammung, Eigenschaften und Verwendung des attenuierten Vaccina-Stammes MVA. *Infection* 3, 6-14.

16. Mayr A & Munz E (1964) [Changes in the vaccinia virus through continuing passages in chick embryo fibroblast cultures]. *Zentralbl.Bakteriol.[Orig.]* 195, 24-35.

17. Mayr A, Stickl H, Muller HK, Danner K, & Singer H (1978) [The smallpox vaccination strain MVA: marker, genetic structure, experience gained with the parenteral vaccination and behavior in organisms with a debilitated defence mechanism (author's transl)].*Zentralbl.Bakteriol.[B]* 167, 375-390.

18. McElwain WP (1972) Complications of smallpox vaccination.*J.Ky.Med.Assoc.* 70, 165-166.

19. Meyer H, Sutter G, & Mayr A (1991) Mapping of deletions in the genome of the highly attenuated vaccinia virus MVA and their influence on virulence. *J.Gen.Virol.* 72 (Pt 5), 1031-1038.

20. Redfield RR, Wright DC, James WD *et al.* (1987) Disseminated vaccinia in a military recruit with human immunodeficiency virus (HIV) disease. *N.Engl.J.Med* 316, 673-676.

21. Rosel JL, Earl PL, Weir JP, & Moss B (1986) Conserved TAAATG sequence at the transcriptional and translational initiation sites of vaccinia virus late genes deduced by structural and functional analysis of the HindIII H genome fragment. *J.Virol.* 60, 436-449.

22. Stickl, H. A. (1974) Smallpox vaccination and its consequences: first experiences with the highly attenuated smallpox vaccine "MVA". Prev.Med, 3[1], 97-101.

23. Stickl H & Hochstein-Mintzel V (1971) [Intracutaneous smallpox vaccination with a weak pathogenic vaccinia virus ("MVA virus")]. *Munch.Med.Wochenschr.* 113, 1149-1153.

24. Stickl H, Hochstein-Mintzel V, Mayr A *et al.* (1974) [MVA vaccination against smallpox: clinical tests with an attenuated live vaccinia virus strain (MVA) (author's transl)]. *Dtsch.Med.Wochenschr.* 99, 2386-2392.

25. Sutter G & Moss B (1992) Nonreplicating vaccinia vector efficiently expresses recombinant genes. *Proc.Natl.Acad.Sci.U.S.A* 89, 10847-10851.

16. Appendices

Appendix I: Toxicity Scale for laboratory values

SERUM CHEMISTRY				
**Lab value**	**Mild (Grade 1)**	**Moderate (Grade 2)**	**Severe (Grade 3)**	**Potentially Life Threatening (Grade 4)**
Sodium – Hyponatremia mmol/L	< LLN - ≥ 132	< 132 - ≥ 130	< 129 - ≥ 125	<125
Sodium – Hypernatremia mmol/L	≥ ULN - < 146	≥ 146 - < 148	≥ 148 - < 150	≥ 150
Potassium – Hyperkalemia mmol/L	≥ ULN - < 5.3	≥ 5.3 - < 5.5	≥ 5.5 - < 5.6	≥ 5.6
Potassium – Hypokalemia mmol/L	< LLN - ≥ 3.5	< 3.5 - ≥ 3.4	< 3.4 - ≥ 3.2	< 3.2
Calcium – Hypercalcaemia	≥ ULN - < 2.9	≥ 2.9 - < 3.1	≥ 3.1 - < 3.4	≥ 3.4
Calcium - Hypocalcaemia	< LLN - ≥ 2.0	< 1.75 - ≥ 2.0	< 1.6 - ≥ 1.75	< 1.5
Blood Urea Nitrogen BUN mg/dL	≥ ULN - < 27	≥ 27 - < 32	≥ 32	
Serum creatinine - mg/dL	≥ ULN - < 1.5 x ULN	≥ 1.5 - < 3 x ULN	≥ 3 - 6 x ULN	> 6 x ULN
Albumin - Hypoalbuminemia g/dL	< LLN - ≥ 2.7	< 2.7 - ≥ 2.5	< 2.5	
Total Protein - Hypoproteinemia g/dL	< LLN - ≥ 5.4	< 5.4 - ≥ 5.0	< 5.0	
Alkaline Phosphatase - increase by factor	> 1.25 - < 2.0 x ULN	≥ 2.0 - < 3.0 x ULN	≥ 3.0 x ULN	
Liver Function Tests - increase by factor	> 1.0 - < 2.5 x ULN	≥ 2.5 - < 4 x ULN	≥ 4 x ULN	
Total Bilirubin - increase by factor	> ULN – 1.5 x ULN	> 1.5 - 3.0 x ULN	> 3.0 - 10.0 x ULN	> 10.0 x ULN
Creatinine Kinase (CK)	>ULN - <2x ULN	≥ 2 - <5x ULN	≥ 5x ULN	
Creatine Kinase Myocardial Band (CK-MB)	>ULN – <2.0 x ULN	≥ 2.0 - < 5.0 x ULN	≥ 5.0 x ULN	
Cardiac Troponin I	>ULN – <2.0 x ULN	≥ 2.0 - < 5.0 x ULN	≥ 5.0 x ULN	
Total Cholesterol	> ULN - 300	> 300 - 400	> 400	

**HEMATOLOGY**				
**Lab Value**	**Mild (Grade 1)**	**Moderate (Grade 2)**	**Severe (Grade 3)**	**Potentially Life Threatening (Grade 4)**
Hemoglobin (Female) - g/dl	< LLN - ≥ 10.5	< 10.5 - ≥ 10.0	< 10.0	
Hemoglobin (Male) – g/dl	< LLN - ≥ 12.0	< 12.0 - ≥ 11.0	< 11.0	
WBC Increase - cell/mm^3	> ULN - < 15,000	≥ 15,000 - < 20,000	≥ 20,000	
WBC Decrease - cell/mm^3	< LLN - ≥ 2,500	< 2,500 - ≥ 1,500	< 1,500	
Lymphocytes Decrease - cell/mm^3	< 1,000 - ≥ 750	< 750 - ≥ 500	< 500	
Neutrophils Decrease - cell/mm^3	< 2,000 - ≥ 1,500	< 1,500 - ≥ 1,000	< 1,000	
Platelets Decreased - cell/mm^3	< LLN - ≥ 75,000	< 75,000 - ≥ 50,000	< 50.000	
**URINE**				
Protein	Trace - ≤ 1+	> 1+ - ≤ 2+	> 2+ - ≤ 3+	≥ 3+ - nephritic syndrome
Glucose	Trace	1+	2+	>2+
Blood (microscopic) – red blood cells per high power field (rbc/hpf)	≥ 0- <10	≥ 10 - < 50	≥ 50	Gross
**SYSTEMIC QUANTITATIVE**				
Tachycardia - beats per minute	101 - 115	116 - 130	≥ 131	
Bradycardia - beats per minute	54 - 50	49 - 45	≤ 44	
Hypertension (systolic) - mm Hg	141 - 155	156 - 165	≥ 166	
Hypertension (diastolic) - mm Hg	91 - 95	96 - 100	≥ 101	
Hypotension (systolic) - mm Hg	89 - 85	84 - 80	≤ 79	

Appendix II: Case Definitions Acute Myocarditis / Pericarditis

Case Definition for Acute Myocarditis

A possible case of acute myocarditis is defined by the following criteria and the absence of evidence of any other likely cause of symptoms:
Presence of dyspnea, palpitations, or chest pain of probable cardiac origin in a patient with either one of the following:

- Electrocardiogram (ECG) abnormalities beyond normal variants, not documented previously, including
  - ST-segment or T-wave abnormalities,
  - Paroxysmal or sustained atrial or ventricular arrhythmias,
  - AV nodal conduction delays or intraventricular conduction defects, or
  - Continuous ambulatory electrocardiographic monitoring that detects frequent atrial or ventricular ectopy

Or

- Evidence of focal or diffuse depressed left-ventricular (LV) function of indeterminate age identified by an imaging study (e.g., echocardiography or radionuclide ventriculography).

A probable case of acute myocarditis, in addition to the above symptoms and in the absence of evidence of any other likely cause of symptoms, has one of the following:

- Elevated cardiac enzymes, specifically, abnormal levels of cardiac troponin I, troponin T, or creatine kinase myocardial band (a troponin test is preferred);
- Evidence of focal or diffuse depressed LV function identified by an imaging study (e.g., echocardiography or radionuclide ventriculography) that is documented to be of new onset or of increased degree of severity (in the absence of a previous study, findings of depressed LV function are considered of new onset if, on follow-up studies, these findings resolve, improve, or worsen); or
- Abnormal result of cardiac radionuclide imaging (e.g., cardiac MRI with gadolinium or gallium-67 imaging) indicating myocardial inflammation.

A case of acute myocarditis is confirmed if histopathologic evidence of myocardial inflammation is found at endomyocardial biopsy or autopsy.

Case Definition for Acute Pericarditis

A possible case of acute pericarditis is defined by the presence of

- Typical chest pain (i.e., pain made worse by lying down and relieved by sitting up and/or leaning forward) and no evidence of any other likely cause of such chest pain.

A probable case of acute pericarditis is a possible case of pericarditis, or a case in a person with pleuritic or other chest pain not characteristic of any other disease, that, in addition, has one or more of the following:

- Pericardial rub, an auscultatory sign with one to three components per beat,
- ECG with diffuse ST-segment elevations or PR depressions without reciprocal ST depressions that are not previously documented, or
- Echocardiogram indicating the presence of an abnormal collection of pericardial fluid (e.g., anterior and posterior pericardial effusion or a large posterior pericardial effusion alone).

A case of acute pericarditis is confirmed if histopathologic evidence of pericardial inflammation is evident from pericardial tissue obtained at surgery or autopsy.

POX-MVA-005

EudraCT No. 2005-001781-14
DMID 05-0128

Amendment #1
to Final Protocol dated 12-Dec-2005

Study title:
A partially randomized, partially double-blind, placebo-controlled Phase II non-inferiority study to evaluate immunogenicity and safety of one and two doses of MVA-BN® (IMVAMUNE™) smallpox vaccine in 18-55 year old healthy subjects

Date of Amendment #1: 26-Mar-2007

PROPERTY OF BAVARIAN NORDIC A/S

CONFIDENTIAL

Site Signature page

Herewith I agree that I have read and fully understood amendment #1 to the final study protocol

POX-MVA-005

A partially randomized, partially double-blind, placebo-controlled Phase II non-inferiority study to evaluate immunogenicity and safety of one and two doses of MVA-BN® (IMVAMUNE™) smallpox vaccine in 18-55 year old healthy subjects.

I agree that I will conduct this study according to the instructions given by the study protocol and by this amendment. Furthermore, I agree that I will conduct this study according to ICH GCP, the current version of the Declaration of Helsinki, Directive 2001/20/EC and the German Drug Law.

Site:
Harrison Clinical Research GmbH
Albrechtstr. 43
80636 München

26/03/2007
Date

Prof. Frank von Sonnenburg, MD
Principal Investigator

Date

Stephan de la Motte, MD
Investigator

Site Signature page

Herewith I agree that I have read and fully understood amendment #1 to the final study protocol

POX-MVA-005

A partially randomized, partially double-blind, placebo-controlled Phase II non-inferiority study to evaluate immunogenicity and safety of one and two doses of MVA-BN® (IMVAMUNE™) smallpox vaccine in 18-55 year old healthy subjects.

I agree that I will conduct this study according to the instructions given by the study protocol and by this amendment. Furthermore, I agree that I will conduct this study according to ICH GCP, the current version of the Declaration of Helsinki, Directive 2001/20/EC and the German Drug Law.

Site:
Harrison Clinical Research GmbH
Albrechtstr. 43
80636 München

______________ Date	______________ Prof. Frank von Sonnenburg, MD Principal Investigator
26 MAR 2007 Date	[signature] Stephan de la Motte, MD Investigator

I. General information

Signature page

POX-MVA-005

A partially randomized, partially double-blind, placebo-controlled Phase II non-inferiority study to evaluate immunogenicity and safety of one and two doses of MVA-BN® (IMVAMUNE™) smallpox vaccine in 18-55 year old healthy subjects

The undersigned parties agree that the amendment was written according to international ethical and scientific quality standards (ICH-GCP), in compliance with the Declaration of Helsinki, Directive 2001/20/EC and the German Drug Law.

Date	Nadine Uebler, MSc<br>Project Coordinator<br>Bavarian Nordic
Date	Garth Virgin, MD<br>Medical Monitor<br>Bavarian Nordic
Date	Joachim Klinger<br>Director Data Management & Statistics,<br>Harrison Clinical Research
Date	Robert Semich, PhD<br>GCP-Specialist, Quality Assurance Development,<br>Bavarian Nordic
26/03/2007<br>Date	Prof. Frank von Sonnenburg, MD<br>Principal Investigator<br>Institute for Tropical Medicine<br>University of Munich

I. General information

Signature page

POX-MVA-005

A partially randomized, partially double-blind, placebo-controlled Phase II non-inferiority study to evaluate immunogenicity and safety of one and two doses of MVA-BN® (IMVAMUNE™) smallpox vaccine in 18-55 year old healthy subjects

The undersigned parties agree that the amendment was written according to international ethical and scientific quality standards (ICH-GCP), in compliance with the Declaration of Helsinki, Directive 2001/20/EC and the German Drug Law.

26 Mar-2007
Date

Nadine Uebler, MSc
Project Coordinator
Bavarian Nordic

27-Mar-07
Date

Garth Virgin, MD
Medical Monitor
Bavarian Nordic

26 Mar 07
Date

Joachim Klinger
Director Data Management & Statistics,
Harrison Clinical Research

26-Mar-2007
Date

Robert Semich, PhD
GCP-Specialist, Quality Assurance Development,
Bavarian Nordic

Date

Prof. Frank von Sonnenburg, MD
Principal Investigator
Institute for Tropical Medicine
University of Munich

II. Rationale

This amendment implements the following changes to Bavarian Nordic protocol POX-MVA-005:

Based on the sample size calculation for this trial, 175 evaluable subjects per group have to be treated to allow a statistically valid analysis. In order to account for drop-outs, it was originally planned to treat 200 subjects per group (800 subjects in total). During the course of the trial it turned out that the actual drop-out rate is substantially lower than expected. Therefore, it was decided to reduce the number of subjects to be finally included to 180 per group to get the initially planned 175 evaluable subjects per group. Since this decision was made when group 4 was already completely enrolled (N = 200), this will only affect study groups 1, 2 and 3 and will result in a total number of 740 subjects to be treated.
The primary hypothesis of the trial is to show that the humoral immune response of the group with history of smallpox vaccination (group 4) is not statistically inferior compared to the group without history of smallpox vaccination (group 1). The decision to reduce the number of subjects to be recruited has no negative impact on primary hypothesis testing, any statistical considerations or statistical analysis of this trial. Although the total number of recruited subjects has been changed, the total number of evaluated subjects for confirmatory statistical testing remains unchanged. Therefore, the reduction in total number of subjects (740 vs. 800) to be recruited does not have any impact on the scientific value or the result of the trial.

In addition, the following minor changes will be implemented:

- The Safety Monitoring Committee (SMC) has been changed to a Data Safety Monitoring Board (DSMB).

- ECGs will be assessed by the investigator and transmitted to a central database. Abnormal and unclear ECG findings will be evaluated and followed-up by a cardiologist. An algorithm for evaluating cardiac events has been added.

- The study halting and termination rules have been extended and the time constraint has been removed, as follows:

  A temporary halting or termination for single study subjects or for the study as a whole can be decided in case of the occurrence of
  - a serious adverse event (SAE)
  - an unexpected grade 3 or higher systemic reaction or grade 3 lab toxicity

  with an at least reasonable possibility of a causal relationship to the administration of MVA-BN®, i.e. the relationship cannot be ruled out.

- The background section is updated with the latest clinical trial results with MVA-BN®.

- The ranges specified for the inclusion criterion “platelets” is adjusted to the normal values of the safety laboratory used in this trial.

- Contact persons at Bavarian Nordic, Interlab and ganiMed are updated and the external cardiologist added.

- Jens Vollmar, MD is replaced by Garth Virgin, MD as Medical Monitor for this trial.

- Correction of typing errors is introduced in the Toxicity Scale for laboratory values (Appendix I).

- Corrections of formal typographical errors and clarification of wording capable of being misunderstood are introduced.

These changes are considered not to have any significant impact on the conduct or management of the trial, on the safety of the study participants or on the scientific value of the trial.

III. Changes

Changed/added terms are highlighted in **bold** letters in the text, deleted text has ~~strikethrough~~ format.

Final study Protocol, version 1.0, dated 12-Dec-2005
Page 3, *'I. General Information, Signature Page'*, previously written and changed to:

…..

Date	~~Jens Vollmar, MD~~ **Garth Virgin, MD**<br>Medical Monitor, Bavarian Nordic

…..

Date	~~Birgitte Dornonville de la Cour~~ **Robert Semich, Ph.D.**<br>~~QA-Specialist~~, **GCP-Specialist,**<br>**Quality Assurance Development**<br>Bavarian Nordic
Date	Prof. Frank von Sonnenburg, MD<br>Principal Investigator<br>**Institute for Tropical Medicine**<br>**University of Munich**

Final study Protocol, version 1.0, dated 12-Dec-2005
Pages 4 - 6 *'Responsibilities'*, previously written and changed to:

……

**STUDY SITE**:	Harrison Clinical Research GmbH<br>Clinical Unit<br>Stephan de la Motte, MD<br>~~Hilblestr. 54~~ **Albrechtstr. 43**

…..

**MEDICAL MONITOR**:	~~Jens Vollmar~~, **Garth Virgin,** MD<br>Bavarian Nordic GmbH

……

Phone:	+49 89 8565 ~~1386~~ **1319**

......

E-mail:	~~jens.vollmar@bavarian-nordic.com~~ **garth.virgin@bavarian-nordic.com**

....

**CRO/PROJECT MANAGER**:	**Marianne Sievers**
	Harrison Clinical Research GmbH
…….	
Phone:	+49-89-12 66 80-**2311**
**Mobile:**	**+49-172-83 65 056**
Fax:	+49-89-12 66 80-2444
E-mail:	**msievers**@harrison-cro.com<br>**or mariannesievers@gmx.net**
**CENTRALIZED ECG PROVIDER:**	~~Dr. Ulrich Herzfeld~~ **Martin Ptacek-Zellmeier**
	ganiMed GmbH
…..	
E-mail:	~~info@ganimed.biz~~ **m.ptacek-zellmeier@ganimed.biz**
**LABORATORY**: **(SAFETY)**	~~Dr. Herrmann Schulz~~ **Holger Schöneborn, PhD**
	INTERLAB GmbH
…..	
Phone:	+49 89 741 393-~~0~~ **37**
Fax:	+49 89 741 393-~~39~~ **40**
E-mail:	~~Dr.Schulz@interlab.de~~ **Dr.Schoeneborn@interlab.de**
LABORATORY (IMMUNOLOGY)	~~Nicole Baedeker, PhD~~ **Thomas Meyer, PhD**
	Bavarian Nordic GmbH<br>…..
Phone:	+49 89 8565 ~~1386~~ **1405**
Fax:	+49 89 8565 1333
E-mail:	~~nicole.baedeker@bavarian-nordic.com~~<br>**thomas.meyer@bavarian-nordic.com**

Added:

**CARDIOLOGIST**	**Eva Zitzmann-Roth, MD**<br>**Richard-Strauss-Str. 56**<br>**81677 Munich**<br>**Germany**
**Phone:**	**+49 89 919095**
**Fax:**	**+49 89 99979800**
**E-mail:**	**Dr.Zitzmann-Roth@t-online.de**

Final study Protocol, version 1.0, dated 12-Dec-2005
Page 11/12, *'II. Protocol Synopsis – previously written and changed to*:

……….

VACCINE DOSAGE, TIMING AND ROUTE OF ADMINISTRATION

Group 1:
~~200~~ **180** vaccinia naive subjects receive two s.c.vaccinations with 0.5 ml MVA-BN® vaccine containing 1 x 10^8 $TCID_{50}$

Group 2:
~~200~~ **180** vaccinia naive subjects receive first vaccination with 0.5 ml MVA-BN® vaccine containing 1 x 10^8 $TCID_{50}$ and second vaccination with placebo (0.5 ml Tris Buffer)

Group 3:
~~200~~ **180** vaccinia naive subjects receive two s.c. vaccinations with placebo (0.5 ml Tris Buffer)

Group 4:
200 vaccinia non-naive subjects receive one s.c. vaccination with 0.5 ml MVA-BN® vaccine containing 1 x 10^8 $TCID_{50}$

Final study Protocol, version 1.0, dated 12-Dec-2005
Page 13, *'II. Protocol Synopsis – Study design'* and page 30, *'3.1. Experimental design' previously written and changed to*:

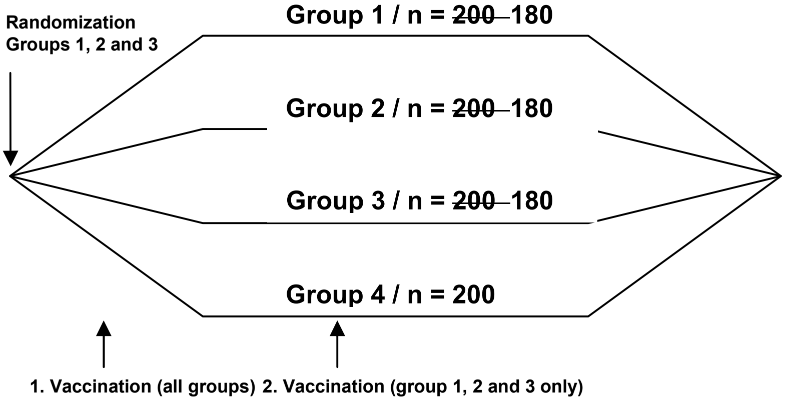

Visit Days (Groups 1, 2, 3)

-28	0	10-15	28 – 35	V3 + 10-15	V3 + 28-35	V3 + 182–210
Scr	V1	V2	V3	V4	V5	F-U

Visit Days (Group 4)

-28	0	10-15	28 - 35	V1 + 182–210
Scr	V1	V2	V3	F-U

Final study Protocol, version 1.0, dated 12-Dec-2005
Page 13, *'II. Protocol Synopsis – Planned total sample size' previously written*:

PLANNED TOTAL SAMPLE SIZE

The ~~sample size is 800~~ (200 subjects ~~per group~~). It is expected that 175 subjects per group are evaluable.

***Changed to*:**

PLANNED TOTAL SAMPLE SIZE

The **number of subjects to be enrolled is 740** (200 subjects **in group 4 and 180 each in groups 1, 2 and 3**). It is expected that 175 subjects per group are evaluable.

Final study Protocol, version 1.0, dated 12-Dec-2005
Page 14 , *'Inclusion criteria'*, and page 36, *'4.2. Inclusion criteria'*, previously written and changed to:

10. Platelets 100 – ~~440~~ **450**/nl.

.

Final study Protocol, version 1.0, dated 12-Dec-2005
Page 17, *'Statistical considerations'* and page 52, *'9.1. Sample Size Calculation'*, previously written and changed to:

Assuming a significance level of 5%, a power of 80%, expected seroconversion rates of 98% in both groups, this yields to a sample size of 175 per group (700 in total). In order to account for drop outs, a total of ~~200 subjects per group (800 in total: 200 for group 1-3, 200 for group 4)~~ **at least 180 subjects per group** will be treated.

Final study Protocol, version 1.0, dated 12-Dec-2005
Page 23, '*1.4.2. Clinical Experience with MVA-BN®* ', previously written and changed to:

To date, ~~11~~ **16** clinical studies with MVA-based vaccines have been initiated and more than ~~500~~ **1600** subjects were vaccinated. The populations vaccinated include risk groups for which conventional smallpox vaccines are contraindicated such as HIV infected patients and patients with atopic dermatitis. The majority of the vaccinated subjects ~~(401)~~ received MVA-BN® ~~and~~ whereas ~~115~~ **150** subjects were vaccinated with several other recombinant MVA-based vaccines.

Final study Protocol, version 1.0, dated 12-Dec-2005
Pages 26-28, '*1.4.2. Clinical Experience with MVA-BN®* '
'*MVA-BN®-based vaccination of populations for which Dryvax® is contraindicated'*, previously written and changed to:

Study POX-MVA-007:

……..

Primary objective of the trial ~~is~~ **was** to expand the available data on the safety of MVA-BN® in a population with atopic disorders. Vaccinia-naïve volunteers between 18 and 40 years were assigned to the following study groups ~~(15 subjects per group)~~ depending on their medical history and current health status:

- Healthy subjects **(n=15)**
- Subjects with history of atopic dermatitis **(n=16)**
- Subjects with mild active atopic dermatitis **(n=15)**
- Subjects with mild allergic rhinitis **(n=14)**

…..

A~~n interim~~ **A full** safety report of **all 60** subjects in this study is available. This ~~interim~~ safety report demonstrates a low number of grade 3 adverse events (AEs) and confirms a favorable safety profile of MVA-BN® even in a population in which conventional smallpox vaccines are contraindicated. Non-serious unsolicited and solicited AEs documented in this report were comparable to typical adverse reactions expected with other modern injectable vaccines (Table III). ~~Importantly, the results of the interim safety analysis revealed no special hazards of MVA-BN® (IMVAMUNE™) smallpox vaccine in subjects with a history of mild or active atopic dermatitis. The interim data were also submitted to CBER (IND # 11596 amendment 6) and CBER confirmed that the study in atopic dermatitis was safe to be continued. All 60 subjects have since received two vaccinations without any serious or unexpected reactions reported.~~

Table III was replaced by the following table with updated numbers:

Table III:Adverse reactions with an occurrence of > 10% in at least one subject group (29-day follow-up period after vaccination) - per subject (full analysis set, N = 60)

Symptom	Healthy (N* = 15)			History of atopic dermatitis (N* = 16)			Active atopic dermatitis (N* = 15)			Allergic rhinitis (N* = 14)		
	n	(%)	n*	n	(%)	n*	n	(%)	n*	n	(%)	n*
Injection site pain	14	(93.3)	27	14	(87.5)	25	15	(100.0)	30	14	(100.0)	27
Injection site induration	13	(86.7)	23	11	(68.8)	17	15	(100.0)	23	12	(85.7)	18
Injection site swelling	12	(80.0)	22	13	(81.3)	20	11	(73.3)	18	11	(78.6)	17
Injection site erythema	12	(80.0)	23	15	(93.8)	26	15	(100.0)	25	14	(100.0)	26
Injection site pruritus	3	(20.0)	5	4	(25.0)	5	5	(33.3)	7	1	(7.1)	1
Inj. site discolouration	2	(13.3)	3	0	(0.0)	0	1	(6.7)	1	1	(7.1)	1
Injection site warmth	0	(0.0)	0	2	(12.5)	2	1	(6.7)	1	0	(0.0)	0
Fatigue	5	(33.3)	6	10	(62.5)	13	7	(46.7)	9	9	(64.3)	13
Headache	6	(40.0)	7	7	(43.8)	12	5	(33.3)	6	6	(42.9)	6
Myalgia	0	(0.0)	0	6	(37.5)	9	2	(13.3)	4	4	(28.6)	4
Nausea	1	(6.7)	1	1	(6.3)	1	2	(13.3)	2	2	(14.3)	2
Pyrexia	1	(6.7)	1	2	(12.5)	2	3	(20.0)	3	0	(0.0)	0
Pruritus	0	(0.0)	0	0	(0.0)	0	2	(13.3)	3	0	(0.0)	0
Dermatitis atopic	0	(0.0)	0	0	(0.0)	0	2	(13.3)	2	0	(0.0)	0

% = Percentages based on N*, N = number of subjects, N* = number of subjects with documented vaccination periods, n = number of subjects with findings in specific category, n* = number of events

Study POX-MVA-010:

……..

The subjects are immunized (s.c.) with a MVA-BN® dose of $1x10^8$ $TCID_{50}$. Group 1 and 3 will receive two vaccine administrations 4 weeks apart; group 2 and 4 a single administration.
The study is being performed at 5 study centers in the US and ~~currently enrolling volunteers~~ **enrollment of volunteers has been completed.**

Solicited local and systemic adverse reactions ≥ grade 3 observed to date are shown in Table IV.

Table IV: POX-MVA-010 (un-cleaned data) -- Solicited local and systemic adverse reactions ≥ grade 3

Symptom	HIV vacc. naïve (N=30)	HIV pre-immune (N=61)	Healthy vacc. naïve (N=30)	Healthy pre-immune (N=30)
	n (%)	n (%)	n (%)	n (%)
Injection site pain	0 (0%)	2 (3.3%)	2 (6.7%)	1 (3.3%)
Injection site erythema	0 (0%)	1 (1.6%)	0 (0%)	1 (3.3%)
Injection site swelling	0 (0%)	0 (0%)	0 (0%)	0 (0%)
Injection site induration	0 (0%)	0 (0%)	0 (0%)	0 (0%)
Fever	0 (0%)	1 (1.6%)	0 (0%)	1 (3.3%)
Headache	1 (3.3%)	0 (0%)	2 (6.7%)	1 (3.3%)
Myalgia	0 (0%)	1 (1.6%)	1 (3.3%)	1 (3.3%)
Chills	1 (3.3%)	1 (1.6%)	2 (6.7%)	1 (3.3%)
Nausea	0 (0%)	1 (1.6%)	0 (0%)	2 (6.7%)
Fatigue	1 (3.3%)	3 (4.9%)	2 (6.7%)	0 (0%)

N=Number of subjects, n=Number of subjects with respective symptom, %=n/N

Study HIV-NEF-004: This phase II study is currently being conducted in Germany to evaluate immunogenicity and safety of two doses of a recombinant MVA vaccine expressing the HIV-1 nef-gene ($1x10^8$ and $5x10^8$ MVA-nef) and one dose of $1x10^8$ $TCID_{50}$ MVA-BN®. ~~By end of Oct. 2005, a total of 52 HIV-1 infected subjects with CD4 counts > 250/µl were enrolled. All subjects are stable on HAART and receive 3 vaccinations of either one of the three vaccines.~~ **A total of 77 HIV-1 infected subjects with CD4 counts > 250/µl have been enrolled and have received all 3 vaccinations.** To date, MVA-nef and MVA-BN® are well tolerated. Most frequent adverse reactions are mild injection site reactions like induration and pain.

Final study Protocol, version 1.0, dated 12-Dec-2005
Page 28, '*1.5 Dose, route of administration, vaccination schedule*', text added:

All subjects in groups 1-3 will receive two vaccinations each with a dose of 0.5 ml MVA-BN® containing $1x10^8$ $TCID_{50}$ or placebo. Injections will be administered subcutaneously according to a 0 – 4 weeks schedule (Day 0 / Day 28-35). Subjects in group 4 will receive one vaccination with a dose of 0.5 ml MVA-BN® containing $1x10^8$ $TCID_{50}$.

Final study Protocol, version 1.0, dated 12-Dec-2005
Page 28, '*1.6 Benefit/risk assessment*', previously written:

…….
Preclinical data with MVA-BN® reveal no special hazard for humans based on conventional studies of safety. Over ~~350~~ **1250** healthy and over **250** subjects with **conditions like** HIV **infection**, **m**elanoma or atopic dermatitis have been vaccinated in clinical trials with vaccines using MVA-BN® alone (to

develop an improved smallpox vaccine) or with MVA-BN® as vector (with HIV specific inserts or inserts to be used as melanoma vaccine).

Final study Protocol, version 1.0, dated 12-Dec-2005
Page 31, '*3.3.1. Screening phase*' text added:

If a subject was screened and could not be enrolled because of a certain transient condition (e.g. abnormal lab value due to an acute condition or a missing lab evaluation due to mishandling of the sample), then the subject may be re-screened and the respective tests have to be repeated as a "partial" re-screening rather than a full re-screening. The re-screening visit must be within the 28 days window started by the first screening visit and the window days -28 to -1 before 1st vaccination must not be exceeded.

If a subject could not be enrolled due to other circumstances (e.g. intermediate closure of the study group because of an interim safety analysis) or the 28 day period is exceeded, a complete re-screening assessment including physical examination, lab examination, and ECG must be performed. The clock then re-starts at the re-screening visit and defines day -28 before 1st vaccination.

Final study Protocol, version 1.0, dated 12-Dec-2005
Page 32, '*3.3.2. Allocation of subjects to treatment groups*' previously written and changed to:

Subjects eligible for Groups 1, 2 and 3 will be randomly allocated to one of these three treatment groups in order of their appearance. The randomization list will be provided by Bavarian Nordic A/S **and will be deposited in the eCRF for randomization**. At investigational site, only the study independent person preparing the study medication **and the medication monitor** will ~~have access to the randomization list~~ **be unblinded**. In case of emergency which makes unblinding necessary, **unblinding of the respective subject can be done by the investigator by means of the eCRF**. **In addition, the Safety Department** ~~the Medical Director~~ of Bavarian Nordic (contact details listed in chapter **8.6**~~7.3~~) has also access to the randomization list **as back up for unblinding**.

Final study Protocol, version 1.0, dated 12-Dec-2005
Page 33 and 34, '*3.3.3. Active study phase*' previously written and changed to:

All Groups, Visit 2

- Collection of the diary card for (1st) vaccination, review together with subject~~ and transfer to the electronic Case Report Form (CRF)~~

Groups 1, 2 and 3, Visit 4

- Collection of the diary card for 2nd vaccination, review together with subject ~~and transfer to the electronic Case Report Form (CRF)~~

Final study Protocol, version 1.0, dated 12-Dec-2005
Page 35, '*4.1. Recruitment procedure*', previously written and changed to:

…..
In total, ~~800~~ **740** individuals will be enrolled as defined in Section 4.2. After signing the Informed Consent, subjects undergo screening procedures to check eligibility ~~due to~~ **regarding** the following inclusion/exclusion criteria. In the event of a screen**ing** failure secondary to mild or limited acute illness or abnormal laboratory values the ~~patient~~ **subject** may be re-screened after resolution of the

event. Re-screening may require only an additional blood draw or a complete re-screening evaluation, depending on the circumstances of and the time interval from the initial screening failure. **Partial re-screening is to be done within the 28 days screening period, while complete re-screening is required in cases where the 28 days screening period has been exceeded**.

……

Bavarian Nordic will be informed about every ~~patient~~ **subject** entered in the study ~~using a subject inclusion form~~ **via the eCRF and weekly status reports**.

Final study Protocol, version 1.0, dated 12-Dec-2005
Page 37, *'5.1. Study halting and termination rule'*, previously written and changed to:

A temporary halting or termination for single study subjects or for the study as a whole can be decided in case of an occurrence of

- a serious adverse event (SAE)
- ~~a~~ **an unexpected** grade 3 or higher systemic reaction or lab toxicity ~~lasting >48 hours~~

with an at least reasonable possibility of a causal relationship to the administration of MVA-BN® , i.e. the relationship cannot be ruled out.

These parameters are not all-inclusive. Other adverse events could occur that would trigger a ~~Safety Monitoring Committee (SMC)~~ **Data Safety Monitoring Board (DSMB)** review. Any member of the ~~SMC~~ **DSMB**, the ~~Principal~~ **Coordinating** Investigator, BN Safety Officer, or DMID Medical Officer could request a ~~Safety Monitoring Committee~~ **DSMB** review based on any observation.

Final study Protocol, version 1.0, dated 12-Dec-2005
Page 38, *'5.3. Safety Monitoring Committee'*,:

~~Safety Monitoring Committee (SMC)~~ was replaced by **Data Safety Monitoring Board (DSMB)** throughout the text.

Final study Protocol, version 1.0, dated 12-Dec-2005
Page 40, *'6.1. Investigational product'* previously written:

MVA-BN® (IMVAMUNE™) is a highly attenuated live vaccinia virus~~. MVA-BN® will be provided in liquid-frozen aliquots of 0.6 ml. One dose of 0.5 ml liquid-frozen vaccine contains 1 x 10^8 $TCID_{50}$ Modified Vaccinia virus Ankara – Bavarian Nordic (MVA-BN®).~~ **The dose is 1 x 10^8 $TCID_{50}$ MVA-BN® per 0.5 ml. The liquid frozen MVA-BN® is provided as 0.6 ml in single dose 2 ml glass vials wherein the extractable volume is equivalent to 0.5 ml.**

For details see current version of **the** Investigator's Brochure.

Final study Protocol, version 1.0, dated 12-Dec-2005
Page 40, *'6.4. Vaccine storage, handling and dispensing'* text added:

Details on vaccine handling can be found in SOP/CLIN/016: "Storage, Handling and Preparation of Liquid Frozen MVA-BN for Vaccination" (Doc No. 10000695).

<u>Final study Protocol, version 1.0, dated 12-Dec-2005</u>
<u>Page 43, *'7.1.4. ECG assessments'*, previously written and changed to:</u>

….
ECGs will be evaluated by a centralized procedure~~. Unclear ECG abnormalities will additionally be evaluated by a central Cardiologist~~. ~~The~~ . **ECGs will be assessed by the investigator of the study site and transmitted to a central database. Abnormal and unclear ECG findings at screening or during the study conduct will additionally be evaluated and followed-up by a Cardiologist**. **Furthermore, in case of cardiac symptoms the following** workflow / communication flow will be ~~as follows~~ **applied**:

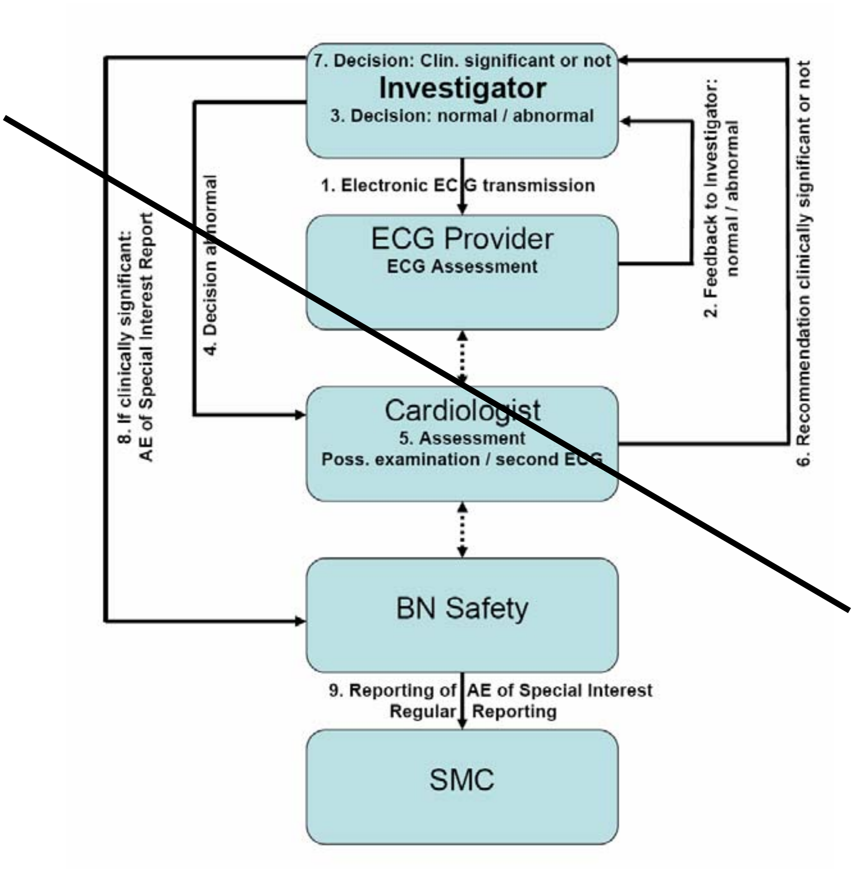

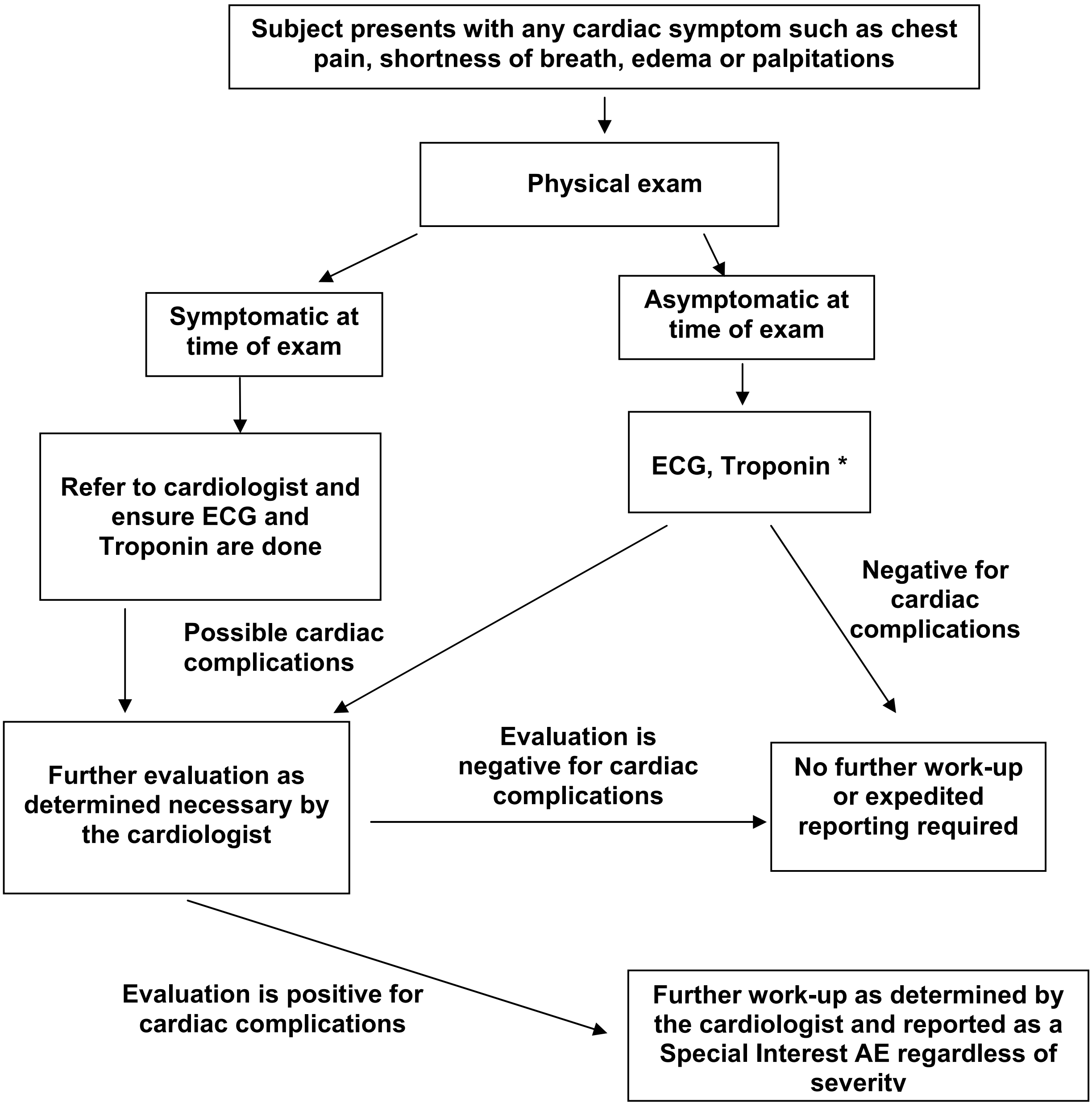

***At any protocol-scheduled ECG and/or Troponin abnormality after vaccination the algorithm will begin at this point.**

If clinically indicated**, e.g.** in case of any kind of cardiac symptoms such as but not limited to chest pain, dyspnea, arrhythmia, or edema **as well as abnormal ECG findings during the treatment phase,** subjects will be referred for a cardiac evaluation by a Cardiologist and further diagnostic tests will be done as recommended by this Cardiologist (e.g. (exercise) ECG, cardiac enzymes, echocardiogram). Subjects will ~~then~~ be followed up for at least one year **if clinically required and** in a frequency determined by the Cardiologist.

Final study Protocol, version 1.0, dated 12-Dec-2005
Page 45, *‘7.1.5. Assessment of solicited adverse events’*, text added:

To standardize procedures, uniform rulers are handed out to all subjects for measurements of erythema, swelling and induration diameters and digital thermometers for oral measurements of body temperature.

Final study Protocol, version 1.0, dated 12-Dec-2005
Page 48, *‘7.2. Assessment of Immunogenicity’*, previously written and changed to:

~~SOP/CA/06: “Standard ELISA for detection of MVA specific antibodies in human sera” (Doc. No. 10000199).~~
~~SOP/PRE/17~~ **SOP/CA/017**: “Vaccinia Virus (VV) Specific Plaque Reduction Assay for the Detection of Neutralizing Antibodies in Human Sera” (Doc. No. 10000210).

Final study Protocol, version 1.0, dated 12-Dec-2005
Page 50&51, *‘8.6. Reporting of SAEs and adverse events of special interest’*, previously written and changed to:

In compliance with **all** regulations Bavarian Nordic will take care of expedited reporting of adverse events ~~as required~~ to the regulatory authorities, **to the ethics committees** and to the responsible NIH-DMID representative.
…….

In case any problems occur ~~when trying to transmit an~~ **during transmittal of a** SAE report or with all questions specifically related to the safety of a subject enrolled in the study please contact one of the below listed responsible persons:

Garth Virgin, MD<br>Drug Safety Officer, Bavarian Nordic<br>Phone: +49-89-8565 1319<br>(working hours)<br>Phone: +49-176-22 633 107<br>(after hours)	or (Backup)	~~Jens Vollmar, MD~~ **Nathaly Arndtz, MD**<br>~~Medical Monitor~~**Vice President Medical Affairs,** Bavarian Nordic<br>Phone: +49-89-8565 1308<br>(working hours)<br>Phone: +49-172-~~86 23 292~~ **834-7130**<br>(after hours)

Final study Protocol, version 1.0, dated 12-Dec-2005
Page 54, *‘9.4. Statistical analysis’, previously written and changed to*:

As soon as the last subject has completed Visit 5 (for Group 1-3), respectively ~~Visit 4~~ **Visit 3** (for Group 4) and after any necessary settlement of queries etc. in the CRFs, data from those ~~patients~~ **subjects** and visits will be locked. A full analysis of the data available will then be performed.

Final study Protocol, version 1.0, dated 12-Dec-2005
Page 55, *'9.4.3. Analysis of safety and reactogenicity'*, previously written and changed to:

Solicited local adverse events

…..
~~The occurrence of any grade 3 or higher adverse reaction probably, possibly or definitely related to the study vaccine within 28 days after vaccination will be compared between treatment groups.~~

Solicited general adverse events

Occurrence of solicited general adverse events within 1 week after each vaccination will be summarized per ~~patient~~ **subject** and vaccination.

The maximum intensity over the 7-day period after vaccination will be used and categorized as follows:

Grade = 0 **/** > 0
Grade < 2 **/** ≥ 2
Grade < 3 / ≥ 3

…..
~~The occurrence of any grade 3 or higher adverse reaction probably, possibly or definitely related to the study vaccine within 28 days after vaccination will be compared between treatment groups.~~
…….

ECG
The ECG**s** will be evaluated by a centralized procedure. **Overall assessment will be done by the investigator and if required by a cardiologist**. The transmitted standard ECG results like PQ, QRS, QT and QTc duration and heart rate will be summarized per visit and treatment group.

Final study Protocol, version 1.0, dated 12-Dec-2005
Page 59, *'12.1. Electronic case report forms'*, previously written and changed to:

eCRFs will be used for all ~~patients~~ **subjects**. The investigator's data will be accessible from the investigator's site throughout the trial. The electronic CRFs must be kept current to reflect ~~patient~~ **subject** status at each phase during the course of the trial. The subjects are identified on the electronic CRF by ~~name and initials~~ **number, gender and date of birth**. The investigator must make a separate confidential record of these details (subject identification and enrollment log). While the trial is ongoing and until the access to the database has been terminated, there will be ~~no documentation~~ **a full audit trail** of changes. All changes to data are done ~~from~~ **by** the investigator through the EDC system. If a change is necessary once the investigator has no further access to the database, a request for change will be sent to the investigator for confirmation of the change.

Final study Protocol, version 1.0, dated 12-Dec-2005
Pages 64, '*Appendices*', previously written and changed to:

16. Appendices

Appendix I: Toxicity Scale for laboratory values

SERUM CHEMISTRY				
**Lab value**	**Mild (Grade 1)**	**Moderate (Grade 2)**	**Severe (Grade 3)**	**Potentially Life Threatening (Grade 4)**
Sodium – Hyponatremia mmol/L	< LLN - ≥ 132	< 132 - ≥ 130	< 129 - ≥ 125	<125
Sodium – Hypernatremia mmol/L	~~≥ ULN - < 146~~<br>**≥ ULN - < 150**	~~≥ 146 - < 148~~<br>**≥ 150 - < 155**	~~≥ 148 - < 150~~<br>**≥ 155 - < 160**	~~≥ 150~~<br>**≥ 160**
Potassium – Hyperkalemia mmol/L	~~≥ ULN - < 5.3~~<br>**≥ ULN - < 5.5**	~~≥ 5.3 - < 5.5~~<br>**≥ 5.5 - < 6.0**	~~≥ 5.5 - < 5.6~~<br>**≥ 6.0 - < 7.0**	~~≥ 5.6~~<br>**≥ 7.0**
Potassium – Hypokalemia mmol/L	< LLN - ≥ 3.5	< 3.5 - ≥ 3.4	< 3.4 - ≥ 3.2	< 3.2
Calcium – Hypercalcaemia **mmol/L**	≥ ULN - < 2.9	≥ 2.9 - < 3.1	≥ 3.1 - < 3.4	≥ 3.4
Calcium - Hypocalcaemia **mmol/L**	< LLN - ≥ 2.0	< 1.75 - ≥ 2.0	< 1.6 - ≥ 1.75	< 1.5
Blood Urea Nitrogen BUN mg/dL	≥ ULN - < 27	≥ 27 - < 32	≥ 32	
Serum creatinine - mg/dL	≥ ULN - < 1.5 x ULN	≥ 1.5 - < 3 x ULN	≥ 3 - 6 x ULN	> 6 x ULN
Albumin - Hypoalbuminemia g/dL	< LLN - ≥ 2.7	< 2.7 - ≥ 2.5	< 2.5	
Total Protein - Hypoproteinemia g/dL	< LLN - ≥ 5.4	< 5.4 - ≥ 5.0	< 5.0	
Alkaline Phosphatase - increase by factor	> 1.25 - < 2.0 x ULN	≥ 2.0 - < 3.0 x ULN	≥ 3.0 x ULN	
Liver Function Tests - increase by factor	> 1.0 - < 2.5 x ULN	≥ 2.5 - < 4 x ULN	≥ 4 x ULN	
Total Bilirubin - increase by factor	> ULN – 1.5 x ULN	> 1.5 - 3.0 x ULN	> 3.0 - 10.0 x ULN	> 10.0 x ULN
Creatinine Kinase (CK)	>ULN - <2x ULN	≥ 2 - <5x ULN	≥ 5x ULN	
Creatine Kinase Myocardial Band (CK-MB)	>ULN – <2.0 x ULN	≥ 2.0 - < 5.0 x ULN	≥ 5.0 x ULN	
Cardiac Troponin I	>ULN – <2.0 x ULN	≥ 2.0 - < 5.0 x ULN	≥ 5.0 x ULN	
Total Cholesterol **mg/dL**	> ULN - 300	> 300 - 400	> 400	

<u>Final study Protocol, version 1.0, dated 12-Dec-2005</u>
<u>Page 65, '*Appendix I: Toxicity Scale for laboratory values*', row added:</u>

URINE				
Protein	Trace - ≤ 1+	> 1+ - ≤ 2+	> 2+ - ≤ 3+	≥ 3+ - nephritic syndrome
Glucose	Trace	1+	2+	>2+
Blood (microscopic) – red blood cells per high power field (rbc/hpf)	≥ 0- <10	≥ 10 - < 50	≥ 50	Gross
**Blood (measured by Combur urine sticks)**	**Trace - ≤ 1+**	**> 1+ - ≤ 2+**	**> 2+ - ≤ 3+**	**≥ 3+**

Harrison
Clinical
Research

Harrison

NOTE TO FILE No.: 01

(please refer to Log sheet for note to files)

**SPONSOR CODE**	POX-MVA-005	**INVESTIGATOR**	N/A ☒
**HCR STUDY CODE**	1029 / BN	**CENTER**	N/A ☒
**DATE**	4 April 2006	**MONITOR**	N/A ☒

☒ **STUDY SPECIFIC**	☐ **CENTER SPECIFIC**	☐ **SUBJECT SPECIFIC**<br>SUBJECT NO.:
**REASON(S):**<br>☐ Issues that are not explicitly defined in the protocol, however need further clarification	☐ Systematically missed assessments<br>☐ Assessment systematically conducted at incorrect time point<br>☐ A signature on a relevant study document could not be obtained<br>☐ GCP/SOP violation (other than above)	☐ Informed consent form incorrectly filled in<br>☐ Waivers for selection criteria<br>☐ Relevant information not covered by the CRF<br>☐ GCP/SOP violation (other than above)
☒ Other: Expired FDA 1572 Form	☐ Other:	☐ Other:

DESCRIPTION:

This NTF concerns the use of the FDA 1572 form for the above mentioned study. This form expires January 31, 2006, but has been given a 30 days extension period, affirmed on the FDA webpage by Mia Prather.

For the 30 day extension period no updated forms are required . Once a three year approval has been given it will be listed on the Federal Register and thereafter updated accordingly.

18 Apr. 06 [signature]

Date / Signature

☐ CRA
☒ Project Manager
☐ Rapporteur

NOTE TO FILE No.: 2

(please refer to Log sheet for note to files)

**SPONSOR CODE**	POX-MVA-005	**INVESTIGATOR**	DR. DE LA MOTTE
**HCR STUDY CODE**	1029 / BN	**CENTER**	01 CLINICAL UNIT
**DATE**	08 JUN 2006	**MONITOR**	DR. ANDREA AUGUSTIN

Harrison

☒ **STUDY SPECIFIC**	☐ **CENTER SPECIFIC**	☐ **SUBJECT SPECIFIC**<br>SUBJECT NO.:
**REASON(S):**<br>☒ Issues that are not explicitly defined in the protocol, however need further clarification	☐ Systematically missed assessments<br>☐ Assessment systematically conducted at incorrect time point<br>☐ A signature on a relevant study document could not be obtained<br>☐ GCP/SOP violation (other than above)	☐ Informed consent form incorrectly filled in<br>☐ Waivers for selection criteria<br>☐ Relevant information not covered by the CRF<br>☐ GCP/SOP violation (other than above)
☐ Other:	☐ Other:	☐ Other:

DESCRIPTION:

The upper limit of platelet normal value range on the Interlab list is 450 G/l. In the inclusion criteria of the study protocol the upper limit is defined as 440/nl. Thus a platelet value between 440 and 450 represents a protocol violation, but on the lab print-out no tick box would appear indicating a value out of range.

At the project meeting on 31 MAY 06 it was agreed to change the upper limit in the inclusion criteria. A protocol amendment dealing with this item is in preparation. Until it is in place, the upper limit of 450 G/l according to the Interlab normal value ranges will be effective.

08.06.06 [signature]

Date
08 JUN 06

Signature

☒ CRA Dr. Andrea Augustin
☐ Project Manager
☐ Rapporteur

Harrison Clinical Research

NOTE TO FILE No.: 03 (please refer to Log sheet for note to files)

**SPONSOR CODE**	POX-MVA-005	**INVESTIGATOR**	DR. DE LA MOTTE
**HCR STUDY CODE**	1029 / BN	**CENTER**	01 CLINICAL UNIT
**DATE**	12 JUN 2006	**MONITOR**	DR. ANDREA AUGUSTIN

Harrison

☒ **STUDY SPECIFIC**	☐ **CENTER SPECIFIC**	☐ **SUBJECT SPECIFIC**<br>SUBJECT NO.:
**REASON(S):**<br>☒ Issues that are not explicitly defined in the protocol, however need further clarification	☐ Systematically missed assessments<br>☐ Assessment systematically conducted at incorrect time point<br>☐ A signature on a relevant study document could not be obtained<br>☐ GCP/SOP violation (other than above)	☐ Informed consent form incorrectly filled in<br>☐ Waivers for selection criteria<br>☐ Relevant information not covered by the CRF<br>☐ GCP/SOP violation (other than above)
☐ Other:	☐ Other:	☐ Other:

DESCRIPTION:

Cholesterol and HDL values for the risk score (National Cholesterol Education Program's risk assessment) have to be calculated as the units on the lab print-out do not correspond to the units required in the score. Up to now the value on the print-out which is mmol/L has been divided through the factor 0,02586 to obtain mg/dl.

Starting from 31 May these values will be calculated by multiplying with factor 38,61 as instructed by Interlab (eMail Dr. Schöneborn 31 MAY 06). This item has been discussed in the project meeting on 31 MAY 06.

12 JUN 06
Date

Signature

☒ CRA Dr. Andrea Augustin
☐ Project Manager
☐ Rapporteur

BAVARIAN NORDIC

NOTE TO FILE #4

**Study number:** POX-MVA-005	

Study Title:
A partially randomized, partially double-blind, placebo-controlled Phase II non-inferiority study to evaluate immunogenicity and safety of one and two doses of MVA-BN® (IMVAMUNE™) smallpox vaccine in 18-55 year old healthy subjects

Issue:
Principal Investigator, Co-Principal Investigator

Description:
This Note to File is to define and clarify the role and responsibility of Prof. Frank von Sonnenburg and Dr. Stephan de la Motte in this trial.
The clinical trial is conducted at one site. This is the Clinical Unit of the CRO Harrison Clinical Research GmbH.
Prof. Frank von Sonnenburg acts as "Leiter der klinischen Prüfung" according to §40 AMG (Arzneimittelgesetz) and as Principal Investigator of this trial, at this site. He was chosen due to his gathered experience with the investigational product MVA-BN® (IMVAMUNE™) in other trials. He mainly has a consulting function in this trial.
Dr. Stephan de La Motte is Chief Medical Officer of the Clinical Unit of Harrison Clinical Research and has therefore the responsibility for the Clinical Unit and the practical conduction of this trial. He acts as Co-Principal Investigator in this trial with the same rights and responsibilities as Prof. von Sonnenburg.

Date: 29-Jun-2006

Name: Nadine Uebler **Signature:** Nadine Uebler

29/06/2006

NOTE TO FILE No.: 5 (please refer to Log sheet for note to files)

**SPONSOR CODE**	POX-MVA-005	**INVESTIGATOR**	DR. DE LA MOTTE
**HCR STUDY CODE**	1029 / BN	**CENTER**	01 CLINICAL UNIT
**DATE**	26 JUL 06	**MONITOR**	N/A

Harrison

☒ **STUDY SPECIFIC**	☐ **CENTER SPECIFIC**	☐ **SUBJECT SPECIFIC**<br>SUBJECT NO.:
**REASON(S):**<br>☒ Issues that are not explicitly defined in the protocol, however need further clarification	☐ Systematically missed assessments<br>☐ Assessment systematically conducted at incorrect time point<br>☐ A signature on a relevant study document could not be obtained<br>☐ GCP/SOP violation (other than above)	☐ Informed consent form incorrectly filled in<br>☐ Waivers for selection criteria<br>☐ Relevant information not covered by the CRF<br>☐ GCP/SOP violation (other than above)
☐ Other:	☐ Other:	☐ Other:

DESCRIPTION:

Confirmation of Exclusion of Vulnerable Subjects

In accordance with GCP and in mutual agreement with the sponsor and the Clinical Trial Director acc. to German Drug Law ("Leiter der klinischen Prüfung gem. AMG") it was confirmed that the following two people will not be included as study subjects, if the investigator should become aware of their relationship:

- Employees of Bavarian Nordic (BN) or Harrison Clinical Research (HCR).

- First degree relatives (children, parents, wife, husband or spouse) of any employee of Bavarian Nordic or of Harrison Clinical Research, if the employee is directly involved in this clinical trial.

For clarification:

Friends or relatives of those employees who are not involved in this trial can participate as study subjects.

During screening, the subjects are being asked about their profession or job, but are not being asked routinely about their employer, although it may be possible that the employer becomes obvious (e.g., for policemen, subway driver, etc.).

It is obvious that the investigator does not ask the study subjects about employers of their friends or relatives and that no such information is recorded.

26 JUL 2006 [signature]

Date SIGNATURE ☒ INVESTIGATOR

NOTE TO FILE No.: 6 (please refer to Log sheet for note to files)

**SPONSOR CODE**	POX-MVA-005	**INVESTIGATOR**	DR. DE LA MOTTE
**HCR STUDY CODE**	1029 / BN	**CENTER**	01 CLINICAL UNIT
**DATE**	26 JUL 06	**MONITOR**	N/A

☒ **STUDY SPECIFIC**	☐ **CENTER SPECIFIC**	☐ **SUBJECT SPECIFIC**<br>SUBJECT NO.:
**REASON(S):**<br>☒ Issues that are not explicitly defined in the protocol, however need further clarification	☐ Systematically missed assessments<br>☐ Assessment systematically conducted at incorrect time point<br>☐ A signature on a relevant study document could not be obtained<br>☐ GCP/SOP violation (other than above)	☐ Informed consent form incorrectly filled in<br>☐ Waivers for selection criteria<br>☐ Relevant information not covered by the CRF<br>☐ GCP/SOP violation (other than above)
☐ Other:	☐ Other:	☐ Other:

DESCRIPTION:

Vaccinia Scar assessment

It was agreed upon with the Bavarian Nordic (BN) and the Clinical trial Director according to German Drug Law (LKP), that only the following subjects are allowed to be enrolled in Group IV

- subjects born in Germany (east and west) before 1976 showing a POX scar
- younger subjects, born after 1975, showing a POX scar without having any further documentation, can only be enrolled after re-confirmation with LKP and BN
- Younger subjects born after 1975, showing a POX Scar confirmed by written documentation (Impfpass)

In cases where the scare is not 100% detectable, the written documentation is mandatory for enrolment in the study.

In cases where the investigator is not sure whether the scare is from a POX or BCG vaccination, a photo should be taken and the case will be discussed in the next weekly meeting.

09-Aug-06 [signature] (PI)
Date Signature ☒ Bavarian Nordic

04 Aug 06 [signature]
Date Signature ☒ Project Manager

Harrison Clinical Research

NOTE TO FILE No.: 7 (please refer to Log sheet for note to files)

**SPONSOR CODE**	POX-MVA-005	**INVESTIGATOR**	DR. DE LA MOTTE
**HCR STUDY CODE**	1029 / BN	**CENTER**	01 CLINICAL UNIT
**DATE**	03 AUG 06	**MONITOR**	M. SIEVERS

Harrison

☐ **STUDY SPECIFIC**	☐ **CENTER SPECIFIC**	☒ **SUBJECT SPECIFIC**<br>SUBJECT NO.: **115**
**REASON(S):**<br>☐ Issues that are not explicitly defined in the protocol, however need further clarification	☐ Systematically missed assessments<br>☐ Assessment systematically conducted at incorrect time point<br>☐ A signature on a relevant study document could not be obtained<br>☐ GCP/SOP violation (other than above)	☐ Informed consent form incorrectly filled in<br>☐ Waivers for selection criteria<br>☐ Relevant information not covered by the CRF<br>☐ GCP/SOP violation (other than above)
☐ Other:	☐ Other:	☒ Other: unclear vaccination status

DESCRIPTION:

Subject no. 115, Scr. No. 254, DOB: 22.08.79

Investigator is not sure whether the vaccination scar is from a POX or from a BCG vaccination. Scar more likely from BCG. Dr. Ilchmann was informed by phone that vaccination card is lost in flood, mother of subject thinks it was no smallpox vaccination.

Subject is included in group 4.

Date of screening visit: 13.06.06
Date of Visit 1: 26.06.06
Date of Visit 2: 05.07.06
Date of Visit 3: 24.07.06

Date	Signature	
03.08.2006	[signature]	☒ CRA Marianne Sievers<br>☐ Project Manager<br>☐ Rapporteur

NOTE TO FILE No.: 8 (please refer to Log sheet for note to files)

**SPONSOR CODE**	POX-MVA-005	**INVESTIGATOR**	DR. DE LA MOTTE
**HCR STUDY CODE**	1029 / BN	**CENTER**	01 CLINICAL UNIT
**DATE**	04 AUG 06	**MONITOR**	N/A

☒ **STUDY SPECIFIC**	☐ **CENTER SPECIFIC**	☐ **SUBJECT SPECIFIC**<br>SUBJECT NO.:
**REASON(S):**<br>☐ Issues that are not explicitly defined in the protocol, however need further clarification	☐ Systematically missed assessments<br>☐ Assessment systematically conducted at incorrect time point<br>☐ A signature on a relevant study document could not be obtained<br>☐ GCP/SOP violation (other than above)	☐ Informed consent form incorrectly filled in<br>☐ Waivers for selection criteria<br>☐ Relevant information not covered by the CRF<br>☐ GCP/SOP violation (other than above)
☒ Other: documentation subject identification and enrolment log	☐ Other:	☐ Other:

DESCRIPTION:

Documentation on the subject identification and enrolment log

By mistake at the beginning of the trial all subjects screened, whether they are screening failures or not, regardless they have consented to participate in the trial or not, have been entered on both the subject screening log and the subject identification and enrolment log. As this is neither in accordance with the HCR SOP nor with ICH- GCP, the enrolment log has to be corrected in the following way.

- For all subjects that have not signed an Informed Consent Form, the names have to be blackened by either a study nurse or an investigator, dated and initialled.
- For all subjects that have signed an Informed Consent , the names have to be cancelled with a single line dated and initialled by either a study nurse or an investigator
- In addition blackened names have to be marked with "NC=NOT CONSENTED" as explanation for the correction and the cancelled names have to be marked with "SF=SCREENING FAILURE"

4 Aug 06

Date Signature ☐ CRA
☒ Project Manager
☐ Rapporteur

NOTE TO FILE No.: 9 (please refer to Log sheet for note to files)

**SPONSOR CODE**	POX-MVA-005	**INVESTIGATOR**	DR. DE LA MOTTE
**HCR STUDY CODE**	1029 / BN	**CENTER**	01 CLINICAL UNIT
**DATE**	28 SEP 06	**MONITOR**	M. SIEVERS

☐ **STUDY SPECIFIC**	☐ **CENTER SPECIFIC**	☒ **SUBJECT SPECIFIC** SUBJECT NO.: **043, 050, 061, 089, 115**
**REASON(S):** ☐ Issues that are not explicitly defined in the protocol, however need further clarification	☐ Systematically missed assessments<br>☐ Assessment systematically conducted at incorrect time point<br>☐ A signature on a relevant study document could not be obtained<br>☐ GCP/SOP violation (other than above)	☐ Informed consent form incorrectly filled in<br>☐ Waivers for selection criteria<br>☐ Relevant information not covered by the CRF<br>☐ GCP/SOP violation (other than above)
☐ Other:	☐ Other:	☒ Other: Inclusion in group IV by mistake

DESCRIPTION:

Subject nos: 043, 050, 061, 089, 115

The vaccination scars of above mentioned subjects are from BCG vaccinations. These subjects don't have any smallpox vaccination history but they were randomised in group IV by mistake.

BCG vaccinations are confirmed in vaccination cards of subjects but no smallpox vaccination is documented. All subjects didn't show their vaccination cards on Screening, V1, V2 and V3. Vaccination cards were shown later on.

28.9.06   G. Sievers

Date 28.09.2006   Signature

☒ CRA Marianne Sievers
☐ Project Manager
☐ Rapporteur

NOTE TO FILE No.: 10 (please refer to Log sheet for note to files)

SPONSOR CODE	POX-MVA-005	INVESTIGATOR	DR. DE LA MOTTE
HCR STUDY CODE	1029 / BN	CENTER	01 CLINICAL UNIT
DATE	06 DEC 06	MONITOR	M. SIEVERS

Harrison

☐ STUDY SPECIFIC	☐ CENTER SPECIFIC	☒ SUBJECT SPECIFIC<br>SUBJECT NO.: **613**
**REASON(S):**<br>☐ Issues that are not explicitly defined in the protocol, however need further clarification	☐ Systematically missed assessments<br>☐ Assessment systematically conducted at incorrect time point<br>☐ A signature on a relevant study document could not be obtained<br>☐ GCP/SOP violation (other than above)	☒ Informed consent form incorrectly filled in<br>☐ Waivers for selection criteria<br>☐ Relevant information not covered by the CRF<br>☐ GCP/SOP violation (other than above)
☐ Other:	☐ Other:	☐ Other:

DESCRIPTION:

Subject no 613:

Informed consent (IC) page 8 and 9 signed without subjects date by mistake. Page 11 of IC (data protection) is signed and dated by subject on 29.09.06 (= date of Screening Visit).

V 1 was on 09.10.06

Subject was not reachable some days and didn't appear to the planned visit 2. Withdrawal of consent on 08.11.06 by phone, Subject was informed by Investigator that a study termination visit is recommended. But subject refused to attend once more and reacts in an unfriendly way.

Date	Signature	
06.12.2006	[signature]	☒ CRA Marianne Sievers<br>☐ Project Manager<br>☐ Rapporteur

NOTE TO FILE No.: 11 (please refer to Log sheet for note to files)

**SPONSOR CODE**	POX-MVA-005	**INVESTIGATOR**	DR. DE LA MOTTE
**HCR STUDY CODE**	1029 / BN	**CENTER**	01 CLINICAL UNIT
**DATE**	08 JAN 07	**MONITOR**	E. KARDALINOU

Harrison

☐ **STUDY SPECIFIC**	☐ **CENTER SPECIFIC**	☒ **SUBJECT SPECIFIC**<br>SUBJECT NO.: **594**
**REASON(S):**<br>☐ Issues that are not explicitly defined in the protocol, however need further clarification	☐ Systematically missed assessments<br>☐ Assessment systematically conducted at incorrect time point<br>☐ A signature on a relevant study document could not be obtained<br>☐ GCP/SOP violation (other than above)	☐ Informed consent form incorrectly filled in<br>☐ Waivers for selection criteria<br>☐ Relevant information not covered by the CRF<br>☐ GCP/SOP violation (other than above)
☐ Other:	☐ Other:	☒ Other: Inclusion in group I-III by mistake

DESCRIPTION:

Subject: 594 (female; DOB:

Subject 594 was assigned in this trial to group 1-3, as neither scars were detected by the physical examination nor small-pox vaccination records seemed to be in her possession.
The subject found apparently and showed her immunisation licence for the first time after the 2. vaccination, at V4. According to the licence she had been vaccinated against smallpox on 14.07.81 and 21.07.81.

The subject will not be included in the statistical analysis of the trial, but for safety reasons she will proceed with the following visits (V5 and follow-up visit), including an additional ECG-assessment and safety lab examination at V5.

12.01.07 E. Kardalinou

Date
08.01.2007

Signature

☒ CRA Efterpi Kardalinou
☐ Project Manager
☐ Rapporteur

Harrison Clinical Research

Harrison

NOTE TO FILE No.: 12 (please refer to Log sheet for note to files)

**SPONSOR CODE**	POX-MVA-005	**INVESTIGATOR**	DR. DE LA MOTTE
**HCR STUDY CODE**	1029 / BN	**CENTER**	01 CLINICAL UNIT
**DATE**	17 JAN 07	**MONITOR**	M. SIEVERS

☐ **STUDY SPECIFIC**	☐ **CENTER SPECIFIC**	☒ **SUBJECT SPECIFIC**<br>SUBJECT NO.: **050**
**REASON(S):**<br>☐ Issues that are not explicitly defined in the protocol, however need further clarification	☐ Systematically missed assessments<br>☐ Assessment systematically conducted at incorrect time point<br>☐ A signature on a relevant study document could not be obtained<br>☐ GCP/SOP violation (other than above)	☒ Informed consent form incorrectly filled in<br>☐ Waivers for selection criteria<br>☐ Relevant information not covered by the CRF<br>☐ GCP/SOP violation (other than above)
☐ Other:	☐ Other:	☐ Other: Inclusion in group I-III by mistake

DESCRIPTION:

Subject: 050 :

Informed consent version 5.0: Signature and date on **page 11** (data protection) is missing.

By mistake on V1 (02.06.06), V2 (14.06.06) and on V3 (30.06.06) it was forgotten to catch subjects dates and signatures on Informed Consent, version 5.0. Therefore the IC, version 5.0, was sent to the subject for dates and signatures by mail twice. The first time was in July 2006. Unfortunately the subject lost this IC and so we sent the updated version 5.0 again in September 2006 by mail. In October 2006 we received the partly signed and dated IC back. The subject forgot to date and sign the section "Data protection" (page 11).

By mistake again it was forgotten by the investigators to catch the missing signature on page 11 on follow-up visit despite some tips (post its) in the source data/ worksheet.

Subjects date (15.05.06) and signature on Informed consent **version 4.0**, page 11 is available and filed in the source data. There were no changes regarding "data protection" in version 5.0.

17.1.07 [signature]

Date 17.01.2007 Signature

☒ CRA Marianne Sievers
☐ Project Manager
☐ Rapporteur

22 JAN 2007 [signature] (Dr. de la Motte)

NOTE TO FILE No.: 13 (please refer to Log sheet for note to files)

**SPONSOR CODE**	POX-MVA-005	**INVESTIGATOR**	DR. DE LA MOTTE
**HCR STUDY CODE**	1029 / BN	**CENTER**	01 CLINICAL UNIT
**DATE**	17 JAN 2007	**MONITOR**	DR. A. AUGUSTIN

Harrison

☐ **STUDY SPECIFIC**	☐ **CENTER SPECIFIC**	☒ **SUBJECT SPECIFIC**<br>SUBJECT NO.: **580**
**REASON(S):**<br>☐ Issues that are not explicitly defined in the protocol, however need further clarification	☐ Systematically missed assessments<br>☐ Assessment systematically conducted at incorrect time point<br>☐ A signature on a relevant study document could not be obtained<br>☐ GCP/SOP violation (other than above)	☐ Informed consent form incorrectly filled in<br>☐ Waivers for selection criteria<br>☐ Relevant information not covered by the CRF<br>☐ GCP/SOP violation (other than above)
☐ Other:	☐ Other:	☒ Other: suspected SIAE

DESCRIPTION:

Subject 580 showed an irregular ECG (VES/extrasystoles) at visit 2 on 16.10.06. She was referred to the cardiologist who detected a mild pericardial effusion in an echo performed on 19.10.06 which was very likely pre-exisiting. The HCR investigators reported this event as an SIAE to Bavarian Nordic to follow the reporting timelines. Retrospectively both events, the VES and pericardial effusion, were judged as not clinically significant by the cardiologist, Dr. Zitzmann-Roth and the PI, Prof. von Sonnenburg. Thus, the events do not fulfill the criteria of an SIAE and no further reporting, e.g. to the DSMB or corrections in the SIAE report are necessary.

However, the subject will not obtain a second study vaccination.

Attachments:
- Initial SIAE Report
- Assessment Dr. Zitzmann-Roth
- Correspondence Dr. Virgin/Prof. von Sonnenburg

Date	Signature	
17 JAN 2007	[signature]	☒ CRA<br>☐ Project Manager<br>☐ Rapporteur

BAVARIAN NORDIC

Drug.Safety@Bavarian-Nordic.com

☐ SERIOUS ADVERSE EVENT ☒ AE OF SPECIAL INTEREST

Study	Country	Centre-No.	Investigator	Report type
POX-MVA-005	Germany	Harrison Clinical Research, Munich	ILCHMANN	☒ initial ☐ follow-up

Subject No. Or Initials	Date of Birth	Age	Gender	Onset of Event	End of Event
05810	04 SEP 1985 (day month year)	21 years	☐ Male ☒ Female	31 OCT 2006 (day month year)	__ __ __ (day month year)

ADVERSE EVENT INFORMATION

Adverse event (Diagnosis): mild pericardial effusion

Description (e.g. symptoms / signs / time course / relevant test / laboratory data), continue on a separate page if necessary and attach copies of relevant documents (e.g. autopsy report, medical reports)
maximal 3mm, no clinical symptoms, diagnosed by ultrasound

Tick all appropriate to adverse event

☐ Requires hospitalisation
Admission date: __ __ __ (day month year)
Discharge date: __ __ __ (day month year)

☐ Prolongs hospitalisation
Discharge date: __ __ __ (day month year)

☐ Persistent or significant disability / incapacity

☐ Life-threatening

☐ Congenital anomaly / birth defect

☐ Serious as assessed by the investigator

☐ Development of cancer

☐ Overdose, intentional or inadvertent

☐ Results in death:
__ __ __ (day month year)

Treatment of the event:
NONE

Outcome

☐ Complete recovery without sequelae
☐ Recovery with sequelae
☐ Ongoing / persistent condition
☐ Death
☐ Death likely due to the reaction
☐ Death not related to the reaction

STUDY VACCINE INFORMATION / RELATIONSHIP

Study medication:	Dose:	Route of administration:	Vaccination dates
MVA-BN / Placebo			

Code broken?	Did event abate after stopping drug?	Did event recur after reintroduction?	Vaccination dates
☐ Yes<br>☒ No<br>☐ Not applicable	☐ Yes<br>☐ No<br>☒ Not applicable	☐ Yes<br>☐ No<br>☒ Not applicable	1st vaccination: 05 OCT 2006 (day month year)<br>2nd vaccination: __ __ __ (day month year)

Relationship to vaccine	Relationship to study procedure	Relationship to other condition/treatment	If related to study procedure or other condition/treatment please specify:
☐ Unrelated<br>☒ Unlikely<br>☐ Possible<br>☐ Probable<br>☐ Definite	☐ Unrelated<br>☒ Unlikely<br>☐ Possible<br>☐ Probable<br>☐ Definite	☐ Unrelated<br>☐ Unlikely<br>☒ Possible<br>☐ Probable<br>☐ Definite	accidental finding, perhaps normal variant

Version: 21-April-2006

BAVARIAN NORDIC

Drug.Safety@Bavarian-Nordic.com

☐ SERIOUS ADVERSE EVENT ☒ AE OF SPECIAL INTEREST

Subject no. or Subject initials 0580 (continued) Study POX-MVA-005

Relevant medical history (e.g. previous diagnosis, surgery, allergies, pregnancy with date of last period (day / month /year)

Nephritis at 2000 (not currently active)

Concomitant drug(s) (exclude those to treat the adverse event; use a second form in case of more than 5 drugs)

Drug	Daily dose	Route of administration	Indication	Therapy dates from (day month year)	Therapy dates to (day month year)	Causal relationship to event
LEIOS	1 tbl.	oral	contraception		unk JUN 2001	☒ none ☐ possible
						☐ none ☐ possible
						☐ none ☐ possible
						☐ none ☐ possible
						☐ none ☐ possible

Comments

Subject is doing sports regularly.

	Date of this report	Investigator's name and address	Investigator's signature
	31 OCT 2006 (day month year)	Harrison Clinical Research Gesellschaft für Klinische Forschung mbH Administration & Operations Albrechtstraße 14 D-80636 München Germany Tel. 49-(0)89-126680-0 Fax 49-(0)89-126680-2444	[signature]

Do NOT fill in shaded area !

Date received by Bavarian Nordic representative: day month year

BN representative's name:

and signature:

Name and address of company organising the clinical trial:

Bavarian Nordic GmbH
Clinical Department
Fraunhoferstrasse 13
82152 Martinsried
GERMANY

FAX: +49-89-8565 1419

Serious	☐ NO ☐ YES	Comments
Unexpected	☐ NO ☐ YES	
Associated with drug	☐ NO ☐ YES	International documentation number:

Name of Med. Monitor / Drug Safety Officer: Signature Date day month year

Final Version 4.0, 17 May 2006

ECG Assessment Form

POX-MVA-005

Screening No. 0979	Subject No. 580	Date of Birth 04.09.1985
ECG recording date & time 16.10.06 11:01:22	Visit V2/2	ECG ID

CARDIOLOGIST

Assessment: ☐ **Normal** ☐ Typical ☐ Variation ☒ **Abnormal**

If abnormal or normal variation please specify:

- ☐☐ Supraventricular arrhythmia
- ☐☐ Ventricular arrhythmia
- ☐☐ AV block
  - ☐ First degree
  - ☐ Second degree Type Mobitz I (Wenckebach)
  - ☐ Second degree Type Mobitz II
  - ☐ Third degree
- ☐☐ Left bundle branch block
  - ☐ Incomplete
  - ☐ Complete
- ☐☐ Right bundle branch block
  - ☐ Incomplete
  - ☐ Complete
- ☐☐ ST elevation
  - V_1 ☐ V_2 ☐ V_3 ☐ V_4 ☐ V_5 ☐ V_6 ☐
  - I ☐ II ☐ III ☐ aVR ☐ aVL ☐ aVF ☐
- ☐☐ ST depression
  - V_1 ☐ V_2 ☐ V_3 ☐ V_4 ☐ V_5 ☐ V_6 ☐
  - I ☐ II ☐ III ☐ aVR ☐ aVL ☐ aVF ☐
- ☐☐ T inversion – pathological
  - V_1 ☐ V_2 ☐ V_3 ☐ V_4 ☐ V_5 ☐ V_6 ☐
  - I ☐ II ☐ III ☐ (aVR ☐) aVL ☐ aVF ☐
- ☐☐ QTc prolongation
- ☐☐ Low voltage
- ☐☐ Other: SCR-ECG!!

Detailed description:

wegen Pö [illegible] [illegible] [illegible] [illegible]
Pericarditis nicht
ausgeprägt; VES nicht
als Impffolge und
bekannt bei der Patient

In case of abnormal ECG – further examinations:

- ☐☐ Echocardiogram
- ☐☐ Holter ECG
- ☐☐ Treadmill ECG
- ☐☐ Other: Echo

Please attach copy of result printout!

Is the ECG change clinically significant?

☐☐ Yes ☐ No → *If No, please specify below, why ECG is abnormal, but not clinically significant!*

Assessment and recommendation:

Echo: geringer Perikarderguss max 3 mm
[illegible] o.B. in Ruhe [illegible]

Does the ECG change meet the case definition for Myocarditis/Pericarditis?

☐ No
☐ Yes → ☐ Possible ☐ Probable ☐ Confirmed

Signature Cardiologist: [signature] 13.10.06 [signature] 31.10.06

Final Version 4.0, 17 May 2006

ECG Assessment Form

POX-MVA-005

Screening No. 0979	Subject No. 580	Date of Birth 04/09/1985
ECG recording date & time 20/09/06 08:47:03	Visit SCR	ECG ID

CARDIOLOGIST

Assessment: ☒ **Normal** ☐ **Abnormal**

☐ Typical ☒ Variation

***If abnormal or normal variation* please specify:**	Detailed description:
☐☐ Supraventricular arrhythmia	
☐☐ Ventricular arrhythmia	
☐☐ AV block: ☐ First degree; ☐ Second degree Type Mobitz I (Wenckebach); ☐ Second degree Type Mobitz II; ☐ Third degree	
☐☐ Left bundle branch block: ☐ Incomplete; ☐ Complete	
☐☐ Right bundle branch block: ☐ Incomplete; ☐ Complete	
☐☐ ST elevation: V_1 ☐ V_2 ☐ V_3 ☐ V_4 ☐ V_5 ☐ V_6 ☐; I ☐ II ☐ III ☐ aVR ☐ aVL ☐ aVF ☐	
☐☐ ST depression: V_1 ☐ V_2 ☐ V_3 ☐ V_4 ☐ V_5 ☐ V_6 ☐; I ☐ II ☐ III ☐ aVR ☐ aVL ☐ aVF ☐	
☐☐ T inversion – pathological: V_1 ☐ V_2 ☐ V_3 ☐ V_4 ☐ V_5 ☐ V_6 ☐; I ☐ II ☐ III ☐ (aVR ☐) aVL ☐ aVF ☐	
☐☐ QTc prolongation	
☐☐ Low voltage	
☐☐ Other: Sinusbradykardie	

In case of abnormal ECG – further examinations:

☐☐ Echocardiogram	**Please attach copy of result printout!**
☐☐ Holter ECG	
☐☐ Treadmill ECG	
☐☐ Other:	

Is the ECG change clinically significant?

☐☐ Yes ☐ No → *If No, please specify below, why ECG is abnormal, but not clinically significant!*

Assessment and recommendation:

Does the ECG change meet the case definition for Myocarditis/Pericarditis?

☐ No

☐ Yes → ☐ Possible ☐ Probable ☐ Confirmed

Signature Cardiologist: [signature] 25.9.06

Betreff: POX-MVA-005, Subject 580: mild pericardial effusion - no SIAE
Absender: "Garth Virgin" <Garth.Virgin@Bavarian-Nordic.com>
Empfänger: "de la Motte, Dr. Stephan" <SdelaMotte@harrison-cro.com>; "Ilchmann, Heiko" <HIlchmann@harrison-cro.com>
Kopie-Empfänger: "F. v. Sonnenburg" <sonnenburg@lrz.uni-muenchen.de>; "Nadine Uebler" <nadine.uebler@bavarian-nordic.com>; "Jens Vollmar" <jens.vollmar@bavarian-nordic.com>; "Alfred von Krempelhuber" <alfred.krempelhuber@bavarian-nordic.com>; "Zillikens, Sabine" <SZillikens@harrison-cro.com>; "Augustin, Andrea" <AJ.Augustin@T-Online.de>
Datum: 15. Nov 2006 16:41

Dear Clinical Team,

The available documentation in conjunction with this reported case of "mild pericardial effusion" diagnosed in a 21 yo female medical student (Subject #580) was forwarded to Professor von Sonnenburg for his review. Due to a slightly irregular ECG the subject had been referred to the local cardiologist, Fr. Dr. Zitzmann-Roth who diagnosed – by chance – a mild pericardial effusion in the echocardiogram.

Based on the assessment by Dr. Zitzmann-Roth stating that the ECG tracing and the mild pericardial effusion are not of clinical significance and the latter being purely a coincidental finding, it was agreed that the reported event actually does not qualify as a Special Interest Adverse Event. According to the protocol: An "adverse event of special interest" is defined in this study as an adverse event fulfilling any of the following features:

- Any cardiac symptom
- ECG changes, which are <u>determined to be clinically significant</u>
- Cardiac enzymes elevated above ULN

Being not clinically significant, this event was not forwarded to the DSMB for their review.

Should there be a need to discuss further, please feel free to contact me.

Cheers – Garth Virgin

Garth Virgin, M.D.
Global Drug Safety Officer

Bavarian Nordic GmbH
Direct phone: +49 89 85 65 1319
Cell phone: +49 172 83 166 04

Fraunhoferstrasse 13 Tel: +49 89 8565 0030
D-82152 Martinsried Fax: +49 89 8565 1333
Germany http://www.bavarian-nordic.com

The information transmitted is intended only for the person or entity to which it is addressed and may contain confidential and/or privileged material. Any review, re-transmission, dissemination or other use of, or tak of any action in reliance upon, this information by persons or entities other than the intended recipient is prohibited. If you received this in error, please contact the sender and delete the material from any computer.

NOTE TO FILE No.: 14 (please refer to Log sheet for note to files)

**SPONSOR CODE**	POX-MVA-005	**INVESTIGATOR**	DR. DE LA MOTTE
**HCR STUDY CODE**	1029 / BN	**CENTER**	01 CLINICAL UNIT
**DATE**	24 JAN 07	**MONITOR**	N/A

☒ **STUDY SPECIFIC**	☐ **CENTER SPECIFIC**	☐ **SUBJECT SPECIFIC**<br>SUBJECT NO.:
**REASON(S):**<br>☐ Issues that are not explicitly defined in the protocol, however need further clarification	☐ Systematically missed assessments<br>☐ Assessment systematically conducted at incorrect time point<br>☐ A signature on a relevant study document could not be obtained<br>☐ GCP/SOP violation (other than above)	☐ Informed consent form incorrectly filled in<br>☐ Waivers for selection criteria<br>☐ Relevant information not covered by the CRF<br>☐ GCP/SOP violation (other than above)
☒ Other: documentation subject identification and enrolment log	☐ Other:	☐ Other:

DESCRIPTION:

The following changes to the corrections to be done on the subject identification and enrolment log are valid as from 24 Jan 07 regarding Note to file #08.

- For all subjects that have not signed an Informed Consent Form, the names have to be blackened by either a study nurse or an investigator, dated and initialled. In addition these entries have to be marked with "NC=NOT CONSENTED"
- At the beginning it was agreed upon with QA department that names of subjects that are screening failures have to be strike-through, after further discussion with the co-PI it was agreed upon that these names will only be marked with SF and / or EX, therefore you will find both names of screening failures that are strike-through but legible or only marked as described below
- For all subjects that have signed an Informed Consent, but the subject is not participating in the study, the names have to be marked with either "SF=SCREENING FAILURE and /or EX=EXCLUDED".

24 Jan 07
Date

[signature]
Signature

☐ CRA
☒ Project Manager
☐ Rapporteur

NOTE TO FILE No.: 15 (please refer to Log sheet for note to files)

SPONSOR CODE	POX-MVA-005	INVESTIGATOR	DR. DE LA MOTTE
HCR STUDY CODE	1029 / BN	CENTER	01 CLINICAL UNIT
DATE	30 JAN 2007	MONITOR	DR. A. AUGUSTIN

Harrison

☒ STUDY SPECIFIC	☐ CENTER SPECIFIC	☐ SUBJECT SPECIFIC
**REASON(S):** ☐ Issues that are not explicitly defined in the protocol, however need further clarification	☐ Systematically missed assessments<br>☐ Assessment systematically conducted at incorrect time point<br>☐ A signature on a relevant study document could not be obtained<br>☐ GCP/SOP violation (other than above)	☐ Informed consent form incorrectly filled in<br>☐ Waivers for selection criteria<br>☐ Relevant information not covered by the CRF<br>☐ GCP/SOP violation (other than above)
☒ Other: Ethical Approval	☐ Other:	☐ Other: suspected SIAE

DESCRIPTION:

Concerning ethical approval of the subject diary, the informed consent and the study participant card ("Studienteilnehmerkarte") the secretary of the ethics committee (Ethikkommission der Medizinischen Fakultät der LMU München") has mixed up dates and version numbers.

In their final approval letter dated 06.04.06, they approved the

- Diary Version 6 dated 03 Mar 2006
- Study participant card ("Studienteilnehmerkarte")
- Informed Consent Version 3.0 dated 28 Feb 2006

although Informed Consent Version 4.0. dated 22 Mar 2006 has already been sent on 22.03.06. Additionally, they refer to a previous letter from BN dated 03.03.06 which did not contain the study participant card.

However, in the ethics approval letter from 28.03.2006, they approved Informed Consent Version 4.0 dated 22 Mar 2006 again referring to the wrong application letter which is dated 03.03.06. Version 4.0 has been sent by BN thereafter namely on 22.03.06.

Upon request the ethical committee sent another approval letter on 02.05.06 with the correct references.

(Concerning the approval of Informed Consent Version 5.0 dated 03 May 2006 the ethics secretary again referred to the BN letter from 03.03.06 but recorded the correct IC version.)

In summary, all actual documents used in the study have been approved by the ethics committee.

Attachments:

- Application letters from BN to the ethics committee from 03.03.06 and 22.03.06
- Approval letters from the ethics committee from 28.03.06, 06.04.06 and 02.05.06

Date	Signature	
30 JAN 2007	[signature]	☒ CRA<br>☐ Project Manager<br>☐ Rapporteur

Ethikkommission der Medizinischen Fakultät
der Ludwig-Maximilians Universität
Vorsitzender: Prof. Dr. Gustav Paumgartner

LMU
Ludwig-Maximilians-Universität München
Marchioninistr. 15
81377 München
Tel: (089) 7095 4609
Fax: (089) 7095 7609
e-mail: Ethikkommission@med.uni-muenchen.de
02.05.2006

Klinikum der Universität München – Großhadern
Marchioninistr. 15 81377 München

Bavarian Nordic GmbH
Herrn Dr. J. Vollmar
Fraunhoferstraße 13
82152 Martinsried

EudraCT-Nr. 2005-001781-14
Teilweise randomisierte, teilweise doppelblinde, placebo-kontrollierte Phase II Nichtunterlegenheitsstudie zur Bewertung der Immunogenität und Sicherheit von ein und zwei Dosen eines Pockenimpfstoffes bei 18-55 Jahre alten gesunden Probanden
Projekt-Nr. 365-05

Sehr geehrter Herr Dr. Vollmar,
sehr geehrte Frau Uebler,

besten Dank für Ihr Schreiben vom 22.03.2006 mit einer nochmals überarbeiteten Version des Probandentagebuchs und der Patienteninformation zu der o.g. Studie. Dabei lagen im Einzelnen zur Begutachtung vor:

- Tagebuch Version 6 (final) 03 Mar 2006
- Studienteilnehmer- Karte
- Probandeninformation und Einverständniserklärung Version 3.0 28 Feb 2006
- Probandeninformation und Einverständniserklärung Version 4.0 22 Mar 2006.

Es bestehen keine ethisch-rechtlichen Bedenken gegen die Verwendung dieser Patienteninformation, so dass die schon ausgesprochene ethisch-rechtliche Unbedenklichkeit weiter Gültigkeit hat.

Ich wünsche Ihrer Studie weiterhin einen guten Verlauf.

Mit freundlichen Grüßen

Prof. Dr. G. Paumgartner
Vorsitzender der Ethikkommission

Mitglieder der Kommission:
Prof. Dr. G. Paumgartner (Vorsitzender), Prof. Dr. E. Held (stellv. Vorsitzender), Prof. Dr. H. U. Gallwas, Prof. Dr. D. Kunze, Dr. V. Mönch, PD Dr. V. Nüßler, Prof. Dr. R. Penning, Prof. Dr. K. Hahn, Prof. Dr. K. Pfeifer

FR 5 325/00 Mh.

Ethikkommission der Medizinischen Fakultät
der Ludwig-Maximilians Universität
Vorsitzender: Prof. Dr. Gustav Paumgartner

LMU
Ludwig-Maximilians-Universität-München
Marchioninistr. 15
81377 München
Tel: (089) 7095 4609
Fax: (089) 7095 7609
e-mail: Ethikkommission@med.uni-muenchen.de

28.03.2006

Klinikum der Universität München – Großhadern
Marchioninistr. 15 81377 München

Bavarian Nordic GmbH
Herrn Dr. J. Vollmar
Fraunhoferstraße 13
82152 Martinsried

EudraCT-Nr. 2005-001781-14
Teilweise randomisierte, teilweise doppelblinde, placebo-kontrollierte Phase II Nichtunterlegenheitsstudie zur Bewertung der Immunogenität und Sicherheit von ein und zwei Dosen eines Pockenimpfstoffes bei 18-55 Jahre alten gesunden Probanden
Projekt-Nr. 365-05

Sehr geehrter Herr Dr. Vollmar,
sehr geehrte Frau Uebler,

besten Dank für Ihr Schreiben vom 03.03.2006 mit einer überarbeiteten Version des Probandentagebuchs und der Patienteninformation zu der o.g. Studie. Dabei lagen im Einzelnen zur Begutachtung vor:

- Tagebuch Version 6 (final) 03 Mar 2006
- Probandeninformation und Einverständniserklärung Version 4.0 22 Mar 2006.

Es bestehen keine ethisch-rechtlichen Bedenken gegen die Verwendung dieser Patienteninformation und des Tagebuchs, so dass die schon ausgesprochene ethisch-rechtliche Unbedenklichkeit weiter Gültigkeit hat.

Ich wünsche Ihrer Studie weiterhin einen guten Verlauf.

Mit freundlichen Grüßen

G. Paumgartner

Prof. Dr. G. Paumgartner
Vorsitzender der Ethikkommission

Mitglieder der Kommission:
Prof. Dr. G. Paumgartner (Vorsitzender), Prof. Dr. E. Held (stellv. Vorsitzender), Prof. Dr. H. U. Gallwas, Prof. Dr. D. Kunze, Dr. V. Mönch, PD Dr. V. Nüßler, Prof. Dr. R. Penning, Prof. Dr. K. Hahn, Prof. Dr. K. Pfeifer

FR 3 325/00 Mü.

Bavarian Nordic GmbH, Fraunhoferstraße 13, 82152 Martinsried, Germany

Ethik-Kommission der Medizinischen Fakultät der LMU München
Prof. Dr. Gustav Paumgartner
Marchioninistr. 15

81377 München

Martinsried, 22. März 2006

Nachrichtlich:
Ethikkommission der Bayerischen
Landesärztekammer
Mühlbaurstraße 16
81677 München

Eudract-Nr. 2005-001781-14
Projekt-Nr. 365-05
Teilweise randomisierte, teilweise doppelblinde, placebo-kontrollierte Phase II Nichtunterlegenheitsstudie zur Bewertung der Immunogenität und Sicherheit von ein und zwei Dosen eines Pockenimpfstoffes bei 18-55 Jahre alten gesunden Probanden

Sehr geehrter Herr Prof. Dr. Paumgartner,

In der Anlage zu diesem Schreiben finden Sie überarbeitete Versionen des Probandentagebuchs und der Patienteninformation zur oben genannten Studie, sowie die Studienteilnehmerkarte dieser Studie mit der Bitte um Kenntnisnahme.

In der Final Version 6 des Probandentagebuchs vom 03. März 2006 wurde das Format verändert und zusätzlich ein Bereich zur Beurteilung des Zusammenhangs der Symptome mit der Prüfmedikation durch den Prüfarzt eingefügt.

In der überarbeiteten Version 3.0 der Probandeninformation vom 28.02.2006 wurde ein zusätzlicher Satz zur Aufwandsentschädigung eingefügt. Demnach werden zusätzliche, vom Prüfarzt veranlasste Studienbesuche mit 50 € entschädigt.
In der überarbeiteten Version 4.0 der Probandeninformation vom 22.03.2006 wurde die Adresse der Clinical Unit von Harrison Clinical Research GmbH geändert. Die Probanden der oben genannten Studie werden in der Tagesklinik der Albrechtstraße 43 (ein weiteres Gebäude von Harrison Clinical Research GmbH) behandelt und untersucht werden.
Die Änderungen in der Probandeninformation 4.0 vom 22.03.2006, im Vergleich zur letzten Ihnen vorgelegten Version 2.0 vom 27.01.2006, sind durch Unterstreichung kenntlich gemacht.

BAVARIAN NORDIC
Bavarian Nordic GmbH, Fraunhoferstrasse 13, D-82152 Martinsried, Germany, Phone +49 (0)89 8565 0030, Fax +49 (0)89 8565 1333
www.bavarian-nordic.com, General Manager: Paul Chaplin Ph.D., VAT ID No.: DE 813 250 971, Reg.: Amtsgericht München, HRB 155872

Ebenfalls in der Anlage finden Sie die Studienteilnehmerkarte mit Kontaktinformationen (inklusive Notrufnummer) zur oben genannten Studie, die dem Probanden ausgehändigt wird.

Mit freundlichen Grüßen,

Dr. Jens Vollmar
Medical Director

Nadine Uebler
Clinical Development Specialist

Anlagen
Probandentagebuch, Final Version 6 vom 03. März 2006
Probandeninformation, Version 3.0 vom 28.02.2006
Probandeninformation, Version 4.0 vom 22.03.2006
Studienteilnehmerkarte

Ethikkommission der Medizinischen Fakultät
der Ludwig-Maximilians Universität
Vorsitzender: Prof. Dr. Gustav Paumgartner

LMU
Ludwig-Maximilians-Universität München

Marchioninistr. 15
81377 München
Tel: (089) 7095 4609
Fax: (089) 7095 7609
e-mail: Ethikkommission@med.uni-muenchen.de

06.04.2006

Klinikum der Universität München – Großhadern
Marchioninistr. 15 81377 München

Bavarian Nordic GmbH
Herrn Dr. J. Vollmar
Fraunhoferstraße 13
82152 Martinsried

EudraCT-Nr. 2005-001781-14
Teilweise randomisierte, teilweise doppelblinde, placebo-kontrollierte Phase II Nichtunterlegenheitsstudie zur Bewertung der Immunogenität und Sicherheit von ein und zwei Dosen eines Pockenimpfstoffes bei 18-55 Jahre alten gesunden Probanden
Projekt-Nr. 365-05

Sehr geehrter Herr Dr. Vollmar,
sehr geehrte Frau Uebler,

besten Dank für Ihr Schreiben vom 03.03.2006 mit einer überarbeiteten Version des Probandentagebuchs und der Patienteninformation zu der o.g. Studie. Dabei lagen im Einzelnen zur Begutachtung vor:

- Tagebuch Version 6 (final) 03 Mar 2006
- Studienteilnehmer- Karte
- Probandeninformation und Einverständniserklärung Version 3.0 28 Feb 2006.

Es bestehen keine ethisch-rechtlichen Bedenken gegen die Verwendung dieser Patienteninformation, so dass die schon ausgesprochene ethisch-rechtliche Unbedenklichkeit weiter Gültigkeit hat.

Ich wünsche Ihrer Studie weiterhin einen guten Verlauf.

Mit freundlichen Grüßen

G. Paumgartner

Prof. Dr. G. Paumgartner
Vorsitzender der Ethikkommission

Mitglieder der Kommission:
Prof. Dr. G. Paumgartner (Vorsitzender), Prof. Dr. E. Held (stellv. Vorsitzender), Prof. Dr. H. U. Gallwas, Prof. Dr. D. Kunze, Dr. V. Mönch, PD Dr. V. Nüßler, Prof. Dr. R. Penning, Prof. Dr. K. Hahn, Prof. Dr. K. Pfeifer

FR 3 325/00 Ml.

Bavarian Nordic GmbH, Fraunhoferstraße 13, 82152 Martinsried, Germany

Ethik-Kommission der Medizinischen Fakultät der LMU München
Prof. Dr. Gustav Paumgartner
Marchioninistr. 15

81377 München

Martinsried, 03. März 2006

Nachrichtlich:
Ethikkommission der Bayerischen
Landesärztekammer
Mühlbaurstraße 16
81677 München

Eudract-Nr. 2005-001781-14
Projekt-Nr. 365-05
Teilweise randomisierte, teilweise doppelblinde, placebo-kontrollierte Phase II Nichtunterlegenheitsstudie zur Bewertung der Immunogenität und Sicherheit von ein und zwei Dosen eines Pockenimpfstoffes bei 18-55 Jahre alten gesunden Probanden

Sehr geehrter Herr Prof. Dr. Paumgartner,

In der Anlage zu diesem Schreiben finden Sie die überarbeiteten Versionen des Probandentagebuchs und der Patienteninformation zur oben genannten Studie mit der Bitte um Kenntnisnahme.

In der Final Version 6 des Probandentagebuchs vom 03. März 2006 wurde das Format verändert und zusätzlich ein Bereich zur Beurteilung des Zusammenhangs der Symptome mit der Prüfmedikation durch den Prüfarzt eingefügt.
In der überarbeiteten Version 3.0 der Probandeninformation vom 28.02.2006 wurde ein zusätzlicher Satz zur Aufwandsentschädigung eingefügt. Demnach werden zusätzliche, vom Prüfarzt veranlasste Studienbesuche mit 50 € entschädigt. Der entsprechende Satz ist in der Probandeninformation durch Unterstreichung kenntlich gemacht (S.7, Version 3.0, 28.02.2006).

Mit freundlichen Grüßen,

i.A. Dr. Jens Vollmar
Medical Director

Nadine Uebler
Clinical Development Specialist

Anlagen

Harrison Clinical Research

NOTE TO FILE No.: 16 (please refer to Log sheet for note to files)

**SPONSOR CODE**	POX-MVA-005	**INVESTIGATOR**	DR. DE LA MOTTE
**HCR STUDY CODE**	1029 / BN	**CENTER**	01 CLINICAL UNIT
**DATE**	14 FEB 07	**MONITOR**	NA

Harrison

☐ **STUDY SPECIFIC**	☐ **CENTER SPECIFIC**	☒ **SUBJECT SPECIFIC**<br>SUBJECT NO.: **NA**
**REASON(S):**<br>☐ Issues that are not explicitly defined in the protocol, however need further clarification	☐ Systematically missed assessments<br>☐ Assessment systematically conducted at incorrect time point<br>☐ A signature on a relevant study document could not be obtained<br>☐ GCP/SOP violation (other than above)	☐ Informed consent form incorrectly filled in<br>☐ Waivers for selection criteria<br>☐ Relevant information not covered by the CRF<br>☐ GCP/SOP violation (other than above)
☐ Other:	☐ Other:	☒ Other: I.C. documentation screening failures

DESCRIPTION:

This note to file refers to the documentation in the Informed consent form done by subjects who were not enrolled / randomised for the study (screening failures).

During the BN Meeting on 06.09.06 it was decided with the sponsor that no follow-up is necessary if an IC from a non-randomised participant is correctly dated and signed on page 8 but not on pages 9 and / or 11.

In one case (ScrNo. 0645) signature and date are missing on page 8 but were given on pages 9 and 11, while the investigator signed and dated the IC correctly, also in this case the signature was not requested as the subject was not randomised. Same applies for subject ScrNos.0093, 0166, 0310, 0665, 0742 and 1280 who have neither dated page 8 correctly or did not sign correctly, while pages 9 and /or 11 are signed and dated correctly.
For ScrNo. 0264 the IC is signed correctly only the year is missing in the date format.

14. Feb. 07 [signature]

Date Signature ☐ CRA ☒ Project Manager ☐ Rapporteur

14 FEB 2007 [signature]

Date SIGNATURE ☒ Investigator

NOTE TO FILE No.: 17 (please refer to Log sheet for note to files)

**SPONSOR CODE**	POX-MVA-005	**INVESTIGATOR**	DR. DE LA MOTTE
**HCR STUDY CODE**	1029 / BN	**CENTER**	01 CLINICAL UNIT
**DATE**	02 FEB 07	**MONITOR**	E. KARDALINOU

Harrison

☐ **STUDY SPECIFIC**	☐ **CENTER SPECIFIC**	☒ **SUBJECT SPECIFIC**<br>SUBJECT NO.: **433**
**REASON(S):**<br>☐ Issues that are not explicitly defined in the protocol, however need further clarification	☐ Systematically missed assessments<br>☐ Assessment systematically conducted at incorrect time point<br>☐ A signature on a relevant study document could not be obtained<br>☐ GCP/SOP violation (other than above)	☐ Informed consent form incorrectly filled in<br>☐ Waivers for selection criteria<br>☐ Relevant information not covered by the CRF<br>☐ GCP/SOP violation (other than above)
☐ Other:	☐ Other:	☒ Other: I.C. Version 5.0 not signed by subject

DESCRIPTION:

Subject no 433:

Subject did sign I.C. Version 4.0 at Screening Visit on 15.05.06.
The subject had a Hepatitis A infection in April 06, the GOT / GPT lab values being elevated at screening visit. In agreement with the sponsor the following enrollment in the trial was postponed for 2 months.
At the Re-Screening Visit on the 07.07.06 the lab values for GOT / GPT were within ref. range and there were no concerns from the investigator side regarding the subject's participation in this trial.
Subject forgot at the Re-Screening Visit to sign the current I.C. Version No. 5.0.

As the subject withdrew her/his consent on 13.09.06, no small-pox vaccination took place.

Date 10.02.2007 Signature

☒ CRA Efterpi Kardalinou
☐ Project Manager
☐ Rapporteur

Harrison Clinical Research

Harrison

NOTE TO FILE No.: 18 (please refer to Log sheet for note to files)

**SPONSOR CODE**	POX-MVA-005	**INVESTIGATOR**	DR. DE LA MOTTE
**HCR STUDY CODE**	1029 / BN	**CENTER**	01 CLINICAL UNIT
**DATE**	14 FEB 07	**MONITOR**	E. KARDALINOU

☐ **STUDY SPECIFIC**	☐ **CENTER SPECIFIC**	☒ **SUBJECT SPECIFIC**<br>SUBJECT NO.: **665**<br>**(SCREENING FAILURE)**
**REASON(S):**<br>☐ Issues that are not explicitly defined in the protocol, however need further clarification	☐ Systematically missed assessments<br>☐ Assessment systematically conducted at incorrect time point<br>☐ A signature on a relevant study document could not be obtained<br>☐ GCP/SOP violation (other than above)	☒ Informed consent form incorrectly filled in<br>☐ Waivers for selection criteria<br>☐ Relevant information not covered by the CRF<br>☐ GCP/SOP violation (other than above)
☐ Other:	☐ Other:	☐ Other:

DESCRIPTION:

This note to file is to document that the Informed Consent Form for Screening No.665 was not correctly signed by mistake by Dr.P.Schmitt. Unfortunately Dr.Schmitt is not longer working for HCR, therefore could only be reached by phone to clarify this issue.
Dr.Schmitt confirmed on the phone that all patients screened have been informed about trial participation in detail before screening assessments have been done, by mistake he forgot to sign on the Informed Consent Form.
Subject 665 signed the Informed Consent correctly but forgot to enter the date on pages 8 and 9, only page 11 is dated and signed by the subject.

14/02/07 [signature]

Date 14.02.2007 Signature ☒ CRA Efterpi Kardalinou
☐ Project Manager
☐ Rapporteur

14 FEB 2007 [signature]

Date 14.02.2007 SIGNATURE ☒ Investigator

NOTE TO FILE No.: 19 (please refer to Log sheet for note to files)

**SPONSOR CODE**	POX-MVA-005	**INVESTIGATOR**	DR. DE LA MOTTE
**HCR STUDY CODE**	1029 / BN	**CENTER**	01 CLINICAL UNIT
**DATE**	14 FEB 07	**MONITOR**	E. KARDALINOU

☐ **STUDY SPECIFIC**	☐ **CENTER SPECIFIC**	☒ **SUBJECT SPECIFIC**<br>SUBJECT NO.: **719**<br>**(SCREENING FAILURE)**
**REASON(S):**<br>☐ Issues that are not explicitly defined in the protocol, however need further clarification	☐ Systematically missed assessments<br>☐ Assessment systematically conducted at incorrect time point<br>☐ A signature on a relevant study document could not be obtained<br>☐ GCP/SOP violation (other than above)	☐ Informed consent form incorrectly filled in<br>☐ Waivers for selection criteria<br>☐ Relevant information not covered by the CRF<br>☐ GCP/SOP violation (other than above)
☐ Other:	☐ Other:	☒ Other: Date of ICF signature

DESCRIPTION:
The investigator has signed this Informed Consent Form (ICF) on the day of informing the subject.
The subject has signed this ICF later, after considering study participation, on the day of the screening examinations, before the start of screening examinations.

14.02.07 [signature]

Date
14.02.2007

Signature

☒ CRA Efterpi Kardalinou
☐ Project Manager
☐ Rapporteur

14 FEB 2007 [signature]

Date
14.02.2007

SIGNATURE

☒ Investigator

NOTE TO FILE No.: 20 (please refer to Log sheet for note to files)

**SPONSOR CODE**	POX-MVA-005	**INVESTIGATOR**	DR. DE LA MOTTE
**HCR STUDY CODE**	1029 / BN	**CENTER**	01 CLINICAL UNIT
**DATE**	28 MAR 07	**MONITOR**	N/A

☒ **STUDY SPECIFIC**	☐ **CENTER SPECIFIC**	☐ **SUBJECT SPECIFIC**<br>SUBJECT NO.:
**REASON(S):**<br>☐ Issues that are not explicitly defined in the protocol, however need further clarification	☐ Systematically missed assessments<br>☐ Assessment systematically conducted at incorrect time point<br>☐ A signature on a relevant study document could not be obtained<br>☐ GCP/SOP violation (other than above)	☐ Informed consent form incorrectly filled in<br>☐ Waivers for selection criteria<br>☐ Relevant information not covered by the CRF<br>☐ GCP/SOP violation (other than above)
☒ Other: Responsibility of Investigator in absence of Principal Investigator	☐ Other:	☐ Other:

DESCRIPTION:

Clarification of the responsibility of the Investigator in the absence of the Principal Investigator

This Note to File is to clarify the role and responsibility of Dr. Stephan de la Motte during the absence of Prof. Frank von Sonnenburg in this trial.

The clinical trial is conducted at one site. This site is the Clinical Unit of the CRO Harrison Clinical Research GmbH.

Prof. Frank von Sonnenburg acts as Principal Investigator of this trial at this site. He was chosen due to his gathered experience with the investigational product MVA-BN® (IMVAMUNE™) in other clinical trials.
Dr. Stephan de la Motte is Chief Medical Officer of the Clinical Unit of Harrison Clinical Research GmbH and he acts as Investigator in this trial.

In the absence of Prof.Frank von Sonnenburg his deputy is Dr. Stephan de la Motte in this trial with all responsibilities as Prof. von Sonnenburg. For example, among other duties, Dr. de la Motte takes over the responsibility of approving and signing-off of "exception to patient entry criteria".

April 30 2007

Date SIGNATURE

PROF. FRANK VON SONNENBURG (PRINCIPAL INVESTIGATOR)

05 APR 2007

Date SIGNATURE

DR. STEPHAN DE LA MOTTE (INVESTIGATOR)

Harrison Clinical Research

Harrison

NOTE TO FILE No.: 21

(please refer to Log sheet for note to files)

**SPONSOR CODE**	POX-MVA-005	**INVESTIGATOR**	DR. DE LA MOTTE
**HCR STUDY CODE**	1029 / BN	**CENTER**	01 CLINICAL UNIT
**DATE**	24/APR/2007	**MONITOR**	BRITTA SCHALLHAMMER

☐ **STUDY SPECIFIC**	☐ **CENTER SPECIFIC**	☒ **SUBJECT SPECIFIC**<br>SUBJECT NO.: **699 AND 702**
**REASON(S):**<br>☐ Issues that are not explicitly defined in the protocol, however need further clarification	☐ Systematically missed assessments<br>☐ Assessment systematically conducted at incorrect time point<br>☐ A signature on a relevant study document could not be obtained<br>☐ GCP/SOP violation (other than above)	☐ Informed consent form incorrectly filled in<br>☐ Waivers for selection criteria<br>☐ Relevant information not covered by the CRF<br>☐ GCP/SOP violation (other than above)
☐ Other:	☐ Other:	☒ Other: lab print outs mixed up

DESCRIPTION:

The lab printout of the Subjects 699 (SCR 1136) and 702 (SCR 1135) were mixed up by a mistake.

In both patient notes the lab print outs of V2/1 from 09/Nov/06 and V2/2 from 17/Nov/06 are files
Both subjects had the identical lab print out, but this could be clarified with the following arguments:

Subject 702 came to the regular V2 on 09/Nov/2007 and blood was taken.

Subject 699 came to the regular V2 on 17/Nov/2007 and also blood was taken.

This blood test dates can be confirmed by the entries of the Visit log sheet.
The lab print out of Subject 699 is congruent with date of birth, screening and patient number of the Lab V2/2 and could be assigned to the accession no.: 317366

The lab print out of Subject 702 was assigned to 699 due to a mistake, because the screening number on the patient number was badly written. So It's possible to read 1136 as screening number instead of 1135. On the basis of the V2 visit date it is possible to assign this lab to the accession no. 316827.

In both labs all values are within normal range.

27.4.07 [signature]

Date | Investigator's Signature

27.4.07 [signature]

Date | Signature

☒ CRA
☐ Project Manager
☐ Rapporteur

NOTE TO FILE **No.:** 22 (please refer to Log sheet for note to files)

**SPONSOR CODE**	POX-MVA-005	**INVESTIGATOR**	DR. DE LA MOTTE
**HCR STUDY CODE**	1029 / BN	**CENTER**	01 CLINICAL UNIT
**DATE**	23 JUL 07	**MONITOR**	E. KARDALINOU

Harrison

☐ STUDY SPECIFIC	☐ CENTER SPECIFIC	☒ SUBJECT SPECIFIC<br>SUBJECT NO.: **594**
**REASON(S):**<br>☐ Issues that are not explicitly defined in the protocol, however need further clarification	☐ Systematically missed assessments<br>☐ Assessment systematically conducted at incorrect time point<br>☐ A signature on a relevant study document could not be obtained<br>☐ GCP/SOP violation (other than above)	☐ Informed consent form incorrectly filled in<br>☐ Waivers for selection criteria<br>☐ Relevant information not covered by the CRF<br>☐ GCP/SOP violation (other than above)
☐ Other:	☐ Other:	☒ Other: Inclusion in group I-III by mistake

DESCRIPTION:

THIS NTF FOLLOWS UP NTF 11. CHANGES WERE INTRODUCED IN THE SECOND PARAGRAPH.

Subject: 594 (female; DOB: 21.11.79)

Subject 594 was assigned in this trial to group 1-3, as neither scars were detected by the physical examination nor small-pox vaccination records seemed to be in her possession.
The subject found apparently and showed her immunisation licence for the first time after the 2. vaccination, at V4. According to the licence she had been vaccinated against smallpox on 14.07.81 and 21.07.81.

For safety reasons the subject will proceed with the following visits (V5 and follow-up visit), including an additional ECG-assessment and safety lab examination at V5.

Date	Signature	
23. July 2007	[signature]	☒ CRA Efterpi Kardalinou<br>☐ Project Manager<br>☐ Rapporteur

NOTE TO FILE **No.: 23** (please refer to Log sheet for note to files)

**SPONSOR CODE**	POX-MVA-005	**INVESTIGATOR**	DR. DE LA MOTTE
**HCR STUDY CODE**	1029 / BN	**CENTER**	01 CLINICAL UNIT
**DATE**	23 JUL 07	**MONITOR**	E. KARDALINOU

☐ **STUDY SPECIFIC**	☐ **CENTER SPECIFIC**	☒ **SUBJECT SPECIFIC** *SCREENING* NO.: 433
**REASON(S):** ☐ Issues that are not explicitly defined in the protocol, however need further clarification	☐ Systematically missed assessments<br>☐ Assessment systematically conducted at incorrect time point<br>☐ A signature on a relevant study document could not be obtained<br>☐ GCP/SOP violation (other than above)	☐ Informed consent form incorrectly filled in<br>☐ Waivers for selection criteria<br>☐ Relevant information not covered by the CRF<br>☐ GCP/SOP violation (other than above)
☐ Other:	☐ Other:	☒ Other: I.C. Version 5.0 not signed by subject

DESCRIPTION:

THIS NTF FOLLOWS UP NTF 17. CHANGES / ADDITIONS ARE MADE IN ITALIC

***Screening* No : 433**

Subject did sign I.C. Version 4.0 at Screening Visit on 15.05.06.
The subject had a Hepatitis A infection in April 06, the GOT / GPT lab values being elevated at screening visit. In agreement with the sponsor the following enrollment in the trial was postponed for 2 months.
At the Re-Screening Visit on the 07.07.06 the lab values for GOT / GPT were within ref. range and there were no concerns from the investigator side regarding the subject's participation in this trial.
Subject forgot at the Re-Screening Visit to sign the current I.C. Version No. 5.0.

As the subject withdrew her/his consent on 13.09.06, no small-pox vaccination took place.

The subject did sign I.C. Version 5.0 on the 15th of February 2007.

Date	Signature	
23. July 2007	[signature]	☒ CRA Efterpi Kardalinou<br>☐ Project Manager<br>☐ Rapporteur

Harrison Clinical Research

NOTE TO FILE No.: 24 (please refer to Log sheet for note to files)

**SPONSOR CODE**	POX-MVA-005	**INVESTIGATOR**	DR. DE LA MOTTE
**HCR STUDY CODE**	1029 / BN	**CENTER**	01 CLINICAL UNIT
**DATE**	23 JUL 07	**MONITOR**	E. KARDALINOU

Harrison

☐ **STUDY SPECIFIC**	☐ **CENTER SPECIFIC**	☒ **SUBJECT SPECIFIC**<br>SUBJECT NO.: 685, 495, 444, 710, 751, 594, 411
**REASON(S):**<br>☐ Issues that are not explicitly defined in the protocol, however need further clarification	☐ Systematically missed assessments<br>☐ Assessment systematically conducted at incorrect time point<br>☐ A signature on a relevant study document could not be obtained<br>☐ GCP/SOP violation (other than above)	☐ Informed consent form incorrectly filled in<br>☐ Waivers for selection criteria<br>☐ Relevant information not covered by the CRF<br>☐ GCP/SOP violation (other than above)
☐ Other:	☐ Other:	☒ Other: SAE forms, page 2 of Version 20.12.05

DESCRIPTION:

The following mistake was done for the SAE reporting:
page 2 / Version 20.12.05 was used, instead of page 2 / Version 21.04.06 of the SAE forms.

The subjects concerned are as follows:
685, 495, 444, 710, 751, 594, 411 (only for the fu-report of 31.05.07).

Date	Signature	
23. July 2007	[signature]	☒ CRA Efterpi Kardalinou<br>☐ Project Manager<br>☐ Rapporteur

Harrison Clinical Research

NOTE TO FILE No.: 25 (please refer to Log sheet for note to files)

**SPONSOR CODE**	POX-MVA-005	**INVESTIGATOR**	DR. DE LA MOTTE
**HCR STUDY CODE**	1029 / BN	**CENTER**	01 CLINICAL UNIT
**DATE**	27 NOV 07	**MONITOR**	DR. E. KARDALINOU

Harrison

☐ **STUDY SPECIFIC**	☒ **CENTER SPECIFIC**	☐ **SUBJECT SPECIFIC**
**REASON(S):**<br>☐ Issues that are not explicitly defined in the protocol, however need further clarification	☐ Systematically missed assessments<br>☐ Assessment systematically conducted at incorrect time point<br>☐ A signature on a relevant study document could not be obtained<br>☐ GCP/SOP violation (other than above)	☐ Informed consent form incorrectly filled in<br>☐ Waivers for selection criteria<br>☐ Relevant information not covered by the CRF<br>☐ GCP/SOP violation (other than above)
☐ Other:	☒ Other: Documentation of temperature for the freezer	☐ Other:

DESCRIPTION:

Temperature graph print-out for the freezer where medication was stored

For the time period 6 February to 23 March 07, no temperature graph print-out for the freezer where medication was stored is available. The last vaccination date of participating subjects was on 20 December 06, whereas the last return-shipment of medication took place on 23 March 07.

In addition, according to the records the temperature between 6 Feb 06 to 6 Feb 07 was at an average of -21°C and herewith consistent with the temperature stipulated in the IMPD. The freezer where medication was stored is connected to an alarm system. No alarm was reported between 6 February to 23 March 07.

29 NOV 2007 [signature]

Date Investigator's Signature

30. Nov. 2007 [signature]

Date Signature

☒ CRA
☐ Project Manager
☐ Rapporteur

16.1.2 Sample case report form and patient diary

Empty CRFs for POX-MVA-005 (HCR: 1029/BN)

Screening

**Form: Visit Date + Subject Characteristics**		
**Visit Date**		
Visit Date	[ ]	
The next visit should be scheduled between the	[ ]	
and the	[ ]	
**Subject Characteristics**		
Screening number	[ ]	
Written informed consent was obtained on:	[ ]	
Date of birth	[ ]	
Age on signing informed consent	[ ]	
Gender	☐ male<br>☐ female	
Child-bearing potential	☐ surgically sterile or post-menopausal<br>☐ of child-bearing potential and using appropriate birth control	
Ethnic group	☐ caucasian<br>☐ asian<br>☐ black<br>☐ arabic<br>☐ other<br>☐ unknown<br>☐ -	
Ethnic group: please specify other	[ ]	
Height (without shoes)	[ ]	cm

Form: Smallpox Vaccination History	
**Smallpox Vaccination**	
History of smallpox vaccination?	☐ yes<br>☐ no
Presence of typical vaccination scar?	☐ yes<br>☐ no<br>☐ -
**Smallpox Vaccination History**	
Vaccination date	[ ]
Or month	☐ 01<br>☐ 02<br>☐ 03<br>☐ 04<br>☐ 05<br>☐ 06<br>☐ 07<br>☐ 08<br>☐ 09<br>☐ 10<br>☐ 11<br>☐ 12<br>☐ unknown<br>☐ -
and year	☐ 2007<br>☐ 2006<br>☐ 2005<br>☐ 2004<br>☐ 2003<br>☐ 2002<br>☐ 2001<br>☐ 2000<br>☐ 1999<br>☐ 1998<br>☐ 1997<br>☐ 1996<br>☐ 1995<br>☐ 1994<br>☐ 1993<br>☐ 1992<br>☐ 1991<br>☐ 1990<br>☐ 1989<br>☐ 1988<br>☐ 1987<br>☐ 1986

	☐ 1985<br>☐ 1984<br>☐ 1983<br>☐ 1982<br>☐ 1981<br>☐ 1980<br>☐ 1979<br>☐ 1978<br>☐ 1977<br>☐ 1976<br>☐ 1975<br>☐ 1974<br>☐ 1973<br>☐ 1972<br>☐ 1971<br>☐ 1970<br>☐ 1969<br>☐ 1968<br>☐ 1967<br>☐ 1966<br>☐ 1965<br>☐ 1964<br>☐ 1963<br>☐ 1962<br>☐ 1961<br>☐ 1960<br>☐ 1959<br>☐ 1958<br>☐ 1957<br>☐ 1956<br>☐ 1955<br>☐ 1954<br>☐ 1953<br>☐ 1952<br>☐ 1951<br>☐ 1950<br>☐ 1949<br>☐ 1948<br>☐ 1947<br>☐ 1946<br>☐ 1945<br>☐ 1944<br>☐ 1943<br>☐ 1942<br>☐ 1941<br>☐ unknown<br>☐ -

Vaccine	[ ]	

Form: Medical History	
**Medical History**	
Skin	☐ abnormal<br>☐ normal<br>☐ unknown<br>☐ -
Head, eyes, ears, nose and throat	☐ abnormal<br>☐ normal<br>☐ unknown<br>☐ -
Respiratory	☐ abnormal<br>☐ normal<br>☐ unknown<br>☐ -
Cardiovascular	☐ abnormal<br>☐ normal<br>☐ unknown<br>☐ -
Gastrointestinal and hepatic	☐ abnormal<br>☐ normal<br>☐ unknown<br>☐ -
Renal and genitourinary	☐ abnormal<br>☐ normal<br>☐ unknown<br>☐ -
Endocrine metabolic	☐ abnormal<br>☐ normal<br>☐ unknown<br>☐ -
Hemopoietic and lymphatic	☐ abnormal<br>☐ normal<br>☐ unknown<br>☐ -
Musculoskeletal	☐ abnormal<br>☐ normal<br>☐ unknown<br>☐ -
Surgical history	☐ abnormal<br>☐ normal<br>☐ unknown<br>☐ -

Psychiatric events	☐ abnormal<br>☐ normal<br>☐ unknown<br>☐ -	
Drug abuse of all kinds	☐ abnormal<br>☐ normal<br>☐ unknown<br>☐ -	
History of allergic reactions	☐ abnormal<br>☐ normal<br>☐ unknown<br>☐ -	
Other	☐ abnormal<br>☐ normal<br>☐ unknown<br>☐ -	

**Description of Medical History**	

	□ 2000<br>□ 1999<br>□ 1998<br>□ 1997<br>□ 1996<br>□ 1995<br>□ 1994<br>□ 1993<br>□ 1992<br>□ 1991<br>□ 1990<br>□ 1989<br>□ 1988<br>□ 1987<br>□ 1986<br>□ 1985<br>□ 1984<br>□ 1983<br>□ 1982<br>□ 1981<br>□ 1980<br>□ 1979<br>□ 1978<br>□ 1977<br>□ 1976<br>□ 1975<br>□ 1974<br>□ 1973<br>□ 1972<br>□ 1971<br>□ 1970<br>□ 1969<br>□ 1968<br>□ 1967<br>□ 1966<br>□ 1965<br>□ 1964<br>□ 1963<br>□ 1962<br>□ 1961<br>□ 1960<br>□ 1959<br>□ 1958<br>□ 1957<br>□ 1956<br>□ 1955<br>□ 1954

	☐ 1953<br>☐ 1952<br>☐ 1951<br>☐ 1950<br>☐ 1949<br>☐ 1948<br>☐ 1947<br>☐ 1946<br>☐ 1945<br>☐ 1944<br>☐ 1943<br>☐ 1942<br>☐ 1941<br>☐ unknown<br>☐ -

Form: Vital Signs + Physical Examination		
**Evaluation of Vital Signs**		
Systolic blood pressure	[ ]	mmHg
Diastolic blood pressure	[ ]	mmHg
Pulse rate	[ ]	bpm
Oral temperature	[ ]	°C (format: _ _ . _)
**Physical Examination - Abnormalities detected?**		
Weight	[ ]	kg (format: _ _ _ . _)
General appearance	☐ abnormal<br>☐ normal<br>☐ not done<br>☐ -	
Throat-Nose-Ears	☐ abnormal<br>☐ normal<br>☐ not done<br>☐ -	
Lung (incl. auscultation)	☐ abnormal<br>☐ normal<br>☐ not done<br>☐ -	
Heart (incl. auscultation)	☐ abnormal<br>☐ normal<br>☐ not done<br>☐ -	
Abdomen general	☐ abnormal<br>☐ normal<br>☐ not done<br>☐ -	
Lymph nodes (axillar/supraclavicular)	☐ abnormal<br>☐ normal<br>☐ not done<br>☐ -	
Short neurological status	☐ abnormal<br>☐ normal<br>☐ not done<br>☐ -	
Other	☐ abnormal<br>☐ normal<br>☐ not done<br>☐ -	
If Other=Abnormal, please specify category:	[ ]	

**Form: ECG**	
**ECG**	
ECG assessment performed by	☐ central cardiologist<br>☐ local cardiologist<br>☐ not required, as not clinically indicated<br>☐ not done<br>☐ -
ECG recording date	[ ]
ECG recording time	[ ]
ECG ID	[ ]
Assessment	☐ normal<br>☐ abnormal<br>☐ not evaluable<br>☐ -
If assessment is normal, please specify:	☐ typical<br>☐ variation<br>☐ -
Supraventricular arrhythmia	☐ yes<br>☐ no<br>☐ -
Ventricular arrhythmia	☐ yes<br>☐ no<br>☐ -
AV block	☐ no<br>☐ first degree<br>☐ second degree Type Mobitz I (Wenckebach)<br>☐ second degree Type Mobitz II<br>☐ third degree<br>☐ -
Left bundle branch block	☐ no<br>☐ incomplete<br>☐ complete<br>☐ -
Right bundle branch block	☐ no<br>☐ incomplete<br>☐ complete<br>☐ -
ST elevation	☐ yes<br>☐ no<br>☐ -
- V1	☐ yes

	☐ no<br>☐ -
- V2	☐ yes<br>☐ no<br>☐ -
- V3	☐ yes<br>☐ no<br>☐ -
- V4	☐ yes<br>☐ no<br>☐ -
- V5	☐ yes<br>☐ no<br>☐ -
- V6	☐ yes<br>☐ no<br>☐ -
- I	☐ yes<br>☐ no<br>☐ -
- II	☐ yes<br>☐ no<br>☐ -
- III	☐ yes<br>☐ no<br>☐ -
- aVR	☐ yes<br>☐ no<br>☐ -
- aVL	☐ yes<br>☐ no<br>☐ -
- aVF	☐ yes<br>☐ no<br>☐ -
ST depression	☐ yes<br>☐ no<br>☐ -
- V1	☐ yes<br>☐ no<br>☐ -
- V2	☐ yes

	☐ no<br>☐ -
- V3	☐ yes<br>☐ no<br>☐ -
- V4	☐ yes<br>☐ no<br>☐ -
- V5	☐ yes<br>☐ no<br>☐ -
- V6	☐ yes<br>☐ no<br>☐ -
- I	☐ yes<br>☐ no<br>☐ -
- II	☐ yes<br>☐ no<br>☐ -
- III	☐ yes<br>☐ no<br>☐ -
- aVR	☐ yes<br>☐ no<br>☐ -
- aVL	☐ yes<br>☐ no<br>☐ -
- aVF	☐ yes<br>☐ no<br>☐ -
T inversion - pathological	☐ yes<br>☐ no<br>☐ -
- V1	☐ yes<br>☐ no<br>☐ -
- V2	☐ yes<br>☐ no<br>☐ -
- V3	☐ yes

	☐ no<br>☐ -
- V4	☐ yes<br>☐ no<br>☐ -
- V5	☐ yes<br>☐ no<br>☐ -
- V6	☐ yes<br>☐ no<br>☐ -
- I	☐ yes<br>☐ no<br>☐ -
- II	☐ yes<br>☐ no<br>☐ -
- III	☐ yes<br>☐ no<br>☐ -
- aVR	☐ yes<br>☐ no<br>☐ -
- aVL	☐ yes<br>☐ no<br>☐ -
- aVF	☐ yes<br>☐ no<br>☐ -
QTc prolongation	☐ yes<br>☐ no<br>☐ -
Low voltage	☐ yes<br>☐ no<br>☐ -
Other	☐ yes<br>☐ no<br>☐ -
Other specify	[ ]
Detailed description	[ ]
Is the ECG change clinically significant?	☐ yes<br>☐ no

	☐ -
Please specify why abnormal ECG is not clinically significant	[ ]
Does the ECG change meet the case definition for Myocarditis/Pericarditis?	☐ yes<br>☐ no<br>☐ -
Yes --->	☐ possible<br>☐ probable<br>☐ confirmed<br>☐ -

Form: Clinical Laboratory	
**Serum Pregnancy Test**	
Sample taken	☐ yes<br>☐ no<br>☐ -
If no, please specify reason	[ ]
Sampling date	[ ]
Sampling time	[ ]
Laboratory order number	[ ]
**Blood Analysis**	
Safety laboratory sample taken (15ml)	☐ yes<br>☐ no<br>☐ not required, as not clinically indicated<br>☐ -
Safety laboratory sample, if not taken please specify reason	[ ]
Antibody analysis sample taken (8ml)	☐ yes<br>☐ no<br>☐ -
Antibody analysis sample, if not taken please specify reason	[ ]
Sampling date	[ ]
Sampling time	[ ]
Laboratory order number	[ ]
**Urine Analysis**	
Urine sample taken	☐ yes<br>☐ no<br>☐ -
If sample not taken, please specify reason	[ ]
Sampling date	[ ]
Sampling time	[ ]
Laboratory order number	[ ]

Form: Inclusion Criteria

Inclusion Criteria		
1. Male and female subjects between 18 and 55 years of age.	☐ yes<br>☐ no<br>☐ -	
2. Women must have a negative serum pregnancy test at screening and a negative urine or serum pregnancy test within 24 hours prior to vaccination.	☐ yes<br>☐ no<br>☐ -	
3. Women of childbearing potential must have used an acceptable method of contraception for 30 days prior to the first vaccination, must agree to use an acceptable method of contraception during the study, and must not become pregnant for at least 28 days after the last vaccination. A woman is considered of childbearing potential unless post-menopausal or surgically sterilized. (Acceptable contraception methods are restricted to abstinence, barrier contraceptives, intrauterine contraceptive devices or licensed hormonal products.)	☐ yes<br>☐ no<br>☐ -	
4. Read, signed and dated informed consent document after being advised of the risks and benefits of the study in a language understood by the subject signed, and prior to performance of any study specific procedure.	☐ yes<br>☐ no<br>☐ -	
5. Troponin I within normal institutional limits.	☐ yes<br>☐ no<br>☐ -	
6. White blood cells >= 2500/mm³ and <= 11,000/mm³.	☐ yes<br>☐ no<br>☐ -	
7. Absolute neutrophil count (ANC) >=1000/mm³.	☐ yes<br>☐ no<br>☐ -	
8. Negative urine glucose by dipstick or urinalysis.	☐ yes<br>☐ no<br>☐ -	
9. Hemoglobin >= LLN	☐ yes<br>☐ no<br>☐ -	
10. Platelets 100-440/nl	☐ yes<br>☐ no<br>☐ -	
11. Adequate renal function defined as: a. Serum creatinine without clinically significant findings b. Urine protein <= 30 mg/dL or none or trace proteinuria (by urinalysis or dip stick)	☐ yes<br>☐ no<br>☐ -	
12. Adequate hepatic function defined as: a. Total bilirubin <= 1.5 x ULN in the absence of other evidence of significant liver disease (healthy subjects without clinical disease with Morbus Meulengracht can be included). b. AST (SGOT), ALT (SGPT) and alkaline phosphatase without clinically significant findings	☐ yes<br>☐ no<br>☐ -	

13. Electrocardiogram (ECG) without abnormal findings (e.g. any kind of atrioventricular or intraventricular conditions or blocks such as complete left or right bundle branch block, AV-node block, QTc or PR prolongation, premature atrial contractions or other atrial arrhythmia, sustained ventricular arrhythmia, 2 premature ventricular contractions (PVC) in a row, ST elevation consistent with ischemia).	☐ yes<br>☐ no<br>☐ -	
14. Availability for follow-up for the planned duration of the study (26 weeks after last vaccination).	☐ yes<br>☐ no<br>☐ -	

**Additional criteria for vaccinia-naïve subjects**		
1. No history of known or suspected previous smallpox vaccination.	☐ yes<br>☐ no<br>☐ -	
2. No detectable vaccinia scar.	☐ yes<br>☐ no<br>☐ -	

**Additional criteria for previously vaccinated subjects**		
1. History of previous smallpox vaccination (documented and/or typical vaccinia scar).	☐ yes<br>☐ no<br>☐ -	
2. Most recent smallpox vaccination >= 5 years.	☐ yes<br>☐ no<br>☐ -	

Form: Exclusion Criteria

Exclusion Criteria		
1. Pregnant or breast-feeding women.	☐ yes<br>☐ no<br>☐ -	
2. Uncontrolled serious infection i.e. not responding to antimicrobial therapy.	☐ yes<br>☐ no<br>☐ -	
3. History of any serious medical condition, which in the opinion of the investigator would compromise the safety of the subject.	☐ yes<br>☐ no<br>☐ -	
4. History of or active autoimmune disease. Persons with vitiligo or thyroid disease taking thyroid replacement are not excluded.	☐ yes<br>☐ no<br>☐ -	
5. Known or suspected impairment of immunologic function including, but not limited to, clinically significant liver disease; diabetes mellitus; moderate to severe kidney impairment.	☐ yes<br>☐ no<br>☐ -	
6. History of malignancy, other than squamous cell or basal cell skin cancer, unless there has been surgical excision that is considered to have achieved cure. Subjects with history of skin cancer at the vaccination site are excluded.	☐ yes<br>☐ no<br>☐ -	
7. History or clinical manifestation of clinically significant and severe hematological, renal, hepatic, pulmonary, central nervous, cardiovascular or gastrointestinal disorders.	☐ yes<br>☐ no<br>☐ -	
8. Clinically significant mental disorder not adequately controlled by medical treatment.	☐ yes<br>☐ no<br>☐ -	
9. Any condition which might interfere with study objectives or would limit the subject's ability to complete the study or to be compliant in the opinion of the investigator.	☐ yes<br>☐ no<br>☐ -	
10. History of coronary heart disease, myocardial infarction, angina, congestive heart failure, cardiomyopathy, stroke or transient ischemic attack, uncontrolled high blood pressure, or any other heart condition under the care of a doctor.	☐ yes<br>☐ no<br>☐ -	
11. History of an immediate family member (father, mother, brother, or sister) who died due to ischemic heart disease before age 50 years.	☐ yes<br>☐ no<br>☐ -	
12. Ten percent or greater risk of developing a myocardial infarction or coronary death within the next 10 years using the National Cholesterol Education Program's risk assessment tool. NOTE: This criterion applies only to volunteers 20 years of age and older.	☐ yes<br>☐ no<br>☐ -	
13. History of intravenous drug abuse.	☐ yes<br>☐ no<br>☐ -	

14. History of allergic disease or reactions likely to be exacerbated by any component of the vaccine.	☐ yes<br>☐ no<br>☐ -	
15. Known allergy to egg or aminoglycoside (gentamicin).	☐ yes<br>☐ no<br>☐ -	
16. History of anaphylaxis or severe allergic reaction.	☐ yes<br>☐ no<br>☐ -	
17. Having received any vaccinations or planned vaccinations with a live vaccine within 30 days prior or after study vaccination.	☐ yes<br>☐ no<br>☐ -	
18. Having received any vaccinations or planned vaccinations with a killed vaccine within 14 days prior or after study vaccination.	☐ yes<br>☐ no<br>☐ -	
19. Chronic administration (defined as more than 14 days) of systemic immuno-suppressant drugs during a period starting from six months prior to administration of the vaccine and ending at study conclusion. (Corticosteroid nasal sprays are permissible. Persons who have used topical and inhaled steroids can be enrolled after their therapy is completed).	☐ yes<br>☐ no<br>☐ -	
20. Post organ transplant subjects whether or not receiving chronic immunosuppressive therapy.	☐ yes<br>☐ no<br>☐ -	
21. Administration or planned administration of immunoglobulins and/or any blood products during a period starting from 3 months prior to administration of the vaccine and ending at study conclusion.	☐ yes<br>☐ no<br>☐ -	
22. Use of any investigational or non-registered drug or vaccine other than the study vaccine within 30 days preceding the first dose of the study vaccine, or planned administration of such a drug during the study period.	☐ yes<br>☐ no<br>☐ -	
23. Study personnel.	☐ yes<br>☐ no<br>☐ -	

Form: Exception to Inclusion/Exclusion Criteria		
**Exception to inclusion/exclusion criteria**		
Inclusion/Exclusion Criteria not fulfilled, but according to sponsor subject is eligible to take part in the study. Date granted by sponsor:	[ ]	

Form: Eligibility + Review of Signs/Symptoms + Prior Medication + General Comments		
**Eligibility**		
Is the subject eligible for the study?	☐ yes<br>☐ no<br>☐ -	
If no, please specify why not:	[ ]	
**Review and General Comment**		
General comment	[ ]	

Baseline Signs/Symptoms

Form: Baseline Signs and Symptoms		
**Baseline Signs and Symptoms**		
Sign/Symptom	[ ]	
**Start**		
Start date	[ ]	
Or month	☐ 01<br>☐ 02<br>☐ 03<br>☐ 04<br>☐ 05<br>☐ 06<br>☐ 07<br>☐ 08<br>☐ 09<br>☐ 10<br>☐ 11<br>☐ 12<br>☐ unknown<br>☐ -	
and year	☐ 2007<br>☐ 2006<br>☐ 2005<br>☐ 2004<br>☐ 2003<br>☐ 2002<br>☐ 2001<br>☐ 2000<br>☐ 1999<br>☐ 1998<br>☐ 1997<br>☐ 1996<br>☐ 1995<br>☐ 1994<br>☐ 1993<br>☐ 1992<br>☐ 1991	

- ☐ 1990
- ☐ 1989
- ☐ 1988
- ☐ 1987
- ☐ 1986
- ☐ 1985
- ☐ 1984
- ☐ 1983
- ☐ 1982
- ☐ 1981
- ☐ 1980
- ☐ 1979
- ☐ 1978
- ☐ 1977
- ☐ 1976
- ☐ 1975
- ☐ 1974
- ☐ 1973
- ☐ 1972
- ☐ 1971
- ☐ 1970
- ☐ 1969
- ☐ 1968
- ☐ 1967
- ☐ 1966
- ☐ 1965
- ☐ 1964
- ☐ 1963
- ☐ 1962
- ☐ 1961
- ☐ 1960
- ☐ 1959
- ☐ 1958
- ☐ 1957
- ☐ 1956
- ☐ 1955
- ☐ 1954
- ☐ 1953
- ☐ 1952
- ☐ 1951
- ☐ 1950
- ☐ 1949
- ☐ 1948
- ☐ 1947
- ☐ 1946
- ☐ 1945
- ☐ 1944

	☐ 1943<br>☐ 1942<br>☐ 1941<br>☐ unknown<br>☐ -
Start time	[ ]
Start time as text	[ ]

**End**	
End date	[ ]
Or month	☐ 01<br>☐ 02<br>☐ 03<br>☐ 04<br>☐ 05<br>☐ 06<br>☐ 07<br>☐ 08<br>☐ 09<br>☐ 10<br>☐ 11<br>☐ 12<br>☐ continued<br>☐ unknown<br>☐ -
and year	☐ 2005<br>☐ 2006<br>☐ 2007<br>☐ continued<br>☐ unknown<br>☐ -
End time	[ ]
End time as text	[ ]

**Sign/Symptom Details**	
Outcome	☐ recovery without sequelae<br>☐ recovery with sequelae<br>☐ ongoing<br>☐ death<br>☐ unknown
SAE or Special Interest AE (SIAE)	☐ no<br>☐ SAE<br>☐ SIAE<br>☐ unknown<br>☐ -
Frequency	☐ [illegible]

Frequency	☐ single episode<br>☐ intermittent<br>☐ continuous<br>☐ unknown<br>☐ -
Intensity	☐ easily tolerated by subject<br>☐ sufficiently discomforting to interfere with daily activities<br>☐ prevents daily activities<br>☐ life threatening or disabling<br>☐ unknown<br>☐ -
Actions taken	☐ no action<br>☐ concomitant medication<br>☐ non-drug therapy<br>☐ hospitalization<br>☐ unknown<br>☐ -

Visit 1

Form: Randomisation + Visit Date + Check of Criteria		
**Confirmation of vaccination visit**		
Has the vaccination visit been confirmed with the subject?	☐ yes<br>☐ no<br>☐ -	
**Planned visit date**		
Planned visit date	[ ]	
**Assignment of vial number**		
Assigned vial number:	[ ]	
Please confirm, that the vial number has been added to the list of vials to be taken out of the freezer by re-entering the vial number	[ ]	
**Visit Date**		
Visit Date	[ ]	
The next visit should be scheduled between the	[ ]	
and the	[ ]	
**Recheck of Criteria**		
Is the subject still eligible for the study after rechecking the inclusion, exclusion and elimination criteria?	☐ yes<br>☐ no	

Form: Vital Signs + Physical Examination		
**Evaluation of Vital Signs**		
Systolic blood pressure	[ ]	mmHg
Diastolic blood pressure	[ ]	mmHg
Pulse rate	[ ]	bpm
Oral temperature	[ ]	°C (format: _ _ . _)
**Physical Examination - Changes since last examination?**		
Weight	[ ]	kg (format: _ _ _ . _)
General appearance	☐ yes<br>☐ no<br>☐ not done<br>☐ -	
Throat-Nose-Ears	☐ yes<br>☐ no<br>☐ not done<br>☐ -	
Lung (incl. auscultation)	☐ yes<br>☐ no<br>☐ not done<br>☐ -	
Heart (incl. auscultation)	☐ yes<br>☐ no<br>☐ not done<br>☐ -	
Abdomen general	☐ yes<br>☐ no<br>☐ not done<br>☐ -	
Lymph nodes (axillar/supraclavicular)	☐ yes<br>☐ no<br>☐ not done<br>☐ -	
Short neurological status	☐ yes<br>☐ no<br>☐ not done<br>☐ -	
Other	☐ yes<br>☐ no<br>☐ not done<br>☐ -	
If Other=Yes, please specify category:	[ ]	

Form: Clinical Laboratory

**Urine Pregnancy Test**	
Sample taken	☐ yes<br>☐ no<br>☐ -
If no, please specify reason	[ ]
Sampling date	[ ]
Sampling time	[ ]
Result of pregnancy test	☐ positive<br>☐ negative<br>☐ -

**Blood Analysis**	
Safety laboratory sample taken (15ml)	☐ yes<br>☐ no<br>☐ not required, as not clinically indicated<br>☐ -
Safety laboratory sample, if not taken please specify reason	[ ]
Antibody analysis sample taken (8ml)	☐ yes<br>☐ no<br>☐ -
Antibody analysis sample, if not taken please specify reason	[ ]
Sampling date	[ ]
Sampling time	[ ]
Laboratory order number	[ ]

Form: Vaccination + Diary Hand-out + Review AE + Concomitant Medication + General Comment

**Vaccination (subcutaneous)**	
Date of vaccination	[ ]
Time of vaccination	[ ]
Please re-enter the vial number of the vial administered!	[ ]
Arm used (non-dominant upper arm)	☐ left upper arm, deltoid region<br>☐ right upper arm, deltoid region<br>☐ -
Route of administration	☐ subcutaneous<br>☐ other<br>☐ -
Route of administration, if other please specify:	[ ]
Was there any deviation from protocol-defined vaccine preparation and administration?	☐ yes<br>☐ no<br>☐ -
If yes, please specify:	[ ]

**Diary Card Hand-Out**	
Diary card handed out?	☐ yes<br>☐ no<br>☐ -
Please provide reason why diary has not been handed-out:	[ ]

**Review and General Comment**	
General comment	[ ]

Visit 2

Form: Visit Date + Vital Signs + Physical Examination		
**Visit Date**		
Visit Date	[ ]	
The next visit should be scheduled between the	[ ]	
and the	[ ]	
**Evaluation of Vital Signs**		
Systolic blood pressure	[ ]	mmHg
Diastolic blood pressure	[ ]	mmHg
Pulse rate	[ ]	bpm
Oral temperature	[ ]	°C (format: _ _ . _)
**Physical Examination - Changes since last examination?**		
Weight	[ ]	kg (format: _ _ _ . _)
General appearance	☐ yes<br>☐ no<br>☐ not done<br>☐ -	
Throat-Nose-Ears	☐ yes<br>☐ no<br>☐ not done<br>☐ -	
Lung (incl. auscultation)	☐ yes<br>☐ no<br>☐ not done<br>☐ -	
Heart (incl. auscultation)	☐ yes<br>☐ no<br>☐ not done<br>☐ -	
Abdomen general	☐ yes<br>☐ no<br>☐ not done<br>☐ -	

Lymph nodes (axillar/supraclavicular)	☐ yes<br>☐ no<br>☐ not done<br>☐ -	
Short neurological status	☐ yes<br>☐ no<br>☐ not done<br>☐ -	
Other	☐ yes<br>☐ no<br>☐ not done<br>☐ -	
If Other=Yes, please specify category:	[ ]	

Form: ECG	
**ECG**	
ECG assessment performed by	☐ central cardiologist<br>☐ local cardiologist<br>☐ not required, as not clinically indicated<br>☐ not done<br>☐ -
ECG recording date	[ ]
ECG recording time	[ ]
ECG ID	[ ]
Assessment	☐ normal<br>☐ abnormal<br>☐ not evaluable<br>☐ -
If assessment is normal, please specify:	☐ typical<br>☐ variation<br>☐ -
Supraventricular arrhythmia	☐ yes<br>☐ no<br>☐ -
Ventricular arrhythmia	☐ yes<br>☐ no<br>☐ -
AV block	☐ no<br>☐ first degree<br>☐ second degree Type Mobitz I (Wenckebach)<br>☐ second degree Type Mobitz II<br>☐ third degree<br>☐ -
Left bundle branch block	☐ no<br>☐ incomplete<br>☐ complete<br>☐ -
Right bundle branch block	☐ no<br>☐ incomplete<br>☐ complete<br>☐ -
ST elevation	☐ yes<br>☐ no<br>☐ -
- V1	☐ yes

	☐ no<br>☐ -
- V2	☐ yes<br>☐ no<br>☐ -
- V3	☐ yes<br>☐ no<br>☐ -
- V4	☐ yes<br>☐ no<br>☐ -
- V5	☐ yes<br>☐ no<br>☐ -
- V6	☐ yes<br>☐ no<br>☐ -
- I	☐ yes<br>☐ no<br>☐ -
- II	☐ yes<br>☐ no<br>☐ -
- III	☐ yes<br>☐ no<br>☐ -
- aVR	☐ yes<br>☐ no<br>☐ -
- aVL	☐ yes<br>☐ no<br>☐ -
- aVF	☐ yes<br>☐ no<br>☐ -
ST depression	☐ yes<br>☐ no<br>☐ -
- V1	☐ yes<br>☐ no<br>☐ -
- V2	☐ yes

	☐ no<br>☐ -
- V3	☐ yes<br>☐ no<br>☐ -
- V4	☐ yes<br>☐ no<br>☐ -
- V5	☐ yes<br>☐ no<br>☐ -
- V6	☐ yes<br>☐ no<br>☐ -
- I	☐ yes<br>☐ no<br>☐ -
- II	☐ yes<br>☐ no<br>☐ -
- III	☐ yes<br>☐ no<br>☐ -
- aVR	☐ yes<br>☐ no<br>☐ -
- aVL	☐ yes<br>☐ no<br>☐ -
- aVF	☐ yes<br>☐ no<br>☐ -
T inversion - pathological	☐ yes<br>☐ no<br>☐ -
- V1	☐ yes<br>☐ no<br>☐ -
- V2	☐ yes<br>☐ no<br>☐ -
- V3	☐ yes

	☐ no<br>☐ -
- V4	☐ yes<br>☐ no<br>☐ -
- V5	☐ yes<br>☐ no<br>☐ -
- V6	☐ yes<br>☐ no<br>☐ -
- I	☐ yes<br>☐ no<br>☐ -
- II	☐ yes<br>☐ no<br>☐ -
- III	☐ yes<br>☐ no<br>☐ -
- aVR	☐ yes<br>☐ no<br>☐ -
- aVL	☐ yes<br>☐ no<br>☐ -
- aVF	☐ yes<br>☐ no<br>☐ -
QTc prolongation	☐ yes<br>☐ no<br>☐ -
Low voltage	☐ yes<br>☐ no<br>☐ -
Other	☐ yes<br>☐ no<br>☐ -
Other specify	[ ]
Detailed description	[ ]
Is the ECG change clinically significant?	☐ yes<br>☐ no

	☐ -
Please specify why abnormal ECG is not clinically significant	[ ]
Does the ECG change meet the case definition for Myocarditis/Pericarditis?	☐ yes<br>☐ no<br>☐ -
Yes --->	☐ possible<br>☐ probable<br>☐ confirmed<br>☐ -

Form: Clinical Laboratory

**Urine Pregnancy Test**	
Sample taken	☐ yes<br>☐ no<br>☐ -
If no, please specify reason	[ ]
Sampling date	[ ]
Sampling time	[ ]
Result of pregnancy test	☐ positive<br>☐ negative<br>☐ -

**Blood Analysis**	
Safety laboratory sample taken (15ml)	☐ yes<br>☐ no<br>☐ not required, as not clinically indicated<br>☐ -
Safety laboratory sample, if not taken please specify reason	[ ]
Antibody analysis sample taken (8ml)	☐ yes<br>☐ no<br>☐ -
Antibody analysis sample, if not taken please specify reason	[ ]
Sampling date	[ ]
Sampling time	[ ]
Laboratory order number	[ ]

Form: Diary Collection + Review of AE's + Concomitant Medication + General Comment		
**Diary Card Collection**		
Diary card collected?	☐ yes<br>☐ no<br>☐ -	
Please provide reason why diary could not be collected:	[ ]	
**Review and General Comment**		
General comment	[ ]	

Visit 3

Form: Randomisation + Visit Date + Check of Criteria		
**Confirmation of vaccination visit**		
Has the vaccination visit been confirmed with the subject?	☐ yes<br>☐ no<br>☐ -	
**Planned visit date**		
Planned visit date	[ ]	
**Assignment of vial number**		
Assigned vial number:	[ ]	
Please confirm, that the vial number has been added to the list of vials to be taken out of the freezer by re-entering the vial number	[ ]	
**Visit Date**		
Visit Date	[ ]	
The next visit should be scheduled between the	[ ]	
and the	[ ]	
**Recheck of Criteria**		
Is the subject still eligible for the study after rechecking the inclusion, exclusion and elimination criteria?	☐ yes<br>☐ no	

Form: Vital Signs + Physical Examination		
**Evaluation of Vital Signs**		
Systolic blood pressure	[ ]	mmHg
Diastolic blood pressure	[ ]	mmHg
Pulse rate	[ ]	bpm
Oral temperature	[ ]	°C (format: _ _ . _)

**Physical Examination - Changes since last examination?**		
Weight	[ ]	kg (format: _ _ _ . _)
General appearance	☐ yes<br>☐ no<br>☐ not done<br>☐ -	
Throat-Nose-Ears	☐ yes<br>☐ no<br>☐ not done<br>☐ -	
Lung (incl. auscultation)	☐ yes<br>☐ no<br>☐ not done<br>☐ -	
Heart (incl. auscultation)	☐ yes<br>☐ no<br>☐ not done<br>☐ -	
Abdomen general	☐ yes<br>☐ no<br>☐ not done<br>☐ -	
Lymph nodes (axillar/supraclavicular)	☐ yes<br>☐ no<br>☐ not done<br>☐ -	
Short neurological status	☐ yes<br>☐ no<br>☐ not done<br>☐ -	
Other	☐ yes<br>☐ no<br>☐ not done<br>☐ -	
If Other=Yes, please specify category:	[ ]	

Form: Visit Date + Vital Signs + Physical Examination

**Visit Date**		
Visit Date	[ ]	
The next visit should be scheduled between the	[ ]	
and the	[ ]	

**Evaluation of Vital Signs**		
Systolic blood pressure	[ ]	mmHg
Diastolic blood pressure	[ ]	mmHg
Pulse rate	[ ]	bpm
Oral temperature	[ ]	°C (format: _ _ . _)

**Physical Examination - Changes since last examination?**		
Weight	[ ]	kg (format: _ _ _ . _)
General appearance	☐ yes<br>☐ no<br>☐ not done<br>☐ -	
Throat-Nose-Ears	☐ yes<br>☐ no<br>☐ not done<br>☐ -	
Lung (incl. auscultation)	☐ yes<br>☐ no<br>☐ not done<br>☐ -	
Heart (incl. auscultation)	☐ yes<br>☐ no<br>☐ not done<br>☐ -	
Abdomen general	☐ yes<br>☐ no<br>☐ not done<br>☐ -	
Lymph nodes (axillar/supraclavicular)	☐ yes<br>☐ no<br>☐ not done<br>☐ -	
Short neurological status	☐ yes<br>☐ no<br>☐ not done<br>☐ -	

Other	☐ yes<br>☐ no<br>☐ not done<br>☐ -	
If Other=Yes, please specify category:	[ ]	

Form: ECG	
**ECG**	
ECG assessment performed by	☐ central cardiologist<br>☐ local cardiologist<br>☐ not required, as not clinically indicated<br>☐ not done<br>☐ -
ECG recording date	[ ]
ECG recording time	[ ]
ECG ID	[ ]
Assessment	☐ normal<br>☐ abnormal<br>☐ not evaluable<br>☐ -
If assessment is normal, please specify:	☐ typical<br>☐ variation<br>☐ -
Supraventricular arrhythmia	☐ yes<br>☐ no<br>☐ -
Ventricular arrhythmia	☐ yes<br>☐ no<br>☐ -
AV block	☐ no<br>☐ first degree<br>☐ second degree Type Mobitz I (Wenckebach)<br>☐ second degree Type Mobitz II<br>☐ third degree<br>☐ -
Left bundle branch block	☐ no<br>☐ incomplete<br>☐ complete<br>☐ -
Right bundle branch block	☐ no<br>☐ incomplete<br>☐ complete<br>☐ -
ST elevation	☐ yes<br>☐ no<br>☐ -
- V1	☐ yes

	☐ no<br>☐ -
- V2	☐ yes<br>☐ no<br>☐ -
- V3	☐ yes<br>☐ no<br>☐ -
- V4	☐ yes<br>☐ no<br>☐ -
- V5	☐ yes<br>☐ no<br>☐ -
- V6	☐ yes<br>☐ no<br>☐ -
- I	☐ yes<br>☐ no<br>☐ -
- II	☐ yes<br>☐ no<br>☐ -
- III	☐ yes<br>☐ no<br>☐ -
- aVR	☐ yes<br>☐ no<br>☐ -
- aVL	☐ yes<br>☐ no<br>☐ -
- aVF	☐ yes<br>☐ no<br>☐ -
ST depression	☐ yes<br>☐ no<br>☐ -
- V1	☐ yes<br>☐ no<br>☐ -
- V2	☐ yes

	☐ no<br>☐ -
- V3	☐ yes<br>☐ no<br>☐ -
- V4	☐ yes<br>☐ no<br>☐ -
- V5	☐ yes<br>☐ no<br>☐ -
- V6	☐ yes<br>☐ no<br>☐ -
- I	☐ yes<br>☐ no<br>☐ -
- II	☐ yes<br>☐ no<br>☐ -
- III	☐ yes<br>☐ no<br>☐ -
- aVR	☐ yes<br>☐ no<br>☐ -
- aVL	☐ yes<br>☐ no<br>☐ -
- aVF	☐ yes<br>☐ no<br>☐ -
T inversion - pathological	☐ yes<br>☐ no<br>☐ -
- V1	☐ yes<br>☐ no<br>☐ -
- V2	☐ yes<br>☐ no<br>☐ -
- V3	☐ yes

	☐ no<br>☐ -
- V4	☐ yes<br>☐ no<br>☐ -
- V5	☐ yes<br>☐ no<br>☐ -
- V6	☐ yes<br>☐ no<br>☐ -
- I	☐ yes<br>☐ no<br>☐ -
- II	☐ yes<br>☐ no<br>☐ -
- III	☐ yes<br>☐ no<br>☐ -
- aVR	☐ yes<br>☐ no<br>☐ -
- aVL	☐ yes<br>☐ no<br>☐ -
- aVF	☐ yes<br>☐ no<br>☐ -
QTc prolongation	☐ yes<br>☐ no<br>☐ -
Low voltage	☐ yes<br>☐ no<br>☐ -
Other	☐ yes<br>☐ no<br>☐ -
Other specify	[ ]
Detailed description	[ ]
Is the ECG change clinically significant?	☐ yes<br>☐ no

	☐ -
Please specify why abnormal ECG is not clinically significant	[ ]
Does the ECG change meet the case definition for Myocarditis/Pericarditis?	☐ yes<br>☐ no<br>☐ -
Yes --->	☐ possible<br>☐ probable<br>☐ confirmed<br>☐ -

Form: Clinical Laboratory

**Urine Pregnancy Test**	
Sample taken	☐ yes<br>☐ no<br>☐ -
If no, please specify reason	[ ]
Sampling date	[ ]
Sampling time	[ ]
Result of pregnancy test	☐ positive<br>☐ negative<br>☐ -

**Blood Analysis**	
Safety laboratory sample taken (15ml)	☐ yes<br>☐ no<br>☐ not required, as not clinically indicated<br>☐ -
Safety laboratory sample, if not taken please specify reason	[ ]
Antibody analysis sample taken (8ml)	☐ yes<br>☐ no<br>☐ -
Antibody analysis sample, if not taken please specify reason	[ ]
Sampling date	[ ]
Sampling time	[ ]
Laboratory order number	[ ]

Form: Vaccination + Diary Hand-out + Review AE + Concomitant Medication + General Comment

**Vaccination (subcutaneous)**	
Date of vaccination	[ ]
Time of vaccination	[ ]
Please re-enter the vial number of the vial administered!	[ ]
Arm used (non-dominant upper arm)	☐ left upper arm, deltoid region<br>☐ right upper arm, deltoid region<br>☐ -
Route of administration	☐ subcutaneous<br>☐ other<br>☐ -
Route of administration, if other please specify:	[ ]
Was there any deviation from protocol-defined vaccine preparation and administration?	☐ yes<br>☐ no<br>☐ -
If yes, please specify:	[ ]
**Diary Card Hand-Out**	
Diary card handed out?	☐ yes<br>☐ no<br>☐ -
Please provide reason why diary has not been handed-out:	[ ]
**Review and General Comment**	
General comment	[ ]

Form: Review of AE's + Concomitant Medication + General Comment		
**Review and General Comment**		
General comment	[ ]	

Visit 4

Form: Visit Date + Vital Signs + Physical Examination			
**Visit Date**			
Visit Date	[ ]		
The next visit should be scheduled between the	[ ]		
and the	[ ]		
**Evaluation of Vital Signs**			
Systolic blood pressure	[ ]	mmHg	
Diastolic blood pressure	[ ]	mmHg	
Pulse rate	[ ]	bpm	
Oral temperature	[ ]	°C (format: _ _ . _)	
**Physical Examination - Changes since last examination?**			
Weight	[ ]	kg (format: _ _ _ . _)	
General appearance	☐ yes<br>☐ no<br>☐ not done<br>☐ -		
Throat-Nose-Ears	☐ yes<br>☐ no<br>☐ not done<br>☐ -		
Lung (incl. auscultation)	☐ yes<br>☐ no<br>☐ not done<br>☐ -		
Heart (incl. auscultation)	☐ yes<br>☐ no<br>☐ not done<br>☐ -		
Abdomen general	☐ yes<br>☐ no<br>☐ not done<br>☐ -		

Lymph nodes (axillar/supraclavicular)	☐ yes<br>☐ no<br>☐ not done<br>☐ -	
Short neurological status	☐ yes<br>☐ no<br>☐ not done<br>☐ -	
Other	☐ yes<br>☐ no<br>☐ not done<br>☐ -	
If Other=Yes, please specify category:	[ ]	

Form: ECG	
**ECG**	
ECG assessment performed by	☐ central cardiologist<br>☐ local cardiologist<br>☐ not required, as not clinically indicated<br>☐ not done<br>☐ -
ECG recording date	[ ]
ECG recording time	[ ]
ECG ID	[ ]
Assessment	☐ normal<br>☐ abnormal<br>☐ not evaluable<br>☐ -
If assessment is normal, please specify:	☐ typical<br>☐ variation<br>☐ -
Supraventricular arrhythmia	☐ yes<br>☐ no<br>☐ -
Ventricular arrhythmia	☐ yes<br>☐ no<br>☐ -
AV block	☐ no<br>☐ first degree<br>☐ second degree Type Mobitz I (Wenckebach)<br>☐ second degree Type Mobitz II<br>☐ third degree<br>☐ -
Left bundle branch block	☐ no<br>☐ incomplete<br>☐ complete<br>☐ -
Right bundle branch block	☐ no<br>☐ incomplete<br>☐ complete<br>☐ -
ST elevation	☐ yes<br>☐ no<br>☐ -
- V1	☐ yes

	☐ no<br>☐ -
- V2	☐ yes<br>☐ no<br>☐ -
- V3	☐ yes<br>☐ no<br>☐ -
- V4	☐ yes<br>☐ no<br>☐ -
- V5	☐ yes<br>☐ no<br>☐ -
- V6	☐ yes<br>☐ no<br>☐ -
- I	☐ yes<br>☐ no<br>☐ -
- II	☐ yes<br>☐ no<br>☐ -
- III	☐ yes<br>☐ no<br>☐ -
- aVR	☐ yes<br>☐ no<br>☐ -
- aVL	☐ yes<br>☐ no<br>☐ -
- aVF	☐ yes<br>☐ no<br>☐ -
ST depression	☐ yes<br>☐ no<br>☐ -
- V1	☐ yes<br>☐ no<br>☐ -
- V2	☐ yes

	☐ no<br>☐ -
- V3	☐ yes<br>☐ no<br>☐ -
- V4	☐ yes<br>☐ no<br>☐ -
- V5	☐ yes<br>☐ no<br>☐ -
- V6	☐ yes<br>☐ no<br>☐ -
- I	☐ yes<br>☐ no<br>☐ -
- II	☐ yes<br>☐ no<br>☐ -
- III	☐ yes<br>☐ no<br>☐ -
- aVR	☐ yes<br>☐ no<br>☐ -
- aVL	☐ yes<br>☐ no<br>☐ -
- aVF	☐ yes<br>☐ no<br>☐ -
T inversion - pathological	☐ yes<br>☐ no<br>☐ -
- V1	☐ yes<br>☐ no<br>☐ -
- V2	☐ yes<br>☐ no<br>☐ -
- V3	☐ yes

	☐ no<br>☐ -
- V4	☐ yes<br>☐ no<br>☐ -
- V5	☐ yes<br>☐ no<br>☐ -
- V6	☐ yes<br>☐ no<br>☐ -
- I	☐ yes<br>☐ no<br>☐ -
- II	☐ yes<br>☐ no<br>☐ -
- III	☐ yes<br>☐ no<br>☐ -
- aVR	☐ yes<br>☐ no<br>☐ -
- aVL	☐ yes<br>☐ no<br>☐ -
- aVF	☐ yes<br>☐ no<br>☐ -
QTc prolongation	☐ yes<br>☐ no<br>☐ -
Low voltage	☐ yes<br>☐ no<br>☐ -
Other	☐ yes<br>☐ no<br>☐ -
Other specify	[ ]
Detailed description	[ ]
Is the ECG change clinically significant?	☐ yes<br>☐ no

	☐ -
Please specify why abnormal ECG is not clinically significant	[ ]
Does the ECG change meet the case definition for Myocarditis/Pericarditis?	☐ yes<br>☐ no<br>☐ -
Yes --->	☐ possible<br>☐ probable<br>☐ confirmed<br>☐ -

Form: Clinical Laboratory

**Urine Pregnancy Test**	
Sample taken	☐ yes<br>☐ no<br>☐ -
If no, please specify reason	[ ]
Sampling date	[ ]
Sampling time	[ ]
Result of pregnancy test	☐ positive<br>☐ negative<br>☐ -

**Blood Analysis**	
Safety laboratory sample taken (15ml)	☐ yes<br>☐ no<br>☐ not required, as not clinically indicated<br>☐ -
Safety laboratory sample, if not taken please specify reason	[ ]
Antibody analysis sample taken (8ml)	☐ yes<br>☐ no<br>☐ -
Antibody analysis sample, if not taken please specify reason	[ ]
Sampling date	[ ]
Sampling time	[ ]
Laboratory order number	[ ]

Form: Diary Collection + Review of AE's + Concomitant Medication + General Comment		
**Diary Card Collection**		
Diary card collected?	☐ yes<br>☐ no<br>☐ -	
Please provide reason why diary could not be collected:	[ ]	
**Review and General Comment**		
General comment	[ ]	

Visit 5

Form: Visit Date + Vital Signs + Physical Examination		
**Visit Date**		
Visit Date	[ ]	
The next visit should be scheduled between the	[ ]	
and the	[ ]	
**Evaluation of Vital Signs**		
Systolic blood pressure	[ ]	mmHg
Diastolic blood pressure	[ ]	mmHg
Pulse rate	[ ]	bpm
Oral temperature	[ ]	°C (format: _ _ . _)
**Physical Examination - Changes since last examination?**		
Weight	[ ]	kg (format: _ _ _ . _)
General appearance	☐ yes<br>☐ no<br>☐ not done<br>☐ -	
Throat-Nose-Ears	☐ yes<br>☐ no<br>☐ not done<br>☐ -	
Lung (incl. auscultation)	☐ yes<br>☐ no<br>☐ not done<br>☐ -	
Heart (incl. auscultation)	☐ yes<br>☐ no<br>☐ not done<br>☐ -	
Abdomen general	☐ yes<br>☐ no<br>☐ not done<br>☐ -	

Lymph nodes (axillar/supraclavicular)	☐ yes<br>☐ no<br>☐ not done<br>☐ -	
Short neurological status	☐ yes<br>☐ no<br>☐ not done<br>☐ -	
Other	☐ yes<br>☐ no<br>☐ not done<br>☐ -	
If Other=Yes, please specify category:	[ ]	

Form: ECG

ECG	
ECG assessment performed by	☐ central cardiologist<br>☐ local cardiologist<br>☐ not required, as not clinically indicated<br>☐ not done<br>☐ -
ECG recording date	[ ]
ECG recording time	[ ]
ECG ID	[ ]
Assessment	☐ normal<br>☐ abnormal<br>☐ not evaluable<br>☐ -
If assessment is normal, please specify:	☐ typical<br>☐ variation<br>☐ -
Supraventricular arrhythmia	☐ yes<br>☐ no<br>☐ -
Ventricular arrhythmia	☐ yes<br>☐ no<br>☐ -
AV block	☐ no<br>☐ first degree<br>☐ second degree Type Mobitz I (Wenckebach)<br>☐ second degree Type Mobitz II<br>☐ third degree<br>☐ -
Left bundle branch block	☐ no<br>☐ incomplete<br>☐ complete<br>☐ -
Right bundle branch block	☐ no<br>☐ incomplete<br>☐ complete<br>☐ -
ST elevation	☐ yes<br>☐ no<br>☐ -
- V1	☐ yes

	☐ no<br>☐ -
- V2	☐ yes<br>☐ no<br>☐ -
- V3	☐ yes<br>☐ no<br>☐ -
- V4	☐ yes<br>☐ no<br>☐ -
- V5	☐ yes<br>☐ no<br>☐ -
- V6	☐ yes<br>☐ no<br>☐ -
- I	☐ yes<br>☐ no<br>☐ -
- II	☐ yes<br>☐ no<br>☐ -
- III	☐ yes<br>☐ no<br>☐ -
- aVR	☐ yes<br>☐ no<br>☐ -
- aVL	☐ yes<br>☐ no<br>☐ -
- aVF	☐ yes<br>☐ no<br>☐ -
ST depression	☐ yes<br>☐ no<br>☐ -
- V1	☐ yes<br>☐ no<br>☐ -
- V2	☐ yes

	☐ no<br>☐ -
- V3	☐ yes<br>☐ no<br>☐ -
- V4	☐ yes<br>☐ no<br>☐ -
- V5	☐ yes<br>☐ no<br>☐ -
- V6	☐ yes<br>☐ no<br>☐ -
- I	☐ yes<br>☐ no<br>☐ -
- II	☐ yes<br>☐ no<br>☐ -
- III	☐ yes<br>☐ no<br>☐ -
- aVR	☐ yes<br>☐ no<br>☐ -
- aVL	☐ yes<br>☐ no<br>☐ -
- aVF	☐ yes<br>☐ no<br>☐ -
T inversion - pathological	☐ yes<br>☐ no<br>☐ -
- V1	☐ yes<br>☐ no<br>☐ -
- V2	☐ yes<br>☐ no<br>☐ -
- V3	☐ yes

	☐ no<br>☐ -
- V4	☐ yes<br>☐ no<br>☐ -
- V5	☐ yes<br>☐ no<br>☐ -
- V6	☐ yes<br>☐ no<br>☐ -
- I	☐ yes<br>☐ no<br>☐ -
- II	☐ yes<br>☐ no<br>☐ -
- III	☐ yes<br>☐ no<br>☐ -
- aVR	☐ yes<br>☐ no<br>☐ -
- aVL	☐ yes<br>☐ no<br>☐ -
- aVF	☐ yes<br>☐ no<br>☐ -
QTc prolongation	☐ yes<br>☐ no<br>☐ -
Low voltage	☐ yes<br>☐ no<br>☐ -
Other	☐ yes<br>☐ no<br>☐ -
Other specify	[ ]
Detailed description	[ ]
Is the ECG change clinically significant?	☐ yes<br>☐ no

	□ -
Please specify why abnormal ECG is not clinically significant	[ ]
Does the ECG change meet the case definition for Myocarditis/Pericarditis?	□ yes<br>□ no<br>□ -
Yes --->	□ possible<br>□ probable<br>□ confirmed<br>□ -

Form: Clinical Laboratory

**Urine Pregnancy Test**	
Sample taken	☐ yes<br>☐ no<br>☐ -
If no, please specify reason	[ ]
Sampling date	[ ]
Sampling time	[ ]
Result of pregnancy test	☐ positive<br>☐ negative<br>☐ -

**Blood Analysis**	
Safety laboratory sample taken (15ml)	☐ yes<br>☐ no<br>☐ not required, as not clinically indicated<br>☐ -
Safety laboratory sample, if not taken please specify reason	[ ]
Antibody analysis sample taken (8ml)	☐ yes<br>☐ no<br>☐ -
Antibody analysis sample, if not taken please specify reason	[ ]
Sampling date	[ ]
Sampling time	[ ]
Laboratory order number	[ ]

Form: Review of AE's + Concomitant Medication + General Comment		
**Review and General Comment**		
General comment	[ ]	

Follow-Up

Form: Visit Date + Vital Signs + Physical Examination		
**Visit Date**		
Visit Date	[ ]	
The next visit should be scheduled between the	[ ]	
and the	[ ]	
**Evaluation of Vital Signs**		
Systolic blood pressure	[ ]	mmHg
Diastolic blood pressure	[ ]	mmHg
Pulse rate	[ ]	bpm
Oral temperature	[ ]	°C (format: _ _ . _)
**Physical Examination - Changes since last examination?**		
Weight	[ ]	kg (format: _ _ _ . _)
General appearance	☐ yes<br>☐ no<br>☐ not done<br>☐ -	
Throat-Nose-Ears	☐ yes<br>☐ no<br>☐ not done<br>☐ -	
Lung (incl. auscultation)	☐ yes<br>☐ no<br>☐ not done<br>☐ -	
Heart (incl. auscultation)	☐ yes<br>☐ no<br>☐ not done<br>☐ -	
Abdomen general	☐ yes<br>☐ no<br>☐ not done<br>☐ -	

Lymph nodes (axillar/supraclavicular)	☐ yes<br>☐ no<br>☐ not done<br>☐ -	
Short neurological status	☐ yes<br>☐ no<br>☐ not done<br>☐ -	
Other	☐ yes<br>☐ no<br>☐ not done<br>☐ -	
If Other=Yes, please specify category:	[ ]	

Form: ECG

**ECG**	
ECG assessment performed by	☐ central cardiologist<br>☐ local cardiologist<br>☐ not required, as not clinically indicated<br>☐ not done<br>☐ -
ECG recording date	[ ]
ECG recording time	[ ]
ECG ID	[ ]
Assessment	☐ normal<br>☐ abnormal<br>☐ not evaluable<br>☐ -
If assessment is normal, please specify:	☐ typical<br>☐ variation<br>☐ -
Supraventricular arrhythmia	☐ yes<br>☐ no<br>☐ -
Ventricular arrhythmia	☐ yes<br>☐ no<br>☐ -
AV block	☐ no<br>☐ first degree<br>☐ second degree Type Mobitz I (Wenckebach)<br>☐ second degree Type Mobitz II<br>☐ third degree<br>☐ -
Left bundle branch block	☐ no<br>☐ incomplete<br>☐ complete<br>☐ -
Right bundle branch block	☐ no<br>☐ incomplete<br>☐ complete<br>☐ -
ST elevation	☐ yes<br>☐ no<br>☐ -
- V1	☐ yes

	☐ no<br>☐ -
- V2	☐ yes<br>☐ no<br>☐ -
- V3	☐ yes<br>☐ no<br>☐ -
- V4	☐ yes<br>☐ no<br>☐ -
- V5	☐ yes<br>☐ no<br>☐ -
- V6	☐ yes<br>☐ no<br>☐ -
- I	☐ yes<br>☐ no<br>☐ -
- II	☐ yes<br>☐ no<br>☐ -
- III	☐ yes<br>☐ no<br>☐ -
- aVR	☐ yes<br>☐ no<br>☐ -
- aVL	☐ yes<br>☐ no<br>☐ -
- aVF	☐ yes<br>☐ no<br>☐ -
ST depression	☐ yes<br>☐ no<br>☐ -
- V1	☐ yes<br>☐ no<br>☐ -
- V2	☐ yes

	☐ no<br>☐ -
- V3	☐ yes<br>☐ no<br>☐ -
- V4	☐ yes<br>☐ no<br>☐ -
- V5	☐ yes<br>☐ no<br>☐ -
- V6	☐ yes<br>☐ no<br>☐ -
- I	☐ yes<br>☐ no<br>☐ -
- II	☐ yes<br>☐ no<br>☐ -
- III	☐ yes<br>☐ no<br>☐ -
- aVR	☐ yes<br>☐ no<br>☐ -
- aVL	☐ yes<br>☐ no<br>☐ -
- aVF	☐ yes<br>☐ no<br>☐ -
T inversion - pathological	☐ yes<br>☐ no<br>☐ -
- V1	☐ yes<br>☐ no<br>☐ -
- V2	☐ yes<br>☐ no<br>☐ -
- V3	☐ yes

	☐ no<br>☐ -
- V4	☐ yes<br>☐ no<br>☐ -
- V5	☐ yes<br>☐ no<br>☐ -
- V6	☐ yes<br>☐ no<br>☐ -
- I	☐ yes<br>☐ no<br>☐ -
- II	☐ yes<br>☐ no<br>☐ -
- III	☐ yes<br>☐ no<br>☐ -
- aVR	☐ yes<br>☐ no<br>☐ -
- aVL	☐ yes<br>☐ no<br>☐ -
- aVF	☐ yes<br>☐ no<br>☐ -
QTc prolongation	☐ yes<br>☐ no<br>☐ -
Low voltage	☐ yes<br>☐ no<br>☐ -
Other	☐ yes<br>☐ no<br>☐ -
Other specify	[ ]
Detailed description	[ ]
Is the ECG change clinically significant?	☐ yes<br>☐ no

	☐ -
Please specify why abnormal ECG is not clinically significant	[ ]
Does the ECG change meet the case definition for Myocarditis/Pericarditis?	☐ yes<br>☐ no<br>☐ -
Yes --->	☐ possible<br>☐ probable<br>☐ confirmed<br>☐ -

Form: Clinical Laboratory

**Urine Pregnancy Test**	
Sample taken	☐ yes<br>☐ no<br>☐ -
If no, please specify reason	[ ]
Sampling date	[ ]
Sampling time	[ ]
Result of pregnancy test	☐ positive<br>☐ negative<br>☐ -

**Blood Analysis**	
Safety laboratory sample taken (15ml)	☐ yes<br>☐ no<br>☐ not required, as not clinically indicated<br>☐ -
Safety laboratory sample, if not taken please specify reason	[ ]
Antibody analysis sample taken (8ml)	☐ yes<br>☐ no<br>☐ -
Antibody analysis sample, if not taken please specify reason	[ ]
Sampling date	[ ]
Sampling time	[ ]
Laboratory order number	[ ]

Form: Review of SAE's + General Comment		
**Review and General Comment**		
General comment	[ ]	

SAE, SIAE, Unresolved AEs

Form: Serious Adverse Events, Special Interest Adverse Events and Follow-Up of Unresolved Study Adverse Events		
**Description**		
Adverse Event (Diagnosis)	[ ]	
**Start**		
Start date	[ ]	
Or month	☐ 01<br>☐ 02<br>☐ 03<br>☐ 04<br>☐ 05<br>☐ 06<br>☐ 07<br>☐ 08<br>☐ 09<br>☐ 10<br>☐ 11<br>☐ 12<br>☐ unknown<br>☐ -	
and year	☐ 2007<br>☐ 2006<br>☐ 2005<br>☐ 2004<br>☐ 2003<br>☐ 2002<br>☐ 2001<br>☐ 2000<br>☐ 1999<br>☐ 1998<br>☐ 1997<br>☐ 1996<br>☐ 1995<br>☐ 1994<br>☐ 1993<br>☐ 1992	

- ☐ 1991
- ☐ 1990
- ☐ 1989
- ☐ 1988
- ☐ 1987
- ☐ 1986
- ☐ 1985
- ☐ 1984
- ☐ 1983
- ☐ 1982
- ☐ 1981
- ☐ 1980
- ☐ 1979
- ☐ 1978
- ☐ 1977
- ☐ 1976
- ☐ 1975
- ☐ 1974
- ☐ 1973
- ☐ 1972
- ☐ 1971
- ☐ 1970
- ☐ 1969
- ☐ 1968
- ☐ 1967
- ☐ 1966
- ☐ 1965
- ☐ 1964
- ☐ 1963
- ☐ 1962
- ☐ 1961
- ☐ 1960
- ☐ 1959
- ☐ 1958
- ☐ 1957
- ☐ 1956
- ☐ 1955
- ☐ 1954
- ☐ 1953
- ☐ 1952
- ☐ 1951
- ☐ 1950
- ☐ 1949
- ☐ 1948
- ☐ 1947
- ☐ 1946
- ☐ 1945

	☐ 1944<br>☐ 1943<br>☐ 1942<br>☐ 1941<br>☐ unknown<br>☐ -	
Start time	[ ]	
Start time as text	[ ]	

**End**		
End date	[ ]	
Or month	☐ 01<br>☐ 02<br>☐ 03<br>☐ 04<br>☐ 05<br>☐ 06<br>☐ 07<br>☐ 08<br>☐ 09<br>☐ 10<br>☐ 11<br>☐ 12<br>☐ continued<br>☐ unknown<br>☐ -	
and year	☐ 2005<br>☐ 2006<br>☐ 2007<br>☐ continued<br>☐ unknown<br>☐ -	
End time	[ ]	
End time as text	[ ]	

**Details**		
Follow-up to previous event or initial event	☐ follow-up<br>☐ initial<br>☐ unknown<br>☐ -	
Outcome	☐ recovery without sequelae<br>☐ recovery with sequelae<br>☐ ongoing<br>☐ death<br>☐ unknown	

SAE or Special Interest AE (SIAE)	☐ no<br>☐ SAE<br>☐ SIAE<br>☐ unknown<br>☐ -
Frequency	☐ single episode<br>☐ intermittent<br>☐ continuous<br>☐ unknown<br>☐ -
Intensity	☐ easily tolerated by subject<br>☐ sufficiently discomforting to interfere with daily activities<br>☐ prevents daily activities<br>☐ life threatening or disabling<br>☐ unknown<br>☐ -
Relationship to vaccine	☐ none<br>☐ unlikely<br>☐ possible<br>☐ probable<br>☐ definite<br>☐ unknown<br>☐ -
Actions taken	☐ no action<br>☐ study drug dosage adjusted<br>☐ study drug discontinued<br>☐ concomitant medication<br>☐ non-drug therapy<br>☐ hospitalization<br>☐ unknown<br>☐ -

Study Closure

Form: Study Closure Form	
**Study Closure Form**	
Main reason for study termination	☐ the volunteer reached the planned observation time<br>☐ adverse event, making it unsafe for the subject to continue<br>☐ withdrawal of consent<br>☐ subject unwilling/unable to comply with study requirements<br>☐ clinical need for concomitant/ancillary therapy not permitted<br>☐ unrelated intercurrent illness, affecting significantly assessment of clinical status<br>☐ lost to follow-up<br>☐ other<br>☐ -
If other, please specify:	[ ]
Date of withdrawal	[ ]
Date of last dosage	[ ]
General comment	[ ]

Adverse Events

Form: Adverse Events		
**Description**		
Adverse Event (Diagnosis)	[ ]	
**Start**		
Start date	[ ]	
Or month	☐ 01<br>☐ 02<br>☐ 03<br>☐ 04<br>☐ 05<br>☐ 06<br>☐ 07<br>☐ 08<br>☐ 09<br>☐ 10<br>☐ 11<br>☐ 12<br>☐ unknown<br>☐ -	
and year	☐ 2007<br>☐ 2006<br>☐ 2005<br>☐ 2004<br>☐ 2003<br>☐ 2002<br>☐ 2001<br>☐ 2000<br>☐ 1999<br>☐ 1998<br>☐ 1997<br>☐ 1996<br>☐ 1995<br>☐ 1994<br>☐ 1993<br>☐ 1992<br>☐ 1991	

- ☐ 1990
- ☐ 1989
- ☐ 1988
- ☐ 1987
- ☐ 1986
- ☐ 1985
- ☐ 1984
- ☐ 1983
- ☐ 1982
- ☐ 1981
- ☐ 1980
- ☐ 1979
- ☐ 1978
- ☐ 1977
- ☐ 1976
- ☐ 1975
- ☐ 1974
- ☐ 1973
- ☐ 1972
- ☐ 1971
- ☐ 1970
- ☐ 1969
- ☐ 1968
- ☐ 1967
- ☐ 1966
- ☐ 1965
- ☐ 1964
- ☐ 1963
- ☐ 1962
- ☐ 1961
- ☐ 1960
- ☐ 1959
- ☐ 1958
- ☐ 1957
- ☐ 1956
- ☐ 1955
- ☐ 1954
- ☐ 1953
- ☐ 1952
- ☐ 1951
- ☐ 1950
- ☐ 1949
- ☐ 1948
- ☐ 1947
- ☐ 1946
- ☐ 1945
- ☐ 1944

	☐ 1943<br>☐ 1942<br>☐ 1941<br>☐ unknown<br>☐ -
Start time	[ ]
Start time as text	[ ]

**End**	
End date	[ ]
Or month	☐ 01<br>☐ 02<br>☐ 03<br>☐ 04<br>☐ 05<br>☐ 06<br>☐ 07<br>☐ 08<br>☐ 09<br>☐ 10<br>☐ 11<br>☐ 12<br>☐ continued<br>☐ unknown<br>☐ -
and year	☐ 2005<br>☐ 2006<br>☐ 2007<br>☐ continued<br>☐ unknown<br>☐ -
End time	[ ]
End time as text	[ ]

**AE Details**	
Outcome	☐ recovery without sequelae<br>☐ recovery with sequelae<br>☐ ongoing<br>☐ death<br>☐ unknown
SAE or Special Interest AE (SIAE)	☐ no<br>☐ SAE<br>☐ SIAE<br>☐ unknown<br>☐ -
Frequency	☐ single episode

Frequency	☐ single episode<br>☐ intermittent<br>☐ continuous<br>☐ unknown<br>☐ -
Intensity	☐ easily tolerated by subject<br>☐ sufficiently discomforting to interfere with daily activities<br>☐ prevents daily activities<br>☐ life threatening or disabling<br>☐ unknown<br>☐ -
Relationship to vaccine	☐ none<br>☐ unlikely<br>☐ possible<br>☐ probable<br>☐ definite<br>☐ unknown<br>☐ -
Actions taken	☐ no action<br>☐ study drug dosage adjusted<br>☐ study drug discontinued<br>☐ concomitant medication<br>☐ non-drug therapy<br>☐ hospitalization<br>☐ unknown<br>☐ -

Prior/Concomitant Medication

Form: Prior and Concomitant Medication	
**Prior and Concomitant Medication**	
Brand name of medication	[ ]
Route of administration	☐ buccal<br>☐ conjunctival<br>☐ dental<br>☐ epidural<br>☐ implant<br>☐ inhalation<br>☐ intra-arterial<br>☐ intra-articular<br>☐ intra-cardiac<br>☐ intra-dermal<br>☐ intra-muscular<br>☐ intra-nasal<br>☐ intra-peritoneal<br>☐ intra-pleural<br>☐ intra-spinal<br>☐ intra-thecal<br>☐ intra-tracheal<br>☐ intra-uterine<br>☐ intra-venous<br>☐ oral<br>☐ rectal<br>☐ subcutaneous<br>☐ sublingual<br>☐ topical<br>☐ transdermal<br>☐ urethral<br>☐ vaginal<br>☐ other<br>☐ unknown<br>☐ -
Other route of administration	[ ]
Dose	[ ]
Unit	☐ cm<br>☐ drops

	☐ g<br>☐ IU<br>☐ l<br>☐ mcg<br>☐ mcl<br>☐ mcmol<br>☐ mg<br>☐ mg/kg<br>☐ ml<br>☐ mm<br>☐ mmol<br>☐ puffs<br>☐ units<br>☐ tablets<br>☐ other<br>☐ unknown<br>☐ -	
Other unit	[ ]	
Frequency of administration	☐ per day<br>☐ per week<br>☐ per month<br>☐ once<br>☐ PRN (as required)<br>☐ other<br>☐ unknown<br>☐ -	
Other frequency of administration	[ ]	
Indication	[ ]	

**Start date**	

- ☐ 2006
- ☐ 2005
- ☐ 2004
- ☐ 2003
- ☐ 2002
- ☐ 2001
- ☐ 2000
- ☐ 1999
- ☐ 1998
- ☐ 1997
- ☐ 1996
- ☐ 1995
- ☐ 1994
- ☐ 1993
- ☐ 1992
- ☐ 1991
- ☐ 1990
- ☐ 1989
- ☐ 1988
- ☐ 1987
- ☐ 1986
- ☐ 1985
- ☐ 1984
- ☐ 1983
- ☐ 1982
- ☐ 1981
- ☐ 1980
- ☐ 1979
- ☐ 1978
- ☐ 1977
- ☐ 1976
- ☐ 1975
- ☐ 1974
- ☐ 1973
- ☐ 1972
- ☐ 1971
- ☐ 1970
- ☐ 1969
- ☐ 1968
- ☐ 1967
- ☐ 1966
- ☐ 1965
- ☐ 1964
- ☐ 1963
- ☐ 1962
- ☐ 1961
- ☐ 1960

	☐ 1959<br>☐ 1958<br>☐ 1957<br>☐ 1956<br>☐ 1955<br>☐ 1954<br>☐ 1953<br>☐ 1952<br>☐ 1951<br>☐ 1950<br>☐ 1949<br>☐ 1948<br>☐ 1947<br>☐ 1946<br>☐ 1945<br>☐ 1944<br>☐ 1943<br>☐ 1942<br>☐ 1941<br>☐ unknown<br>☐ -
**End date**	
End date	[ ]
Or month	☐ 01<br>☐ 02<br>☐ 03<br>☐ 04<br>☐ 05<br>☐ 06<br>☐ 07<br>☐ 08<br>☐ 09<br>☐ 10<br>☐ 11<br>☐ 12<br>☐ continued<br>☐ unknown<br>☐ -
and year	☐ 2005<br>☐ 2006<br>☐ 2007<br>☐ continued<br>☐ unknown<br>☐ -

Empty CRFs for POX-MVA-005 (HCR: 1029/BN)

BAVARIAN NORDIC

Tagebuch

Probandennummer |_|_|_|

Screeningnummer |_|_|_|_|

Geburtsdatum |_|_| |_|_| |_|_|_|_|

Vertraulich

Studiennummer: POX-MVA-005

☐ **1. Impfung** ☐ **2. Impfung** |_|_| |_|_| |2,0,0|_|
t t mm j j j j

Prüfarzt: ____________________

Liebe Teilnehmerin, lieber Teilnehmer,

Wir freuen uns, dass Sie an unserer klinischen Prüfung zur Erforschung eines neuen verbesserten Pockenimpfstoffs teilnehmen. Der Studienablauf sieht vor, dass jeder Teilnehmer ein Tagebuch führt. In diesem Tagebuch sollen *lokale und allgemeine Reaktionen auf die Impfung* dokumentiert werden (mögliche Symptome sind z. B. Schmerzen, Rötung, Schwellung oder Verhärtung an der Einstichstelle, Kopfschmerzen, Muskelschmerzen, Übelkeit, Ermüdung oder Ähnliches). Bitte tragen Sie alle Symptome ein, die Sie empfinden, auch wenn Sie meinen, diese Symptome seien unwichtig. Zur Bestimmung der Körpertemperatur wird Ihnen ein Thermometer ausgehändigt. Bitte messen Sie die Temperatur immer gegen Abend in der Mundhöhle. Das Thermometer ist rechts oder links unter der Zunge zu platzieren. Die Messdauer sollte 1 Minute betragen, am Ende der Messung ertönt ein Signalton. Wenn Sie mehrere Messungen an einem Tag durchführen, tragen Sie bitte den Höchstwert ein. Durchmesser von Rötungen, Schwellungen oder Verhärtungen sollen mit dem Lineal gemessen werden. Wenn Sie mehrere Messungen an einem Tag durchführen, tragen Sie den Höchstwert ein. Medikamente zur Behandlung der aufgetretenen Symptome müssen ebenfalls an entsprechender Stelle ins Tagebuch eingetragen werden. Füllen Sie das Tagebuch bitte mit einem Kugelschreiber aus. Bleistifteintragungen sind nicht erlaubt. Bitte füllen Sie jedes Feld aus. Wenn das entsprechende Symptom nicht aufgetreten ist, tragen Sie bei der Bewertung eine "0" ein. Korrekturen sind wie folgt vorzunehmen: Der falsche Eintrag wird mit einer einfachen Linie durchgestrichen und die korrekte Information daneben eingetragen. Das ausgefüllte Tagebuch muss zum nächsten Termin beim Prüfarzt mitgebracht werden. Bei diesem Gespräch werden dann die eingetragenen Informationen besprochen.

Nächster Termin beim Prüfarzt: |_|_|.|_|_|.|_|_|_|_|
Tag Monat Jahr

Im Fall von schweren und unerwarteten Reaktionen kontaktieren Sie bitte sofort den zuständigen Prüfarzt (Telefon ____________).

Vielen Dank für Ihre Teilnahme.

Medikamente zur Behandlung der Symptome				
**Name des Medikaments**	**Tägliche Dosis**	**Startdatum**	**Enddatum**	**Grund der Behandlung**
		|_|_||_|_| |2,0,0|_| t t mm j j j j	|_|_||_|_| |2,0,0|_| t t mm j j j j	
		|_|_||_|_| |2,0,0|_| t t mm j j j j	|_|_||_|_| |2,0,0|_| t t mm j j j j	
		|_|_||_|_| |2,0,0|_| t t mm j j j j	|_|_||_|_| |2,0,0|_| t t mm j j j j	
		|_|_||_|_| |2,0,0|_| t t mm j j j j	|_|_||_|_| |2,0,0|_| t t mm j j j j	
		|_|_||_|_| |2,0,0|_| t t mm j j j j	|_|_||_|_| |2,0,0|_| t t mm j j j j	
		|_|_||_|_| |2,0,0|_| t t mm j j j j	|_|_||_|_| |2,0,0|_| t t mm j j j j	
		|_|_||_|_| |2,0,0|_| t t mm j j j j	|_|_||_|_| |2,0,0|_| t t mm j j j j	

Bitte füllen Sie die Tabelle auf dieser Seite aus und beurteilen Sie das Auftreten der folgenden Symptome anhand der aufgelisteten Kriterien.

Lokale Symptome (an der Einstichstelle)	Bewertung	Tag der Impfung	Tag 1	Tag 2	Tag 3	Tag 4	Tag 5	Tag 6	Tag 7	Symptome nach Tag 7: Nach Tag 7 andauernd	Symptome nach Tag 7: Letzter Tag mit Symptomen	Symptome nach Tag 7: Höchste Bewertung	Vom Prüfarzt auszufüllen! Outcome[1]	Vom Prüfarzt auszufüllen! Relationship to vaccine[2]	Vom Prüfarzt auszufüllen! SAE If yes complete SAE Form	Vom Prüfarzt auszufüllen! Actions taken[3]
**Schmerzen**	**0 = Keine Schmerzen**<br>**1 = Schmerz bei Berührung**<br>**2 = Schmerz bei Bewegung des Impfarmes**<br>**3 = Spontan schmerzhaft, verhindert normale Aktivitäten**									☐ nein ☐ ja	tt mm 200_ jjjj			Not applicable	☐ no ☐ yes	
**Rötung**	**Bitte geben Sie den größten Durchmesser an, wenn Sie mehrmals an einem Tag messen.**	mm	mm	mm	mm	mm	mm	mm	mm	☐ nein ☐ ja	tt mm 200_ jjjj	mm			☐ no ☐ yes	
**Schwellung**		mm	mm	mm	mm	mm	mm	mm	mm	☐ nein ☐ ja	tt mm 200_ jjjj	mm			☐ no ☐ yes	
**Verhärtung**		mm	mm	mm	mm	mm	mm	mm	mm	☐ nein ☐ ja	tt mm 200_ jjjj	mm			☐ no ☐ yes	
**Andere (Welche?)** ______	**0 = Keine Symptome**<br>**1 = Leicht:** Tagesablauf nicht beeinträchtigt<br>**2 = Mäßig:** Tagesablauf beeinträchtigt<br>**3 = Stark:** verhindern normalen Tagesablauf									☐ nein ☐ ja	tt mm 200_ jjjj					
______										☐ nein ☐ ja	tt mm 200_ jjjj					
**Allgemeine Symptome**	Bewertung	Tag der Impfung	Tag 1	Tag 2	Tag 3	Tag 4	Tag 5	Tag 6	Tag 7	Nach Tag 7 andauernd	Letzter Tag mit Symptomen	Höchste Bewertung				
**Körpertemperatur (°C)** (Mund)	**Bitte geben Sie die höchste gemessene Temperatur an, wenn Sie mehrmals an einem Tag messen.**	__ __ , __	__ __ , __	__ __ , __	__ __ , __	__ __ , __	__ __ , __	__ __ , __	__ __ , __	☐ nein ☐ ja	tt mm 200_ jjjj	__ __ , __			☐ no ☐ yes	
**Kopfschmerzen**	**0 = Keine Symptome**<br>**1 = Leicht:** Tagesablauf nicht beeinträchtigt<br>**2 = Mäßig:** Tagesablauf beeinträchtigt<br>**3 = Stark:** verhindern normalen Tagesablauf									☐ nein ☐ ja	tt mm 200_ jjjj				☐ no ☐ yes	
**Muskelschmerzen**										☐ nein ☐ ja	tt mm 200_ jjjj				☐ no ☐ yes	
**Übelkeit**										☐ nein ☐ ja	tt mm 200_ jjjj				☐ no ☐ yes	
**Müdigkeit**										☐ nein ☐ ja	tt mm 200_ jjjj				☐ no ☐ yes	
**Andere (Welche?)** ______										☐ nein ☐ ja	tt mm 200_ jjjj					
______										☐ nein ☐ ja	tt mm 200_ jjjj					

[1]: 1 = recovery without sequelae, 2 = recovery with sequelae, 3 = ongoing, 4 = death, 9=unknown

[2]: 1 = none, 2 = unlikely, 3 = possible, 4 = probable, 5 = definite

[3]: 0 = no action, 1 = study drug dosage adjusted, 2 = study drug discontinued, 3 = concomitant medication, 4 = non-drug therapy, 5 = hospitalization

Please transfer all symptoms recorded in the section 'Andere' as 'unsolicited adverse event' into the respective section of the source data and into the eCRF.

16.1.3 Ethical and regulatory issues

List of IECs and authorities consulted or informed:

Ethics committee

Ethikkommission der
Medizinischen Fakultät der LMU München
Marchioninistr. 15
81377 München

Federal Agency for Sera and Vaccines

Paul-Ehrlich-Institut, Bundesamt für Sera und Impfstoffe
Paul-Ehrlich-Straße 51-59
D-63225 Langen
Postfach D-63207 Langen

Local Authority

Regierung von Oberbayern
Sachgebiet 212-Pharmazie
Maximilianstraße 39
80534 München, Germany

Ethikkommission der Medizinischen Fakultät
der Ludwig-Maximilians Universität
Vorsitzender: Prof. Dr. Gustav Paumgartner

Ludwig-
Maximilians-
Universität
München
LMU

Marchioninistr. 15
81377 München
Tel: (089) 7095 4609
Fax: (089) 7095 7609
e-mail: Ethikkommission@med.uni-muenchen.de
17.04.2007 Hb/ cp

Klinikum der Universität München – Großhadern
Marchioninistr. 15 81377 München

Bavarian Nordic GmbH
Frau Nadine Uebler
Fraunhoferstraße 13
82152 Martinsried

EudraCT-Nr. 2005-001781-14
Teilweise randomisierte, teilweise doppelblinde, placebo-kontrollierte Phase II Nichtunterlegenheitsstudie zur Bewertung der Immunogenität und Sicherheit von ein und zwei Dosen eines Pockenimpfstoffes bei 18-55 Jahre alten gesunden Probanden
Projekt-Nr. 365-05

Sehr geehrte Frau Uebler,

besten Dank für Ihr Schreiben vom 02.04.2007 mit dem Amendment 1 zum Prüfplan vom 12.12.2005 zu der o.g. Studie.

Zur Bewertung haben folgende Unterlagen vorgelegen:

- Amendment # 1, 26.03.2007

Vor dem Hintergrund Ihrer Stellungnahme bestehen keine ethisch-rechtlichen Bedenken gegen dieses Amendment, so dass die schon ausgesprochene ethisch-rechtliche Unbedenklichkeit weiter Gültigkeit hat.

Die Stellungnahme zu Amendment 1 erfolgte im Benehmen mit der lokal beteiligten Ethikkommission.

Ich wünsche Ihrer Studie weiterhin einen guten Verlauf.

Mit freundlichen Grüßen

Prof. Dr. G. Paumgartner
Vorsitzender der Ethikkommission

i.A. Dr. B. Henrikus
Geschäftsstelle Ethikkommission

Mitglieder der Kommission:
Prof. Dr. G. Paumgartner (Vorsitzender), Prof. Dr. E. Held (stellv. Vorsitzender), Prof. Dr. H. U. Gallwas,
Prof. Dr. D. Kunze, Dr. V. Mönch, PD Dr. V. Nüßler, Prof. Dr. R. Penning, Prof. Dr. K. Hahn, Prof. Dr. K. Pfeifer, Dr. Ch. Zach

Ethikkommission der Medizinischen Fakultät
der Ludwig-Maximilians Universität
Vorsitzender: Prof. Dr. Gustav Paumgartner

LMU
Ludwig-Maximilians-Universität-München
Marchioninistr. 15
81377 München
Tel: (089) 7095 4609
Fax: (089) 7095 7609
e-mail: Ethikkommission@med.uni-muenchen.de

20.08.2006 Hb/ cp

Klinikum der Universität München – Großhadern
Marchioninistr. 15 81377 München

Bavarian Nordic GmbH
Frau Nadine Uebler
Fraunhoferstraße 13
82152 Martinsried

EudraCT-Nr. 2005-001781-14
Teilweise randomisierte, teilweise doppelblinde, placebo-kontrollierte Phase II Nichtunterlegenheitsstudie zur Bewertung der Immunogenität und Sicherheit von ein und zwei Dosen eines Pockenimpfstoffes bei 18-55 Jahre alten gesunden Probanden
Projekt-Nr. 365-05

Sehr geehrte Frau Uebler,

besten Dank für Ihr Schreiben vom 11.08.2006 mit Unterlagen, mit denen die Rekrutierung von Patienten für die o. g. Studie unterstützt werden soll.

In der Anlage sende ich Ihnen unsere „Hinweise zu rekrutierungsunterstützenden Maßnahmen" und teile Ihnen mit, dass die uns zugesandten Unterlagen nicht im Widerspruch dazu stehen.

Ich wünsche Ihrer Studie weiterhin einen guten Verlauf.

Mit freundlichen Grüßen

i.V. [illegible]
Prof. Dr. G. Paumgartner
Vorsitzender der Ethikkommission

Anlage

Mitglieder der Kommission:
Prof. Dr. G. Paumgartner (Vorsitzender), Prof. Dr. E. Held (stellv. Vorsitzender), Prof. Dr. H. U. Gallwas,
Prof. Dr. D. Kunze, Dr. V. Mönch, PD Dr. V. Nüßler, Prof. Dr. R. Penning, Prof. Dr. K. Hahn, Prof. Dr. K. Pfeifer, Dr. Ch. Zach

FR 3 323/09 Ma

Hinweise zu rekrutierungsunterstützende Maßnahmen

Der Aufgabenbereich der Ethikkommission wird durch die für sie geltenden Rechtsgrundlagen festgelegt. Das sind neben der Geschäfts- und Verfahrensordnung der Ethikkommission das Arzneimittelgesetz bzw. das Medizinprodukte- Gesetz und die EG-GCP. Diese sieht vor, dass die Ethikkommission die Art und Weise, in der die Rekrutierung der Patienten/ Probanden durchgeführt wird, begutachten soll, weil derartigen Werbematerialien im Einzelfall eine ethische und (arzt-) berufsrechtliche Relevanz zukommen könnte. Deshalb gibt die Ethikkommission folgende allgemeine Hinweise zu Inhalten und Verfahren rekrutierungsunterstützender Informationen in allgemein zugänglichen Medien:

- Über das (primär fremd- oder allenfalls gruppennützige) Studienziel „Erkenntnisgewinn" (sei es hinsichtlich Sicherheit, Wirksamkeit, geeigneter Dosis o.ä.) darf nicht mit Informationen über den erwarteten, aber noch nicht belegten persönlichen „Gewinn" aus der Perspektive des Studienteilnehmers hinweg getäuscht werden, zumal, wenn nicht alle Studienteilnehmer diese Behandlung erhalten, sondern die Studie einen Placeboarm hat.

  Im Hinblick auf die teilnehmenden Prüfärzte ist in diesem Zusammenhang auf das Schutzgut der vertrauensvollen Arzt- Patientenbeziehung in der Berufsordnung hinzuweisen, dessen Sicherung unter anderem die Bestimmungen des §11 Abs. 2 der Berufsordnung für die Ärzte Bayerns dient:

  Der ärztliche Berufsauftrag verbietet es, diagnostische oder therapeutische Methoden unter missbräuchlicher Ausnutzung des Vertrauens, der Unwissenheit, der Leichtgläubigkeit oder der Hilflosigkeit von Patienten anzuwenden. Unzulässig ist es auch, Heilerfolge, insbesondere bei nicht heilbaren Krankheiten, als gewiss zuzusichern.

- Die werbende Aussage, der Studienteilnehmer habe den Vorteil intensiverer ärztlicher Betreuung, findet zum einen eine berufsrechtliche Grenze der Zulässigkeit in einer anpreisenden Werbung mit Selbstverständlichkeiten, die für den Arzt, dem sie zugute kommt, als berufswidrig im Sinne des §27 Abs. 1 der Berufsordnung für die Ärzte Bayerns anzusehen ist und zum anderen in der gebotenen Ausgewogenheit der Aussage im Hinblick auf die zusätzlichen Belastungen, die der Patient studienhalber auf sich nimmt.

- Personenbezogene Daten des potentiell teilnahmewilligen Patienten /Probanden sollten nicht vom Sponsor oder von ihm beauftragten Instituten abgefragt werden, sondern nur dem vom Patienten /Probanden ggf. gewählten Prüfarzt zugänglich sein.

- Im Hinblick auf das für den Arzt geltende berufsrechtliche Verbot, berufswidrige Werbung zu dulden, sollte möglichst darauf verzichtet werden, die teilnehmenden Prüfärzte unmittelbar zu veröffentlichen. Es erscheint adäquater, wenn dem Anfragenden, potentiell teilnahmewilligen Patienten /Probanden vom Sponsor bzw. einem von ihm beauftragten Institut eine Liste der teilnehmenden Prüfärzte zur Verfügung gestellt wird.

Ethikkommission der Medizinischen Fakultät
der Ludwig-Maximilians Universität
Vorsitzender: Prof. Dr. Gustav Paumgartner

Ludwig-
Maximilians-
Universität
München
Marchioninistr. 15
81377 München
Tel: (089) 7095 4609
Fax: (089) 7095 7609
e-mail: Ethikkommission@
med.uni-muenchen.de
17.05.2006

Klinikum der Universität München – Großhadern
Marchioninistr. 15 81377 München

Bavarian Nordic GmbH
Herrn Dr. J. Vollmar
Fraunhoferstraße 13
82152 Martinsried

EudraCT-Nr. 2005-001781-14
Teilweise randomisierte, teilweise doppelblinde, placebo-kontrollierte Phase II Nichtunterlegenheitsstudie zur Bewertung der Immunogenität und Sicherheit von ein und zwei Dosen eines Pockenimpfstoffes bei 18-55 Jahre alten gesunden Probanden
Projekt-Nr. 365-05

Sehr geehrter Herr Dr. Vollmar,
sehr geehrte Frau Uebler,

besten Dank für Ihr Schreiben vom 03.03.2006 mit einer überarbeiteten Version der Patienteninformation zu der o.g. Studie. Dabei lagen im Einzelnen zur Begutachtung vor:

- Probandeninformation und Einverständniserklärung Version 5.0 05 May 2006
- Werbebrief zur Patientenrekrutierung Apr 2006.

Es bestehen keine ethisch-rechtlichen Bedenken gegen die Verwendung dieser Patienteninformation und des Informationsbriefes zur Studie 1046/BN, so dass die schon ausgesprochene ethisch-rechtliche Unbedenklichkeit weiter Gültigkeit hat.

Im Zusammenhang mit der Bewertung des „Werbebriefs" beachten Sie bitte unsere „Hinweise zu rekrutierungsunterstützenden Maßnahmen".

Ich wünsche Ihrer Studie weiterhin einen guten Verlauf.

Mit freundlichen Grüßen

Prof. Dr. G. Paumgartner
Vorsitzender der Ethikkommission

Mitglieder der Kommission:
Prof. Dr. G. Paumgartner (Vorsitzender), Prof. Dr. E. Held (stellv. Vorsitzender), Prof. Dr. H. U. Gallwas,
Prof. Dr. D. Kunze, Dr. V. Mönch, PD Dr. V. Nüßler, Prof. Dr. R. Penning, Prof. Dr. K. Hahn, Prof. Dr. K. Pfeifer, Dr. Ch. Zach

FR 3 325/00 Mä.

Hinweise zu rekrutierungsunterstützende Maßnahmen

Der Aufgabenbereich der Ethikkommission wird durch die für sie geltenden Rechtsgrundlagen festgelegt. Das sind neben der Geschäfts- und Verfahrensordnung der Ethikkommission das Arzneimittelgesetz bzw. das Medizinprodukte- Gesetz und die EG-GCP. Diese sieht vor, dass die Ethikkommission die Art und Weise, in der die Rekrutierung der Patienten/ Probanden durchgeführt wird, begutachten soll, weil derartigen Werbematerialien im Einzelfall eine ethische und (arzt-) berufsrechtliche Relevanz zukommen könnte. Deshalb gibt die Ethikkommission folgende allgemeine Hinweise zu Inhalten und Verfahren rekrutierungsunterstützender Informationen in allgemein zugänglichen Medien:

- Über das (primär fremd- oder allenfalls gruppennützige) Studienziel „Erkenntnisgewinn" (sei es hinsichtlich Sicherheit, Wirksamkeit, geeigneter Dosis o.ä.) darf nicht mit Informationen über den erwarteten, aber noch nicht belegten persönlichen „Gewinn" aus der Perspektive des Studienteilnehmers hinweg getäuscht werden, zumal, wenn nicht alle Studienteilnehmer diese Behandlung erhalten, sondern die Studie einen Placeboarm hat.

  Im Hinblick auf die teilnehmenden Prüfärzte ist in diesem Zusammenhang auf das Schutzgut der vertrauensvollen Arzt- Patientenbeziehung in der Berufsordnung hinzuweisen, dessen Sicherung unter anderem die Bestimmungen des §11 Abs. 2 der Berufsordnung für die Ärzte Bayerns dient:

  Der ärztliche Berufsauftrag verbietet es, diagnostische oder therapeutische Methoden unter missbräuchlicher Ausnutzung des Vertrauens, der Unwissenheit, der Leichtgläubigkeit oder der Hilflosigkeit von Patienten anzuwenden. Unzulässig ist es auch, Heilerfolge, insbesondere bei nicht heilbaren Krankheiten, als gewiss zuzusichern.

- Die werbende Aussage, der Studienteilnehmer habe den Vorteil intensiverer ärztlicher Betreuung, findet zum einen eine berufsrechtliche Grenze der Zulässigkeit in einer anpreisenden Werbung mit Selbstverständlichkeiten, die für den Arzt, dem sie zugute kommt, als berufswidrig im Sinne des §27 Abs. 1 der Berufsordnung für die Ärzte Bayerns anzusehen ist und zum anderen in der gebotenen Ausgewogenheit der Aussage im Hinblick auf die zusätzlichen Belastungen, die der Patient studienhalber auf sich nimmt.

- Personenbezogene Daten des potentiell teilnahmewilligen Patienten /Probanden sollten nicht vom Sponsor oder von ihm beauftragten Instituten abgefragt werden, sondern nur dem vom Patienten /Probanden ggf. gewählten Prüfarzt zugänglich sein.

- Im Hinblick auf das für den Arzt geltende berufsrechtliche Verbot, berufswidrige Werbung zu dulden, sollte möglichst darauf verzichtet werden, die teilnehmenden Prüfärzte unmittelbar zu veröffentlichen. Es erscheint adäquater, wenn dem Anfragenden, potentiell teilnahmewilligen Patienten /Probanden vom Sponsor bzw. einem von ihm beauftragten Institut eine Liste der teilnehmenden Prüfärzte zur Verfügung gestellt wird.

Das rekrutierungsunterstützende Material steht nicht im Widerspruch zu den oben genannten Hinweisen.

Ethikkommission der Medizinischen Fakultät
der Ludwig-Maximilians Universität
Vorsitzender: Prof. Dr. Gustav Paumgartner

Ludwig-
Maximilians-
Universität
München
Marchioninistr. 15
81377 München
Tel: (089) 7095 4609
Fax: (089) 7095 7609
e-mail: Ethikkommission@
med.uni-muenchen.de
02.05.2006

Klinikum der Universität München – Großhadern
Marchioninistr. 15 81377 München

Bavarian Nordic GmbH
Herrn Dr. J. Vollmar
Fraunhoferstraße 13
82152 Martinsried

EudraCT-Nr. 2005-001781-14
Teilweise randomisierte, teilweise doppelblinde, placebo-kontrollierte Phase II Nichtunterlegenheitsstudie zur Bewertung der Immunogenität und Sicherheit von ein und zwei Dosen eines Pockenimpfstoffes bei 18-55 Jahre alten gesunden Probanden
Projekt-Nr. 365-05

Sehr geehrter Herr Dr. Vollmar,
sehr geehrte Frau Uebler,

besten Dank für Ihr Schreiben vom 22.03.2006 mit einer nochmals überarbeiteten Version des Probandentagebuchs und der Patienteninformation zu der o.g. Studie. Dabei lagen im Einzelnen zur Begutachtung vor:

- Tagebuch Version 6 (final) 03 Mar 2006
- Studienteilnehmer- Karte
- Probandeninformation und Einverständniserklärung Version 3.0 28 Feb 2006
- Probandeninformation und Einverständniserklärung Version 4.0 22 Mar 2006.

Es bestehen keine ethisch-rechtlichen Bedenken gegen die Verwendung dieser Patienteninformation, so dass die schon ausgesprochene ethisch-rechtliche Unbedenklichkeit weiter Gültigkeit hat.

Ich wünsche Ihrer Studie weiterhin einen guten Verlauf.

Mit freundlichen Grüßen

Prof. Dr. G. Paumgartner
Vorsitzender der Ethikkommission

Mitglieder der Kommission:
Prof. Dr. G. Paumgartner (Vorsitzender), Prof. Dr. E. Held (stellv. Vorsitzender), Prof. Dr. H. U. Gallwas,
Prof. Dr. D. Kunze, Dr. V. Mönch, PD Dr. V. Nüßler, Prof. Dr. R. Penning, Prof. Dr. K. Hahn, Prof. Dr. K. Pfeifer

Ethikkommission der Medizinischen Fakultät
der Ludwig-Maximilians Universität
Vorsitzender: Prof. Dr. Gustav Paumgartner

Ludwig-
Maximilians-
Universität
München
Marchioninistr. 15
81377 München
Tel: (089) 7095 4609
Fax: (089) 7095 7609
e-mail: Ethikkommission@
med.uni-muenchen.de
21.03.2006

Klinikum der Universität München – Großhadern
Marchioninistr. 15 81377 München

Bavarian Nordic GmbH
Herrn Dr. J. Vollmar
Fraunhoferstraße 13
82152 Martinsried

EudraCT-Nr. 2005-001781-14
Teilweise randomisierte, teilweise doppelblinde, placebo-kontrollierte Phase II Nichtunterlegenheitsstudie zur Bewertung der Immunogenität und Sicherheit von ein und zwei Dosen eines Pockenimpfstoffes bei 18-55 Jahre alten gesunden Probanden
Projekt-Nr. 365-05

Sehr geehrter Herr Dr. Vollmar,
sehr geehrte Frau Uebler,

besten Dank für Ihr Schreiben vom 03.03.2006 mit einer überarbeiteten Version des Probandentagebuchs und der Patienteninformation zu der o.g. Studie. Dabei lagen im Einzelnen zur Begutachtung vor:

- Tagebuch Version 6 (final) 03 Mar 2006
- Probandeninformation und Einverständniserklärung Version 3.0 28 Feb 2006.

Es bestehen keine ethisch-rechtlichen Bedenken gegen die Verwendung dieser Patienteninformation, so dass die schon ausgesprochene ethisch-rechtliche Unbedenklichkeit weiter Gültigkeit hat.

Ich wünsche Ihrer Studie weiterhin einen guten Verlauf.

Mit freundlichen Grüßen

G. Paumgartner

Prof. Dr. G. Paumgartner
Vorsitzender der Ethikkommission

Mitglieder der Kommission:
Prof. Dr. G. Paumgartner (Vorsitzender), Prof. Dr. E. Held (stellv. Vorsitzender), Prof. Dr. H. U. Gallwas, Prof. Dr. D. Kunze, Dr. V. Mönch, PD Dr. V. Nüßler, Prof. Dr. R. Penning, Prof. Dr. K. Hahn, Prof. Dr. K. Pfeifer

Ethikkommission der Medizinischen Fakultät
der Ludwig-Maximilians Universität
Vorsitzender: Prof. Dr. Gustav Paumgartner

Ludwig-
Maximilians-
Universität
München
Marchioninistr. 15
81377 München
Tel: (089) 7095 4609
Fax: (089) 7095 7609
e-mail: Ethikkommission@med.uni-muenchen.de
07.02.2006 Hb/sc

Klinikum der Universität München – Großhadern
Marchioninistr. 15 81377 München

Bavarian Nordic GmbH
Frau Nadine Uebler
Fraunhoferstraße 13
82152 Martinsried

N/ Paul- Ehrlich- Institut
N/ Mitglieder der Ethikkommission
N/ mitbeteiligte Ethikkommission (en)

EudraCT-Nr. 2005-001781-14
Teilweise randomisierte, teilweise doppelblinde, placebo-kontrollierte Phase II Nichtunterlegenheitsstudie zur Bewertung der Immunogenität und Sicherheit von ein und zwei Dosen eines Pockenimpfstoffes bei 18-55 Jahre alten gesunden Probanden
Projekt-Nr. 365-05

Sehr geehrte Frau Uebler,

die Ethikkommission der Med. Fakultät der LMU München ist mit der Federführung für die Bewertung der o.g. multizentrischen klinische Arzneimittelprüfung betraut. Sie hat auf der Basis der eingereichten unten gelisteten Unterlagen beraten. Zur Bewertung hat folgende beteiligte Ethikkommission beigetragen:

- Ethikkommission der Bayerischen Landesärztekammer

Die Begutachtung durch die Ethikkommission der Med. Fakultät der LMU München wird mit einer zustimmenden Bewertung abgeschlossen. Es sind keine Versagensgründe gemäß §42 Abs. 1, Satz 7, Ziffer 1-3 AMG gegeben.

Die oben aufgeführte beteiligte Ethikkommissionen hat der Durchführung an den jeweiligen Prüfzentren zugestimmt:

- Prof. Dr. F. von Sonnenburg, Abtl. f. Tropenmedizin der LMU München, Leopoldstr. 5, 80802 München –
- Dr. St. de la Motte, Harrison Clinical Research GmbH, Hilblestr. 4, 83636 München.

Unabhängig vom Beratungsergebnis möchten wir darauf hinweisen, dass die ärztliche und juristische Verantwortung für die Durchführung des Projektes uneingeschränkt beim Leiter der klinischen Prüfung und auch bei allen an der Prüfung teilnehmenden Ärzten verbleibt.

Eingereichte Unterlagen:

- Begleitschreiben 19 Dec 2005
- Kostenübernahmeerklärung 16 Dec 2005

FR 3 325/00 Ml.

- Checkliste signed16 Dec 2005
- Modul 1signed 16 Dec 2005
- Modul 2 signed 16 Dec 2005
- Studienprotokoll Version 1.0 12 Dec 2005
- Investigator's Brochure Version 08 22 Nov 2005
- Probandeninformation u. Einverständniserklärung inkl. Datenschutzerklärung und Einwilligung zum Datenschutz Version 1.0 12 Dec 2005
- Probandentagebuch
- Kopie der Versicherungspolice inkl. Versicherungsbedingungen 06 Dec 2005
- Kopie des Bestätigungsschreibens zur Vergabe der EudraCT- Nr. 15 Apr 2005
- Beispieltexte zur Probandenrekrutierung
- Lebensläufe der Prüfer
  Angaben zu wirtschaftlichen Interessen der Prüfer (Financial Disclosure) signed 15 Dec 2005
  Eignung der Prüfstelle
- Vergütung der Prüfer /Vertrag zwischen Sponsor und Prüfstelle
- Zwischenauswertung der Studie POX-MVA-007 21 Sep 2005

- Antwortschreiben auf das Schreiben der EK (23 Jan 2006) vom 30 Jan 2006
- Probandeninformation und Einverständniserklärung Version 2.0 27 Jan 2006.

Folgende Mitglieder der federführenden Ethikkommission haben an der Beratung des o.g. Antrags mitgewirkt:

Prof. Dr. G. Paumgartner (Vorsitzender, Internist/Gastroenterologe/Klinischer Pharmakologe)
Prof. Dr. E. Held (stellv. Vorsitzender, Internist/Nephrologe/Kardiologe)
Prof. Dr. H. U. Gallwas (Jurist)
Prof. Dr. D. Kunze (Pädiater)
Frau Dr. V. Mönch (Apothekerin/Mitglied mit Schwerpunkt außerhalb der Wissenschaft)
PD Dr. V. Nüßler (Internist/Onkologe)
Prof. Dr. R. Penning (Rechtsmediziner)
Prof. Dr. K. Hahn (Nuklearmediziner/Strahlenschutzexperte)
Prof. Dr. K. J. Pfeifer (Radiologe/Strahlenschutzexperte)

Die Ethikkommission der Med. Fakultät der LMU München ist nach Landesrecht gebildet und nach GCP-ICH zusammengesetzt. Sie arbeitet entsprechend GCP-V.

Mit freundlichen Grüßen

G. Paumgartner

Prof. Dr. G. Paumgartner
Vorsitzender der Ethikkommission

Mitglieder der Kommission:
Prof. Dr. G. Paumgartner (Vorsitzender), Prof. Dr. E. Held (stellv. Vorsitzender), Prof. Dr. H. U. Gallwas,
Prof. Dr. D. Kunze, Dr. V. Mönch, PD Dr. V. Nüßler, Prof. Dr. R. Penning, Prof. Dr. K. Hahn, Prof. Dr. K. Pfeifer

Paul-Ehrlich-Institut
Bundesamt für Sera und Impfstoffe

Paul-Ehrlich-Institut Postfach D-63207 Langen

Bavarian Nordic GmbH

Herr Dr. Klaus Tressl
Fraunhoferstr. 13
82152 Martinsried

AZ (bitte angeben)	Telefon-Durchwahl	Telefax-Durchwahl	Datum
23510	+49(0) 6103 771811	+49(0) 6103 771277	28.02.2007

Genehmigung der Änderung der klinischen Prüfung gem. § 10 Abs. 1 GCP-V
Prüfsubstanz: IMVAMUNE
Studien-Nr.: POX-MVA-005
EudraCT-Nr.: 2006-001781-14 Vorlage-Nr.: 192/02

Sehr geehrte Damen und Herren,

im Anhang übersenden wir Ihnen den Genehmigungsbescheid vom 28.02.2007 des Paul-Ehrlich Instituts zur Änderung der oben genannten klinischen Prüfung mit dem Studientitel:

A partially randomized, partially double-blind, placebo-controlled Phase II non-inferiority study to evaluate immunogenicity and safety of one and two doses of MVA-BN (IMVAMUNE) smallpox vaccine in 18-55 year old healthy subjects

Mit freundlichen Grüßen
Im Auftrag

Dr. H. Krafft

Paul-Ehrlich-Institut
Bundesamt für Sera und Impfstoffe

Paul-Ehrlich-Institut, Postfach D-63207 Langen

Bavarian Nordic GmbH

Herr Dr. Klaus Tressl
Fraunhoferstr. 13
82152 Martinsried

EudraCT-Nr.: 2005-001781-14
Vorlage-Nr.: 192/02
Eingang des Antrages: 09.02.2007

Langen, den 28.02.2007

Genehmigung der Änderung der klinischen Prüfung gem. § 10 Abs. 1 GCP-V
Kurz-Titel: POX-MVA-005
Ihr Antrag vom 08.02.2007

Bescheid

Die Genehmigung zur Änderung der klinischen Prüfung mit dem Studientitel:

A partially randomized, partially double-blind, placebo-controlled Phase II non-inferiority study to evaluate immunogenicity and safety of one and two doses of MVA-BN (IMVAMUNE) smallpox vaccine in 18-55 year old healthy subjects

zur Prüfung der Prüfsubstanz IMVAMUNE, die mit Schreiben vom 08.02.2007 beim Paul-Ehrlich-Institut beantragt wurde, wird erteilt.

Der Antrag betrifft die Änderungen der Prüferinformationen.

Eine Entscheidung über die Kosten ergeht durch gesonderten Bescheid.

Rechtsbehelfsbelehrung

Gegen diesen Bescheid kann innerhalb eines Monats nach Bekanntgabe Widerspruch erhoben werden. Der Widerspruch ist beim Paul-Ehrlich-Institut, Bundesamt für Sera und Impfstoffe, Paul-Ehrlich-Str. 51-59, 63325 Langen, schriftlich oder zur Niederschrift einzulegen.

Mit freundlichen Grüßen
Im Auftrag

Dr. H. Krafft

****
*** SENDEBERICHT ***
****

SENDUNG OK

SE/EM NR	1085
NR. GEGENSTELLE	+49610377
SUBADRESSE	
NAME GEGENSTELLE	
ANF.ZEIT	12/04 09:11
ÜB.ZEIT	00'26
S.	1
ERGEBNIS	OK

Paul-Ehrlich-Institut
Bundesamt für Sera und Impfstoffe

Paul-Ehrlich-Institut Postfach D-63207 Langen

Bavarian Nordic GmbH

Herr Dr. Klaus Tressl
Fraunhoferstr. 13
82152 Martinsried

FAX 089 8565 1333

→ FaxAntwort Herr Dr. Krafft – Vielen Dank! Mit freundlichen Grüßen Klaus Tressl 12.04.06

Empfangsbestätigung

Das übermittelte Schreiben des

Paul-Ehrlich-Instituts,
Paul-Ehrlich-Str. 51 - 59,
63225 Langen

vom 11.04.2006 zur klinischen Prüfung mit dem Titel:

A partially randomized, partially double-blind, placebo-controlled Phase II non-inferiority study to evaluate immunogenicity and safety of one and two doses of MVA-BN (IMVAMUNE) smallpox vaccine in 18-55 year old healthy subjects

zur Prüfsubstanz **IMVAMUNE**

EudraCT-Nr.: **2005-001781-14** Vorlage-Nr.: **192/01** Kurztitel: **POX-MVA-005**

habe ich am 12.04.2006 **um** 9:05 **Uhr empfangen.**

Das übermittelte Schreiben bestand aus 3 **Seiten und ist**

X **vollständig empfangen worden**

0 **nicht vollständig empfangen worden. Es fehlten die Seiten ....**

Martinsried, **den** 12.04.2006

Paul-Ehrlich-Institut
Bundesamt für Sera und Impfstoffe

Paul-Ehrlich-Institut Postfach D-63207 Langen

Bavarian Nordic GmbH

Herr Dr. Klaus Tressl
Fraunhoferstr. 13
82152 Martinsried

FAX 089 8565 1333

→ Fax Antwort Herr Dr. Krafft – Vielen Dank! Mit freundlichen Grüßen, Klaus Tressl 12.04.06

Empfangsbestätigung

Das übermittelte Schreiben des

Paul-Ehrlich-Instituts,
Paul-Ehrlich-Str. 51 - 59,
63225 Langen

vom 11.04.2006 zur klinischen Prüfung mit dem Titel:

A partially randomized, partially double-blind, placebo-controlled Phase II non-inferiority study to evaluate immunogenicity and safety of one and two doses of MVA-BN (IMVAMUNE) smallpox vaccine in 18-55 year old healthy subjects

zur Prüfsubstanz **IMVAMUNE**

EudraCT-Nr.: **2005-001781-14** Vorlage-Nr.: **192/01** Kurztitel: **POX-MVA-005**

habe ich am 12.04.2006 **um** 9⁰⁵ **Uhr empfangen.**

Das übermittelte Schreiben bestand aus 3 **Seiten und ist**

X **vollständig empfangen worden**

0 **nicht vollständig empfangen worden. Es fehlten die Seiten ....**

Martinsried ...., **den** 12.04.2006

Bavarian Nordic GmbH
Fraunhoferstraße 13
82152 Martinsried
Tel. 089 / 85 65 00 30, Fax 089 / 85 65 13 33

i.A. Klaus Tressl (Monitor RA)

(Stempel und Unterschrift des Empfängers)

Bavarian Nordic GmbH
Fraunhoferstraße 13
82152 Martinsried
Tel. 089 / 85 65 00 30, Fax 089 / 85 65 13 33

Bitte übersenden Sie die Empfangsbestätigung umgehend nach dem Empfang des Schreibens zurück an das Paul-Ehrlich-Institut,
→ Telefax-Nr.: +49 (0)6103 771277, zH. Dr. H. Krafft

Dienstag, 11. April 2006

Paul-Ehrlich-Institut
Bundesamt für Sera und Impfstoffe

Paul-Ehrlich-Institut Postfach D-63207 Langen

Bavarian Nordic GmbH

Herr Dr. Klaus Tressl
Fraunhoferstr. 13
82152 Martinsried

AZ (bitte angeben)	Telefon-Durchwahl	Telefax-Durchwahl	Datum
23510	+49(0) 6103 771811	+49(0) 6103 771277	11.04.2006

Genehmigung klinischer Prüfungen gem. § 42 Abs. 2 AMG
Prüfsubstanz: IMVAMUNE
Studien-Nr.: POX-MVA-005
EudraCT-Nr.: 2005-001781-14 Vorlage-Nr.: 192/01

Sehr geehrte Damen und Herren,

im Anhang übersenden wir Ihnen den Genehmigungsbescheid vom 11.04.2006 des Paul-Ehrlich Instituts zu oben genannter klinischer Prüfung mit dem Studientitel:

A partially randomized, partially double-blind, placebo-controlled Phase II non-inferiority study to evaluate immunogenicity and safety of one and two doses of MVA-BN (IMVAMUNE) smallpox vaccine in 18-55 year old healthy subjects

Mit freundlichen Grüßen
Im Auftrag

Dr. H. Krafft

Paul-Ehrlich-Institut
Bundesamt für Sera und Impfstoffe
Federal Agency for Sera and Vaccines

Paul-Ehrlich-Straße 51-59
D-63225 Langen
Postfach D-63207 Langen

Telefon 0 6103/77-1811
Telefax 0 6103/77-1277

Paul-Ehrlich-Institut
Bundesamt für Sera und Impfstoffe

Paul-Ehrlich-Institut Postfach D-63207 Langen

Bavarian Nordic GmbH

Herr Dr. Klaus Tressl
Fraunhoferstr. 13
82152 Martinsried

EudraCT-Nr.: 2005-001781-14
Vorlage-Nr.: 192/01
Eingang des Antrages: 31.01.2006

Langen, den 11.04.2006

Genehmigung der klinischen Prüfung gem. § 42 Abs. 2 AMG
Kurz-Titel: POX-MVA-005
Ihr Antrag vom 27.01.2006

Bescheid

Die Genehmigung der klinischen Prüfung mit dem Studientitel:

A partially randomized, partially double-blind, placebo-controlled Phase II non-inferiority study to evaluate immunogenicity and safety of one and two doses of MVA-BN (IMVAMUNE) smallpox vaccine in 18-55 year old healthy subjects

zur Prüfung der Prüfsubstanz IMVAMUNE, die mit Schreiben vom 27.01.2006 beim Paul-Ehrlich-Institut beantragt wurde, wird erteilt.

Eine Entscheidung über die Kosten ergeht durch gesonderten Bescheid.

Rechtsbehelfsbelehrung:

Gegen diesen Bescheid kann innerhalb eines Monats nach Bekanntgabe Widerspruch erhoben werden. Der Widerspruch ist beim Paul-Ehrlich-Institut, Bundesamt für Sera und Impfstoffe, Paul-Ehrlich-Str. 51-59, 63325 Langen, schriftlich oder zur Niederschrift einzulegen.

Mit freundlichen Grüßen

PAUL-EHRLICH-INSTITUT LANGEN

Prof. Dr. J. Löwer
Präsident
Paul-Ehrlich-[illegible]
Bundesamt für Sera und [illegible]
Paul-Ehrlich-Str. 51-59
63225 Langen

Paul-Ehrlich-Institut
Bundesamt für Sera und Impfstoffe
Federal Agency for Sera and Vaccines

Paul-Ehrlich-Straße 51-59
D-63225 Langen
Postfach D-63207 Langen

Telefon 0 6103/77-1811
Telefax 0 6103/77-1277

Harrison Clinical Research / Clinical Research Unit
Gesellschaft für klinische Forschung mbH

Albrechtstraße 43
80636 München

Medizinischer Leiter: Dr. med. S. de la Motte

Telefonnummer: 0800-8063680 (gebührenfrei)
Notfallnummer: 0163-8588599

Internet: www.harrison.de

Probandeninformation und Einverständniserklärung

Studienprotokoll vom 12. Dezember 2005

Teilweise randomisierte, teilweise doppelblinde, placebo-kontrollierte Phase II Nichtunterlegenheitsstudie zur Bewertung der Immunogenität und Sicherheit von ein und zwei Dosen eines Pockenimpfstoffs bei 18-55 Jahre alten gesunden Probanden

Prüfärzte:

Prof. Dr. med. Frank von Sonnenburg<br>Abteilung für Infektions- und Tropenmedizin<br>der Ludwig-Maximilians-Universität<br>Leopoldstraße 5<br>80802 München<br>Tel. 089-2180 3830	Dr. med. Stephan de la Motte<br>Harrison Clinical Research GmbH<br>Clinical Unit<br>Albrechtstraße 43<br>80636 München<br>Tel. 089-126680 1210

Probanden-Nummer: |__|__|__|　　Screening-Nummer: |__|__|__|__|

Sehr geehrte Studienteilnehmerin,
sehr geehrter Studienteilnehmer,

wir möchten Ihnen anbieten an einer klinischen Prüfung (Studie) zur Erforschung eines neuen verbesserten Pockenimpfstoffs teilzunehmen. Diese klinische Prüfung wird von Harrison Clinical Research im Auftrag von Bavarian Nordic durchgeführt. Leitender Prüfarzt ist Herr Prof. von Sonnenburg. Alle in der Studie anfallenden Untersuchungen werden am Prüfzentrum, d.h. in den Räumlichkeiten von Harrison Clinical Research unter Mitarbeit der dort tätigen Prüfärzte durchgeführt.
Mit dieser Information möchten wir Sie über den Studienablauf sowie den mit einer Teilnahme verbundenen möglichen Nutzen und die möglichen Risiken informieren, damit Sie sich entscheiden können, ob Sie an der Studie teilnehmen wollen oder nicht.

Information zu Pocken und zum Pockenimpfstoff

Die Pocken, eine gefährliche, bislang unbehandelbare Infektionskrankheit mit einer Sterblichkeit von bis zu 30%, sind als natürlicherweise auftretende Infektion seit 1977 ausgerottet. Dies konnte durch eine konsequente Impfung gegen Pocken erreicht werden. Das Pockenvirus steht jedoch seit langem an der Spitze potentieller biologischer Kampfstoffe. Spätestens seit dem Einsatz von Milzbrand als biologische Waffe wird befürchtet, dass auch Pockenviren von Bioterroristen als Waffe eingesetzt werden könnten.

Da es weltweit keine ausreichenden Vorräte an Pockenimpfstoffen mehr gibt und die traditionellen Pockenimpfstoffe eine hohe Rate an zum Teil schweren Nebenwirkungen besitzen, besteht die Notwendigkeit, neue gut verträgliche Pockenimpfstoffe zu entwickeln. Der in dieser Studie zur Anwendung kommende Impfstoff ist eine stark abgeschwächte Variante eines traditionell zur Pockenimpfung verwendeten Vacciniavirus. Nach der klinischen Überprüfung an Mensch und Tier erhielt er den Namen MVA (= Modified Vaccinia Ankara). Im Gegensatz zu traditionellen Pockenimpfstoffen vermehrt sich MVA nicht im Menschen. Es kann deshalb auch keine durch eine Vermehrung des Impfvirus bedingte Erkrankung auslösen und nicht von Mensch zu Mensch übertragen werden.

Insgesamt wurden zwischen 1972 und 1980 im Rahmen eines Stufenimpfprogramms mehr als 120.000 Menschen mit MVA geimpft. Die Verträglichkeit war dabei gut.

Aus diesem MVA-Impfstoff wurde der in dieser Studie verwendete Impfstoff entwickelt. Nach den bisher vorliegenden Forschungsergebnissen, kann man davon ausgehen, dass dieser Impfstoff ähnlich gut verträglich ist wie der in den 70er Jahren verwendete MVA Vorläuferimpfstoff. Bisher wurden mit diesem neu entwickelten Impfstoff in vier Studien insgesamt über 350 gesunde Probanden geimpft, wobei sich der Impfstoff als sicher und gut verträglich erwies.

Dieser Impfstoff befindet sich im Stadium der klinischen Erprobung und ist noch nicht zugelassen. Eine Zulassung des Impfstoffs wird aber angestrebt.

Ziel dieser klinischen Prüfung

Das primäre Ziel dieser klinischen Prüfung ist der Vergleich der Immunantwort von Probanden mit und ohne Vorgeschichte einer Pockenimpfung. Dadurch soll gezeigt werden, dass bei Probanden, die bereits gegen Pocken geimpft wurden, eine einzelne Impfung mit dem neu entwickelten Impfstoff ausreichend ist, um eine vergleichbare Immunität zu erreichen, wie bei Probanden ohne Pockenimpfung in der Vorgeschichte, die zwei Impfungen erhalten. Des Weiteren sollen Sicherheit und Verträglichkeit des Impfstoffs, insbesondere in Bezug auf EKG–Veränderungen und andere Symptome die auf Herzprobleme hinweisen, nachgewiesen werden.

Die genaue Kontrolle von Impfungen mit dem herkömmlichen Impfstoff gegen Pocken ergab den Verdacht, dass der herkömmliche Impfstoff eine Herzentzündung (Myokarditis), eine Entzündung der Herzhaut (Perikarditis) und/oder eine Kombination beider Entzündungen (Myoperikaditis) auslösen kann. Es ist zurzeit nicht bekannt, ob diese Symptome durch die Vermehrung des herkömmlichen Pockenimpstoffes oder durch einen anderen Mechanismus ausgelöst werden.

Obwohl diese Entzündungen mit MVA nicht beobachtet wurden, werden vorsorglich Probanden, bei denen von einen Arzt Herzbeschwerden mit oder ohne Symptome festgestellt wurden oder die ein Risiko haben, solche Beschwerden zu entwickeln, nicht an dieser Studie teilnehmen.

Im Gegensatz zu traditionellen Pockenimpfstoffen kann sich der in dieser Studie eingesetzte Impfstoff im Menschen nicht vermehren. Deshalb ist auch nicht möglich, dass die schwerwiegenden Nebenwirkungen der traditionellen Pockenimpfstoffe auftreten, die durch die Vermehrung des Impfvirus ausgelöst werden.

Ablauf der klinischen Prüfung

Wenn Sie sich entscheiden an dieser Studie teilzunehmen, werden Sie nach einer Voruntersuchung einer von vier möglichen Gruppen zugeteilt.

Im Rahmen dieser klinischen Prüfung sollen insgesamt 800 Teilnehmer geimpft werden (200 pro Gruppe).

Sollten Sie bereits gegen Pocken geimpft sein, werden Sie in die Gruppe der bereits geimpften aufgenommen und erhalten eine Impfung mit dem Pockenimpfstoff.

Sollten Sie noch nicht gegen Pocken geimpft sein, werden Sie durch ein Zufallsprinzip einer

der übrigen drei Gruppen zugeteilt. Dadurch soll verhindert werden, dass individuelle Faktoren in die Entscheidung der Gruppenzuteilung einbezogen werden. Je nachdem welcher Gruppe Sie während der Studie zugeteilt werden erhalten Sie entweder:

- 2 Impfungen mit dem Pockenimpfstoff
- 1 Impfung mit dem Pockenimpfstoff und 1 Impfung mit Placebo
- 2 Impfungen mit Placebo

Ein Placebo ist ein Scheinmedikament, das keinen Wirkstoff enthält.
Weder Sie noch Ihr Prüfarzt wissen während der Studie zu welcher der drei Gruppen Sie gehören. Dies schützt vor ungewollten Beeinflussungen des Ergebnisses. Im Notfall kann aber festgestellt werden welche Behandlung Sie erhalten haben.

Je nachdem, ob Sie bereits in der Vorgeschichte gegen Pocken geimpft wurden oder nicht, ergibt sich für Sie folgender Ablauf:

Probanden **ohne** Pockenimpfung in der Vorgeschichte:
Die Studiendauer beträgt einschließlich der Nachuntersuchung ca. 8 Monate.
Es sind 7 Untersuchungstermine vorgesehen. Sie erhalten im Abstand von vier Wochen zwei Impfungen unter die Haut des Oberarms (subkutan). Nach jeder Impfung erfolgt eine 30-minütige Nachbeobachtungszeit.
Im Verlauf der Studie wird Ihnen bei jedem Besuchstermin Blut abgenommen, um Laborparameter zu kontrollieren und die Immunantwort zu überprüfen. Die während der Studie abgenommene Menge an Blut beträgt insgesamt ca. 93-123 ml (zum Vergleich: bei einer Blutspende werden 400-500 ml Blut auf einmal entnommen).

Die genauen Untersuchungstermine entnehmen Sie bitte der tabellarischen Übersicht (**Besuchschema 1**).

Probanden **mit** Pockenimpfung in der Vorgeschichte:
Die Studiendauer beträgt einschließlich der Nachuntersuchung ca. 7 Monate.

Es sind 5 Untersuchungstermine vorgesehen. Sie erhalten eine Impfung unter die Haut des Oberarms (subkutan). Nach der Impfung erfolgt eine 30-minütige Nachbeobachtungszeit.
Im Verlauf der Studie wird Ihnen bei jedem Besuchstermin Blut abgenommen, um Laborparameter zu kontrollieren und die Immunantwort zu überprüfen. Die während der Studie abgenommene Menge an Blut beträgt insgesamt ca. 62-77 ml (zum Vergleich: bei einer Blutspende werden 400-500 ml Blut auf einmal entnommen).

Die genauen Untersuchungstermine entnehmen Sie bitte der tabellarischen Übersicht (**Besuchsschema 2**).

Besuchsschema 1:

Besuch	0	1	2	3	4	5	6
Woche	-4 - 0	0	2	4	6	8	30
Impfung		I		II			
Körperliche Untersuchung	X	X	X	X	X	X	X
EKG	X		X		X	(X)	(X)
Schwangerschaftstest	X	X		X		X	
Blutabnahme	X	X	X	X	X	X	X
Urinuntersuchung	X						
Tagebuchkarte (Ausgabe)		X		X			
Tagebuchkarte (Rückgabe)			X		X		
Befragung zu Nebenwirkungen und Medikamenten	X	X	X	X	X	X	X

(X) = falls notwendig

Besuchsschema 2:

Besuch	0	1	2	3	4
Woche	-4 - 0	0	2	4	26
Impfung		I			
Körperliche Untersuchung	X	X	X	X	X
EKG	X		X	(X)	(X)
Schwangerschaftstest	X	X		X	
Blutabnahme	X	X	X	X	X
Urinuntersuchung	X				
Tagebuchkarte (Ausgabe)		X			
Tagebuchkarte (Rückgabe)			X		
Befragung zu Nebenwirkungen und Medikamenten	X	X	X	X	X

(X) = falls notwendig

Alle Probanden:

An den Besuchstagen finden Sie sich bitte jeweils morgens nüchtern im Studienzentrum ein. Es werden dann die entsprechenden Untersuchungen vorgenommen. Anschließend können Sie das Studienzentrum wieder verlassen.

Bitte bringen Sie zu jedem Besuch Ihren Personalausweis mit.

An den Tagen der Impfung und den darauf folgenden 7 Tagen müssen Sie ein Tagebuch führen, in dem Sie eventuelle lokale Symptome an der Injektionsstelle wie Rötung, Schwellung, Verhärtung und Schmerzen, sowie allgemeine Symptome wie Fieber, Kopfschmerzen, Müdigkeit, Muskelschmerzen oder Übelkeit verzeichnen. Dieses Tagebuch müssen Sie nach der 7-tägigen Dokumentation bei Ihrem nächsten Besuch wieder im Studienzentrum abgeben.

Für den Fall, dass bei Ihnen während der Studie Symptome festgestellt werden, die auf Herzprobleme hinweisen könnten, werden Sie zusätzlich von einem Herzspezialisten (Kardiologen) untersucht. Hierbei werden weitere Untersuchungen (z.B. Belastungs-EKG, Untersuchung der Herzenzyme, Ultraschall des Herzens) durchgeführt. Durch diese Untersuchungen können zusätzliche Besuchstermine bei diesem Kardiologen notwendig werden.

Sie erhalten eine Studienteilnehmerkarte ausgehändigt, auf der eine Telefonnummer aufgeführt ist, unter der Sie zu jeder Zeit Ihren Prüfarzt erreichen können. Diese Karte sollten Sie zu Ihrer eigenen Sicherheit bis zur Abschlussuntersuchung immer bei sich tragen.

Mögliche Risiken die sich aus der Studienteilnahme ergeben

Historisch wurde der Vorgängerimpfstoff als erster Schritt eines zweistufigen Impfprogramms in die Haut (intrakutan) oder unter die Haut (subkutan) verabreicht. Die mit dem Vorgängerimpfstoff durchgeführte Immunisierung von mehr als 120.000 Kleinkindern (unter 2 Jahren) und Erwachsenen wurde im Allgemeinen gut vertragen. Als unerwünschte Ereignisse wurden am häufigsten lokale Reaktionen an der Injektionsstelle angegeben. Dabei traten vorübergehend Rötung, Schwellung, Schmerzen, Verhärtung und Juckreiz auf. Als unerwünschte Ereignisse wurden z.B. grippeähnliche Symptome oder leichtes vorübergehendes Fieber gemeldet.

Zu dem in dieser Studie verwendeten Impfstoff liegen Daten aus klinischen Studien mit über 350 Probanden im Alter von 18 bis 55 Jahren vor, die bis zu zweimal mit Impfstoff geimpft wurden. Neben lokalen Reaktionen wie Rötung, Schwellung und Schmerz an der Injektionsstelle die sehr häufig auftraten, wurden in einzelnen Fällen folgende Symptome beobachtet bei denen ein Zusammenhang mit der Impfung nicht ausgeschlossen werden kann:

Unwohlsein, Fieber, Kopfschmerzen, Schwindel, Müdigkeit, Übelkeit, Lymphknotenschwellung, Muskel- und Gelenkschmerzen, Hitzewallung / Kältegefühl. Dies sind Symptome wie sie auch nach anderen Impfungen (wie z.B. Grippe oder Tetanus) beobachtet werden.
Keines dieser Symptome war schwerwiegend und alle bildeten sich innerhalb kurzer Zeit zurück.

Eine im Einzelfall nicht vorhersehbare unerwünschte Reaktion kann bei einer Impfung – wie bei jeder anderen Injektion mit biologischem Material – nicht ausgeschlossen werden. Prinzipiell kann es, wie bei allen Injektionen oder Arzneimitteleinnahmen, zu **sofort oder verzögert auftretenden Überempfindlichkeitsreaktionen** (bis hin zu Schockreaktionen) oder zu unerwarteten Wirkungen kommen. Aus diesem Grund werden Sie nach jeder Impfung 30 Minuten lang beobachtet. Medikamente zur Behandlung möglicher allergischer Reaktionen sind bei Ihrem Prüfarzt verfügbar.

Es kann ebenfalls nicht ausgeschlossen werden, dass auch bisher unbekannte, neue Nebenwirkungen auftreten können. Um diese im Fall des Auftretens rechtzeitig zu erkennen, werden sie in regelmäßigen Abständen engmaschig betreut und untersucht. Sollten diesbezüglich vor oder während der Studie neue Informationen bekannt werden, werden Ihnen diese unverzüglich mitgeteilt.

Bitte nehmen Sie nicht eigenständig andere Medikamente ein, ohne den Prüfarzt zu informieren. Medikamente zur Behandlung anderer Krankheiten sollen in Absprache mit dem Prüfarzt unverändert weiter eingenommen werden.

Bedingt durch die Blutabnahmen kann es an den Punktionsstellen zu Hautverletzungen und Hämatomen (Blutergüssen) kommen. Die Gefahr einer Infektion an der Punktionsstelle ist außerordentlich gering.

Möglicher Nutzen aus der Teilnahme an der klinischen Prüfung

Zurzeit gibt es keinerlei medizinische Indikation/Nutzen für Sie eine Pockenschutzimpfung durchzuführen, es sei denn, in Ihrem persönlichen Sicherheitsbedürfnis gegenüber möglichen Biowaffen-Angriffen.

Ziel dieser Studie ist es, durch die Impfung mit dem Pockenimpfstoff eine Schutzwirkung vor einer Infektion mit Pockenviren hervorzurufen. Sollten Sie in der Vergangenheit nicht gegen Pocken geimpft worden sein, besteht jedoch die Möglichkeit, dass Sie statt dem Pockenimpfstoff ein Scheinmedikament (Placebo) erhalten. Die Wahrscheinlichkeit, dass Sie im Rahmen der Studie gar keinen Pockenimpfstoff sondern nur Placebo erhalten beträgt 33%. Weitere 33% erhalten nur eine Impfung mit dem Pockenimpfstoff, gefolgt von Placebo, die restlichen 33% erhalten zwei Impfungen mit dem Pockenimpfstoff.

Wenn Sie in der Vergangenheit gegen Pocken geimpft wurden erhalten Sie in jedem Fall den Pockenimpfstoff und es besteht die Möglichkeit, dass Ihr Impfschutz aufgefrischt wird.

Da sich der in der Studie eingesetzte Impfstoff noch in der klinischen Entwicklungsphase befindet, kann keine Aussage über die Wirksamkeit des Impfschutzes gemacht werden.

Die Resultate der während der Studie durchgeführten Tests und Untersuchungen (Blutuntersuchungen und EKGs) werden Ihnen kostenlos zur Verfügung gestellt.

Darüber hinaus tragen Sie mit Ihrer Studienteilnahme zur Entwicklung eines besser verträglichen Pockenimpfstoffes bei, eine Maßnahme die im Falle eines Wiederauftretens der Pocken Millionen von Menschen vor den teils schweren Nebenwirkungen der traditionellen Pockenimpfstoffe bewahren könnte.

Andere Behandlungsmöglichkeiten

In Deutschland gibt es keinen zugelassenen Impfstoff gegen Pocken. Die Bundesregierung hat für den Fall eines Pockenausbruchs einen auf einem historischen Impfvirus basierenden Pockenimpfstoff eingelagert. Da dieser Impfstoff auf einem vermehrungsfähigen Impfvirus basiert, kann er schwerwiegende Nebenwirkungen auslösen und ist deshalb nur für Notfälle bestimmt.

Frauen im gebärfähigem Alter

Frauen im gebärfähigen Alter müssen mindestens 30 Tage lang vor der ersten Impfung eine zuverlässige Verhütungsmethode angewendet haben, und müssen zustimmen während der Studie eine zuverlässige Verhütungsmethode weiter anzuwenden. Zuverlässige Verhütungsmethoden sind: Enthaltsamkeit, Barrieremethoden (z.B. Kondom, Diaphragma), Intrauterin-Systeme (Hormonspirale), hormonelle Methoden (z.B. Antibabypille).

Sollten Sie während dieser klinischen Studie schwanger werden, melden Sie dies bitte sofort Ihrem Prüfarzt.

Sollten Sie nach Ende dieser klinischen Studie erfahren, dass Sie während dieser Studie schwanger geworden sind, melden Sie dies bitte ebenfalls sofort Ihrem Prüfarzt.

Datenschutz und Vertraulichkeit

Bei dieser Studie werden die Vorschriften über die ärztliche Schweigepflicht und den Datenschutz eingehalten. Von Ihrem Prüfarzt werden persönliche Daten und Befunde über Sie erhoben und am Prüfzentrum aufbewahrt. Ihre Daten werden im Prüfzentrum von Harrison Clinical Research verschlossen aufbewahrt und können nur von Ihrem Prüfarzt oder von ihm beauftragten Personen eingesehen werden. Ihre persönlichen Daten werden vor dem Zugriff unbefugter Dritter durch ein Zugangskontrollsystem am Prüfzentrum geschützt.

Vor Weiterverarbeitung und Weitergabe von Studiendaten an den Sponsor Bavarian Nordic, die Ethikkommission oder Behörden, werden diese von Ihrem Prüfarzt, oder einer von ihm beauftragten Person verschlüsselt (pseudonymisiert). Der Verschlüsselungscode besteht aus einer dreistelligen Zahl, die keinen Rückschluss auf Ihre Person zulässt.

Um eine vollständige und korrekte Übertragung der für die wissenschaftliche Bewertung wichtigen Daten zu gewährleisten, dürfen Mitarbeiter des Auftraggebers oder von ihm beauftragte Fachleute, für die klinische Prüfung zuständige in- und ausländische Überwachungsbehörden sowie ggf. Mitglieder der Ethikkommission beim Prüfarzt Einblick in Ihre personenbezogenen Krankenunterlagen nehmen. Hierfür ist Ihre ausdrückliche Einwilligung erforderlich. Die korrekte Aufzeichnung von Messwerten und klinischen Befunden ist besonders wichtig zur Überwachung der Studiensicherheit, für den Forschungszweck und dient der Verbesserung der Arzneimittelsicherheit. Alle mit der Datenüberprüfung beauftragten Personen sind zur strengen Vertraulichkeit und zur Beachtung des Datenschutzes verpflichtet. Ihre Studienteilnahme wird vertraulich behandelt. Sie werden nicht namentlich in irgendeinem Bericht der Studie genannt. Im Falle von Veröffentlichungen der Studienergebnisse bleibt die Vertraulichkeit Ihrer persönlichen Daten ebenfalls gewährleistet, wenn überhaupt, werden die Daten in verschlüsselter Form verwendet. Ihre Identität wird von Ihrem Prüfarzt niemanden bekannt gegeben außer zu o.g. Zwecken, in medizinischen Notfällen oder wenn dies vom Gesetzgeber gefordert wird.

Sollten Sie Ihre Einwilligung zur Studienteilnahme während der Studie widerrufen, dürfen die bis dahin gespeicherten Daten weiterhin verwendet werden, soweit dies erforderlich ist, um Wirkungen des zu prüfenden Arzneimittels festzustellen, um sicherzustellen, dass Ihre schutzwürdigen Interessen nicht beeinträchtigt werden und um der Pflicht zur Vorlage vollständiger Zulassungsunterlagen zu genügen.

Die Unterlagen werden mindestens zehn Jahre nach Beendigung oder Abbruch der Prüfung am Prüfzentrum von Harrison Clinical Research GmbH aufbewahrt.

Haben Sie weitere Fragen?

Sie haben das Recht, sich jederzeit bei Ihrem Prüfarztarzt persönlich über die möglichen oder bekannten Risiken dieser Studie zu informieren. Sie erhalten vom Prüfarztarzt alle neuen Informationen, die sich im Laufe der Untersuchung ergeben und Auswirkungen auf Ihre Studienteilnahme haben könnten. Falls Sie irgendwelche Fragen zu dieser Studie haben, so wenden Sie sich bitte an Ihren aufklärenden Prüfarzt oder den leitenden Prüfarzt:

Prof. Dr. med. Frank von Sonnenburg
Abteilung für Infektions- und Tropenmedizin
der Ludwig-Maximilians-Universität
Leopoldstraße 5
80802 München
Tel.: 089-2180 3830

Freiwilligkeit der Teilnahme / Vorzeitige Beendigung der klinischen Prüfung

Ihre Teilnahme an dieser klinischen Prüfung ist freiwillig und Sie haben jederzeit das Recht, die Teilnahme zu beenden. Eine Begründung ist nicht erforderlich, und die Beendigung der Teilnahme wird Ihnen keinerlei Nachteile bringen. Ihre ärztliche Behandlung wird durch Ihre Entscheidung nicht beeinträchtigt, insbesondere wird Ihnen keine Behandlung vorenthalten.

Ihr Prüfarzt kann Sie jederzeit aus der Studie nehmen, falls dies medizinisch angezeigt ist. Er wird Ihnen dazu die Gründe für den Studienabbruch mitteilen.

Sollten Sie die Teilnahme aus eigenem Entschluss oder auf Anraten Ihres Prüfarztes beenden, werden Sie möglicherweise nach Ihren Erfahrungen mit dem Prüfmedikament befragt. Ihr Prüfarzt kann Sie auch darum bitten, sich weiteren, von ihm als notwendig befundenen Körper- und Laboruntersuchungen zu unterziehen.

Versicherung

Als Teilnehmer an dieser klinischen Prüfung besteht für Sie der gesetzlich vorgeschriebene Versicherungsschutz (Personenschadenversicherung nach dem Arzneimittelgesetz, maximale Deckungssumme 500.000 € pro Teilnehmer), der alle Schäden abdeckt, die an Ihrem Leben oder Ihrer Gesundheit durch die an Ihnen durchgeführten Maßnahmen der klinischen Prüfung verursacht werden können; ausgenommen sind genetische Schäden. Die Versicherung wurde für Sie bei der Gerling Allgemeine Versicherungs AG (Versicherungsschein-Nr. 70-005878165-6) abgeschlossen. Die Kontaktperson in Deutschland ist Angelika Beck, Gerling Allgemeine Versicherungs-AG, GIS-Haftpflicht/Markmanagement 2-2, Postfach 10 15 44, 50587 Köln, Tel.: 0221-144-7943, PC-Fax: 0221-144-607943. Im Schadensfall können Sie sich direkt an den Versicherer wenden und Ihre Ansprüche selbständig geltend machen. Um den Versicherungsschutz nicht zu gefährden, dürfen Sie sich während der Dauer der klinischen Prüfung einer anderen medizinischen Behandlung nur im Einvernehmen mit Ihrem behandelnden Prüfarzt unterziehen; ausgenommen davon sind Notfälle. Dies gilt auch für die zusätzliche Einnahme von Medikamenten. Auch müssen Sie eine Gesundheitsschädigung, die als Folge der klinischen Prüfung eingetreten sein könnte, dem behandelnden Prüfarzt und der oben genannten Versicherungsgesellschaft unverzüglich mitteilen.

Aufwandsentschädigung

Um Ihnen den durch die Studienteilnahme entstehenden Aufwand (z.B. Zeit, Fahrtkosten) zu ersetzen, erhalten Sie eine Unkostenpauschale.
Probanden **ohne** Pockenimpfung in der Vorgeschichte erhalten 100 € *pro Besuch* für die Besuche 1, 3, 5 und 6 und 50 € *pro Besuch* für die Besuche 2 und 4. Dies ergibt für die Teilnahme an der gesamten klinischen Prüfung *500 €.*
Probanden **mit** Pockenimpfung in der Vorgeschichte erhalten 100 € *pro Besuch* für die Besuche 1, 3 und 4 und 50 € *pro Besuch* für den Besuch 2. Dies ergibt für die Teilnahme an der gesamten klinischen Prüfung *350 €.*
Vom Prüfarzt veranlasste zusätzliche studienbezogene Besuchstermine (z.B. beim Kardiologen) werden mit 50 € pro Besuch entschädigt.

Rahmenbedingungen

Die Studie wird entsprechend der Deklaration von Helsinki des Weltärztebundes, sowie den Vorschriften des deutschen Arzneimittelgesetzes und den Richtlinien zu „Good Clinical Practice" durchgeführt. Diese Dokumente können jeweils bei Ihrem Prüfarzt eingesehen werden.
An einer weiteren klinischen Prüfung für ein anderes, noch nicht zugelassenes Medikament oder eine in der Erprobung befindliche medizinische Maßnahme dürfen Sie nicht gleichzeitig teilnehmen.

Harrison Clinical Research / Clinical Research Unit
Gesellschaft für klinische Forschung mbH

Albrechtsraße 43
80636 München

Medizinischer Leiter: Dr. med. S. de la Motte

Telefonnummer: 0800-8063680 (gebührenfrei)
Notfallnummer: 0163-8588599

Internet: www.harrison.de

Harrison Clinical Research

Harrison

Einverständniserklärung

Teilweise randomisierte, teilweise doppelblinde, placebo-kontrollierte Phase II Nichtunterlegenheitsstudie zur Bewertung der Immunogenität und Sicherheit von ein und zwei Dosen eines Pockenimpfstoffs bei 18-55 Jahre alten gesunden Probanden

Name des Probanden (in Druckbuchstaben): ________________________________

Geburtsdatum: |__|__||__|__||__|__|__|__|
TAG MONAT JAHR

Probanden-Nummer: |__|__|__| Screening-Nummer: |__|__|__|__|

Ich erkläre freiwillig meine Einwilligung, an dieser klinischen Studie teilzunehmen und stimme zu, mit dem Prüfarzt vertrauensvoll zusammenzuarbeiten und ihn sofort über das Auftreten irgendwelcher unerwarteten Symptome zu informieren.

Mir ist bekannt, dass ich die Untersuchung jederzeit ohne Angaben von Gründen abbrechen kann, und dass dies keinerlei Einfluss auf meine weitere Behandlung oder medizinische Versorgung hat.

Ich bin durch den Arzt / die Ärztin, Herrn / Frau. .... in einem persönlichen Gespräch ausführlich und verständlich über das Prüfpräparat, mögliche Belastungen und Risiken sowie über Wesen, Bedeutung und Tragweite der klinischen Prüfung, die bestehende Versicherung sowie die sich für mich daraus ergebenden Anforderungen aufgeklärt worden.

Ich habe den Text der Probandeninformation und Einverständniserklärung gelesen und verstanden.

Aufgetretene Fragen wurden mir vom Prüfarzt verständlich und ausreichend beantwortet. Ich hatte auch ausreichend Zeit, mich für eine Studienteilnahme zu entscheiden.

Eine Kopie der Probandeninformation und Einverständniserklärung sowie der Versicherungsbedingungen habe ich erhalten. (Das Original verbleibt beim Prüfarzt).

NUR VOM PROBANDEN AUSZUFÜLLEN!

____
Datum und Unterschrift des Probanden

____
Datum und Unterschrift des **aufklärenden Prüfarztes**

Ich habe verstanden, dass es für die Impfung und deren Risiken derzeit keine medizinische Begründung gibt, außer meinem persönlichen Sicherheitsbedürfnis gegenüber Bio-Waffen-Angriffen und dem Nutzen für die Forschung.

NUR VOM PROBANDEN AUSZUFÜLLEN!

____
Datum und Unterschrift des Probanden

Harrison Clinical Research / Clinical Research Unit
Gesellschaft für klinische Forschung mbH

Albrechtsraße 43
80636 München

Medizinischer Leiter: Dr. med. S. de la Motte

Telefonnummer: 0800-8063680 (gebührenfrei)
Notfallnummer: 0163-8588599

Internet: www.harrison.de

Harrison Clinical Research

Harrison

Datenschutzerklärung und Einwilligung zum Datenschutz

Name des Probanden (in Druckbuchstaben): ____________________

Geburtsdatum: |__|__||__|__||__|__|__|__|
TAG MONAT JAHR

Probanden-Nummer: |__|__|__| Screening-Nummer: |__|__|__|__|

Datenschutzerklärung

Teilweise randomisierte, teilweise doppelblinde, placebo-kontrollierte Phase II Nichtunterlegenheitsstudie zur Bewertung der Immunogenität und Sicherheit von ein und zwei Dosen eines Pockenimpfstoffs bei 18-55 Jahre alten gesunden Probanden

Mir ist bekannt, dass im Rahmen dieser klinischen Prüfung personenbezogene Daten, insbesondere medizinische Befunde, über mich erhoben, gespeichert und ausgewertet werden sollen. Die Verwendung der Angaben über meine Person und meine Gesundheit erfolgt nach gesetzlichen Bestimmungen und setzt vor der Teilnahme an der klinischen Prüfung folgende freiwillig abgegebene Einwilligungserklärung voraus, d.h. ohne die nachfolgende Einwilligung kann ich nicht an der klinischen Prüfung teilnehmen.

Einwilligung zum Datenschutz

1. Ich erkläre mich damit einverstanden, dass im Rahmen dieser klinischen Prüfung erhobene Daten, insbesondere Angaben über meine Person und meine Gesundheit, in Papierform und auf elektronischen Datenträgern am Prüfzentrum in München aufgezeichnet und hinreichend gesichert aufbewahrt werden. Soweit erforderlich, dürfen die erhobenen Daten allerdings grundsätzlich nur pseudonymisiert (verschlüsselt) weiter gegeben werden:

a) an den Sponsor Bavarian Nordic oder eine von diesem beauftragte Stelle zum Zwecke der wissenschaftlichen Auswertung im Rahmen dieser Studie
b) im Falle eines Antrags auf Zulassung: an den Antragsteller und die für die Zulassung zuständige Behörde (z.B. Paul-Ehrlich-Institut, Zulassungsbehörde der USA)

c) im Falle unerwünschter Ereignisse: an den Sponsor Bavarian Nordic, an die jeweils zuständige Ethik-Kommission und die zuständige Bundesoberbehörde (Paul-Ehrlich-Institut, Langen) sowie von dieser an die Europäische Datenbank.

2. Außerdem erkläre ich mich damit einverstanden, dass autorisierte und zur Verschwiegenheit verpflichtete Beauftragte des Sponsors Bavarian Nordic sowie die zuständigen inländischen und ausländischen Überwachungsbehörden in meine beim Prüfarzt vorhandenen personenbezogenen Daten, insbesondere meine Gesundheitsdaten, Einsicht nehmen, soweit dies für die Überprüfung der ordnungsgemäßen Durchführung der Studie notwendig ist. Für diese Maßnahme entbinde ich den Prüfarzt von der ärztlichen Schweigepflicht.

3. Die Einwilligung zur Erhebung und Verarbeitung meiner personenbezogenen Daten, insbesondere der Angaben über meine Gesundheit, ist unwiderruflich. Ich bin bereits darüber aufgeklärt worden, dass ich jederzeit die Teilnahme an der klinischen Prüfung beenden kann. Im Fall eines solchen Widerrufs meiner Einwilligung, an der Studie teilzunehmen, erkläre ich mich damit einverstanden, dass die bis zu diesem Zeitpunkt gespeicherten Daten ohne Namensnennung weiterhin verwendet werden dürfen, soweit dies erforderlich ist, um

a) Wirkungen des zu prüfenden Arzneimittels festzustellen,
b) sicherzustellen, dass meine schutzwürdigen Interessen nicht beeinträchtigt werden,
c) der Pflicht zur Vorlage vollständiger Zulassungsunterlagen zu genügen.

4. Ich erkläre mich damit einverstanden, dass meine Daten nach Beendigung oder Abbruch der Prüfung mindestens zehn Jahre aufbewahrt werden, wie es die Vorschriften über die klinische Prüfung von Arzneimitteln bestimmen. Danach werden meine personenbezogenen Daten gelöscht, soweit nicht gesetzliche, satzungsmäßige oder vertragliche Aufbewahrungsfristen entgegenstehen.

5. Ich bin über folgende gesetzliche Regelung informiert: Falls ich meine Einwilligung, an der Studie teilzunehmen, widerrufe, müssen alle Stellen, die meine personenbezogenen Daten, insbesondere Gesundheitsdaten gespeichert haben, unverzüglich prüfen, inwieweit die gespeicherten Daten für die in Nr. 3 a) bis c) genannten Zwecke noch erforderlich sind. Nicht mehr benötigte Daten sind unverzüglich zu löschen.

Name ---

Datum ---

Unterschrift ---

16.1.4 Study personnel and curricula vitae

List of names and addresses

Principal Investigator:	Prof. Frank von Sonnenburg<br>Section of International Medicine & Public Health<br>Department of Infectious Diseases and Tropical Medicine<br>Georgenstrasse 5<br>80799 Munich<br>Germany<br>Phone: +49-89-2180 3830<br>Fax: +49-89-33 60 38
Investigator:	Dr. Stephan de la Motte<br>Harrison Clinical Research Deutschland GmbH<br>Albrechtstr. 14<br>80636 Munich, Germany<br>Phone: +49.89 126680-2509<br>Fax: +49 89-126680-2444
Sponsor:	Bavarian Nordic A/S<br>Bøgeskovvej 9<br>DK-3490 Kvistgård<br>Denmark
Project Coordinator	Alfred von Krempelhuber, Ph.D., Bavarian Nordic GmbH, Martinsried, Germany
Medical Monitor	Garth Virgin, M.D., Bavarian Nordic GmbH, Martinsried, Germany
Chief Scientific Officer	Paul Chaplin, Ph.D., Bavarian Nordic GmbH, Martinsried, Germany
GCP Specialist	Robert Semich, Ph.D., Bavarian Nordic GmbH, Martinsried, Germany
Contract Research Organisation:	Harrison Clinical Research Deutschland GmbH<br>Albrechtstr. 14, 80636 Munich, Germany<br>Phone: +49 89-126680-0<br>Fax: +49 89-126680-3444<br>www.harrisonclinical.com
Project Manager	Marianne Sievers, Harrison Clinical Research Deutschland GmbH, Munich, Germany
Statistician:	Joachim Klinger, qualified statistician, Harrison Clinical Research Deutschland GmbH, Munich, Germany
Report writing:	Caterina Milazzo, PhD, Harrison Clinical Research Deutschland GmbH, Munich, Germany
Centralized ECG Provider:	Martin Ptacek-Zellmeier, ganiMed GmbH, Munzinger Strasse 1, 79111 Freiburg, Germany
Cardiologist	Eva Zitzmann-Roth, MD, Richard-Strauss-Str. 56, 81677 Munich, Germany

Laboratories:	Holger Schöneborn, PhD, INTERLAB GmbH<br>Bayerstrasse 53, 80335 Munich, Germany
	Thomas Meyer, Ph.D, Bavarian Nordic GmbH<br>Fraunhoferstrasse 13, 82152 Martinsried, Germany
Routine haematology and clinical chemistry:	Laboratory Dr. Becker, Dr. Olgemöller, Dr. Hermeyer<br>Führichstraße 70<br>81671 Munich, Germany

Curriculum Vitae

Harrison Clinical Research

Harrison

**NAME**	Dr. Stephan de la Motte
**POSITION AT HCR**	Chief Medical Officer / Head of Clinical Unit
**Date of medical license:**	03 July 1985

WORK EXPERIENCES

2001 - present	Chief Medical Officer at Harrison Clinical Research, Munich
2001	Project manager at the Pharmalog Institute, Munich
1993 – 2000	Scientific Documentation Director at Harrison Clinical Research, Munich
1990 - 1992	Medical Assistant in Anesthesiology at the Federal Armed Forces Hospital, Munich
	Teaching medical students in physiology, University of Munich
1988 - 1990	Medical Assistant in Pharmacology & Toxicology at the Federal Armed Forces Medical College, Institute for Pharmacology and Toxicology, Munich
	Teaching physicians, dentists, pharmacists, paramedics, Federal Armed Forces Medical College
1988-1993	Author for software journals (test reports, trainings, book reviews, etc.)
1987 - 1988	Assistant in Neurophysiology at the Institute for Neurophysiology, University of Munich
1985 - 1987	Air Force Staff Surgeon at the German Air Force, Freising

AREAS OF THERAPEUTIC EXPERIENCE

General medicine, clinical pharmacology, pharmacology and toxicology, anesthesia, internal medicine, psychiatry, neurosurgery, neurophysiology, military medicine

QUALIFICATIONS

1999	Certificate Medical Biometrics
1997 - 1999	Post-graduate courses in medical biometrics
1990	Post-graduate courses in emergency medicine
1987-1988	Dissertation at the Institute for Neurophysiology at University of Munich<br>1988: Medical doctorate
1985	State Exam in medicine and Physician's Practice License
1977-1985	Study of medicine, University of Munich

POST-GRADUATE EDUCATIONS

2004	Clinical Trials Directive – Implementation in Germany, Bonn
2004	Adverse Reaction Reporting, Nürnberg
2004	EU Clinical Trials Directive – Status of Implementation, Munich
2003	MedDRA Coding, Munich
1990	Basic training on radiation protection, Munich
1990	Special training on radiation protection when handling open radioactive material, Munich
1990	Special training on radiation protection during X-ray examinations, Munich
1990	Course on occupational medicine - part B, Munich
1988	Advanced Cardiac Life Support Provider Course (ACLS), American Heart Associaton / Military Training Network-Europe, Augsburg

Curriculum Vitae

1983 - 1984	Flight physiology, special questions on practical physiology (24 hours), Munich University<br>Sports medicine (40 hours), University Munich<br>Tropical medicine (60 hours), University Munich
**LANGUAGES**	German (mother tongue), English, Latin
**MEMBERSHIPS**	German Society for Medical Informatics, Biometrics and Epidemiology (GMDS), German Society for Applied Human Pharmacology (AGAH), German Society for Pharmaceutical Medicine (DGPharMed), German Society for Clinical Pharmacology and Therapy (GKPharm), Bavarian Medical Doctors Association (BLÄK),"Ärzlicher Kreis- und Bezirksverband München" (ÄKBV)

PUBLICATIONS AND PRESENTATIONS

2004	*de la Motte S, Gianella-Borradori A.*, Pharmacokinetic model of R-roscovitine and its metabolite in healthy male subjects., Int J Clin Pharmacol Ther 2004; 42:232-239.
2002	*de la Motte S, Plum J, Passlick-Deetjen J, Grabensee B.*, One-compartment model for amino acids and other biological molecules in peritoneal dialysis.<br>Int J Clin Pharmacol Ther 2002; 40:60-68.
2001	*de la Motte S, Klinger J, Kefer G, King T, Harrison F.*, Pharmacokinetics of human growth hormone administered subcutaneously with two different injection systems, Arzneim.-Forschung / Drug Res 2001; 51:613-617.
2001	*Harrison F, Egilmez MV, de la Motte S, Eierman D.*, Ioequivalence determination of two ofloxacin (400 mg) tablets manufactured in Turkey and Germany, FABAD J Pharm Sci 2001; 26:173-177.
2000	*Barenbrock M, Hausberg M, Matzkies F, de la Motte S, Schaefer RM.*, Effects of bicarbonate- and lactate-buffered replacement fluids on cardiovascular outcome in CVVH patients, Kidney Int 2000; 58:1751-1757.
1999	*de la Motte S, Alberich X, Harrison F.*, Non-linear pharmacokinetic modelling of intravenous elgodipine., Int J Clin Pharmacol Ther 1999; 37:20-27.
1998	*Pfaffenrath V, de la Motte S, Harrison F, Rüthning C.*, Effects of pentaerithritol tetranitrate, isosorbide mononitrate and placebo on the headache and on the ability to work of healthy volunteers, Arzneim.-Forschung / Drug Res 1998; 48:646-650.
1997	*de la Motte S, Böse-O'Reilly S, Heinisch M, Harrison F.*<br>Double-blind comparison of a preparation of pectin/chamomile extract and placebo in children with diarrhea. Arzneim.-Forschung / Drug Res 1997; 47:1247-1249.
1996	*Unkauf M, Rehn D, Klinger J, de la Motte S, Großmann K.*, Investigation of the efficacy of oxerutins compared to placebo in patients with chronic venous insufficiency treated with compression stockings, Arzneim.-Forschung / Drug Res 1996; 46:478-482.
1996	*Sridhara BS, Senior Roxy, Anagnostou E, de la Motte S, Harrison FJJ, Raftery EB, Lahiri A.*, Saterinone: A new dual-action drug in the acute management of chronic heart failure, Am J Ther 1996; 3:597-601
1996	*Silke B, de la Motte S, Spiers P, Herity NA, Drake M, Kelly J, Harrison FJ.* Hemodynamic effects of intravenous elgodipine in coronary artery disease during rest and exercise, and basic pharmacokinetic parameters. Cardiovasc Drugs Ther 1996; 10:573-580.

1995	*Heirler F, de la Motte S, Popp W.*, Influence of metronidazole and tamoxifen in a case of otherwise untreated ulcerous mamma carcinoma, Eur J Gynaecol Oncol 1995; 6:448-452.
1995	*Heirler F, de la Motte S, Popp W.*, Influence of metronidazole and tamoxifen in a case of otherwise untreated ulcerous mamma carcinoma, Eur J Gynaecol Oncol 1995; 6:448-452.
1991	*de la Motte S, Szinicz L.*, Effects of Pyridinium,1-[[(4-Carbamoyl-Pyridinio) Methoxy] Methyl]-2-(Hydroxyimino-Methyl) Dichloride Monohydrate (HI6) and Atropine on the Circulation and Respiration of Anesthetized Guinea Pigs., In: Chambers et al (eds.), Recent Developments in Toxicology: Trends, Methods and Problems. Springer Verlag, Berlin, 1991, pp. 266-268.
1989	*Fritz N, Illert M, de la Motte S, Reeh P, Saggau P.*, Pattern of monosynaptic Ia connections in the cat forelimb., J Physiol 1989; 419:321-351.
General	10 abstracts, 3 articles in congress proceedings, 13 presentations without abstract.

27 JUL 2005 [signature]

Date Signature

Curriculum Vitae

Professor FRANK (FRANZ-JOSEF FALKNER) von SONNENBURG
Doctor of Internal Medicine and Tropical Medicine

1 **Personal Data**

Date of birth:	May 8, 1946
Place of birth:	Munich
Nationality:	German
Civil status:	married, 2 children
Languages:	German: mother tongue<br>English: fluent<br>French: working knowledge<br>Spanish: basic knowledge
Hobbies:	Chess, sailing and wind and kite surfing, skiing

2 **Summary**

After studies in medicine at the universities of Cologne and Munich, license to practice medicine. Subsequently research and clinical practice in Internal Medicine, specialization in Infectious Diseases and Tropical Medicine. License as a Doctor in Internal Medicine and Doctor in Tropical Medicine.

1987 further training as "Master of Public Health/Epidemiology" with specialization in the field of Infectious Diseases Epidemiology.

Currently, Deputy Director of the Department of Infectious Diseases and Tropical Medicine (Director: Prof. Dr. med. Th. Löscher) of the University of Munich. Head of the Section International Medicine and Public Health. Main fields of experience in infectious diseases: HIV/AIDS/STD, Malaria, Lyme disease, travelers' diarrhea, adult and travel medicine related vaccines. Extensive experience in developing countries and in international working environments (WHO, European Community), experience in working groups and steering groups, appointed member of German advisory board on vaccine recommendations.

3 **Education and Academic Titles**

1967	Abitur (permitting university entrance) in Fürstenfeldbruck, Germany
1967 - 1972	Studies in Medicine at the Universities of Cologne and Munich, Germany Diploma: "Staatsexamen" (state examination in medicine)
1972 - 1975	Medical doctorate at the urological clinic of the University of Munich: "The antibiotic sensitivity of infectious agents of the uro-genital tract at the Urologic Clinic of the University of Munich"
1975	"Approbation" (medical licence)
1984	Licence to practice as a doctor in Internal Medicine
1985	Licence to practice as a doctor in Tropical Medicine
1986	Master of Public Health/ Epidemiology with specialisation in the field of the epidemiology of infectious diseases at the School of Public Health in Honolulu, USA, supported by the Federal Ministry of Health
1991	PhD in Tropical Medicine, University of Munich
1997	Full professor at the University of Munich

4 **Professional Experience Record**

1992 - to date Deputy Director, Institute of Infectious Diseases and Tropical Medicine of the University of Munich, Head of the Section International Medicine and Public Health

- Member of the national advisory board on vaccinations (STIKO, appointed by the German Minister of Health)
- PI and /or supervisor in a number of phase I-III studies on adult vaccines in cooperation with the industry. Member of safety board of various vaccine trials
- Site director for the GeoSentinel program in collaboration with the International Society of Travel Medicine and the Centers of Disease Control

- PI of the international study on travelers' diarrhea
- Supervision of the HIV/AIDS/STD intervention site in the Mbeya region, Tanzania (founded in 1987, see below). The intervention program is linked with research on HIV subtypes and double infection. The program is supported by the EU and conducted in collaboration with the Walter-Reed-Institute. A cohort is developed for further phase II and III HIV vaccine trials.
- Advisor for the German Government on AIDS/STD research and intervention programs in developing countries. Member of the AIDS-Enquête- Commission of the German Government with regard to the AIDS epidemic in developing countries. Team leader of a "Joint Evaluation" (on behalf of BMZ and WHO) for assessing the country-specific component of the "Global Programme on AIDS" (GPA) and of biomedical research activities.
- Reviewer of WHO supported Programs (e.g. Malaria Control Program in Cambodia)
- Head of the research program "Malaria Control within the Framework of Primary Health Care" in Mali, Mauritania, Uganda and Vietnam supported by the German Government. Close cooperation with and additional promotion through the WHO programs CTD and TDR
- Co-ordination and supervision of several AIDS/STD Control Projects in the Caribbean and Africa
- Cooperation with WHO and UNAIDS in the areas Sentinel Surveillance of HIV infection, STD Control and Malaria Control
- Teaching of students in epidemiology and control of infectious diseases and health systems in developing countries
- Academic supervision of doctoral students
- Examination of medical students

1988 - 1991 Medical Officer (P5), World Health Organisation, Global Programme on AIDS (director: J. Mann. M. Merson), Biomedical Research Unit (J. Esparza). Main fields of work:

- Academic supervision of doctoral students
- Examination of medical students Identification of research priorities on opportunistic infections of HIV infected persons. Development and promotion of research on the interaction of HIV infection and tropical diseases
- Participation in the development of a WHO staging system for HIV infected persons
- Participation in WHO's first reflections on HIV vaccine trials in endemic countries
- Developing projections of AIDS cases and change of mortality due to AIDS in Tanzania

1986 - 1987 Research fellow, later assistant professor at the Schools of Public Health in Hawaii, USA

1987 Setting up of a comprehensive regional AIDS Control Programme in Mbeya Region (Tanzania), a cooperation project of the Ministry of Health of Tanzania, the German government and the Department of Infectious Diseases and Tropical Medicine of the University of Munich with the aim to conduct operational research on the HIV/AIDS/STD-epidemic in developing countries

1981 - 1985 Assistant Professor, Institute of Infectious Diseases and Tropical Medicine, University of Munich Duties:

- Planning and implementation of a Schistosomiasis Research Project on the Philippines (1981-1982)
- Counselling and treatment of travellers with tropical diseases
- I/c of the serological routine laboratory of the institute
- Teaching of medical students

1974 - 1980 Internship and Senior House Officer at various hospitals in Munich, Berlin and New York, practicing internal medicine with focus on infectious diseases.

Curriculum Vitae

Professor FRANK (FRANZ JOSEF FALKNER) von SONNENBURG
Doctor of Internal Medicine and Tropical Medicine

5 **Publications**

91 peer reviewed publications (mainly infectious diseases and epidemiology) and 67 abstracts in international conferences.

6 **Membership of Professional Bodies**

- International AIDS Society
- German Society of Tropical Medicine
- Austrian Society of Tropical Medicine and Parasitic Disease
- American Society of Tropical Medicine and Hygiene
- German Society of Infectious Diseases
- German Society of Epidemiology
- International Society of Travel Medicine, President Elect

7 **Fields of Research**

- Epidemiological and molecular epidemiological research on HIV/AIDS/STD (Tanzania, Uganda, Namibia, Jamaica)
- Study on the development of semi-immunity against Malaria in different epidemiological strata and P. falciparum strain variations (Uganda, Mali, Vietnam)
- Diagnosis and treatment of imported Kala-Azar
- Treatment of complicated P. falciparum Malaria of non-immune travellers
- Phase III vaccine trials (on Hepatitis A/B and Typhoid fever and other vaccines in adults)
- Incidence and prevalence of Lyme Disease in Germany
- Incidence and causes of travellers' diarrhea
- Study on the epidemiology of Echinococcosis in Bavaria
- Treatment of echinococcosis with mebendazole
- IgE-specific antibodies as a tool for monitoring intervention programmes on Schistosoma japonicum (Philippines)

Frank v. Sonnenburg

Munich, September 28, 2005

confirmed
April 26, 2006

v. [illegible]

16.1.5 Signatures of relevant study personnel

Please refer to Section 1 of this report for signatures.

16.1.6 Listing of subjects receiving the test drug(s)/investigational product(s) from specific batches, where more than one batch was used

The following subjects received the following batch numbers:

Subjects in group 1 and 4 received batch no. 170505 (test product).
Subjects in group 2 received batch no. 170505 (test product) and batch no. 030505 (placebo).
Subjects in group 3 received batch no. 030505 (placebo).

16.1.7 Randomization codes

Subject randomisation list for BN study POX-MVA-005 (P031363)
Seed= 18655143

Container	Subject Randomization Number	Treatment Sequence	
1	1001	Placebo	Placebo
	1002	MVA	Placebo
	1003	Placebo	Placebo
	1004	MVA	MVA
	1005	MVA	MVA
	1006	Placebo	Placebo
	1007	MVA	MVA
	1008	MVA	Placebo
	1009	MVA	Placebo
2	1010	MVA	Placebo
	1011	MVA	MVA
	1012	MVA	MVA
	1013	MVA	MVA
	1014	Placebo	Placebo
	1015	Placebo	Placebo
	1016	MVA	Placebo
	1017	Placebo	Placebo
	1018	MVA	Placebo
3	1019	MVA	MVA
	1020	MVA	Placebo
	1021	Placebo	Placebo
	1022	MVA	MVA
	1023	Placebo	Placebo
	1024	MVA	MVA
	1025	MVA	Placebo
	1026	Placebo	Placebo
	1027	MVA	Placebo
4	1028	Placebo	Placebo
	1029	MVA	Placebo
	1030	MVA	MVA
	1031	Placebo	Placebo
	1032	Placebo	Placebo
	1033	MVA	MVA
	1034	MVA	Placebo
	1035	MVA	MVA
	1036	MVA	Placebo
5	1037	MVA	MVA
	1038	MVA	MVA
	1039	Placebo	Placebo
	1040	MVA	MVA
	1041	MVA	Placebo
	1042	MVA	Placebo
	1043	Placebo	Placebo
	1044	MVA	Placebo
	1045	Placebo	Placebo
6	1046	MVA	MVA
	1047	Placebo	Placebo
	1048	MVA	Placebo
	1049	Placebo	Placebo
	1050	Placebo	Placebo
	1051	MVA	Placebo
	1052	MVA	MVA
	1053	MVA	MVA
	1054	MVA	Placebo

Subject randomisation list for BN study POX-MVA-005 (P031363)
Seed= 18655143

Container	Subject Randomization Number	Treatment Sequence	
7	1055	MVA	MVA
	1056	Placebo	Placebo
	1057	Placebo	Placebo
	1058	Placebo	Placebo
	1059	MVA	MVA
	1060	MVA	MVA
	1061	MVA	Placebo
	1062	MVA	Placebo
	1063	MVA	Placebo
8	1064	MVA	Placebo
	1065	MVA	Placebo
	1066	MVA	Placebo
	1067	Placebo	Placebo
	1068	Placebo	Placebo
	1069	Placebo	Placebo
	1070	MVA	MVA
	1071	MVA	MVA
	1072	MVA	MVA
9	1073	MVA	Placebo
	1074	Placebo	Placebo
	1075	MVA	Placebo
	1076	MVA	Placebo
	1077	MVA	MVA
	1078	Placebo	Placebo
	1079	MVA	MVA
	1080	MVA	MVA
	1081	Placebo	Placebo
10	1082	MVA	Placebo
	1083	MVA	MVA
	1084	MVA	Placebo
	1085	MVA	Placebo
	1086	Placebo	Placebo
	1087	Placebo	Placebo
	1088	Placebo	Placebo
	1089	MVA	MVA
	1090	MVA	MVA
11	1091	Placebo	Placebo
	1092	Placebo	Placebo
	1093	MVA	Placebo
	1094	MVA	Placebo
	1095	Placebo	Placebo
	1096	MVA	MVA
	1097	MVA	Placebo
	1098	MVA	MVA
	1099	MVA	MVA
12	1100	Placebo	Placebo
	1101	MVA	Placebo
	1102	MVA	Placebo
	1103	MVA	MVA
	1104	MVA	MVA
	1105	MVA	MVA
	1106	Placebo	Placebo
	1107	MVA	Placebo
	1108	Placebo	Placebo

Subject randomisation list for BN study POX-MVA-005 (P031363)
Seed= 18655143

Container	Subject Randomization Number	Treatment Sequence	
13	1109	MVA	MVA
	1110	MVA	Placebo
	1111	MVA	Placebo
	1112	MVA	Placebo
	1113	MVA	MVA
	1114	Placebo	Placebo
	1115	MVA	MVA
	1116	Placebo	Placebo
	1117	Placebo	Placebo
14	1118	MVA	MVA
	1119	MVA	Placebo
	1120	Placebo	Placebo
	1121	Placebo	Placebo
	1122	MVA	MVA
	1123	MVA	Placebo
	1124	Placebo	Placebo
	1125	MVA	MVA
	1126	MVA	Placebo
15	1127	MVA	Placebo
	1128	MVA	Placebo
	1129	MVA	Placebo
	1130	Placebo	Placebo
	1131	MVA	MVA
	1132	MVA	MVA
	1133	Placebo	Placebo
	1134	Placebo	Placebo
	1135	MVA	MVA
16	1136	Placebo	Placebo
	1137	MVA	MVA
	1138	MVA	Placebo
	1139	Placebo	Placebo
	1140	MVA	MVA
	1141	MVA	Placebo
	1142	MVA	Placebo
	1143	Placebo	Placebo
	1144	MVA	MVA
17	1145	MVA	MVA
	1146	MVA	MVA
	1147	Placebo	Placebo
	1148	Placebo	Placebo
	1149	Placebo	Placebo
	1150	MVA	Placebo
	1151	MVA	MVA
	1152	MVA	Placebo
	1153	MVA	Placebo
18	1154	MVA	Placebo
	1155	MVA	MVA
	1156	Placebo	Placebo
	1157	MVA	MVA
	1158	Placebo	Placebo
	1159	MVA	Placebo
	1160	MVA	Placebo
	1161	Placebo	Placebo
	1162	MVA	MVA

Subject randomisation list for BN study POX-MVA-005 (P031363)
Seed= 18655143

Container	Subject Randomization Number	Treatment Sequence	
19	1163	MVA	Placebo
	1164	Placebo	Placebo
	1165	MVA	Placebo
	1166	Placebo	Placebo
	1167	MVA	MVA
	1168	Placebo	Placebo
	1169	MVA	MVA
	1170	MVA	Placebo
	1171	MVA	MVA
20	1172	MVA	MVA
	1173	Placebo	Placebo
	1174	MVA	Placebo
	1175	MVA	MVA
	1176	Placebo	Placebo
	1177	MVA	MVA
	1178	MVA	Placebo
	1179	MVA	Placebo
	1180	Placebo	Placebo
21	1181	MVA	Placebo
	1182	MVA	MVA
	1183	Placebo	Placebo
	1184	MVA	MVA
	1185	Placebo	Placebo
	1186	MVA	Placebo
	1187	MVA	Placebo
	1188	MVA	MVA
	1189	Placebo	Placebo
22	1190	MVA	Placebo
	1191	MVA	MVA
	1192	MVA	Placebo
	1193	Placebo	Placebo
	1194	MVA	Placebo
	1195	MVA	MVA
	1196	Placebo	Placebo
	1197	Placebo	Placebo
	1198	MVA	MVA
23	1199	MVA	MVA
	1200	Placebo	Placebo
	1201	MVA	MVA
	1202	MVA	Placebo
	1203	MVA	Placebo
	1204	Placebo	Placebo
	1205	MVA	Placebo
	1206	MVA	MVA
	1207	Placebo	Placebo
24	1208	MVA	MVA
	1209	MVA	Placebo
	1210	MVA	Placebo
	1211	MVA	MVA
	1212	Placebo	Placebo
	1213	MVA	MVA
	1214	MVA	Placebo
	1215	Placebo	Placebo
	1216	Placebo	Placebo

Subject randomisation list for BN study POX-MVA-005 (P031363)
Seed= 18655143

Container	Subject Randomization Number	Treatment Sequence	
25	1217	MVA	Placebo
	1218	MVA	MVA
	1219	Placebo	Placebo
	1220	Placebo	Placebo
	1221	MVA	MVA
	1222	MVA	Placebo
	1223	Placebo	Placebo
	1224	MVA	Placebo
	1225	MVA	MVA
26	1226	MVA	Placebo
	1227	MVA	Placebo
	1228	MVA	Placebo
	1229	MVA	MVA
	1230	MVA	MVA
	1231	MVA	MVA
	1232	Placebo	Placebo
	1233	Placebo	Placebo
	1234	Placebo	Placebo
27	1235	MVA	MVA
	1236	Placebo	Placebo
	1237	MVA	Placebo
	1238	MVA	Placebo
	1239	MVA	Placebo
	1240	Placebo	Placebo
	1241	MVA	MVA
	1242	MVA	MVA
	1243	Placebo	Placebo
28	1244	MVA	Placebo
	1245	MVA	Placebo
	1246	Placebo	Placebo
	1247	MVA	MVA
	1248	Placebo	Placebo
	1249	MVA	MVA
	1250	MVA	MVA
	1251	Placebo	Placebo
	1252	MVA	Placebo
29	1253	MVA	Placebo
	1254	MVA	Placebo
	1255	Placebo	Placebo
	1256	MVA	Placebo
	1257	MVA	MVA
	1258	MVA	MVA
	1259	MVA	MVA
	1260	Placebo	Placebo
	1261	Placebo	Placebo
30	1262	MVA	Placebo
	1263	MVA	Placebo
	1264	Placebo	Placebo
	1265	MVA	Placebo
	1266	MVA	MVA
	1267	Placebo	Placebo
	1268	MVA	MVA
	1269	Placebo	Placebo
	1270	MVA	MVA

Subject randomisation list for BN study POX-MVA-005 (P031363)
Seed= 18655143

Container	Subject Randomization Number	Treatment Sequence	
31	1271	Placebo	Placebo
	1272	MVA	Placebo
	1273	MVA	MVA
	1274	MVA	Placebo
	1275	MVA	MVA
	1276	MVA	Placebo
	1277	MVA	MVA
	1278	Placebo	Placebo
	1279	Placebo	Placebo
32	1280	MVA	MVA
	1281	MVA	Placebo
	1282	MVA	Placebo
	1283	MVA	MVA
	1284	Placebo	Placebo
	1285	Placebo	Placebo
	1286	Placebo	Placebo
	1287	MVA	MVA
	1288	MVA	Placebo
33	1289	MVA	MVA
	1290	Placebo	Placebo
	1291	MVA	MVA
	1292	MVA	Placebo
	1293	MVA	Placebo
	1294	Placebo	Placebo
	1295	MVA	Placebo
	1296	MVA	MVA
	1297	Placebo	Placebo
34	1298	MVA	MVA
	1299	MVA	Placebo
	1300	Placebo	Placebo
	1301	MVA	Placebo
	1302	MVA	Placebo
	1303	Placebo	Placebo
	1304	Placebo	Placebo
	1305	MVA	MVA
	1306	MVA	MVA
35	1307	MVA	Placebo
	1308	Placebo	Placebo
	1309	MVA	MVA
	1310	Placebo	Placebo
	1311	MVA	MVA
	1312	Placebo	Placebo
	1313	MVA	Placebo
	1314	MVA	MVA
	1315	MVA	Placebo
36	1316	MVA	MVA
	1317	MVA	MVA
	1318	MVA	MVA
	1319	Placebo	Placebo
	1320	Placebo	Placebo
	1321	MVA	Placebo
	1322	MVA	Placebo
	1323	MVA	Placebo
	1324	Placebo	Placebo

Subject randomisation list for BN study POX-MVA-005 (P031363)
Seed= 18655143

Container	Subject Randomization Number	Treatment Sequence	
37	1325	MVA	Placebo
	1326	Placebo	Placebo
	1327	Placebo	Placebo
	1328	MVA	Placebo
	1329	Placebo	Placebo
	1330	MVA	Placebo
	1331	MVA	MVA
	1332	MVA	MVA
	1333	MVA	MVA
38	1334	MVA	Placebo
	1335	MVA	Placebo
	1336	Placebo	Placebo
	1337	MVA	MVA
	1338	Placebo	Placebo
	1339	MVA	MVA
	1340	MVA	MVA
	1341	MVA	Placebo
	1342	Placebo	Placebo
39	1343	MVA	MVA
	1344	MVA	MVA
	1345	MVA	Placebo
	1346	MVA	Placebo
	1347	Placebo	Placebo
	1348	MVA	MVA
	1349	Placebo	Placebo
	1350	Placebo	Placebo
	1351	MVA	Placebo
40	1352	Placebo	Placebo
	1353	MVA	MVA
	1354	MVA	Placebo
	1355	Placebo	Placebo
	1356	MVA	MVA
	1357	Placebo	Placebo
	1358	MVA	MVA
	1359	MVA	Placebo
	1360	MVA	Placebo
41	1361	MVA	Placebo
	1362	Placebo	Placebo
	1363	MVA	Placebo
	1364	Placebo	Placebo
	1365	MVA	MVA
	1366	MVA	MVA
	1367	Placebo	Placebo
	1368	MVA	Placebo
	1369	MVA	MVA
42	1370	Placebo	Placebo
	1371	MVA	MVA
	1372	MVA	Placebo
	1373	MVA	Placebo
	1374	MVA	MVA
	1375	Placebo	Placebo
	1376	Placebo	Placebo
	1377	MVA	MVA
	1378	MVA	Placebo

Subject randomisation list for BN study POX-MVA-005 (P031363)
Seed= 18655143

Container	Subject Randomization Number	Treatment Sequence	
43	1379	MVA	Placebo
	1380	MVA	MVA
	1381	MVA	Placebo
	1382	MVA	MVA
	1383	MVA	MVA
	1384	Placebo	Placebo
	1385	Placebo	Placebo
	1386	Placebo	Placebo
	1387	MVA	Placebo
44	1388	Placebo	Placebo
	1389	MVA	MVA
	1390	MVA	Placebo
	1391	MVA	Placebo
	1392	MVA	Placebo
	1393	Placebo	Placebo
	1394	MVA	MVA
	1395	Placebo	Placebo
	1396	MVA	MVA
45	1397	Placebo	Placebo
	1398	MVA	Placebo
	1399	MVA	MVA
	1400	Placebo	Placebo
	1401	MVA	MVA
	1402	MVA	MVA
	1403	MVA	Placebo
	1404	Placebo	Placebo
	1405	MVA	Placebo
46	1406	Placebo	Placebo
	1407	MVA	MVA
	1408	MVA	MVA
	1409	Placebo	Placebo
	1410	MVA	Placebo
	1411	MVA	Placebo
	1412	MVA	Placebo
	1413	Placebo	Placebo
	1414	MVA	MVA
47	1415	MVA	Placebo
	1416	MVA	Placebo
	1417	MVA	MVA
	1418	MVA	MVA
	1419	Placebo	Placebo
	1420	MVA	Placebo
	1421	Placebo	Placebo
	1422	Placebo	Placebo
	1423	MVA	MVA
48	1424	MVA	MVA
	1425	MVA	Placebo
	1426	MVA	MVA
	1427	Placebo	Placebo
	1428	MVA	MVA
	1429	MVA	Placebo
	1430	MVA	Placebo
	1431	Placebo	Placebo
	1432	Placebo	Placebo

Subject randomisation list for BN study POX-MVA-005 (P031363)
Seed= 18655143

Container	Subject Randomization Number	Treatment Sequence	
49	1433	MVA	MVA
	1434	Placebo	Placebo
	1435	MVA	MVA
	1436	MVA	MVA
	1437	MVA	Placebo
	1438	MVA	Placebo
	1439	Placebo	Placebo
	1440	MVA	Placebo
	1441	Placebo	Placebo
50	1442	Placebo	Placebo
	1443	MVA	Placebo
	1444	MVA	MVA
	1445	MVA	MVA
	1446	Placebo	Placebo
	1447	MVA	Placebo
	1448	Placebo	Placebo
	1449	MVA	MVA
	1450	MVA	Placebo
51	1451	MVA	MVA
	1452	MVA	Placebo
	1453	MVA	MVA
	1454	Placebo	Placebo
	1455	MVA	Placebo
	1456	MVA	Placebo
	1457	Placebo	Placebo
	1458	Placebo	Placebo
	1459	MVA	MVA
52	1460	MVA	Placebo
	1461	MVA	MVA
	1462	MVA	MVA
	1463	MVA	Placebo
	1464	Placebo	Placebo
	1465	MVA	MVA
	1466	MVA	Placebo
	1467	Placebo	Placebo
	1468	Placebo	Placebo
53	1469	MVA	Placebo
	1470	MVA	MVA
	1471	MVA	MVA
	1472	MVA	Placebo
	1473	Placebo	Placebo
	1474	Placebo	Placebo
	1475	MVA	MVA
	1476	MVA	Placebo
	1477	Placebo	Placebo
54	1478	Placebo	Placebo
	1479	Placebo	Placebo
	1480	MVA	MVA
	1481	MVA	MVA
	1482	MVA	Placebo
	1483	MVA	Placebo
	1484	MVA	MVA
	1485	MVA	Placebo
	1486	Placebo	Placebo

Subject randomisation list for BN study POX-MVA-005 (P031363)
Seed= 18655143

Container	Subject Randomization Number	Treatment Sequence	
55	1487	MVA	MVA
	1488	Placebo	Placebo
	1489	MVA	Placebo
	1490	Placebo	Placebo
	1491	MVA	MVA
	1492	MVA	Placebo
	1493	MVA	MVA
	1494	MVA	Placebo
	1495	Placebo	Placebo
56	1496	Placebo	Placebo
	1497	MVA	MVA
	1498	Placebo	Placebo
	1499	MVA	Placebo
	1500	MVA	Placebo
	1501	MVA	MVA
	1502	Placebo	Placebo
	1503	MVA	Placebo
	1504	MVA	MVA
57	1505	MVA	MVA
	1506	Placebo	Placebo
	1507	MVA	Placebo
	1508	MVA	MVA
	1509	MVA	MVA
	1510	Placebo	Placebo
	1511	Placebo	Placebo
	1512	MVA	Placebo
	1513	MVA	Placebo
58	1514	MVA	Placebo
	1515	Placebo	Placebo
	1516	MVA	Placebo
	1517	Placebo	Placebo
	1518	MVA	MVA
	1519	MVA	MVA
	1520	Placebo	Placebo
	1521	MVA	Placebo
	1522	MVA	MVA
59	1523	MVA	Placebo
	1524	MVA	MVA
	1525	Placebo	Placebo
	1526	MVA	Placebo
	1527	MVA	MVA
	1528	Placebo	Placebo
	1529	MVA	Placebo
	1530	Placebo	Placebo
	1531	MVA	MVA
60	1532	Placebo	Placebo
	1533	MVA	MVA
	1534	Placebo	Placebo
	1535	Placebo	Placebo
	1536	MVA	Placebo
	1537	MVA	Placebo
	1538	MVA	Placebo
	1539	MVA	MVA
	1540	MVA	MVA

Subject randomisation list for BN study POX-MVA-005 (P031363)
Seed= 18655143

Container	Subject Randomization Number	Treatment Sequence	
61	1541	MVA	Placebo
	1542	MVA	MVA
	1543	MVA	Placebo
	1544	Placebo	Placebo
	1545	MVA	MVA
	1546	MVA	Placebo
	1547	MVA	MVA
	1548	Placebo	Placebo
	1549	Placebo	Placebo
62	1550	Placebo	Placebo
	1551	MVA	Placebo
	1552	Placebo	Placebo
	1553	MVA	Placebo
	1554	MVA	MVA
	1555	MVA	MVA
	1556	Placebo	Placebo
	1557	MVA	MVA
	1558	MVA	Placebo
63	1559	MVA	Placebo
	1560	Placebo	Placebo
	1561	MVA	Placebo
	1562	Placebo	Placebo
	1563	Placebo	Placebo
	1564	MVA	MVA
	1565	MVA	Placebo
	1566	MVA	MVA
	1567	MVA	MVA
64	1568	MVA	Placebo
	1569	MVA	Placebo
	1570	MVA	MVA
	1571	Placebo	Placebo
	1572	Placebo	Placebo
	1573	MVA	MVA
	1574	MVA	MVA
	1575	MVA	Placebo
	1576	Placebo	Placebo
65	1577	MVA	MVA
	1578	Placebo	Placebo
	1579	MVA	Placebo
	1580	Placebo	Placebo
	1581	MVA	Placebo
	1582	MVA	MVA
	1583	Placebo	Placebo
	1584	MVA	Placebo
	1585	MVA	MVA
66	1586	Placebo	Placebo
	1587	MVA	MVA
	1588	MVA	MVA
	1589	MVA	Placebo
	1590	MVA	Placebo
	1591	MVA	Placebo
	1592	MVA	MVA
	1593	Placebo	Placebo
	1594	Placebo	Placebo

Subject randomisation list for BN study POX-MVA-005 (P031363)
Seed= 18655143

Container	Subject Randomization Number	Treatment Sequence	
67	1595	Placebo	Placebo
	1596	Placebo	Placebo
	1597	MVA	Placebo
	1598	MVA	MVA
	1599	MVA	Placebo
	1600	MVA	MVA
	1601	MVA	MVA
	1602	MVA	Placebo
	1603	Placebo	Placebo
68	1604	Placebo	Placebo
	1605	MVA	MVA
	1606	MVA	Placebo
	1607	Placebo	Placebo
	1608	MVA	MVA
	1609	MVA	Placebo
	1610	Placebo	Placebo
	1611	MVA	MVA
	1612	MVA	Placebo
69	1613	MVA	MVA
	1614	MVA	Placebo
	1615	Placebo	Placebo
	1616	Placebo	Placebo
	1617	Placebo	Placebo
	1618	MVA	MVA
	1619	MVA	Placebo
	1620	MVA	MVA
	1621	MVA	Placebo
70	1622	MVA	Placebo
	1623	MVA	MVA
	1624	Placebo	Placebo
	1625	Placebo	Placebo
	1626	MVA	MVA
	1627	MVA	Placebo
	1628	Placebo	Placebo
	1629	MVA	MVA
	1630	MVA	Placebo
71	1631	MVA	Placebo
	1632	Placebo	Placebo
	1633	MVA	MVA
	1634	Placebo	Placebo
	1635	Placebo	Placebo
	1636	MVA	Placebo
	1637	MVA	MVA
	1638	MVA	Placebo
	1639	MVA	MVA
72	1640	MVA	Placebo
	1641	Placebo	Placebo
	1642	Placebo	Placebo
	1643	MVA	MVA
	1644	MVA	Placebo
	1645	MVA	Placebo
	1646	MVA	MVA
	1647	Placebo	Placebo
	1648	MVA	MVA

Subject randomisation list for BN study POX-MVA-005 (P031363)
Seed= 18655143

Container	Subject Randomization Number	Treatment Sequence	
73	1649	Placebo	Placebo
	1650	MVA	MVA
	1651	Placebo	Placebo
	1652	Placebo	Placebo
	1653	MVA	MVA
	1654	MVA	Placebo
	1655	MVA	Placebo
	1656	MVA	Placebo
	1657	MVA	MVA
74	1658	MVA	Placebo
	1659	Placebo	Placebo
	1660	MVA	MVA
	1661	MVA	MVA
	1662	MVA	Placebo
	1663	MVA	Placebo
	1664	MVA	MVA
	1665	Placebo	Placebo
	1666	Placebo	Placebo
75	1667	MVA	Placebo
	1668	MVA	Placebo
	1669	Placebo	Placebo
	1670	MVA	MVA
	1671	MVA	Placebo
	1672	MVA	MVA
	1673	MVA	MVA
	1674	Placebo	Placebo
	1675	Placebo	Placebo
76	1676	Placebo	Placebo
	1677	MVA	Placebo
	1678	Placebo	Placebo
	1679	MVA	MVA
	1680	MVA	Placebo
	1681	Placebo	Placebo
	1682	MVA	Placebo
	1683	MVA	MVA
	1684	MVA	MVA
77	1685	MVA	Placebo
	1686	MVA	Placebo
	1687	MVA	MVA
	1688	Placebo	Placebo
	1689	MVA	Placebo
	1690	MVA	MVA
	1691	Placebo	Placebo
	1692	MVA	MVA
	1693	Placebo	Placebo
78	1694	MVA	MVA
	1695	MVA	MVA
	1696	MVA	MVA
	1697	Placebo	Placebo
	1698	MVA	Placebo
	1699	Placebo	Placebo
	1700	MVA	Placebo
	1701	MVA	Placebo
	1702	Placebo	Placebo

Subject randomisation list for BN study POX-MVA-005 (P031363)
Seed= 18655143

Container	Subject Randomization Number	Treatment Sequence	
79	1703	Placebo	Placebo
	1704	MVA	MVA
	1705	MVA	Placebo
	1706	MVA	MVA
	1707	Placebo	Placebo
	1708	Placebo	Placebo
	1709	MVA	Placebo
	1710	MVA	MVA
	1711	MVA	Placebo
80	1712	MVA	Placebo
	1713	Placebo	Placebo
	1714	MVA	Placebo
	1715	Placebo	Placebo
	1716	MVA	Placebo
	1717	Placebo	Placebo
	1718	MVA	MVA
	1719	MVA	MVA
	1720	MVA	MVA
81	1721	MVA	MVA
	1722	MVA	Placebo
	1723	Placebo	Placebo
	1724	MVA	Placebo
	1725	MVA	MVA
	1726	MVA	Placebo
	1727	Placebo	Placebo
	1728	MVA	MVA
	1729	Placebo	Placebo
82	1730	MVA	MVA
	1731	MVA	MVA
	1732	MVA	Placebo
	1733	MVA	Placebo
	1734	MVA	MVA
	1735	Placebo	Placebo
	1736	Placebo	Placebo
	1737	MVA	Placebo
	1738	Placebo	Placebo
83	1739	MVA	Placebo
	1740	Placebo	Placebo
	1741	Placebo	Placebo
	1742	MVA	MVA
	1743	Placebo	Placebo
	1744	MVA	MVA
	1745	MVA	MVA
	1746	MVA	Placebo
	1747	MVA	Placebo
84	1748	MVA	MVA
	1749	MVA	Placebo
	1750	Placebo	Placebo
	1751	MVA	Placebo
	1752	MVA	Placebo
	1753	Placebo	Placebo
	1754	MVA	MVA
	1755	Placebo	Placebo
	1756	MVA	MVA

Subject randomisation list for BN study POX-MVA-005 (P031363)
Seed= 18655143

Container	Subject Randomization Number	Treatment Sequence	
85	1757	MVA	MVA
	1758	Placebo	Placebo
	1759	Placebo	Placebo
	1760	MVA	MVA
	1761	MVA	MVA
	1762	Placebo	Placebo
	1763	MVA	Placebo
	1764	MVA	Placebo
	1765	MVA	Placebo
86	1766	MVA	Placebo
	1767	MVA	MVA
	1768	Placebo	Placebo
	1769	MVA	Placebo
	1770	MVA	MVA
	1771	Placebo	Placebo
	1772	Placebo	Placebo
	1773	MVA	MVA
	1774	MVA	Placebo
87	1775	MVA	MVA
	1776	MVA	Placebo
	1777	MVA	MVA
	1778	Placebo	Placebo
	1779	MVA	MVA
	1780	Placebo	Placebo
	1781	MVA	Placebo
	1782	Placebo	Placebo
	1783	MVA	Placebo
88	1784	MVA	Placebo
	1785	MVA	MVA
	1786	Placebo	Placebo
	1787	Placebo	Placebo
	1788	Placebo	Placebo
	1789	MVA	MVA
	1790	MVA	MVA
	1791	MVA	Placebo
	1792	MVA	Placebo
89	1793	MVA	MVA
	1794	MVA	MVA
	1795	MVA	MVA
	1796	Placebo	Placebo
	1797	Placebo	Placebo
	1798	Placebo	Placebo
	1799	MVA	Placebo
	1800	MVA	Placebo
	1801	MVA	Placebo
90	1802	MVA	MVA
	1803	Placebo	Placebo
	1804	MVA	Placebo
	1805	Placebo	Placebo
	1806	MVA	Placebo
	1807	Placebo	Placebo
	1808	MVA	Placebo
	1809	MVA	MVA
	1810	MVA	MVA

Subject randomisation list for BN study POX-MVA-005 (P031363)
Seed= 18655143

Container	Subject Randomization Number	Treatment Sequence	
91	1811	MVA	Placebo
	1812	Placebo	Placebo
	1813	MVA	MVA
	1814	Placebo	Placebo
	1815	MVA	Placebo
	1816	MVA	MVA
	1817	Placebo	Placebo
	1818	MVA	Placebo
	1819	MVA	MVA
92	1820	MVA	MVA
	1821	Placebo	Placebo
	1822	MVA	Placebo
	1823	MVA	Placebo
	1824	Placebo	Placebo
	1825	MVA	MVA
	1826	MVA	MVA
	1827	MVA	Placebo
	1828	Placebo	Placebo
93	1829	Placebo	Placebo
	1830	MVA	MVA
	1831	Placebo	Placebo
	1832	MVA	MVA
	1833	MVA	MVA
	1834	MVA	Placebo
	1835	Placebo	Placebo
	1836	MVA	Placebo
	1837	MVA	Placebo
94	1838	MVA	MVA
	1839	MVA	Placebo
	1840	Placebo	Placebo
	1841	MVA	Placebo
	1842	MVA	MVA
	1843	MVA	Placebo
	1844	Placebo	Placebo
	1845	Placebo	Placebo
	1846	MVA	MVA
95	1847	MVA	MVA
	1848	MVA	Placebo
	1849	MVA	MVA
	1850	Placebo	Placebo
	1851	MVA	Placebo
	1852	MVA	MVA
	1853	Placebo	Placebo
	1854	MVA	Placebo
	1855	Placebo	Placebo
96	1856	MVA	MVA
	1857	MVA	MVA
	1858	MVA	MVA
	1859	MVA	Placebo
	1860	MVA	Placebo
	1861	Placebo	Placebo
	1862	Placebo	Placebo
	1863	Placebo	Placebo
	1864	MVA	Placebo

Subject randomisation list for BN study POX-MVA-005 (P031363)
Seed= 18655143

Container	Subject Randomization Number	Treatment Sequence	
97	1865	MVA	Placebo
	1866	Placebo	Placebo
	1867	MVA	MVA
	1868	Placebo	Placebo
	1869	MVA	Placebo
	1870	MVA	Placebo
	1871	MVA	MVA
	1872	Placebo	Placebo
	1873	MVA	MVA
98	1874	MVA	Placebo
	1875	MVA	Placebo
	1876	Placebo	Placebo
	1877	Placebo	Placebo
	1878	MVA	Placebo
	1879	Placebo	Placebo
	1880	MVA	MVA
	1881	MVA	MVA
	1882	MVA	MVA
99	1883	MVA	Placebo
	1884	MVA	Placebo
	1885	MVA	Placebo
	1886	Placebo	Placebo
	1887	Placebo	Placebo
	1888	Placebo	Placebo
	1889	MVA	MVA
	1890	MVA	MVA
	1891	MVA	MVA
100	1892	Placebo	Placebo
	1893	MVA	Placebo
	1894	Placebo	Placebo
	1895	MVA	Placebo
	1896	MVA	MVA
	1897	Placebo	Placebo
	1898	MVA	Placebo
	1899	MVA	MVA
	1900	MVA	MVA
101	1901	MVA	MVA
	1902	MVA	Placebo
	1903	Placebo	Placebo
	1904	MVA	MVA
	1905	Placebo	Placebo
	1906	MVA	Placebo
	1907	Placebo	Placebo
	1908	MVA	MVA
	1909	MVA	Placebo
102	1910	Placebo	Placebo
	1911	MVA	MVA
	1912	Placebo	Placebo
	1913	MVA	Placebo
	1914	Placebo	Placebo
	1915	MVA	Placebo
	1916	MVA	Placebo
	1917	MVA	MVA
	1918	MVA	MVA

Subject randomisation list for BN study POX-MVA-005 (P031363)
Seed= 18655143

Container	Subject Randomization Number	Treatment Sequence	
103	1919	MVA	Placebo
	1920	MVA	Placebo
	1921	MVA	Placebo
	1922	Placebo	Placebo
	1923	Placebo	Placebo
	1924	MVA	MVA
	1925	MVA	MVA
	1926	Placebo	Placebo
	1927	MVA	MVA
104	1928	MVA	Placebo
	1929	Placebo	Placebo
	1930	MVA	MVA
	1931	MVA	Placebo
	1932	Placebo	Placebo
	1933	MVA	MVA
	1934	MVA	MVA
	1935	Placebo	Placebo
	1936	MVA	Placebo
105	1937	MVA	Placebo
	1938	Placebo	Placebo
	1939	Placebo	Placebo
	1940	MVA	MVA
	1941	MVA	MVA
	1942	MVA	MVA
	1943	MVA	Placebo
	1944	MVA	Placebo
	1945	Placebo	Placebo
106	1946	MVA	MVA
	1947	Placebo	Placebo
	1948	MVA	MVA
	1949	Placebo	Placebo
	1950	MVA	MVA
	1951	Placebo	Placebo
	1952	MVA	Placebo
	1953	MVA	Placebo
	1954	MVA	Placebo
107	1955	Placebo	Placebo
	1956	MVA	MVA
	1957	MVA	MVA
	1958	MVA	Placebo
	1959	MVA	Placebo
	1960	MVA	MVA
	1961	Placebo	Placebo
	1962	MVA	Placebo
	1963	Placebo	Placebo
108	1964	MVA	Placebo
	1965	Placebo	Placebo
	1966	MVA	MVA
	1967	MVA	MVA
	1968	Placebo	Placebo
	1969	MVA	MVA
	1970	MVA	Placebo
	1971	Placebo	Placebo
	1972	MVA	Placebo

Subject randomisation list for BN study POX-MVA-005 (P031363)
Seed= 18655143

Container	Subject Randomization Number	Treatment Sequence	
109	1973	MVA	Placebo
	1974	MVA	MVA
	1975	MVA	MVA
	1976	MVA	Placebo
	1977	Placebo	Placebo
	1978	Placebo	Placebo
	1979	Placebo	Placebo
	1980	MVA	MVA
	1981	MVA	Placebo
110	1982	Placebo	Placebo
	1983	MVA	Placebo
	1984	MVA	Placebo
	1985	MVA	MVA
	1986	Placebo	Placebo
	1987	MVA	MVA
	1988	Placebo	Placebo
	1989	MVA	MVA
	1990	MVA	Placebo
111	1991	MVA	Placebo
	1992	MVA	Placebo
	1993	MVA	MVA
	1994	MVA	MVA
	1995	Placebo	Placebo
	1996	MVA	Placebo
	1997	MVA	MVA
	1998	Placebo	Placebo
	1999	Placebo	Placebo
112	2000	MVA	Placebo
	2001	MVA	Placebo
	2002	MVA	MVA
	2003	Placebo	Placebo
	2004	MVA	MVA
	2005	Placebo	Placebo
	2006	Placebo	Placebo
	2007	MVA	Placebo
	2008	MVA	MVA
113	2009	MVA	MVA
	2010	MVA	Placebo
	2011	MVA	MVA
	2012	Placebo	Placebo
	2013	Placebo	Placebo
	2014	Placebo	Placebo
	2015	MVA	Placebo
	2016	MVA	MVA
	2017	MVA	Placebo
114	2018	MVA	Placebo
	2019	MVA	MVA
	2020	Placebo	Placebo
	2021	Placebo	Placebo
	2022	MVA	Placebo
	2023	MVA	MVA
	2024	Placebo	Placebo
	2025	MVA	Placebo
	2026	MVA	MVA

Subject randomisation list for BN study POX-MVA-005 (P031363)
Seed= 18655143

Container	Subject Randomization Number	Treatment Sequence	
115	2027	MVA	Placebo
	2028	MVA	Placebo
	2029	Placebo	Placebo
	2030	Placebo	Placebo
	2031	MVA	MVA
	2032	MVA	MVA
	2033	MVA	Placebo
	2034	Placebo	Placebo
	2035	MVA	MVA
116	2036	MVA	MVA
	2037	MVA	Placebo
	2038	MVA	MVA
	2039	MVA	Placebo
	2040	Placebo	Placebo
	2041	MVA	MVA
	2042	MVA	Placebo
	2043	Placebo	Placebo
	2044	Placebo	Placebo
117	2045	Placebo	Placebo
	2046	MVA	Placebo
	2047	Placebo	Placebo
	2048	MVA	MVA
	2049	MVA	MVA
	2050	MVA	Placebo
	2051	Placebo	Placebo
	2052	MVA	Placebo
	2053	MVA	MVA
118	2054	MVA	Placebo
	2055	Placebo	Placebo
	2056	MVA	MVA
	2057	MVA	Placebo
	2058	Placebo	Placebo
	2059	MVA	MVA
	2060	MVA	Placebo
	2061	Placebo	Placebo
	2062	MVA	MVA
119	2063	MVA	Placebo
	2064	MVA	Placebo
	2065	Placebo	Placebo
	2066	MVA	Placebo
	2067	MVA	MVA
	2068	Placebo	Placebo
	2069	MVA	MVA
	2070	MVA	MVA
	2071	Placebo	Placebo
120	2072	MVA	Placebo
	2073	MVA	MVA
	2074	Placebo	Placebo
	2075	MVA	Placebo
	2076	MVA	MVA
	2077	MVA	MVA
	2078	MVA	Placebo
	2079	Placebo	Placebo
	2080	Placebo	Placebo

Subject randomisation list for BN study POX-MVA-005 (P031363)
Seed= 18655143

Container	Subject Randomization Number	Treatment Sequence	
121	2081	MVA	Placebo
	2082	MVA	MVA
	2083	MVA	Placebo
	2084	Placebo	Placebo
	2085	Placebo	Placebo
	2086	Placebo	Placebo
	2087	MVA	MVA
	2088	MVA	MVA
	2089	MVA	Placebo
122	2090	MVA	MVA
	2091	Placebo	Placebo
	2092	Placebo	Placebo
	2093	Placebo	Placebo
	2094	MVA	Placebo
	2095	MVA	MVA
	2096	MVA	Placebo
	2097	MVA	MVA
	2098	MVA	Placebo
123	2099	Placebo	Placebo
	2100	MVA	Placebo
	2101	MVA	MVA
	2102	MVA	MVA
	2103	MVA	Placebo
	2104	Placebo	Placebo
	2105	MVA	Placebo
	2106	Placebo	Placebo
	2107	MVA	MVA
124	2108	MVA	Placebo
	2109	Placebo	Placebo
	2110	Placebo	Placebo
	2111	MVA	MVA
	2112	MVA	MVA
	2113	Placebo	Placebo
	2114	MVA	MVA
	2115	MVA	Placebo
	2116	MVA	Placebo
125	2117	Placebo	Placebo
	2118	MVA	Placebo
	2119	MVA	Placebo
	2120	MVA	MVA
	2121	MVA	MVA
	2122	Placebo	Placebo
	2123	MVA	MVA
	2124	MVA	Placebo
	2125	Placebo	Placebo
126	2126	MVA	MVA
	2127	Placebo	Placebo
	2128	MVA	MVA
	2129	Placebo	Placebo
	2130	Placebo	Placebo
	2131	MVA	Placebo
	2132	MVA	Placebo
	2133	MVA	MVA
	2134	MVA	Placebo

Subject randomisation list for BN study POX-MVA-005 (P031363)
Seed= 18655143

Container	Subject Randomization Number	Treatment Sequence	
127	2135	MVA	Placebo
	2136	MVA	MVA
	2137	MVA	Placebo
	2138	MVA	MVA
	2139	MVA	Placebo
	2140	MVA	MVA
	2141	Placebo	Placebo
	2142	Placebo	Placebo
	2143	Placebo	Placebo
128	2144	Placebo	Placebo
	2145	Placebo	Placebo
	2146	MVA	Placebo
	2147	MVA	Placebo
	2148	MVA	MVA
	2149	MVA	MVA
	2150	MVA	Placebo
	2151	Placebo	Placebo
	2152	MVA	MVA
129	2153	Placebo	Placebo
	2154	MVA	MVA
	2155	MVA	Placebo
	2156	MVA	MVA
	2157	MVA	Placebo
	2158	Placebo	Placebo
	2159	Placebo	Placebo
	2160	MVA	Placebo
	2161	MVA	MVA
130	2162	MVA	Placebo
	2163	Placebo	Placebo
	2164	MVA	MVA
	2165	MVA	MVA
	2166	MVA	Placebo
	2167	MVA	Placebo
	2168	Placebo	Placebo
	2169	MVA	MVA
	2170	Placebo	Placebo
131	2171	MVA	Placebo
	2172	MVA	MVA
	2173	MVA	MVA
	2174	Placebo	Placebo
	2175	Placebo	Placebo
	2176	MVA	Placebo
	2177	Placebo	Placebo
	2178	MVA	MVA
	2179	MVA	Placebo
132	2180	MVA	Placebo
	2181	Placebo	Placebo
	2182	MVA	MVA
	2183	Placebo	Placebo
	2184	MVA	MVA
	2185	Placebo	Placebo
	2186	MVA	Placebo
	2187	MVA	MVA
	2188	MVA	Placebo

Subject randomisation list for BN study POX-MVA-005 (P031363)
Seed= 18655143

Container	Subject Randomization Number	Treatment Sequence	
133	2189	MVA	MVA
	2190	MVA	Placebo
	2191	MVA	MVA
	2192	MVA	Placebo
	2193	Placebo	Placebo
	2194	Placebo	Placebo
	2195	MVA	MVA
	2196	Placebo	Placebo
	2197	MVA	Placebo

Vial randomisation list for BN study POX-MVA-005 (P031363)
Seed= 83461674

Container	Vial Randomization Number	Treatment
1	1	MVA
	2	Placebo
	3	MVA
	4	MVA
	5	MVA
	6	Placebo
	7	MVA
	8	Placebo
	9	MVA
	10	Placebo
	11	MVA
	12	Placebo
	13	Placebo
	14	MVA
	15	Placebo
	16	Placebo
	17	MVA
	18	Placebo
2	19	Placebo
	20	Placebo
	21	MVA
	22	MVA
	23	Placebo
	24	MVA
	25	Placebo
	26	MVA
	27	Placebo
	28	Placebo
	29	Placebo
	30	Placebo
	31	Placebo
	32	MVA
	33	MVA
	34	MVA
	35	MVA
	36	MVA
3	37	MVA
	38	MVA
	39	Placebo
	40	Placebo
	41	Placebo
	42	Placebo
	43	MVA
	44	Placebo
	45	MVA
	46	MVA
	47	Placebo
	48	MVA
	49	MVA
	50	Placebo
	51	MVA
	52	MVA
	53	Placebo
	54	Placebo

Vial randomisation list for BN study POX-MVA-005 (P031363)
Seed= 83461674

Container	Vial Randomization Number	Treatment
4	55	MVA
	56	MVA
	57	Placebo
	58	MVA
	59	Placebo
	60	Placebo
	61	MVA
	62	MVA
	63	Placebo
	64	Placebo
	65	Placebo
	66	MVA
	67	Placebo
	68	MVA
	69	MVA
	70	Placebo
	71	Placebo
	72	MVA
5	73	Placebo
	74	Placebo
	75	Placebo
	76	Placebo
	77	Placebo
	78	MVA
	79	MVA
	80	Placebo
	81	MVA
	82	Placebo
	83	MVA
	84	MVA
	85	MVA
	86	Placebo
	87	MVA
	88	MVA
	89	Placebo
	90	MVA
6	91	MVA
	92	MVA
	93	MVA
	94	MVA
	95	Placebo
	96	Placebo
	97	Placebo
	98	MVA
	99	MVA
	100	Placebo
	101	Placebo
	102	MVA
	103	Placebo
	104	MVA
	105	Placebo
	106	Placebo
	107	MVA
	108	Placebo

Vial randomisation list for BN study POX-MVA-005 (P031363)
Seed= 83461674

Container	Vial Randomization Number	Treatment
7	109	MVA
	110	MVA
	111	Placebo
	112	MVA
	113	Placebo
	114	MVA
	115	MVA
	116	Placebo
	117	MVA
	118	Placebo
	119	Placebo
	120	MVA
	121	Placebo
	122	MVA
	123	MVA
	124	Placebo
	125	Placebo
	126	Placebo
8	127	Placebo
	128	MVA
	129	Placebo
	130	MVA
	131	Placebo
	132	Placebo
	133	Placebo
	134	Placebo
	135	MVA
	136	MVA
	137	Placebo
	138	MVA
	139	Placebo
	140	MVA
	141	MVA
	142	Placebo
	143	MVA
	144	MVA
9	145	Placebo
	146	MVA
	147	Placebo
	148	MVA
	149	MVA
	150	MVA
	151	MVA
	152	Placebo
	153	Placebo
	154	Placebo
	155	Placebo
	156	MVA
	157	MVA
	158	Placebo
	159	Placebo
	160	MVA
	161	MVA
	162	Placebo

Vial randomisation list for BN study POX-MVA-005 (P031363)
Seed= 83461674

Container	Vial Randomization Number	Treatment
10	163	MVA
	164	Placebo
	165	Placebo
	166	Placebo
	167	Placebo
	168	MVA
	169	MVA
	170	MVA
	171	MVA
	172	Placebo
	173	Placebo
	174	Placebo
	175	MVA
	176	Placebo
	177	MVA
	178	Placebo
	179	MVA
	180	MVA
11	181	MVA
	182	MVA
	183	MVA
	184	MVA
	185	Placebo
	186	Placebo
	187	MVA
	188	Placebo
	189	MVA
	190	MVA
	191	MVA
	192	MVA
	193	Placebo
	194	Placebo
	195	Placebo
	196	Placebo
	197	Placebo
	198	Placebo
12	199	Placebo
	200	Placebo
	201	Placebo
	202	MVA
	203	MVA
	204	Placebo
	205	MVA
	206	MVA
	207	MVA
	208	MVA
	209	Placebo
	210	Placebo
	211	Placebo
	212	MVA
	213	Placebo
	214	Placebo
	215	MVA
	216	MVA

Vial randomisation list for BN study POX-MVA-005 (P031363)
Seed= 83461674

Container	Vial Randomization Number	Treatment
13	217	Placebo
	218	Placebo
	219	Placebo
	220	MVA
	221	Placebo
	222	MVA
	223	Placebo
	224	MVA
	225	MVA
	226	Placebo
	227	Placebo
	228	MVA
	229	MVA
	230	Placebo
	231	MVA
	232	MVA
	233	Placebo
	234	MVA
14	235	MVA
	236	Placebo
	237	Placebo
	238	MVA
	239	MVA
	240	Placebo
	241	MVA
	242	MVA
	243	Placebo
	244	MVA
	245	Placebo
	246	Placebo
	247	MVA
	248	Placebo
	249	Placebo
	250	MVA
	251	Placebo
	252	MVA
15	253	Placebo
	254	Placebo
	255	Placebo
	256	Placebo
	257	MVA
	258	MVA
	259	MVA
	260	MVA
	261	Placebo
	262	MVA
	263	MVA
	264	MVA
	265	Placebo
	266	Placebo
	267	MVA
	268	MVA
	269	Placebo
	270	Placebo

Vial randomisation list for BN study POX-MVA-005 (P031363)
Seed= 83461674

Container	Vial Randomization Number	Treatment
16	271	MVA
	272	Placebo
	273	MVA
	274	MVA
	275	MVA
	276	Placebo
	277	Placebo
	278	Placebo
	279	MVA
	280	Placebo
	281	MVA
	282	Placebo
	283	Placebo
	284	Placebo
	285	Placebo
	286	MVA
	287	MVA
	288	MVA
17	289	Placebo
	290	MVA
	291	MVA
	292	Placebo
	293	MVA
	294	MVA
	295	Placebo
	296	MVA
	297	MVA
	298	MVA
	299	MVA
	300	Placebo
	301	Placebo
	302	Placebo
	303	Placebo
	304	Placebo
	305	MVA
	306	Placebo
18	307	MVA
	308	Placebo
	309	MVA
	310	Placebo
	311	MVA
	312	MVA
	313	Placebo
	314	Placebo
	315	Placebo
	316	MVA
	317	MVA
	318	Placebo
	319	MVA
	320	Placebo
	321	MVA
	322	MVA
	323	Placebo
	324	Placebo

Vial randomisation list for BN study POX-MVA-005 (P031363)
Seed= 83461674

Container	Vial Randomization Number	Treatment
19	325	Placebo
	326	Placebo
	327	Placebo
	328	Placebo
	329	Placebo
	330	Placebo
	331	MVA
	332	MVA
	333	MVA
	334	MVA
	335	MVA
	336	Placebo
	337	MVA
	338	Placebo
	339	Placebo
	340	MVA
	341	MVA
	342	MVA
20	343	MVA
	344	Placebo
	345	Placebo
	346	MVA
	347	MVA
	348	Placebo
	349	Placebo
	350	MVA
	351	MVA
	352	MVA
	353	Placebo
	354	MVA
	355	MVA
	356	Placebo
	357	Placebo
	358	Placebo
	359	Placebo
	360	MVA
21	361	MVA
	362	MVA
	363	Placebo
	364	MVA
	365	Placebo
	366	MVA
	367	MVA
	368	Placebo
	369	Placebo
	370	MVA
	371	Placebo
	372	MVA
	373	Placebo
	374	MVA
	375	Placebo
	376	Placebo
	377	MVA
	378	Placebo

Vial randomisation list for BN study POX-MVA-005 (P031363)
Seed= 83461674

Container	Vial Randomization Number	Treatment
22	379	MVA
	380	Placebo
	381	Placebo
	382	MVA
	383	Placebo
	384	MVA
	385	MVA
	386	MVA
	387	MVA
	388	Placebo
	389	Placebo
	390	Placebo
	391	MVA
	392	MVA
	393	Placebo
	394	MVA
	395	Placebo
	396	Placebo
23	397	Placebo
	398	MVA
	399	MVA
	400	Placebo
	401	Placebo
	402	MVA
	403	MVA
	404	Placebo
	405	MVA
	406	MVA
	407	Placebo
	408	Placebo
	409	MVA
	410	Placebo
	411	MVA
	412	MVA
	413	Placebo
	414	Placebo
24	415	MVA
	416	MVA
	417	MVA
	418	Placebo
	419	MVA
	420	Placebo
	421	Placebo
	422	MVA
	423	MVA
	424	MVA
	425	Placebo
	426	Placebo
	427	MVA
	428	Placebo
	429	Placebo
	430	MVA
	431	Placebo
	432	Placebo

Vial randomisation list for BN study POX-MVA-005 (P031363)
Seed= 83461674

Container	Vial Randomization Number	Treatment
25	433	MVA
	434	Placebo
	435	MVA
	436	MVA
	437	Placebo
	438	Placebo
	439	MVA
	440	Placebo
	441	MVA
	442	Placebo
	443	MVA
	444	Placebo
	445	MVA
	446	Placebo
	447	MVA
	448	Placebo
	449	Placebo
	450	MVA
26	451	MVA
	452	Placebo
	453	MVA
	454	MVA
	455	Placebo
	456	MVA
	457	MVA
	458	Placebo
	459	MVA
	460	MVA
	461	Placebo
	462	Placebo
	463	MVA
	464	Placebo
	465	Placebo
	466	Placebo
	467	MVA
	468	Placebo
27	469	Placebo
	470	Placebo
	471	MVA
	472	MVA
	473	Placebo
	474	MVA
	475	MVA
	476	MVA
	477	MVA
	478	Placebo
	479	Placebo
	480	MVA
	481	Placebo
	482	Placebo
	483	Placebo
	484	MVA
	485	Placebo
	486	MVA

Vial randomisation list for BN study POX-MVA-005 (P031363)
Seed= 83461674

Container	Vial Randomization Number	Treatment
28	487	MVA
	488	MVA
	489	Placebo
	490	MVA
	491	MVA
	492	Placebo
	493	Placebo
	494	Placebo
	495	Placebo
	496	Placebo
	497	Placebo
	498	MVA
	499	MVA
	500	Placebo
	501	Placebo
	502	MVA
	503	MVA
	504	MVA
29	505	Placebo
	506	MVA
	507	Placebo
	508	MVA
	509	Placebo
	510	MVA
	511	MVA
	512	Placebo
	513	MVA
	514	Placebo
	515	MVA
	516	Placebo
	517	Placebo
	518	Placebo
	519	MVA
	520	Placebo
	521	MVA
	522	MVA
30	523	MVA
	524	MVA
	525	Placebo
	526	Placebo
	527	Placebo
	528	Placebo
	529	Placebo
	530	Placebo
	531	MVA
	532	MVA
	533	MVA
	534	MVA
	535	MVA
	536	Placebo
	537	MVA
	538	MVA
	539	Placebo
	540	Placebo

Vial randomisation list for BN study POX-MVA-005 (P031363)
Seed= 83461674

Container	Vial Randomization Number	Treatment
31	541	Placebo
	542	Placebo
	543	Placebo
	544	Placebo
	545	Placebo
	546	MVA
	547	MVA
	548	MVA
	549	MVA
	550	Placebo
	551	MVA
	552	Placebo
	553	Placebo
	554	MVA
	555	Placebo
	556	MVA
	557	MVA
	558	MVA
32	559	MVA
	560	Placebo
	561	Placebo
	562	MVA
	563	Placebo
	564	MVA
	565	Placebo
	566	MVA
	567	Placebo
	568	MVA
	569	Placebo
	570	MVA
	571	Placebo
	572	Placebo
	573	MVA
	574	Placebo
	575	MVA
	576	MVA
33	577	MVA
	578	Placebo
	579	Placebo
	580	Placebo
	581	MVA
	582	Placebo
	583	MVA
	584	Placebo
	585	Placebo
	586	Placebo
	587	MVA
	588	MVA
	589	MVA
	590	MVA
	591	Placebo
	592	Placebo
	593	MVA
	594	MVA

Vial randomisation list for BN study POX-MVA-005 (P031363)
Seed= 83461674

Container	Vial Randomization Number	Treatment
34	595	Placebo
	596	Placebo
	597	Placebo
	598	MVA
	599	MVA
	600	Placebo
	601	Placebo
	602	MVA
	603	Placebo
	604	MVA
	605	Placebo
	606	Placebo
	607	MVA
	608	Placebo
	609	MVA
	610	MVA
	611	MVA
	612	MVA
35	613	Placebo
	614	MVA
	615	Placebo
	616	MVA
	617	MVA
	618	Placebo
	619	MVA
	620	Placebo
	621	Placebo
	622	MVA
	623	MVA
	624	MVA
	625	Placebo
	626	MVA
	627	Placebo
	628	Placebo
	629	MVA
	630	Placebo
36	631	MVA
	632	Placebo
	633	Placebo
	634	Placebo
	635	MVA
	636	MVA
	637	Placebo
	638	MVA
	639	MVA
	640	Placebo
	641	MVA
	642	MVA
	643	Placebo
	644	MVA
	645	MVA
	646	Placebo
	647	Placebo
	648	Placebo

Vial randomisation list for BN study POX-MVA-005 (P031363)
Seed= 83461674

Container	Vial Randomization Number	Treatment
37	649	MVA
	650	Placebo
	651	MVA
	652	Placebo
	653	Placebo
	654	Placebo
	655	MVA
	656	MVA
	657	Placebo
	658	MVA
	659	MVA
	660	Placebo
	661	MVA
	662	MVA
	663	Placebo
	664	Placebo
	665	MVA
	666	Placebo
38	667	Placebo
	668	MVA
	669	Placebo
	670	MVA
	671	Placebo
	672	MVA
	673	MVA
	674	Placebo
	675	Placebo
	676	MVA
	677	Placebo
	678	Placebo
	679	Placebo
	680	MVA
	681	Placebo
	682	MVA
	683	MVA
	684	MVA
39	685	MVA
	686	MVA
	687	Placebo
	688	MVA
	689	Placebo
	690	MVA
	691	Placebo
	692	Placebo
	693	MVA
	694	Placebo
	695	MVA
	696	Placebo
	697	MVA
	698	Placebo
	699	MVA
	700	Placebo
	701	Placebo
	702	MVA

Vial randomisation list for BN study POX-MVA-005 (P031363)
Seed= 83461674

Container	Vial Randomization Number	Treatment
40	703	Placebo
	704	Placebo
	705	Placebo
	706	MVA
	707	Placebo
	708	MVA
	709	Placebo
	710	Placebo
	711	Placebo
	712	MVA
	713	MVA
	714	Placebo
	715	Placebo
	716	MVA
	717	MVA
	718	MVA
	719	MVA
	720	MVA
41	721	Placebo
	722	Placebo
	723	MVA
	724	MVA
	725	MVA
	726	Placebo
	727	MVA
	728	MVA
	729	MVA
	730	Placebo
	731	MVA
	732	Placebo
	733	Placebo
	734	Placebo
	735	Placebo
	736	Placebo
	737	MVA
	738	MVA
42	739	Placebo
	740	Placebo
	741	MVA
	742	MVA
	743	Placebo
	744	MVA
	745	MVA
	746	Placebo
	747	Placebo
	748	Placebo
	749	MVA
	750	Placebo
	751	MVA
	752	Placebo
	753	Placebo
	754	MVA
	755	MVA
	756	MVA

Vial randomisation list for BN study POX-MVA-005 (P031363)
Seed= 83461674

Container	Vial Randomization Number	Treatment
43	757	MVA
	758	MVA
	759	Placebo
	760	Placebo
	761	MVA
	762	Placebo
	763	MVA
	764	Placebo
	765	MVA
	766	MVA
	767	Placebo
	768	Placebo
	769	Placebo
	770	Placebo
	771	MVA
	772	MVA
	773	MVA
	774	Placebo
44	775	MVA
	776	Placebo
	777	Placebo
	778	MVA
	779	Placebo
	780	Placebo
	781	Placebo
	782	Placebo
	783	MVA
	784	MVA
	785	Placebo
	786	MVA
	787	MVA
	788	MVA
	789	MVA
	790	Placebo
	791	Placebo
	792	MVA
45	793	Placebo
	794	MVA
	795	Placebo
	796	MVA
	797	MVA
	798	MVA
	799	Placebo
	800	Placebo
	801	MVA
	802	Placebo
	803	MVA
	804	MVA
	805	MVA
	806	Placebo
	807	MVA
	808	Placebo
	809	Placebo
	810	Placebo

Vial randomisation list for BN study POX-MVA-005 (P031363)
Seed= 83461674

Container	Vial Randomization Number	Treatment
46	811	Placebo
	812	Placebo
	813	MVA
	814	Placebo
	815	MVA
	816	Placebo
	817	MVA
	818	MVA
	819	Placebo
	820	MVA
	821	Placebo
	822	Placebo
	823	MVA
	824	MVA
	825	MVA
	826	MVA
	827	Placebo
	828	Placebo
47	829	Placebo
	830	MVA
	831	MVA
	832	Placebo
	833	MVA
	834	MVA
	835	Placebo
	836	MVA
	837	MVA
	838	Placebo
	839	MVA
	840	Placebo
	841	Placebo
	842	MVA
	843	Placebo
	844	Placebo
	845	MVA
	846	Placebo
48	847	Placebo
	848	MVA
	849	Placebo
	850	MVA
	851	Placebo
	852	MVA
	853	MVA
	854	MVA
	855	Placebo
	856	MVA
	857	Placebo
	858	Placebo
	859	MVA
	860	Placebo
	861	MVA
	862	Placebo
	863	MVA
	864	Placebo

Vial randomisation list for BN study POX-MVA-005 (P031363)
Seed= 83461674

Container	Vial Randomization Number	Treatment
49	865	Placebo
	866	MVA
	867	Placebo
	868	Placebo
	869	MVA
	870	MVA
	871	Placebo
	872	Placebo
	873	Placebo
	874	Placebo
	875	MVA
	876	Placebo
	877	MVA
	878	MVA
	879	MVA
	880	Placebo
	881	MVA
	882	MVA
50	883	Placebo
	884	Placebo
	885	MVA
	886	Placebo
	887	Placebo
	888	MVA
	889	MVA
	890	MVA
	891	Placebo
	892	Placebo
	893	MVA
	894	MVA
	895	MVA
	896	Placebo
	897	Placebo
	898	Placebo
	899	MVA
	900	MVA
51	901	Placebo
	902	MVA
	903	Placebo
	904	Placebo
	905	MVA
	906	MVA
	907	Placebo
	908	Placebo
	909	MVA
	910	MVA
	911	MVA
	912	MVA
	913	Placebo
	914	Placebo
	915	MVA
	916	Placebo
	917	Placebo
	918	MVA

Vial randomisation list for BN study POX-MVA-005 (P031363)
Seed= 83461674

Container	Vial Randomization Number	Treatment
52	919	MVA
	920	MVA
	921	Placebo
	922	MVA
	923	Placebo
	924	Placebo
	925	Placebo
	926	MVA
	927	MVA
	928	MVA
	929	Placebo
	930	Placebo
	931	MVA
	932	MVA
	933	Placebo
	934	Placebo
	935	Placebo
	936	MVA
53	937	Placebo
	938	MVA
	939	MVA
	940	MVA
	941	MVA
	942	MVA
	943	Placebo
	944	MVA
	945	MVA
	946	MVA
	947	Placebo
	948	Placebo
	949	Placebo
	950	Placebo
	951	Placebo
	952	Placebo
	953	MVA
	954	Placebo
54	955	Placebo
	956	Placebo
	957	Placebo
	958	Placebo
	959	MVA
	960	MVA
	961	Placebo
	962	Placebo
	963	Placebo
	964	MVA
	965	Placebo
	966	MVA
	967	Placebo
	968	MVA
	969	MVA
	970	MVA
	971	MVA
	972	MVA

Vial randomisation list for BN study POX-MVA-005 (P031363)
Seed= 83461674

Container	Vial Randomization Number	Treatment
55	973	Placebo
	974	MVA
	975	MVA
	976	Placebo
	977	Placebo
	978	MVA
	979	Placebo
	980	MVA
	981	MVA
	982	Placebo
	983	MVA
	984	Placebo
	985	Placebo
	986	MVA
	987	Placebo
	988	MVA
	989	MVA
	990	Placebo
56	991	MVA
	992	MVA
	993	MVA
	994	MVA
	995	MVA
	996	Placebo
	997	MVA
	998	Placebo
	999	MVA
	1000	MVA
	1001	MVA
	1002	Placebo
	1003	Placebo
	1004	Placebo
	1005	Placebo
	1006	Placebo
	1007	Placebo
	1008	Placebo
57	1009	MVA
	1010	MVA
	1011	Placebo
	1012	Placebo
	1013	Placebo
	1014	Placebo
	1015	Placebo
	1016	Placebo
	1017	MVA
	1018	MVA
	1019	Placebo
	1020	MVA
	1021	MVA
	1022	Placebo
	1023	Placebo
	1024	MVA
	1025	MVA
	1026	MVA

Vial randomisation list for BN study POX-MVA-005 (P031363)
Seed= 83461674

Container	Vial Randomization Number	Treatment
58	1027	MVA
	1028	Placebo
	1029	MVA
	1030	MVA
	1031	MVA
	1032	Placebo
	1033	Placebo
	1034	Placebo
	1035	Placebo
	1036	MVA
	1037	MVA
	1038	Placebo
	1039	Placebo
	1040	MVA
	1041	Placebo
	1042	MVA
	1043	Placebo
	1044	MVA
59	1045	MVA
	1046	Placebo
	1047	Placebo
	1048	MVA
	1049	Placebo
	1050	Placebo
	1051	Placebo
	1052	Placebo
	1053	MVA
	1054	Placebo
	1055	MVA
	1056	MVA
	1057	MVA
	1058	MVA
	1059	Placebo
	1060	MVA
	1061	MVA
	1062	Placebo
60	1063	MVA
	1064	MVA
	1065	MVA
	1066	Placebo
	1067	Placebo
	1068	Placebo
	1069	Placebo
	1070	Placebo
	1071	Placebo
	1072	MVA
	1073	MVA
	1074	Placebo
	1075	MVA
	1076	MVA
	1077	MVA
	1078	MVA
	1079	Placebo
	1080	Placebo

Vial randomisation list for BN study POX-MVA-005 (P031363)
Seed= 83461674

Container	Vial Randomization Number	Treatment
61	1081	Placebo
	1082	Placebo
	1083	Placebo
	1084	Placebo
	1085	MVA
	1086	MVA
	1087	MVA
	1088	MVA
	1089	Placebo
	1090	Placebo
	1091	MVA
	1092	Placebo
	1093	Placebo
	1094	Placebo
	1095	MVA
	1096	MVA
	1097	MVA
	1098	MVA
62	1099	Placebo
	1100	MVA
	1101	Placebo
	1102	Placebo
	1103	Placebo
	1104	MVA
	1105	MVA
	1106	MVA
	1107	Placebo
	1108	MVA
	1109	Placebo
	1110	MVA
	1111	Placebo
	1112	MVA
	1113	MVA
	1114	MVA
	1115	Placebo
	1116	Placebo
63	1117	Placebo
	1118	Placebo
	1119	Placebo
	1120	MVA
	1121	MVA
	1122	Placebo
	1123	MVA
	1124	Placebo
	1125	Placebo
	1126	Placebo
	1127	Placebo
	1128	MVA
	1129	Placebo
	1130	MVA
	1131	MVA
	1132	MVA
	1133	MVA
	1134	MVA

Vial randomisation list for BN study POX-MVA-005 (P031363)
Seed= 83461674

Container	Vial Randomization Number	Treatment
64	1135	MVA
	1136	Placebo
	1137	MVA
	1138	MVA
	1139	Placebo
	1140	Placebo
	1141	Placebo
	1142	Placebo
	1143	Placebo
	1144	MVA
	1145	Placebo
	1146	Placebo
	1147	MVA
	1148	MVA
	1149	MVA
	1150	Placebo
	1151	MVA
	1152	MVA
65	1153	MVA
	1154	Placebo
	1155	Placebo
	1156	Placebo
	1157	Placebo
	1158	MVA
	1159	Placebo
	1160	Placebo
	1161	Placebo
	1162	MVA
	1163	MVA
	1164	MVA
	1165	MVA
	1166	MVA
	1167	Placebo
	1168	MVA
	1169	Placebo
	1170	MVA
66	1171	MVA
	1172	Placebo
	1173	MVA
	1174	MVA
	1175	Placebo
	1176	Placebo
	1177	Placebo
	1178	Placebo
	1179	MVA
	1180	Placebo
	1181	MVA
	1182	MVA
	1183	Placebo
	1184	Placebo
	1185	MVA
	1186	MVA
	1187	MVA
	1188	Placebo

Vial randomisation list for BN study POX-MVA-005 (P031363)
Seed= 83461674

Container	Vial Randomization Number	Treatment
67	1189	MVA
	1190	Placebo
	1191	Placebo
	1192	Placebo
	1193	Placebo
	1194	MVA
	1195	MVA
	1196	Placebo
	1197	MVA
	1198	MVA
	1199	MVA
	1200	Placebo
	1201	MVA
	1202	MVA
	1203	Placebo
	1204	Placebo
	1205	Placebo
	1206	MVA
68	1207	MVA
	1208	MVA
	1209	MVA
	1210	MVA
	1211	MVA
	1212	MVA
	1213	Placebo
	1214	MVA
	1215	MVA
	1216	Placebo
	1217	Placebo
	1218	Placebo
	1219	Placebo
	1220	Placebo
	1221	Placebo
	1222	Placebo
	1223	MVA
	1224	Placebo
69	1225	MVA
	1226	MVA
	1227	Placebo
	1228	Placebo
	1229	MVA
	1230	MVA
	1231	Placebo
	1232	Placebo
	1233	Placebo
	1234	Placebo
	1235	MVA
	1236	MVA
	1237	Placebo
	1238	MVA
	1239	Placebo
	1240	MVA
	1241	MVA
	1242	Placebo

Vial randomisation list for BN study POX-MVA-005 (P031363)
Seed= 83461674

Container	Vial Randomization Number	Treatment
70	1243	Placebo
	1244	MVA
	1245	Placebo
	1246	MVA
	1247	MVA
	1248	MVA
	1249	MVA
	1250	Placebo
	1251	Placebo
	1252	Placebo
	1253	Placebo
	1254	MVA
	1255	MVA
	1256	MVA
	1257	MVA
	1258	Placebo
	1259	Placebo
	1260	Placebo
71	1261	MVA
	1262	Placebo
	1263	MVA
	1264	MVA
	1265	Placebo
	1266	Placebo
	1267	Placebo
	1268	Placebo
	1269	MVA
	1270	MVA
	1271	Placebo
	1272	MVA
	1273	Placebo
	1274	Placebo
	1275	MVA
	1276	Placebo
	1277	MVA
	1278	MVA
72	1279	Placebo
	1280	Placebo
	1281	Placebo
	1282	Placebo
	1283	Placebo
	1284	MVA
	1285	Placebo
	1286	MVA
	1287	Placebo
	1288	MVA
	1289	Placebo
	1290	MVA
	1291	MVA
	1292	MVA
	1293	MVA
	1294	Placebo
	1295	MVA
	1296	MVA

Vial randomisation list for BN study POX-MVA-005 (P031363)
Seed= 83461674

Container	Vial Randomization Number	Treatment
73	1297	MVA
	1298	Placebo
	1299	Placebo
	1300	MVA
	1301	MVA
	1302	Placebo
	1303	MVA
	1304	MVA
	1305	Placebo
	1306	MVA
	1307	Placebo
	1308	MVA
	1309	Placebo
	1310	Placebo
	1311	Placebo
	1312	MVA
	1313	Placebo
	1314	MVA
74	1315	Placebo
	1316	Placebo
	1317	MVA
	1318	MVA
	1319	MVA
	1320	Placebo
	1321	Placebo
	1322	Placebo
	1323	Placebo
	1324	Placebo
	1325	MVA
	1326	MVA
	1327	MVA
	1328	MVA
	1329	Placebo
	1330	Placebo
	1331	MVA
	1332	MVA
75	1333	MVA
	1334	Placebo
	1335	MVA
	1336	Placebo
	1337	Placebo
	1338	Placebo
	1339	MVA
	1340	MVA
	1341	Placebo
	1342	MVA
	1343	MVA
	1344	Placebo
	1345	MVA
	1346	Placebo
	1347	MVA
	1348	Placebo
	1349	MVA
	1350	Placebo

Vial randomisation list for BN study POX-MVA-005 (P031363)
Seed= 83461674

Container	Vial Randomization Number	Treatment
76	1351	Placebo
	1352	MVA
	1353	MVA
	1354	Placebo
	1355	Placebo
	1356	MVA
	1357	Placebo
	1358	Placebo
	1359	Placebo
	1360	MVA
	1361	MVA
	1362	Placebo
	1363	Placebo
	1364	MVA
	1365	MVA
	1366	MVA
	1367	Placebo
	1368	MVA
77	1369	Placebo
	1370	MVA
	1371	Placebo
	1372	Placebo
	1373	MVA
	1374	Placebo
	1375	MVA
	1376	MVA
	1377	MVA
	1378	Placebo
	1379	MVA
	1380	MVA
	1381	Placebo
	1382	Placebo
	1383	Placebo
	1384	Placebo
	1385	MVA
	1386	MVA
78	1387	MVA
	1388	MVA
	1389	MVA
	1390	Placebo
	1391	Placebo
	1392	Placebo
	1393	MVA
	1394	MVA
	1395	Placebo
	1396	Placebo
	1397	Placebo
	1398	Placebo
	1399	MVA
	1400	Placebo
	1401	MVA
	1402	MVA
	1403	MVA
	1404	Placebo

Vial randomisation list for BN study POX-MVA-005 (P031363)
Seed= 83461674

Container	Vial Randomization Number	Treatment
79	1405	Placebo
	1406	MVA
	1407	Placebo
	1408	MVA
	1409	MVA
	1410	Placebo
	1411	MVA
	1412	Placebo
	1413	Placebo
	1414	MVA
	1415	Placebo
	1416	Placebo
	1417	MVA
	1418	Placebo
	1419	MVA
	1420	Placebo
	1421	MVA
	1422	MVA
80	1423	Placebo
	1424	Placebo
	1425	Placebo
	1426	MVA
	1427	MVA
	1428	MVA
	1429	Placebo
	1430	MVA
	1431	MVA
	1432	MVA
	1433	MVA
	1434	Placebo
	1435	Placebo
	1436	MVA
	1437	Placebo
	1438	Placebo
	1439	Placebo
	1440	MVA
81	1441	MVA
	1442	Placebo
	1443	Placebo
	1444	MVA
	1445	Placebo
	1446	MVA
	1447	Placebo
	1448	MVA
	1449	MVA
	1450	Placebo
	1451	Placebo
	1452	MVA
	1453	Placebo
	1454	Placebo
	1455	MVA
	1456	MVA
	1457	MVA
	1458	Placebo

Vial randomisation list for BN study POX-MVA-005 (P031363)
Seed= 83461674

Container	Vial Randomization Number	Treatment
82	1459	MVA
	1460	Placebo
	1461	Placebo
	1462	Placebo
	1463	MVA
	1464	Placebo
	1465	Placebo
	1466	MVA
	1467	MVA
	1468	MVA
	1469	Placebo
	1470	Placebo
	1471	MVA
	1472	Placebo
	1473	MVA
	1474	Placebo
	1475	MVA
	1476	MVA
83	1477	MVA
	1478	MVA
	1479	Placebo
	1480	MVA
	1481	Placebo
	1482	MVA
	1483	MVA
	1484	Placebo
	1485	Placebo
	1486	Placebo
	1487	MVA
	1488	MVA
	1489	MVA
	1490	Placebo
	1491	Placebo
	1492	Placebo
	1493	Placebo
	1494	MVA
84	1495	Placebo
	1496	MVA
	1497	MVA
	1498	MVA
	1499	MVA
	1500	Placebo
	1501	MVA
	1502	Placebo
	1503	Placebo
	1504	MVA
	1505	Placebo
	1506	MVA
	1507	MVA
	1508	Placebo
	1509	Placebo
	1510	Placebo
	1511	MVA
	1512	Placebo

Vial randomisation list for BN study POX-MVA-005 (P031363)
Seed= 83461674

Container	Vial Randomization Number	Treatment
85	1513	MVA
	1514	Placebo
	1515	MVA
	1516	Placebo
	1517	MVA
	1518	MVA
	1519	MVA
	1520	MVA
	1521	Placebo
	1522	Placebo
	1523	Placebo
	1524	MVA
	1525	MVA
	1526	Placebo
	1527	Placebo
	1528	Placebo
	1529	Placebo
	1530	MVA
86	1531	Placebo
	1532	Placebo
	1533	Placebo
	1534	MVA
	1535	MVA
	1536	MVA
	1537	MVA
	1538	Placebo
	1539	MVA
	1540	Placebo
	1541	MVA
	1542	Placebo
	1543	MVA
	1544	Placebo
	1545	MVA
	1546	MVA
	1547	Placebo
	1548	Placebo
87	1549	MVA
	1550	MVA
	1551	MVA
	1552	Placebo
	1553	MVA
	1554	MVA
	1555	Placebo
	1556	MVA
	1557	Placebo
	1558	Placebo
	1559	Placebo
	1560	Placebo
	1561	MVA
	1562	MVA
	1563	Placebo
	1564	MVA
	1565	Placebo
	1566	Placebo

Vial randomisation list for BN study POX-MVA-005 (P031363)
Seed= 83461674

Container	Vial Randomization Number	Treatment
88	1567	MVA
	1568	MVA
	1569	MVA
	1570	Placebo
	1571	Placebo
	1572	Placebo
	1573	MVA
	1574	MVA
	1575	MVA
	1576	MVA
	1577	Placebo
	1578	Placebo
	1579	MVA
	1580	Placebo
	1581	MVA
	1582	Placebo
	1583	Placebo
	1584	Placebo
89	1585	Placebo
	1586	MVA
	1587	MVA
	1588	Placebo
	1589	Placebo
	1590	Placebo
	1591	MVA
	1592	MVA
	1593	MVA
	1594	MVA
	1595	Placebo
	1596	Placebo
	1597	Placebo
	1598	MVA
	1599	Placebo
	1600	Placebo
	1601	MVA
	1602	MVA
90	1603	Placebo
	1604	Placebo
	1605	MVA
	1606	MVA
	1607	MVA
	1608	Placebo
	1609	Placebo
	1610	MVA
	1611	Placebo
	1612	Placebo
	1613	MVA
	1614	MVA
	1615	Placebo
	1616	Placebo
	1617	MVA
	1618	MVA
	1619	MVA
	1620	Placebo

Vial randomisation list for BN study POX-MVA-005 (P031363)
Seed= 83461674

Container	Vial Randomization Number	Treatment
91	1621	Placebo
	1622	Placebo
	1623	MVA
	1624	MVA
	1625	Placebo
	1626	Placebo
	1627	MVA
	1628	MVA
	1629	Placebo
	1630	MVA
	1631	MVA
	1632	MVA
	1633	Placebo
	1634	Placebo
	1635	Placebo
	1636	MVA
	1637	Placebo
	1638	MVA
92	1639	Placebo
	1640	Placebo
	1641	MVA
	1642	MVA
	1643	Placebo
	1644	Placebo
	1645	Placebo
	1646	MVA
	1647	Placebo
	1648	Placebo
	1649	MVA
	1650	Placebo
	1651	Placebo
	1652	MVA
	1653	MVA
	1654	MVA
	1655	MVA
	1656	MVA
93	1657	Placebo
	1658	Placebo
	1659	Placebo
	1660	Placebo
	1661	MVA
	1662	MVA
	1663	MVA
	1664	Placebo
	1665	MVA
	1666	MVA
	1667	Placebo
	1668	MVA
	1669	MVA
	1670	Placebo
	1671	MVA
	1672	MVA
	1673	Placebo
	1674	Placebo

Vial randomisation list for BN study POX-MVA-005 (P031363)
Seed= 83461674

Container	Vial Randomization Number	Treatment
94	1675	Placebo
	1676	Placebo
	1677	MVA
	1678	Placebo
	1679	MVA
	1680	Placebo
	1681	Placebo
	1682	Placebo
	1683	MVA
	1684	MVA
	1685	Placebo
	1686	Placebo
	1687	MVA
	1688	Placebo
	1689	MVA
	1690	MVA
	1691	MVA
	1692	MVA
95	1693	Placebo
	1694	MVA
	1695	Placebo
	1696	Placebo
	1697	MVA
	1698	Placebo
	1699	Placebo
	1700	MVA
	1701	Placebo
	1702	Placebo
	1703	MVA
	1704	MVA
	1705	MVA
	1706	Placebo
	1707	Placebo
	1708	MVA
	1709	MVA
	1710	MVA
96	1711	Placebo
	1712	MVA
	1713	Placebo
	1714	Placebo
	1715	MVA
	1716	MVA
	1717	MVA
	1718	MVA
	1719	Placebo
	1720	MVA
	1721	Placebo
	1722	MVA
	1723	Placebo
	1724	MVA
	1725	MVA
	1726	Placebo
	1727	Placebo
	1728	Placebo

Vial randomisation list for BN study POX-MVA-005 (P031363)
Seed= 83461674

Container	Vial Randomization Number	Treatment
97	1729	MVA
	1730	MVA
	1731	Placebo
	1732	Placebo
	1733	Placebo
	1734	MVA
	1735	Placebo
	1736	MVA
	1737	MVA
	1738	MVA
	1739	MVA
	1740	Placebo
	1741	MVA
	1742	Placebo
	1743	Placebo
	1744	MVA
	1745	Placebo
	1746	Placebo
98	1747	MVA
	1748	MVA
	1749	MVA
	1750	MVA
	1751	Placebo
	1752	Placebo
	1753	Placebo
	1754	MVA
	1755	Placebo
	1756	Placebo
	1757	MVA
	1758	MVA
	1759	Placebo
	1760	Placebo
	1761	MVA
	1762	MVA
	1763	Placebo
	1764	Placebo
99	1765	MVA
	1766	Placebo
	1767	Placebo
	1768	Placebo
	1769	Placebo
	1770	MVA
	1771	Placebo
	1772	Placebo
	1773	MVA
	1774	Placebo
	1775	Placebo
	1776	Placebo
	1777	MVA
	1778	MVA
	1779	MVA
	1780	MVA
	1781	MVA
	1782	MVA

Vial randomisation list for BN study POX-MVA-005 (P031363)
Seed= 83461674

Container	Vial Randomization Number	Treatment
100	1783	MVA
	1784	Placebo
	1785	MVA
	1786	Placebo
	1787	Placebo
	1788	MVA
	1789	MVA
	1790	Placebo
	1791	Placebo
	1792	Placebo
	1793	MVA
	1794	MVA
	1795	MVA
	1796	Placebo
	1797	MVA
	1798	Placebo
	1799	MVA
	1800	Placebo
101	1801	Placebo
	1802	Placebo
	1803	Placebo
	1804	Placebo
	1805	MVA
	1806	Placebo
	1807	MVA
	1808	MVA
	1809	MVA
	1810	Placebo
	1811	MVA
	1812	MVA
	1813	MVA
	1814	MVA
	1815	Placebo
	1816	Placebo
	1817	Placebo
	1818	MVA
102	1819	MVA
	1820	MVA
	1821	MVA
	1822	Placebo
	1823	MVA
	1824	Placebo
	1825	Placebo
	1826	MVA
	1827	MVA
	1828	MVA
	1829	Placebo
	1830	MVA
	1831	Placebo
	1832	Placebo
	1833	Placebo
	1834	Placebo
	1835	Placebo
	1836	MVA

Vial randomisation list for BN study POX-MVA-005 (P031363)
Seed= 83461674

Container	Vial Randomization Number	Treatment
103	1837	MVA
	1838	MVA
	1839	MVA
	1840	Placebo
	1841	Placebo
	1842	MVA
	1843	MVA
	1844	MVA
	1845	Placebo
	1846	Placebo
	1847	Placebo
	1848	MVA
	1849	Placebo
	1850	Placebo
	1851	Placebo
	1852	MVA
	1853	Placebo
	1854	MVA
104	1855	Placebo
	1856	Placebo
	1857	MVA
	1858	Placebo
	1859	MVA
	1860	Placebo
	1861	Placebo
	1862	MVA
	1863	MVA
	1864	Placebo
	1865	Placebo
	1866	MVA
	1867	Placebo
	1868	MVA
	1869	Placebo
	1870	MVA
	1871	MVA
	1872	MVA
105	1873	MVA
	1874	MVA
	1875	Placebo
	1876	Placebo
	1877	Placebo
	1878	MVA
	1879	Placebo
	1880	Placebo
	1881	MVA
	1882	Placebo
	1883	MVA
	1884	MVA
	1885	MVA
	1886	Placebo
	1887	MVA
	1888	Placebo
	1889	Placebo
	1890	MVA

Vial randomisation list for BN study POX-MVA-005 (P031363)
Seed= 83461674

Container	Vial Randomization Number	Treatment
106	1891	MVA
	1892	MVA
	1893	Placebo
	1894	Placebo
	1895	Placebo
	1896	MVA
	1897	Placebo
	1898	MVA
	1899	MVA
	1900	Placebo
	1901	Placebo
	1902	Placebo
	1903	Placebo
	1904	MVA
	1905	Placebo
	1906	MVA
	1907	MVA
	1908	MVA
107	1909	MVA
	1910	Placebo
	1911	MVA
	1912	Placebo
	1913	Placebo
	1914	MVA
	1915	Placebo
	1916	MVA
	1917	Placebo
	1918	MVA
	1919	MVA
	1920	MVA
	1921	MVA
	1922	Placebo
	1923	Placebo
	1924	Placebo
	1925	Placebo
	1926	MVA
108	1927	MVA
	1928	Placebo
	1929	Placebo
	1930	MVA
	1931	MVA
	1932	Placebo
	1933	Placebo
	1934	Placebo
	1935	Placebo
	1936	Placebo
	1937	MVA
	1938	MVA
	1939	MVA
	1940	MVA
	1941	Placebo
	1942	Placebo
	1943	MVA
	1944	MVA

Vial randomisation list for BN study POX-MVA-005 (P031363)
Seed= 83461674

Container	Vial Randomization Number	Treatment
109	1945	MVA
	1946	Placebo
	1947	MVA
	1948	Placebo
	1949	MVA
	1950	Placebo
	1951	Placebo
	1952	MVA
	1953	Placebo
	1954	Placebo
	1955	MVA
	1956	Placebo
	1957	MVA
	1958	Placebo
	1959	MVA
	1960	MVA
	1961	Placebo
	1962	MVA
110	1963	Placebo
	1964	Placebo
	1965	Placebo
	1966	MVA
	1967	MVA
	1968	MVA
	1969	MVA
	1970	MVA
	1971	MVA
	1972	MVA
	1973	Placebo
	1974	Placebo
	1975	Placebo
	1976	Placebo
	1977	Placebo
	1978	Placebo
	1979	MVA
	1980	MVA
111	1981	MVA
	1982	Placebo
	1983	MVA
	1984	MVA
	1985	MVA
	1986	Placebo
	1987	Placebo
	1988	Placebo
	1989	MVA
	1990	Placebo
	1991	Placebo
	1992	Placebo
	1993	MVA
	1994	MVA
	1995	MVA
	1996	Placebo
	1997	Placebo
	1998	MVA

Vial randomisation list for BN study POX-MVA-005 (P031363)
Seed= 83461674

Container	Vial Randomization Number	Treatment
112	1999	MVA
	2000	Placebo
	2001	MVA
	2002	MVA
	2003	Placebo
	2004	Placebo
	2005	Placebo
	2006	Placebo
	2007	Placebo
	2008	Placebo
	2009	MVA
	2010	MVA
	2011	MVA
	2012	MVA
	2013	MVA
	2014	MVA
	2015	Placebo
	2016	Placebo
113	2017	MVA
	2018	Placebo
	2019	Placebo
	2020	Placebo
	2021	Placebo
	2022	Placebo
	2023	MVA
	2024	Placebo
	2025	MVA
	2026	Placebo
	2027	MVA
	2028	MVA
	2029	Placebo
	2030	MVA
	2031	Placebo
	2032	MVA
	2033	MVA
	2034	MVA
114	2035	Placebo
	2036	Placebo
	2037	MVA
	2038	MVA
	2039	Placebo
	2040	MVA
	2041	MVA
	2042	Placebo
	2043	MVA
	2044	Placebo
	2045	Placebo
	2046	Placebo
	2047	MVA
	2048	Placebo
	2049	MVA
	2050	MVA
	2051	MVA
	2052	Placebo

Vial randomisation list for BN study POX-MVA-005 (P031363)
Seed= 83461674

Container	Vial Randomization Number	Treatment
115	2053	MVA
	2054	Placebo
	2055	Placebo
	2056	MVA
	2057	MVA
	2058	Placebo
	2059	Placebo
	2060	MVA
	2061	MVA
	2062	MVA
	2063	Placebo
	2064	Placebo
	2065	MVA
	2066	MVA
	2067	MVA
	2068	Placebo
	2069	Placebo
	2070	Placebo
116	2071	MVA
	2072	Placebo
	2073	MVA
	2074	Placebo
	2075	Placebo
	2076	MVA
	2077	Placebo
	2078	Placebo
	2079	Placebo
	2080	MVA
	2081	MVA
	2082	MVA
	2083	MVA
	2084	MVA
	2085	Placebo
	2086	Placebo
	2087	MVA
	2088	Placebo
117	2089	MVA
	2090	Placebo
	2091	MVA
	2092	MVA
	2093	Placebo
	2094	MVA
	2095	Placebo
	2096	Placebo
	2097	MVA
	2098	Placebo
	2099	Placebo
	2100	Placebo
	2101	MVA
	2102	MVA
	2103	MVA
	2104	Placebo
	2105	Placebo
	2106	MVA

Vial randomisation list for BN study POX-MVA-005 (P031363)
Seed= 83461674

Container	Vial Randomization Number	Treatment
118	2107	Placebo
	2108	Placebo
	2109	MVA
	2110	MVA
	2111	MVA
	2112	Placebo
	2113	MVA
	2114	Placebo
	2115	MVA
	2116	Placebo
	2117	MVA
	2118	Placebo
	2119	Placebo
	2120	MVA
	2121	Placebo
	2122	MVA
	2123	Placebo
	2124	MVA
119	2125	Placebo
	2126	Placebo
	2127	Placebo
	2128	MVA
	2129	Placebo
	2130	MVA
	2131	Placebo
	2132	MVA
	2133	MVA
	2134	MVA
	2135	Placebo
	2136	Placebo
	2137	MVA
	2138	MVA
	2139	MVA
	2140	MVA
	2141	Placebo
	2142	Placebo
120	2143	Placebo
	2144	MVA
	2145	Placebo
	2146	Placebo
	2147	Placebo
	2148	MVA
	2149	Placebo
	2150	MVA
	2151	MVA
	2152	MVA
	2153	Placebo
	2154	Placebo
	2155	MVA
	2156	MVA
	2157	Placebo
	2158	MVA
	2159	MVA
	2160	Placebo

Vial randomisation list for BN study POX-MVA-005 (P031363)
Seed= 83461674

Container	Vial Randomization Number	Treatment
121	2161	MVA
	2162	MVA
	2163	Placebo
	2164	MVA
	2165	Placebo
	2166	Placebo
	2167	MVA
	2168	MVA
	2169	MVA
	2170	Placebo
	2171	Placebo
	2172	MVA
	2173	Placebo
	2174	Placebo
	2175	Placebo
	2176	Placebo
	2177	MVA
	2178	MVA
122	2179	Placebo
	2180	Placebo
	2181	MVA
	2182	Placebo
	2183	MVA
	2184	MVA
	2185	MVA
	2186	MVA
	2187	MVA
	2188	Placebo
	2189	Placebo
	2190	Placebo
	2191	Placebo
	2192	MVA
	2193	Placebo
	2194	MVA
	2195	MVA
	2196	Placebo
123	2197	Placebo
	2198	MVA
	2199	Placebo
	2200	MVA
	2201	Placebo
	2202	Placebo
	2203	Placebo
	2204	MVA
	2205	MVA
	2206	MVA
	2207	MVA
	2208	MVA
	2209	MVA
	2210	Placebo
	2211	Placebo
	2212	MVA
	2213	Placebo
	2214	Placebo

Vial randomisation list for BN study POX-MVA-005 (P031363)
Seed= 83461674

Container	Vial Randomization Number	Treatment
124	2215	Placebo
	2216	MVA
	2217	Placebo
	2218	MVA
	2219	Placebo
	2220	MVA
	2221	MVA
	2222	Placebo
	2223	MVA
	2224	MVA
	2225	Placebo
	2226	Placebo
	2227	MVA
	2228	Placebo
	2229	MVA
	2230	Placebo
	2231	Placebo
	2232	MVA
125	2233	MVA
	2234	MVA
	2235	Placebo
	2236	MVA
	2237	Placebo
	2238	Placebo
	2239	Placebo
	2240	Placebo
	2241	Placebo
	2242	MVA
	2243	MVA
	2244	Placebo
	2245	MVA
	2246	MVA
	2247	MVA
	2248	MVA
	2249	Placebo
	2250	Placebo
126	2251	MVA
	2252	Placebo
	2253	MVA
	2254	Placebo
	2255	Placebo
	2256	MVA
	2257	MVA
	2258	Placebo
	2259	MVA
	2260	MVA
	2261	Placebo
	2262	Placebo
	2263	MVA
	2264	Placebo
	2265	Placebo
	2266	MVA
	2267	MVA
	2268	Placebo

Vial randomisation list for BN study POX-MVA-005 (P031363)
Seed= 83461674

Container	Vial Randomization Number	Treatment
127	2269	Placebo
	2270	MVA
	2271	Placebo
	2272	MVA
	2273	MVA
	2274	MVA
	2275	MVA
	2276	MVA
	2277	Placebo
	2278	Placebo
	2279	Placebo
	2280	Placebo
	2281	Placebo
	2282	Placebo
	2283	Placebo
	2284	MVA
	2285	MVA
	2286	MVA
128	2287	Placebo
	2288	Placebo
	2289	Placebo
	2290	Placebo
	2291	Placebo
	2292	MVA
	2293	Placebo
	2294	MVA
	2295	Placebo
	2296	MVA
	2297	MVA
	2298	Placebo
	2299	MVA
	2300	MVA
	2301	MVA
	2302	MVA
	2303	MVA
	2304	Placebo
129	2305	MVA
	2306	MVA
	2307	Placebo
	2308	Placebo
	2309	MVA
	2310	MVA
	2311	Placebo
	2312	Placebo
	2313	Placebo
	2314	MVA
	2315	Placebo
	2316	Placebo
	2317	MVA
	2318	MVA
	2319	Placebo
	2320	Placebo
	2321	MVA
	2322	MVA

Vial randomisation list for BN study POX-MVA-005 (P031363)
Seed= 83461674

Container	Vial Randomization Number	Treatment
130	2323	MVA
	2324	MVA
	2325	Placebo
	2326	MVA
	2327	MVA
	2328	Placebo
	2329	MVA
	2330	Placebo
	2331	MVA
	2332	Placebo
	2333	Placebo
	2334	Placebo
	2335	MVA
	2336	Placebo
	2337	Placebo
	2338	MVA
	2339	Placebo
	2340	MVA
131	2341	MVA
	2342	Placebo
	2343	Placebo
	2344	Placebo
	2345	MVA
	2346	MVA
	2347	MVA
	2348	MVA
	2349	MVA
	2350	Placebo
	2351	Placebo
	2352	MVA
	2353	Placebo
	2354	Placebo
	2355	Placebo
	2356	MVA
	2357	Placebo
	2358	MVA
132	2359	MVA
	2360	MVA
	2361	Placebo
	2362	Placebo
	2363	MVA
	2364	Placebo
	2365	MVA
	2366	Placebo
	2367	MVA
	2368	Placebo
	2369	MVA
	2370	Placebo
	2371	MVA
	2372	Placebo
	2373	MVA
	2374	MVA
	2375	Placebo
	2376	Placebo

Vial randomisation list for BN study POX-MVA-005 (P031363)
Seed= 83461674

Container	Vial Randomization Number	Treatment
133	2377	MVA
	2378	Placebo
	2379	MVA
	2380	MVA
	2381	Placebo
	2382	MVA
	2383	MVA
	2384	MVA
	2385	Placebo
	2386	MVA
	2387	Placebo
	2388	Placebo
	2389	Placebo
	2390	Placebo
	2391	Placebo
	2392	Placebo
	2393	MVA
	2394	MVA

16.1.8 Audit Certificates

AUDIT CERTIFICATE

It is hereby certified that the following Clinical Trial was subject to evaluation by the independent Quality Assurance department of Bavarian Nordic.

**Clinical Trial Number:**	**POX-MVA-005 (EudraCT No 2005-001781-14), DMID 05-0128**
**Title of Clinical Trial:**	**A Partially Randomized, Partially Double-Blind, Placebo-Controlled Phase II Non-Inferiority Study to Evaluate Immunogenicity and Safety of One and Two Doses of MVA-BN® (IMVAMUNE™) Smallpox Vaccine in 18-55 Year Old Healthy Subjects**
**Contract Research Organization (CRO, on-site)**	**Harrison Clinical Research GmbH, Albrechtstrasse 43, 80636 München**

I confirm that the audit was carried out in accordance with generally accepted auditing standards reflecting ethical rules and GCP guidelines.

The results of the audit were reported to the responsible personnel and corrective actions were performed when considered necessary. The corrective actions were deemed to be appropriate as reviewed and approved by Bavarian Nordic Quality Assurance. The audit was performed on the following dates:

Item audited	Audit Date	Date of Audit Report
General conduct of the trial, Trial Master File, CRO System	February 15, 2007	March 01, 2007

05-July 2007
(DATE)

(SIGNATURE) ROBERT SEMICH, GCP SPECIALIST,
QUALITY ASSURANCE DEVELOPMENT

16.1.9 Documentation of statistical methods

16.1.9 Statistical Considerations

The statistical analysis was followed according to the statistical analysis plan (SAP) Vers. 2.0 (attached to this section). In following, further specifications, the results of statistical tests and the output of statistical software is listed.

Description of the randomisation method

The randomisation list allocated each subject number to one of the four treatment sequences as described in the protocol.
The randomisation list was prepared by Kendle. The list allocated each subject to one of the three sequences (Group 1-3). A separate list of vials ranging from 0001 to 2394 assigns each vial number to one of the two treatments 'MVA-BN' and 'Placebo'.
The block length for the allocation of sequences was 9, i.e. after 9 subjects, 3 in each sequence group were allocated, the block length for the vial list was 18, i.e. after 18 vials, 9 of each treatment were used.

The subject randomisation number used in the Kendle list could not be used in the e-CRF for technical reasons. Therefore, the treatment sequence was strongly followed, however, the e-CRF system has assigned consecutive numbers starting with 001.

Population data sets

The data sets Safety, Full analysis set (FA) and Per Protocol (PP) were classified according to the definition in the protocol and the SAP. Relevant protocol deviations were considered during a data review meeting.

Statistical Analysis

The statistical plan of analysis was followed according to the protocol and the amendments.

Special considerations

Diameter size measurements for solicited adverse events (redness, swelling, induration) have been translated into intensity grades as described in the CRF. The original diameter sizes are not listed in section 16.2 but just the intensity categories.

Index of tables

16.1.9.1. Statistical Output for primary efficacy parameter 7
16.1.9.1.1. ELISA 7
16.1.9.1.2. PRNT 15
16.1.9.2. Statistical Output for descriptive comparison of seroconversion rates among other groups23
16.1.9.2.1. ELISA 23
16.1.9.2.2. PRNT 43

Statistical Results for the primary immunological parameters

Table 1: Primary efficacy parameter: MVA -specific seroconversion rate derived from the **ELISA** specific antibody titers **2 weeks after the last vaccination** (Group 1-3: Visit 4, Group 4: Visit 2)

Population	Seroconversion rate Group 1 (MM)	Seroconversion rate Group 4 (M-)	Difference Group 4 – Group 1	p-value for the test of the null hypothesis of equality of proportions[1] (Fisher's exact test)	p-value for the rejection of the null hypothesis of inferiority	Exact one-sided 97.5% confidence interval for the difference of rates between Group 4 and Group 1
FA	98.9% (174/176)	95.5% (191/200)	-3.4%	0.0675	0.2162	(-7.36%; +∞)
PP	98.8% (166/168)	96.4% (186/193)	-2.4%	0.1837	0.0857	(-6.27%; +∞)

Unconditional exact test for non-inferiority for a binomial difference
The predefined non-inferiority margin was (-5%; +∞).

Table 2: Secondary efficacy parameter: MVA -specific seroconversion rate derived from the **ELISA** specific antibody titers **4 weeks after the last vaccination** (Group 1 3: Visit 5, Group 4: Visit 3)

Population	Seroconversion rate Group 1 (MM)	Seroconversion rate Group 4 (M-)	Difference Group 4 – Group 1	p-value for the test of the null hypothesis of equality of proportions[2] (Fisher's exact test)	p-value for the rejection of the null hypothesis of inferiority	Exact one-sided 97.5% confidence interval for the difference of rates between Group 4 and Group 1
FA	98.9% (176/178)	93.0% (185/199)	-5.9%	**0.0043**	0.7055	(-10.52%; +∞)
PP	98.8% (166/168)	92.7% (178/192)	-6.1%	**0.0046**	0.7452	(-10.84%; +∞)

Unconditional exact test for non-inferiority for a binomial difference
The predefined non-inferiority margin was (-5%; +∞).

[1] This is only done for descriptive purposes and is NOT the p-value for the actual primary null and alternative hypothesis for testing the non-inferiority
[2] This is only done for descriptive purposes and is NOT the p-value for the actual primary null and alternative hypothesis for testing the non-inferiority

Table 3: Primary efficacy parameter: MVA -specific seroconversion rate derived from the **PRNT** specific antibody titers **2 weeks after the last vaccination** (Group 1-3: Visit 4, Group 4: Visit 2)

Population	Seroconversion rate<br>Group 1 (MM)	Seroconversion rate<br>Group 4 (M-)	Difference Group 4 – Group 1	p-value for the test of the null hypothesis of equality of proportions[3] (Fisher's exact test)	p-value for the rejection of the null hypothesis of inferiority	Exact one-sided 97.5% confidence interval for the difference of rates between Group 4 and Group 1
FA	89.2% (157/176)	78.5% (157/200)	-10.7%	**0.0054**	0.9353	(-18.18%; +∞)
PP	88.7% (149/168)	78.8% (152/193)	-9.9%	**0.0155**	0.9017	(-17.60%; +∞)

Unconditional exact test for non-inferiority for a binomial difference
The predefined non-inferiority margin was (-5%; +∞).

Table 4: Secondary efficacy parameter: MVA -specific seroconversion rate derived from the **PRNT** specific antibody titers **4 weeks after the last vaccination** (Group 1 3: Visit 5, Group 4: Visit 3)

Population	Seroconversion rate<br>Group 1 (MM)	Seroconversion rate<br>Group 4 (M-)	Difference Group 4 – Group 1	p-value for the test of the null hypothesis of equality of proportions[4] (Fisher's exact test)	p-value for the rejection of the null hypothesis of inferiority	Exact one-sided 97.5% confidence interval for the difference of rates between Group 4 and Group 1
FA	86.0% (153/178)	69.8% (139/199)	-16.2%	**0.0002**	0.9957	(-24.40%; +∞)
PP	85.7% (144/168)	68.8% (132/192)	-16.9%	**0.0002**	0.9969	(-25.54%; +∞)

Unconditional exact test for non-inferiority for a binomial difference
The predefined non-inferiority margin was (-5%; +∞).

[3] This is only done for descriptive purposes and is NOT the p-value for the actual primary null and alternative hypothesis for testing the non-inferiority
[4] This is only done for descriptive purposes and is NOT the p-value for the actual primary null and alternative hypothesis for testing the non-inferiority

Table 5: Fisher's exact test for MVA -specific seroconversion rate derived from the ELISA specific antibody titers 2 weeks after the last vaccination (Group 1-3: Visit 4, Group 4: Visit 2)

**Population**	**Comparison**	**Fisher's Exact Test P-value (2-Tail)**
FA	Group 1 <-> 4	0.0675
	Group 1 <-> 2	**<.0001**
	Group 1 <-> 3	**<.0001**
	Group 2 <-> 4	**<.0001**
	Group 3 <-> 4	**<.0001**
	Group 2 <-> 3	**<.0001**
PP	Group 1 <-> 4	0.1837
	Group 1 <-> 2	**<.0001**
	Group 1 <-> 3	**<.0001**
	Group 2 <-> 4	**<.0001**
	Group 3 <-> 4	**<.0001**
	Group 2 <-> 3	**<.0001**

Significant p-values (<0.05) are marked bold

Table 6: Fisher's exact test for MVA -specific seroconversion rate derived from the ELISA specific antibody titers 4 weeks after the last vaccination (Group 1 3: Visit 5, Group 4: Visit 3

**Population**	**Comparison**	**Fisher's Exact Test P-value (2-Tail)**
FA	Group 1 <-> 4	**0.0043**
	Group 1 <-> 2	**<.0001**
	Group 1 <-> 3	**<.0001**
	Group 2 <-> 4	**<.0001**
	Group 3 <-> 4	**<.0001**
	Group 2 <-> 3	**<.0001**
PP	Group 1 <-> 4	**0.0046**
	Group 1 <-> 2	**<.0001**
	Group 1 <-> 3	**<.0001**
	Group 2 <-> 4	**<.0001**
	Group 3 <-> 4	**<.0001**
	Group 2 <-> 3	**<.0001**

Significant p-values (<0.05) are marked bold

Table 7: Fisher's exact test for MVA -specific seroconversion rate derived from the PRNT specific antibody titers 2 weeks after the last vaccination (Group 1-3: Visit 4, Group 4: Visit 2)

**Population**	**Comparison**	**Fisher's Exact Test P-value (2-Tail)**
FA	Group 1 <-> 4	**0.0054**
	Group 1 <-> 2	**<.0001**
	Group 1 <-> 3	**<.0001**
	Group 2 <-> 4	**<.0001**
	Group 3 <-> 4	**<.0001**
	Group 2 <-> 3	**<.0001**
PP	Group 1 <-> 4	**0.0155**
	Group 1 <-> 2	**<.0001**
	Group 1 <-> 3	**<.0001**
	Group 2 <-> 4	**<.0001**
	Group 3 <-> 4	**<.0001**
	Group 2 <-> 3	**<.0001**

Significant p-values (<0.05) are marked bold

Table 8: Fisher's exact test for MVA -specific seroconversion rate derived from the PRNT specific antibody titers 4 weeks after the last vaccination (Group 1 3: Visit 5, Group 4: Visit 3

**Population**	**Comparison**	**Fisher's Exact Test P-value (2-Tail)**
FA	Group 1 <-> 4	**0.0002**
	Group 1 <-> 2	**<.0001**
	Group 1 <-> 3	**<.0001**
	Group 2 <-> 4	**<.0001**
	Group 3 <-> 4	**<.0001**
	Group 2 <-> 3	**<.0001**
PP	Group 1 <-> 4	**0.0002**
	Group 1 <-> 2	**<.0001**
	Group 1 <-> 3	**<.0001**
	Group 2 <-> 4	**<.0001**
	Group 3 <-> 4	**<.0001**
	Group 2 <-> 3	**<.0001**

Significant p-values (<0.05) are marked bold

The raw output of statistical software is given on the following pages.

Index of Statistical Output:

16.1.9.1. Statistical Output for primary efficacy parameter 7

16.1.9.1.1. ELISA 7

16.1.9.1.2. PRNT 15

16.1.9.2. Statistical Output for descriptive comparison of seroconversion rates among other groups23

16.1.9.2.1. ELISA 23

16.1.9.1. Statistical Output for primary efficacy parameter

16.1.9.1.1. ELISA

```
Seroconversion rate, 2 weeks after last vaccination, FA dataset
Fisher's exact test for testing superiority

Output from StatXact (r) PROCs (v7.0) _SAS9.1_
Copyright (c) 1987-2005 Cytel Software Corporation, Cambridge, MA, USA.

FISHER'S EXACT TEST

Data file name : < ELISA >
Population Variable Name : treat
Outcome Variable Name : sero2wk



Statistic based on the observed  2 x  2 table(x) :
Two-sided Tests                Obs.Stat.  AsympP-val   ExactP-val   Pt.Prob.
Pearson ChiSquare Test          3.7296      0.0535      0.0675      0.0394
Pearson with Yates CC           2.6392
Fisher's Exact Test             3.6502      0.0561      0.0675      0.0394
Hypergeometric Prob.            0.0394
Likelihood Ratio Test           4.0773      0.0435      0.0675      0.0394

One-sided : Let y be the value in Row 1 and Column 1
    y = 191 min(Y) = 189 max(Y) = 200 mean(Y) =    194.1489 std(Y) =      1.6327

          Pr { Y .LE.  191 } =      0.0489
          Pr { Y .EQ.  191 } =      0.0394
```

Geometric mean calculated from computing the arithmetic mean of the log-transformed values (i.e. the arithmetic mean on the log scale)
and then using the exponentiation to return the geometric mean
Other descriptive statistics are based on log-transformed values

```
Seroconversion rate, 2 weeks after last vaccination, FA dataset
Unconditional, exact test and confidence interval for non-inferiority

Output from StatXact (r) PROCs (v7.0) _SAS9.1_
Copyright (c) 1987-2005 Cytel Software Corporation, Cambridge, MA, USA.

UNCONDITIONAL TEST OF NON-INFERIORITY USING DIFFERENCE OF TWO BINOMIAL PROPORTIONS

Data file name : < ELISA >
Population Variable Name : treat
Outcome Variable Name : sero2wk

    HO:(pi_2-pi_1) .GE. delta_0 vs H1: (pi_2-pi_1) .LT. delta_0

Statistics based on the observed  2 by  2 table :

    Observed proportion for population <        1> : piHat_1             =      0.9550
    Observed proportion for population <        2> : piHat_2             =      0.9886
    Observed difference of proportions : piHat_2-piHat_1                 =      0.0336
    Maximum  margin of non-inferiority : pi_2-pi_1 = delta_0             =      0.0500
    Stderr (restricted mle of stdev of pi_2-pi_1-delta_0 given delta_0)  =      0.0181
    Standardized test statistic (t):(piHat_2-piHat_1-delta_0)/Stderr     =     -0.9050

Results:
---------------------------------------------------------------------
                1-sided P-value              97.50% Upper Confidence
     Method       Pr{T .LE. t}                 Bound for pi_2-pi_1
---------------------------------------------------------------------
      Asymp            0.1827                       0.0735
      Exact            0.2162                       0.0736
```

Geometric mean calculated from computing the arithmetic mean of the log-transformed values (i.e. the arithmetic mean on the log scale)
and then using the exponentiation to return the geometric mean
Other descriptive statistics are based on log-transformed values

```
Seroconversion rate, 2 weeks after last vaccination, PP dataset
Fisher's exact test for testing superiority

Output from StatXact (r) PROCs (v7.0) _SAS9.1_
Copyright (c) 1987-2005 Cytel Software Corporation, Cambridge, MA, USA.

FISHER'S EXACT TEST

Data file name : < ELISA >
Population Variable Name : treat
Outcome Variable Name : sero2wk



Statistic based on the observed  2 x  2 table(x) :
Two-sided Tests                Obs.Stat.  AsympP-val   ExactP-val   Pt.Prob.
Pearson ChiSquare Test          2.1934      0.1386      0.1837      0.0958
Pearson with Yates CC           1.3056
Fisher's Exact Test             2.0715      0.1501      0.1837      0.0958
Hypergeometric Prob.            0.0958
Likelihood Ratio Test           2.3462      0.1256      0.1837      0.0958

One-sided : Let y be the value in Row 1 and Column 1
    y = 186 min(Y) = 184 max(Y) = 193 mean(Y) =    188.1884 std(Y) =      1.4797

          Pr { Y .LE.  186 } =      0.1258
          Pr { Y .EQ.  186 } =      0.0958
```

Geometric mean calculated from computing the arithmetic mean of the log-transformed values (i.e. the arithmetic mean on the log scale)
and then using the exponentiation to return the geometric mean
Other descriptive statistics are based on log-transformed values

```
Seroconversion rate, 2 weeks after last vaccination, PP dataset
Unconditional, exact test and confidence interval for non-inferiority

Output from StatXact (r) PROCs (v7.0) _SAS9.1_
Copyright (c) 1987-2005 Cytel Software Corporation, Cambridge, MA, USA.

UNCONDITIONAL TEST OF NON-INFERIORITY USING DIFFERENCE OF TWO BINOMIAL PROPORTIONS

Data file name : < ELISA >
Population Variable Name : treat
Outcome Variable Name : sero2wk

    HO:(pi_2-pi_1) .GE. delta_0 vs H1: (pi_2-pi_1) .LT. delta_0

Statistics based on the observed  2 by  2 table :

    Observed proportion for population <        1> : piHat_1             =      0.9637
    Observed proportion for population <        2> : piHat_2             =      0.9881
    Observed difference of proportions : piHat_2-piHat_1                 =      0.0244
    Maximum  margin of non-inferiority : pi_2-pi_1 = delta_0             =      0.0500
    Stderr (restricted mle of stdev of pi_2-pi_1-delta_0 given delta_0)  =      0.0182
    Standardized test statistic (t):(piHat_2-piHat_1-delta_0)/Stderr     =     -1.4065

Results:
---------------------------------------------------------------------
                1-sided P-value              97.50% Upper Confidence
     Method       Pr{T .LE. t}                 Bound for pi_2-pi_1
---------------------------------------------------------------------
      Asymp            0.0798                        0.0627
      Exact            0.0857                        0.0627
```

Geometric mean calculated from computing the arithmetic mean of the log-transformed values (i.e. the arithmetic mean on the log scale)
and then using the exponentiation to return the geometric mean
Other descriptive statistics are based on log-transformed values

```
Seroconversion rate, 4 weeks after last vaccination, FA dataset
Fisher's exact test for testing superiority

Output from StatXact (r) PROCs (v7.0) _SAS9.1_
Copyright (c) 1987-2005 Cytel Software Corporation, Cambridge, MA, USA.

FISHER'S EXACT TEST

Data file name : < ELISA >
Population Variable Name : treat
Outcome Variable Name : sero4wk



Statistic based on the observed  2 x  2 table(x) :
Two-sided Tests                Obs.Stat.  AsympP-val   ExactP-val   Pt.Prob.
Pearson ChiSquare Test          8.0797      0.0045      0.0043      0.0030
Pearson with Yates CC           6.6905
Fisher's Exact Test             8.4412      0.0037      0.0043      0.0030
Hypergeometric Prob.            0.0030
Likelihood Ratio Test           9.1781      0.0024      0.0043      0.0030

One-sided : Let y be the value in Row 1 and Column 1
    y = 185 min(Y) = 183 max(Y) = 199 mean(Y) =    190.5544 std(Y) =      1.9567

          Pr { Y .LE.  185 } =      0.0034
          Pr { Y .EQ.  185 } =      0.0030
```

Geometric mean calculated from computing the arithmetic mean of the log-transformed values (i.e. the arithmetic mean on the log scale)
and then using the exponentiation to return the geometric mean
Other descriptive statistics are based on log-transformed values

```
Seroconversion rate, 4 weeks after last vaccination, FA dataset
Unconditional, exact test and confidence interval for non-inferiority

Output from StatXact (r) PROCs (v7.0) _SAS9.1_
Copyright (c) 1987-2005 Cytel Software Corporation, Cambridge, MA, USA.

UNCONDITIONAL TEST OF NON-INFERIORITY USING DIFFERENCE OF TWO BINOMIAL PROPORTIONS

Data file name : < ELISA >
Population Variable Name : treat
Outcome Variable Name : sero4wk

    H0:(pi_2-pi_1) .GE. delta_0 vs H1: (pi_2-pi_1) .LT. delta_0

Statistics based on the observed  2 by  2 table :

    Observed proportion for population <        1> : piHat_1              =      0.9296
    Observed proportion for population <        2> : piHat_2              =      0.9888
    Observed difference of proportions : piHat_2-piHat_1                  =      0.0591
    Maximum  margin of non-inferiority : pi_2-pi_1 = delta_0              =      0.0500
    Stderr (restricted mle of stdev of pi_2-pi_1-delta_0 given delta_0)   =      0.0192
    Standardized test statistic (t):(piHat_2-piHat_1-delta_0)/Stderr      =      0.4746

Results:
---------------------------------------------------------------------
                1-sided P-value              97.50% Upper Confidence
     Method       Pr{T .LE. t}                Bound for pi_2-pi_1
---------------------------------------------------------------------
      Asymp            0.6825                        0.1049
      Exact            0.7055                        0.1052
```

Geometric mean calculated from computing the arithmetic mean of the log-transformed values (i.e. the arithmetic mean on the log scale)
and then using the exponentiation to return the geometric mean
Other descriptive statistics are based on log-transformed values

```
Seroconversion rate, 4 weeks after last vaccination, PP dataset
Fisher's exact test for testing superiority

Output from StatXact (r) PROCs (v7.0) _SAS9.1_
Copyright (c) 1987-2005 Cytel Software Corporation, Cambridge, MA, USA.

FISHER'S EXACT TEST

Data file name : < ELISA >
Population Variable Name : treat
Outcome Variable Name : sero4wk



Statistic based on the observed  2 x  2 table(x) :
Two-sided Tests                Obs.Stat.  AsympP-val   ExactP-val   Pt.Prob.
Pearson ChiSquare Test          7.8535      0.0051      0.0082      0.0034
Pearson with Yates CC           6.4826
Fisher's Exact Test             8.2068      0.0042      0.0046      0.0034
Hypergeometric Prob.            0.0034
Likelihood Ratio Test           8.9416      0.0028      0.0046      0.0034

One-sided : Let y be the value in Row 1 and Column 1
    y = 178 min(Y) = 176 max(Y) = 192 mean(Y) =    183.4667 std(Y) =      1.9534

          Pr { Y .LE.  178 } =      0.0039
          Pr { Y .EQ.  178 } =      0.0034
```

Geometric mean calculated from computing the arithmetic mean of the log-transformed values (i.e. the arithmetic mean on the log scale)
and then using the exponentiation to return the geometric mean
Other descriptive statistics are based on log-transformed values

```
Seroconversion rate, 4 weeks after last vaccination, PP dataset
Unconditional, exact test and confidence interval for non-inferiority

Output from StatXact (r) PROCs (v7.0) _SAS9.1_
Copyright (c) 1987-2005 Cytel Software Corporation, Cambridge, MA, USA.

UNCONDITIONAL TEST OF NON-INFERIORITY USING DIFFERENCE OF TWO BINOMIAL PROPORTIONS

Data file name : < ELISA >
Population Variable Name : treat
Outcome Variable Name : sero4wk

    HO:(pi_2-pi_1) .GE. delta_0 vs H1: (pi_2-pi_1) .LT. delta_0

Statistics based on the observed  2 by  2 table :

    Observed proportion for population <        1> : piHat_1             =      0.9271
    Observed proportion for population <        2> : piHat_2             =      0.9881
    Observed difference of proportions : piHat_2-piHat_1                 =      0.0610
    Maximum  margin of non-inferiority : pi_2-pi_1 = delta_0             =      0.0500
    Stderr (restricted mle of stdev of pi_2-pi_1-delta_0 given delta_0)  =      0.0199
    Standardized test statistic (t):(piHat_2-piHat_1-delta_0)/Stderr     =      0.5530

Results:
---------------------------------------------------------------------
                1-sided P-value              97.50% Upper Confidence
     Method       Pr{T .LE. t}                 Bound for pi_2-pi_1
---------------------------------------------------------------------
      Asymp            0.7099                        0.1084
      Exact            0.7452                        0.1084
```

Geometric mean calculated from computing the arithmetic mean of the log-transformed values (i.e. the arithmetic mean on the log scale)
and then using the exponentiation to return the geometric mean
Other descriptive statistics are based on log-transformed values

16.1.9.1.2. PRNT

Fisher's exact test for testing superiority

Output from StatXact (r) PROCs (v7.0) _SAS9.1_
Copyright (c) 1987-2005 Cytel Software Corporation, Cambridge, MA, USA.

FISHER'S EXACT TEST

Data file name : < PRNT >
Population Variable Name : treat
Outcome Variable Name : sero2wk

Statistic based on the observed 2 x 2 table(x) :

Two-sided Tests	Obs.Stat.	AsympP-val	ExactP-val	Pt.Prob.
Pearson ChiSquare Test	7.7901	0.0053	0.0054	0.0022
Pearson with Yates CC	7.0322			
Fisher's Exact Test	7.8606	0.0051	0.0054	0.0022
Hypergeometric Prob.	0.0022			
Likelihood Ratio Test	8.0045	0.0047	0.0054	0.0022

One-sided : Let y be the value in Row 1 and Column 1
y = 157 min(Y) = 138 max(Y) = 200 mean(Y) = 167.0213 std(Y) = 3.5952

Pr { Y .LE. 157 } = 0.0037
Pr { Y .EQ. 157 } = 0.0022

Geometric mean calculated from computing the arithmetic mean of the log-transformed values (i.e. the arithmetic mean on the log scale)
and then using the exponentiation to return the geometric mean
Other descriptive statistics are based on log-transformed values

```
Seroconversion rate, 2 weeks after last vaccination, FA dataset
Unconditional, exact test and confidence interval for non-inferiority

Output from StatXact (r) PROCs (v7.0) _SAS9.1_
Copyright (c) 1987-2005 Cytel Software Corporation, Cambridge, MA, USA.

UNCONDITIONAL TEST OF NON-INFERIORITY USING DIFFERENCE OF TWO BINOMIAL PROPORTIONS

Data file name : < PRNT >
Population Variable Name : treat
Outcome Variable Name : sero2wk

    HO:(pi_2-pi_1) .GE. delta_0 vs H1: (pi_2-pi_1) .LT. delta_0

Statistics based on the observed  2 by  2 table :

    Observed proportion for population <        1> : piHat_1              =      0.7850
    Observed proportion for population <        2> : piHat_2              =      0.8920
    Observed difference of proportions : piHat_2-piHat_1                  =      0.1070
    Maximum  margin of non-inferiority : pi_2-pi_1 = delta_0              =      0.0500
    Stderr (restricted mle of stdev of pi_2-pi_1-delta_0 given delta_0)   =      0.0376
    Standardized test statistic (t):(piHat_2-piHat_1-delta_0)/Stderr      =      1.5166

Results:
---------------------------------------------------------------------
                1-sided P-value              97.50% Upper Confidence
     Method       Pr{T .LE. t}                 Bound for pi_2-pi_1
---------------------------------------------------------------------
      Asymp            0.9353                       0.1809
      Exact            0.9353                       0.1818
```

Geometric mean calculated from computing the arithmetic mean of the log-transformed values (i.e. the arithmetic mean on the log scale)
and then using the exponentiation to return the geometric mean
Other descriptive statistics are based on log-transformed values

```
Seroconversion rate, 2 weeks after last vaccination, PP dataset
Fisher's exact test for testing superiority

Output from StatXact (r) PROCs (v7.0) _SAS9.1_
Copyright (c) 1987-2005 Cytel Software Corporation, Cambridge, MA, USA.

FISHER'S EXACT TEST

Data file name : < PRNT >
Population Variable Name : treat
Outcome Variable Name : sero2wk



Statistic based on the observed  2 x  2 table(x) :
Two-sided Tests                Obs.Stat.  AsympP-val   ExactP-val   Pt.Prob.
Pearson ChiSquare Test          6.3959      0.0114      0.0155      0.0045
Pearson with Yates CC           5.6992
Fisher's Exact Test             6.4306      0.0112      0.0155      0.0045
Hypergeometric Prob.            0.0045
Likelihood Ratio Test           6.5551      0.0105      0.0155      0.0045

One-sided : Let y be the value in Row 1 and Column 1
    y = 152 min(Y) = 133 max(Y) = 193 mean(Y) =    160.9224 std(Y) =      3.5329

          Pr { Y .LE.  152 } =      0.0080
          Pr { Y .EQ.  152 } =      0.0045
```

Geometric mean calculated from computing the arithmetic mean of the log-transformed values (i.e. the arithmetic mean on the log scale)
and then using the exponentiation to return the geometric mean
Other descriptive statistics are based on log-transformed values

```
Seroconversion rate, 2 weeks after last vaccination, PP dataset
Unconditional, exact test and confidence interval for non-inferiority

Output from StatXact (r) PROCs (v7.0) _SAS9.1_
Copyright (c) 1987-2005 Cytel Software Corporation, Cambridge, MA, USA.

UNCONDITIONAL TEST OF NON-INFERIORITY USING DIFFERENCE OF TWO BINOMIAL PROPORTIONS

Data file name : < PRNT >
Population Variable Name : treat
Outcome Variable Name : sero2wk

    HO:(pi_2-pi_1) .GE. delta_0 vs H1: (pi_2-pi_1) .LT. delta_0

Statistics based on the observed  2 by  2 table :

    Observed proportion for population <       1> : piHat_1             =      0.7876
    Observed proportion for population <       2> : piHat_2             =      0.8869
    Observed difference of proportions : piHat_2-piHat_1                =      0.0993
    Maximum  margin of non-inferiority : pi_2-pi_1 = delta_0            =      0.0500
    Stderr (restricted mle of stdev of pi_2-pi_1-delta_0 given delta_0)  =      0.0386
    Standardized test statistic (t):(piHat_2-piHat_1-delta_0)/Stderr     =      1.2797

Results:
--------------------------------------------------------------------
                1-sided P-value             97.50% Upper Confidence
     Method       Pr{T .LE. t}               Bound for pi_2-pi_1
--------------------------------------------------------------------
      Asymp            0.8997                     0.1750
      Exact            0.9017                     0.1760
```

Geometric mean calculated from computing the arithmetic mean of the log-transformed values (i.e. the arithmetic mean on the log scale)
and then using the exponentiation to return the geometric mean
Other descriptive statistics are based on log-transformed values

```
Seroconversion rate, 4 weeks after last vaccination, FA dataset
Fisher's exact test for testing superiority

Output from StatXact (r) PROCs (v7.0) _SAS9.1_
Copyright (c) 1987-2005 Cytel Software Corporation, Cambridge, MA, USA.

FISHER'S EXACT TEST

Data file name : < PRNT >
Population Variable Name : treat
Outcome Variable Name : sero4wk



Statistic based on the observed  2 x  2 table(x) :
Two-sided Tests                Obs.Stat.  AsympP-val   ExactP-val   Pt.Prob.
Pearson ChiSquare Test         13.9565      0.0002      0.0002      0.0001
Pearson with Yates CC          13.0495
Fisher's Exact Test            14.1766      0.0002      0.0002      0.0001
Hypergeometric Prob.            0.0001
Likelihood Ratio Test          14.3506      0.0002      0.0002      0.0001

One-sided : Let y be the value in Row 1 and Column 1
    y = 139 min(Y) = 114 max(Y) = 199 mean(Y) =    154.1326 std(Y) =      4.0560

          Pr { Y .LE.  139 } =      0.0001
          Pr { Y .EQ.  139 } =      0.0001
```

Geometric mean calculated from computing the arithmetic mean of the log-transformed values (i.e. the arithmetic mean on the log scale)
and then using the exponentiation to return the geometric mean
Other descriptive statistics are based on log-transformed values

Seroconversion rate, 4 weeks after last vaccination, FA dataset
Unconditional, exact test and confidence interval for non-inferiority

Output from StatXact (r) PROCs (v7.0) _SAS9.1_
Copyright (c) 1987-2005 Cytel Software Corporation, Cambridge, MA, USA.

UNCONDITIONAL TEST OF NON-INFERIORITY USING DIFFERENCE OF TWO BINOMIAL PROPORTIONS

```
Data file name : < PRNT >
Population Variable Name : treat
Outcome Variable Name : sero4wk

    HO:(pi_2-pi_1) .GE. delta_0 vs H1: (pi_2-pi_1) .LT. delta_0

Statistics based on the observed  2 by  2 table :

    Observed proportion for population <        1> : piHat_1              =      0.6985
    Observed proportion for population <        2> : piHat_2              =      0.8596
    Observed difference of proportions : piHat_2-piHat_1                  =      0.1611
    Maximum  margin of non-inferiority : pi_2-pi_1 = delta_0              =      0.0500
    Stderr (restricted mle of stdev of pi_2-pi_1-delta_0 given delta_0)   =      0.0425
    Standardized test statistic (t):(piHat_2-piHat_1-delta_0)/Stderr      =      2.6116
```

Results:

Method	1-sided P-value Pr{T .LE. t}	97.50% Upper Confidence Bound for pi_2-pi_1
Asymp	0.9955	0.2424
Exact	0.9957	0.2440

Geometric mean calculated from computing the arithmetic mean of the log-transformed values (i.e. the arithmetic mean on the log scale)
and then using the exponentiation to return the geometric mean
Other descriptive statistics are based on log-transformed values

```
Seroconversion rate, 4 weeks after last vaccination, PP dataset
Fisher's exact test for testing superiority

Output from StatXact (r) PROCs (v7.0) _SAS9.1_
Copyright (c) 1987-2005 Cytel Software Corporation, Cambridge, MA, USA.

FISHER'S EXACT TEST

Data file name : < PRNT >
Population Variable Name : treat
Outcome Variable Name : sero4wk



Statistic based on the observed  2 x  2 table(x) :
Two-sided Tests                Obs.Stat.  AsympP-val   ExactP-val   Pt.Prob.
Pearson ChiSquare Test         14.4144      0.0001      0.0002      0.0001
Pearson with Yates CC          13.4817
Fisher's Exact Test            14.6724      0.0001      0.0002      0.0001
Hypergeometric Prob.            0.0001
Likelihood Ratio Test          14.8602      0.0001      0.0002      0.0001

One-sided : Let y be the value in Row 1 and Column 1
    y = 132 min(Y) = 108 max(Y) = 192 mean(Y) =    147.2000 std(Y) =      4.0091

          Pr { Y .LE.  132 } =      0.0001
          Pr { Y .EQ.  132 } =      0.0001
```

Geometric mean calculated from computing the arithmetic mean of the log-transformed values (i.e. the arithmetic mean on the log scale)
and then using the exponentiation to return the geometric mean
Other descriptive statistics are based on log-transformed values

```
Seroconversion rate, 4 weeks after last vaccination, PP dataset
Unconditional, exact test and confidence interval for non-inferiority

Output from StatXact (r) PROCs (v7.0) _SAS9.1_
Copyright (c) 1987-2005 Cytel Software Corporation, Cambridge, MA, USA.

UNCONDITIONAL TEST OF NON-INFERIORITY USING DIFFERENCE OF TWO BINOMIAL PROPORTIONS

Data file name : < PRNT >
Population Variable Name : treat
Outcome Variable Name : sero4wk

    HO:(pi_2-pi_1) .GE. delta_0 vs H1: (pi_2-pi_1) .LT. delta_0

Statistics based on the observed  2 by  2 table :

    Observed proportion for population <        1> : piHat_1              =      0.6875
    Observed proportion for population <        2> : piHat_2              =      0.8571
    Observed difference of proportions : piHat_2-piHat_1                  =      0.1696
    Maximum  margin of non-inferiority : pi_2-pi_1 = delta_0              =      0.0500
    Stderr (restricted mle of stdev of pi_2-pi_1-delta_0 given delta_0)   =      0.0441
    Standardized test statistic (t):(piHat_2-piHat_1-delta_0)/Stderr      =      2.7155

Results:
---------------------------------------------------------------------
                1-sided P-value              97.50% Upper Confidence
     Method       Pr{T .LE. t}                 Bound for pi_2-pi_1
---------------------------------------------------------------------
      Asymp             0.9967                       0.2533
      Exact             0.9969                       0.2554
```

Geometric mean calculated from computing the arithmetic mean of the log-transformed values (i.e. the arithmetic mean on the log scale)
and then using the exponentiation to return the geometric mean
Other descriptive statistics are based on log-transformed values

16.1.9.2. Statistical Output for descriptive comparison of seroconversion rates among other groups

16.1.9.2.1. ELISA

Fisher's exact test for descriptive comparison of seroconversion rates among other groups

The FREQ Procedure

Table of treat by sero2wk

treat(Group) Frequency / Percent / Row Pct / Col Pct	sero2wk 1	2	Total
Group 4 (M-)	191<br>50.80<br>95.50<br>52.33	9<br>2.39<br>4.50<br>81.82	200<br>53.19
Group 1 (MM)	174<br>46.28<br>98.86<br>47.67	2<br>0.53<br>1.14<br>18.18	176<br>46.81
Total	365<br>97.07	11<br>2.93	376<br>100.00

Fisher's exact test for descriptive comparison of seroconversion rates among other groups

The FREQ Procedure

Statistics for Table of treat by sero2wk

Statistic	DF	Value	Prob
Chi-Square	1	3.7296	0.0535
Likelihood Ratio Chi-Square	1	4.0773	0.0435
Continuity Adj. Chi-Square	1	2.6392	0.1043
Mantel-Haenszel Chi-Square	1	3.7197	0.0538
Phi Coefficient		-0.0996	
Contingency Coefficient		0.0991	
Cramer's V		-0.0996	

Fisher's Exact Test	
Cell (1,1) Frequency (F)	191
Left-sided Pr <= F	0.0489
Right-sided Pr >= F	0.9905
Table Probability (P)	0.0394
Two-sided Pr <= P	0.0675

Sample Size = 376

Fisher's exact test for descriptive comparison of seroconversion rates among other groups

The FREQ Procedure

Table of treat by sero2wk

treat(Group) sero2wk

Frequency<br>Percent<br>Row Pct<br>Col Pct	1	2	Total
Group 4 (M-)	191<br>51.07<br>95.50<br>57.19	9<br>2.41<br>4.50<br>22.50	200<br>53.48
Group 2 (MP)	143<br>38.24<br>82.18<br>42.81	31<br>8.29<br>17.82<br>77.50	174<br>46.52
Total	334<br>89.30	40<br>10.70	374<br>100.00

Fisher's exact test for descriptive comparison of seroconversion rates among other groups

The FREQ Procedure

Statistics for Table of treat by sero2wk

Statistic	DF	Value	Prob
Chi-Square	1	17.2742	<.0001
Likelihood Ratio Chi-Square	1	17.9119	<.0001
Continuity Adj. Chi-Square	1	15.9082	<.0001
Mantel-Haenszel Chi-Square	1	17.2280	<.0001
Phi Coefficient		0.2149	
Contingency Coefficient		0.2101	
Cramer's V		0.2149	

Fisher's Exact Test	
Cell (1,1) Frequency (F)	191
Left-sided Pr <= F	1.0000
Right-sided Pr >= F	2.578E-05
Table Probability (P)	2.058E-05
Two-sided Pr <= P	3.659E-05

Sample Size = 374

Fisher's exact test for descriptive comparison of seroconversion rates among other groups

The FREQ Procedure

Table of treat by sero2wk

treat(Group) sero2wk

Frequency<br>Percent<br>Row Pct<br>Col Pct	1	2	Total
Group 4 (M-)	191<br>50.93<br>95.50<br>96.95	9<br>2.40<br>4.50<br>5.06	200<br>53.33
Group 3 (PP)	6<br>1.60<br>3.43	169<br>45.07<br>96.57	175<br>46.67

	3.05	94.94	
Total	197	178	375
	52.53	47.47	100.00

Fisher's exact test for descriptive comparison of seroconversion rates among other groups

The FREQ Procedure

Statistics for Table of treat by sero2wk

Statistic	DF	Value	Prob
Chi-Square	1	317.2947	<.0001
Likelihood Ratio Chi-Square	1	393.2206	<.0001
Continuity Adj. Chi-Square	1	313.6131	<.0001
Mantel-Haenszel Chi-Square	1	316.4486	<.0001
Phi Coefficient		0.9198	
Contingency Coefficient		0.6770	
Cramer's V		0.9198	

Fisher's Exact Test	
Cell (1,1) Frequency (F)	191
Left-sided Pr <= F	1.0000
Right-sided Pr >= F	2.197E-86
Table Probability (P)	1.012E-83
Two-sided Pr <= P	2.197E-86

Sample Size = 375

Fisher's exact test for descriptive comparison of seroconversion rates among other groups

The FREQ Procedure

Table of treat by sero2wk

treat(Group) sero2wk

Frequency<br>Percent<br>Row Pct<br>Col Pct	1	2	Total
Group 1 (MM)	174<br>49.71<br>98.86<br>54.89	2<br>0.57<br>1.14<br>6.06	176<br>50.29
Group 2 (MP)	143<br>40.86<br>82.18<br>45.11	31<br>8.86<br>17.82<br>93.94	174<br>49.71
Total	317<br>90.57	33<br>9.43	350<br>100.00

Fisher's exact test for descriptive comparison of seroconversion rates among other groups

The FREQ Procedure

Statistics for Table of treat by sero2wk

Statistic	DF	Value	Prob
Chi-Square	1	28.5059	<.0001
Likelihood Ratio Chi-Square	1	33.6830	<.0001

Statistic	DF	Value	Prob
Continuity Adj. Chi-Square	1	26.5861	<.0001
Mantel-Haenszel Chi-Square	1	28.4245	<.0001
Phi Coefficient		0.2854	
Contingency Coefficient		0.2744	
Cramer's V		0.2854	

Fisher's Exact Test

Cell (1,1) Frequency (F)	174
Left-sided Pr <= F	1.0000
Right-sided Pr >= F	1.505E-08
Table Probability (P)	1.430E-08
Two-sided Pr <= P	1.615E-08

Sample Size = 350

Fisher's exact test for descriptive comparison of seroconversion rates among other groups

The FREQ Procedure

Table of treat by sero2wk

treat(Group) sero2wk

Frequency / Percent / Row Pct / Col Pct	1	2	Total
Group 1 (MM)	174<br>49.57<br>98.86<br>96.67	2<br>0.57<br>1.14<br>1.17	176<br>50.14
Group 3 (PP)	6<br>1.71<br>3.43<br>3.33	169<br>48.15<br>96.57<br>98.83	175<br>49.86
Total	180<br>51.28	171<br>48.72	351<br>100.00

Fisher's exact test for descriptive comparison of seroconversion rates among other groups

The FREQ Procedure

Statistics for Table of treat by sero2wk

Statistic	DF	Value	Prob
Chi-Square	1	319.8933	<.0001
Likelihood Ratio Chi-Square	1	412.2038	<.0001
Continuity Adj. Chi-Square	1	316.0848	<.0001
Mantel-Haenszel Chi-Square	1	318.9819	<.0001
Phi Coefficient		0.9547	
Contingency Coefficient		0.6905	
Cramer's V		0.9547	

Fisher's Exact Test

Cell (1,1) Frequency (F)	174
Left-sided Pr <= F	1.0000
Right-sided Pr >= F	3.239E-90
Table Probability (P)	4.534E-87

Two-sided Pr <= P 3.277E-90

Sample Size = 351

Fisher's exact test for descriptive comparison of seroconversion rates among other groups

The FREQ Procedure

Table of treat by sero2wk

treat(Group) sero2wk

Frequency / Percent / Row Pct / Col Pct	1	2	Total
Group 2 (MP)	143<br>40.97<br>82.18<br>95.97	31<br>8.88<br>17.82<br>15.50	174<br>49.86
Group 3 (PP)	6<br>1.72<br>3.43<br>4.03	169<br>48.42<br>96.57<br>84.50	175<br>50.14
Total	149<br>42.69	200<br>57.31	349<br>100.00

Fisher's exact test for descriptive comparison of seroconversion rates among other groups

The FREQ Procedure

Statistics for Table of treat by sero2wk

Statistic	DF	Value	Prob
Chi-Square	1	221.1854	<.0001
Likelihood Ratio Chi-Square	1	260.9986	<.0001
Continuity Adj. Chi-Square	1	217.9782	<.0001
Mantel-Haenszel Chi-Square	1	220.5516	<.0001
Phi Coefficient		0.7961	
Contingency Coefficient		0.6228	
Cramer's V		0.7961	

Fisher's Exact Test	
Cell (1,1) Frequency (F)	143
Left-sided Pr <= F	1.0000
Right-sided Pr >= F	6.358E-58
Table Probability (P)	6.807E-56
Two-sided Pr <= P	6.622E-58

Sample Size = 349

Fisher's exact test for descriptive comparison of seroconversion rates among other groups

The FREQ Procedure

Table of treat by sero2wk

treat(Group) sero2wk

Frequency
Percent
Row Pct

Col Pct	1	2	Total
Group 4 (M-)	186	7	193
	51.52	1.94	53.46
	96.37	3.63	
	52.84	77.78	
Group 1 (MM)	166	2	168
	45.98	0.55	46.54
	98.81	1.19	
	47.16	22.22	
Total	352	9	361
	97.51	2.49	100.00

Fisher's exact test for descriptive comparison of seroconversion rates among other groups

The FREQ Procedure

Statistics for Table of treat by sero2wk

Statistic	DF	Value	Prob
Chi-Square	1	2.1934	0.1386
Likelihood Ratio Chi-Square	1	2.3462	0.1256
Continuity Adj. Chi-Square	1	1.3056	0.2532
Mantel-Haenszel Chi-Square	1	2.1873	0.1392
Phi Coefficient		-0.0779	
Contingency Coefficient		0.0777	
Cramer's V		-0.0779	

WARNING: 50% of the cells have expected counts less than 5. Chi-Square may not be a valid test.

Fisher's Exact Test

Cell (1,1) Frequency (F)	186
Left-sided Pr <= F	0.1258
Right-sided Pr >= F	0.9701
Table Probability (P)	0.0958
Two-sided Pr <= P	0.1837

Sample Size = 361

Fisher's exact test for descriptive comparison of seroconversion rates among other groups

The FREQ Procedure

Table of treat by sero2wk

treat(Group) sero2wk

Frequency<br>Percent<br>Row Pct<br>Col Pct	1	2	Total
Group 4 (M-)	186	7	193
	51.24	1.93	53.17
	96.37	3.63	
	57.23	18.42	
Group 2 (MP)	139	31	170
	38.29	8.54	46.83
	81.76	18.24	
	42.77	81.58	

Total	325	38	363
	89.53	10.47	100.00

Fisher's exact test for descriptive comparison of seroconversion rates among other groups

The FREQ Procedure

Statistics for Table of treat by sero2wk

Statistic	DF	Value	Prob
Chi-Square	1	20.5801	<.0001
Likelihood Ratio Chi-Square	1	21.7351	<.0001
Continuity Adj. Chi-Square	1	19.0510	<.0001
Mantel-Haenszel Chi-Square	1	20.5234	<.0001
Phi Coefficient		0.2381	
Contingency Coefficient		0.2316	
Cramer's V		0.2381	

Fisher's Exact Test

Cell (1,1) Frequency (F)	186
Left-sided Pr <= F	1.0000
Right-sided Pr >= F	3.977E-06
Table Probability (P)	3.351E-06
Two-sided Pr <= P	4.586E-06

Sample Size = 363

Fisher's exact test for descriptive comparison of seroconversion rates among other groups

The FREQ Procedure

Table of treat by sero2wk

treat(Group) sero2wk

Frequency<br>Percent<br>Row Pct<br>Col Pct	1	2	Total
Group 4 (M-)	186<br>52.10<br>96.37<br>96.88	7<br>1.96<br>3.63<br>4.24	193<br>54.06
Group 3 (PP)	6<br>1.68<br>3.66<br>3.13	158<br>44.26<br>96.34<br>95.76	164<br>45.94
Total	192<br>53.78	165<br>46.22	357<br>100.00

Fisher's exact test for descriptive comparison of seroconversion rates among other groups

The FREQ Procedure

Statistics for Table of treat by sero2wk

Statistic	DF	Value	Prob
Chi-Square	1	306.6053	<.0001
Likelihood Ratio Chi-Square	1	381.2102	<.0001
Continuity Adj. Chi-Square	1	302.8868	<.0001

Mantel-Haenszel Chi-Square	1	305.7465	<.0001
Phi Coefficient		0.9267	
Contingency Coefficient		0.6797	
Cramer's V		0.9267	

Fisher's Exact Test

Cell (1,1) Frequency (F)	186
Left-sided Pr <= F	1.0000
Right-sided Pr >= F	9.782E-84
Table Probability (P)	5.126E-81
Two-sided Pr <= P	9.782E-84

Sample Size = 357

Fisher's exact test for descriptive comparison of seroconversion rates among other groups

The FREQ Procedure

Table of treat by sero2wk

treat(Group) sero2wk

Frequency / Percent / Row Pct / Col Pct	1	2	Total
Group 1 (MM)	166<br>49.11<br>98.81<br>54.43	2<br>0.59<br>1.19<br>6.06	168<br>49.70
Group 2 (MP)	139<br>41.12<br>81.76<br>45.57	31<br>9.17<br>18.24<br>93.94	170<br>50.30
Total	305<br>90.24	33<br>9.76	338<br>100.00

Fisher's exact test for descriptive comparison of seroconversion rates among other groups

The FREQ Procedure

Statistics for Table of treat by sero2wk

Statistic	DF	Value	Prob
Chi-Square	1	27.8642	<.0001
Likelihood Ratio Chi-Square	1	33.0395	<.0001
Continuity Adj. Chi-Square	1	25.9630	<.0001
Mantel-Haenszel Chi-Square	1	27.7817	<.0001
Phi Coefficient		0.2871	
Contingency Coefficient		0.2760	
Cramer's V		0.2871	

Fisher's Exact Test

Cell (1,1) Frequency (F)	166
Left-sided Pr <= F	1.0000
Right-sided Pr >= F	2.080E-08
Table Probability (P)	1.975E-08
Two-sided Pr <= P	3.500E-08

Sample Size = 338
Fisher's exact test for descriptive comparison of seroconversion rates among other groups

The FREQ Procedure

Table of treat by sero2wk

treat(Group) sero2wk

Frequency / Percent / Row Pct / Col Pct	1	2	Total
Group 1 (MM)	166<br>50.00<br>98.81<br>96.51	2<br>0.60<br>1.19<br>1.25	168<br>50.60
Group 3 (PP)	6<br>1.81<br>3.66<br>3.49	158<br>47.59<br>96.34<br>98.75	164<br>49.40
Total	172<br>51.81	160<br>48.19	332<br>100.00

Fisher's exact test for descriptive comparison of seroconversion rates among other groups

The FREQ Procedure

Statistics for Table of treat by sero2wk

Statistic	DF	Value	Prob
Chi-Square	1	300.9327	<.0001
Likelihood Ratio Chi-Square	1	386.6415	<.0001
Continuity Adj. Chi-Square	1	297.1337	<.0001
Mantel-Haenszel Chi-Square	1	300.0263	<.0001
Phi Coefficient		0.9521	
Contingency Coefficient		0.6895	
Cramer's V		0.9521	

Fisher's Exact Test

Cell (1,1) Frequency (F)	166
Left-sided Pr <= F	1.0000
Right-sided Pr >= F	1.121E-84
Table Probability (P)	1.400E-81
Two-sided Pr <= P	1.164E-84

Sample Size = 332
Fisher's exact test for descriptive comparison of seroconversion rates among other groups

The FREQ Procedure

Table of treat by sero2wk

treat(Group) sero2wk

Frequency / Percent / Row Pct / Col Pct	1	2	Total

Group 2 (MP)	139<br>41.62<br>81.76<br>95.86	31<br>9.28<br>18.24<br>16.40	170<br>50.90
Group 3 (PP)	6<br>1.80<br>3.66<br>4.14	158<br>47.31<br>96.34<br>83.60	164<br>49.10
Total	145<br>43.41	189<br>56.59	334<br>100.00

Fisher's exact test for descriptive comparison of seroconversion rates among other groups

The FREQ Procedure

Statistics for Table of treat by sero2wk

Statistic	DF	Value	Prob
Chi-Square	1	207.2908	<.0001
Likelihood Ratio Chi-Square	1	244.2535	<.0001
Continuity Adj. Chi-Square	1	204.1236	<.0001
Mantel-Haenszel Chi-Square	1	206.6702	<.0001
Phi Coefficient		0.7878	
Contingency Coefficient		0.6188	
Cramer's V		0.7878	

Fisher's Exact Test

Cell (1,1) Frequency (F)	139
Left-sided Pr <= F	1.0000
Right-sided Pr >= F	2.709E-54
Table Probability (P)	2.634E-52
Two-sided Pr <= P	3.766E-54

Sample Size = 334

Fisher's exact test for descriptive comparison of seroconversion rates among other groups

The FREQ Procedure

Table of treat by sero4wk

treat(Group) sero4wk

Frequency<br>Percent<br>Row Pct<br>Col Pct	1	2	Total
Group 4 (M-)	185<br>49.07<br>92.96<br>51.25	14<br>3.71<br>7.04<br>87.50	199<br>52.79
Group 1 (MM)	176<br>46.68<br>98.88<br>48.75	2<br>0.53<br>1.12<br>12.50	178<br>47.21
Total	361<br>95.76	16<br>4.24	377<br>100.00

Fisher's exact test for descriptive comparison of seroconversion rates among other groups

The FREQ Procedure

Statistics for Table of treat by sero4wk

Statistic	DF	Value	Prob
Chi-Square	1	8.0797	0.0045
Likelihood Ratio Chi-Square	1	9.1781	0.0024
Continuity Adj. Chi-Square	1	6.6905	0.0097
Mantel-Haenszel Chi-Square	1	8.0583	0.0045
Phi Coefficient		-0.1464	
Contingency Coefficient		0.1449	
Cramer's V		-0.1464	

Fisher's Exact Test

Cell (1,1) Frequency (F)	185
Left-sided Pr <= F	0.0034
Right-sided Pr >= F	0.9996
Table Probability (P)	0.0030
Two-sided Pr <= P	0.0043

Sample Size = 377

Fisher's exact test for descriptive comparison of seroconversion rates among other groups

The FREQ Procedure

Table of treat by sero4wk

treat(Group) sero4wk

Frequency<br>Percent<br>Row Pct<br>Col Pct	1	2	Total
Group 4 (M-)	185<br>49.47<br>92.96<br>59.49	14<br>3.74<br>7.04<br>22.22	199<br>53.21
Group 2 (MP)	126<br>33.69<br>72.00<br>40.51	49<br>13.10<br>28.00<br>77.78	175<br>46.79
Total	311<br>83.16	63<br>16.84	374<br>100.00

Fisher's exact test for descriptive comparison of seroconversion rates among other groups

The FREQ Procedure

Statistics for Table of treat by sero4wk

Statistic	DF	Value	Prob
Chi-Square	1	29.2176	<.0001
Likelihood Ratio Chi-Square	1	30.3134	<.0001
Continuity Adj. Chi-Square	1	27.7401	<.0001
Mantel-Haenszel Chi-Square	1	29.1395	<.0001
Phi Coefficient		0.2795	
Contingency Coefficient		0.2692	
Cramer's V		0.2795	

Fisher's Exact Test

Cell (1,1) Frequency (F)	185
Left-sided Pr <= F	1.0000
Right-sided Pr >= F	4.298E-08
Table Probability (P)	3.501E-08
Two-sided Pr <= P	6.483E-08

Sample Size = 374

Fisher's exact test for descriptive comparison of seroconversion rates among other groups

The FREQ Procedure

Table of treat by sero4wk

treat(Group) sero4wk

Frequency / Percent / Row Pct / Col Pct	1	2	Total
Group 4 (M-)	185<br>49.20<br>92.96<br>97.37	14<br>3.72<br>7.04<br>7.53	199<br>52.93
Group 3 (PP)	5<br>1.33<br>2.82<br>2.63	172<br>45.74<br>97.18<br>92.47	177<br>47.07
Total	190<br>50.53	186<br>49.47	376<br>100.00

Fisher's exact test for descriptive comparison of seroconversion rates among other groups

The FREQ Procedure

Statistics for Table of treat by sero4wk

Statistic	DF	Value	Prob
Chi-Square	1	304.4966	<.0001
Likelihood Ratio Chi-Square	1	374.3695	<.0001
Continuity Adj. Chi-Square	1	300.9012	<.0001
Mantel-Haenszel Chi-Square	1	303.6867	<.0001
Phi Coefficient		0.8999	
Contingency Coefficient		0.6689	
Cramer's V		0.8999	

Fisher's Exact Test

Cell (1,1) Frequency (F)	185
Left-sided Pr <= F	1.0000
Right-sided Pr >= F	2.427E-82
Table Probability (P)	8.562E-80
Two-sided Pr <= P	2.655E-82

Sample Size = 376

Fisher's exact test for descriptive comparison of seroconversion rates among other groups

The FREQ Procedure

Table of treat by sero4wk

treat(Group) sero4wk

Frequency / Percent / Row Pct / Col Pct	1	2	Total
Group 1 (MM)	176	2	178
	49.86	0.57	50.42
	98.88	1.12	
	58.28	3.92	
Group 2 (MP)	126	49	175
	35.69	13.88	49.58
	72.00	28.00	
	41.72	96.08	
Total	302	51	353
	85.55	14.45	100.00

Fisher's exact test for descriptive comparison of seroconversion rates among other groups

The FREQ Procedure

Statistics for Table of treat by sero4wk

Statistic	DF	Value	Prob
Chi-Square	1	51.5701	<.0001
Likelihood Ratio Chi-Square	1	62.1167	<.0001
Continuity Adj. Chi-Square	1	49.4186	<.0001
Mantel-Haenszel Chi-Square	1	51.4240	<.0001
Phi Coefficient		0.3822	
Contingency Coefficient		0.3570	
Cramer's V		0.3822	

Fisher's Exact Test

Cell (1,1) Frequency (F)	176
Left-sided Pr <= F	1.0000
Right-sided Pr >= F	1.011E-14
Table Probability (P)	9.770E-15
Two-sided Pr <= P	1.087E-14

Sample Size = 353

Fisher's exact test for descriptive comparison of seroconversion rates among other groups

The FREQ Procedure

Table of treat by sero4wk

treat(Group) sero4wk

Frequency / Percent / Row Pct / Col Pct	1	2	Total
Group 1 (MM)	176	2	178
	49.58	0.56	50.14
	98.88	1.12	

	97.24	1.15	
Group 3 (PP)	5	172	177
	1.41	48.45	49.86
	2.82	97.18	
	2.76	98.85	
Total	181	174	355
	50.99	49.01	100.00

Fisher's exact test for descriptive comparison of seroconversion rates among other groups

The FREQ Procedure

Statistics for Table of treat by sero4wk

Statistic	DF	Value	Prob
Chi-Square	1	327.6442	<.0001
Likelihood Ratio Chi-Square	1	424.5399	<.0001
Continuity Adj. Chi-Square	1	323.8119	<.0001
Mantel-Haenszel Chi-Square	1	326.7213	<.0001
Phi Coefficient		0.9607	
Contingency Coefficient		0.6928	
Cramer's V		0.9607	

Fisher's Exact Test	
Cell (1,1) Frequency (F)	176
Left-sided Pr <= F	1.0000
Right-sided Pr >= F	7.433E-93
Table Probability (P)	1.250E-89
Two-sided Pr <= P	7.519E-93

Sample Size = 355

Fisher's exact test for descriptive comparison of seroconversion rates among other groups

The FREQ Procedure

Table of treat by sero4wk

treat(Group) sero4wk

Frequency<br>Percent<br>Row Pct<br>Col Pct	1	2	Total
Group 2 (MP)	126	49	175
	35.80	13.92	49.72
	72.00	28.00	
	96.18	22.17	
Group 3 (PP)	5	172	177
	1.42	48.86	50.28
	2.82	97.18	
	3.82	77.83	
Total	131	221	352
	37.22	62.78	100.00

Fisher's exact test for descriptive comparison of seroconversion rates among other groups

The FREQ Procedure

Statistics for Table of treat by sero4wk

Statistic	DF	Value	Prob
Chi-Square	1	180.2148	<.0001
Likelihood Ratio Chi-Square	1	211.6485	<.0001
Continuity Adj. Chi-Square	1	177.2664	<.0001
Mantel-Haenszel Chi-Square	1	179.7029	<.0001
Phi Coefficient		0.7155	
Contingency Coefficient		0.5819	
Cramer's V		0.7155	

Fisher's Exact Test

Cell (1,1) Frequency (F)	126
Left-sided Pr <= F	1.0000
Right-sided Pr >= F	3.018E-47
Table Probability (P)	2.156E-45
Two-sided Pr <= P	3.426E-47

Sample Size = 352

Fisher's exact test for descriptive comparison of seroconversion rates among other groups

The FREQ Procedure

Table of treat by sero4wk

treat(Group) sero4wk

Frequency<br>Percent<br>Row Pct<br>Col Pct	1	2	Total
Group 4 (M-)	178<br>49.44<br>92.71<br>51.74	14<br>3.89<br>7.29<br>87.50	192<br>53.33
Group 1 (MM)	166<br>46.11<br>98.81<br>48.26	2<br>0.56<br>1.19<br>12.50	168<br>46.67
Total	344<br>95.56	16<br>4.44	360<br>100.00

Fisher's exact test for descriptive comparison of seroconversion rates among other groups

The FREQ Procedure

Statistics for Table of treat by sero4wk

Statistic	DF	Value	Prob
Chi-Square	1	7.8535	0.0051
Likelihood Ratio Chi-Square	1	8.9416	0.0028
Continuity Adj. Chi-Square	1	6.4826	0.0109
Mantel-Haenszel Chi-Square	1	7.8317	0.0051
Phi Coefficient		-0.1477	
Contingency Coefficient		0.1461	
Cramer's V		-0.1477	

Fisher's Exact Test

Cell (1,1) Frequency (F)	178
Left-sided Pr <= F	0.0039
Right-sided Pr >= F	0.9995
Table Probability (P)	0.0034
Two-sided Pr <= P	0.0046

Sample Size = 360

Fisher's exact test for descriptive comparison of seroconversion rates among other groups

The FREQ Procedure

Table of treat by sero4wk

treat(Group) / Frequency / Percent / Row Pct / Col Pct	sero4wk: 1	2	Total
Group 4 (M-)	178<br>49.17<br>92.71<br>59.33	14<br>3.87<br>7.29<br>22.58	192<br>53.04
Group 2 (MP)	122<br>33.70<br>71.76<br>40.67	48<br>13.26<br>28.24<br>77.42	170<br>46.96
Total	300<br>82.87	62<br>17.13	362<br>100.00

Fisher's exact test for descriptive comparison of seroconversion rates among other groups

The FREQ Procedure

Statistics for Table of treat by sero4wk

Statistic	DF	Value	Prob
Chi-Square	1	27.8644	<.0001
Likelihood Ratio Chi-Square	1	28.8916	<.0001
Continuity Adj. Chi-Square	1	26.4084	<.0001
Mantel-Haenszel Chi-Square	1	27.7874	<.0001
Phi Coefficient		0.2774	
Contingency Coefficient		0.2673	
Cramer's V		0.2774	

Fisher's Exact Test

Cell (1,1) Frequency (F)	178
Left-sided Pr <= F	1.0000
Right-sided Pr >= F	8.835E-08
Table Probability (P)	7.154E-08
Two-sided Pr <= P	1.189E-07

Sample Size = 362

Fisher's exact test for descriptive comparison of seroconversion rates among other groups

The FREQ Procedure

Table of treat by sero4wk

treat(Group) sero4wk

Frequency<br>Percent<br>Row Pct<br>Col Pct	1	2	Total
Group 4 (M-)	178<br>50.00<br>92.71<br>97.27	14<br>3.93<br>7.29<br>8.09	192<br>53.93
Group 3 (PP)	5<br>1.40<br>3.05<br>2.73	159<br>44.66<br>96.95<br>91.91	164<br>46.07
Total	183<br>51.40	173<br>48.60	356<br>100.00

Fisher's exact test for descriptive comparison of seroconversion rates among other groups

The FREQ Procedure

Statistics for Table of treat by sero4wk

Statistic	DF	Value	Prob
Chi-Square	1	284.6368	<.0001
Likelihood Ratio Chi-Square	1	348.2199	<.0001
Continuity Adj. Chi-Square	1	281.0589	<.0001
Mantel-Haenszel Chi-Square	1	283.8372	<.0001
Phi Coefficient		0.8942	
Contingency Coefficient		0.6666	
Cramer's V		0.8942	

Fisher's Exact Test

Cell (1,1) Frequency (F)	178
Left-sided Pr <= F	1.0000
Right-sided Pr >= F	1.129E-76
Table Probability (P)	3.541E-74
Two-sided Pr <= P	1.337E-76

Sample Size = 356

Fisher's exact test for descriptive comparison of seroconversion rates among other groups

The FREQ Procedure

Table of treat by sero4wk

treat(Group) sero4wk

Frequency<br>Percent<br>Row Pct<br>Col Pct	1	2	Total
Group 1 (MM)	166<br>49.11<br>98.81<br>57.64	2<br>0.59<br>1.19<br>4.00	168<br>49.70
Group 2 (MP)	122<br>36.09	48<br>14.20	170<br>50.30

	71.76	28.24	
	42.36	96.00	
Total	288	50	338
	85.21	14.79	100.00

Fisher's exact test for descriptive comparison of seroconversion rates among other groups

The FREQ Procedure

Statistics for Table of treat by sero4wk

Statistic	DF	Value	Prob
Chi-Square	1	49.0321	<.0001
Likelihood Ratio Chi-Square	1	59.2571	<.0001
Continuity Adj. Chi-Square	1	46.9099	<.0001
Mantel-Haenszel Chi-Square	1	48.8870	<.0001
Phi Coefficient		0.3809	
Contingency Coefficient		0.3559	
Cramer's V		0.3809	

Fisher's Exact Test	
Cell (1,1) Frequency (F)	166
Left-sided Pr <= F	1.0000
Right-sided Pr >= F	4.232E-14
Table Probability (P)	4.108E-14
Two-sided Pr <= P	6.456E-14

Sample Size = 338

Fisher's exact test for descriptive comparison of seroconversion rates among other groups

The FREQ Procedure

Table of treat by sero4wk

treat(Group) sero4wk

Frequency<br>Percent<br>Row Pct<br>Col Pct	1	2	Total
Group 1 (MM)	166	2	168
	50.00	0.60	50.60
	98.81	1.19	
	97.08	1.24	
Group 3 (PP)	5	159	164
	1.51	47.89	49.40
	3.05	96.95	
	2.92	98.76	
Total	171	161	332
	51.51	48.49	100.00

Fisher's exact test for descriptive comparison of seroconversion rates among other groups

The FREQ Procedure

Statistics for Table of treat by sero4wk

Statistic	DF	Value	Prob
Chi-Square	1	304.6802	<.0001

Likelihood Ratio Chi-Square	1	393.4988	<.0001
Continuity Adj. Chi-Square	1	300.8584	<.0001
Mantel-Haenszel Chi-Square	1	303.7625	<.0001
Phi Coefficient		0.9580	
Contingency Coefficient		0.6918	
Cramer's V		0.9580	

Fisher's Exact Test

Cell (1,1) Frequency (F)	166
Left-sided Pr <= F	1.0000
Right-sided Pr >= F	3.960E-86
Table Probability (P)	5.804E-83
Two-sided Pr <= P	4.160E-86

Sample Size = 332

Fisher's exact test for descriptive comparison of seroconversion rates among other groups

The FREQ Procedure

Table of treat by sero4wk

treat(Group) Frequency / Percent / Row Pct / Col Pct	sero4wk 1	2	Total
Group 2 (MP)	122<br>36.53<br>71.76<br>96.06	48<br>14.37<br>28.24<br>23.19	170<br>50.90
Group 3 (PP)	5<br>1.50<br>3.05<br>3.94	159<br>47.60<br>96.95<br>76.81	164<br>49.10
Total	127<br>38.02	207<br>61.98	334<br>100.00

Fisher's exact test for descriptive comparison of seroconversion rates among other groups

The FREQ Procedure

Statistics for Table of treat by sero4wk

Statistic	DF	Value	Prob
Chi-Square	1	167.2553	<.0001
Likelihood Ratio Chi-Square	1	196.5678	<.0001
Continuity Adj. Chi-Square	1	164.3521	<.0001
Mantel-Haenszel Chi-Square	1	166.7546	<.0001
Phi Coefficient		0.7076	
Contingency Coefficient		0.5776	
Cramer's V		0.7076	

Fisher's Exact Test

Cell (1,1) Frequency (F)	122
Left-sided Pr <= F	1.0000
Right-sided Pr >= F	5.637E-44

```
Table Probability (P)    3.674E-42
Two-sided Pr <= P        5.848E-44

Sample Size = 334
```

16.1.9.2.2. PRNT

The FREQ Procedure

Table of treat by sero2wk

treat(Group) sero2wk

Frequency<br>Percent<br>Row Pct<br>Col Pct	1	2	Total
Group 4 (M-)	157<br>41.76<br>78.50<br>50.00	43<br>11.44<br>21.50<br>69.35	200<br>53.19
Group 1 (MM)	157<br>41.76<br>89.20<br>50.00	19<br>5.05<br>10.80<br>30.65	176<br>46.81
Total	314<br>83.51	62<br>16.49	376<br>100.00

Fisher's exact test for descriptive comparison of seroconversion rates among other groups

The FREQ Procedure

Statistics for Table of treat by sero2wk

Statistic	DF	Value	Prob
Chi-Square	1	7.7901	0.0053
Likelihood Ratio Chi-Square	1	8.0045	0.0047
Continuity Adj. Chi-Square	1	7.0322	0.0080
Mantel-Haenszel Chi-Square	1	7.7694	0.0053
Phi Coefficient		-0.1439	
Contingency Coefficient		0.1425	
Cramer's V		-0.1439	

Fisher's Exact Test

Cell (1,1) Frequency (F)	157
Left-sided Pr <= F	0.0037
Right-sided Pr >= F	0.9985
Table Probability (P)	0.0022
Two-sided Pr <= P	0.0054

Sample Size = 376

Fisher's exact test for descriptive comparison of seroconversion rates among other groups

The FREQ Procedure

Table of treat by sero2wk

treat(Group) sero2wk

Frequency<br>Percent<br>Row Pct<br>Col Pct	1	2	Total
Group 4 (M-)	157	43	200

	1	2	Total
	41.98	11.50	53.48
	78.50	21.50	
	61.57	36.13	
Group 2 (MP)	98	76	174
	26.20	20.32	46.52
	56.32	43.68	
	38.43	63.87	
Total	255	119	374
	68.18	31.82	100.00

Fisher's exact test for descriptive comparison of seroconversion rates among other groups

The FREQ Procedure

Statistics for Table of treat by sero2wk

Statistic	DF	Value	Prob
Chi-Square	1	21.0967	<.0001
Likelihood Ratio Chi-Square	1	21.2388	<.0001
Continuity Adj. Chi-Square	1	20.0868	<.0001
Mantel-Haenszel Chi-Square	1	21.0403	<.0001
Phi Coefficient		0.2375	
Contingency Coefficient		0.2311	
Cramer's V		0.2375	

Fisher's Exact Test	
Cell (1,1) Frequency (F)	157
Left-sided Pr <= F	1.0000
Right-sided Pr >= F	3.479E-06
Table Probability (P)	2.304E-06
Two-sided Pr <= P	4.772E-06

Sample Size = 374

Fisher's exact test for descriptive comparison of seroconversion rates among other groups

The FREQ Procedure

Table of treat by sero2wk

treat(Group) sero2wk

Frequency<br>Percent<br>Row Pct<br>Col Pct	1	2	Total
Group 4 (M-)	157	43	200
	41.87	11.47	53.33
	78.50	21.50	
	100.00	19.72	
Group 3 (PP)	0	175	175
	0.00	46.67	46.67
	0.00	100.00	
	0.00	80.28	
Total	157	218	375
	41.87	58.13	100.00

Fisher's exact test for descriptive comparison of seroconversion rates among other groups

The FREQ Procedure

Statistics for Table of treat by sero2wk

Statistic	DF	Value	Prob
Chi-Square	1	236.3102	<.0001
Likelihood Ratio Chi-Square	1	301.6909	<.0001
Continuity Adj. Chi-Square	1	233.0959	<.0001
Mantel-Haenszel Chi-Square	1	235.6800	<.0001
Phi Coefficient		0.7938	
Contingency Coefficient		0.6217	
Cramer's V		0.7938	

Fisher's Exact Test	
Cell (1,1) Frequency (F)	157
Left-sided Pr <= F	1.0000
Right-sided Pr >= F	5.059E-66
Table Probability (P)	3.159E-63
Two-sided Pr <= P	5.191E-66

Sample Size = 375

Fisher's exact test for descriptive comparison of seroconversion rates among other groups

The FREQ Procedure

Table of treat by sero2wk

treat(Group) sero2wk

Frequency<br>Percent<br>Row Pct<br>Col Pct	1	2	Total
Group 1 (MM)	157<br>44.86<br>89.20<br>61.57	19<br>5.43<br>10.80<br>20.00	176<br>50.29
Group 2 (MP)	98<br>28.00<br>56.32<br>38.43	76<br>21.71<br>43.68<br>80.00	174<br>49.71
Total	255<br>72.86	95<br>27.14	350<br>100.00

Fisher's exact test for descriptive comparison of seroconversion rates among other groups

The FREQ Procedure

Statistics for Table of treat by sero2wk

Statistic	DF	Value	Prob
Chi-Square	1	47.8411	<.0001
Likelihood Ratio Chi-Square	1	50.3855	<.0001
Continuity Adj. Chi-Square	1	46.1928	<.0001
Mantel-Haenszel Chi-Square	1	47.7044	<.0001
Phi Coefficient		0.3697	
Contingency Coefficient		0.3468	
Cramer's V		0.3697	

Fisher's Exact Test

Cell (1,1) Frequency (F)	157
Left-sided Pr <= F	1.0000
Right-sided Pr >= F	1.654E-12
Table Probability (P)	1.404E-12
Two-sided Pr <= P	2.296E-12

Sample Size = 350

Fisher's exact test for descriptive comparison of seroconversion rates among other groups

The FREQ Procedure

Table of treat by sero2wk

treat(Group) sero2wk

Frequency / Percent / Row Pct / Col Pct	1	2	Total
Group 1 (MM)	157<br>44.73<br>89.20<br>100.00	19<br>5.41<br>10.80<br>9.79	176<br>50.14
Group 3 (PP)	0<br>0.00<br>0.00<br>0.00	175<br>49.86<br>100.00<br>90.21	175<br>49.86
Total	157<br>44.73	194<br>55.27	351<br>100.00

Fisher's exact test for descriptive comparison of seroconversion rates among other groups

The FREQ Procedure

Statistics for Table of treat by sero2wk

Statistic	DF	Value	Prob
Chi-Square	1	282.4427	<.0001
Likelihood Ratio Chi-Square	1	362.2213	<.0001
Continuity Adj. Chi-Square	1	278.8460	<.0001
Mantel-Haenszel Chi-Square	1	281.6381	<.0001
Phi Coefficient		0.8970	
Contingency Coefficient		0.6677	
Cramer's V		0.8970	

Fisher's Exact Test

Cell (1,1) Frequency (F)	157
Left-sided Pr <= F	1.0000
Right-sided Pr >= F	4.985E-79
Table Probability (P)	6.848E-76
Two-sided Pr <= P	5.523E-79

Sample Size = 351

Fisher's exact test for descriptive comparison of seroconversion rates among other groups

The FREQ Procedure

Table of treat by sero2wk

treat(Group) sero2wk

Frequency<br>Percent<br>Row Pct<br>Col Pct	1	2	Total
Group 2 (MP)	98<br>28.08<br>56.32<br>100.00	76<br>21.78<br>43.68<br>30.28	174<br>49.86
Group 3 (PP)	0<br>0.00<br>0.00<br>0.00	175<br>50.14<br>100.00<br>69.72	175<br>50.14
Total	98<br>28.08	251<br>71.92	349<br>100.00

Fisher's exact test for descriptive comparison of seroconversion rates among other groups

The FREQ Procedure

Statistics for Table of treat by sero2wk

Statistic	DF	Value	Prob
Chi-Square	1	137.0461	<.0001
Likelihood Ratio Chi-Square	1	175.9830	<.0001
Continuity Adj. Chi-Square	1	134.2714	<.0001
Mantel-Haenszel Chi-Square	1	136.6534	<.0001
Phi Coefficient		0.6266	
Contingency Coefficient		0.5310	
Cramer's V		0.6266	

Fisher's Exact Test

Cell (1,1) Frequency (F)	98
Left-sided Pr <= F	1.0000
Right-sided Pr >= F	7.831E-39
Table Probability (P)	1.744E-36
Two-sided Pr <= P	7.831E-39

Sample Size = 349

Fisher's exact test for descriptive comparison of seroconversion rates among other groups

The FREQ Procedure

Table of treat by sero2wk

treat(Group) sero2wk

Frequency<br>Percent<br>Row Pct<br>Col Pct	1	2	Total
Group 4 (M-)	152<br>42.11<br>78.76<br>50.50	41<br>11.36<br>21.24<br>68.33	193<br>53.46

Group 1 (MM)	149<br>41.27<br>88.69<br>49.50	19<br>5.26<br>11.31<br>31.67	168<br>46.54
Total	301<br>83.38	60<br>16.62	361<br>100.00

Fisher's exact test for descriptive comparison of seroconversion rates among other groups

The FREQ Procedure

Statistics for Table of treat by sero2wk

Statistic	DF	Value	Prob
Chi-Square	1	6.3959	0.0114
Likelihood Ratio Chi-Square	1	6.5551	0.0105
Continuity Adj. Chi-Square	1	5.6992	0.0170
Mantel-Haenszel Chi-Square	1	6.3782	0.0116
Phi Coefficient		-0.1331	
Contingency Coefficient		0.1319	
Cramer's V		-0.1331	

Fisher's Exact Test	
Cell (1,1) Frequency (F)	152
Left-sided Pr <= F	0.0080
Right-sided Pr >= F	0.9966
Table Probability (P)	0.0045
Two-sided Pr <= P	0.0155

Sample Size = 361

Fisher's exact test for descriptive comparison of seroconversion rates among other groups

The FREQ Procedure

Table of treat by sero2wk

treat(Group) sero2wk

Frequency<br>Percent<br>Row Pct<br>Col Pct	1	2	Total
Group 4 (M-)	152<br>41.87<br>78.76<br>61.54	41<br>11.29<br>21.24<br>35.34	193<br>53.17
Group 2 (MP)	95<br>26.17<br>55.88<br>38.46	75<br>20.66<br>44.12<br>64.66	170<br>46.83
Total	247<br>68.04	116<br>31.96	363<br>100.00

Fisher's exact test for descriptive comparison of seroconversion rates among other groups

The FREQ Procedure

Statistics for Table of treat by sero2wk

Statistic	DF	Value	Prob

Statistic	DF	Value	Prob
Chi-Square	1	21.7494	<.0001
Likelihood Ratio Chi-Square	1	21.9282	<.0001
Continuity Adj. Chi-Square	1	20.7101	<.0001
Mantel-Haenszel Chi-Square	1	21.6895	<.0001
Phi Coefficient		0.2448	
Contingency Coefficient		0.2378	
Cramer's V		0.2448	

Fisher's Exact Test

Cell (1,1) Frequency (F)	152
Left-sided Pr <= F	1.0000
Right-sided Pr >= F	2.470E-06
Table Probability (P)	1.663E-06
Two-sided Pr <= P	3.458E-06

Sample Size = 363

Fisher's exact test for descriptive comparison of seroconversion rates among other groups

The FREQ Procedure

Table of treat by sero2wk

treat(Group) sero2wk

Frequency<br>Percent<br>Row Pct<br>Col Pct	1	2	Total
Group 4 (M-)	152<br>42.58<br>78.76<br>100.00	41<br>11.48<br>21.24<br>20.00	193<br>54.06
Group 3 (PP)	0<br>0.00<br>0.00<br>0.00	164<br>45.94<br>100.00<br>80.00	164<br>45.94
Total	152<br>42.58	205<br>57.42	357<br>100.00

Fisher's exact test for descriptive comparison of seroconversion rates among other groups

The FREQ Procedure

Statistics for Table of treat by sero2wk

Statistic	DF	Value	Prob
Chi-Square	1	224.9285	<.0001
Likelihood Ratio Chi-Square	1	287.3838	<.0001
Continuity Adj. Chi-Square	1	221.7188	<.0001
Mantel-Haenszel Chi-Square	1	224.2984	<.0001
Phi Coefficient		0.7938	
Contingency Coefficient		0.6217	
Cramer's V		0.7938	

Fisher's Exact Test

Cell (1,1) Frequency (F)	152
Left-sided Pr <= F	1.0000

Right-sided Pr >= F	6.467E-63
Table Probability (P)	3.838E-60
Two-sided Pr <= P	7.057E-63

Sample Size = 357

Fisher's exact test for descriptive comparison of seroconversion rates among other groups

The FREQ Procedure

Table of treat by sero2wk

treat(Group)   sero2wk

Frequency / Percent / Row Pct / Col Pct	1	2	Total
Group 1 (MM)	149<br>44.08<br>88.69<br>61.07	19<br>5.62<br>11.31<br>20.21	168<br>49.70
Group 2 (MP)	95<br>28.11<br>55.88<br>38.93	75<br>22.19<br>44.12<br>79.79	170<br>50.30
Total	244<br>72.19	94<br>27.81	338<br>100.00

Fisher's exact test for descriptive comparison of seroconversion rates among other groups

The FREQ Procedure

Statistics for Table of treat by sero2wk

Statistic	DF	Value	Prob
Chi-Square	1	45.3023	<.0001
Likelihood Ratio Chi-Square	1	47.7227	<.0001
Continuity Adj. Chi-Square	1	43.6828	<.0001
Mantel-Haenszel Chi-Square	1	45.1682	<.0001
Phi Coefficient		0.3661	
Contingency Coefficient		0.3438	
Cramer's V		0.3661	

Fisher's Exact Test

Cell (1,1) Frequency (F)	149
Left-sided Pr <= F	1.0000
Right-sided Pr >= F	6.323E-12
Table Probability (P)	5.336E-12
Two-sided Pr <= P	8.800E-12

Sample Size = 338

Fisher's exact test for descriptive comparison of seroconversion rates among other groups

The FREQ Procedure

Table of treat by sero2wk

treat(Group)   sero2wk

Frequency<br>Percent<br>Row Pct<br>Col Pct	1	2	Total
Group 1 (MM)	149<br>44.88<br>88.69<br>100.00	19<br>5.72<br>11.31<br>10.38	168<br>50.60
Group 3 (PP)	0<br>0.00<br>0.00<br>0.00	164<br>49.40<br>100.00<br>89.62	164<br>49.40
Total	149<br>44.88	183<br>55.12	332<br>100.00

Fisher's exact test for descriptive comparison of seroconversion rates among other groups

The FREQ Procedure

Statistics for Table of treat by sero2wk

Statistic	DF	Value	Prob
Chi-Square	1	263.8808	<.0001
Likelihood Ratio Chi-Square	1	338.1745	<.0001
Continuity Adj. Chi-Square	1	260.3078	<.0001
Mantel-Haenszel Chi-Square	1	263.0860	<.0001
Phi Coefficient		0.8915	
Contingency Coefficient		0.6655	
Cramer's V		0.8915	

Fisher's Exact Test

Cell (1,1) Frequency (F)	149
Left-sided Pr <= F	1.0000
Right-sided Pr >= F	8.104E-74
Table Probability (P)	9.901E-71
Two-sided Pr <= P	9.639E-74

Sample Size = 332

Fisher's exact test for descriptive comparison of seroconversion rates among other groups

The FREQ Procedure

Table of treat by sero2wk

treat(Group) sero2wk

Frequency<br>Percent<br>Row Pct<br>Col Pct	1	2	Total
Group 2 (MP)	95<br>28.44<br>55.88<br>100.00	75<br>22.46<br>44.12<br>31.38	170<br>50.90
Group 3 (PP)	0<br>0.00<br>0.00<br>0.00	164<br>49.10<br>100.00<br>68.62	164<br>49.10

Total	95	239	334
	28.44	71.56	100.00

Fisher's exact test for descriptive comparison of seroconversion rates among other groups

The FREQ Procedure

Statistics for Table of treat by sero2wk

Statistic	DF	Value	Prob
Chi-Square	1	128.0758	<.0001
Likelihood Ratio Chi-Square	1	165.5444	<.0001
Continuity Adj. Chi-Square	1	125.3449	<.0001
Mantel-Haenszel Chi-Square	1	127.6923	<.0001
Phi Coefficient		0.6192	
Contingency Coefficient		0.5265	
Cramer's V		0.6192	

Fisher's Exact Test

Cell (1,1) Frequency (F)	95
Left-sided Pr <= F	1.0000
Right-sided Pr >= F	1.436E-36
Table Probability (P)	2.945E-34
Two-sided Pr <= P	1.446E-36

Sample Size = 334

Fisher's exact test for descriptive comparison of seroconversion rates among other groups

The FREQ Procedure

Table of treat by sero4wk

treat(Group) sero4wk

Frequency<br>Percent<br>Row Pct<br>Col Pct	1	2	Total
Group 4 (M-)	139<br>36.87<br>69.85<br>47.60	60<br>15.92<br>30.15<br>70.59	199<br>52.79
Group 1 (MM)	153<br>40.58<br>85.96<br>52.40	25<br>6.63<br>14.04<br>29.41	178<br>47.21
Total	292<br>77.45	85<br>22.55	377<br>100.00

Fisher's exact test for descriptive comparison of seroconversion rates among other groups

The FREQ Procedure

Statistics for Table of treat by sero4wk

Statistic	DF	Value	Prob
Chi-Square	1	13.9565	0.0002
Likelihood Ratio Chi-Square	1	14.3506	0.0002
Continuity Adj. Chi-Square	1	13.0495	0.0003

Statistic	DF	Value	Prob
Mantel-Haenszel Chi-Square	1	13.9195	0.0002
Phi Coefficient		-0.1924	
Contingency Coefficient		0.1889	
Cramer's V		-0.1924	

Fisher's Exact Test

Cell (1,1) Frequency (F)	139
Left-sided Pr <= F	1.278E-04
Right-sided Pr >= F	1.0000
Table Probability (P)	8.222E-05
Two-sided Pr <= P	1.979E-04

Sample Size = 377

Fisher's exact test for descriptive comparison of seroconversion rates among other groups

The FREQ Procedure

Table of treat by sero4wk

treat(Group) sero4wk

Frequency / Percent / Row Pct / Col Pct	1	2	Total
Group 4 (M-)	139<br>37.17<br>69.85<br>62.61	60<br>16.04<br>30.15<br>39.47	199<br>53.21
Group 2 (MP)	83<br>22.19<br>47.43<br>37.39	92<br>24.60<br>52.57<br>60.53	175<br>46.79
Total	222<br>59.36	152<br>40.64	374<br>100.00

Fisher's exact test for descriptive comparison of seroconversion rates among other groups

The FREQ Procedure

Statistics for Table of treat by sero4wk

Statistic	DF	Value	Prob
Chi-Square	1	19.4028	<.0001
Likelihood Ratio Chi-Square	1	19.5262	<.0001
Continuity Adj. Chi-Square	1	18.4845	<.0001
Mantel-Haenszel Chi-Square	1	19.3509	<.0001
Phi Coefficient		0.2278	
Contingency Coefficient		0.2221	
Cramer's V		0.2278	

Fisher's Exact Test

Cell (1,1) Frequency (F)	139
Left-sided Pr <= F	1.0000
Right-sided Pr >= F	8.109E-06
Table Probability (P)	5.087E-06
Two-sided Pr <= P	1.404E-05

Sample Size = 374
Fisher's exact test for descriptive comparison of seroconversion rates among other groups

The FREQ Procedure

Table of treat by sero4wk

treat(Group) sero4wk

Frequency<br>Percent<br>Row Pct<br>Col Pct	1	2	Total
Group 4 (M-)	139<br>36.97<br>69.85<br>100.00	60<br>15.96<br>30.15<br>25.32	199<br>52.93
Group 3 (PP)	0<br>0.00<br>0.00<br>0.00	177<br>47.07<br>100.00<br>74.68	177<br>47.07
Total	139<br>36.97	237<br>63.03	376<br>100.00

Fisher's exact test for descriptive comparison of seroconversion rates among other groups

The FREQ Procedure

Statistics for Table of treat by sero4wk

Statistic	DF	Value	Prob
Chi-Square	1	196.1438	<.0001
Likelihood Ratio Chi-Square	1	251.7766	<.0001
Continuity Adj. Chi-Square	1	193.1576	<.0001
Mantel-Haenszel Chi-Square	1	195.6221	<.0001
Phi Coefficient		0.7223	
Contingency Coefficient		0.5855	
Cramer's V		0.7223	

Fisher's Exact Test	
Cell (1,1) Frequency (F)	139
Left-sided Pr <= F	1.0000
Right-sided Pr >= F	3.070E-55
Table Probability (P)	1.238E-52
Two-sided Pr <= P	3.522E-55

Sample Size = 376
Fisher's exact test for descriptive comparison of seroconversion rates among other groups

The FREQ Procedure

Table of treat by sero4wk

treat(Group) sero4wk

Frequency<br>Percent<br>Row Pct<br>Col Pct	1	2	Total

Group 1 (MM)	153<br>43.34<br>85.96<br>64.83	25<br>7.08<br>14.04<br>21.37	178<br>50.42
Group 2 (MP)	83<br>23.51<br>47.43<br>35.17	92<br>26.06<br>52.57<br>78.63	175<br>49.58
Total	236<br>66.86	117<br>33.14	353<br>100.00

Fisher's exact test for descriptive comparison of seroconversion rates among other groups

The FREQ Procedure

Statistics for Table of treat by sero4wk

Statistic	DF	Value	Prob
Chi-Square	1	59.1090	<.0001
Likelihood Ratio Chi-Square	1	61.8535	<.0001
Continuity Adj. Chi-Square	1	57.3831	<.0001
Mantel-Haenszel Chi-Square	1	58.9416	<.0001
Phi Coefficient		0.4092	
Contingency Coefficient		0.3787	
Cramer's V		0.4092	

Fisher's Exact Test	
Cell (1,1) Frequency (F)	153
Left-sided Pr <= F	1.0000
Right-sided Pr >= F	4.962E-15
Table Probability (P)	4.330E-15
Two-sided Pr <= P	9.296E-15

Sample Size = 353

Fisher's exact test for descriptive comparison of seroconversion rates among other groups

The FREQ Procedure

Table of treat by sero4wk

treat(Group) sero4wk

Frequency<br>Percent<br>Row Pct<br>Col Pct	1	2	Total
Group 1 (MM)	153<br>43.10<br>85.96<br>100.00	25<br>7.04<br>14.04<br>12.38	178<br>50.14
Group 3 (PP)	0<br>0.00<br>0.00<br>0.00	177<br>49.86<br>100.00<br>87.62	177<br>49.86
Total	153<br>43.10	202<br>56.90	355<br>100.00

Fisher's exact test for descriptive comparison of seroconversion rates among other groups

The FREQ Procedure

Statistics for Table of treat by sero4wk

Statistic	DF	Value	Prob
Chi-Square	1	267.3755	<.0001
Likelihood Ratio Chi-Square	1	340.8923	<.0001
Continuity Adj. Chi-Square	1	263.8821	<.0001
Mantel-Haenszel Chi-Square	1	266.6224	<.0001
Phi Coefficient		0.8679	
Contingency Coefficient		0.6554	
Cramer's V		0.8679	

Fisher's Exact Test

Cell (1,1) Frequency (F)	153
Left-sided Pr <= F	1.0000
Right-sided Pr >= F	1.900E-74
Table Probability (P)	1.979E-71
Two-sided Pr <= P	2.167E-74

Sample Size = 355

Fisher's exact test for descriptive comparison of seroconversion rates among other groups

The FREQ Procedure

Table of treat by sero4wk

treat(Group) sero4wk

Frequency<br>Percent<br>Row Pct<br>Col Pct	1	2	Total
Group 2 (MP)	83<br>23.58<br>47.43<br>100.00	92<br>26.14<br>52.57<br>34.20	175<br>49.72
Group 3 (PP)	0<br>0.00<br>0.00<br>0.00	177<br>50.28<br>100.00<br>65.80	177<br>50.28
Total	83<br>23.58	269<br>76.42	352<br>100.00

Fisher's exact test for descriptive comparison of seroconversion rates among other groups

The FREQ Procedure

Statistics for Table of treat by sero4wk

Statistic	DF	Value	Prob
Chi-Square	1	109.8509	<.0001
Likelihood Ratio Chi-Square	1	142.3756	<.0001
Continuity Adj. Chi-Square	1	107.2346	<.0001
Mantel-Haenszel Chi-Square	1	109.5388	<.0001
Phi Coefficient		0.5586	
Contingency Coefficient		0.4877	
Cramer's V		0.5586	

Fisher's Exact Test

Cell (1,1) Frequency (F)	83
Left-sided Pr <= F	1.0000
Right-sided Pr >= F	1.461E-31
Table Probability (P)	2.308E-29
Two-sided Pr <= P	1.461E-31

Sample Size = 352

Fisher's exact test for descriptive comparison of seroconversion rates among other groups

The FREQ Procedure

Table of treat by sero4wk

treat(Group) sero4wk

Frequency / Percent / Row Pct / Col Pct	1	2	Total
Group 4 (M-)	132<br>36.67<br>68.75<br>47.83	60<br>16.67<br>31.25<br>71.43	192<br>53.33
Group 1 (MM)	144<br>40.00<br>85.71<br>52.17	24<br>6.67<br>14.29<br>28.57	168<br>46.67
Total	276<br>76.67	84<br>23.33	360<br>100.00

Fisher's exact test for descriptive comparison of seroconversion rates among other groups

The FREQ Procedure

Statistics for Table of treat by sero4wk

Statistic	DF	Value	Prob
Chi-Square	1	14.4144	0.0001
Likelihood Ratio Chi-Square	1	14.8602	0.0001
Continuity Adj. Chi-Square	1	13.4817	0.0002
Mantel-Haenszel Chi-Square	1	14.3743	0.0001
Phi Coefficient		-0.2001	
Contingency Coefficient		0.1962	
Cramer's V		-0.2001	

Fisher's Exact Test

Cell (1,1) Frequency (F)	132
Left-sided Pr <= F	9.923E-05
Right-sided Pr >= F	1.0000
Table Probability (P)	6.492E-05
Two-sided Pr <= P	1.625E-04

Sample Size = 360

Fisher's exact test for descriptive comparison of seroconversion rates among other groups

The FREQ Procedure

Table of treat by sero4wk

treat(Group) sero4wk

Frequency / Percent / Row Pct / Col Pct	1	2	Total
Group 4 (M-)	132<br>36.46<br>68.75<br>61.97	60<br>16.57<br>31.25<br>40.27	192<br>53.04
Group 2 (MP)	81<br>22.38<br>47.65<br>38.03	89<br>24.59<br>52.35<br>59.73	170<br>46.96
Total	213<br>58.84	149<br>41.16	362<br>100.00

Fisher's exact test for descriptive comparison of seroconversion rates among other groups

The FREQ Procedure

Statistics for Table of treat by sero4wk

Statistic	DF	Value	Prob
Chi-Square	1	16.5798	<.0001
Likelihood Ratio Chi-Square	1	16.6733	<.0001
Continuity Adj. Chi-Square	1	15.7199	<.0001
Mantel-Haenszel Chi-Square	1	16.5340	<.0001
Phi Coefficient		0.2140	
Contingency Coefficient		0.2093	
Cramer's V		0.2140	

Fisher's Exact Test

Cell (1,1) Frequency (F)	132
Left-sided Pr <= F	1.0000
Right-sided Pr >= F	3.525E-05
Table Probability (P)	2.135E-05
Two-sided Pr <= P	4.908E-05

Sample Size = 362

Fisher's exact test for descriptive comparison of seroconversion rates among other groups

The FREQ Procedure

Table of treat by sero4wk

treat(Group) sero4wk

Frequency / Percent / Row Pct / Col Pct	1	2	Total
Group 4 (M-)	132<br>37.08<br>68.75	60<br>16.85<br>31.25	192<br>53.93

	100.00	26.79	
Group 3 (PP)	0	164	164
	0.00	46.07	46.07
	0.00	100.00	
	0.00	73.21	
Total	132	224	356
	37.08	62.92	100.00

Fisher's exact test for descriptive comparison of seroconversion rates among other groups

The FREQ Procedure

Statistics for Table of treat by sero4wk

Statistic	DF	Value	Prob
Chi-Square	1	179.1920	<.0001
Likelihood Ratio Chi-Square	1	230.9764	<.0001
Continuity Adj. Chi-Square	1	176.2573	<.0001
Mantel-Haenszel Chi-Square	1	178.6886	<.0001
Phi Coefficient		0.7095	
Contingency Coefficient		0.5786	
Cramer's V		0.7095	

Fisher's Exact Test

Cell (1,1) Frequency (F)	132
Left-sided Pr <= F	1.0000
Right-sided Pr >= F	9.902E-51
Table Probability (P)	3.514E-48
Two-sided Pr <= P	1.374E-50

Sample Size = 356

Fisher's exact test for descriptive comparison of seroconversion rates among other groups

The FREQ Procedure

Table of treat by sero4wk

treat(Group) sero4wk

Frequency<br>Percent<br>Row Pct<br>Col Pct	1	2	Total
Group 1 (MM)	144	24	168
	42.60	7.10	49.70
	85.71	14.29	
	64.00	21.24	
Group 2 (MP)	81	89	170
	23.96	26.33	50.30
	47.65	52.35	
	36.00	78.76	
Total	225	113	338
	66.57	33.43	100.00

Fisher's exact test for descriptive comparison of seroconversion rates among other groups

The FREQ Procedure

Statistics for Table of treat by sero4wk

Statistic	DF	Value	Prob
Chi-Square	1	55.0195	<.0001
Likelihood Ratio Chi-Square	1	57.6517	<.0001
Continuity Adj. Chi-Square	1	53.3223	<.0001
Mantel-Haenszel Chi-Square	1	54.8567	<.0001
Phi Coefficient		0.4035	
Contingency Coefficient		0.3742	
Cramer's V		0.4035	

Fisher's Exact Test

Cell (1,1) Frequency (F)	144
Left-sided Pr <= F	1.0000
Right-sided Pr >= F	4.142E-14
Table Probability (P)	3.531E-14
Two-sided Pr <= P	5.401E-14

Sample Size = 338

Fisher's exact test for descriptive comparison of seroconversion rates among other groups

The FREQ Procedure

Table of treat by sero4wk

treat(Group) sero4wk

Frequency / Percent / Row Pct / Col Pct	1	2	Total
Group 1 (MM)	144	24	168
	43.37	7.23	50.60
	85.71	14.29	
	100.00	12.77	
Group 3 (PP)	0	164	164
	0.00	49.40	49.40
	0.00	100.00	
	0.00	87.23	
Total	144	188	332
	43.37	56.63	100.00

Fisher's exact test for descriptive comparison of seroconversion rates among other groups

The FREQ Procedure

Statistics for Table of treat by sero4wk

Statistic	DF	Value	Prob
Chi-Square	1	248.2432	<.0001
Likelihood Ratio Chi-Square	1	316.6021	<.0001
Continuity Adj. Chi-Square	1	244.7656	<.0001
Mantel-Haenszel Chi-Square	1	247.4954	<.0001
Phi Coefficient		0.8647	
Contingency Coefficient		0.6541	
Cramer's V		0.8647	

Fisher's Exact Test

Cell (1,1) Frequency (F)	144
Left-sided Pr <= F	1.0000
Right-sided Pr >= F	3.536E-69
Table Probability (P)	3.341E-66
Two-sided Pr <= P	4.890E-69

Sample Size = 332

Fisher's exact test for descriptive comparison of seroconversion rates among other groups

The FREQ Procedure

Table of treat by sero4wk

treat(Group) / Frequency, Percent, Row Pct, Col Pct	sero4wk 1	sero4wk 2	Total
Group 2 (MP)	81<br>24.25<br>47.65<br>100.00	89<br>26.65<br>52.35<br>35.18	170<br>50.90
Group 3 (PP)	0<br>0.00<br>0.00<br>0.00	164<br>49.10<br>100.00<br>64.82	164<br>49.10
Total	81<br>24.25	253<br>75.75	334<br>100.00

Fisher's exact test for descriptive comparison of seroconversion rates among other groups

The FREQ Procedure

Statistics for Table of treat by sero4wk

Statistic	DF	Value	Prob
Chi-Square	1	103.1587	<.0001
Likelihood Ratio Chi-Square	1	134.7529	<.0001
Continuity Adj. Chi-Square	1	100.5813	<.0001
Mantel-Haenszel Chi-Square	1	102.8498	<.0001
Phi Coefficient		0.5558	
Contingency Coefficient		0.4858	
Cramer's V		0.5558	

Fisher's Exact Test

Cell (1,1) Frequency (F)	81
Left-sided Pr <= F	1.0000
Right-sided Pr >= F	6.589E-30
Table Probability (P)	9.726E-28
Two-sided Pr <= P	6.714E-30

Sample Size = 334

Statistical Analysis Plan
Study: 1029/BN

Title: A PARTIALLY RANDOMIZED, PARTIALLY DOUBLE-BLIND, PLACEBO-CONTROLLED PHASE II NON-INFERIORITY STUDY TO EVALUATE IMMUNOGENICITY AND SAFETY OF ONE AND TWO DOSES OF MVA-BN® (IMVAMUNE™) SMALLPOX VACCINE IN 18-55 YEAR OLD HEALTHY SUBJECTS

Protocol-No.: POX-MVA-005 (HCR CODE 1029/BN)

Sponsor: BAVARIAN NORDIC A/S

Prepared by: JOACHIM KLINGER, DIPL.-STAT.

HARRISON CLINICAL RESEARCH GMBH

Date: 8 MAY 2007

Version 2.0

Table of Contents

1 PURPOSE 2

2 METHODS 2

3 PRESENTATION OF DATA 2

3.1 General 2
3.2 Layout of tables, listings and figures 3
3.2.1 Quantitative variables: 3
3.2.2 Qualitative variables. 4
3.2.3 Raw data listings 5

4 STATISTICAL ANALYSIS 6

4.1 Primary objective 6
4.2 Sample size calculation 6
4.3 Variables 7
4.3.1 Immunogenicity variables 7
4.3.2 Safety and reactogenicity variables 7
4.4 Study cohorts/data sets to be evaluated 7
4.5 Statistical analysis 8
4.5.1 Timing of statistical analysis 8
4.5.2 Analysis of demographics and baseline characteristics 8
4.5.3 Analysis of Immunogenicity 8
4.5.4 Analysis of safety and reactogenicity 9
4.5.5 Data handling 11
4.6 Appendix: Index of section 14 of the clinical report 14
4.7 Appendix: Index of section 16.2 of the clinical report 18

1 Purpose

This statistical analysis plan should specify the planned statistical analyses in the protocol in more depth. Furthermore, special considerations or deviations (that are not necessary to be included in a protocol amendment) should be documented.

2 Methods

Raw data listings, summary tables, graphs and statistical tests will be generated by means of the program SAS Vers. 9.1. Special non-parametric tests will done with Proc-StatXact 7® from ©Cytel Software, which is completely linked to SAS.

Data from the SAP-DB database (MARVIN e-CRF) will be transferred to HCR over a secured SSL-connection from the host server to the HCR file server. The data will be transferred in CDISC ODM XML Vers. 1.2 compliant format (see http://www.cdisc.org/models/odm/v1.2.1/index.html for details).

This ODM XML file is then imported into SAS via the SAS procedure proc CDISC.

Appropriate SAS programs will be prepared and validated according to HCR SOPs.

3 Presentation of data

3.1 General

All raw data will be listed in section 16.2 of the clinical report. Additional text entries in the CRF, which did not clearly belong to pre-defined CRF and database fields, will be disregarded. Summary tables for all parameters of interest are presented in section 14 of the clinical report.

Non-mandatory empty data fields in the CRF are generally treated as missing values. Mandatory CRF fields which remained empty at the time of e-CRF data entry are always automatically queried and resolved.

3.2 Layout of tables, listings and figures

If not otherwise specified in the tables, the following format will be used for all tables:

3.2.1 Quantitative variables:

x-axis: Treatment group: Group 1: (MM), Group 2: (MP), Group 3: (PP), Group 4: (M-)
y-axis: Variable
Cells: Mean, Median, StdDev (Standard deviation), SEM (Standard error of mean), Min, Max, N (Valid cases)

Example:

Subject characteristics - Safety Data Set

		Number of Patients (%) / Descriptive Statistics					
		Group 1 (MM) (N=153)	Group 2 (MP) (N=221)	Group 3 (PP) (N=171)	Group 4 (M-) (N=200)	Total (N=745)	p-value
Age [years]							
	Mean	25.8	25.6	25.3	41.5	29.8	<0.001#
	Median	25.0	24.0	25.0	42.0	27.0	
	Std	4.68	5.01	4.75	7.59	9.07	
	SEM	0.38	0.34	0.36	0.54	0.33	
	Min	18.0	18.0	18.0	22.0	18.0	
	Max	44.0	50.0	50.0	55.0	55.0	
	N	153	221	171	200	745	
Height [cm]							
	Mean	174.4	173.0	173.6	172.2	173.2	0.195#
	Median	173.0	173.0	173.0	171.0	172.0	
	Std	10.74	8.90	9.35	9.36	9.54	
	SEM	0.87	0.60	0.72	0.66	0.35	
	Min	152.0	151.0	150.0	153.0	150.0	
	Max	204.0	196.0	200.0	195.0	204.0	
	N	153	221	171	200	745	
Weight at screening visit							

~ Chi-square test
^ Fisher's Exact test
# F-test

3.2.2 Qualitative variables.

x-axis:	Treatment group: Group 1: (MM), Group 2: (MP), Group 3: (PP), Group 4: (M-)
y-axis:	Categories/classes of a specific variable
Cells:	Absolute and relative count within each treatment and class
Percentage basis:	Depending on the variable, percentages are based on number of events if not otherwise specified. For special tables (e.g. AEs) percentages are based on no. of subjects within a treatment group

Example:

Solicited Local Adverse Events (per Vaccination period):
Summary of Maximum Intensity (7-Day Follow-up Period after Vaccination), Safety Data Set

Dose=1st vaccination

Preferred term	Group1 (MM) (N=33)		Group 2 (MP) (N=73)	
	n	%	n	%
Injection site induration				
Missing	-	0.0	-	0.0
Grade 0	31	68.9	24	32.9
Grade 1	11	24.4	37	50.7
Grade 2	3	6.7	12	16.4
Grade 3	-	0.0	-	0.0
Grade 4	-	0.0	-	0.0
Total	45	100.0	73	100.0

-→ further groups to the right

N: Number of subjects in the specified group with patient diaries recording local symptoms
n: Number of events
%: Percentage based on total number of events

3.2.3 Raw data listings

All CRF data will be listed in raw data listings (section 16.2 of clinical report).
If not otherwise specified, all data listings will be sorted by treatment, vaccination and visit/time (if applicable/ necessary) and subject no.

Listing xy Solicited General Adverse Events: Body Temperature (oral) >= Grade 2 - Safety Data Set

Group=Group 1 Dose=1st vaccination

	Body temperature [°C]								Intensity									Relation		Last day of sympto after day 7		Max. intens. after day7		Day of	Durati	
Subj	Day0	Day1	Day2	Day3	Day4	Day5	Day6	Day7	Day0	Day1	Day2	Day3	Day4	Day5	Day6	Day7	Out- come	ship to vaccine	SAE	Date	Relative to vacc.	[°C]	Intens.	onset (rel. to vacc.)	on [days] *	Action taken
080	36.5	36.3	36.9	36.7	36.3	36.5	36.5	38.5	0	0	0	0	0	0	0	2	1	3	no	23JUN2006	10	38.5	2	7	1	3

Intensity: 0= <37.5°C
1= >=37.5 - <38.0 °C
2= >=38.0 - <39.0 °C
3= >=39.0 - <40.0 °C
4= >=40.0 °C

Outcome: 1=recovery without sequelae
2=recovery with sequelae
3=ongoing
4=death

Action: 0=no action
1=study drug dosage adjusted
2=study drug discontinued
3=concomitant medication
4=non-drug therapy
5=hospitalization

Relationship: 1=none
2=unlikely
3=possible
4=probable
5=definite

*: Number of days symptom present within one week after vaccination (incl. day of vaccination)

4 Statistical analysis

The statistical plan of the study protocol will be followed completely.

In the following section, the statistical analysis from the study protocol and its amendments will be detailed. If necessary, modifications or specifications will be done and documented.

4.1 Primary objective

The primary objective of this trial is to compare the ELISA specific humoral immune response of the group with history of smallpox vaccination versus the group without history of smallpox vaccination, i.e. to prove that a single dose schedule would be sufficient to boost the immunity in a previously vaccinated population.

4.2 Sample size calculation

The sample size calculation is based on the primary immunogenicity variable 'MVA -specific seroconversion rate'. This parameter is derived from the ELISA specific antibody titers. Seroconversion is defined as the appearance of antibody titers ≥ 1:50 in a vaccinia specific IgG ELISA for initially seronegative subjects or twofold increase of the antibody titer compared to the pre-existing baseline titer for subjects with a pre-existing antibody titer in the ELISA.

The primary hypothesis is to show that the humoral immune response of the group with history of smallpox vaccination (Group 4) is not statistically inferior compared to the group without history of smallpox vaccination (Group 1). The study should demonstrate that the Group 4 seroconversion rate is not worse than the Group 1 seroconversion rate by more than a pre-specified amount. This amount is called the non-inferiority margin (D).

Suppose p_1 is the seroconversion rate in the group without history of smallpox vaccination and p_4 is the seroconversion rate in the subjects with history of smallpox vaccination.
The test on non-inferiority will be applied for the following hypothesis:

H_0: $p_4 - p_1 \leq -\Delta$ versus H_1: $p_4 - p_1 > -\Delta$, where

Δ is the non-inferiority margin and is chosen in this trial as 5%.
From the experience in a pilot trial with MVA-BN® in healthy subjects it is anticipated that the seroconversion rate in healthy vaccinia-naive subjects reaches 98-100%.

Assuming a significance level of 5%, a power of 80%, expected seroconversion rates of 98% in both groups, this yields to a sample size of 175 per group (700 in total). The number of subjects to be enrolled was **740** (200 subjects in group 4 and 180 each in groups 1, 2 and 3). It is expected that 175 subjects per group are evaluable.

For reference see Agresti A, Min Y. (2001) and Chan I (1998). This sample size estimation was performed using StatXact®, Vers. 7 using PROC SXPOWERBIN with an internal significance level of 2.5% to consider the one-sided testing situation.

4.3 Variables

4.3.1 Immunogenicity variables

Primary variable	MVA -specific seroconversion rate derived from the ELISA specific antibody titers 2 weeks after the last vaccination (Group 1-3: Visit 4, Group 4: Visit 2).<br>Seroconversion is defined as the appearance of antibody titers ≥ 1:50 in a vaccinia specific IgG ELISA for initially seronegative subjects or twofold increase of the antibody titer compared to the pre-existing baseline titer for subjects with a pre-existing antibody titer in the ELISA.
Secondary variables:	MVA -specific seroconversion rate derived from the ELISA specific antibody titers 4 weeks after the last vaccination (Group 1-3: Visit 5, Group 4: Visit 3).<br><br>MVA -specific seroconversion rate derived from the PRNT specific antibody titers 2 weeks after the last vaccination (Group 1-3: Visit 4, Group 4: Visit 2) and 4 weeks after the last vaccination (Group 1-3: Visit 5, Group 4: Visit 3).<br>Seroconversion is defined as appearance of antibody titers ≥ 1:20 in a vaccinia specific plaque reduction neutralization assay for initially seronegative subjects or twofold increase of the antibody titer compared to the pre-existing baseline titer for subjects with a pre-existing antibody titer in the PRNT.

4.3.2 Safety and reactogenicity variables

Primary variables:	Occurrence, relationship and intensity of any specific or unspecific ECG change at any time during the study.<br><br>Occurrence, relationship and intensity of any other cardiac symptom at any time during the study.
Secondary variables:	Occurrence of any serious adverse event possibly, probably or definitely related to the study vaccine at any time during the study.<br><br>Occurrence of any grade 3 or higher adverse reaction possibly, probably or definitely related to the study vaccine within 28 days after vaccination.<br><br>Occurrence of solicited local adverse events within 1 week (Days 0-7) after vaccination: Intensity, duration, and relationship to vaccination.<br><br>Occurrence of solicited general adverse events within 1 week (Days 0-7) after vaccination: Intensity, duration, and relationship to vaccination.<br><br>Occurrence of unsolicited non-serious adverse events within 28 days after vaccination: Intensity, duration and relationship to vaccination.

4.4 Study cohorts/data sets to be evaluated

For the statistical analysis the included subjects will be divided up into the following datasets:

Safety population: This is the subset of subjects who are randomized, have received at least one dose of study vaccine and for whom safety data are available.

The main analysis of safety will be performed on this population.

Full-analysis (FA): This is the subset of subjects who are randomized and had received at least one dose of study vaccine. Relevant protocol violators are included into this dataset. Patients who drop out before Visit 5 (for Group 1-3) or Visit 3 (for Group 4) are included here.

Per Protocol (PP): This is the subset of subjects who are randomized and had received two doses of study vaccine (group 1-3), respectively one dose of study vaccine (group 4). Subjects have to complete Visit 5 (for Group 1-3), respectively Visit 3 (for Group 4). Subjects have to adhere to all protocol conditions, however mild (not relevant) protocol violators can be included into this dataset.

The decision whether a protocol deviation is relevant or not for the classification of subjects to subsets should be made case-by-case in a blind review meeting.

The primary population dataset will be the FA Set. All confirmatory testing is based on this subgroup. For further descriptive purposes, the same statistical procedures will be applied to the PP dataset.

Analysis of immunogenicity variables will be done on a valid case basis, i.e. for missing observations no imputation technique like LOCF will be applied, because this would imply a too optimistic approach.

4.5 Statistical analysis

4.5.1 Timing of statistical analysis

As soon as the last subject has completed Visit 5 (for Group 1-3), respectively Visit 3 (for Group 4) and after any necessary settlement of queries etc. in the CRFs, data from those patients and visits will be locked. A full analysis of the data available will then be performed.
Once all subjects have completed the follow-up (Visit F-U), an addendum to the clinical report will be prepared including all follow-up data.

4.5.2 Analysis of demographics and baseline characteristics

An analysis of variance (ANOVA) for the main baseline characteristics (age, weight, height) will be performed. The ANOVA will be performed to detect any global treatment effect. The model does not include a term for location by treatment interaction (according to ICH guideline E9, Statistical Principles for Clinical Trials, p.16).

4.5.3 Analysis of Immunogenicity

Antibody titers and resulting seroconversion rates will be assessed by direct ELISA and PRNT method as described in section 7.2 of the study protocol.
The primary immunogenicity variable is the MVA -specific seroconversion rate. This parameter is derived from the ELISA specific antibody titers.

The primary hypothesis is to show that the humoral immune response of the group with history of smallpox vaccination (Group 4) is not statistically inferior compared to the group without history of smallpox vaccination (Group 1).

The above hypothesis will be tested based on an exact, unconditional test for binomial differences. In addition an exact one-sided 97.5% unconditional confidence interval for the difference of proportions will be calculated. Both involve the use of a special score test, which is described in detail in the given references. If the lower limit of this confidence interval is greater than ̃ 5% (or equivalent the p-value of the non-inferiority test is less than 5%), then the null hypothesis will be rejected (StatXact®, Chan (1998), Agresti and Min (2001)).

In order to limit the overall type-I-error to a nominal level of 5%, a hierarchical test procedure will be chosen:

In a first step, the primary null hypothesis as stated in section 4.2 will be tested on the data points collected 2 weeks after the last vaccination (Group 1-3: Visit 4, Group 4: Visit 2). Only if this comparison shows a significant result, the comparison of data at 4 weeks after vaccination (Group 1-3: Visit 5, Group 4: Visit 3) will be done.

A secondary analysis will be done with the neutralization assay (Plaque Reduction Neutralization Test, PRNT) specific seroconversion rates similar to the procedure explained above.

In addition to the main comparison of Group 4 versus Group 1, secondary comparisons will be made among all other groups.

All statistical tests for secondary time points and comparisons are regarded descriptive. No adjustment for multiple testing will therefore be done.

Tables and graphs showing the seroconversion rates for each group at each visit will be prepared.
Descriptive statistics and graphical displays will be given for the geometric mean titer for all groups at all visits.

4.5.4 Analysis of safety and reactogenicity

Solicited local adverse events

The occurrence of solicited local adverse events within 1 week after each vaccination will be summarized on a per patient and per vaccination basis.
The maximum intensity over the 7-day period after vaccination will be used and categorized as follows:

Pain:
- Grade = 0 / > 0
- Grade < 2 / ≥ 2
- Grade < 3 / = 3

For measurements of diameter size:
- Diameter = 0 / > 0
- Diameter < 30mm / ≥ 30 mm
- Diameter < 100mm/ ≥ 100 mm

These categories will be compared between treatment groups by means of the exact test of Fisher (or its normal approximation, the chi-squared test).

Solicited general adverse events

Occurrence of solicited general adverse events within 1 week after each vaccination will be summarized per patient and vaccination.

The maximum intensity over the 7-day period after vaccination will be used and categorized as follows:
- Grade = 0 / > 0

Grade < 2 / ≥ 2
Grade < 3 / ≥ 3

These categories will be compared between treatment groups by means of the exact test of Fisher (or its normal approximation, the chi-squared test).

Unsolicited adverse events:

Unsolicited adverse events will be coded with the MedDRA coding terminology. The intensity of adverse events will be graded according to section 7.1.7. of the study protocol.

The number of adverse events and number of patients with at least one adverse event for each preferred term will be descriptively compared between treatment groups.

The occurrence of any grade 3 or higher adverse reaction possibly, probably or definitely related to the study vaccine within 28 days after vaccination will be compared between treatment groups.

Adverse events of special interest will be separately listed and tabulated. The incidence of such AEs will be compared between treatment groups by means of the exact test of Fisher (or its normal approximation, the chi-squared test).

Serious adverse events will be listed separately. Each SAE will be described individually in detail. The number of subjects with at least one SAE will be compared between treatment groups by means of the exact test of Fisher (or its normal approximation, the chi-squared test).

The occurrence, relationship and intensity of any other cardiac symptom at any time during the study will be listed. The number of subjects with at least one cardiac symptom will be compared between treatment groups by means of the exact test of Fisher (or its normal approximation, the chi-squared test).

Safety laboratory and urine analysis:
Clinical laboratory test results will be marked whether the result is below, within or above the respective reference range. The number of values outside of the reference range will be counted.

ECG
The ECG will be evaluated by a centralized procedure. The transmitted standard ECG results like PQ, QRS, QT and QTc duration and heart rate will be summarized per visit and treatment group.

a) Overall assessment:
    a. The number of subjects with normal/abnormal ECGs and in case of abnormal results clinically significant ECGs (resulting in AEs) will be compared between treatment groups by means of the exact test of Fisher (or its normal approximation, the chi-squared test).
    b. The number of subjects with Myo-Pericarditis will be compared between treatment groups by means of the exact test of Fisher (or its normal approximation, the chi-squared test).
    c. For all clinically significant ECGs, individual data listings showing the type of ECG abnormality will be prepared.

b) Analysis of QTc

According to FDA concept paper for *'Clinical Evaluation Of Qt/Qtc Interval Prolongation And Proarrhythmic Potential For Non-Antiarrhythmic'* and the EMEA *'Guidance for Industry E14 Clinical Evaluation of QT/QTc Interval Prolongation and Proarrhythmic Potential for Non-Antiarrhythmic Drugs'* a detailed QTc analysis will be performed:

    a. Correction of QT interval data:

QT interval data will be corrected for heart rate based upon Fridericia's formula:

Fridericia's correction: $QTc = QT/RR^{0.33}$, where RR is the resting rate.

The QTc-values reported from the conventional ECG are based on Bazett's correction ($QTc = QT/RR^{0.5}$)
In general, however, Bazett's correction overcorrects at elevated heart rates and under corrects at heart rates below 60 bpm and hence is not an ideal correction. Fridericia's correction is more accurate than Bazett's correction in subjects with such altered heart rates.
Therefore, Fridericia's correction only will be used for summary tables.

b. Analyses of Central Tendency

Absolute and baseline-adjusted QTc-values tabulated with descriptive statistics per treatment group and per visit. In addition, the maximum absolute and baseline-adjusted change will be tabulated per treatment group.

c. Categorical Analyses

Categorical analyses of QTc interval data are based on the number and percentage of subjects meeting or exceeding some predefined upper limit value.
The limits are defined as follows:

→ Absolute QTc interval prolongation:

- QTc interval > 450
- QTc interval > 480
- QTc interval > 500

→ Change from baseline in QTc interval:

- QTc interval increases from baseline ≥30
- QTc interval increases from baseline ≥60

Frequency tables showing the absolute and relative frequency for each category per treatment group and visit are prepared. In addition, the maximal QTc prolongation, respectively the maximum change from baseline over time will be used for classification into the categories.

d. Further ECG data
PR-, QT- QRS, reported QTc (Bazett) duration and heart rate will be listed and tabulated per treatment group and visit.

4.5.5 Data handling

All data obtained in this study and documented in the CRF's will be listed. For parameters of interest, summary tables with descriptive group statistics (mean, standard deviation, minimum, maximum, number of valid cases) for metrical variables will be prepared. For ordinal/dichotomous variables summary tables showing the absolute and relative count in each category will be prepared.
Further statistical analysis may be performed as appropriate.

5 References

1) Agresti A, Min Y. (2001). On small-sample confidence intervals for parameters in discretedistributions. Biometrics. 2001 Sep;57(3):963-71

2) Chan I (1998). Exact tests of equivalence and efficacy with a non-zero lower bound for comparative studies. Statistics in Medicine 17, 1403-1413.
3) Proc StatXact User Manual, Statistical Software For Exact Nonparametric Inference, Cytel Statistical Software, 2005
4) Guidance for Industry E14 Clinical Evaluation of QT/QTc Interval Prolongation and Proarrhythmic Potential for Non-Antiarrhythmic Drugs, October 2005
5) FDA Concept Paper, 'Clinical Evaluation Of Qt/Qtc Interval Prolongation And Proarrhythmic Potential For Non-Antiarrhythmic Drugs, http://www.fda.gov/ohrms/dockets/ac/03/briefing/pubs%5Cprelim.pdf, 2002

Signature page

Statistical Analysis Plan approved:

Signatures:

11 May 07<br>Date	J. Klinger, Statistician – HCR
10-Aug-2007<br>Date	N. Uebler, Project Coordinator – Sponsor
10 - May - 2007<br>Date	A. v. Krempelhuber,<br>Manager Clinical Development – Sponsor
10-May-2007<br>Date	G. Virgin,<br>Medical Monitor – Sponsor

5.1 Appendix: Index of section 14 of the clinical report

The following proposal for section 14 and 16.2 is completely done according to the pre-defined ICH-format. Minor changes from this planned index do not need to amended in the SAP.

Comments in Italics will not be printed in table headers or footers.
Formal organization of tabulations may be changed during programming if appropriate, e.g., tables for the different variables may be combined into a single table, or tables with more than one variable may be split into several tables.

SECTION NUMBER	TITLE	TYPE
**14.**	**TABLES, FIGURES AND GRAPHS REFERRED TO BUT NOT INCLUDED IN THE TEXT**	
**14.1**	**DEMOGRAPHIC AND BASELINE DATA**	
14.1.1	Exception to inclusion/ exclusion criteria, safety data set	Count
14.1.2	Subject characteristics, Safety population	Descr. stats
14.1.3	Overall Assessment (until Visit 3/Visit 5), Reason for Premature Discontinuation - All Subjects	Count
14.1.4	Baseline signs and symptoms	
14.1.4.1	Baseline signs and symptoms - outcome	Count
14.1.4.2	Baseline signs and symptoms - intensity	Count
14.1.4.3	Baseline signs and symptoms - action taken	Count
14.1.4.4	Baseline signs and symptoms - SAE or Special Interest AE (SIAE)	Count
14.1.5	History of smallpox vaccination by presence of vaccination scar	Count
14.1.6	Medical history - number of normal/ abnormal findings per category	Count
**14.2**	**IMMUNOGENICITY DATA**	**all tables of this section for FA and PP**
**14.2.1.**	**Antibody responses against MVA-BN® using a direct ELISA**	
14.2.1.1	Antibodies against MVA-BN, descriptive statistics for log-transformed raw data	Descr. stats
14.2.1.2	Antibodies against MVA-BN, serconversion rates, responders	Descr. stats
14.2.1.3	Antibodies against MVA-BN, figure of log-transformed data	Figure
**14.2.2.**	**Antibody responses against MVA-BN® using a plaque reduction neutralization test (PRNT)**	
14.2.2.1	Antibodies against MVA-BN, descriptive statistics for log-transformed raw data	Descr. stats
14.2.2.2	Antibodies against MVA-BN, serconversion rates, responders	Descr. stats
14.2.2.3	Antibodies against MVA-BN, figure of log-transformed data	Figure

SECTION NUMBER	TITLE	TYPE
**14.4**	**LABORATORY DATA**	**all tables of this section for safety population**
14.4.1	Pregnancy test	Count
14.4.2	Laboratory abnormalities - Clinical Chemistry	Count, Descr. stats
14.4.3	Laboratory abnormalities - Haematology	Count, Descr. stats
14.4.4	Laboratory abnormalities - Urinalysis (pH, specific gravity)	Count
14.4.5	Laboratory abnormalities - Urinalysis (qualitative result parameters)	
14.4.6.	Laboratory abnormalities, shift tables	Count
**14.4**	**ECG DATA**	**all tables of this section for safety population**
14.4.1	Overall assessment of ECG	Count
14.4.1.1	Count and percentage of subjects with normal/ abnormal and clinically significant ECG findings	Count
14.4.1.2	Count and percentage of subjects with Myo-/Pericarditis	Count
14.4.1.3	Listing of abnormal ECG findings	Listing
14.4.2	QTc analysis: categorical analysis	Count
14.4.2.1	Absolute QTc values - threshold <=/> 450ms (Fridericia´s correction)	Count
14.4.2.2	Absolute QTc values - threshold <=/> 480ms (Fridericia´s correction)	Count
14.4.2.3	Absolute QTc values - threshold <=/> 500ms (Fridericia´s correction)<br>Change from baseline - threshold </>= 30ms (Fridericia´s correction)<br>Change from baseline - threshold </>= 60ms (Fridericia´s correction)	Count
14.4.2.4	Maximum change from baseline	Count
14.4.3	QTc analysis: analysis of central tendency	
14.4.3.1	Absolute QTc values (Fridericia´s correction)	Descr. stats
14.4.3.2	Maximum post-dose values (Fridericia´s correction)	Descr. stats
14.4.3.3	Change from baseline (Fridericia´s correction)	Descr. stats
14.4.3.4	Mean maximal change from baseline (Fridericia´s correction)	Descr. stats
14.4.4	Further ECG data	
14.4.4.1	PR-duration	Descr. stats
14.4.4.2	QRS-duration	Descr. stats
14.4.4.3	QT-duration	Descr. stats
14.4.4.4	QTc-duration (Bazett correction)	Descr. stats
14.4.4.5	RR Interval	Descr. stats
**14.5**	**ADVERSE EVENT DATA**	**all tables of this section for safety population**
**14.5.1**	**Unsolicited AEs**	

SECTION NUMBER	TITLE	TYPE
14.5.1.1	Listings of deaths, other serious and significant adverse events	Listing
14.5.1.2	Narratives of deaths, other serious and certain other significant adverse events	Listing
14.5.1.3	Unsolicited adverse events: Overview of unsolicited adverse events	Count
14.5.1.4	Unsolicited adverse events: MedDRA coding by SOC, PT and subject	Count
14.5.1.5	Unsolicited adverse events: MedDRA coding by SOC and subject	Count
14.5.1.6	Unsolicited adverse events: MedDRA coding by PT and subject	Count
14.5.1.7	Unsolicited adverse events: MedDRA coding by SOC, PT, intensity and relationship to study medication and subject	Count
14.5.1.8	Unsolicited serious adverse events: MedDRA coding by SOC, PT and subject	Count
14.5.1.9	Unsolicited special interest adverse events: MedDRA coding by SOC, PT and subject	Count
**14.5.2**	**Solicited General Adverse Events**	
14.5.2.1	Summary of Maximum Intensity (7-Day Follow-up Period after Vaccination) by vaccination period	Count
14.5.2.2	Summary of Maximum Intensity (7-Day Follow-up Period after Vaccination) by subject	Count
14.5.2.3	Summary of Maximum Intensity by subject: categories grade=0/ >0	Count
14.5.2.4	Summary of Maximum Intensity by subject: categories grade<2/ >=2	Count
14.5.2.5	Summary of Maximum Intensity by subject: categories grade<3/ >=3	Count
14.5.2.6	Solicited general adverse reactions >= grade 3 by symptom and subject	Count
**14.5.2**	**Solicited Local Adverse Events**	
14.5.2.1	Summary of Maximum Intensity (7-Day Follow-up Period after Vaccination) by vaccination period	Count
14.5.2.2	Summary of Maximum Intensity (7-Day Follow-up Period after Vaccination) by subject	Count
14.5.2.3	Summary of Maximum Intensity by subject: categories grade=0/ >0 (resp. Diameter =0mm/>0mm)	Count
14.5.2.4	Summary of Maximum Intensity by subject: categories grade<2/ >=2 (resp. Diameter <30mm/>=30mm)	Count
14.5.2.5	Summary of Maximum Intensity by subject: categories grade<3/ >=3 (resp. Diameter <100mm/>=100mm)	Count
14.5.2.6	Solicited general adverse reactions >= grade 3 by symptom and subject	Count
**14.5.3**	**Possibly/probably/definitely Related Adverse Events**	
14.5.3.1	Solicited (general / local) and unsolicited adverse events: MedDRA coding by SOC, PT, possible/probable/definite relationship to study medication	Count
**14.6**	**FURTHER SAFETY DATA**	**all tables of this section for safety population**

SECTION NUMBER	TITLE	TYPE
14.6.1	Vital signs	
14.6.1.1	Vital signs – systolic blood pressure	Descr. stats
14.6.1.2	Vital signs – diastolic blood pressure	Descr. stats
14.6.1.3	Vital signs – pulse rate	Descr. stats
14.6.1.4	Vital signs – oral body temperature	Descr. stats
14.6.2	Total study duration	Descr. stats
16.6.3.	Prior and concomitant medication by brand name	Count

5.2 Appendix: Index of section 16.2 of the clinical report

Section number	Title
**16.2**	**SUBJECT DATA LISTINGS**
**16.2.1.**	**DISCONTINUED SUBJECTS**
16.2.1.1.	Study closure form
**16.2.2.**	**PROTOCOL DEVIATIONS**
**16.2.3.**	**SUBJECTS EXCLUDED FROM THE EFFICACY ANALYSIS**
**16.2.4.**	**DEMOGRAPHIC AND BASELINE DATA**
16.2.4.1.	Subject characteristics
16.2.4.2.	History of smallpox vaccination
16.2.4.3.	Medical history
16.2.4.3.	Baseline Signs and Symptoms
**16.2.5.**	**COMPLIANCE AND DRUG CONCENTRATION DATA**
16.2.5.1.	Fulfilment of inclusion/exclusion criteria
16.2.5.2.	Exception to inclusion/exclusion criteria
16.2.5.3.	Confirmation of Eligibility at end of screening
16.2.5.4.	Re-check of eligibility criteria at Visit 1/3
16.2.5.5.	Visit dates
16.2.5.6.	Time between visits
16.2.5.7.	Total study duration
16.2.5.8.	Assignment of vial numbers and vaccination
**16.2.6.**	**IMMUNOGENICITY DATA**
16.2.6.1.	Antibody responses against MVA-BN® using a direct ELISA
16.2.6.2.	Antibody responses against MVA-BN® using a plaque reduction neutralization test (PRNT)
**16.2.7.**	**ADVERSE EVENT LISTING**
16.2.7.1.	Soliceted adverse events
16.2.7.1.1	Solicited adverse events - local symptom: pain
16.2.7.1.2	Solicited adverse events - local symptoms: redness, swelling, induration
16.2.7.1.3	Solicited adverse events - general symptom: body temperature
16.2.7.1.4	Solicited adverse events - general symptoms: headache, myalgia, nausea, fatigue
16.2.7.2.	Unsoliceted adverse events
16.2.7.2.1	Unsoliceted adverse events: Details
16.2.7.2.2	Unsoliceted adverse events: MedDRA coding
**16.2.8.**	**LISTING OF INDIVIDUAL LABORATORY MEASUREMENTS BY SUBJECT**
16.2.8.1.	Laboratory tests, reference ranges
16.2.8.2.	Date and time of blood analysis, check for sample taken, laboratory order number
16.2.8.3.	Date and time of urine analysis, check for sample taken, laboratory order number
16.2.8.4.	Date and time of serum pregnancy test, check for sample taken, laboratory order number

Section number	Title
16.2.8.5.	Date and time of urine pregnancy test, check for sample taken, result
16.2.8.6.	Central laboratory tests, results
**16.2.9.**	**SAFETY DATA**
16.2.9.1.	Physical examination
16.2.9.1.1	Physical examination at screening
16.2.9.1.2	Physical examination – changes since last examination
16.2.9.2.	Vital signs
16.2.9.3.	ECG
16.2.9.3.1	ECG, date, time, overall assessment by investigator and local cardiologist
16.2.9.3.2	ECG data, details abnormal ECGs
16.2.9.3.3	ECG data, details ST-elevation
16.2.9.3.4	ECG data, details ST-depression
16.2.9.3.5	ECG data, details T-inversion, pathological
16.2.9.3.6	Central ECG raw data
16.2.9.3.7	Central ECG data: PR, QRS, RR interval, QTc Bazett and Fridericia correction
16.2.9.6.	Check for hand-out and collection of diary
16.2.9.7.	General comments
16.2.9.8.	Prior and concomitant medication

16.1.10 Laboratory methods, quality assurance and reference ranges

Laboratory methods, quality assurance and reference ranges

- Laboratory methods:

  standard methods

- Reference ranges:

  see Appendix 16.2.8.1 and 16.2.10.2.1 of this report

2382

Certificate

External Quality Assessment Scheme October 2006

INSTAND e.V.
Gesellschaft zur Förderung
der Qualitätssicherung in
medizinischen Laboratorien e. V.
(vormals Hämometerprüfstelle)
Ubier - Str. 20 / PF 250211
40223 / 40093 Düsseldorf
Tel. (0211) 159213 - 0
FAX (0211) 159213 - 30

This is to certify that the undermentioned participant has fullfilled the requirements for the following analyts/parameters;

DIFFERENTIAL BLOOD COUNT : blood cell differentiation - percent

THYROID HORMONES : T3 (M.41) (R), free T3 (M.41), T4 (M.41) (R), free T4 (M.41)
TSH (M.41) (R), TBG (M.22)

2382
Laborgemeinschaft
Bayerischer Aerzte
Abt. QM
Bayerstr. 53
80335 Muenchen

INTERLAB
central lab services–worldwide
INTERLAB GmbH – Bayerstr. 53 - D-80335 München
Phone: +49 741 393-0 - Fax +49 741 393-39

Gesellschaft zur Förderung der Qualitätssicherung in medizinischen Laboratorien e.V.
INSTAND e.V.
Abt. Qualitätskontrolle
und Ringversuche
Düsseldorf

Düsseldorf, der 21.12.2006

Prof. Dr. med. Reinauer
Von der Bundesärztekammer
ernannter Ringversuchsleiter

Prof. Dr. med. Geisen
Ringversuchsleiter
Immunhämatologie

(R) : entspricht RiliBÄK.

212 294

02382 1517

Bl. 1517 Rv=606, Crvz/Lzen 21.12.2006 vp161644

**Ringversuch:**	**CM2/06**
**im Monat:**	**September**
**Teilnehmer-Nr.:**	**6326050**
**ausgestellt am:**	**23.09.2006**

DGKL
Deutsche Vereinte Gesellschaft für Klinische Chemie und Laboratoriumsmedizin e.V.

Referenzinstitut für Bioanalytik

Deutscher Akkreditierungs Rat
DAR
DAC-IS-004-06-00

Medizinisches Versorgungszentrum
Dres. Tiller und Kollegen
Abteilung QM
Bayerstr. 53
80335 München

Ringversuchsleiter
Prof. Dr. K. Kleesiek
Prof. Dr. Dr. K. P. Kohse
Prof. Dr. M. Neumaier

INTERLAB
central lab services–worldwide
INTERLAB GmbH – Bayerstr. 53 - D-80335 München
Phone: +49 741 393-0 - Fax +49 741 393-39

25.10.06

Bonn, 17. Oktober 2006

Zertifikat

Wir bestätigen Ihnen, dass Sie am Ringversuch für kardiale Marker im Serum teilgenommen haben.
Sie haben die Anforderungen des Ringversuches für folgende Messgrößen erfüllt:

CK-MB Akt.	(7)	Myoglobin	(3)	Troponin I	(2)
Troponin T	(5)	ProBNP	(5)		

Dieses Zertifikat ist gültig bis einschließlich September 2007.

K. Kleesiek

Die hinter jedem Analyten angegebene Ziffer kennzeichnet die angewendete Analysenmethode.
Die Zuordnung der Ziffer zur jeweiligen Methode ist der Gesamtauswertung zu entnehmen.

**Ringversuch:**	**KS7/06**
**im Monat:**	**Oktober**
**Teilnehmer-Nr.:**	**630650L**
**ausgestellt am:**	**09.10.2006**

DGKL
Deutsche Vereinte Gesellschaft
für Klinische Chemie und Laboratoriumsmedizin e.V.

Referenzinstitut für Bioanalytik

Deutscher Akkreditierungs Rat
DAR
DAC-IS-004-06-00

Herr
PD Dr. med. Tiller u. Kollegen
Laborgemeinschaft Bayer. Ärzte
Bayerstr. 53
80335 München

Ringversuchsleiter
Prof. Dr. K. Kleesiek
Prof. Dr. Dr. K. P. Kohse
Prof. Dr. M. Neumaier

INTERLAB
central lab services–worldwide

INTERLAB GmbH – Bayerstr. 53 - D-80335 München
Phone: +49 741 393-0 - Fax +49 741 393-39

Bonn, 27. Oktober 2006

Zertifikat

Wir bestätigen Ihnen, dass Sie am Ringversuch für klinisch-chemische Analyte im Serum teilgenommen haben.
Sie haben die Anforderungen des Ringversuches für folgende Messgrößen erfüllt:

Bilirubin, ges.	(2)	Cholesterin	(1)	Glucose	(1)
Harnsäure	(3)	Harnstoff	(2)	Kreatinin	(1)
Lactat	(2)	Triglyceride	(2)	Gesamtprotein	(1)
Albumin (CRM)	(1)	Elektrophorese	(7)	Calcium	(5)
Chlorid	(5)	Kalium	(2)	Lithium	(3)
Magnesium	(4)	Natrium	(2)	Phosphor, anorg.	(2)
Eisen	(2)	Kupfer	(2)	Pankr. Amylase	(1)
Cholinesterase	(1)	Alk. P.ase	(1)	CK	(1)
GLDH	(1)	AST (GOT)	(1)	ALT (GPT)	(1)
Alpha-HBDH	(1)	LDH	(1)	Gamma-GT	(1)
Lipase	(3)	Saure P.ase	(1)	Prostata SP	(1)

Dieses Zertifikat ist gültig bis einschließlich April 2007.

K. Kleesiek

Die hinter jedem Analyten angegebene Ziffer kennzeichnet die angewendete Analysenmethode.
Die Zuordnung der Ziffer zur jeweiligen Methode ist der Gesamtauswertung zu entnehmen.

**Ringversuch:**	KS5/06
**im Monat:**	Juli
**Teilnehmer-Nr.:**	630650L
**ausgestellt am:**	15.07.2006

DGKL
Deutsche Vereinte Gesellschaft für Klinische Chemie und Laboratoriumsmedizin e.V.

Referenzinstitut für Bioanalytik

Deutscher Akkreditierungs Rat
DAR
DAC-IS-004-06-00

Herr
PD Dr. med. Tiller u. Kollegen
Laborgemeinschaft Bayer. Ärzte
Bayerstr. 53
80335 München

Ringversuchsleiter
Prof. Dr. K. Kleesiek
Prof. Dr. Dr. K. P. Kohse
Prof. Dr. M. Neumaier

INTERLAB
central lab services–worldwide

INTERLAB GmbH – Bayerstr. 53 - D-80335 München
Phone: +49 741 393-0 - Fax +49 741 393-39

B. Gruner
02.10.06

Bonn, 2. August 2006

Zertifikat

Wir bestätigen Ihnen, dass Sie am Ringversuch für klinisch-chemische Analyte im Serum teilgenommen haben.
Sie haben die Anforderungen des Ringversuches für folgende Messgrößen erfüllt:

Bilirubin, ges.	(2)	Cholesterin	(1)	Glucose	(1)
Harnsäure	(3)	Harnstoff	(2)	Kreatinin	(1)
Lactat	(2)	Triglyceride	(2)	Gesamtprotein	(1)
Albumin (CRM)	(1)	Elektrophorese	(7)	Calcium	(5)
Chlorid	(5)	Kalium	(2)	Lithium	(3)
Magnesium	(4)	Natrium	(2)	Phosphor, anorg.	(2)
Eisen	(2)	Kupfer	(2)	Pankr. Amylase	(1)
Cholinesterase	(1)	Alk. P.ase	(1)	CK	(1)
GLDH	(1)	AST (GOT)	(1)	ALT (GPT)	(1)
Alpha-HBDH	(1)	LDH	(1)	Gamma-GT	(1)
Lipase	(3)	Saure P.ase	(1)	Prostata SP	(1)

Dieses Zertifikat ist gültig bis einschließlich Januar 2007.

K. Kleesiek

Die hinter jedem Analyten angegebene Ziffer kennzeichnet die angewendete Analysenmethode.
Die Zuordnung der Ziffer zur jeweiligen Methode ist der Gesamtauswertung zu entnehmen.

Ringversuch:	KS3/06
im Monat:	April
Teilnehmer-Nr.:	630650L
ausgestellt am:	24.04.2006

DGKL
Deutsche Vereinte Gesellschaft für Klinische Chemie und Laboratoriumsmedizin e.V.

Referenzinstitut für Bioanalytik

Deutscher Akkreditierungs Rat
DAR
DAC-IS-004-06-00

Herr
PD Dr. med. Tiller u. Kollegen
Laborgemeinschaft Bayer. Ärzte
Bayerstr. 53
80335 München

Ringversuchsleiter
Prof. Dr. K. Kleesiek
Prof. Dr. Dr. K. P. Kohse
Prof. Dr. M. Neumaier

INTERLAB
central lab services–worldwide

INTERLAB GmbH – Bayerstr. 53 - D-80335 München
Phone: +49 741 393-0 - Fax +49 741 393-39

Bonn, 11. Mai 2006

B. Grimm 01.08.06

Zertifikat

Wir bestätigen Ihnen, dass Sie am Ringversuch für klinisch-chemische Analyte im Serum teilgenommen haben.
Sie haben die Anforderungen des Ringversuches für folgende Messgrößen erfüllt:

Bilirubin, ges.	(2)	Cholesterin	(1)	Glucose	(1)
Harnsäure	(3)	Harnstoff	(2)	Kreatinin	(1)
Lactat	(2)	Triglyceride	(2)	Gesamtprotein	(1)
Albumin (CRM)	(1)	Elektrophorese	(7)	Calcium	(5)
Chlorid	(5)	Kalium	(2)	Lithium	(3)
Magnesium	(4)	Natrium	(2)	Phosphor, anorg.	(2)
Eisen	(2)	Kupfer	(2)	Pankr. Amylase	(1)
Cholinesterase	(1)	Alk. P.ase	(1)	CK	(1)
GLDH	(1)	AST (GOT)	(1)	ALT (GPT)	(1)
Alpha-HBDH	(1)	LDH	(1)	Gamma-GT	(1)
Lipase	(3)	Saure P.ase	(1)	Prostata SP	(1)

Dieses Zertifikat ist gültig bis einschließlich Oktober 2006.

K. Kleesiek

Die hinter jedem Analyten angegebene Ziffer kennzeichnet die angewendete Analysenmethode.
Die Zuordnung der Ziffer zur jeweiligen Methode ist der Gesamtauswertung zu entnehmen.

**Ringversuch:**	**KS1/06**
**im Monat:**	**Januar**
**Teilnehmer-Nr.:**	**630650L**
**ausgestellt am:**	**14.01.2006**

DGKL
Deutsche Vereinte Gesellschaft für Klinische Chemie und Laboratoriumsmedizin e.V.

Referenzinstitut für Bioanalytik

Herr
PD Dr. med. Tiller u. Kollegen
Laborgemeinschaft Bayer. Ärzte
Bayerstr. 53
80335 München

Ringversuchsleiter
Prof. Dr. K. Kleesiek
Prof. Dr. Dr. K. P. Kohse
Prof. Dr. M. Neumaier

INTERLAB
central lab services–worldwide

INTERLAB GmbH – Bayerstr. 53 - D-80335 München
Phone: +49 741 393-0 - Fax +49 741 393-39

3. 5. 06 S. Plever

Bonn, 8. Februar 2006

Zertifikat

Wir bestätigen Ihnen, dass Sie am Ringversuch für klinisch-chemische Analyte im Serum teilgenommen haben.
Sie haben die Anforderungen des Ringversuches für folgende Messgrößen erfüllt:

Bilirubin, ges.	(2)	Cholesterin	(1)	Glucose	(1)
Harnsäure	(3)	Harnstoff	(2)	Kreatinin	(1)
Lactat	(2)	Triglyceride	(2)	Gesamtprotein	(1)
Albumin (CRM)	(1)	Elektrophorese	(7)	Calcium	(5)
Chlorid	(5)	Kalium	(2)	Lithium	(3)
Magnesium	(4)	Natrium	(2)	Phosphor, anorg.	(2)
Eisen	(2)	Kupfer	(2)	Pankr. Amylase	(1)
Cholinesterase	(1)	Alk. P.ase	(1)	CK	(1)
AST (GOT)	(1)	ALT (GPT)	(1)	Alpha-HBDH	(1)
LDH	(1)	Gamma-GT	(1)	Lipase	(3)
Saure P.ase	(1)	Prostata SP	(1)		

Dieses Zertifikat ist gültig bis einschließlich Juli 2006.

K. Kleesiek

Die hinter jedem Analyten angegebene Ziffer kennzeichnet die angewendete Analysenmethode.
Die Zuordnung der Ziffer zur jeweiligen Methode ist der Gesamtauswertung zu entnehmen.

Ringversuch:	KS7/05
im Monat:	Oktober
Teilnehmer-Nr.:	630650L
ausgestellt am:	10.10.2005

DGKL
Deutsche Vereinte Gesellschaft für Klinische Chemie und Laboratoriumsmedizin e.V.

Referenzinstitut für Bioanalytik

Herr
PD Dr. med. Tiller u. Kollegen
Laborgemeinschaft Bayer. Ärzte
Bayerstr. 53

80335 München

Ringversuchsleiter
Prof. Dr. K. Kleesiek
Prof. Dr. Dr. K. P. Kohse
Prof. Dr. M. Neumaier

INTERLAB
central lab services – worldwide

INTERLAB GmbH – Bayerstr. 53 - D-80335 München
Phone: +49 741 393-0 - Fax +49 741 393-39
2006-03-29 A. [illegible]

Bonn, 3. November 2005

Zertifikat

Wir bestätigen Ihnen, dass Sie am Ringversuch für klinisch-chemische Analyte im Serum teilgenommen haben.
Sie haben die Anforderungen des Ringversuches für folgende Messgrößen erfüllt:

Bilirubin, ges.	(2)	Cholesterin	(1)	Glucose	(1)
Harnsäure	(3)	Harnstoff	(2)	Kreatinin	(1)
Lactat	(2)	Triglyceride	(2)	Gesamtprotein	(1)
Albumin (CRM)	(1)	Elektrophorese	(7)	Calcium	(5)
Chlorid	(5)	Kalium	(2)	Lithium	(3)
Magnesium	(4)	Natrium	(2)	Phosphor, anorg.	(2)
Eisen	(2)	Kupfer	(2)	Pankr. Amylase	(1)
Cholinesterase	(1)	Alk. P.ase	(1)	CK	(1)
GLDH	(1)	AST (GOT)	(1)	ALT (GPT)	(1)
Alpha-HBDH	(1)	LDH	(1)	Gamma-GT	(1)
Lipase	(3)	Saure P.ase	(1)	Prostata SP	(1)

Dieses Zertifikat ist gültig bis einschließlich April 2006.

K. Kleesiek

Die hinter jedem Analyten angegebene Ziffer kennzeichnet die angewendete Analysenmethode.
Die Zuordnung der Ziffer zur jeweiligen Methode ist der Gesamtauswertung zu entnehmen.

Ringversuch:	LP3/06
im Monat:	August
Teilnehmer-Nr.:	6326050
ausgestellt am:	19.08.2006

DGKL
Deutsche Vereinte Gesellschaft für Klinische Chemie und Laboratoriumsmedizin e.V.

Referenzinstitut für Bioanalytik

Deutscher Akkreditierungs Rat
DAR
DAC-IS-004-06-00

Medizinisches Versorgungszentrum
Dres. Tiller und Kollegen
Abteilung QM
Bayerstr. 53
80335 München

Ringversuchsleiter
Prof. Dr. K. Kleesiek
Prof. Dr. Dr. K. P. Kohse
Prof. Dr. M. Neumaier

INTERLAB
central lab services–worldwide
INTERLAB GmbH – Bayerstr. 53 - D-80335 München
Phone: +49 741 393-0 - Fax +49 741 393-39

B. [illegible] 02.10.06

Bonn, 26. September 2006

Zertifikat

Wir bestätigen Ihnen, dass Sie am Ringversuch für Lipoproteine teilgenommen haben.
Sie haben die Anforderungen des Ringversuches für folgende Messgrößen erfüllt:

Cholesterin	(2)	Triglyceride	(2)	HDL-Cholesterin	(1)
LDL-Cholesterin	(1)	Apolipoprot. A1	(1)	Apolipoprot. B	(1)
Lipoprotein (a)	(1)	Homocystein	(3)	HDL-Chol., Elpho	(5)
LDL-Chol., Elpho	(4)				

Dieses Zertifikat ist gültig bis einschließlich Februar 2007.

K. Kleesiek

Die hinter jedem Analyten angegebene Ziffer kennzeichnet die angewendete Analysenmethode.
Die Zuordnung der Ziffer zur jeweiligen Methode ist der Gesamtauswertung zu entnehmen.

**Ringversuch:**	**LP1/06**
**im Monat:**	**März**
**Teilnehmer-Nr.:**	**6326050**
**ausgestellt am:**	**11.03.2006**

DGKL Deutsche Vereinte Gesellschaft für Klinische Chemie und Laboratoriumsmedizin e.V.

Referenzinstitut für Bioanalytik

Deutscher Akkreditierungs Rat
DAR
DAC-IS-004-06-00

Ringversuchsleiter
Prof. Dr. K. Kleesiek
Prof. Dr. Dr. K. P. Kohse
Prof. Dr. M. Neumaier

Medizinisches Versorgungszentrum
Dres. Tiller und Kollegen
Abteilung QM
Bayerstr. 53
80335 München

INTERLAB
central lab services–worldwide

INTERLAB GmbH – Bayerstr. 53 - D-80335 München
Phone: +49 741 393-0 - Fax +49 741 393-39

Bonn, 5. April 2006

3.5.06 s. [illegible]

Zertifikat

Wir bestätigen Ihnen, dass Sie am Ringversuch für Lipoproteine teilgenommen haben.
Sie haben die Anforderungen des Ringversuches für folgende Messgrößen erfüllt:

Cholesterin	(2)	Triglyceride	(2)	HDL-Cholesterin	(1)
LDL-Cholesterin	(1)	Apolipoprot. A1	(1)	Apolipoprot. B	(1)
Lipoprotein (a)	(1)	Homocystein	(3)	HDL-Chol., Elpho	(5)
LDL-Chol., Elpho	(4)				

Dieses Zertifikat ist gültig bis einschließlich September 2006.

K. Kleesiek

Die hinter jedem Analyten angegebene Ziffer kennzeichnet die angewendete Analysenmethode.
Die Zuordnung der Ziffer zur jeweiligen Methode ist der Gesamtauswertung zu entnehmen.

Certificate

External Quality Assessment Scheme October 2006

INSTAND e.V.
Gesellschaft zur Förderung der Qualitätssicherung in medizinischen Laboratorien e. V.
(vormals Hämometerprüfstelle)
Ubier - Str. 20 / PF 250211
40223 / 40093 Düsseldorf
Tel. (0211) 159213 - 0
FAX (0211) 159213 - 30

(R) = RiliBÄK; valid 6 months

This is to certify that the undermentioned participant has fullfilled the requirements for the following analyts/parameters:

**GLYCATED PROTEINS:**	HbA1C (M.9) (R)	HbA1C (M.4) (R)	fructosamine
**BLOOD CELL COUNT :**	haemoglobin (M.7) (R)	erythrocytes (M.1) (R)	MCV
haematocrit el.count (R)	WBC (leucocytes) (M.1) (R)	platelet count (M.1) (R)	

2382
Laborgemeinschaft
Bayerischer Aerzte
Abt. QM
Bayerstr. 53
80335 Muenchen

INTERLAB
central lab services–worldwide
INTERLAB GmbH – Bayerstr. 53 - D-80335 München
Phone: +49 741 393-0 - Fax +49 741 393-39

Gesellschaft zur Förderung der Qualitätssicherung in medizinischen Laboratorien e.V.
INSTAND e.V.
Abt. Qualitätskontrolle und Ringversuche
Düsseldorf

Düsseldorf, der 13.12.2006

Prof. Dr. med. Reinauer
(Director of the board)

(R) : RiliBÄK

145 146 211

02382 1805

Bl. 1805 Rv=606, Crvz/Lzert 12.12.2006 vp135732

2382

Certificate

External Quality Assessment Scheme July 2006

(R) = RiliBÄK: Gültigkeitsdauer 6 Monate

INSTAND e.V.
Gesellschaft zur Förderung
der Qualitätssicherung in
medizinischen Laboratorien e. V.
(vormals Hämometerprüfstelle)
Ubier - Str. 20 / PF 250211
40223 / 40093 Düsseldorf
Tel. (0211) 159213 - 0
FAX (0211) 159213 - 30

This is to certify that the undermentioned participant has fullfilled the requirements for the following analyts/parameters:

**BLOOD CELL COUNT :**	**haemoglobin (M.7) (R)**	**erythrocytes (M.1) (R)**	**MCV**
**haematocrit el.count (R)**	**WBC (leucocytes) (M.1) (R)**	**platelet count (M.1) (R)**	**neutrophil granulocytes**
**lymphocytes**	**eosinophil granulocytes**	**basophile granulocytes**	**monocytes**

2382
Laborgemeinschaft
Bayerischer Aerzte
Abt. QM
Bayerstr. 53
80335 Muenchen

INTERLAB
central lab services – worldwide

INTERLAB GmbH – Bayerstr. 53 - D-80335 München
Phone: +49 741 393-0 - Fax +49 741 393-39

(R) : entspricht RiliBÄK B. Gruiun 25.10.06

Gesellschaft zur Förderung der Qualitätssicherung in medizinischen Laboratorien e.V. INSTAND e.V. Abt. Qualitätskontrolle und Ringversuche Düsseldorf

Düsseldorf, der 25.08.2006

Reinauer

Prof. Dr. med. Reinauer
(Director of the board)

02382 511

612

Bl. 511 Rv=604, Crvz/Lzert 24.8.2006 DAC

2382

INSTAND e.V.
Gesellschaft zur Förderung der Qualitätssicherung in medizinischen Laboratorien e. V.
(vormals Hämometerprüfstelle)
Ubier - Str. 20 / PF 250211
40223 / 40093 Düsseldorf
Tel. (0211) 159213 - 0
FAX (0211) 159213 - 30

Certificate

External Quality Assessment Scheme May 2006

(R) = RiliBÄK: Gültigkeitsdauer 6 Monate

This is to certify that the undermentioned participant has fullfilled the requirements for the following analyts/parameters:

**BLOOD CELL COUNT :**	haemoglobin (M.7) (R)	erythrocytes (M.1) (R)	MCV
haematocrit el.count (R)	WBC (leucocytes) (M.1) (R)	platelet count (M.1) (R)	
**DIFFERENTIAL BLOOD COUNT :**		blood cell differentiation - percent	
blood cell differentiation - diagnosis		blood cell differentiation - morphology	

2382
Laborgemeinschaft
Bayerischer Aerzte
Abt. QM
Bayerstr. 53
80335 Muenchen

INTERLAB
central lab services–worldwide

INTERLAB GmbH – Bayerstr. 53 - D-80335 München
Phone: +49 741 393-0 - Fax +49 741 393-39

B. Grimm 01.08.06

(R) : entspricht RiliBÄK

Gesellschaft zur Förderung der Qualitätssicherung in medizinischen Laboratorien e.V.
INSTAND e.V.
Abt. Qualitätskontrolle und Ringversuche
Düsseldorf

Düsseldorf, der 18.7.2006

Reinauer

Prof. Dr. med. Reinauer
(Director of the board)

02382 605

Certificate

External Quality Assessment Scheme October 2005

(R) = RiliBÄK: Gültigkeitsdauer 6 Monate

INSTAND e.V.
Institut für Standardisierung und Dokumentation im medizinischen Laboratorium e. V.
Ubier - Str. 20 / PF 250211
40223 / 40093 Düsseldorf
Tel. (0211) 159213 - 0
FAX (0211) 159213 - 30

This is to certify that the undermentioned participant has fullfilled the requirements for the following analyts/parameters:

**GLYCATED PROTEINS:**	HbA1C (M.4) (R)	fructosamine	
**BLOOD CELL COUNT :**	haemoglobin (M.7) (R)	erythrocytes (M.1) (R)	MCV
haematocrit el.count (R)	WBC (leucocytes) (M.1) (R)	platelet count (M.1) (R)	
blood cell differentiation - percent		blood cell differentiation - morphology	
**MYOCARDIAL MARKERS :**	CK-MB (M.11)	CK-MB (M.11)	myoglobin (M.2)
troponin T	troponin T		

2382
Laborgemeinschaft
Bayerischer Aerzte
Abt. QM
Bayerstr. 53
80335 Muenchen

INTERLAB
central lab services–worldwide
INTERLAB GmbH – Bayerstr. 53 - D-80335 München
Phone: +49 741 393-0 - Fax +49 741 393-39
2006-03-29 A. [illegible]
(R) : entspricht RiliBÄK

145 211 212 760

02382 1643

INSTAND Abt. Qualitätskontrolle und Ringversuche Düsseldorf — Institut f. Standardisierung und Dokumentation im medizin. Laboratorium e.V.

Düsseldorf, der 14.12.2005

Reinauer

Prof. Dr. med. Reinauer
(Director of the board)

Bl. 1643 Rv=506, Crvz/Lzert 13.12.2005 DAC

Certificate

External Quality Assessment Scheme October 2006

INSTAND e.V.
Gesellschaft zur Förderung
der Qualitätssicherung in
medizinischen Laboratorien e. V.
(vormals Hämometerprüfstelle)
Ubier - Str. 20 / PF 250211
40223 / 40093 Düsseldorf
Tel. (0211) 159213 - 0
FAX (0211) 159213 - 30

This is to certify that the undermentioned participant has fullfilled the requirements for the following analyts/parameters:

URINE CHEMISTRY:
HCG (M.1), specif.gravity (M.1), bilirubin (M.1), ketone bodies (M.1), urobilinogen (M.1), protein (M.1), leucocytes (M.1), erythrocytes (M.1), nitrite (M.1), glucose (M.1), pH (M.1)

URINE CONTROL - SEDIMENT :
Urine Control - sediment

URINE CHEMISTRY:
metanephrines (M.72), vanillylmandelic acid (M.72), epinephrine (M.72), norepinephrine (M.72), dopamine (M.72), normetanephrine (M.72), homovanillic acid (M.72), 5-hydroxyindolea.acid (M.72)

TRICYCLIC ANTIDEPRESSANTS :
nortriptyline, doxepin, imipramine, nordoxepin, amitriptyline, desipramine

TRACE ELEMENTS:
chromium (M.3), copper (M.14), zinc (M.2), cadmium in plasma (M.14), selenium in plasma (M.3), molybdaen in plasma (M.14), lead in whole blood (M.14), cobalt in whole blood (M.14), selenium in whole blood (M.3), arsenic (M.3), cadmium (M.14), nickel (M.3), chromium in plasma (M.3), cobalt in plasma (M.14), zinc in plasma (M.2), tin in plasma (M.14), chromium in whole blood (M.3), manganese in whole blood (M.3), zinc in whole blood (M.2), lead (M.14), cobalt (M.14), mercury inorganic (M.13), gold in plasma (M.14), copper in plasma (M.2), antimon in plasma (M.14), antimon in whole blood (M.14), cadmium in whole blood (M.14), mercury in whole blood (M.13)

RETICULOCYTE DETERM.

196
Dr. med. Tiller & Kollegen
Abt. QM
Med.Versorgungszentrum
Bayerstr. 53
80335 Muenchen

Düsseldorf, der 21.12.2006

Prof. Dr. med. Reinauer
Von der Bundesärztekammer
ernannter Ringversuchsleiter

Prof. Dr. med. Geisen
Ringversuchsleiter
Immunhämatologie

Gesellschaft zur Förderung der Qualitätssicherung in medizinischen Laboratorien e.V.
INSTAND e.V.
Abt. Qualitätskontrolle
und Ringversuche
Düsseldorf

INTERLAB
central lab services – worldwide
INTERLAB GmbH – Bayerstr. 53 - D-80335 München
Phone: +49 741 393-0 - Fax +49 741 393-39

(R) : entspricht RiliBÄK.

171 172 175 199 205 206 207 208 216 217 297 301 455 720 760

Bl. 175 Rv=606, Crvz/Lzeit 21.12.2006 vp103710

00196 175

Certificate

External Quality Assessment Scheme May 2006

(R) = RiliBÄK: Gültigkeitsdauer 6 Monate

INSTAND e.V.
Gesellschaft zur Förderung
der Qualitätssicherung in
medizinischen Laboratorien e. V.
(vormals Hämometerprüfstelle)
Ubier - Str. 20 / PF 250211
40223 / 40093 Düsseldorf
Tel. (0211) 159213 - 0
FAX (0211) 159213 - 30

This is to certify that the undermentioned participant has fullfilled the requirements for the following analyts/parameters:

**URINE CHEMISTRY:**	**bilirubin (M.1)**	**protein (M.1)**	**glucose (M.1)**	**HCG (M.1)**
ketone bodies (M.1)	leucocytes (M.1)	nitrite (M.1)	specif.gravity (M.1)	urobilinogen (M.1)
**URINE CONTROL - SEDIMENT :**		Urine Control - sediment		
**URINE CHEMISTRY:**	epinephrine (M.72)	dopamine (M.72)	homovanillic acid (M.72)	5-hydroxyindolea.acid (M.72)
metanephrines (M.72)	norepinephrine (M.72)	normetanephrine (M.72)	vanillylmandelic acid (M.72)	
**PREGNANCY / FERTILITY ANALYTES :**		AFP (M.41) (R)	hCG total+b-hCG free (M.41) (R)	
LH (M.41)	FSH (M.41)			
**PANCREAS / GASTROINT.TRACT :**		insulin (M.22)		
C-peptide (M.22)	gastrin (M.22)	pro-insuline (M.20)		
**CARBOHYDRATE DEFICIENT TRANSF. :**		CDT (M.2)		

196
Dr. med. Tiller & Kollegen
Abt. QM
Med.Versorgungszentrum
Bayerstr. 53
80335 Muenchen

INTERLAB
central lab services – worldwide

INTERLAB GmbH – Bayerstr. 53 - D-80335 München
Phone: +49 741 393-0 - Fax +49 741 393-39

(R) : entspricht RiliBÄK

171 172 175 297 300 750

B. Grimm 02.08.06

Gesellschaft zur Förderung der Qualitätssicherung in medizinischen Laboratorien e.V.
INSTAND e.V.
Abt. Qualitätskontrolle
und Ringversuche
Düsseldorf

Düsseldorf, der 10.7.2006

Reinauer

Prof. Dr. med. Reinauer
(Director of the board)

00196 115

Bl. 115 Rv=603, Crvz/Lzert 10.7.2006 DAC

Certificate

External Quality Assessment Scheme October 2005

(R) = RiliBÄK: Gültigkeitsdauer 6 Monate

INSTAND e.V.
Institut für Standardisierung und Dokumentation im medizinischen Laboratorium e. V.
Ubier - Str. 20 / PF 250211
40223 / 40093 Düsseldorf
Tel. (0211) 159213 - 0
FAX (0211) 159213 - 30

This is to certify that the undermentioned participant has fullfilled the requirements for the following analyts/parameters:

URINE CHEMISTRY:
HCG (M.1)
urobilinogen (M.1)
epithelium by microscope
5-hydroxyindolea.acid (M.72)
bilirubin (M.1)
ketone bodies (M.1)
erythrocytes by microscope
cylinders by microscope
metanephrines (M.72)
protein (M.1)
leucocytes (M.1)
leucocytes by microscope
epinephrine (M.72)
norepinephrine (M.72)
erythrocytes (M.1)
nitrite (M.1)
crystals by microscope
dopamine (M.72)
normetanephrine (M.72)
glucose (M.1)
specif.gravity (M.1)
bacteria by microscope
homovanillic acid (M.72)
vanillylmandelic acid (M.72)

TRICYCLIC ANTIDEPRESSANTS :
desipramine
doxepin
amitriptyline
nordoxepin
nortriptyline
imipramine

TRACE ELEMENTS:
copper in plasma (M.2)
Platin in plasma (M.14)
antimon in plasma (M.14)
arsenic in whole blood (M.3)
cadmium in whole blood (M.3)
nickel in whole blood (M.3)
zinc in whole blood (M.2)
arsenic in plasma (M.3)
manganese in plasma (M.3)
selenium in plasma (M.3)
molybdaen in plasma (M.14)
lead in whole blood (M.3)
cobalt in whole blood (M.14)
mercury in whole blood (M.13)
cobalt in plasma (M.14)
nickel in plasma (M.3)
zinc in plasma (M.2)
antimon in whole blood (M.14)
chromium in whole blood (M.3)
manganese in whole blood (M.3)
selenium in whole blood (M.3)

FREE HAEMOGLOBIN :
haemoglobin free (M.3)

PREGNANCY / FERTILITY ANALYTES :
hCG total+b-hCG free (M.41) (R)
FSH (M.41)
17-b-estradiol (M.41) (R)
AFP (M.41) (R)
LH (M.41)
progesterone (M.41) (R)

196 (KV. 6326047)
Dr.med. Tiller & Kollegen
Abt. QM
Bayerstr. 53
80335 Muenchen

Düsseldorf, der 20.12.2005

INSTAND Abt. Qualitätskontrolle und Ringversuche Düsseldorf — Institut f. Standardisierung und Dokumentation im medizin. Laboratorium e.V.

Reinauer

Prof. Dr. med. Reinauer
(Director of the board)

INTERLAB
central lab services–worldwide

INTERLAB GmbH – Bayerstr. 53 - D-80335 München
Phone: +49 741 393-0 - Fax +49 741 393-39

(R) : entspricht RiliBÄK

171 175 199 206 207 208 236 297 2006-03-29 A.

00196 133

Bl. 133 Rv=506, Crvz/Lzert 19.12.2005 DAC

INTERLAB
central lab services – worldwide

Translation

DACH Deutsche Akkreditierungsstelle Chemie GmbH

Signatory to EA Multilateral Agreement and ILAC Mutual Recognition Arrangement

represented in the

Deutschen AkkreditierungsRat

Accreditation

The DACH Deutsche Akkreditierungsstelle Chemie GmbH herewith confirms that the laboratories

**Laborgemeinschaft Bayerischer Ärzte GbR**	**Medizinisches Versorgungszentrum Labor Dr. Tiller & Kollegen GbR**	**Praxisgemeinschaft Medizinisch Genetisches Zentrum**

Bayerstraße 53

D-80335 München

are competent according to DIN EN ISO/IEC 17025 and DIN EN ISO 15189 to carry out testing

**in the fields**	**medical laboratory diagnosis**<br>**forensic genetik (paternity tests)**<br>**biology**
in the analytical field	clinical chemistry, immunology, microbiology, virology, human genetics, transfusion medicine, hygiene
for the types of tests	analyses of chromosoms, microscopy, amplification, agglutination testing, flow cytometry, electrophoresis, ligand assays, nephelometry, GC-MS, HPLC, electrochemical examinations, coagulation tests, characteristics of particels, AAS, photometry, turbidimetry, filtration, differentiation- / identification of germs, culture examinations, sensitivity tests of bacteria and fungus, CFT, PCR
for individual test methods of the	immunhistochemistry, cell function tests, aggregometry, thin layer chromatography, GC, functional tests at patients, osmometry, qualitative examinations with visual evaluation, rheology, sediment examinations, AES, ICP-MS, hybridisation procedures, neutralisation tests, centrifugation, unspecific culturing methods, specific culturing methods, sampling of drinking water
for analysis of	human specimen, rinsing liquids, bioindicators, dialysis extracts, catheder materials, cultures, water, swimming-, bathing and drinking water.

This accreditation is valid until: 04.12.2010

The annex is part of this certificate and comprises 38 pages.

DAR registration number: DAC-ML-0431-00-10

Frankfurt, 3rd of February 2006

Dr. A. Steinhorst
Managing director

Deutsche Akkreditierungsstelle Chemie GmbH DACH

Translation for information purposes only. The German Accreditation Certificate is authoritative.

See notes overleaf.

This accreditation has been awarded on the basis of an assessment and pursuant to the agreement concluded with the accreditation body with respect to the accreditation of a testing laboratory in accordance with the rules and procedures of the German Accreditation System, in conformity with the standards DIN EN ISO 15189, DIN EN ISO/IEC 17025 and DIN EN ISO/IEC 17011.

The requirements in terms of materials and personnel as specified in DIN EN ISO 15189 and DIN EN ISO/IEC 17025 for the specific tests indicated in the accreditation certificate, as well as for the procedures described in the annex to the accreditation certificate, have been met.

Details on the scope of the accreditation (test fields, procedures and specifications) are given in the annex to this accreditation certificate.

The annex and the documents submitted in connection with the accreditation are deemed to form an integral part of it. Any amendments are to be made in writing.

The accreditation is awarded subject to revocation at any time on the fundamental change or lapse of any conditions defined in the agreement and in the annex to this accreditation certificate.

The Deutsche Akkreditierungsstelle Chemie (DACH) has signed the Multilateral Agreement (MLA) of the European Organisation of Accreditation Bodies (EA, European co-operation for Accreditation) and the International Agreement for Mutual Recognition of ILAC (International Laboratory Accreditation Cooperation).
Signatories of the MLA are requested to promote the recognition of accreditations granted in all the other member states in their own country. Therefore laboratories accredited by DACH bear international recognition in the following countries:

Australia, Belgium, Brazil, Denmark, Finland, France, Greek, United Kingdom, Hong Kong (China), India, Indonesia, Ireland, Israel, Italy, Japan, Canada, Korea, Lithuania, New Zealand, The Netherlands, Norway, Austria, Poland, Romania, Spain, Sweden, Switzerland, Singapore, Slovakia, Slovenia, South Africa, Thailand, Taipei (China), Czech Republic, United States of America, Vietnam, People's Republic of China.

Accreditation certificate and annex are not to be disseminated in any form other than the present one. The publication of extracts is subject to approval from the accreditation body.
The impression shall not be given that the inspection of the testing laboratory also extends to products and services of the certificate holder which are not covered by this accreditation. If such an impression is given, the accreditation body is entitled to demand that changes be made.

16.1.11 Publications based on this study

Publications based on this study

Not applicable.

16.1.12 Important publications referenced in the study

BIOMETRICS 57, 963–971
September 2001

CONSULTANT'S FORUM

On Small-Sample Confidence Intervals for Parameters in Discrete Distributions

Alan Agresti* and Yongyi Min

Department of Statistics, University of Florida,
Gainesville, Florida 32611-8545, U.S.A.
* *email:* aa@stat.ufl.edu

SUMMARY. The traditional definition of a confidence interval requires the coverage probability at any value of the parameter to be at least the nominal confidence level. In constructing such intervals for parameters in discrete distributions, less conservative behavior results from inverting a single two-sided test than inverting two separate one-sided tests of half the nominal level each. We illustrate for a variety of discrete problems, including interval estimation of a binomial parameter, the difference and the ratio of two binomial parameters for independent samples, and the odds ratio.

KEY WORDS: Binomial distribution; Clopper–Pearson interval; Difference of proportions; Exact test; Odds ratio; Proportion; Relative risk; Tail method.

1. Introduction

Let T be a discrete statistic with probability mass function $f(t;\theta)$ and cumulative distribution function $F(t;\theta)$ indexed by a parameter θ. Some applications, especially in legal or regulatory environments, require interval estimators for θ to guarantee coverage probability of at least $1-\alpha$, for some fixed α, for all θ. Such methodology is also useful with small samples when one is unwilling to trust the uncertain performance of a large-sample approximation.

The usual approach inverts a family of tests having size at most α. For such a test, for each value θ_0 of θ, let $A(\theta_0)$ denote the acceptance region for testing H_0: $\theta = \theta_0$. Then for each value t of T, let $C(t) = \{\theta_0 : t \in A(\theta_0)\}$. This is a confidence region with the desired property. For a typical θ_0, $A(\theta_0)$ does not achieve probability of Type I error exactly equal to α because of discreteness. Hence, such confidence intervals are conservative. The actual coverage probability varies for different values of θ but exceeds $1-\alpha$ (Neyman, 1935) unless one artificially transforms T to a continuous variable using supplementary randomization (e.g., Anscombe, 1948; Stevens, 1950). These confidence intervals and the related significance tests are often referred to as exact because they use the true null distribution of T rather than an approximation based on large-sample normality. However, the actual coverage probability is not exact but only guaranteed to be bounded below by the nominal confidence level.

The approach to constructing such an interval commonly presented in theory of statistics texts inverts two separate one-sided tests each having size at most $\alpha/2$. For instance, if $F(t;\theta)$ is a decreasing function of θ for each t, the interval (θ_L, θ_U) is defined by the equations

$$P(T \leq t_o; \theta_U) = \alpha/2, \qquad P(T \geq t_o; \theta_L) = \alpha/2, \qquad (1)$$

where t_o is the observed value of T. This method is often called the tail method. When T is continuous, method (1) yields coverage probability $1-\alpha$ at all θ, but when T is discrete, $1-\alpha$ is a lower bound. The latter behavior results from the distribution of $F(T;\theta)$ being stochastically larger than uniform when T is discrete (Casella and Berger, 1990, p. 421).

This article shows that, for constructing confidence intervals with discrete distributions, it is usually better to invert a single two-sided test than to invert two separate one-sided tests. Here, "better" means that intervals tend to be shorter and coverage probabilities tend to be closer to the nominal level. We first discuss potential disadvantages of the tail method. Using particular examples of coverage probability graphs, we then illustrate the advantages of basing a confidence interval on inversion of a two-sided test. We use such tests with two-sided P-values that order the sample space (1) by null probabilities or (2) by null tail probabilities or (3) by a criterion measuring distance from the null, such as a score statistic. We show examples with the binomial parameter, the difference and the ratio of two binomial parameters for independent samples, and the odds ratio.

2. Inverting Two One-Sided Tests Versus Inverting a Two-Sided Test

In constructing a confidence interval by inverting a test, forming acceptance regions such that

$$P_{\theta_0}[T \in A(\theta_0)] \geq 1 - \alpha$$

for all θ_0 guarantees that the confidence level is at least the nominal level. Inverting the family of tests corresponds to forming the confidence region from the set of θ_0 for which the test's P-value exceeds α. The tail method (1) requires that the probability be no greater than $\alpha/2$ that T falls below $A(\theta_0)$ and no greater than $\alpha/2$ that T falls above $A(\theta_0)$. The interval is then the set of θ_0 for which each one-sided P-value exceeds $\alpha/2$. Equivalently, it corresponds to forming the confidence region from the set of θ_0 for which an overall P-value defined as $P = 2 \times \min[P_{\theta_0}(T \geq t_o), P_{\theta_0}(T \leq t_o)]$ exceeds α (taking $P = 1.0$ if this exceeds 1.0).

A disadvantage of the tail method is that, for sufficiently small and sufficiently large θ, the lower bound on the coverage probability is actually $1 - \alpha/2$ rather than $1 - \alpha$. For sufficiently small θ, e.g., the interval can never exclude θ by falling below it. To illustrate, suppose T has the geometric distribution $f(t;\theta) = (1-\theta)\theta^t, t = 0, 1, 2, \ldots$. Then $F(t;\theta) = 1 - \theta^{t+1}$, and using (1) yields the tail interval $((\alpha/2)^{1/t_o}, (1-\alpha/2)^{1/(t_o+1)})$. All θ between 0 and $1 - \alpha/2$ never fall above a confidence interval, and the coverage probability exceeds $1 - \alpha/2$ over this region.

To construct a confidence region using a single two-sided test, one approach enters the test statistic values t in $A(\theta_0)$ in order of their null probabilities, starting with the highest, stopping when the total probability is at least $1-\alpha$, i.e., $A(\theta_0)$ contains the smallest possible number of most likely outcomes (under $\theta = \theta_0$). This leads to optimality in terms of minimizing total length (Sterne, 1954; Crow, 1956). The intervals also satisfy a nestedness property, an interval with larger nominal confidence level necessarily containing one with a smaller nominal level. A slight complication is the lack of a unique way of forming $A(\theta_0)$ in many cases. In its crudest partitioning of the sample space, it corresponds to using the P-value

$$P_{\theta_0}[f(T;\theta_0) \leq f(t_o;\theta_0)], \qquad (2)$$

the sum of null probabilities of outcomes no more likely than the observed outcome. The confidence interval is the set of θ_0 for which

$$P_{\theta_0}[f(T;\theta_0) \leq f(t_o;\theta_0)] > \alpha. \qquad (3)$$

An endpoint θ_U (or θ_L) of this interval then satisfies

$$P_{\theta_U}[f(T;\theta_U) \leq f(t_o;\theta_U)] = \alpha.$$

In a related approach, Blaker (2000) defined

$$\gamma(t,\theta) = \min[P_\theta(T \geq t), P_\theta(T \leq t)]$$

and suggested forming the confidence interval as the set of θ_0 for which

$$P_{\theta_0}[\gamma(T,\theta_0) \leq \gamma(t_o,\theta_0)] > \alpha. \qquad (4)$$

This corresponds to using a P-value that equals $\min[P_{\theta_0}(T \geq t_o), P_{\theta_0}(T \leq t_o)]$ plus an attainable probability in the other tail that is as close as possible to, but not greater than, that one-tailed probability. Blaker showed that such intervals also have the nestedness property but may not have length optimality. The P-value for this approach is necessarily no greater than the P-value for the tail method, which uses $2\min[P_{\theta_0}(T \geq t_o), P_{\theta_0}(T \leq t_o)]$. Thus, the intervals based on (4) have the important advantage of necessarily being contained in intervals obtained with the tail method.

A third two-sided approach orders the sample space according to the distance of t_0 from H_0. One forms P-values according to a statistic that describes this distance, such as a score or likelihood-ratio statistic. To reduce conservativeness, it is preferable to use a statistic that tends to be less discrete, as discussed in Sections 4 and 5.

These two-sided approaches usually provide sensible results. The confidence regions do not have the tail method disadvantage of a lower bound of $1-\alpha/2$ for the coverage probability over part of the parameter space. However, anomalies can occur. For instance, a confidence region based on two-sided tests is not guaranteed to be an interval because the endpoints of the acceptance region need not be monotone in θ_0. Casella and Berger (1990, p. 417) and Santner and Duffy (1989, p. 37) discussed this in the context of the binomial parameter. For the two-sided method (3) based on ordered null probabilities, an endpoint from inverting a two-sided test with nominal confidence level of $1 - \alpha$ can be identical to an endpoint from the tail method with nominal confidence level of $1-2\alpha$. In the geometric case, e.g., because of the monotone decrease in the probabilities, this method yields $[\alpha^{1/t_o}, 1]$. More generally, suppose θ_L is such that

$$P_{\theta_L}[f(T;\theta_L) \leq f(t_o;\theta_L)] = \sum_{t \geq t_o} f(t;\theta_L) = \alpha.$$

Then θ_L is the lower endpoint from two-sided approach (3) with nominal confidence level $1 - \alpha$ and the lower endpoint using one-sided approach (1) with nominal level $1 - 2\alpha$. This happens when $f(t;\theta_L)$ is monotone decreasing in t, the geometric distribution being an extreme example in which this occurs for all θ.

Unfortunately, no single method for constructing confidence regions with discrete distributions can have optimality simultaneously in length, necessarily yielding an interval, and nestedness (Blaker, 2000). For the cases discussed below, similar results occurred from inverting an exact test using method (3) with P-value based on ordered null probabilities, method (4) with P-value based on two-tail probabilities, or the method based on P-value for the score statistic. The latter two methods may yield slightly wider intervals than method (3) based on ordered null probabilities, but in our experience, they have fewer anomalous cases.

3. Confidence Intervals for a Binomial Parameter

Let T be a binomial variate for n trials with parameter π, denoted bin(n, π). The tail method (1) gives the most commonly cited exact confidence interval, the Clopper–Pearson interval (Clopper and Pearson, 1934). The endpoints satisfy

$$\sum_{k=t_o}^{n} \binom{n}{k} \pi_L^k (1-\pi_L)^{n-k} = \alpha/2$$

and

$$\sum_{k=0}^{t_o} \binom{n}{k} \pi_U^k (1-\pi_U)^{n-k} = \alpha/2$$

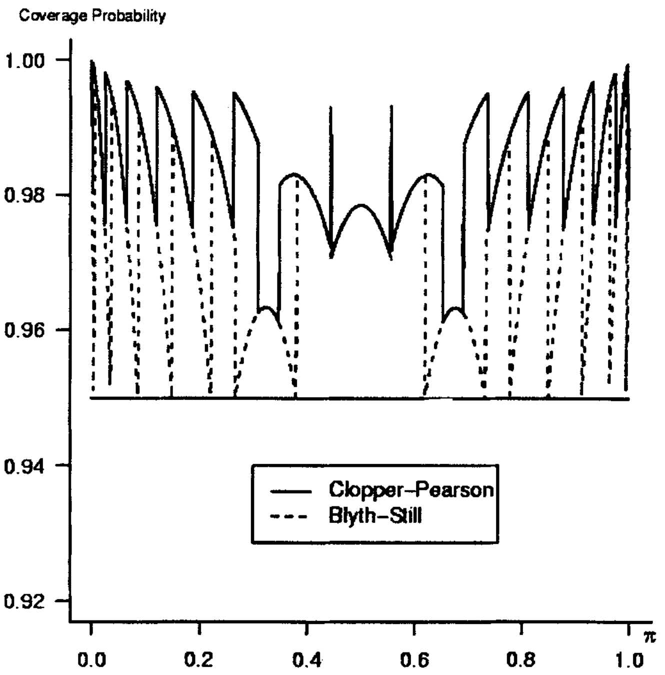

Figure 1. Coverage probabilities for 95% confidence intervals for a binomial parameter π with $n = 10$.

except that $\pi_L = 0$ when $t_o = 0$ and $\pi_U = 1$ when $t_o = n$. When $t_o = 0$, this confidence interval is $[0, 1 - (\alpha/2)^{1/n}]$. The actual coverage probability necessarily exceeds $1 - \alpha/2$ for π below $1 - (\alpha/2)^{1/n}$ and above $(\alpha/2)^{1/n}$. This is the entire parameter space when $n \leq \log(\alpha/2)/\log(0.5)$, e.g., $n \leq 5$ for $\alpha = .05$.

Sterne (1954) proposed method (3) of inverting a single test with outcomes ordered by their null probabilities. Blyth and Still (1983) and Casella (1986) amended this method slightly so that the confidence region cannot contain unconnected intervals and so natural symmetry and invariance properties are satisfied. The Blaker (2000) two-tailed probability approach (4) yields similar intervals that, unlike the Blyth–Still–Casella intervals, necessarily have the nestedness property with confidence levels, are contained within the Clopper–Pearson intervals, and are relatively simple to compute (Blaker's article contains short S-plus functions for doing this). The Blyth–Still interval is available in StatXact 4 (Cytel, 1999), the software having greatest scope for small-sample inference in discrete problems.

Figure 1 illustrates the superiority of forming the confidence interval by inverting a single two-sided test. It shows the actual coverage probabilities of the Clopper–Pearson and Blyth–Still intervals for nominal 95% confidence intervals, plotted as a function of π, when $n = 10$. Table 1 shows the 11 confidence intervals for each method. When $n = 10$, the ratio of expected lengths of the Blyth–Still and Clopper–Pearson intervals varies between 0.865 and .954, with a mean of 0.935. For comparison, Table 1 also shows the intervals using Blaker's (2000) two-sided approach (4) and by inverting the exact test using the score statistic. These are similar to the Blyth–Still intervals.

4. Confidence Intervals for Odds Ratio

When there are nuisance parameters, construction of an interval is more complicated. For exact inference with contingency tables, historically, the most popular approach is the conditional one that eliminates nuisance parameters by conditioning on their sufficient statistics.

For instance, consider inference for the odds ratio θ in a 2×2 contingency table. Assuming a multinomial distribution for the cell counts $\{n_{ij}\}$ or assuming $\{n_{ij}\}$ are independent Poisson or assuming the rows or the columns are independent binomials, conditioning on row and column marginal totals yields the hypergeometric distribution

$$P(n_{11} = t \mid \{n_{i+}\}, \{n_{+j}\}; \theta) = \frac{\binom{n_{1+}}{t}\binom{n - n_{1+}}{n_{+1} - t}\theta^t}{\sum_s \binom{n_{1+}}{s}\binom{n - n_{1+}}{n_{+1} - s}\theta^s}.$$

For this problem, Cornfield (1956) suggested the tail approach (1). This is the most commonly used exact method in practice (in fact, it is the only option in StatXact).

In forming a confidence interval for θ, Baptista and Pike (1977) adapted the approach (3) of inverting a single test based on ordered null probabilities. Table 2 shows this and the tail interval when $n = 20$ and each marginal count is 10. Figure 2 plots coverage probabilities for $\log(\theta)$ for the two approaches. Again, inverting a single test gives much better

Table 1
Nominal 95% confidence intervals for a binomial proportion with t successes in n = 10 trials

	Clopper–Pearson interval		Blyth–Still interval		Blaker interval		Score-test interval	
t	Lower	Upper	Lower	Upper	Lower	Upper	Lower	Upper
0	0.000	0.308	0.000	0.267	0.000	0.283	0.000	0.300
1	0.002	0.445	0.005	0.444	0.005	0.444	0.005	0.450
2	0.025	0.556	0.037	0.556	0.037	0.556	0.037	0.550
3	0.067	0.652	0.087	0.619	0.087	0.619	0.087	0.619
4	0.122	0.738	0.150	0.733	0.150	0.717	0.150	0.700
5	0.187	0.813	0.222	0.778	0.222	0.778	0.222	0.778

Note: Blyth–Still intervals obtained using StatXact. For count $6 \leq t \leq 10$, limits equal $(1 - \theta_U, 1 - \theta_L)$ for limits given for $10 - t$.

Table 2
Nominal 95% confidence intervals for odds ratio with count n_{11} when each row and column marginal total is 10

	Cornfield conditional interval		Invert two-sided conditional test		Invert two-sided conditional score test		Mid-P adapted Cornfield	
t	Lower	Upper	Lower	Upper	Lower	Upper	Lower	Upper
0	0.000	0.090	0.000	0.069	0.000	0.066	0.000	0.064
1	0.0003	0.309	0.0005	0.296	0.0005	0.290	0.0005	0.235
2	0.004	0.764	0.006	0.676	0.006	0.669	0.006	0.600
3	0.018	1.683	0.025	1.480	0.025	1.494	0.024	1.340
4	0.052	3.605	0.069	3.380	0.063	3.449	0.068	2.870
5	0.126	7.942	0.158	6.350	0.157	6.350	0.160	6.253

Note: For count $6 \leq n_{11} \leq 10$, limits equal $(1/\theta_U, 1/\theta_L)$ for limits given for $10 - n_{11}$.

results. For $\log(\theta)$ between zero and four, we computed the expected lengths for the two methods, conditional on $0 < n_{11} < 10$ (with $n_{1+} = n_{+1} = 10$) so the interval width is finite. On the log scale, their ratio varies between 0.894 and 0.905, with a mean of 0.899. Similar results occur by inverting the test using the exact conditional distribution but with the score statistic (Cornfield, 1956) or inverting the test using Blaker's two-tailed P-value. Table 2 also shows the score-based intervals.

A related problem is constructing a confidence interval for an odds ratio that is assumed constant in a set of 2×2 tables. Gart (1970) described the tail interval of form (1). For computing and software, see Mehta, Patel, and Gray (1985), Vollset, Hirji, and Elashoff (1991), and StatXact. For examples of the advantage of instead inverting a single two-sided test, see Kim and Agresti (1995), who used approach (3) with ordered null probabilities. When possibly many points in the sample space have the same value of the test statistic, they showed one can reduce the conservativeness by using the null probability to form a finer partitioning within fixed values of the test statistic. For instance, to illustrate the tail method, Gart gave a 95% confidence interval of (0.05, 1.16) for a $2 \times 2 \times 18$ table. Inverting the two-sided test, the Kim and Agresti interval yields (0.06, 1.14), and it reduces further to (0.09, 0.99) with a more finely partitioned P-value.

5. Confidence Intervals for Difference of Proportions

Next consider the difference of proportions for two independent binomial samples, where X_1 is bin(n_1, π_1), X_2 is bin(n_2, π_2), and $\hat{\pi}_i = X_i/n_i$. The joint probability mass function can be expressed in terms of $\theta = \pi_1 - \pi_2$ and a nuisance parameter such as π_1 or π_2 or $(\pi_1 + \pi_2)/2$, e.g.,

$$f(x_1, x_2; n_1, n_2, \theta, \pi_2) = \binom{n_1}{x_1}(\theta + \pi_2)^{x_1}(1 - \theta - \pi_2)^{n_1 - x_1} \times \binom{n_2}{x_2}\pi_2^{x_2}(1 - \pi_2)^{n_2 - x_2}.$$

The conditional approach for eliminating the nuisance parameter π_2 does not apply here since the canonical parameter is the difference of logits rather than the difference of proportions. One can eliminate π_2 using the unconditional approach of maximizing the P-value over its possible values. For instance, with a direction-sensitive statistic T for testing H_0: $\theta = \theta_0$, one-sided P-values for H_a: $\theta > \theta_0$ and H_a: $\theta < \theta_0$ are

$$P_U(\theta_0) = \sup_{\pi_2} P[T \geq t_0; \theta_0, \pi_2],$$
$$P_L(\theta_0) = \sup_{\pi_2} P[T \leq t_0; \theta_0, \pi_2], \quad (5)$$

where the supremum is taken over the permissible π_2 for the fixed θ_0.

For the tail method, the confidence interval satisfies $\theta_L = \sup\{\theta_0 : P_U(\theta_0) > \alpha/2\}$ and $\theta_U = \inf\{\theta_0 : P_L(\theta_0) > \alpha/2\}$. Santner and Snell (1980) proposed this interval using $T = \hat{\pi}_1 - \hat{\pi}_2$. Soms (1989a,b) discussed and programmed their interval and one inverting the Wald statistic. StatXact 4 provides the Santner and Snell interval. Santner and Snell (1980) also discussed the approach of inverting tests based on ordered null probabilities. They noted that it usually gave shorter in-

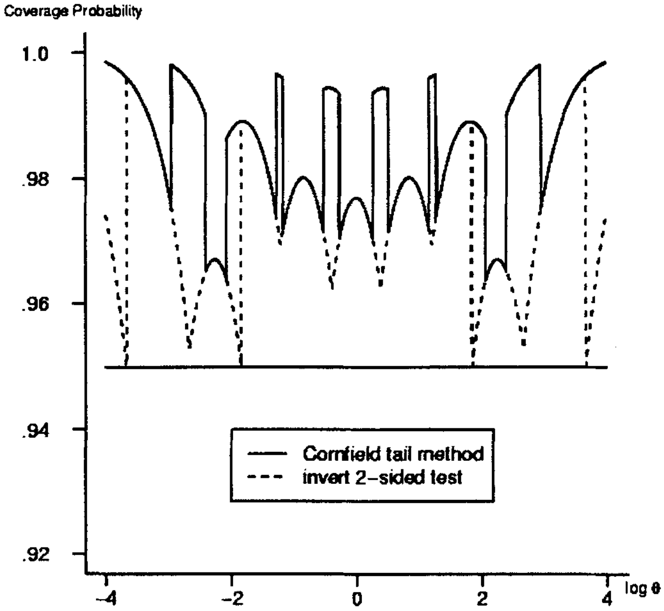

Figure 2. Coverage probabilities for 95% confidence intervals for $\pi_1 - \pi_2$ based on independent binomials, with $n_1 = n_2 = 10$.

Table 3
Nominal 95% confidence intervals for difference of proportions with binomial outcomes x_1 and x_2 in $n_1 = n_2 = 10$ independent trials

		Santner–Snell Interval		Chan–Zhang Interval		Invert two-sided Score test	
x_1	x_2	Lower	Upper	Lower	Upper	Lower	Upper
5	0	0.014	0.829	0.118	0.813	0.132	0.778
5	1	−0.089	0.764	−0.020	0.741	−0.001	0.700
5	2	−0.188	0.695	−0.146	0.671	−0.142	0.646
5	3	−0.283	0.620	−0.256	0.601	−0.249	0.560
5	4	−0.373	0.542	−0.369	0.539	−0.349	0.507
5	5	−0.459	0.459	−0.456	0.456	−0.419	0.419
2	0	−0.283	0.620	−0.129	0.556	−0.132	0.525
2	1	−0.373	0.542	−0.280	0.464	−0.265	0.441
2	2	−0.459	0.459	−0.386	0.386	−0.377	0.377
2	3	−0.542	0.373	−0.490	0.309	−0.455	0.296
2	4	−0.620	0.283	−0.585	0.229	−0.551	0.224

tervals and was preferable but was computationally infeasible (in 1980) except for very small n_i.

Chan and Zhang (1999) showed that conservativeness of the Santner and Snell tail method was exacerbated by the severe discreteness of $T = \hat{\pi}_1 - \hat{\pi}_2$ for small samples. For that application of the tail method, each sample with the same value of $\hat{\pi}_1 - \hat{\pi}_2$ has the same interval (for the given sample sizes). Chan and Zhang showed that better performance results from using a less discrete statistic, such as the score statistic (Mee, 1984; Miettinen and Nurminen, 1985)

$$T = \frac{(\hat{\pi}_1 - \hat{\pi}_2) - \theta_0}{\sqrt{\tilde{\pi}_1(1-\tilde{\pi}_1)/n_1 + \tilde{\pi}_2(1-\tilde{\pi}_2)/n_2}},$$

where $\tilde{\pi}_1$ and $\tilde{\pi}_2$ denote the maximum likelihood estimates of π_1 and π_2 subject to $\pi_1 - \pi_2 = \theta_0$. However, Chan and Zhang (1999) used only the tail method for this statistic. Better performance yet results from using the score statistic with a single two-sided test, in which the P-value compares $|T|$ to $|t_o|$.

Table 3 shows some intervals for the Santner–Snell and Chan–Zhang tail methods and for the two-sided inversion using the score statistic for various (x_1, x_2) values with $n_1 = n_2 = 10$. Similar improvements occurred from inverting a single likelihood-ratio test. In implementing the two-sided inversion, first we adapt the bisection method used in a FORTRAN program by Chan and Zhang (1999) to search for the lower and upper limits. Because of the theoretical possibility that disjoint intervals may exist, we supplement results from the bisection method with an intensive random search over the parameter space complement to that part represented by the bisection-based interval. This random search applies relatively greater weight to parameter points near those at the boundary obtained with the bisection method in order to enhance the chance of finding additionally needed points. In the rare cases

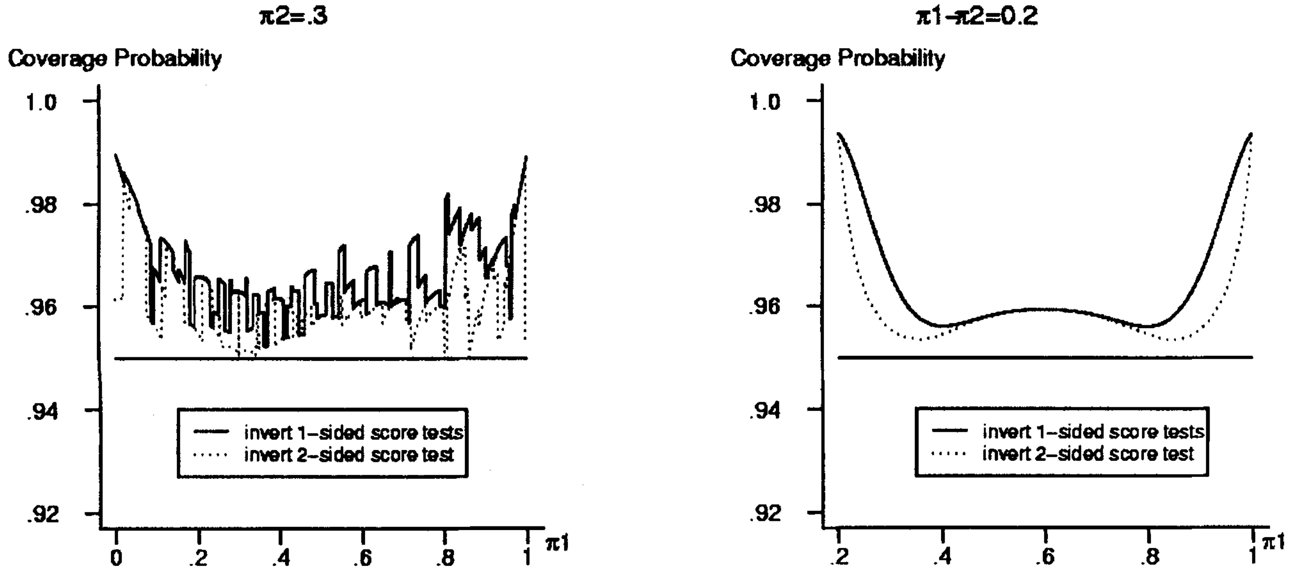

Figure 3. Coverage probabilities for 95% confidence intervals for $\pi_1 - \pi_2$ based on independent binomials, with $n_1 = n_2 = 10$.

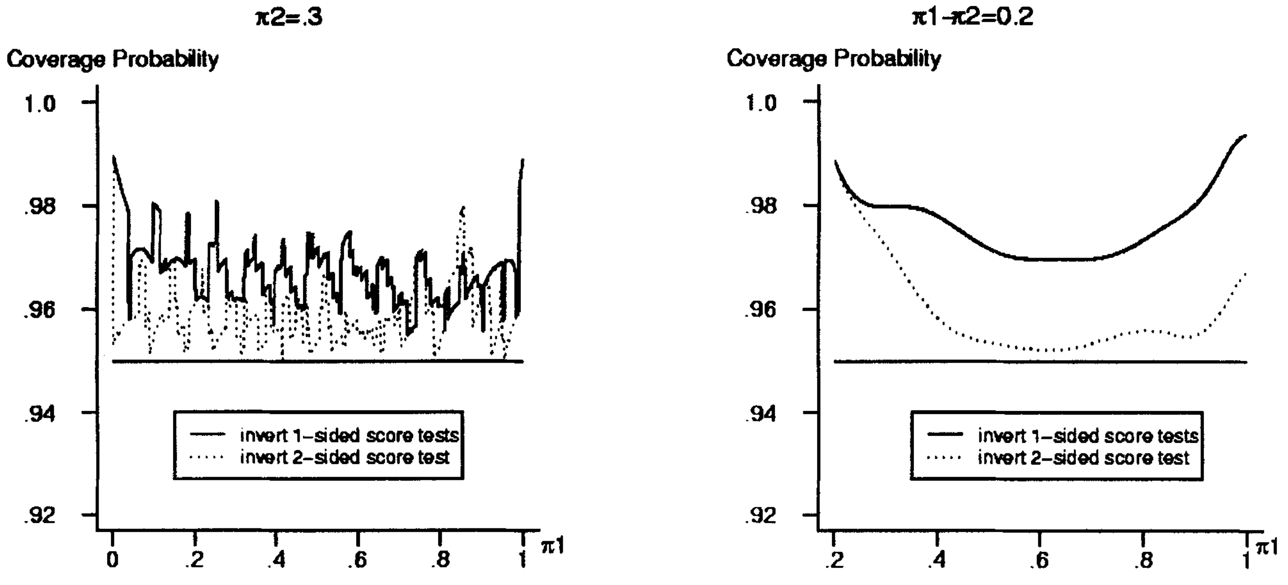

Figure 4. Coverage probabilities for 95% confidence intervals for $\pi_1 - \pi_2$ based on independent binomials, with $n_1 = 20$, $n_2 = 10$.

that this process determines the existence of disjoint intervals, we use the interval for $\pi_1 - \pi_2$ from the lowest lower bound to the highest upper bound. Testing of our algorithm showed accuracy to the fourth decimal place. As a check, in cases studied, we obtained confidence limits for all possible sample outcomes with the given sample sizes and evaluated and plotted coverage probability curves to analyze whether the nominal confidence level uniformly provided a lower bound; we did not observe any violations.

Figure 3 illustrates performance, plotting the coverage probability for the Chan–Zhang tail method and the two-sided score test approach as a function of π_1. The first panel in Figure 3 holds $\pi_2 = 0.3$ fixed and the second panel holds $\pi_1 - \pi_2 = 0.2$ fixed. When $\pi_1 - \pi_2 = 0.2$, the ratio of expected length varies between 0.937 and 0.948, with a mean of 0.945, as π_1 varies between 0.2 and 1. Even greater differences in coverage probability curves can occur with unbalanced sample sizes. Figure 4 illustrates this, making the same comparisons but with $n_1 = 20$ and $n_2 = 10$. Both methods tend to be very conservative when both parameters are near zero or one.

An alternative way to invert unconditional tests uses the Berger and Boos (1994) method of eliminating the nuisance parameter. That method takes the supremum in (5) over a high confidence region (e.g., 99.9%) for the nuisance parameter and adjusts the P-value (e.g., by adding 0.001) so that the overall nominal size is not exceeded. StatXact has the option of adapting the Santner–Snell interval in this manner. We considered it for the two-sided score interval, but this did not provide improved performance over the score interval based on taking the supremum over the entire space for the nuisance parameter. Likewise, Chan and Zhang (1999) noted that using this sort of restricted search over values of the nuisance parameter did not improve performance of the interval based on the tail method with score tests.

Interestingly, Coe and Tamhane (1993) and Santner and Yamagami (1993) also dealt with the problem of interval estimation of $\pi_1 - \pi_2$ with a generalized version of approach (3) based on ordered null probabilities. Surprisingly, their methods have not received much attention in the subsequent literature or in statistical practice. These methods also provide intervals with better coverage properties than the Santner and Snell (1980) or Chan and Zhang (1999) tail-method intervals. The Coe–Tamhane and Santner–Yamagami methods used different approaches in constructing the acceptance regions. The result is that the Coe–Tamhane intervals tend to be shorter for small to moderate $|\hat{p}_1 - \hat{p}_2|$ whereas the Santner–Yamagami intervals tend to be shorter for large $|\hat{p}_1 - \hat{p}_2|$. Lee, Serachitopol, and Brown (1997) evaluated these and other intervals for $\pi_1 - \pi_2$ and showed that, over a broad range of cases, the Coe–Tamhane interval had the best performance. Coe (1998) provided a SAS macro for the Coe–Tamhane approach.

6. Confidence Intervals for Other Parameters

Similar results occur for other parameters in discrete data problems. For instance, the discussion of the previous section on the difference of two binomial parameters also applies to their ratio, the relative risk $\theta = \pi_1/\pi_2$. Again, an unconditional approach can eliminate the nuisance parameter in the test to be inverted. We illustrate by inverting tests using the score statistic (Koopman, 1984; Miettinen and Nurminen, 1985), which has good performance for large-sample confidence intervals (Gart and Nam, 1988). Figure 5 compares coverage probabilities of 95% confidence intervals based on the tail method and based on inverting a single two-sided score test when $n_1 = n_2 = 10$. One panel refers to $\pi_2 = 0.3$ and the other to $\theta = 2$. When $\theta = 2$, the ratio of expected widths on the log scale, conditional on $x_1 > 0$ and $x_2 > 0$, varies between 0.801 and 0.864, with a mean of 0.833, as π_1 varies between zero and one.

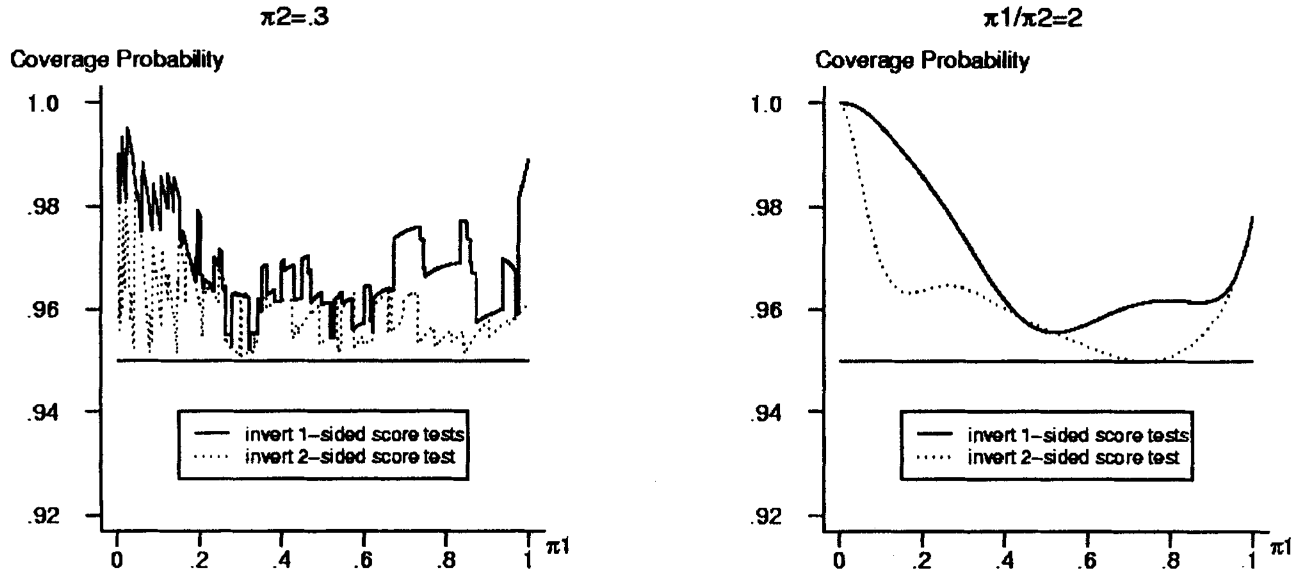

Figure 5. Coverage probabilities for 95% confidence intervals for π_1/π_2 based on independent binomials, with $n_1 = n_2 = 10$.

Another case analyzed with the tail method in most theory of statistics textbooks is the Poisson parameter. The tail method yields an interval specified with chi-squared percentage points (Garwood, 1936), but a plot in Casella and Berger (1990, p. 422) illustrates its conservativeness. Crow and Gardner (1959), Walton (1970), Casella and Robert (1989), Blaker (2000), and Kabaila and Byrne (2001) discussed improved intervals based on two-sided tests.

A class of parameters that includes the odds ratio is the set of parameters for logistic regression models. Cox (1970, p. 48) applied the tail method, using the conditional distribution to eliminate other parameters. Inverting a two-sided test has the benefits illustrated above for the odds ratio.

For independent binomial samples, we used the unconditional approach to obtain confidence intervals for the difference or ratio of proportions. In principle, one can also apply the unconditional approach to the odds ratio and more generally to logistic regression parameters even though the conditional approach is available. An open question is whether the unconditional two-sided approach may provide improvement over the conditional two-sided approach in some cases. The potential for improvement exists because of a reduction in discreteness. This is the case for testing equality of two independent binomials (e.g., Suissa and Shuster, 1985). However, there is also the potential for increased conservativism because of the approach of eliminating the nuisance parameter by taking a supremum of P-values (with respect to the nuisance parameter) instead of conditioning it out. Our preliminary study of this, for the odds ratio, suggests that the unconditional approach tends to provide intervals with coverage probabilities nearer the nominal levels. This will be addressed in detail in a future article.

7. A Limitation of the Two-Sided Approach

The past four sections showed examples of advantages of basing confidence intervals on two-sided tests. While the main theme of this article is recommending this over the tail method, a referee has suggested a qualification. In many studies, the goal is to show that a new treatment is better than a standard one. Such studies often use one-sided tests. Interval estimation is consistent with the test when the interval is based on inverting two one-sided tests (e.g., a 95% interval is then consistent with the result of the one-sided test with nominal size 0.025). Also, in some noninferiority trials, it is a regulatory requirement to use a confidence interval for the difference of proportions and compare one bound to a prespecified value. Again, using an interval based on inverting one-sided tests guarantees that the size of an implicit one-sided test does not exceed the nominal size.

A related comment is that users often are particularly interested in one of the bounds (say, the lower one) and interpret 95% intervals as imparting 97.5% confidence that the parameter falls above that bound. This inference is not appropriate with intervals based on inverting a two-sided test. Of course, in discrete cases, 97.5% is a lower bound and the actual confidence may be considerably higher than one prefers.

For such goals, one can argue in favor of simply calculating a one-sided confidence bound instead of a confidence interval. This may be a psychological barrier for many statisticians because most statistical texts discuss one-sided tests but few discuss one-sided confidence bounds.

8. Summary and Recommendations

In summary, discreteness has the effect of making exact confidence intervals more conservative than desired. We make the following recommendations for reducing the effects of that discreteness. First, apart from the caveat of the previous section, invert a two-sided test rather than two one-sided tests (the tail method). Second, in that test, use a test statistic that alleviates the discreteness (e.g., for comparing two proportions, use the score statistic rather than $\hat{\pi}_1 - \hat{\pi}_2$). Third, when appropriate, use an unconditional rather than conditional method of eliminating nuisance parameters.

A fourth recommendation is relevant if one is willing to relax slightly the requirement that the actual coverage probability have the nominal level as a lower bound at every parameter value. We then recommend inverting a two-sided exact test but using the mid P-value. The mid P-value has form $P(T > t_o) + (1/2)P(T = t_o)$, which is the expected value of the Stevens (1950) randomized P-value $P(T > t_o) + U \times P(T = t_o)$, where U is a uniform(0, 1) random variable, which achieves the nominal size. The mid P-value has null expected value of 0.5, and the sum of the two one-sided mid P-values equals 1.0. Evaluations for a variety of problems (e.g., Mehta and Walsh, 1992; Newcombe, 1998a) have shown that, although this method no longer guarantees coverage probabilities of at least the nominal level, it still tends to be somewhat conservative, although necessarily less so than using the ordinary P-value. An advantage over ordinary asymptotic methods is that it uses the exact distribution and provides an essentially exact method for moderate sample size since the difference between the mid P-value and ordinary exact P-value diminishes as the sample size increases and the discreteness in the tails diminishes. This recommendation is particularly relevant for the conditional approach, which has greater discreteness than the unconditional approach. Table 2 shows the confidence interval for the odds ratio that StatXact reports using the mid P adjustment of the Cornfield interval, which is a conditional one. Comparing this to the ordinary Cornfield interval in this table illustrates the shortening of intervals that can occur with the mid P method.

Related to this last point, we emphasize in closing that, except for the last paragraph, this article has discussed only confidence interval methods that attain at least the nominal confidence level. More generally, for three types of situations in which a statistician might select a method, we believe the preferred method differs. One situation is that dealt with in this article, in which one needs to guarantee a lower bound on the coverage probability. A second situation, more important for most statistical practice, is when one wants the actual coverage probability to be close to the nominal level but not necessarily to have it as a lower bound. A third situation is that of teaching basic statistical methods in a classroom or of consulting environment, for which one may be willing to sacrifice quality of performance somewhat in favor of simplicity.

For most statistical practice (i.e., situation two), for interval estimation of a proportion or a difference or ratio of proportions, the inversion of the asymptotic score test seems a good choice (e.g., Gart and Nam, 1988; Newcombe, 1998a,b). This tends to have an actual level fluctuating around the nominal level; if one prefers that level to be a bit more conservative, mid P adaptations of exact methods work well. For situations that require a bound on the error (i.e., situation one), basing conservative intervals on inverting the exact score test or the test using Blaker's (2000) two-tailed P-value has reasonable performance. For teaching (i.e., situation three), the Wald-type interval of point estimate plus and minus a normal-score multiple of a standard error is simplest. Unfortunately, this can perform poorly, but simple adjustments sometimes result in much improved performance, e.g., see Agresti and Caffo (2000).

Acknowledgements

This research was partially supported by grants from NIH and NSF. The authors thank George Casella, Cyrus Mehta, Thomas Santner, Roger Berger, and a referee for helpful comments.

Résumé

La définition traditionnelle d'un intervalle de confiance requiert de la probabilité de recouvrement de toute valeur du paramètre d'être au moins égale au niveau de confiance nominal. Pour des paramètres de distributions discrètes, on adopte un comportement moins conservatif en construisant de tels intervalles à partir d'une famille de tests bilatéraux plutôt qu'à partir de deux familles de tests unilatéraux séparés dont le niveau est la moitié du niveau nominal. Nous illustrons cela avec un certain nombre de problèmes discrets incluant l'estimation par intervalle d'un paramètre de binomiale, la différence et le rapport de deux paramètres de binomiales à partir d'échantillons indépendants, ainsi que le rapport de chances.

References

Agresti, A. and Caffo, B. (2000). Simple and effective confidence intervals for proportions and difference of proportions result from adding two successes and two failures. *The American Statistician* **54,** 280–288.

Anscombe, F. J. (1948). The validity of comparative experiments (with discussion). *Journal of the Royal Statistical Society, Series A* **111,** 181–211.

Baptista, J. and Pike, M. C. (1977). Exact two-sided confidence limits for the odds ratio in a 2 × 2 table. *Journal of the Royal Statistical Society, Series C* **26,** 214–220.

Berger, R. L. and Boos, D. D. (1994). *P* values maximized over a confidence set for the nuisance parameter. *Journal of the American Statistical Association* **89,** 1012–1016.

Blaker, H. (2000). Confidence curves and improved exact confidence intervals for discrete distributions. *Canadian Journal of Statistics* **28,** 783–798.

Blyth, C. R. and Still, H. A. (1983). Binomial confidence intervals. *Journal of the American Statistical Association* **78,** 108–116.

Casella, G. (1986). Refining binomial confidence intervals. *Canadian Journal of Statistics* **14,** 113–129.

Casella, G. and Berger, R. (1990). *Statistical Inference.* Pacific Grove, California: Wadsworth.

Casella, G. and Robert, C. (1989). Refining Poisson confidence intervals. *Canadian Journal of Statistics* **17,** 45–57.

Chan, I. S. F. and Zhang, Z. (1999). Test-based exact confidence intervals for the difference of two binomial proportions. *Biometrics* **55,** 1202–1209.

Clopper, C. J. and Pearson, E. S. (1934). The use of confidence or fiducial limits illustrated in the case of the binomial. *Biometrika* **26,** 404–413.

Coe, P. R. (1998). A SAS macro to calculate exact confidence intervals for the difference of two proportions. *Proceedings of the 23rd Annual SAS Users Group International Conference,* 1400–1405.

Coe, P. R. and Tamhane, A. C. (1993). Small sample confidence intervals for the difference, ratio and odds ratio of two success probabilities. *Communications in Statistics—Simulation and Computation* **22**, 925–938.

Cornfield, J. (1956). A statistical problem arising from retrospective studies. *Proceedings of the Third Berkeley Symposium on Mathematical Statistics and Probability,* **4**, 135–148.

Cox, D. R. (1970). *Analysis of Binary Data.* London: Chapman and Hall.

Crow, E. L. (1956). Confidence intervals for a proportion. *Biometrika* **43**, 423–435.

Crow, E. L. and Gardner, R. S. (1959). Confidence intervals for the expectation of a Poisson variable. *Biometrika* **46**, 441–447.

Cytel. (1999). *StatXact 4 for Windows.* Cytel Software: Cambridge, Massachusetts.

Gart, J. J. (1970). Point and interval estimation of the common odds ratio in the combination of 2×2 tables with fixed marginals. *Biometrika* **57**, 471–475.

Gart, J. J. and Nam, J. (1988). Approximate interval estimation of the ratio of binomial parameters: A review and corrections for skewness. *Biometrics* **44**, 323–338.

Garwood, F. (1936). Fiducial limits for the Poisson distribution. *Biometrika* **28**, 437–442.

Kabaila, P. and Byrne, J. (2001). Exact short Poisson confidence intervals. *Canadian Journal of Statistics* **29**, in press.

Kim, D. and Agresti, A. (1995). Improved exact inference about conditional association in three-way contingency tables. *Journal of the American Statistical Association* **90**, 632–639.

Koopman, P. A. R. (1984). Confidence intervals for the ratio of two binomial proportions. *Biometrics* **40**, 513–517.

Lee, J. J., Serachitopol, D. M., and Brown, B. W. (1997). Likelihood-weighted confidence intervals for the difference of two binomial proportions. *Biometrical Journal* **39**, 387–407.

Mee, R. W. (1984). Confidence bounds for the difference between two probabilities (letter). *Biometrics* **40**, 1175–1176.

Mehta, C. R. and Walsh, S. J. (1992). Comparison of exact, mid-p, and Mantel–Haenszel confidence intervals for the common odds ratio across several 2×2 contingency tables. *The American Statistician* **46**, 146–150.

Mehta, C. R., Patel, N. R., and Gray, R. (1985). Computing an exact confidence interval for the common odds ratio in several 2 by 2 contingency tables. *Journal of the American Statistical Association* **80**, 969–973.

Miettinen, O. and Nurminen, M. (1985). Comparative analysis of two rates. *Statistics in Medicine* **4**, 213–226.

Newcombe, R. (1998a). Two-sided confidence intervals for the single proportion: Comparison of seven methods. *Statistics in Medicine* **17**, 857–872.

Newcombe, R. (1998b). Interval estimation for the difference between independent proportions: Comparison of eleven methods. *Statistics in Medicine* **17**, 873–890.

Neyman, J. (1935). On the problem of confidence limits. *Annals of Mathematical Statistics* **6**, 111–116.

Santner, T. J. and Duffy, D. E. (1989). *The Statistical Analysis of Discrete Data.* Berlin: Springer-Verlag.

Santner, T. J. and Snell, M. K. (1980). Small-sample confidence intervals for $p_1 - p_2$ and p_1/p_2 in 2×2 contingency tables. *Journal of the American Statistical Association* **75**, 386–394.

Santner, T. J. and Yamagami, S. (1993). Invariant small sample confidence-intervals for the difference of 2 success probabilities. *Communications in Statistics—Simulation and Computation* **22**, 33–59.

Soms, A. P. (1989a). Exact confidence intervals, based on the Z statistic, for the difference between two proportions. *Communications in Statistics—Simulation and Computation* **18**, 1325–1341.

Soms, A. P. (1989b). Some recent results for exact confidence intervals for the difference between two proportions. *Communications in Statistics—Simulation and Computation* **18**, 1343–1357.

Sterne, T. E. (1954). Some remarks on confidence or fiducial limits. *Biometrika* **41**, 275–278.

Stevens, W. L. (1950). Fiducial limits of the parameter of a discontinuous distribution. *Biometrika* **37**, 117–129.

Suissa, S. and Shuster, J. J. (1985). Exact unconditional sample sizes for the 2 by 2 binomial trial. *Journal of the Royal Statistical Society, Series A* **148**, 317–327.

Vollset, S. E., Hirji, K. F., and Elashoff, R. M. (1991). Fast computation of exact confidence limits for the common odds ratio in a series of 2×2 tables. *Journal of the American Statistical Association* **86**, 404–409.

Walton, G. S. (1970). A note on nonrandomized Neyman-shortest unbiased confidence intervals for the binomial and Poisson parameters. *Biometrika* **57**, 223–224.

Received October 2000. *Revised January* 2001.
Accepted January 2001.

STATISTICS IN MEDICINE
Statist. Med. **17**, 1403–1413 (1998)

EXACT TESTS OF EQUIVALENCE AND EFFICACY WITH A NON-ZERO LOWER BOUND FOR COMPARATIVE STUDIES

IVAN S. F. CHAN*

Merck Research Laboratories, BL 3-2, West Point, PA 19486, U.S.A.

SUMMARY

Exact tests of equivalence and efficacy with a non-zero lower bound based on two independent binomial proportions for comparative trials are proposed. These exact tests are desirable for studies with small sample sizes. They generalize classical methods to include testing of null hypotheses of prespecified differences and can be used to demonstrate a new treatment's efficacy or its equivalence to a standard treatment. The proposed exact tests use unconditional distributions of the test statistics. Variances of test statistics are estimated via a constrained maximum likelihood method (Farrington and Manning). Data from oncology and vaccine clinical trials are used to illustrate the exact tests. © 1998 John Wiley & Sons, Ltd.

1. INTRODUCTION

Many clinical trials are designed to compare a new treatment to a standard treatment or a placebo control in terms of dichotomous endpoints. In vaccine trials, for example, one often assesses whether or not a subject develops certain levels of antibody responses, any systemic adverse experiences, or certain clinical symptoms of disease after receiving a vaccine. In oncology trials, one often assesses whether or not a treatment stops tumour development or induces certain toxicity in patients. Since endpoints are dichotomous, the comparison focuses on proportions of responses in the two groups. For example, Rodary *et al.*[1] reported a randomized trial in childhood nephroblastoma to show that a chemotherapy (new treatment) is equivalent to a radiation therapy (standard treatment) in terms of reducing tumour rupture rates. Fries *et al.*[2] conducted a viral challenge study to compare the incidence rates of influenza among subjects who received a new influenza vaccine and those subjects who received a placebo injection. We discuss these two examples in more detail later in Section 3. In this report we consider a binomial trial that compares two rates P_1 and P_2 from groups of size N_1 and N_2, respectively. In particular, we focus on tests of hypotheses regarding the difference $(P_1 - P_2)$ and the ratio P_2/P_1.

When the difference of proportions is the focus of comparison, a classical analysis is to test the null hypothesis of no difference between the two proportions. In some situations, however, the

* Correspondence to: Ivan S. F. Chan, Merck Research Laboratories, BL 3-2, West Point, PA 19486, U.S.A. E-mail: Ivan_Chan@Merck.Com

CCC 0277–6715/98/121403–11$17.50
© 1998 John Wiley & Sons, Ltd.
Received March 1997
Revised September 1997

primary interest is to demonstrate that the new treatment is equivalent (or not substantially inferior) to the standard treatment, instead of showing a difference between the two treatments. In fact, in assessing a new multicomponent combination vaccine where the individual component vaccines have been licensed, it is a regulatory requirement to show that each component in the combination vaccine induces adequate (or not substantially inferior) immune responses when compared to the monovalent vaccines.[3] Formulation of such hypotheses requires some generalizations in which the null hypothesis specifies that the difference between the two proportions is at least some prespecified small quantity and the alternative specifies that the difference is smaller than the prespecified quantity. This formulation leads to a one-sided equivalence hypothesis, intended to show that one treatment is not substantially inferior to the other. Suppose P_1 and P_2 represent true response rates of the standard and new treatments, respectively, then the test of equivalence (one-sided) focuses on the hypothesis:

$$H_0\colon P_1 - P_2 \geqslant \delta \text{ versus } H_1\colon P_1 - P_2 < \delta \tag{1}$$

where δ is a prespecified small quantity. This quantity is usually so chosen that a difference of δ is deemed clinically important. Rejection of the null hypothesis, which rules out the prespecified small difference δ, demonstrates that the new treatment is equivalent (or not substantially inferior) to the standard treatment. When $\delta = 0$, hypothesis (1) reduces to the classical one-sided hypothesis.

In vaccine efficacy studies where subjects are randomized to receive either the vaccine or a placebo injection, the response is defined as whether or not the subject develops the disease of interest during the study. If P_1 and P_2 represent the disease incidence rates among placebo controls and vaccinees, respectively, then the vaccine efficacy, denoted by π, is commonly measured by 1 – relative risk for the disease among the vaccinees:

$$\pi = 1 - P_2/P_1.$$

We assume that $P_2 \leqslant P_1$. The new vaccine is said to have 100 percent efficacy if $P_2 = 0$ ($\pi = 1$), and has no efficacy if $P_2 = P_1$ ($\pi = 0$). A test of efficacy focuses on the hypothesis

$$H_0\colon \pi \leqslant \pi_0 \text{ versus } H_1\colon \pi > \pi_0 \tag{2}$$

where π_0 denotes the minimal level of efficacy anticipated for the new vaccine. The value of π_0 could be 0 as is typically the case in establishing therapeutic efficacy of a drug. In designing vaccine efficacy trials, however, one often chooses a non-zero efficacy lower bound ($\pi_0 > 0$) to more precisely estimate the benefit of the vaccine and justify the risk of vaccinating healthy subjects. Hypothesis (2) relates directly to the hypothesis test on the ratio of two rates. Let $R = P_2/P_1$, then hypothesis (2) is equivalent to H_0: $R \geqslant 1 - \pi_0$ versus H_1: $R < 1 - \pi_0$.

Several studies[4–8] have considered these two types of statistical tests and proposed the following statistics:

$$Z_{\text{EQ}} = \frac{\hat{P}_1 - \hat{P}_2 - \delta}{\hat{\sigma}_{\text{EQ}}}, \qquad Z_{\text{EF}} = \frac{\hat{P}_2 - (1 - \pi_0)\hat{P}_1}{\hat{\sigma}_{\text{EF}}}$$

for test of equivalence (Z_{EQ}) with hypothesis (1) and for test of efficacy (Z_{EF}) with hypothesis (2), respectively. Here $\hat{P}_1$ and $\hat{P}_2$ are the observed proportions for the two groups, respectively. The $\hat{\sigma}_{\text{EQ}}$ and $\hat{\sigma}_{\text{EF}}$ are standard error estimates of the numerators under the null hypotheses (1) and (2), respectively. Small values of the test statistic favour the alternative hypothesis in both cases. When $\delta = 0$ and $\pi_0 = 0$, these two tests are equivalent and reduce to the classical one-sided Z-test. One can obtain the standard error estimates in three ways: (i) use the observed values

© 1998 John Wiley & Sons, Ltd.

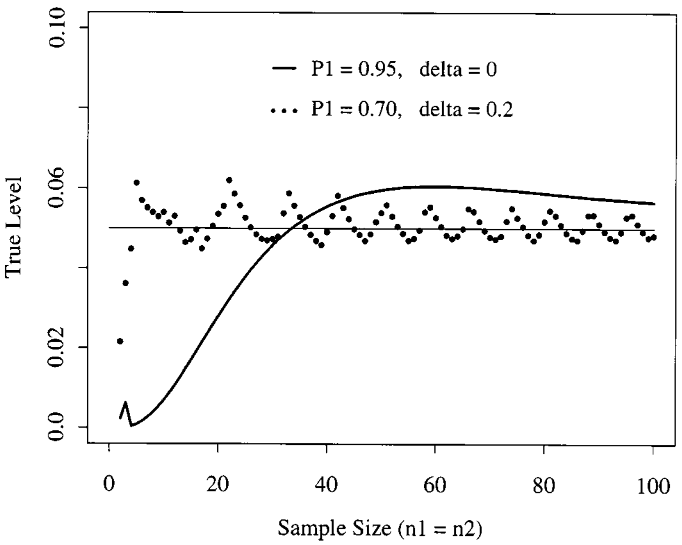

Figure 1. True levels of the test of equivalence at the 0·05 nominal level based on the normal approximation using the constrained ML estimate of the variance[4]

$\hat{P}_1$ and $\hat{P}_2$;[5] (ii) use estimates of P_1 and P_2 under the null hypothesis subject to the constraint of fixed marginal total of responses;[6] or (iii) use maximum likelihood (ML) estimates of P_1 and P_2 constrained under the null hypothesis.[4] It has been pointed out that the constrained ML method performs better in terms of confidence interval coverage.[4,8]

Both Z_{EQ} and Z_{EF} are asymptotically distributed as standard normal under null hypotheses (1) and (2), respectively. However, the performance of these tests is uncertain in small samples. To illustrate, let us look at the true levels of a 0·05 level test of equivalence hypothesis (1) based on the normal approximation using the constrained ML estimate of the variance.[4] We consider two hypothetical set-ups, ($P_1 = 0{\cdot}95$, $\delta = 0$) and ($P_1 = 0{\cdot}7$, $\delta = 0{\cdot}2$), and assume the two groups have equal sample sizes. We obtain the true level by enumerating all possible combinations of responses given the sample sizes N_1 and N_2, the true probability for group 1 (P_1), and the prespecified minimum difference (δ) between the two groups. Figure 1 plots the relationship of true levels and sample sizes for the equivalence test (Z_{EQ}) based on normal approximation. The graph suggests that the true type I error rate of the equivalence test (Z_{EQ}) may well exceed the nominal level and may approach the nominal level very slowly. In addition, the type I error rate may depend on the sample size in ways that are not predictable. Therefore, it is desirable to study the exact distribution of the test statistic in order to control the true level of the test. In Section 2 we present the likelihood of all possible outcomes and describe the exact test procedures. Section 3 discusses some illustrative examples including the nephroblastoma study and the influenza vaccine study introduced earlier. We give some discussion in Section 4.

2. LIKELIHOOD FOR EXACT TEST

In this report we consider exact tests based on Z_{EQ} and Z_{EF}. We estimate the standard errors using the constrained ML method. Details of the formulae for $\hat{\sigma}_{EQ}$ and $\hat{\sigma}_{EF}$ are given by

© 1998 John Wiley & Sons, Ltd.

Farrington and Manning.[4] It is worth noting that we could also construct exact tests based on numerators of Z_{EQ} and Z_{EF} only. However, there are more ties in the values of the numerator than in its standardized score. As a result, the distribution based on the numerator alone would be more discrete and the corresponding test would be less sensitive. Simulations by Storer and Kim[9] also confirmed that tests based on the numerator only were less powerful than tests based on its standardized value in the classical case of testing the null hypothesis of no difference.

We consider exact distributions of the test statistics (Z_{EQ} and Z_{EF}), which consist of all possible outcomes of the two binomial responses given the sample sizes N_1 and N_2. Let X and Y be the independent response variables distributed as binomial (N_1, P_1) and binomial (N_2, P_2), respectively. Then each outcome (X, Y) corresponds to a 2×2 table. Since X and Y are independent, the probability of observing a particular outcome (i, j) is the product of two individual binomial probabilities

$$\Pr(X = i, Y = j) = \binom{N_1}{i}\binom{N_2}{j} P_1^i (1 - P_1)^{N_1 - i} P_2^j (1 - P_2)^{N_2 - j}. \quad (3)$$

This likelihood can be rearranged under the null hypothesis for the test of equivalence and the test of efficacy. Let $P = P_2$, then we can write the null likelihood for the test of equivalence hypothesis (1) as

$$\Pr(X = i, Y = j \mid H_0) = \binom{N_1}{i}\binom{N_2}{j} (P + \delta)^i (1 - P - \delta)^{N_1 - i} P^j (1 - P)^{N_2 - j}. \quad (4)$$

Similarly, we can express the null likelihood for the test of efficacy hypothesis (2) as

$$\Pr(X = i, Y = j \mid H_0)$$
$$= \binom{N_1}{i}\binom{N_2}{j} (1 - \pi_0)^{-i} P^{i+j} (1 - (1 - \pi_0)^{-1} P)^{N_1 - i} (1 - P)^{N_2 - j}. \quad (5)$$

We base the exact inference on Z_{EQ} and Z_{EF} on likelihoods (4) and (5), respectively. The exact significance level is the sum of probabilities of tables that are at least as 'extreme' as the observed table. In this report, we will use the test statistics Z_{EQ} and Z_{EF} to order the tables. For hypothesis (1) or (2), small values of the test statistic favour the alternatives. Let Z be the test statistic of interest (Z_{EQ} or Z_{EF}), and let Z_{obs} be the Z statistic calculated for the observed table. Then the tail (or the as extreme region) of the observed table includes all tables for which $Z \leqslant Z_{obs}$. We note that ranking by the likelihood (see, for example, Agresti[10] and Zelterman *et al.*[11]) is difficult to implement because the likelihoods (4) and (5) depend on the unknown parameter P. In addition, the tables with smaller likelihoods do not necessarily favour the alternative hypothesis and vice versa.

Notice that the likelihoods (4) and (5) depend on P, which is an unknown nuisance parameter for the exact inference. In the special case of testing the null hypothesis of no difference ($\delta = 0$ or $\pi_0 = 0$), a simple sufficient statistic for P is the marginal total $T = X + Y$. We could then conduct exact inference by conditioning on T, in which case the test simplifies to Fisher's exact test.[12] However, no simple sufficient statistics for P exist for the general case where $\delta \neq 0$ or $\pi_0 \neq 0$. To eliminate the effect of this nuisance parameter P, we use the maximization method[13] which calculates the exact significance level by maximizing the null likelihood over the domain of the

© 1998 John Wiley & Sons, Ltd.

nuisance parameter P. The domain of P is $D = [0, 1-\delta]$ for the test of equivalence and $D = [0, 1 - \pi_0]$ for the test of efficacy. Specifically, we define the exact significance level as

$$pvalue = \max_{P \in D} \Pr(Z \leqslant Z_{\mathrm{obs}} \mid P).$$

Since the maximization method does not condition on a particular sufficient statistic and uses the unconditional distribution of the test statistic, this test is called an exact unconditional test. Suissa and Shuster[14] and Haber[15] studied the exact unconditional test of null hypotheses of no difference ($\delta = 0$) in 2×2 tables and showed that the maximization method increased the power when compared to Fisher's exact test. The proposed exact test of equivalence provides an extension to their work to include null hypotheses of a prespecified difference ($\delta \neq 0$).

In summary, we can calculate the exact p-value in the following steps:

1. Compute the Z statistic (Z_{EQ} or Z_{EF}) for all tables and order them. Let Z_{obs} be the Z statistic calculated at the observed table. The tail of the observed table includes those tables whose Z statistics are smaller than or equal to Z_{obs}.
2. For a given value of the nuisance parameter P, calculate the tail probability by summing up the probabilities of those tables in the tail using likelihood (4) or (5).
3. Repeat step 2 for every value of P in its domain. Then the exact p-value is the maximum of the tail probability over the domain of P. Since the domain of P is continuous, we suggest dividing the domain into a large number (say 1000) of equally spaced subintervals and calculation of the tail probability at every increment. This provides adequate accuracy for most practical uses.

For a nominal α level test, we reject the null hypothesis if the exact p-value is less than or equal to α. To obtain the true level of the exact test, we first convert the test procedure to find the critical value given the nominal α level and the sample sizes N_1 and N_2. This critical value does not depends on any specific value of the nuisance parameter, and the true level is the maximum (over the domain of the nuisance parameter) null probability of those tables of which the test statistics are less than or equal to the critical value. We have implemented the exact test procedures in FORTRAN programs.

3. EXAMPLES

Now let us revisit the two hypothetical examples of equivalence tests discussed in Section 1. Throughout we use 1000 uniform grid points over the nuisance parameter space to search for the maximum tail probability. We calculate the true levels of the exact equivalence test at the nominal 0·05 level for these two examples: ($P_1 = 0{\cdot}95$, $\delta = 0$) and ($P_1 = 0{\cdot}7$, $\delta = 0{\cdot}2$) under hypothesis (1). The true levels are plotted in Figures 2 and 3, respectively. By definition, the true level of the exact test never exceeds the nominal level. In addition, the true level approaches the nominal level as sample size increases. Tables I and II give more examples of true levels of the exact tests of equivalence and efficacy in comparison to the true levels of the corresponding tests based on the normal approximation. We observe that the exact tests are not too conservative in most cases. By construction, their true levels are obtained as the maximum over all possible values of the nuisanec parameter (or the incidence rate in Group 1). On the other hand, the true levels of the normal Z-tests are higher than the nominal level of 0·05 (5%) in many cases. The last column in

© 1998 John Wiley & Sons, Ltd.

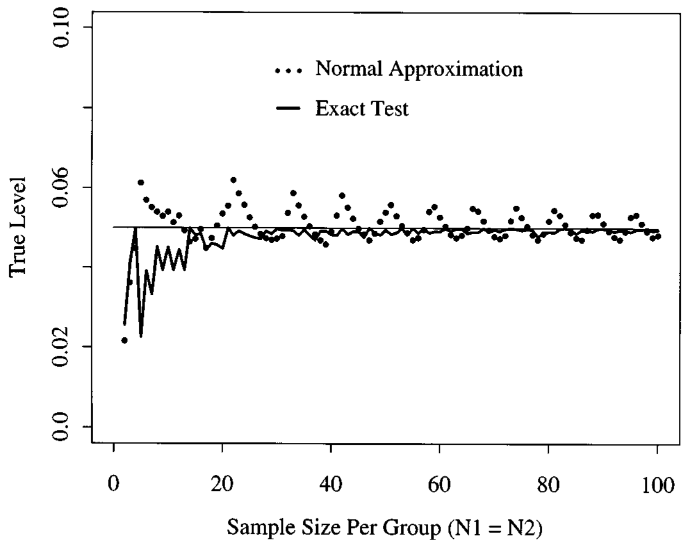

Figure 2. True levels of the test of equivalence at the 0·05 nominal level given $P_1 = 0{\cdot}7$ and $\delta = 0{\cdot}2$

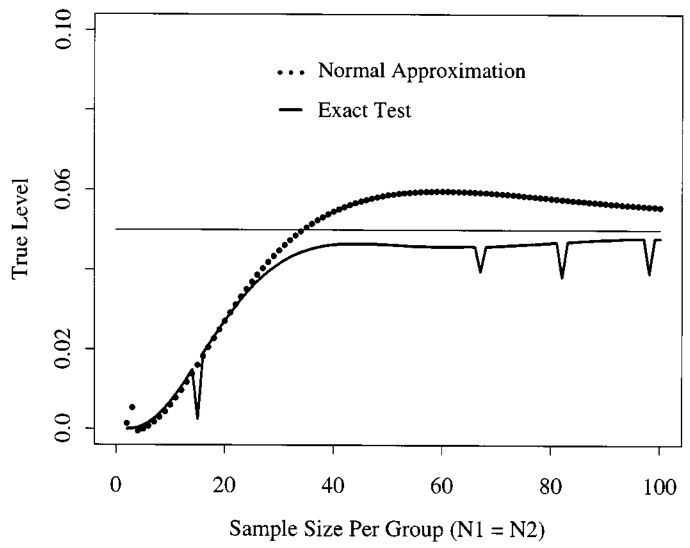

Figure 3. True levels of the test of equivalence at the 0·05 nominal level given $P_1 = 0{\cdot}95$ and $\delta = 0$

these two tables gives the maximum type I error rate of the normal Z-test over all possible values of the incidence rate in group 1 (P_1). The results show that the type I error rate of the normal Z-test may be substantially higher than the nominal level if the true rate in group 1 deviates from what one expects when designing the study.

© 1998 John Wiley & Sons, Ltd.

Table I. True levels of the exact test and the Z_{EQ} test via normal approximation performed at the nominal 5 percent level for the equivalence hypothesis $H_0: P_1 - P_2 \geqslant \delta$

Incidence rate in group 1 (P_1)	Minimum difference under $H_0(\delta)$	Sample size per group ($N_1 = N_2$)	Exact test true level (%)	Z_{EQ} test via normal approximation	
				True level (%)	Maximum level (%)
0·95	0·1	35	4·85	5·00	5·36
0·95	0·1	70	4·98	4·48	5·36
0·95	0·2	35	4·96	4·64	5·74
0·8	0·1	100	4·91	5·09	5·16
0·8	0·15	25	4·56	5·20	6·73
0·8	0·15	85	4·91	5·08	5·64
0·6	0·2	90	4·99	5·58	5·58
0·6	0·25	20	4·84	5·05	5·91
0·6	0·25	50	4·80	5·03	5·27

Note: The normal approximation uses the constrained ML estimate of the variance[4]

Table II. True levels of the exact test and the Z_{EF} test via normal approximation performed at the nominal 5 percent level for the efficacy hypothesis $H_0: \pi \leqslant \pi_0$

Incidence rate in control group 1 (P_1)	Efficacy lower bound (π_0)	Sample size per group ($N_1 = N_2$)	Exact test true level (%)	Z_{EF} test via normal approximation	
				True level (%)	Maximum level (%)
0·9	0	10	4·74	2·51	5·79
0·9	0·4	14	3·74	5·39	5·81
0·9	0·6	50	4·98	4·96	4·98
0·6	0	20	4·30	4·57	5·37
0·6	0·3	20	4·66	5·33	5·35
0·6	0·3	40	4·97	4·89	6·31
0·4	0	30	4·63	4·54	5·76
0·4	0·1	55	4·87	5·08	6·01
0·4	0·1	70	4·88	5·06	5·76

Note: The normal approximation uses the constrained ML estimate of the variance[4]

We now apply these exact tests to analyse the two medical examples introduced in Section 1. The goal of the childhood nephroblastoma study[1] was to demonstrate that a new pre-operative chemotherapy with less severe general disadvantages is equivalent to (or not inferior to) the standard radiation therapy in terms of reducing tumour rupture rates following nephrectomy. The equivalence criterion required that the difference in the proportion of subjects who did not develop tumour rupture between the radiation group (P_1) and the chemotherapy group (P_2) be less than 0·1. The corresponding hypothesis was $H_0: P_1 - P_2 \geqslant 0{\cdot}1$ versus $H_1: P_1 - P_2 < 0{\cdot}1$.

© 1998 John Wiley & Sons, Ltd.

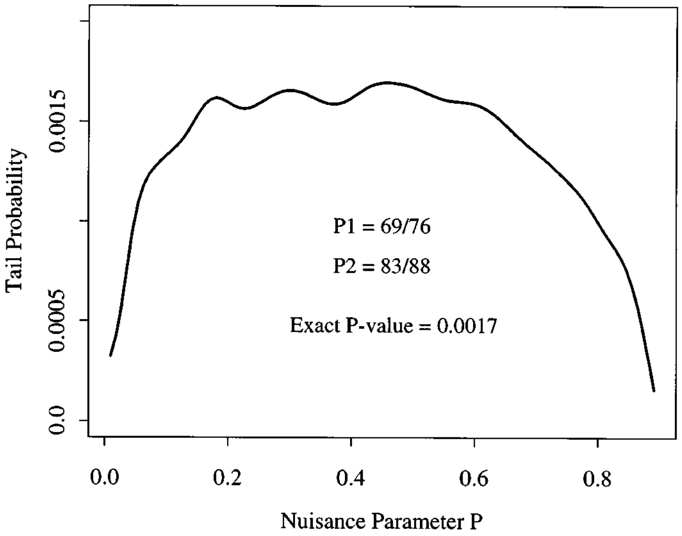

Figure 4. Tail probabilities of the exact test of efficacy for the childhood nephroblastoma study

A total of 164 patients were randomized in the study. Surprisingly the radiation group had a lower response ($\hat{P}_1 = 0{\cdot}9079[69/76]$) than the chemotherapy group ($\hat{P}_2 = 0{\cdot}9432[83/88]$), and the observed difference of $(P_1 - P_2)$ was $-0{\cdot}0353$. Rodary *et al.*[1] analysed these data using the method proposed by Dunnett and Gent[6] and concluded equivalence between the two treatments. As an illustrative example, we re-analyse the data using the Z_{EQ} test, which yields a statistic of $-2{\cdot}9595$ with a p-value of 0·0016 based on the normal approximation. The exact test based on the unconditional distribution of Z_{EQ} yields a p-value of 0·0017. Figure 4 plots the exact tail probabilities as a function of the nuisance parameter P for this example. The exact p-value, which is the maximum, occurs at $P = 0{\cdot}441$. These results provide very strong evidence for equivalence between the two treatments. They also suggest that the exact test could be as sensitive as the asymptotic test based on the normal approximation.

Fries *et al.*[2] reported an experiment to evaluate protective efficacy of a Recombinant protein Influenza A vaccine against wild-type H1N1 virus challenge. In this study subjects were randomized to receive either the experimental vaccine or the placebo injection, and they were subsequently challenged intranasally with influenza virus. All subjects were closely monitored in the next 9 days for any viral infection and subsequently any clinical symptoms that included fever, upper respiratory infection, recurrent cough or lower respiratory infection, and myalgias. The observed incidence rates of any clinical illness among subjects who had viral infection were $\hat{P}_1 = 0{\cdot}8000(12/15)$ in the placebo group and $\hat{P}_2 = 0{\cdot}4667(7/15)$ in the vaccine group. The observed protective efficacy was $\hat{\pi} = 0{\cdot}4167$. The Fisher's exact test yields a one-sided p-value of 0·0641, which gives some evidence of vaccine efficacy. On the other hand, the efficacy test for $H_0: \pi = 0$ versus $H_0: \pi > 0$ results in a test statistic (Z_{EF}) of $-1{\cdot}8943$ with a p-value of 0·0291 based on the normal approximation. The exact p-value is 0·0341, which indicates that the exact unconditional test is more sensitive than the Fisher's exact test and provides stronger evidence for

© 1998 John Wiley & Sons, Ltd.

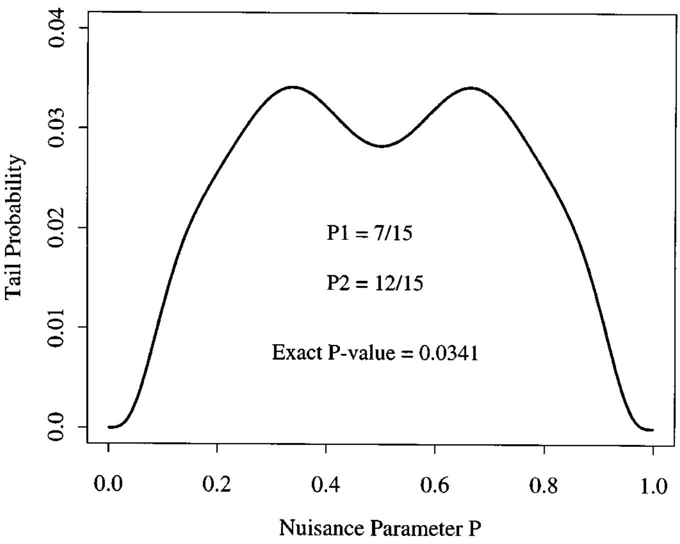

Figure 5. Tail probabilities of the exact test of efficacy for the influenza vaccine study

protective efficacy of the influenza vaccine. Figure 5 plots the exact tail probabilities as a function of the nuisance parameter P for this example, and the maximum occurs at $P = 0{\cdot}336$ or $P = 0{\cdot}667$. To illustrate the test of efficacy with a non-zero lower bound, we perform the test for $H_0 : \pi = 0{\cdot}1$ versus $H_0 : \pi > 0{\cdot}1$ based on the data from the influenza vaccine study. The test statistic (Z_{EF}) is $-1{\cdot}5255$, and the associated exact p-value is 0·0857. The normal approximation of Z_{EF} yields a p-value of 0·0636.

4. DISCUSSION

The exact methods described in this paper provide alternatives for large sample approaches to tests of equivalence and efficacy in binomial trials. These tests generalize classical methods to include testing of null hypotheses of prespecified differences. It is very important that one specify the minimum difference (δ) or minimum efficacy (π_0) in the protocol prior to the start of the study. The proposed exact tests use unconditional distributions of the test statistic, and are more sensitive than Fisher's type exact tests, which are based on conditional distributions.

We obtain the exact p-value by maximizing over the domain of the nuisance parameter. It casts for the worst configuration of the sample outcomes, but sometimes the p-value could be overconservative if the peak occurs at a place far away from the true value of P. Berger and Boos[16] proposed a method that maximizes the p-value over a confidence set rather than the whole domain for the nuisance parameter. Their method can reduce the conservatism if the peak (often near the extreme) is not in the confidence set. StatXact[17] has incorporated this method for classical analyses of two binomial proportions. One can also implement the method in the proposed tests. First, construct a $100(1-\gamma)$ percent confidence interval, denoted by C_γ, for the nuisance parameter P. Second, compute the maximum tail probability in the confidence interval,

© 1998 John Wiley & Sons, Ltd.

and the exact p-value is given as

$$pvalue_\gamma = \max_{P \in C_\gamma} \Pr(Z \leqslant Z_{\text{obs}} \,|\, P) + \gamma$$

where γ serves as a penalty for restricting the search for the maximum tail probability to the confidence set. For the influenza example discussed in Section 3, a 99·99 percent ($\gamma = 0{\cdot}0001$) exact confidence interval for the nuisance parameter P under the null hypothesis $H_0 : \pi = 0$ is (0·2772, 0·9601). The maximum tail probability in this confidence interval is 0·0341, and the exact p-value ($pvalue_\gamma$) is 0·0342. Note that this p-value is actually greater than the p-value obtained from the whole domain simply because the peaks (at $P = 0{\cdot}336$ and 0·664) lie within the confidence interval.

The proposed tests use numerical search for the maximum tail probability, and the computational effort required increases drastically with the sample size. As a result, the exact unconditional test may not be feasible in studies that involve a very large number of subjects. Liddell[18] and Storer and Kim[9] proposed approximate exact tests by obtaining the p-value at the maximum likelihood estimate of the nuisance parameter. Their methods reduce the computational effort substantially and provide reasonable approximation to the exact p-value. However, they do not guarantee the level of tests. In the rare event studies where one can assume a Poisson approximation to the event rate, it is possible to obtain an exact test based on the conditional distribution given the total number of events observed in the two groups.[7,19] One can greatly reduce the computational effort for the Poisson case from that required for the exact unconditional test.

One can also invert the exact test procedures to construct exact confidence intervals for the true difference of proportions ($\delta = P_1 - P_2$) and the true efficacy (π) using the duality of hypothesis testing and interval estimation. To construct a $(1 - \alpha)$ 100 percent exact confidence interval for δ, for instance, one can obtain the upper limit as the value of δ for which the exact p-value is $\alpha/2$ for testing hypothesis (1), and similarly one can obtain the lower limit as the value of δ for which the exact p-value is $\alpha/2$ for testing the other one-sided hypothesis $H_0 : P_1 - P_2 \leqslant \delta$ versus $H_1 : P_1 - P_2 > \delta$. For a given observed outcome, the number of tables in its tail may change with different values of δ. The computational effort involved in calculating confidence intervals is much more complicated than performing hypothesis tests because it requires numerical search on the parameter (δ or π) space as well as the nuisance parameter (P) space. In addition, one needs to update the tail with each new value of the parameter. We are currently working on this problem in an attempt to develop more efficient methods to construct test-based exact confidence intervals.

ACKNOWLEDGEMENTS

The author would like to thank Dan Zelterman, the referee, and the editor for very helpful suggestions that greatly improved the paper.

REFERENCES

1. Rodary, C., Com-Nougue, C. and Tournade, M. 'How to establish equivalence between treatments: A one-sided clinical trial in paediatric oncology', *Statistics in Medicine*, **8**, 593–598 (1989).
2. Fries, L. F. *et. al.* 'Safety and immunogenicity of a recombinant protein influenza A vaccine in adult human volunteers and protective efficacy against wild-type H1N1 virus challenge', *Journal of Infectious Diseases*, **167**, 593–601 (1993).

© 1998 John Wiley & Sons, Ltd.

3. Food and Drug Administration. 'Points to consider for the evaluation of combination vaccines: production, testing and clinical study', draft, 1995.
4. Farrington, C. P. and Manning, G. 'Test statistics and sample size formulae for comparative binomial trials with null hypothesis of non-zero risk difference or non-unity relative risk', *Statistics in Medicine*, **9**, 1447–1454 (1990).
5. Blackwelder, W. C. 'Proving the null hypothesis in clinical trials', *Controlled Clinical Trials*', **3**, 345–353 (1982).
6. Dunnett, C. W. and Gent, M. 'Significance testing to establish equivalence between treatments with special reference to data in the form of 2×2 tables', *Biometrics*, **33**, 593–602 (1977).
7. Blackwelder, W. C. 'Sample size and power for prospective analysis of relative risk', *Statistics in Medicine*, **12**, 691–698 (1993).
8. Miettinen, O. and Nurminen, M. 'Comparative analysis of two rates', *Statistics in Medicine*, **4**, 213–226 (1985).
9. Storer, B. E. and Kim, C. 'Exact properties of some exact test statistics for comparing two binomial proportions', *Journal of the American Statistical Association*, **85**, 146–155 (1990).
10. Agresti, A. 'A survey of exact inference for contingency tables' (with discussion), *Statistical Science*, **7**, 131–177 (1992).
11. Zelterman, D., Chan, I. S. F. and Mielke, P. W. Jr. 'Exact tests of significance in higher dimensional tables', *American Statistician*, **49**, 357–361 (1995).
12. Fisher, R. A. *The Design of Experiment*, Oliver & Boyd, Edinburgh, 1935.
13. Basu, D. 'On the elimination of nuisance parameters', *Journal of the American Statistical Association*, **72**, 355 (1977).
14. Suissa, S. and Shuster, J. J. 'Exact unconditional sample sizes for the 2×2 binomial trial', *Journal of the Royal Statistical Society*, *Series A*, **148**, 317–327 (1985).
15. Haber, M. 'An exact unconditional test for the 2×2 comparative trials', *Psychological Bulletin*, **99**, 129–132 (1986).
16. Berger, R. L. and Boos, D. D. 'P values maximized over a confidence set for the nuisance parameter', *Journal of the American Statistical Association*, **89**, 1012–1016 (1994).
17. *StatXact* 3. Cytel Software Corporation, MA, 1995.
18. Liddell, D. 'Practical tests of 2×2 contingency tables', *Statistician*, **25**, 295–304 (1978).
19. Bohidar, N. and Chan, I. S. F. 'An evaluation of exact power calculations for vaccine efficacy trials', Abstract, International Biometric Conference, Amsterdam, Netherlands, 1996.

© 1998 John Wiley & Sons, Ltd.

Step 1 Draft 2 (July 17, 2003) Draft 3 (November 12, 2003), Draft 4 (June 10, 2004)

THE CLINICAL EVALUATION OF QT/QTc INTERVAL PROLONGATION AND PROARRHYTHMIC POTENTIAL FOR NON-ANTIARRHYTHMIC DRUGS

Preliminary Concept Paper
For Discussion Purposes Only

This draft guidance, when finalized, will represent the Food and Drug Administration's (FDA's) current thinking on this topic. It does not create or confer any rights for or on any person and does not operate to bind FDA or the public. You can use an alternative approach if it satisfies the requirements of the applicable statutes and regulations. If you want to discuss an alternative approach, contact the FDA staff responsible for implementing this guidance. If you cannot identify the appropriate FDA staff, call the appropriate number listed on the title page of this guidance.

TABLE OF CONTENTS

INTRODUCTION

1.1 Background

1.2 Objectives

1.3 Scope

2.0 CLINICAL TRIALS

2.1 Design Considerations

2.1.1 Subject Enrollment, Safety Monitoring, and Discontinuation Criteria

2.1.2 'Thorough QT/QTc Study': Dose-Effect

and Time Course Relationships

2.1.3 Clinical Trial Evaluation After the 'Thorough QT/QTc Study

2.2 Collection, Assessment and Submission of Electrocardiographic Data

2.2.1 Collection of Standard 12-Lead Electrocardiograms (ECGs)

2.2.2 Assessment of Standard 12-Lead ECGs

2.2.3 Ambulatory ECG Monitoring

2.2.4 Submission of Interval and Waveform Data

3.0 ANALYSIS OF ECG DATA FROM CLINICAL TRIALS

3.1 QT Interval Correction Formulae

3.1.1 Population-Derived Correction Formulae

3.1.2 Correction Formulae Derived from Within-Subject Data

3.2 Analysis of QT/QTc Interval Data

3.2.1 Analyses of Central Tendency

3.2.2 Categorical Analyses

3.2.3 QT/QTc Interval Dispersion

3.3 Morphological Analyses of ECG Waveforms

4.0 ADVERSE EVENTS

4.1 Clinical Trial Adverse Event Reports

4.2 Premature Discontinuations or Dosage Reductions

4.3 Pharmacogenetic Considerations

4.4 Post-Marketing Adverse Event Reports

5.0 REGULATORY IMPLICATIONS, LABELLING, AND RISK MANAGEMENT STRATEGIES

5.1 Relevance of QT/QTc Interval Prolonging Effects to the

Approval Process

5.2 Labelling Issues for Drugs that Prolong the QT/QTc Interval

5.3 Post-Marketing Risk Management for Drugs that Prolong the QT/QTc Interval

THE CLINICAL EVALUATION OF QT/QTc INTERVAL PROLONGATION AND PROARRHYTHMIC POTENTIAL FOR NON-ANTIARRHYTHMIC DRUGS

1.0 INTRODUCTION

1.1 Background

An undesirable feature of some non-antiarrhythmic drugs is their ability to delay cardiac repolarization, an effect that can be measured as prolongation of the QT interval on the surface electrocardiogram (ECG). The QT interval represents the duration of ventricular depolarization and subsequent repolarization, beginning at the initiation of the QRS complex and ending where the T wave returns to isoelectric baseline. A delay in cardiac repolarization creates an electrophysiological environment that favors the development of cardiac arrhythmias, most clearly torsade de pointes, but possibly other ventricular arrhythmias as well. Torsade de pointes (TdP) is a polymorphic ventricular tachyarrhythmia that appears on the ECG as continuous twisting of the vector of the QRS complex around the isoelectric baseline. A feature of TdP is pronounced prolongation of the QT interval in the supraventricular beat preceding the arrhythmia. TdP can degenerate into ventricular fibrillation, leading to sudden death.

While the degree of QT prolongation is recognized as an imperfect biomarker for proarrhythmic risk, there is a qualitative relationship between QT prolongation and the risk of TdP, especially for drugs that cause substantial prolongation of the QT/QTc interval.

Because of its inverse relationship to heart rate, the QT interval is routinely transformed (normalized) by means of various formulae into a heart rate independent "corrected" value known as the QTc interval. The QTc interval is intended to represent the QT interval at a standardized heart rate of 60 bpm. For drugs that prolong the QT/QTc interval, the mean degree of prolongation has been roughly correlated with the observed risk of clinical proarrhythmic events. It is not clear, however, whether arrhythmia development is more closely related to an increase in the absolute QT interval or an increase in the relative ("corrected") QT

interval (QTc). Most drugs that have caused TdP clearly increase both the absolute QT and the QTc (hereafter called QT/QTc). The combination of QT/QTc interval prolongation and documented cases of TdP (fatal and non-fatal) associated with the use of a drug has resulted in regulatory actions, including withdrawal from the market, relegation to second-line status or denial of marketing authorization. Because prolongation of the QT/QTc interval is the ECG finding associated with the increased susceptibility to these arrhythmias, an adequate pre-marketing investigation of the safety of a new pharmaceutical agent should include rigorous characterization of its effects on the QT/QTc interval. The relevant non-clinical and clinical data will be used to make an integrated assessment of proarrhythmic risk for novel drug therapies.

1.2 Objectives

This document provides recommendations to sponsors concerning the design, conduct, analysis, and interpretation of clinical studies to assess the potential of a drug to delay cardiac repolarization. This assessment should include testing the effects of new agents on the QT/QTc interval as well as the collection of cardiovascular adverse events. The investigational approach used for a particular drug should be individualized, depending on the pharmacodynamic, pharmacokinetic, and safety characteristics of the product, as well as on its proposed clinical use.

The assessment of the effects of drugs on cardiac repolarization is the subject of active investigation. When additional data (non-clinical and clinical) are accumulated in the future, this document may be reevaluated and revised.

1.3 Scope

The recommendations contained in this document are generally applicable to new drugs having systemic bioavailability. The focus is on agents being developed for uses other than the control of arrhythmias, as antiarrhythmic drugs can prolong the QT/QTc interval as a part of their mechanism of clinical efficacy. While this document is concerned primarily with the development of novel agents, the recommendations might also be applicable to approved drugs when a new dose or route of administration is being developed that results in significantly higher C_{max} or AUC values. Additional ECG data might also be considered appropriate if a new indication or patient population were being pursued. The evaluation of the effect of a drug on the QT interval would also be considered important if the drug or members of its chemical or pharmacological class have been associated with QT/QTc interval prolongation, TdP, or sudden cardiac death during post-marketing surveillance.

2.0 CLINICAL TRIALS

2.1 Design Considerations

In general, drugs should receive an electrocardiographic evaluation, beginning early in clinical development, typically including a single trial dedicated to evaluating their effect on cardiac repolarization ('thorough QT/QTc study'). The ability of a drug to prolong the QT/QTc interval is linked to pharmacologic effects that can be investigated in non-clinical models as well as clinically. At present, whether non-clinical testing can exclude a clinical risk for QT/QTc prolongation is controversial. Conduct of the 'thorough QT/QTc study', as described in section 2.1.2, would be needed in almost all cases for regions where non-clinical data are not considered able to preclude risk of QT/QTc prolongation. For regions where non-clinical data are considered informative enough about the risk of QT/QTc prolongation in humans, the recommendations in this guidance for the clinical evaluation of QT/QTc could be modified. Additional factors that could influence the need for such a study include duration of treatment, metabolic profile, pharmacodynamic duration of action, and previous experience with other members of the same chemical or pharmacological class.

As discussed below, the results of the 'thorough QT/QTc study' will influence the amount of information collected in later stages of development:

- A negative 'thorough QT/QTc study', even in the presence of non-clinical data of concern, will almost alwaysallow the collection of on-therapy ECGs in accordance with the current practices in each therapeutic area to constitute sufficient evaluation during subsequent stages of drug development (see section 2.1.3);

- A positive 'thorough QT/QTc study' will almost always call for an expanded ECG safety evaluation during later stages of drug development (see section 2.1.3).

2.1.1 Subject Enrollment, Safety Monitoring, and Discontinuation Criteria

Subject enrollment, discontinuation criteria and safety monitoring for a given trial would be influenced by the clinical and non-clinical information available on the effects of the drug on cardiac repolarization.

Regarding subject enrollment, until the effects of the drug on the QT/QTc interval have been characterized, the following exclusion criteria are suggested:

- A marked baseline prolongation of QT/QTc interval (*e.g.*, repeated demonstration of a QTc interval >450);

- A history of additional risk factors for TdP (*e.g.*, heart failure, hypokalemia, family history of Long QT Syndrome);

- The use of concomitant medications that prolong the QT/QTc

interval;

If supported by the QT/QTc interval safety data from the early studies, later clinical trials could expand the eligibility criteria to include a broader spectrum of patients who are likely to receive the drug once approved.

Regarding safety monitoring, the procedures to follow if a patient experiences an adverse event suggestive of TdP should be specified in the clinical trial protocol.

Discontinuation of a subject from a clinical trial should be considered if there is a marked prolongation of the QT/QTc interval during treatment with the study drug, especially if the measurement is obtained from more than one ECG. While increases in QT/QTc to >500 ms or of >60 ms over baseline are commonly used as thresholds for potential discontinuation, the exact criteria chosen for a given trial will depend on the risk-tolerance level considered appropriate for the indication and patient group in question.

2.1.2 The 'Thorough QT/QTc Study': Dose-Effect and Time Course Relationships

An adequate drug development programme should ensure that the dose-response and generally the concentration-response relationship for QT/QTc prolongation have been characterized, including exploration of concentrations that are higher than those achieved following the anticipated therapeutic doses. Data on the drug concentrations around the time of ECG assessment would aid this assessment. If not precluded by considerations of safety or tolerability due to adverse effects, the drug should be tested at substantial multiples of the anticipated maximum therapeutic exposure. Alternatively, if the concentrations of a drug can be increased by drug-drug or drug-food interactions involving metabolizing enzymes (*e.g.*, CYP3A4, CYP2D6) or transporters (*e.g.*, P-glycoprotein), these studies can be performed under conditions of maximum inhibition. This approach calls for a detailed understanding of the absorption, distribution, metabolism and excretion of the drug. In general, the duration of dosing or dosing regimen should be sufficient to characterize the effects of the drug and its active metabolites at relevant concentrations.

The 'thorough QT/QTc study' is intended to determine whether the drug has a threshold pharmacologic effect on cardiac repolarization, as detected by QT/QTc prolongation. The study is typically carried out in healthy volunteers (as opposed to individuals at increased risk of arrhythmias) and is used to determine whether or not the effect of a drug on the QT/QTc interval in target patient populations need to be studied intensively during later stages of drug development. Although data are limited, it is expected that the results of the 'thorough QT/QTc study' would not be affected by ethnic factors.

The 'thorough QT/QTc study' would typically be conducted early in clinical development to provide maximum guidance for later trials,

although the precise timing will depend on the specifics of the drug under development. It would usually not be the first study, as it is important to have basic clinical data for its design and conduct, including tolerability and pharmacokinetics. It would often be conducted in healthy volunteers. Some drugs might not be suitable for study in healthy volunteers because of issues related to tolerability (*e.g.*, neuroleptic agents, chemotherapeutics).

The timing of the collection of ECGs and the study design (*e.g.*, single or multiple dose, duration) of the 'thorough QT/QTc study' should be guided by the available information about the pharmacokinetic profile of the drug. For drugs with short half-lives and no metabolites, a single dose study might be sufficient. Studies should characterize the effect of a drug on the QT/QTc throughout the dosing interval. While the peak serum concentration does not always correspond to the peak effect on QT/QTc interval, care should be taken to perform ECG recordings at time points around the Cmax. As one intent of a positive control is to establish assay sensitivity, in multiple dose studies of new drugs a positive control only needs to be used long enough to have its expected effect.

The 'thorough QT/QTc study' should be adequate and well-controlled, with mechanisms to deal with potential bias, including use of randomization, appropriate blinding, and concurrent placebo control group. As this study has a critical role in determining the intensity of data collection during later stages of drug development, it is important to have a high degree of confidence in the ability of the study to detect differences of clinical significance. The confidence in the ability of the study to detect QT/QTc prolongation can be greatly enhanced by the use of a concurrent positive control group to establish assay sensitivity. Absence of a positive control should be justified and alternative methods to establish assay sensitivity provided. It is difficult to determine whether there is an effect on the mean QT/QTc interval that is so small as to be of no consequence. However, drugs that prolong the mean QT/QTc interval by around 5 ms or less do not appear to cause TdP. On that basis, the positive control (whether pharmacological or non-pharmacological) should be well-characterized and consistently produce an effect corresponding to the largest change in the QT/QTc interval that is currently viewed as clinically not important to detect (a mean change of around 5 ms or less)[1].

Based on similar considerations, a negative 'thorough QT/QTc study' is one where the largest time-matched mean difference between the drug and placebo (baseline-subtracted) for the QTc interval is around 5 ms or less, with a one-sided 95% confidence interval that excludes an effect >8.0 ms[2]. This upper bound was chosen to reflect the uncertainty related to the variability of repeated measurements. As with other data, the presence of outliers (see section 3.2.2) should also be explored.

If an investigational drug belongs to a chemical or pharmacological class that has been associated with QT/QTc prolongation, a positive control selected from other members of the same class is recommended to

permit a comparison of effect sizes, preferably at equipotent therapeutic doses.

Crossover or parallel group study designs can be suitable for trials assessing the potential of a drug to cause QT/QTc interval prolongation. Crossover studies at least have two potential advantages:

- They usually call for smaller numbers of subjects than parallel group studies, as the subjects serve as their own controls and hence reduce variability of differences related to diurnal variations and inter-subject variability;

- They might facilitate heart rate correction approaches based on individual subject data.

Parallel group studies might be preferred under certain circumstances:

- For drugs with long elimination half-lives for which lengthy time intervals would be required to achieve steady-state or complete washout;

- If carryover effects are prominent for other reasons, such as irreversible receptor binding or long-lived active metabolites;

- If multiple doses or treatment groups are to be compared.

A critical problem in the measurement of the QT/QTc interval is its intrinsic variability. This variability results from many factors, including activity level, postural changes, circadian patterns, and food ingestion. It is considered essential to address intrinsic variability in the conduct of the 'thorough QT/QTc study'. This can be accomplished in several ways, including the collection of multiple ECGs at baseline and during the study.

2.1.3 Clinical Trial Evaluation After the 'Thorough QT/QTc Study'

In the absence of QT/QTc interval prolongation in the 'thorough QT/QTc study' (see section 2.1.2), the collection of baseline and periodic on-therapy ECGs in accordance with the current investigational practices in each therapeutic field is, in general, considered appropriate.

If the 'thorough QT/QTc study' is positive, additional evaluation in subsequent clinical studies should be performed. One objective of this evaluation should be to fully characterize the dose-, concentration-, and time- relationships of the drug on the QT/QTc interval in the target patient population(s) at therapeutic and supratherapeutic serum concentrations. The latter can be achieved in two ways: through administration of high doses or use of metabolic inhibitors (if applicable).

Another objective of this evaluation should be to collect information on

the adverse events that occur in the trials following the positive 'thorough QT/QTc study'. This would include patients who develop marked QT/QTc prolongation (*e.g.*, >500 ms) or experience a serious cardiovascular adverse event that suggests an arrhythmia (*e.g.*, TdP). Such patients should be evaluated closely for risk factors that might have contributed to this event (*e.g.*, genotyping for Long QT Syndromes, see section 4.3).

If the 'thorough QT/QTc study' is positive, analyses of the ECG and adverse event data from certain patient sub-groups are of particular interest, such as:

- Patients with electrolyte abnormalities (*e.g.*, hypokalemia);
- Patients with congestive heart failure;
- Patients with impaired drug metabolizing capacity or clearance (*e.g.*, renal or hepatic impairment, drug interactions);
- Female patients;
- Patients aged <16 and over 65 years.

Even if the 'thorough QT/QTc study' is negative, if other evidence of an effect in a patient population from subsequent studies (*e.g.*, marked QT/QTc interval prolongation, TdP) were to emerge, then additional investigation would be needed.

2.2 Collection, Assessment and Submission of Electrocardiographic Data

The recommendations below are most relevant to the 'thorough QT/QTc study' and to any studies investigating a drug with a known effect on cardiac repolarization.

2.2.1 Collection of Standard 12-Lead Electrocardiograms (ECGs)

Until better ways are established to assess proarrhythmic risk during drug development, the measurement of the QT/QTc interval on the surface ECG is central to the detection of that risk. The clinical ECG database is typically derived from the collection of 12-lead surface ECGs, although ambulatory ECG techniques show promise (see section 2.2.3).

2.2.2 Assessment of Standard 12-Lead ECGs

Several methods for measuring ECG intervals have been used in clinical trials, and for a given trial, the sponsor should describe the accuracy and precision of QT/QTc interval measurements using the selected system. The method chosen will depend on the level of precision needed for a

given trial. For example, the 'thorough QT/QTc study' would warrant particularly careful attention to interval measurement. At present, this would usually involve the measurement by a few skilled readers operating from a centralized ECG laboratory, although other methods (*e.g.*, semi-automated ECG reading) can be acceptable when appropriately supported. Readers of ECGs should be blinded to time, treatment and subject identifier, and one reader should read all the ECG recordings from a given subject. The degree of inter- and intra-reader variability should be established by having the assessors reread a subset of the data (both normal and abnormal) under blinded conditions. Criteria for ECG diagnoses and for identification of adverse events should be pre-defined by the sponsor. If well-characterized data validating the use of fully-automated technologies become available, the recommendations in the guidance for the measurement of ECG intervals could be modified. In the absence of a concern in the early clinical trial (s), automated ECG readings have a role in the rapid assessment of ECGs for safety.

The quality of the ECG database can depend on the use of modern equipment with the capacity for digital signal processing. Such equipment should be recently serviced and calibrated. Machine calibration records and performance data should be maintained on file. In the case of multicentre trials, training sessions are encouraged to ensure consistency of operator technique (e.g., skin preparation, lead placement, patient position) and data acquisition practices.

While the most appropriate lead(s) and methodology to measure the QT interval have not been established, lead II is often used. A consistent approach should be used for a given trial.

Morphological changes in the T-U complex might occur. Information should be provided on changes in T and U wave morphologies (see section 3.3). Discrete U waves should be excluded from the QT/QTc interval measurement

2.2.3 Ambulatory ECG Monitoring

While ambulatory ECG monitoring has historically not been sufficiently validated to be considered as the primary assessment ECG for QT/QTc interval effects, newer systems that allow for the collection of multiple leads that more closely approximate a surface ECG have potential value to collect interval data. The use of ambulatory ECG monitors might additionally allow detection of extreme QT/QTc interval events that occur infrequently during the day and asymptomatic arrhythmias. Data on the QT/RR from ambulatory ECG monitoring can also prove useful in the calculation of individualized QT corrections. However, as QT/QTc intervals measured by this methodology might not correspond quantitatively to those from standard surface ECGs, data obtained from the two methodologies might not be suitable for direct comparison, pooling, or interpretation using the same thresholds of concern.

2.2.4 Submission of Interval and Waveform Data

Regional guidance should be sought for information on the submission of ECG interval data and overall assessments.

3.0 ANALYSIS OF ECG DATA FROM CLINICAL TRIALS

Evaluation of the effects of a drug on the standard ECG intervals and waveforms is considered a fundamental component of the safety database of any new drug application.

Regardless of the outcome of the ‘thorough QT/QTc study’, ECG changes recorded as adverse events should be pooled from all studies for analysis. ECG interval data from the ‘thorough QT/QTc study’ should only be pooled with subsequent trials of similar rigor with regard to ECG data collection and analysis, but should not be pooled with trials using less rigorous ECG collection. Standardization of ECG collection for similar studies within a clinical trial programme will facilitate pooled analyses.

3.1 QT Interval Correction Formulae

As the QT interval has an inverse relationship to heart rate, the measured QT intervals are generally corrected for heart rate in order to determine whether they are prolonged relative to baseline. Various correction formulae have been suggested, of which Bazett’s and Fridericia’s corrections are the most widely used. In early trials evaluating the effects of a new drug on the QT/QTc interval in healthy volunteers, designed to detect relatively small effects (*e.g.*, 5 ms), it is important to apply the most accurate correction available (*e.g.*, methods using individually-derived relationships between RR and QT intervals). For later trials, where less ECG information is available, population-derived corrections, including standard correction formulae, can provide useful information.

Because the best correction approach is a subject of controversy, uncorrected QT and RR interval data, heart rate data, as well as QT interval data corrected using Bazett’s and Fridericia’s corrections should be submitted in all applications, in addition to QT interval data corrected using any other formulae. The sponsor should pre-specify the primary correction method. A concurrent positive control group is strongly encouraged to support the use of newer correction approaches (*e.g.*, individual subject correction) in order to demonstrate the ability of the correction method to allow detection of relevant effects on the QT/QTc interval.

3.1.1 Population-Derived Correction Formulae

Examples of such corrections include the following:

1) Bazett’s correction: $QTc = QT/RR^{0.5}$

2) Fridericia’s correction: $QTc = QT/RR^{0.33}$

Bazett's correction is frequently used in clinical practice and in the medical literature. In general, however, Bazett's correction overcorrects at elevated heart rates and under corrects at heart rates below 60 bpm and hence is not an ideal correction. Fridericia's correction is more accurate than Bazett's correction in subjects with such altered heart rates.

3) Corrections based on linear regression techniques

Application of linear regression techniques to plots of QT/RR data for the placebo or baseline study population allows for the estimation of the slope (b), which can be used for standardizing the data from both the drug and control groups to a normalized heart rate of 60 beats per minute, using the equation QT = a + b(RR). The Framingham correction [QTc = QT + 0.154(1-RR)] is one example of a correction derived by linear regression.

4) Corrections using linear or non-linear regression modeling on pooled data from large databases

3.1.2 Correction Formulae Derived from Within-Subject Data

Corrections for heart rate using individual subject data have been developed, applying regression analysis techniques to individual pre-therapy QT and RR interval data over a range of heart rates, then applying this correction to on-treatment QT values. These approaches are considered most suitable for the 'thorough QT/QTc study' and early clinical studies, where it is possible to obtain many QT interval measurements for each study subject. As adaptation of the QT/QTc interval to changes in heart rate is not instantaneous, care should be taken to exclude ECG recordings collected during times of rapid heart rate changes due to this QT/RR hysteresis effect.

3.2 Analysis of QT/QTc Interval Data

Although increases from baseline in the QT/QTc interval constitute signals of interest, interpretation of these differences is complicated by the potential for changes not related to drug therapy, including regression toward the mean and choice of extreme values. Regression toward the mean refers to the tendency of subjects with high baseline values to have lower values at later time points, while subjects with low baseline values tend to experience increases. The direction of regression depends on initial selection criteria (for example, if subjects with high baseline QT/QTc interval values are excluded from the trial, values recorded during treatment will tend to rise relative to baseline levels). The process of choosing the highest of multiple observed values will also almost invariably cause an apparent change from any single baseline value, a phenomenon found in both drug and placebo-treated groups.

The QT/QTc interval data should be presented both as analyses of central tendency (*e.g.*, means, medians) and categorical analyses. Both can provide relevant information on clinical risk assessment.

3.2.1 Analyses of Central Tendency

The effect of an investigational drug on the QT/QTc interval is most commonly analyzed using the largest time-matched mean difference between the drug and placebo (baseline-subtracted) over the collection period (*e.g.*, hourly, weekly, monthly). Additional approaches to the assessment of central tendency could include analysis of time-averaged QT/QTc intervals or analysis of changes occurring at the Cmax for each individual.

3.2.2 Categorical Analyses

Categorical analyses of QT/QTc interval data are based on the number and percentage of patients meeting or exceeding some predefined upper limit value. Clinically noteworthy QT/QTc interval signals might be defined in terms of absolute QT/QTc intervals or changes from baseline. Absolute interval signals are QT/QTc interval readings in excess of some specified threshold value. Separate analyses should be provided for patients with normal and elevated baseline QT/QTc intervals. As with all QT/QTc interval analyses, categorical analyses are most informative when it is possible to compare the rate of supra-threshold readings in the treatment and control groups.

There is no consensus concerning the choice of upper limit values for absolute interval signals and change from baseline signals. While lower limits increase the false-positive rate, higher limits increase the risk of failing to detect a signal. In clinical trials, a prolongation of QTc > 500 ms during therapy has been a threshold of particular concern. Multiple analyses using different signal values are a reasonable approach to this uncertainty, including:

- Absolute QTc interval prolongation:
    - QTc interval > 450
    - QTc interval > 480
    - QTc interval > 500
- Change from baseline in QTc interval:
    - QTc interval increases from baseline ≥30
    - QTc interval increases from baseline ≥60

3.2.3 QT/QTc Interval Dispersion

QT/QTc interval dispersion, defined as the difference between the shortest and the longest QT/QTc interval measured on the 12-lead ECG, has been thought to reflect the regional heterogeneity of cardiac repolarization. Normal values are typically in the range of 40-60 ms.

Absolute values of ≥100 ms and changes from baseline of >100% have been suggested as clinically noteworthy signals for categorical analyses. The value of assessment of QT/QTc interval dispersion as a measure of proarrhythmic risk of a drug is, however, the subject of debate, and the predictive value of this parameter has yet to be demonstrated. Analyses of QT/QTc dispersion should therefore be used, if at all, to supplement more standard analyses of QT/QTc interval duration.

3.3 Morphological Analyses of ECG Waveforms

While the predictive value of changes in ECG morphology, such as the development of U waves, has not been established, morphological abnormalities should be described and the data presented in terms of the number and percentage of subjects in each treatment group having changes from baseline that represent the appearance or worsening of the morphological abnormality. Typically these data will be obtained as a part of the 'thorough QT/QTc study'.

4.0 ADVERSE EVENTS

In addition to data on changes in ECG intervals, adverse event data can be another source of information on proarrhythmic potential, including:

- Premature discontinuations and dosage adjustments during clinical studies;
- Post-marketing adverse event reports if available.

4.1 Clinical Trial Adverse Events

Although drug-induced prolongation of the QT/QTc interval is usually asymptomatic, an increased rate of certain adverse events in patients taking an investigational agent can signal potential proarrhythmic effects. The rates of the following clinical events should be compared in the treated and control patients, particularly when there is evidence of an effect on the QT/QTc interval:

- Torsade de pointes;
- Sudden death;
- Ventricular tachycardia;
- Ventricular fibrillation and flutter;
- Syncope;
- Dizziness;
- Seizures.

Torsade de pointes (TdP) is infrequently captured in most clinical

databases, even those for drugs known to have significant proarrhythmic effects. Given this, the failure to observe an episode of TdP in a drug application database is not considered sufficient grounds for dismissing the possible arrhythmogenic risks of a drug when these are suspected on the basis of ECG and other clinical data. The other adverse events listed above, while less specific for an effect on cardiac repolarization, are more commonly captured in clinical trials, and an imbalance in their frequency between study groups can signal a potential proarrhythmic effect of the investigational agent. Sub-group analyses should be conducted in terms of age, gender, pre-existing cardiac disease, electrolyte disturbances, and concomitant medications. Comparing cause-specific rates of death is difficult, but a difference in the fraction of total deaths qualifying as "sudden" has also been proposed as a marker for proarrhythmic potential.

Detailed patient narratives should be provided for all serious cardiac adverse events, as would be the case for any serious event or events leading to discontinuation. In assessing the possible causal relationship of drug-induced QT/QTc interval prolongation to the event, attention should be directed to considerations such as temporal relationship and ECG results collected at the time of the event. As the QT/QTc interval is subject to considerable fluctuation, a possible role for QT/QTc interval prolongation should not be dismissed on the basis of normal on-therapy ECG measurements performed prior to, or near the time of the adverse event. In addition to an appropriate adverse reaction report, patients with marked QT/QTc prolongation or an episode of TdP might provide useful information on risk management. When identified, they should therefore be examined closely for other risk factors (*e.g.*, genetic predisposition, see section 4.3). Rechallenge with the investigational drug under appropriately monitored conditions can provide useful information on dose- and concentration-response relationships.

In evaluating the safety database of a new drug, consideration should be given to the extent to which the inclusion and exclusion criteria for patient eligibility might have influenced the study population with respect to the risk of QT/QTc interval prolongation and associated adverse events (*e.g.*, exclusion of patients with cardiac co-morbidities or renal/hepatic impairment, prohibition of diuretics as concomitant medications). Ideally, the major clinical studies should include an adequate representation of female and elderly patients, as well as patients with co-morbidities and concomitant medications typical of the expected user population.

If a subject experiences symptoms or ECG findings suggestive of an arrhythmia during a clinical trial, immediate evaluation by a cardiac specialist is recommended, both for the purposes of treating the patient and for discussions related to continuation/ re-institution of the therapy.

4.2 Premature Discontinuations or Dosage Reductions

Particular attention should be directed to subjects or patients who are discontinued from clinical trials due to QT/QTc interval prolongation. Information should be provided on the basis for premature

discontinuation of the patient (*e.g.*, a QT/QTc interval value in excess of a protocol-defined upper limit, occurrence of QT/QTc interval prolongation in association with symptoms of arrhythmia), as well as the dose and duration of treatment, plasma levels if available, demographic characteristics, and the presence or absence of risk factors for arrhythmia.

Dosage reductions prompted by QT/QTc interval prolongation should also be documented.

4.3 Pharmacogenetic Considerations

Many forms of Long QT Syndrome are now known to be linked to mutations in genes encoding cardiac ion channel proteins. Because of incomplete penetrance, not all carriers of mutated ion channel genes will manifest QT/QTc interval prolongation in screening ECG evaluations. Common polymorphisms can affect ion channels, leading to an increased sensitivity to drugs that affect repolarization. When possible, and following informed consent, patients who experience marked prolongation of the QT/QTc or TdP while on drug therapy should be genotyped.

4.4 Post-Marketing Adverse Event Reports

Because documented cases of TdP are relatively rare, even for drugs that prolong the QT/QTc, they are often not reported until large populations of patients have received the agent in post-marketing settings. The available post-marketing adverse event data should be examined for evidence of QT/QTc interval prolongation and TdP and for adverse events possibly related to QT/QTc interval prolongation, such as cardiac arrest, sudden cardiac death and ventricular arrhythmias (*e.g.*, ventricular tachycardia and ventricular fibrillation). A well-characterized episode of TdP has a high probability of being related to drug use, whereas the other events that are reported more commonly would be of particular concern if reported in a population at low risk for them (*e.g.*, young men experiencing sudden death).

5.0 REGULATORY IMPLICATIONS, LABELLING, AND RISK MANAGEMENT STRATEGIES

5.1 Relevance of QT/QTc Interval Prolonging Effects to the Approval Process

Substantial prolongation of the QT/QTc interval, with or without documented arrhythmias, could be the basis for non-approval of a drug or discontinuation of its clinical development, particularly when the drug has no clear advantage over available therapy and available therapy appears to meet the needs of most patients. Failure to perform an adequate non-clinical and clinical assessment of the potential QT/QTc interval prolonging properties of a drug can likewise be justification to delay or deny marketing authorization. For non-antiarrhythmic drugs, the outcome of the risk benefit assessment will generally be influenced by the size of the QT/QTc interval prolongation effect, whether the

effect occurs in most patients or only in certain defined outliers, the overall benefit of the drug, and the utility and feasibility of risk management options. The inclusion of precautionary material in the prescribing information will not necessarily be considered an adequate risk management strategy, if implementation of the recommendations in a clinical use setting is judged to be unlikely.

If QT/QTc interval prolongation is a feature shared by other drugs of the therapeutic class in question, evaluation of the new drug could usefully involve a comparison of the magnitude and incidence of any QT/QTc interval prolongation effects relative to those of other members of its class in concurrent positive control groups.

It is difficult to determine whether there is an effect on the mean QT/QTc interval that is so small as to be inconsequential, but the risk of arrhythmias appears to increase with the extent of QT/QTc prolongation. Drugs that prolong the mean QT/QTc interval by around 5 ms or less do not appear to cause TdP. Whether this signifies that no increased risk exists for these compounds or simply that the increased risk has been too small to detect is not clear. The data on drugs that prolong the mean QT/QTc interval by more than around 5 and less than 20 ms are inconclusive, but some of these compounds have been associated with proarrhythmic risk. Drugs that prolong the mean QT/QTc interval by >20 ms have a substantially increased likelihood of being proarrhythmic, and might have clinical arrhythmic events captured during drug development.[3]

Regardless of the degree to which a drug prolongs the QT/QTc interval, decisions about its development and approval will depend upon the morbidity and mortality associated with the untreated disease or disorder and the demonstrated clinical benefits of the drug, especially as they compare with available therapeutic modalities. Demonstrated benefits of the drug in resistant populations or in patients who are intolerant of, or have a labeled contraindication to, approved drugs for the same disease represent additional relevant clinical considerations that might justify approval of the drug, if the indication were limited to use in such patients.

Some factors have been proposed that can modify the risk of QT/QTc prolongation. For instance, it has been suggested that some drugs might prolong the QT/QTc interval up to a "plateau" value, above which there is no dose-dependent increase, although this has not been demonstrated adequately to date. It has also been suggested that proarrhythmic risk might be influenced by other pharmacologic effects (*e.g.*, other channel effects). In any case, it is important to identify the "worst case scenario" for drugs that have demonstrated effects on QT/QTc interval as a part of risk assessment (*i.e.*, the QT/QTc interval measured in the target patient population at the time of peak effect and under conditions of the highest blood levels that can be attained during therapy).

5.2 Labelling Issues for Drugs that Prolong the QT/QTc Interval

It is recognized that there will be regional differences in labelling. However, it is recommended that the following be considered:

- A warning/precautionary statement about the risk;
- A description of the design and results of the trials investigating the effect on the QT/QTc interval, including the absence of demonstrated effect;
- The dosage recommendations;
- A list of conditions known to increase the proarrhythmic risk (*e.g.*, congestive heart failure, Long QT Ssyndrome, hypokalemia);
- A precautionary statement regarding the concomitant use of two or more QT/QTc interval prolonging drugs and other interactions increasing the risk.
- Recommendations for patient monitoring (ECG and electrolytes) and management of patients with QT/QTc prolongation or symptoms suggestive of an arrhythmia.

5.3 Post-Marketing Risk Management for Drugs that Prolong the QT/QTc Interval

The use of dosing adjustments following institution of therapy appears to materially decrease the risk of TdP in hospitalized patients receiving an antiarrhythmic drug; no similar data are available for drugs of other therapeutic classes. For approved drugs that prolong the QT/QTc interval, risk-management strategies aimed at minimizing the occurrence of arrhythmias associated with their use have focused on education of the health-care providers and patients.

---

[1] Comment is requested on the choice of the 5 ms as a threshold for clinical and regulatory concern.

[2] Comment is requested on the statistics here, including the 8 ms upper bound of the confidence interval. This interval is derived from an analysis of the variability observed for placebo data from five 'thorough QT/QTc studies'.

[3] Comment is requested on the risk categorization described in this paragraph.

Back to Top Back to Guidance Page

Date created: October 21, 2004

CDER Home Page | CDER Site Info | Contact CDER | What's New @ CDER
FDA Home Page | Search FDA Site | FDA A-Z Index | Contact FDA | Privacy | Accessibility |
HHS Home Page

FDA/Center for Drug Evaluation and Research

US Military Smallpox Vaccination Program Experience

John D. Grabenstein, RPh, PhD
William Winkenwerder, Jr, MD, MBA

Context The United States recently implemented smallpox vaccination of selected military personnel in a national program of preparedness against use of smallpox as a biological weapon. The resumption of smallpox vaccinations raises important questions regarding implementation and safety.

Objective To describe the US military smallpox vaccination program.

Design Descriptive study of the vaccination program from its inception on December 13, 2002, through May 28, 2003.

Setting US Department of Defense (DoD) fixed and field medical treatment facilities on multiple continents and ships at sea.

Subjects US service members and DoD civilian workers eligible for smallpox vaccination.

Main Outcome Measures Numbers of vaccinations and rates of vaccination exemptions, symptoms, and adverse events. Data were collected via reports to headquarters and rigorous surveillance for sentinel events.

Results In 5.5 months, the DoD administered 450293 smallpox vaccinations (70.5% primary vaccinees and 29.5% revaccinees). In 2 settings, 0.5% and 3.0% of vaccine recipients needed short-term sick leave. Most adverse events occurred at rates below historical rates. One case of encephalitis and 37 cases of acute myopericarditis developed after vaccination; all cases recovered. Among 19461 worker-months of clinical contact, there were no cases of transmission of vaccinia from worker to patient, no cases of eczema vaccinatum or progressive vaccinia, and no attributed deaths.

Conclusions Mass smallpox vaccinations can be conducted safely with very low rates of serious adverse events. Program implementation emphasized human factors: careful staff training, contraindication screening, recipient education, and attention to bandaging. Our experience suggests that broad smallpox vaccination programs may be implemented with fewer serious adverse events than previously believed.

JAMA. 2003;289:3278-3282 www.jama.com

In December 2002, the United States implemented a program of smallpox vaccinations for approximately 500000 military personnel. The directive came as part of a national program of preparedness against biological attack.[1] Preattack vaccination was determined to be the best way to personally protect troops so they can continue their missions.[2,3] The program was therefore mandatory for designated service members and employees except those with contraindications. This article describes the first 450293 vaccinations.

METHODS

To develop vaccination policy, the US Department of Defense (DoD) drew from its own physicians, scientists, and administrators[3] as well as colleagues in government and academia. The military vaccination program included vaccination for smallpox epidemic response teams (2000-5000 people) to assist with epidemic control and contact tracing in an outbreak, medical teams for hospitals and clinics (10000-25000 people) to care for smallpox cases, and operational forces (up to 500000 people) to preserve critical capabilities.

Education, Training, and Screening

The US Army conducted a medical training conference in October 2002. This conference was videotaped and posted at http://www.smallpox.army.mil. Medical directors completed 8 hours of training; physicians and supervising nurses completed 6 hours; vaccinating nurses and medics completed 3 hours. Leaders educated vaccine candidates and reinforced messages after vaccination. We provided brochures and curricula as well as customized information services via telephone (877-GET-VACC) or e-mail (vaccines@amedd.army.mil).

To identify people with contraindications to vaccination,[4-8] clinicians used a standardized recording form to screen for skin conditions, immunosuppression, pregnancy or breastfeeding, and allergies (http://www.smallpox.army.mil/resource/forms.asp). Completed forms were reviewed by medics, nurses, nurse practitioners, physician assistants, and physicians in a triaged manner before vaccination. The DoD used its periodic testing program for human immunodeficiency virus (HIV) infection to identify that contraindication. Women were advised that pregnancy is a contraindication, asked about pregnancy status, and provided pregnancy tests, if requested.

See also pp 3283, 3290, 3295, and 3306.

Author Affiliations: Military Vaccine Agency, US Army Medical Command, Falls Church, Va (Dr Grabenstein); Assistant Secretary of Defense (Health Affairs), Pentagon, Washington, DC (Dr Winkenwerder).
Corresponding Author and Reprints: John D. Grabenstein, RPh, PhD, Military Vaccine Agency, 5111 Leesburg Pike, Suite 401, Falls Church, VA 22041 (e-mail: john.grabenstein@us.army.mil).

Infection Control

Each hospital and clinic established bandage evaluation stations.[6,7] For non–health care workers, infection control practices focused on bandages (eg, simple Band-Aid type bandages with 20 × 25-mm pads), sleeves, and hand washing. To calculate time at risk, each vaccinee was assumed capable of spreading vaccinia for 21 days after vaccination.

Quality Control and Implementation

The licensed full-strength smallpox vaccine (Dryvax, Wyeth Laboratories, Marietta, Pa) containing the New York City Board of Health strain of vaccinia was used in this program.[4,8] First-time vaccination entailed 3 punctures with a bifurcated needle. Previous vaccinees received 15 punctures. Those who did not respond with a major reaction as defined by the World Health Organization (WHO)[5,8] were vaccinated again.

Smallpox vaccinations began at 4 pilot sites: Walter Reed Army Medical Center, Washington, DC; Aberdeen Proving Ground, Md; Wilford Hall Air Force Medical Center, Lackland Air Force Base, San Antonio, Tex; and the National Naval Medical Center, Bethesda, Md. For quality control, clinics tracked the vaccination response rates of the first 25 people for each vaccinator.

Surveillance for Adverse Events

Postvaccination surveillance assesses the screening process, health events shortly after vaccination, and events months or years after vaccination. Vaccine Healthcare Centers evaluate complex cases. A registry was established for women vaccinated before recognition of pregnancy, a joint project with the Centers for Disease Control and Prevention (CDC).[9] An independent safety monitoring panel, coordinated by the Armed Forces Epidemiological Board and the Advisory Committee on Immunization Practices, reviews weekly updates.[10] We shared experience weekly with CDC and state health departments.

Adverse events reported here were collected via symptom surveys from each vaccinated member of the first 7 operational units vaccinated (n=526), plus reports to headquarters and rigorous surveillance for sentinel events. Frequencies are reported for the units described. The number of adverse events identified was divided by the size of the vaccinated cohort. Exact confidence intervals were calculated for Poisson distribution of rare events.

Definitions of adverse events were derived from expert consultants. Generalized vaccinia involved vesicles or pustules in several locations distant from the vaccination site that evolved like vaccinial vesicles and pustules. Encephalitis involved altered consciousness for at least 24 hours with more than 5 white blood cells/µL in cerebrospinal fluid. Myopericarditis involved chest pain and elevated cardiac enzymes (eg, troponin, creatine kinase–MB), as reported by Halsell et al[11] elsewhere in this issue of THE JOURNAL.

RESULTS

From December 13, 2002, through May 28, 2003, a total of 450293 personnel were vaccinated against smallpox with the New York City Board of Health strain (TABLE 1). Vaccinations occurred in fixed and field medical facilities in North America, Europe, Asia, Africa, and Oceania and on ships at sea. At peak, more than 50000 vaccinations were administered per week.

Table 1. Demographic Characteristics of Smallpox Vaccinees, December 13, 2002, to May 28, 2003 (n = 450293)*

Characteristics	
Age, y	
Mean (SD)	28.8 (8.3)
Median (range)	26 (17-76)
Sex	
Female	57460 (12.8)
Male	392833 (87.2)
Ethnicity	
Non-Hispanic	394857 (87.7)
Hispanic	39461 (8.8)
Unspecified	15975 (3.5)
Race	
White	328612 (73.0)
African American	80796 (17.9)
Asian/Pacific Islander	10459 (2.3)
Native American	3927 (0.9)
Other	26499 (5.9)
Vaccination status	
Primary vaccinees	317637 (70.5)
Revaccinees	132656 (29.5)

*Vaccinations continue. Current number of vaccinees is larger. Data are No. (%) unless otherwise specified.

The median age of vaccinees was 26 years and 87% were men; 70.5% were receiving their primary (first) vaccination. Contraindications due to either personal conditions or conditions among household contacts varied by military unit, temporarily deferring vaccination in 11% to 34% of eligible personnel. Skin conditions predominated, followed by pregnancy and immune conditions. Exemptions ranged from 4.9% to 7.8% among deployed units, where household contacts were not present.

Military immunization clinics reported high rates of successful vaccination (major reactions, response rates).[5,8] Some clinicians sought advice to confirm that small dermal responses fit the WHO definition. At the 4 pilot clinics, 971 of 1017 primary vaccinees (95.5%) were successfully vaccinated with a single vaccination. Among 975 people who had been vaccinated decades earlier, 934 (95.8%) were successfully vaccinated with a single vaccination. Around the world, 2 vaccine recipients were treated successfully with epinephrine for acute allergic events.

Vaccinated health care workers remained on the job (including surgery and labor and delivery), using bandages, sleeves, hand washing, and evaluation stations. Vaccinated workers were not prohibited from caring for immunocompromised patients. No transmission of vaccinia from a health care worker to a patient was identified during an estimated 19461 worker-months of interaction with patients. Conversely, no vaccinee later treated as a patient transmitted vaccinia to a health care worker.

Expected temporary symptoms occurred after vaccination, notably itching, muscle ache, "feeling lousy," headache, swollen lymph nodes, irritation from bandages, subjectively reported fever, and local rash (TABLE 2). Approximately 1% of vaccine recipients developed cutaneous eruptions beyond the vaccination site.

Among carefully observed workers at Walter Reed Army Medical Center, the frequency of vaccinated workers

taking sick leave was 3.0% (16/530), typically 8 to 12 days after vaccination. The most common duration was 1 day (mean, 1.5 days; maximum, 4 days). Eleven of 198 primary vaccinees (5.5%) took sick leave, as did 5 of 332 revaccinees (1.5%). Among 16 deployed US Air Force clinics serving 20 272 vaccinees, 0.5% took 1 or 2 days of sick leave after vaccination. Other sites reported similar results.

Skin eruptions or rashes caused concern among clinicians. These rashes were generally mild and treated symptomatically. In 16 cases with maculopapular rashes, vaccine recipients were hospitalized, in part because of uncertainty about contagiousness or clinical course. This was especially true early in the program. In each case, patients returned to duty promptly. Subsequent patients of comparable acuity were typically not hospitalized. Six service members developed cellulitis as diagnosed by physicians, were treated with intravenous antibiotics, and returned to duty.

Table 2. Expected Temporary Symptoms and Short-term Outcomes After Smallpox Vaccination*

	No. (%)
Symptoms	
Local itching	313 (60)
Muscle ache	110 (21)
Feeling lousy	109 (20)
Headache	95 (18)
Swollen lymph nodes	74 (14)
Bandage reaction	39 (7.4)
Itchy all over	29 (5.5)
Fever (subjective)	28 (5.3)
Local rash	28 (5.3)
Body rash	8 (1.1)
Outcomes	
Took medication	89 (17)
Restricted activity	7 (1.3)
Outpatient visit	4 (0.8)
Missed work	1 (0.2)

*Via survey completed 6 to 8 days after vaccination among all 526 vaccinated personnel assigned to the 2nd (New York), 35th (West Virginia), and 103rd (Alaska) Civil Support Teams; Aberdeen Proving Ground, Md; Fort Jackson, SC; Lackland Air Force Base, Tex; and Schofield Barracks, Hawaii.

Noteworthy adverse reactions are summarized in TABLE 3. Thirty-six vaccine recipients may qualify as mild cases of generalized vaccinia (based on reports to the Vaccine Adverse Event Reporting System), all among primary vaccinees (35 men and 1 woman). Each was treated symptomatically (eg, with antihistamines, analgesics, antipyretics) and returned to duty. Nine were hospitalized for observation, notably by clinicians seeing their first such patient. Erythema multiforme major confirmed by skin biopsy developed in a 20-year-old service member 19 days after receiving smallpox and several other vaccines. This case was deemed to possibly be associated with smallpox vaccination, given multiple possible causes.

Thirty-eight reports of inadvertent implantation ("autoinoculation") of the skin were submitted, with varying certainty of association to vaccinia. Affected sites included chest, cheek, arm, finger, wrist, genitals, groin, and mouth. Ten others involved possible or probable ocular vaccinia, typically treated with trifluridine eye drops. Four (skin=3, eye=1) were confirmed by culture and polymerase chain reaction (PCR) test.

We identified 21 instances of contact transfer of vaccinia, all involving primary vaccinees, in 306 673 person-months of potential contact with vaccinia. These included 10 spouses, 2 children, 5 intimate contacts, and 4 friends. No cases of contact vaccinia were observed in patients. The most commonly named mechanism involved failure to observe bandaging discipline (eg, touching vaccination sites, touching bandages). Most cases were clinically mild, manifesting as pustules on arms, shoulders, or hips. In 2 cases, conjunctivitis or blepharitis developed; each was treated with trifluridine. One was treated with vaccinia immune globulin (VIG) and discharged from the hospital the following day.[12] Hundreds of thousands of vaccinees flew from North America to southwest Asia before their vaccinial scabs had fallen off, yet no spread to passengers or flight attendants was reported.

Two cases of encephalitis as defined by attending physicians presented 7 and 9 days after vaccination, the first in a 23-year-old after primary vaccination and the second in a 38-year-old after revaccination. In both cases, the patients recovered and returned to duty. The first case met objective criteria for encephalitis but the second did not. Diagnostic studies in the first case by viral culture and PCR did not establish vaccinia or 9 other viruses as a cause. Twenty-three other neurologic events occurred, 1 fatal. Their association to vaccination is unclear, given multiple possible causes (neuromuscular weakness or palsy=6, seizures=6, meningitis=4, optic neuritis=2, demyelinating conditions [eg, Guillain-Barré syndrome]=2, altered cognition=2, transient visual defect=1).

Thirty-seven suspected, probable, or confirmed cases diagnosed as acute myopericarditis developed in male primary vaccinees aged 21 to 33 years at 7 to 19 days after vaccination. In each of the initial 18 cases described by Halsell et al,[11] the patient presented with chest pain, and elevated cardiac enzymes were found;

Table 3. Noteworthy Adverse Events After Smallpox Vaccination

Event Type	No. of Events	DoD Rate per Million Vaccinees (95% Confidence Interval)	Historical Rate per Million Vaccinees
Mild or temporary			
Generalized vaccinia, mild	36	80 (63-100)	45-212*
Erythema multiforme	1	NA	NA
Inadvertent inoculation, self	48†	107 (88-129)	606*
Vaccinia transfer to contact	21	47 (35-63)	8-27*
Moderate or serious			
Encephalitis	1	2.2 (0.6-7.2)	2.6-8.7*
Acute myopericarditis	37	82 (65-102)	100‡
Eczema vaccinatum	0	0 (0-3.7)	2-35*
Progressive vaccinia	0	0 (0-3.7)	1-7*
Death	0	0 (0-3.7)	1-2*

Abbreviations: DoD, US Department of Defense; NA, not available.
*Based on adolescent and adult smallpox vaccinations from 1968 studies (both primary and revaccination).[27-29]
†Includes 38 inadvertent innoculations of the skin and 10 of the eye.
‡Based on case series in Finnish military recruits given the Finnish strain of smallpox vaccine.[30]

electrocardiogram (ECG) and echocardiogram findings varied. Clinical conditions ranged from mild (no ECG or echocardiogram changes) to severe (1 case of transient lowered ejection fraction).[13] All survived and either have or are expected to return to duty.

Eight additional cardiac events occurred 2 to 12 days after vaccination (myocardial infarction=4, angina=2, coronary artery spasm=1, atrial fibrillation=1). A 55-year-old male service member died after a myocardial infarction 5 days after vaccination. Autopsy findings showed 3-vessel coronary occlusion, left ventricular hypertrophy, and cardiomegaly.[13]

Despite extensive efforts to avoid vaccinating women who were pregnant, 85 of 62622 vaccine-eligible women (0.14%) were vaccinated before they knew they were pregnant. Once found to be pregnant, these women were offered clinical support and medical counseling, and enrolled in a prospective registry.[9,14]

Ten male service members aged 23 to 37 years were vaccinated before recognition that they were infected with HIV. Each responded successfully to vaccination and their sites healed at the expected pace. Later studies showed that their CD4 cell counts ranged from 303 to 751 cells/μL shortly after vaccination.

One service member received extensive burns 5 days after vaccination. To preclude vaccinial complications of his recovery, this man received VIG prophylactically. He developed new vesicles 13 days after receiving VIG, but the vesicles tested negative for vaccinia by PCR and culture. Another burned service member whose vaccination site had scabbed over was not treated with VIG. He did not develop other vaccinial lesions.

No cases of eczema vaccinatum or progressive vaccinia occurred. We attribute no deaths to smallpox vaccination to date.[13]

COMMENT

Smallpox affected soldiers and sailors for centuries.[4,8,15,16] United States service members received smallpox vaccination from the War of 1812 until 1990.[17,18] A 1977 report notes 54 hospitalizations per 1 million vaccinations (mainly secondary infections).[19] Between 1965 and 1975, the US Air Force recorded 6 encephalitis cases after vaccination.[19] Service members spread vaccinia to several civilians in the 1980s.[20-23] After extensive searching, we could not confirm any deaths due to smallpox vaccination of military personnel since 1943.[17-19,24-26]

Surveillance of the current US military smallpox vaccination program identified an increased risk of myopericarditis after smallpox vaccination.[11] We believe myopericarditis resulted from smallpox vaccination in the 1960s but was underrecognized because cardiac enzyme and imaging technologies were still being developed. We observed ischemic events after vaccination but at a rate that was less than the age-adjusted rate expected among unvaccinated military personnel. Allowing for underreporting, we found no causal association between vaccination and ischemic events.

The incidence of adverse events in Table 3 reflects rates largely below those reported in the 1960s and 1970 (although such comparisons must be interpreted with caution given differences in age, prior vaccination, population immunity, and other variables).[27-29] In addition, underreporting may affect our rates and historical rates,[27-31] but our global communications and surveillance would tend to reduce underreporting. Military commanders were vigorous in identifying troops needing treatment. Teleconsultation identified noteworthy cases that may have been missed in earlier eras. Historical rates are not directly applicable to contact transfer because of differences in population immunity and underlying disease.

Our individualized screening for contraindications may explain the lack of eczema vaccinatum or progressive vaccinia.[6,7] Extensive education may have contributed to low rates of inadvertent inoculation. Interestingly, reports of inadvertent inoculation from the United States did not differ substantially from rates reported in Asia, where sanitary conditions are rudimentary. Medical officers attributed the low rates overseas to disciplined wearing of long sleeves, attention to personal hygiene, and training. We observed 1 probable case of autoinoculation among 27801 military health care workers, comparable with the 3 confirmed cases reported among 34541 civilian health care workers.[32]

Successful implementation of bandage evaluation stations in our health care settings validates this approach.[6,7] Our experience suggests that no disruption of routine clinical care need occur after vaccination given vigorous infection control.

Similarly, we observed a low rate of contact transfer of vaccinia, despite the close living conditions associated with mobilization and deployment. These conditions are known risk factors for other contagious diseases. Most vaccine recipients used simple bandages, not semipermeable dressings, suggesting the primacy of human factors and postvaccination discipline. Health care workers in both military and civilian settings have been comparably successful in not spreading vaccinia to their contacts.[32]

This descriptive report cannot establish the efficacy of the screening, educational, and surveillance methods used because it lacks an explicit control group. Additional efforts will be needed to evaluate the relative efficiency of alternative approaches.

The DoD is responsible for protecting and preserving the health of US service members. We vaccinated with this responsibility in mind. Because smallpox vaccine is associated with temporary unpleasant symptoms and carries a small risk of serious adverse events, we sought to implement our program with due attention to safety. We offer our experience to help the civilian community.

Author Contributions: *Study concept and design:* Grabenstein, Winkenwerder.
Acquisition of data: Grabenstein.
Analysis and interpretation of data: Grabenstein, Winkenwerder.
Drafting of the manuscript: Grabenstein, Winkenwerder.

Critical revision of the manuscript for important intellectual content: Grabenstein, Winkenwerder.
Statistical expertise: Grabenstein, Winkenwerder.
Obtained funding: Grabenstein, Winkenwerder.
Administrative, technical, or material support: Grabenstein, Winkenwerder.
Study supervision: Grabenstein, Winkenwerder.

Disclaimer: The views expressed in this article are those of the authors and do not reflect the official policy or position of the Department of the Defense or the US Government. This work is approved for public release.

Acknowledgment: We gratefully acknowledge the health care providers, planners, laboratorians, and especially the medics implementing the smallpox vaccination program around the world, especially Douglas G. Smith, MD, Kevin L. Pehr, MD, Jason A. Lees, MD, Larry J. Godfrey, MD, US Central Command; George W. Christopher, MD, Landstuhl Regional Medical Center, Landstuhl, Germany; Renata J. M. Engler, MD, Lisa A. Black, DO, Thomas J. White, MBA, Scott A. Norton, MD, K. Scott Bower, MD, William P. Madigan, MD, Walter Reed Army Medical Center, Washington, DC; Timothy P. Endy, MD, Niranjan Kanesa-Thasan, MD, US Army Medical Research Institute of Infectious Diseases, Fort Detrick, Md; Caroline C. DeWitt, MD, John P. Dice, MD, Wilford Hall Medical Center, Lackland Air Force Base, San Antonio, Tex; Kylee V. Sutherlin, RN, Aberdeen Proving Ground, Md; Richard D. Ares, PA-C, Schofield Barracks, Oahu, Hawaii; Margaret A. K. Ryan, MD, Naval Health Research Center, San Diego, Calif; James R. Riddle, DVM, Armed Forces Epidemiological Board, Falls Church, Va; Benedict M. Diniega, MD, Kenneth Hoffman, MD, Roger L. Gibson, DVM, PhD, Office of the Secretary of Defense, Falls Church, Va; and Christine G. Casey, MD, Penina Haber, MPH, CDC, Atlanta, Ga.

REFERENCES

1. Centers for Disease Control and Prevention. Smallpox response plan and guidelines. September 23, 2002. Available at: http://www.bt.cdc.gov/DocumentsApp/Smallpox/RPG/. Accessed March 5, 2003.
2. US Army Regulation 40-562; Navy Bureau of Medicine and Surgery Instruction 6230.15; Air Force Joint Instruction 48-110; Coast Guard Commandant Instruction M6230.4E. *Immunizations & Chemoprophylaxis.* Washington, DC: US Dept of Defense; November 1, 1995.
3. US Department of Defense smallpox response plan. September 29, 2002. Available at: http://www.smallpox.army.mil/resource/SMAplan/SMAplan.asp. Accessed March 5, 2003.
4. Smallpox vaccine, dried, calf lymph type (Dryvax) [product labeling]. Marietta, Pa: Wyeth Laboratories; October 25, 2002. Available at: http://www.fda.gov/cber/label/smalwye102502LB.pdf. Accessed March 5, 2003.
5. Advisory Committee on Immunization Practices. Vaccinia (smallpox) vaccine. *MMWR Morb Mortal Wkly Rep.* 2001;50(RR-10):1-25.
6. Advisory Committee on Immunization Practices, Healthcare Infection Control Practices Advisory Committee. Recommendations for using smallpox vaccine in a pre-event vaccination program: supplemental recommendations. *MMWR Morb Mortal Wkly Rep.* 2003;52(RR-7):1-16.
7. Centers for Disease Control and Prevention. Smallpox vaccination and adverse events: guidance for clinicians. *MMWR Morb Mortal Wkly Rep.* 2003;52(RR-4):1-30.
8. Fenner F, Henderson DA, Arita I, Jezek Z, Ladnyi ID. *Smallpox and Its Eradication.* Geneva, Switzerland: World Health Organization; 1988.
9. Centers for Disease Control and Prevention. National Smallpox Vaccine in Pregnancy Registry. *MMWR Morb Mortal Wkly Rep.* 2003;52:256.
10. Institute of Medicine. *Review of the Centers for Disease Control & Prevention's Smallpox Vaccination Program Implementation—Letter Report #1.* Washington, DC: National Academy of Sciences; January 2003.
11. Halsell JS, Riddle JR, Atwood JE, et al, and the Department of Defense Smallpox Vaccination Clinical Evaluation Team. Myopericarditis following smallpox vaccination among vaccinia-naive US military personnel. *JAMA.* 2003;289:3283-3289.
12. Centers for Disease Control and Prevention. Smallpox vaccine adverse events among civilians—United States, February 25 to March 3, 2003. *MMWR Morb Mortal Wkly Rep.* 2003;52:180-181, 191.
13. Centers for Disease Control and Prevention. Update: adverse events following smallpox vaccination—United States, 2003. *MMWR Morb Mortal Wkly Rep.* 2003;52:278-282.
14. Centers for Disease Control and Prevention. Women with smallpox vaccine exposure during pregnancy reported to the National Smallpox Vaccine in Pregnancy Registry—United States, 2003. *MMWR Morb Mortal Wkly Rep.* 2003;52:386-388.
15. Gillett MC. *The Army Medical Department: 1775-1818.* Washington, DC: US Army Center of Military History; 1981:56-65, 74-75, 82-89, 191.
16. Duncan LC. The days gone by: sketches of the medical service in the War of 1812. *Milit Surg.* 1932;71:439.
17. Coates JB Jr, Hoff EC, eds. *Medical Department, US Army, in World War II—Preventive Medicine in World War II, Volume III: Personal Health Measures and Immunization.* Washington, DC: US Dept of the Army; 1955:280-287, 351-353.
18. Coates JB Jr, Hoff EC, eds. *Medical Department, US Army, in World War II—Preventive Medicine in World War II, Volume VII: Communicable Diseases—Arthropodborne Diseases Other Than Malaria.* Washington, DC: US Dept of the Army; 1964:17-18.
19. Armed Forces Epidemiological Board. *Report of the Ad Hoc Subcommittee on Smallpox.* Falls Church, Va: Armed Forces Epidemiological Board; May 12, 1977.
20. Centers for Disease Control. Disseminated vaccinia infection in a college student—Tennessee. *MMWR Morb Mortal Wkly Rep.* 1982;31:682-683.
21. Centers for Disease Control. Vaccinia outbreak—Nevada. *MMWR Morb Mortal Wkly Rep.* 1983;32:403-404.
22. Centers for Disease Control. Contact spread of vaccinia from a recently vaccinated Marine—Louisiana. *MMWR Morb Mortal Wkly Rep.* 1984;33:37-38.
23. Centers for Disease Control. Contact spread of vaccinia from a National Guard vaccinee—Wisconsin. *MMWR Morb Mortal Wkly Rep.* 1985;34:182-183.
24. Lane JM, Millar JD, Neff JM. Smallpox and smallpox vaccination policy. *Annu Rev Med.* 1971;22:251-272.
25. Armed Forces Epidemiological Board. *Minutes of the June 11, 1981, Meeting.* Falls Church, Va: Armed Forces Epidemiological Board; June 11, 1981.
26. Redfield RR, Wright DC, James WD, Jones TS, Brown C, Burke DS. Disseminated vaccinia in a military recruit with human immunodeficiency virus (HIV) disease. *N Engl J Med.* 1987;316:673-676.
27. Lane JM, Ruben FL, Neff JM, Millar JD. Complications of smallpox vaccination, 1968: national surveillance in the United States. *N Engl J Med.* 1969;281:1201-1208.
28. Lane JM, Ruben FL, Neff JM, Millar JD. Complications of smallpox vaccination, 1968: results of ten statewide surveys. *J Infect Dis.* 1970;122:303-309.
29. Lane JM, Millar JD. Risks of smallpox vaccination complications in the United States. *Am J Epidemiol.* 1971;93:238-240.
30. Karjalainen J, Heikkila J, Nieminen MS, et al. Etiology of mild acute infectious myocarditis. *Acta Med Scand.* 1983;213:65-73.
31. Lane JM, Goldstein J. Evaluation of 21st-century risks of smallpox vaccination and policy options. *Ann Intern Med.* 2003;138:488-493.
32. Centers for Disease Control and Prevention. Adverse events following civilian smallpox vaccination—United States, 2003. *MMWR Morb Mortal Wkly Rep.* 2003;52:419-420.

European Medicines Agency

November 2005
CHMP/ICH/2/04

ICH Topic E 14
The Clinical Evaluation of QT/QTc Interval Prolongation and Proarrhythmic Potential for Non-Antiarrhythmic Drugs

Step 5

NOTE FOR GUIDANCE ON THE CLINICAL EVALUATION OF QT/QTc INTERVAL PROLONGATION AND PROARRHYTHMIC POTENTIAL FOR NON-ANTIARRHYTHMIC DRUGS
(CHMP/ICH/2/04)

TRANSMISSION TO CHMP	June 2004
TRANSMISSION TO INTERESTED PARTIES	June 2004
DEADLINE FOR COMMENTS	December 2004
FINAL APPROVAL BY CHMP	May 2005
DATE FOR COMING INTO OPERATION	November 2005

7 Westferry Circus, Canary Wharf, London, E14 4HB, UK
Tel. (44-20) 74 18 85 75 Fax (44-20) 75 23 70 40
E-mail: mail@emea.eu.int http://www.emea.eu.int
©EMEA 2006 Reproduction and/or distribution of this document is authorised for non commercial purposes only provided the EMEA is acknowledged

THE CLINICAL EVALUATION OF QT/QTc INTERVAL PROLONGATION AND PROARRHYTHMIC POTENTIAL FOR NON-ANTIARRHYTHMIC DRUGS

TABLE OF CONTENTS

1. INTRODUCTION 3

1.1 BACKGROUND 3
1.2 OBJECTIVES 3
1.3 SCOPE 4

2. CLINICAL TRIALS 4

2.1 OVERALL APPROACHES TO EVALUATING DRUG EFFECTS ON QT/QTC INTERVAL 4
2.1.1 Subject Enrollment 4
2.1.2 Safety Monitoring, and Discontinuation Criteria 4
2.2 The 'Thorough QT/QTc Study' 4
2.2.1 Design of the 'Thorough QT/QTc Study' 5
2.2.2 Dose-Effect and Time Course Relationships in the 'Thorough QT/QTc Study' 6
2.2.3 Timing of ECGs in the 'Thorough QT/QTc Study' 6
2.2.4 Interpretation of the 'Thorough QT/QTc Study' 6
2.2.5 Alternative Strategies to Assess QT/QTc Interval Effects 7
2.3 CLINICAL TRIAL EVALUATION AFTER THE 'THOROUGH QT/QTC STUDY' 7
2.5 COLLECTION, ASSESSMENT AND SUBMISSION OF ELECTROCARDIOGRAPHIC DATA 8
2.5.1 Collection and Assessment of Standard 12-Lead ECGs 8
2.5.2 Ambulatory ECG Monitoring 8
2.5.3 Submission of Interval and Waveform Data 9

3. ANALYSIS OF ECG DATA FROM CLINICAL TRIALS 9

3.1 QT INTERVAL CORRECTION FORMULAE 9
3.1.1 Population-Derived Correction Formulae 9
3.1.2 Correction Formulae Derived from Within-Subject Data 10
3.2 ANALYSIS OF QT/QTC INTERVAL DATA 10
3.2.1 Analyses of Central Tendency 10
3.2.2 Categorical Analyses 10
3.2.3 Analysis of Relationship Between Drug Exposure and QT/QTc Interval Changes 11
3.3 MORPHOLOGICAL ANALYSES OF ECG WAVEFORMS 11

4. ADVERSE EVENTS 11

4.1 CLINICAL TRIAL ADVERSE EVENTS 11
4.2 PREMATURE DISCONTINUATIONS OR DOSAGE REDUCTIONS 12
4.3 PHARMACOGENETIC CONSIDERATIONS 12
4.4 POST-MARKETING ADVERSE EVENT REPORTS 12

5. REGULATORY IMPLICATIONS, LABELLING, AND RISK MANAGEMENT STRATEGIES 13

5.1 RELEVANCE OF QT/QTC INTERVAL PROLONGING EFFECTS TO THE EVALUATION PROCESS 13
5.2 LABELLING ISSUES FOR DRUGS THAT PROLONG THE QT/QTC INTERVAL 13
5.3 POST-MARKETING RISK MANAGEMENT FOR DRUGS THAT PROLONG THE QT/QTC INTERVAL 14

© EMEA 2006

THE CLINICAL EVALUATION OF QT/QTc INTERVAL PROLONGATION AND PROARRHYTHMIC POTENTIAL FOR NON-ANTIARRHYTHMIC DRUGS

1. INTRODUCTION

1.1 Background

An undesirable property of some non-antiarrhythmic drugs is their ability to delay cardiac repolarization, an effect that can be measured as prolongation of the QT interval on the surface electrocardiogram (ECG). The QT interval represents the duration of ventricular depolarization and subsequent repolarization, and is measured from the beginning of the QRS complex to the end of the T wave. A delay in cardiac repolarization creates an electrophysiological environment that favors the development of cardiac arrhythmias, most clearly torsade de pointes (TdP), but possibly other ventricular tachyarrhythmias as well. TdP is a polymorphic ventricular tachyarrhythmia that appears on the ECG as continuous twisting of the vector of the QRS complex around the isoelectric baseline. A feature of TdP is pronounced prolongation of the QT interval in the supraventricular beat preceding the arrhythmia. TdP can degenerate into ventricular fibrillation, leading to sudden death.

While the degree of QT prolongation is recognized as an imperfect biomarker for proarrhythmic risk, in general there is a qualitative relationship between QT prolongation and the risk of TdP, especially for drugs that cause substantial prolongation of the QT interval. Because of its inverse relationship to heart rate, the measured QT interval is routinely corrected by means of various formulae to a less heart rate dependent value known as the QTc interval. It is not clear, however, whether arrhythmia development is more closely related to an increase in the absolute QT interval or QTc. Most drugs that have caused TdP clearly increase both the absolute QT and the QTc (hereafter called QT/QTc). Documented cases of TdP (fatal and non-fatal) associated with the use of a drug have resulted in the withdrawal from the market of several drugs and relegation of other drugs to second-line status. Because prolongation of the QT/QTc interval is the ECG finding associated with the increased susceptibility to these arrhythmias, an adequate pre-marketing investigation of the safety of a new pharmaceutical agent should include rigorous characterization of its effects on the QT/QTc interval.

1.2 Objectives

This document provides recommendations to sponsors concerning the design, conduct, analysis, and interpretation of clinical studies to assess the potential of a drug to delay cardiac repolarization. This assessment should include testing the effects of new agents on the QT/QTc interval as well as the collection of cardiovascular adverse events. The investigational approach used for a particular drug should be individualized, depending on the pharmacodynamic, pharmacokinetic, and safety characteristics of the product, as well as on its proposed clinical use.

The assessment of the effects of drugs on cardiac repolarization is the subject of active investigation. When additional data (non-clinical and clinical) are accumulated in the future, this document may be reevaluated and revised.

© EMEA 2006

1.3 Scope

The recommendations contained in this document are generally applicable to new drugs having systemic bioavailability, but may not apply to products with highly localized distribution and those administered topically and not absorbed. The focus is on agents being developed for uses other than the control of arrhythmias, as antiarrhythmic drugs can prolong the QT/QTc interval as a part of their mechanism of clinical efficacy. While this document is concerned primarily with the development of novel agents, the recommendations might also be applicable to approved drugs when a new dose or route of administration is being developed that results in significantly higher exposure (i.e., C_{max} or AUC). Additional ECG data might also be considered appropriate if a new indication or patient population were being pursued. The evaluation of the effect of a drug on the QT interval would also be considered important if the drug or members of its chemical or pharmacological class have been associated with QT/QTc interval prolongation, TdP, or sudden cardiac death during post-marketing surveillance.

2. CLINICAL TRIALS

2.1 Overall Approaches to Evaluating Drug Effects on QT/QTc Interval

Drugs are expected to receive a clinical electrocardiographic evaluation, beginning early in clinical development, typically including a single trial dedicated to evaluating their effect on cardiac repolarization ('thorough QT/QTc study'). Factors that could reduce the need for such a study include the inability to conduct the study in healthy volunteers or patients, how the drug is studied and used (e.g., administered under continuous monitoring), as well as nonclinical data.

2.1.1 Subject Enrollment

Subject enrollment for a given trial would be influenced by the clinical and non-clinical information available on the effects of the drug on cardiac repolarization. Until the effects of the drug on the QT/QTc interval have been characterized, the following exclusion criteria are suggested:

- A marked baseline prolongation of QT/QTc interval (e.g., repeated demonstration of a QTc interval >450 ms);
- A history of additional risk factors for TdP (e.g., heart failure, hypokalemia, family history of Long QT Syndrome);
- The use of concomitant medications that prolong the QT/QTc interval.

If supported by the QT/QTc interval data from the early clinical studies, later clinical trials could expand the eligibility criteria to include a broader spectrum of patients who are likely to receive the drug once approved.

2.1.2 Safety Monitoring, and Discontinuation Criteria

The clinical trial protocol should specify the procedures to follow if a patient experiences an adverse event suggestive of TdP.

Discontinuation of a subject from a clinical trial should be considered if there is a marked prolongation of the QT/QTc interval during treatment with the study drug, especially if the measurement is obtained from more than one ECG. While increases in QT/QTc to >500 ms or of >60 ms over baseline are commonly used as thresholds for potential discontinuation, the exact criteria chosen for a given trial will depend on the risk-tolerance level considered appropriate for the indication and patient group in question.

2.2 The 'Thorough QT/QTc Study'

The 'thorough QT/QTc study' is intended to determine whether the drug has a threshold pharmacologic effect on cardiac repolarization, as detected by QT/QTc prolongation. The threshold level of regulatory concern, discussed further below, is around 5 ms as evidenced by an upper bound of the 95% confidence interval around the mean effect on QTc of 10 ms. The

© EMEA 2006

study is typically carried out in healthy volunteers (as opposed to individuals at increased risk of arrhythmias) and is used to determine whether or not the effect of a drug on the QT/QTc interval in target patient populations should be studied intensively during later stages of drug development. It is not intended to identify drugs as being pro-arrhythmic. Although data are limited, it is not expected that the results of the 'thorough QT/QTc study' would be affected by ethnic factors.

The 'thorough QT/QTc study' would typically be conducted early in clinical development to provide maximum guidance for later trials, although the precise timing will depend on the specifics of the drug under development. It would usually not be the first study, as it is important to have basic clinical data for its design and conduct, including tolerability and pharmacokinetics. Some drugs might not be suitable for study in healthy volunteers because of issues related to tolerability (e.g., neuroleptic agents, chemotherapeutics).

The results of the 'thorough QT/QTc study' will influence the amount of information collected in later stages of development:

- A negative 'thorough QT/QTc study' will almost always allow the collection of on-therapy ECGs in accordance with the current practices in each therapeutic area to constitute sufficient evaluation during subsequent stages of drug development (see section 2.3);
- A positive 'thorough QT/QTc study' will almost always call for an expanded ECG safety evaluation during later stages of drug development (see section 2.3).

There could be very unusual cases in which the 'thorough QT/QTc study' is negative but the available nonclinical data are strongly positive (e.g., hERG positive at low concentrations and *in vivo* animal model results that are strongly positive). If this discrepancy cannot be explained by other data, and the drug is in a class of pharmacological concern, expanded ECG safety evaluation during later stages of drug development might be appropriate (section 2.3).

2.2.1 Design of the 'Thorough QT/QTc Study'

The 'thorough QT/QTc study' should be adequate and well-controlled, with mechanisms to deal with potential bias, including use of randomization, appropriate blinding, and concurrent placebo control group. As this study has a critical role in determining the intensity of ECG data collection during later stages of drug development, it is important to have a high degree of confidence in the ability of the study to detect differences of clinical significance. The confidence in the ability of the study to detect QT/QTc prolongation can be greatly enhanced by the use of a concurrent positive control group (pharmacological or non-pharmacological) to establish assay sensitivity. The positive control should have an effect on the mean QT/QTc interval of about 5 ms (i.e., an effect that is close to the QT/QTc effect that represents the threshold of regulatory concern, around 5 ms). Detecting the positive control's effect will establish the ability of the study to detect such an effect of the study drug. Absence of a positive control should be justified and alternative methods to establish assay sensitivity provided.

If an investigational drug belongs to a chemical or pharmacological class that has been associated with QT/QTc prolongation, a positive control selected from other members of the same class should be considered to permit a comparison of effect sizes, preferably at equipotent therapeutic doses.

Crossover or parallel group study designs can be suitable for trials assessing the potential of a drug to cause QT/QTc interval prolongation. Crossover studies at least have two potential advantages:

- They usually call for smaller numbers of subjects than parallel group studies, as the subjects serve as their own controls and hence reduce variability of differences related to inter-subject variability;
- They might facilitate heart rate correction approaches based on individual subject data.

Parallel group studies might be preferred under certain circumstances:

- For drugs with long elimination half-lives for which lengthy time intervals would be required to achieve steady-state or complete washout;
- If carryover effects are prominent for other reasons, such as irreversible receptor binding or long-lived active metabolites;
- If multiple doses or treatment groups are to be compared.

An important problem in the measurement of the QT/QTc interval is its intrinsic variability. This variability results from many factors, including activity level, postural changes, circadian patterns, and food ingestion. It is critical to address intrinsic variability in the conduct of the 'thorough QT/QTc study'. This can be accomplished in several ways, including the collection of multiple ECGs at baseline and during the study.

2.2.2 Dose-Effect and Time Course Relationships in the 'Thorough QT/QTc Study'

An adequate drug development programme should ensure that the dose-response and generally the concentration-response relationship for QT/QTc prolongation have been characterized, including exploration of concentrations that are higher than those achieved following the anticipated therapeutic doses. Data on the drug concentrations around the time of ECG assessment would aid this assessment. If not precluded by considerations of safety or tolerability due to adverse effects, the drug should be tested at substantial multiples of the anticipated maximum therapeutic exposure. Alternatively, if the concentrations of a drug can be increased by drug-drug or drug-food interactions involving metabolizing enzymes (e.g., CYP3A4, CYP2D6) or transporters (e.g., P-glycoprotein), these effects could be studied under conditions of maximum inhibition. This approach calls for an understanding of the pharmacokinetic and pharmacodynamic properties of the parent and significant human metabolites. In general, the duration of dosing or dosing regimen should be sufficient to characterize the effects of the drug and its active metabolites at relevant concentrations.

2.2.3 Timing of ECGs in the 'Thorough QT/QTc Study'

The timing of the collection of ECGs and the study design (e.g., single or multiple dose, duration) of the 'thorough QT/QTc study' should be guided by the available information about the pharmacokinetic profile of the drug. For drugs with short half-lives and no metabolites, a single dose study might be sufficient. Studies should characterize the effect of a drug on the QT/QTc throughout the dosing interval. While the peak serum concentration does not always correspond to the peak effect on QT/QTc interval, care should be taken to perform ECG recordings at time points around the Cmax. As one intent of a positive control is to establish assay sensitivity, in multiple dose studies of new drugs a positive control needs to be used only long enough to have its expected effect.

2.2.4 Interpretation of the 'Thorough QT/QTc Study'

It is difficult to determine whether there is an effect on the mean QT/QTc interval that is so small as to be of no consequence. However, drugs that prolong the mean QT/QTc interval by around 5 ms or less do not appear to cause TdP. On that basis, the positive control (whether pharmacological or non-pharmacological) should be well-characterized and consistently produce an effect on the QT/QTc interval that is around the threshold of regulatory concern (5 ms, section 2.2).

Based on similar considerations, a negative 'thorough QT/QTc study' is one in which the upper bound of the 95% one-sided confidence interval for the largest time-matched mean

effect of the drug on the QTc interval excludes 10 ms. This definition is chosen to provide reasonable assurance that the mean effect of the study drug on the QT/QTc interval is not greater than around 5 ms. When the largest time-matched difference exceeds the threshold, the study is termed 'positive'. A positive study influences the evaluations carried out during later stages of drug development, but does not imply that the drug is pro-arrhythmic.

As with other data, the presence of outliers (see section 3.2.2) should also be explored.

2.2.5 Alternative Strategies to Assess QT/QTc Interval Effects

Alternatives to the use of the 'thorough QT/QTc study' are under active investigation. Examples include evaluating the relationship between concentration and QT/QTc effects or more intensively evaluating ECGs, based on data collected during early phase clinical studies.

2.3 Clinical Trial Evaluation After the 'Thorough QT/QTc Study'

If the 'thorough QT/QTc study' is negative (see section 2.2), the collection of baseline and periodic on-therapy ECGs in accordance with the current investigational practices in each therapeutic field is almost always sufficient evaluation during subsequent stages of drug development.

If the 'thorough QT/QTc study' is positive, additional evaluation in subsequent clinical studies should be performed. One objective of this evaluation should be to fully describe the effect of the drug on the QT/QTc interval in the target patient population with particular attention to dose- and concentration-related effects. It is important to include patients in these analyses exposed to the full range of potential doses and patients with additional risk factors for TdP. These analyses would ordinarily focus on outliers as well as on changes in mean QT/QTc. Depending on the size of the effect seen in the 'thorough QT/QTc study', more intense monitoring of patients with additional risk factors for TdP might be needed.

While what constitutes adequate ECG assessment to accomplish this is not fully established, additional ECGs collected appropriately in substantial numbers of patients in late phase clinical trials could provide this information. In this case, it would be important to collect ECGs at the anticipated time of peak drug effects, as determined in the 'thorough QT/QTc study' and pharmacokinetic information about the patient population.

Another objective of this evaluation should be to collect information on the adverse events that occur in the trials following the positive 'thorough QT/QTc study'. This would include patients who develop marked QT/QTc prolongation (e.g., >500 ms) or experience a serious cardiovascular adverse event that suggests an arrhythmia (e.g., TdP). Such patients should be evaluated closely for risk factors that might have contributed to this event (e.g., genotyping for Long QT Syndromes, see section 4.3).

If the 'thorough QT/QTc study' is positive, analyses of the ECG and adverse event data from certain patient sub-groups are of particular interest, such as:

- Patients with electrolyte abnormalities (e.g., hypokalemia);
- Patients with congestive heart failure;
- Patients with impaired drug metabolizing capacity or clearance (e.g., renal or hepatic impairment, drug interactions);
- Female patients;
- Patients aged <16 and over 65 years.

Even if the 'thorough QT/QTc study' is negative, if other evidence of an effect in a patient population from subsequent studies (e.g., marked QT/QTc interval prolongation, TdP) were to emerge, then additional investigation would be needed.

© EMEA 2006

2.4 Clinical Development When the 'Thorough QT/QTc Study' Cannot be Performed in Healthy Volunteers

There are some drugs that cannot be studied in a 'thorough QT/QTc study' in healthy volunteers due to safety or tolerability concerns (e.g., cytotoxic cancer drugs). In such cases, the 'thorough QT/QTc study' can often be conducted in patient populations. When this is not possible, the importance of detecting and modifying this safety risk means that other ways of detecting effects on the QT/QTc interval need to be developed. These might include the collection of ECGs at multiple timepoints under tightly controlled settings that target a broad range of doses early in development.

2.5 Collection, Assessment and Submission of Electrocardiographic Data

The recommendations below are most relevant to the 'thorough QT/QTc study' and to any studies investigating a drug with a known effect on cardiac repolarization and when the 'thorough QT/QTc study' cannot be performed.

2.5.1 Collection and Assessment of Standard 12-Lead ECGs

The clinical ECG database is typically derived from the collection of 12-lead surface ECGs, although ambulatory ECG techniques show promise (see section 2.5.2). The quality of the ECG database can depend on the use of modern equipment with the capacity for digital signal processing. Such equipment should be recently serviced and calibrated. Machine calibration records and performance data should be maintained on file. In the case of multicentre trials, training sessions are encouraged to ensure consistency of operator technique (e.g., skin preparation, lead placement, patient position) and data acquisition practices.

Several methods for measuring ECG intervals have been used in clinical trials, and for a given trial, the sponsor should describe the accuracy and precision of QT/QTc interval measurements using the selected system. The method chosen will depend on the level of precision appropriate for a given trial. For example, the 'thorough QT/QTc study' would warrant particularly careful attention to interval measurement. At present, this would usually involve the measurement by a few skilled readers (whether or not assisted by computer) operating from a centralized ECG laboratory. If well-characterized data validating the use of fully-automated technologies become available, the recommendations in the guidance for the measurement of ECG intervals could be modified. Readers of ECGs should be blinded to time, treatment and subject identifier, and one reader should read all the ECG recordings from a given subject. The degree of inter- and intra-reader variability should be established by having the assessors reread a subset of the data (both normal and abnormal) under blinded conditions.

In the absence of a concern in the early clinical trial(s), ECG reading by machine have a role in the rapid assessment of ECGs for safety. Criteria for ECG diagnoses and for identification of adverse events should be pre-defined by the sponsor.

While the most appropriate lead(s) and methodology to measure the QT interval have not been established, pre-cordial leads and lead II are often used. A consistent approach should be used for a given trial.

Morphological changes in the T-U complex might occur. Information should be provided on changes in T and U wave morphologies (see section 3.3).

Discrete U waves should be excluded from the QT/QTc interval measurement.

2.5.2 Ambulatory ECG Monitoring

While ambulatory ECG monitoring has historically not been considered sufficiently validated to be used as the primary assessment of QT/QTc interval, newer systems that allow for the collection of multiple leads that more closely approximate a surface ECG have potential value to collect interval data. The use of ambulatory ECG monitors might additionally allow detection of extreme QT/QTc interval events that occur infrequently during the day and

© EMEA 2006

asymptomatic arrhythmias. Data on the QT/RR from ambulatory ECG monitoring can also prove useful in the calculation of individualized QT corrections. However, as QT/QTc intervals measured by this methodology might not correspond quantitatively to those from standard surface ECGs, data obtained from the two methodologies might not be suitable for direct comparison, pooling, or interpretation using the same thresholds of concern.

2.5.3 Submission of Interval and Waveform Data

Regional guidance should be sought for information on the submission of ECG interval data and overall assessments.

3. ANALYSIS OF ECG DATA FROM CLINICAL TRIALS

Evaluation of the effects of a drug on the standard ECG intervals and waveforms is considered a fundamental component of the safety database of any new drug application.

Regardless of the outcome of the 'thorough QT/QTc study', ECG changes recorded as adverse events should be pooled from all studies for analysis. ECG interval data from the 'thorough QT/QTc study' should only be pooled with subsequent trials of similar rigor with regard to ECG data collection and analysis, but should not be pooled with trials using less rigorous ECG collection. Standardization of ECG collection for similar studies within a clinical trial programme will facilitate pooled analyses.

3.1 QT Interval Correction Formulae

As the QT interval has an inverse relationship to heart rate, the measured QT intervals are generally corrected for heart rate in order to determine whether they are prolonged relative to baseline. Various correction formulae have been suggested, of which Bazett's and Fridericia's corrections are the most widely used. In early trials evaluating the effects of a new drug on the QT/QTc interval in healthy volunteers, designed to detect relatively small effects (e.g., 5 ms), it is important to apply the most accurate correction available.

Because the best correction approach is a subject of controversy, uncorrected QT and RR interval data, heart rate data, as well as QT interval data corrected using Bazett's and Fridericia's corrections should be submitted in all applications, in addition to QT interval data corrected using any other formulae. A concurrent positive control group is strongly encouraged to support the use of newer correction approaches (e.g., individual subject correction) in order to demonstrate the ability of the correction method to allow detection of relevant effects on the QT/QTc interval.

3.1.1 Population-Derived Correction Formulae

Examples of such corrections include the following:

1) Fridericia's correction: $QTc = QT/RR^{0.33}$

2) Bazett's correction: $QTc = QT/RR^{0.5}$

Bazett's correction is frequently used in clinical practice and in the medical literature. In general, however, Bazett's correction overcorrects at elevated heart rates and under corrects at heart rates below 60 bpm and hence is not an ideal correction. Fridericia's correction is more accurate than Bazett's correction in subjects with such altered heart rates.

3) Corrections based on linear regression techniques

Application of linear regression techniques to plots of QT/RR data for the placebo or baseline study population allows for the estimation of the slope (b), which can be used for standardizing the data from both the drug and control groups to a normalized heart rate of 60 beats per minute, using the equation $QT = a + b(1-RR)$. The Framingham correction [$QTc = QT + 0.154(1-RR)$] is one example of a correction derived by linear regression.

4) Corrections using linear or non-linear regression modeling on pooled data from large databases.

© EMEA 2006

3.1.2 Correction Formulae Derived from Within-Subject Data

Corrections for heart rate using individual subject data have been developed, applying regression analysis techniques to individual pre-therapy QT and RR interval data over a range of heart rates, then applying this correction to on-treatment QT values. These approaches are considered most suitable for the 'thorough QT/QTc study' and early clinical studies, where it is possible to obtain many QT interval measurements for each study subject over a broad range of heart rates. As adaptation of the QT/QTc interval to changes in heart rate is not instantaneous, care should be taken to exclude ECG recordings collected during times of rapid heart rate changes due to this QT/RR hysteresis effect.

3.2 Analysis of QT/QTc Interval Data

Although increases from baseline in the QT/QTc interval constitute signals of interest, interpretation of these differences is complicated by the potential for changes not related to drug therapy, including regression toward the mean and choice of extreme values. Regression toward the mean refers to the tendency of subjects with high baseline values to have lower values at later time points, while subjects with low baseline values tend to experience increases. The direction of regression depends on initial selection criteria (for example, if subjects with high baseline QT/QTc interval values are excluded from the trial, values recorded during treatment will tend to rise relative to baseline levels). The process of choosing the highest of multiple observed values will also almost invariably cause an apparent change from any single baseline value, a phenomenon found in both drug and placebo-treated groups.

The QT/QTc interval data should be presented both as analyses of central tendency (e.g., means, medians) and categorical analyses. Both can provide relevant information on clinical risk assessment.

3.2.1 Analyses of Central Tendency

The effect of an investigational drug on the QT/QTc interval is most commonly analyzed using the largest time-matched mean difference between the drug and placebo (baseline-adjusted) over the collection period. Additional approaches to the assessment of central tendency could include analysis of changes occurring around the Cmax for each individual. This last analysis would be especially important if the drug has large between-subject variability in the rate of absorption or metabolism.

3.2.2 Categorical Analyses

Categorical analyses of QT/QTc interval data are based on the number and percentage of patients meeting or exceeding some predefined upper limit value. Clinically noteworthy QT/QTc interval changes might be defined in terms of absolute QT/QTc intervals or changes from baseline. Separate analyses should be provided for patients with normal and elevated baseline QT/QTc intervals. As with all QT/QTc interval analyses, categorical analyses are most informative when it is possible to compare the rate of supra-threshold readings in the treatment and control groups.

There is no consensus concerning the choice of upper limit values for absolute QT/QTc interval and changes from baseline. While lower limits increase the false-positive rate, higher limits increase the risk of failing to detect a signal of concern. In clinical trials, a prolongation of QTc > 500 ms during therapy has been a threshold of particular concern. Multiple analyses using different limits are a reasonable approach to this uncertainty, including:

- Absolute QTc interval prolongation:
  - QTc interval > 450
  - QTc interval > 480
  - QTc interval > 500

© EMEA 2006

- Change from baseline in QTc interval:
    - QTc interval increases from baseline > 30
    - QTc interval increases from baseline > 60

3.2.3 Analysis of Relationship Between Drug Exposure and QT/QTc Interval Changes

Establishing the relationship of drug concentrations to changes in QT/QTc interval may provide additional information to assist the planning and interpretation of studies assessing cardiac repolarization. This area is under active investigation.

3.3 Morphological Analyses of ECG Waveforms

While the predictive value of changes in ECG morphology, such as the development of U waves, has not been established, morphological abnormalities should be described and the data presented in terms of the number and percentage of subjects in each treatment group having changes from baseline that represent the appearance or worsening of the morphological abnormality. Typically these data will be obtained as a part of the 'thorough QT/QTc study'.

4. ADVERSE EVENTS

In addition to data on changes in ECG intervals, adverse event data can be another source of information on proarrhythmic potential, including:

- Premature discontinuations and dosage adjustments during clinical studies;
- Post-marketing adverse event reports if available.

4.1 Clinical Trial Adverse Events

Although drug-induced prolongation of the QT/QTc interval is usually asymptomatic, an increased rate of certain adverse events in patients taking an investigational agent can signal potential proarrhythmic effects. The rates of the following clinical events should be compared in the treated and control patients, particularly when there is evidence of an effect on the QT/QTc interval:

- Torsade de pointes;
- Sudden death;
- Ventricular tachycardia;
- Ventricular fibrillation and flutter;
- Syncope;
- Seizures.

Torsade de pointes is very infrequently captured in clinical databases, even those for drugs known to have significant proarrhythmic effects. Given this, the failure to observe an episode of TdP in a drug application database is not considered sufficient grounds for dismissing the possible arrhythmogenic risks of a drug when these are suspected on the basis of ECG and other clinical data. The other adverse events listed above, while less specific for an effect on cardiac repolarization, are more commonly captured in clinical trials, and an imbalance in their frequency between study groups can signal a potential proarrhythmic effect of the investigational agent. Sub-group analyses should be conducted in terms of age, gender, pre-existing cardiac disease, electrolyte disturbances, and concomitant medications. Comparing cause-specific rates of death is difficult, but a difference in the fraction of total deaths qualifying as "sudden" has also been proposed as a marker for proarrhythmic potential.

Detailed patient narratives should be provided for all serious cardiac adverse events, as would be the case for any serious event or events leading to discontinuation. In assessing the possible causal relationship of drug-induced QT/QTc interval prolongation to the event, attention should be directed to considerations such as temporal relationship and ECG results collected at the time of the event. As the QT/QTc interval is subject to considerable

fluctuation, a possible role for QT/QTc interval prolongation should not be dismissed on the basis of normal on-therapy ECG measurements performed prior to, or near the time of the adverse event. In addition to an appropriate adverse reaction report, patients with marked QT/QTc prolongation or an episode of TdP might provide useful information on risk management. When identified, they should therefore be examined closely for other risk factors (e.g., genetic predisposition, see section 4.3). Rechallenge with the investigational drug under appropriately monitored conditions can provide useful information on dose- and concentration-response relationships.

In evaluating the safety database of a new drug, consideration should be given to the extent to which the inclusion and exclusion criteria for patient eligibility might have influenced the study population with respect to the risk of QT/QTc interval prolongation and associated adverse events (e.g., exclusion of patients with cardiac co-morbidities or renal/hepatic impairment, prohibition of diuretics as concomitant medications). Ideally, the major clinical studies should include an adequate representation of female and elderly patients, as well as patients with co-morbidities and concomitant medications typical of the expected user population.

If a subject experiences symptoms or ECG findings suggestive of an arrhythmia during a clinical trial, immediate evaluation by a cardiac specialist is recommended, both for the purposes of treating the patient and for discussions related to continuation/ re-institution of the therapy.

4.2 Premature Discontinuations or Dosage Reductions

Particular attention should be directed to subjects or patients who are discontinued from clinical trials due to QT/QTc interval prolongation. Information should be provided on the basis for premature discontinuation of the patient (e.g., a QT/QTc interval value in excess of a protocol-defined upper limit, occurrence of QT/QTc interval prolongation in association with symptoms of arrhythmia), as well as the dose and duration of treatment, plasma levels if available, demographic characteristics, and the presence or absence of risk factors for arrhythmia.

Dosage reductions prompted by QT/QTc interval prolongation should also be documented.

4.3 Pharmacogenetic Considerations

Many forms of Long QT Syndrome are now known to be linked to mutations in genes encoding cardiac ion channel proteins. Because of incomplete penetrance, not all carriers of mutated ion channel genes will manifest QT/QTc interval prolongation in screening ECG evaluations. Polymorphisms can affect ion channels, leading to an increased sensitivity to drugs that affect repolarization. Genotyping patients who experience marked prolongation of the QT/QTc or TdP while on drug therapy should be considered.

4.4 Post-Marketing Adverse Event Reports

Because documented cases of TdP are relatively rare, even for drugs that prolong the QT/QTc, they are often not reported until large populations of patients have received the agent in post-marketing settings. The available post-marketing adverse event data should be examined for evidence of QT/QTc interval prolongation and TdP and for adverse events possibly related to QT/QTc interval prolongation, such as cardiac arrest, sudden cardiac death and ventricular arrhythmias (e.g., ventricular tachycardia and ventricular fibrillation). A well-characterized episode of TdP has a high probability of being related to drug use, whereas the other events that are reported more commonly would be of particular concern if reported in a population at low risk for them (e.g., young men experiencing sudden death).

5. REGULATORY IMPLICATIONS, LABELLING, AND RISK MANAGEMENT STRATEGIES

5.1 Relevance of QT/QTc Interval Prolonging Effects to the Evaluation Process

Substantial prolongation of the QT/QTc interval, with or without documented arrhythmias, could be the basis for non-approval of a drug or discontinuation of its clinical development, particularly when the drug has no clear advantage over available therapy and available therapy appears to meet the needs of most patients. Failure to perform an adequate clinical assessment of the potential QT/QTc interval prolonging properties of a drug can likewise be justification to delay or deny marketing authorization. For non-antiarrhythmic drugs, the outcome of the risk benefit assessment will generally be influenced by the size of the QT/QTc interval prolongation effect, whether the effect occurs in most patients or only in certain defined outliers, the overall benefit of the drug, and the utility and feasibility of risk management options. The inclusion of precautionary material in the prescribing information will not necessarily be considered an adequate risk management strategy, if implementation of the recommendations in a clinical use setting is judged to be unlikely.

If QT/QTc interval prolongation is a feature shared by other drugs of the therapeutic class in question, evaluation of the new drug could usefully involve a comparison of the magnitude and incidence of any QT/QTc interval prolongation effects relative to those of other members of its class in concurrent positive control groups.

It is difficult to determine whether there is an effect on the mean QT/QTc interval that is so small as to be inconsequential, but the risk of arrhythmias appears to increase with the extent of QT/QTc prolongation. Drugs that prolong the mean QT/QTc interval by around 5 ms or less do not appear to cause TdP. Whether this signifies that no increased risk exists for these compounds or simply that the increased risk has been too small to detect is not clear. The data on drugs that prolong the mean QT/QTc interval by more than around 5 and less than 20 ms are inconclusive, but some of these compounds have been associated with proarrhythmic risk. Drugs that prolong the mean QT/QTc interval by >20 ms have a substantially increased likelihood of being proarrhythmic, and might have clinical arrhythmic events captured during drug development.

Regardless of the degree to which a drug prolongs the QT/QTc interval, decisions about its development and approval will depend upon the morbidity and mortality associated with the untreated disease or disorder and the demonstrated clinical benefits of the drug, especially as they compare with available therapeutic modalities. Demonstrated benefits of the drug in resistant populations or in patients who are intolerant of, or have a labeled contraindication to, approved drugs for the same disease represent additional relevant clinical considerations that might justify approval of the drug, if the indication were limited to use in such patients.

Some factors have been proposed that can modify the risk of QT/QTc prolongation. For instance, it has been suggested that some drugs might prolong the QT/QTc interval up to a "plateau" value, above which there is no dose-dependent increase, although this has not been demonstrated adequately to date. It has also been suggested that proarrhythmic risk might be influenced by other pharmacologic effects (e.g., other channel effects). In any case, it is important to identify the "worst case scenario" for drugs that have demonstrated effects on QT/QTc interval as a part of risk assessment (i.e., the QT/QTc interval measured in the target patient population at the time of peak effect and under conditions of the highest blood levels that can be attained during therapy).

5.2 Labelling Issues for Drugs that Prolong the QT/QTc Interval

It is recognized that there will be regional differences in labelling. However, it is recommended that the following be considered:

- A warning/precautionary statement about the risk;

© EMEA 2006

- A description of the design and results of the trials investigating the effect on the QT/QTc interval, including the absence of demonstrated effect;
- The dosage recommendations;
- A list of conditions known to increase the proarrhythmic risk (e.g., congestive heart failure, Long QT Syndrome, hypokalemia);
- A precautionary statement regarding the concomitant use of two or more QT/QTc interval prolonging drugs and other interactions increasing the risk;
- Recommendations for patient monitoring (ECG and electrolytes) and management of patients with QT/QTc prolongation or symptoms suggestive of an arrhythmia.

5.3 Post-Marketing Risk Management for Drugs that Prolong the QT/QTc Interval

The use of dosing adjustments following institution of therapy appears to materially decrease the risk of TdP in hospitalized patients receiving an antiarrhythmic drug; no similar data are available for drugs of other therapeutic classes. For approved drugs that prolong the QT/QTc interval, risk-management strategies aimed at minimizing the occurrence of arrhythmias associated with their use have focused on education of the health-care providers and patients.

THE JOURNAL OF INFECTIOUS DISEASES • VOL. 122, NO. 4 • OCTOBER 1970
© 1970 by the University of Chicago. All rights reserved.

Complications of Smallpox Vaccination, 1968: Results of Ten Statewide Surveys

J. Michael Lane,* Frederick L. Ruben, John M. Neff, and J. D. Millar

From the Smallpox Eradication Program, National Communicable Disease Center, Health Services and Mental Health Administration, Public Health Services, United States Department of Health, Education and Welfare, Atlanta, Georgia; and the Department of Pediatrics, Johns Hopkins Hospital, Baltimore, Maryland

In this country little attention was given to complications of smallpox vaccination until Greenberg reported a mortality rate of approximately one per 1,000,000 in 1947 [1]. Reports following this one included Kempe's [2] and that of Sussman and Grossman [3], each citing cases and reporting on the effects of vaccinia immune globulin (VIG). The first published data on complications for the entire United States were for the year 1963 [4, 5]. A recent review reports deaths from smallpox vaccination in the United States since 1959 [6].

Complications of vaccination against smallpox are not considered as officially reportable diseases, and statistics on incidence are not gathered systematically in the United States. A comparison of finding of the cases through the records on the use of VIG in 1963 with cases found through retrospective surveys of physicians in 4 small states indicates that complications of smallpox vaccination occur more frequently than can be detected by the system of VIG distribution alone [4, 5]. The VIG distribution system misses most of the patients with less severe clinical illness and fails to detect many patients with postvaccinial disease of the central nervous system [7].

Contacting practicing physicians directly by mail is a useful way of obtaining information that is not available in regularly reported statistics. Such surveys may have the disadvantage of low rates of return, which decrease their validity. Poor return rates may bias the results because the physicians not responding may have had widely varying experiences.

In mid-1967 the Smallpox Eradication Program of the National Communicable Disease Center (NCDC) initiated studies to determine the incidence of complications following smallpox vaccination in calendar year 1968. Investigation of patients receiving VIG and analysis of clinical information obtained from a variety of other sources was instituted nationwide [7]. In addition, 10 states wtih relatively small populations were chosen as sites for direct statewide surveys to determine the validity of the data from national surveillance. Previous surveys had indicated that states with a cumulative population considerably in excess of 5% of the nation's population would have to be covered to allow calculation of meaningful rates of incidence for rare complications such as postvaccinial disease of the central nervous system and vaccinia necrosum [5].

Methods

Table 1 lists the 10 states selected for direct surveys of physicians and the estimated populations for 1968 [8]. The states were chosen

Received for publication April 3, 1970 and in revised form June 26, 1970.

The authors are indebted to the following state officials and Epidemic Intelligence Service Officers for conducting state surveys: Drs. W. J. Donald, M. C. Sinclair, D. K. Freedman, P. Clark, A. M. Reeve, R. G. Sharrar, L. Anderson, J. L. Nitzkin, D. H. Fisher, P. J. Leadley, H. J. Garber, H. Mellin, J. H. Janney, J. E. Cannon, H. D. Scott, G. E. McDaniel, W. M. Johnson, B. J. Francis, R. S. Thompson, N. H. Dyer, and J. M. Wolff.

* Present address: Department of Epidemiology, University of California School of Public Health, Berkeley, California 94720. Please address requests for reprints to Dr. Lane at this address.

Table 1. Estimated Populations of the 10 survey states in 1968

State	1968 Mid-year population
Alabama	3,558,000
Alaska	274,000
Iowa	2,774,000
Kentucky	3,220,000
Maine	976,000
Maryland	3,754,000
Rhode Island	914,000
South Carolina	2,664,000
Washington	3,276,000
West Virginia	1,802,000
Total	23,212,000*

NOTE.—Populations are estimated as of July 1, 1968.
* Total is 11.6% of estimated population for the United States.

partly for their size, but also because of the interest shown by their state epidemiologists, the presence of Epidemic Intelligence Service Officers assigned from NCDC, and the relatively few numbers of physicians. Direct mail surveys in states with more than 4,000 physicians were considered unwieldy.

In each state, a list of practicing physicians was obtained from the state health department or state medical society. In some states this list included a small number of physicians who had retired or had moved from the area. Although such nonpracticing physicians were included in the initial mailings, they are excluded from our calculations. In Washington, South Carolina, and Kentucky, certain groups of physicians (psychiatrists, orthopedic surgeons, obstetricians, etc.), who were not expected to see complications of smallpox vaccination, were excluded from the mailings. In Alaska and Maryland, public health nurses were included in the mailing list.

All physicians on the mailing lists were sent an initial "sensitizing" letter in late 1967 or early January 1968. The letter was signed by representatives of the state health department and state medical society. It explained briefly the need for data on complications of smallpox vaccination and requested that each physician record the clinical and epidemiologic characteristics of patients with complications following vaccination whom they saw during calendar year 1968. The letter informed the physicians that they would be asked early in 1969 for information on such patients. The Iowa State Health Department surveyed Iowa physicians quarterly rather than at the end of the year, and Maine conducted a monthly survey.

Early in January 1969, all physicians on the mailing lists were sent a combination letter and checksheet with a stamped, addressed return envelope. The letter reminded the recipient of the survey for vaccination complications and asked him to check the enclosed form. The physician was asked to check *yes* or *no* to indicate whether he had seen any patients with any vaccination complications during 1968. If he checked *yes,* he then checked the diagnostic type or types of the complications he had seen. Physicians who had not responded approximately 8 weeks after this mailing were sent a second mailing of the same letter and form. Each physician's name was typed on the reporting form so that returned forms could be matched with the master lists and the physician contacted if further information was required. These 2 mailings were primarily for identification of physicians who might have seen complications; they were designed to get a high rate of return, and they did not request detailed clinical information.

Physicians who responded to these 2 mailings indicating that they had seen or treated a complication of smallpox vaccination were again contacted by mail or telephone. State health department physicians asked them to provide the age, sex, date of vaccination, vaccination history, and date of onset of complication of each patient. A brief clinical description of each patient was elicited, and this was supplemented by a hospital chart review of hospitalized patients. The diagnosis of the local physician was accepted in most instances of minor complications. In the case of major complications, particularly encephalitis or vaccinia necrosum, clinical data were thoroughly reviewed to corroborate the diagnosis. Cases were rejected when these data proved insufficient to confirm the diagnosis. The resulting clinical information was tabulated according to the patient's age, vaccination status, and type of complication.

As indicated above, several patients detected by this survey are not included in the analysis reported here. Most of these were excluded because insufficient data on the patient's age and year of vaccination made it impossible to be sure that they had been vaccinated in 1968. A smaller

number were definitely found to have been vaccinated in 1967 or 1969 and were, therefore, excluded.

Results

Detailed results by individual state, including full clinical details of typical patients and particularly unusual patients, are in preparation and will be presented elsewhere. This paper will emphasize the rates of incidence for the states grouped as a whole.

Response to the surveys. In 8 of the 10 states surveyed, questionnaires were mailed at the end of 1968. Table 2 presents the numbers of questionnaires sent and responses received in these 8 states; 83.9% of the physicians returned their mailed questionnaires. Approximately 5% of those physicians responding claimed to have seen or treated at least 1 complication of vaccination during 1968.

In Iowa and Maine, quarterly and monthly questionnaires, respectively, were mailed. In Iowa a total of 9,852 questionnaires were mailed to 3,076 physicians, and 4,855, or 49%, were returned. In Maine, 14,400 questionnaires were mailed to 1,200 physicians, with 6,333, or 44%, returned. The survey methods did not permit calculation of the percentages of physicians in these 2 states who never returned their questionnaires.

Complications in primary vaccinees. Table 3 shows the numbers of complications by diagnosis and by age and vaccination status of the patients. The diagnostic classifications used were those defined by Neff et al. [4].

Table 2. Numbers of questionnaires sent and responses received by state

State	Physicians sent questionnaires	Physicians responding	% Response
Alabama	2,411	2,089	86.6
Alaska	234	206	88.0
Kentucky	2,565	2,144	83.6
Maryland	3,800	2,960	77.8
Rhode Island	1,548	997	64.4
South Carolina	1,047	1,026	97.9
Washington	1,824	1,691	92.7
West Virginia	1,550	1,464	94.5
Total	14,979	12,577	83.9

NOTE.—This listing excludes Iowa and Maine, which performed quarterly and monthly surveys, respectively.

Accidental implantation was the most frequent complication of primary vaccination, accounting for 42% of all such complications. The involved sites included virtually all parts of the body surface, but lesions on the eyelid were reported most frequently.

Generalized vaccinia comprised 19% of the primary-vaccination complications. Only patients with vesicular lesions away from the site of vaccination were included in this category. In the descriptions of some patients, it was difficult to distinguish generalized vaccinia from erythema multiforme, because of inadequate descriptions of the rash. The diagnosis by the reporting physician was accepted in such instances.

"Other" complications accounted for 21% of complications of primary vaccination. This category includes patients with severe reactions to vaccination which were either superinfected with bacteria or which made the patient uncomfortable enough to consult a physician. Several unusual complications are also included in this group: a patient with fetal vaccinia, a patient with a melanoma developing in the scar from vaccination in 1968, and a patient with monoarticular arthritis following vaccination.

Erthema multiforme (sometimes called "roseola vaccinia," "toxic urticaria," etc.) accounted for 13% of all complications of primary vaccination. It was invariably transient and mild.

The 25 cases of eczema vaccinatum accounted for only 3% of complications following primary vaccination. The patient with vaccinia necrosum and the 8 patients with postvaccinial encephalitis together accounted for only 1% of the complications of primary vaccination.

Complications in revaccinated individuals. Table 4 shows that individuals receiving revaccinations, with 108.2 complications per 1,000,000 vaccinations, developed fewer complications than did primary vaccinees who had 1,253.8 complications per 1,000,000 vaccinations. Accidental implantation and "other" complications comprised up to 75% of complications following revaccination. Forty-four percent of revaccinations were given to adults, who developed 46% of the complications among those individuals revaccinated.

Complications in contacts of vaccinated persons. Most of the complications resulting from contact were accidental implantations of vaccinia

Table 3. Reported complications of smallpox vaccination by diagnosis and vaccination status

Age (yrs.) and status	Estimated number of vaccinations	Vaccinia necrosum	Postvaccinial encephalitis	Eczema vaccinatum	Generalized vaccinia	Accidental implantation	Erythema multiforme	Other	Total
Primary vaccination:*									
<1	71,000	0	3	1	28	36	31	11	110
1–4	317,000	1	3	14	74	183	50	75	400
5–19	229,000	0	2	8	32	85	20	49	196
⩾20	33,000	0	0	1	7	20	1	21	50
Unknown	...	0	0	1	16	20	5	17	59
Total	650,000	1	8	25	157	344	107	173	815
Revaccination:									
<1	0	0	0	0	0	0	0	0	0
1–4	55,000	0	0	0	0	6	4	1	11
5–19	503,000	0	0	1	5	24	1	12	43
⩾20	440,000	3	2	2	4	11	4	24	50
Unknown	...	0	0	0	0	1	1	2	4
Total	998,000	3	2	3	9	42	10	39	108
Contact:									
<1	...	0	0	0	0	1	0	1	2
1–4	...	0	0	4	1	12	1	0	18
5–19	...	0	0	6	0	5	0	0	11
⩾20	...	0	0	3	0	11	0	0	14
Unknown	...	0	0	0	0	0	0	0	0
Total	...	0	0	13	1	29	1	1	45
Grand total	1,648,000	4	10	41	167	415	118	213	968

* Includes 65 patients with unknown vaccination status.

virus in otherwise healthy people. Approximately 29% of the complications were in patients with eczema who came in contact with recently vaccinated siblings or parents.

Severe complications. Postvaccinial encephalitis was documented in 8 patients who received primary vaccination and in 2 patients who had received revaccination. One patient, an infant vaccinated at 9 months, died. Other cases of suspected postvaccinial encephalitis were excluded because clinical documentation was insufficient to establish the diagnosis.

Vaccinia necrosum developed in 1 primary vaccinee (a child with Bruton's type hypogammaglobulinemia) and 3 revaccinated persons (a man with a depressed immunoglobulin M while receiving anticoagulants and 2 elderly people each with chronic lymphatic leukemia). All required therapy with either VIG, gamma globulin, or thiosemicarbazone, and none died. Vaccinia necrosum was reported frequently by physicians whose patients, upon investigation, proved to have suffered severe primary takes. No patient was included as having vaccinia necrosum unless he had an underlying immunologic deficiency, or unless the course of his illness was typical of vaccinia necrosum. Patients whose sites of vaccination healed spontaneously were not accepted as cases of vaccinia necrosum.

Fetal vaccinia is a very rare complication of smallpox vaccination. One patient with probable fetal vaccinia was detected in this survey. A young primagravida received a primary smallpox vaccination in the first month of an unsuspected pregnancy. She gave birth at 5 months to a premature baby weighing slightly under 3 pounds. The only abnormality noted at birth was marked pock-like scarring of the skin over the entire body and a few loosely adherent crusts. The baby did well after delivery and has developed normally. Details of this patient will be published later.

Incidence rates for complications of vaccination. Table 4 presents the rates of incidence for complications of primary vaccination and revaccination by age and diagnostic category. Complications were at least 10 times more frequent in

Table 4. Rates of reported complications associated with smallpox vaccination, cases per 1,000,000 vaccinations

Age (yrs.) and status	Vaccinia necrosum	Post-vaccinial encephalitis	Eczema vaccinatum	Generalized vaccinia	Accidental implantation	Erythema multiforme	Other	Total
Primary vaccination:								
<1	...	42.3	14.1	394.4	507.0	436.6	154.9	1,549.3
1–4	3.2	9.5	44.2	233.4	577.3	157.7	236.6	1,261.8
5–19	...	8.7	34.9	139.7	371.2	87.3	214.0	855.9
⩾20	...	...	30.3	212.1	606.1	30.3	636.4	1,515.2
Total*	1.5	12.3	38.5	241.5	529.2	164.6	266.2	1,253.8
Revaccination:								
<1	...	...	...	...	...	...	...	...
1–4	...	...	...	...	109.1	72.7	18.2	200.0
5–19	...	...	2.0	9.9	47.7	2.0	23.9	85.5
⩾20	6.8	4.5	4.5	9.1	25.0	9.1	54.5	113.6
Total†	3.0	2.0	3.0	9.0	42.1	10.0	39.1	108.2

NOTE.—These data omit 45 patients with contact-acquired vaccinia.
* Total includes 59 patients of unknown age.
† Total includes 4 patients of unknown age.

primary vaccinees than in revaccinated individuals. The cases on which these rates are based are those presented in table 3. The estimates of the numbers of vaccinations were calculated from data from the National Immunization Survey, which is conducted for the National Communicable Disease Center by the Bureau of the Census.[1] The numbers of vaccinees in each age category throughout the United States have been adjusted by multiplication by the percentage of the population of the United States contained in the 10 states surveyed (11.6%).

Comparison of results of techniques for national surveillance with results of techniques for direct physician survey. In the national surveillance study for 1968 [7], 572 persons were detected who had confirmed complications of vaccination. Of these, 82.5% had received VIG. In these direct surveys of physicians in 10 states with 11.6% of the U.S. population, 968 patients were detected; only 6.6% of the patients had received VIG. The rates of complications per million vaccinations are, therefore, considerably higher in this study, particularly those for the less severe complications.

[1] United States Immunization Survey, United States Public Health Service, National Communicable Disease Center, Atlanta, Georgia, December 1968, p. 24.

Discussion

Most studies of the complications of smallpox vaccination are biased toward the relatively more severe complications, especially postvaccinial encephalitis, vaccinia necrosum, and eczema vaccinatum. The purpose of this study was to uncover the less severe, as well as the severe, complications by a direct survey of physicians.

The techniques applied in these state surveys allowed many of the clinical details of cases to be lost. The surveys should be regarded as reports of diagnoses of the physicians, rather than as reports of proven disease entities. A vigorous effort was made to gather detailed clinical information on all patients reported as having vaccinia necrosum, encephalitis, or unusual complications of vaccination. The details of these efforts and their results by state are in preparation and will be published later. The diagnoses of the physicians were accepted in most reports of generalized vaccinia, accidental implantation, erythema multiforme, and other complications. Although the exact date of vaccination was sought for each patient, some were not available. A small number of patients included may have been vaccinated in 1967 or 1969. The rates, therefore, may overestimate slightly the true incidence of complications.

The National Immunization Survey is not de-

signed to be applicable to individual states. However, major errors in the estimate of numbers of vaccinations performed in the 10 survey states would not greatly influence the rates because of the low incidence of complications. The National Immunization Survey has a standard error of estimate of less than 1%. Shifts of even 10% within each age category would not greatly influence the absolute rates or the relative frequency of the various complications between diagnostic categories or within age groups.

The incidence rates for the various complications as calculated from these 10-state direct surveys of physicians are considerably higher than those reported in the comparison national surveillance study [7]. There were minor variations in rates from state to state, but these were not felt to be significant. The less severe complications were poorly reported in the national surveillance data. Complication rates for primary vaccineces with generalized vaccinia, accidental implantation, and "other" complications are 10–22 times higher in the direct surveys than in the national surveillance work. Eczema vaccinatum in primary vaccinees was found 4 times more frequently in the state surveys than in the national surveillance, but the majority of the severe or hospitalized cases of eczema vaccinatum was reported through the system of VIG distribution. The rates for vaccinia necrosum in the surveys and the National Surveillance Program are similar, although 2 patients were discovered in the statewide surveys which were not reported nationally. All viral or postviral diseases of the central nervous system are theoretically reportable through the National Encephalitis Surveillance Program. Postvaccinial encephalitis is not believed to be treatable with VIG [2, 9] and, therefore, the VIG distribution system does not detect it frequently. The state surveys uncovered patients with encephalitis at a rate approximately 4 times that of the National Surveillance Program.

Vaccination complications occurred more frequently in infants than in children vaccinated after their first birthday. This higher rate for infants has been reported consistently in recent studies [4, 5, 7]. Although the American Academy of Pediatrics and the Surgeon General's Advisory Committee on Immunization Practices of the Public Health Service recommend that vaccination be deferred until the second year of life, approximately 614,000 infants were vaccinated during 1968.

The occurrence of a case of fetal vaccinia reemphasizes the dangers of vaccination of pregnant women. Studies of the incidence of congenital malformations and stillbirths in association with vaccination during pregnancy are inconclusive, and they fail to establish an adverse effect of vaccination [10]. However, unless a pregnant woman has had contact with a smallpox patient, vaccination should be deferred until after her delivery.

Summary

Direct surveys of physicians were used to identify patients with complications of smallpox vaccination occurring in 1968 in 10 states, which comprise 11.6% of the total population of the United States. In 8 of the states a survey at the end of the year yielded a total response from 83%. In 2 states, where quarterly or monthly surveys were conducted, 49% and 44%, respectively, of the total number of questionnaires were returned.

Eighty-four percent of complications occurred in primary vaccinees. The rate of accidental implantation and of generalized vaccinia, the nonfatal complications, was 529.2 and 241.5 cases, respectively, per million primary vaccinations. These rates are 10 and 21 times as great as the rates computed from data from national surveillance of complications. The rate of postvaccinial encephalitis and vaccinia necrosum, potentially fatal complications, was 12.3 and 1.5 cases, respectively, per million primary vaccinees. These rates are 4 and 1.6 times greater than rates from national surveillance data. One death (from postvaccinial encephalitis) and 1 case of fetal vaccinia were detected.

References

1. Greenberg, M. 1948. Complications of vaccination against smallpox. Amer. J. Dis. Child. 76:492–502.
2. Kempe, C. H. 1960. Studies on smallpox and complications of smallpox vaccination. Pediatrics 26: 176–189.
3. Sussman, S., and M. Grossman. 1965. Complications of smallpox vaccination. Effects of vaccinia immune globulin therapy. J. Pediat. 67:1168–1173.
4. Neff, J. M., J. M. Lane, J. H. Pert, R. Moore, J. D. Millar, and D. A. Henderson. 1967. Complica-

tions of smallpox vaccination. I. National survey in the United States, 1963. New Eng. J. Med. 276:125–132.

5. Neff, J. M., R. H. Levine, J. M. Lane, E. A. Ager, H. Moore, B. J. Rosenstein, J. D. Millar, and D. A. Henderson. 1967. Complications of smallpox vaccination United States 1963. II. Results obtained by four statewide surveys. Pediatrics 39: 916–923.
6. Lane, J. M., F. L. Ruben, E. Abrutyn, and J. D. Millar. 1970. Deaths attributable to smallpox vaccination, 1959 to 1966, and 1968. J.A.M.A. 212: 441–444.
7. Lane, J. M., F. L. Ruben, J. M. Neff, and J. D. Millar. 1969. Complications of smallpox vaccination, 1968. National surveillance in the United States. New Eng. J. Med. 281:1201–1208.
8. Current Population Reports. April, 1969. Series P-25, No. 420.
9. Fulginiti, V. A. 1970. Smallpox, p. 58–64. *In* H. F. Conn [ed.] Current therapy 1970. W. B. Saunders, Philadelphia.
10. Saxen, L., K. Cantell, and M. Hakama. 1968. Relation between smallpox vaccination and outcome of pregnancy. Amer. J. Public Health 58:1910–1921.

ACAM2000
Smallpox Vaccine

Vaccines and Related Biological Products Advisory Committee (VRBPAC) Briefing Document

Sponsor: Acambis Inc.
38 Sidney Street
Cambridge, MA 02139
Telephone: (617) 761-4200
Fax: (617) 494-1741

Report Date: 18 April 2007

Advisory Committee Briefing Materials: Available for Public Release

TABLE OF CONTENTS

1. HISTORY OF SMALLPOX AND CURRENT NEED FOR VACCINE 8
1.1. Smallpox (Variola Virus) Infection and Smallpox (Vaccinia Virus) Vaccination 8
1.2. Threat of Smallpox as a Biological Weapon 8
1.3. Measurements of the Protective Efficacy of Vaccination against Smallpox 9
1.4. Expected Adverse Events with Smallpox Vaccination 10
2. PRODUCT OVERVIEW 13
2.1. ACAM2000 Product Description 13
2.2. Indication 13
2.3. Strategic National Stockpile (SNS) 14
3. DEVELOPMENT OF ACAM2000 (SMALLPOX VACCINE) 15
3.1. Required Vaccine Specifications 15
3.2. Development of ACAM2000 15
4. NON-CLINICAL STUDIES OF ACAM2000 17
4.1. Non-Clinical Evaluations 17
4.1.1. Non-Clinical Efficacy 17
5. CLINICAL DEVELOPMENT OF ACAM2000 20
5.1. Overview 20
5.2. Standard Procedures in Clinical Studies of ACAM2000 20
5.2.1. Study Design 20
5.2.2. Administration of Vaccine 20
5.2.3. Subject Populations for Efficacy Studies 21
5.2.4. Primary Efficacy Endpoints 22
5.2.5. Evaluations of Vaccine Safety 23
6. CLINICAL EFFICACY RESULTS 24
6.1. Immunogenicity Results from Phase 1 Studies 24
6.2. Summary of Efficacy Results from Phase 2 Studies 25
6.2.1. Study H-400-005: Vaccinia-Naïve Subjects 25
6.2.2. Study H-400-003: Previously Vaccinated (Dryvax®) Subjects 28
6.3. Summary of Efficacy Results from Phase 3 Studies 29
6.3.1. Phase 3 Study H-400-009: Vaccinia-Naïve Subjects 30
6.3.2. Phase 3 Study H-400-012: Previously Vaccinated Subjects 32
6.4. Integrated Efficacy Analysis 36

6.4.1. Summary of Efficacy Results 36
6.4.2. Effect of Baseline Immunity on Efficacy Outcome Measures 39
6.4.3. ACAM2000 Clinical Consistency and Dosing Recommendations 40
6.5. Efficacy Conclusions 40
7. CLINICAL SAFETY 42
7.1. Safety Population 42
7.2. Serious Adverse Experiences (SAEs) and Deaths 42
7.2.1. Myocarditis/pericarditis 42
7.2.2. Other Serious Cardiac Events 44
7.2.3. Pregnancy 44
7.2.4. Other Serious Adverse Events 44
7.2.5. Other Relevant Safety Information 45
7.3. Common Adverse Experiences 45
7.3.1. Vaccination Site Reactions 47
7.3.2. Constitutional Symptoms 47
7.3.3. Lymphadenitis 47
7.3.4. Gastrointestinal Symptoms 48
7.4. Virus Shedding 48
7.5. Induction of Non-Specific Serologic Responses 48
7.6. Safety in Special Populations 49
7.7. Conclusions Regarding the Safety of ACAM2000 49
8. POST-LICENSURE SURVEILLANCE 50
9. OVERALL SUMMARY AND CONCLUSIONS: BENEFITS VERSUS RISKS 51
10. LIST OF REFERENCES 54
11. APPENDICES 58
11.1. Appendix 1 - Analysis of Efficacy Data from ITT Population 58
11.2. Appendix 2 - Demographics 60
11.2.1. Summary of Demographics 60
11.3. Appendix 3 – Subjects with Suspect or Probable Myocarditis 63
11.4. Appendix 4 – Cardiac Algorithm 70

LIST OF TABLES

Table 1: Rates of Reported Complications Associated with Vaccinia Vaccinations (Cases/Million Vaccinations) [a] ... 11

Table 2: Anticipated and Unanticipated SAEs Associated With Large-scale Immunization Programs by the DoD and DHHS ... 12

Table 3: Non-Clinical Studies Conducted with ACAM2000 Vaccine, and Master and Production Virus Banks ... 19

Table 4: Clinical Studies Conducted in the ACAM2000 Clinical Program ... 20

Table 5: Analysis Populations in Clinical Studies of ACAM2000 ... 21

Table 6: Proportion of Subjects with Positive T Cell Responses in Study H-400-002, by Treatment Group ... 25

Table 7: Vaccination Success and Neutralizing Antibody Response as a Function of ACAM2000 Dose for Study H-400-005: Vaccinia-Naïve Subjects ... 26

Table 8: Vaccination Success based on Cutaneous Response and Neutralizing Antibody Response as a Function of ACAM2000 Dose for Study H-400-003: Previously Vaccinated (Dryvax®) Subjects ... 28

Table 9: Phase 3 Efficacy Results for ACAM2000 versus Dryvax® in Vaccinia-Naïve Subjects (Study H-400-009) ... 31

Table 10: Phase 3 Efficacy Results for ACAM2000 versus Dryvax® in Previously Vaccinated Subjects (Study H-400-012) ... 34

Table 11: Phase 3 Efficacy Results for ACAM2000 and Dryvax®, Adjusted for Baseline Neutralizing Antibody Titer (Study H-400-012) ... 36

Table 12: Immunogenicity and Efficacy Results for Vaccinia-Naïve Subjects ... 38

Table 13: Immunogenicity and Efficacy Results for Previously Vaccinated Subjects ... 39

Table 14: Commonly Reported (≥10% in the ACAM2000 or Dryvax® Groups) AEs by MedDRA Preferred Term, Baseline Vaccination Status and Treatment Group, Phase 3 Studies, Total Population ... 46

Table 15: Phase 3 Efficacy Results for ACAM2000 versus Dryvax® in Vaccinia-Naïve Subjects from the ITT population (Study H-400-009) ... 58

Table 16: Phase 3 Efficacy Results for ACAM2000 versus Dryvax® in Previously Vaccinated Subjects from the ITT population (Study H-400-012) ... 59

Table 17: Demographics and Baseline Characteristics, By Subject Population, Treatment Group, and ACAM2000 Lot, Final Analysis Safety Population ... 61

Table 18: Subjects with Treatment-Emergent Suspect or Probable Myocarditis, by Treatment Group and Study ... 63

LIST OF FIGURES

Figure 1: Passage History of ACAM1000 and ACAM2000 16

Figure 2: ACAM2000 Dose Response and Comparison to Dryvax®: Vaccination Success based on Cutaneous Response in Vaccinia-Naïve Subjects (Study H-400-005) 27

Figure 3: ACAM2000 Dose Response and Comparison to Dryvax®: Neutralizing Antibody GMT in Vaccinia-Naïve Subjects (Study H-400-005) 27

Figure 4: ACAM2000 GMTs for Vaccine-Naïve vs. Previously Vaccinated Subjects (Studies H-400-003 and H-400-005) 29

Figure 5: Graphical Presentation of Phase 3 Efficacy Results in Vaccinia-Naïve Subjects (Study H-400-009) 31

Figure 6: Reverse Cumulative Frequency Distribution of Neutralizing Antibody Titers on Day 30 (Study H-400-009) 32

Figure 7: Graphical Presentation of Phase 3 Efficacy Results in Previously Vaccinated Subjects (Study H-400-012) 34

Figure 8: Reverse Cumulative Frequency Distribution of Neutralizing Antibody Titers on Day 30 (Study H-400-012) 35

Figure 9: Algorithm for Diagnosis and Management of Subjects with Suspect Myocarditis/Pericarditis 70

LIST OF ABBREVIATIONS

Abbreviation	Definition
ACAM2000	ACAM2000 Smallpox Vaccine (Vero Cells), lyophilized Smallpox Vaccine
ACIP	Advisory Committee on Immunization Practices
AE	Adverse Event
AnE	Antibody evaluable
ANOVA	Analysis of variance
AST	Average Survival Time
AV	Atrio-ventricular
CAP	Cardiology Advisory Panel
CDC	Centers for Disease Control and Prevention
CI	Confidence interval
CK	Creatine kinase
CK-MB	Creatine kinase-myocardial band
CNS	Central nervous system
CPK	Creatine Phosphokinase
CTL	Cytotoxic T lymphocyte
DoD	Department of Defense
DHHS	Department of Health and Human Services
DSMB	Data Safety Monitoring Board
ECG	Electrocardiogram
ED_{90}	90% Effective Dose
EV	Eczema vaccinatum
FDA	Food and Drug Administration
GMP	Good Manufacturing Practice
GMT	Geometric mean neutralizing antibody titer
GV	Generalized vaccinia
HBsAb	Hepatitis B Surface Antibody
HBsAg	Hepatitis B Surface Antigen
HCV	Hepatitis C Virus
HIV	Human immunodeficiency virus
IRC	Independent Review Committee
ITT	Intent-to-treat
LD_{50}	Lethal Dose, 50%
LPA	Lymphoproliferation Assay
MedDRA	Medical Dictionary for Regulatory Activities

Abbreviation	Definition
NA	Not applicable
NYCBH	New York City Board of Health
P	Passage
PFU	Plaque-forming units
$PRNT_{50}$	50% Plaque reduction neutralization test
PV	Progressive vaccinia
PVE	Post-vaccinial encephalopathy
PVG	Pharmacovigilance
RFP	Request for Proposal
SAE	Serious adverse event
SD	Standard deviation
SNS	Strategic National Stockpile
US	United States
USP	United States Pharmacopeia
VAERS	Vaccine Adverse Event Reporting System
VIG	Vaccinia immune globulin
VRBPAC	Vaccine and Related Biological Products Advisory Committee
WHO	World Health Organization
WR	Western Reserve

1. HISTORY OF SMALLPOX AND CURRENT NEED FOR VACCINE

1.1. Smallpox (Variola Virus) Infection and Smallpox (Vaccinia Virus) Vaccination

Smallpox was one of the most important causes of morbidity and mortality worldwide through the first half of the 20th century. Smallpox in humans is caused by the variola virus, which, along with vaccinia (cowpox) and monkeypox, is a member of the Orthopoxvirus genus. The disease is defined by an acute onset of fever >101°F (38.3°C) followed by a rash characterized by firm, deep-seated vesicles or pustules in the same stage of development (i.e., on any one part of the body, all lesions are vesicles or all are pustules) without other apparent cause. Infection is spread person-to-person via the respiratory route by contact (droplets), and possibly by aerosol [1]. However, since the virus can survive on objects for approximately 1 week, infection can be spread by contact with contaminated objects such as bed linens. Smallpox is fatal in approximately 30% of cases.

Between 1967 and 1977, a global program of smallpox eradication resulted in the elimination of the natural disease. The systematic use of vaccine (live, attenuated vaccinia virus) contributed significantly to this effort [1]. The vaccines used in the eradication campaign were derived from several strains of vaccinia, including the New York City Board of Health (NYCBH) strain. Dryvax®, the only smallpox vaccine currently approved in the United States (US), was derived from the NYCBH strain of vaccinia. In turn, ACAM2000 (Acambis' live, second generation smallpox vaccine), the subject of this briefing document, was derived from Dryvax®.

The last cases of smallpox in the US occurred in 1949 in Texas [1]. Because of the absence of smallpox and the risk of vaccine-associated adverse events, routine vaccination of children ceased by 1972 and of hospital personnel by 1976. Vaccinations of military populations ceased in 1989, but were subsequently renewed in 2002 in the context of the imminent war in Iraq. By the mid 1980s, there were only 2 known repositories of variola virus: the Institute of Virus Preparations in Moscow, Russia, and the United States Centers for Disease Control and Prevention (US CDC) in Atlanta, GA.

1.2. Threat of Smallpox as a Biological Weapon

In the mid- to late 1990s, the US government became concerned about the threat of smallpox as an agent of biological terrorism [2]. In the US, approximately half of the population was born after the cessation of vaccination, and therefore has no immunity to smallpox. The remaining population has variable residual immunity. The eradication of smallpox and the cessation of vaccination, therefore, have created vulnerability to covert attack or biowarfare using variola virus.

The threat of a deliberate release of smallpox is a high-consequence event that is considered credible and possible. As a result, numerous governments around the world are building stockpiles of smallpox vaccine as a deterrent and management measure should such an attack occur. The terrorist attacks in New York and Washington in September 2001 and the anthrax

attacks of October 2001 in the US heightened the urgency of developing stockpiles of vaccines and treatments for potential bioweapon agents, and in November of 2001, Acambis was awarded a contract from the CDC for the development and manufacture of a second-generation smallpox vaccine using modern methodology and quality controls (see Section 2.3).

1.3. Measurements of the Protective Efficacy of Vaccination against Smallpox

Smallpox vaccination was started by Edward Jenner at the end of the 18th century before there was any understanding of measurable immunity. His classic studies on the protection of vaccination against smallpox were based on a dermal response to percutaneous vaccination manifested by pustule formation followed by a scar. This dermal response remained the standard for measurement of protection for over a hundred years. In the 20th century, standardized smallpox vaccines were developed and the dermal reaction that was observed to correspond to the development or the presence of humoral antibodies was defined by the World Health Organization (WHO) as major dermal reaction (6-8 days after vaccination the presence of a pustular lesion or an area of definite induration surrounding a central ulcer or scab) [3].

Although the measurement of dermal response is well established as an indicator of immunity for primary vaccination, it does have its drawbacks since the absence of a major cutaneous reaction can result either from poor vaccination technique or from an existing degree of immunity [4]. Dermal response rates, particularly in the previously vaccinated, are highly dependent upon the attributes of the vaccine and the technique of administration. Subjects who have been previously vaccinated and are revaccinated may manifest a reduced cutaneous response compared to vaccinia-naïve subjects, yet also have an immune response to the vaccine. Therefore, the measurement of neutralizing antibody is considered by many to be a better correlate to immunity against smallpox in the previously vaccinated [5]. Nevertheless, the measure of immunity has historically been based on the appearance of a major cutaneous reaction, confirmed by the presence of a vaccination scar.

Using this standard of dermal response, there were several retrospective studies that established that absolute protection against smallpox lasted 3 to 5 years after vaccination, then waned with protection appearing to persist for up to 50 years [6, 7, 8].

It is not entirely clear what the separate role of T cell response and circulating antibodies play in protection against smallpox. According to Fenner’s extensive review of orthopox virus infections in animal models, circulating antibodies do have an effect on viral replication and spread of pox virus infection but cell mediated immunity may be more important [9]. In all probability both are very important. There is evidence to support the protective value of neutralizing antibodies against smallpox infection in humans [10, 5]. The observations of the value of neutralizing antibodies also are supported in the findings of another study where the administration of VIG (human gamma globulin serum with high titer neutralizing antibodies from vaccinated individuals) after exposure to smallpox during the incubation period seems to have modified the disease [11].

While the level of neutralizing antibody as determined by plaque reduction assays in cell culture may potentially provide a better correlate of immunity than dermal response, there is not an extensive database to precisely establish the neutralizing antibody titer required for protection

based on qualified assays. However, historical data and limited prospective studies suggest a putative titer of greater than 32 [1:32 dilution of plasma giving a 50% reduction in plaque-forming units (PFUs)] as protective [5].

1.4. Expected Adverse Events with Smallpox Vaccination

Frequent and non-serious adverse events (AEs) historically associated with smallpox vaccination include erythema, pruritus, and swelling at the vaccination site with or without regional lymphadenopathy or lymphangitis, as well as constitutional symptoms, including fatigue, headache, and myalgia [12,13].

Other possible, more severe and potentially serious adverse reactions to smallpox vaccination include inadvertent inoculation (nonocular), superinfection of the vaccination site or regional lymph nodes, ocular vaccinia [14], generalized vaccinia (GV), eczema vaccinatum (EV), progressive vaccinia (PV), postvaccinial central nervous system (CNS) diseases including encephalitis, post-vaccinial encephalopathy (PVE) and encephalomyelitis (PVEM), myocarditis and/or pericarditis, and fetal vaccinia [15,16,17,18,19]. Such complications may result in severe disability, permanent neurological sequelae, and/or death [20, 21, 22].

Estimates of the risks of occurrence of serious complications after primary vaccination and revaccination based on safety surveillance studies conducted when smallpox vaccine was routinely administered are presented in Table 1 [15]. ACAM2000 is a live vaccinia virus that can be transmitted to persons who have close contact with the vaccine or vaccinees. The risks for close contacts who are exposed to a vaccinee are the same as those stated for vaccinees.

Table 1: Rates of Reported Complications Associated with Vaccinia Vaccinations (Cases/Million Vaccinations) [a]

Age (yrs)	<1		1-4		5-19		≥20		Overall rates [g]	
Vaccination status	Vaccinia-naïve	Previously vaccinated	Vaccinia-naïve	Previously vaccinated	Vaccinia-naïve	Previously vaccinated	Vaccinia-naïve	Previously vaccinated	Vaccinia-naïve	Previously vaccinated
**Inadvertent Inoculation [b]**	507.0	-- [f]	577.3	109.1	371.2	47.7	606.1	25.0	**529.2**	**42.1**
**Generalized vaccinia**	394.4	-- [f]	233.4	-- [f]	139.7	9.9	212.1	9.1	**241.5**	**9.0**
**Eczema vaccinatum**	14.1	-- [f]	44.2	-- [f]	34.9	2.0	30.3	4.5	**38.5**	**3.0**
**Progressive Vaccinia [c]**	-- [f]	-- [f]	3.2	-- [f]	-- [f]	-- [f]	-- [f]	6.8	**1.5**	**3.0**
**Post-vaccinial encephalitis**	42.3	-- [f]	9.5	-- [f]	8.7	-- [f]	-- [f]	4.5	**12.3**	**2.0**
**Death [d]**	5	--	0.5	--	0.5	--	unknown	--	**--**	**--**
**Total [e]**	**1549.3**	**-- [f]**	**1261.8**	**200.0**	**855.9**	**85.5**	**1515.2**	**113.6**	**1253.8**	**108.2**

a. Adapted from Lane JM, Ruben FL, Neff JM, Millar JD. Complications of smallpox vaccination, 1968: results of ten statewide surveys. J Infect Dis. 1970; 122:303-309. [17]
b. Referenced as accidental implantation.
c. Referenced as vaccinia necrosum.
d. Death from all complications.
e. Rates of overall complications by age group include complications not provided in this table, including severe local reactions, bacterial superinfection of the vaccination site, and erythema multiforme.
f. No instances of this complication were identified during the 1968 10-state survey.
g. Overall rates for each complication include persons of unknown age.

More recent data on the incidence of AEs among adult military personnel and civilian first responders vaccinated with Dryvax® during pre-event programs starting in December 2002/January 2003 are presented in Table 2. These data show significant decreases in the incidence of preventable AEs (eczema vacinatum, contact transmission, and auto-inoculation) as compared to historical data, probably reflecting better screening procedures and routine use of protective bandages over the inoculation site. Myocarditis and/or pericarditis was not a common AE reported in the surveys of the 1960s [23], but emerged as the most frequent serious adverse event (SAE) in the US Department of Defense (DoD) and Department of Health and Human Services (DHHS) programs with incidence rates of 117.71 [24] and 519.52 [25] cases per million vaccinations, respectively.

Table 2: Anticipated and Unanticipated SAEs Associated With Large-scale Immunization Programs by the DoD and DHHS

**Adverse event**	**DoD program (n=730,5801 [a]) as of Jan05**		**DHHS program (n=40, 422) [b] as of Jan04**	
	**N**	**Incidence /million**	**N**	**Incidence /million**
Myo/pericarditis	89	117.71	21	519.52
Post-vaccinal encephalitis	1	1.37	1	24.74
Eczema vaccinatum	0	0	0	0
Generalized vaccinia	43	58.86	3	74.22
Progressive vaccinia	0	0	0	0
Fetal vaccinia	0	0	0	0
Contact transmission	52	71.18	0	0
Auto-inoculation (nonocular)	62	84.86	20	494.78
Ocular vaccinia	16	21.9	3	74.22

a. 71% primary vaccination; 89% male; median age 28.5 yr.
b. 36% primary vaccination; 36% male; median age 47.1 yr.

2. PRODUCT OVERVIEW

2.1. ACAM2000 Product Description

Acambis Inc. has developed a live Smallpox Vaccine, ACAM2000 [Proper name: Smallpox Vaccine (Vero Cells), Lyophilized], containing live vaccinia virus derived by plaque purification cloning from the licensed calf-lymph produced vaccine, Dryvax® Dried Smallpox Vaccine (Dryvax®, Wyeth Laboratories, Marietta, PA). The goal in developing ACAM2000 was to produce a clonally pure virus derived from Dryvax® that would provide effective protection against smallpox disease, possess an acceptable safety profile, and which could be manufactured efficiently in serum-free tissue culture (Vero cells) to produce a second generation, purified, and lyophilized smallpox vaccine. The advantages with cell culture based manufacturing over production in calf skin include better control for adventitious agents and a more consistent product quality with higher purity.

ACAM2000 is manufactured in compliance with current Good Manufacturing Practice (cGMP) regulations with cell harvest production performed by Baxter International, Inc. and bulk processing/purification and formulation being carried out by Acambis. Consistent cGMP manufacturing based on a well controlled process has been demonstrated with approximately 75 lots, representing over 190 million doses, being supplied to the UUS Strategic National Stockpile (SNS).

ACAM2000 is a lyophilized preparation of purified live vaccinia virus containing the following non-active excipients:

- 6-8 mM HEPES (pH 6.5-7.5)
- 2% human serum albumin United States Pharmacopeia (USP)
- 0.5 -0.7% sodium chloride USP
- 5% mannitol USP

Trace levels of residual neomycin and polymyxin B from the manufacturing process may be present in the vaccine. The diluent for ACAM2000 contains 50% (v/v) Glycerin USP, 0.25% (v/v) Phenol USP in Water for Injection USP, and is supplied in 3 mL clear glass vials containing 0.6 mL of diluent.

Each vial of ACAM2000 is reconstituted using 0.3 mL of the supplied diluent. After reconstitution, each vial of ACAM2000 vaccine contains 100 nominal doses (0.0025 mL/dose) and is stable at 2-8 °C for up to 30 days. The concentration of vaccinia virus in the reconstituted solution is $1.0\text{-}5.0 \times 10^8$ PFU/mL determined by plaque assay in Vero cells. One vial of diluent, 100 bifurcated needles and a 1-mL syringe are necessary for use of each vial of vaccine.

2.2. Indication

ACAM2000 is indicated for active immunization against smallpox disease for persons determined to be at risk for smallpox infection. The determinations of those at risk are developed and managed in accordance with the policies governing the use of a SNS product and DoD regulations.

2.3. Strategic National Stockpile (SNS)

ACAM2000 was produced under a contract awarded by the CDC in November 2001 for the development and manufacture of a second generation smallpox vaccine. Under this contract more than 192.5 million doses have been manufactured and supplied to the US Government SNS. This vaccine may be provided for military vaccination and to others at risk of smallpox infection post licensure. Acambis is also awaiting a contract grant (pending licensure) to maintain an ongoing manufacturing capacity of ACAM2000

ACAM2000 will not be marketed for general commercial distribution. The product will be manufactured solely for the following groups:

1. The CDC of the US Government for stockpiling at the SNS and for limited use in accordance with CDC and DoD regulations,
2. The World Health Organization (WHO) for stockpiling, and
3. Foreign governments for stockpiling and potential use outside the US.

3. DEVELOPMENT OF ACAM2000 (SMALLPOX VACCINE)

3.1. Required Vaccine Specifications

As part of the Request for Proposal (RFP) issued by the CDC on November 1, 2001, specifications for a new smallpox vaccine were established. Acambis modeled the development of a second generation smallpox vaccines based on these specifications. These specifications included, but were not limited to:

- Vaccine administration via the percutaneous route by bifurcated needle.
- Packaged as wet-frozen or freeze-dried preparation in units of 100 doses per container.
- Minimum potency of 1×10^8 PFU/mL.
- Produced in cell-culture from fully qualified, current cGMP compliant cell banks.
- Derived from the NYCBH Laboratory strain of vaccinia virus, or comparable strains of known efficacy against smallpox virus.
- Scalable manufacture capable of production of up to 250 million doses in a 12 month period.

3.2. Development of ACAM2000

In order to develop a modern smallpox vaccine, Acambis first characterized six cloned vaccine candidates, isolated by plaque-purification, and a polyclonal virus; all were derived from the Dryvax® NYCBH vaccinia virus strain and grown in cell cultures of diploid human embryonic lung (MRC-5) cells [26]. Based on its reduced neurovirulence in suckling mice and similarity to Dryvax® in other characteristics, clone number 2 was selected as the best candidate for further development and was renamed ACAM1000.

The ACAM1000 master virus seed (Passage 7) was used to prepare the second candidate vaccine, ACAM2000, by growth in a continuous line of African green monkey kidney (Vero) cells under serum-free conditions (cell line established and provided by Baxter International, Inc.). The ACAM2000 production virus seed and smallpox vaccine are 8 and 10 passages, respectively, from Dryvax® (Figure 1). The ACAM2000 virus is treated with endonuclease enzyme (Benzonase) to digest cellular nucleic acids, and is purified by ultrafiltration and diafiltration to produce the vaccine product.

The vaccine product was shown to be negative for a variety of adventitious agents including mycoplasma and viruses. The genomes of ACAM1000 master virus seed (Passage 7) and ACAM2000 vaccine (Passage 10) were sequenced at St. Louis University, St. Louis, MO, and CDC, Atlanta, GA, respectively, and found to be identical [27]. Therefore, ACAM1000 (Passage 9) and ACAM2000 (Passage 10) may be considered equivalent. ACAM1000 and ACAM2000 were shown to have similar safety and immunogenicity profiles in nonclinical studies and in Phase 1 and 2 clinical studies. Based on these findings and the recommendation by a Joint Down-Selection Working Group of the National Vaccine Advisory Committee (NVAC) and the

Figure 1: Passage History of ACAM1000 and ACAM2000

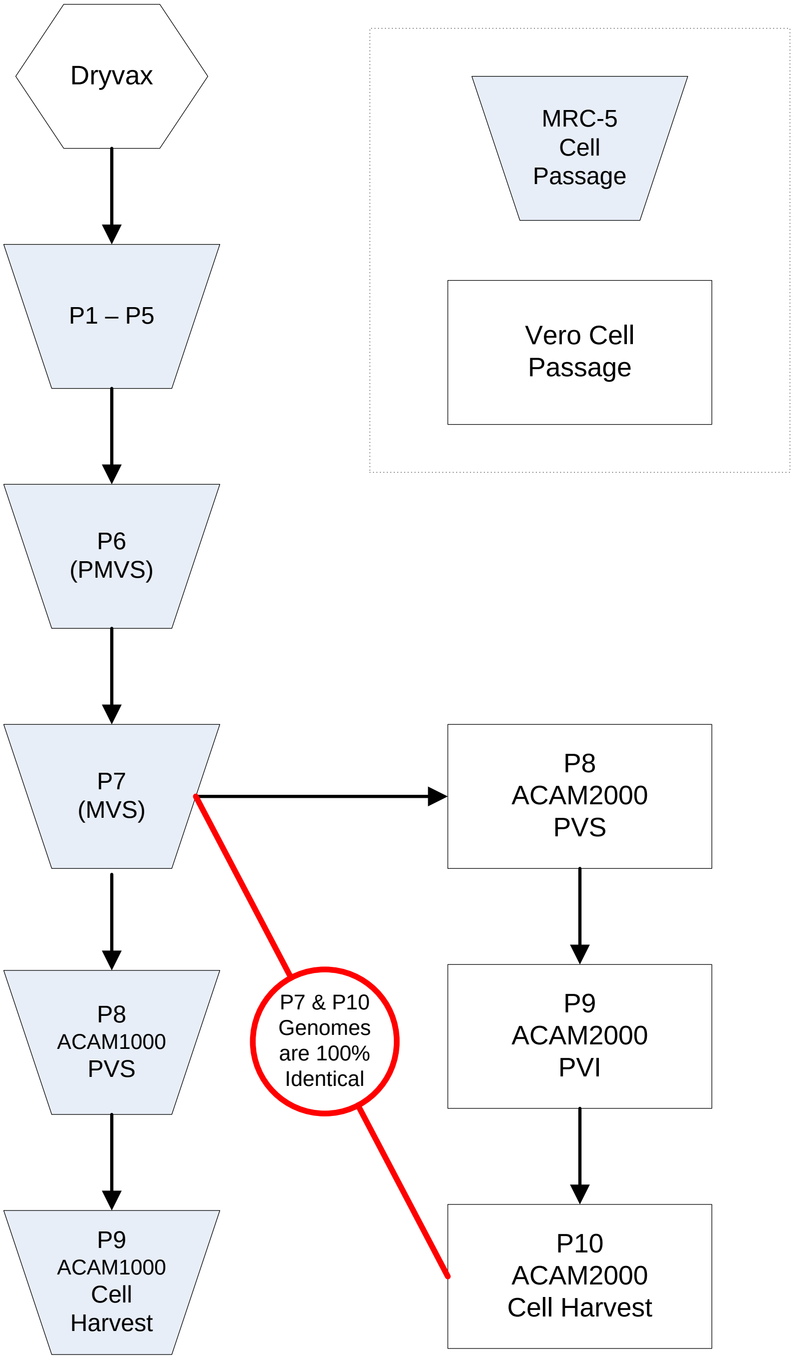

MVS = Master Virus Seed; P = Passage; PMVS = Pre-master Virus Seed; PVI = Production Virus Inoculum; PVS = Production Virus Seed

Defense Science Board, the CDC recommended in February 2003 that Acambis stop the co-development of ACAM1000 and focus its manufacturing and clinical study resources solely on the production and development program of ACAM2000. Data obtained in nonclinical and clinical studies of ACAM1000 prior to selection of ACAM2000 are considered to be supportive of data obtained with ACAM2000.

4. NON-CLINICAL STUDIES OF ACAM2000

4.1. Non-Clinical Evaluations

ACAM2000 was tested in nine non-clinical studies in order to evaluate its safety and efficacy. In designing the non-clinical testing program, Dryvax® treated groups were included in accordance with the CDC requirement that the vaccine candidate be developed to molecular and non-clinical biological criteria similar to DryVax®. Dryvax® is the only US Food and Drug Administration (FDA)-licensed smallpox vaccine in the US and provided a reliable standard against which to measure immunogenicity and virulence potential in animal systems. Table 3 presents a summary of the non-clinical studies conducted with ACAM2000 and with the ACAM2000 Master Seed Virus (ACAM1000).

4.1.1. Non-Clinical Efficacy

With the worldwide eradication of smallpox, protective efficacy studies are no longer possible for smallpox vaccine candidates. Therefore, the animal challenge studies, as summarized in Table 3, provide very important supportive data for the efficacy of ACAM2000.

The primate challenge study performed at the Battelle Memorial Institute is of particular note relative to vaccine efficacy. In this study twenty four (24) cynomolgus monkeys, were randomized into three groups (8 monkeys per group). Each group received inoculations of either ACAM2000 (potency=4.4 x 10^8 PFU/mL), Dryvax® (potency=1.5 x 10^8 PFU/mL), or a negative control material (an equivalent volume of ACAM2000 diluent). All were vaccinated on Day 0 and were challenged with a virulent dose (3.8 x 10^7 PFU) of monkeypox virus at Day 61. Monkeys were observed for morbidity and mortality for 30 days post challenge. The following study observations were made:

- Seroconversion (≥4-fold rise in neutralizing antibodies between Days 0 and 30) rates were identical for ACAM2000 and Dryvax® (100% each) whereas placebo treatment resulted in 0% seroconversion. The geometric mean titer (GMT) for ACAM2000 was160 (range 40-320) and Dryvax® was 174 (range 40-640).
- Those vaccinated with either ACAM2000 or Dryvax® developed cutaneous lesions characteristic of a “take” or positive dermal response as observed in human subjects.
- Post-challenge (day 91) antibody results for both ACAM2000 and Dryvax® showed greater than 200-fold increases in GMT. The control group also developed a detectable antibody titer post-challenge (Day 67 or 69) with a GMT of 227 (range 70 to 946); however, this was not protective as all 8 controls succumbed to the infection.
- All monkeys (8) vaccinated with the ACAM2000 vaccine survived the lethal challenge with little to no apparent clinical signs of infection, as did the 8 monkeys in the Dryvax® group.
- No detectable viremia was found in animals vaccinated with either the ACAM2000 or the Dryvax® vaccine at any point during the post-challenge period.
- No relevant clinical symptoms or pathological changes were observed in either the ACAM2000 or the Dryvax® vaccinated animals.

Based upon the results of this study, ACAM2000 was determined to be immunogenic and fully efficacious in protecting cynomolgus monkeys from a fatal monkeypox challenge. No post-challenge viral replication was observed in any biological sample tested from the ACAM2000 treatment group and no significant clinical symptoms were observed in any of the ACAM2000 vaccinated animals. All control monkeys receiving placebo (ACAM2000 diluent) succumbed to the virulent monkeypox challenge with viremia tissue virus replication, and numerous monkeypox-specific clinical symptoms.

In summary, the non-clinical studies summarized in Table 3 demonstrate that ACAM2000 elicits neutralizing antibody and T cell responses in mice and monkeys that are sufficient to provide protection against a lethal challenge dose of orthopox virus. The non-clinical studies further support the conclusion that the immunogenicity of ACAM2000 is comparable to Dryvax® based on dose response, and that ACAM2000 has less neurovirulence and cutaneous virulence potential than Dryvax® at similar dose levels.

Table 3: Non-Clinical Studies Conducted with ACAM2000 Vaccine, and Master and Production Virus Banks

Study Type/Test Article	Study Results
Immunogenicity in mice following percutaneous administration / ACAM2000 Vaccine	ACAM2000 (1×10^6 or 1×10^8 PFU/mL) was associated with an increase in neutralizing antibody titer and a T cell response comparable to that observed for Dryvax® at similar doses.
Protection against Vaccinia Western Reserve (WR) Virus challenge in mice following percutaneous administration / ACAM2000 Vaccine	ACAM2000 provided equivalentprotection to Dryvax® based on the survival of all vaccinia WR virus-challenged mice at ACAM2000 doses of 1×10^6 and 1×10^7 PFU/mL
Immunogenicity and protection against Monkeypox Virus challenge in monkeys following percutaneous administration / ACAM2000 Vaccine	ACAM2000 (4.4×10^8 PFU/mL) was associated with an increase in neutralizing antibody titer comparable to that observed for Dryvax® (1.5×10^8 PFU/mL) and provided equal protection of all monkeypox-challenged monkeys.
Cutaneous virulence in rabbits following percutaneous administration / ACAM2000 Vaccine	Average erythema and lesion diameters observed in the ACAM2000 groups were less than or equivalent to those in the Dryvax® groups at the same dose levels.
Neurovirulence in mice following intracerebral administration / Unpurified ACAM2000 Vaccine	Average survival times (AST) in ACAM2000-treated mice were greater than AST in Dryvax®-treated mice, except at the highest dose administered (2,000 PFU/mouse). 50% lethal dose (LD_{50}) value was higher for ACAM2000 than for Dryvax®.
Neurovirulence in mice following intracerebral administration / ACAM2000 Vaccine	AST were greater in the ACAM2000 treated mice at all doses compared to the Dryvax® treated mice. LD_{50} value was higher for ACAM2000 than for Dryvax®.
Neurovirulence in mice following intracerebral administration / ACAM2000 Vero Production Virus Bank P8	Mortality was significantly lower (p=0.0003) in mice treated with the ACAM2000 Production Virus Bank compared to mice treated with Dryvax®.
Neurovirulence in mice following intracerebral administration / ACAM2000 Vero-Vaccinia TFF Retentate (ACAM2000 Drug Substance)	Mortality was similar and not significantly lower in mice treated with the ACAM2000 Drug Substance compared to mice treated with Dryvax®.
Neurovirulence in monkeys following intrathalamic administration / ACAM1000 Master Seed Virus	Three of six monkeys treated with Dryvax® (4.9×10^7 PFU) died during the study. There were no deaths among six monkeys treated with ACAM1000 Master Seed Virus (1.25×10^7 PFU). Clinical illness scores were lower and clinical signs of vaccine-related meningitis were less severe in the ACAM1000 Master Seed Virus treated animals than the Dryvax® treated animals.

5. CLINICAL DEVELOPMENT OF ACAM2000

5.1. Overview

ACAM2000 was evaluated in six clinical studies sponsored by Acambis Inc., summarized in Table 4. A total of 3881 subjects received either ACAM2000 (2983), ACAM1000 (30, all in Study H-400-002), or Dryvax® (868) in these studies. Of these subjects, 2981 were enrolled in two Phase 3 studies.

Table 4: Clinical Studies Conducted in the ACAM2000 Clinical Program

	Study Number	Subject Population	Total Subjects
**Phase 1**	H-400-002	Vaccinia-Naïve	90
	H-400-008	Vaccinia-Naïve	100
**Phase 2**	H-400-003	Previously Vaccinated	357
	H-400-005	Vaccinia-Naïve	353
**Phase 3**	H-400-009	Vaccinia-Naïve	1162
	H-400-012	Previously Vaccinated	1819
		**Overall Total**	3881

The Phase 1 studies were designed to evaluate the safety, tolerability, and immunogenicity of the ACAM1000 and ACAM2000 vaccines in vaccinia-naïve subjects (note that H-400-002 compared ACAM1000 to ACAM2000 to Dryvax®). The Phase 2 studies further assessed the safety and efficacy of ACAM2000 compared to Dryvax® in both vaccinia-naïve and previously vaccinated subjects and evaluated the dose response to the vaccine. The Phase 3 studies were designed to assess the safety and efficacy of ACAM2000 based on non-inferiority to Dryvax® in the vaccinia-naïve and the previously vaccinated populations, as well as the evaluation of clinical lot consistency for the ACAM2000 vaccine.

5.2. Standard Procedures in Clinical Studies of ACAM2000

The following research procedures and criteria were used in clinical studies conducted with ACAM2000.

5.2.1. Study Design

With the exception of Study H-400-008, all clinical studies in this series were parallel group, double-blind studies with Dryvax® as an active control. Following a screening period, all subjects were vaccinated on Day 0 and safety and efficacy assessments were obtained through Day 30 (Day 45 for study H-400-002) as described below.

5.2.2. Administration of Vaccine

Smallpox vaccine was administered percutaneously via fifteen vigorous strokes with a bifurcated needle in the skin of the upper arm over the deltoid muscle, with a small amount of blood subsequently appearing at the vaccination site. In all studies in the ACAM2000 clinical

program, subjects received a single administration of smallpox vaccine via 15 pokes with a bifurcated needle.

5.2.3. Subject Populations for Efficacy Studies

ACAM 2000 has been studied in two subject populations: 1. those who have not been previously vaccinated for smallpox (vaccinia-naïve), and 2. those previously vaccinated with vaccinia.

Subjects enrolled into studies designed to evaluate responses in vaccinia-naïve populations were aged 18 to 30 years and did not have a smallpox vaccination scar at Baseline.

Those enrolled into studies designed to evaluate responses in previously vaccinated populations ranged in age from 29 to 84 years. The majority (≥97%) had a confirmed vaccination scar at Baseline. All subjects in this population met the protocol criterion of >10 years since previous vaccination. In studies with previously vaccinated subjects, the mean time between study vaccination and their last previous vaccination was similar in the ACAM2000 (42 years) and Dryvax® (41 years) groups.

Specific analysis populations were defined in each of the studies conducted with ACAM2000 to assist in interpretation of the study data. These populations are listed and defined in Table 5.

Table 5: Analysis Populations in Clinical Studies of ACAM2000

Analysis Population	Definition	
	**Vaccinia-Naïve subjects**	**Previously vaccinated subjects**
Intent to Treat (ITT)	All subjects who received vaccination regardless of any post-vaccination assessments. These subjects may or may not have had cutaneous reaction assessments.	
Safety	All subjects who received vaccination (same as ITT population) and had follow-up for safety	
Dermal Evaluable	All subjects who received vaccination, had no major protocol violations, and were assessed for local cutaneous reaction between Days 6 and 11.	All subjects who received study vaccination, had no major protocol violations, were assessed for local cutaneous reaction between Days 6 and 8, inclusive, and were evaluated for vaccination response by the Independent Review Committee, based on photographic evidence.
Antibody Evaluable (AnE)	All subjects who were randomly assigned to have serum samples analyzed for neutralizing antibody response, received vaccination, were seronegative for vaccinia at Baseline [i.e., had a neutralizing antibody titer <10 (set to 5 for analysis purposes)], had no major protocol violations, and had sera collected for vaccinia antibody assessments at Baseline (Day 0) and on Day 30 (±3 days).	All subjects who were randomly assigned to have serum samples analyzed for neutralizing antibody response, received vaccination, had no major protocol violations, and had sera collected for vaccinia antibody assessments at Baseline (Day 0) and on Day 30 (±3 days).

5.2.4. Primary Efficacy Endpoints

Based on FDA guidance pertaining to the development of second generation smallpox vaccines [28] and communications with the Agency, Acambis' clinical trials have been designed to evaluate the ACAM2000 vaccine based on two surrogate endpoints for efficacy, specifically, the rate of vaccination success based on the proportion of subjects displaying a major cutaneous response, and the vaccinia-specific neutralizing antibody GMT.

The two surrogate endpoints for efficacy were established based upon the following observations:

a. In historical practice, vaccinated individuals were considered fully protected against smallpox after a major cutaneous reaction was observed [29].
b. Two prospective studies have correlated higher titers of neutralizing antibodies with lower susceptibility to smallpox upon contact with a smallpox victim [10, 5]. Although the methods used to measure neutralizing antibody titers differ between these published studies and Acambis' clinical trials, the data indicate that neutralizing antibody titer serves as a qualitative, if not a quantitative, correlate of protection. The limited data from these studies suggested that neutralizing antibody titers greater than 32 may correlate with protection [10, 5] although this value cannot be validated due to eradication of the disease.

Definition of Successful Vaccination and Revaccination Based on Dermal Response

The definitions of successful vaccination and revaccination used in ACAM2000 clinical studies were consistent with the Advisory Committee on Immunization Practices (ACIP) [29] and WHO [3] definitions. Successful vaccination was defined as a vesicular or pustular lesion or an area of definite palpable induration or congestion surrounding a central lesion that might be a crust or an ulcer. Since skin reactions may be less pronounced for revaccination, an independent review committee (IRC) was used in the evaluation of vaccination success for the previously vaccinated subjects in the Phase 3 study. The IRC reviewed digital photographs of the vaccination site taken by study center personnel and assessed subjects' responses to vaccination. The review was blinded to study treatment. This evaluation was conducted because of the acknowledged difficulty in determining successful revaccination based on the examination of the cutaneous response and the need to objectively assess vaccination success in a standardized fashion across all subjects in a multicenter study. The IRC consisted of 3 smallpox experts.

Determination of Neutralizing Antibody Response

Neutralizing antibody response to smallpox vaccination was determined using a 50% plaque reduction neutralization test ($PRNT_{50}$). The method determines the dilution of a test serum which, when pre-incubated with a fixed number of PFU of the vaccine virus strain, results in a 50% reduction in plaques when the virus-serum mixture is plated on Vero cells. The method was developed and validated by Acambis. The test serum titer is reported as the reciprocal of the dilution resulting in 50% plaque reduction. An arbitrary neutralizing antibody titer of 5 was assigned to a serum sample with no detectable neutralizing antibody at a dilution of 1:10. In order to determine the neutralizing antibody response in vaccinated populations, GMTs were calculated.

5.2.5. Evaluations of Vaccine Safety

The safety of study vaccine was assessed by structured interviews and subject diaries following vaccination, recording of concomitant medications, assessment of vital signs including body temperature, physical examination findings, and clinical laboratory tests. In a Phase 1 study (H-400-002) this was supplemented by measurements of virus shedding from the vaccination site, and in the Phase 3 studies and Study H-400-002, assessments were made post-vaccination of electrocardiogram (ECG) findings and cardiac troponin I measurements. Female subjects of child-bearing age were routinely tested for pregnancy using standard serum β-human chorionic gonadotropin (β-HCG) assays.

AEs were solicited via structured interviews at scheduled study center visits, with specific prompts for AEs historically associated with smallpox vaccination. A diary to be completed by the subject served as an aid to memory for the interview In order to identify potential symptoms of myocarditis/pericarditis, subjects in the Phase 2 studies (after 28 March 2003) and Phase 3 studies were also questioned for symptoms of chest pain, shortness of breath, heart palpitations, and reduced tolerance to exercise.

Surveillance for selected cardiac, dermatologic and neurologic AEs was intensified due to reports of cardiac AEs in the civilian and US DoD vaccination programs utilizing Dryvax® [30,31]. In the Phase 3 studies (Studies H-400-009 and H-400-012) and one Phase 1 study (Study H-400-002), clinical algorithms were included to help the Investigator with the recognition of clinically apparent vaccinia-related myocarditis or pericarditis, specific dermatologic reactions, and neurologic complications associated with smallpox vaccination.

In clinical studies of ACAM2000, with the exception of Phase 1 Study H-400-008, serious and non-serious AE data were reviewed at scheduled intervals by a Data Safety Monitoring Board (DSMB) during the conduct of the studies, with stopping rules in place in the event any SAE was considered to be study-vaccine related in subjects or in contacts of subjects. Furthermore, a blinded Cardiology Advisory Panel (CAP) reviewed all serious cardiac events, abnormal ECG findings and troponin I levels in Studies H-400-009 and H-400-012 at the request of the DSMB, with the goal of providing a consensus diagnosis for each case of suspected/probable myocarditis or pericarditis and identifying additional cases by review of ECG and troponin I abnormalities.

6. CLINICAL EFFICACY RESULTS

This section provides a brief description of the major results and conclusions from each of the clinical studies which pertain to the clinical efficacy of the ACAM2000 smallpox vaccine. The Phase 3 efficacy results presented in this section are based on the analysis of clinical data which excluded four clinical sites (all treated subjects were considered for safety analyses). The FDA requested that these sites not be included due to possible Good Clinical Practice (GCP) violations. However, there were no differences in efficacy conclusions from the Phase 3 studies, with or without the inclusion of data from these four clinical study sites. Data from an efficacy analysis that includes all sites is provided as an appendix in Section 11.1 of this briefing document for reference. An integrated assessment of the efficacy of ACAM2000 based on all of the clinical studies, including comparison to the currently licensed vaccine, Dryvax®, is provided in Section 6.4. The list of clinical studies conducted with ACAM2000 was provided in Section 5.1, Table 4.

6.1. Immunogenicity Results from Phase 1 Studies

Two Phase 1 studies, H-400-002 and H-400-008, were conducted in vaccinia-naïve subjects. Study H-400-008 was a Phase 1, open-label, single-arm, fixed-dose study designed to evaluate the safety, tolerability and immunogenicity of ACAM2000. The clinical lot used in this study had a potency of 7.7×10^{7} PFU/mL, which was slightly below the nominal target of 1.0×10^{8} PFU/mL. One hundred adults, aged 18 to 29 years, who were naïve to smallpox vaccine, were enrolled in the study. Ninety-nine percent of subjects (99 of 100 subjects) experienced a successful vaccination based on their cutaneous reactions. The GMT on Day 30 was 225.

Study H-400-002 was a randomized, double-blind study to compare the immunogenicity of ACAM1000 or ACAM2000 to Dryvax® in adults 18 to 29 years of age. A dose of 1.0×10^{8} PFU/mL was delivered in all treatment groups. Thirty (30) subjects were vaccinated in each treatment group. All subjects in each treatment group (30 of 30 subjects, 100%) experienced a successful vaccination based on cutaneous reaction. The GMT on Day 45 was 124.1, 103.2 and 171.5 in the ACAM1000, ACAM2000 and Dryvax® groups, respectively; the differences between groups were not significant ($p \geq 0.2273$).

Protection against smallpox disease is mediated by neutralizing antibodies and cytotoxic T cells, and both B and T cells provide long-term memory [32]. In study H-400-002, all subjects were evaluated at Day 45 for cytotoxic T lymphocytes (CTL assay), cytokine producing cells (γ-interferon ELISPOT assay), and vaccinia-specific lymphoproliferation (LPA assay). The results are summarized in Table 6. All 30 subjects (100%) in the ACAM2000 group, and 28 (93%) of 30 subjects in the Dryvax® group demonstrated a positive cell-mediated immune response to smallpox vaccine in at least one of the three T-cell assays. These results are in agreement with studies performed with Dryvax® for development of cytotoxic T-cell responses and increases in virus specific γ-interferon-producing cells in vaccinia-naïve subjects [33, 34].

Table 6: Proportion of Subjects with Positive T Cell Responses in Study H-400-002, by Treatment Group

Assay Method	Treatment Group	
	ACAM2000 (n = 30)	Dryvax® (n = 30)
CTL assay, n (%)	26 (87)	22 (73)
γ-interferon ELISPOT assay, n (%)	30 (100)	27 (90)
LPA assay, n (%)	29 (97)	26 (87)
At least one assay, n (%)	30 (100)	28 (93)

6.2. Summary of Efficacy Results from Phase 2 Studies

The immune response to ACAM2000 as a function of administered dose was evaluated and compared to Dryvax® in two Phase 2 studies. Study H-400-005 assessed the dose response to ACAM2000 in vaccinia-naïve subjects, while study H-400-003 evaluated the dose response in previously vaccinated (Dryvax®) individuals. The results of these studies are summarized below.

6.2.1. Study H-400-005: Vaccinia-Naïve Subjects

Study H-400-005 was a Phase 2, double-blind, randomized, active controlledtudy evaluating the safety, tolerability and immunogenicity of 4 dose levels of ACAM2000 compared to Dryvax® in 353 healthy vaccinia-naïve adults, ages 18-30. The comparator vaccine, Dryvax®, was administered at a dose of 1.6×10^8 PFU/mL and doses of ACAM2000 ranged from a high of 6.8×10^7 PFU/mL to a low of 3.4×10^6 PFU/mL. No data were collected on the dose response to Dryvax® in this study.

Subjects were vaccinated on Day 0 and asked to return to the clinic on study Days 3, 7, 10, 15 and 30. The efficacy of each vaccine dose was evaluated based on the rates of major cutaneous reactions on Days 7 or 10 and the neutralizing antibody responses on Day 30. The results of the study are presented below, in Table 7.

Table 7: Vaccination Success and Neutralizing Antibody Response as a Function of ACAM2000 Dose for Study H-400-005: Vaccinia-Naïve Subjects

Treatment group (PFU/Dose)	Dryvax® (1.6×10^8)	ACAM2000 (6.8×10^7)	ACAM2000 (1.4×10^7)	ACAM2000 (6.8×10^6)	ACAM2000 (3.4×10^6)
**Vaccination Success based on Cutaneous Response**					
Evaluable Subjects, n	49	51	100	100	49
Vaccination Success, n (%)	49 (100)	51 (100)	86 (86)	80 (80)	29 (59)
Difference from Dryvax® [a]	--	Undefined [b]	-22, -6	-29, -11	-57, -25
**Neutralizing Antibody Titer**					
Evaluable Subjects, n	49	51	100	100	50
GMT	158	154	101	61	31
Ratio of GMT [c]	--	0.468, 1.995	0.339, 1.202	0.209, 0.741	0.095, 0.914
Log_{10} GMT: Difference from Dryvax® [a]	--	-0.33, 0.30	-0.47, 0.08	-0.68, -0.13	-1.02, -0.39

a. Lower and upper bound of 95% CI on ACAM2000 difference from Dryvax®, as derived by normal approximation
b. Because the successful vaccination rates and the CIs for the ACAM2000 6.8×10^7 dose group and Dryvax® were identical, the difference between ACAM2000 6.8×10^7 versus Dryvax® could not be computed based on the normal approximation to the binomial.
c. Lower and upper bound of 95% CI on the ratio of ACAM2000 GMT/Dryvax® GMT

With ACAM2000, rates of successful vaccination based on cutaneous response ranged from a low of 59% in subjects administered the lowest dose of vaccine to a high of 100% in subjects administered the highest dose, as presented in Table 7 and graphically in Figure 2. Based on a probit analysis, which models the probability of a successful reaction as a function of dose, the ACAM2000 dose necessary to achieve a successful vaccination in 90% of vaccinia-naïve subjects was 1.5×10^7 PFU/mL, with an upper limit of the 95% confidence interval (CI) of 2.7×10^7 PFU/mL. Vaccination success (as determined by cutaneous response) was also found to be dose dependent in a study performed with Dryvax® in vaccinia-naïve subjects [13]. Similarly, GMTs were sensitive to the administered dose of ACAM2000. In the highest dose ACAM2000 group, the GMT was considered equivalent to that in the Dryvax® group. The neutralizing antibody titer declined with decreasing doses of ACAM2000 (Table 7 and Figure 3).

Figure 2: ACAM2000 Dose Response and Comparison to Dryvax®: Vaccination Success based on Cutaneous Response in Vaccinia-Naïve Subjects (Study H-400-005)

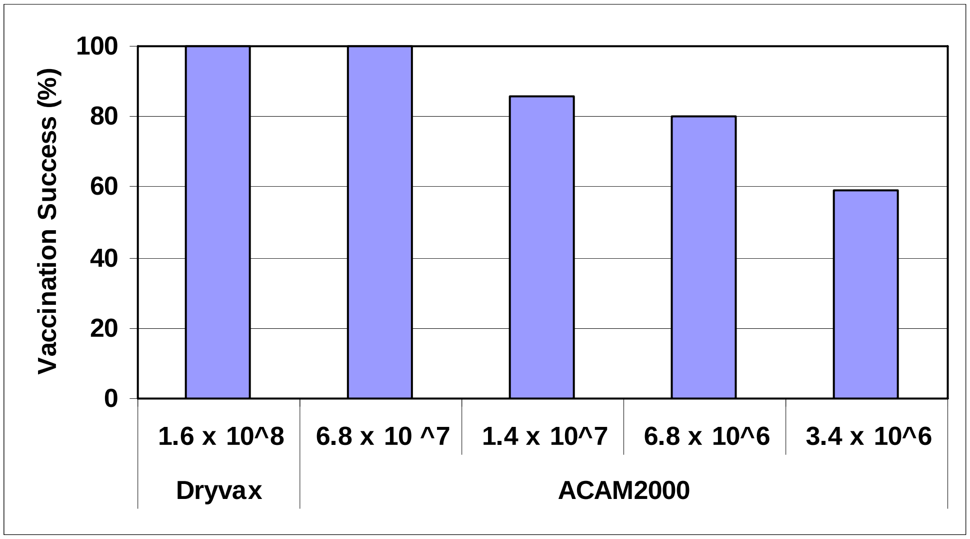

Figure 3: ACAM2000 Dose Response and Comparison to Dryvax®: Neutralizing Antibody GMT in Vaccinia-Naïve Subjects (Study H-400-005)

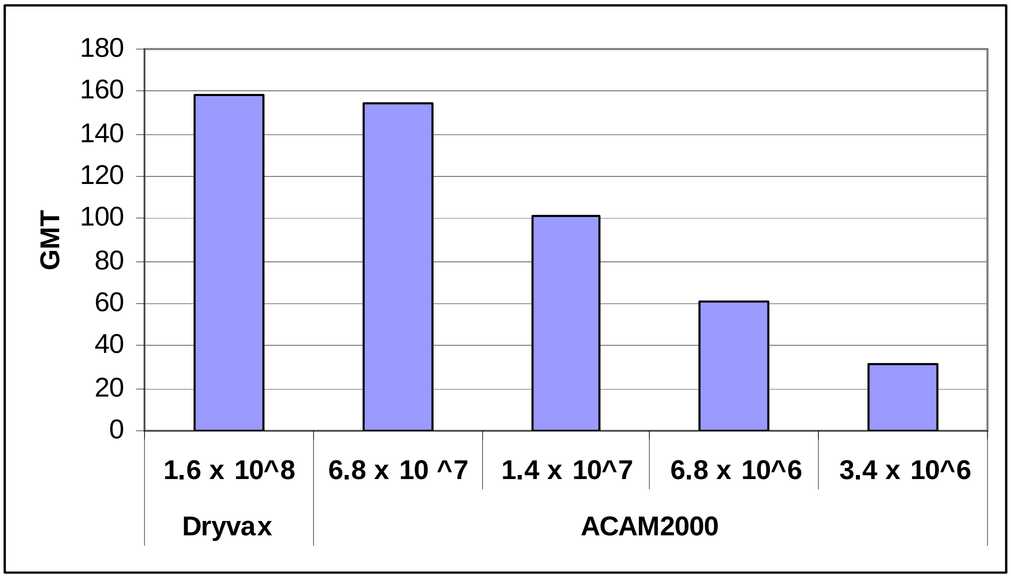

The highest dose ACAM2000 group (6.8×10^7 PFU/mL) was found to be substantially equivalent to Dryvax® for both measures of efficacy. The percentage of subjects with successful vaccination in the ACAM2000 6.8×10^7 PFU/mL dose group and the Dryvax® 1.6×10^8 group was 100% (Table 7). Each group had identical 2 sided exact 95% CIs of 93% and 100%. Similarly, the GMTs for the ACAM2000 6.8×10^7 dose group and the Dryvax® group (154 and 158, respectively) may be considered equivalent since the 2-sided 95% CI on the ratio of the geometric mean titers fell approximately between 0.5 and 2.0 (actual value 0.468 to 1.995). In contrast, all lower dose groups of ACAM2000 were found to be not statistically equivalent to Dryvax® for both primary efficacy measures.

6.2.2. Study H-400-003: Previously Vaccinated (Dryvax®) Subjects

Study H-400-003 was a Phase 2, double-blind, randomized study designed to evaluate the safety, tolerability and immunogenicity of four dose levels of ACAM2000 versus Dryvax® in subjects previously vaccinated with Dryvax® smallpox vaccine. Healthy adults, aged 28 years or older, who had received smallpox vaccine more than 10 years previously, were enrolled in the study at 3 centers in the US. There were no significant differences between subjects in each treatment with respect to demographic variables or baseline characteristics. The majority of subjects in all groups were seropositive for vaccinia antibodies at Baseline.

No data were collected on the dose response to Dryvax® in this study.

Subjects were inoculated on Day 0 and instructed to return to the clinic on Days 3, 7, 10, 15 and 30. At each visit, the size and appearance of the local cutaneous reaction was assessed and recorded. Blood samples were collected on Day 30 for serum neutralizing antibody assays.

Table 8 presents the rates of successful revaccination (based on cutaneous response) and the neutralizing antibody GMTs observed for each dose group at Day 30.

Table 8: Vaccination Success based on Cutaneous Response and Neutralizing Antibody Response as a Function of ACAM2000 Dose for Study H-400-003: Previously Vaccinated (Dryvax®) Subjects

Treatment group (PFU/Dose)	Dryvax® (1.6×10^8)	ACAM2000 (6.8×10^7)	ACAM2000 (1.4×10^7)	ACAM2000 (6.8×10^6)	ACAM2000 (3.4×10^6)
**Revaccination Success based on Cutaneous Response**					
Evaluable Subjects	52	49	101	100	49
Revaccination Success, n (%)	52 (100)	43 (88)	52 (51)	40 (40)	13 (27)
Difference from Dryvax® [a]	--	-23, -1	-60, -37	-71, -49	-88, -59
**Neutralizing Antibody Titer**					
Evaluable Subjects	52	50	102	102	51
GMT	447	256	115	84	59
Ratio of GMT [b]	--	0.309, 1.072	0.151, 0.447	0.110, 0.324	0.071, 0.245
Log_{10} GMT: Difference from Dryvax® [a]	--	-0.51, 0.03	-0.82, -0.35	-0.96, -0.49	-1.15, -0.61

a. Lower and upper bound of 95% CI on ACAM2000 difference from Dryvax®, as derived by normal approximation.
b. Lower and upper bound of 95% CI on the ratio of ACAM2000 GMT/Dryvax® GMT.

The rates of successful revaccination based on cutaneous response were 88%, 51%, 40%, and 27% in the ACAM2000 6.8×10^7 PFU/dose, 1.4×10^7, 6.8×10^6 PFU/dose, and 3.4×10^6 PFU/dose groups, respectively, and 100% in the Dryvax® group (1.6×10^8 PFU/dose). As was observed for the vaccinia-naïve population, a dose response was seen for ACAM2000 with respect to revaccination success rate and GMT. This dose-dependency of the vaccination success rates is also consistent with the results of a study in previously vaccinated subjects when Dryvax® was diluted prior to administration [35]. In summary, this study demonstrated that in previously vaccinated individuals, the highest ACAM2000 dose group (6.8×10^7) was not equivalent to Dryvax® with respect to revaccination success (as determined by cutaneous response) or GMT.

However, it should be noted that at all dose levels of ACAM2000 the GMTs were higher for the revaccinated subjects than the vaccinia-naïve subjects. (Figure 4).

Figure 4: ACAM2000 GMTs for Vaccine-Naïve vs. Previously Vaccinated Subjects (Studies H-400-003 and H-400-005)

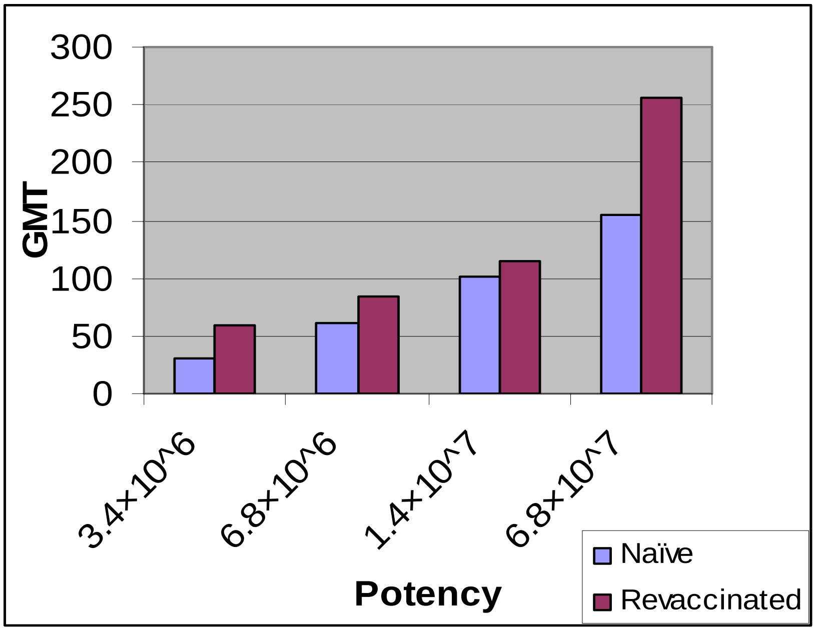

6.3. Summary of Efficacy Results from Phase 3 Studies

The efficacy of ACAM2000 was evaluated and compared to Dryvax® in two Phase 3 studies. Study H-400-009 assessed the response to ACAM2000 and Dryvax® in a vaccinia-naïve population, while Study H-400-012 compared the response to both products in a previously vaccinated population. Both studies used ACAM2000 lots with potencies ranging from 1.3-2.2×10^8 PFU/mL and Dryvax® with a potency of 1.5×10^8 PFU/mL.

Study H-400-009 (vaccinia-naïve subjects) found that ACAM2000 was non-inferior to Dryvax® with respect to cutaneous response, but narrowly missed the endpoint for non-inferiority with respect to GMT (97.5% CI lower bound on the difference in $\log_{10}$ GMT for non-inferiority was pre-defined at ≥-0.301 and the study outcome was -0.307). On the other hand, study H-400-012 (previously vaccinated subjects) found that ACAM2000 was statistically non-inferior to Dryvax® with respect to GMT, but did not meet the endpoint for non-inferiority with respect to the cutaneous response.

The results of the final analysis of each study are summarized in further detail below, including a discussion of why ACAM2000 is considered efficacious despite the fact that the GMT of vaccinia-naïve subjects receiving ACAM2000 and Dryvax narrowly missed the statistical margin to demonstrate non-inferiority and that ACAM2000 does not elicit the same frequency of cutaneous response as Dryvax® in previously vaccinated subjects.

6.3.1. Phase 3 Study H-400-009: Vaccinia-Naïve Subjects

Study H-400-009 was a Phase 3, double-blind, randomized study designed to evaluate the safety and immunogenicity of ACAM2000 versus Dryvax® in adults 18 to 30 years of age, inclusive, who were naïve to smallpox vaccine. Subjects were randomized 3:1 to receive ACAM2000 and Dryvax®, respectively. In addition, consistency was confirmed by comparing 3 conformance lots of ACAM2000. The original design called for enrollment of 2720 healthy subjects. However, observed rates of occurrence of myocarditis/pericarditis were unexpectedly higher than those previously published for smallpox vaccine in both the ACAM2000 and Dryvax® treatment groups. Cosequently, Acambis voluntarily suspended enrollment into this study. Following the DSMB review of the safety data, in conjunction with the analyses provided by a CAP, the DSMB concluded that if more data were needed to meet the primary efficacy end points, then the studies could continue as planned, with a revised informed consent describing the new findings on the risk of myocarditis/ pericarditis. However, Acambis calculated at the time that there was sufficient power to meet the objectives of the Phase 3 studies, and with the concurrence of the FDA, closed the study, resulting in a smaller sample size than originally planned.

Calculations performed after the studies were prematurely terminated and the 4 clinical sites removed (per FDA's request) indicate that the power for study H-400-009 (Vaccinia-Naïve) vaccination success (cutaneous response) was ≥ 80% and the power for neutralizing antibody GMT was >90%. For Study H-400-012 (Previously Vaccinated), the power for revaccination success (cutaneous response) and neutralizing antibody GMT was 72% and 85%, respectively.

A total of 1162 subjects were enrolled at 69 study centers in the US and its territories of which 1033 subjects at 65 sites qualified for the final analysis for dermal response There were no significant differences between the Dryvax® and ACAM2000 groups with regard to demographic or baseline characteristics. A summary of demographic information for all patients enrolled in ACAM2000 studies, including Phase 3, is provided as an appendix in Section 11.2.

At Baseline (Day 0), subjects received a double-blind percutaneous vaccination in the deltoid region with ACAM2000 or Dryvax®. On Days 7, 10, 21, and 30 post-vaccination, study personnel examined the vaccination site and scored the local cutaneous reaction. Blood samples for assay of serum neutralizing antibodies were obtained at the final study center visit on Day 30.

The effectiveness of study vaccine was determined by two co-primary endpoints: the proportion of subjects with a successful vaccination based on the investigators' assessments of cutaneous reaction on Day 7 and/or 10, and GMT of neutralizing antibodies on Day 30. The endpoints were evaluated based on statistical tests for the non-inferiority of ACAM2000 compared to Dryvax®. Table 9 summarizes the efficacy results of study H-400-009 for ACAM2000 versus Dryvax®. Vaccination success (as determined by cutaneous response) and GMT results are plotted in Figure 5.

Table 9: Phase 3 Efficacy Results for ACAM2000 versus Dryvax® in Vaccinia-Naïve Subjects (Study H-400-009)

Treatment group	ACAM2000	Dryvax®
**Vaccination Success based on Cutaneous Response**		
Evaluable Subjects (Dermal Evaluable Population)	776	257
Vaccination Success, n (%)	747 (96)	255 (99)
97.5% CI [a] (Criterion for Non-Inferiority)	-4.67 (>-5.00)	
**Neutralizing Antibody Titer**		
Evaluable Subjects (Antibody Evaluable Population)	565	190
GMT	166	255
97.5% CI [b] (Criterion for Non-Inferiority)	-0.307 (>-0.301)	

a. Lower bound of the 97.5% one-sided CI on the difference between ACAM2000 and Dryvax® derived by normal approximation to the binomial distribution.
b. Lower bound of the 97.5% one-sided CI on the difference in the mean log_{10} GMT between ACAM2000 and Dryvax® derived using analysis of variance (ANOVA).

Figure 5: Graphical Presentation of Phase 3 Efficacy Results in Vaccinia-Naïve Subjects (Study H-400-009)

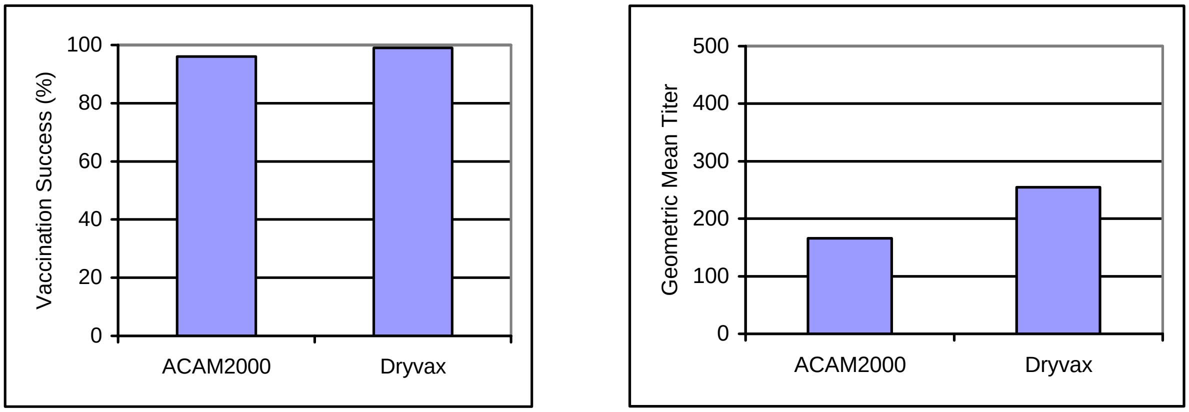

Panel A: Vaccination Success **Panel B: Neutralizing Antibody Titer**

ACAM2000 was found to be non-inferior to Dryvax® with respect to vaccination success, as determined by cutaneous response rates, in the vaccinia-naïve population. Ninety-six percent (96%) of subjects in the ACAM2000 dose group and 99% of subjects in the Dryvax® group experienced a major cutaneous response after vaccination.

The GMT of the neutralizing antibody response was higher in the Dryvax® group (255) compared to the ACAM2000 group (166). Although only a 1.5-fold difference in GMT was observed between the two groups, ACAM2000 did not quite meet the statistical criterion for non-

inferiority relative to Dryvax®. The 97.5% CI (-0.307) was just below the lower bound required to establish non-inferiority (≥-0.301).

A comparison of distribution of ACAM2000 and Dryvax® neutralizing antibody titers for the study is illustrated in Figure 6. The reverse cumulative frequency distribution curves for the two vaccines tracked well together with comparable profiles. It is interesting to note that about 93% of these subjects had neutralizing antibody titers above 20, a value that at least in one incomplete clinical study suggested as protective [10]. If one assumes that the positive cutaneous response observed with ACAM2000 (96%) correlates with protective immunity, then it is also worth noting that all subjects who did not have a positive dermal response and were evaluated for neutralizing antibody levels had titers ≤20.

Figure 6: Reverse Cumulative Frequency Distribution of Neutralizing Antibody Titers on Day 30 (Study H-400-009)

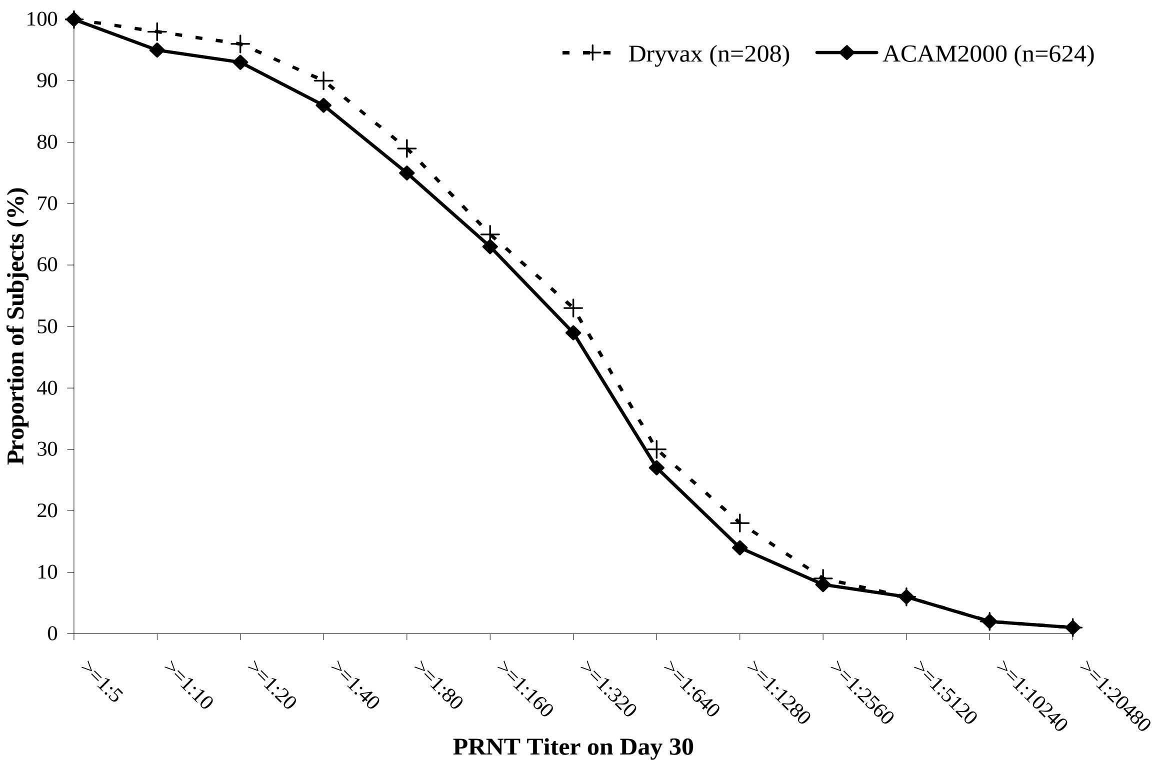

6.3.2. Phase 3 Study H-400-012: Previously Vaccinated Subjects

Study H-400-012 was a Phase 3, double-blind, randomized study designed to evaluate the safety and immunogenicity of ACAM2000 versus Dryvax®. Subjects were randomized to receive either ACAM2000 or Dryvax® at a 3:1 ratio. The original design called for enrollment of 2720 healthy male and female subjects, at least 31 years of age, who were previously vaccinated against smallpox. However, due to the early termination of the study, as described in Section 6.3.1, a total of 1819 subjects was enrolled at 70 study centers in the US and its territories of which 1577 subjects at 66 sites were evaluable for efficacy.

Co-primary efficacy measures were the proportion of subjects with a successful cutaneous reaction on Day 7 (±1), and neutralizing antibody GMT on Day 30. Vaccination success (based on cutaneous response) was determined by an IRC's assessment of digital photographs.

The treatment groups were well-balanced with respect to demographic variables and baseline characteristics, with the exception of baseline neutralizing titer. The baseline GMT was significantly higher in the ACAM2000 group than in the Dryvax® group (34 versus 28, respectively; p = 0.036). A summary of demographic information for all patients enrolled in ACAM2000 studies, including Phase 3, is provided as an appendix in Section 11.2. Table 10 summarizes the efficacy results in previously vaccinated subjects for ACAM2000 versus Dryvax®. These data are plotted in Figure 7.

Table 10: Phase 3 Efficacy Results for ACAM2000 versus Dryvax® in Previously Vaccinated Subjects (Study H-400-012)

Treatment group	ACAM2000	Dryvax®
**Revaccination Success based on Cutaneous Response**		
Evaluable Subjects (Dermal Evaluable Population)	1189	388
Revaccination Success, n (%)	998 (84)	381 (98)
97.5% CI [a] (Criterion for Non-Inferiority)	-17.00 (>-10)	
**Neutralizing Antibody Titer**		
Evaluable Subjects (Antibody Evaluable Population)	734	376
GMT	286	445
97.5% CI [b] (Criterion for Non-Inferiority)	-0.275 (>-0.301)	

a. Lower bound of the 97.5% one-sided CI on the difference between ACAM2000 and Dryvax® derived by normal approximation to the binomial distribution.
b. Lower bound of the 97.5% one-sided CI on the difference in the mean log_{10} GMT between ACAM2000 and Dryvax® derived using ANOVA.

Figure 7: Graphical Presentation of Phase 3 Efficacy Results in Previously Vaccinated Subjects (Study H-400-012)

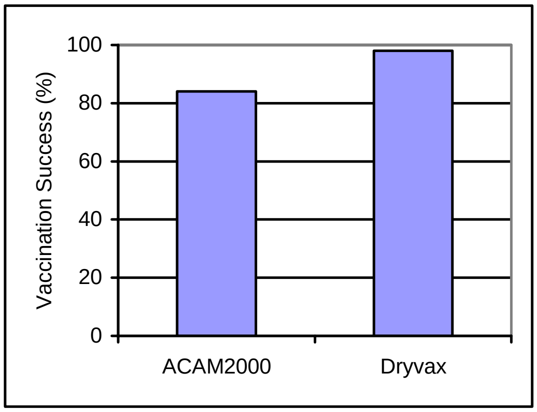

Panel A: Vaccination Success

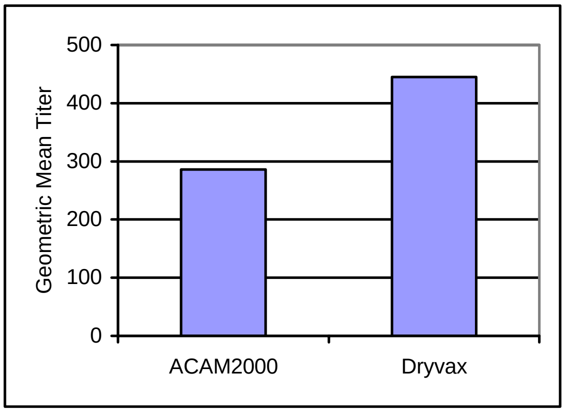

Panel B: Neutralizing Antibody Titer

While the majority of subjects in the ACAM2000 evaluable population developed a cutaneous response after revaccination (84%), the response rate was lower than in the Dryvax® group (98%). Statistically, ACAM2000 failed to meet the criterion for non-inferiority to Dryvax® with regard to revaccination success rates based on cutaneous response, as indicated by a lower bound of the one-sided 97.5% CI on the ACAM2000 – Dryvax difference that did not exceed -10% (Table 10). The observation that the dermal response with ACAM2000 was somewhat suppressed in previously vaccinated subjects was not unexpected based on Phase 2 data and is consistent with experience with other smallpox vaccines where the cutaneous reaction is

affected by the level of neutralizing antibody in the vaccinee [29, 36] (see discussion of baseline immunity, below).

With regard to neutralizing antibody GMT on Day 30, ACAM2000 was statistically non-inferior to Dryvax® in this study, as indicated by a lower bound of the 97.5% one-sided CI that exceeded -0.301 (Table 10). Consistent with the findings in the vaccinia-naïve subjects, the neutralizing antibody response to ACAM2000 was approximately 1.5-fold lower than to Dryvax®.

The reverse cumulative frequency distribution for ACAM2000 and Dryvax® neutralizing antibody titers demonstrate that these vaccines have similar response slopes, although there are generally higher titers in the Dryvax® treated group (Figure 8). However, it is important to emphasize that with both vaccines greater than 97% of the subjects had neutralizing antibody titers ≥20.

Figure 8: Reverse Cumulative Frequency Distribution of Neutralizing Antibody Titers on Day 30 (Study H-400-012)

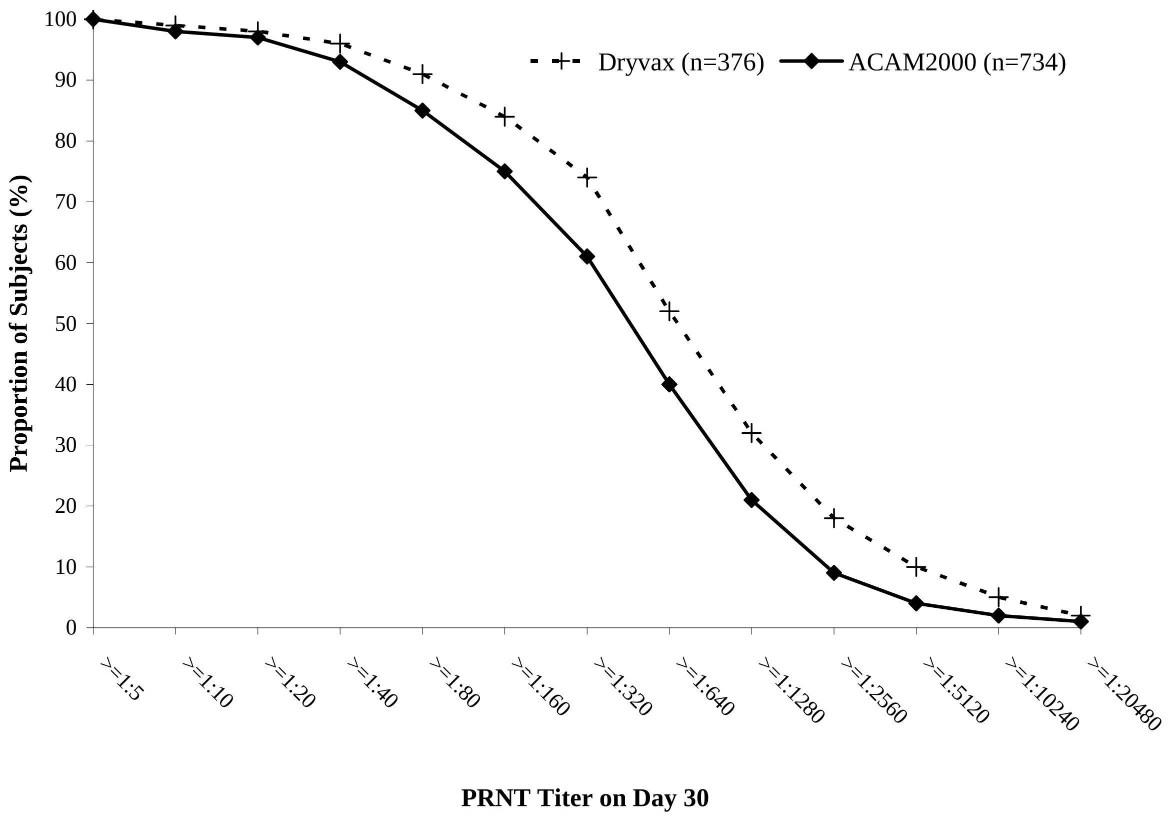

Influence of Baseline Immunity

It has been shown that pre-existing immunity can modulate the response to revaccination [36]. Therefore, a prospectively planned analysis of the relative influence of baseline immunity on the response to revaccination with ACAM2000 or Dryvax® was carried out using a covariate analysis of subjects who were in the evaluable populations. For ACAM2000, cutaneous response rates were found to be inversely proportional to baseline immunity, ranging from a high of 94% in subjects who were seronegative at baseline, to a low of 73% in subjects with

baseline titers of 160 or more (Table 11). Baseline immunity, however, had little effect on the cutaneous response to Dryvax®. It is possible that the restriction of cutaneous response by preexisting immunity is greater in the case of a slightly more attenuated virus (ACAM2000).

The magnitude of the antibody response to revaccination was also dependent on baseline immunity (Table 11). In both treatment groups, the mean fold-increase in neutralizing antibody titer was found to be inversely proportional to the titer of neutralizing antibodies at baseline. When mean fold-increases in neutralizing antibody titer were adjusted for baseline titer, a significant difference was found between the treatment groups (p<0.0001), with a higher fold-increase in the Dryvax® group than in the ACAM2000 group in each baseline titer category. Thus, the influence of baseline immunity was greater on ACAM2000 than on Dryvax®. This could again be due to a slight attenuation of the ACAM2000 virus, which would result in a more modest immune response as compared to Dryvax®.

Table 11: Phase 3 Efficacy Results for ACAM2000 and Dryvax®, Adjusted for Baseline Neutralizing Antibody Titer (Study H-400-012)

Baseline Titer	Revaccination Success n/N, (%)		GMT Mean Fold-Increase from Baseline to Day 30	
	ACAM2000 (n = 706)	Dryvax® (n = 364)	ACAM2000 (n = 734)	Dryvax® (n = 376)
< 10	151/161 (94)	86/88 (98)	29.6	36.4
10-20	158/186 (85)	107/108 (99)	13.3	23.8
40-80	168/199 (84)	93/94 (99)	5.9	10.9
≥160	116/160 (73)	72/74 (97)	2.2	4.8
All subjects ≥10	442/545 (81)	272/276 (99)	5.8	12.0
Overall total	593/706 (84)	358/364 (98)	8.5	15.7
p-value	<0.0001 [a]		<0.0001 [b]	

a. Overall test of difference between ACAM2000 and Dryvax®.
b. p-value on difference between ACAM2000 and Dryvax® derived by Analysis of Covariance, adjusting for baseline neutralizing antibody titer.

6.4. Integrated Efficacy Analysis

This section presents a summary of the efficacy results across the entire clinical program with an interpretation of those results.

6.4.1. Summary of Efficacy Results

The clinical trial results for the principal efficacy endpoints (cutaneous response and neutralizing antibody GMTs) are summarized in Table 12 for vaccinia-naïve subjects and in Table 13 for previously vaccinated subjects. The data are presented by study phase, study number and treatment group.

From a clinical perspective, ACAM2000 elicited a strong immune response in all study populations. Across all studies, ACAM2000 induced positive cutaneous responses in >96% of vaccinia-naïve subjects and in >84% of previously vaccinated subjects. In addition, ACAM2000

induced a robust neutralizing antibody response across all studies, with GMTs >100 in vaccinia-naïve subjects and >250 in the previously vaccinated population. ACAM2000 also induced a positive cell-mediated immune response as determined by at least one assay method in 100% of subjects tested for this response (n=30, see Table 6).

There was no significant difference between the ACAM2000 and Dryvax® groups in respect to neutralizing antibody titer in the Phase I and Phase 2 clinical studies of vaccinia-naïve subjects. Indeed, the Phase 2 comparative study of these vaccines (Study H-400-005) showed nearly identical GMTs (154 and 158) for the two vaccine groups. In an earlier open label, Phase 1 study (H-400-008) with ACAM2000 the GMP was 225 (n=100), however, in the Phase 3 trial (Study H-400-009) the GMT was 166 (n=776). In part these differences reflect the inconsistency of vaccination techniques, the diversity of individual responses, the variability of serological assays, and explain why immune responses are often reported in log units.

Plots for the reverse cumulative frequency distribution of neutralizing antibody titers in the Phase 3 studies for vaccinia-naïve and previously vaccinated subjects are provided in Figure 6 and Figure 8, respectively. These graphs illustrate similar profiles for both vaccines, with Dryvax® having slightly higher antibody titers. Although the neutralizing antibody titers of the ACAM2000 group (GMT=166) in the vaccinia-naïve study (H-400-009) was within approximately 1.5 fold of the Dryvax® group (GMT=255), it narrowly missed the primary endpoint for non-inferiority (CI = -0.307 vs. >-0.301) in the Phase 3 study.

In regards to the previously vaccinated subjects, the Phase 2 study (H-400-003) indicates that the titers of the ACAM2000 and Dryvax® were not statistically equivalent, although, in the Phase 3 study (Study H-400-012) the primary endpoint for non-inferiority, based on GMT, was met (Table 13). The important point is that in all studies the previously vaccinated groups had higher GMTs than the primary vaccination (vaccinia-naïve) groups. Therefore, if protection is provided to the primary vaccination group it follows that the previously vaccinated group would also be protected (based on antibody titer).

With respect to the dermal response, no statistical difference was observed between ACAM2000 and Dryvax® groups in the Phase I and Phase 2 studies (H-400-002 & H-400-005) for primary vaccination, where there was a 100% positive dermal response in all groups. In the Phase 3 study of the vaccinia-naïve (Study H-400-009) there was a 96% positive dermal response with ACAM2000 and the primary endpoint for non-inferiority was met. This is a very significant outcome since it is recognized as the primary correlate for immunity with smallpox vaccines [29].

The greatest difference between the two vaccines was observed in the cutaneous response rates in previously vaccinated subjects. Eighty-four percent (84%) of subjects in the ACAM2000 group versus 98% of subjects in the Dryvax® group were scored for a positive cutaneous response. In this population, ACAM2000 did not meet the Phase 3 statistical endpoint of non-inferiority, relative to Dryvax®, for dermal response. However, not meeting this endpoint is not considered negative from an efficacy standpoint since those with low neutralizing antibody titers (<10) prior to vaccination had 94% positive cutaneous responses and overall the neutralizing antibody levels were higher than in the primary vaccination group (see Section 6.4.2 and Section 6.5).

Table 12: Immunogenicity and Efficacy Results for Vaccinia-Naïve Subjects

Study Phase	Phase 1			Phase 2		Phase 3	
Study No.	H-400-002		H-400-008	H-400-005		H-400-009	
Treatment group (PFU/Dose)	ACAM 2000 ($1.0x10^8$)	Dryvax® ($1.6x10^8$)	ACAM 2000 ($7.7x10^7$)	ACAM 2000 ($6.8x10^7$)	Dryvax® ($1.6x10^8$)	ACAM 2000 (1.3-2.2 $x10^8$)	Dryvax® ($1.6x10^8$)
**Vaccination Success based on Cutaneous Response**							
Evaluable Subjects (n)	30	30	100	51	49	776	257
Vaccination success, n (%)	30 (100)	30 (100)	99 (99)	51 (100)	49 (100)	747 (96)	255 (99)
Statistical Interpretation	No difference between groups		NA	No difference between groups		ACAM2000 was non-inferior to Dryvax®	
**Neutralizing Antibody Titer [a]**							
Evaluable Subjects (n)	30	30	100	51	49	565	190
Geometric Mean Titer	103	172	225	154	158	166	255
Statistical Interpretation	No significant difference between groups		NA	No significant difference between groups		ACAM2000 did not meet the endpoint of non-inferiority to Dryvax®	

Key: NA = Not applicable
a. $PRNT_{50}$ titer was determined on Day 45 in Study H-400-002 and on Day 30 in all other studies.

Table 13: Immunogenicity and Efficacy Results for Previously Vaccinated Subjects

**Study Phase**	**Phase 2**		**Phase 3**	
**Study No.**	**H-400-003**		**H-400-012**	
**Treatment group (PFU/Dose)**	**ACAM2000 (6.8 x 10^7)**	**Dryvax® (1.6 x 10^8)**	**ACAM2000 (1.3-2.2 x 10^8)**	**Dryvax® (1.6 x 10^8)**
**Vaccination Success based on Cutaneous Response**				
Evaluable Subjects	n = 49	n = 52	n = 1189	n = 388
Vaccination success, n (%)	43 (88)	52 (100)	998 (84)	381 (98)
Statistical Interpretation	ACAM2000 not equivalent to Dryvax®		ACAM2000 did not meet the endpoint of non-inferiority to Dryvax®	
**Neutralizing Antibody Titer**				
Evaluable Subjects	n = 50	n = 52	n = 734	n = 376
Geometric Mean Titer	256	447	286	445
Statistical Interpretation	Groups not equivalent		ACAM2000 was non-inferior to Dryvax®	

6.4.2. Effect of Baseline Immunity on Efficacy Outcome Measures

Clinical experience with other smallpox vaccines has shown that previously vaccinated subjects with baseline neutralizing antibody titers ≥10 are less likely to exhibit a primary-type cutaneous response upon revaccination than are subjects with undetectable levels of neutralizing antibody at the time of revaccination [29]. Consistent with these historical observations, cutaneous and neutralizing antibody responses to ACAM2000 in previously vaccinated subjects were strongly dependent on the level of pre-existing immunity to vaccinia in each subject. Both the cutaneous response rate and the mean fold change in neutralizing antibody titer from Baseline to Day 30 were inversely proportional to the titer at Baseline (Table 11). Revaccination success based on cutaneous response with Dryvax® was comparatively unaffected by baseline titer, which contributed to the overall differences observed between the two treatment groups. A recent study in Israel demonstrated that a lack of response to revaccination with Lister vaccine correlated with the interval since last vaccination [37]. In that study, 77.5% of subjects vaccinated <20 years previously responded to revaccination versus 97.2% for persons vaccinated >20 years before. Unlike ACAM2000 or the Israeli Lister vaccine, Dryvax® is able to elicit a more active cutaneous response that is independent of residual immunity [38]. Nevertheless, in previously vaccinated subjects where immunity has waned so that smallpox neutralizing antibody titers are below 10 or in vaccinia-naïve subjects, ACAM2000 elicited a positive cutaneous response in greater than 94% of the subjects, similar to Dryvax® (Table 11).

6.4.3. ACAM2000 Clinical Consistency and Dosing Recommendations

Three lots of ACAM2000, with potencies of 1.3×10^8, 1.9×10^8, and 2.2×10^8 PFU/mL, were released according to the proposed potency specification ($1.0\text{-}5.0\times10^8$ PFU/mL) and utilized in the Phase 3 studies. Given that all three ACAM2000 lots utilized in the Phase 3 studies produced a cutaneous response in ≥94% of vaccinia-naïve subjects and in previously vaccinated subjects who lacked baseline antibody, and given that data from Phase 2 study H-400-005 demonstrated that a lot with 4-fold lower potency (6.8×10^7 PFU/mL) than the lower limit of the release specification elicited responses comparable to Dryvax®, the sponsor has concluded that ACAM2000 released according to the potency specification of $1.0\text{-}5.0\times10^8$ PFU/mL is effective in eliciting a protective response (cutaneous and serological) against smallpox infection in the majority of vaccinia-naïve and previously vaccinated individuals. This potency specification is also consistent with the requirements outlined in the original RFP issued by the CDC in 2001.

6.5. Efficacy Conclusions

Based on historical evidence, vaccinated individuals are considered protected against smallpox after a major cutaneous reaction is observed following primary vaccination [29]. Further, two prospective studies have correlated higher titers of neutralizing antibodies with lower susceptibility to smallpox upon contact with a smallpox victim [10, 5]. Although the methods used to measure neutralizing antibody titers differ between these published studies and Acambis' clinical trials, the limited data suggest that neutralizing antibody titer serves as a qualitative, if not a quantitative, correlate of protection with titers greater than 32 proposed as nominally protective [5].

Both dermal reactivity and neutralizing antibodies to smallpox infection were used as co-primary endpoints in two Phase 3 trials designed to show the non-inferiority of ACAM2000 to Dryvax®. In these Phase 3 studies the non-inferiority endpoint for cutaneous response was met and the neutralizing antibody titer was narrowly missed in the vaccinia-naïve population whereas in the previously vaccinated population the non-inferiority endpoint for neutralizing antibody titer was met and the cutaneous response was not met, consistent with ACAM2000 being a purified vaccine that is slightly more attenuated than Dryvax®.

Although the Phase 3 clinical endpoints for non-inferiority were not all met, these results when taken together with historical information, earlier clinical and non-clinical studies, and further analysis of individual baseline immunity provide a preponderance of data supporting the efficacy of this vaccine. The following points further support this conclusion:

1. The ACIP guidance for protective efficacy against smallpox states: "Clinically, persons are considered fully protected after a successful response is demonstrated at the site of vaccination" [29 Primary vaccination efficacy, based on this criterion, was demonstrated to be greater than 96% with ACAM2000 and was determined to be statistically non-inferior to Dryvax®.
2. Neutralizing antibody titers greater than a putative value of 32 have been suggested as a correlate to immunity [5, 10]. Although this value cannot be validated due to the elimination of the disease, it is noted that >93% of subjects in the primary vaccination group had titers greater than 20. In the same group 96% were evaluated to be protected

based on a positive cutaneous response. Of those subjects who did not have a positive dermal response and were evaluated serologically (n=20), all had neutralizing antibody titers ≤20.

3. The Phase 3 study resulted in a neutralizing antibody GMTs of 166 and 286 for vaccinia-naïve subjects and previously vaccinated subjects, respectively. Therefore, if the primary vaccination group is considered protected (based on cutaneous response), so also should the previously vaccinated group with ever higher titers (GMT=286), assuming similar distribution curves for the antibody titer (Figure 6 and Figure 8). Even though ACAM2000 had a subdued dermal response with previously vaccinated, one may conclude that the neutralizing antibody titer for this group provides a reasonable indication of immunity.
4. In the previously vaccinated group, those at highest risk for infection (lowest neutralizing antibody titer) also exhibited a dermal response as defined by the ACIP. Of the 161 previously vaccinated subjects who had waning immunity, (titers <10), 151 (94%) of them had a positive dermal response with ACAM2000.
5. Plots of reverse cumulative frequency distribution of neutralizing antibody titers show similar profiles for both vaccines with Dryvax® having a slightly higher antibody level (Figure 6 and Figure 8). Since differences in immune responses are often measured in logs, these distribution curves are illustrative of product similarity. In the case of the previously vaccinated subjects, the vaccines directly overlapped in the frequency distribution curves for those with low neutralizing antibody titers (Figure 8).
6. Phase 1 clinical studies demonstrated that all 30 subjects (100%) in the ACAM2000 group, and 28 (93%) of 30 subjects in the Dryvax® group had a positive cell-mediated immune response to smallpox vaccine in at least one of the three T-cell conducted
7. Because of the serious nature of this disease and the inability to perform traditional efficacy studies it is important to consider supportive animal challenge studies as part of the efficacy assessment. The ability to provide complete protection of primates at lethal challenge doses of monkeypox virus (Orthopoxvirus genus) with no signs of disease, as described in Section 4, provides critical support for ACAM2000 effectiveness.

Since ACAM2000 is a purified vaccine derived from Dryvax® through 10 cell culture passages, it provides a slightly more attenuated vaccine and therefore a milder cutaneous reaction than does Dryvax®. Nevertheless, ACAM2000 elicited a robust immune response with neutralizing antibody GMTs >100 in vaccinia naïve subjects and >250 in previously vaccinated subjects across all clinical studies.

In conclusion, the immunogenicity of ACAM2000 and its efficacy compared to Dryvax® are adequate to support ACAM2000 vaccination against smallpox disease for those at risk of infection.

7. CLINICAL SAFETY

7.1. Safety Population

The Safety Population consists of all subjects who received vaccination during the clinical trials. The safety profile of ACAM2000 was evaluated in a total of 2983 subjects (1307 vaccinia-naïve and 1676 previously vaccinated) in doses ranging from 3.4×10^6 to 2.2×10^8 PFU/mL. A total of 868 subjects (368 vaccinia-naïve and 500 previously vaccinated) received single cutaneous doses of Dryvax® vaccine administered at doses ranging from 1.0×10^8 to 1.6×10^8 PFU/mL.

Comparison of the safety profile of ACAM2000 obtained from the Phase 3 studies with the safety profile of ACAM2000 when the safety data from all clinical studies were pooled indicated that the safety profile of ACAM2000 in the Phase 3 studies was similar to that seen in all studies overall. Therefore this summary will focus on Phase 3 results for the most common AEs, but the discussion regarding SAEs will include data from all clinical studies.

7.2. Serious Adverse Experiences (SAEs) and Deaths

There were no fatalities during any clinical study of ACAM2000. Other SAEs occurred rarely (<1%) with ACAM2000, with the most commonly reported SAEs being myocarditis and pregnancy (considered an SAE due to the potential risk of congenital infection, although no cases of congenital infection were documented). Not all reported events of myocarditis or pregnancy met the International Conference on Harmonization (ICH) definition of an SAE. However, Acambis treated all such events as SAEs as a conservative safety measure.

7.2.1. Myocarditis/pericarditis

Ten cases, seven in subjects treated with ACAM2000 (5.73 events per thousand vaccinations) and three in subjects treated with Dryvax® (10.38 events per thousand vaccinations), were reported in a total vaccinia-naïve population of 1675 subjects, for a combined calculated incidence of 5.97 cases per thousand vaccinations.

Recently, it was reported that myocarditis and/or pericarditis (myocarditis/pericarditis) is associated with administration of the Dryvax® vaccine with incidence rates of 0.11 cases per thousand vaccinations (cumulative for both vaccinia-naïve and previously vaccinated subjects) [24]. Reports of myocarditis/pericarditisiari in the US civilian and military smallpox vaccination programs using Dryvax® were issued in 2003 [30]. In addition, evidence of subclinical myocarditis/pericarditis following smallpox vaccination from the literature was highlighted at that time [30, 4]. Thus in 2003, when the ACAM2000 Phase 2 studies were being conducted, the structured interview for all ongoing and planned ACAM2000 clinical studies was revised to included symptoms of myocarditis/pericarditis [30]. Subjects with such symptoms were to have additional evaluations performed according to a cardiac algorithm to determine whether myocarditis/pericarditis had occurred . These studies excluded subjects with cardiac disease and specific risk factors for ischemic cardiac disease. (See 11.4, Appendix 4 – Cardiac Algorithm)

Scheduled serial ECGs and cardiac troponin I evaluations (capable of detecting subclinical myocarditis) were incorporated for all subjects in the Phase 3 studies and the H-400-002 Phase

1 study, to complement the structured interview. These studies also excluded subjects with cardiac disease and specific risk factors for ischemic cardiac disease.

The incidence rate for myocarditis in Acambis' Phase 3 clinical studies was higher than previously reported in Department of Defense (DoD) and civilian vaccination programs (0.11 [1] and 0.54 [25] cases per thousand vaccinations, respectively), which used only passive surveillance measures and did not prospectively test for sub-clinical cases.

Subjects with suspect or probable myocarditis as confirmed by the CAP or the Sponsor, are presented as an appendix in Section 11.3. Both local and central read ECGs and clinical laboratory findings obtained at the time of onset of the event (or at the first time-point after the event onset) are presented.

A total of 10 cases of suspect or probable myocarditis, 7 (5.73 events per thousand vaccinations) in subjects treated with ACAM2000 and 3 (10.38 events per thousand vaccinations) in subjects treated with Dryvax®, were identified in the ACAM2000 clinical program (Phase 1 through 3). No ACAM2000 subjects were diagnosed with confirmed myocarditis or pericarditis (requiring histological evidence of myocardial or pericardial inflammation). All subjects who experienced myocarditis were previously naïve to vaccinia; no cases were detected in previously vaccinated subjects. Of these 10 subjects, 9 were male and 7 were Caucasian. The mean age of subjects was 22 years. No subject had a known history of cardiac disease. However, 2 subjects had at least 1 risk factor for ischemic coronary disease. The mean time to onset of myocarditis from vaccination was 11 days, with a range of 9 to 20 days. It is noted however that the exact time to onset of these events is unknown, because 8 of 10 cases were not characterized by acute clinical signs and were identified by routine study evaluation at the Day 10 study center visit, the first post-vaccination time-point at which cardiovascular evaluations were conducted.

Although the myocarditis incidence rate was higher in the Dryvax® group (10.38 events per thousand vaccinations) there was no statistically significant difference from the ACAM2000 group (5.73 events per thousand vaccinations). Of the ten subjects, four (three in the ACAM2000 group and one in the Dryvax® group) were symptomatic, of whom two (one each in the ACAM2000 and Dryvax® groups) were hospitalized with acute cardiac symptoms. In the 2 hospitalized subjects, these cardiovascular events were considered resolved with sequelae for the ACAM2000 subject and resolved without sequelae for the Dryvax® subject. At discharge the ECG for the ACAM2000 subject was still abnormal with nonspecific ST-T wave changes and he was put on Coreg 6.25 mg and aspirin 81 mg.

The remaining 8 subjects with suspect or probable myocarditis were neither hospitalized nor treated with medications. Suspect or probable myocarditis was considered resolved without sequelae at the last follow-up for all but one Dryvax® recipient, the sole female subject, who remains with persistent echocardiogram evidence of diminished ejection fraction (27-32%) and global hypokinesis. The subject has been followed by her cardiologist for 2.5 years after vaccination and remains asymptomatic with normal cardiac examination findings, and continuing abnormal echocardiogram findings (see Table 18).

7.2.2. Other Serious Cardiac Events

Serious cardiac events other than myocarditis were reported within 30 days after vaccination for 4 previously vaccinated subjects, and included single reports of atrial fibrillation, chest discomfort, chest pain, and coronary artery disease. All 3 events, except coronary artery disease, were considered to have a possible relationship to ACAM2000 as determined by the clinical investigator, and are described below. The event of coronary artery disease was considered by the clinical investigatory to have no relationship to the study vaccine (Dryvax®).

The subject hospitalized with atrial fibrillation 31 days after vaccination converted to normal sinus rhythm while in the hospital and the event was considered resolved. The subject had a normal ejection fraction on echocardiogram, normal troponin and slightly elevated CK.

One subject was hospitalized with chest pressure beginning 3 days after vaccination. Follow-up testing was normal and the event was considered resolved 4 days after onset.

One subject was hospitalized with severe chest pain 35 days after vaccination. The subject's Day 12 ECG was abnormal; however the specific abnormality was not reported. Follow-up testing revealed normal ejection fraction on echocardiogram, normal chest x-ray and normal cardiac enzymes. The subject was discharged with nitroglycerin, beta blockers, aspirin and diet change.

7.2.3. Pregnancy

As reported in the literature [39], congenital infection, a very rare occurrence, principally occurring during the first trimester, may be associated with generalized vaccinia of the fetus (termed fetal vaccinia), resulting in early delivery of a stillborn infant or a high risk of perinatal death. Due to this potential risk, women of child-bearing potential who were pregnant or lactating were excluded from participation in any clinical study of ACAM2000. Reports of several pregnancies within 30 days of vaccination during the Phase 2 studies resulted in a two week interruption of enrollment while the protocols were amended to require more stringent birth control methods and pregnancy testing. In addition, a comprehensive plan to reduce the risk of inadvertent vaccination of pregnant women was developed and implemented, including more comprehensive screening procedures and enhanced subject education materials. These procedures also were incorporated in the Phase 3 studies.

In the ACAM2000 clinical program, a total of five subjects who were determined to be non-pregnant prior to vaccination subsequently were determined to be pregnant within 30 days after vaccination. The five pregnancies after vaccination resulted in two spontaneous abortions, one live birth, and one elective termination; the remaining subject was lost to follow-up. There were no reports of fetal vaccinia, congenital anomalies, or birth defects. Of the two spontaneous abortions, one was considered to be unrelated to study vaccine. In the other case, neither smallpox vaccination nor VIG administration could be ruled out as risk factors.

7.2.4. Other Serious Adverse Events

One vaccinia-naïve subject treated with ACAM2000 experienced a new-onset seizure 8 days after vaccination. Follow-up physical and neurological examination findings and imaging studies were normal, and the subject did not experience any subsequent seizures during the study period. Other predisposing factors (sleep deprivation, photic stimulation, paternal history of

seizures) were considered contributory to new-onset seizures in this subject, in addition to vaccination with ACAM2000. This event in this subject was not considered to be a symptom of a significant CNS disorder historically associated with smallpox vaccine (e.g., aseptic meningitis, post-vaccinal encephalitis, or myelitis). A neurologic algorithm was subsequently established in the Phase 3 studies in order to monitor closely for neurologic events associated with smallpox vaccination. No additional subject experienced convulsions of any etiology in any clinical study of ACAM2000 or any other significant CNS event historically associated with smallpox vaccine. This is consistent with the experience from the government smallpox vaccination programs [40].

Two cases of appendicitis were reported with ACAM2000, one each in a vaccinia-naïve and previously vaccinated subject. Both cases were considered unrelated to ACAM2000. One previously vaccinated subject treated with ACAM2000 was determined to be human immunodeficiency virus (HIV)-positive one day after vaccination. (Samples for screening virology tests had been collected ten days before vaccination; however, the test results were not available at the time of vaccination.) This subject received prophylactic treatment with VIG. No notable complications were reported through last follow-up approximately three months post-vaccination.

7.2.5. Other Relevant Safety Information

Urticaria was rarely (<1%) reported with ACAM2000; and no cases were considered to be serious. No cases of urticaria had an acute temporal relationship with ACAM2000 administration. No cases of anaphylaxis, erythema multiforme major/Stevens-Johnson syndrome, or other significant allergic responses were reported with ACAM2000. Urticarial rash and hypersensitivity were noted after Dryvax® in the Phase 3 studies.

7.3. Common Adverse Experiences

ACAM2000 was well tolerated by both vaccinia-naïve and previously vaccinated subjects. ACAM2000 and Dryvax® were associated with a high rate of AEs, with the majority (99%) of subjects, regardless of baseline vaccination status and vaccine dose, experiencing at least one treatment-emergent AE after vaccination.

As expected with a smallpox vaccine, the most commonly reported AEs among subjects generally were local signs or symptoms related to the cutaneous reaction or associated systemic symptoms. The incidence of these events was higher among subjects who experienced a successful vaccination based on dermal response than among those who did not, regardless of dose or baseline vaccination status. Solicited, as well as unsolicited events were collected, although the most common events came from a solicited checklist.

AEs commonly reported after vaccination with ACAM2000 generally fell into four distinct categories: reactions at the vaccination site, lymphadenitis, constitutional "flu-like" symptoms, and minor gastrointestinal symptoms. These commonly reported AEs generally occurred at a higher incidence in vaccinia-naïve subjects than in previously vaccinated subjects receiving revaccination. This finding is not unexpected, given that the clinical course of the response to revaccination is typically milder than that to primary vaccination. Furthermore, the incidence of these events generally was lower, in many cases statistically significantly lower, with ACAM2000 than with Dryvax®, regardless of baseline vaccination status, suggesting that the

former is more attenuated. However, the clinical relevance of this difference is limited, considering the relatively benign nature of these events.

AEs reported by ≥10% of subjects in either the ACAM2000 or Dryvax® group in the Phase 3 studies are summarized in Table 14 for the safety population by baseline vaccination status and treatment group.

Table 14: Commonly Reported (≥10% in the ACAM2000 or Dryvax® Groups) AEs by MedDRA Preferred Term, Baseline Vaccination Status and Treatment Group, Phase 3 Studies, Total Population

	Vaccinia-Naïve Subjects			Previously Vaccinated Subjects		
**MedDRA Preferred Term [a]**	**ACAM 2000 (n=873) n (%)**	**Dryvax® (n=289) n (%)**	**p-value [b]**	**ACAM 2000 (n=1371) n (%)**	**Dryvax® (n=448) n (%)**	**p-value [b]**
At least 1 AE	864 (99)	288 (100)	0.4661	1325 (97)	443 (99)	0.0124
Injection site pruritus*	804 (92)	277 (96)	0.0324	1130 (82)	416 (93)	<.0001
Injection site erythema*	649 (74)	229 (79)	0.0976	841 (61)	324 (72)	<.0001
Injection site pain*	582 (67)	208 (72)	0.0950	505 (37)	209 (47)	0.0003
Lymph node pain*	494 (57)	199 (69)	0.0002	261 (19)	119 (27)	0.0008
Headache*	433 (50)	150 (52)	0.4984	437 (32)	166 (37)	0.0493
Fatigue*	423 (48)	161 (56)	0.0354	468 (34)	184 (41)	0.0090
Injection site swelling*	422 (48)	165 (57)	0.0100	384 (28)	188 (42)	<.0001
Myalgia*	404 (46)	147 (51)	0.1966	374 (27)	148 (33)	0.0222
Malaise*	327 (37)	122 (42)	0.1634	381 (28)	147 (33)	0.0478
Feeling hot*	276 (32)	97 (34)	0.5611	271 (20)	114 (25)	0.0114
Erythema*	190 (22)	69 (24)	0.4636	329 (24)	107 (24)	1.000
Rigors*	185 (21)	66 (23)	0.5643	171 (12)	76 (17)	0.0173
Nausea*	170 (19)	65 (22)	0.2728	142 (10)	63 (14)	0.0386
Diarrhea*	144 (16)	34 (12)	0.0593	158 (12)	77 (17)	0.0026
Exercise tolerance decreased*	98 (11)	35 (12)	0.6709	105 (8)	50 (11)	0.0248
Rash*	94 (11)	30 (10)	0.9128	80 (6)	29 (6)	0.6466
Lymphadenopathy*	72 (8)	35 (12)	0.0598	78 (6)	29 (6)	0.5632

* Event was listed on a checklist included in subject diaries and is therefore considered prompted.

a. Medical Dictionary for Regulatory Activities (MedDRA) Preferred Terms are listed in descending order of occurrence in the ACAM2000 Naïve Subject Group.

b. Fisher's Exact test.

AEs commonly reported with ACAM2000 generally were assessed by the Investigator as mild or moderate in intensity and study vaccine-related. Furthermore, the great majority of commonly reported AEs occurred within the first week (Days 0 to 6) after vaccination, with a general decline in the incidence of AEs thereafter.

A dose-response relationship was not seen with regard to the incidence of AEs overall across the range of doses evaluated in the Phase 3 program, 1.3×10^8 to 2.2×10^8 PFU/mL, in either vaccinia-naïve or previously vaccinated subjects.

7.3.1. Vaccination Site Reactions

After vaccination with ACAM2000, the majority (92%) of subjects experienced at least 1 vaccination site reaction, with the most common reactions being pruritus, erythema, pain, and swelling at the vaccination site; the incidence of all of these events was notably higher in vaccinia-naïve subjects than in previously vaccinated subjects. Less common vaccination site reactions with ACAM2000 included inflammation, burning, and vesicles at the vaccination site or another vaccination complication (2% each). All other vaccination site reactions reported with ACAM2000, including robust takes, satellite lesions, and autoinoculation/distal lesions, were uncommon (<1%).

A relatively small proportion (5%) of ACAM2000-treated subjects experienced a severe vaccination site reaction, with a higher incidence of such events in vaccinia-naïve subjects (9%) than in previously vaccinated subjects (2%). Approximately three-fourths (73%) of these subjects with a severe vaccination site reactions experienced severe injection site erythema, which was conservatively defined as an area of redness ≥1.5 inches (3.8 cm) in diameter. No significant vaccination site reactions or complications historically associated with smallpox vaccination, including GV, PV, erythema multiforme or EV, were reported with ACAM2000 in either vaccinia-naïve or previously vaccinated subjects. One case of GV was reported for a Dryvax®-treated subject.

7.3.2. Constitutional Symptoms

Commonly reported constitutional symptoms associated with the systemic response to smallpox vaccine included fatigue, headache, myalgia, malaise, feeling hot, and rigors. Constitutional "flu-like" symptoms were reported for approximately three-fourths (76%) of vaccinia-naïve subjects and 55% of previously vaccinated subjects treated with ACAM2000. All of these events occurred at a higher incidence ($p<0.05$) in the Dryvax® group than in the ACAM2000 group in both vaccinia-naïve and previously vaccinated subjects (Table 14).

Fever is an expected reaction to primary vaccination and revaccination with smallpox vaccine, although it has been reported to occur less commonly in adults than in children after both primary vaccination and revaccination [12]. Of note, body temperature increase and pyrexia were uncommonly (≤1%) reported as an AE with either the ACAM2000 or Dryvax® groups. Oral body temperature measurement ≥102°F (38.9°C) at any time-point were uncommon (<1%) in both vaccinia-naïve and previously vaccinated subjects in either group.

7.3.3. Lymphadenitis

Lymph node pain, a prompted AE, was commonly reported with ACAM2000, occurring at an overall incidence of 57% and 19% in vaccinia-naïve and previously vaccinated subjects,

respectively. Lymphadenopathy was seen less commonly than lymph node pain (8% and 6% of vaccinia-naïve and previously vaccinated subjects, respectively). Specific reports of lymphadenitis were uncommon (1 subject; <1%) with ACAM2000.

As would be expected, lymph node enlargement and tenderness occurred more commonly ipsilateral rather than contralateral to the vaccination site.

7.3.4. Gastrointestinal Symptoms

Commonly reported gastrointestinal symptoms included nausea and diarrhea, which were reported at an incidence of 19% and 16%, respectively in vaccinia-naïve subjects and 10% and 12%, respectively, in previously vaccinated subjects treated with ACAM2000. In contrast with other commonly reported AEs, the incidence of diarrhea was higher with ACAM2000 (16%) than with Dryvax® (12%) in vaccinia-naïve subjects. However, in previously vaccinated subjects, the incidence of this event was lower with ACAM2000 than with Dryvax®. The incidence of nausea in both subject populations was lower with ACAM2000 than Dryvax®. Most cases of nausea were mild or moderate in intensity and were unaccompanied by vomiting.

7.4. Virus Shedding

Because virus shedding can lead to AEs for both the recipient and the recipient's contacts, this parameter was evaluated in Phase 1 study H-400-002 at the vaccination site and outside the vaccination site bandage.

ACAM2000 was compared to ACAM1000 and Dryvax®. The pattern of virus shedding over time was similar in each treatment group, with virus detectable at the vaccination site in all 3 treatment groups by Day 3, the first post-vaccination time-point assessed. The proportion of subjects with evidence of virus shedding was notably higher by Day 7 and remained high through Day 15. By Day 30, a relatively small proportion of subjects in each treatment group (3%, 10%, and 13% of subjects in the ACAM2000, ACAM1000, or Dryvax® groups) had evidence of virus shedding at the vaccination site. The median duration of virus shedding at the vaccination site, as determined by Kaplan-Meier estimates, was 16, 18, and 20 days with ACAM2000, ACAM1000, and Dryvax®, respectively. Although the median duration of virus shedding was 4 days longer in the Dryvax® group compared to the ACAM2000 group, the p-value for the log rank comparison of the Kaplan-Meier curves for duration of virus shedding from the inoculation site missed the criterion for significance (p=0.0586).

Only 1 (3%) subject treated with ACAM2000 in Study H-400-002 had evidence of virus shedding outside the vaccination site dressing detected at any time-point.

7.5. Induction of Non-Specific Serologic Responses

In the Phase 1 Study H-400-002, serologic evaluations were performed before and after vaccination to determine whether subjects vaccinated with smallpox vaccine developed non-specific positive serological responses to unrelated viruses (HIV, hepatitis B and C viruses) and bacteria (syphilis). These tests were conducted at the request of the FDA based on historical data that indicated that other vaccines (e.g., influenza) occasionally cause non-specific false positive serological tests for HIV and that smallpox vaccine specifically caused a relatively high incidence of false positive tests for syphilis [41,45].

Serologic evaluations at Screening and on Days 15 and 45 included tests for HIV antibody (using an FDA approved HIV enzyme-linked immunoassay [EIA] test kit); hepatitis B surface antigen (HBsAg) and hepatitis B surface antibody (HBsAb) (assessed only at Screening unless either was positive); hepatitis C virus (HCV); and syphilis antibody (using the Rapid Plasma Reagin test with reflex to titer). Samples that tested positive for HIV were confirmed by Western Blot, positive tests for HBsAg were confirmed by neutralization, positive tests for HBsAb were confirmed by endpoint, positive tests for HCV were confirmed by Recombinant Immunoblot Assay if there were no risk factors, and by polymerase chain reaction quantitation if there were risk factors, and positive tests for syphilis were retested using Florescent Treponemal Antibody. All subjects' retest results were negative. The results indicate that smallpox vaccination using calf lymph or cell culture derived vaccines, can elicit acute (i.e. transient) biologic false positive tests for syphilis (BFP) in a relatively high proportion of subjects.

7.6. Safety in Special Populations

Subgroup analyses of safety data were performed for the following factors: sex, race, body surface area, age, baseline neutralizing antibody titer (previously vaccinated subjects only), vaccination success (as determined by cutaneous response), and seroconversion (≥4-fold rise in neutralizing antibodies between Days 0 and 30) status. As discussed in Section 7.3, the incidence of injection site reactions and constitutional symptoms was higher among subjects who experienced a successful vaccination and among subjects who seroconverted than among those who did not, regardless of dose or baseline vaccination status. No notable difference in AE rates was seen by race, sex, age, body surface area, or baseline neutralizing antibody titer.

7.7. Conclusions Regarding the Safety of ACAM2000

ACAM2000 was well tolerated and less reactogenicity than Dryvax®. These observations when taken in conjunction with the neurovirulence testing in animals (Table 3) suggest that ACAM200 is more attenuated than Dryvax®. Therefore, the increased safety potential with ACAM2000 is balanced by a more modest cutaneous response observed in previously vaccinated subjects and slightly lower GMTs overall.

The AEs associated with ACAM2000 were, in general, predictable and manageable, with the most commonly reported events being mild to moderate vaccination site reactions, lymphadenitis, and constitutional and gastrointestinal symptoms. The incidence of these commonly reported AEs was higher in vaccinia-naïve subjects than in those who were previously vaccinated. Furthermore, a higher incidence of these events was generally seen in subjects who experienced a successful vaccination based on dermal response compared to those who did not, regardless of baseline vaccination status.

Although uncommon, myocarditis was associated with smallpox vaccination in vaccinia-naïve subjects, with an incidence of 5.73 cases per thousand vaccinations of ACAM2000 in the Phase 3 study. This event has only been identified in more recent years with Dryvax® vaccinated subjects [24] but has not been evaluated with the detail these studies have provided. No other SAEs historically associated with smallpox vaccine, including GV, ocular vaccinia, postvaccinial encephalitis, PV, erythema multiforme, or EV, were reported with ACAM2000.

8. POST-LICENSURE SURVEILLANCE

In accordance with established practice, Acambis will work with the various government agencies (DoD and CDC) to ensure complete reporting of safety information following incorporation of ACAM2000 post-licensure into their vaccination programs. Based upon the current reporting scheme for Dryvax® smallpox vaccine, most spontaneously reported ACAM2000 AEs will be captured by the CDC and/or DoD smallpox vaccine programs, and reported directly to the FDA using Vaccine Adverse Event Reporting System (VAERS) forms. Summaries of DoD and CDC spontaneous reports for ACAM2000 will be downloaded from the VAERS database by Acambis for safety review.

ACAM2000 AEs reported directly to Acambis will be entered, tracked and reported on FDA VAERS form via a validated pharmacovigilance (PVG) database. ACAM2000 spontaneous events reported directly by the CDC or DoD smallpox vaccination programs will not be entered into the Acambis PVG database to avoid duplication of reporting. During the early post-marketing period (first two years after licensure), all known 15 day reports provided directly to Acambis will be furnished to the FDA on a monthly basis.

As ACAM2000 is a new product, Acambis proposes additional safety measures in coordination with governmental agencies, to include the following three modules:

- A prospective post-marketing study with appropriate power estimates (minimum 10,000 subjects) for the specific primary endpoint of myocarditis and secondary endpoints of superinfection, contact transmission, autoinoculation, and rash.
- A myocarditis registry for extended follow-up of defined cases out to two years following onset of disease, to ascertain rates of long term serious outcomes (i.e. dilated cardiomyopathy (DCM), heart failure, and death associated smallpox vaccination-associated myocarditis/pericarditis).
- An enhanced surveillance program of between 1-2 years duration to facilitate signal detection following the introduction of ACAM2000.

9. OVERALL SUMMARY AND CONCLUSIONS: BENEFITS VERSUS RISKS

The eradication of naturally occurring smallpox was a major milestone in global public health, eliminating a disease with a case fatality rate of 30% [46]. However, the credible and possible risk of a deliberate release of smallpox necessitates a continued supply of vaccine against this virus. Dryvax®, the only US-licensed vaccine for the prevention of smallpox, is effective and was instrumental in eliminating smallpox in the US, but is no longer manufactured. Consequently, an alternative smallpox vaccine is urgently needed to protect individuals at risk of exposure to smallpox and as an emergency safeguard stockpile for use in the general population in the event of a deliberate smallpox release.

Clinical and non-clinical studies conducted with ACAM2000 provide data supporting the safety and efficacy of this vaccine. While efficacy cannot be proven in the absence of naturally occurring smallpox, ACAM2000 provided protection from lethal challenges with other Orthopox viruses in two animal models and induced positive responses in the great majority of human subjects according to two markers of efficacy, cutaneous response and neutralizing antibody response. Among vaccinia-naïve subjects and previously vaccinated subjects in the Phase 3 studies, vaccination success (as determined by cutaneous response) rates of 96% and 84%, respectively, were observed. Notably, among previously vaccinated subjects where pre-existing immunity had waned (i.e., Baseline neutralizing antibody titers <10), successful rates were higher (94%) and more closely resembled the responses observed in vaccinia-naïve subjects. Robust neutralizing antibody responses to ACAM2000 were found in both vaccinia-naïve and previously vaccinated populations (GMTs of 166 and 286, respectively, in Phase 3 studies). In addition, vaccinia-specific T-cell responses were detected in all subjects assessed for this response (n=30) by at least one of three cell-mediated immunity assays (Table 6).

Overall, the most significant difference between ACAM2000 and Dryvax® is the lower rate of cutaneous response in previously vaccinated subjects: 84% vs. 98%, respectively. As noted, the lower response rate appears to be principally due to the confounding effect of residual immunity. A similar finding was reported in a recent study in Israel, where lack of response to revaccination with Lister vaccine correlated with the interval since last vaccination [37]. In that study, 77.5% of subjects vaccinated <20 years previously responded to revaccination versus 97.2% for persons vaccinated >20 years before. Unlike ACAM2000 or the Israeli Lister vaccine, Dryvax® is able to elicit a more active cutaneous response that is independent of residual immunity [38]. For ACAM2000, an inverse correlation was seen between response rate and level of residual immunity, i.e. response rates were higher in individuals with the lowest baseline neutralizing antibody titers.

As described in earlier sections of this package, not all statistical efficacy endpoints were met in the Phase 3 studies. ACAM2000 narrowly missed the criterion for non-inferiority to Dryvax® with regard to GMTs in the vaccinia-naïve population and did not meet the dermal response endpoint in the previously vaccinated population. Nevertheless, the entirety of the efficacy database supports the conclusion that ACAM2000 will be effective in providing protection against smallpox infection in these individuals. For example, if one accepts that the data are sufficient to support efficacy in the primary vaccination group (vaccinia naïve) based on the ACIP

definition for cutaneous reaction at the site of vaccination, then it follows that the previously vaccinated group should also be protected based on higher neutralizing antibody levels in that group. Furthermore, when one correlates the baseline antibody titer of the previously vaccinated group with the cutaneous reactivity, it is evident that the dermal reaction occurs in greater than 94% of the subjects who lack immunity (based on low neutralizing antibody titer).

ACAM2000 appeared to be somewhat more attenuated than Dryvax® in terms of immunogenicity and safety parameters. In both subject populations, cutaneous reaction rates and the magnitude of the antibody responses (GMT) were lower for ACAM2000 than Dryvax®. This phenomenon was also revealed by greater sensitivity to interference in cutaneous response in previously vaccinated persons with demonstrable residual immunity (neutralizing antibodies). Finally, ACAM2000 was less reactogenic than Dryvax® with respect to inoculation site, lymph node-related, and constitutional symptoms (see Risk Assessment discussion, below).

Revaccination

The CDC currently recommends revaccination of individuals who demonstrate no cutaneous response after Dryvax®. Acambis subscribes to the CDC's recommendation and has included it in the draft package insert for ACAM2000.

Risk Assessment

In the ACAM2000 program, risks of the vaccine were assessed in both the non-clinical and clinical studies. The non-clinical program demonstrated that ACAM2000 is associated with reduced neurovirulence relative to Dryvax®. It is unknown whether this non-clinical finding would translate into a lower risk of PVE. However, previous studies have demonstrated a relationship between mouse neurovirulence and the incidence of PVE associated with different vaccinia vaccine strains [48].

In the clinical program, common AEs were generally expected local signs or symptoms related to the cutaneous reaction to vaccination, or associated systemic symptoms indicative of vaccinia virus replication. These commonly reported AEs included pruritus, erythema, pain, and swelling at the vaccination site, constitutional symptoms (fatigue, headache, myalgia, malaise, feeling hot, and rigors), and lymphadenitis. These commonly reported AEs were generally predictable with regard to time of onset, mild or moderate in intensity, and tolerable. A higher incidence of these events was seen in vaccinia-naïve subjects than in previously vaccinated subjects. This finding is not unexpected, given that the clinical course of the response to revaccination is modulated by preexisting immunity and is typically milder than that to primary vaccination. In both subject populations, the incidence of these common AEs generally was lower, in many cases significantly lower, with ACAM2000 than with Dryvax®.

Mycarditis and/or pericarditis is associated with smallpox vaccination (0.11 [24] and 0.54 [25] cases per thousand vaccinations in the DoD and civilian vaccination programs, respectively), which is a vaccinia virus-specific complication resulting from vaccination with a number of vaccine strains [18]. ACAM2000 was associated with myocarditis at an incidence of 5.73 cases per thousand vaccinations in vaccinia-naïve subjects in the Phase 3 study. The incidence was lower than that seen with Dryvax® (10.38 cases per thousand vaccinations), but the difference was not significant. Myocarditis/pericarditis was not seen in previously vaccinated subjects. The relatively high incidence of myocarditis in the Phase 3 program compared to the DoD and

civilian vaccination programs, where surveillance was largely passive, was attributed to prospective case finding and serial ECG and troponin I monitoring. These measures identified six asymptomatic cases (of the ten total cases reported) that would likely not have been identified by a passive surveillance program. Myocarditis resolved without permanent sequelae in all but one subject who received Dryvax®. It could not be determined whether that subject had preexisting heart disease.

No other SAEs historically associated with smallpox vaccine, including GV, fetal vaccinia, ocular vaccinia, PVE, PV, erythema multiforme, or EV, were reported with ACAM2000, although the studies were not powered to detect these SAEs historically associated with smallpox vaccine. Other adverse reactions noted after recent vaccination programs (including erythema multiforme major/ Stevens-Johnson syndrome, dilated cardiomyopathy, and contact transmission of vaccinia) have not been observed with ACAM2000 [23, 30, 31].

No fatalities were reported in the ACAM2000 clinical program. Historically, the rate of death as a result of complications from smallpox vaccination has been estimated to be 1/1,000,000 among primary vaccinees and 0.25/1,000,000 among revaccinees [15]. The ACIP has indicated that the risk associated with the receipt of smallpox vaccine should be weighed against the risk of exposure to Orthopoxviruses [49].

Conclusion

The license application for ACAM2000 Smallpox Vaccine is based on the safety and vaccination response (cutaneous and serological) demonstrated by the comprehensive clinical development program. There is strong evidence of efficacy in both vaccinia-naïve and previously vaccinated subject populations. The safety risks for ACAM2000 were expected based on historic experience with smallpox vaccines, with the obvserved incidence of myo/pericarditis with both Dryvax and ACAM2000attributed to the increased surveillance that was performed in clinical trials for ACAM2000. On the basis of this clinical program, it is proposed that ACAM2000 vaccine be indicated for the prevention of smallpox disease in populations determined to be at risk for smallpox infection.

10. LIST OF REFERENCES

1. Henderson, DA, Moss, B. Smallpox and vaccinia. In: SA Plotkin, WA Orenstein, eds. Vaccines 3rd Edition. Philadelphia, PA WB Saunders; 1999:74-97.
2. Henderson DA, Inglesby TV, Bartlett JG, Ascher MS, Eitzen E, Jahrling PB, et al. Smallpox as a biological weapon: medical and public health management. JAMA 1999;281:2127-37.
3. WHO Expert Committee on Smallpox. First report. Geneva: World Health Organization;1964. World Health Organization Technical Report Series, No. 283.
4. Benenson, A. S., Kempe, C. H., and Wheeter, R. E., 1952. Problems in Maintaining immunity to smallpox. Am. J. Public Health, 42: 535-541
5. Mack TM, Noble J Jr, Thomas DB. A prospective study of serum antibody and protection against smallpox. Am J Trop Med Hyg. 1972 Mar;21(2):214-8.
6. Hanna, W. Studies in Smallpox and vaccination, Bristol, Wright; 1913
7. Fenner F, Henderson DA, Arita I, Jazek Z, Ladnyi ID. *Smallpox and its Eradication. Chapter 1 (52-53).* Geneva: World Health Organization; 1988.
8. Mack TM. Smallpox in Europe, 1950-1971. Journal of Infectious Diseases 125: 161-169; 1972
9. Fenner F, Henderson DA, Arita I, Jazek Z, Ladnyi ID. *Smallpox and its Eradication. Chapter 3 (152-161).* Geneva: World Health Organization; 1988.
10. Sarkar JK, Mitra AC, Mukherjee MK. Bull World Health Organ. 1975;52(3):307-11.
11. Kempe, C.H., Bowles, C., Meilklejohn, G.,et al. The use of vaccinia hyperimmune gamma globulin in the prophylaxis of smallpox. Bulletin of the World Health Organization, 25: 41-48; 1961
12. Dryvax® Dried Smallpox Vaccine. Prescribing information. Wyeth Pharmaceuticals, Inc., October, 2004.
13. Frey SE, Couch RB, Tacket CO, Treanor JJ, Wolff M, Newman FK, et al. Clinical responses to undiluted and diluted smallpox vaccine. N Engl J Med. 2002;346(17):1265-74.
14. Fulginiti VA, Papier A, Lane JM, Neff JM, Henderson DA. Smallpox vaccination: a review, part II. Adverse events. Clin Infect Dis 2003;37(2):251-71.
15. Lane JM, Ruben FL, Neff JM, and Millar JD. Complications of smallpox vaccination, 1968. N Engl J Med 1969;281(22): 1201-1208.
16. Neff JM, Lane JM, Pert JH, Moore R, Millar JD, Henderson DA. Complications of smallpox vaccination. National survey in the United States, 1963. N Engl J Med 1967;276:125-132.
17. Lane JM, Ruben FL, Neff JM, Millar JD. Complications of smallpox vaccination, 1968: results of 10 state-wide surveys. J Infect Dis 1970;122(4):303-309.

18. Casey C, Vellozzi C, Mootrey GT, Chapman LE, McCauley M, Roper MH, et al. Surveillance guidelines for smallpox vaccine (vaccinia) adverse reactions. MMWR Recomm Rep. 2006;55(RR-1):1-16.

19. Lane JM, Goldstein J. Evaluation of 21st-century risks of smallpox vaccination and policy options. Annals of Internal Medicine 2003;138(6):488-494.

20. Centers for Disease Control and Prevention Recommendations of the Advisory Committee on Immunization Practices (ACIP): use of vaccines and immune globulins in persons with altered immunocompetence. MMWR Morb Mortal Wkly Rep. 1993;42(No. RR-4):1-24.

21. Centers for Disease Control and Prevention. Notice to readers: Supplemental recommendations on adverse events following smallpox vaccine in the pre-event vaccination program: recommendations of the Advisory Committee on Immunization Practices. MMWR Morb Mortal Wkly Rep. 2003;52(13):282-284.

22. Lane J, Millar J. Risks of smallpox vaccination complications in the United States. Am J Epidemiol. 1971;93:238-240.

23. Feery BJ. Adverse reactions after smallpox vaccination. Med J Aust 1977;2(6):180-3.

24. Department of Defense. Smallpox Vaccination Program Safety Summary, June 20, 2006. Retrieved from http://www.smallpox.mil/documents/SPSafetySumJun2006.pdf

25. Centers for Disease Control. Update: adverse events following civilian smallpox vaccination – United States, 2003. MMWR Morb Mortal Wkly Rep 2004;53(05):106-107.

26. Weltzin R, Liu J, Pugachev KV, Myers GA, Coughlin B, Blum PS, et al. Clonal vaccinia virus grown in cell culture as a new smallpox vaccine. Nature Med 2003;9:1125-30.

27. Monath TP, Caldwell JR, Mundt W, Fusco J, Johnson CS, Buller M, et al. ACAM2000 clonal Vero cell culture vaccinia virus (New York City Board of Health strain) – a second-generation smallpox vaccine for biological defense. Int J Infect Dis 2004;Suppl 2:S31-44.

28. Rosenthal SR, Merchlinsky M, Kleppinger C, and Goldenthal KL. Developing New Smallpox Vaccines. Emerg Infect Dis 2001;7(6):920-926.

29. Centers for Disease Control and Prevention. Vaccinia (smallpox) vaccine. Recommendations of the Advisory Committee in Immunization Practices (ACIP), 2001. MMWR 2001;50(RR10):1-25.

30. Centers for Disease Control. Cardiac adverse events following smallpox vaccination – United States 2003. MMWR Morb Mortal Wkly Rep 2003;52(12):248-50.

31. Centers for Disease Control. Update: adverse events following smallpox vaccination – United States, 2003. MMWR Morb Mortal Wkly Rep 2003;52(13):278-82.

32. Cole G, Blanden R. Immunology of poxviruses. In: .Nahmias AJ, O'Reilly RJ, eds Comprehensive Immunology, Immunology of Human Infection, Part II, Viruses and Parasites. Vol 9 New York, NY:Plenum; 1982:1-19.

33. Frey SE, Newman FK, Cruz J, Shelton WB, Tennant JM, Polach T, Rothman AL, Kennedy JS, Wolff M, Belshe RB, Ennis FA. Dose-related effects of smallpox vaccine. N Engl J Med. 2002 Apr 25;346(17):1275-80.

34. Rock MT, Yoder SM, Talbot TR, Edwards KM, Crowe JE Jr. Cellular immune responses to diluted and undiluted aventis pasteur smallpox vaccine. J Infect Dis. 2006 Aug 15;194(4):435-43.

35. Frey SE, Newman FK, Yan L, Lottenbach KR, Belshe RB. Response to smallpox vaccine in persons immunized in the distant past. JAMA. 2003 Jun 25;289(24):3295-9.

36. McIntosh K, Cherry JD, Benenson AS, Connor JD, Alling DW, Rolfe UT, et al. Clinical and serologic study of four smallpox vaccines comparing variations of dose and route of administration. Standard percutaneous revaccination of children who receive primary percutaneous vaccination. J Infect Dis 1977;135:155–66.

37. Orr N, Forman M, Marcus H, et al. Clinical and immune responses after revaccination of israeli adults with the Lister strain of vaccinia virus. J Infect Dis. 2004 Oct 1;190(7):1295-302.

38. Treanor J, Wu H, Liang H, Topham DJ. Immune responses to vaccinia and influenza elicited during primary versus recent or distant secondary smallpox vaccination of adults. Vaccine. 2006 Nov 17;24(47-48):6913-23.

39. Netter, R: Vaccinia. In: R Debré and J Celers, eds. Clinical Virology, W.B. Saunders, Philadelphia, pp 436-304, 1970.

40. Sejvar JJ, Labutta RJ, Chapman LE, Grabenstein JD, Iskander J, Lane JM. Neurologic Adverse Events Associated With Smallpox Vaccination in the United States, 2002-2004. JAMA 2005;294:2744-2750.

41. Salo OP, Somer T, Aho K, Cantell K: Studies of false positive serological tests for syphilis following smallpox vaccination. Ann Med Exp Biol Fenn, 44:304-306, 1966.

42. Apparent transient false-positive FTA-ABS test following smallpox vaccination. OK State Med Assoc, p 372.

43. Grossman LJ, Peery TM. Biologically false positive serologic tests for syphilis due to smallpox vaccination. Am J Clin Pathol, 51(3):375-378, 1968.

44. Seet LC, Sng EH, Thomas M. False positive results in V D R L slide test following smallpox immunization. Asian J Infect Dis, 3:137-138, 1979.

45. Collard P, Evans AJ. Smallpox as a cause of false positive reactions to the Kahn test. Brit J Vener Dis, 34:256, 1958.

46. Breman JG, Henderson DA. Diagnosis and management of smallpox. N Engl J Med 2002;346(17):1300-1308.

47. Centers for Disease Control and Prevention (2002). Public health emergency preparedness and response, FAQs about smallpox vaccine.

48. Fenner F, Henderson DA, Arita I, Jazek Z, Ladnyi ID. *Smallpox and its Eradication. Chapter 11 (539-592).* Geneva: World Health Organization; 1988.

49. Wharton M, Strikas RA, Harpaz R, Rotz LD, Schwartz B, Casey CG, et al. Recommendations for Using Smallpox Vaccine in a Pre-Event Vaccination Program. Supplemental Recommendations of the Advisory Committee on Immunization Practices (ACIP) and the Healthcare Infection Control Practices Advisory Committee (HICPAC). MMWR 2003;52(RR07);1-16.

11. APPENDICES

11.1. Appendix 1 - Analysis of Efficacy Data from ITT Population

This appendix presents a summary of the data and conclusions from the two Phase 3 studies comparing ACAM2000 to Dryvax®, using data collected from all subjects vaccinated with either ACAM2000 or Dryvax (ITT population) at all sites, including the four sites excluded from the final analysis presented in Section 6 of this briefing document. ITT subjects with no evaluations on Day 7 or after were considered vaccination failures. The conclusions from the final analysis presented in Section 6 and the analysis presented below, based on the expanded data set, do not differ with respect to the assessment of non-inferiority of ACAM2000 relative to Dryvax® in both the vaccinia-naïve and previously vaccinated populations. The data for primary vaccination and for previously vaccinated subjects are provided in Tables 15 and 16, respectively.

Table 15: Phase 3 Efficacy Results for ACAM2000 versus Dryvax® in Vaccinia-Naïve Subjects from the ITT population (Study H-400-009)

Treatment group	ACAM2000	Dryvax®
**Vaccination Success based on Cutaneous Response**		
ITT Population, n	873	289
Vaccination Success, n (%)	839 (96)	287 (99)
97.5% CI [a] (Criterion for Non-Inferiority)	-4.80 (>-5.00)	
**Neutralizing Antibody Titer**		
AnE Population, n	624	208
GMT	168	259
97.5% CI [b] (Criterion for Non-Inferiority)	-0.305 (>-0.301)	

a. Lower bound of the 97.5% one-sided CI on the difference between ACAM2000 and Dryvax® derived by normal approximation to the binomial distribution.
b. Lower bound of the 97.5% one-sided CI on the difference in the mean log10 GMT between ACAM2000 and Dryvax® derived using analysis of variance (ANOVA).

Table 16: Phase 3 Efficacy Results for ACAM2000 versus Dryvax® in Previously Vaccinated Subjects from the ITT population (Study H-400-012)

Treatment group	ACAM2000	Dryvax®
**Revaccination Success based on Cutaneous Response**		
ITT Population, n	1371	448
Revaccination Success, n (%)	1113 (81)	427 (95)
97.5% CI [a]<br>(Criterion for Non-Inferiority)	-17.00 (>-10)	
**Neutralizing Antibody Titer**		
AnE Population, n	803	412
GMT	279	425
97.5% CI [b]<br>(Criterion for Non-Inferiority)	-0.262 (>-0.301)	

a. Lower bound of the 97.5% one-sided CI on the difference between ACAM2000 and Dryvax® derived by normal approximation to the binomial distribution.
b. Lower bound of the 97.5% one-sided CI on the difference in the mean $\log_{10}$ GMT between ACAM2000 and Dryvax® derived using ANOVA.

11.2. Appendix 2 - Demographics

11.2.1. Summary of Demographics

Table 17 presents a summary of demographic and baseline characteristics by baseline vaccination status for subjects included in the safety population for the final analysis for each treatment regimen (ACAM2000 or Dryvax®) combined and by treatment groups. A total of 873 vaccinia-naïve subjects and 1371 previously vaccinated subjects were treated with ACAM2000 in the Phase 3 studies. A total of 289 vaccinia-naïve and 448 previously vaccinated subjects were treated with Dryvax® in these Phase 3 studies.

The racial distribution in the studies was generally similar to that of the overall US population (76.8% Caucasian, 10.1% African-American, and 9.3% Hispanic).

As anticipated, the vaccinia-naïve subjects were younger (mean age of 23.0 versus 49.0 years) than previously vaccinated subjects. The vaccinia-naïve subjects were slightly taller (174 cm versus 171 cm) and had a slightly lower mean body weight (81 kg versus 85 kg) than the previously vaccinated subjects. There was a higher percentage of male vaccinia-naïve subjects than male previously vaccinated subjects (65% versus 49%).

Table 17: Demographics and Baseline Characteristics, By Subject Population, Treatment Group, and ACAM2000 Lot, Final Analysis Safety Population

Parameter / Statistic	Subject Population/Treatment Group									
	Vaccinia-Naïve Subjects (Study H-400-009)					Previously Vaccinated Subjects (Study H-400-012)				
	ACAM 2000 $2.2{\times}10^8$ (n=289)	ACAM 2000 $1.9{\times}10^8$ (n=298)	ACAM 2000 $1.3{\times}10^8$ (n=286)	ACAM 2000 All Doses (n=873)	Dryvax® $1.5{\times}10^8$ (n=289)	ACAM 2000 $2.2{\times}10^8$ (n=455)	ACAM 2000 $1.9{\times}10^8$ (n=459)	ACAM 2000 $1.3{\times}10^8$ (n=457)	ACAM 2000 All Doses (n=1371)	Dryvax® $1.5{\times}10^8$ (n=448)
**Sex, n (%)**										
Male	200 (69)	187 (63)	185 (65)	572 (66)	182 (63)	218 (48)	247 (54)	220 (48)	685 (50)	214 (48)
Female	89 (31)	111 (37)	101 (35)	301 (34)	107 (37)	237 (52)	212 (46)	237 (52)	686 (50)	234 (52)
**Race, n (%)**										
Caucasian	220 (76)	219 (73)	221 (77)	660 (76)	205 (71)	362 (80)	365 (80)	348 (76)	1075 (78)	348 (78)
Afr-Amer	32 (11)	43 (14)	25 (9)	100 (11)	40 (14)	38 (8)	40 (9)	58 (13)	136 (10)	51 (11)
Hispanic	27 (9)	28 (9)	31 (11)	86 (10)	32 (11)	39 (9)	43 (9)	39 (9)	121 (9)	38 (8)
Asian	2 (1)	4 (1)	4 (1)	10 (1)	4 (1)	5 (1)	3 (1)	3 (1)	11 (1)	4 (1)
Other	8 (3)	4 (1)	5 (2)	17 (2)	8 (3)	11 (2)	8 (2)	9 (2)	28 (2)	7 (2)
**Age, (years)**										
N	289	298	286	873	289	455	459	457	1371	448
Mean (±SD)	22.9 (3.5)	23.2 (3.6)	22.8 (3.5)	23.0 (3.5)	22.9 (3.5)	44.8 (10.0)	49.0 (10.4)	48.9 (9.3)	48.9 (10.0)	49.2 (9.8)
Min, Max	18, 30	18, 30	18, 30	18, 30	18, 30	31, 82	31, 78	31, 80	31, 82	31, 84
**Height, (cm)**										
N	288	298	286	872	289	455	458	456	1369	448
Mean (±SD)	174 (9.7)	174 (9.8)	174 (10.4)	174 (10.0)	173 (9.8)	171 (10.1)	171 (9.5)	170 (10.3)	171 (10.0)	171 (10.1)
Min, Max	150, 201	147, 199	146, 203	146, 203	132, 203	149, 201	144, 196	142, 203	142, 203	142, 208

Table 17: Demographics and Baseline Characteristics, By Subject Population, Treatment Group, and ACAM2000 Lot, Final Analysis Safety Population (Continued)

Parameter / Statistic	Subject Population/Treatment Group									
	Vaccinia-Naïve Subjects (Study H-400-009)					Previously Vaccinated Subjects (Study H-400-012)				
	ACAM 2000 2.2×10^8 (n=258)	ACAM 2000 1.9×10^8 (n=264)	ACAM 2000 1.3×10^8 (n=258)	ACAM 2000 All Doses (n=780)	Dryvax® 1.5×10^8 (n=257)	ACAM 2000 2.2×10^8 (n=258)	ACAM 2000 1.9×10^8 (n=264)	ACAM 2000 1.3×10^8 (n=258)	ACAM 2000 All Doses (n=780)	Dryvax® 1.5×10^8 (n=257)
**Weight, (kg)**										
N	288	298	286	872	289	455	458	456	1369	448
Mean (±SD)	81 (20.7)	82 (21.1)	81 (19.4)	81 (20.4)	81 (19.1)	85 (20.9)	86 (21.1)	83 (20.1)	85 (20.7)	85 (21.6)
Min, Max	44, 158	43, 159	42, 159	42, 159	48, 158	45, 163	46, 173	47, 201	45, 201	46, 272

11.3. Appendix 3 – Subjects with Suspect or Probable Myocarditis

Table 18: Subjects with Treatment-Emergent Suspect or Probable Myocarditis, by Treatment Group and Study

Subject No. (Dose) Sex/Race/Age	Notable Medical History	CAP Classification	Clinical Signs	ECG and ECHO Findings		Laboratory Findings	PV Day of Onset and Outcome
				Local	Core		
ACAM2000 (Vaccinia-Naïve Subjects)							
**Study H-400-002**							
2063<br>($1.0x10^8$)<br>Male<br>Caucasian<br>19 years	None	Suspect subclinical myocarditis **[a]**	None reported	ECG: mild T wave flattening with very slight elevation of ST segment in Leads I and aVL consistent with but not diagnostic of myocarditis<br>3 month F/U ECHO<br>LVEF = 64%	ECG Not done<br>3 month F/U ECHO<br>LVEF = 55%	Troponin and creatine phosphokinase (CPK) normal	14<br>Resolved without sequelae

Table 18: Subjects with Treatment-Emergent Suspect or Probable Myocarditis, by Treatment Group and Study (Continued)

Subject No. (Dose) Sex/Race/Age	Notable Medical History	CAP Classification	Clinical Signs	ECG and ECHO Findings		Laboratory Findings	PV Day of Onset and Outcome
				Local	Core		
ACAM2000 (Vaccinia-Naïve Subjects)							
**Study H-400-005**							
1238<br>($6.8x10^7$)<br>Male<br>Caucasian<br>18 years	None	Probable myocarditis **[a]**	Chest tightness	ECG: T-wave inversion in Lead III and nonspecific changes in Leads II and aVF<br>ECHO 16 month F/U<br>LVEF = 60%	ECG Not done<br>ECHO 16 month F/U<br>LVEF = >55%	CPK elevated (400 IU/L; normal range 38 to 199 IU/L), troponin I evaluated (3.8 ng/mL; normal range <0.5 ng/mL, CPK-MM fraction elevated (88%); and CPK-MB elevated (12%; normal range 0%)	10<br>Resolved without sequelae

Table 18: Subjects with Treatment-Emergent Suspect or Probable Myocarditis, by Treatment Group and Study (Continued)

Subject No. (Dose) Sex/Race/Age	Notable Medical History	CAP Classification	Clinical Signs	ECG and ECHO Findings		Laboratory Findings	PV Day of Onset and Outcome
				Local	Core		
ACAM2000 (Vaccinia-Naïve Subjects)							
**Study H-400-009**							
023-190<br><br>($1.9x10^8$)<br><br>Male<br>African American<br>22 years	Current Smoker	Suspect subclinical myocarditis	None Reported	ECG: sinus arrhythmia, ST elevation, T-wave inversion<br><br>Day 10 ECHO normal	Significant anterior T-wave inversion compared with baseline ECG<br>Day 10 ECHO<br>LVEF = 55 to 60%	Troponin I and CK-MB normal	9<br><br>Resolved without sequelae
048-116<br><br>($1.3x10^8$)<br><br>Male<br>Caucasian<br>23 years	None	Probable myocarditis	Chest pain, exercise tolerance decreased	ECG: sinus rhythm with sinus arrhythmia, minimal voltage criteria for LVH; ST-T elevations leads I, aVL, V5, V6; inverted T wave lead III<br>Day 10 ECHO<br>LVEF = 50%	Significant inferolateral T-wave inversion on Day 21 as compared to Day 10 ECG<br>Day 10 ECHO<br>LVEF = $\geq$ 60%	Troponin I elevated (4.8 ng/mL; normal range 0.0 to 0.4 ng/mL), CK-MB 16.7 ng/mL (normal range 0.0 to 3.3 ng/mL)	11<br><br>Resolved with sequelae (abnormal ECG on discharge with nonspecific ST-T wave changes)

Table 18: Subjects with Treatment-Emergent Suspect or Probable Myocarditis, by Treatment Group and Study (Continued)

Subject No. (Dose) Sex/Race/Age	Notable Medical History	CAP Classification	Clinical Signs	ECG and ECHO Findings		Laboratory Findings	PV Day of Onset and Outcome
				Local	Core		
ACAM2000 (Vaccinia-Naïve Subjects)							
**Study H-400-009**							
056-111<br>($1.9X10^8$)<br>Male<br>Caucasian<br>23 years	None	Probable subclinical myocarditis	None reported	Elevated ST segments on ECG<br>Day 56 ECHO<br>LVEF = 60%	No significant ECG findings<br>Day 56 ECHO<br>LVEF = $\geq$ 60%	Troponin I elevated (1.8 ng/mL; normal range <0.5 ng/mL)	10<br>Resolved without sequelae
080-112<br>($1.9X10^8$)<br>Male<br>Hispanic<br>18 years	None	Probable myocarditis	Dyspnea; palpitations	ECG: 2 mm ST elevation in Leads I and aVL, suggesting lateral injury; T-wave inversion and 1 mm ST depression in Leads III and F<br>Day 22 ECHO<br>LVEF = 52%	Significant inferior T-wave inversion compared to Baseline ECG<br>Day 22 ECHO<br>LVEF = 60%<br>12 month Follow-up<br>LVEF = $\geq$ 60%	Troponin I, CK-MB, and CPK and isoenzymes normal	9<br>Resolved without sequelae

Table 18: Subjects with Treatment-Emergent Suspect or Probable Myocarditis, by Treatment Group and Study (Continued)

Subject No. (Dose) Sex/Race/Age	Notable Medical History	CAP Classification	Clinical Signs	ECG and ECHO Findings		Laboratory Findings	PV Day of Onset and Outcome
				Local	Core		
ACAM2000 (Vaccinia-Naïve Subjects)							
**Study H-400-009**							
094-114<br>(1.3X10^8)<br>Male<br>Caucasian<br>21 years	None	Probable subclinical myocarditis	None reported	ECG: sinus bradycardia with sinus arrhythmia and first degree AV block<br>Day 20 ECHO<br>LVEF = 48%	No significant ECG findings<br>Day 20 ECHO<br>LVEF= $\geq$ 60%	Troponin I elevated (0.11; normal range <0.4)	9<br>Resolved without sequelae
Dryvax® (Vaccinia-Naïve Subjects)							
**Study H-400-009**							
004-103<br>(1.5X10^8)<br>Male<br>Asian<br>20 years	None	Suspect myocarditis	None reported	Significant ST and T wave changes on ECG	Significant anterior T wave inversion on ECG<br>9 months post-vaccination<br>LVEF= $\geq$ 60%	Troponin I normal; “all cardiac labs normal”	20<br>Resolved without sequelae

Table 18: Subjects with Treatment-Emergent Suspect or Probable Myocarditis, by Treatment Group and Study (Continued)

Subject No. (Dose) Sex/Race/Age	Notable Medical History	CAP Classification	Clinical Signs	ECG Findings/ECHO		Laboratory Findings	PV Day of Onset and Outcome
				Local	Core		
Dryvax® (Vaccinia-Naïve Subjects)							
**Study H-400-009**							
054-106<br>(1.5x10^{8})<br>Male<br>Caucasian<br>23 years	None	Probable myocarditis	Exercise tolerance decreased; palpitations; "crushing" chest pain; dyspnea	Abnormal ST-T wave elevations on ECG	Day 21 ECG showed minimal decrease in T-wave amplitude and ST flattening (possibly consistent with myopericarditis) compared with Day 10 ECG<br>Day 42 MUGA LVEF = 70%	Troponin I elevated (6.7 ng/mL; normal range <0.2 ng/mL); CK-MB normal	11<br>Resolved without sequelae

Table 18: Subjects with Treatment-Emergent Suspect or Probable Myocarditis, by Treatment Group and Study (Continued)

Subject No. (Dose) Sex/Race/Age	Notable Medical History	CAP Classification	Clinical Signs	ECG Findings/ECHO		Laboratory Findings	PV Day of Onset and Outcome
				Local	Core		
Dryvax® (Vaccinia-Naïve Subjects)							
**Study H-400-009**							
065-137<br>($1.5x10^8$)<br>Female<br>Caucasian<br>21 years	Smoker, obesity; borderline high blood pressure; occasional sinus tachycardia	Probable myocarditis	None reported	No significant findings on ECG<br>Day 15 ECHO<br>LVEF = 52%<br>Day 49 ECHO<br>LVEF = 44%<br>1 year F/U<br>LVEF = 35-42%<br>2 year F/U<br>LVEF = 27-32%	No significant ECG findings<br>Day 15 ECHO<br>LVEF = 35%<br>Day 49 ECHO<br>LVEF = 30%<br>1 year F/U<br>LVEF = 35%<br>2 year F/U<br>LVEF = 25%	Troponin I elevated (3.2 ng/mL; normal range <0.3 ng/mL); CPK normal	9<br>Ongoing

AV=atrioventricular; CPK=creatine phosphokinase; CK-MB=creatine kinase-myocardial band; CPK-MM= creatine phosphokinase skeletal muscle; PV=Post vaccination.

a. This case was not reviewed by the CAP, but was classified by the Sponsor’s medical officer.

11.4. Appendix 4 – Cardiac Algorithm

The algorithm for diagnosis and management of subjects with suspect myocarditis/pericarditis, that was used in the ACAM2000 Phase 3 studies (H-400-009 and H-400-012) is presented in Figure 9 below.

Figure 9: Algorithm for Diagnosis and Management of Subjects with Suspect Myocarditis/Pericarditis

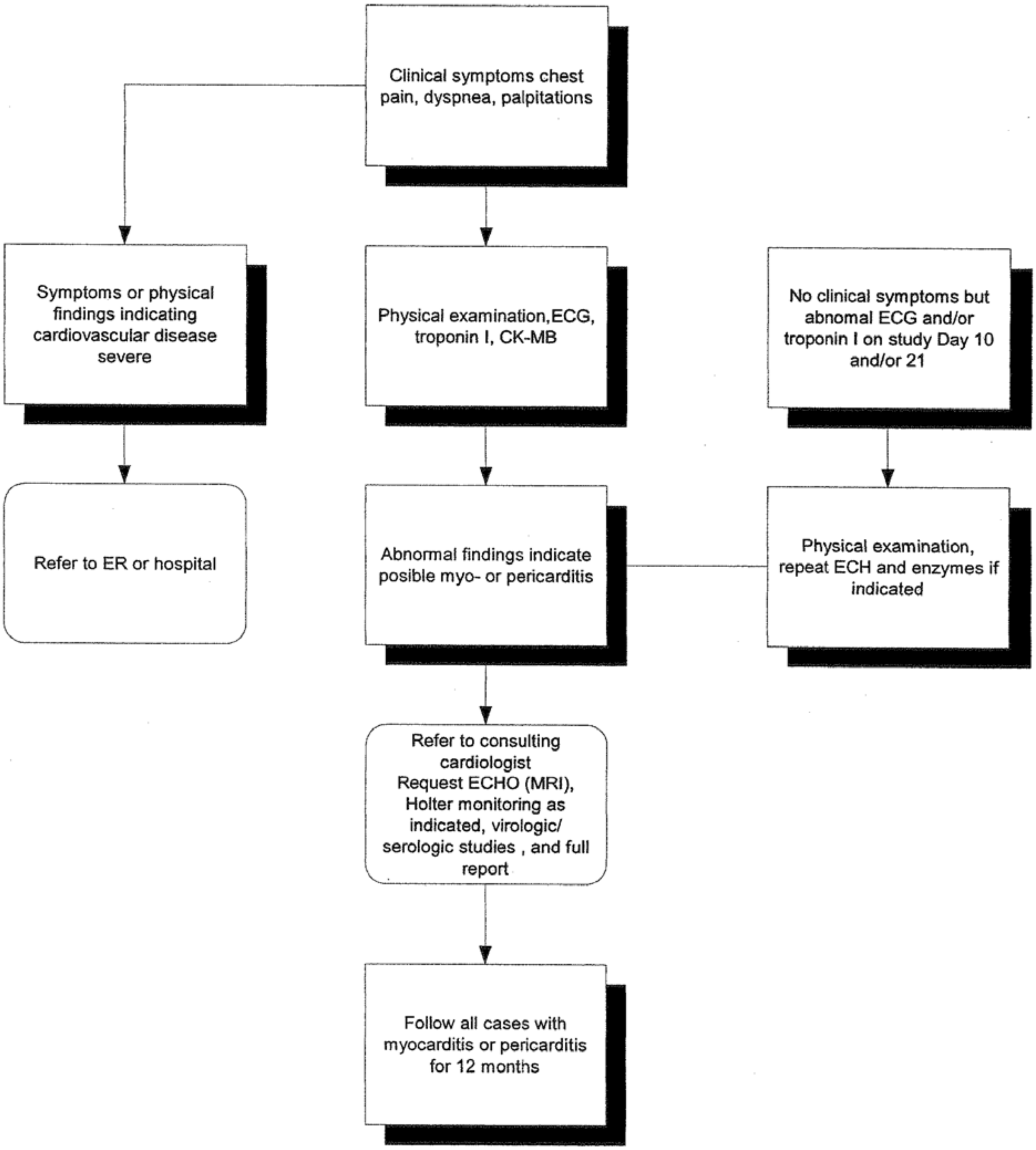

16.2 SUBJECT DATA LISTINGS

INDEX OF LISTINGS

16.2.1. DISCONTINUED SUBJECTS....3

16.2.1.1. Discontinued subjects....3
16.2.1.2. Overall Assessment of Drop-outs (until Visit 3/Visit 5)....6

16.2.2. PROTOCOL DEVIATIONS....9

16.2.2.1. Known violations of Inclusion/Exclusion Criteria (i.e. documented in the e-CRF by ticking relevant criterion)....9
16.2.2.2. Violations of inclusion/exclusion criteria revealed by DM....11
16.2.2.3. Date of informed consent is after date of screening....12
16.2.2.4. Note to files (indicating a PV)....13
16.2.2.5. Violation against visit windows....14
16.2.2.5.1. Days between V1 and Screening (<-28 d)....14
16.2.2.5.2. Days between V2 and V1 (outside 10-15 d)....15
16.2.2.5.3. Days between V3 and V1 (outside 28-35 d)....16
16.2.2.5.4. Days between V4 and V3 (outside 10-15 d)....17
16.2.2.5.5. Days between V5 and V3....17
16.2.2.6. Prohibited medical history....18
16.2.2.7. Prohibited previous/ concomitant treatments....18
16.2.2.8. Other protocol deviations from the e-CRF comments....19
16.2.2.9. Deviations during vaccination....19
16.2.2.10. Not evaluable antibody sample detected at BN analytical laboratory:....19

16.2.3. SUBJECTS EXCLUDED FROM THE ANALYSIS....21

16.2.3.1. Subjects excluded from the safety analysis....21
16.2.3.2. Subjects excluded from per protocol analysis....21

16.2.4. DEMOGRAPHIC DATA....24

16.2.4.1. Demographic data....24
16.2.4.2. History of smallpox vaccination....51
16.2.4.3. Medical history....79
16.2.4.4. Baseline signs and symptoms....432

16.2.5. COMPLIANCE AND/OR DRUG CONCENTRATION DATA....449

16.2.5.1. Fulfillment of inclusion criteria....449
16.2.5.2. Fulfillment of exclusion criteria....451
16.2.5.3. Exception to inclusion/ exclusion criteria....452
16.2.5.4. Confirmation of eligibility at end of screening....454
16.2.5.5. Re-check of eligibility criteria at Visit 1/3....478
16.2.5.6. Visit dates....517
16.2.5.7. Time between visits....540
16.2.5.8. Assignment of vial numbers....570
16.2.5.9. Vaccination....635

16.2.6. IMMUNOGENECITY DATA....707

16.2.6.1. Antibody responses against MVA-BN® using a direct ELISA, Safety (FA) dataset....707
16.2.6.2. Antibody responses against MVA-BN® using a PRNT assay, Safety (FA) dataset....733

16.2.7. ADVERSE EVENT LISTING....758

16.2.7.1. Solicited adverse events....758
16.2.7.1.1. Solicited adverse events - local symptom: pain....758
16.2.7.1.2. Solicited adverse events - local symptoms: redness, swelling, induration....831
16.2.7.1.3. Solicited adverse events - local symptoms: other symptoms....1084
16.2.7.1.4. Solicited adverse events - general symptom: body temperature....1091

16.2.7.1.5. Solicited adverse events - general symptoms: headache, myalgia, nausea, fatigue 1205
16.2.7.1.6. Solicited adverse events - general symptoms: other symptoms 1528
16.2.7.2. Unsolicited adverse events 1560
16.2.7.2.1. Adverse events: details 1560
16.2.7.2.2. Adverse events: MedDRA coding 1647
16.2.7.2.3. Deaths, other serious and significant adverse events: MedDRA coding 1719

16.2.8. LISTING OF INDIVIDUAL LABORATORY MEASUREMENTS BY SUBJECT 1723

16.2.8.1. Laboratory Normal Ranges 1723
16.2.8.2. Date and time of blood analysis, check for sample taken, laboratory order number 1726
16.2.8.3. Date and time of urine analysis, check for sample taken, laboratory order number 1926
16.2.8.4. Date and time of serum pregnancy test, check for sample taken, laboratory order number 1950
16.2.8.5. Date and time of urine pregnancy test, check for sample taken, result 1963
16.2.8.6. Central laboratory tests, results 2010

16.2.9. SAFETY DATA 4413

16.2.9.1. Study closure form 4413
16.2.9.2. Physical examination 4440
16.2.9.2.1. Physical examination at Screening 4440
16.2.9.2.2. Physical examination - changes since last examination 4466
16.2.9.3. Vital signs 4596
16.2.9.4. ECG 4740
16.2.9.4.1. ECG - date, time and overall result of investigator and cardiologist 4740
16.2.9.4.2. Central ECG raw data 4849
16.2.9.4.3. Central ECG data: PR, QRS, RR interval, QTc Bazett and Fridericia correction 5388
16.2.9.5. Check for hand-out and collection of diary 5494
16.2.9.6. General comments 5562
16.2.9.7. Prior and concomitant medication 5708

16.2.10. FOLLOW-UP DATA 5767

16.2.10.1. Follow Up: SAE, SIAE and unresolved AEs: MedDRA coding 5767
16.2.10.2. Listing of Individual Laboratory Measurements by Subject 5775
16.2.10.2.1. Laboratory Normal Ranges 5775
16.2.10.2.2. Date and time of blood analysis, check for sample taken, laboratory order number 5779
16.2.10.2.3. Central laboratory tests, results 5826
16.2.10.3. Safety data 5865
16.2.10.3.1. Physical examination - changes since last examination 5865
16.2.10.3.2. Vital signs 5890
16.2.10.3.3. ECG 5913
16.2.10.3.3.1. ECG - date, time and overall result of investigator and cardiologist 5913
16.2.10.3.3.2. Central ECG raw data 5959
16.2.10.3.4. General comments 5966

16.2.1. Discontinued subjects

16.2.1.1. Discontinued subjects

Group	Subj	Screening number	Main reason for study termination	If other, please specify:	Date of withdrawal	Date of last dosage	General comment
Group 1 (MM)	148	293	subject unwilling/unable to comply with study requirements		30AUG2006	01AUG2006	PHONE CALL MADE ON 28.09.06 TO RULE OUT PREGNANCY. SUBJECT HAD HER PERIOD AFTER THE 30.08.06
	268	518	withdrawal of consent		16AUG2006	03AUG2006	SUBJECT DOES NOT WANT TO CONTINUE THE STUDY BEAUSE OF "HIGH BLOOD PRESSURE WHEN AT HCR". SUBJECT FEELS WELL AND SAYS HE HAS NO COMPLAINTS.
	306	574	subject unwilling/unable to comply with study requirements		31OCT2006	28SEP2006	
	400	587	subject unwilling/unable to comply with study requirements		19SEP2006	19SEP2006	DUE TO MEETING NOTES FROM 07.03.07 THE "DATE OF WITHDRAWAL" IS THE DATE OF THE "LAST FACE-TO-FACE CONTACT" WITH THE SUBJECT. A FU-VISIT SHOULD BE DONE FOR THE SUBJECT'S SAFETY. 2ND DIARY NOT BROUGHT.
	433	766	withdrawal of consent		09OCT2006	27SEP2006	SUBJECT MOVES TO CANADA AND CAN NOT CONTINUE THE STUDY
	643	1081	withdrawal of consent		18OCT2006	18OCT2006	
	670	1090	subject unwilling/unable to comply with study requirements		23JAN2007	23NOV2006	SUBJ. DOES NOT ANSWER PHONE CALLS, CERTIFIED MAIL SENT TO SUBJECT. THEREFORE DIARY FROM 2ND VACCINATION CANNOT BE COLLECTED.
	734	1120	subject unwilling/unable to comply with study requirements		03JAN2007	09NOV2006	

Discontinued subjects

Group	Subj	Screening number	Main reason for study termination	If other, please specify:	Date of withdrawal	Date of last dosage	General comment
Group 2 (MP)	105	225	withdrawal of consent		18JUL2006	03JUL2006	SUBJECT WENT TO CONTROL ECG WITH ERGOMETRY ON 07/08/06 AND REFUSED SECOND VACCINATION. SUBJ. INFORMED VIA CERTIFIED MAIL THAT SHE SHOULD UNDERGO A FINAL SAFETY EXAMINATION.
	284	487	withdrawal of consent		01AUG2006	01AUG2006	SUBJ. INFORMED VIA CERTIFIED MAIL THAT HE SHOUL UNDERGO A FINAL SAFETY EXAMINATION.
	328	560	withdrawal of consent		10AUG2006	10AUG2006	
	385	598	subject unwilling/unable to comply with study requirements		19DEC2006	22AUG2006	
	536	926	adverse event, making it unsafe for the subject to continue		16OCT2006	27SEP2006	
	699	1136	withdrawal of consent		15DEC2006	04DEC2006	WITHDRAWAL OF CONSENT DUE TO PERSONAL REASONS (NEW JOB IN OTHER CITY)
	738	1225	withdrawal of consent		20NOV2006	07NOV2006	SUBJECT IS NOT WILLING TO COMPLY ANY MORE.
	781	1269	withdrawal of consent		25JAN2007	09NOV2006	
Group 3 (PP)	399	699	lost to follow-up		12SEP2006	12SEP2006	SUBJ. COULD NEVER BE REACHED AFTER V1
	580	979	adverse event, making it unsafe for the subject to continue		07NOV2006	05OCT2006	DUE TO MEETING NOTES FROM 07.03.07 THE "DATE OF WITHDRAWAL" IS THE DATE OF THE LAST "FACE-TO-FACE CONTACT" WITH THE SUBJECT. NEVERTHELESS THERE HAS TO BE A FU-VISIT FOR SAFETY.
	613	1042	withdrawal of consent		09OCT2006	09OCT2006	SUBJECT WAS ASKED TO COME TO AN FOLLOW UP EXAMINATION, BUT SUBJECT DOES NOT WANT ANY FURTHER EXAMINATION

Discontinued subjects

Group	Subj	Screening number	Main reason for study termination	If other, please specify:	Date of withdrawal	Date of last dosage	General comment
Group 3 (PP)	661	1082	withdrawal of consent		30OCT2006	18OCT2006	SUBJECT MOVED TO HAMBURG AND THEREFORE CANNOT PARTICIPATE ANY MORE.
	687	1164	subject unwilling/unable to comply with study requirements		20DEC2006	20OCT2006	
	782	1181	subject unwilling/unable to comply with study requirements		11DEC2006	10NOV2006	WITHDRAWAL OF CONSENT AND INCOMPLIANCE TO PERFORM VISIT 5 AND FOLLOW-UP. SUBJECT IS HEALTHY AND DOING WELL.

16.2.1.2. Overall Assessment of Drop-outs (until Visit 3/Visit 5)

Group	Subj	Returned for visit 2	Returned for/ completed visit 3	Returned for visit 4	Completed visit 5/ Final Examination	Date of withdrawal	Study Day*	Premature termination: Main reason for study termination	Premature termination: If other, please specify:	General comment
Group 1 (MM)	148	yes	yes	no	yes	30AUG2006	58	subject unwilling/unable to comply with study requirements		PHONE CALL MADE ON 28.09.06 TO RULE OUT PREGNANCY. SUBJECT HAD HER PERIOD AFTER THE 30.08.06
	268	yes	no	no	no	16AUG2006	13	withdrawal of consent		SUBJECT DOES NOT WANT TO CONTINUE THE STUDY BEAUSE OF "HIGH BLOOD PRESSURE WHEN AT HCR". SUBJECT FEELS WELL AND SAYS HE HAS NO COMPLAINTS.
	306	yes	yes	no	yes	31OCT2006	67	subject unwilling/unable to comply with study requirements		
	400	yes	yes	no	no	19SEP2006	29	subject unwilling/unable to comply with study requirements		DUE TO MEETING NOTES FROM 07.03.07 THE "DATE OF WITHDRAWAL" IS THE DATE OF THE "LAST FACE-TO-FACE CONTACT" WITH THE SUBJECT. A FU-VISIT SHOULD BE DONE FOR THE SUBJECT'S SAFETY. 2ND DIARY NOT BROUGHT.
	433	yes	yes	yes	no	09OCT2006	41	withdrawal of consent		SUBJECT MOVES TO CANADA AND CAN NOT CONTINUE THE STUDY
	643	no	no	no	no	18OCT2006	0	withdrawal of consent		
	670	yes	yes	no	no	23JAN2007	92	subject unwilling/unable to comply with study requirements		SUBJ. DOES NOT ANSWER PHONE CALLS, CERTIFIED MAIL SENT TO SUBJECT. THEREFORE DIARY FROM 2ND VACCINATION CANNOT BE COLLECTED.

*: Date of premature termination - Date of visit 1

Overall Assessment of Drop-outs (until Visit 3/Visit 5)

Group	Subj	Returned for visit 2	Returned for/ completed visit 3	Returned for visit 4	Completed visit 5/ Final Examination	Date of withdrawal	Study Day*	Premature termination: Main reason for study termination	Premature termination: If other, please specify:	General comment
Group 1 (MM)	734	yes	no	no	yes	03JAN2007	55	subject unwilling/unable to comply with study requirements		
Group 2 (MP)	105	yes	no	no	no	18JUL2006	15	withdrawal of consent		SUBJECT WENT TO CONTROL ECG WITH ERGOMETRY ON 07/08/06 AND REFUSED SECOND VACCINATION. SUBJ. INFORMED VIA CERTIFIED MAIL THAT SHE SHOULD UNDERGO A FINAL SAFETY EXAMINATION.
	284	no	no	no	no	01AUG2006	0	withdrawal of consent		SUBJ. INFORMED VIA CERTIFIED MAIL THAT HE SHOUL UNDERGO A FINAL SAFETY EXAMINATION.
	328	no	no	no	no	10AUG2006	0	withdrawal of consent		
	385	yes	no	no	no	19DEC2006	119	subject unwilling/unable to comply with study requirements		
	536	yes	no	no	yes	16OCT2006	19	adverse event, making it unsafe for the subject to continue		
	699	yes	yes	yes	no	15DEC2006	42	withdrawal of consent		WITHDRAWAL OF CONSENT DUE TO PERSONAL REASONS (NEW JOB IN OTHER CITY)
	738	yes	no	no	no	20NOV2007	378	withdrawal of consent		SUBJECT IS NOT WILLING TO COMPLY ANY MORE.
	781	yes	no	no	yes	25JAN2007	77	withdrawal of consent		
Group 3 (PP)	399	no	no	no	no	12SEP2006	0	lost to follow-up		SUBJ. COULD NEVER BE REACHED AFTER V1

*: Date of premature termination - Date of visit 1

Overall Assessment of Drop-outs (until Visit 3/Visit 5)

Group	Subj	Returned for visit 2	Returned for/ completed visit 3	Returned for visit 4	Completed visit 5/ Final Examination	Date of withdrawal	Study Day*	Premature termination: Main reason for study termination	Premature termination: If other, please specify:	General comment
Group 3 (PP)	580	yes	no	no	yes	07NOV2006	33	adverse event, making it unsafe for the subject to continue		DUE TO MEETING NOTES FROM 07.03.07 THE "DATE OF WITHDRAWAL" IS THE DATE OF THE LAST "FACE-TO-FACE CONTACT" WITH THE SUBJECT. NEVERTHELESS THERE HAS TO BE A FU-VISIT FOR SAFETY.
	613	no	no	no	no	09OCT2006	0	withdrawal of consent		SUBJECT WAS ASKED TO COME TO AN FOLLOW UP EXAMINATION, BUT SUBJECT DOES NOT WANT ANY FURTHER EXAMINATION
	661	yes	no	no	no	30OCT2006	12	withdrawal of consent		SUBJECT MOVED TO HAMBURG AND THEREFORE CANNOT PARTICIPATE ANY MORE.
	687	yes	no	no	yes	20DEC2006	61	subject unwilling/unable to comply with study requirements		
	782	yes	no	no	no	11DEC2006	31	subject unwilling/unable to comply with study requirements		WITHDRAWAL OF CONSENT AND INCOMPLIANCE TO PERFORM VISIT 5 AND FOLLOW-UP. SUBJECT IS HEALTHY AND DOING WELL.

*: Date of premature termination - Date of visit 1

16.2.2. Protocol deviations

Original CRF entries in capital letters

SUBJECT.-NO.	SCREENING NO.	PROTOCOL DEVIATION	DETAILS, FURTHER DESCRIPTION OR SPECIFICATION	Relevant for exclusion of subject from PP population?
		16.2.2.1. Known violations of Inclusion/Exclusion Criteria (i.e. documented in the e-CRF by ticking relevant criterion)<br>*- sorted by subject number -*		
006	14	Inclusion criteria no. 9 not fulfilled (Hemoglobin >= LLN) not fulfilled)	11.9 g/dl, female<br>Waiver granted by sponsor	No
023	50	Inclusion criteria no. 2 not fulfilled (Women must have a negative serum pregnancy test at screening and a negative urine or serum pregnancy test within 24 hours prior to vaccination)	Waiver granted by sponsor<br>(URINE PREGNANCY TEST BEFORE VACCINATION WAS NEGATIVE.)	No
053	111	Inclusion criteria no. 2 not fulfilled (Women must have a negative serum pregnancy test at screening and a negative urine or serum pregnancy test within 24 hours prior to vaccination)	Waiver granted by sponsor<br>(URINE PREGNANCY TEST BEFORE VACCINATION WAS NEGATIVE.)	No
134	222	Inclusion criteria no. 9 not fulfilled (Hemoglobin >= LLN) not fulfilled)	11.4 g/dl, female<br>Waiver granted by sponsor	No
146	376	Inclusion criteria no. 9 not fulfilled (Hemoglobin >= LLN) not fulfilled)	11.9 g/dl; female<br>Waiver granted by sponsor	No
151	287	Inclusion criteria no. 9 not fulfilled (Hemoglobin >= LLN) not fulfilled)	12.9 g/dl, male<br>Waiver granted by sponsor	No
186	321	Inclusion criteria no. 9 not fulfilled (Hemoglobin >= LLN) not fulfilled)	11.5 g/dl, female<br>Waiver granted by sponsor	No
205	394	Inclusion criteria no. 9 not fulfilled (Hemoglobin >= LLN) not fulfilled)	11.9 g/dl, female<br>Waiver granted by sponsor	No
229	413	Inclusion criteria no. 9 not fulfilled (Hemoglobin >= LLN) not	11.9 g/dl, female	No

SUBJECT.-NO.	SCREENING NO.	PROTOCOL DEVIATION	DETAILS, FURTHER DESCRIPTION OR SPECIFICATION	Relevant for exclusion of subject from PP population?
		fulfilled)	Waiver granted by sponsor	
265	383	Inclusion criteria no. 9 not fulfilled (Hemoglobin >= LLN) not fulfilled)	11.4 g/dl, female<br>Waiver granted by sponsor	No
291	440	Inclusion criteria no. 9 not fulfilled (Hemoglobin >= LLN) not fulfilled)	10.9 g/dl, female<br>Waiver granted by sponsor	No
296	423	Inclusion criteria no. 6 not fulfilled (White blood cells >= 2500/mm³ and <= 11,000/mm³)	11.8 10^9/l, 11.9 10^9/l<br>Waiver granted by sponsor	No
303	492	Inclusion criteria no. 9 not fulfilled (Hemoglobin >= LLN) not fulfilled)	11.0 g/dl, female<br>Waiver granted by sponsor	No
308	654	Inclusion criteria no. 9 not fulfilled (Hemoglobin >= LLN) not fulfilled)	11.9 g/dl, female<br>Waiver granted by sponsor	No
311	548	Inclusion criteria no. 9 not fulfilled (Hemoglobin >= LLN) not fulfilled)	11.6 g/dl, female<br>Waiver granted by sponsor	No
326	520	Inclusion criteria no. 9 not fulfilled (Hemoglobin >= LLN) not fulfilled)	12.9 g/dl, male<br>Waiver granted by sponsor	No
339	539	Inclusion criteria no. 2 not fulfilled (Women must have a negative serum pregnancy test at screening and a negative urine or serum pregnancy test within 24 hours prior to vaccination)	Waiver granted by sponsor<br>(URINE PREGNANCY TEST BEFORE VACCINATION WAS NEGATIVE.)	No
347	555	Inclusion criteria no. 9 not fulfilled (Hemoglobin >= LLN) not fulfilled)	11.0 g/dl, female<br>Waiver granted by sponsor	No
353	575	Inclusion criteria no. 9 not fulfilled (Hemoglobin >= LLN) not fulfilled)	11.6 g/dl, female<br>Waiver granted by sponsor	No
358	561	Inclusion criteria no. 9 not fulfilled (Hemoglobin >= LLN) not fulfilled)	10.2 g/dl, female<br>Waiver granted by sponsor	No
456	713	Inclusion criteria no. 9 not fulfilled (Hemoglobin >= LLN) not fulfilled)	10.7 g/dl, female<br>Waiver granted by sponsor	No
469	828	Inclusion criteria no. 9 not fulfilled (Hemoglobin >= LLN) not fulfilled)	12.7 g/dl, male<br>Waiver granted by sponsor	No
506	783	Inclusion criteria no. 9 not fulfilled (Hemoglobin >= LLN) not fulfilled)	11.9 g/dl, female<br>Waiver granted by sponsor	No
613	1042	Inclusion criteria no. 9 not fulfilled (Hemoglobin >= LLN) not fulfilled)	11.8 g/dl, female	No

SUBJECT.-NO.	SCREENING NO.	PROTOCOL DEVIATION	DETAILS, FURTHER DESCRIPTION OR SPECIFICATION	Relevant for exclusion of subject from PP population?
			Waiver granted by sponsor	
623	1025	Inclusion criteria no. 9 not fulfilled (Hemoglobin >= LLN) not fulfilled)	11.5 g/dl, female<br>Waiver granted by sponsor	No
626	1060	Inclusion criteria no. 9 not fulfilled (Hemoglobin >= LLN) not fulfilled)	11.6 g/dl, female<br>Waiver granted by sponsor	No
661	1082	Inclusion criteria no. 9 not fulfilled (Hemoglobin >= LLN) not fulfilled)	11.9 g/dl, female<br>Waiver granted by sponsor	No
744	1088	Inclusion criteria no. 9 not fulfilled (Hemoglobin >= LLN))	11.9 g/dl, female<br>Waiver granted by sponsor	No
228	430	Exclusion criteria no. 12 not fulfilled (Ten percent or greater risk of developing a myocardial infarction or coronary death within the next 10 years using the National Cholesterol Education Program's risk assessment tool. NOTE: This criterion applies only to volunteers 20 years of age and older.	Waiver granted by sponsor	No
		16.2.2.2. Violations of inclusion/exclusion criteria revealed by DM		
83	117	Absolute neutrophil count (ANC) < 1000/mm3	Result: 0.9x10E3/µl;<br>Clinical significance judged by investigator: 1=Outside the reference range, but without clinical relevance	No
158	291	Hemoglobin < LLN (f: 11.9, m: 13)	11.7 g/dl, female (NCS)	No
186	321	Hemoglobin < LLN (f: 11.9, m: 13)	11.5 g/dl, female (NCS)<br>Waiver granted by sponsor	No
265	383	Hemoglobin < LLN (f: 11.9, m: 13)	11.4 g/dl, female (NCS)<br>Waiver granted by sponsor<br>10.8 g/dl, female (NCS)<br>Waiver granted by sponsor	No
303	492	Hemoglobin < LLN (f: 11.9, m: 13)	11 g/dl, female (NCS)	No

SUBJECT.-NO.	SCREENING NO.	PROTOCOL DEVIATION	DETAILS, FURTHER DESCRIPTION OR SPECIFICATION	Relevant for exclusion of subject from PP population?
			Waiver granted by sponsor	
308	654	Hemoglobin < LLN (f: 11.9, m: 13)	11.9 g/dl, male (NCS)<br>Waiver granted by sponsor	No
353	575	Hemoglobin < LLN (f: 11.9, m: 13)	10.6 g/dl, female (NCS)<br>Waiver granted by sponsor	No
456	713	Hemoglobin < LLN (f: 11.9, m: 13)	10.7 g/dl, female (NCS)<br>Waiver granted by sponsor	No
464	764	Hemoglobin < LLN (f: 11.9, m: 13)	12.8 g/dl, male (NCS)	No
469	828	Hemoglobin < LLN (f: 11.9, m: 13)	12.7 g/dl, male (NCS)<br>Waiver granted by sponsor	No
613	1042	Hemoglobin < LLN (f: 11.9, m: 13)	11.8 g/dl, female (NCS)<br>Waiver granted by sponsor	No
623	1025	Hemoglobin < LLN (f: 11.9, m: 13)	11.5 g/dl, female (NCS)<br>Waiver granted by sponsor	No
626	1060	Hemoglobin < LLN (f: 11.9, m: 13)	11.6 g/dl, female (NCS)<br>Waiver granted by sponsor	No
134	222	Hemoglobin < LLN (f: 11.9, m: 13)	11.4 g/dl, female (NCS)<br>Waiver granted by sponsor	No
146	376	Hemoglobin < LLN (f: 11.9, m: 13)	11.4 g/dl, female (NCS)<br>Waiver granted by sponsor	No
151	287	Hemoglobin < LLN (f: 11.9, m: 13)	12.9 g/dl, male (NCS)<br>Waiver granted by sponsor	No
291	440	Hemoglobin < LLN (f: 11.9, m: 13)	10.9 g/dl, female (NCS)<br>Waiver granted by sponsor	No
311	548	Hemoglobin < LLN (f: 11.9, m: 13)	11.6 g/dl, female (NCS)<br>Waiver granted by sponsor	No
326	520	Hemoglobin < LLN (f: 11.9, m: 13)	12.9 g/dl, male (NCS)<br>Waiver granted by sponsor	No
347	555	Hemoglobin < LLN (f: 11.9, m: 13)	11 g/dl, female (NCS)<br>Waiver granted by sponsor	No
358	561	Hemoglobin < LLN (f: 11.9, m: 13)	10.2 g/dl, female (NCS)<br>Waiver granted by sponsor	No
63	120	Platelets outside 100 – 440 10E3/µl	460 (normal range: 140-450) (NCS)	No
		16.2.2.3. Date of informed consent is		

SUBJECT.-NO.	SCREENING NO.	PROTOCOL DEVIATION	DETAILS, FURTHER DESCRIPTION OR SPECIFICATION	Relevant for exclusion of subject from PP population?
		after date of screening<br>*- sorted by subject number -*		
613	1042	Page 8 and 9 of IC not dated by subject (NTF. No. 10)		No
665	1067	Page 8 and 9 of IC not dated by subject (NTF. No. 18)		No
050	098	Informed consent version 5.0: signature and date on page 11 (data protection) missing (NTF. No. 12)		No
		16.2.2.4. Note to files (indicating a PV)<br>*- sorted by subject number -*		
594	1009	False classification into Group 1-3	SUBJECT SHOWED US HER IMMUNISATION-LICENCES TODAY! THERE WAS A VACCINATION FOR SMALLPOX AT 14.07.1981 AND 21.07.1981. WE WILL CONTINUE VISITS FOR SAFETY. CASE HAS TO BE DROPPED OUT.<br>SUBJECT WAS IN WRONG GROUP. AT FIRST SUBJECT WAS SURE AND STATED THERE WAS NO SMALL POX VACCINATION. LATER SUBJECT FOUND AND PROVIDED A DOCUMENT THAT THERE WAS A VACCINATION AGAINST SMALL POX IN 1981.	Yes
115	254	Possible false classification into Group 4	UNCLEAR VACCINATION STATUS; AT DATE OF SCREENING IT WAS NOT KNOWN THAT BCG-VACCINATION COULD ALSO BE THE REASON FOR THE VACCINATION SCAR. SUBJECT'S MOTHER THINKS SHE HAD NO SMALL POX VACCINATION	Yes
043	75	False classification into Group 4	SUBJECT HAS NO SMALLPOX VACCINATION BUT HAS A BCG VACCINATION. WAS INCLUDED BY MISTAKE IN GROUP 4. VACCINATION CARD WAS SENT ON 16.08.06. DOCUMENTED BCG VACCINATION ON 20.02.84.	Yes

SUBJECT.-NO.	SCREENING NO.	PROTOCOL DEVIATION	DETAILS, FURTHER DESCRIPTION OR SPECIFICATION	Relevant for exclusion of subject from PP population?
050	98	False classification into Group 4	SCARS ON LEFT UPPER ARM FROM DOCUMENTED BCG-VACCINATION (28.08.1979). SUBJECT INCLUDED IN GROUP 4 BY MISTAKE. VACCINATION CARD SEEN ON 28.07.2006.	Yes
061	131	False classification into Group 4	ONE SCAR, DOCUMENTED BCG VACCINATION ON 18.12.1981. ON VACCINATION DATE NO MORE SMALLPOX VACCINATIONS IN GDR. INCLUDED TO GROUP IV BY MISTAKE	Yes
089	195	False classification into Group 4	Vaccination scar is from BCG vaccination (1980)	Yes
		16.2.2.5. Violation against visit windows		
		16.2.2.5.1. Days between V1 and Screening (<-28 d)<br>*- sorted by days between visit*		
136	165	Days between V1 and Screening	-35	No
641	933	Days between V1 and Screening	-33	No
762	1068	Days between V1 and Screening	-33	No
140	185	Days between V1 and Screening	-32	No
736	1041	Days between V1 and Screening	-32	No
763	1078	Days between V1 and Screening	-32	No
529	756	Days between V1 and Screening	-31	No
603	1017	Days between V1 and Screening	-31	No
637	908	Days between V1 and Screening	-31	No
797	1130	Days between V1 and Screening	-31	No
58	126	Days between V1 and Screening	-31	No
78	133	Days between V1 and Screening	-31	No
91	132	Days between V1 and Screening	-31	No
163	304	Days between V1 and Screening	-30	No
164	303	Days between V1 and Screening	-30	No
561	848	Days between V1 and Screening	-30	No
785	1113	Days between V1 and Screening	-30	No
135	173	Days between V1 and Screening	-29	No
141	181	Days between V1 and Screening	-29	No

SUBJECT.-NO.	SCREENING NO.	PROTOCOL DEVIATION	DETAILS, FURTHER DESCRIPTION OR SPECIFICATION	Relevant for exclusion of subject from PP population?
152	215	Days between V1 and Screening	-29	No
236	353	Days between V1 and Screening	-29	No
306	574	Days between V1 and Screening	-29	No
309	427	Days between V1 and Screening	-29	No
425	664	Days between V1 and Screening	-29	No
517	855	Days between V1 and Screening	-29	No
542	875	Days between V1 and Screening	-29	No
552	861	Days between V1 and Screening	-29	No
598	865	Days between V1 and Screening	-29	No
734	1120	Days between V1 and Screening	-29	No
751	1084	Days between V1 and Screening	-29	No
776	1125	Days between V1 and Screening	-29	No
792	1114	Days between V1 and Screening	-29	No
97	183	Days between V1 and Screening	-29	No
224	289	Days between V1 and Screening	-29	No
272	372	Days between V1 and Screening	-29	No
315	426	Days between V1 and Screening	-29	No
344	542	Days between V1 and Screening	-29	No
		16.2.2.5.2. Days between V2 and V1 (outside 10-15 d)<br>*- sorted by days between visit*	Time window of 9-18 d considered as not relevant	
357	606	Days between V2 and V1	7	Yes
372	644	Days between V2 and V1	9	No
493	801	Days between V2 and V1	9	No
633	930	Days between V2 and V1	9	No
115	254	Days between V2 and V1	9	No
088	174	Days between V2 and V1	16	No
130	253	Days between V2 and V1	16	No
301	582	Days between V2 and V1	16	No
494	791	Days between V2 and V1	16	No
501	858	Days between V2 and V1	16	No
556	953	Days between V2 and V1	16	No
605	972	Days between V2 and V1	16	No
641	933	Days between V2 and V1	16	No
662	1119	Days between V2 and V1	16	No
051	107	Days between V2 and V1	16	No
058	126	Days between V2 and V1	16	No

SUBJECT.-NO.	SCREENING NO.	PROTOCOL DEVIATION	DETAILS, FURTHER DESCRIPTION OR SPECIFICATION	Relevant for exclusion of subject from PP population?
524	771	Days between V2 and V1	16	No
082	199	Days between V2 and V1	17	No
254	424	Days between V2 and V1	17	No
530	883	Days between V2 and V1	17	No
075	162	Days between V2 and V1	17	No
081	91	Days between V2 and V1	17	No
108	217	Days between V2 and V1	17	No
123	362	Days between V2 and V1	17	No
315	426	Days between V2 and V1	17	No
381	651	Days between V2 and V1	17	No
422	735	Days between V2 and V1	17	No
147	340	Days between V2 and V1	18	No
477	812	Days between V2 and V1	18	No
001	2	Days between V2 and V1	18	No
420	703	Days between V2 and V1	18	No
567	962	Days between V2 and V1	19	Yes
669	1074	Days between V2 and V1	19	Yes
512	887	Days between V2 and V1	22	Yes
724	1192	Days between V2 and V1	30	Yes
782	1181	Days between V2 and V1	31	Yes
		16.2.2.5.3. Days between V3 and V1 (outside 28-35 d)<br>*- sorted by days between visit*	Time window of 25-38 d considered as not relevant	
419	732	Days between V3 and V1	24	Yes
462	743	Days between V3 and V1	26	No
297	551	Days between V3 and V1	27	No
049	97	Days between V3 and V1	27	No
092	186	Days between V3 and V1	27	No
269	499	Days between V3 and V1	27	No
547	922	Days between V3 and V1	36	No
557	938	Days between V3 and V1	36	No
657	1103	Days between V3 and V1	36	No
683	1052	Days between V3 and V1	38	No
751	1084	Days between V3 and V1	38	No
685	1001	Days between V3 and V1	40	Yes
686	1145	Days between V3 and V1	40	Yes
405	694	Days between V3 and V1	41	Yes

SUBJECT.-NO.	SCREENING NO.	PROTOCOL DEVIATION	DETAILS, FURTHER DESCRIPTION OR SPECIFICATION	Relevant for exclusion of subject from PP population?
502	857	Days between V3 and V1	41	Yes
755	1261	Days between V3 and V1	42	Yes
752	1196	Days between V3 and V1	43	Yes
562	964	Days between V3 and V1	44	Yes
368	649	Days between V3 and V1	52	Yes
		16.2.2.5.4. Days between V4 and V3 (outside 10-15 d)<br>*- sorted by days between visit*	Time window of 9-18 d considered as not relevant	
724	1192	Days between V4 and V3	9	No
730	1207	Days between V4 and V3	9	No
795	1304	Days between V4 and V3	9	No
149	344	Days between V4 and V3	16	No
428	570	Days between V4 and V3	16	No
437	615	Days between V4 and V3	16	No
503	830	Days between V4 and V3	16	No
505	823	Days between V4 and V3	16	No
548	931	Days between V4 and V3	16	No
585	982	Days between V4 and V3	16	No
651	1011	Days between V4 and V3	16	No
610	959	Days between V4 and V3	17	No
574	889	Days between V4 and V3	18	No
513	832	Days between V4 and V3	19	Yes
357	606	Days between V4 and V3	20	Yes
486	803	Days between V4 and V3	21	Yes
527	903	Days between V4 and V3	22	Yes
462	743	Days between V4 and V3	26	Yes
533	892	Days between V4 and V3	28	Yes
534	876	Days between V4 and V3	34	Yes
		16.2.2.5.5. Days between V5 and V3<br>*- sorted by days between visit*	Time window of 25-38 d considered as not relevant	
067	155	Days between V5 and V3	25	No
474	835	Days between V5 and V3	26	No
080	152	Days between V5 and V3	27	No
125	309	Days between V5 and V3	27	No
128	245	Days between V5 and V3	27	No

SUBJECT.-NO.	SCREENING NO.	PROTOCOL DEVIATION	DETAILS, FURTHER DESCRIPTION OR SPECIFICATION	Relevant for exclusion of subject from PP population?
175	357	Days between V5 and V3	27	No
253	432	Days between V5 and V3	27	No
348	550	Days between V5 and V3	27	No
450	748	Days between V5 and V3	27	No
478	838	Days between V5 and V3	27	No
510	899	Days between V5 and V3	27	No
518	870	Days between V5 and V3	27	No
535	911	Days between V5 and V3	27	No
570	948	Days between V5 and V3	27	No
589	1003	Days between V5 and V3	27	No
604	1021	Days between V5 and V3	27	No
646	1031	Days between V5 and V3	27	No
696	1156	Days between V5 and V3	27	No
462	743	Days between V5 and V3	36	No
559	895	Days between V5 and V3	36	No
658	1035	Days between V5 and V3	36	No
756	1239	Days between V5 and V3	36	No
187	349	Days between V5 and V3	38	No
511	869	Days between V5 and V3	38	No
596	980	Days between V5 and V3	38	No
101	211	Days between V5 and V3	39	Yes
464	764	Days between V5 and V3	39	Yes
		16.2.2.6. Prohibited medical history<br>*- sorted by subject number -*		
		**No deviations occurred.**		
		16.2.2.7. Prohibited previous/ concomitant treatments<br>*- sorted by subject number -*		
442	688	Exclusion criterion 19 TRIAMGALEN	ECZEMA BOTH HANDS (17SEP2006-Ongoing, prn); Eczema reported in adverse events section on 07.09.2006, on 09.09.2006 and from 17.09.2006 to 19.09.2006	No
120	248	Exclusion criterion 19 TRIAMGALEN	ECZEMA RIGHT HAND; 18JUL2006-	No

SUBJECT.-NO.	SCREENING NO.	PROTOCOL DEVIATION	DETAILS, FURTHER DESCRIPTION OR SPECIFICATION	Relevant for exclusion of subject from PP population?
			23JUL2006 (prn)	
342	596	Exclusion criterion 19<br>Tetanol Pur	Vaccination on 26.09.2006 (11 days after visit 3, 2nd vaccination), because of laceration at eyebrow	No
		16.2.2.8. Other protocol deviations from the e-CRF comments<br>*- sorted by subject number-*		
148	293	No urine pregnancy test done at V5	WAS NOT DONE BECAUSE DURING THIS VISIT ONLY ASSESSMENTS REQUIERED AT VISIT 4 HAVE BEEN PERFORMED. SUBJ. HAD MENSTRUATION AFTER 30.08.06	No
305	522	ON 27.08.06 AT DAY 26 OF CYCLE RUPTURE OF CONDOME WITHOUT CONTRACEPTIONAL MEDICATION. "PILL AFTER" WITHIN 24H. CASE DISCUSSED IN BN-MEETING. CAN BE VACCINATED AFTER B-HCG URINE TEST.	Pregnany test negative	No
797	1130	SUBJECT LOST DIARY (2ND VACCINATION). SHE TRIED TO REMEBER THE VALUES AND ENTERED "GUESSED" DATA INTO A NEW DIARY.		No
344	542	ECG ASSESSED BY CARDIOLOGIST IN COMB. WITH ERGOMETRY. VACC. PLANNED ON 22.08.06 DUE TO TETANUS-DIPHTERIA VACC. ON 08.08.06. TIME EXCEEDING OF 1 DAY FROM SCR. WINDOW ACCEPTABLE ACCORDING TO DR. VIRGIN	V1 on 22AUG2006	No
		16.2.2.9. Deviations during vaccination<br>*- sorted by subject number-*		
		**No deviations occurred.**		
		16.2.2.10. Not evaluable antibody sample detected at BN analytical laboratory:<br>*- sorted by subject number-*		

SUBJECT.-<br>NO.	SCREENING NO.	PROTOCOL DEVIATION	DETAILS, FURTHER DESCRIPTION OR SPECIFICATION	Relevant for exclusion of subject from PP population?
Group 1-3		For all samples of visit 4 result obtained (N=525)		N/A
037 (S0049)<br>Group 4 (M-)		Sample taken at V2, however never received at BN, i.e. no result available		No

16.2.3. Subjects excluded from the analysis

16.2.3.1. Subjects excluded from the safety analysis

8 subjects have been randomised, but did not receive any dose of study vaccine. These subjects will be treated as screening failures.

SUBJECT.-NO.	SCREENING NO.	REASON FOR EXCLUSION FROM EFFICACY ANALYSIS	CLASSIFICATION
040	43	SUBJECT WAS NO MORE ELIGIBLE AFTER RE-CHECK OF CRITERIA	SCREENING FAILURE
025	55	SUBJECT WAS NO MORE ELIGIBLE AFTER RE-CHECK OF CRITERIA	SCREENING FAILURE
608	1051	SUBJECT WAS NO MORE ELIGIBLE AFTER RE-CHECK OF CRITERIA	SCREENING FAILURE
331	563	SUBJECT WAS NO MORE ELIGIBLE AFTER RE-CHECK OF CRITERIA	SCREENING FAILURE
558	868	SUBJECT WAS NO MORE ELIGIBLE AFTER RE-CHECK OF CRITERIA	SCREENING FAILURE
155	365	SUBJECT WAS NO MORE ELIGIBLE AFTER RE-CHECK OF CRITERIA	SCREENING FAILURE
258	408	SUBJECT WAS NO MORE ELIGIBLE AFTER RE-CHECK OF CRITERIA	SCREENING FAILURE
264	382	SUBJECT WAS NO MORE ELIGIBLE AFTER RE-CHECK OF CRITERIA	SCREENING FAILURE

16.2.3.2. Subjects excluded from per protocol analysis

The following **50** subjects will be excluded from the Per-Protocol analysis.

SUBJECT.-NO.	SCREENING NO.	REASON FOR EXCLUSION FROM EFFICACY ANALYSIS	CLASSIFICATION
043	75	False classification into Group 4	ONLY SAFETY AND FA
050	98	False classification into Group 4	ONLY SAFETY AND FA
061	131	False classification into Group 4	ONLY SAFETY AND FA
089	195	False classification into Group 4	ONLY SAFETY AND FA
101	211	Days between V5 and V3 = 39	ONLY SAFETY AND FA
105	225	SUBJECT DROPPED OUT BEFORE V5 (GROUP 1-3).	ONLY SAFETY AND FA
115	254	Possible false classification into Group 4	ONLY SAFETY AND FA
148	293	SUBJECT DROPPED OUT BEFORE V5 (GROUP 1-3).	ONLY SAFETY AND FA

SUBJECT.-NO.	SCREENING NO.	REASON FOR EXCLUSION FROM EFFICACY ANALYSIS	CLASSIFICATION
268	518	SUBJECT DROPPED OUT BEFORE V5 (GROUP 1-3).	ONLY SAFETY AND FA
284	487	SUBJECT DROPPED OUT BEFORE V5 (GROUP 1-3).	ONLY SAFETY AND FA
306	574	SUBJECT DROPPED OUT BEFORE V5 (GROUP 1-3).	ONLY SAFETY AND FA
328	560	SUBJECT DROPPED OUT BEFORE V5 (GROUP 1-3).	ONLY SAFETY AND FA
357	606	Days between V2 and V1 = 7<br>Days between V4 and V3 = 20	ONLY SAFETY AND FA
368	649	Days between V3 and V1 = 52	ONLY SAFETY AND FA
385	598	SUBJECT DROPPED OUT BEFORE V5 (GROUP 1-3).	ONLY SAFETY AND FA
399	699	SUBJECT DROPPED OUT BEFORE V5 (GROUP 1-3).	ONLY SAFETY AND FA
400	587	SUBJECT DROPPED OUT BEFORE V5 (GROUP 1-3).	ONLY SAFETY AND FA
405	694	Days between V3 and V1 = 41	ONLY SAFETY AND FA
419	732	Days between V3 and V1 = 24	ONLY SAFETY AND FA
433	766	SUBJECT DROPPED OUT BEFORE V5 (GROUP 1-3).	ONLY SAFETY AND FA
462	743	Days between V4 and V3 = 26	ONLY SAFETY AND FA
464	764	Days between V5 and V3 = 39	ONLY SAFETY AND FA
486	803	Days between V4 and V3 = 21	ONLY SAFETY AND FA
502	857	Days between V3 and V1 = 41	ONLY SAFETY AND FA
512	887	Days between V2 and V1 = 22	ONLY SAFETY AND FA
513	832	Days between V4 and V3 = 19	ONLY SAFETY AND FA
527	903	Days between V4 and V3 = 22	ONLY SAFETY AND FA
533	892	Days between V4 and V3 = 28	ONLY SAFETY AND FA
534	876	Days between V4 and V3 = 34	ONLY SAFETY AND FA
536	926	SUBJECT DROPPED OUT BEFORE V5 (GROUP 1-3).	ONLY SAFETY AND FA
562	964	Days between V3 and V1 = 44	ONLY SAFETY AND FA
567	962	Days between V2 and V1 = 19	ONLY SAFETY AND FA
580	979	SUBJECT DROPPED OUT BEFORE V5 (GROUP 1-3).	ONLY SAFETY AND FA
594	1009	False classification into Group 1-3	ONLY SAFETY AND FA
613	1042	SUBJECT DROPPED OUT BEFORE V5 (GROUP 1-3).	ONLY SAFETY AND FA
643	1081	SUBJECT DROPPED OUT BEFORE V5 (GROUP 1-3).	ONLY SAFETY AND FA
661	1082	SUBJECT DROPPED OUT BEFORE V5 (GROUP 1-3).	ONLY SAFETY AND FA
669	1074	Days between V2 and V1 = 19	ONLY SAFETY AND FA
670	1090	SUBJECT DROPPED OUT BEFORE V5 (GROUP 1-3).	ONLY SAFETY AND FA
685	1001	Days between V3 and V1 = 40	ONLY SAFETY AND FA
686	1145	Days between V3 and V1 = 40	ONLY SAFETY AND FA
687	1164	SUBJECT DROPPED OUT BEFORE V5 (GROUP 1-3).	ONLY SAFETY AND FA
699	1136	SUBJECT DROPPED OUT BEFORE V5 (GROUP 1-3).	ONLY SAFETY AND FA
724	1192	Days between V2 and V1 = 30	ONLY SAFETY AND FA
734	1120	SUBJECT DROPPED OUT BEFORE V5 (GROUP 1-3).	ONLY SAFETY AND FA

SUBJECT.-NO.	SCREENING NO.	REASON FOR EXCLUSION FROM EFFICACY ANALYSIS	CLASSIFICATION
738	1225	SUBJECT DROPPED OUT BEFORE V5 (GROUP 1-3).	ONLY SAFETY AND FA
752	1196	Days between V3 and V1 = 43	ONLY SAFETY AND FA
755	1261	Days between V3 and V1 = 42	ONLY SAFETY AND FA
781	1269	SUBJECT DROPPED OUT BEFORE V5 (GROUP 1-3).	ONLY SAFETY AND FA
782	1181	SUBJECT DROPPED OUT BEFORE V5 (GROUP 1-3).<br>Days between V2 and V1 = 31	ONLY SAFETY AND FA

16.2.4. Demographic data

16.2.4.1. Demographic data

Group	Subj	Screening number	Centre	Date of birth	Written informed consent was obtained on:	Age [years]	Gender	Child-bearing potential [1]	Height [cm]	Weight [kg]	Body mass index [kg/ m²]	Ethnic group	Ethnic group: please specify other
Group 1 (MM)	070	142	1	20DEC1977	22MAY2006	28	female	B	168	54.2	19	caucasian	
	080	152	1	27SEP1982	23MAY2006	23	male	.	175	83.5	27	caucasian	
	084	149	1	05FEB1985	23MAY2006	21	female	B	179	64.4	20	caucasian	
	088	174	1	21OCT1983	30MAY2006	22	female	B	157	48.4	20	caucasian	
	093	201	1	06MAR1971	02JUN2006	35	male	.	174	63.3	21	caucasian	
	096	203	1	02JUL1979	02JUN2006	26	male	.	181	85.5	26	caucasian	
	102	216	1	18JUN1986	06JUN2006	19	male	.	185	101.4	30	caucasian	
	103	224	1	05JAN1984	07JUN2006	22	male	.	181	82.1	25	caucasian	
	107	227	1	27JUN1977	08JUN2006	28	female	B	185	112.9	33	caucasian	
	129	272	1	16JUN1985	14JUN2006	20	female	B	175	67.4	22	caucasian	
	137	283	1	10SEP1979	19JUN2006	26	female	B	161	53.7	21	caucasian	
	140	185	1	20MAR1978	01JUN2006	28	male	.	186	80.5	23	caucasian	
	143	313	1	30OCT1974	21JUN2006	31	male	.	186	92.3	27	caucasian	
	144	320	1	16MAR1970	22JUN2006	36	male	.	179	86.0	27	caucasian	
	148	293	1	30NOV1978	20JUN2006	27	female	B	176	56.9	18	caucasian	
	154	367	1	10APR1977	28JUN2006	29	female	B	161	58.5	23	caucasian	
	156	360	1	24MAY1979	27JUN2006	27	female	B	175	72.9	24	caucasian	
	161	229	1	06MAY1974	08JUN2006	32	male	.	186	93.1	27	caucasian	
	176	377	1	31DEC1980	29JUN2006	25	male	.	166	58.6	21	arabic	
	180	380	1	12MAR1956	29JUN2006	50	female	B	168	57.6	20	caucasian	
	182	359	1	05AUG1982	27JUN2006	23	female	B	162	62.2	24	caucasian	
	189	312	1	07NOV1984	21JUN2006	21	male	.	177	72.9	23	caucasian	
	196	384	1	27SEP1980	29JUN2006	25	female	B	158	68.8	28	caucasian	
	199	366	1	27DEC1980	28JUN2006	25	female	B	165	53.0	19	caucasian	
	204	398	1	04APR1983	03JUL2006	23	female	B	167	67.6	24	caucasian	
	205	394	1	31MAR1976	03JUL2006	30	female	B	158	52.2	21	caucasian	
	215	411	1	08JUL1982	04JUL2006	23	female	B	168	69.7	25	caucasian	
	216	417	1	13AUG1980	05JUL2006	25	female	B	163	54.0	20	caucasian	

[1] Child-bearing potential: A: surgically sterile or post-menopausal B: of child-bearing potential and using appropriate birth control

Demographic data

Group	Subj	Screening number	Centre	Date of birth	Written informed consent was obtained on:	Age [years]	Gender	Child-bearing potential [1]	Height [cm]	Weight [kg]	Body mass index [kg/m²]	Ethnic group	Ethnic group: please specify other
Group 1 (MM)	218	403	1	02APR1986	04JUL2006	20	female	B	166	56.6	21	caucasian	
	225	414	1	26FEB1974	05JUL2006	32	female	B	171	61.3	21	caucasian	
	226	341	1	16NOV1977	26JUN2006	28	female	B	173	56.3	19	caucasian	
	227	381	1	05APR1979	29JUN2006	27	male	.	188	68.1	19	caucasian	
	236	353	1	15NOV1986	27JUN2006	19	female	B	177	54.9	18	caucasian	
	237	437	1	01JUL1981	06JUN2006	24	female	B	160	45.1	18	caucasian	
	248	445	1	30JAN1984	10JUL2006	22	male	.	180	77.9	24	caucasian	
	254	424	1	25FEB1981	06JUL2006	25	male	.	181	85.9	26	caucasian	
	255	463	1	10MAY1978	12JUL2006	28	male	.	191	82.6	23	caucasian	
	256	472	1	29JUN1982	13JUL2006	24	female	B	173	56.5	19	caucasian	
	268	518	1	28NOV1981	20JUL2006	24	male	.	177	69.6	22	caucasian	
	274	404	1	02FEB1984	04JUL2006	22	male	.	177	97.1	31	caucasian	
	281	532	1	25FEB1983	21JUL2006	23	male	.	188	83.2	24	caucasian	
	283	507	1	28MAR1985	19JUL2006	21	female	B	167	64.0	23	caucasian	
	287	519	1	10NOV1977	20JUL2006	28	female	B	160	63.0	25	caucasian	
	297	551	1	30SEP1986	25JUL2006	19	female	B	168	59.4	21	caucasian	
	304	482	1	29MAR1965	17JUL2006	41	female	B	164	56.4	21	caucasian	
	306	574	1	29OCT1981	27JUL2006	24	male	.	185	67.0	20	caucasian	
	317	488	1	27NOV1960	17JUL2006	45	male	.	179	89.0	28	caucasian	
	318	538	1	09MAR1981	24JUL2006	25	female	B	171	52.4	18	caucasian	
	323	471	1	30APR1982	08JUN2006	24	female	B	168	52.0	18	caucasian	
	330	611	1	01MAR1968	02AUG2006	38	male	.	181	75.5	23	caucasian	
	334	494	1	01APR1986	18JUL2006	20	female	B	162	122.6	47	caucasian	
	342	596	1	04AUG1981	01AUG2006	24	female	B	161	56.8	22	caucasian	
	353	575	1	04DEC1981	27JUL2006	24	female	B	176	66.5	21	caucasian	
	356	632	1	17MAR1982	04AUG2006	24	male	.	179	71.0	22	caucasian	
	386	648	1	27MAY1977	08AUG2006	29	female	B	160	52.8	21	caucasian	
	398	698	1	24DEC1980	16AUG2006	25	male	.	166	70.7	26	caucasian	

[1] Child-bearing potential: A: surgically sterile or post-menopausal B: of child-bearing potential and using appropriate birth control

Demographic data

Group	Subj	Screening number	Centre	Date of birth	Written informed consent was obtained on:	Age [years]	Gender	Child-bearing potential [1]	Height [cm]	Weight [kg]	Body mass index [kg/ m²]	Ethnic group	Ethnic group: please specify other
Group 1 (MM)	400	587	1	08AUG1980	31JUL2006	25	male	.	182	70.8	21	caucasian	
	415	717	1	21SEP1982	17AUG2006	23	female	B	172	61.4	21	caucasian	
	416	593	1	21JUL1983	01AUG2006	23	male	.	178	65.7	21	caucasian	
	417	671	1	05OCT1964	10AUG2006	41	female	B	160	61.4	24	caucasian	
	427	723	1	29JUN1985	17AUG2006	21	male	.	167	62.0	22	caucasian	
	428	570	1	11MAY1984	27JUL2006	22	male	.	180	88.5	27	caucasian	
	429	762	1	05OCT1979	22AUG2006	26	female	B	159	58.6	23	caucasian	
	432	705	1	21FEB1978	16AUG2006	28	female	B	162	61.3	23	caucasian	
	433	766	1	18DEC1979	22AUG2006	26	female	B	176	79.8	26	caucasian	
	438	638	1	24JAN1979	07AUG2006	27	female	B	175	76.4	25	caucasian	
	441	773	1	03DEC1983	23AUG2006	22	male	.	186	83.0	24	caucasian	
	442	688	1	30SEP1976	14AUG2006	29	female	B	168	75.9	27	caucasian	
	447	806	1	18MAR1975	25AUG2006	31	female	B	176	68.1	22	caucasian	
	448	747	1	11FEB1975	21AUG2006	31	male	.	186	94.8	27	caucasian	
	449	782	1	13JAN1977	07JUL2006	29	female	B	154	55.2	23	caucasian	
	455	687	1	09MAR1978	14AUG2006	28	male	.	183	94.2	28	caucasian	
	457	795	1	03NOV1980	24AUG2006	25	female	B	166	56.8	21	caucasian	
	459	790	1	28JUL1983	24AUG2006	23	female	B	166	62.0	22	caucasian	
	462	743	1	03MAY1987	21AUG2006	19	female	B	170	56.8	20	caucasian	
	471	831	1	18APR1972	31AUG2006	34	female	B	173	68.6	23	caucasian	
	472	822	1	01NOV1983	30AUG2006	22	female	B	174	70.6	23	caucasian	
	473	833	1	07JAN1984	31AUG2006	22	female	B	171	67.2	23	caucasian	
	479	819	1	27MAY1978	29AUG2006	28	female	B	171	70.0	24	caucasian	
	482	804	1	21MAR1972	25AUG2006	34	male	.	195	86.4	23	caucasian	
	483	854	1	21FEB1985	05SEP2006	21	male	.	177	87.2	28	caucasian	
	484	817	1	07NOV1983	29AUG2006	22	male	.	179	63.6	20	caucasian	
	488	843	1	09OCT1983	01SEP2006	22	female	B	165	65.6	24	caucasian	
	489	700	1	05JUL1974	25AUG2006	32	female	B	162	87.4	33	caucasian	

[1]Child-bearing potential: A: surgically sterile or post-menopausal B: of child-bearing potential and using appropriate birth control

Demographic data

Group	Subj	Screening number	Centre	Date of birth	Written informed consent was obtained on:	Age [years]	Gender	Child-bearing potential [1]	Height [cm]	Weight [kg]	Body mass index [kg/m²]	Ethnic group	Ethnic group: please specify other
Group 1 (MM)	495	816	1	12AUG1984	29AUG2006	22	female	B	162	55.3	21	caucasian	
	497	844	1	23OCT1987	01SEP2006	18	male	.	179	69.8	22	caucasian	
	502	857	1	25MAR1977	05SEP2006	29	male	.	184	76.9	23	caucasian	
	505	823	1	16AUG1966	30AUG2006	40	female	A	166	103.9	38	caucasian	
	510	899	1	22NOV1976	11SEP2006	29	female	B	167	83.6	30	caucasian	
	512	887	1	19MAY1987	08SEP2006	19	female	B	171	60.5	21	other	HISPANIC
	518	870	1	01OCT1981	01AUG2006	24	female	B	172	70.6	24	caucasian	
	519	872	1	10AUG1983	07SEP2006	23	male	.	181	86.1	26	caucasian	
	521	802	1	03OCT1976	25AUG2006	29	male	.	190	78.6	22	caucasian	
	525	886	1	05JUN1979	08SEP2006	27	male	.	182	104.3	31	caucasian	
	532	890	1	01JUL1985	08SEP2006	21	female	B	155	51.1	21	caucasian	
	534	876	1	10FEB1982	07SEP2006	24	male	.	178	67.4	21	caucasian	
	537	928	1	26JUN1975	14SEP2006	31	male	.	175	71.0	23	caucasian	
	543	877	1	07NOV1981	07SEP2006	24	female	B	169	64.5	23	caucasian	
	545	918	1	05DEC1978	13SEP2006	27	male	.	174	78.1	26	caucasian	
	555	947	1	26FEB1981	15SEP2006	25	female	B	172	64.2	22	caucasian	
	556	953	1	07APR1982	18SEP2006	24	female	B	162	52.0	20	caucasian	
	557	938	1	31MAY1971	15SEP2006	35	male	.	166	69.3	25	caucasian	
	562	964	1	17AUG1979	18SEP2006	27	male	.	185	95.7	28	caucasian	
	565	965	1	12JUN1982	18SEP2006	24	female	B	170	64.9	22	caucasian	
	567	962	1	05JUL1982	18SEP2006	24	female	B	168	54.0	19	caucasian	
	568	992	1	14FEB1986	22SEP2006	20	male	.	175	83.1	27	caucasian	
	571	907	1	11FEB1981	12SEP2006	25	female	B	166	81.8	30	caucasian	
	572	991	1	08SEP1982	21SEP2006	24	female	B	154	58.8	25	arabic	
	574	889	1	16JAN1984	08SEP2006	22	male	.	181	77.6	24	caucasian	
	582	951	1	13OCT1982	15SEP2006	23	male	.	185	72.8	21	caucasian	
	583	949	1	04DEC1979	15SEP2006	26	male	.	177	71.9	23	caucasian	
	586	990	1	08JUL1980	21SEP2006	26	male	.	193	120.8	32	caucasian	

[1]Child-bearing potential: A: surgically sterile or post-menopausal B: of child-bearing potential and using appropriate birth control

Demographic data

Group	Subj	Screening number	Centre	Date of birth	Written informed consent was obtained on:	Age [years]	Gender	Child-bearing potential [1]	Height [cm]	Weight [kg]	Body mass index [kg/m²]	Ethnic group	Ethnic group: please specify other
Group 1 (MM)	587	1002	1	17FEB1981	22SEP2006	25	male	.	198	97.1	25	caucasian	
	595	1026	1	14FEB1980	27SEP2006	26	male	.	170	70.0	24	caucasian	
	597	1004	1	06DEC1982	25SEP2006	23	female	B	165	70.0	26	caucasian	
	604	1021	1	04JUN1986	26SEP2006	20	female	B	173	71.8	24	caucasian	
	605	972	1	01MAR1981	19SEP2006	25	female	B	162	72.2	28	caucasian	
	607	1049	1	14NOV1981	29SEP2006	24	female	B	167	57.7	21	caucasian	
	609	1018	1	04DEC1981	26SEP2006	24	male	.	173	68.7	23	caucasian	
	610	959	1	13MAY1982	18SEP2006	24	male	.	184	73.7	22	caucasian	
	612	968	1	20APR1988	19SEP2006	18	female	B	181	82.6	25	caucasian	
	616	915	1	26JUN1982	12SEP2006	24	male	.	180	73.1	23	caucasian	
	625	1007	1	02JAN1984	25SEP2006	22	female	B	162	57.7	22	caucasian	
	627	1062	1	13DEC1982	04OCT2006	23	female	B	167	59.8	21	caucasian	
	629	1073	1	27OCT1979	05OCT2006	26	female	B	166	59.2	21	caucasian	
	634	1072	1	30JUL1974	05OCT2006	32	male	.	181	78.6	24	caucasian	
	636	1037	1	06AUG1978	28SEP2006	28	male	.	180	77.2	24	caucasian	
	637	908	1	24MAR1984	12SEP2006	22	male	.	184	77.0	23	caucasian	
	641	933	1	15JAN1985	15SEP2006	21	female	B	168	64.8	23	caucasian	
	643	1081	1	18JAN1980	05OCT2006	26	female	B	162	53.4	20	caucasian	
	646	1031	1	02APR1982	18AUG2006	24	female	B	155	55.4	23	caucasian	
	648	946	1	11FEB1976	15SEP2006	30	male	.	183	75.4	23	caucasian	
	649	961	1	26APR1987	18SEP2006	19	male	.	182	71.1	21	caucasian	
	654	1099	1	25AUG1981	09OCT2006	25	female	B	165	73.7	27	caucasian	
	655	1098	1	16FEB1984	09OCT2006	22	male	.	179	77.0	24	caucasian	
	662	1119	1	28APR1986	07SEP2006	20	male	.	180	67.2	21	caucasian	
	663	1112	1	25NOV1986	11OCT2006	19	male	.	204	91.2	22	caucasian	
	666	1089	1	17MAR1985	06OCT2006	21	female	B	159	103.0	41	caucasian	
	669	1074	1	12APR1982	05OCT2006	24	male	.	181	86.4	26	caucasian	
	670	1090	1	12JAN1980	06OCT2006	26	female	B	177	71.0	23	caucasian	

[1] Child-bearing potential: A: surgically sterile or post-menopausal B: of child-bearing potential and using appropriate birth control

Demographic data

Group	Subj	Screening number	Centre	Date of birth	Written informed consent was obtained on:	Age [years]	Gender	Child-bearing potential [1]	Height [cm]	Weight [kg]	Body mass index [kg/m²]	Ethnic group	Ethnic group: please specify other
Group 1 (MM)	673	1054	1	02FEB1983	02OCT2006	23	female	B	170	56.7	20	other	LATINO
	676	1124	1	18DEC1980	12OCT2006	25	male	.	181	70.3	21	caucasian	
	678	1131	1	09JUN1982	13OCT2006	24	male	.	183	81.1	24	caucasian	
	683	1052	1	15JAN1985	02OCT2006	21	male	.	178	63.8	20	caucasian	
	688	1143	1	17MAY1984	16OCT2006	22	female	B	165	70.4	26	caucasian	
	690	1111	1	11JUL1977	11OCT2006	29	female	B	170	71.7	25	caucasian	
	691	1123	1	11JUN1982	12OCT2006	24	female	B	162	63.6	24	caucasian	
	697	1163	1	11APR1977	17OCT2006	29	male	.	188	88.9	25	caucasian	
	698	1151	1	27JUL1981	16OCT2006	25	male	.	192	96.8	26	caucasian	
	701	1057	1	22NOV1968	23AUG2006	37	female	B	168	68.5	24	caucasian	
	703	1153	1	15AUG1983	16OCT2006	23	male	.	191	86.1	24	caucasian	
	705	1197	1	25OCT1985	20OCT2006	20	female	B	168	51.7	18	caucasian	
	707	1158	1	26SEP1983	17OCT2006	23	male	.	190	69.2	19	caucasian	
	716	1159	1	16APR1981	17OCT2006	25	female	B	164	52.6	20	caucasian	
	719	1190	1	21OCT1983	20OCT2006	22	male	.	183	80.0	24	caucasian	
	721	1178	1	07JUN1985	18OCT2006	21	female	B	170	69.1	24	caucasian	
	725	1194	1	29MAY1982	20OCT2006	24	male	.	178	75.5	24	caucasian	
	729	1128	1	15JAN1977	12OCT2006	29	male	.	191	86.0	24	caucasian	
	730	1207	1	07JUL1983	23OCT2006	23	female	B	182	72.1	22	caucasian	
	733	1229	1	04OCT1977	25OCT2006	29	male	.	190	82.0	23	caucasian	
	734	1120	1	13JUL1988	11OCT2006	18	male	.	189	76.5	21	caucasian	
	735	1233	1	17DEC1984	25OCT2006	21	male	.	183	73.4	22	caucasian	
	742	1191	1	27FEB1984	20OCT2006	22	male	.	198	117.6	30	caucasian	
	743	1226	1	14APR1981	25OCT2006	25	male	.	180	77.8	24	caucasian	
	746	1172	1	03JUL1984	18OCT2006	22	female	B	167	61.8	22	caucasian	
	748	1169	1	04SEP1982	18OCT2006	24	female	B	165	59.6	22	caucasian	
	749	1216	1	07DEC1982	24OCT2006	23	male	.	183	79.3	24	caucasian	
	750	1243	1	19AUG1986	26OCT2006	20	female	B	163	53.0	20	caucasian	

[1] Child-bearing potential: A: surgically sterile or post-menopausal B: of child-bearing potential and using appropriate birth control

Demographic data

Group	Subj	Screening number	Centre	Date of birth	Written informed consent was obtained on:	Age [years]	Gender	Child-bearing potential [1]	Height [cm]	Weight [kg]	Body mass index [kg/m²]	Ethnic group	Ethnic group: please specify other
Group 1 (MM)	758	1179	1	08JUL1988	19OCT2006	18	female	B	176	65.6	21	caucasian	
	765	1222	1	29NOV1983	24OCT2006	22	male	.	187	99.6	28	caucasian	
	768	1250	1	24NOV1972	26OCT2006	33	female	B	168	61.2	22	asian	
	771	1171	1	20FEB1979	18OCT2006	27	male	.	182	68.3	21	caucasian	
	774	1246	1	01JUL1982	26OCT2006	24	male	.	182	73.8	22	caucasian	
	776	1125	1	06JUN1980	12OCT2006	26	male	.	186	77.9	23	caucasian	
	778	1272	1	18FEB1987	30OCT2006	19	female	B	165	71.0	26	caucasian	
	779	1262	1	20AUG1983	27OCT2006	23	male	.	175	61.9	20	caucasian	
	780	1220	1	22JAN1976	24OCT2006	30	male	.	198	99.6	25	caucasian	
	792	1114	1	11FEB1983	11OCT2006	23	male	.	172	60.8	21	caucasian	
	795	1304	1	20AUG1976	03NOV2006	30	female	B	173	84.1	28	caucasian	
	797	1130	1	21OCT1979	13OCT2006	26	female	B	177	67.4	22	caucasian	
	803	1281	1	09AUG1982	31OCT2006	24	male	.	180	74.6	23	caucasian	
	805	1223	1	20OCT1985	25OCT2006	21	female	B	171	77.2	26	caucasian	
	809	1248	1	16NOV1978	26OCT2006	27	female	B	174	81.3	27	caucasian	
Group 2 (MP)	069	144	1	31MAR1978	22MAY2006	28	female	B	170	70.2	24	caucasian	
	082	199	1	28JUL1964	02JUN2006	41	female	A	161	58.2	22	caucasian	
	085	158	1	05NOV1981	24MAY2006	24	male	.	178	67.6	21	caucasian	
	090	192	1	05MAR1983	01JUN2006	23	female	B	168	65.0	23	caucasian	
	095	200	1	21FEB1981	02JUN2006	25	female	B	165	65.4	24	caucasian	
	104	223	1	12JUL1986	07JUN2006	19	female	B	176	62.7	20	caucasian	
	105	225	1	02APR1986	07JUN2006	20	female	B	169	62.3	22	caucasian	
	114	231	1	12JUL1983	08JUN2006	22	female	B	163	55.4	21	caucasian	
	125	309	1	04APR1987	21JUN2006	19	female	B	178	60.4	19	caucasian	
	128	245	1	23NOV1982	09JUN2006	23	female	B	174	57.3	19	caucasian	
	130	253	1	31MAR1982	13JUN2006	24	female	B	171	59.6	20	caucasian	

[1]Child-bearing potential: A: surgically sterile or post-menopausal B: of child-bearing potential and using appropriate birth control

Demographic data

Group	Subj	Screening number	Centre	Date of birth	Written informed consent was obtained on:	Age [years]	Gender	Child-bearing potential [1]	Height [cm]	Weight [kg]	Body mass index [kg/m²]	Ethnic group	Ethnic group: please specify other
Group 2 (MP)	136	165	1	14SEP1978	29MAY2006	27	male	.	182	91.1	28	caucasian	
	141	181	1	27NOV1977	31MAY2006	28	male	.	196	100.8	26	caucasian	
	142	270	1	13APR1979	14JUN2006	27	male	.	171	71.2	24	caucasian	
	164	303	1	29JAN1979	21JUN2006	27	male	.	174	74.8	25	caucasian	
	165	300	1	20OCT1984	21JUN2006	21	male	.	186	76.0	22	caucasian	
	167	348	1	13JAN1980	20JUN2006	26	female	B	164	57.6	21	arabic	
	169	378	1	22SEP1981	29JUN2006	24	male	.	177	75.5	24	caucasian	
	171	373	1	26OCT1984	28JUN2006	21	female	B	156	53.0	22	caucasian	
	177	347	1	26JUN1979	26JUN2006	27	female	B	182	66.8	20	caucasian	
	184	330	1	24DEC1967	23JUN2006	38	male	.	176	70.1	23	caucasian	
	187	349	1	03AUG1979	27JUN2006	26	female	B	171	53.2	18	caucasian	
	188	352	1	12MAR1977	27JUN2006	29	female	B	169	72.5	25	caucasian	
	192	250	1	13APR1977	12JUN2006	29	female	B	157	69.9	28	asian	
	198	363	1	01JUL1979	28JUN2006	26	female	B	165	54.2	20	caucasian	
	202	327	1	26JAN1978	22JUN2006	28	male	.	182	85.9	26	caucasian	
	203	393	1	28SEP1981	03JUL2006	24	female	B	156	60.3	25	caucasian	
	209	419	1	04DEC1978	05JUL2006	27	male	.	183	76.0	23	caucasian	
	213	387	1	05OCT1981	30JUN2006	24	female	B	162	53.8	20	caucasian	
	217	346	1	27SEP1978	26JUN2006	27	female	B	174	75.0	25	caucasian	
	220	316	1	04MAY1980	22JUN2006	26	female	B	162	58.9	22	caucasian	
	228	430	1	24DEC1973	06JUL2006	32	male	.	186	84.8	25	caucasian	
	229	413	1	10SEP1983	05JUL2006	22	female	B	170	72.4	25	caucasian	
	247	468	1	10FEB1977	13JUL2006	29	female	B	178	100.7	32	caucasian	
	250	466	1	05JAN1976	12JUL2006	30	male	.	182	76.5	23	caucasian	
	251	337	1	25NOV1976	26JUN2006	29	male	.	187	91.7	26	caucasian	
	253	432	1	08JAN1980	07JUL2006	26	female	B	159	60.4	24	caucasian	
	273	401	1	25APR1980	03JUL2006	26	female	B	167	68.9	25	caucasian	
	278	465	1	19AUG1980	12JUL2006	25	female	B	160	48.4	19	caucasian	

[1]Child-bearing potential: A: surgically sterile or post-menopausal B: of child-bearing potential and using appropriate birth control

Demographic data

Group	Subj	Screening number	Centre	Date of birth	Written informed consent was obtained on:	Age [years]	Gender	Child-bearing potential [1]	Height [cm]	Weight [kg]	Body mass index [kg/m²]	Ethnic group	Ethnic group: please specify other
Group 2 (MP)	284	487	1	10SEP1985	17JUL2006	20	male	.	188	91.9	26	caucasian	
	285	490	1	23JAN1977	17JUL2006	29	female	B	164	65.4	24	caucasian	
	290	525	1	10MAR1977	21JUL2006	29	female	B	167	60.2	22	caucasian	
	292	444	1	18JUL1977	28JUN2006	28	female	B	169	65.3	23	caucasian	
	301	582	1	29JUL1984	28JUL2006	21	male	.	180	82.0	25	other	CAUCASIAN/ ARABIC
	312	579	1	25FEB1977	28JUL2006	29	female	B	173	60.0	20	caucasian	
	313	642	1	30APR1976	08AUG2006	30	female	B	165	52.3	19	caucasian	
	316	562	1	13OCT1978	26JUL2006	27	female	B	169	61.4	21	caucasian	
	328	560	1	30NOV1982	26JUL2006	23	male	.	188	106.6	30	caucasian	
	329	547	1	18MAR1978	25JUL2006	28	female	B	169	61.7	22	asian	
	341	527	1	10JUN1985	21JUL2006	21	female	B	170	66.7	23	caucasian	
	355	605	1	30NOV1984	02AUG2006	21	female	B	167	58.0	21	caucasian	
	361	616	1	11NOV1978	03AUG2006	27	female	B	161	71.9	28	caucasian	
	362	607	1	12APR1974	02AUG2006	32	female	B	164	59.2	22	caucasian	
	364	629	1	17DEC1973	04AUG2006	32	male	.	181	79.0	24	caucasian	
	372	644	1	05JAN1984	04JUL2006	22	female	B	169	63.3	22	caucasian	
	376	623	1	30AUG1975	04AUG2006	30	male	.	166	85.0	31	caucasian	
	385	598	1	11SEP1979	01AUG2006	26	female	B	169	63.2	22	caucasian	
	389	625	1	02AUG1985	04AUG2006	21	female	B	161	81.4	31	caucasian	
	391	599	1	22FEB1980	01AUG2006	26	male	.	178	78.5	25	caucasian	
	406	701	1	08FEB1983	16AUG2006	23	female	B	159	52.0	21	caucasian	
	423	739	1	03SEP1980	18AUG2006	25	female	B	159	54.3	21	caucasian	
	425	664	1	20JUN1986	10AUG2006	20	female	B	167	74.9	27	caucasian	
	426	740	1	08MAY1980	21AUG2006	26	female	B	165	47.4	17	caucasian	
	435	730	1	04AUG1977	18AUG2006	29	female	B	171	60.2	21	caucasian	
	436	760	1	23AUG1976	22AUG2006	29	male	.	180	75.6	23	caucasian	
	445	737	1	21AUG1971	18AUG2006	34	male	.	188	78.5	22	caucasian	

[1]Child-bearing potential: A: surgically sterile or post-menopausal B: of child-bearing potential and using appropriate birth control

Demographic data

Group	Subj	Screening number	Centre	Date of birth	Written informed consent was obtained on:	Age [years]	Gender	Child-bearing potential [1]	Height [cm]	Weight [kg]	Body mass index [kg/m²]	Ethnic group	Ethnic group: please specify other
Group 2 (MP)	446	796	1	22APR1980	24AUG2006	26	male	.	172	79.0	27	caucasian	
	453	805	1	16MAY1982	25AUG2006	24	male	.	173	61.2	20	caucasian	
	458	808	1	14JUL1981	28AUG2006	25	female	B	161	63.2	24	caucasian	
	460	811	1	09DEC1981	28AUG2006	24	female	B	171	63.9	22	caucasian	
	461	793	1	06MAR1981	24AUG2006	25	male	.	185	66.8	20	caucasian	
	466	815	1	05AUG1974	29AUG2006	32	male	.	179	84.2	26	caucasian	
	470	663	1	14OCT1978	09AUG2006	27	female	B	173	66.0	22	caucasian	
	474	835	1	30MAY1984	31AUG2006	22	male	.	183	68.1	20	caucasian	
	477	812	1	11APR1982	28AUG2006	24	male	.	184	80.0	24	caucasian	
	478	838	1	11NOV1981	27JUL2006	24	female	B	165	59.0	22	caucasian	
	480	820	1	13APR1978	29AUG2006	28	female	B	167	76.3	27	caucasian	
	481	810	1	24FEB1979	28AUG2006	27	male	.	189	84.1	24	caucasian	
	486	803	1	01APR1986	25AUG2006	20	female	B	167	99.8	36	caucasian	
	487	788	1	20DEC1982	24AUG2006	23	female	B	158	65.1	26	caucasian	
	490	834	1	08JUL1976	23JUN2006	29	female	B	166	70.1	25	caucasian	
	491	825	1	25JUN1983	30AUG2006	23	female	B	171	84.8	29	caucasian	
	492	840	1	17NOV1981	01SEP2006	24	male	.	186	80.1	23	caucasian	
	493	801	1	27JUN1982	25AUG2006	24	male	.	185	73.6	22	caucasian	
	499	849	1	25AUG1983	04SEP2006	23	female	B	173	52.8	18	caucasian	
	504	860	1	19FEB1972	05SEP2006	34	female	B	186	82.4	24	caucasian	
	506	783	1	16DEC1984	24AUG2006	21	female	B	175	67.8	22	caucasian	
	511	869	1	23NOV1979	07SEP2006	26	female	B	170	56.8	20	caucasian	
	515	878	1	10MAY1982	07SEP2006	24	male	.	180	69.4	21	caucasian	
	516	896	1	05MAR1978	11SEP2006	28	male	.	183	85.0	25	caucasian	
	520	874	1	28AUG1985	07SEP2006	21	male	.	178	63.0	20	caucasian	
	522	863	1	21AUG1978	06SEP2006	28	male	.	189	114.7	32	caucasian	
	526	900	1	25JUN1975	11SEP2006	31	female	B	160	76.0	30	caucasian	
	527	903	1	12JUL1979	11SEP2006	27	female	A	170	65.4	23	caucasian	

[1]Child-bearing potential: A: surgically sterile or post-menopausal B: of child-bearing potential and using appropriate birth control

Demographic data

Group	Subj	Screening number	Centre	Date of birth	Written informed consent was obtained on:	Age [years]	Gender	Child-bearing potential [1]	Height [cm]	Weight [kg]	Body mass index [kg/ m²]	Ethnic group	Ethnic group: please specify other
Group 2 (MP)	528	924	1	17SEP1975	13SEP2006	30	female	B	166	63.4	23	caucasian	
	529	756	1	08DEC1986	22AUG2006	19	male	.	183	86.8	26	caucasian	
	530	883	1	09JAN1984	08SEP2006	22	male	.	181	78.3	24	caucasian	
	535	911	1	22APR1981	12SEP2006	25	female	B	168	93.3	33	caucasian	
	536	926	1	19JAN1968	14SEP2006	38	female	B	173	62.0	21	caucasian	
	540	950	1	11AUG1985	15SEP2006	21	male	.	197	94.2	24	caucasian	
	542	875	1	19JUN1975	07SEP2006	31	male	.	196	97.7	25	caucasian	
	544	893	1	25AUG1973	11SEP2006	33	male	.	175	81.7	27	caucasian	
	548	931	1	01OCT1983	14SEP2006	22	male	.	180	71.2	22	caucasian	
	550	941	1	21OCT1982	15SEP2006	23	male	.	177	60.7	19	caucasian	
	552	861	1	06DEC1983	05SEP2006	22	male	.	180	82.7	26	caucasian	
	553	936	1	26MAR1962	15SEP2006	44	female	A	164	62.4	23	caucasian	
	559	895	1	22NOV1980	11SEP2006	25	female	B	163	53.7	20	caucasian	
	561	848	1	23OCT1981	04SEP2006	24	female	B	168	51.7	18	caucasian	
	563	966	1	12FEB1981	18SEP2006	25	female	B	165	73.9	27	caucasian	
	573	971	1	13OCT1985	19SEP2006	20	female	B	166	56.8	21	caucasian	
	576	904	1	07OCT1974	11SEP2006	31	male	.	182	66.1	20	caucasian	
	579	987	1	10MAY1985	21SEP2006	21	male	.	181	78.0	24	caucasian	
	584	910	1	28NOV1986	12SEP2006	19	female	B	172	61.3	21	caucasian	
	585	982	1	20JAN1979	20SEP2006	27	female	B	169	64.1	22	caucasian	
	588	1020	1	16SEP1984	26SEP2006	22	female	B	177	72.6	23	caucasian	
	590	1033	1	18JAN1985	28SEP2006	21	male	.	175	72.4	24	caucasian	
	591	985	1	01DEC1979	21SEP2006	26	female	B	167	64.9	23	caucasian	
	598	865	1	07JUN1987	06SEP2006	19	female	B	159	60.8	24	caucasian	
	602	1027	1	28AUG1988	27SEP2006	18	male	.	175	66.5	22	caucasian	
	614	1047	1	17MAY1979	29SEP2006	27	male	.	182	69.5	21	caucasian	
	615	1032	1	12DEC1976	28SEP2006	29	female	B	165	95.4	35	caucasian	
	619	1012	1	24MAR1981	25SEP2006	25	female	B	165	54.4	20	caucasian	

[1]Child-bearing potential: A: surgically sterile or post-menopausal B: of child-bearing potential and using appropriate birth control

Demographic data

Group	Subj	Screening number	Centre	Date of birth	Written informed consent was obtained on:	Age [years]	Gender	Child-bearing potential [1]	Height [cm]	Weight [kg]	Body mass index [kg/m²]	Ethnic group	Ethnic group: please specify other
Group 2 (MP)	622	994	1	16APR1983	22SEP2006	23	male	.	183	84.2	25	caucasian	
	623	1025	1	27NOV1977	27SEP2006	28	female	B	166	68.1	25	caucasian	
	624	944	1	12OCT1964	15SEP2006	41	female	B	157	82.0	33	caucasian	
	626	1060	1	20JUN1984	04OCT2006	22	female	B	168	63.9	23	caucasian	
	630	1075	1	31MAR1978	05OCT2006	28	male	.	183	83.7	25	caucasian	
	633	930	1	08NOV1977	14SEP2006	28	female	B	167	71.9	26	caucasian	
	635	1092	1	15FEB1983	06OCT2006	23	female	B	170	67.7	23	caucasian	
	639	1000	1	15JAN1986	22SEP2006	20	female	B	172	65.8	22	caucasian	
	642	1080	1	26APR1985	05OCT2006	21	female	B	160	61.6	24	caucasian	
	647	996	1	05JAN1976	22SEP2006	30	male	.	190	90.0	25	caucasian	
	660	1105	1	23MAR1981	10OCT2006	25	female	B	170	61.9	21	caucasian	
	664	1107	1	30DEC1982	10OCT2006	23	male	.	177	76.4	24	caucasian	
	667	1083	1	09SEP1984	05OCT2006	22	female	B	175	68.7	22	caucasian	
	668	1058	1	24JUL1978	04OCT2006	28	female	B	166	49.7	18	caucasian	
	672	1046	1	14SEP1983	29SEP2006	23	male	.	178	89.6	28	black	
	674	1142	1	11JUN1985	16OCT2006	21	male	.	176	77.6	25	caucasian	
	675	1140	1	31JUL1981	16OCT2006	25	female	B	163	64.5	24	caucasian	
	677	1013	1	16DEC1981	25SEP2006	24	male	.	183	73.1	22	caucasian	
	679	1022	1	19MAR1981	26SEP2006	25	female	B	170	65.2	23	caucasian	
	680	1043	1	09JUN1985	29SEP2006	21	female	B	176	96.2	31	caucasian	
	693	1126	1	05DEC1979	12OCT2006	26	female	B	160	58.5	23	caucasian	
	694	1141	1	24MAR1978	16OCT2006	28	female	B	162	50.8	19	caucasian	
	696	1156	1	30DEC1984	04JUL2006	21	female	B	172	69.5	23	caucasian	
	699	1136	1	14JUN1980	13OCT2006	26	female	B	176	79.7	26	caucasian	
	706	1170	1	26JUL1988	18OCT2006	18	male	.	177	70.2	22	caucasian	
	708	1157	1	21SEP1980	17OCT2006	26	male	.	183	78.5	23	caucasian	
	709	1188	1	12JUL1978	20OCT2006	28	male	.	187	85.5	24	caucasian	
	710	1118	1	14NOV1980	11OCT2006	25	female	B	166	63.0	23	caucasian	

[1] Child-bearing potential: A: surgically sterile or post-menopausal B: of child-bearing potential and using appropriate birth control

Demographic data

Group	Subj	Screening number	Centre	Date of birth	Written informed consent was obtained on:	Age [years]	Gender	Child-bearing potential [1]	Height [cm]	Weight [kg]	Body mass index [kg/ m²]	Ethnic group	Ethnic group: please specify other
Group 2 (MP)	712	1186	1	25NOV1980	20OCT2006	25	male	.	180	73.8	23	caucasian	
	713	1149	1	10SEP1987	16OCT2006	19	male	.	178	77.4	24	caucasian	
	714	1144	1	09FEB1981	16OCT2006	25	female	B	172	59.5	20	caucasian	
	722	1189	1	21NOV1981	20OCT2006	24	female	B	153	62.0	26	caucasian	
	731	1211	1	03MAY1983	23OCT2006	23	female	B	167	55.3	20	caucasian	
	732	1109	1	10FEB1977	11OCT2006	29	female	B	167	94.6	34	caucasian	
	736	1041	1	04AUG1981	29SEP2006	25	female	B	172	75.4	25	caucasian	
	738	1225	1	12DEC1983	25OCT2006	22	female	B	167	57.3	21	caucasian	
	747	1234	1	25JUL1981	25OCT2006	25	male	.	180	94.3	29	caucasian	
	752	1196	1	27JAN1977	20OCT2006	29	female	B	172	75.6	26	caucasian	
	754	1249	1	27JUL1983	26OCT2006	23	female	B	163	59.6	22	caucasian	
	757	1094	1	09NOV1983	06OCT2006	22	female	B	161	49.8	19	caucasian	
	759	1256	1	25DEC1983	27OCT2006	22	male	.	184	78.4	23	caucasian	
	761	1245	1	16DEC1983	26OCT2006	22	female	B	187	95.7	27	caucasian	
	763	1078	1	02MAY1985	05OCT2006	21	female	B	164	55.0	20	caucasian	
	764	1252	1	05APR1981	22SEP2006	25	female	B	176	85.8	28	caucasian	
	770	1146	1	02SEP1968	16OCT2006	38	female	B	160	75.9	30	caucasian	
	777	1205	1	23AUG1976	23OCT2006	30	male	.	177	86.5	28	caucasian	
	781	1269	1	24SEP1985	30OCT2006	21	female	B	168	74.8	27	caucasian	
	783	1129	1	29SEP1979	12OCT2006	27	male	.	179	79.7	25	caucasian	
	785	1113	1	05DEC1985	11OCT2006	20	male	.	175	60.2	20	caucasian	
	787	1227	1	17OCT1977	25OCT2006	29	male	.	169	69.7	24	caucasian	
	789	1182	1	24SEP1983	19OCT2006	23	female	B	167	58.5	21	caucasian	
	790	1231	1	29SEP1983	25OCT2006	23	male	.	185	77.4	23	caucasian	
	794	1277	1	02FEB1985	31OCT2006	21	female	B	175	62.3	20	caucasian	
	798	1152	1	26APR1982	16OCT2006	24	male	.	183	83.3	25	caucasian	
	800	1275	1	15APR1982	31OCT2006	24	female	B	170	59.8	21	caucasian	
	804	1289	1	25JUL1984	02NOV2006	22	female	B	169	61.3	21	caucasian	

[1]Child-bearing potential: A: surgically sterile or post-menopausal B: of child-bearing potential and using appropriate birth control

Demographic data

Group	Subj	Screening number	Centre	Date of birth	Written informed consent was obtained on:	Age [years]	Gender	Child-bearing potential [1]	Height [cm]	Weight [kg]	Body mass index [kg/m²]	Ethnic group	Ethnic group: please specify other
Group 2 (MP)	806	1206	1	23SEP1983	23OCT2006	23	male	.	178	77.4	24	caucasian	
	807	1213	1	13MAR1983	24OCT2006	23	female	B	161	56.7	22	caucasian	
	815	1271	1	21AUG1984	30OCT2006	22	male	.	165	70.5	26	caucasian	
Group 3 (PP)	067	155	1	01DEC1956	24MAY2006	49	female	B	160	47.7	19	caucasian	
	068	122	1	27SEP1979	18MAY2006	26	male	.	194	87.0	23	caucasian	
	072	140	1	02MAY1979	22MAY2006	27	male	.	180	79.8	25	caucasian	
	094	163	1	24FEB1981	29MAY2006	25	male	.	183	69.6	21	caucasian	
	099	214	1	24SEP1980	06JUN2006	25	female	B	168	62.3	22	caucasian	
	101	211	1	29JAN1986	06JUN2006	20	female	B	163	54.4	20	caucasian	
	110	220	1	04FEB1988	07JUN2006	18	female	B	168	61.7	22	caucasian	
	112	324	1	11OCT1980	22JUN2006	25	female	B	166	62.9	23	caucasian	
	120	248	1	21JAN1975	12JUN2006	31	male	.	183	67.1	20	caucasian	
	126	268	1	05SEP1981	14JUN2006	24	female	B	171	63.3	22	caucasian	
	131	241	1	09SEP1974	09JUN2006	31	male	.	175	76.3	25	caucasian	
	135	173	1	24JUL1984	30MAY2006	21	male	.	188	71.2	20	caucasian	
	138	282	1	10SEP1979	19JUN2006	26	female	B	163	52.4	20	caucasian	
	147	340	1	10JUN1981	26JUN2006	25	male	.	177	72.7	23	caucasian	
	149	344	1	16NOV1982	26JUN2006	23	male	.	187	81.3	23	caucasian	
	152	215	1	02OCT1986	06JUN2006	19	female	B	163	65.2	25	caucasian	
	158	291	1	06JUL1977	20JUN2006	28	female	B	166	62.4	23	caucasian	
	159	295	1	12SEP1979	20JUN2006	26	male	.	175	65.4	21	caucasian	
	163	304	1	02AUG1983	21JUN2006	22	female	B	171	88.2	30	caucasian	
	166	298	1	25JUL1980	20JUN2006	25	female	B	170	54.3	19	caucasian	
	172	351	1	29SEP1981	27JUN2006	24	male	.	181	90.0	27	caucasian	
	175	357	1	23DEC1983	27JUN2006	22	male	.	193	85.6	23	caucasian	
	186	321	1	08MAY1981	22JUN2006	25	female	B	175	61.4	20	caucasian	

[1]Child-bearing potential: A: surgically sterile or post-menopausal B: of child-bearing potential and using appropriate birth control

Demographic data

Group	Subj	Screening number	Centre	Date of birth	Written informed consent was obtained on:	Age [years]	Gender	Child-bearing potential [1]	Height [cm]	Weight [kg]	Body mass index [kg/m²]	Ethnic group	Ethnic group: please specify other
Group 3 (PP)	190	233	1	02SEP1983	08JUN2006	22	female	B	170	72.9	25	caucasian	
	191	314	1	02SEP1977	22JUN2006	28	female	B	157	58.3	24	caucasian	
	200	396	1	21JUL1986	03JUL2006	19	female	B	151	52.8	23	caucasian	
	201	368	1	26DEC1981	28JUN2006	24	male	.	188	89.3	25	caucasian	
	221	402	1	20APR1978	03JUL2006	28	male	.	189	95.7	27	caucasian	
	232	412	1	22DEC1974	04JUL2006	31	male	.	192	86.2	23	caucasian	
	233	429	1	03MAR1984	06JUL2006	22	female	B	165	58.5	21	caucasian	
	239	395	1	01DEC1982	03JUL2006	23	female	B	163	65.2	25	caucasian	
	241	439	1	04MAR1985	07JUN2006	21	male	.	196	80.9	21	caucasian	
	265	383	1	05NOV1982	29JUN2006	23	female	B	177	61.5	20	caucasian	
	266	510	1	27FEB1986	19JUL2006	20	female	B	166	60.0	22	caucasian	
	276	513	1	23MAY1980	20JUL2006	26	female	B	181	61.3	19	caucasian	
	288	526	1	23NOV1977	21JUL2006	28	female	B	177	66.5	21	caucasian	
	294	511	1	04FEB1981	20JUL2006	25	female	B	168	69.1	24	asian	
	296	423	1	01FEB1968	06JUL2006	38	female	B	168	80.6	29	caucasian	
	300	469	1	26MAY1978	13JUL2006	28	male	.	192	84.3	23	caucasian	
	303	492	1	07JUL1979	18JUL2006	27	female	B	178	68.9	22	caucasian	
	305	522	1	11OCT1985	21JUL2006	20	female	B	163	63.4	24	caucasian	
	307	577	1	23MAR1978	28JUL2006	28	male	.	190	80.4	22	caucasian	
	308	654	1	18APR1983	05JUL2006	23	male	.	170	66.9	23	caucasian	
	309	427	1	05SEP1975	06JUL2006	30	male	.	178	74.0	23	caucasian	
	310	491	1	07NOV1982	17JUL2006	23	female	B	170	94.2	33	caucasian	
	321	567	1	01MAR1980	27JUL2006	26	female	B	164	56.0	21	caucasian	
	337	572	1	10JAN1983	27JUL2006	23	female	B	167	54.3	19	caucasian	
	339	539	1	04DEC1975	24JUL2006	30	female	B	167	57.1	20	caucasian	
	348	550	1	20NOV1982	25JUL2006	23	male	.	171	80.1	27	caucasian	
	350	603	1	04FEB1985	02AUG2006	21	female	B	164	49.1	18	caucasian	
	357	606	1	15MAR1979	02AUG2006	27	female	B	169	61.9	22	caucasian	

[1]Child-bearing potential: A: surgically sterile or post-menopausal B: of child-bearing potential and using appropriate birth control

Demographic data

Group	Subj	Screening number	Centre	Date of birth	Written informed consent was obtained on:	Age [years]	Gender	Child-bearing potential [1]	Height [cm]	Weight [kg]	Body mass index [kg/m²]	Ethnic group	Ethnic group: please specify other
Group 3 (PP)	360	597	1	18JUL1980	01AUG2006	26	female	B	164	63.8	24	caucasian	
	367	610	1	30NOV1979	02AUG2006	26	male	.	173	59.9	20	caucasian	
	374	591	1	15JAN1983	31JUL2006	23	female	B	164	56.7	21	caucasian	
	384	639	1	23JUN1978	07AUG2006	28	female	B	178	101.4	32	caucasian	
	387	617	1	31OCT1981	03AUG2006	24	male	.	182	78.1	24	caucasian	
	390	586	1	29APR1978	31JUL2006	28	male	.	189	82.4	23	caucasian	
	392	662	1	27JUN1980	09AUG2006	26	female	B	166	94.8	34	caucasian	
	394	667	1	23MAY1983	10AUG2006	23	female	B	169	60.1	21	caucasian	
	399	699	1	15DEC1975	16AUG2006	30	male	.	186	82.9	24	caucasian	
	405	694	1	12SEP1979	14AUG2006	26	female	B	174	72.0	24	caucasian	
	431	641	1	19AUG1972	07AUG2006	33	male	.	173	80.4	27	caucasian	
	437	615	1	14NOV1984	03AUG2006	21	female	B	150	53.8	24	caucasian	
	440	722	1	17JUL1982	17AUG2006	24	male	.	177	74.3	24	caucasian	
	443	715	1	19OCT1980	17AUG2006	25	male	.	190	80.4	22	caucasian	
	444	714	1	12MAR1966	17AUG2006	40	male	.	181	80.7	25	caucasian	
	450	748	1	28MAR1982	21AUG2006	24	male	.	190	96.5	27	caucasian	
	454	934	1	07APR1982	22AUG2006	24	male	.	184	88.1	26	caucasian	
	456	713	1	16DEC1979	17AUG2006	26	female	B	171	63.2	22	caucasian	
	463	789	1	30JUN1981	24AUG2006	25	female	B	170	66.7	23	caucasian	
	464	764	1	03SEP1980	22AUG2006	25	male	.	184	75.8	22	caucasian	
	465	637	1	21SEP1984	07AUG2006	21	female	B	160	61.0	24	caucasian	
	467	841	1	12DEC1974	01SEP2006	31	female	B	177	70.7	23	caucasian	
	468	792	1	22AUG1982	24AUG2006	24	female	B	160	55.7	22	caucasian	
	469	828	1	02MAR1979	28JUL2006	27	male	.	176	71.7	23	caucasian	
	475	696	1	02MAR1983	14AUG2006	23	male	.	191	87.8	24	caucasian	
	476	836	1	15JUN1984	31AUG2006	22	male	.	181	66.0	20	caucasian	
	485	862	1	02JUL1983	06SEP2006	23	female	B	161	56.4	22	caucasian	
	494	791	1	26JUL1956	24AUG2006	50	male	.	179	78.1	24	caucasian	

[1]Child-bearing potential: A: surgically sterile or post-menopausal B: of child-bearing potential and using appropriate birth control

Demographic data

Group	Subj	Screening number	Centre	Date of birth	Written informed consent was obtained on:	Age [years]	Gender	Child-bearing potential [1]	Height [cm]	Weight [kg]	Body mass index [kg/m²]	Ethnic group	Ethnic group: please specify other
Group 3 (PP)	496	845	1	25AUG1980	01SEP2006	26	male	.	176	68.8	22	caucasian	
	498	853	1	14NOV1978	04SEP2006	27	female	B	164	88.2	33	caucasian	
	500	837	1	11JAN1963	31AUG2006	43	male	.	181	109.8	34	caucasian	
	501	858	1	07MAR1979	05SEP2006	27	female	B	171	62.1	21	caucasian	
	503	830	1	11AUG1988	30AUG2006	18	female	B	162	48.4	18	caucasian	
	507	851	1	24MAY1973	04SEP2006	33	male	.	181	116.8	36	caucasian	
	508	882	1	08MAY1987	08SEP2006	19	female	B	165	71.1	26	caucasian	
	509	879	1	11SEP1977	07SEP2006	28	male	.	176	69.8	23	caucasian	
	513	832	1	25AUG1984	31AUG2006	22	male	.	171	66.3	23	caucasian	
	514	894	1	29JAN1982	11SEP2006	24	female	B	170	73.5	25	caucasian	
	517	855	1	14JUL1966	05SEP2006	40	female	B	162	120.9	46	caucasian	
	523	842	1	22JUN1984	01SEP2006	22	male	.	190	74.6	21	caucasian	
	531	921	1	27AUG1978	13SEP2006	28	male	.	183	86.8	26	caucasian	
	533	892	1	14JUN1978	11SEP2006	28	male	.	174	70.2	23	caucasian	
	538	909	1	18DEC1979	12SEP2006	26	female	B	175	87.7	29	caucasian	
	539	912	1	22OCT1978	12SEP2006	27	female	B	164	61.8	23	caucasian	
	541	956	1	02MAR1976	18SEP2006	30	male	.	183	79.6	24	caucasian	
	546	943	1	30NOV1978	15SEP2006	27	male	.	180	74.0	23	caucasian	
	547	922	1	20DEC1980	13SEP2006	25	female	B	173	59.3	20	caucasian	
	549	940	1	06AUG1973	15SEP2006	33	female	B	175	69.8	23	caucasian	
	551	935	1	24MAR1972	15SEP2006	34	female	B	173	90.2	30	caucasian	
	554	919	1	19MAY1978	13SEP2006	28	female	B	182	67.1	20	caucasian	
	560	945	1	30DEC1982	15SEP2006	23	female	B	165	66.2	24	caucasian	
	564	963	1	27FEB1978	18SEP2006	28	female	B	161	53.6	21	caucasian	
	566	958	1	04MAY1982	18SEP2006	24	female	B	176	82.0	26	caucasian	
	570	948	1	01AUG1976	15SEP2006	30	male	.	180	75.1	23	caucasian	
	577	884	1	17MAY1986	08SEP2006	20	male	.	175	57.8	19	caucasian	
	578	888	1	30NOV1977	11SEP2006	28	male	.	188	82.6	23	caucasian	

[1]Child-bearing potential: A: surgically sterile or post-menopausal B: of child-bearing potential and using appropriate birth control

Demographic data

Group	Subj	Screening number	Centre	Date of birth	Written informed consent was obtained on:	Age [years]	Gender	Child-bearing potential [1]	Height [cm]	Weight [kg]	Body mass index [kg/m²]	Ethnic group	Ethnic group: please specify other
Group 3 (PP)	580	979	1	04SEP1985	20SEP2006	21	female	B	184	67.6	20	caucasian	
	581	916	1	11SEP1976	13SEP2006	30	male	.	181	84.7	26	caucasian	
	589	1003	1	02FEB1977	22SEP2006	29	female	B	172	68.4	23	caucasian	
	592	969	1	12JUL1979	19SEP2006	27	male	.	183	68.3	20	caucasian	
	593	1005	1	19NOV1983	25SEP2006	22	female	B	167	67.7	24	caucasian	
	594	1009	1	21NOV1979	25SEP2006	26	female	B	178	65.9	21	caucasian	
	596	980	1	15JUN1986	20SEP2006	20	male	.	181	68.8	21	caucasian	
	599	995	1	11OCT1987	22SEP2006	18	female	B	171	62.4	21	caucasian	
	600	1029	1	07JAN1983	27SEP2006	23	male	.	197	88.5	23	caucasian	
	601	975	1	09JUL1976	19SEP2006	30	male	.	184	79.9	24	caucasian	
	603	1017	1	04AUG1979	26SEP2006	27	female	B	170	71.3	25	arabic	
	611	1045	1	02JUN1983	29SEP2006	23	female	B	158	77.2	31	caucasian	
	613	1042	1	03JUL1980	29SEP2006	26	female	B	168	56.8	20	caucasian	
	617	1014	1	16JUN1983	26SEP2006	23	female	B	164	86.1	32	caucasian	
	620	1093	1	18JAN1983	06OCT2006	23	female	B	168	55.9	20	caucasian	
	628	1065	1	12APR1976	04OCT2006	30	female	B	172	56.6	19	caucasian	
	631	1079	1	14JUN1975	05OCT2006	31	female	B	154	53.6	23	caucasian	
	632	1095	1	11APR1986	06OCT2006	20	female	B	167	52.5	19	caucasian	
	638	1028	1	10OCT1979	27SEP2006	26	female	B	173	69.4	23	caucasian	
	640	1038	1	14MAR1981	28SEP2006	25	female	B	167	62.5	22	caucasian	
	644	1085	1	04NOV1979	05OCT2006	26	male	.	175	70.0	23	caucasian	
	650	1117	1	11AUG1974	11OCT2006	32	female	B	177	78.2	25	caucasian	
	651	1011	1	12FEB1983	25SEP2006	23	male	.	174	65.2	22	caucasian	
	652	1023	1	10MAY1984	26SEP2006	22	female	B	165	107.8	40	caucasian	
	653	1069	1	28OCT1983	04OCT2006	22	female	B	171	66.0	23	caucasian	
	656	1059	1	28JUN1965	04OCT2006	41	male	.	183	82.0	24	caucasian	
	657	1103	1	22MAR1977	10OCT2006	29	male	.	173	77.2	26	caucasian	
	658	1035	1	11NOV1980	28SEP2006	25	male	.	186	111.1	32	caucasian	

[1]Child-bearing potential: A: surgically sterile or post-menopausal B: of child-bearing potential and using appropriate birth control

Demographic data

Group	Subj	Screening number	Centre	Date of birth	Written informed consent was obtained on:	Age [years]	Gender	Child-bearing potential [1]	Height [cm]	Weight [kg]	Body mass index [kg/ m²]	Ethnic group	Ethnic group: please specify other
Group 3 (PP)	661	1082	1	15JUL1976	05OCT2006	30	female	B	169	67.8	24	caucasian	
	671	1015	1	17SEP1982	26SEP2006	24	female	B	164	68.7	26	caucasian	
	682	1061	1	07SEP1982	04OCT2006	24	female	B	158	60.5	24	caucasian	
	684	1106	1	01OCT1983	10OCT2006	23	male	.	199	95.4	24	caucasian	
	685	1001	1	13JAN1980	22SEP2006	26	female	B	167	63.3	23	caucasian	
	686	1145	1	02NOV1985	16OCT2006	20	female	B	171	47.3	16	caucasian	
	687	1164	1	03DEC1962	17OCT2006	43	female	B	160	62.5	24	caucasian	
	689	1133	1	24MAY1976	13OCT2006	30	male	.	183	82.0	24	caucasian	
	695	1134	1	12MAR1976	13OCT2006	30	female	B	171	72.4	25	other	COMBINATIO N
	700	1154	1	08OCT1980	16OCT2006	26	male	.	172	62.8	21	caucasian	
	702	1135	1	10OCT1980	13OCT2006	26	female	B	152	77.4	34	caucasian	
	704	1166	1	20AUG1982	18OCT2006	24	female	B	173	61.1	20	caucasian	
	711	1193	1	07JUL1983	20OCT2006	23	female	B	172	57.1	19	caucasian	
	715	1150	1	20SEP1982	16OCT2006	24	male	.	172	80.9	27	caucasian	
	717	1160	1	09DEC1977	17OCT2006	28	female	B	173	63.0	21	caucasian	
	718	1184	1	08APR1985	20OCT2006	21	female	B	172	77.0	26	caucasian	
	723	1167	1	20AUG1979	18OCT2006	27	female	B	166	58.4	21	caucasian	
	724	1192	1	22NOV1982	20OCT2006	23	female	B	178	98.5	31	caucasian	
	726	1195	1	01SEP1982	20OCT2006	24	female	B	161	48.7	19	other	SOUTH AMERICA
	727	1175	1	04SEP1985	18OCT2006	21	female	B	162	63.0	24	caucasian	
	728	1139	1	19JUL1981	13OCT2006	25	male	.	181	71.2	22	caucasian	
	737	1138	1	23MAR1979	13OCT2006	27	male	.	181	91.3	28	caucasian	
	739	1203	1	28MAR1977	23OCT2006	29	female	B	160	56.1	22	caucasian	
	740	1221	1	17OCT1982	24OCT2006	24	male	.	186	80.7	23	caucasian	
	741	1183	1	08JUL1980	19OCT2006	26	female	B	177	90.2	29	caucasian	
	744	1088	1	25AUG1977	06OCT2006	29	female	B	175	65.6	21	caucasian	

[1]Child-bearing potential: A: surgically sterile or post-menopausal B: of child-bearing potential and using appropriate birth control

Demographic data

Group	Subj	Screening number	Centre	Date of birth	Written informed consent was obtained on:	Age [years]	Gender	Child-bearing potential [1]	Height [cm]	Weight [kg]	Body mass index [kg/m²]	Ethnic group	Ethnic group: please specify other
Group 3 (PP)	745	1235	1	06APR1978	25OCT2006	28	female	B	172	63.9	22	caucasian	
	751	1084	1	25APR1975	05OCT2006	31	male	.	200	109.9	27	caucasian	
	755	1261	1	16MAY1987	27OCT2006	19	female	B	173	65.7	22	caucasian	
	756	1239	1	09AUG1976	26OCT2006	30	male	.	176	90.2	29	caucasian	
	760	1264	1	28FEB1986	27OCT2006	20	male	.	179	77.5	24	caucasian	
	762	1068	1	28JAN1983	04OCT2006	23	female	B	162	59.4	23	caucasian	
	766	1251	1	16OCT1980	26OCT2006	26	female	B	168	72.8	26	caucasian	
	767	1209	1	12MAY1974	23OCT2006	32	male	.	178	74.4	23	caucasian	
	769	1266	1	19AUG1977	30OCT2006	29	female	B	167	63.4	23	caucasian	
	773	1215	1	19OCT1983	24OCT2006	23	male	.	180	66.0	20	caucasian	
	782	1181	1	15NOV1986	19OCT2006	19	female	B	161	54.9	21	caucasian	
	784	1273	1	15NOV1986	30OCT2006	19	male	.	173	63.9	21	caucasian	
	786	1165	1	23FEB1984	18OCT2006	22	female	B	167	67.4	24	caucasian	
	788	1174	1	23MAR1984	18OCT2006	22	male	.	190	88.5	25	caucasian	
	793	1286	1	02JUL1980	31OCT2006	26	female	B	171	136.0	47	caucasian	
	796	1299	1	13SEP1979	03NOV2006	27	female	B	173	68.0	23	caucasian	
	799	1212	1	17MAY1982	23OCT2006	24	male	.	177	82.0	26	caucasian	
	801	1276	1	07NOV1981	31OCT2006	24	female	B	174	70.2	23	caucasian	
	802	1294	1	10FEB1986	02NOV2006	20	female	B	165	52.2	19	caucasian	
	814	1309	1	04JAN1980	03NOV2006	26	male	.	183	75.0	22	caucasian	
Group 4 (M-)	001	2	1	17MAY1959	27APR2006	46	female	A	174	68.0	22	caucasian	
	002	5	1	01DEC1970	02MAY2006	35	female	B	167	92.1	33	caucasian	
	004	7	1	23MAR1978	02MAY2006	28	male	.	178	64.7	20	black	
	005	9	1	18DEC1953	02MAY2006	52	female	A	163	61.8	23	caucasian	
	006	14	1	30JUL1961	03MAY2006	44	female	A	166	58.2	21	caucasian	
	007	21	1	05APR1971	03MAY2006	35	male	.	175	64.3	21	caucasian	

[1] Child-bearing potential: A: surgically sterile or post-menopausal B: of child-bearing potential and using appropriate birth control

Demographic data

Group	Subj	Screening number	Centre	Date of birth	Written informed consent was obtained on:	Age [years]	Gender	Child-bearing potential [1]	Height [cm]	Weight [kg]	Body mass index [kg/m²]	Ethnic group	Ethnic group: please specify other
Group 4 (M-)	008	16	1	09NOV1958	03MAY2006	47	male	.	181	93.4	29	caucasian	
	009	15	1	03DEC1966	03MAY2006	39	male	.	187	85.6	24	caucasian	
	010	11	1	06JUL1955	02MAY2006	50	female	A	169	69.1	24	caucasian	
	011	23	1	21DEC1961	04MAY2006	44	male	.	180	92.2	28	caucasian	
	012	25	1	12JAN1965	04MAY2006	41	female	B	169	59.6	21	caucasian	
	014	44	1	30SEP1969	05MAY2006	36	female	B	167	54.1	19	caucasian	
	015	48	1	21NOV1965	08MAY2006	40	male	.	165	55.2	20	caucasian	
	016	56	1	11SEP1977	09MAY2006	28	male	.	187	94.2	27	caucasian	
	017	62	1	21FEB1957	09MAY2006	49	female	A	172	69.2	23	caucasian	
	019	74	1	11MAR1974	10MAY2006	32	male	.	176	67.7	22	caucasian	
	020	31	1	31JAN1967	04MAY2006	39	male	.	192	74.1	20	caucasian	
	021	38	1	23MAY1958	05MAY2006	47	female	A	178	67.9	21	caucasian	
	022	73	1	20JUN1959	10MAY2006	46	female	A	165	60.0	22	caucasian	
	023	50	1	07JUN1966	08MAY2006	39	female	B	170	74.8	26	caucasian	
	024	67	1	06JUL1959	09MAY2006	46	female	B	180	83.7	26	caucasian	
	026	99	1	17SEP1964	15MAY2006	41	male	.	178	101.3	32	caucasian	
	028	85	1	22DEC1959	11MAY2006	46	male	.	184	71.7	21	caucasian	
	029	53	1	23SEP1962	08MAY2006	43	male	.	175	96.8	32	caucasian	
	030	71	1	27MAY1967	10MAY2006	38	female	B	175	67.7	22	caucasian	
	031	33	1	03JAN1951	04MAY2006	55	female	A	164	55.9	21	caucasian	
	032	40	1	06JUN1956	05MAY2006	49	female	B	167	58.6	21	caucasian	
	033	41	1	26DEC1960	05MAY2006	45	male	.	180	79.9	25	caucasian	
	034	34	1	03JAN1969	05MAY2006	37	male	.	170	62.0	21	caucasian	
	035	30	1	12MAY1974	04MAY2006	31	male	.	176	65.7	21	caucasian	
	036	58	1	06SEP1977	09MAY2006	28	female	B	158	58.0	23	caucasian	
	037	49	1	30JAN1967	08MAY2006	39	male	.	181	75.2	23	caucasian	
	038	81	1	02MAY1954	11MAY2006	52	female	B	173	63.3	21	caucasian	
	039	84	1	28SEP1974	11MAY2006	31	male	.	177	71.4	23	caucasian	

[1] Child-bearing potential: A: surgically sterile or post-menopausal B: of child-bearing potential and using appropriate birth control

Demographic data

Group	Subj	Screening number	Centre	Date of birth	Written informed consent was obtained on:	Age [years]	Gender	Child-bearing potential [1]	Height [cm]	Weight [kg]	Body mass index [kg/m²]	Ethnic group	Ethnic group: please specify other
Group 4 (M-)	041	101	1	21FEB1966	15MAY2006	40	male	.	167	63.8	23	caucasian	
	042	26	1	02MAR1978	04MAY2006	28	female	B	158	55.5	22	caucasian	
	043	75	1	18FEB1984	10MAY2006	22	female	B	172	63.5	21	caucasian	
	044	52	1	15DEC1954	08MAY2006	51	male	.	189	77.6	22	caucasian	
	045	69	1	29JUL1958	09MAY2006	47	male	.	186	75.1	22	caucasian	
	047	105	1	29JUN1955	16MAY2006	50	male	.	179	75.0	23	caucasian	
	048	110	1	28AUG1972	17MAY2006	33	male	.	181	77.8	24	caucasian	
	049	97	1	05JAN1963	15MAY2006	43	female	B	163	53.4	20	caucasian	
	050	98	1	25AUG1979	15MAY2006	26	female	B	176	65.3	21	caucasian	
	051	107	1	23DEC1953	16MAY2006	52	male	.	183	94.6	28	caucasian	
	052	108	1	02FEB1960	16MAY2006	46	female	B	173	76.5	26	caucasian	
	053	111	1	14APR1963	17MAY2006	43	female	B	162	60.0	23	caucasian	
	055	119	1	02JAN1965	18MAY2006	41	male	.	174	69.3	23	caucasian	
	056	123	1	01JUN1951	18MAY2006	54	male	.	179	69.4	22	caucasian	
	057	124	1	12JUL1968	18MAY2006	37	male	.	174	65.4	22	caucasian	
	058	126	1	29MAR1959	19MAY2006	47	female	B	176	58.1	19	caucasian	
	059	127	1	21JUL1962	19MAY2006	43	female	B	165	61.2	22	caucasian	
	060	130	1	01FEB1960	19MAY2006	46	female	B	162	53.8	20	caucasian	
	061	131	1	17DEC1981	19MAY2006	24	female	B	164	61.8	23	caucasian	
	063	120	1	02OCT1962	18MAY2006	43	female	B	159	81.2	32	caucasian	
	064	139	1	02APR1963	22MAY2006	43	female	B	167	51.4	18	caucasian	
	065	159	1	02NOV1954	24MAY2006	51	female	B	165	75.2	28	caucasian	
	066	154	1	11FEB1971	24MAY2006	35	female	B	163	51.2	19	caucasian	
	073	169	1	30SEP1962	30MAY2006	43	female	B	171	71.8	25	caucasian	
	074	164	1	01MAR1964	29MAY2006	42	male	.	186	94.0	27	caucasian	
	075	162	1	07NOV1981	29MAY2006	24	female	B	167	60.7	22	caucasian	
	077	138	1	15DEC1955	22MAY2006	50	male	.	193	88.5	24	caucasian	
	078	133	1	28FEB1959	19MAY2006	47	male	.	185	95.1	28	caucasian	

[1]Child-bearing potential: A: surgically sterile or post-menopausal B: of child-bearing potential and using appropriate birth control

Demographic data

Group	Subj	Screening number	Centre	Date of birth	Written informed consent was obtained on:	Age [years]	Gender	Child-bearing potential [1]	Height [cm]	Weight [kg]	Body mass index [kg/m²]	Ethnic group	Ethnic group: please specify other
Group 4 (M-)	081	91	1	18MAR1956	12MAY2006	50	male	.	183	85.9	26	caucasian	
	083	117	1	30JAN1965	18MAY2006	41	female	B	167	61.7	22	caucasian	
	086	177	1	01JAN1973	31MAY2006	33	female	B	162	58.8	22	caucasian	
	089	195	1	16MAR1980	02JUN2006	26	female	B	162	59.7	23	caucasian	
	091	132	1	28MAY1959	19MAY2006	46	male	.	195	108.6	29	caucasian	
	092	186	1	17SEP1967	01JUN2006	38	female	B	153	60.5	26	caucasian	
	097	183	1	15MAR1958	31MAY2006	48	female	B	168	88.9	31	caucasian	
	100	219	1	05NOV1963	07JUN2006	42	female	B	169	60.0	21	caucasian	
	106	221	1	21JAN1958	07JUN2006	48	male	.	181	78.5	24	caucasian	
	108	217	1	30DEC1965	07JUN2006	40	male	.	182	82.0	25	caucasian	
	111	243	1	26OCT1950	09JUN2006	55	male	.	182	86.8	26	caucasian	
	113	208	1	28AUG1967	06JUN2006	38	male	.	177	83.3	27	caucasian	
	115	254	1	22AUG1979	13JUN2006	26	female	B	171	64.6	22	caucasian	
	116	257	1	21MAY1973	13JUN2006	33	male	.	180	70.0	22	caucasian	
	117	259	1	23JUN1978	13JUN2006	27	male	.	183	80.0	24	caucasian	
	119	256	1	11MAR1971	13JUN2006	35	male	.	183	81.2	24	caucasian	
	122	267	1	04APR1969	14JUN2006	37	male	.	178	89.7	28	caucasian	
	123	362	1	20SEP1972	08JUN2006	33	male	.	187	83.9	24	caucasian	
	127	239	1	01JAN1972	09JUN2006	34	female	A	161	62.9	24	caucasian	
	132	236	1	12MAY1959	09JUN2006	47	female	A	163	68.1	26	caucasian	
	134	222	1	11FEB1968	07JUN2006	38	female	B	170	59.6	21	caucasian	
	139	286	1	28JUN1954	19JUN2006	51	male	.	186	102.4	30	caucasian	
	145	332	1	17DEC1961	23JUN2006	44	female	B	164	66.4	25	caucasian	
	146	376	1	27DEC1966	22MAY2006	39	female	B	163	62.8	24	caucasian	
	150	331	1	25JUL1958	23JUN2006	47	female	B	170	62.3	22	caucasian	
	151	287	1	22FEB1957	19JUN2006	49	male	.	183	84.9	25	caucasian	
	153	260	1	31JUL1951	13JUN2006	54	female	A	168	69.2	25	caucasian	
	157	328	1	29DEC1959	23JUN2006	46	female	B	173	56.8	19	caucasian	

[1]Child-bearing potential: A: surgically sterile or post-menopausal B: of child-bearing potential and using appropriate birth control

Demographic data

Group	Subj	Screening number	Centre	Date of birth	Written informed consent was obtained on:	Age [years]	Gender	Child-bearing potential [1]	Height [cm]	Weight [kg]	Body mass index [kg/m²]	Ethnic group	Ethnic group: please specify other
Group 4 (M-)	160	339	1	12MAR1982	26JUN2006	24	female	B	166	60.4	22	caucasian	
	162	308	1	29JUN1963	21JUN2006	42	female	B	159	57.2	23	caucasian	
	168	294	1	01FEB1954	20JUN2006	52	female	A	156	73.7	30	caucasian	
	170	246	1	20MAY1970	12JUN2006	36	male	.	171	80.2	27	caucasian	
	173	297	1	27NOV1963	20JUN2006	42	male	.	190	87.8	24	caucasian	
	174	232	1	16SEP1963	08JUN2006	42	female	B	160	60.2	24	caucasian	
	178	252	1	09MAR1960	13JUN2006	46	male	.	180	80.9	25	caucasian	
	181	278	1	12MAY1974	19JUN2006	32	male	.	186	77.9	23	caucasian	
	183	370	1	22DEC1953	28JUN2006	52	male	.	188	84.6	24	caucasian	
	185	290	1	18SEP1979	20JUN2006	26	female	B	175	57.3	19	caucasian	
	193	416	1	14APR1951	23MAY2006	55	female	B	163	56.8	21	caucasian	
	194	284	1	11FEB1951	19JUN2006	55	female	A	170	78.3	27	caucasian	
	195	364	1	12FEB1967	28JUN2006	39	female	B	169	60.2	21	caucasian	
	206	343	1	07JAN1972	26JUN2006	34	male	.	184	106.6	31	caucasian	
	207	406	1	07AUG1961	04JUL2006	44	male	.	187	89.1	25	caucasian	
	208	288	1	04NOV1960	20JUN2006	45	female	A	160	72.7	28	caucasian	
	210	435	1	11MAY1960	07JUL2006	46	female	B	160	74.6	29	caucasian	
	211	392	1	27MAR1966	03JUL2006	40	female	B	168	92.6	33	caucasian	
	212	447	1	01NOV1967	10JUL2006	38	male	.	188	79.0	22	caucasian	
	214	446	1	11APR1955	31MAY2006	51	male	.	177	92.7	30	caucasian	
	222	442	1	09SEP1971	07JUL2006	34	female	B	171	64.3	22	caucasian	
	224	289	1	22MAY1960	20JUN2006	46	male	.	183	108.0	32	caucasian	
	230	369	1	01OCT1969	28JUN2006	36	female	B	169	52.9	19	caucasian	
	231	475	1	23MAR1976	13JUL2006	30	male	.	186	75.0	22	caucasian	
	234	379	1	10MAR1967	29JUN2006	39	male	.	173	72.6	24	caucasian	
	235	452	1	01AUG1976	11JUL2006	29	female	B	173	61.4	21	caucasian	
	238	334	1	24FEB1980	23JUN2006	26	female	B	166	80.4	29	caucasian	
	240	317	1	18JAN1973	22JUN2006	33	male	.	158	60.9	24	caucasian	

[1]Child-bearing potential: A: surgically sterile or post-menopausal B: of child-bearing potential and using appropriate birth control

Demographic data

Group	Subj	Screening number	Centre	Date of birth	Written informed consent was obtained on:	Age [years]	Gender	Child-bearing potential [1]	Height [cm]	Weight [kg]	Body mass index [kg/ m²]	Ethnic group	Ethnic group: please specify other
Group 4 (M-)	242	459	1	18JUL1951	11JUL2006	54	female	A	156	57.7	24	caucasian	
	243	322	1	14APR1970	22JUN2006	36	female	B	160	57.8	23	caucasian	
	244	462	1	30MAY1957	12JUL2006	49	female	A	157	60.0	24	caucasian	
	245	385	1	11JAN1968	30JUN2006	38	male	.	188	88.8	25	caucasian	
	246	479	1	18JUN1952	14JUL2006	54	male	.	180	96.7	30	caucasian	
	249	464	1	31JAN1956	12JUL2006	50	male	.	170	83.8	29	caucasian	
	252	329	1	23APR1965	23JUN2006	41	female	B	177	88.3	28	caucasian	
	257	399	1	07DEC1976	03JUL2006	29	female	B	172	60.1	20	caucasian	
	259	478	1	09FEB1967	19MAY2006	39	male	.	181	86.9	27	caucasian	
	261	451	1	17APR1960	11JUL2006	46	male	.	180	90.9	28	caucasian	
	262	407	1	27AUG1978	04JUL2006	27	female	B	168	71.5	25	caucasian	
	267	476	1	09NOV1965	13JUL2006	40	female	B	162	61.7	24	caucasian	
	269	499	1	27APR1964	18JUL2006	42	female	A	164	63.3	24	caucasian	
	270	431	1	18MAY1965	06JUL2006	41	female	B	169	83.3	29	caucasian	
	271	474	1	13APR1964	13JUL2006	42	female	B	155	75.2	31	caucasian	
	272	372	1	05JUL1967	28JUN2006	38	male	.	188	95.0	27	caucasian	
	275	485	1	25APR1966	17JUL2006	40	male	.	164	90.0	33	caucasian	
	277	448	1	22AUG1959	10JUL2006	46	male	.	189	110.2	31	caucasian	
	279	529	1	31DEC1957	21JUL2006	48	male	.	175	77.9	25	caucasian	
	286	473	1	29MAR1960	13JUL2006	46	male	.	187	89.0	25	caucasian	
	289	545	1	03NOV1953	24JUL2006	52	male	.	186	135.4	39	caucasian	
	291	440	1	20APR1970	07JUL2006	36	female	B	166	59.5	22	caucasian	
	293	500	1	23JAN1966	18JUL2006	40	female	A	177	77.0	25	caucasian	
	295	498	1	02MAY1961	18JUL2006	45	female	B	164	49.1	18	caucasian	
	298	523	1	21JAN1970	21JUL2006	36	male	.	185	83.5	24	caucasian	
	299	559	1	19OCT1973	26JUL2006	32	male	.	179	75.9	24	caucasian	
	302	455	1	18MAY1968	11JUL2006	38	female	B	167	71.9	26	caucasian	
	311	548	1	13JUL1961	25JUL2006	45	female	B	173	67.7	23	caucasian	

[1]Child-bearing potential: A: surgically sterile or post-menopausal B: of child-bearing potential and using appropriate birth control

Demographic data

Group	Subj	Screening number	Centre	Date of birth	Written informed consent was obtained on:	Age [years]	Gender	Child-bearing potential [1]	Height [cm]	Weight [kg]	Body mass index [kg/m²]	Ethnic group	Ethnic group: please specify other
Group 4 (M-)	314	576	1	30APR1960	27JUL2006	46	female	B	163	56.0	21	caucasian	
	315	426	1	18AUG1962	06JUL2006	43	male	.	183	84.3	25	caucasian	
	320	588	1	27MAY1966	31JUL2006	40	male	.	165	67.3	25	caucasian	
	324	508	1	05MAR1955	08JUN2006	51	female	B	171	70.6	24	caucasian	
	325	564	1	22APR1954	26JUL2006	52	female	B	162	54.7	21	caucasian	
	326	520	1	02JUL1962	09MAY2006	43	male	.	181	81.1	25	caucasian	
	327	580	1	10JUL1956	28JUL2006	50	female	A	162	46.1	18	caucasian	
	332	483	1	27FEB1969	17JUL2006	37	male	.	191	87.9	24	caucasian	
	333	524	1	06JUN1966	04MAY2006	39	female	B	176	80.7	26	caucasian	
	335	543	1	15JAN1966	24JUL2006	40	female	B	178	63.1	20	caucasian	
	336	531	1	10JUN1970	21JUL2006	36	male	.	168	81.9	29	other	CAUCASIAN/ INDIAN
	338	556	1	04OCT1967	26JUL2006	38	female	B	167	86.5	31	caucasian	
	344	542	1	01FEB1970	24JUL2006	36	female	B	159	85.6	34	caucasian	
	345	635	1	21JAN1967	07AUG2006	39	male	.	182	70.6	21	caucasian	
	346	614	1	04JAN1963	03AUG2006	43	female	B	163	66.7	25	caucasian	
	347	555	1	22MAY1957	22JUN2006	49	female	B	171	57.6	20	caucasian	
	349	633	1	15JUL1967	07AUG2006	39	female	B	168	114.8	41	caucasian	
	358	561	1	06AUG1959	26JUL2006	46	female	B	168	66.4	24	caucasian	
	359	622	1	31OCT1952	04AUG2006	53	female	A	165	62.3	23	caucasian	
	363	620	1	15APR1971	03AUG2006	35	female	B	167	52.7	19	caucasian	
	365	608	1	05FEB1956	02AUG2006	50	female	B	155	56.5	24	caucasian	
	368	649	1	13OCT1972	08AUG2006	33	female	B	158	61.2	25	caucasian	
	369	546	1	08APR1965	14JUN2006	41	female	B	161	84.5	33	caucasian	
	370	609	1	29MAR1964	02AUG2006	42	female	B	161	57.8	22	caucasian	
	371	549	1	21DEC1957	25JUL2006	48	female	B	173	70.6	24	caucasian	
	373	677	1	09JAN1958	11AUG2006	48	female	B	168	63.3	22	caucasian	
	378	690	1	25FEB1964	14AUG2006	42	male	.	178	79.6	25	caucasian	

[1] Child-bearing potential: A: surgically sterile or post-menopausal B: of child-bearing potential and using appropriate birth control

Demographic data

Group	Subj	Screening number	Centre	Date of birth	Written informed consent was obtained on:	Age [years]	Gender	Child-bearing potential [1]	Height [cm]	Weight [kg]	Body mass index [kg/m²]	Ethnic group	Ethnic group: please specify other
Group 4 (M-)	379	668	1	24MAY1963	10AUG2006	43	female	A	170	61.7	21	caucasian	
	380	652	1	21OCT1959	08AUG2006	46	male	.	182	103.3	31	caucasian	
	381	651	1	03FEB1960	08AUG2006	46	male	.	180	82.8	26	caucasian	
	382	643	1	23AUG1978	08AUG2006	27	female	B	164	70.6	26	caucasian	
	388	678	1	02JAN1955	11AUG2006	51	male	.	168	85.8	30	caucasian	
	395	672	1	27JAN1952	10AUG2006	54	male	.	173	82.8	28	caucasian	
	396	674	1	15APR1973	11AUG2006	33	female	B	163	58.2	22	caucasian	
	397	682	1	04SEP1968	11AUG2006	37	female	B	170	56.8	20	caucasian	
	401	569	1	23AUG1968	27JUL2006	37	female	B	175	82.9	27	caucasian	
	404	686	1	03MAR1978	14AUG2006	28	female	B	165	57.2	21	caucasian	
	407	702	1	05NOV1961	16AUG2006	44	female	A	160	52.9	21	caucasian	
	408	670	1	14FEB1956	10AUG2006	50	female	A	168	102.1	36	caucasian	
	409	658	1	18NOV1967	09AUG2006	38	female	B	158	63.5	25	caucasian	
	410	711	1	03DEC1959	17AUG2006	46	female	B	167	73.0	26	caucasian	
	411	683	1	16JAN1959	11AUG2006	47	female	B	164	53.8	20	caucasian	
	412	692	1	19NOV1957	14AUG2006	48	female	B	173	84.0	28	caucasian	
	413	619	1	22DEC1963	03AUG2006	42	male	.	177	68.4	22	caucasian	
	414	718	1	14NOV1965	17AUG2006	40	female	B	163	50.0	19	caucasian	
	419	732	1	03MAR1962	18AUG2006	44	female	A	168	67.8	24	caucasian	
	420	703	1	14OCT1961	16AUG2006	44	male	.	184	89.4	26	caucasian	
	421	734	1	12OCT1963	18AUG2006	42	female	A	169	60.5	21	caucasian	
	422	735	1	08JUN1961	18AUG2006	45	female	A	166	72.5	26	caucasian	
	430	581	1	02JUL1968	28JUL2006	38	male	.	179	75.3	24	caucasian	
	439	585	1	25NOV1953	31JUL2006	52	female	B	158	64.5	26	caucasian	
	451	721	1	24JUN1959	17AUG2006	47	male	.	185	85.8	25	caucasian	
	452	731	1	16AUG1958	18AUG2006	48	male	.	165	67.6	25	caucasian	
	524	771	1	08JUN1965	23AUG2006	41	male	.	171	82.2	28	caucasian	

[1]Child-bearing potential: A: surgically sterile or post-menopausal B: of child-bearing potential and using appropriate birth control

16.2.4.2. History of smallpox vaccination

			Presence of smallpox vaccination		Smallpox vaccination history		
Group	Subj	Screening number	History of smallpox vaccination?	Presence of typical vaccination scar?	ID	Vaccination date	Vaccine
Group 1 (MM)	070	142	no	.	.		
	080	152	no	.	.		
	084	149	no	.	.		
	088	174	no	.	.		
	093	201	no	.	.		
	096	203	no	.	.		
	102	216	no	.	.		
	103	224	no	.	.		
	107	227	no	.	.		
	129	272	no	.	.		
	137	283	no	.	.		
	140	185	no	.	.		
	143	313	no	.	.		
	144	320	no	.	.		
	148	293	no	.	.		
	154	367	no	.	.		
	156	360	no	.	.		
	161	229	no	.	.		
	176	377	no	.	.		
	180	380	no	.	.		
	182	359	no	.	.		
	189	312	no	.	.		
	196	384	no	.	.		
	199	366	no	.	.		
	204	398	no	.	.		
	205	394	no	.	.		
	215	411	no	.	.		
	216	417	no	.	.		
	218	403	no	.	.		

History of smallpox vaccination

			Presence of smallpox vaccination		Smallpox vaccination history		
Group	Subj	Screening number	History of smallpox vaccination?	Presence of typical vaccination scar?	ID	Vaccination date	Vaccine
Group 1 (MM)	225	414	no	.	.		
	226	341	no	.	.		
	227	381	no	.	.		
	236	353	no	.	.		
	237	437	no	.	.		
	248	445	no	.	.		
	254	424	no	.	.		
	255	463	no	.	.		
	256	472	no	.	.		
	268	518	no	.	.		
	274	404	no	.	.		
	281	532	no	.	.		
	283	507	no	.	.		
	287	519	no	.	.		
	297	551	no	.	.		
	304	482	no	.	.		
	306	574	no	.	.		
	317	488	no	.	.		
	318	538	no	.	.		
	323	471	no	.	.		
	330	611	no	.	.		
	334	494	no	.	.		
	342	596	no	.	.		
	353	575	no	.	.		
	356	632	no	.	.		
	386	648	no	.	.		
	398	698	no	.	.		
	400	587	no	.	.		
	415	717	no	.	.		

History of smallpox vaccination

			Presence of smallpox vaccination		Smallpox vaccination history		
Group	Subj	Screening number	History of smallpox vaccination?	Presence of typical vaccination scar?	ID	Vaccination date	Vaccine
Group 1 (MM)	416	593	no	.	.		
	417	671	no	.	.		
	427	723	no	.	.		
	428	570	no	.	.		
	429	762	no	.	.		
	432	705	no	.	.		
	433	766	no	.	.		
	438	638	no	.	.		
	441	773	no	.	.		
	442	688	no	.	.		
	447	806	no	.	.		
	448	747	no	.	.		
	449	782	no	.	.		
	455	687	no	.	.		
	457	795	no	.	.		
	459	790	no	.	.		
	462	743	no	.	.		
	471	831	no	.	.		
	472	822	no	.	.		
	473	833	no	.	.		
	479	819	no	.	.		
	482	804	no	.	.		
	483	854	no	.	.		
	484	817	no	.	.		
	488	843	no	.	.		
	489	700	no	.	.		
	495	816	no	.	.		
	497	844	no	.	.		
	502	857	no	.	.		

History of smallpox vaccination

Group	Subj	Screening number	Presence of smallpox vaccination: History of smallpox vaccination?	Presence of smallpox vaccination: Presence of typical vaccination scar?	Smallpox vaccination history: ID	Smallpox vaccination history: Vaccination date	Smallpox vaccination history: Vaccine
Group 1 (MM)	505	823	no	.	.		
	510	899	no	.	.		
	512	887	no	.	.		
	518	870	no	.	.		
	519	872	no	.	.		
	521	802	no	.	.		
	525	886	no	.	.		
	532	890	no	.	.		
	534	876	no	.	.		
	537	928	no	.	.		
	543	877	no	.	.		
	545	918	no	.	.		
	555	947	no	.	.		
	556	953	no	.	.		
	557	938	no	.	.		
	562	964	no	.	.		
	565	965	no	.	.		
	567	962	no	.	.		
	568	992	no	.	.		
	571	907	no	.	.		
	572	991	no	.	.		
	574	889	no	.	.		
	582	951	no	.	.		
	583	949	no	.	.		
	586	990	no	.	.		
	587	1002	no	.	.		
	595	1026	no	.	.		
	597	1004	no	.	.		
	604	1021	no	.	.		

History of smallpox vaccination

Group	Subj	Screening number	Presence of smallpox vaccination: History of smallpox vaccination?	Presence of smallpox vaccination: Presence of typical vaccination scar?	Smallpox vaccination history: ID	Smallpox vaccination history: Vaccination date	Smallpox vaccination history: Vaccine
Group 1 (MM)	605	972	no	.	.		
	607	1049	no	.	.		
	609	1018	no	.	.		
	610	959	no	.	.		
	612	968	no	.	.		
	616	915	no	.	.		
	625	1007	no	.	.		
	627	1062	no	.	.		
	629	1073	no	.	.		
	634	1072	no	.	.		
	636	1037	no	.	.		
	637	908	no	.	.		
	641	933	no	.	.		
	643	1081	no	.	.		
	646	1031	no	.	.		
	648	946	no	.	.		
	649	961	no	.	.		
	654	1099	no	.	.		
	655	1098	no	.	.		
	662	1119	no	.	.		
	663	1112	no	.	.		
	666	1089	no	.	.		
	669	1074	no	.	.		
	670	1090	no	.	.		
	673	1054	no	.	.		
	676	1124	no	.	.		
	678	1131	no	.	.		
	683	1052	no	.	.		
	688	1143	no	.	.		

History of smallpox vaccination

Group	Subj	Screening number	Presence of smallpox vaccination: History of smallpox vaccination?	Presence of smallpox vaccination: Presence of typical vaccination scar?	Smallpox vaccination history: ID	Smallpox vaccination history: Vaccination date	Smallpox vaccination history: Vaccine
Group 1 (MM)	690	1111	no	.	.		
	691	1123	no	.	.		
	697	1163	no	.	.		
	698	1151	no	.	.		
	701	1057	no	.	.		
	703	1153	no	.	.		
	705	1197	no	.	.		
	707	1158	no	.	.		
	716	1159	no	.	.		
	719	1190	no	.	.		
	721	1178	no	.	.		
	725	1194	no	.	.		
	729	1128	no	.	.		
	730	1207	no	.	.		
	733	1229	no	.	.		
	734	1120	no	.	.		
	735	1233	no	.	.		
	742	1191	no	.	.		
	743	1226	no	.	.		
	746	1172	no	.	.		
	748	1169	no	.	.		
	749	1216	no	.	.		
	750	1243	no	.	.		
	758	1179	no	.	.		
	765	1222	no	.	.		
	768	1250	no	.	.		
	771	1171	no	.	.		
	774	1246	no	.	.		
	776	1125	no	.	.		

History of smallpox vaccination

			Presence of smallpox vaccination		Smallpox vaccination history		
Group	Subj	Screening number	History of smallpox vaccination?	Presence of typical vaccination scar?	ID	Vaccination date	Vaccine
Group 1 (MM)	778	1272	no	.	.		
	779	1262	no	.	.		
	780	1220	no	.	.		
	792	1114	no	.	.		
	795	1304	no	.	.		
	797	1130	no	.	.		
	803	1281	no	.	.		
	805	1223	no	.	.		
	809	1248	no	.	.		
Group 2 (MP)	069	144	no	.	.		
	082	199	no	.	.		
	085	158	no	.	.		
	090	192	no	.	.		
	095	200	no	.	.		
	104	223	no	.	.		
	105	225	no	.	.		
	114	231	no	.	.		
	125	309	no	.	.		
	128	245	no	.	.		
	130	253	no	.	.		
	136	165	no	.	.		
	141	181	no	.	.		
	142	270	no	.	.		
	164	303	no	.	.		
	165	300	no	.	.		
	167	348	no	.	.		
	169	378	no	.	.		

History of smallpox vaccination

			Presence of smallpox vaccination		Smallpox vaccination history		
Group	Subj	Screening number	History of smallpox vaccination?	Presence of typical vaccination scar?	ID	Vaccination date	Vaccine
Group 2 (MP)	171	373	no	.	.		
	177	347	no	.	.		
	184	330	no	.	.		
	187	349	no	.	.		
	188	352	no	.	.		
	192	250	no	.	.		
	198	363	no	.	.		
	202	327	no	.	.		
	203	393	no	.	.		
	209	419	no	.	.		
	213	387	no	.	.		
	217	346	no	.	.		
	220	316	no	.	.		
	228	430	no	.	.		
	229	413	no	.	.		
	247	468	no	.	.		
	250	466	no	.	.		
	251	337	no	.	.		
	253	432	no	.	.		
	273	401	no	.	.		
	278	465	no	.	.		
	284	487	no	.	.		
	285	490	no	.	.		
	290	525	no	.	.		
	292	444	no	.	.		
	301	582	no	.	.		
	312	579	no	.	.		
	313	642	no	.	.		
	316	562	no	.	.		

History of smallpox vaccination

Group	Subj	Screening number	Presence of smallpox vaccination: History of smallpox vaccination?	Presence of smallpox vaccination: Presence of typical vaccination scar?	Smallpox vaccination history: ID	Smallpox vaccination history: Vaccination date	Smallpox vaccination history: Vaccine
Group 2 (MP)	328	560	no	.	.		
	329	547	no	.	.		
	341	527	no	.	.		
	355	605	no	.	.		
	361	616	no	.	.		
	362	607	no	.	.		
	364	629	no	.	.		
	372	644	no	.	.		
	376	623	no	.	.		
	385	598	no	.	.		
	389	625	no	.	.		
	391	599	no	.	.		
	406	701	no	.	.		
	423	739	no	.	.		
	425	664	no	.	.		
	426	740	no	.	.		
	435	730	no	.	.		
	436	760	no	.	.		
	445	737	no	.	.		
	446	796	no	.	.		
	453	805	no	.	.		
	458	808	no	.	.		
	460	811	no	.	.		
	461	793	no	.	.		
	466	815	no	.	.		
	470	663	no	.	.		
	474	835	no	.	.		
	477	812	no	.	.		
	478	838	no	.	.		

History of smallpox vaccination

			Presence of smallpox vaccination		Smallpox vaccination history		
Group	Subj	Screening number	History of smallpox vaccination?	Presence of typical vaccination scar?	ID	Vaccination date	Vaccine
Group 2 (MP)	480	820	no	.	.		
	481	810	no	.	.		
	486	803	no	.	.		
	487	788	no	.	.		
	490	834	no	.	.		
	491	825	no	.	.		
	492	840	no	.	.		
	493	801	no	.	.		
	499	849	no	.	.		
	504	860	no	.	.		
	506	783	no	.	.		
	511	869	no	.	.		
	515	878	no	.	.		
	516	896	no	.	.		
	520	874	no	.	.		
	522	863	no	.	.		
	526	900	no	.	.		
	527	903	no	.	.		
	528	924	no	.	.		
	529	756	no	.	.		
	530	883	no	.	.		
	535	911	no	.	.		
	536	926	no	.	.		
	540	950	no	.	.		
	542	875	no	.	.		
	544	893	no	.	.		
	548	931	no	.	.		
	550	941	no	.	.		
	552	861	no	.	.		

History of smallpox vaccination

Group	Subj	Screening number	Presence of smallpox vaccination: History of smallpox vaccination?	Presence of smallpox vaccination: Presence of typical vaccination scar?	Smallpox vaccination history: ID	Smallpox vaccination history: Vaccination date	Smallpox vaccination history: Vaccine
Group 2 (MP)	553	936	no	.	.		
	559	895	no	.	.		
	561	848	no	.	.		
	563	966	no	.	.		
	573	971	no	.	.		
	576	904	no	.	.		
	579	987	no	.	.		
	584	910	no	.	.		
	585	982	no	.	.		
	588	1020	no	.	.		
	590	1033	no	.	.		
	591	985	no	.	.		
	598	865	no	.	.		
	602	1027	no	.	.		
	614	1047	no	.	.		
	615	1032	no	.	.		
	619	1012	no	.	.		
	622	994	no	.	.		
	623	1025	no	.	.		
	624	944	no	.	.		
	626	1060	no	.	.		
	630	1075	no	.	.		
	633	930	no	.	.		
	635	1092	no	.	.		
	639	1000	no	.	.		
	642	1080	no	.	.		
	647	996	no	.	.		
	660	1105	no	.	.		
	664	1107	no	.	.		

History of smallpox vaccination

Group	Subj	Screening number	Presence of smallpox vaccination: History of smallpox vaccination?	Presence of smallpox vaccination: Presence of typical vaccination scar?	Smallpox vaccination history: ID	Smallpox vaccination history: Vaccination date	Smallpox vaccination history: Vaccine
Group 2 (MP)	667	1083	no	.	.		
	668	1058	no	.	.		
	672	1046	no	.	.		
	674	1142	no	.	.		
	675	1140	no	.	.		
	677	1013	no	.	.		
	679	1022	no	.	.		
	680	1043	no	.	.		
	693	1126	no	.	.		
	694	1141	no	.	.		
	696	1156	no	.	.		
	699	1136	no	.	.		
	706	1170	no	.	.		
	708	1157	no	.	.		
	709	1188	no	.	.		
	710	1118	no	.	.		
	712	1186	no	.	.		
	713	1149	no	.	.		
	714	1144	no	.	.		
	722	1189	no	.	.		
	731	1211	no	.	.		
	732	1109	no	.	.		
	736	1041	no	.	.		
	738	1225	no	.	.		
	747	1234	no	.	.		
	752	1196	no	.	.		
	754	1249	no	.	.		
	757	1094	no	.	.		
	759	1256	no	.	.		

History of smallpox vaccination

			Presence of smallpox vaccination		Smallpox vaccination history		
Group	Subj	Screening number	History of smallpox vaccination?	Presence of typical vaccination scar?	ID	Vaccination date	Vaccine
Group 2 (MP)	761	1245	no	.	.		
	763	1078	no	.	.		
	764	1252	no	.	.		
	770	1146	no	.	.		
	777	1205	no	.	.		
	781	1269	no	.	.		
	783	1129	no	.	.		
	785	1113	no	.	.		
	787	1227	no	.	.		
	789	1182	no	.	.		
	790	1231	no	.	.		
	794	1277	no	.	.		
	798	1152	no	.	.		
	800	1275	no	.	.		
	804	1289	no	.	.		
	806	1206	no	.	.		
	807	1213	no	.	.		
	815	1271	no	.	.		
Group 3 (PP)	067	155	no	.	.		
	068	122	no	.	.		
	072	140	no	.	.		
	094	163	no	.	.		
	099	214	no	.	.		
	101	211	no	.	.		
	110	220	no	.	.		
	112	324	no	.	.		
	120	248	no	.	.		

History of smallpox vaccination

			Presence of smallpox vaccination		Smallpox vaccination history		
Group	Subj	Screening number	History of smallpox vaccination?	Presence of typical vaccination scar?	ID	Vaccination date	Vaccine
Group 3 (PP)	126	268	no	.	.		
	131	241	no	.	.		
	135	173	no	.	.		
	138	282	no	.	.		
	147	340	no	.	.		
	149	344	no	.	.		
	152	215	no	.	.		
	158	291	no	.	.		
	159	295	no	.	.		
	163	304	no	.	.		
	166	298	no	.	.		
	172	351	no	.	.		
	175	357	no	.	.		
	186	321	no	.	.		
	190	233	no	.	.		
	191	314	no	.	.		
	200	396	no	.	.		
	201	368	no	.	.		
	221	402	no	.	.		
	232	412	no	.	.		
	233	429	no	.	.		
	239	395	no	.	.		
	241	439	no	.	.		
	265	383	no	.	.		
	266	510	no	.	.		
	276	513	no	.	.		
	288	526	no	.	.		
	294	511	no	.	.		
	296	423	no	.	.		

History of smallpox vaccination

Group	Subj	Screening number	Presence of smallpox vaccination: History of smallpox vaccination?	Presence of smallpox vaccination: Presence of typical vaccination scar?	Smallpox vaccination history: ID	Smallpox vaccination history: Vaccination date	Smallpox vaccination history: Vaccine
Group 3 (PP)	300	469	no	.	.		
	303	492	no	.	.		
	305	522	no	.	.		
	307	577	no	.	.		
	308	654	no	.	.		
	309	427	no	.	.		
	310	491	no	.	.		
	321	567	no	.	.		
	337	572	no	.	.		
	339	539	no	.	.		
	348	550	no	.	.		
	350	603	no	.	.		
	357	606	no	.	.		
	360	597	no	.	.		
	367	610	no	.	.		
	374	591	no	.	.		
	384	639	no	.	.		
	387	617	no	.	.		
	390	586	no	.	.		
	392	662	no	.	.		
	394	667	no	.	.		
	399	699	no	.	.		
	405	694	no	.	.		
	431	641	no	.	.		
	437	615	no	.	.		
	440	722	no	.	.		
	443	715	no	.	.		
	444	714	no	.	.		
	450	748	no	.	.		

History of smallpox vaccination

			Presence of smallpox vaccination		Smallpox vaccination history		
Group	Subj	Screening number	History of smallpox vaccination?	Presence of typical vaccination scar?	ID	Vaccination date	Vaccine
Group 3 (PP)	454	934	no	.	.		
	456	713	no	.	.		
	463	789	no	.	.		
	464	764	no	.	.		
	465	637	no	.	.		
	467	841	no	.	.		
	468	792	no	.	.		
	469	828	no	.	.		
	475	696	no	.	.		
	476	836	no	.	.		
	485	862	no	.	.		
	494	791	no	.	.		
	496	845	no	.	.		
	498	853	no	.	.		
	500	837	no	.	.		
	501	858	no	.	.		
	503	830	no	.	.		
	507	851	no	.	.		
	508	882	no	.	.		
	509	879	no	.	.		
	513	832	no	.	.		
	514	894	no	.	.		
	517	855	no	.	.		
	523	842	no	.	.		
	531	921	no	.	.		
	533	892	no	.	.		
	538	909	no	.	.		
	539	912	no	.	.		
	541	956	no	.	.		

History of smallpox vaccination

Group	Subj	Screening number	Presence of smallpox vaccination: History of smallpox vaccination?	Presence of smallpox vaccination: Presence of typical vaccination scar?	Smallpox vaccination history: ID	Smallpox vaccination history: Vaccination date	Smallpox vaccination history: Vaccine
Group 3 (PP)	546	943	no	.	.		
	547	922	no	.	.		
	549	940	no	.	.		
	551	935	no	.	.		
	554	919	no	.	.		
	560	945	no	.	.		
	564	963	no	.	.		
	566	958	no	.	.		
	570	948	no	.	.		
	577	884	no	.	.		
	578	888	no	.	.		
	580	979	no	.	.		
	581	916	no	.	.		
	589	1003	no	.	.		
	592	969	no	.	.		
	593	1005	no	.	.		
	594	1009	no	.	.		
	596	980	no	.	.		
	599	995	no	.	.		
	600	1029	no	.	.		
	601	975	no	.	.		
	603	1017	no	.	.		
	611	1045	no	.	.		
	613	1042	no	.	.		
	617	1014	no	.	.		
	620	1093	no	.	.		
	628	1065	no	.	.		
	631	1079	no	.	.		
	632	1095	no	.	.		

History of smallpox vaccination

			Presence of smallpox vaccination		Smallpox vaccination history		
Group	Subj	Screening number	History of smallpox vaccination?	Presence of typical vaccination scar?	ID	Vaccination date	Vaccine
Group 3 (PP)	638	1028	no	.	.		
	640	1038	no	.	.		
	644	1085	no	.	.		
	650	1117	no	.	.		
	651	1011	no	.	.		
	652	1023	no	.	.		
	653	1069	no	.	.		
	656	1059	no	.	.		
	657	1103	no	.	.		
	658	1035	no	.	.		
	661	1082	no	.	.		
	671	1015	no	.	.		
	682	1061	no	.	.		
	684	1106	no	.	.		
	685	1001	no	.	.		
	686	1145	no	.	.		
	687	1164	no	.	.		
	689	1133	no	.	.		
	695	1134	no	.	.		
	700	1154	no	.	.		
	702	1135	no	.	.		
	704	1166	no	.	.		
	711	1193	no	.	.		
	715	1150	no	.	.		
	717	1160	no	.	.		
	718	1184	no	.	.		
	723	1167	no	.	.		
	724	1192	no	.	.		
	726	1195	no	.	.		

History of smallpox vaccination

			Presence of smallpox vaccination		Smallpox vaccination history		
Group	Subj	Screening number	History of smallpox vaccination?	Presence of typical vaccination scar?	ID	Vaccination date	Vaccine
Group 3 (PP)	727	1175	no	.	.		
	728	1139	no	.	.		
	737	1138	no	.	.		
	739	1203	no	.	.		
	740	1221	no	.	.		
	741	1183	no	.	.		
	744	1088	no	.	.		
	745	1235	no	.	.		
	751	1084	no	.	.		
	755	1261	no	.	.		
	756	1239	no	.	.		
	760	1264	no	.	.		
	762	1068	no	.	.		
	766	1251	no	.	.		
	767	1209	no	.	.		
	769	1266	no	.	.		
	773	1215	no	.	.		
	782	1181	no	.	.		
	784	1273	no	.	.		
	786	1165	no	.	.		
	788	1174	no	.	.		
	793	1286	no	.	.		
	796	1299	no	.	.		
	799	1212	no	.	.		
	801	1276	no	.	.		
	802	1294	no	.	.		
	814	1309	no	.	.		

History of smallpox vaccination

Group	Subj	Screening number	Presence of smallpox vaccination: History of smallpox vaccination?	Presence of smallpox vaccination: Presence of typical vaccination scar?	Smallpox vaccination history: ID	Smallpox vaccination history: Vaccination date	Smallpox vaccination history: Vaccine
Group 4 (M-)	001	2	yes	yes	1	03JUN1960	UNKNOWN
					2	20APR1972	UNKNOWN
	002	5	yes	yes	1	25APR1972	UNKNOWN
	004	7	yes	yes	1	Unknown	UNKNOWN
	005	9	yes	yes	1	Unknown	UNKNOWN
	006	14	yes	yes	1	1971	UNKNOWN
	007	21	yes	yes	1	1986	UNKNOWN
	008	16	yes	yes	1	1968	UNKNOWN
	009	15	yes	yes	1	Unknown	UNKNOWN
	010	11	yes	yes	1	25APR1958	UNKNOWN
					2	03MAR1966	UNKNOWN
	011	23	yes	yes	1	Unknown	UNKNOWN
	012	25	yes	yes	1	11MAY1966	UNKNOWN
	014	44	yes	yes	1	26SEP1970	UNKNOWN
	015	48	yes	yes	1	23MAY1967	NOT KNOWN
	016	56	yes	yes	1	Unknown	UNKNOWN
	017	62	yes	yes	1	Unknown	UNKNOWN
	019	74	yes	yes	1	Unknown	UNKNOWN
	020	31	yes	yes	1	1972	UNKNOWN
	021	38	yes	yes	1	Unknown	UNKNOWN
	022	73	yes	yes	1	01MAR1960	UNKNOWN
					2	15FEB1971	UNKNOWN
	023	50	yes	yes	1	Unknown	UNKNOWN
	024	67	yes	yes	1	Unknown	UNKNOWN
	026	99	yes	yes	1	21MAY1965	NOT WRITTEN IN VACCINATION CARD
	028	85	yes	yes	1	25SEP1960	NOT WRITTEN IN VACCINATION CARD
	029	53	yes	yes	1	Unknown	UNKNOWN
	030	71	yes	yes	1	02MAY1968	UNKNOWN
	031	33	yes	yes	1	Unknown	UNKNOWN

History of smallpox vaccination

Group	Subj	Screening number	Presence of smallpox vaccination: History of smallpox vaccination?	Presence of smallpox vaccination: Presence of typical vaccination scar?	Smallpox vaccination history: ID	Smallpox vaccination history: Vaccination date	Smallpox vaccination history: Vaccine
Group 4 (M-)	032	40	yes	yes	1	Unknown	UNKNOWN
	033	41	yes	yes	1	Unknown	UNKNOWN
	034	34	yes	yes	1	Unknown	UNKNOWN
	035	30	yes	yes	1	Unknown	UNKNOWN
	036	58	yes	yes	1	1987	UNKNOWN
	037	49	yes	yes	1	Unknown	UNKNOWN
	038	81	yes	yes	1	Unknown	UNKNOWN
	039	84	yes	yes	1	Unknown	UNKNOWN
	041	101	yes	yes	1	Unknown	UNKNOWN
	042	26	yes	yes	1	24MAR1979	UNKNOWN
	043	75	yes	yes	1	1984	UNKNOWN
	044	52	yes	yes	1	13AUG1975	NOT WRITTEN IN VACCINATION CARD
	045	69	yes	yes	1	1960	UNKNOWN
	047	105	yes	yes	1	Unknown	UNKNOWN
					2	Unknown	NOT KNOWN
	048	110	yes	yes	1	Unknown	UNKNOWN
	049	97	yes	yes	1	Unknown	UNKNOWN
	050	98	yes	yes	1	Unknown	UNKNOWN
	051	107	yes	yes	1	Unknown	UNKNOWN
	052	108	yes	yes	1	1964	NOT KNOWN
	053	111	yes	yes	1	06APR1964	NOT KNOWN
	055	119	yes	yes	1	14SEP1965	UNKNOWN
					2	31OCT1973	UNKNOWN
	056	123	yes	yes	1	07MAY1952	UNKNOWN
					2	22MAY1963	UNKNOWN
	057	124	yes	yes	1	Unknown	UNKNOWN
	058	126	yes	yes	1	1966	UNKNOWN
	059	127	yes	yes	1	23JUL1963	UNKNOWN
					2	28FEB1974	UNKNOWN

History of smallpox vaccination

Group	Subj	Screening number	Presence of smallpox vaccination: History of smallpox vaccination?	Presence of smallpox vaccination: Presence of typical vaccination scar?	Smallpox vaccination history: ID	Smallpox vaccination history: Vaccination date	Smallpox vaccination history: Vaccine
Group 4 (M-)	060	130	yes	yes	1	28APR1961	UNKNOWN
					2	21FEB1972	UNKNOWN
	061	131	yes	yes	1	1985	UNKNOWN
	063	120	yes	yes	1	Unknown	UNKNOWN
	064	139	yes	yes	1	1969	UNKNOWN
	065	159	yes	yes	1	1961	UNKNOWN
	066	154	yes	yes	1	Unknown	UNKNOWN
	073	169	yes	yes	1	05MAR1963	UNKNOWN
					2	30APR1974	UNKNOWN
	074	164	yes	yes	1	Unknown	UNKNOWN
	075	162	yes	yes	1	Unknown	UNKNOWN
	077	138	yes	yes	1	21AUG1975	UNKNOWN
	078	133	yes	yes	1	23APR1971	UNKNOWN
	081	91	yes	yes	1	Unknown	UNKNOWN
	083	117	yes	yes	1	Unknown	NK
	086	177	yes	yes	1	Unknown	UNKNOWN
	089	195	yes	yes	1	Unknown	UNKNOWN
	091	132	yes	yes	1	Unknown	UNKNOWN
	092	186	yes	yes	1	1967	UNKNOWN
	097	183	yes	yes	1	Unknown	UNKNOWN
	100	219	yes	yes	1	1970	UNKNOWN
	106	221	yes	yes	1	Unknown	UNKNOWN
	108	217	yes	yes	1	Unknown	UNKNOWN
	111	243	yes	yes	1	15DEC1978	UNKNOWN
	113	208	yes	yes	1	18OCT1968	UNKNOWN
	115	254	yes	yes	1	Unknown	UNKNOWN
	116	257	yes	yes	1	Unknown	UNKNOWN
	117	259	yes	yes	1	Unknown	UNKNOWN
	119	256	yes	yes	1	Unknown	UNKNOWN

History of smallpox vaccination

Group	Subj	Screening number	Presence of smallpox vaccination: History of smallpox vaccination?	Presence of smallpox vaccination: Presence of typical vaccination scar?	Smallpox vaccination history: ID	Smallpox vaccination history: Vaccination date	Smallpox vaccination history: Vaccine
Group 4 (M-)	122	267	yes	yes	1	Unknown	UNKNOWN
	123	362	yes	yes	1	04OCT1973	UNKNOWN
	127	239	yes	yes	1	Unknown	UNKNOWN
	132	236	yes	yes	1	Unknown	UNKNOWN
	134	222	yes	yes	1	Unknown	N.K.
	139	286	yes	yes	1	Unknown	UNKNOWN
	145	332	yes	yes	1	14MAY1973	UNKNOWN
	146	376	yes	yes	1	1975	UNKNOWN
	150	331	yes	yes	1	Unknown	UNKNOWN
	151	287	yes	yes	1	Unknown	UNKNOWN
	153	260	yes	yes	1	Unknown	UNKNOWN
	157	328	yes	yes	1	09JUN1971	UNKNOWN
	160	339	yes	yes	1	1992	UNKNOWN, VACCINATION IN RUMAENIA
					2	1993	UNKNOWN, VACCINATION IN RUMAENIA
	162	308	yes	yes	1	Unknown	UNKNOWN
	168	294	yes	yes	1	1961	UNKNOWN
	170	246	yes	yes	1	Unknown	UNKNOWN
	173	297	yes	yes	1	Unknown	UNKNOWN
	174	232	yes	yes	1	Unknown	UNKNOWN
	178	252	yes	yes	1	1979	UNKNOWN
	181	278	yes	yes	1	Unknown	UNKNOWN
	183	370	yes	yes	1	Unknown	UNKNOWN
	185	290	yes	yes	1	Unknown	N.K. (USSR)
	193	416	yes	yes	1	Unknown	UNKNOWN
	194	284	yes	yes	1	Unknown	NK
	195	364	yes	yes	1	Unknown	NK
	206	343	yes	yes	1	Unknown	NK
	207	406	yes	yes	1	09MAY1962	UNKNOWN
	208	288	yes	yes	1	09MAY1961	UNKNOWN

History of smallpox vaccination

			Presence of smallpox vaccination		Smallpox vaccination history		
Group	Subj	Screening number	History of smallpox vaccination?	Presence of typical vaccination scar?	ID	Vaccination date	Vaccine
Group 4 (M-)	210	435	yes	yes	1	Unknown	UNKNOWN
	211	392	yes	yes	1	02NOV1967	UNKNOWN
					2	29NOV1977	UNKNOWN
	212	447	yes	yes	1	29APR1969	UNKNOWN
	214	446	yes	yes	1	Unknown	UNKNOWN
	222	442	yes	yes	1	29APR1974	N.K.
	224	289	yes	yes	1	Unknown	N.K.
	230	369	yes	yes	1	Unknown	NK
	231	475	yes	yes	1	Unknown	UNKNOWN
	234	379	yes	yes	1	10JUN1968	UNKNOWN
	235	452	yes	yes	1	07JUN1978	UNKNOWN
	238	334	yes	yes	1	Unknown	UNKNOWN
	240	317	yes	yes	1	Unknown	UNKNOWN
	242	459	yes	yes	1	Unknown	UNKNOWN
	243	322	yes	yes	1	15JUN1971	UNKNOWN
	244	462	yes	yes	1	31JUL1973	UNKNOWN
	245	385	yes	yes	1	18APR1969	UNKNOWN
	246	479	yes	yes	1	Unknown	UNKNOWN
	249	464	yes	yes	1	Unknown	UNKNOWN
	252	329	yes	yes	1	1970	UNKNOWN
	257	399	yes	yes	1	11MAY1978	NK
	259	478	yes	yes	1	Unknown	UNKNOWN
	261	451	yes	yes	1	Unknown	UNKNOWN
	262	407	yes	yes	1	02OCT1979	CHNR: 221178
	267	476	yes	yes	1	Unknown	N.K.
	269	499	yes	yes	1	15FEB1966	24063/3
					2	30APR1976	N.K.
	270	431	yes	yes	1	Unknown	N.K.
	271	474	yes	yes	1	Unknown	UNKNOWN

History of smallpox vaccination

Group	Subj	Screening number	Presence of smallpox vaccination: History of smallpox vaccination?	Presence of smallpox vaccination: Presence of typical vaccination scar?	Smallpox vaccination history: ID	Smallpox vaccination history: Vaccination date	Smallpox vaccination history: Vaccine
Group 4 (M-)	272	372	yes	yes	1	Unknown	UNKNOWN
	275	485	yes	yes	1	1976	NK
	277	448	yes	yes	1	Unknown	NK
	279	529	yes	yes	1	23AUG1978	UNKNOWN
	286	473	yes	yes	1	27JUN1973	N.K.
					2	29APR1974	UNKNOWN
	289	545	yes	yes	1	Unknown	N.K.
	291	440	yes	yes	1	Unknown	N.K.
	293	500	yes	yes	1	31JAN1967	N.K.
					2	11APR1967	N.K.
	295	498	yes	yes	1	Unknown	N.K.
	298	523	yes	yes	1	18JUL1971	UNKNOWN
	299	559	yes	yes	1	11MAR1977	UNKNOWN
					2	23APR1980	UNKNOWN (SAUDI ARABIA)
	302	455	yes	yes	1	Unknown	UNKNOWN
	311	548	yes	yes	1	Unknown	UNKNOWN
	314	576	yes	yes	1	Unknown	NK
	315	426	yes	yes	1	13FEB1963	NK
					2	21MAY1974	NK
	320	588	yes	yes	1	Unknown	UNKNOWN
	324	508	yes	yes	1	17APR1956	UNKNOWN
					2	02MAY1967	UNKNOWN
	325	564	yes	yes	1	Unknown	UNKNOWN
	326	520	yes	yes	1	Unknown	UNKNOWN
	327	580	yes	yes	1	10MAY1968	UNKNOWN
					2	04MAY1963	UNKNOWN
					3	22JUL1974	UNKNOWN
	332	483	yes	no	1	22SEP1969	UNKNOWN
	333	524	yes	yes	1	Unknown	UNKNOWN

History of smallpox vaccination

			Presence of smallpox vaccination		Smallpox vaccination history		
Group	Subj	Screening number	History of smallpox vaccination?	Presence of typical vaccination scar?	ID	Vaccination date	Vaccine
Group 4 (M-)	335	543	yes	yes	1	16MAY1966	UNKNOWN
	336	531	yes	yes	1	Unknown	UNKNOWN
	338	556	yes	yes	1	02FEB1990	MVA
					2	16MAR1990	MVA
					3	07JAN1969	UNKNOWN
	344	542	yes	yes	1	20APR1971	NK
	345	635	yes	yes	1	21MAY1968	NK
	346	614	yes	yes	1	22OCT1963	UNKNOWN
					2	28AUG1964	UNKNOWN
	347	555	yes	yes	1	Unknown	UNKNOWN
					2	14FEB1975	UNKNOWN
	349	633	yes	yes	1	02OCT1968	NK
	358	561	yes	yes	1	Unknown	UNKNOWN
	359	622	yes	yes	1	Unknown	UNKNOWN
	363	620	yes	yes	1	Unknown	UNKNOWN
	365	608	yes	yes	1	25MAR1958	N.K.
	368	649	yes	yes	1	Unknown	N.K.
	369	546	yes	no	1	01APR1968	N.K.
	370	609	yes	yes	1	1969	UNKNOWN
					2	1976	UNKNOWN
	371	549	yes	yes	1	06MAY1960	NK
					2	05MAY1969	NK
	373	677	yes	yes	1	21MAR1960	UNKNOWN
					2	13APR1970	UNKNOWN
	378	690	yes	yes	1	11SEP1964	NK
					2	24JUN1976	NK
	379	668	yes	yes	1	10JAN1975	NK
					2	17APR1975	NK
	380	652	yes	yes	1	05JAN1960	NK

History of smallpox vaccination

Group	Subj	Screening number	Presence of smallpox vaccination: History of smallpox vaccination?	Presence of smallpox vaccination: Presence of typical vaccination scar?	Smallpox vaccination history: ID	Smallpox vaccination history: Vaccination date	Smallpox vaccination history: Vaccine
Group 4 (M-)	380	652	yes	yes	2	04FEB1969	NK
					3	05OCT1978	NK
	381	651	yes	yes	1	10MAY1963	SMALLPOX
					2	02MAY1972	SMALLPOX
	382	643	yes	yes	1	07DEC1979	NK
	388	678	yes	yes	1	Unknown	UNKNOWN
	395	672	yes	yes	1	Unknown	N.K.
	396	674	yes	yes	1	24OCT1974	N.K.
	397	682	yes	yes	1	Unknown	N.K.
	401	569	yes	yes	1	23APR1969	N.K.
					2	30APR1969	N.K.
	404	686	yes	yes	1	29MAY1979	UNKNOWN
	407	702	yes	yes	1	06MAY1963	UNKNOWN
					2	27MAR1973	UNKNOWN
	408	670	yes	yes	1	Unknown	UNKNOWN
	409	658	yes	yes	1	24FEB1969	SMALLPOX
	410	711	yes	yes	1	24OCT1960	UNKNOWN
					2	24MAR1971	UNKNOWN
	411	683	yes	yes	1	Unknown	UNKNOWN
	412	692	yes	yes	1	19NOV1976	UNKNOWN
	413	619	yes	yes	1	Unknown	UNKNOWN
	414	718	yes	yes	1	Unknown	UNKNOWN
	419	732	yes	yes	1	Unknown	UNKNOWN
	420	703	yes	yes	1	Unknown	UNKNOWN
	421	734	yes	yes	1	Unknown	N.K.
	422	735	yes	yes	1	24APR1962	N.K.
					2	18AUG1970	N.K.
	430	581	yes	yes	1	1974	NK
	439	585	yes	yes	1	05MAY1965	N.K.

History of smallpox vaccination

			Presence of smallpox vaccination		Smallpox vaccination history		
Group	Subj	Screening number	History of smallpox vaccination?	Presence of typical vaccination scar?	ID	Vaccination date	Vaccine
Group 4 (M-)	451	721	yes	yes	1	Unknown	UNKNOWN
	452	731	yes	yes	1	Unknown	UNKNOWN
	524	771	yes	yes	1	Unknown	UNKNOWN

16.2.4.3. Medical history

Group	Subj	Screening number	Category	Result	Diagnosis/pathological finding	Year of first diagnosis (yyyy)	Currently active/present
Group 1 (MM)	070	142	skin	normal		.	.
			head, eyes, ears, nose and throat	normal		.	.
			respiratory	normal		.	.
			cardiovascular	normal		.	.
			gastrointestinal and hepatic	normal		.	.
			renal and genitourinary	normal		.	.
			endocrine metabolic	normal		.	.
			hemopoietic and lymphatic	normal		.	.
			musculoskeletal	normal		.	.
			surgical history	normal		.	.
			psychiatric events	normal		.	.
			drug abuse of all kinds	normal		.	.
			history of allergic reactions	normal		.	.
			other	normal		.	.
	080	152	skin	normal		.	.
			head, eyes, ears, nose and throat	abnormal	TINNITUS	2004	yes
			respiratory	normal		.	.
			cardiovascular	normal		.	.
			gastrointestinal and hepatic	abnormal	M MEULENGRACHT	-	yes
			renal and genitourinary	normal		.	.
			endocrine metabolic	normal		.	.
			hemopoietic and lymphatic	normal		.	.
			musculoskeletal	abnormal	OSTEOCHONDROSIS DISSECANS	1996	no
			surgical history	normal		.	.
			psychiatric events	normal		.	.
			drug abuse of all kinds	normal		.	.
			history of allergic reactions	normal		.	.
			other	normal		.	.
	084	149	skin	normal		.	.
			head, eyes, ears, nose and throat	normal		.	.
			respiratory	normal		.	.

Medical history

Group	Subj	Screening number	Category	Result	Diagnosis/pathological finding	Year of first diagnosis (yyyy)	Currently active/present
Group 1 (MM)	084	149	cardiovascular	normal		.	.
			gastrointestinal and hepatic	normal		.	.
			renal and genitourinary	normal		.	.
			endocrine metabolic	normal		.	.
			hemopoietic and lymphatic	normal		.	.
			musculoskeletal	normal		.	.
			surgical history	normal		.	.
			psychiatric events	normal		.	.
			drug abuse of all kinds	normal		.	.
			history of allergic reactions	normal		.	.
			other	normal		.	.
	088	174	skin	normal		.	.
			head, eyes, ears, nose and throat	normal		.	.
			respiratory	normal		.	.
			cardiovascular	normal		.	.
			gastrointestinal and hepatic	abnormal	GASTRITIS	2006	yes
			renal and genitourinary	normal		.	.
			endocrine metabolic	normal		.	.
			hemopoietic and lymphatic	normal		.	.
			musculoskeletal	normal		.	.
			surgical history	normal		.	.
			psychiatric events	normal		.	.
			drug abuse of all kinds	normal		.	.
			history of allergic reactions	normal		.	.
			other	normal		.	.
	093	201	skin	normal		.	.
			head, eyes, ears, nose and throat	normal		.	.
			respiratory	normal		.	.
			cardiovascular	normal		.	.
			gastrointestinal and hepatic	normal		.	.
			renal and genitourinary	normal		.	.

Medical history

Group	Subj	Screening number	Category	Result	Diagnosis/pathological finding	Year of first diagnosis (yyyy)	Currently active/present
Group 1 (MM)	093	201	endocrine metabolic	normal		.	.
			hemopoietic and lymphatic	normal		.	.
			musculoskeletal	normal		.	.
			surgical history	normal		.	.
			psychiatric events	normal		.	.
			drug abuse of all kinds	normal		.	.
			history of allergic reactions	normal		.	.
			other	normal		.	.
	096	203	skin	normal		.	.
			head, eyes, ears, nose and throat	normal		.	.
			respiratory	normal		.	.
			cardiovascular	normal		.	.
			gastrointestinal and hepatic	normal		.	.
			renal and genitourinary	normal		.	.
			endocrine metabolic	normal		.	.
			hemopoietic and lymphatic	normal		.	.
			musculoskeletal	normal		.	.
			surgical history	normal		.	.
			psychiatric events	normal		.	.
			drug abuse of all kinds	normal		.	.
			history of allergic reactions	abnormal	ALLERGY TO ATROPINE EYE DROPS WITH RED PUSTULES	unknown	no
				abnormal	ALLERGY TO NARCISSUS WITH RED PUSTULES ON BODY	unknown	no
			other	abnormal	HEADACHE INTERMITTENT	2004	no
	102	216	skin	normal		.	.
			head, eyes, ears, nose and throat	normal		.	.
			respiratory	normal		.	.
			cardiovascular	normal		.	.
			gastrointestinal and hepatic	normal		.	.
			renal and genitourinary	normal		.	.

Medical history

Group	Subj	Screening number	Category	Result	Diagnosis/pathological finding	Year of first diagnosis (yyyy)	Currently active/present
Group 1 (MM)	102	216	endocrine metabolic	normal		.	.
			hemopoietic and lymphatic	normal		.	.
			musculoskeletal	normal		.	.
			surgical history	abnormal	TYMPANOPLASTIC (DUE TO POLYPS)	1991	no
			psychiatric events	normal		.	.
			drug abuse of all kinds	normal		.	.
			history of allergic reactions	abnormal	ALLERGIC RHINITIS	unknown	no
			other	normal		.	.
	103	224	skin	normal		.	.
			head, eyes, ears, nose and throat	abnormal	TINNITUS	2001	yes
			respiratory	normal		.	.
			cardiovascular	normal		.	.
			gastrointestinal and hepatic	normal		.	.
			renal and genitourinary	normal		.	.
			endocrine metabolic	normal		.	.
			hemopoietic and lymphatic	normal		.	.
			musculoskeletal	normal		.	.
			surgical history	normal		.	.
			psychiatric events	normal		.	.
			drug abuse of all kinds	normal		.	.
			history of allergic reactions	normal		.	.
			other	normal		.	.
	107	227	skin	normal		.	.
			head, eyes, ears, nose and throat	normal		.	.
			respiratory	normal		.	.
			cardiovascular	normal		.	.
			gastrointestinal and hepatic	normal		.	.
			renal and genitourinary	normal		.	.
			endocrine metabolic	normal		.	.
			hemopoietic and lymphatic	normal		.	.

Medical history

Group	Subj	Screening number	Category	Result	Diagnosis/pathological finding	Year of first diagnosis (yyyy)	Currently active/present
Group 1 (MM)	107	227	musculoskeletal	normal		.	.
			surgical history	normal		.	.
			psychiatric events	normal		.	.
			drug abuse of all kinds	normal		.	.
			history of allergic reactions	abnormal	ALLERGY TO SUN	unknown	no
			other	normal		.	.
	129	272	skin	normal		.	.
			head, eyes, ears, nose and throat	abnormal	CRANIOCEREBRAL TRAUMA	1995	no
			respiratory	normal		.	.
			cardiovascular	normal		.	.
			gastrointestinal and hepatic	normal		.	.
			renal and genitourinary	normal		.	.
			endocrine metabolic	normal		.	.
			hemopoietic and lymphatic	normal		.	.
			musculoskeletal	abnormal	SMALL LUMBAL DISC PROLAPSE	2005	no
			surgical history	normal		.	.
			psychiatric events	normal		.	.
			drug abuse of all kinds	normal		.	.
			history of allergic reactions	normal		.	.
			other	normal		.	.
	137	283	skin	normal		.	.
			head, eyes, ears, nose and throat	normal		.	.
			respiratory	normal		.	.
			cardiovascular	normal		.	.
			gastrointestinal and hepatic	normal		.	.
			renal and genitourinary	normal		.	.
			endocrine metabolic	normal		.	.
			hemopoietic and lymphatic	normal		.	.
			musculoskeletal	normal		.	.
			surgical history	normal		.	.
			psychiatric events	normal		.	.

Medical history

Group	Subj	Screening number	Category	Result	Diagnosis/pathological finding	Year of first diagnosis (yyyy)	Currently active/present
Group 1 (MM)	137	283	drug abuse of all kinds	normal		.	.
			history of allergic reactions	normal		.	.
			other	normal		.	.
	140	185	skin	normal		.	.
			head, eyes, ears, nose and throat	normal		.	.
			respiratory	normal		.	.
			cardiovascular	normal		.	.
			gastrointestinal and hepatic	normal		.	.
			renal and genitourinary	normal		.	.
			endocrine metabolic	normal		.	.
			hemopoietic and lymphatic	normal		.	.
			musculoskeletal	normal		.	.
			surgical history	normal		.	.
			psychiatric events	normal		.	.
			drug abuse of all kinds	normal		.	.
			history of allergic reactions	normal		.	.
			other	normal		.	.
	143	313	skin	normal		.	.
			head, eyes, ears, nose and throat	normal		.	.
			respiratory	normal		.	.
			cardiovascular	normal		.	.
			gastrointestinal and hepatic	normal		.	.
			renal and genitourinary	normal		.	.
			endocrine metabolic	normal		.	.
			hemopoietic and lymphatic	normal		.	.
			musculoskeletal	normal		.	.
			surgical history	normal		.	.
			psychiatric events	normal		.	.
			drug abuse of all kinds	normal		.	.
			history of allergic reactions	abnormal	HAY FEVER	1994	no
			other	normal		.	.

Medical history

Group	Subj	Screening number	Category	Result	Diagnosis/pathological finding	Year of first diagnosis (yyyy)	Currently active/present
Group 1 (MM)	144	320	skin	normal		.	.
			head, eyes, ears, nose and throat	normal		.	.
			respiratory	normal		.	.
			cardiovascular	normal		.	.
			gastrointestinal and hepatic	normal		.	.
			renal and genitourinary	normal		.	.
			endocrine metabolic	normal		.	.
			hemopoietic and lymphatic	normal		.	.
			musculoskeletal	normal		.	.
			surgical history	abnormal	FRACTURE LOWER ARM	1973	no
				abnormal	FRACTURE NASAL BONE	1997	no
			psychiatric events	normal		.	.
			drug abuse of all kinds	normal		.	.
			history of allergic reactions	normal		.	.
			other	normal		.	.
	148	293	skin	normal		.	.
			head, eyes, ears, nose and throat	normal		.	.
			respiratory	normal		.	.
			cardiovascular	abnormal	HYPOTENSION, MILD	unknown	yes
			gastrointestinal and hepatic	normal		.	.
			renal and genitourinary	normal		.	.
			endocrine metabolic	normal		.	.
			hemopoietic and lymphatic	normal		.	.
			musculoskeletal	normal		.	.
			surgical history	normal		.	.
			psychiatric events	normal		.	.
			drug abuse of all kinds	normal		.	.
			history of allergic reactions	abnormal	ALLERGY TO NICKEL	unknown	no
				abnormal	ALLERGY TO COBALT	unknown	no
				abnormal	ALLERGIC RHINITIS	unknown	no
			other	normal		.	.

Medical history

Group	Subj	Screening number	Category	Result	Diagnosis/pathological finding	Year of first diagnosis (yyyy)	Currently active/present
Group 1 (MM)	154	367	skin	normal		.	.
			head, eyes, ears, nose and throat	normal		.	.
			respiratory	normal		.	.
			cardiovascular	normal		.	.
			gastrointestinal and hepatic	normal		.	.
			renal and genitourinary	normal		.	.
			endocrine metabolic	normal		.	.
			hemopoietic and lymphatic	normal		.	.
			musculoskeletal	normal		.	.
			surgical history	normal		.	.
			psychiatric events	normal		.	.
			drug abuse of all kinds	normal		.	.
			history of allergic reactions	normal		.	.
			other	abnormal	HEADACHE INTERMITTENT	1997	no
	156	360	skin	normal		.	.
			head, eyes, ears, nose and throat	normal		.	.
			respiratory	normal		.	.
			cardiovascular	normal		.	.
			gastrointestinal and hepatic	normal		.	.
			renal and genitourinary	normal		.	.
			endocrine metabolic	normal		.	.
			hemopoietic and lymphatic	normal		.	.
			musculoskeletal	normal		.	.
			surgical history	normal		.	.
			psychiatric events	normal		.	.
			drug abuse of all kinds	normal		.	.
			history of allergic reactions	normal		.	.
			other	normal		.	.
	161	229	skin	normal		.	.
			head, eyes, ears, nose and throat	normal		.	.
			respiratory	normal		.	.

Medical history

Group	Subj	Screening number	Category	Result	Diagnosis/pathological finding	Year of first diagnosis (yyyy)	Currently active/present
Group 1 (MM)	161	229	cardiovascular	normal		.	.
			gastrointestinal and hepatic	normal		.	.
			renal and genitourinary	normal		.	.
			endocrine metabolic	normal		.	.
			hemopoietic and lymphatic	normal		.	.
			musculoskeletal	normal		.	.
			surgical history	abnormal	SURGERY OF SHOULDER (RIGHT)	2004	no
			psychiatric events	normal		.	.
			drug abuse of all kinds	normal		.	.
			history of allergic reactions	normal		.	.
			other	normal		.	.
	176	377	skin	normal		.	.
			head, eyes, ears, nose and throat	normal		.	.
			respiratory	normal		.	.
			cardiovascular	normal		.	.
			gastrointestinal and hepatic	normal		.	.
			renal and genitourinary	normal		.	.
			endocrine metabolic	normal		.	.
			hemopoietic and lymphatic	normal		.	.
			musculoskeletal	normal		.	.
			surgical history	normal		.	.
			psychiatric events	normal		.	.
			drug abuse of all kinds	normal		.	.
			history of allergic reactions	abnormal	CAT ALLERGY	1986	no
				abnormal	HAY FEVER, MILD	2005	no
			other	normal		.	.
	180	380	skin	normal		.	.
			head, eyes, ears, nose and throat	normal		.	.
			respiratory	normal		.	.
			cardiovascular	normal		.	.

Medical history

Group	Subj	Screening number	Category	Result	Diagnosis/pathological finding	Year of first diagnosis (yyyy)	Currently active/present
Group 1 (MM)	180	380	gastrointestinal and hepatic	normal		.	.
			renal and genitourinary	normal		.	.
			endocrine metabolic	normal		.	.
			hemopoietic and lymphatic	normal		.	.
			musculoskeletal	normal		.	.
			surgical history	normal		.	.
			psychiatric events	normal		.	.
			drug abuse of all kinds	normal		.	.
			history of allergic reactions	abnormal	ALLERGY: HONEYMELONE,PINEAPPLE,GLUTAMATE	2001	no
			other	normal		.	.
	182	359	skin	normal		.	.
			head, eyes, ears, nose and throat	abnormal	CHRON. SINUSITIS MAXILLARIS	2001	no
			respiratory	normal		.	.
			cardiovascular	normal		.	.
			gastrointestinal and hepatic	normal		.	.
			renal and genitourinary	normal		.	.
			endocrine metabolic	normal		.	.
			hemopoietic and lymphatic	normal		.	.
			musculoskeletal	normal		.	.
			surgical history	normal		.	.
			psychiatric events	normal		.	.
			drug abuse of all kinds	normal		.	.
			history of allergic reactions	normal		.	.
			other	abnormal	MIGRAINE	1995	no
	189	312	skin	normal		.	.
			head, eyes, ears, nose and throat	normal		.	.
			respiratory	normal		.	.
			cardiovascular	normal		.	.

Medical history

Group	Subj	Screening number	Category	Result	Diagnosis/pathological finding	Year of first diagnosis (yyyy)	Currently active/present
Group 1 (MM)	189	312	gastrointestinal and hepatic	normal		.	.
			renal and genitourinary	normal		.	.
			endocrine metabolic	normal		.	.
			hemopoietic and lymphatic	normal		.	.
			musculoskeletal	normal		.	.
			surgical history	normal		.	.
			psychiatric events	normal		.	.
			drug abuse of all kinds	normal		.	.
			history of allergic reactions	normal		.	.
			other	normal		.	.
	196	384	skin	normal		.	.
			head, eyes, ears, nose and throat	normal		.	.
			respiratory	normal		.	.
			cardiovascular	normal		.	.
			gastrointestinal and hepatic	normal		.	.
			renal and genitourinary	abnormal	MULTIPLE INFECTIONS OF RENAL PELVIS	1986	no
			endocrine metabolic	normal		.	.
			hemopoietic and lymphatic	normal		.	.
			musculoskeletal	normal		.	.
			surgical history	abnormal	SURGERY ON URETRAL VALVES OF BOTH SIDES	1986	no
			psychiatric events	normal		.	.
			drug abuse of all kinds	normal		.	.
			history of allergic reactions	normal		.	.
			other	abnormal	HEADACHE INTERMITTENT	1994	no
	199	366	skin	normal		.	.
			head, eyes, ears, nose and throat	normal		.	.
			respiratory	normal		.	.
			cardiovascular	normal		.	.
			gastrointestinal and hepatic	normal		.	.

Medical history

Group	Subj	Screening number	Category	Result	Diagnosis/pathological finding	Year of first diagnosis (yyyy)	Currently active/present
Group 1 (MM)	199	366	renal and genitourinary	normal		.	.
			endocrine metabolic	normal		.	.
			hemopoietic and lymphatic	normal		.	.
			musculoskeletal	abnormal	MENISCAL DAMAGE BOTH KNEES	2003	yes
				abnormal	LOWER BACK PAIN	2004	no
			surgical history	normal		.	.
			psychiatric events	normal		.	.
			drug abuse of all kinds	normal		.	.
			history of allergic reactions	abnormal	ALLERGIC RHINITIS	2006	no
			other	abnormal	HEADACHE INTERMITTENT	2006	yes
	204	398	skin	normal		.	.
			head, eyes, ears, nose and throat	normal		.	.
			respiratory	normal		.	.
			cardiovascular	normal		.	.
			gastrointestinal and hepatic	normal		.	.
			renal and genitourinary	normal		.	.
			endocrine metabolic	abnormal	HYPOTHYREOSIS	1999	no
			hemopoietic and lymphatic	normal		.	.
			musculoskeletal	normal		.	.
			surgical history	abnormal	HALLUX VALGUS SURGERY	2005	no
			psychiatric events	normal		.	.
			drug abuse of all kinds	normal		.	.
			history of allergic reactions	normal		.	.
			other	normal		.	.
	205	394	skin	normal		.	.
			head, eyes, ears, nose and throat	normal		.	.
			respiratory	normal		.	.
			cardiovascular	normal		.	.
			gastrointestinal and hepatic	normal		.	.
			renal and genitourinary	normal		.	.
			endocrine metabolic	normal		.	.

Medical history

Group	Subj	Screening number	Category	Result	Diagnosis/pathological finding	Year of first diagnosis (yyyy)	Currently active/present
Group 1 (MM)	205	394	hemopoietic and lymphatic	normal		.	.
			musculoskeletal	abnormal	ARTHIROSCOPY AFTER LUXATION OF PATELLA	1990	no
			surgical history	normal		.	.
			psychiatric events	normal		.	.
			drug abuse of all kinds	normal		.	.
			history of allergic reactions	normal		.	.
			other	normal		.	.
	215	411	skin	normal		.	.
			head, eyes, ears, nose and throat	normal		.	.
			respiratory	normal		.	.
			cardiovascular	normal		.	.
			gastrointestinal and hepatic	normal		.	.
			renal and genitourinary	normal		.	.
			endocrine metabolic	normal		.	.
			hemopoietic and lymphatic	normal		.	.
			musculoskeletal	normal		.	.
			surgical history	normal		.	.
			psychiatric events	normal		.	.
			drug abuse of all kinds	normal		.	.
			history of allergic reactions	normal		.	.
			other	abnormal	INTERMITTENT HEADACHE	2004	no
	216	417	skin	normal		.	.
			head, eyes, ears, nose and throat	normal		.	.
			respiratory	normal		.	.
			cardiovascular	normal		.	.
			gastrointestinal and hepatic	normal		.	.
			renal and genitourinary	normal		.	.
			endocrine metabolic	normal		.	.
			hemopoietic and lymphatic	normal		.	.
			musculoskeletal	normal		.	.

Medical history

Group	Subj	Screening number	Category	Result	Diagnosis/pathological finding	Year of first diagnosis (yyyy)	Currently active/present
Group 1 (MM)	216	417	surgical history	normal		.	.
			psychiatric events	normal		.	.
			drug abuse of all kinds	normal		.	.
			history of allergic reactions	normal		.	.
			other	normal		.	.
	218	403	skin	normal		.	.
			head, eyes, ears, nose and throat	normal		.	.
			respiratory	normal		.	.
			cardiovascular	normal		.	.
			gastrointestinal and hepatic	normal		.	.
			renal and genitourinary	abnormal	INTERMITTENT PAIN DURING MENSTRUATION	2000	no
			endocrine metabolic	normal		.	.
			hemopoietic and lymphatic	normal		.	.
			musculoskeletal	normal		.	.
			surgical history	normal		.	.
			psychiatric events	normal		.	.
			drug abuse of all kinds	normal		.	.
			history of allergic reactions	normal		.	.
			other	normal		.	.
	225	414	skin	normal		.	.
			head, eyes, ears, nose and throat	abnormal	TYMPANIC INFECTION LEFTSIDE	2005	no
				abnormal	MILD DECREASE OF HEARING LEFTSIDE	2005	yes
				abnormal	MILD FEELING OF PRESSURE IN LEFT EAR	2005	yes
			respiratory	normal		.	.
			cardiovascular	normal		.	.
			gastrointestinal and hepatic	normal		.	.
			renal and genitourinary	normal		.	.

Medical history

Group	Subj	Screening number	Category	Result	Diagnosis/pathological finding	Year of first diagnosis (yyyy)	Currently active/present
Group 1 (MM)	225	414	endocrine metabolic	normal		.	.
			hemopoietic and lymphatic	normal		.	.
			musculoskeletal	normal		.	.
			surgical history	normal		.	.
			psychiatric events	normal		.	.
			drug abuse of all kinds	normal		.	.
			history of allergic reactions	normal		.	.
			other	normal		.	.
	226	341	skin	normal		.	.
			head, eyes, ears, nose and throat	normal		.	.
			respiratory	normal		.	.
			cardiovascular	normal		.	.
			gastrointestinal and hepatic	normal		.	.
			renal and genitourinary	normal		.	.
			endocrine metabolic	normal		.	.
			hemopoietic and lymphatic	normal		.	.
			musculoskeletal	normal		.	.
			surgical history	abnormal	NOSE SURGERY AFTER FRACTURE	1996	no
				abnormal	4X TOOTH EXTRACTION	1994	no
			psychiatric events	normal		.	.
			drug abuse of all kinds	normal		.	.
			history of allergic reactions	normal		.	.
			other	normal		.	.
	227	381	skin	normal		.	.
			head, eyes, ears, nose and throat	normal		.	.
			respiratory	normal		.	.
			cardiovascular	normal		.	.
			gastrointestinal and hepatic	normal		.	.
			renal and genitourinary	normal		.	.
			endocrine metabolic	normal		.	.

Medical history

Group	Subj	Screening number	Category	Result	Diagnosis/pathological finding	Year of first diagnosis (yyyy)	Currently active/present
Group 1 (MM)	227	381	hemopoietic and lymphatic	normal		.	.
			musculoskeletal	normal		.	.
			surgical history	normal		.	.
			psychiatric events	normal		.	.
			drug abuse of all kinds	normal		.	.
			history of allergic reactions	normal		.	.
			other	normal		.	.
	236	353	skin	normal		.	.
			head, eyes, ears, nose and throat	normal		.	.
			respiratory	normal		.	.
			cardiovascular	abnormal	HYPOTENSION	1998	no
			gastrointestinal and hepatic	normal		.	.
			renal and genitourinary	abnormal	BLADDERY INFECTION INTERMITTENT	2005	no
			endocrine metabolic	normal		.	.
			hemopoietic and lymphatic	normal		.	.
			musculoskeletal	normal		.	.
			surgical history	normal		.	.
			psychiatric events	normal		.	.
			drug abuse of all kinds	normal		.	.
			history of allergic reactions	abnormal	ALLERGIC RHINITIS	1989	no
				abnormal	ALLERGY TO DUST	1989	no
				abnormal	ALLERGY TO NUTS	1989	no
			other	normal		.	.
	237	437	skin	normal		.	.
			head, eyes, ears, nose and throat	abnormal	ABLATIO RETINAE LEFT>RIGHT	2004	no
			respiratory	normal		.	.
			cardiovascular	normal		.	.
			gastrointestinal and hepatic	normal		.	.
			renal and genitourinary	normal		.	.
			endocrine metabolic	normal		.	.

Medical history

Group	Subj	Screening number	Category	Result	Diagnosis/pathological finding	Year of first diagnosis (yyyy)	Currently active/present
Group 1 (MM)	237	437	hemopoietic and lymphatic	normal		.	.
			musculoskeletal	normal		.	.
			surgical history	normal		.	.
			psychiatric events	normal		.	.
			drug abuse of all kinds	normal		.	.
			history of allergic reactions	normal		.	.
			other	normal		.	.
	248	445	skin	normal		.	.
			head, eyes, ears, nose and throat	normal		.	.
			respiratory	normal		.	.
			cardiovascular	normal		.	.
			gastrointestinal and hepatic	normal		.	.
			renal and genitourinary	normal		.	.
			endocrine metabolic	normal		.	.
			hemopoietic and lymphatic	normal		.	.
			musculoskeletal	normal		.	.
			surgical history	normal		.	.
			psychiatric events	normal		.	.
			drug abuse of all kinds	normal		.	.
			history of allergic reactions	abnormal	POLLINOSIS	1990	no
			other	abnormal	MOEBIUS SYNDROME, CONGENITAL	1984	yes
	254	424	skin	normal		.	.
			head, eyes, ears, nose and throat	normal		.	.
			respiratory	normal		.	.
			cardiovascular	normal		.	.
			gastrointestinal and hepatic	normal		.	.
			renal and genitourinary	normal		.	.
			endocrine metabolic	normal		.	.
			hemopoietic and lymphatic	normal		.	.
			musculoskeletal	normal		.	.

Medical history

Group	Subj	Screening number	Category	Result	Diagnosis/pathological finding	Year of first diagnosis (yyyy)	Currently active/present
Group 1 (MM)	254	424	surgical history	normal		.	.
			psychiatric events	normal		.	.
			drug abuse of all kinds	normal		.	.
			history of allergic reactions	normal		.	.
			other	normal		.	.
	255	463	skin	normal		.	.
			head, eyes, ears, nose and throat	normal		.	.
			respiratory	normal		.	.
			cardiovascular	normal		.	.
			gastrointestinal and hepatic	normal		.	.
			renal and genitourinary	normal		.	.
			endocrine metabolic	normal		.	.
			hemopoietic and lymphatic	normal		.	.
			musculoskeletal	normal		.	.
			surgical history	abnormal	CRUCIATE LIGAMENT RUPTURE LEFT	2002	no
			psychiatric events	normal		.	.
			drug abuse of all kinds	normal		.	.
			history of allergic reactions	abnormal	ANTIBIOTICS: AMOXICILLIN	2005	no
			other	normal		.	.
	256	472	skin	normal		.	.
			head, eyes, ears, nose and throat	normal		.	.
			respiratory	normal		.	.
			cardiovascular	normal		.	.
			gastrointestinal and hepatic	abnormal	LACTOSE INTOLERANCE	2006	no
			renal and genitourinary	abnormal	CYST AT RIGHT KIDNEY	2000	no
			endocrine metabolic	normal		.	.
			hemopoietic and lymphatic	normal		.	.
			musculoskeletal	abnormal	BACK PAIN, INTERMITTENT	1998	no
			surgical history	normal		.	.
			psychiatric events	normal		.	.

Medical history

Group	Subj	Screening number	Category	Result	Diagnosis/pathological finding	Year of first diagnosis (yyyy)	Currently active/present
Group 1 (MM)	256	472	drug abuse of all kinds	normal		.	.
			history of allergic reactions	normal		.	.
			other	abnormal	MIGRAINE	2001	no
				abnormal	HEADACHE	1994	no
	268	518	skin	normal		.	.
			head, eyes, ears, nose and throat	normal		.	.
			respiratory	normal		.	.
			cardiovascular	normal		.	.
			gastrointestinal and hepatic	normal		.	.
			renal and genitourinary	normal		.	.
			endocrine metabolic	normal		.	.
			hemopoietic and lymphatic	normal		.	.
			musculoskeletal	normal		.	.
			surgical history	normal		.	.
			psychiatric events	normal		.	.
			drug abuse of all kinds	normal		.	.
			history of allergic reactions	normal		.	.
			other	normal		.	.
	274	404	skin	normal		.	.
			head, eyes, ears, nose and throat	abnormal	TINNITUS	2006	no
			respiratory	normal		.	.
			cardiovascular	normal		.	.
			gastrointestinal and hepatic	normal		.	.
			renal and genitourinary	normal		.	.
			endocrine metabolic	normal		.	.
			hemopoietic and lymphatic	normal		.	.
			musculoskeletal	normal		.	.
			surgical history	normal		.	.
			psychiatric events	normal		.	.
			drug abuse of all kinds	normal		.	.
			history of allergic reactions	abnormal	ALLERGIC RHINITIS	unknown	no

Medical history

Group	Subj	Screening number	Category	Result	Diagnosis/pathological finding	Year of first diagnosis (yyyy)	Currently active/present
Group 1 (MM)	274	404	other	normal		.	.
	281	532	skin	normal		.	.
			head, eyes, ears, nose and throat	normal		.	.
			respiratory	normal		.	.
			cardiovascular	normal		.	.
			gastrointestinal and hepatic	normal		.	.
			renal and genitourinary	normal		.	.
			endocrine metabolic	normal		.	.
			hemopoietic and lymphatic	normal		.	.
			musculoskeletal	normal		.	.
			surgical history	normal		.	.
			psychiatric events	normal		.	.
			drug abuse of all kinds	normal		.	.
			history of allergic reactions	abnormal	HAY FEVER	unknown	no
			other	normal		.	.
	283	507	skin	normal		.	.
			head, eyes, ears, nose and throat	normal		.	.
			respiratory	normal		.	.
			cardiovascular	normal		.	.
			gastrointestinal and hepatic	normal		.	.
			renal and genitourinary	abnormal	CYSTITIS	1995	no
			endocrine metabolic	normal		.	.
			hemopoietic and lymphatic	normal		.	.
			musculoskeletal	normal		.	.
			surgical history	normal		.	.
			psychiatric events	normal		.	.
			drug abuse of all kinds	normal		.	.
			history of allergic reactions	abnormal	HAY FEVER	1990	no
				abnormal	ALLERGY TO SUN	1990	no
				abnormal	ALLERGY TO BAND-AID	1990	no
			other	normal		.	.

Medical history

Group	Subj	Screening number	Category	Result	Diagnosis/pathological finding	Year of first diagnosis (yyyy)	Currently active/present
Group 1 (MM)	287	519	skin	normal		.	.
			head, eyes, ears, nose and throat	normal		.	.
			respiratory	normal		.	.
			cardiovascular	normal		.	.
			gastrointestinal and hepatic	normal		.	.
			renal and genitourinary	normal		.	.
			endocrine metabolic	abnormal	HYPOTHYREOSIS	2004	no
			hemopoietic and lymphatic	normal		.	.
			musculoskeletal	normal		.	.
			surgical history	normal		.	.
			psychiatric events	normal		.	.
			drug abuse of all kinds	normal		.	.
			history of allergic reactions	abnormal	ALLERGY TO AMALGAM	2002	no
				abnormal	ALLERGY TO PALLADIUMCHLORID	2002	no
				abnormal	ALLERGY TO ETHYLENGLYCOL - DIMETHACRYLAT	2002	no
			other	abnormal	DIZINESS	2005	no
	297	551	skin	normal		.	.
			head, eyes, ears, nose and throat	normal		.	.
			respiratory	normal		.	.
			cardiovascular	normal		.	.
			gastrointestinal and hepatic	normal		.	.
			renal and genitourinary	normal		.	.
			endocrine metabolic	normal		.	.
			hemopoietic and lymphatic	normal		.	.
			musculoskeletal	normal		.	.
			surgical history	normal		.	.
			psychiatric events	abnormal	MENTAL BREAKDOWN	2002	no
			drug abuse of all kinds	normal		.	.
			history of allergic reactions	abnormal	ALLERGY TO HAZELNUT POLLEN	1989	no
				abnormal	ALLERGY TO BERBERIS POLLEN	1989	no

Medical history

Group	Subj	Screening number	Category	Result	Diagnosis/pathological finding	Year of first diagnosis (yyyy)	Currently active/present
Group 1 (MM)	297	551	history of allergic reactions	abnormal	ALLERGY TO CURRY	2001	no
			other	normal		.	.
	304	482	skin	normal		.	.
			head, eyes, ears, nose and throat	normal		.	.
			respiratory	normal		.	.
			cardiovascular	abnormal	HYPOTENSION	2005	yes
			gastrointestinal and hepatic	normal		.	.
			renal and genitourinary	normal		.	.
			endocrine metabolic	normal		.	.
			hemopoietic and lymphatic	normal		.	.
			musculoskeletal	normal		.	.
			surgical history	normal		.	.
			psychiatric events	normal		.	.
			drug abuse of all kinds	normal		.	.
			history of allergic reactions	normal		.	.
			other	normal		.	.
	306	574	skin	normal		.	.
			head, eyes, ears, nose and throat	normal		.	.
			respiratory	normal		.	.
			cardiovascular	normal		.	.
			gastrointestinal and hepatic	normal		.	.
			renal and genitourinary	normal		.	.
			endocrine metabolic	normal		.	.
			hemopoietic and lymphatic	normal		.	.
			musculoskeletal	abnormal	INTERMITTENT BACK PAIN	1991	no
			surgical history	normal		.	.
			psychiatric events	normal		.	.
			drug abuse of all kinds	normal		.	.
			history of allergic reactions	abnormal	ALLERGY/POLLENOSIS	1994	no
			other	normal		.	.

Medical history

Group	Subj	Screening number	Category	Result	Diagnosis/pathological finding	Year of first diagnosis (yyyy)	Currently active/present
Group 1 (MM)	317	488	skin	normal		.	.
			head, eyes, ears, nose and throat	normal		.	.
			respiratory	normal		.	.
			cardiovascular	normal		.	.
			gastrointestinal and hepatic	normal		.	.
			renal and genitourinary	normal		.	.
			endocrine metabolic	normal		.	.
			hemopoietic and lymphatic	normal		.	.
			musculoskeletal	normal		.	.
			surgical history	normal		.	.
			psychiatric events	normal		.	.
			drug abuse of all kinds	normal		.	.
			history of allergic reactions	normal		.	.
			other	normal		.	.
	318	538	skin	normal		.	.
			head, eyes, ears, nose and throat	normal		.	.
			respiratory	normal		.	.
			cardiovascular	normal		.	.
			gastrointestinal and hepatic	normal		.	.
			renal and genitourinary	normal		.	.
			endocrine metabolic	normal		.	.
			hemopoietic and lymphatic	normal		.	.
			musculoskeletal	normal		.	.
			surgical history	normal		.	.
			psychiatric events	normal		.	.
			drug abuse of all kinds	normal		.	.
			history of allergic reactions	abnormal	HAY FEVER TO HAZELNUT, BIRCH, ALDER	2001	no
			other	normal		.	.
	323	471	skin	normal		.	.
			head, eyes, ears, nose and throat	abnormal	RECURRENT DIZZINESS	1996	no

Medical history

Group	Subj	Screening number	Category	Result	Diagnosis/pathological finding	Year of first diagnosis (yyyy)	Currently active/present
Group 1 (MM)	323	471	respiratory	normal		.	.
			cardiovascular	normal		.	.
			gastrointestinal and hepatic	normal		.	.
			renal and genitourinary	normal		.	.
			endocrine metabolic	normal		.	.
			hemopoietic and lymphatic	normal		.	.
			musculoskeletal	normal		.	.
			surgical history	normal		.	.
			psychiatric events	normal		.	.
			drug abuse of all kinds	normal		.	.
			history of allergic reactions	normal		.	.
			other	normal		.	.
	330	611	skin	normal		.	.
			head, eyes, ears, nose and throat	normal		.	.
			respiratory	normal		.	.
			cardiovascular	normal		.	.
			gastrointestinal and hepatic	normal		.	.
			renal and genitourinary	normal		.	.
			endocrine metabolic	normal		.	.
			hemopoietic and lymphatic	normal		.	.
			musculoskeletal	abnormal	HIP DYSPLASIA WITH INTERMITTENT PAIN	2003	no
			surgical history	normal		.	.
			psychiatric events	normal		.	.
			drug abuse of all kinds	normal		.	.
			history of allergic reactions	normal		.	.
			other	normal		.	.
	334	494	skin	normal		.	.
			head, eyes, ears, nose and throat	normal		.	.
			respiratory	normal		.	.
			cardiovascular	normal		.	.

Medical history

Group	Subj	Screening number	Category	Result	Diagnosis/pathological finding	Year of first diagnosis (yyyy)	Currently active/present
Group 1 (MM)	334	494	gastrointestinal and hepatic	normal		.	.
			renal and genitourinary	normal		.	.
			endocrine metabolic	abnormal	HYPOTHYREOIDISM	2003	yes
			hemopoietic and lymphatic	normal		.	.
			musculoskeletal	normal		.	.
			surgical history	abnormal	CARPAL TUNNEL SYNDROME	2002	no
			psychiatric events	normal		.	.
			drug abuse of all kinds	normal		.	.
			history of allergic reactions	abnormal	CONTACT ALLERGY TO NICKEL, COBALT	unknown	no
			other	abnormal	HEADACHE	1998	no
	342	596	skin	normal		.	.
			head, eyes, ears, nose and throat	normal		.	.
			respiratory	normal		.	.
			cardiovascular	normal		.	.
			gastrointestinal and hepatic	normal		.	.
			renal and genitourinary	normal		.	.
			endocrine metabolic	normal		.	.
			hemopoietic and lymphatic	normal		.	.
			musculoskeletal	abnormal	SLIGHT SCOLIOSIS WITH INTERMITTENT BACKPAIN.	1986	no
				abnormal	INTERMITTENT TENSIONS ON THORAX	1986	no
			surgical history	normal		.	.
			psychiatric events	normal		.	.
			drug abuse of all kinds	normal		.	.
			history of allergic reactions	normal		.	.
			other	abnormal	HEADACHE, INTERMITTENT	2001	no
	353	575	skin	normal		.	.
			head, eyes, ears, nose and throat	abnormal	HARDNESS OF HEARING RIGHT EAR	1981	yes

Medical history

Group	Subj	Screening number	Category	Result	Diagnosis/pathological finding	Year of first diagnosis (yyyy)	Currently active/present
Group 1 (MM)	353	575	respiratory	normal		.	.
			cardiovascular	normal		.	.
			gastrointestinal and hepatic	normal		.	.
			renal and genitourinary	normal		.	.
			endocrine metabolic	normal		.	.
			hemopoietic and lymphatic	normal		.	.
			musculoskeletal	normal		.	.
			surgical history	normal		.	.
			psychiatric events	normal		.	.
			drug abuse of all kinds	normal		.	.
			history of allergic reactions	abnormal	HAY FEVER	1993	no
			other	abnormal	RECURRENT DIZZINESS	1988	no
	356	632	skin	normal		.	.
			head, eyes, ears, nose and throat	normal		.	.
			respiratory	normal		.	.
			cardiovascular	normal		.	.
			gastrointestinal and hepatic	normal		.	.
			renal and genitourinary	normal		.	.
			endocrine metabolic	normal		.	.
			hemopoietic and lymphatic	normal		.	.
			musculoskeletal	normal		.	.
			surgical history	normal		.	.
			psychiatric events	normal		.	.
			drug abuse of all kinds	normal		.	.
			history of allergic reactions	abnormal	ALLERGY TO DUST MITES	2000	no
			other	normal		.	.
	386	648	skin	normal		.	.
			head, eyes, ears, nose and throat	normal		.	.
			respiratory	normal		.	.
			cardiovascular	normal		.	.
			gastrointestinal and hepatic	normal		.	.

Medical history

Group	Subj	Screening number	Category	Result	Diagnosis/pathological finding	Year of first diagnosis (yyyy)	Currently active/present
Group 1 (MM)	386	648	renal and genitourinary	normal		.	.
			endocrine metabolic	normal		.	.
			hemopoietic and lymphatic	normal		.	.
			musculoskeletal	normal		.	.
			surgical history	normal		.	.
			psychiatric events	normal		.	.
			drug abuse of all kinds	normal		.	.
			history of allergic reactions	normal		.	.
			other	normal		.	.
	398	698	skin	normal		.	.
			head, eyes, ears, nose and throat	normal		.	.
			respiratory	normal		.	.
			cardiovascular	normal		.	.
			gastrointestinal and hepatic	normal		.	.
			renal and genitourinary	normal		.	.
			endocrine metabolic	normal		.	.
			hemopoietic and lymphatic	normal		.	.
			musculoskeletal	normal		.	.
			surgical history	normal		.	.
			psychiatric events	normal		.	.
			drug abuse of all kinds	normal		.	.
			history of allergic reactions	abnormal	NEURODERMITIS	1982	yes
			other	normal		.	.
	400	587	skin	normal		.	.
			head, eyes, ears, nose and throat	normal		.	.
			respiratory	normal		.	.
			cardiovascular	normal		.	.
			gastrointestinal and hepatic	normal		.	.
			renal and genitourinary	normal		.	.
			endocrine metabolic	normal		.	.
			hemopoietic and lymphatic	normal		.	.

Medical history

Group	Subj	Screening number	Category	Result	Diagnosis/pathological finding	Year of first diagnosis (yyyy)	Currently active/present
Group 1 (MM)	400	587	musculoskeletal	normal		.	.
			surgical history	normal		.	.
			psychiatric events	normal		.	.
			drug abuse of all kinds	normal		.	.
			history of allergic reactions	abnormal	HAYFEVER	1984	no
			other	normal		.	.
	415	717	skin	normal		.	.
			head, eyes, ears, nose and throat	normal		.	.
			respiratory	normal		.	.
			cardiovascular	normal		.	.
			gastrointestinal and hepatic	normal		.	.
			renal and genitourinary	normal		.	.
			endocrine metabolic	normal		.	.
			hemopoietic and lymphatic	normal		.	.
			musculoskeletal	normal		.	.
			surgical history	normal		.	.
			psychiatric events	normal		.	.
			drug abuse of all kinds	normal		.	.
			history of allergic reactions	normal		.	.
			other	normal		.	.
	416	593	skin	normal		.	.
			head, eyes, ears, nose and throat	abnormal	CONCUSSION	2001	no
			respiratory	normal		.	.
			cardiovascular	normal		.	.
			gastrointestinal and hepatic	normal		.	.
			renal and genitourinary	normal		.	.
			endocrine metabolic	normal		.	.
			hemopoietic and lymphatic	normal		.	.
			musculoskeletal	normal		.	.
			surgical history	normal		.	.
			psychiatric events	abnormal	DEPRESSION	2003	no

Medical history

Group	Subj	Screening number	Category	Result	Diagnosis/pathological finding	Year of first diagnosis (yyyy)	Currently active/present
Group 1 (MM)	416	593	drug abuse of all kinds	normal		.	.
			history of allergic reactions	normal		.	.
			other	abnormal	INTERMITTENT HEADACHE	1993	no
	417	671	skin	normal		.	.
			head, eyes, ears, nose and throat	normal		.	.
			respiratory	normal		.	.
			cardiovascular	normal		.	.
			gastrointestinal and hepatic	normal		.	.
			renal and genitourinary	normal		.	.
			endocrine metabolic	normal		.	.
			hemopoietic and lymphatic	normal		.	.
			musculoskeletal	normal		.	.
			surgical history	normal		.	.
			psychiatric events	normal		.	.
			drug abuse of all kinds	normal		.	.
			history of allergic reactions	abnormal	ALLERGY AGAINST NICKEL	1996	no
				abnormal	ALLERGY AGAINST PENICILLIN	1996	no
			other	abnormal	INTERMITTENT HEADACHE	2002	no
	427	723	skin	normal		.	.
			head, eyes, ears, nose and throat	normal		.	.
			respiratory	normal		.	.
			cardiovascular	normal		.	.
			gastrointestinal and hepatic	normal		.	.
			renal and genitourinary	normal		.	.
			endocrine metabolic	normal		.	.
			hemopoietic and lymphatic	normal		.	.
			musculoskeletal	normal		.	.
			surgical history	normal		.	.
			psychiatric events	normal		.	.
			drug abuse of all kinds	normal		.	.
			history of allergic reactions	normal		.	.

Medical history

Group	Subj	Screening number	Category	Result	Diagnosis/pathological finding	Year of first diagnosis (yyyy)	Currently active/present
Group 1 (MM)	427	723	other	normal		.	.
	428	570	skin	normal		.	.
			head, eyes, ears, nose and throat	normal		.	.
			respiratory	normal		.	.
			cardiovascular	normal		.	.
			gastrointestinal and hepatic	normal		.	.
			renal and genitourinary	normal		.	.
			endocrine metabolic	normal		.	.
			hemopoietic and lymphatic	normal		.	.
			musculoskeletal	abnormal	INTERMITTENT BACK PAIN	2004	no
			surgical history	normal		.	.
			psychiatric events	normal		.	.
			drug abuse of all kinds	normal		.	.
			history of allergic reactions	normal		.	.
			other	normal		.	.
	429	762	skin	normal		.	.
			head, eyes, ears, nose and throat	normal		.	.
			respiratory	normal		.	.
			cardiovascular	normal		.	.
			gastrointestinal and hepatic	normal		.	.
			renal and genitourinary	abnormal	THIN BASEMENT MEMBRANE NEPHROPATHY	1979	no
			endocrine metabolic	normal		.	.
			hemopoietic and lymphatic	normal		.	.
			musculoskeletal	abnormal	LUMBAL DISCUS PROLAPSE	2005	no
			surgical history	normal		.	.
			psychiatric events	normal		.	.
			drug abuse of all kinds	normal		.	.
			history of allergic reactions	abnormal	PENICILLIN ALLERGY	2001	no
			other	abnormal	MIGRAINE	2003	no
				abnormal	MONONUCLEOSIS	1999	no

Medical history

Group	Subj	Screening number	Category	Result	Diagnosis/pathological finding	Year of first diagnosis (yyyy)	Currently active/present
Group 1 (MM)	429	762	other	abnormal	INTERMITTENT "PALPITATIONS"	2004	no
	432	705	skin	normal		.	.
			head, eyes, ears, nose and throat	normal		.	.
			respiratory	normal		.	.
			cardiovascular	normal		.	.
			gastrointestinal and hepatic	normal		.	.
			renal and genitourinary	normal		.	.
			endocrine metabolic	normal		.	.
			hemopoietic and lymphatic	normal		.	.
			musculoskeletal	normal		.	.
			surgical history	normal		.	.
			psychiatric events	abnormal	DEPRESSION	2005	no
			drug abuse of all kinds	normal		.	.
			history of allergic reactions	abnormal	NICKEL ALLERGY	2006	no
				abnormal	ALLERGY TO EPOXIDHARZ	2006	no
			other	normal		.	.
	433	766	skin	normal		.	.
			head, eyes, ears, nose and throat	normal		.	.
			respiratory	normal		.	.
			cardiovascular	normal		.	.
			gastrointestinal and hepatic	normal		.	.
			renal and genitourinary	normal		.	.
			endocrine metabolic	normal		.	.
			hemopoietic and lymphatic	normal		.	.
			musculoskeletal	normal		.	.
			surgical history	normal		.	.
			psychiatric events	normal		.	.
			drug abuse of all kinds	normal		.	.
			history of allergic reactions	abnormal	HAYFEVER	1993	yes
			other	normal		.	.

Medical history

Group	Subj	Screening number	Category	Result	Diagnosis/pathological finding	Year of first diagnosis (yyyy)	Currently active/present
Group 1 (MM)	438	638	skin	normal		.	.
			head, eyes, ears, nose and throat	normal		.	.
			respiratory	normal		.	.
			cardiovascular	normal		.	.
			gastrointestinal and hepatic	normal		.	.
			renal and genitourinary	abnormal	MENSTRUAL PAIN	1996	no
			endocrine metabolic	normal		.	.
			hemopoietic and lymphatic	normal		.	.
			musculoskeletal	normal		.	.
			surgical history	normal		.	.
			psychiatric events	normal		.	.
			drug abuse of all kinds	normal		.	.
			history of allergic reactions	normal		.	.
			other	normal		.	.
	441	773	skin	normal		.	.
			head, eyes, ears, nose and throat	normal		.	.
			respiratory	normal		.	.
			cardiovascular	normal		.	.
			gastrointestinal and hepatic	normal		.	.
			renal and genitourinary	normal		.	.
			endocrine metabolic	normal		.	.
			hemopoietic and lymphatic	normal		.	.
			musculoskeletal	normal		.	.
			surgical history	normal		.	.
			psychiatric events	abnormal	DEPRESSION	2005	no
			drug abuse of all kinds	normal		.	.
			history of allergic reactions	normal		.	.
			other	normal		.	.
	442	688	skin	normal		.	.
			head, eyes, ears, nose and throat	normal		.	.
			respiratory	normal		.	.

Medical history

Group	Subj	Screening number	Category	Result	Diagnosis/pathological finding	Year of first diagnosis (yyyy)	Currently active/present
Group 1 (MM)	442	688	cardiovascular	normal		.	.
			gastrointestinal and hepatic	normal		.	.
			renal and genitourinary	normal		.	.
			endocrine metabolic	abnormal	HYPOTHYROIDISM	2004	yes
			hemopoietic and lymphatic	normal		.	.
			musculoskeletal	normal		.	.
			surgical history	normal		.	.
			psychiatric events	abnormal	ANXIETY DISORDER	2005	yes
			drug abuse of all kinds	normal		.	.
			history of allergic reactions	abnormal	HAY FEVER	unknown	yes
				abnormal	ALLERGIC ASTHMA	2006	yes
			other	normal		.	.
	447	806	skin	normal		.	.
			head, eyes, ears, nose and throat	normal		.	.
			respiratory	normal		.	.
			cardiovascular	normal		.	.
			gastrointestinal and hepatic	normal		.	.
			renal and genitourinary	normal		.	.
			endocrine metabolic	normal		.	.
			hemopoietic and lymphatic	normal		.	.
			musculoskeletal	normal		.	.
			surgical history	normal		.	.
			psychiatric events	normal		.	.
			drug abuse of all kinds	normal		.	.
			history of allergic reactions	normal		.	.
			other	normal		.	.
	448	747	skin	normal		.	.
			head, eyes, ears, nose and throat	normal		.	.
			respiratory	normal		.	.
			cardiovascular	normal		.	.
			gastrointestinal and hepatic	normal		.	.

Medical history

Group	Subj	Screening number	Category	Result	Diagnosis/pathological finding	Year of first diagnosis (yyyy)	Currently active/present
Group 1 (MM)	448	747	renal and genitourinary	normal		.	.
			endocrine metabolic	normal		.	.
			hemopoietic and lymphatic	normal		.	.
			musculoskeletal	abnormal	BACK PAIN, INTERMITTENT	2005	no
			surgical history	normal		.	.
			psychiatric events	normal		.	.
			drug abuse of all kinds	normal		.	.
			history of allergic reactions	normal		.	.
			other	normal		.	.
	449	782	skin	normal		.	.
			head, eyes, ears, nose and throat	normal		.	.
			respiratory	normal		.	.
			cardiovascular	normal		.	.
			gastrointestinal and hepatic	normal		.	.
			renal and genitourinary	normal		.	.
			endocrine metabolic	normal		.	.
			hemopoietic and lymphatic	normal		.	.
			musculoskeletal	normal		.	.
			surgical history	normal		.	.
			psychiatric events	normal		.	.
			drug abuse of all kinds	normal		.	.
			history of allergic reactions	normal		.	.
			other	normal		.	.
	455	687	skin	normal		.	.
			head, eyes, ears, nose and throat	normal		.	.
			respiratory	normal		.	.
			cardiovascular	normal		.	.
			gastrointestinal and hepatic	normal		.	.
			renal and genitourinary	normal		.	.
			endocrine metabolic	normal		.	.
			hemopoietic and lymphatic	normal		.	.

Medical history

Group	Subj	Screening number	Category	Result	Diagnosis/pathological finding	Year of first diagnosis (yyyy)	Currently active/present
Group 1 (MM)	455	687	musculoskeletal	normal		.	.
			surgical history	normal		.	.
			psychiatric events	normal		.	.
			drug abuse of all kinds	normal		.	.
			history of allergic reactions	normal		.	.
			other	normal		.	.
	457	795	skin	normal		.	.
			head, eyes, ears, nose and throat	abnormal	CONCUSSION	1993	no
			respiratory	normal		.	.
			cardiovascular	normal		.	.
			gastrointestinal and hepatic	normal		.	.
			renal and genitourinary	normal		.	.
			endocrine metabolic	normal		.	.
			hemopoietic and lymphatic	normal		.	.
			musculoskeletal	normal		.	.
			surgical history	normal		.	.
			psychiatric events	normal		.	.
			drug abuse of all kinds	normal		.	.
			history of allergic reactions	abnormal	ALLERGY TO NICKEL	1998	no
			other	normal		.	.
	459	790	skin	normal		.	.
			head, eyes, ears, nose and throat	normal		.	.
			respiratory	normal		.	.
			cardiovascular	normal		.	.
			gastrointestinal and hepatic	normal		.	.
			renal and genitourinary	normal		.	.
			endocrine metabolic	normal		.	.
			hemopoietic and lymphatic	normal		.	.
			musculoskeletal	normal		.	.
			surgical history	normal		.	.
			psychiatric events	normal		.	.

Medical history

Group	Subj	Screening number	Category	Result	Diagnosis/pathological finding	Year of first diagnosis (yyyy)	Currently active/present
Group 1 (MM)	459	790	drug abuse of all kinds	normal		.	.
			history of allergic reactions	abnormal	HAY FEVER	1988	no
			other	normal		.	.
	462	743	skin	normal		.	.
			head, eyes, ears, nose and throat	normal		.	.
			respiratory	normal		.	.
			cardiovascular	normal		.	.
			gastrointestinal and hepatic	normal		.	.
			renal and genitourinary	normal		.	.
			endocrine metabolic	normal		.	.
			hemopoietic and lymphatic	normal		.	.
			musculoskeletal	normal		.	.
			surgical history	normal		.	.
			psychiatric events	normal		.	.
			drug abuse of all kinds	normal		.	.
			history of allergic reactions	abnormal	NICKEL ALLERGY	1990	no
			other	normal		.	.
	471	831	skin	normal		.	.
			head, eyes, ears, nose and throat	normal		.	.
			respiratory	normal		.	.
			cardiovascular	normal		.	.
			gastrointestinal and hepatic	normal		.	.
			renal and genitourinary	normal		.	.
			endocrine metabolic	normal		.	.
			hemopoietic and lymphatic	normal		.	.
			musculoskeletal	normal		.	.
			surgical history	normal		.	.
			psychiatric events	abnormal	ANXIETY DISORDER	2006	no
			drug abuse of all kinds	normal		.	.
			history of allergic reactions	abnormal	PENICILLIN ALLERGY	unknown	no
			other	abnormal	MIGRAINE	1978	no

Medical history

Group	Subj	Screening number	Category	Result	Diagnosis/pathological finding	Year of first diagnosis (yyyy)	Currently active/present
Group 1 (MM)	472	822	skin	normal		.	.
			head, eyes, ears, nose and throat	normal		.	.
			respiratory	normal		.	.
			cardiovascular	normal		.	.
			gastrointestinal and hepatic	normal		.	.
			renal and genitourinary	normal		.	.
			endocrine metabolic	normal		.	.
			hemopoietic and lymphatic	normal		.	.
			musculoskeletal	normal		.	.
			surgical history	normal		.	.
			psychiatric events	normal		.	.
			drug abuse of all kinds	normal		.	.
			history of allergic reactions	normal		.	.
			other	normal		.	.
	473	833	skin	normal		.	.
			head, eyes, ears, nose and throat	normal		.	.
			respiratory	normal		.	.
			cardiovascular	normal		.	.
			gastrointestinal and hepatic	normal		.	.
			renal and genitourinary	normal		.	.
			endocrine metabolic	normal		.	.
			hemopoietic and lymphatic	normal		.	.
			musculoskeletal	normal		.	.
			surgical history	normal		.	.
			psychiatric events	normal		.	.
			drug abuse of all kinds	normal		.	.
			history of allergic reactions	normal		.	.
			other	normal		.	.
	479	819	skin	normal		.	.
			head, eyes, ears, nose and throat	normal		.	.
			respiratory	normal		.	.

Medical history

Group	Subj	Screening number	Category	Result	Diagnosis/pathological finding	Year of first diagnosis (yyyy)	Currently active/present
Group 1 (MM)	479	819	cardiovascular	normal		.	.
			gastrointestinal and hepatic	normal		.	.
			renal and genitourinary	normal		.	.
			endocrine metabolic	normal		.	.
			hemopoietic and lymphatic	normal		.	.
			musculoskeletal	normal		.	.
			surgical history	normal		.	.
			psychiatric events	normal		.	.
			drug abuse of all kinds	normal		.	.
			history of allergic reactions	abnormal	PENICILLIN ALLERGY	2001	no
			other	normal		.	.
	482	804	skin	normal		.	.
			head, eyes, ears, nose and throat	normal		.	.
			respiratory	normal		.	.
			cardiovascular	normal		.	.
			gastrointestinal and hepatic	normal		.	.
			renal and genitourinary	normal		.	.
			endocrine metabolic	normal		.	.
			hemopoietic and lymphatic	normal		.	.
			musculoskeletal	normal		.	.
			surgical history	normal		.	.
			psychiatric events	normal		.	.
			drug abuse of all kinds	normal		.	.
			history of allergic reactions	abnormal	CAT HAIR ALLERGY	1989	no
				abnormal	ALLERGY TO BUNNY HAIR	1989	no
			other	normal		.	.
	483	854	skin	normal		.	.
			head, eyes, ears, nose and throat	normal		.	.
			respiratory	normal		.	.
			cardiovascular	normal		.	.
			gastrointestinal and hepatic	normal		.	.

Medical history

Group	Subj	Screening number	Category	Result	Diagnosis/pathological finding	Year of first diagnosis (yyyy)	Currently active/present
Group 1 (MM)	483	854	renal and genitourinary	normal		.	.
			endocrine metabolic	normal		.	.
			hemopoietic and lymphatic	normal		.	.
			musculoskeletal	abnormal	BACK PAIN, LUMBAR	2001	no
			surgical history	normal		.	.
			psychiatric events	normal		.	.
			drug abuse of all kinds	normal		.	.
			history of allergic reactions	normal		.	.
			other	normal		.	.
	484	817	skin	normal		.	.
			head, eyes, ears, nose and throat	normal		.	.
			respiratory	normal		.	.
			cardiovascular	normal		.	.
			gastrointestinal and hepatic	abnormal	INTERMITTENT DIARRHOEA	2000	no
			renal and genitourinary	normal		.	.
			endocrine metabolic	normal		.	.
			hemopoietic and lymphatic	normal		.	.
			musculoskeletal	normal		.	.
			surgical history	normal		.	.
			psychiatric events	normal		.	.
			drug abuse of all kinds	normal		.	.
			history of allergic reactions	normal		.	.
			other	normal		.	.
	488	843	skin	abnormal	NEURODERMATITIS	2006	no
			head, eyes, ears, nose and throat	normal		.	.
			respiratory	normal		.	.
			cardiovascular	normal		.	.
			gastrointestinal and hepatic	abnormal	INTERMITTENT PAIN IN LOWER ABDOMEN	2006	no
			renal and genitourinary	normal		.	.
			endocrine metabolic	normal		.	.

Medical history

Group	Subj	Screening number	Category	Result	Diagnosis/pathological finding	Year of first diagnosis (yyyy)	Currently active/present
Group 1 (MM)	488	843	hemopoietic and lymphatic	normal		.	.
			musculoskeletal	normal		.	.
			surgical history	normal		.	.
			psychiatric events	normal		.	.
			drug abuse of all kinds	normal		.	.
			history of allergic reactions	normal		.	.
			other	abnormal	HEADACHE INTERMITTENT	2000	no
				abnormal	INTERMITTENT DIZZINESS	2006	no
	489	700	skin	normal		.	.
			head, eyes, ears, nose and throat	normal		.	.
			respiratory	normal		.	.
			cardiovascular	normal		.	.
			gastrointestinal and hepatic	normal		.	.
			renal and genitourinary	normal		.	.
			endocrine metabolic	normal		.	.
			hemopoietic and lymphatic	normal		.	.
			musculoskeletal	normal		.	.
			surgical history	normal		.	.
			psychiatric events	normal		.	.
			drug abuse of all kinds	normal		.	.
			history of allergic reactions	normal		.	.
			other	normal		.	.
	495	816	skin	normal		.	.
			head, eyes, ears, nose and throat	normal		.	.
			respiratory	normal		.	.
			cardiovascular	normal		.	.
			gastrointestinal and hepatic	normal		.	.
			renal and genitourinary	normal		.	.
			endocrine metabolic	normal		.	.
			hemopoietic and lymphatic	normal		.	.
			musculoskeletal	normal		.	.

Medical history

Group	Subj	Screening number	Category	Result	Diagnosis/pathological finding	Year of first diagnosis (yyyy)	Currently active/present
Group 1 (MM)	495	816	surgical history	normal		.	.
			psychiatric events	normal		.	.
			drug abuse of all kinds	normal		.	.
			history of allergic reactions	normal		.	.
			other	normal		.	.
	497	844	skin	normal		.	.
			head, eyes, ears, nose and throat	normal		.	.
			respiratory	normal		.	.
			cardiovascular	normal		.	.
			gastrointestinal and hepatic	normal		.	.
			renal and genitourinary	normal		.	.
			endocrine metabolic	normal		.	.
			hemopoietic and lymphatic	normal		.	.
			musculoskeletal	normal		.	.
			surgical history	normal		.	.
			psychiatric events	normal		.	.
			drug abuse of all kinds	normal		.	.
			history of allergic reactions	abnormal	ALLERGY TO THE SUN	1987	no
			other	normal		.	.
	502	857	skin	normal		.	.
			head, eyes, ears, nose and throat	normal		.	.
			respiratory	normal		.	.
			cardiovascular	normal		.	.
			gastrointestinal and hepatic	normal		.	.
			renal and genitourinary	normal		.	.
			endocrine metabolic	normal		.	.
			hemopoietic and lymphatic	normal		.	.
			musculoskeletal	abnormal	DECREASED MOBILITY RIGHT SHOULDER DUE TO LUXATION	2006	yes
				abnormal	RECURRENT PAIN RIGHT KNEE	1996	no
			surgical history	normal		.	.

Medical history

Group	Subj	Screening number	Category	Result	Diagnosis/pathological finding	Year of first diagnosis (yyyy)	Currently active/present
Group 1 (MM)	502	857	psychiatric events	normal		.	.
			drug abuse of all kinds	normal		.	.
			history of allergic reactions	normal		.	.
			other	abnormal	RECURRENT HEADACHE	2002	no
	505	823	skin	normal		.	.
			head, eyes, ears, nose and throat	normal		.	.
			respiratory	normal		.	.
			cardiovascular	normal		.	.
			gastrointestinal and hepatic	normal		.	.
			renal and genitourinary	normal		.	.
			endocrine metabolic	normal		.	.
			hemopoietic and lymphatic	normal		.	.
			musculoskeletal	normal		.	.
			surgical history	abnormal	HYSTERECTOMY	1997	no
			psychiatric events	normal		.	.
			drug abuse of all kinds	normal		.	.
			history of allergic reactions	abnormal	ALLERGY TO PENICILLIN (NO ANAPHYLAXIS)	1977	no
			other	normal		.	.
	510	899	skin	normal		.	.
			head, eyes, ears, nose and throat	normal		.	.
			respiratory	normal		.	.
			cardiovascular	normal		.	.
			gastrointestinal and hepatic	normal		.	.
			renal and genitourinary	normal		.	.
			endocrine metabolic	normal		.	.
			hemopoietic and lymphatic	normal		.	.
			musculoskeletal	normal		.	.
			surgical history	normal		.	.
			psychiatric events	normal		.	.
			drug abuse of all kinds	normal		.	.

Medical history

Group	Subj	Screening number	Category	Result	Diagnosis/pathological finding	Year of first diagnosis (yyyy)	Currently active/present
Group 1 (MM)	510	899	history of allergic reactions	normal		.	.
			other	normal		.	.
	512	887	skin	normal		.	.
			head, eyes, ears, nose and throat	normal		.	.
			respiratory	normal		.	.
			cardiovascular	normal		.	.
			gastrointestinal and hepatic	normal		.	.
			renal and genitourinary	normal		.	.
			endocrine metabolic	normal		.	.
			hemopoietic and lymphatic	normal		.	.
			musculoskeletal	normal		.	.
			surgical history	normal		.	.
			psychiatric events	normal		.	.
			drug abuse of all kinds	normal		.	.
			history of allergic reactions	normal		.	.
			other	normal		.	.
	518	870	skin	normal		.	.
			head, eyes, ears, nose and throat	normal		.	.
			respiratory	normal		.	.
			cardiovascular	normal		.	.
			gastrointestinal and hepatic	normal		.	.
			renal and genitourinary	normal		.	.
			endocrine metabolic	abnormal	HYPOTHYREOSIS	2001	yes
			hemopoietic and lymphatic	normal		.	.
			musculoskeletal	normal		.	.
			surgical history	normal		.	.
			psychiatric events	abnormal	BULIMIA	2001	no
			drug abuse of all kinds	normal		.	.
			history of allergic reactions	abnormal	ALLERGY TO SOY AND NUTS	2006	no
			other	normal		.	.

Medical history

Group	Subj	Screening number	Category	Result	Diagnosis/pathological finding	Year of first diagnosis (yyyy)	Currently active/present
Group 1 (MM)	519	872	skin	normal		.	.
			head, eyes, ears, nose and throat	normal		.	.
			respiratory	normal		.	.
			cardiovascular	normal		.	.
			gastrointestinal and hepatic	normal		.	.
			renal and genitourinary	normal		.	.
			endocrine metabolic	normal		.	.
			hemopoietic and lymphatic	normal		.	.
			musculoskeletal	normal		.	.
			surgical history	normal		.	.
			psychiatric events	normal		.	.
			drug abuse of all kinds	normal		.	.
			history of allergic reactions	abnormal	ALLERGIC REACTION (RHINITIS) AGAINST HOME DUST, CAT HAIR, POLLEN	1996	no
			other	normal		.	.
	521	802	skin	normal		.	.
			head, eyes, ears, nose and throat	normal		.	.
			respiratory	normal		.	.
			cardiovascular	normal		.	.
			gastrointestinal and hepatic	normal		.	.
			renal and genitourinary	normal		.	.
			endocrine metabolic	normal		.	.
			hemopoietic and lymphatic	normal		.	.
			musculoskeletal	normal		.	.
			surgical history	normal		.	.
			psychiatric events	normal		.	.
			drug abuse of all kinds	normal		.	.
			history of allergic reactions	normal		.	.
			other	normal		.	.
	525	886	skin	normal		.	.

Medical history

Group	Subj	Screening number	Category	Result	Diagnosis/pathological finding	Year of first diagnosis (yyyy)	Currently active/present
Group 1 (MM)	525	886	head, eyes, ears, nose and throat	normal		.	.
			respiratory	abnormal	PNEUMONIA	1995	no
			cardiovascular	normal		.	.
			gastrointestinal and hepatic	abnormal	HEART BURN	2005	no
			renal and genitourinary	normal		.	.
			endocrine metabolic	normal		.	.
			hemopoietic and lymphatic	normal		.	.
			musculoskeletal	normal		.	.
			surgical history	normal		.	.
			psychiatric events	normal		.	.
			drug abuse of all kinds	normal		.	.
			history of allergic reactions	abnormal	HOME DUST MITE ALLERGY	1986	no
			other	normal		.	.
	532	890	skin	normal		.	.
			head, eyes, ears, nose and throat	normal		.	.
			respiratory	abnormal	COUGHING	2006	no
			cardiovascular	normal		.	.
			gastrointestinal and hepatic	normal		.	.
			renal and genitourinary	normal		.	.
			endocrine metabolic	normal		.	.
			hemopoietic and lymphatic	normal		.	.
			musculoskeletal	abnormal	SHORTENED LEFT ARM WITH WEAKNESS	1985	yes
			surgical history	normal		.	.
			psychiatric events	normal		.	.
			drug abuse of all kinds	normal		.	.
			history of allergic reactions	normal		.	.
			other	abnormal	NEURODERMATITIS	1993	no
	534	876	skin	normal		.	.
			head, eyes, ears, nose and throat	normal		.	.
			respiratory	normal		.	.

Medical history

Group	Subj	Screening number	Category	Result	Diagnosis/pathological finding	Year of first diagnosis (yyyy)	Currently active/present
Group 1 (MM)	534	876	cardiovascular	normal		.	.
			gastrointestinal and hepatic	normal		.	.
			renal and genitourinary	normal		.	.
			endocrine metabolic	normal		.	.
			hemopoietic and lymphatic	normal		.	.
			musculoskeletal	normal		.	.
			surgical history	normal		.	.
			psychiatric events	normal		.	.
			drug abuse of all kinds	normal		.	.
			history of allergic reactions	abnormal	HAY FEVER	1983	no
			other	normal		.	.
	537	928	skin	normal		.	.
			head, eyes, ears, nose and throat	abnormal	CONCUSSION	1988	no
			respiratory	normal		.	.
			cardiovascular	normal		.	.
			gastrointestinal and hepatic	abnormal	HEARTBURN	1993	no
			renal and genitourinary	normal		.	.
			endocrine metabolic	normal		.	.
			hemopoietic and lymphatic	normal		.	.
			musculoskeletal	normal		.	.
			surgical history	normal		.	.
			psychiatric events	normal		.	.
			drug abuse of all kinds	normal		.	.
			history of allergic reactions	normal		.	.
			other	normal		.	.
	543	877	skin	normal		.	.
			head, eyes, ears, nose and throat	normal		.	.
			respiratory	normal		.	.
			cardiovascular	normal		.	.
			gastrointestinal and hepatic	abnormal	RECURRENT HEARTBURN	2000	no
				abnormal	RECURRENT ABDOMINAL PAIN	2001	no

Medical history

Group	Subj	Screening number	Category	Result	Diagnosis/pathological finding	Year of first diagnosis (yyyy)	Currently active/present
Group 1 (MM)	543	877	renal and genitourinary	normal		.	.
			endocrine metabolic	normal		.	.
			hemopoietic and lymphatic	normal		.	.
			musculoskeletal	normal		.	.
			surgical history	normal		.	.
			psychiatric events	normal		.	.
			drug abuse of all kinds	normal		.	.
			history of allergic reactions	abnormal	ALLERGY TO AMOXICILLIN	1990	no
			other	abnormal	RECURRENT DIZZINESS	2000	no
	545	918	skin	normal		.	.
			head, eyes, ears, nose and throat	normal		.	.
			respiratory	normal		.	.
			cardiovascular	normal		.	.
			gastrointestinal and hepatic	normal		.	.
			renal and genitourinary	normal		.	.
			endocrine metabolic	normal		.	.
			hemopoietic and lymphatic	normal		.	.
			musculoskeletal	normal		.	.
			surgical history	normal		.	.
			psychiatric events	normal		.	.
			drug abuse of all kinds	normal		.	.
			history of allergic reactions	abnormal	RASH AFTER PENICILLIN	1988	no
			other	normal		.	.
	555	947	skin	normal		.	.
			head, eyes, ears, nose and throat	normal		.	.
			respiratory	normal		.	.
			cardiovascular	normal		.	.
			gastrointestinal and hepatic	normal		.	.
			renal and genitourinary	normal		.	.
			endocrine metabolic	normal		.	.
			hemopoietic and lymphatic	normal		.	.

Medical history

Group	Subj	Screening number	Category	Result	Diagnosis/pathological finding	Year of first diagnosis (yyyy)	Currently active/present
Group 1 (MM)	555	947	musculoskeletal	normal		.	.
			surgical history	normal		.	.
			psychiatric events	normal		.	.
			drug abuse of all kinds	normal		.	.
			history of allergic reactions	normal		.	.
			other	normal		.	.
	556	953	skin	normal		.	.
			head, eyes, ears, nose and throat	normal		.	.
			respiratory	normal		.	.
			cardiovascular	normal		.	.
			gastrointestinal and hepatic	normal		.	.
			renal and genitourinary	normal		.	.
			endocrine metabolic	normal		.	.
			hemopoietic and lymphatic	normal		.	.
			musculoskeletal	normal		.	.
			surgical history	normal		.	.
			psychiatric events	normal		.	.
			drug abuse of all kinds	normal		.	.
			history of allergic reactions	abnormal	ALLERGY TO SOME FRUITS	unknown	no
			other	normal		.	.
	557	938	skin	normal		.	.
			head, eyes, ears, nose and throat	normal		.	.
			respiratory	normal		.	.
			cardiovascular	normal		.	.
			gastrointestinal and hepatic	normal		.	.
			renal and genitourinary	normal		.	.
			endocrine metabolic	abnormal	HYPOTHYREOSIS	1995	yes
			hemopoietic and lymphatic	normal		.	.
			musculoskeletal	normal		.	.
			surgical history	normal		.	.
			psychiatric events	normal		.	.

Medical history

Group	Subj	Screening number	Category	Result	Diagnosis/pathological finding	Year of first diagnosis (yyyy)	Currently active/present
Group 1 (MM)	557	938	drug abuse of all kinds	normal		.	.
			history of allergic reactions	abnormal	RASH AFTER APPLICATION OF (UNKNOWN) OINTMENT	1982	no
			other	abnormal	HEADACHE, INTERMITTENT	2002	no
	562	964	skin	normal		.	.
			head, eyes, ears, nose and throat	normal		.	.
			respiratory	normal		.	.
			cardiovascular	normal		.	.
			gastrointestinal and hepatic	normal		.	.
			renal and genitourinary	normal		.	.
			endocrine metabolic	normal		.	.
			hemopoietic and lymphatic	normal		.	.
			musculoskeletal	normal		.	.
			surgical history	abnormal	FRACTURE OF LOWER LEG RIGHT SIDE	2005	no
			psychiatric events	normal		.	.
			drug abuse of all kinds	normal		.	.
			history of allergic reactions	abnormal	HOUSE DUST MITE ALLERGY	2005	no
				abnormal	ALLERGY AGAINST CATS HAIR	2005	no
			other	normal		.	.
	565	965	skin	normal		.	.
			head, eyes, ears, nose and throat	normal		.	.
			respiratory	normal		.	.
			cardiovascular	normal		.	.
			gastrointestinal and hepatic	normal		.	.
			renal and genitourinary	normal		.	.
			endocrine metabolic	normal		.	.
			hemopoietic and lymphatic	normal		.	.
			musculoskeletal	normal		.	.
			surgical history	normal		.	.
			psychiatric events	abnormal	DEPRESSION	2005	no

Medical history

Group	Subj	Screening number	Category	Result	Diagnosis/pathological finding	Year of first diagnosis (yyyy)	Currently active/present
Group 1 (MM)	565	965	drug abuse of all kinds	normal		.	.
			history of allergic reactions	normal		.	.
			other	abnormal	RECURRENT HEADACHE	2006	no
	567	962	skin	normal		.	.
			head, eyes, ears, nose and throat	normal		.	.
			respiratory	normal		.	.
			cardiovascular	normal		.	.
			gastrointestinal and hepatic	normal		.	.
			renal and genitourinary	normal		.	.
			endocrine metabolic	normal		.	.
			hemopoietic and lymphatic	normal		.	.
			musculoskeletal	normal		.	.
			surgical history	normal		.	.
			psychiatric events	normal		.	.
			drug abuse of all kinds	normal		.	.
			history of allergic reactions	normal		.	.
			other	abnormal	RECURRENT HEADACHE	2003	no
	568	992	skin	normal		.	.
			head, eyes, ears, nose and throat	normal		.	.
			respiratory	normal		.	.
			cardiovascular	normal		.	.
			gastrointestinal and hepatic	normal		.	.
			renal and genitourinary	normal		.	.
			endocrine metabolic	normal		.	.
			hemopoietic and lymphatic	normal		.	.
			musculoskeletal	normal		.	.
			surgical history	normal		.	.
			psychiatric events	normal		.	.
			drug abuse of all kinds	normal		.	.
			history of allergic reactions	normal		.	.
			other	normal		.	.

Medical history

Group	Subj	Screening number	Category	Result	Diagnosis/pathological finding	Year of first diagnosis (yyyy)	Currently active/present
Group 1 (MM)	571	907	skin	normal		.	.
			head, eyes, ears, nose and throat	normal		.	.
			respiratory	normal		.	.
			cardiovascular	normal		.	.
			gastrointestinal and hepatic	abnormal	RECURRENT DIARRHEA	unknown	no
				abnormal	GASTRALGIA	unknown	no
			renal and genitourinary	normal		.	.
			endocrine metabolic	normal		.	.
			hemopoietic and lymphatic	normal		.	.
			musculoskeletal	abnormal	DISC PROLAPSE	2006	no
			surgical history	normal		.	.
			psychiatric events	normal		.	.
			drug abuse of all kinds	normal		.	.
			history of allergic reactions	abnormal	ALLERGY TO WASP VENOM	1986	no
				abnormal	URTICARIA	2001	no
			other	abnormal	MIGRAINE	1995	no
				abnormal	DIZZINESS RECURRENT	1995	no
				abnormal	INSOMNIA, INTERMITTENT	unknown	yes
				abnormal	RECURRENT HEADACHE	1995	no
	572	991	skin	normal		.	.
			head, eyes, ears, nose and throat	normal		.	.
			respiratory	normal		.	.
			cardiovascular	normal		.	.
			gastrointestinal and hepatic	normal		.	.
			renal and genitourinary	normal		.	.
			endocrine metabolic	normal		.	.
			hemopoietic and lymphatic	normal		.	.
			musculoskeletal	normal		.	.
			surgical history	normal		.	.
			psychiatric events	normal		.	.
			drug abuse of all kinds	normal		.	.
			history of allergic reactions	normal		.	.

Medical history

Group	Subj	Screening number	Category	Result	Diagnosis/pathological finding	Year of first diagnosis (yyyy)	Currently active/present
Group 1 (MM)	572	991	other	normal		.	.
	574	889	skin	normal		.	.
			head, eyes, ears, nose and throat	normal		.	.
			respiratory	abnormal	ASTHMA	1994	no
			cardiovascular	normal		.	.
			gastrointestinal and hepatic	normal		.	.
			renal and genitourinary	normal		.	.
			endocrine metabolic	normal		.	.
			hemopoietic and lymphatic	normal		.	.
			musculoskeletal	normal		.	.
			surgical history	normal		.	.
			psychiatric events	normal		.	.
			drug abuse of all kinds	normal		.	.
			history of allergic reactions	abnormal	ALLERGY TO CAT HAIR	1990	no
			other	abnormal	MONONUCLEOSIS INFECTIOSA	2004	no
	582	951	skin	normal		.	.
			head, eyes, ears, nose and throat	normal		.	.
			respiratory	normal		.	.
			cardiovascular	normal		.	.
			gastrointestinal and hepatic	normal		.	.
			renal and genitourinary	normal		.	.
			endocrine metabolic	normal		.	.
			hemopoietic and lymphatic	normal		.	.
			musculoskeletal	normal		.	.
			surgical history	normal		.	.
			psychiatric events	normal		.	.
			drug abuse of all kinds	normal		.	.
			history of allergic reactions	normal		.	.
			other	normal		.	.
	583	949	skin	normal		.	.

Medical history

Group	Subj	Screening number	Category	Result	Diagnosis/pathological finding	Year of first diagnosis (yyyy)	Currently active/present
Group 1 (MM)	583	949	head, eyes, ears, nose and throat	normal		.	.
			respiratory	normal		.	.
			cardiovascular	normal		.	.
			gastrointestinal and hepatic	normal		.	.
			renal and genitourinary	normal		.	.
			endocrine metabolic	normal		.	.
			hemopoietic and lymphatic	normal		.	.
			musculoskeletal	normal		.	.
			surgical history	normal		.	.
			psychiatric events	normal		.	.
			drug abuse of all kinds	normal		.	.
			history of allergic reactions	abnormal	HAY FEVER	2000	no
			other	normal		.	.
	586	990	skin	normal		.	.
			head, eyes, ears, nose and throat	normal		.	.
			respiratory	normal		.	.
			cardiovascular	normal		.	.
			gastrointestinal and hepatic	normal		.	.
			renal and genitourinary	normal		.	.
			endocrine metabolic	normal		.	.
			hemopoietic and lymphatic	normal		.	.
			musculoskeletal	normal		.	.
			surgical history	normal		.	.
			psychiatric events	normal		.	.
			drug abuse of all kinds	normal		.	.
			history of allergic reactions	abnormal	ALLERGY AGAINST COMBINATION OF MERCUROCHROM AND BAND-AID	1992	no
			other	normal		.	.
	587	1002	skin	normal		.	.
			head, eyes, ears, nose and throat	normal		.	.

Medical history

Group	Subj	Screening number	Category	Result	Diagnosis/pathological finding	Year of first diagnosis (yyyy)	Currently active/present
Group 1 (MM)	587	1002	respiratory	normal		.	.
			cardiovascular	normal		.	.
			gastrointestinal and hepatic	normal		.	.
			renal and genitourinary	normal		.	.
			endocrine metabolic	normal		.	.
			hemopoietic and lymphatic	normal		.	.
			musculoskeletal	normal		.	.
			surgical history	normal		.	.
			psychiatric events	normal		.	.
			drug abuse of all kinds	normal		.	.
			history of allergic reactions	abnormal	HAY FEVER	1991	no
				abnormal	ALLERGY TO ANIMAL HAIRS (CATS...)	1991	no
			other	normal		.	.
	595	1026	skin	normal		.	.
			head, eyes, ears, nose and throat	normal		.	.
			respiratory	normal		.	.
			cardiovascular	normal		.	.
			gastrointestinal and hepatic	normal		.	.
			renal and genitourinary	normal		.	.
			endocrine metabolic	normal		.	.
			hemopoietic and lymphatic	normal		.	.
			musculoskeletal	normal		.	.
			surgical history	normal		.	.
			psychiatric events	normal		.	.
			drug abuse of all kinds	normal		.	.
			history of allergic reactions	normal		.	.
			other	normal		.	.
	597	1004	skin	normal		.	.
			head, eyes, ears, nose and throat	normal		.	.
			respiratory	normal		.	.

Medical history

Group	Subj	Screening number	Category	Result	Diagnosis/pathological finding	Year of first diagnosis (yyyy)	Currently active/present
Group 1 (MM)	597	1004	cardiovascular	normal		.	.
			gastrointestinal and hepatic	normal		.	.
			renal and genitourinary	normal		.	.
			endocrine metabolic	normal		.	.
			hemopoietic and lymphatic	normal		.	.
			musculoskeletal	normal		.	.
			surgical history	normal		.	.
			psychiatric events	normal		.	.
			drug abuse of all kinds	normal		.	.
			history of allergic reactions	abnormal	ALLERGY AGAINST HOUSE DUST	1989	no
				abnormal	ALLERGY AGAINST ANIMAL HAIR (GUINEA PIG)	1989	no
			other	normal		.	.
	604	1021	skin	normal		.	.
			head, eyes, ears, nose and throat	normal		.	.
			respiratory	normal		.	.
			cardiovascular	normal		.	.
			gastrointestinal and hepatic	abnormal	HEARTBURN	2005	no
			renal and genitourinary	normal		.	.
			endocrine metabolic	normal		.	.
			hemopoietic and lymphatic	abnormal	IRON DEFICIENCY ANAEMIA	2001	no
			musculoskeletal	abnormal	INTERMITTENT PAIN LOWER SPINE	2003	no
			surgical history	normal		.	.
			psychiatric events	normal		.	.
			drug abuse of all kinds	normal		.	.
			history of allergic reactions	normal		.	.
			other	normal		.	.
	605	972	skin	normal		.	.
			head, eyes, ears, nose and throat	normal		.	.
			respiratory	normal		.	.

Medical history

Group	Subj	Screening number	Category	Result	Diagnosis/pathological finding	Year of first diagnosis (yyyy)	Currently active/present
Group 1 (MM)	605	972	cardiovascular	normal		.	.
			gastrointestinal and hepatic	abnormal	INTERMITTENT HEARTBURN	unknown	no
				abnormal	INTERMITTENT ABDOMINAL PAIN	unknown	no
			renal and genitourinary	normal		.	.
			endocrine metabolic	normal		.	.
			hemopoietic and lymphatic	normal		.	.
			musculoskeletal	normal		.	.
			surgical history	normal		.	.
			psychiatric events	normal		.	.
			drug abuse of all kinds	normal		.	.
			history of allergic reactions	abnormal	SUSPECTED ALLERGY TO PRESERVATIVES	unknown	no
			other	abnormal	SUSPECTED LACTOSE INTOLERANCE	unknown	no
	607	1049	skin	abnormal	ACNE ON BACK	1997	no
			head, eyes, ears, nose and throat	normal		.	.
			respiratory	normal		.	.
			cardiovascular	normal		.	.
			gastrointestinal and hepatic	normal		.	.
			renal and genitourinary	normal		.	.
			endocrine metabolic	normal		.	.
			hemopoietic and lymphatic	normal		.	.
			musculoskeletal	normal		.	.
			surgical history	normal		.	.
			psychiatric events	normal		.	.
			drug abuse of all kinds	normal		.	.
			history of allergic reactions	normal		.	.
			other	normal		.	.
	609	1018	skin	normal		.	.
			head, eyes, ears, nose and throat	normal		.	.

Medical history

Group	Subj	Screening number	Category	Result	Diagnosis/pathological finding	Year of first diagnosis (yyyy)	Currently active/present
Group 1 (MM)	609	1018	respiratory	normal		.	.
			cardiovascular	normal		.	.
			gastrointestinal and hepatic	abnormal	M. MEULENGRACHT	unknown	yes
			renal and genitourinary	normal		.	.
			endocrine metabolic	normal		.	.
			hemopoietic and lymphatic	normal		.	.
			musculoskeletal	normal		.	.
			surgical history	normal		.	.
			psychiatric events	normal		.	.
			drug abuse of all kinds	normal		.	.
			history of allergic reactions	normal		.	.
			other	abnormal	HEADACHE, INTERMITTENT	unknown	no
	610	959	skin	normal		.	.
			head, eyes, ears, nose and throat	normal		.	.
			respiratory	normal		.	.
			cardiovascular	normal		.	.
			gastrointestinal and hepatic	abnormal	M. MEULENGRACHT	unknown	yes
			renal and genitourinary	normal		.	.
			endocrine metabolic	normal		.	.
			hemopoietic and lymphatic	normal		.	.
			musculoskeletal	normal		.	.
			surgical history	abnormal	SURGERY DUE TO PECTUS EXCAVATUM	1997	no
			psychiatric events	normal		.	.
			drug abuse of all kinds	normal		.	.
			history of allergic reactions	abnormal	HAY FEVER	2000	no
			other	normal		.	.
	612	968	skin	normal		.	.
			head, eyes, ears, nose and throat	abnormal	SKULL BASE FRACTURE	2006	no
			respiratory	normal		.	.
			cardiovascular	normal		.	.

Medical history

Group	Subj	Screening number	Category	Result	Diagnosis/pathological finding	Year of first diagnosis (yyyy)	Currently active/present
Group 1 (MM)	612	968	gastrointestinal and hepatic	normal		.	.
			renal and genitourinary	normal		.	.
			endocrine metabolic	normal		.	.
			hemopoietic and lymphatic	normal		.	.
			musculoskeletal	normal		.	.
			surgical history	normal		.	.
			psychiatric events	normal		.	.
			drug abuse of all kinds	normal		.	.
			history of allergic reactions	normal		.	.
			other	normal		.	.
	616	915	skin	normal		.	.
			head, eyes, ears, nose and throat	normal		.	.
			respiratory	normal		.	.
			cardiovascular	normal		.	.
			gastrointestinal and hepatic	normal		.	.
			renal and genitourinary	normal		.	.
			endocrine metabolic	normal		.	.
			hemopoietic and lymphatic	normal		.	.
			musculoskeletal	normal		.	.
			surgical history	normal		.	.
			psychiatric events	normal		.	.
			drug abuse of all kinds	normal		.	.
			history of allergic reactions	abnormal	HAY FEVER	2003	no
			other	normal		.	.
	625	1007	skin	normal		.	.
			head, eyes, ears, nose and throat	normal		.	.
			respiratory	normal		.	.
			cardiovascular	normal		.	.
			gastrointestinal and hepatic	abnormal	M. MEULENGRACHT	unknown	yes
			renal and genitourinary	normal		.	.
			endocrine metabolic	normal		.	.

Medical history

Group	Subj	Screening number	Category	Result	Diagnosis/pathological finding	Year of first diagnosis (yyyy)	Currently active/present
Group 1 (MM)	625	1007	hemopoietic and lymphatic	normal		.	.
			musculoskeletal	normal		.	.
			surgical history	normal		.	.
			psychiatric events	normal		.	.
			drug abuse of all kinds	normal		.	.
			history of allergic reactions	abnormal	ALLERGY AGAINST PENICILLIN	1990	no
				abnormal	ALLERGY AGAINST STINGER'S POISON	1990	no
			other	abnormal	HEADACHE	1998	no
	627	1062	skin	normal		.	.
			head, eyes, ears, nose and throat	normal		.	.
			respiratory	normal		.	.
			cardiovascular	normal		.	.
			gastrointestinal and hepatic	normal		.	.
			renal and genitourinary	normal		.	.
			endocrine metabolic	normal		.	.
			hemopoietic and lymphatic	normal		.	.
			musculoskeletal	normal		.	.
			surgical history	normal		.	.
			psychiatric events	normal		.	.
			drug abuse of all kinds	normal		.	.
			history of allergic reactions	abnormal	HAY FEVER	1992	no
			other	normal		.	.
	629	1073	skin	normal		.	.
			head, eyes, ears, nose and throat	normal		.	.
			respiratory	normal		.	.
			cardiovascular	normal		.	.
			gastrointestinal and hepatic	normal		.	.
			renal and genitourinary	abnormal	CYSTITIS	2001	no
			endocrine metabolic	normal		.	.
			hemopoietic and lymphatic	normal		.	.

Medical history

Group	Subj	Screening number	Category	Result	Diagnosis/pathological finding	Year of first diagnosis (yyyy)	Currently active/present
Group 1 (MM)	629	1073	musculoskeletal	normal		.	.
			surgical history	normal		.	.
			psychiatric events	normal		.	.
			drug abuse of all kinds	normal		.	.
			history of allergic reactions	normal		.	.
			other	normal		.	.
	634	1072	skin	normal		.	.
			head, eyes, ears, nose and throat	normal		.	.
			respiratory	normal		.	.
			cardiovascular	normal		.	.
			gastrointestinal and hepatic	normal		.	.
			renal and genitourinary	normal		.	.
			endocrine metabolic	normal		.	.
			hemopoietic and lymphatic	normal		.	.
			musculoskeletal	normal		.	.
			surgical history	normal		.	.
			psychiatric events	normal		.	.
			drug abuse of all kinds	normal		.	.
			history of allergic reactions	normal		.	.
			other	normal		.	.
	636	1037	skin	normal		.	.
			head, eyes, ears, nose and throat	normal		.	.
			respiratory	normal		.	.
			cardiovascular	normal		.	.
			gastrointestinal and hepatic	normal		.	.
			renal and genitourinary	normal		.	.
			endocrine metabolic	normal		.	.
			hemopoietic and lymphatic	normal		.	.
			musculoskeletal	normal		.	.
			surgical history	normal		.	.
			psychiatric events	abnormal	DEPRESSION	2005	no

Medical history

Group	Subj	Screening number	Category	Result	Diagnosis/pathological finding	Year of first diagnosis (yyyy)	Currently active/present
Group 1 (MM)	636	1037	drug abuse of all kinds	normal		.	.
			history of allergic reactions	abnormal	HAY FEVER	1990	no
				abnormal	ALLERGY TO ANIMAL HAIR AND FEATHER	1990	no
			other	normal		.	.
	637	908	skin	normal		.	.
			head, eyes, ears, nose and throat	normal		.	.
			respiratory	normal		.	.
			cardiovascular	normal		.	.
			gastrointestinal and hepatic	normal		.	.
			renal and genitourinary	normal		.	.
			endocrine metabolic	normal		.	.
			hemopoietic and lymphatic	normal		.	.
			musculoskeletal	normal		.	.
			surgical history	normal		.	.
			psychiatric events	normal		.	.
			drug abuse of all kinds	normal		.	.
			history of allergic reactions	abnormal	HAY FEVER	1995	no
			other	normal		.	.
	641	933	skin	normal		.	.
			head, eyes, ears, nose and throat	normal		.	.
			respiratory	normal		.	.
			cardiovascular	normal		.	.
			gastrointestinal and hepatic	abnormal	INTERMITTENT HEARTBURN	unknown	no
			renal and genitourinary	normal		.	.
			endocrine metabolic	normal		.	.
			hemopoietic and lymphatic	normal		.	.
			musculoskeletal	normal		.	.
			surgical history	normal		.	.
			psychiatric events	normal		.	.
			drug abuse of all kinds	normal		.	.

Medical history

Group	Subj	Screening number	Category	Result	Diagnosis/pathological finding	Year of first diagnosis (yyyy)	Currently active/present
Group 1 (MM)	641	933	history of allergic reactions	abnormal	HYPERSENSITIVITYTO IBUHEXAL 400 AKUT	2002	no
				abnormal	HYPERSENSITIVITY TO BEE AND WASP VENOM	2002	no
			other	abnormal	INTERMITTENT HEADACHE	2002	no
	643	1081	skin	normal		.	.
			head, eyes, ears, nose and throat	normal		.	.
			respiratory	normal		.	.
			cardiovascular	normal		.	.
			gastrointestinal and hepatic	normal		.	.
			renal and genitourinary	normal		.	.
			endocrine metabolic	normal		.	.
			hemopoietic and lymphatic	normal		.	.
			musculoskeletal	normal		.	.
			surgical history	normal		.	.
			psychiatric events	normal		.	.
			drug abuse of all kinds	normal		.	.
			history of allergic reactions	normal		.	.
			other	normal		.	.
	646	1031	skin	normal		.	.
			head, eyes, ears, nose and throat	normal		.	.
			respiratory	normal		.	.
			cardiovascular	normal		.	.
			gastrointestinal and hepatic	normal		.	.
			renal and genitourinary	normal		.	.
			endocrine metabolic	normal		.	.
			hemopoietic and lymphatic	normal		.	.
			musculoskeletal	normal		.	.
			surgical history	normal		.	.
			psychiatric events	normal		.	.
			drug abuse of all kinds	normal		.	.
			history of allergic reactions	abnormal	HAY FEVER	2002	no

Medical history

Group	Subj	Screening number	Category	Result	Diagnosis/pathological finding	Year of first diagnosis (yyyy)	Currently active/present
Group 1 (MM)	646	1031	other	normal		.	.
	648	946	skin	normal		.	.
			head, eyes, ears, nose and throat	normal		.	.
			respiratory	normal		.	.
			cardiovascular	normal		.	.
			gastrointestinal and hepatic	normal		.	.
			renal and genitourinary	normal		.	.
			endocrine metabolic	normal		.	.
			hemopoietic and lymphatic	normal		.	.
			musculoskeletal	normal		.	.
			surgical history	normal		.	.
			psychiatric events	normal		.	.
			drug abuse of all kinds	normal		.	.
			history of allergic reactions	normal		.	.
			other	normal		.	.
	649	961	skin	normal		.	.
			head, eyes, ears, nose and throat	normal		.	.
			respiratory	normal		.	.
			cardiovascular	normal		.	.
			gastrointestinal and hepatic	abnormal	MB. MEULENGRACHT (GILBERT)	2006	no
			renal and genitourinary	normal		.	.
			endocrine metabolic	normal		.	.
			hemopoietic and lymphatic	normal		.	.
			musculoskeletal	normal		.	.
			surgical history	normal		.	.
			psychiatric events	normal		.	.
			drug abuse of all kinds	normal		.	.
			history of allergic reactions	abnormal	HAY FEVER	1995	no
			other	normal		.	.
	654	1099	skin	normal		.	.

Medical history

Group	Subj	Screening number	Category	Result	Diagnosis/pathological finding	Year of first diagnosis (yyyy)	Currently active/present
Group 1 (MM)	654	1099	head, eyes, ears, nose and throat	normal		.	.
			respiratory	normal		.	.
			cardiovascular	normal		.	.
			gastrointestinal and hepatic	normal		.	.
			renal and genitourinary	normal		.	.
			endocrine metabolic	normal		.	.
			hemopoietic and lymphatic	normal		.	.
			musculoskeletal	normal		.	.
			surgical history	normal		.	.
			psychiatric events	normal		.	.
			drug abuse of all kinds	normal		.	.
			history of allergic reactions	abnormal	ALLERGY TO NICKEL	1990	no
			other	normal		.	.
	655	1098	skin	normal		.	.
			head, eyes, ears, nose and throat	normal		.	.
			respiratory	normal		.	.
			cardiovascular	normal		.	.
			gastrointestinal and hepatic	normal		.	.
			renal and genitourinary	normal		.	.
			endocrine metabolic	normal		.	.
			hemopoietic and lymphatic	normal		.	.
			musculoskeletal	normal		.	.
			surgical history	normal		.	.
			psychiatric events	normal		.	.
			drug abuse of all kinds	normal		.	.
			history of allergic reactions	abnormal	ALLERGY TO GUINEA PIG	2003	no
			other	normal		.	.
	662	1119	skin	normal		.	.
			head, eyes, ears, nose and throat	normal		.	.
			respiratory	normal		.	.
			cardiovascular	normal		.	.

Medical history

Group	Subj	Screening number	Category	Result	Diagnosis/pathological finding	Year of first diagnosis (yyyy)	Currently active/present
Group 1 (MM)	662	1119	gastrointestinal and hepatic	normal		.	.
			renal and genitourinary	normal		.	.
			endocrine metabolic	normal		.	.
			hemopoietic and lymphatic	normal		.	.
			musculoskeletal	normal		.	.
			surgical history	normal		.	.
			psychiatric events	normal		.	.
			drug abuse of all kinds	normal		.	.
			history of allergic reactions	normal		.	.
			other	abnormal	HEADACHE INTERMITTENT	1998	no
	663	1112	skin	normal		.	.
			head, eyes, ears, nose and throat	normal		.	.
			respiratory	normal		.	.
			cardiovascular	normal		.	.
			gastrointestinal and hepatic	normal		.	.
			renal and genitourinary	normal		.	.
			endocrine metabolic	normal		.	.
			hemopoietic and lymphatic	normal		.	.
			musculoskeletal	normal		.	.
			surgical history	normal		.	.
			psychiatric events	normal		.	.
			drug abuse of all kinds	normal		.	.
			history of allergic reactions	normal		.	.
			other	normal		.	.
	666	1089	skin	normal		.	.
			head, eyes, ears, nose and throat	abnormal	TINNITUS INTERMITTENT	2004	no
			respiratory	normal		.	.
			cardiovascular	normal		.	.
			gastrointestinal and hepatic	normal		.	.
			renal and genitourinary	normal		.	.
			endocrine metabolic	normal		.	.

Medical history

Group	Subj	Screening number	Category	Result	Diagnosis/pathological finding	Year of first diagnosis (yyyy)	Currently active/present
Group 1 (MM)	666	1089	hemopoietic and lymphatic	normal		.	.
			musculoskeletal	normal		.	.
			surgical history	normal		.	.
			psychiatric events	normal		.	.
			drug abuse of all kinds	normal		.	.
			history of allergic reactions	normal		.	.
			other	abnormal	RECURRENT HEADACHE	1991	no
	669	1074	skin	normal		.	.
			head, eyes, ears, nose and throat	normal		.	.
			respiratory	normal		.	.
			cardiovascular	normal		.	.
			gastrointestinal and hepatic	abnormal	M. MEULENGRACHT	unknown	yes
			renal and genitourinary	normal		.	.
			endocrine metabolic	normal		.	.
			hemopoietic and lymphatic	normal		.	.
			musculoskeletal	normal		.	.
			surgical history	normal		.	.
			psychiatric events	normal		.	.
			drug abuse of all kinds	normal		.	.
			history of allergic reactions	abnormal	HAY FEVER	1987	no
				abnormal	ALLERGY TO PENICILLIN	1990	no
			other	normal		.	.
	670	1090	skin	normal		.	.
			head, eyes, ears, nose and throat	normal		.	.
			respiratory	normal		.	.
			cardiovascular	normal		.	.
			gastrointestinal and hepatic	normal		.	.
			renal and genitourinary	normal		.	.
			endocrine metabolic	normal		.	.
			hemopoietic and lymphatic	normal		.	.
			musculoskeletal	abnormal	RECURRENT PAIN LOWER SPINE	2002	no

Medical history

Group	Subj	Screening number	Category	Result	Diagnosis/pathological finding	Year of first diagnosis (yyyy)	Currently active/present
Group 1 (MM)	670	1090	surgical history	normal		.	.
			psychiatric events	normal		.	.
			drug abuse of all kinds	normal		.	.
			history of allergic reactions	abnormal	HAY FEVER	1989	no
			other	normal		.	.
	673	1054	skin	normal		.	.
			head, eyes, ears, nose and throat	normal		.	.
			respiratory	normal		.	.
			cardiovascular	normal		.	.
			gastrointestinal and hepatic	normal		.	.
			renal and genitourinary	normal		.	.
			endocrine metabolic	normal		.	.
			hemopoietic and lymphatic	normal		.	.
			musculoskeletal	normal		.	.
			surgical history	normal		.	.
			psychiatric events	normal		.	.
			drug abuse of all kinds	normal		.	.
			history of allergic reactions	normal		.	.
			other	normal		.	.
	676	1124	skin	normal		.	.
			head, eyes, ears, nose and throat	normal		.	.
			respiratory	normal		.	.
			cardiovascular	normal		.	.
			gastrointestinal and hepatic	normal		.	.
			renal and genitourinary	normal		.	.
			endocrine metabolic	normal		.	.
			hemopoietic and lymphatic	normal		.	.
			musculoskeletal	normal		.	.
			surgical history	normal		.	.
			psychiatric events	normal		.	.
			drug abuse of all kinds	normal		.	.

Medical history

Group	Subj	Screening number	Category	Result	Diagnosis/pathological finding	Year of first diagnosis (yyyy)	Currently active/present
[truncated: 359,700 more chars]
