# Supplementary material for: Cardiac Safety of Modified Vaccinia Ankara for Vaccination against Smallpox in a Young, Healthy Study Population
Source: PLoS One. 2015 Apr 16;10(4):e0122653. doi: 10.1371/journal.pone.0122653 (PMC4399887; doi:10.1371/journal.pone.0122653)
Supplement: S1 Protocol — (PDF) [file pone.0122653.s002.pdf]

POX-MVA-005
EudraCT No. 2005-001781-14
DMID 05-0128

A partially randomized, partially double-blind, placebo-controlled Phase II non-inferiority study to evaluate immunogenicity and safety of one and two doses of MVA-BN® (IMVAMUNE™) smallpox vaccine in 18-55 year old healthy subjects

PROPERTY OF BAVARIAN NORDIC A/S

CONFIDENTIAL

Site Signature page

Herewith I agree that I have read and fully understood this Protocol:

A partially randomized, partially double-blind, placebo-controlled Phase II non-inferiority study to evaluate immunogenicity and safety of one and two doses of MVA-BN® (IMVAMUNE™) smallpox vaccine in 18-55 year old healthy subjects Version 2.0, dated 26-Mar-2007, EudraCT No. 2005-001781-14, DMID 05-0128,

which reveals all the Information necessary to conduct the study. I agree that I will conduct the study according to the instructions given by this protocol. Furthermore I agree that I will conduct this study according to ICH GCP, the current version of the declaration of Helsinki, Directive 2001/20/EC and the German Drug Law.

I agree that all Information revealed in this protocol is handled strictly confidential.

Other than that I will permit trial related monitoring, audits, Ethics Committee review and regulatory inspections, providing direct access to source data/documents.

26/03/2007

Date

Prof. Frank von Sonnenburg, MD
Principal Investigator

Date

Stephan de la Motte, MD
Investigator

Site Signature page

Herewith I agree that I have read and fully understood this Protocol:

A partially randomized, partially double-blind, placebo-controlled Phase II non-inferiority study to evaluate immunogenicity and safety of one and two doses of MVA-BN® (IMVAMUNE™) smallpox vaccine in 18-55 year old healthy subjects Version 2.0, dated 26-Mar-2007, EudraCT No. 2005-001781-14, DMID 05-0128,

which reveals all the Information necessary to conduct the study. I agree that I will conduct the study according to the instructions given by this protocol. Furthermore I agree that I will conduct this study according to ICH GCP, the current version of the declaration of Helsinki, Directive 2001/20/EC and the German Drug Law.

I agree that all Information revealed in this protocol is handled strictly confidential.

Other than that I will permit trial related monitoring, audits, Ethics Committee review and regulatory inspections, providing direct access to source data/documents.

Date	Prof. Frank von Sonnenburg, MD<br>Principal Investigator
26 MAR 2007<br>Date	[signature]<br>Stephan de la Motte, MD<br>Investigator

I. General information

Signature page

By signing the protocol:

A partially randomized, partially double-blind, placebo-controlled Phase II non-inferiority study to evaluate immunogenicity and safety of one and two doses of MVA-BN® (IMVAMUNE™) smallpox vaccine in 18-55 year old healthy subjects, Version 2.0, dated 26-Mar-2007, EudraCT No. 2005-001781-14, DMID 05-0128,

the undersigned parties agree, that the protocol was written according to international ethical and scientific quality standards (ICH-GCP), in compliance with Declaration of Helsinki, Directive 2001/20/EC and the German Drug Law.

26-Mar-2007
Date

Nadine Uebler, MSc
Protocol Author, Bavarian Nordic

27-Mar-07
Date

Garth Virgin, MD
Medical Monitor, Bavarian Nordic

26 Mar 07
Date

Joachim Klinger
Director Data Management & Statistics,
Harrison Clinical Research

26-Mar-2007
Date

Robert Semich, PhD
GCP-Specialist, Quality Assurance Development,
Bavarian Nordic

Date

Prof. Frank von Sonnenburg, MD
Principal Investigator
Institute for Tropical Medicine
University of Munich

I. General information

Signature page

By signing the protocol:

A partially randomized, partially double-blind, placebo-controlled Phase II non-inferiority study to evaluate immunogenicity and safety of one and two doses of MVA-BN® (IMVAMUNE™) smallpox vaccine in 18-55 year old healthy subjects, Version 2.0, dated 26-Mar-2007, EudraCT No. 2005-001781-14, DMID 05-0128,

the undersigned parties agree, that the protocol was written according to international ethical and scientific quality standards (ICH-GCP), in compliance with Declaration of Helsinki, Directive 2001/20/EC and the German Drug Law.

Date	Nadine Uebler, MSc<br>Protocol Author, Bavarian Nordic
Date	Garth Virgin, MD<br>Medical Monitor, Bavarian Nordic
Date	Joachim Klinger<br>Director Data Management & Statistics,<br>Harrison Clinical Research
Date	Robert Semich, PhD<br>GCP-Specialist, Quality Assurance Development,<br>Bavarian Nordic
26/03/2007<br>Date	Prof. Frank von Sonnenburg, MD<br>Principal Investigator<br>Institute for Tropical Medicine<br>University of Munich

Responsibilities

**STUDY NUMBER**:	POX-MVA-005<br>DMID 05-0128
**STUDY TITLE**:	A partially randomized, partially double-blind, placebo-controlled Phase II non-inferiority study to evaluate immunogenicity and safety of one and two doses of MVA-BN® (IMVAMUNE™) smallpox vaccine in 18-55 year old healthy subjects.
**PRINCIPAL INVESTIGATOR**:	Prof. Frank von Sonnenburg<br>Section of International Medicine & Public Health<br>Department of Infectious Diseases and Tropical Medicine<br>Georgenstrasse 5<br>80799 Munich<br>Germany
Phone:	+49-89-2180 3830
Fax:	+49-89-33 60 38
E-mail:	sonnenburg@lrz.uni-muenchen.de
**STUDY SITE:**	Harrison Clinical Research GmbH<br>Clinical Unit<br>Stephan de la Motte, MD<br>Albrechtstr. 43<br>80636 Munich<br>Germany.
Phone:	+49-89-126680-1210
Fax:	+49-89-126680-1444
**SPONSOR and PRODUCT SUPPLY:**	Bavarian Nordic A/S<br>Bøgeskovvej 9<br>DK-3490 Kvistgård<br>Denmark
Phone:	+45-33-268383
Fax:	+45-33-268380
**MEDICAL MONITOR**:	Garth Virgin, MD<br>Bavarian Nordic GmbH<br>Fraunhoferstrasse 13<br>82152 Martinsried<br>Germany
Phone:	+49-89-8565 1319
Fax:	+49-89-8565 1333
E-mail:	garth.virgin@bavarian-nordic.com

PROJECT COORDINATOR: Nadine Uebler, M.Sc.
Bavarian Nordic GmbH
Fraunhoferstrasse 13
82152 Martinsried
Germany
Phone: +49-89-85651323
Fax: +49-89-85651333
E-mail: nadine.uebler@bavarian-nordic.com

STUDY STATISTICIAN(S): Joachim Klinger
Harrison Clinical Research GmbH
Albrechtstrasse 14
80636 Munich
Germany
Phone: +49-89-12 66 80-2190
Fax: +49-89-12 66 80-2444
E-mail: JKlinger@harrison-cro.com

Niels Holger Wulff, M.Sc.
Bavarian Nordic GmbH
Fraunhoferstrasse 13
82152 Martinsried
Germany
Phone: +49-89-85651366
Fax: +49-89-85651333
E-mail: niels.wulff@bavarian-nordic.com

CRO/PROJECT MANAGER: Marianne Sievers
Harrison Clinical Research GmbH
Albrechtstrasse 14
80636 Munich
Germany
Phone: +49-89-12 66 80-2311
Mobile: +49-172-83 65 056
Fax: +49-89-12 66 80-2444
E-mail: msievers@harrison-cro.com
or mariannesievers@gmx.net

**CENTRALIZED ECG PROVIDER:**	Martin Ptacek-Zellmeier<br>ganiMed GmbH<br>Munzinger Strasse 1<br>79111 Freiburg<br>Germany
Phone:	+49-761-120 666 3
Fax:	+49-761-120 666 4
E-mail:	m.ptacek-zellmeier@ganimed.biz

**LABORATORY**:<br>**(SAFETY)**	Dr. Holger Schöneborn<br>INTERLAB GmbH<br>Bayerstrasse 53<br>80335 Munich<br>Germany
Phone:	+49 89 741 393-37
Fax:	+49 89 741 393-40
E-mail:	Dr.Schoeneborn@interlab.de

**LABORATORY**<br>**(IMMUNOLOGY)**	Thomas Meyer, PhD<br>Bavarian Nordic GmbH Frau<br>Germany
Phone:	+49-89-8565 1405
Fax:	+49-89-8565 1333
E-mail:	thomas.meyer@bavarian-nordic.com

**CARDIOLOGIST**	Eva-Zitzmann-Roth, MD<br>Richard-Strauss-Str. 56<br>81677 Munich<br>Germany
Phone:	+49 89 919095
Fax:	+49 89 99979800
E-mail:	Dr.Zitzmann-Roth@t-online.de

Table of contents

I. GENERAL INFORMATION 3

SIGNATURE PAGE 3

II. PROTOCOL SYNOPSIS 11

III. FLOW CHART 18

IV. SCIENTIFIC SECTION 19
1. Introduction 19
1.1. Background information 19
1.2. Origin and characteristics of MVA 20
1.3. Summary of Preclinical Data 21
1.4. Summary of Clinical Data 22
1.5. Dose, route of administration, vaccination schedule 28
1.6. Benefit/risk assessment 28
2. Objectives 29
3. Study design 30
3.1. Experimental design 30
3.2. Discussion of study design and choice of control groups 31
3.3. Description of study procedures 32
3.4. Study duration 36
4. Selection of subjects 36
4.1. Recruitment procedure 36
4.2. Inclusion criteria 37
4.3. Exclusion criteria 37
5. Study halting/termination and withdrawal of subjects 39
5.1. Study halting and termination rule 39
5.2. Reporting of events fulfilling the study halting criteria 39
5.3. Data Safety Monitoring Board (DSMB) 39
5.4. Individual withdrawal criteria during the study 39
5.5. Subject withdrawal procedure 40
5.6. Contraindications and precautions for further study vaccinations 40
6. Study treatment 41
6.1. Investigational product 41
6.2. Placebo 41
6.3. Packaging and labeling 41
6.4. Vaccine storage, handling and dispensing 41
6.5. Dose, vaccination schedule and route of administration 42
6.6. Accountability and disposal 42
6.7. Concomitant medication 43
7. Clinical and laboratory assessments 43
7.1. Assessment of safety and reactogenicity 43
7.2. Assessment of immunogenicity: Antibody Response 48
8. Adverse event definitions and reporting 49
8.1. Definition of adverse event 49
8.2. Definition of solicited adverse events 50
8.3. Definition of unsolicited adverse events 50
8.4. Definition of “adverse event of special interest” 50
8.5. Definition of serious adverse events (SAEs) 50
8.6. Reporting of SAEs and adverse events of special interest 51
9. Statistical Considerations 52

9.1. Sample size calculation 52
9.2. Variables 53
9.3. Study cohorts/data sets to be evaluated 54
9.4. Statistical analysis 54
10. Ethical aspects 57
10.1. Ethical and legal regulations 57
10.2. Approval of an ethics committee 57
10.3. Confidentiality and data protection 58
11. Informed consent 58
12. Case report forms and retention of records 59
12.1. Electronic case report forms 59
12.2. Retention of records 59
13. Monitoring of the study 59
14. Responsibilities of the investigator 60
15. References 62
16. Appendices 64

List of abbreviations

AE	Adverse Event
AIDS	Acquired Immunodeficiency Syndrome
ALT	Alanine aminotransferase
AST	Aspartate aminotransferase
CD	Cluster of differentiation
CDISC	Clinical Data Interchange Standards Consortium
CEF	Chicken embryo fibroblast
CI	Confidence interval
CRF(s)	Case Report Form(s); can either be hardcopy or an electronic document
eCRF	Electronic CRF
eSource	Source data captured initially into a permanent electronic record
CRO	Contract Research Organization
CVA	Chorioallantois vaccinia virus Ankara
DMID	Division of Microbiology and Infectious Diseases, National Institute of Allergy and Infectious Diseases
DSMB	Data Safety Monitoring Board
ECG	Electrocardiogram
ELISA	Enzyme-linked Immunosorbent Assay
FU	Follow-up
GMT	Geometric mean titer
HIV	Human immunodeficiency virus
ICH	International Conference of Harmonization of Technical Requirements for Registration of Pharmaceuticals for Human Use
ICS	Intracellular Cytokine Staining
IgG	immunoglobulin G
i.d.	Intra-dermal
IFN	Interferon
i.m.	Intra-muscular
i.t.	Intra-tracheal
FA	Full Analysis
LLN	Lower Limit of Normal
LMU	Ludwig Maximilian University of Munich
MedDRA	Medical Dictionary for Regulatory Activities
MVA	Modified Vaccinia Ankara Strain

MVA-BN®	Modified Vaccinia Ankara – Bavarian Nordic
n/N	Number
NCI	National Cancer Institute
NYCBH	New York City Board of Health
ODM	Operational data model
PP	Per protocol
PRNT	Plaque reduction neutralization test
PVC	Premature ventricular contractions
SAE	Serious Adverse Event
s.c.	Subcutaneous
SCR	Screening
ST	ST segment in electrocardiogram
$TCID_{50}$	Tissue culture infectious dose 50
ULN	Upper Limit of Normal
V	Visit
VV	Vaccinia Virus
WBC	White Blood Cell Count
WHO	World Health Organization

II. Protocol Synopsis

TITLE	A partially randomized, partially double-blind, placebo-controlled Phase II non-inferiority study to evaluate immunogenicity and safety of one and two doses of MVA-BN® (IMVAMUNE™) smallpox vaccine in 18-55 year old healthy subjects.
SPONSOR	Bavarian Nordic A/S<br>Bøgeskovvej 9<br>3490 Kvistgård<br>Denmark
PRINCIPAL INVESTIGATOR	Prof. Frank von Sonnenburg<br>Section of International Medicine & Public Health<br>Department of Infectious Diseases and Tropical Medicine<br>Georgenstrasse 5<br>80799 Munich<br>Germany
Phone:	+49-89-2180 3830
Fax:	+49-89-33 60 38
E-mail:	sonnenburg@lrz.uni-muenchen.de
PROJECT PHASE	Phase II
INVESTIGATIONAL DRUG	MVA-BN® liquid-frozen, containing 1 x 10^8 $TCID_{50}$ Modified Vaccinia virus Ankara per 0.5 ml dose.
PLACEBO	0.5 ml Tris Buffer<br>(1.21 g/l Tris, 8.526 g/l sodium chloride, 19.2 g/l Dextran FP-40, 45.2 g/l Sucrose, 0.108 g/l L-Glutamic acid monopotassium salt, pH 7.5)
VACCINE DOSAGE, TIMING AND ROUTE OF ADMINISTRATION	**Group 1:**<br>180 vaccinia naive subjects receive two s.c.vaccinations with 0.5 ml MVA-BN® vaccine containing 1 x 10^8 $TCID_{50}$<br><br>**Group 2:**<br>180 vaccinia naive subjects receive first vaccination with 0.5 ml MVA-BN® vaccine containing 1 x 10^8 $TCID_{50}$ and second vaccination with placebo (0.5 ml Tris Buffer)

Group 3:
180 vaccinia naive subjects receive two s.c. vaccinations with placebo (0.5 ml Tris Buffer)

Group 4:
200 vaccinia non-naive subjects receive one s.c. vaccination with 0.5 ml MVA-BN® vaccine containing 1 x 10^8 $TCID_{50}$

PRIMARY IMMUNOGENICITY OBJECTIVE

To compare the ELISA specific humoral immune response of the group with history of smallpox vaccination (Group 4: 1 dose MVA-BN®) versus the group without history of smallpox vaccination (Group 1: 2 doses MVA-BN®), i.e. to prove that a single dose schedule would be sufficient to boost the immunity in a previously vaccinated population.

PRIMARY SAFETY OBJECTIVE

To compare the four different vaccination groups with regard to ECG changes and cardiac symptoms.

SECONDARY OBJECTIVES

IMMUNOGENICITY

To compare the ELISA specific immune response of the four different vaccination groups - comparison combinations not done for the primary objective.

To compare the four different vaccination groups with regard to immune response measured with a plaque reduction neutralization test.

To assess the kinetics of the immune responses after 1 (Group 2) and 2 (Group 1) doses of MVA-BN® in vaccinia naïve subjects.

SAFETY

To compare the four different vaccination groups with regard to safety and reactogenicity.

To compare the safety of one (Group 2) and two (Group 1) vaccinations with MVA-BN® in vaccinia-naïve subjects with placebo (Group 3) and historical / recently published data of Dryvax® immunization in vaccinia-naive subjects.

STUDY DESIGN

Partially randomized, partially double-blind, placebo-controlled, 4 study groups, non-inferiority trial:

Double-blinded:

Group 1: Subjects without history of smallpox vaccination (2 doses MVA-BN®)

Group 2: Subjects without history of smallpox vaccination (1st dose MVA-BN® / 2nd dose placebo)

Group 3: Subjects without history of smallpox vaccination (2 doses placebo)

Open-label:

Group 4: Subjects with history of smallpox vaccination (1 dose MVA-BN®)

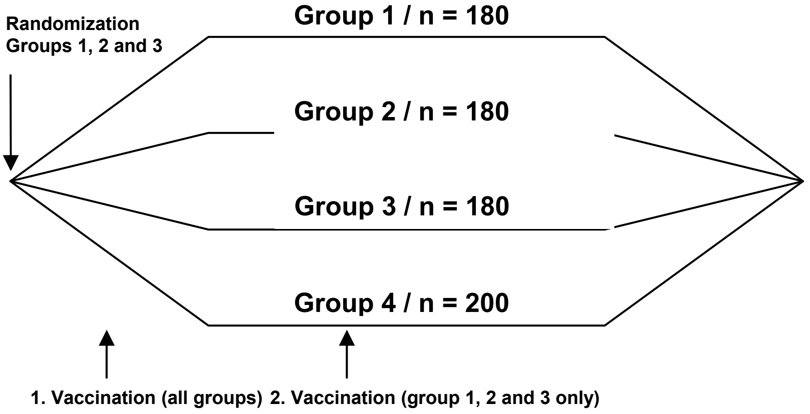

Visit Days (Groups 1, 2, 3)

-28	0	10-15	28 – 35	V3 + 10-15	V3 + 28-35	V3 + 182–210
Scr	V1	V2	V3	V4	V5	F-U

Visit Days (Group 4)

-28	0	10-15	28 - 35	V1 + 182–210
Scr	V1	V2	V3	F-U

STUDY DURATION

Groups 1/2/3: Up to 39 weeks for each subject
Groups 4: Up to 34 weeks for each subject

PLANNED TOTAL SAMPLE SIZE

The number of subjects to be enrolled is 740 (200 subjects in group 4 and 180 each in groups 1, 2 and 3). It is expected that 175 subjects per group are evaluable.

INCLUSION CRITERIA

All groups

1. Male and female subjects between 18 and 55 years of age.
2. Women must have a negative serum pregnancy test at screening and a negative urine or serum pregnancy test within 24 hours prior to vaccination.
3. Women of childbearing potential must have used an acceptable method of contraception for 30 days prior to the first vaccination, must agree to use an acceptable method of contraception during the study, and must not become pregnant for at least 28 days after the last vaccination. A woman is considered of childbearing potential unless post-menopausal or surgically sterilized. (Acceptable contraception methods are restricted to abstinence, barrier contraceptives, intrauterine contraceptive devices or licensed hormonal products.)
4. Read, signed and dated informed consent document after being advised of the risks and benefits of the study in a language understood by the subject signed, and prior to performance of any study specific procedure.
5. Troponin I within normal institutional limits.
6. White blood cells ≥ 2500/mm^3 and ≤ 11,000/ mm^3.
7. Absolute neutrophil count (ANC) ≥ 1000/mm^3.
8. Negative urine glucose by dipstick or urinalysis.
9. Hemoglobin ≥ LLN.
10. Platelets 100 – 450/nl.
11. Adequate renal function defined as:
    a. Serum creatinine without clinically significant findings.
    b. Urine protein ≤ 30 mg/dL or none or trace proteinuria (by urinalysis or dip stick).
12. Adequate hepatic function defined as:
    a. Total bilirubin ≤ 1.5 x ULN in the absence of other evidence of significant liver disease (healthy subjects without clinical disease with Morbus Meulengracht can be included).
    b. AST (SGOT), ALT (SGPT) and alkaline phosphatase without clinically significant findings.
13. Electrocardiogram (ECG) without abnormal findings (e.g. any kind of atrioventricular or intraventricular conditions or blocks such as complete left or right bundle branch block, AV-node block, QTc or PR prolongation, premature atrial contractions or other atrial arrhythmia, sustained ventricular arrhythmia, 2 premature ventricular contractions (PVC) in a row, ST elevation consistent with ischemia).
14. Availability for follow-up for the planned duration of the study (26 weeks after last vaccination).

Groups 1, 2 and 3 (All vaccinia-naïve subjects) additionally:

1. No history of known or suspected previous smallpox vaccination.
2. No detectable vaccinia scar.

Group 4 (All previously vaccinated subjects) additionally:

1. History of previous smallpox vaccination (documented and/or typical vaccinia scar).
2. Most recent smallpox vaccination ≥ 5 years.

EXCLUSION CRITERIA

Groups 1, 2, 3, and 4 (All subjects):

1. Pregnant or breast-feeding women.
2. Uncontrolled serious infection i.e. not responding to antimicrobial therapy.
3. History of any serious medical condition, which in the opinion of the investigator would compromise the safety of the subject.
4. History of or active autoimmune disease. Persons with vitiligo or thyroid disease taking thyroid replacement are not excluded.
5. Known or suspected impairment of immunologic function including, but not limited to, clinically significant liver disease; diabetes mellitus; moderate to severe kidney impairment.
6. History of malignancy, other than squamous cell or basal cell skin cancer, unless there has been surgical excision that is considered to have achieved cure. Subjects with history of skin cancer at the vaccination site are excluded.
7. History or clinical manifestation of clinically significant and severe hematological, renal, hepatic, pulmonary, central nervous, cardiovascular or gastrointestinal disorders.
8. Clinically significant mental disorder not adequately controlled by medical treatment.
9. Any condition which might interfere with study objectives or would limit the subject's ability to complete the study or to be compliant in the opinion of the investigator.
10. History of coronary heart disease, myocardial infarction, angina, congestive heart failure, cardiomyopathy, stroke or transient ischemic attack, uncontrolled high blood pressure, or any other heart condition under the care of a doctor.
11. History of an immediate family member (father, mother, brother, or sister) who died due to ischemic heart disease before age 50 years.
12. Ten percent or greater risk of developing a myocardial infarction or coronary death within the next 10 years using the National Cholesterol Education Program's risk assessment tool. (http://hin.nhlbi.nih.gov/atpiii/calculator.asp?usertype=prof) NOTE: This criterion applies only to volunteers 20 years of age and older.
13. History of intravenous drug abuse.
14. History of allergic disease or reactions likely to be exacerbated by any component of the vaccine.
15. Known allergy to egg or aminoglycoside (gentamicin).
16. History of anaphylaxis or severe allergic reaction.
17. Having received any vaccinations or planned vaccinations with a live vaccine within 30 days prior or after study vaccination.
18. Having received any vaccinations or planned vaccinations with a killed vaccine within14 days prior or after study vaccination.
19. Chronic administration (defined as more than 14 days) of systemic immuno-suppressant drugs during a period starting from six months prior to administration of the vaccine and ending at study conclusion. (Corticosteroid nasal sprays are permissible. Persons who have used topical and inhaled steroids can be enrolled after their therapy is completed).
20. Post organ transplant subjects whether or not receiving chronic immunosuppressive therapy.
21. Administration or planned administration of immunoglobulins and/or any blood products during a period starting from 3 months prior to administration of the vaccine and ending at study conclusion.
22. Use of any investigational or non-registered drug or vaccine other than the study vaccine within 30 days preceding the first dose of the study vaccine, or planned administration of such a drug during the study period.
23. Study personnel.

PRIMARY IMMUNOGENICITY VARIABLE

MVA -specific seroconversion rate derived from the ELISA specific antibody titers 2 weeks after the last vaccination (Group 1-3: Visit 4, Group 4: Visit 2).

Seroconversion is defined as the appearance of antibody titers ≥ 1:50 in a vaccinia specific IgG ELISA for initially seronegative subjects or twofold increase of the antibody titer compared to the pre-existing baseline titer for subjects with a pre-existing antibody titer in the ELISA.

PRIMARY SAFETY VARIABLES

Occurrence, relationship and intensity of any specific or unspecific ECG change at any time during the study.

Occurrence, relationship and intensity of any other cardiac symptom at any time during the study.

SECONDARY VARIABLES

IMMUNOGENICITY

MVA -specific seroconversion rate derived from the ELISA specific antibody titers 4 weeks after the last vaccination (Group 1-3: Visit 5, Group 4: Visit 3).

MVA -specific seroconversion rate derived from the PRNT specific antibody titers 2 weeks after the last vaccination (Group 1-3: Visit 4, Group 4: Visit 2) and 4 weeks after the last vaccination (Group 1-3: Visit 5, Group 4: Visit 3).

Seroconversion is defined as appearance of antibody titers ≥ 1:20 in a vaccinia specific plaque reduction neutralization assay for initially seronegative subjects or twofold increase of the antibody titer compared to the pre-existing baseline titer for subjects with a pre-existing antibody titer in the PRNT.

SAFETY

Occurrence of any serious adverse event probably, possibly or definitely related to the study vaccine at any time during the study.

Occurrence of any grade 3 or higher adverse reaction probably, possibly or definitely related to the study vaccine within 28 days after vaccination.

Occurrence of solicited local adverse events within 1 week (Days 0-7) after vaccination: Intensity, duration, and relationship to vaccination.

Occurrence of solicited general adverse events within 1 week (Days 0-7) after vaccination: Intensity, duration, and relationship to vaccination.

Occurrence of unsolicited non-serious adverse events within 28 days after vaccination: Intensity, duration and relationship to vaccination.

STATISTICAL CONSIDERATIONS

The sample size calculation is based on the primary immunogenicity variable '**MVA -specific seroconversion rate'**. This parameter is derived from the ELISA specific antibody titers.

The primary hypothesis is to show that the humoral immune response of the group with history of smallpox vaccination (Group 4) is not statistically inferior compared to the group without history of smallpox vaccination (Group 1). The study should demonstrate that the Group 4 seroconversion rate is not worse than the Group 1 seroconversion rate by more than a pre-specified amount. This amount is called the non-inferiority margin (Δ).

Suppose p_1 is the seroconversion rate in the group without history of smallpox vaccination and p_4 is the seroconversion rate in the subjects with history of smallpox vaccination.
The test on non-inferiority will be applied for the following hypothesis:

H_0: $p_4 - p_1 \leq -\Delta$ versus H_1: $p_4 - p_1 > -\Delta$,
where

Δ is the non-inferiority margin and is chosen in this trial as 5%.
From the experience in a pilot trial with MVA-BN® in healthy subjects it is anticipated that the seroconversion rate in healthy vaccinia-naive subjects reaches 98-100%.

Assuming a significance level of 5%, a power of 80%, expected seroconversion rates of 98% in both groups, this yields to a sample size of 175 per group (700 in total). In order to account for drop-outs, a total of at least 180 subjects per group will be treated.

III. Flow Chart

GROUP 1, 2 AND 3 (SUBJECTS WITHOUT HISTORY OF SMALLPOX VACCINATION: 2 DOSES OF MVA-BN® OR 1 DOSE MVA-BN® / 1 DOSE PLACEBO OR 2 DOSES PLACEBO)

VISIT (V)	SCR	V 1	V 2	V 3	V 4	V 5	V F-U
	DAY -28- -1	DAY 0	V 1 + 10-15 DAYS	V 1 + 28-35 Days	V 3 + 10-15 DAYS	V 3 + 28-35 DAYS	V 3 + 182-210 DAYS
Informed consent	X						
Check incl. / excl. criteria	X	X		X			
Medical History	X						
Vaccination		I		II			
Phys. exam incl. vital signs	X	X	X	X	X	X	X
ECG	X		X		X	(X)[2]	(X)[2]
Pregnancy test[1]	X	X		X		X	
Urine analysis	X						
Safety Laboratory	X		X		X	(X)[2]	(X)[2]
Antibody analysis		X	X	X	X	X	X
Handout of diary card[3]		X		X			
Collection of diary card			X		X		
AE-reporting		X	X	X	X	X	X[4]
Review baseline signs & symptoms	X	X					
Review prior / concomitant medication	X	X	X	X	X	X	
Blood sampling in ml[5]	15	8	23	8	23	8 (+15 ML)[2]	8(+15 ML)[2]

GROUP 4 (SUBJECTS WITH HISTORY OF SMALLPOX VACCINATION): 1 DOSE OF MVA-BN®

VISIT (V)	SCR	V 1	V 2	V 3	V F-U
	DAY -28 - 1	DAY 0	V1 + 10-15 DAYS	V 1 + 28-35 DAYS	V 1 + 182-210 DAYS
Informed consent	X				
Check incl. / excl. criteria	X	X			
Medical History	X				
Vaccination		I			
Phys. exam incl. vital signs	X	X	X	X	X
ECG	X		X	(X)[2]	(X)[2]
Pregnancy test[1]	X	X		X	
Urine analysis	X				
Safety Laboratory	X		X	(X)[2]	(X)[2]
Antibody analysis		X	X	X	X
Handout of diary card[3]		X			
Collection of diary card			X		
AE-reporting		X	X	X	X[4]
Review baseline signs & symptoms	X	X			
Review concomitant medication	X	X	X	X	
Blood sampling in ml[5]	15	8	23	8	8 (+15 ML)[2]

[1] Urine or serum pregnancy test, women of childbearing potential only. At screening visit, a serum test must be performed.
[2] Only required if clinically indicated.
[3] The Diary Card should be completed daily for 7 days. If symptoms persist at day 7, temperature/symptom measurements should be recorded each day until resolved.
[4] Serious adverse events only.
[5] Amount of single blood draws: Safety laboratory: 15 ml; Antibody analysis: 8 ml. Total amount of blood taken during study: Up to 123 ml for vaccinia naïve subjects; up to 77ml for preimmune subjects

IV. Scientific Section

1. Introduction

1.1. Background information

In Germany during the period from 1960 to 1974, Professor Anton Mayr succeeded in attenuating the dermal vaccinia strain Ankara (CVA) by over 500 continuous passages in primary chicken embryo fibroblast cultures (CEF). The rationale was to attenuate the smallpox vaccine so that the complications associated with vaccination could be reduced or completely eliminated and make safer parenteral vaccination possible. This highly attenuated virus of CVA was named MVA (modified vaccinia Ankara). It was demonstrated that it had retained its original immunogenicity and its protective effect against variola and general Orthopox infections and offers a greatly diminished virulence for humans and animals combined with the loss of contagiousness. It was shown that vaccination-induced disease does not occur in newborn animals (mice, rabbits, and chickens) and immunosuppressed vaccinated animals (by radiation, iatrogenic immune suppression). Local (cutaneous, oral, and intranasal) and parenteral (subcutaneous, intramuscular) administration of MVA are both possible, safe and retain immunizing efficacy (Mayr & Munz 1964; Stickl *et al.* 1974; Stickl, Hochstein-Mintzel, Mayr, Huber, Schafer, & Holzner 1974; Mayr *et al.* 1975).

In Germany, the MVA strain was officially authorized for two-stage parenteral smallpox vaccination in children in 1976. It was used in approximately 120,000 Caucasian individuals with no reported side effects, even though many of the subjects were among the population with high risk of developing complications (Mayr *et al.* 1978).

In May 1980, the World Health Organization (WHO) declared the global eradication of smallpox. Since 1982, smallpox vaccination has not been required for international travelers' vaccination and International Certificates of Vaccination no longer include smallpox. As smallpox vaccination was deleted from the vaccination schedules, production of the vaccine was no longer required.

Despite the fact that the WHO officially declared smallpox eradicated in 1980, a new threat exists from the use of variola in biological warfare and/or bio-terrorism. Indeed, as mass vaccinations programs halted more than 20 years ago, most people have no existing immunity to smallpox, and as such the release of this highly contagious virus would have devastating effects. As a consequence, there is an urgent need by many Governments for a safe efficacious vaccine to protect the public against smallpox.

The original smallpox vaccines were based on a number of different vaccinia strains e.g. Lister-Elstree strain recommended by the WHO and used in Germany or the New York City Board of Health (NYCBH) strain used in the United States. While these proved to be highly effective immunizing agents they also showed considerable side effects. Besides from local reactions with scab development and scarring, general symptoms observed frequently after smallpox vaccination have been fever, weakness, muscular pain, headache, swelling and soreness of local lymph nodes and rashes. Fever occurred in the majority of vaccinees, especially in small children: up to 70% got temperatures of 38°C, 15-20% showed even higher rates. Apart from less dramatic and transient side effects like erythematous or urticarial rashes, severe and potentially fatal cutaneous complications of vaccinia vaccination include eczema vaccinatum and progressive vaccinia. Most feared are complications of the central nervous system, especially post-vaccinal encephalitis, followed in 15-25% by death and in 25% by neurological sequelae (Goldstein *et al.* 1975;Lane *et al.* 1969;Lane *et al.*

1970). In Germany, the occurrence of neurological complications in primary vaccinees was reported in 1:20,000 to 30,000 vaccinees and while other countries such as the United States excluded high-risk individuals from vaccination, an average of seven persons a year still died from complications due to smallpox vaccination (McElwain 1972). Another consideration for the discontinued use of the classical smallpox vaccine is the world prevalence of HIV and AIDS. The classical replication competent smallpox vaccine could be lethal if given to immune compromised individuals. A study published in 1991 (Guillaume *et al.* 1991) reported the case of two HIV infected individuals that received an experimental immunotherapy in the form of paraformaldehyde fixed autologous T cells previously infected with recombinant vaccinia viruses. Both these patients were immune compromised and experienced necrotic skin lesions due to generalized vaccinia infections that led to death. However, complications can also occur in HIV infected individuals that have a good T cell count and appear healthy following vaccinations with vaccinia (Redfield *et al.* 1987) demonstrating the severe implications of a wide spread use of vaccinia based vaccines.

Bavarian Nordic's proprietary Modified Vaccinia Ankara vector (MVA-BN®) is a live attenuated pox virus derived from Anton Mayr's seed virus used to vaccinate more than 120,000 people in Germany during the smallpox vaccination program in the late seventies. This vaccine possesses a number of characteristics that makes it highly suitable for prophylactic and therapeutic vaccination. The vaccine has a superior safety record in comparison to other viruses. The vaccine's immunogenic potential has been demonstrated in animal studies and is known from data in humans vaccinated during the two-step vaccination program in Germany (Mayr et al. 1978).

Bavarian Nordic has established a state of the art cGMP (current Good Manufacturing Practice) rated production process for MVA-BN® as well as for MVA-BN® derived recombinant viruses.

1.2. Origin and characteristics of MVA

Vaccinia virus (VV) is considered the best known member of the poxvirus family and the prototype live viral vaccine. VV replicates in the cytoplasm of the host cell, its DNA does not integrate into the host cell genome and it is non-oncogenic.

MVA was derived from the VV strain Ankara by 574 serial passages in CEF. From passage 530 the virus is named MVA and has a stabilized immunogenicity, extremely reduced transmission and no induction of a carrier status. During passaging, MVA has suffered a multitude of mutations within its genome and six major deletions resulting in the loss of 15% (30kbp) of original genetic information (Antoine *et al.* 1998). The deletions affect a number of virulence and host range genes as well as the gene for the Type A inclusion bodies (Antoine, Scheiflinger, Dorner, & Falkner 1998;Rosel *et al.* 1986;Meyer *et al.* 1991). As a consequence, MVA exhibits a severely restricted host range, and replicates only very poorly, if at all, in most mammalian cell types, including primary human cells and most transformed human cell lines (Sutter & Moss 1992;Carroll & Moss 1997;Blanchard *et al.* 1998;Drexler *et al.* 1998).

Although MVA exhibits a strongly attenuated replication in these cell types its genes are efficiently transcribed, with the block in viral replication being at the level of virus assembly and egress (Sutter & Moss 1992; Carroll & Moss 1997). Genetic reconstitution of one of the deletions in the MVA genome affecting, besides other genes, the VV host range gene K1L restored MVA replication in some mammalian cell lines, but failed to restore the growth capacity of MVA in human cells (Meyer et al. 1991; Carroll & Moss 1997).

Taken together with the loss of 15% of its genome, MVA is therefore unlikely to be able to spontaneously regain its replication competency following injection into humans. Despite its high attenuation and reduced virulence, MVA has retained its stable immunogenic properties and variola-protective effect.

MVA is a potent inducer of type I interferon (IFN) in human cells and it expresses a soluble interleukin-1 receptor (Blanchard et al. 1998), which has been implicated as an anti-virulence factor for certain poxviruses (Alcami & Smith 1992;Alcami & Smith 1996). In contrast to VV, MVA does not express soluble receptors for IFN γ, IFN α/β, tumor necrosis factor and CC chemokines (Antoine et al. 1998; Blanchard et al. 1998). MVA is known to have protective immunogenic potential against any kind of orthopox virus.

Taken together with its restricted host range these factors may explain the avirulent phenotype observed for MVA *in vivo* in a wide variety of mammalian species including humans (see below). As a consequence, MVA was used in more than 120,000 human subjects as part of a two-step vaccination protocol, combined with conventional vaccinia virus, against smallpox in Germany (Mayr et al. 1978).

MVA-BN® is a highly attenuated, purified live vaccine produced under serum-free conditions in CEF cells. Extensive in-house studies have demonstrated that MVA-BN® has superior characteristics compared to other MVA strains, which include:

- MVA-BN® has a superior attenuation compared to other MVA strains that replicate in various human cells.
- MVA-BN® vaccinations are safe in severely immune compromised animals, whereas other MVA strains replicate resulting in pathology and death.
- MVA-BN® has a stable genotype, whereas other MVA strains rapidly lose their attenuation when cultured on mammalian cells.

Therefore, it can be concluded that MVA-BN® is a promising safe human smallpox vaccine candidate.

1.3. Summary of Preclinical Data

Bavarian Nordic has performed an extensive preclinical development program for MVA-BN® that has demonstrated the safety, efficacy and bio-equivalence of MVA-BN® compared to other traditional smallpox vaccines (e.g. Dryvax® and MVA-571). Bavarian Nordic has also developed challenge models including an intratracheal monkeypox challenge model and two murine orthopox challenge models. Some key features include:

- MVA-BN® has a superior attenuation compared to other MVA strains that replicate in various human cells.
- MVA-BN® vaccinations are safe in rabbits with minimal and (in case of subcutaneous administration: “potentially”) reversible effects.
- Administration of MVA-BN® resulted in the detection of vaccine transcripts in distinct tissues with clearance of those transcripts within 7 days (with only the injection site skin and muscle being positive to a low frequency).
- MVA-BN® vaccinations are safe in severely immune compromised animals, whereas other MVA strains replicate resulting in disease outbreak and death.
- MVA-BN® demonstrated a superior efficacy in a lethal Vaccinia virus challenge model in mice compared to other MVA strains.

- MVA-BN® vaccinations induce the same efficacy as traditional smallpox vaccine (e.g. Dryvax®) against a lethal orthopox challenge given via various routes (intraperitoneal and intranasal).
- A single vaccination with MVA-BN® induces equivalent protection in mice following intranasal challenge with high lethal doses of Ectromelia virus compared to a traditional smallpox vaccine (Dryvax®).
- MVA-BN® vaccinated mice are protected from a lethal Vaccinia virus challenge applied within 4 days, whereas Dryvax vaccinated mice are not protected.
- MVA-BN® is a potent inducer of both antibody and T cells responses and induces equivalent levels of immunity as traditional smallpox vaccines (Dryvax® and Elstree) in both murine and non-human primates.
- BN has developed the first intra-tracheal (i.t.) monkeypox challenge model that more closely mimics the natural route of a smallpox infection and results in a smallpox-like disease.
- Non-human primates vaccinated with MVA-BN® are protected from a lethal i.t. monkeypox challenge demonstrating an equivalent protection to traditional smallpox vaccines (Dryvax®).
- BN is in the process of validating the monkeypox and selected murine orthopox virus challenge models according to FDA approved protocols.

For more detailed information on preclinical data please refer to the respective section of the investigator brochure.

1.4. Summary of Clinical Data

1.4.1. Clinical Experience with the MVA precursor vaccine

Non-recombinant MVA has been extensively used in humans, forming part of a two-step smallpox vaccination program in combination with wild-type vaccinia virus in Germany in 1978. More than 120,000 human subjects received i.d. and s.c. injections with MVA as part of this program, with the majority of subjects being children and adults at risk for adverse reaction from the wild-type vaccine without prior immunization with an avirulent MVA (Mahnel & Mayr 1994;Mayr, Stickl, Muller, Danner, & Singer 1978;Stickl, Hochstein-Mintzel, Mayr, Huber, Schafer, & Holzner 1974; Stickl & Hochstein-Mintzel 1971). Mild local reactions including local reddening of the skin and infiltration were observed at the site of injection in ~75% of individuals vaccinated by the intradermal route (Mayr et al. 1978). Side effects on the injection site after MVA vaccination seen in 5308 vaccinia-naive individuals (0.2 ml intradermally in the forearm) are shown in table I.

Table I: Local reactogenicity 24-48 hours and 5-7 days after vaccination with MVA

LOCAL REACTION	24-48 HOURS	5-7 DAYS
No reaction	7.25%	9.17%
Reddening up to 10 mm in diameter	54.9%	75.45%
Reddening 10-20 mm in diameter	31.45%	14.05%
Reddening more than 20 mm in diameter	6.40%	1.53 %

No blistering, pustula or ulceration were observed at virus doses that produce such reactions in approximately 50% of naive individuals after application of conventional vaccinia. This indicated that the virulence of MVA for skin is very low. No encephalitic reaction was observed (Stickl et al. 1974).

Approximately 2-4% of the subjects (n = 7098) had slight fever and felt unwell (fever > 38°C in 2.28%, non-specific systemic symptoms in 4.11%), but the symptoms were much less than those observed with the normal smallpox vaccine. No serious adverse events were reported. In addition, vaccination with MVA before administering the normal smallpox vaccine (replication competent wild-type vaccinia virus) resulted in a reduced number of side effects from this administration and the development of smaller wild-type vaccinia-derived pocks.

Single dose vaccination with MVA elicited only weak hemagglutination inhibiting or virus neutralizing antibody response. However, after vaccination with wild-type vaccinia virus vaccinees presented with a strong booster effect, indicating the priming by MVA with specific humoral and cellular immune response (Mayr et al. 1978). In vaccinees having received a second dose of MVA, an increased level of antibody response was observed as well. It could be shown in animal experiments that protection against variola was provided even in the absence of an antibody response (Stickl et al. 1974).

1.4.2. Clinical Experience with MVA-BN®

To date, 16 clinical studies with MVA-based vaccines have been initiated and more than 1600 subjects were vaccinated. The populations vaccinated include risk groups for which conventional smallpox vaccines are contraindicated such as HIV infected patients and patients with atopic dermatitis. The majority of the vaccinated subjects received MVA-BN® **whereas** 150 subjects were vaccinated with several other recombinant MVA-based vaccines.

Vaccination of healthy population with MVA-BN®

Study POX-MVA-001: 86 healthy volunteers were vaccinated in this clinical phase I trial conducted in Europe. The subjects were healthy males, stratified based on the presence or absence of a prior history of smallpox vaccination. Primary objective was to assess safety and tolerability of MVA-BN® with secondary objectives comparing various routes of administration (subcutaneous [s.c.] versus intramuscular [i.m.]) with three different doses of vaccine being tested. There were five cohorts, with three cohorts receiving the vaccine s.c. at a dose of 10^6, 10^7, and 10^8 tissue culture infectious dose 50 ($TCID_{50}$), respectively. A fourth cohort received the vaccine i.m. at a dose of 10^8 $TCID_{50}$. All four cohorts were naïve to smallpox vaccination and received vaccine at baseline and week 4. A fifth cohort of subjects, who had been previously vaccinated against smallpox, received a single dose of $1x10^8$ $TCID_{50}$ of MVA-BN® s.c. In this study the vaccine induced a strong and dose dependent immune response and was safe and well tolerated even at high doses.

Study POX-MVA-004: This double-blind randomized, phase II, dose finding trial in 165 healthy, vaccinia naïve male and female volunteers aged 18–32 years evaluated the immunogenicity and safety of MVA-BN® at doses of $2x10^7$, $5x10^7$ or $1x10^8$ $TCID_{50}$. Subjects were randomly assigned to one of three treatment groups and were immunized twice at a 4 week interval using MVA-BN® (s.c.). Results of PRNT and ELISA, detecting neutralizing antibodies and total IgG against vaccinia respectively are shown in Table II.

Table II: POX-MVA-004 Humoral immune response (per protocol population

			ELISA			PRNT		
**Group**	**Visit Day**	**N**	**n (%)**	**GMT**	**95% CI (LL / UL)**	**n (%)**	**GMT**	**95% CI (LL / UL)**
Group 1 (N=54) $2x10^7$ $TCID_{50}$	0	54	0 (0%)	1.00	- / -	0 (0%)	1.00	- / -
	28	54	32 (59.3%)	14.37	7.71 / 26.8	4 (7.4%)	1.31	1.01 / 1.70
	42	54	54 (100%)	377.22	288.33 / 493.53	23 (42.6%)	5.51	3.17 / 9.59
	84	54	51 (94.4%)	134.33	91.05 / 198.19	10 (18.5%)	1.94	1.31 / 2.85
Group 2 (N=49) $5x10^7$ $TCID_{50}$	0	49	0 (0%)	1.00	- / -	0 (0%)	1.00	- / -
	28	49	40 (81.6%)	53.21	29.86 / 94.87	6 (12.2%)	1.55	1.10 / 2.19
	42	49	49 (100%)	583.62	461.58 / 737.94	29 (59.2%)	10.31	5.78 / 18.40
	84	49	49 (100%)	227.76	176.40 / 294.07	11 (22.4%)	2.32	1.47 / 3.66
Group 3 (N=52) $1x10^8$ $TCID_{50}$	0	52	0 (0%)	1.00	- / -	0 (0%)	1.00	- / -
	28	52	49 (94.2%)	98.52	67.57 / 143.65	5 (9.6%)	1.39	1.05 / 1.85
	42	52	52 (100%)	813.77	628.74 / 1053.26	37 (71.2%)	19.43	11.05 / 34.16
	84	52	52 (100%)	323.63	246.84 / 424.30	15 (28.8%)	2.94	1.81 / 4.76

N = number of subjects with samples for antibody response, n (%) = number of seropositive subjects / percentage of seropositive subjects based on N, GMT = Geometric Mean Titer, CI = Confidence Interval, LL: Lower Limit, UL: Upper Limit
Seropositivity rate was defined as the percentage of subjects with antibody titers ≥ the assay cut-off value.
ELISA: The assay cut-off value is 1:50; PRNT: The assay cut-off value is 1:20.

Study POX-MVA-004 revealed that all three dose levels induced a 100% seroconversion after the second vaccination. Seroconversion was defined as the appearance of antibody titers ≥ 1:50 in a vaccinia specific IgG ELISA for initially seronegative subjects. The strongest immune response with earliest onset of seroconversion was achieved with the highest dose tested. In this group, 94% of the subjects already seroconverted after only 1 vaccination. The data obtained with neutralizing antibodies closely correlated to the ELISA results (Figure I). A significance test of the correlation shows a highly significant result: p = 1.5E-18 and confirms that both assays are highly correlated. T cell response data: Cellular immune responses were analyzed in this trial with intracellular cytokine staining (ICS) detecting vaccinia-specific IFN-γ producing CD4+ / CD8+ cells. Cellular immune response resulted in a dose-dependent CD8+ response (Figure II).

Figure I: POX-MVA-004 ELISA/PRNT Correlation analysis

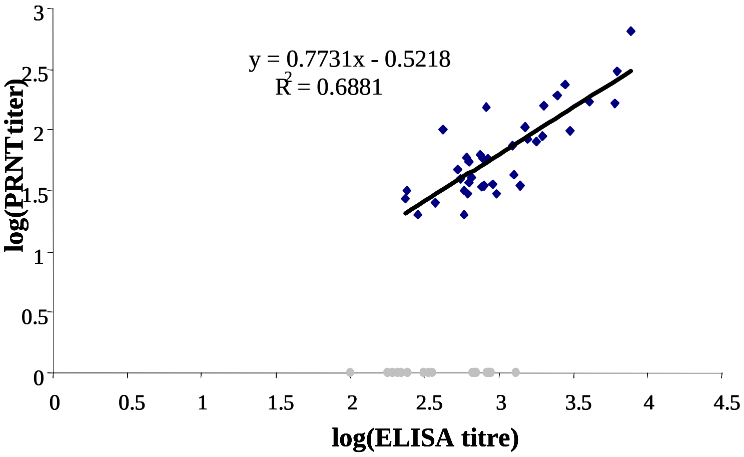

The ELISA titer versus the PRNT titer for all 162 available day 42 samples. The correlation calculation is based only on the samples where both titer values are positive – 94 positive pairs in all. 75% of the negative PRNT values had (log10 of) ELISA titers below 2.73, whereas all the samples with a positive PRNT titer had (log10 of) ELISA titers above 2.34.

Figure II: POX-MVA-004 Intracellular cytokine staining / vaccinia specific immunity

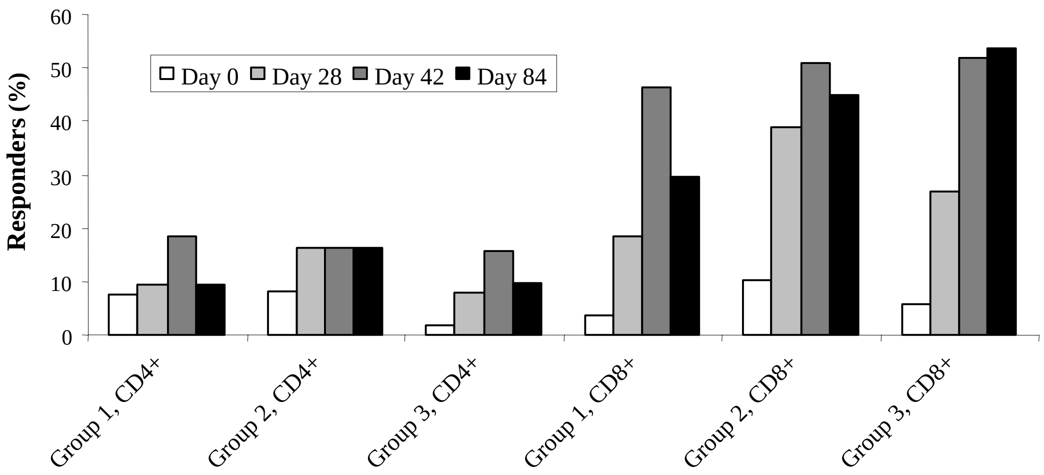

Group 1: 2x 10^7 $TCID_{50}$, subcutaneous in subjects not vaccinated against smallpox
Group 2: 5x 10^7 $TCID_{50}$, subcutaneous in subjects not vaccinated against smallpox
Group 3: 1x 10^8 $TCID_{50}$, subcutaneous in subjects not vaccinated against smallpox
Analysis was performed on the per protocol population

Study POX-MVA-002: In addition MVA-BN® is currently being tested in the US in a Phase I study (POX-MVA-002) in healthy subjects in combination with the licensed smallpox vaccine Dryvax®.

A total of 90 subjects were enrolled 75 of whom have received MVA-BN® in different doses. Currently, the study is still blinded and it is expected that final results of the study will become available during 2006. However no serious and unexpected adverse events related to MVA-BN® have been reported. One subject reported shortness of breath 2 weeks after vaccination with Dryvax® and this cardiac adverse event was assessed by the investigator as definitely being related to Dryvax®.

MVA-BN®-based vaccination of populations for which Dryvax® is contraindicated

Patients belonging to risk populations such as immune compromised (e.g. HIV infected patients) or otherwise immunologically impaired (e.g. patients with atopic disorders) are normally excluded from vaccination with traditional smallpox vaccines (e.g. Dryvax®). To show that MVA-BN® is safe and immunogenic even in these patients, Bavarian Nordic up to date initiated three clinical trials in HIV infected subjects and/or subjects with atopic dermatitis with MVA-BN® or MVA-BN® based vaccines. Initial trials in HIV-1 infected subjects using the recombinant MVA-BN® based Nef vaccine are completed and study reports available.

Studies HIV-NEF-001 and HIV-NEF-002: Both phase I vaccination studies conducted in Germany had the objective to evaluate safety and tolerability of a recombinant MVA vaccine expressing the HIV-1 nef-gene (MVA-nef) in HIV-1 infected patients on HAART. In study HIV-NEF-002 the subjects underwent a structured therapy interruption after the third vaccination.
Twenty-four patients with CD4 counts ≥ 400/µl were enrolled and immunized with $5x10^8$ $TCID_{50}$ of the MVA-nef vaccine, administered subcutaneously.
In both studies it was demonstrated that $5x10^8$ $TCID_{50}$ MVA-nef was safe and well tolerated in HIV-1 infected patients with CD4 counts ≥ 400/µl. The most frequent adverse reactions were mild injection site reactions like induration or pain.

Study POX-MVA-007: To evaluate safety and immunogenicity of MVA-BN® smallpox vaccine in subjects with atopic disorders, this open-label, controlled phase I pilot study was started in Germany in May 2004.

Primary objective of the trial was to expand the available data on the safety of MVA-BN® in a population with atopic disorders. Vaccinia-naïve volunteers between 18 and 40 years were assigned to the following study groups depending on their medical history and current health status:

- Healthy subjects (n=15)
- Subjects with history of atopic dermatitis (n=16)
- Subjects with mild active atopic dermatitis (n=15)
- Subjects with mild allergic rhinitis (n=14)

All subjects are immunized twice at a 4 week interval using MVA-BN® subcutaneously in a dose of $1x10^8$ $TCID_{50.}$ The study duration is up to 36 weeks for each subject. All subjects will be followed for at least 24 weeks after having received the last study vaccination.

A full safety report of all 60 subjects in this study is available. This safety report demonstrates a low number of grade 3 adverse events (AEs) and confirms a favorable safety profile of MVA-BN® (IMVAMUNE™) even in a population in which conventional smallpox vaccines are contraindicated. Non-serious unsolicited and solicited AEs documented in this report were comparable to typical adverse reactions expected with other modern injectable vaccines (Table III).

Table III:Adverse reactions with an occurrence of > 10% in at least one subject group (29-day follow-up period after vaccination) - per subject (full analysis set, N = 60)

Symptom	Healthy (N* = 15)			History of atopic dermatitis (N* = 16)			Active atopic dermatitis (N* = 15)			Allergic rhinitis (N* = 14)		
	n	(%)	n*	n	(%)	n*	n	(%)	n*	n	(%)	n*
Injection site pain	14	(93.3)	27	14	(87.5)	25	15	(100.0)	30	14	(100.0)	27
Injection site induration	13	(86.7)	23	11	(68.8)	17	15	(100.0)	23	12	(85.7)	18
Injection site swelling	12	(80.0)	22	13	(81.3)	20	11	(73.3)	18	11	(78.6)	17
Injection site erythema	12	(80.0)	23	15	(93.8)	26	15	(100.0)	25	14	(100.0)	26
Injection site pruritus	3	(20.0)	5	4	(25.0)	5	5	(33.3)	7	1	(7.1)	1
Inj. site discolouration	2	(13.3)	3	0	(0.0)	0	1	(6.7)	1	1	(7.1)	1
Injection site warmth	0	(0.0)	0	2	(12.5)	2	1	(6.7)	1	0	(0.0)	0
Fatigue	5	(33.3)	6	10	(62.5)	13	7	(46.7)	9	9	(64.3)	13
Headache	6	(40.0)	7	7	(43.8)	12	5	(33.3)	6	6	(42.9)	6
Myalgia	0	(0.0)	0	6	(37.5)	9	2	(13.3)	4	4	(28.6)	4
Nausea	1	(6.7)	1	1	(6.3)	1	2	(13.3)	2	2	(14.3)	2
Pyrexia	1	(6.7)	1	2	(12.5)	2	3	(20.0)	3	0	(0.0)	0
Pruritus	0	(0.0)	0	0	(0.0)	0	2	(13.3)	3	0	(0.0)	0
Dermatitis atopic	0	(0.0)	0	0	(0.0)	0	2	(13.3)	2	0	(0.0)	0

% = Percentages based on N*, N = number of subjects, N* = number of subjects with documented vaccination periods, n = number of subjects with findings in specific category, n* = number of events

Study POX-MVA-010: In July 2005, BN initiated a multi-center, open-label phase I/II study with the objective to generate safety and immunogenicity data with MVA-BN® in HIV infected subjects with CD4+ counts >350 / µl compared to healthy subjects. 150 male and female vaccinia-naïve and pre-immune volunteers are assigned to the following study groups depending on their medical history and health status:

Group 1: 30 HIV infected subjects without history of previous smallpox vaccination
Group 2: 60 HIV infected subjects with history of previous smallpox vaccination
Group 3: 30 Healthy subjects without history of previous smallpox vaccination
Group 4: 30 Healthy subjects with history of previous smallpox vaccination

The subjects are immunized (s.c.) with a MVA-BN® (IMVAMUNE™) dose of 1 x 10^8 $TCID_{50}$. Group 1 and 3 will receive two vaccine administrations 4 weeks apart; group 2 and 4 a single administration.
The study is being performed at 5 study centers in the US and enrollment of volunteers has been completed.

Solicited local and systemic adverse reactions ≥ grade 3 observed to date are shown in Table IV.

Table IV:POX-MVA-010 (un-cleaned data) -- Solicited local and systemic adverse reactions ≥ grade 3

Symptom	HIV vacc. naïve (N=30)	*HIV pre-immune (N=61)*	Healthy vacc. naïve (N=30)	Healthy pre-immune (N=30)
	n (%)	*n (%)*	*n (%)*	*n (%)*
Injection site pain	***0 (0%)***	***2 (3.3%)***	***2 (6.7%)***	***1 (3.3%)***
Injection site erythema	***0 (0%)***	***1 (1.6%)***	***0 (0%)***	***1 (3.3%)***
Injection site swelling	***0 (0%)***	***0 (0%)***	***0 (0%)***	***0 (0%)***
Injection site induration	***0 (0%)***	***0 (0%)***	***0 (0%)***	***0 (0%)***
Fever	***0 (0%)***	***1 (1.6%)***	***0 (0%)***	***1 (3.3%)***
Headache	***1 (3.3%)***	***0 (0%)***	***2 (6.7%)***	***1 (3.3%)***
Myalgia	***0 (0%)***	***1 (1.6%)***	***1 (3.3%)***	***1 (3.3%)***
Chills	***1 (3.3%)***	***1 (1.6%)***	***2 (6.7%)***	***1 (3.3%)***
Nausea	***0 (0%)***	***1 (1.6%)***	***0 (0%)***	***2 (6.7%)***
Fatigue	***1 (3.3%)***	***3 (4.9%)***	***2 (6.7%)***	***0 (0%)***

N=Number of subjects, n=Number of subjects with respective symptom, %=n/N

Study HIV-NEF-004: This phase II study is currently being conducted in Germany to evaluate immunogenicity and safety of two doses of a recombinant MVA vaccine expressing the HIV-1 nef-gene ($1x10^8$ and $5x10^8$ MVA-nef) and one dose of $1x10^8$ $TCID_{50}$ MVA-BN®. A total of 77 HIV-1 infected subjects with CD4 counts > 250/µl have been enrolled and have received all 3 vaccinations. To date, MVA-nef and MVA-BN® are well tolerated. Most frequent adverse reactions are mild injection site reactions like induration and pain.

1.5. Dose, route of administration, vaccination schedule

MVA-BN® will be given subcutaneously. The rationale for the selected dose and the route of administration comes from historical use as well as preclinical and clinical data (study V-POX-00.1 (POX-MVA-001)) that showed satisfactory immune responses after a $1x10^8$ $TCID_{50}$ dose and a good safety profile of MVA-BN® if administered via the subcutaneous route.
All subjects in groups 1-3 will receive two vaccinations each with a dose of 0.5 ml MVA-BN® containing $1x10^8$ $TCID_{50}$ or placebo. Injections will be administered subcutaneously according to a 0 – 4 weeks schedule (Day 0 / Day 28-35). Subjects in group 4 will receive one vaccination with a dose of 0.5 ml MVA-BN® containing $1x10^8$ $TCID_{50}$.

1.6. Benefit/risk assessment

The safety profile of MVA-BN® is supported by the historical experience with the MVA-BN® precursor vaccine, which was used in Germany during the smallpox vaccination for two-stage parenteral vaccination in children in 1976. MVA was used in over 120,000 Caucasian individuals with no reported major side effects, even though some of the subjects were among the population with high risk of developing complications (Mayr et al., 1978).

It could be concluded that from the previous experience with MVA based vaccines, adverse reactions to MVA-BN® are expected to be comparable to typical adverse reactions seen with other modern vaccines.

The severe and life-threatening adverse reactions like progressive vaccinia, eczema vaccinatum, generalized vaccinia and inadvertent inoculation that are observed after the administration of conventional smallpox vaccines are due to the replication of the vaccinia strains. Since MVA-BN® is replication incompetent in human cells it has a better safety and tolerability profile and can consequently not induce the severe side effects like progressive vaccinia which are associated with the replication of the vaccine virus. Apart from the better safety profile with regard to severe reactions the available clinical experience with MVA-BN® shows that it is generally better tolerated (e.g. local reaction without scar formation) than conventional smallpox vaccines.

Based on the available data with MVA-BN® the risk for the study participants is not expected to be higher than the risk observed with other modern vaccines. The main risk is the development of local reactions at the vaccination site.

In addition, the study participants can contribute significantly to the development of a safe smallpox vaccine. They might acquire a potential protection against smallpox. However, it cannot be concluded that the vaccine is efficacious against this disease, as MVA based vaccines are currently in an early phase of development. In view of the expected side effects of MVA-BN® the potential risks associated for the study participants in this study seem to be limited and justify the potential benefit for society.

Preclinical data with MVA-BN® reveal no special hazard for humans based on conventional studies of safety. Over 1250 healthy and over 250 subjects with conditions like HIV infection, melanoma or atopic dermatitis have been vaccinated in clinical trials with vaccines using MVA-BN® alone (to develop an improved smallpox vaccine) or with MVA-BN® as vector (with HIV specific inserts or inserts to be used as melanoma vaccine).

2. Objectives

The primary immunogenicity objective of the trial is:

- To compare the ELISA specific humoral immune response of the group with history of smallpox vaccination (Group 4: 1 dose MVA-BN®) versus the group without history of smallpox vaccination (Group 1: 2 doses MVA-BN®), i.e. to prove that a single dose schedule would be sufficient to boost the immunity in a previously vaccinated population.

The primary safety objective of the trial is:

- To compare the four different vaccination groups with regard to ECG changes and cardiac symptoms.

The secondary objectives of the trial are:

- To compare the ELISA specific immune response of the four different vaccination groups - comparison combinations not done for the primary objective.
- To compare the four different vaccination groups with regard to immune response measured with a plaque reduction neutralization test.
- To assess the kinetics of the immune responses after 1 (Group 2) and 2 (Group 1) doses of MVA-BN® in vaccinia naïve subjects.
- To compare the four different vaccination groups with regard to safety and reactogenicity.
- To compare the safety of one (Group 2) and two (Group 1) vaccinations with MVA-BN® in vaccinia-naïve subjects with placebo (Group 3) and historical / recently published data of Dryvax® immunization in vaccinia-naive subjects.

3. Study design

3.1. Experimental design

This study is a partially randomized, partially double-blind, placebo-controlled, phase II non-inferiority trial to evaluate immunogenicity and safety of one and two doses of MVA-BN® smallpox vaccine in 18-55 year old healthy subjects.

In this study the following four subject groups will be treated as described below:

Double-blind:

Group 1: Subjects without history of smallpox vaccination (2 doses MVA-BN®)

Group 2: Subjects without history of smallpox vaccination (1st dose MVA-BN® / 2nd dose placebo)

Group 3: Subjects without history of smallpox vaccination (2 doses placebo)

Open-label:

Group 4: Subjects with history of smallpox vaccination (1 dose MVA-BN®)

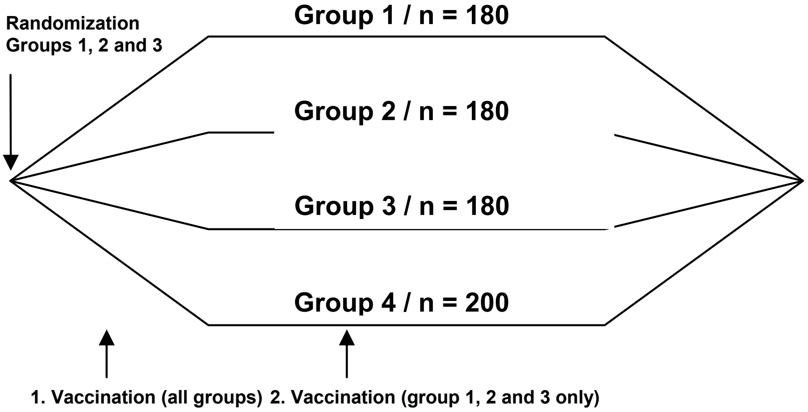

Visit Days (Groups 1, 2, 3)

**-28**	**0**	**10-15**	**28 – 35**	**V3 + 10-15**	**V3 + 28-35**	**V3 + 182–210**
**Scr**	**V1**	**V2**	**V3**	**V4**	**V5**	**F-U**

Visit Days (Group 4)

**-28**	**0**	**10-15**	**28 - 35**	**V1 + 182–210**
**Scr**	**V1**	**V2**	**V3**	**F-U**

3.2. Discussion of study design and choice of control groups

The primary objective of this study is to demonstrate that the booster immune response elicited by a single vaccination in a population previously vaccinated against smallpox (Group 4) is non-inferior to a vaccinia-naive population which receives two vaccinations (Group 1), i.e. to prove that a single dose schedule would be sufficient to boost the immunity in a previously vaccinated population.

Group 3 serves as an internal control to validate the trial results and to show sensitivity of the trial. In addition Groups 2 and 3 are important for the assessment of the secondary variables of the study and to generate hypotheses which might prove to be important and which should be addressed in the further clinical development plan for MVA-BN®.

Group 2, which receives only a single vaccination with MVA-BN® followed by a placebo vaccination, is necessary for the comparison of the immune response after only one vaccination in a vaccinia-naive population with the immune response induced by two vaccinations in such a population (Group 1) and the immune response after a single vaccination in a previously vaccinated population (Group 4).

In addition the study is designed to further investigate the safety profile of MVA-BN® in a healthy population. Recent findings of the US vaccination smallpox campaign (Grabenstein et. al.) and previous clinical trials indicated that after vaccination with the replication

competent NYCBOH vaccinia strain, cardiac side effects occurred in an unexpected high frequency. Results of controlled clinical trials with the first and second generation smallpox vaccines Dryvax® and ACAM2000 demonstrated 8 cases of myo-/pericarditis in 1162 vaccinated study subjects, i.e. an incidence of 1:145 or 0.69% (Acambis news release Sep. 20th, 2004). Importantly, the events occurred only in a vaccinia-naive population, whereas a population previously vaccinated against smallpox did not report any cardiac adverse reactions. While the underlying mechanism of NYCBOH-related myo-/pericarditis is unknown, specific mechanisms (“molecular mimicry”) as well as unspecific immunologically triggered mechanisms are being discussed. Thus, it has been stipulated by regulatory authorities to focus on the cardiac safety profile of all other vaccinia-based vaccines in development.
To date, >500 subjects have been vaccinated in clinical trials with MVA-BN®. No case of myo-/pericarditis or any other unexpected adverse reactions have been detected. To further evaluate the cardiac safety profile of MVA-BN®, the comparison of the four groups with regard to ECG changes and cardiac symptoms was chosen as co-primary safety objective. The main comparisons will be Group 1 (vaccinia naïve, 2 vaccinations) with placebo (Group 3), Group 2 (1 vaccination) with placebo and Group 4 with placebo. Additionally, a comparison of Group 1 vs. Group 2 and Group 1 vs. Group 4, as well as pooled analyses of Groups 1, 2 and Groups 1, 2, 4 vs. Group 3 will be performed.

A sample size of 200, 400 and 600 will be sufficient to detect reactions reported at a frequency of less than 1/50, 1/100 and 1/200 respectively.

3.3. Description of study procedures

The study will be conducted according to the Study Flow Chart (section III).

Visits should be scheduled within the given intervals.

3.3.1. Screening phase

Screening Visit (SCR, day -28 to –1 / week –4 to -1)

The informed consent must be reviewed with and signed and dated by the subject (volunteer) prior to the initiation of any evaluations or procedures required by the protocol. All subjects must be thoroughly informed of all aspects of the study (e.g. study visit schedule, required evaluations and procedures) as described in the informed consent document.

After informed consent has been collected, subjects will enter a screening period of up to four weeks before the first vaccination.

An extensive screening assessment will be performed during the screening period (visit SCR).

All Groups
**VISIT SCR (DAYS -28 TO -1)**<br>• Informed Consent<br>• Check inclusion/exclusion criteria<br>• Check medical history<br>• Physical examination including evaluation of vital signs, especially listening to heart and lung<br>• Perform baseline ECG<br>• Serum pregnancy test (if applicable)<br>• Urine analysis<br>• Safety laboratory<br>• Review baseline signs & symptoms<br>• Review prior medication

If a subject was screened and could not be enrolled because of a certain transient condition (e.g. abnormal lab value due to an acute condition or a missing lab evaluation due to mishandling of the sample), then the subject may be re-screened and the respective tests have to be repeated as a "partial" re-screening rather than a full re-screening. The re-screening visit must be within the 28 days window started by the first screening visit and the window days -28 to -1 before 1st vaccination must not be exceeded.

If a subject could not be enrolled due to other circumstances (e.g. intermediate closure of the study group because of an interim safety analysis) or the 28 day period is exceeded, a complete re-screening assessment including physical examination, lab examination, ECG must be performed. The clock then re-starts at the re-screening visit with day -28 before 1st vaccination.

3.3.2. Allocation of subjects to treatment groups

Subjects eligible for Groups 1, 2 and 3 will be randomly allocated to one of these three treatment groups in order of their appearance. The randomization list will be provided by Bavarian Nordic A/S and will be deposited in the eCRF for randomization. At investigational site, only the study independent person preparing the study medication and the medication monitor will be unblinded. In case of emergency which makes unblinding necessary, unblinding of the respective subject can be done by the investigator by means of the eCRF. In addition, the Safety Department of Bavarian Nordic (contact details listed in chapter 8.6) has also access to the randomization list as back up for unblinding.

Subjects eligible for Group 4 will be allocated to Group 4 in order of their appearance.

3.3.3. Active study phase

After successful screening period the subject will run in the active study phase starting with Visit 1.
The procedures performed at Visit 1 and all following visits are listed below. Blood draws and all other tasks mentioned in the list above the vaccination event must always be performed **prior** to vaccination!

Each immunization consists of 1 x 10^8 $TCID_{50}$ MVA-BN® vaccine or placebo (0.5 ml Tris

Buffer), administered subcutaneously in the non-dominant upper arm. Subjects in Groups 1, 2 and 3 will receive two doses each, four weeks apart (optional up to +7 days); depending on the group two doses of MVA-BN®, one dose MVA-BN® and one dose placebo, or two doses of placebo (see section 3.1). Subjects in Groups 1, 2 and 3 will be double-blinded.
Study Group 4 will receive one vaccination with MVA-BN® at Visit 1. This group will be open-label.

Following vaccination subjects will be kept under close observation for at least 30 minutes, with appropriate medical treatment readily available in case of a rare anaphylactic reaction following the administration of vaccines.

Any adverse events (AE) that occur during or after vaccination will be recorded. The subjects will be issued with a diary card for the recording of solicited AEs occurring on the vaccination day and the seven days following vaccination.

<table>
<tr><th colspan="2">All Groups</th></tr>
<tr><td colspan="2">VISIT 1 (DAY 0)
Re-check inclusion/exclusion criteria
Physical examination including evaluation of vital signs, especially listening to heart and lung
Urine pregnancy test (if applicable)
Blood for Antibody analysis
Review baseline signs & symptoms
Review prior medication
Blood draws and all other tasks mentioned above must always be performed prior to vaccination!
1st vaccination
Handout of diary card for 1st vaccination
Recording and documentation of AEs
Review concomitant medication</td></tr>
<tr><td colspan="2">VISIT 2 (VISIT 1 + 10-15 DAYS)
Physical examination including evaluation of vital signs, especially listening to heart and lung
Perform ECG
Safety laboratory
Blood for Antibody analysis
Collection of the diary card for (1st) vaccination, review together with subject
Recording and documentation of AEs
Review concomitant medication</td></tr>
<tr><th>Groups 1, 2 and 3</th><th>Group 4</th></tr>
</table>

**VISIT 3 (VISIT 1 + 28-35 DAYS)**<br>• Re-check inclusion/exclusion criteria<br>• Physical examination including evaluation of vital signs, especially listening to heart and lung<br>• Urine pregnancy test (if applicable)<br>• Blood for Antibody analysis<br><br>Blood draws and all other tasks mentioned above must always be performed prior to vaccination!<br><br>• 2nd vaccination<br>• Handing over of the diary card for 2nd vaccination<br>• Recording and documentation of AEs<br>• Review concomitant medication	**VISIT 3 (VISIT 1 + 28-35 DAYS)**<br>• Physical examination including evaluation of vital signs, especially listening to heart and lung<br>• Urine pregnancy test (if applicable)<br>• ECG, only if clinically indicated<br>• Safety laboratory, only if clinically indicated<br>• Blood for Antibody analysis<br>• Recording and documentation of AEs<br>• Review concomitant medication
**VISIT 4 (VISIT 3 + 10-15 DAYS)**<br>• Physical examination including evaluation of vital signs, especially listening to heart and lung<br>• Perform ECG<br>• Safety laboratory<br>• Blood for Antibody analysis<br>• Collection of the diary card for 2nd vaccination, review together with subject<br>• Recording and documentation of AEs<br>• Review concomitant medication	
**VISIT 5 (VISIT 3 + 28-35 DAYS)**<br>• Physical examination including evaluation of vital signs, especially listening to heart and lung<br>• Urine pregnancy test (if applicable)<br>• ECG, only if clinically indicated<br>• Safety laboratory, only if clinically indicated<br>• Blood for Antibody analysis<br>• Recording and documentation of AEs<br>• Review concomitant medication	

3.3.4. Follow-up phase

To secure long-term safety, the subject has to come in for an examination in a follow-up phase 26 (+4) weeks after the last vaccination.

Groups 1, 2 and 3<br>VISIT F-U (VISIT 3 + 182 – 210 DAYS)	Group 4<br>VISIT F-U (VISIT 1 + 182 – 210 DAYS)
• Physical examination including evaluation of vital signs, especially listening to heart and lung<br>• ECG, only if clinically indicated<br>• Safety laboratory only if clinically indicated<br>• Blood for Antibody analysis<br>• Recording and documentation of Serious Adverse Events (SAEs)	

3.4. Study duration

The total duration of the study including the screening period and follow-up visit will be

- Up to 39 weeks for each subject in study Groups 1, 2 and 3 (vaccinia naïve subjects) and
- Up to 34 weeks for each subject in study Group 4 (pre-immune subjects)

All subjects will be followed for at least 26 weeks after having received the last study vaccination. It is expected to recruit subjects within a reasonable short time, to limit the total study duration to 11 months.

4. Selection of subjects

4.1. Recruitment procedure

Volunteers will be recruited actively. Methods of recruitment may include letters, e-mails, posters, paid advertisements, announcements on campuses and distribution of flyers.

In total, 740 individuals will be enrolled as defined in Section 4.2. After signing the Informed Consent, subjects undergo screening procedures to check eligibility regarding to the following inclusion/exclusion criteria. In the event of a screening failure secondary to mild or limited acute illness or abnormal laboratory values the subject may be re-screened after resolution of the event. Re-screening may require only an additional blood draw or a complete re-screening evaluation, depending on the circumstances of and the time interval from the initial screening failure. Partial rescreening is to be done within the 28 days screening period, while complete re-screening is required in case the 28 days screening period has exceeded.

The investigator will keep a log of subjects screened for the study, but not enrolled, and provide the reason for exclusion.

Bavarian Nordic will be informed about every subject entered in the study via the eCRF and weekly status reports.

4.2. Inclusion criteria

All groups

1. Male and female subjects between 18 and 55 years of age.
2. Women must have a negative serum pregnancy test at screening and a negative urine or serum pregnancy test within 24 hours prior to vaccination.
3. Women of childbearing potential must have used an acceptable method of contraception for 30 days prior to the first vaccination, must agree to use an acceptable method of contraception during the study, and must not become pregnant for at least 28 days after the last vaccination. A woman is considered of childbearing potential unless post-menopausal or surgically sterilized. (Acceptable contraception methods are restricted to abstinence, barrier contraceptives, intrauterine contraceptive devices or licensed hormonal products.)
4. Read, signed and dated informed consent document after being advised of the risks and benefits of the study in a language understood by the subject signed, and prior to performance of any study specific procedure.
5. Troponin I within normal institutional limits.
6. White blood cells ≥ 2500/mm^3 and ≤ 11,000/ mm^3.
7. Absolute neutrophil count (ANC) ≥ 1000/mm^3.
8. Negative urine glucose by dipstick or urinalysis.
9. Hemoglobin ≥ LLN.
10. Platelets 100 – 450/nl.
11. Adequate renal function defined as:
    c. Serum creatinine without clinically significant findings.
    d. Urine protein ≤ 30 mg/dL or none or trace proteinuria (by urinalysis or dip stick).
12. Adequate hepatic function defined as:
    c. Total bilirubin ≤ 1.5 x ULN in the absence of other evidence of significant liver disease (healthy subjects without clinical disease with Morbus Meulengracht can be included).
    d. AST (SGOT), ALT (SGPT) and alkaline phosphatase without clinically significant findings.
13. Electrocardiogram (ECG) without abnormal findings (e.g. any kind of atrioventricular or intraventricular conditions or blocks such as complete left or right bundle branch block, AV-node block, QTc or PR prolongation, premature atrial contractions or other atrial arrhythmia, sustained ventricular arrhythmia, 2 premature ventricular contractions (PVC) in a row, ST elevation consistent with ischemia).
14. Availability for follow-up for the planned duration of the study (26 weeks after last vaccination).

Groups 1, 2 and 3 (All vaccinia-naïve subjects) additionally:

1. No history of known or suspected previous smallpox vaccination.
2. No detectable vaccinia scar.

Group 4 (All previously vaccinated subjects) additionally:

1. History of previous smallpox vaccination (documented and/or typical vaccinia scar).
2. Most recent smallpox vaccination ≥ 5 years.

4.3. Exclusion criteria

Groups 1, 2, 3, and 4 (All subjects):

1. Pregnant or breast-feeding women.
2. Uncontrolled serious infection i.e. not responding to antimicrobial therapy.
3. History of any serious medical condition, which in the opinion of the investigator would compromise the safety of the subject.
4. History of or active autoimmune disease. Persons with vitiligo or thyroid disease taking thyroid replacement are not excluded.
5. Known or suspected impairment of immunologic function including, but not limited to, clinically significant liver disease; diabetes mellitus; moderate to severe kidney impairment.
6. History of malignancy, other than squamous cell or basal cell skin cancer, unless there has been surgical excision that is considered to have achieved cure. Subjects with history of skin cancer at the vaccination site are excluded.
7. History or clinical manifestation of clinically significant and severe hematological, renal, hepatic, pulmonary, central nervous, cardiovascular or gastrointestinal disorders.
8. Clinically significant mental disorder not adequately controlled by medical treatment.
9. Any condition which might interfere with study objectives or would limit the subject's ability to complete the study or to be compliant in the opinion of the investigator.
10. History of coronary heart disease, myocardial infarction, angina, congestive heart failure, cardiomyopathy, stroke or transient ischemic attack, uncontrolled high blood pressure, or any other heart condition under the care of a doctor.
11. History of an immediate family member (father, mother, brother, or sister) who died due to ischemic heart disease before age 50 years.
12. Ten percent or greater risk of developing a myocardial infarction or coronary death within the next 10 years using the National Cholesterol Education Program's risk assessment tool. (http://hin.nhlbi.nih.gov/atpiii/calculator.asp?usertype=prof) NOTE: This criterion applies only to volunteers 20 years of age and older.
13. History of intravenous drug abuse.
14. History of allergic disease or reactions likely to be exacerbated by any component of the vaccine.
15. Known allergy to egg or aminoglycoside (gentamicin).
16. History of anaphylaxis or severe allergic reaction.
17. Having received any vaccinations or planned vaccinations with a live vaccine within 30 days prior or after study vaccination.
18. Having received any vaccinations or planned vaccinations with a killed vaccine within14 days prior or after study vaccination.
19. Chronic administration (defined as more than 14 days) of systemic immuno-suppressant drugs during a period starting from six months prior to administration of the vaccine and ending at study conclusion. (Corticosteroid nasal sprays are permissible. Persons who have used topical and inhaled steroids can be enrolled after their therapy is completed).
20. Post organ transplant subjects whether or not receiving chronic immunosuppressive therapy.
21. Administration or planned administration of immunoglobulins and/or any blood products during a period starting from 3 months prior to administration of the vaccine and ending at study conclusion.
22. Use of any investigational or non-registered drug or vaccine other than the study vaccine within 30 days preceding the first dose of the study vaccine, or planned administration of such a drug during the study period.
23. Study personnel.

5. Study halting/termination and withdrawal of subjects

5.1. Study halting and termination rule

A temporary halting or termination for single study subjects or for the study as a whole can be decided in case of an occurrence of

- a serious adverse event (SAE)
- an unexpected grade 3 or higher systemic reaction or lab toxicity

with an at least reasonable possibility of a causal relationship to the administration of MVA-BN®, i.e. the relationship cannot be ruled out.

These parameters are not all-inclusive. Other adverse events could occur that would trigger a Data Safety Monitoring Board (DSMB) review. Any member of the DSMB, the Principal Investigator, BN Safety Officer, or DMID Medical Officer could request a DSMB review based on any observation.

5.2. Reporting of events fulfilling the study halting criteria

If an event fulfilling the study halting criteria reaches the investigator's attention, the investigator has the liability to alert Bavarian Nordic's Safety Department immediately (within 24 hours) and provide a comprehensive documentation of the event.

5.3. Data Safety Monitoring Board (DSMB)

The DSMB is an independent board that oversees the safety of volunteers participating in the study. The members of the DSMB are selected by Bavarian Nordic and DMID in accordance to DMID guidelines. The primary responsibilities of the DSMB are to periodically review and evaluate the accumulated study data for participant safety, study conduct and progress, and make recommendations to Bavarian Nordic, DMID and the Principal Investigator concerning the continuation, modification, or termination of the trial program. The DSMB considers study specific data as well as relevant background knowledge about the disease, test agent, and patient population under study. A separate charter describes in detail relevant operational procedures, communication pathways, roles and responsibilities of the DSMB.

In case an event occurs which fulfills the study halting criteria the Data Safety Monitoring Board will review the event in a timely manner and give a recommendation to Bavarian Nordic, DMID and the Principal Investigator to halt, resume or terminate the study participation of the affected subject and/or the study as a whole.

5.4. Individual withdrawal criteria during the study

Subjects may withdraw or be removed from the study for any of the reasons cited below:

- An adverse event occurs that, in the opinion of the investigator, makes it unsafe for the subject to continue the study. In this case, the appropriate measures will be taken.
- Subject's request to withdraw.
- Subject unwilling or unable to comply with study requirements.
- Clinical need for concomitant or ancillary therapy not permitted in the study.

- Unrelated intercurrent illness that, in the judgment of the investigator, will affect assessment of clinical status to a significant degree.

The following criteria should be checked at any visit after enrollment into the study. If any become applicable during the study, the subject may be withdrawn:

1. Use of any investigational or non-registered drug or vaccine other than the study vaccine(s).
2. Administration of a licensed vaccine not foreseen by the study protocol during the study period.
3. Start of chronic administration (defined as more than 14 days) of systemic immuno-suppressant drugs during the study period (Corticosteroid nasal sprays are permissible. Persons who have used topical and inhaled steroids can be enrolled after their therapy is completed).
4. Administration of immunoglobulins and/or any blood products during the study period.

5.5. Subject withdrawal procedure

If a subject discontinues prior to completion of the study, the date and reason for the discontinuation will be obtained. The date of the last dose of study medication will also be obtained.

Once a subject receives MVA-BN® he/she must be followed for safety as stated in the protocol. From the time of discontinuation, all diagnostic procedures and evaluations scheduled for Visit 5 for Group 1, 2 and 3 and Visit 3 for Group 4 should be performed (see III. FLOW CHART).

As a general rule, subjects who discontinued the trial after having received at least one vaccination will not be replaced.

Subjects included in the study but already discontinued the trial prior to the first vaccination should be replaced.

5.6. Contraindications and precautions for further study vaccinations

Absolute contra-indications:
The following events constitute absolute contra-indications to administration of the study vaccine; if any of these occur or become known during the study, the subject must not receive (additional) doses of the vaccine, but may continue other study procedures at the discretion of the investigator:

- Anaphylactic reaction following the administration of vaccine(s).

- Pregnancy up to and including 4 weeks (minimum 28 days) after receiving the last vaccine dose.

Subjects should be instructed to notify the investigator if it is determined after completion of the study that they became pregnant during the study or within 4 weeks (minimum 28 days) after receiving the last vaccine dose. Pregnancy must be reported to Bavarian Nordic on a Pregnancy Form within 24 hours of the investigator's becoming aware of the event.

A pregnancy should be followed to term, any premature terminations reported, and the health status of the mother and child including date of delivery and the child's gender and weight should be reported to Bavarian Nordic after delivery.

Temporary deferral of vaccination:
If the following symptom is present at the time scheduled for vaccination, the subject may be vaccinated at a later date or withdrawn at the discretion of the investigator:

- Acute disease at the time of vaccination. (Acute disease is defined as the presence of a moderate or severe illness with or without fever. The vaccine can be administered to persons with a minor illness such as diarrhea, mild upper respiratory infection with or without low-grade febrile illness, i.e., oral temperature ≤ 100.4°F (≤ 38.0°C).

6. Study treatment

6.1. Investigational product

MVA-BN® (IMVAMUNE™) is a highly attenuated live vaccinia virus. The dose is 1 x 10^8 $TCID_{50}$ MVA-BN® per 0.5 ml. The liquid frozen MVA-BN® is provided as 0.6 ml in single dose 2 ml glass vials wherein the extractable volume is equivalent to 0.5 ml.

For details see current version of the Investigator's Brochure.

6.2. Placebo

For this study 0.5 ml Tris Buffer will be used as placebo.

The Tris Buffer contains 1.21 g/l Tris, 8.526 g/l sodium chloride, 19.2 g/l Dextran FP-40, 45.2 g/l Sucrose and 0.108 g/l L-Glutamic acid monopotassium salt, pH 7.5.

6.3. Packaging and labeling

MVA-BN® and placebo are manufactured and labeled by IDT- Impfstoffwerke Dessau-Tornau GmbH.

Address:

IDT - Impfstoffwerke Dessau-Tornau GmbH
Dr. Margrit Gehrt
Streetzer Weg 15a
06862 Rodleben
Germany
Phone: +49-34901-885 0

The packages and vials will be labeled according to the national law.

6.4. Vaccine storage, handling and dispensing

The liquid frozen MVA-BN® vaccine has to be stored at -20 ± 5°C. Do not re-freeze a vial once it has been thawed.

For administration, the vaccine vial will be thawed at room temperature. To ensure homogeneity, upon thawing the vial will be swirled gently (not shaken!) for at least 30 seconds. After thawing, the drug product should appear as a pale milky colored suspension. The liquid vaccine should be visually inspected for any foreign particulate matter prior to administration. In case foreign particulate matter is visible, the vaccine must not be used anymore.
The injection volume of 0.5 ml per dose will be withdrawn with a syringe using an injection needle long enough to reach the bottom of the vial. After withdrawal of the vaccine, the injection needle should be changed and the vaccine administered to the subject immediately.

If the vaccine cannot be administered immediately, it is recommended to administer the product within 12 hours after thawing. During this time the thawed vaccine has to be stored at +2 to +8°C in the dark.

Details on vaccine handling can be found in SOP/CLIN/016: “Storage, Handling and Preparation of Liquid Frozen MVA-BN for Vaccination” (Doc No. 10000695).

6.5. Dose, vaccination schedule and route of administration

Depending on the group subjects are allocated to, subjects will receive MVA-BN® vaccine and/or placebo as follows in the upper arm (deltoid region):

Group 1:
Two s.c. vaccinations with 0.5 ml MVA-BN® vaccine containing 1 x 10^8 $TCID_{50}$, four weeks apart (Visit 1/Day 0 and Visit 3/Visit 1 + 28-35 days)

Group 2:
First s.c. vaccination with 0.5 ml MVA-BN® vaccine containing 1 x 10^8 $TCID_{50}$ and second s.c. vaccination with placebo (0.5 ml Tris Buffer), four weeks apart (Visit 1/Day 0 and Visit 3/Visit 1 + 28-35 days)

Group 3:
Two s.c. vaccinations with placebo (0.5 ml Tris Buffer), four weeks apart (Visit 1/Day 0 and Visit 3/Visit 1 + 28-35 days)

Group 4:
One s.c. vaccination with 0.5 ml MVA-BN® vaccine containing 1 x 10^8 $TCID_{50}$ (Visit 1/Day 0)

6.6. Accountability and disposal

Used and unused vials should be stored in a safe place and remain the property of Bavarian Nordic. The Principal Investigator of the respective site or his designee is responsible for ensuring adequate accountability of all used and unused study drug. This includes acknowledgement of receipt of each shipment of study drug (quantity and condition) and drug inventory log. The drug inventory log will document quantity of study drug received from Bavarian Nordic, quantity of study drug used for vaccination (including lot number, date dispensed, subject identification number and initials of the person dispensing the study medication) and quantity of study drug returned to Bavarian Nordic or destroyed.

In case destruction is agreed, material should be discarded at site according to local regulations.

6.7. Concomitant medication

All concomitant medication must be recorded in the CRF with the reason for administration, the dosage regimen, and the onset and end of treatment.

7. Clinical and laboratory assessments

7.1. Assessment of safety and reactogenicity

Taking into account the medical history of the subject, safety will be monitored by performing physical examinations including vital signs, routine laboratory measurements and ECGs as well as by evaluating local and general solicited adverse events and unsolicited adverse events.

Details regarding definitions and reporting of adverse events are described in section 8 of this protocol.

7.1.1. Medical history

The baseline assessment for safety parameters will be performed during screening visit. Medical history will be documented during the screening period (visit SCR). History will focus particularly on any important diseases and in case of infections or tumors, the pathogen involved or the pathological diagnosis, respectively, if available. A special attention should be given to history of prior allergic reactions, especially to vaccines.

For subjects in group 4, the presence of a typical vaccinia scar and/or data about smallpox vaccination (if any vaccination certificate is available) should be documented in the CRF.

7.1.2. Physical examination and vital signs

A physical examination and a check of vital signs will be performed during the screening period (visit SCR) for baseline assessment and on every following visit (Visits 1 – 5 and F-U).

PHYSICAL EXAMINATION:

The exam includes a review of major organ systems and weight. The examination should be directed at finding evidence of any infections, tumors and lymphadenopathy.

In addition, listening to the heart and lungs specifically for heart failure, presence of rubs, gallops, murmurs, crackles, and rales will be performed.

VITAL SIGNS:

Blood pressure and pulse rate will be taken after the subject was seated for two minutes, and oral temperature will be taken.

7.1.3. Laboratory measurements

SAFETY LABORATORY:

Safety laboratory is determined at the screening visit (Visit SCR) and 10 – 15 days after each vaccination (groups 1, 2 and 3: Visits 2 and 4 / Group 4: Visit 2). At Visit 5 (only Groups 1, 2 and 3) and at follow-up visit (all groups: Visit F-U) safety laboratory is only done if clinically indicated. The safety laboratory measurements are performed at a central laboratory. Laboratory normal ranges are provided by the central laboratory and filed in the investigator file. Safety laboratory parameters to be evaluated are:

Hematology:
Red blood cell count, hemoglobin, total and differential WBC, platelet count.

Serum chemistry:
Total bilirubin, alkaline phosphatase, AST, ALT, serum creatinine, sodium, potassium, calcium, troponin I.

The intensity of laboratory / systemic quantitatively measured toxicities will be graded according to the toxicity scale in Appendix I. These grading scales include the laboratory values determined with the routine safety parameters. In case of other laboratory values not included in the routine safety laboratory and not listed in Appendix I, the NCI Common Toxicity Criteria table, Version 2.0, published April 30, 1999 will be used for grading of laboratory toxicities.

PREGNANCY TEST:

β-HCG pregnancy test will be conducted for all women with reproductive potential at screening (Visit SCR), within 24 hours prior to each vaccination (Groups 1, 2 and 3: Visits 1 and 3 / Group 4: Visit 1) and 28-35 days after the last vaccination (Group 1-3: Visit 5 / Group 4: Visit 3). At screening a serum β-HCG pregnancy test will be conducted and all other pregnancy test will be conducted as urine β-HCG test.

The following parameters will only be evaluated during the screening period (Visit SCR) for check of inclusion / exclusion criteria:

CHOLESTEROL:

Total, HDL and LDL.

URINE ANALYSIS (E.G. WITH COMBUR-10 STICK):

Protein, glucose, occult blood, nitrites, leucocytes, pH, bilirubin, ketones, urobilinogen, specific gravity.

7.1.4. ECG assessments

A standard 12-lead ECG will be taken on all subjects at screening (Visit SCR) and 10-15 days after each vaccination (Groups 1, 2 and 3: Visits 2 and 4 / Group 4: Visit 2). At Visit 5 (only Groups 1, 2 and 3) and at follow-up visit (all groups: Visit F-U) an ECG is only done if clinically indicated.

ECGs will be evaluated by a centralized procedure. ECGs will be assessed by the investigator of the study site and transmitted to a central database. Abnormal and unclear

ECG findings at screening or during the study conduct will additionally be evaluated and followed-up by a Cardiologist. Furthermore, in case of cardiac symptoms the following workflow / communication flow will be applied:

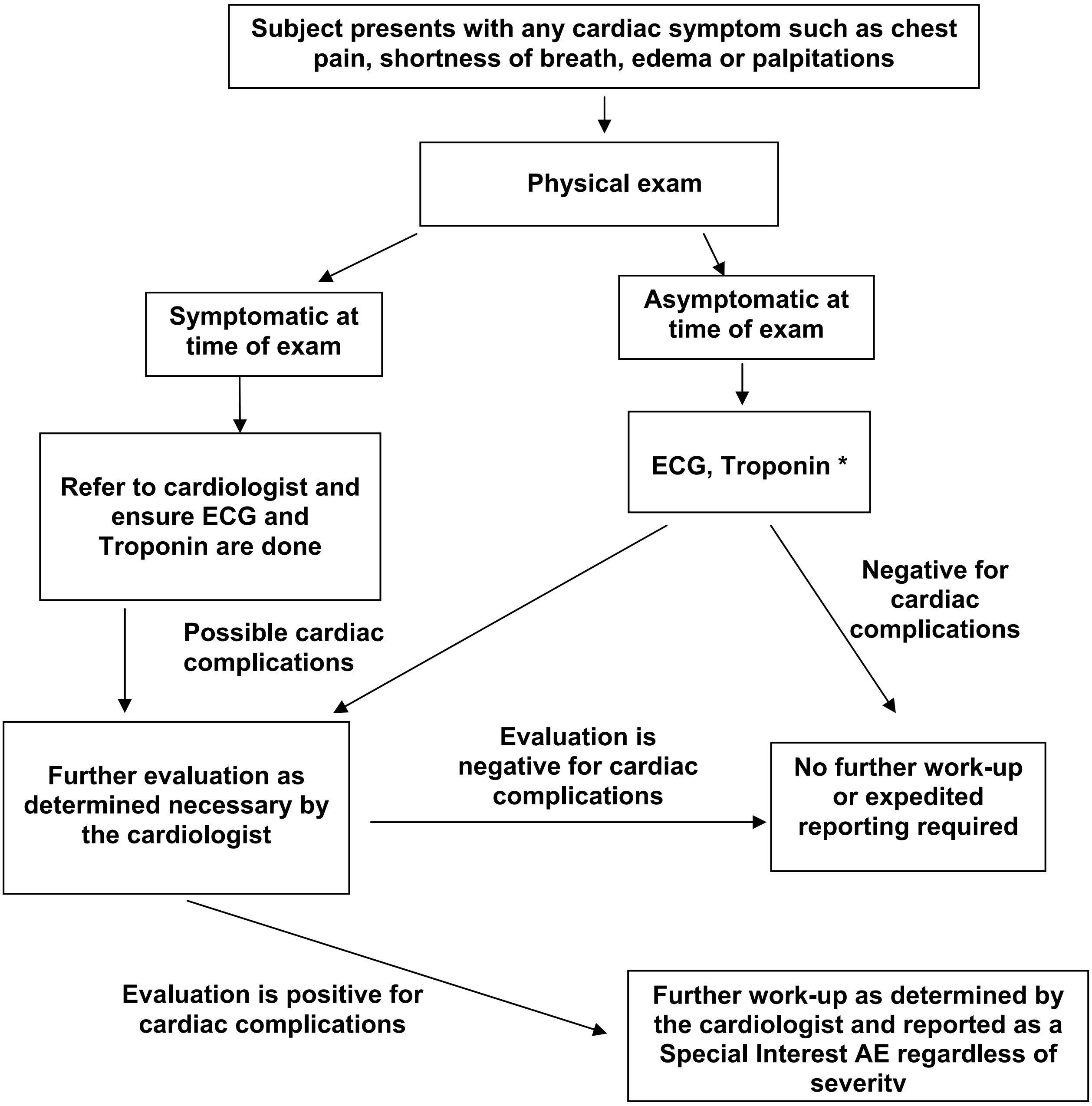

***At any protocol-scheduled ECG and/or Troponin abnormality after vaccination the algorithm will begin at this point.**

If clinically indicated, e.g. in case of any kind of cardiac symptoms such as but not limited to chest pain, dyspnea, arrhythmia, or edema as well as abnormal ECG findings during the treatment phase, subjects will be referred for a cardiac evaluation by a Cardiologist and further diagnostic tests will be done as recommended by this Cardiologist (e.g. (exercise) ECG, cardiac enzymes, echocardiogram). Subjects will be followed up for at least one year if clinically required and in a frequency determined by the Cardiologist.

Such adverse events fulfill the definition of an “adverse event of special interest” and are to

be handled respectively reported as described under section 8.4.

Using replication-competent vaccinia-based smallpox vaccines during smallpox vaccination programs in the US during the last years, cases of acute myocarditis and pericarditis were observed (Grabenstein and Winkenwerder, 2003).

Case definitions as published by the US Center of Disease Control (CDC) ("Update: Cardiac-Related Events During the Civilian Smallpox Vaccination Program --- United States, MMWR May 30, 2003, Vol. 52, No. 21, p. 494") are provided in Appendix II to recognize possible cases of acute myocarditis and pericarditis and to distinguish unspecific and isolated ECG changes without or with unclear clinical meaning and ECG changes related to possible or probable cases of acute myocarditis and/or pericarditis.

7.1.5. Assessment of solicited adverse events (Diary)

After each vaccination each subject receives a diary to record solicited local and general adverse events most likely to occur on the day of vaccination or the following 7 days. All solicited adverse events observed after vaccination with details concerning the intensity and the course of the event should be documented there. The investigator will collect this information during the next scheduled visit and will assess the relationship of the solicited general events to study medication.

In case of severe and unexpected local and systemic reactions, the study physician should be contacted outside from scheduled study visits.

To standardize procedures uniform rulers have been handed out to all subjects for measurements of erythema, swelling and induration diameters and digital thermometers for oral measurements of body temperature.

Solicited Local Adverse Events

The solicited local symptoms erythema, swelling, induration and pain at the injection site have to be documented in the diary by the subject and intensity is assessed in the following way:

Injection site erythema: size measured in diameter
Injection site swelling: size measured in diameter
Injection site induration: size measured in diameter

The maximum intensity will be scored as follows:

0 = 0
1 = < 30 mm
2 = ≥ 30 – <100 mm
3 = ≥ 100 mm

Injection site pain:

0 = Absent
1 = Painful on touch
2 = Painful when limb is moved
3 = Spontaneously painful / prevents normal activity

SOLICITED GENERAL ADVERSE EVENTS

Subjects are asked to document the solicited general adverse events as described in the table below on the day of vaccination and the following 7 days on their diary card.

In the subject's diary the grading of maximum symptom intensity is described in basic, easily understood language based on the following descriptions.

MedDRA coded Preferred Term General Adverse Events	Grade	Maximum Intensity
Body temperature*	0	< 99.5°F (< 37.5°C)
	1	≥ 99.5 - <100.4°F (≥ 37.5° – < 38.0°C)
	2	≥ 100.4 – 102.2°F (≥ 38.0 – < 39.0°C)
	3	≥ 102.2 - 104°F (≥ 39.0 - < 40.0°C)
	4	≥ 104°F (40.0°C)
Headache, Myalgia, Nausea and Fatigue	0	None
	1	Mild: easily tolerated, minimal discomfort and no interference with daily activity
	2	Moderate: Some interference with daily activity
	3	Severe: Prevents daily activity
	4	Life-threatening or disabling

***Pyrexia** is defined as oral temperature ≥ 100.4°F (≥38.0°C).

Causal relationship between solicited general adverse events and the study vaccine will be assessed by the investigator.

7.1.6. Assessment of unsolicited adverse events

During every study visit following screening, the investigator has to report any unsolicited adverse event experienced by the subject.

Unsolicited adverse events following the vaccination will generally be recorded by the subjects in a special section of the diary card and transferred by the investigator to the adverse event section of the CRF. In addition, all intercurrent diseases not recorded on the diary card but reported when the investigator actively inquires of the subject will be documented in the respective section of the case report form.

Adverse Events will be documented and followed up until completion of the study.
Serious adverse events ongoing at the time of the F-U Visit will be followed up until resolution or achievement of stable clinical conditions.

7.1.7. Assessment of intensity for adverse events

The scale for grading the maximum intensity of all adverse events will be based on the following descriptions:

1 = An adverse event which is easily tolerated by the subject, causing minimal discomfort and not interfering with daily activities.
2 = An adverse event which is sufficiently discomforting to interfere with daily activities.
3 = An adverse event which prevents daily activities. (Such an adverse event would, for example, prevent attendance at work and would necessitate the administration of corrective therapy.)

4 = Life threatening or disabling

7.1.8. Assessment of causality for adverse events

The relationship between the occurrence of an adverse event and the study drug will be assessed using the following categories:

**None**	The time interval between the administration of the study drug and the occurrence or worsening of the AE rules out a relationship
	and/or
	another cause is established and there is no evidence of a (concomitant) causal connection with or worsening caused by the study medication.
**Unlikely**	The time interval between administration of the study drug and the occurrence or worsening of the AE makes a causal relationship unlikely
	and/or
	the known effects of the study medication or substance class provide no indication of a (concomitant) causal connection with or worsening caused by the study medication and there is another cause which serves as an adequate explanation
	and/or
	although the known effects of the study medication or substance class make it possible to derive a plausible causal chain with regard to a (concomitant) causal connection or worsening, however, another cause is considerably more likely
	and/or
	another cause of the AE has been identified and a (concomitant) causal connection with or worsening caused by the study medication is unlikely.
**Possible**	A plausible causal chain with regard to a (concomitant) causal connection with / worsening of the AE can be derived from the pharmacological properties of the study medication or substance class. However, other approximately equally likely causes are known
	or
	although the pharmacological properties of the study medication or substance class provide no indication of a (concomitant) causal connection with / worsening of the AE, there is no other known cause which provides an adequate explanation.
**Probable**	The pharmacological properties of the study medication or substance class
	and/or
	the course of the AE after discontinuation of the study drug and possible subsequent re-exposure
	and/or
	specific findings (e.g. positive allergy test or antibodies against the trial drug / metabolites) suggest a (concomitant) causal connection with / worsening of the AE resulting from the study medication, however another cause cannot completely be ruled out.
**Definite**	The pharmacological properties of the study medication or substance class
	and/or
	the course of the AE after discontinuation of the study drug and possible subsequent re-exposure
	and/or
	specific findings (e.g. positive allergy test or antibodies against the trial drug / metabolites) definitely indicate that there is a (concomitant) causal connection with / worsening of the AE resulting from the study medication and there are no indications of other causes.

7.2. Assessment of immunogenicity: Antibody Response

Immune response analysis is planned at any study visit except the screening visit. The baseline assessment for immunogenicity parameters will be performed during Visit 1 (before vaccination).

The method of collection, storage and handling of laboratory specimen for the immune analysis is specified in SOP/CA/020: "Collection preparation and storage instructions for immune response serum specimens" (Doc. No. 10000213). A written instruction for this procedure will be provided to the investigators before enrollment. Additionally, the procedure will be introduced during the investigator meeting and/or at the initiation visit.

Antibody responses against MVA-BN® will be measured using a direct ELISA and a plaque reduction neutralization test (PRNT) using established and validated in house assays.

Immune analyses will be performed at Bavarian Nordic's laboratory at Bavarian Nordic GmbH, 82152 Martinsried, Germany. The protocols for the analytical tests performed are detailed in the following SOPs and will be provided in the Trial Master File:

SOP/CA/017: "Vaccinia Virus (VV) Specific Plaque Reduction Assay for the Detection of Neutralizing Antibodies in Human Sera" (Doc. No. 10000210).
SOP/CA/029: "(Automated) Standard ELISA for detection of MVA specific antibodies in human sera" (Doc. No. 10000262).

The names and titles of the mentioned SOPs can be subject to changes or updates during the trial. A changed or updated SOP will always retain the same document number and will immediately be provided to the investigator upon availability.

ELISA
Seroconversion is defined as:
Appearance of antibody titers ≥ 1:50 in a vaccinia specific IgG ELISA for initially seronegative subjects or twofold increase of the antibody titer compared to the pre-existing baseline titer for subjects with a pre-existing antibody titer in the ELISA.

The geometric mean titer (GMT) is calculated by taking the antilogarithm of the mean of the log10 titer transformations. Antibody titers below the cut-off of the assay will be given an arbitrary value of one for the purpose of calculation.

PRNT
Seroconversion is defined as:
Appearance of antibody titers ≥ 1:20 in a vaccinia specific plaque reduction neutralization assay for initially seronegative subjects or twofold increase of the antibody titer compared to the pre-existing baseline titer for subjects with a pre-existing antibody titer in the PRNT.

The GMT is calculated by taking the antilogarithm of the mean of the log10 titer transformations. Antibody titers below the cut-off of the assay will be given an arbitrary value of one for the purpose of calculation.

8. Adverse event definitions and reporting

Any signs and symptoms that occur before first vaccination will be recorded in the baseline signs and symptoms section and will not be considered as adverse events.

8.1. Definition of adverse event

Adverse events are defined as any untoward (undesirable) medical occurrence in a clinical trial subject administered a medicinal product and which does not necessarily have a causal relationship with this medication. All adverse events (e.g. feeling of ill-health, subjective symptoms and objective signs, intercurrent diseases, accidents, etc.) observed by the

investigator and/or reported by the subject must be recorded in the CRF regardless of the assessment of causality in relationship with the study drug.

Abnormal laboratory values that were assessed as clinically significant by the investigator are to be documented as AEs. In addition, abnormal laboratory values fulfilling the Grade 3 criterion according to the toxicity scale (Appendix I) are to be documented as AEs, regardless of whether they are considered clinically relevant or not.

8.2. Definition of solicited adverse events

Within this study protocol solicited adverse events are defined as all events recorded by the subjects in the diary provided to them following every vaccination. For each of the 7 days post-vaccination the subjects are requested to monitor and record local symptoms, i.e. erythema, swelling, induration and pain at the site of injection as well as general symptoms, i.e. body temperature, headache, myalgia, nausea and fatigue.

8.3. Definition of unsolicited adverse events

At every study visit the investigator should ask the subject if they have experienced any adverse events since their last visit. All intercurrent diseases reported by the subject, regardless of whether recorded in the subject diary or not, need to be recorded by the investigator in the appropriate page of the case report form.

The intensity and causality of the events will be graded according to the procedures described under 7.1.7 and 7.1.8 respectively.

8.4. Definition of “adverse event of special interest”

An “adverse event of special interest” is defined in this study as an adverse event fulfilling any of the following features:

- Any cardiac symptom
- ECG changes, which are determined to be clinically significant
- Cardiac enzymes elevated above ULN

Adverse events of special interest are to be reported according to the procedures and timelines applicable for serious adverse events.

8.5. Definition of serious adverse events (SAEs)

A serious adverse event (experience) or reaction is any untoward medical occurrence or effect that at any dose:

1. Results in death,
2. Is life-threatening*,
3. Requires inpatient hospitalization or prolongation of existing hospitalization,
4. Results in persistent or significant disability or incapacity,
5. Is a congenital anomaly or birth defect,
6. or is an otherwise important medical event.

**The term "life-threatening" in the definition of "serious" refers to an event in which the patient was at risk of death at the time of the event; it does not refer to an event which hypothetically might have caused death if it were more severe.*

Medical and scientific judgment should be exercised in deciding whether expedited reporting is appropriate in other situations, such as important medical events that may not be immediately life-threatening or result in death or hospitalization but may jeopardize the patient or may require intervention to prevent one of the other outcomes listed in the definition above. These should also usually be considered serious.

Examples of such events are intensive treatment in an emergency room or at home for allergic bronchospasm; blood dyscrasias or convulsions that do not result in hospitalization; or development of drug dependency or drug abuse.

8.6. Reporting of SAEs and adverse events of special interest

All serious adverse events (SAEs) or adverse events of special interest (see definition 8.4) occurring throughout the entire course of the study have to be reported to Bavarian Nordic´s Drug Safety Department. The study site has to send by e-mail or fax the completed serious adverse event form to Bavarian Nordic's Drug Safety Department within 24 hours of becoming aware of the adverse event.

In compliance with all regulations Bavarian Nordic will take care of expedited reporting of adverse events to the regulatority authorities, to the ethics committees and to the responsible NIH-DMID representative.

SAEs: BN will send copies of all SAEs and any supporting documents electronically to DMID within 3 business days of receipt by BN.
Safety Reports: BN will provide DMID with copies of all IND safety report submissions within 3 business days of the submission to the FDA.
Site Monitoring Reports: BN will provide DMID with a copy of all site monitoring reports on a monthly basis.

Please mail SAE reports to the following address: drug.safety@bavarian-nordic.com

***OR* fax the completed SAE report to the number: +49-89-8565 1419**

In case any problems occur during transmittal of a SAE report or with all questions specifically related to the safety of a subject enrolled in the study please contact one of the below listed responsible persons:

Garth Virgin, MD<br>Drug Safety Officer, Bavarian Nordic<br>Phone: +49-89-8565 1319<br>(working hours)<br>Phone: +49-172-831 6604<br>(after hours)	or (Backup)	Nathaly Arndtz, MD<br>Vice President Medical Affairs<br>Phone: +49-89-8565 1308<br>(working hours)<br>Phone: +49-172 834-7130<br>(after hours)

The investigator should not delay reporting because of missing information. Nonetheless, the report should be as complete as possible. This initial notification should include, as a

minimum, sufficient information to permit identification of the following:

- the reporter
- adverse event(s)
- involved study medication
- the subject
- date of onset

9. Statistical Considerations

The primary objective of this trial is to compare the ELISA specific humoral immune response of the group with history of smallpox vaccination versus the group without history of smallpox vaccination, i.e. to prove that a single dose schedule would be sufficient to boost the immunity in a previously vaccinated population.

9.1. Sample size calculation

The sample size calculation is based on the **primary immunogenicity variable 'MVA - specific seroconversion rate'**. This parameter is derived from the ELISA specific antibody titers. Seroconversion is defined as the appearance of antibody titers ≥ 1:50 in a vaccinia specific IgG ELISA for initially seronegative subjects or twofold increase of the antibody titer compared to the pre-existing baseline titer for subjects with a pre-existing antibody titer in the ELISA.

The primary hypothesis is to show that the humoral immune response of the group with history of smallpox vaccination (Group 4) is not statistically inferior compared to the group without history of smallpox vaccination (Group 1). The study should demonstrate that the Group 4 seroconversion rate is not worse than the Group 1 seroconversion rate by more than a pre-specified amount. This amount is called the non-inferiority margin (Δ).

Suppose p_1 is the seroconversion rate in the group without history of smallpox vaccination and p_4 is the seroconversion rate in the subjects with history of smallpox vaccination.
The test on non-inferiority will be applied for the following hypothesis:

H_0: $p_4 - p_1 \leq -\Delta$ versus H_1: $p_4 - p_1 > -\Delta$, where

Δ is the non-inferiority margin and is chosen in this trial as 5%.
From the experience in a pilot trial with MVA-BN® in healthy subjects it is anticipated that the seroconversion rate in healthy vaccinia-naive subjects reaches 98-100%.

Assuming a significance level of 5%, a power of 80%, expected seroconversion rates of 98% in both groups, this yields to a sample size of 175 per group (700 in total). In order to account for drop-outs, a total of at least 180 subjects per group will be treated.

For reference see Agresti A, Min Y. (2001) and Chan I (1998).

9.2. Variables

9.2.1. Immunogenicity variables

Primary variable

MVA -specific seroconversion rate derived from the ELISA specific antibody titers 2 weeks after the last vaccination (Group 1-3: Visit 4, Group 4: Visit 2).

Seroconversion is defined as the appearance of antibody titers ≥ 1:50 in a vaccinia specific IgG ELISA for initially seronegative subjects or twofold increase of the antibody titer compared to the pre-existing baseline titer for subjects with a pre-existing antibody titer in the ELISA.

Secondary variables:

MVA -specific seroconversion rate derived from the ELISA specific antibody titers 4 weeks after the last vaccination (Group 1-3: Visit 5, Group 4: Visit 3).

MVA -specific seroconversion rate derived from the PRNT specific antibody titers 2 weeks after the last vaccination (Group 1-3: Visit 4, Group 4: Visit 2) and 4 weeks after the last vaccination (Group 1-3: Visit 5, Group 4: Visit 3).

Seroconversion is defined as appearance of antibody titers ≥ 1:20 in a vaccinia specific plaque reduction neutralization assay for initially seronegative subjects or twofold increase of the antibody titer compared to the pre-existing baseline titer for subjects with a pre-existing antibody titer in the PRNT.

9.2.2. Safety and reactogenicity variables

Primary variables:

Occurrence, relationship and intensity of any specific or unspecific ECG change at any time during the study.

Occurrence, relationship and intensity of any other cardiac symptom at any time during the study.

Secondary variables:

Occurrence of any serious adverse event probably, possibly or definitely related to the study vaccine at any time during the study.

Occurrence of any grade 3 or higher adverse reaction probably, possibly or definitely related to the study vaccine within 28 days after vaccination.

Occurrence of solicited local adverse events within 1 week (Days 0-7) after vaccination: Intensity, duration, and relationship to vaccination.

Occurrence of solicited general adverse events within 1 week (Days 0-7) after vaccination: Intensity, duration, and relationship to vaccination.

Occurrence of unsolicited non-serious adverse events within 28 days after vaccination: Intensity, duration and relationship to vaccination.

9.3. Study cohorts/data sets to be evaluated

For the statistical analysis the included subjects will be divided up into the following datasets:

Safety population: This is the subset of subjects who are randomized, have received at least one dose of study vaccine and for whom safety data are available.

The main analysis of safety will be performed on this population.

Full-analysis (FA): This is the subset of subjects who are randomized and had received at least one dose of study vaccine. Relevant protocol violators are included into this dataset. Subjects who drop out before Visit 5 (for Group 1-3) or Visit 3 (for Group 4) are included here.

Per Protocol (PP): This is the subset of subjects who are randomized and had received two doses of study vaccine (group 1-3), respectively one dose of study vaccine (group 4). Subjects have to complete Visit 5 (for Group 1-3), respectively Visit 3 (for Group 4). Subjects have to adhere to all protocol conditions, however mild (not relevant) protocol violators can be included into this dataset.

The decision whether a protocol deviation is relevant or not for the classification of subjects to subsets should be made case-by case in a blind review meeting.

The primary population dataset will be the FA Set. All confirmatory testing is based on this subgroup. For further descriptive purposes, the same statistical procedures will be applied to the PP dataset.

Analysis of immunogenicity variables will be done on a valid case basis, i.e. for missing observations no imputation technique like LOCF will be applied, because this would imply a too optimistic approach.

9.4. Statistical analysis

As soon as the last subject has completed Visit 5 (for Group 1-3), respectively Visit 3 (for Group 4) and after any necessary settlement of queries etc. in the CRFs, data from those subjects and visits will be locked. A full analysis of the data available will then be performed. Once all subjects have completed the follow-up (Visit F-U), an addendum to the clinical report will be prepared including all follow-up data.

9.4.1. Analysis of demographics and baseline characteristics

An analysis of variance (ANOVA) for the main baseline characteristics (age, weight, height) will be performed. The ANOVA includes treatment and location effects and will be performed to detect any global treatment or location effect. The model does not include a term for

location by treatment interaction (according to ICH guideline E9, Statistical Principles for Clinical Trials, p.16).

9.4.2. Analysis of Immunogenicity

Antibody titers and resulting seroconversion rates will be assessed by direct ELISA and PRNT method as described in section 7.2.
The primary immunogenicity variable is the MVA -specific seroconversion rate. This parameter is derived from the ELISA specific antibody titers.

The primary hypothesis is to show that the humoral immune response of the group with history of smallpox vaccination (Group 4) is not statistically inferior compared to the group without history of smallpox vaccination (Group 1).

The above hypothesis will be tested based on an exact, unconditional test for binomial differences. In addition an exact one-sided 97.5% unconditional confidence interval for the difference of proportions will be calculated. If the lower limit of this confidence interval is greater than 5% (or equivalent the p-value of the non-inferiority test is less than 5%) then the null hypothesis will be rejected (StatXact®, Chan (1998), Agresti and Min (2001)).

In order to limit the overall type-I-error to a nominal level of 5%, a **hierarchical test procedure** will be chosen:

In a first step, the primary null hypothesis as stated in section 9.1 will be tested 2 weeks after the last vaccination (Group 1-3: Visit 4, Group 4: Visit 2). Only if this comparison shows a significant result, the comparison at 4 weeks after vaccination (Group 1-3: Visit 5, Group 4: Visit 3) will be done.

A secondary analysis will be done with the neutralization assay specific seroconversion rates (from PRNT) as above.

In addition to the main comparison of Group 4 versus Group 1, additional, secondary comparisons will be made among all other groups.

All statistical tests for secondary time points and comparisons are regarded descriptive. No adjustment for multiple testing will therefore be done.

Tables and graphs showing the seroconversion rates for each group at visit will be prepared.
Descriptive statistics and graphical displays will be given for the geometric mean titer for all groups at all visits.

9.4.3. Analysis of safety and reactogenicity

<u>Solicited local adverse events</u>

The occurrence of solicited local adverse events within 1 week after each vaccination will be summarized on a per subject and per vaccination basis.
The maximum intensity over the 7-day period after vaccination will be used and categorized as follows:

Pain:

Grade = 0 / > 0
Grade < 2 / ≥ 2
Grade < 3 / = 3

For measurements of diameter size:

Diameter = 0 / > 0
Diameter < 30mm / ≥ 30 mm
Diameter < 100mm/ ≥ 100 mm

These categories will be compared between treatment groups by means of the exact test of Fisher (or its normal approximation, the chi-squared test).

Solicited general adverse events

Occurrence of solicited general adverse events within 1 week after each vaccination will be summarized per subject and vaccination.

The maximum intensity over the 7-day period after vaccination will be used and categorized as follows:

Grade = 0 / > 0
Grade < 2 / ≥ 2
Grade < 3 / ≥ 3

These categories will be compared between treatment groups by means of the exact test of Fisher (or its normal approximation, the chi-squared test).

Unsolicited adverse events:

Unsolicited adverse events will be coded with the MedDRA coding terminology. The intensity of adverse events will be graded according to section 7.1.7.
The number of adverse events and number of Subjects with at least one adverse event for each preferred term will be descriptively compared between treatment groups.

The occurrence of any grade 3 or higher adverse reaction probably, possibly or definitely related to the study vaccine within 28 days after vaccination will be compared between treatment groups.

Adverse events of special interest will be separately listed and tabulated. The incidence of such AEs will be compared between treatment groups by means of the exact test of Fisher (or its normal approximation, the chi-squared test).

Serious adverse events will be listed separately. Each SAE will be described individually in detail. The number of subjects with at least one SAE will be compared between treatment groups by means of the exact test of Fisher (or its normal approximation, the chi-squared test).

The occurrence, relationship and intensity of any *other cardiac symptom* at any time during the study will be listed. The number of subjects with at least one cardiac symptom will be compared between treatment groups by means of the exact test of Fisher (or its normal approximation, the chi-squared test).

Safety laboratory and urine analysis:
Clinical laboratory test results will be marked whether the result is below, within or above the respective reference range. The number of values outside of the reference range will be counted.

ECG
The ECGs will be evaluated by a centralized procedure. Overall assessment will be done by the investigators or if required by a cardiologist. The transmitted standard ECG results like PQ, QRS, QT and QTc duration and heart rate will be summarized per visit and treatment group.

The number of subjects with normal/abnormal ECGs and clinically significant ECGs (resulting in AEs) will be compared between treatment groups by means of the exact test of Fisher (or its normal approximation, the chi-squared test).
Detailed descriptive analysis of the reasons (category) of abnormality will be done.

9.4.4. Data handling

All data obtained in this study and documented in the CRF's will be listed. For parameters of interest, summary tables with descriptive group statistics (mean, standard deviation, minimum, maximum, number of valid cases) for metrical variables will be prepared. For ordinal/dichotomous variables summary tables showing the absolute and relative count in each category will be prepared.
Further statistical analysis may be performed as appropriate.

10. Ethical aspects

10.1. Ethical and legal regulations

The Principal Investigators make sure that this clinical trial is conducted in complete accordance with the provisions of the Declaration of Helsinki (and its amendments of Tokyo, Venice, Hong Kong, Somerset West and Edinburgh), the national laws and other guidelines for the conduct of clinical studies like the ICH Harmonized Tripartite Guideline for Good Clinical Practice to guarantee the greatest possible subject protection.

10.2. Approval of an ethics committee

The protocol must be reviewed by the competent ethics committee according to the national law of the respective site before the first subject is included in this study.

If one of the investigators is a member of one of these committees, he may not vote on any aspect of the review of this protocol.

The Sponsor will assure that the ethics committee is informed of any amendment to the protocol and any unanticipated problems involving risks to human subjects included in the study. Such information will be provided to the committee at intervals appropriate to the degree of subject risk involved, but not less than once a year. Copies of all correspondence between the investigator and the committee must be forwarded immediately to the sponsor. In case of withdrawal of ethics committee approval of the study, the sponsor has to be

contacted immediately by facsimile or telephone.

10.3. Confidentiality and data protection

The Principal Investigator of the respective site is obliged to ensure anonymity of the subject. He/she has to make sure that all documents including CRFs provided (e.g. in the course of a marketing authorization procedure) to third parties (in this case: to the manufacturer of MVA-BN® or to an authority) contain no subject names.
Only a subject and centre number, not by their name or clinic and subject's file number, may identify subjects respectively. The Principal Investigators keep separate confidential subject logs for study enrollment, which allows subject numbers to be matched with names and addresses of subjects at any time. Documents not meant to be passed on to third parties have to be stored confidentially by the Principal Investigator.

Any information collected in the course of the study may be made available only to persons directly involved in this study (Principal Investigator and his staff members, monitor, statistician) or to authorized persons by the sponsor or the Principal Investigator or authorities.

11. Informed consent

No subject can participate in this study without having given informed consent in writing after the investigator or his delegate has informed the subject clearly and completely, verbally and in writing, over the purpose, procedures, potential benefits and risks of the current study and prior to study drug administration.

One signed copy of the informed consent must be given to each subject and one signed copy must remain in the study documentation file and be available for verification by the monitor or competent regulatory authorities at any time.

Subjects must be informed unequivocally that they may refuse participation in the study and that they may withdraw from the study at any time and for whatever reason and that withdrawal of consent will not affect their subsequent medical treatment or relationship with the treating physician.

Subjects also consent to authorize the monitor, quality assurance personnel and regulatory authorities to inspect source documents for quality assurance purposes. Such verifications will always be conducted on site and under the ethical supervision of the investigator. All aspects of the confidentiality of the subject's data will be guaranteed.

The informed consent form must be submitted to the ethics committee. Subjects will be informed about the following:

- Purpose of the research, duration of participation, procedures to be followed, including any that are experimental
- Reasonable foreseeable risks or discomforts
- Extent, if any, to which confidentiality of record will be maintained, and notification that regulatory authorities, the sponsor and the monitor may inspect the records
- Whether any compensation or medical treatments are available if injury occurs

- Who to contact for answers to question or in event of injury
- Statement that participation is voluntary and the subject has the right to withdraw at any time without penalty or loss of benefits
- Subject insurance & insurance conditions.

12. Case report forms and retention of records

12.1. Electronic case report forms

All of the clinical data will be captured via electronic data capture (EDC) using a web-based tool. The investigator site staff will enter and edit the data via a secure network, with secure access features (username and password). A complete electronic audit trail will be maintained. The investigator will approve the data using an electronic signature (Ref: 21 CFR Part 11), and this approval is used to confirm the accuracy of the data recorded.

eCRFs will be used for all subjects. The investigator's data will be accessible from the investigator's site throughout the trial. The electronic CRFs must be kept current to reflect subject status at each phase during the course of the trial. The subjects are identified on the electronic CRF by number, gender and date of birth. The investigator must make a separate confidential record of these details (subject identification and enrollment log). While the trial is ongoing and until the access to the database has been terminated, there will be a full audit trail of changes. All changes to data are done by the investigator through the EDC system. If a change is necessary once the investigator has no further access to the database, a request for change will be sent to the investigator for confirmation of the change.

It is the responsibility of the Principal Investigator of the respective site to ensure that all subject discontinuations or changes in study or other medications entered on the subject's eCRF are also made on the subjects medical records.

The eCRFs for any subject leaving the study should be completed at the time of the final visit or shortly thereafter.

12.2. Retention of records

The Principal Investigator shall maintain the records of disposition of drug receipts and drug inventory logs, subject files and regulatory documents (informed consents, ethical approval) for 15 years after the end of the study.

To meet regulatory requirements, the eCRF data will be electronically stored at sites. The CDISC ODM (see www.cdisc.org for details) will be used to store and archive all electronic data at the sites. Since CDISC ODM is also the source for the EDC-web-based system, no transcription of data is necessary.
CDISC ODM is a plat-form independent standardized data format including the complete study metadata and audit trail. The data can be reviewed at a later stage using off-the-shelf tools. CDISC is providing a complete CDISC ODM Viewer for these purposes.
If needed, PDF-files can be created from the ODM file.

13. Monitoring of the study

The monitor is responsible for obtaining an overview of the course of the trial in co-operation with the investigator, checking if the trial protocol is being observed, and helping the investigators to solve any problems which may arise. All documents in the context with this clinical trial will be handled confidentially at any time.

Data reported in the eCRFs (electronic CRFs) that are transcribed from source documents must be consistent with the source documents or the discrepancies must be explained.

For data that is directly captured in the eCRF (eSource), the eCRF itself becomes source data and source data verification is not applicable.

The Principal Investigator of the respective site agrees to give the monitor access to relevant hospital or clinical records to confirm their consistency with the CRF entries and to obtain an adequate overview of the course of the trial. The monitor checks entries on the CRF for completeness, accuracy and correctness. The entries on the CRF will be verified against source documents. This will be done under preservation of data protection.

The items for source data verification will be specified in detail in the monitoring manual.

The source data verification must be performed by direct viewing. If a subject refuses to consent to this procedure he/she must not be enrolled in the study.
The investigator (or a representative) has further agreed to support the monitor in solving any problems he/she discovers during his/her visits.

Monitoring of drug accountability will be performed by an additional independent monitor to ensure that the monitor responsible for source data verification is kept blinded.

14. Responsibilities of the investigator

The Principal Investigator of the respective site agrees to carry out the study in accordance with the guidelines and procedures outlined in this trial protocol. The Principal Investigator especially consents to strictly adhere to the ethical principles (see section 10 of this protocol).

The Principal Investigator knows that he/she must, according to professional regulations for physicians, obtain the approval of the relevant Ethics Committee.

Any deviation from the trial protocol must, before its implementation, be agreed to by the sponsor in writing, and by the Ethics Committee initially consulted.

Changes to the protocol require written ”Amendments to the protocol” and written approval by the Principal Investigator. Changes are allowed only if study value is not reduced and if they are ethically justifiable. The statistician must agree to the amendment, if appropriate, his statement is to be submitted to the Ethics Committee. The amendment must be passed on to all participating investigators with the obligation to adhere to its provisions. If warranted, the subject information has to be changed accordingly.

It is within the responsibility of the Principal Investigator that a CRF is completed and signed after the subject has finished the trial for each subject participating in the study.

Since the electronic signature on the eCRF is a legally binding signature which is equivalent to a handwritten signature and dating on a document, it is the responsibility of the investigator not to pass any information about username and password to other persons.

At the conclusion of the study, the investigator will return all partly used, unused and empty drug containers to the sponsor.

The Principal Investigator may ask to terminate the study due to administrative or other reasons. If this should be the case, appropriate measures which safeguard the interests of the participating subjects must be taken after verification.

By signing this protocol, the Principal Investigator confirms that he/she has read the entire trial protocol, agrees to its procedures, and will comply strictly with the formulated guidelines.

15. References

1. Agresti A, Min Y. (2001). On small-sample confidence intervals for parameters in discretedistributions. *Biometrics. 2001 Sep;57(3):963-71*

2. Alcami A & Smith GL (1992) A soluble receptor for interleukin-1 beta encoded by vaccinia virus: a novel mechanism of virus modulation of the host response to infection.*Cell* 71, 153-167.

3. Alcami A & Smith GL (1996) A mechanism for the inhibition of fever by a virus.*Proc.Natl.Acad.Sci.U.S.A* 93, 11029-11034.

4. Antoine G, Scheiflinger F, Dorner F, & Falkner FG (1998) The complete genomic sequence of the modified vaccinia Ankara strain: comparison with other orthopoxviruses.*Virology* 244, 365-396.

5. Blanchard TJ, Alcami A, Andrea P, & Smith GL (1998) Modified vaccinia virus Ankara undergoes limited replication in human cells and lacks several immunomodulatory proteins: implications for use as a human vaccine. *J.Gen.Virol.* 79 (Pt 5), 1159-1167.

6. Carroll MW & Moss B (1997) Host range and cytopathogenicity of the highly attenuated MVA strain of vaccinia virus: propagation and generation of recombinant viruses in a nonhuman mammalian cell line. *Virology* 238, 198-211.

7. Chan I (1998). Exact tests of equivalence and efficacy with a non-zero lower bound for comparative studies. *Statistics in Medicine* 17, 1403-1413.

8. Drexler I, Heller K, Wahren B, Erfle V, & Sutter G (1998) Highly attenuated modified vaccinia virus Ankara replicates in baby hamster kidney cells, a potential host for virus propagation, but not in various human transformed and primary cells. *J.Gen.Virol.* 79 (Pt 2), 347-352.

9. Goldstein JA, Neff JM, Lane JM, & Koplan JP (1975) Smallpox vaccination reactions, prophylaxis, and therapy of complications. *Pediatrics* 55, 342-347.

10. Guillaume JC, Saiag P, Wechsler J, Lescs MC, & Roujeau JC (1991) Vaccinia from recombinant virus expressing HIV genes. *Lancet* 337, 1034-1035.

11. Grabenstein JD, Winkenwerder W. Jr. (2003) US Military Smallpox Vaccination Program Experience. JAMA 2003, Vol. 289, No. 24, 3278 - 3282

12. Lane JM, Ruben FL, Neff JM, & Millar JD (1969) Complications of smallpox vaccination, 1968.*N.Engl.J.Med.* 281, 1201-1208.

13. Lane JM, Ruben FL, Neff JM, & Millar JD (1970) Complications of smallpox vaccination, 1968: results of ten statewide surveys. *J.Infect.Dis.* 122, 303-309.

14. Mahnel H & Mayr A (1994) [Experiences with immunization against orthopox viruses of humans and animals using vaccine strain MVA]. *Berl Munch.Tierarztl.Wochenschr.* 107, 253-256.

15. Mayr A, Hochstein-Mintzel V, & Stickl H (1975) Passage History: Abstammung, Eigenschaften und Verwendung des attenuierten Vaccina-Stammes MVA. *Infection* 3, 6-14.

16. Mayr A & Munz E (1964) [Changes in the vaccinia virus through continuing passages in chick embryo fibroblast cultures]. *Zentralbl.Bakteriol.[Orig.]* 195, 24-35.

17. Mayr A, Stickl H, Muller HK, Danner K, & Singer H (1978) [The smallpox vaccination strain MVA: marker, genetic structure, experience gained with the parenteral vaccination and behavior in organisms with a debilitated defence mechanism (author's transl)].*Zentralbl.Bakteriol.[B]* 167, 375-390.

18. McElwain WP (1972) Complications of smallpox vaccination.*J.Ky.Med.Assoc.* 70, 165-166.

19. Meyer H, Sutter G, & Mayr A (1991) Mapping of deletions in the genome of the highly attenuated vaccinia virus MVA and their influence on virulence. *J.Gen.Virol.* 72 (Pt 5), 1031-1038.

20. Redfield RR, Wright DC, James WD *et al.* (1987) Disseminated vaccinia in a military recruit with human immunodeficiency virus (HIV) disease. *N.Engl.J.Med* 316, 673-676.

21. Rosel JL, Earl PL, Weir JP, & Moss B (1986) Conserved TAAATG sequence at the transcriptional and translational initiation sites of vaccinia virus late genes deduced by structural and functional analysis of the HindIII H genome fragment. *J.Virol.* 60, 436-449.

22. Stickl, H. A. (1974) Smallpox vaccination and its consequences: first experiences with the highly attenuated smallpox vaccine "MVA". Prev.Med, 3[1], 97-101.

23. Stickl H & Hochstein-Mintzel V (1971) [Intracutaneous smallpox vaccination with a weak pathogenic vaccinia virus ("MVA virus")]. *Munch.Med.Wochenschr.* 113, 1149-1153.

24. Stickl H, Hochstein-Mintzel V, Mayr A *et al.* (1974) [MVA vaccination against smallpox: clinical tests with an attenuated live vaccinia virus strain (MVA) (author's transl)]. *Dtsch.Med.Wochenschr.* 99, 2386-2392.

25. Sutter G & Moss B (1992) Nonreplicating vaccinia vector efficiently expresses recombinant genes. *Proc.Natl.Acad.Sci.U.S.A* 89, 10847-10851.

16. Appendices

Appendix I: Toxicity Scale for laboratory values

SERUM CHEMISTRY				
**Lab value**	**Mild (Grade 1)**	**Moderate (Grade 2)**	**Severe (Grade 3)**	**Potentially Life Threatening (Grade 4)**
Sodium – Hyponatremia mmol/L	< LLN - ≥ 132	< 132 - ≥ 130	< 129 - ≥ 125	<125
Sodium – Hypernatremia mmol/L	≥ ULN - < 150	≥ 150 - < 155	≥ 155 - < 160	≥ 160
Potassium – Hyperkalemia mmol/L	≥ ULN - < 5.5	≥ 5.5 - < 6.0	≥ 6.0 - < 7.0	≥ 7.0
Potassium – Hypokalemia mmol/L	< LLN - ≥ 3.5	< 3.5 - ≥ 3.4	< 3.4 - ≥ 3.2	< 3.2
Calcium – Hypercalcaemia mmol/L	≥ ULN - < 2.9	≥ 2.9 - < 3.1	≥ 3.1 - < 3.4	≥ 3.4
Calcium - Hypocalcaemia mmol/L	< LLN - ≥ 2.0	< 1.75 - ≥ 2.0	< 1.6 - ≥ 1.75	< 1.5
Blood Urea Nitrogen BUN mg/dL	≥ ULN - < 27	≥ 27 - < 32	≥ 32	
Serum creatinine - mg/dL	≥ ULN - < 1.5 x ULN	≥ 1.5 - < 3 x ULN	≥ 3 - 6 x ULN	> 6 x ULN
Albumin - Hypoalbuminemia g/dL	< LLN - ≥ 2.7	< 2.7 - ≥ 2.5	< 2.5	
Total Protein - Hypoproteinemia g/dL	< LLN - ≥ 5.4	< 5.4 - ≥ 5.0	< 5.0	
Alkaline Phosphatase - increase by factor	> 1.25 - < 2.0 x ULN	≥ 2.0 - < 3.0 x ULN	≥ 3.0 x ULN	
Liver Function Tests - increase by factor	> 1.0 - < 2.5 x ULN	≥ 2.5 - < 4 x ULN	≥ 4 x ULN	
Total Bilirubin - increase by factor	> ULN – 1.5 x ULN	> 1.5 - 3.0 x ULN	> 3.0 - 10.0 x ULN	> 10.0 x ULN
Creatinine Kinase (CK)	>ULN - <2x ULN	≥ 2 - <5x ULN	≥ 5x ULN	
Creatine Kinase Myocardial Band (CK-MB)	>ULN – <2.0 x ULN	≥ 2.0 - < 5.0 x ULN	≥ 5.0 x ULN	
Cardiac Troponin I	>ULN – <2.0 x ULN	≥ 2.0 - < 5.0 x ULN	≥ 5.0 x ULN	
Total Cholesterol mg/dL	> ULN - 300	> 300 - 400	> 400	

HEMATOLOGY				
**Lab Value**	**Mild (Grade 1)**	**Moderate (Grade 2)**	**Severe (Grade 3)**	**Potentially Life Threatening (Grade 4)**
Hemoglobin (Female) - g/dl	< LLN - ≥ 10.5	< 10.5 - ≥ 10.0	< 10.0	
Hemoglobin (Male) – g/dl	< LLN - ≥ 12.0	< 12.0 - ≥ 11.0	< 11.0	
WBC Increase - cell/mm^3	> ULN - < 15,000	≥ 15,000 - < 20,000	≥ 20,000	
WBC Decrease - cell/mm^3	< LLN - ≥ 2,500	< 2,500 - ≥ 1,500	< 1,500	
Lymphocytes Decrease - cell/mm^3	< 1,000 - ≥ 750	< 750 - ≥ 500	< 500	
Neutrophils Decrease - cell/mm^3	< 2,000 - ≥ 1,500	< 1,500 - ≥ 1,000	< 1,000	
Platelets Decreased - cell/mm^3	< LLN - ≥ 75,000	< 75,000 - ≥ 50,000	< 50.000	
**URINE**				
Protein	Trace - ≤ 1+	> 1+ - ≤ 2+	> 2+ - ≤ 3+	≥ 3+ - nephritic syndrome
Glucose	Trace	1+	2+	>2+
Blood (microscopic) – red blood cells per high power field (rbc/hpf)	≥ 0- ≤10	≥ 10 - < 50	≥ 50	Gross
Blood (measured by Combur urine sticks)	Trace - ≤ 1+	> 1+ - ≤ 2+	> 2+ - ≤ 3+	≥ 3+
**SYSTEMIC QUANTITATIVE**				
Tachycardia - beats per minute	101 - 115	116 - 130	≥ 131	
Bradycardia - beats per minute	54 - 50	49 - 45	≤ 44	
Hypertension (systolic) - mm Hg	141 - 155	156 - 165	≥ 166	
Hypertension (diastolic) - mm Hg	91 - 95	96 - 100	≥ 101	
Hypotension (systolic) - mm Hg	89 - 85	84 - 80	≤ 79	

Appendix II: Case Definitions Acute Myocarditis / Pericarditis

Case Definition for Acute Myocarditis

A possible case of acute myocarditis is defined by the following criteria and the absence of evidence of any other likely cause of symptoms:
Presence of dyspnea, palpitations, or chest pain of probable cardiac origin in a patient with either one of the following:

- Electrocardiogram (ECG) abnormalities beyond normal variants, not documented previously, including
  - ST-segment or T-wave abnormalities,
  - Paroxysmal or sustained atrial or ventricular arrhythmias,
  - AV nodal conduction delays or intraventricular conduction defects, or
  - Continuous ambulatory electrocardiographic monitoring that detects frequent atrial or ventricular ectopy

Or

- Evidence of focal or diffuse depressed left-ventricular (LV) function of indeterminate age identified by an imaging study (e.g., echocardiography or radionuclide ventriculography).

A probable case of acute myocarditis, in addition to the above symptoms and in the absence of evidence of any other likely cause of symptoms, has one of the following:

- Elevated cardiac enzymes, specifically, abnormal levels of cardiac troponin I, troponin T, or creatine kinase myocardial band (a troponin test is preferred);
- Evidence of focal or diffuse depressed LV function identified by an imaging study (e.g., echocardiography or radionuclide ventriculography) that is documented to be of new onset or of increased degree of severity (in the absence of a previous study, findings of depressed LV function are considered of new onset if, on follow-up studies, these findings resolve, improve, or worsen); or
- Abnormal result of cardiac radionuclide imaging (e.g., cardiac MRI with gadolinium or gallium-67 imaging) indicating myocardial inflammation.

A case of acute myocarditis is confirmed if histopathologic evidence of myocardial inflammation is found at endomyocardial biopsy or autopsy.

Case Definition for Acute Pericarditis

A possible case of acute pericarditis is defined by the presence of

- Typical chest pain (i.e., pain made worse by lying down and relieved by sitting up and/or leaning forward) and no evidence of any other likely cause of such chest pain.

A probable case of acute pericarditis is a possible case of pericarditis, or a case in a person with pleuritic or other chest pain not characteristic of any other disease, that, in addition, has one or more of the following:

- Pericardial rub, an auscultatory sign with one to three components per beat,
- ECG with diffuse ST-segment elevations or PR depressions without reciprocal ST depressions that are not previously documented, or
- Echocardiogram indicating the presence of an abnormal collection of pericardial fluid (e.g., anterior and posterior pericardial effusion or a large posterior pericardial effusion alone).

A case of acute pericarditis is confirmed if histopathologic evidence of pericardial inflammation is evident from pericardial tissue obtained at surgery or autopsy.
